# A westerly wind dominated Puna Plateau during deposition of upper Pleistocene loessic sediments in the subtropical Andes, South America

**DOI:** 10.1038/s41467-022-31118-5

**Published:** 2022-06-14

**Authors:** Alex Pullen, David L. Barbeau, Andrew L. Leier, Jordan T. Abell, Madison Ward, Austin Bruner, Mary Kate Fidler

**Affiliations:** 1 Department of Environmental Engineering and Earth Sciences, Clemson University, Clemson, SC 29634 USA; 2 School of the Earth, Ocean and Environment, University of South Carolina, Columbia, SC 29208 USA; 3 Department of Geosciences, University of Arizona, Tucson, AZ 85721 USA; 4 Department of Geology, University of Kansas, Lawrence, KS 66045 USA

**Keywords:** Palaeoclimate, Sedimentology

## Abstract

The Tafí del Valle depression (~27° S) in the eastern Andes of Argentina provides a record of late Pleistocene dust deposition in the subtropics of South America. We present large-*n* U-Pb geochronology data for detrital zircons from upper Pleistocene loess-paleosol deposits. When compared to regional data, the age spectra from the Tafí del Valle samples are most like the southern Puna Plateau, supporting derivation largely from the west and northwest. This runs counter to hypotheses suggesting these loessic sediments were derived from the low elevation plains to the east or extra-Andean Patagonia. Mapping of linear wind erosion features on the Puna Plateau yield a mean orientation of 125.7° (1 s.d. = 12.4°). These new data and existing records are consistent with a westerly-northwesterly dominated (upper- and lower-level) wind system over the southern Puna Plateau (to at least ~27° S) during periods of high dust accumulation in Tafí del Valle.

## Abstract (web summary)

Detrital zircon ages in Pleistocene sediments and wind erosion patterns indicate the Puna Plateau was dominated by westerly winds during intervals of high dust accumulation in the eastern subtropical Andes.

## Introduction

Determining the provenance of loess and loessic paleosols provides an opportunity to better understand ancient wind and precipitation patterns, surface conditions, and atmospheric dust loading which impacts the Earth’s radiative forcing budget (e.g., ref. ^1^). Once deposited, lithogenic dust influences biogeochemical processes in terrestrial and ocean environments (e.g., refs. ^2–4^). Dust in the Southern Hemisphere is of particular importance because of the Southern Ocean’s iron limitation on the productivity of photosynthetic organisms and disproportionate role in modulating Pleistocene climate (e.g., refs. ^5,6^). A resolved understanding of Pleistocene dust production and transport in South American provides critical information about atmospheric circulation in the Southern Hemisphere and the nature of dust transported to the Southern Ocean and Antarctica during that interval.

**Fig. 1:**
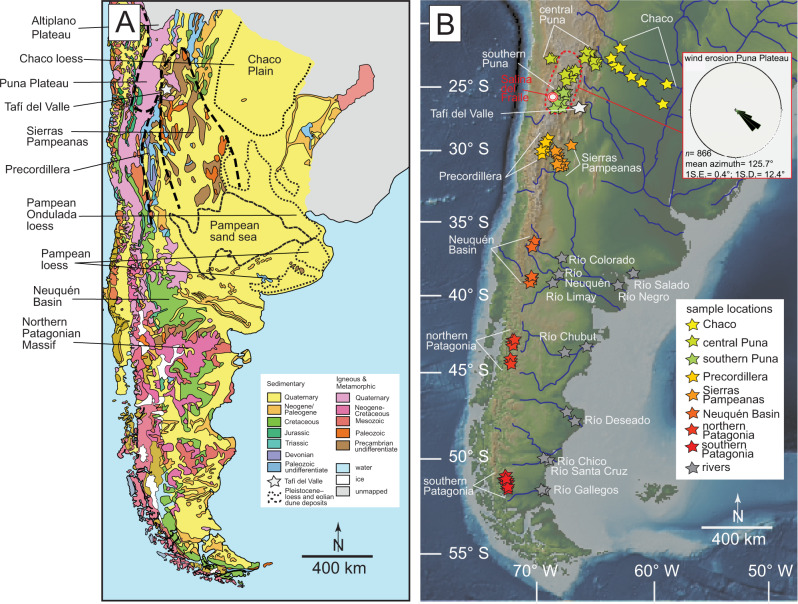
Maps of southern South America. **A** Simplified geologic map of Argentina and central and southern Chile based on compilation maps^54,55^. Distribution of La Pampa loess and Pampean Sand Sea after Iriondo (^11,46^) and Zárate (^7^). **B** Digital elevation model for southern portion of South America. Color filled stars denote locations of U-Pb zircon samples for comparison with Tafí del Valle samples: southern Puna Plateau^37,38^; central Puna Plateau^56–60^; Chaco^36,61^; Sierras Pampeanas^62–67^; Precordillera^67–69^; Neuquén basin^70^; northern Patagonia^71^; southern Patagonia^72^; Ríos Neuquén, Salado, Limay, Colorado, Negro Chubut, Gallegos, Santa Cruz, Chico, and Deseado^36^. Area of wind erosion study of Puna Plateau reported here highlighted by dashed red line; linear wind erosion data shown in inset rose plot.

The distribution of southern South America’s Quaternary dust deposits have been well documented (e.g., ref. ^7^), and broadly include the southern Pampa loess^8^, the Chaco Plain loess^9^, eolian deposits of the northern Pampa—Río de la Plata region^10,11^ and smaller deposits in the eastern Andes (or pre-Andes; Fig. 1A, B)^12^. Less certain, however, is the sediment provenance of these loess provinces, which is currently interpreted by competing models. Satellite data show that recent dust plumes originating from the Altiplano–Puna Plateau generally track towards the southeast across the low elevation plains crossing locations with upper Pleistocene–Holocene loessic strata (Fig. 1A, B)^13^. But synoptic winds, lake levels, ice volumes, and precipitation were much different for long intervals during the late Pleistocene to early Holocene (e.g., ref. ^14^), making the practice of applying a post-industrial dust transport model to the paleoclimate problematic.

Similarities in bulk geochemistry between loess deposits along the eastern flank of the Andes with lower elevation loess deposits in the Pampean plains to the east led to the interpretation that the loess deposits along the eastern flank of the subtropical Andes were derived from the south^15^. In this scenario, late Pleistocene loess deposits along the eastern flank of the Andes are envisioned to mark the western edge of a dust source in the extra-Andean regions of Patagonia carried by southerly winds^15,16^. Alternatively, provenance of the late Pleistocene loess deposits along the eastern flank of the Andes have also been attributed to the Chaco Plain, including parts of eastern Bolivia, western Paraguay, and northwest Argentina^11^; this ‘Chaco model’ implicates northerly winds. Widely documented wind erosion of the southern Altiplano–Puna Plateau (e.g., refs. ^17–21^) suggests that the loess deposits along the eastern flank of the Andes were largely sourced from the west and northwest, and that low elevation plains of southern South America played a negligible role in the sourcing of this eolian detritus. Satellite based studies show the feasibility—at least under present-day-like climate conditions—of the Puna-Altiplano Plateau supplying dust to the eastern flank of the Andes and Chaco and Pampean plains to the east (e.g., refs. ^13,22^). Many modern dust events between 28°–37° S along the eastern flank of the Andes out to the Argentine plains result from short duration polar front outbreaks^23^. These short duration shifts, on the order of several hours, most frequently occur in the austral autumn–winter months and result in westerly katabatic (referred to as Zonda) winds crossing the Andes and transporting dust towards the Argentine plains^24,25^. Today, the core of the dust-carrying modern Zonda winds tends to be around 32° S^26^.

Modern observations add to the ambiguity surrounding the provenance of the loess deposits along the eastern flank of the Andes. In addition to dust production within the high elevation Andes, present-day dust plumes originate at lower elevations (e.g., Mar Chiquita, a large saline lake in the central Argentine plains). These plumes indicate components of north- and south-directed atmospheric transport^27^, further highlighting the uncertainty of southern South American loess provenance. This is particularly important considering the variability and differences between atmospheric circulation during the late Pleistocene and the present^14^. To better understand Quaternary dust provenance and wind erosion in southern South America and to help elucidate synoptic wind patterns during periods of dust accumulation over that time interval, we present new detrital-zircon geochronology data from an upper Pleistocene loess-paleosol succession near Tafí del Valle, Argentina, along with supporting wind erosion data from the Puna Plateau.

## Geologic setting

**Fig. 2:**
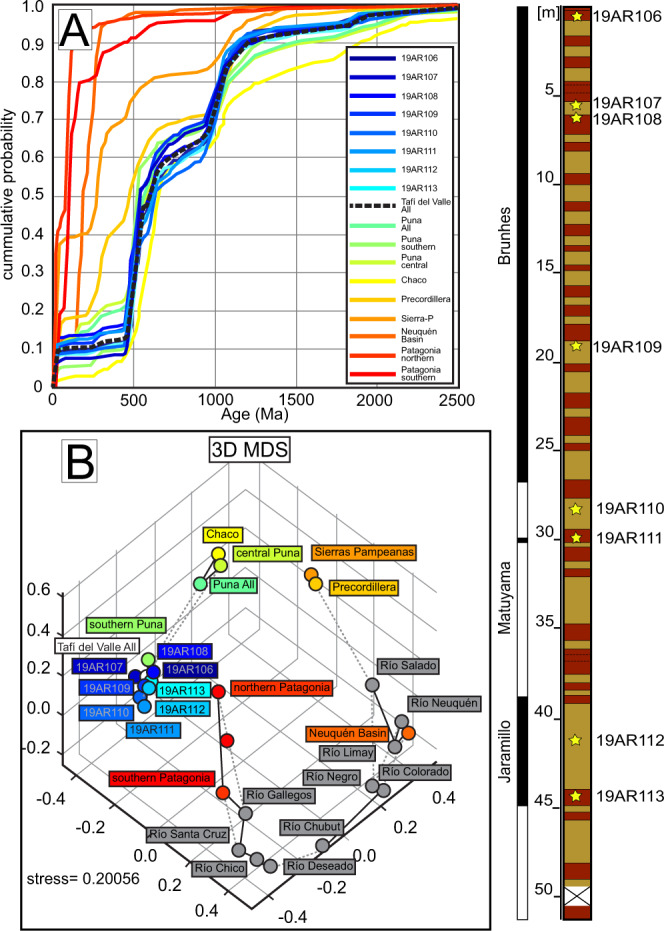
U-Pb detrital zircon data. **A** U-Pb detrital zircon cumulative density functions for Tafí del Valle samples and comparison data. References for comparison data in the Fig. 1 caption with descriptions in Supplementary Table S2. **B** 3-dimensional multi-dimensional scaling (MDS) plot of U-Pb detrital zircon data for Tafí del Valle samples and comparison data. Measured and sampled stratigraphy, correlated with magnetostratigraphic timescale from Schellenbreger et al. (ref. ^29^); tan denotes loess and crimson denotes paleosol layers. 0 meters represents the top of the section and youngest strata.

Tafí del Valle is located at ~2200 m a.s.l. in a ~100 km^2^ topographical depression in northwestern Argentina along the boundary between the arid southern Puna Plateau and semi-arid Chaco Plain (Fig. 1A, B). This area receives orographic precipitation when moisture-rich air of the South American low-level jet travels southward along the eastern slope of the Andes^28^. Like other notable desert-fringing loess provinces such as the Chinese Loess Plateau and the Negev Desert, the Tafí del Valle loess has accumulated in an area along a steep precipitation gradient. Several exposures of laterally and vertically continuous interbedded loess and paleosol beds have been documented within the Tafí del Valle area^12,15^. The Las Carreras section, studied here, contains a ~50 m thick stratigraphic section with 32 unique paleosol horizons interbedded with loess^29,30^ (Fig. 1B). The base of the Las Carreras section is assigned a minimum age of 1.15 Ma based on magnetostratigraphy^29^; an age that is corroborated by optically-stimulated luminescence dating of a nearby 42-m loess-paleosol succession^31^. The age and number of interbedded loess and paleosol beds exposed in Las Carreras suggests a sub-100-ky orbital forcing signal on pedogenesis^30^. The *N* = 8 loess and paleosol samples collected for this study range in age from ~6 ka to ~1.05 Ma based on the Schellenberger et al. (ref. ^29^) age model (Fig. 2). This sampling strategy was taken to sample strata presumably deposited around drier/cooler and wetter/warmer intervals in Tafí del Valle across the late Pleistocene and early Holocene.

The surface of the Puna Plateau to the west and northwest of the field area indicates extensive wind modification. Geomorphic features include yardangs, ventifacts, wind megaripples, and bedrock keeling (‘wind tails’). Organization of gravel-mantled ripples on the Puna Plateau and linear erosional bedrock features indicate principally northwesterly geomorphically effective near-surface winds^18,19,32^ (Fig. 1; data presented herein). The southern Puna Plateau includes up to 1.95 km of vertical wind deflation in the Salina del Fraile—approximately 250 km northwest of the Tafí del Valle field area—since the ~mid-Miocene^21^ (Fig. 1B).

**Fig. 3:**
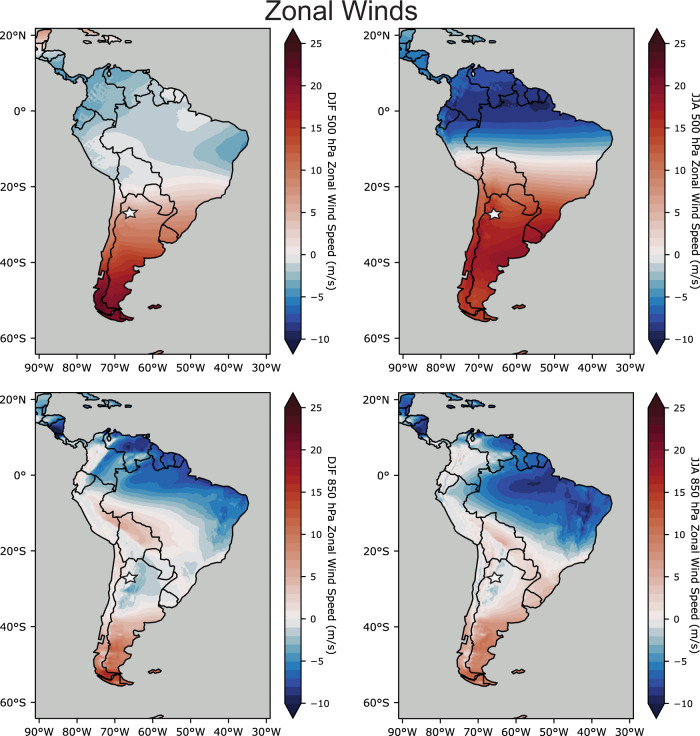
The zonal components of wind at 500 hPa and 850 hPa for 1979–2021. Data from the ERA5 Reanalysis product^73^. Positive values (red) denote westerly winds, whereas negative values (blue) denote easterly winds. The white star indicates the field location in Tafí del Valle.

The field area in Tafí del Valle (~27° S) is located beneath the northernmost position of the present-day subtropical westerly jet stream^33,34^. During austral winter months, the subtropical jet shifts northward, such that the middle-to-upper tropospheric westerly winds move equatorward and further encompass Tafí del Valle^33^ (Fig. 3). However, the interannual mean position of the lower-level Southern Hemisphere westerlies remains south of Tafí del Valle throughout the year with only periodic incursions (Fig. 3). This puts Tafí del Valle to the north of most sustained lower-level westerly wind activity, including Zonda winds, today.

## Results

Linear wind erosion features on the southern Puna Plateau were mapped using composite satellite images to better understand the spatial distribution and orientations of these features with respect to the Tafí del Valle field area. These features include yardangs, wind streaks, and eolian modified interfluves (e.g., ref. ^35^). The *n* = 866 measurements yielded a mean orientation of 125.7° with low variability (1 s.d. = 12.4°; Fig. 1B; location and orientation of mapped elements are provided in Supplementary Table S1).

Detrital zircon geochronology was applied to Tafí del Valle deposits for its usefulness in assessing the provenance of highly mixed loessic sediments. The detrital zircon ages range from Pleistocene to Archean (Fig. 2A). The major age modes (and the range in percent of sample composition) are as follows: 0–23 Ma (6.5–11.8%); 450–650 Ma (38.5–50.0%), and 950–1200 Ma (20.5–40.7%; Supplementary Table S2; Table S3; Supplementary Fig. S1, S2). These major age modes also compose major components in potential source areas for the Las Carreras loess-paleosol sequence and are widely associated with the magmatic history of western South America^36^. However, the major age modes along with minor age components can be recognized in different relative proportions by location through large-observation (large-*n*) data sets (Fig. 2A; S2) like those presented here (*n* = 391–526).

## Discussion

Comparison of the detrital zircon age spectra from Tafí del Valle with those of potential source areas inform the provenance of dust deposited in the subtropics along the eastern flank of the Andes during the late Pleistocene and early Holocene. In Fig. 2, we examine the age similarities and differences between the Tafí del Valle deposits and potential source areas along observed and proposed dust transport pathways (e.g., refs. ^11,13,15^). This is aimed at testing whether the upper Pleistocene and Holocene loessic deposits in Tafí del Valle were sourced from: (1) the lowlands of the Chaco Plain to the east^11^, presumably when the Chaco was drier; (2) the extra-Andean areas of Patagonia (e.g., ref. ^15^), with possible storage of sediment in the Pampean plains; or (3) from the west-northwest on the Puna Plateau.

Zircon geochronology data from the southern Puna Plateau^37,38^ are most similar to data for individual and aggregate cumulative distributions of Tafí del Valle samples (Fig. 2A and Supplementary Fig. S1, S2). These similarities are supported by their locations in multi-dimensional scaling (MDS) space (Fig. 2B). We interpret this to mean that the southern Puna Plateau, to the west-northwest of the field area, contributed a large portion of the >20 μm detritus—20 μm being the analytical limit of the U-Pb measurements—to the upper Pleistocene and Holocene Tafí del Valle loess-paleosol strata. Considering the proximity of the southern Puna Plateau to Tafí del Valle, as well as the intervening high topography of the Eastern Cordillera between wind eroded areas of the Puna Plateau and Tafí del Valle, we speculate that most of the fine-grained fraction of detritus was likely sourced from this area as well. Rivers regionally sample bedrock, and river floodplains are often sources for desert-fringing loess deposits^39,40^. To that end, in addition to using bedrock samples for detrital zircon age comparisons, we quantitatively compare our data with river samples from La Pampa and the extra-Andean Patagonia regions as a proxy for detrital zircon populations that could have been derived by winds to the south of Tafí del Valle (Fig. 2B, S1, S2). The detrital zircon data from the Sierra Pampeanas foreland, northern and southern Patagonian Andes—all areas south of Tafí del Valle—indicate poor quantitative similarities to the Tafí del Valle sediments (Fig. 2B). We interpret this to mean that most of the silt and larger fraction of detritus in the upper Pleistocene to Holocene Tafí del Valle loess-paleosol strata was not derived from southerly winds as previously proposed. Published data from the Chaco Plain is also a poor fit for the Tafi del Valle data, suggesting alternative wind orientations played a limited role in supplying the dust deposited in Tafí del Valle (Fig. 2B). Notably, the mobilization of Puna Plateau sediments within the westerly-northwesterly winds is consistent with inferences from bulk geochemical analysis of late Pleistocene dust sampled from the Antarctic ice sheet that largely point to the Altiplano–Puna Plateau as a prominent proto-source for this dust^41^. Such a sediment routing pathway is also largely consistent with last glacial maximum (LGM) South American dust flux in general circulation models (e.g., ref. ^42^).

The high concentration of volcanic-derived particles in Tafí del Valle strata has been used to argue for a significant direct volcanic contribution to the loess-paleosol succession^12,30^. However, only *n* = 2 detrital zircons (of *n* = 4011) in the Tafí del Valle samples yielded U-Pb ages that overlap (within uncertainty) the depositional ages of the respective samples. We interpret this to mean that direct volcanic contribution to the silt and larger fraction of the loess-paleosol strata was perhaps lower than previously thought—present but likely overwhelmed by the signal from older (e.g., Miocene) volcanic bedrock. Despite the absence of directly deposited volcanic zircons in our data, the percentage of detrital zircon with ages younger than 23 Ma is significant. This, coupled with observations of wind eroded volcanic deposits on the southern Puna Plateau, suggests substantial eolian reworking of Miocene and younger volcanic rocks.

Near surface wind orientations reconstructed from bedrock erosion features on the Puna Plateau and the volumetrically large wind deflation documented in areas like Salina del Fraile^21^ (Fig. 1B) could explain the abundance of loessic deposits along the eastern flank of the subtropical Andes. Tafí del Valle, at least presently, is located along a steep precipitation gradient, but synoptic changes in moisture are needed to explain the interbedding of loess and paleosol strata.

In addition to evidence of westerly-northwesterly wind-supplied eolian sediment to the subtropical eastern Andes, the Tafí del Valle data do not indicate a significant shift in provenance throughout the stratigraphic section studied here at large-*n*. This observation is valuable for several reasons. (1) It implies little change to the sourcing of loessic sediment to Tafí del Valle across the Mid-Pleistocene Transition—an important change in the forcing of Earth’s glacial climate^43,44^. (2) The lack of variability in detrital zircon provenance between loess and paleosol strata implies either little variation in sediment sourcing between dry and humid periods (assuming a relatively continuous accumulation of dust)—albeit with potentially different accumulation rates—or a preponderance of dust accumulation during drier periods with pedogenesis occurring during more humid intervals (as noted by ref. ^30^).

Presumably dust production in central and southern South America looked different throughout much of the late Pleistocene relative to present-day because of much different climatic and surficial conditions (e.g., ref. ^14^). This would include changes in wind intensity and position across spatial and temporal scales. Geomorphic evidence from the low elevation Pampean plains point toward important differences in near surface winds and surficial conditions during intervals of the late Pleistocene and early Holocene. Eolian dune fields of the Pampean Sand Sea were periodically active during the late Pleistocene and early Holocene but are mostly vegetation-stabilized at present^45^. The Pampean Sand Sea covers >2*10^5^ km^2^ (Fig. 1A)^46^, which includes extensive low-amplitude (<10 m), longitudinal, kilometer-scale deflationary zones. Referred to as ‘las cubetas de deflación’, these deflationary depressions exhibit an anticlockwise pattern in satellite images across >1*10^5^ km^2^ of the Pampean plains, retaining the geomorphically effective wind pattern that formed them, which may be a poor fit for present-day winds^47^. A higher present-day water table compared to when the deflation depressions formed has resulted in widespread shallow lakes across the Pampean plains, highlighting a lack of ongoing deflation. These geomorphic features in the Argentine lowlands are an indication of much different surface, moisture, synoptic wind, and dust production conditions during much of the late Pleistocene and early Holocene. To that end, applying a present-day-like dust model to South America for the Pleistocene would be imprudent.

Westerly-northwesterly winds supplied dust to Tafí del Valle during the late Pleistocene and early Holocene. But the nature of this westerly-northwesterly system(s) and the duration of its emplacement when dust was generated on the southern Puna and deposited in Tafí del Valle is uncertain. Paleoclimate data demonstrate that conditions in South America during the Pleistocene and Holocene were, for long intervals, much different than the present-day, with only brief periods approximating present-day-like conditions (e.g., ref. ^14^). A model aimed at explaining central South American dust dynamics across most of the late Pleistocene to early Holocene would need to consider not only the data presented here, but also explain (1) a < 100-ky periodicity in pedogenesis in Tafí del Valle, and (2) major differences between the present-day and late Pleistocene and early Holocene in the low elevation plains of southern South America to include large-scale eolian dune mobilization and deflation. To that end, we provide a brief discussion of the possible scenarios which could satisfy (most of) the available data.

Tafí del Valle is located at or slightly beyond the northern influence of present-day Zonda winds^23,25^ and within the dust generating influence of the Southern Hemisphere subtropical jet, particularly during the austral winter months. In a seminal study, Gaiero et al.^13^ utilized satellite data, a particle transport model, and geochemical analyses to suggest that dust storms in 2009 and 2010 originating from the Puna Plateau were associated with northward incursions of polar frontal systems, and experienced subsequent eastward transport related to the subtropical jet. While dust can be generated in this region year-round, today it is these short-term (hour- to day-long) events that drive substantial dust production. If this same concept was proposed for longer-term dust generation (i.e., the time interval covered by the geochemical data in this study), it would explain the uniformly westerly-northwesterly derived dust at Tafí del Valle, but it would be insufficient to explain the periodicity of loess accumulation and pedogenesis, which would have to be controlled by longer duration synoptic changes. Additionally, short-term displacements in zonal winds would not (alone) explain prolonged periods of greater-than-present aridity, wind erosion, and dust production in the Pampean plains.

Alternatively, longer-term, orbitally-forced processes may explain the loessic sediments and pedogenic cycles in Tafí del Valle, and provide a mechanism for changes in eolian transport and wind erosion at lower elevations in the Pampean plains. One possibility is that the frequency of the storm systems driving cold air advection to lower latitudes, and thus generating dust outbreaks on the Puna Plateau, varies temporally, which would be associated with changes in the jet streams and midlatitude westerlies^48^. An additional mechanism could relate to a time-varied mean position of the Zonda winds, which today predominantly affect ~32–33° S^23^, but have been suggested to shift with climate changes in the past (e.g., ref. ^49^). These local features of atmospheric circulation are fundamentally connected to the subtropical jet stream and/or the midlatitude westerlies, and there is abundant evidence for variations in both of these systems at precessional (~19–23 ky), obliquity (~41 ky), and eccentricity (~100 ky) timescales across the Pleistocene and Holocene^49–52^. Considering the new geochemical and geomorphologic data produced here, as well as existing records of pedogenesis, eolian activity, and hydrologic changes in nearby regions, we suggest that longer-term displacements in synoptic winds likely need to be invoked to explain the provenance of the loessic strata in Tafí del Valle. We note that our records of dust provenance cannot distinguish the timescales of atmospheric circulation changes, but sub-eccentricity variability is likely present^30^.

In summary, comparisons of U-Pb detrital zircon age spectra from upper Pleistocene loess and paleosol strata in the Tafí del Valle area of the Andean foothills with potential source areas indicate these crystals were sourced to the west and northwest on the Puna Plateau. This challenges previous assertions that the subtropical loess along the eastern flank of the Andes in South America was primarily sourced from extra-Andean Patagonia or the Chaco Plain. However, westerly-northwesterly derivation is consistent with extensive bedrock wind erosion on the Puna Plateau. If this provenance scenario is valid, it implies the emplacement of a westerly-northwesterly dominated wind system at ~27° S during periods of high dust accumulation at Tafí del Valle in the late Pleistocene and early Holocene.

## Methods

### Sampling and mineral separation

U-Pb detrital zircon samples were collected from the Las Carreras upper Pleistocene to Holocene section described in Schellenberger et al^29^. and Schellenberger and Veit^30^. A handheld GPS and photos from Schellenberger and Veit^30^ were used to confirm the location of the Las Carreras section. The field investigation and sample collection were completed with permission of the landowner. Samples were exported with permission of Argentine Customs Authority for academic use. Following the convention of Schellenberger and Veit^30^, the section was measured from the stratigraphically youngest paleosol (S0) down to the base of the measured section. We used two investigators on the outcrop and one spotter to improve reproducibility of the stratigraphic measurement. *N* = 8 samples, weighing 2–3 kg each, were collected at 0.2 m (19AR106), 5.1 m (19AR107), 5.9 m (19AR108), 19 m (19AR109), 28.7 m (19AR110), 30.2 m (19AR111), 42 m (19AR112), and 43.2 m (19AR113) depth.

Zircon crystals were separated from the loess and paleosol samples using low hydraulic energies and ultrasonic disruption to minimize mineral separation induced grain-size age biases. Once the clay fraction was isolated from the zircon containing mineral fraction, methylene iodide was used to isolate the dense minerals. The zircon fraction was then isolated from the dense fraction using a barrier Frantz instrument. Aliquots of samples at different magnet settings were investigated under a reflected light microscope to check for the presence of zircon in the ‘magnetic’ fraction thus minimizing the introduction of mineral separation induced age bias. Zircon separates were homogenized and then poured onto double-sided tape to create 2.5 cm diameter cylindrical epoxy mounts for each sample. The epoxy zircon mounts were imaged using BSE on a Hitachi 3400 N SEM. These images were used to identify and avoid non-zircon on mounts and to avoid inclusions within zircon during LA-ICP-MS analysis.

### Laser-ablation inductively-coupled-plasma mass-spectrometry

A Photon Analyte-G2 193 nm Excimer laser with a HelEx sample cell was used to ablate the detrital zircon crystals. Laser energy was set to 7 mJ. A He carrier gas was used to deliver analyte to the plasma. MFC1 was set to 0.10 l min^−1^ and MFC2 was set to 0.30 l min^−1^. A 3 burst 50 μm circular pre-ablation pass was used to clean sample surfaces and a 20 μm circular spot was used for analysis. Isotope ratios were measured on a Nu Instruments HR Multi-collection ICP-MS. Cool gas was set to 13.0 l min^−1^, auxiliary gas to 0.80 l min^−1^, and sample/make-up gas to 1.06 l min^−1^. RF power was set to 1300 W. Faraday collectors were used to measure ^206^Pb, ^207^Pb, ^208^Pb, ^232^Th, ^238^U, whereas ^202^Hg and ^204^Pb + Hg were measured on ion counters. Isotope ratios were determined from a 6 s ablation window using a total counts method. Elemental and mass fractionation, initial-Pb correction, and instrument drift were corrected using the FC-1 and R33 zircon reference materials and the open-source data reduction AgeCalcML MATLAB code^53^.

External uncertainties on ^206^Pb/^238^U for all samples ranged from 0.80% (2σ) to 0.38% (2σ), whereas the external uncertainties on ^206^Pb/^207^Pb ranged from 0.50% (2σ) to 0.36% (2σ). Following convention, the uncertainties shown below with the ages include only the internal uncertainties shown at 1σ. The ‘Best Age’ was assigned as ^206^Pb/^238^U age when the ^206^Pb/^238^U was <900 Ma, whereas the ^206^Pb/^207^Pb age was used when ^206^Pb/^238^U was >900 Ma. Ages >600 Ma with >20% discordance or >5% reverse discordance were not considered; as were dates with > ±10% internal uncertainty.

### Geomorphic mapping

Linear wind erosion features were mapped on Landsat images in Google Earth (GE) Pro. Measurements between Landsat images in GE Pro were avoided to minimize errors induced from georegistration problems. Linear features were mapped as lines in GE Pro defining the long axis of the lineation. The geomorphic nature of linear features was determined through analysis of landform geometry, size, local- and regional slope, and relief in GE. Geographic coordinates for the starting and ending positions of lines were converted to azimuth (°) in Excel.

## Supplementary information

Supplementary Information

## Data Availability

All new data generated in this study are available in the manuscript and supplementary material.
