# Supplementary material for: A westerly wind dominated Puna Plateau during deposition of upper Pleistocene loessic sediments in the subtropical Andes, South America
Source: Nat Commun. 2022 Jun 14;13:3411. doi: 10.1038/s41467-022-31118-5 (PMC9197825; doi:10.1038/s41467-022-31118-5)
Supplement: Supplementary file 1 — Supplementary Information [file 41467_2022_31118_MOESM1_ESM.pdf]

Figure S1

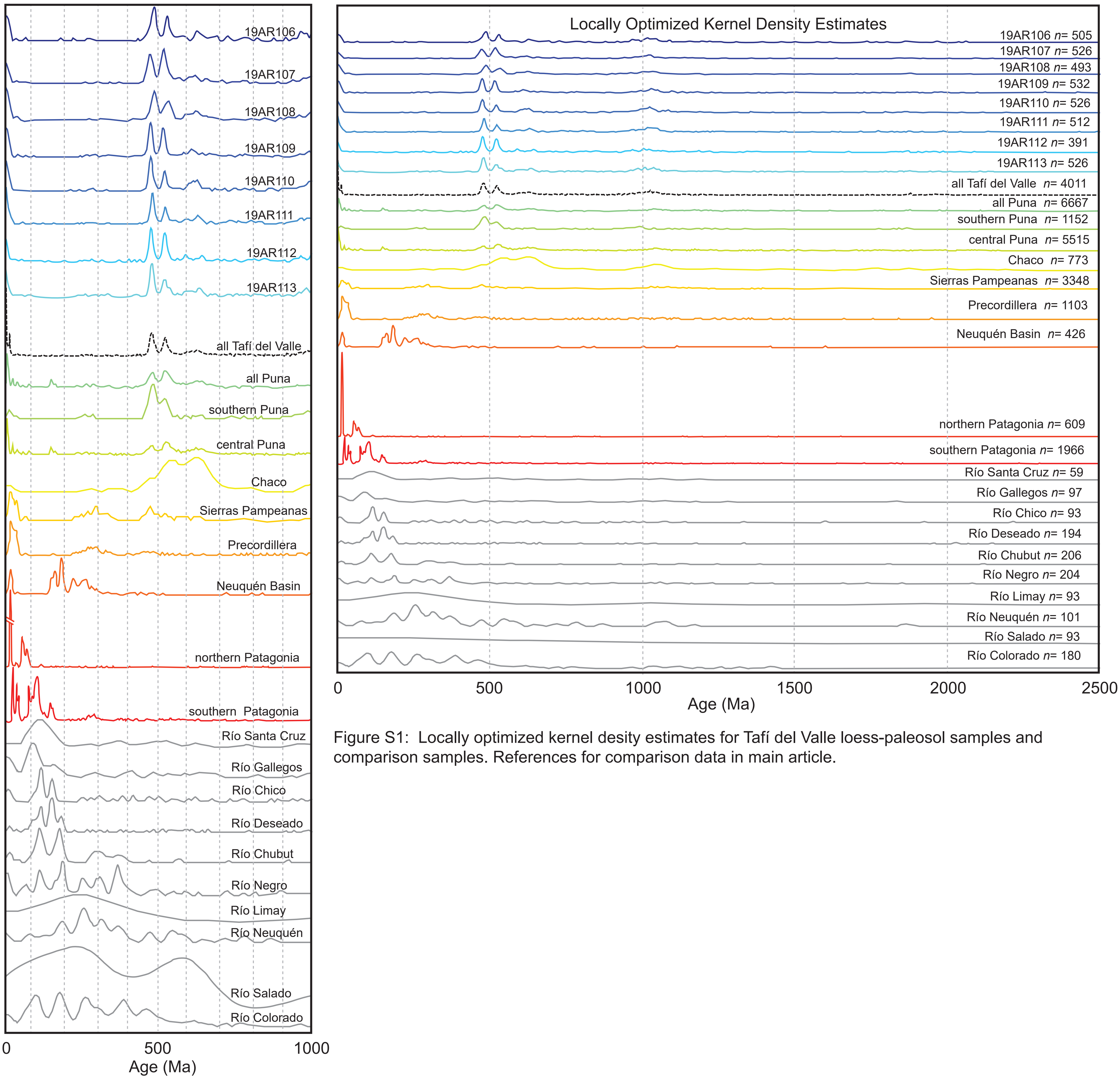

Figure S1: Locally optimized kernel desity estimates for Tafí del Valle loess-paleosol samples and comparison samples. References for comparison data in main article.

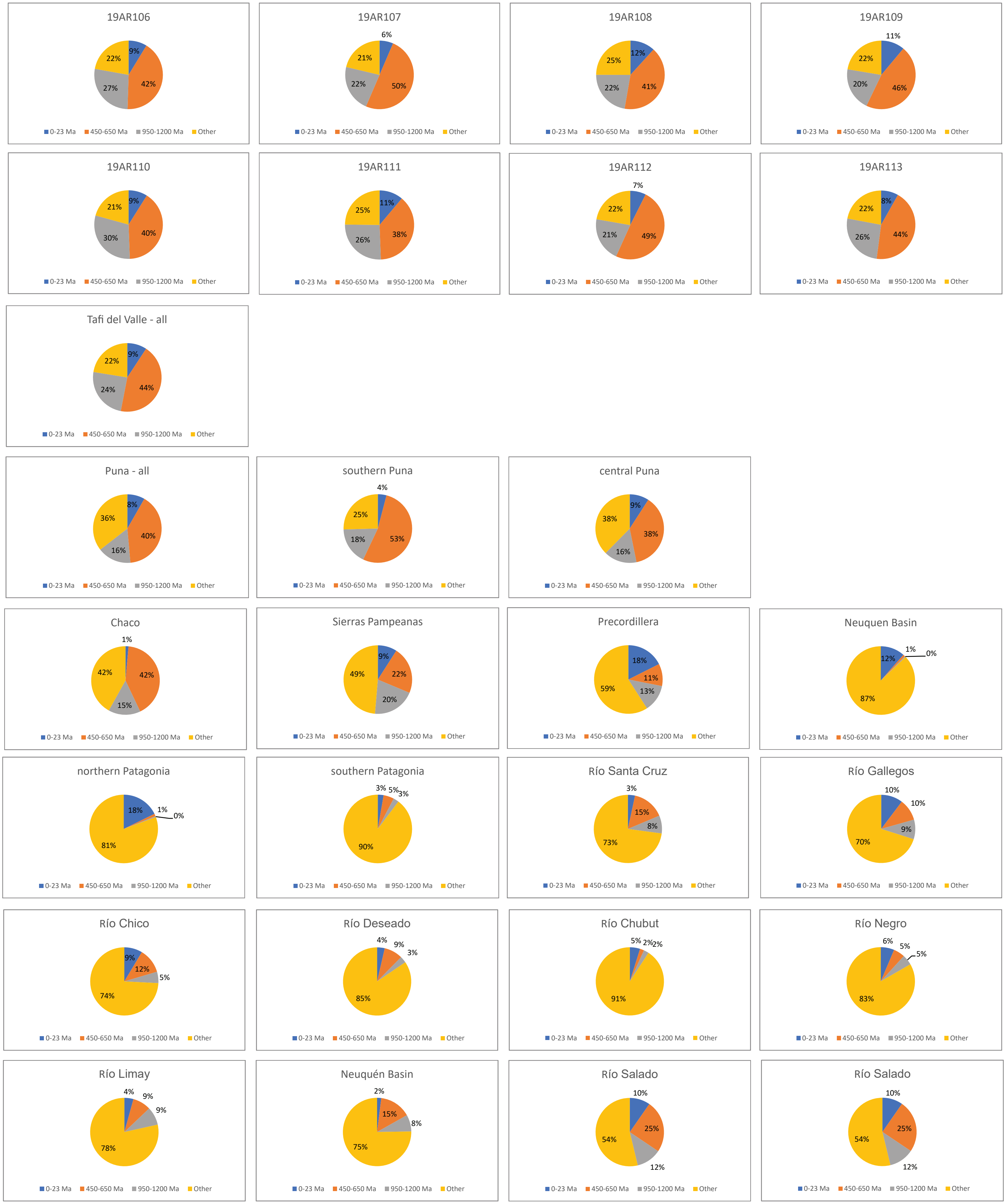

Figure S2

Figure S2: U-Pb detrital zircon data for Tafí del Valle and comparison data.

Supplementary Table S1

Location, nature, and orientations of wind erosion features

	Name	Type of feature	Starting Long.	Starting Lat.	Long axis azimuth (°)
1	Puna--wind erosion features	yardang	-67.45447735	-25.28390287	**133.6**
2	Puna--wind erosion features	yardang	-67.44787535	-25.27799636	**154.8**
3	Puna--wind erosion features	yardang	-67.44415029	-25.27590279	**138.9**
4	Puna--wind erosion features	yardang	-67.44263415	-25.28157435	**139.3**
5	Puna--wind erosion features	yardang	-67.44200116	-25.27812326	**130.4**
6	Puna--wind erosion features	yardang	-67.44441163	-25.25810214	**139.0**
7	Puna--wind erosion features	yardang	-67.4478775	-25.25871471	**137.5**
8	Puna--wind erosion features	yardang	-67.44675262	-25.25938712	**130.3**
9	Puna--wind erosion features	yardang	-67.46645451	-25.24961272	**136.8**
10	Puna--wind erosion features	yardang	-67.46466893	-25.25114301	**131.9**
11	Puna--wind erosion features	yardang	-67.46602683	-25.24764425	**137.4**
12	Puna--wind erosion features	yardang	-67.46403982	-25.24769313	**129.1**
13	Puna--wind erosion features	yardang	-67.46344869	-25.24871652	**126.3**
14	Puna--wind erosion features	yardang	-67.46520661	-25.24975374	**128.4**
15	Puna--wind erosion features	yardang	-67.4626135	-25.25055623	**125.0**
16	Puna--wind erosion features	yardang	-67.46214328	-25.25073663	**129.1**
17	Puna--wind erosion features	yardang	-67.46062826	-25.25185148	**135.6**
18	Puna--wind erosion features	yardang	-67.46043944	-25.25174841	**136.0**
19	Puna--wind erosion features	yardang	-67.45966052	-25.25205909	**127.9**
20	Puna--wind erosion features	yardang	-67.46109207	-25.25086199	**128.2**
21	Puna--wind erosion features	yardang	-67.46034072	-25.25362505	**133.1**
22	Puna--wind erosion features	yardang	-67.45898644	-25.25289044	**136.0**
23	Puna--wind erosion features	yardang	-67.45924656	-25.25176519	**129.8**
24	Puna--wind erosion features	yardang	-67.4590637	-25.24769518	**128.8**
25	Puna--wind erosion features	yardang	-67.4580888	-25.25250748	**134.9**
26	Puna--wind erosion features	yardang	-67.46706785	-25.24645294	**133.2**
27	Puna--wind erosion features	yardang	-67.47147494	-25.24382628	**115.0**
28	Puna--wind erosion features	yardang	-67.47305051	-25.24200207	**140.3**
29	Puna--wind erosion features	yardang	-67.47222998	-25.24175478	**126.3**
30	Puna--wind erosion features	yardang	-67.47384074	-25.23985516	**136.3**
31	Puna--wind erosion features	yardang	-67.46976786	-25.24319556	**127.5**
32	Puna--wind erosion features	yardang	-67.47019688	-25.24687075	**130.4**
33	Puna--wind erosion features	yardang	-67.46846017	-25.24688854	**142.5**
34	Puna--wind erosion features	yardang	-67.46824423	-25.24569527	**132.4**
35	Puna--wind erosion features	yardang	-67.47195567	-25.24509431	**131.5**
36	Puna--wind erosion features	yardang	-67.47228997	-25.24731157	**133.6**
37	Puna--wind erosion features	yardang	-67.47089064	-25.24885532	**124.7**
38	Puna--wind erosion features	yardang	-67.46876158	-25.25034039	**122.5**
39	Puna--wind erosion features	yardang	-67.47247893	-25.24676438	**135.0**
40	Puna--wind erosion features	yardang	-67.47348597	-25.2463784	**124.6**
41	Puna--wind erosion features	yardang	-67.47402892	-25.24599147	**132.4**
42	Puna--wind erosion features	yardang	-67.46643812	-25.24365073	**126.4**
43	Puna--wind erosion features	yardang	-67.46477076	-25.24360498	**124.6**
44	Puna--wind erosion features	yardang	-67.465686	-25.24373192	**147.8**
45	Puna--wind erosion features	yardang	-67.46420078	-25.24344503	**132.9**
46	Puna--wind erosion features	yardang	-67.46858817	-25.23833468	**131.9**
47	Puna--wind erosion features	yardang	-67.46290221	-25.24133732	**140.3**
48	Puna--wind erosion features	yardang	-67.46635708	-25.23700166	**133.4**
49	Puna--wind erosion features	yardang	-67.47031191	-25.23753363	**126.2**
50	Puna--wind erosion features	yardang	-67.45498856	-25.23517096	**135.1**
51	Puna--wind erosion features	yardang	-67.45632343	-25.23476586	**129.3**
52	Puna--wind erosion features	yardang	-67.45530822	-25.23479093	**133.0**
53	Puna--wind erosion features	yardang	-67.45465216	-25.23573253	**123.4**
54	Puna--wind erosion features	yardang	-67.45319278	-25.23591416	**130.0**
55	Puna--wind erosion features	yardang	-67.45310241	-25.2361815	**136.5**
56	Puna--wind erosion features	yardang	-67.45279109	-25.23634248	**135.7**
57	Puna--wind erosion features	yardang	-67.45876214	-25.23005803	**125.2**
58	Puna--wind erosion features	yardang	-67.45532778	-25.23141342	**141.0**
59	Puna--wind erosion features	yardang	-67.45914731	-25.22952535	**128.3**
60	Puna--wind erosion features	yardang	-67.45807957	-25.23026218	**133.6**
61	Puna--wind erosion features	yardang	-67.46022589	-25.22999345	**134.0**
62	Puna--wind erosion features	yardang	-67.45632096	-25.22846909	**143.4**

63	Puna--wind erosion features	yardang	-67.45602296	-25.2284641	**137.9**
64	Puna--wind erosion features	yardang	-67.45503523	-25.2285839	**137.4**
65	Puna--wind erosion features	yardang	-67.45762338	-25.23108329	**120.8**
66	Puna--wind erosion features	yardang	-67.46156146	-25.227709	**137.0**
67	Puna--wind erosion features	yardang	-67.46203297	-25.22842705	**119.2**
68	Puna--wind erosion features	yardang	-67.46168901	-25.22890488	**117.3**
69	Puna--wind erosion features	yardang	-67.45672221	-25.22980375	**131.2**
70	Puna--wind erosion features	yardang	-67.45009124	-25.23672781	**145.8**
71	Puna--wind erosion features	yardang	-67.44936342	-25.23770067	**138.6**
72	Puna--wind erosion features	yardang	-67.4490416	-25.23739092	**147.6**
73	Puna--wind erosion features	yardang	-67.44362362	-25.23822554	**146.9**
74	Puna--wind erosion features	yardang	-67.46437278	-25.22126706	**122.0**
75	Puna--wind erosion features	yardang	-67.46225928	-25.22169855	**113.6**
76	Puna--wind erosion features	yardang	-67.44439842	-25.21453768	**129.4**
77	Puna--wind erosion features	yardang	-67.44330744	-25.21496139	**126.8**
78	Puna--wind erosion features	yardang	-67.44967474	-25.21149637	**120.9**
79	Puna--wind erosion features	yardang	-67.441874	-25.20467341	**133.1**
80	Puna--wind erosion features	yardang	-67.45198536	-25.2072865	**129.4**
81	Puna--wind erosion features	yardang	-67.4555111	-25.20266273	**154.0**
82	Puna--wind erosion features	yardang	-67.45374169	-25.20475553	**136.9**
83	Puna--wind erosion features	yardang	-67.45406728	-25.20128006	**132.4**
84	Puna--wind erosion features	yardang	-67.45327579	-25.20270961	**136.8**
85	Puna--wind erosion features	yardang	-67.4561669	-25.19909819	**153.4**
86	Puna--wind erosion features	yardang	-67.45645268	-25.19704806	**136.2**
87	Puna--wind erosion features	yardang	-67.45546567	-25.20023445	**151.4**
88	Puna--wind erosion features	yardang	-67.4552345	-25.201164	**143.4**
89	Puna--wind erosion features	yardang	-67.45655282	-25.19272749	**133.5**
90	Puna--wind erosion features	yardang	-67.45834778	-25.18588725	**126.9**
91	Puna--wind erosion features	yardang	-67.46009594	-25.18645547	**132.5**
92	Puna--wind erosion features	yardang	-67.46213838	-25.18374168	**136.2**
93	Puna--wind erosion features	yardang	-67.46283234	-25.1823961	**136.7**
94	Puna--wind erosion features	yardang	-67.4623409	-25.18225921	**136.2**
95	Puna--wind erosion features	yardang	-67.44756449	-25.18347503	**130.2**
96	Puna--wind erosion features	yardang	-67.44922634	-25.18197991	**122.4**
97	Puna--wind erosion features	yardang	-67.4501062	-25.18234153	**129.6**
98	Puna--wind erosion features	yardang	-67.44756312	-25.17924046	**128.9**
99	Puna--wind erosion features	yardang	-67.46379391	-25.18113855	**140.6**
100	Puna--wind erosion features	yardang	-67.46597591	-25.17794257	**169.1**
101	Puna--wind erosion features	yardang	-67.46482491	-25.1783486	**151.7**
102	Puna--wind erosion features	yardang	-67.46366037	-25.17931442	**134.1**
103	Puna--wind erosion features	yardang	-67.46356433	-25.17978717	**138.0**
104	Puna--wind erosion features	yardang	-67.4670058	-25.17660789	**180.1**
105	Puna--wind erosion features	yardang	-67.46650407	-25.17649144	**168.1**
106	Puna--wind erosion features	yardang	-67.46439837	-25.17685826	**154.6**
107	Puna--wind erosion features	eolian modified interfluve	-68.08666419	-26.22295202	**105.0**
108	Puna--wind erosion features	eolian modified interfluve	-68.09142965	-26.22538319	**106.9**
109	Puna--wind erosion features	eolian modified interfluve	-68.07327744	-26.22686033	**119.6**
110	Puna--wind erosion features	eolian modified interfluve	-68.06551377	-26.23136275	**108.6**
111	Puna--wind erosion features	eolian modified interfluve	-68.06380882	-26.23229548	**112.9**
112	Puna--wind erosion features	eolian modified interfluve	-68.06558045	-26.23304593	**108.5**
113	Puna--wind erosion features	eolian modified interfluve	-68.06548059	-26.2336634	**108.3**
114	Puna--wind erosion features	eolian modified interfluve	-68.06244003	-26.23120849	**109.0**
115	Puna--wind erosion features	eolian modified interfluve	-68.06392195	-26.2344795	**104.8**
116	Puna--wind erosion features	eolian modified interfluve	-68.06554931	-26.23427588	**105.2**
117	Puna--wind erosion features	eolian modified interfluve	-68.0636211	-26.23416205	**103.9**
118	Puna--wind erosion features	eolian modified interfluve	-68.06130333	-26.22769699	**111.8**
119	Puna--wind erosion features	eolian modified interfluve	-68.06529297	-26.2282673	**112.3**
120	Puna--wind erosion features	eolian modified interfluve	-68.09338012	-26.22699089	**109.6**
121	Puna--wind erosion features	eolian modified interfluve	-68.08745117	-26.22427018	**110.8**
122	Puna--wind erosion features	eolian modified interfluve	-68.06547096	-26.23077755	**111.6**
123	Puna--wind erosion features	eolian modified interfluve	-68.08762564	-26.22609957	**113.1**
124	Puna--wind erosion features	eolian modified interfluve	-68.11067563	-26.20009093	**115.2**
125	Puna--wind erosion features	eolian modified interfluve	-68.09090526	-26.20342487	**113.8**
126	Puna--wind erosion features	eolian modified interfluve	-68.1011588	-26.19962469	**124.4**

127	Puna--wind erosion features	eolian modified interfluve	-68.10891204	-26.19949725	**115.2**
128	Puna--wind erosion features	eolian modified interfluve	-68.11279615	-26.20351996	**115.9**
129	Puna--wind erosion features	eolian modified interfluve	-68.11057481	-26.20403107	**111.0**
130	Puna--wind erosion features	eolian modified interfluve	-68.11499832	-26.20427577	**111.5**
131	Puna--wind erosion features	eolian modified interfluve	-68.11477475	-26.19454184	**118.4**
132	Puna--wind erosion features	eolian modified interfluve	-68.11100899	-26.19456397	**114.2**
133	Puna--wind erosion features	eolian modified interfluve	-68.11196314	-26.19531323	**117.6**
134	Puna--wind erosion features	eolian modified interfluve	-68.10909615	-26.19640188	**113.2**
135	Puna--wind erosion features	eolian modified interfluve	-68.10668347	-26.1974364	**116.4**
136	Puna--wind erosion features	eolian modified interfluve	-68.09412987	-26.21424271	**102.7**
137	Puna--wind erosion features	eolian modified interfluve	-68.10174898	-26.2024774	**114.5**
138	Puna--wind erosion features	eolian modified interfluve	-68.12676849	-26.41547852	**102.5**
139	Puna--wind erosion features	eolian modified interfluve	-68.13293059	-26.41299585	**99.1**
140	Puna--wind erosion features	eolian modified interfluve	-68.12444996	-26.41168551	**106.5**
141	Puna--wind erosion features	eolian modified interfluve	-68.12169885	-26.41124319	**101.6**
142	Puna--wind erosion features	eolian modified interfluve	-68.12055282	-26.41740551	**110.5**
143	Puna--wind erosion features	eolian modified interfluve	-68.12245238	-26.41434599	**103.8**
144	Puna--wind erosion features	eolian modified interfluve	-68.13034898	-26.41306976	**103.5**
145	Puna--wind erosion features	eolian modified interfluve	-68.0958232	-26.25033957	**114.9**
146	Puna--wind erosion features	eolian modified interfluve	-68.09810791	-26.25190752	**108.9**
147	Puna--wind erosion features	eolian modified interfluve	-68.08704341	-26.2557977	**103.4**
148	Puna--wind erosion features	eolian modified interfluve	-68.09283981	-26.24806119	**108.8**
149	Puna--wind erosion features	eolian modified interfluve	-68.08660695	-26.25225936	**106.0**
150	Puna--wind erosion features	eolian modified interfluve	-68.07674361	-26.26254495	**107.2**
151	Puna--wind erosion features	eolian modified interfluve	-68.07637361	-26.26638152	**105.9**
152	Puna--wind erosion features	eolian modified interfluve	-68.09608558	-26.22951746	**113.9**
153	Puna--wind erosion features	eolian modified interfluve	-68.0952468	-26.23193599	**111.2**
154	Puna--wind erosion features	eolian modified interfluve	-68.13177817	-26.40113962	**111.6**
155	Puna--wind erosion features	eolian modified interfluve	-68.12465816	-26.40356961	**110.1**
156	Puna--wind erosion features	eolian modified interfluve	-68.12317483	-26.40494045	**110.4**
157	Puna--wind erosion features	eolian modified interfluve	-68.11991522	-26.40901021	**109.5**
158	Puna--wind erosion features	eolian modified interfluve	-68.13316417	-26.40857951	**104.3**
159	Puna--wind erosion features	eolian modified interfluve	-68.13405938	-26.41335216	**103.1**
160	Puna--wind erosion features	eolian modified interfluve	-68.1217953	-26.39981467	**102.0**
161	Puna--wind erosion features	eolian modified interfluve	-68.13503718	-26.40562961	**108.9**
162	Puna--wind erosion features	eolian modified interfluve	-68.12570953	-26.40512551	**109.3**
163	Puna--wind erosion features	yardang	-68.11658637	-26.18456573	**107.5**
164	Puna--wind erosion features	wind streak	-67.52194794	-26.66666505	**133.8**
165	Puna--wind erosion features	yardang	-67.57964735	-26.73782798	**119.7**
166	Puna--wind erosion features	yardang	-67.57169158	-26.74083867	**140.5**
167	Puna--wind erosion features	yardang	-67.57163169	-26.73556227	**131.1**
168	Puna--wind erosion features	yardang	-67.5718628	-26.7317863	**126.0**
169	Puna--wind erosion features	yardang	-67.57189977	-26.73107756	**125.0**
170	Puna--wind erosion features	yardang	-67.57198899	-26.73278801	**132.9**
171	Puna--wind erosion features	yardang	-67.56730404	-26.74093835	**139.6**
172	Puna--wind erosion features	yardang	-67.59135234	-26.73006399	**127.5**
173	Puna--wind erosion features	yardang	-67.58881228	-26.73052134	**135.9**
174	Puna--wind erosion features	yardang	-67.58389908	-26.73304779	**130.0**
175	Puna--wind erosion features	yardang	-67.58031846	-26.73385899	**135.8**
176	Puna--wind erosion features	yardang	-67.58338846	-26.73592615	**128.3**
177	Puna--wind erosion features	yardang	-67.58040239	-26.73217144	**121.1**
178	Puna--wind erosion features	yardang	-67.58842477	-26.72570014	**123.2**
179	Puna--wind erosion features	yardang	-67.58793076	-26.72339827	**127.8**
180	Puna--wind erosion features	yardang	-67.57909618	-26.72301114	**130.6**
181	Puna--wind erosion features	yardang	-67.57973637	-26.72384314	**128.8**
182	Puna--wind erosion features	yardang	-67.57949902	-26.72177081	**131.3**
183	Puna--wind erosion features	yardang	-67.57876388	-26.72168431	**134.3**
184	Puna--wind erosion features	yardang	-67.57974521	-26.72034565	**132.3**
185	Puna--wind erosion features	yardang	-67.57985607	-26.71983337	**135.5**
186	Puna--wind erosion features	yardang	-67.57963822	-26.71873475	**121.5**
187	Puna--wind erosion features	yardang	-67.57892543	-26.71751455	**118.3**
188	Puna--wind erosion features	yardang	-67.57894085	-26.71789504	**118.3**
189	Puna--wind erosion features	yardang	-67.58744115	-26.71744455	**136.0**
190	Puna--wind erosion features	yardang	-67.58695866	-26.71458118	**127.5**

191	Puna--wind erosion features	yardang	-67.58411816	-26.71502903	**117.0**
192	Puna--wind erosion features	yardang	-67.58273643	-26.71416667	**117.8**
193	Puna--wind erosion features	yardang	-67.59073316	-26.71336961	**125.1**
194	Puna--wind erosion features	yardang	-67.58904196	-26.71673996	**127.6**
195	Puna--wind erosion features	yardang	-67.5883602	-26.71724922	**127.2**
196	Puna--wind erosion features	yardang	-67.58461861	-26.70959168	**127.2**
197	Puna--wind erosion features	yardang	-67.5859292	-26.70732409	**123.4**
198	Puna--wind erosion features	yardang	-67.58524354	-26.70598599	**124.1**
199	Puna--wind erosion features	yardang	-67.58486774	-26.70601534	**116.9**
200	Puna--wind erosion features	yardang	-67.58203914	-26.70573846	**128.4**
201	Puna--wind erosion features	yardang	-67.58064254	-26.70624335	**128.4**
202	Puna--wind erosion features	yardang	-67.57758868	-26.70576355	**125.7**
203	Puna--wind erosion features	yardang	-67.57112899	-26.7016596	**126.4**
204	Puna--wind erosion features	yardang	-67.58211289	-26.69977958	**124.4**
205	Puna--wind erosion features	yardang	-67.58039546	-26.70535555	**131.9**
206	Puna--wind erosion features	yardang	-67.57086104	-26.69492833	**123.6**
207	Puna--wind erosion features	yardang	-67.55213818	-26.67896493	**130.6**
208	Puna--wind erosion features	yardang	-67.55229066	-26.67821785	**133.3**
209	Puna--wind erosion features	yardang	-67.55428036	-26.67929917	**138.4**
210	Puna--wind erosion features	yardang	-67.54872803	-26.66952722	**129.3**
211	Puna--wind erosion features	yardang	-67.53275474	-26.67187962	**130.2**
212	Puna--wind erosion features	yardang	-67.52987415	-26.66976375	**121.1**
213	Puna--wind erosion features	yardang	-67.5295096	-26.67082989	**128.3**
214	Puna--wind erosion features	yardang	-67.48426321	-26.62838292	**118.3**
215	Puna--wind erosion features	yardang	-67.48537499	-26.62746956	**121.3**
216	Puna--wind erosion features	yardang	-67.48388133	-26.62574844	**133.3**
217	Puna--wind erosion features	yardang	-67.58828571	-24.75979232	**121.6**
218	Puna--wind erosion features	yardang	-67.58430525	-24.76076621	**120.2**
219	Puna--wind erosion features	yardang	-67.5851468	-24.7615501	**112.9**
220	Puna--wind erosion features	yardang	-67.57967437	-24.76050662	**120.6**
221	Puna--wind erosion features	yardang	-67.58072842	-24.7615712	**124.4**
222	Puna--wind erosion features	yardang	-67.58108795	-24.76035132	**116.9**
223	Puna--wind erosion features	yardang	-67.57188814	-24.76245492	**124.4**
224	Puna--wind erosion features	yardang	-67.57689823	-24.76593148	**127.9**
225	Puna--wind erosion features	yardang	-67.57762563	-24.76748266	**125.3**
226	Puna--wind erosion features	yardang	-67.57805596	-24.76772158	**123.3**
227	Puna--wind erosion features	yardang	-67.57151563	-24.76362507	**125.6**
228	Puna--wind erosion features	yardang	-67.56969631	-24.76523707	**120.3**
229	Puna--wind erosion features	yardang	-67.56998677	-24.76542228	**128.0**
230	Puna--wind erosion features	yardang	-67.56931314	-24.76569856	**119.8**
231	Puna--wind erosion features	yardang	-67.56858798	-24.76543927	**121.2**
232	Puna--wind erosion features	yardang	-67.57036719	-24.76447691	**122.5**
233	Puna--wind erosion features	yardang	-67.56867019	-24.76832486	**119.3**
234	Puna--wind erosion features	yardang	-67.56892393	-24.7679243	**119.8**
235	Puna--wind erosion features	yardang	-67.57148912	-24.76967931	**129.2**
236	Puna--wind erosion features	yardang	-67.57385075	-24.7701142	**127.1**
237	Puna--wind erosion features	yardang	-67.57150283	-24.77110914	**130.8**
238	Puna--wind erosion features	yardang	-67.57020434	-24.77114773	**125.1**
239	Puna--wind erosion features	yardang	-67.56787716	-24.77147166	**124.1**
240	Puna--wind erosion features	yardang	-67.56444648	-24.76925757	**127.8**
241	Puna--wind erosion features	yardang	-67.56786699	-24.7727662	**126.2**
242	Puna--wind erosion features	yardang	-67.56665025	-24.77353522	**122.4**
243	Puna--wind erosion features	yardang	-67.56388818	-24.77423004	**121.0**
244	Puna--wind erosion features	yardang	-67.56770777	-24.77362659	**125.0**
245	Puna--wind erosion features	yardang	-67.56948486	-24.77314079	**124.3**
246	Puna--wind erosion features	yardang	-67.5700065	-24.77321203	**125.1**
247	Puna--wind erosion features	yardang	-67.56575376	-24.77247128	**122.3**
248	Puna--wind erosion features	yardang	-67.56425943	-24.77030369	**126.3**
249	Puna--wind erosion features	yardang	-67.56561293	-24.77546718	**125.4**
250	Puna--wind erosion features	yardang	-67.56095277	-24.77675197	**123.0**
251	Puna--wind erosion features	yardang	-67.56683932	-24.77592777	**126.4**
252	Puna--wind erosion features	yardang	-67.56745196	-24.77763327	**123.3**
253	Puna--wind erosion features	yardang	-67.56673793	-24.77769736	**122.7**
254	Puna--wind erosion features	yardang	-67.56715036	-24.77643699	**128.0**

255	Puna--wind erosion features	yardang	-67.56806613	-24.77503799	**118.9**
256	Puna--wind erosion features	yardang	-67.56813662	-24.77566152	**123.5**
257	Puna--wind erosion features	yardang	-67.56460195	-24.77618946	**120.4**
258	Puna--wind erosion features	yardang	-67.56462717	-24.77713975	**123.8**
259	Puna--wind erosion features	yardang	-67.56133703	-24.77695311	**122.9**
260	Puna--wind erosion features	yardang	-67.56387672	-24.77977277	**121.8**
261	Puna--wind erosion features	yardang	-67.56347908	-24.77960585	**126.2**
262	Puna--wind erosion features	yardang	-67.56783515	-24.77956181	**123.9**
263	Puna--wind erosion features	yardang	-67.56763009	-24.77805631	**127.1**
264	Puna--wind erosion features	yardang	-67.56629035	-24.77528996	**123.8**
265	Puna--wind erosion features	yardang	-67.56413317	-24.77797067	**123.8**
266	Puna--wind erosion features	yardang	-67.56379464	-24.7756466	**129.7**
267	Puna--wind erosion features	yardang	-67.56344591	-24.77667075	**124.4**
268	Puna--wind erosion features	yardang	-67.56320757	-24.77609696	**124.2**
269	Puna--wind erosion features	yardang	-67.56185009	-24.7751575	**124.3**
270	Puna--wind erosion features	yardang	-67.56387618	-24.77839665	**121.7**
271	Puna--wind erosion features	yardang	-67.56346515	-24.77803723	**127.8**
272	Puna--wind erosion features	yardang	-67.56998343	-24.78124153	**124.3**
273	Puna--wind erosion features	yardang	-67.56793798	-24.78241309	**121.2**
274	Puna--wind erosion features	yardang	-67.56985495	-24.78232037	**119.1**
275	Puna--wind erosion features	yardang	-67.57263219	-24.78506748	**126.0**
276	Puna--wind erosion features	yardang	-67.56466229	-24.78373399	**123.6**
277	Puna--wind erosion features	yardang	-67.57117089	-24.7871304	**120.0**
278	Puna--wind erosion features	yardang	-67.57014382	-24.78367307	**120.5**
279	Puna--wind erosion features	yardang	-67.56841127	-24.78294247	**125.4**
280	Puna--wind erosion features	yardang	-67.56834975	-24.78402722	**127.3**
281	Puna--wind erosion features	yardang	-67.56840666	-24.78470168	**127.8**
282	Puna--wind erosion features	yardang	-67.56468048	-24.78337979	**118.2**
283	Puna--wind erosion features	yardang	-67.56407782	-24.78178052	**121.8**
284	Puna--wind erosion features	yardang	-67.56560876	-24.78589079	**118.3**
285	Puna--wind erosion features	yardang	-67.56578687	-24.78502448	**124.5**
286	Puna--wind erosion features	yardang	-67.57130729	-24.78883902	**114.2**
287	Puna--wind erosion features	yardang	-67.57369862	-24.79082617	**121.8**
288	Puna--wind erosion features	yardang	-67.56848917	-24.78972147	**122.8**
289	Puna--wind erosion features	yardang	-67.56691978	-24.79036177	**119.7**
290	Puna--wind erosion features	yardang	-67.56860506	-24.79152465	**124.3**
291	Puna--wind erosion features	yardang	-67.56895976	-24.79164549	**119.9**
292	Puna--wind erosion features	yardang	-67.57138502	-24.7897581	**121.1**
293	Puna--wind erosion features	yardang	-67.57569451	-24.79544035	**119.4**
294	Puna--wind erosion features	yardang	-67.57586126	-24.79569853	**104.8**
295	Puna--wind erosion features	yardang	-67.53346772	-24.84731249	**108.6**
296	Puna--wind erosion features	yardang	-67.53744073	-24.84900607	**120.6**
297	Puna--wind erosion features	yardang	-67.53604662	-24.84957808	**112.3**
298	Puna--wind erosion features	yardang	-67.53441987	-24.84795352	**122.3**
299	Puna--wind erosion features	yardang	-67.53360081	-24.84863317	**108.6**
300	Puna--wind erosion features	yardang	-67.53953501	-24.84775779	**116.4**
301	Puna--wind erosion features	yardang	-67.53801707	-24.84780779	**122.8**
302	Puna--wind erosion features	yardang	-67.53263577	-24.84922961	**110.2**
303	Puna--wind erosion features	yardang	-67.5321677	-24.85116199	**110.6**
304	Puna--wind erosion features	yardang	-67.53289918	-24.85344172	**139.2**
305	Puna--wind erosion features	yardang	-67.52615952	-24.85509182	**112.4**
306	Puna--wind erosion features	yardang	-67.53356537	-24.85285779	**126.7**
307	Puna--wind erosion features	yardang	-67.53512265	-24.853752	**125.9**
308	Puna--wind erosion features	yardang	-67.52672582	-24.85334895	**109.6**
309	Puna--wind erosion features	yardang	-67.52830572	-24.85508263	**119.3**
310	Puna--wind erosion features	yardang	-67.52935015	-24.85284847	**111.3**
311	Puna--wind erosion features	yardang	-67.52917031	-24.85634736	**115.6**
312	Puna--wind erosion features	yardang	-67.53336097	-24.85478861	**117.1**
313	Puna--wind erosion features	yardang	-67.52452303	-24.85358467	**114.0**
314	Puna--wind erosion features	yardang	-67.52592269	-24.85317192	**114.9**
315	Puna--wind erosion features	yardang	-67.52712468	-24.85603605	**130.8**
316	Puna--wind erosion features	yardang	-67.51061291	-24.89080749	**134.1**
317	Puna--wind erosion features	yardang	-67.51552824	-24.89284861	**117.2**
318	Puna--wind erosion features	yardang	-67.51173863	-24.89529289	**137.8**

319	Puna--wind erosion features	yardang	-67.51364526	-24.89484786	**129.9**
320	Puna--wind erosion features	yardang	-67.50880782	-24.89435955	**123.1**
321	Puna--wind erosion features	yardang	-67.50876975	-24.89595402	**131.8**
322	Puna--wind erosion features	yardang	-67.50817203	-24.89234675	**130.8**
323	Puna--wind erosion features	yardang	-67.50941049	-24.89727154	**126.8**
324	Puna--wind erosion features	yardang	-67.51188113	-24.8970483	**131.1**
325	Puna--wind erosion features	yardang	-67.50769868	-24.89361383	**143.8**
326	Puna--wind erosion features	yardang	-67.50629148	-24.89420406	**132.9**
327	Puna--wind erosion features	yardang	-67.50647806	-24.89468483	**132.0**
328	Puna--wind erosion features	yardang	-67.50904064	-24.89495165	**130.0**
329	Puna--wind erosion features	yardang	-67.5169117	-24.88960366	**139.8**
330	Puna--wind erosion features	yardang	-67.51556169	-24.88925379	**130.5**
331	Puna--wind erosion features	yardang	-67.51277013	-24.8847162	**126.8**
332	Puna--wind erosion features	yardang	-67.52088278	-24.91719385	**119.9**
333	Puna--wind erosion features	yardang	-67.52237393	-24.91935507	**127.7**
334	Puna--wind erosion features	yardang	-67.52244038	-24.91683555	**134.6**
335	Puna--wind erosion features	yardang	-67.52292743	-24.91508856	**134.5**
336	Puna--wind erosion features	yardang	-67.52067672	-24.91546361	**130.3**
337	Puna--wind erosion features	yardang	-67.51853707	-24.92381193	**129.8**
338	Puna--wind erosion features	yardang	-67.52329751	-24.92271445	**142.4**
339	Puna--wind erosion features	yardang	-67.51966735	-24.92537878	**122.3**
340	Puna--wind erosion features	yardang	-67.51827777	-24.92645034	**125.1**
341	Puna--wind erosion features	yardang	-67.51827205	-24.92732682	**121.5**
342	Puna--wind erosion features	yardang	-67.52148543	-24.92295051	**116.6**
343	Puna--wind erosion features	yardang	-67.3754684	-24.62010495	**152.6**
344	Puna--wind erosion features	yardang	-67.37512857	-24.62045124	**149.5**
345	Puna--wind erosion features	yardang	-67.37541783	-24.62054179	**148.9**
346	Puna--wind erosion features	yardang	-67.37543749	-24.62081073	**142.8**
347	Puna--wind erosion features	wind streak	-67.28057854	-26.71239054	**134.2**
348	Puna--wind erosion features	wind streak	-67.33899119	-26.6551191	**132.4**
349	Puna--wind erosion features	wind streak	-67.15342073	-26.88295671	**147.5**
350	Puna--wind erosion features	wind streak	-67.28404814	-26.85201706	**139.4**
351	Puna--wind erosion features	wind streak	-67.1456984	-26.82492492	**140.6**
352	Puna--wind erosion features	wind streak	-67.1004962	-26.87761528	**130.6**
353	Puna--wind erosion features	wind streak	-67.33101559	-26.79304223	**137.5**
354	Puna--wind erosion features	wind streak	-67.3233108	-26.89019308	**138.5**
355	Puna--wind erosion features	wind streak	-67.16704692	-26.94504747	**135.2**
356	Puna--wind erosion features	wind streak	-67.23255756	-27.06717595	**140.1**
357	Puna--wind erosion features	wind streak	-68.14655876	-27.00455614	**103.2**
358	Puna--wind erosion features	eolian modified interfluve	-68.37970581	-26.29725028	**119.7**
359	Puna--wind erosion features	eolian modified interfluve	-68.38155534	-26.29760326	**118.7**
360	Puna--wind erosion features	eolian modified interfluve	-68.38172412	-26.29519638	**122.8**
361	Puna--wind erosion features	eolian modified interfluve	-68.37992635	-26.30655506	**125.1**
362	Puna--wind erosion features	eolian modified interfluve	-68.37822505	-26.29491522	**121.5**
363	Puna--wind erosion features	eolian modified interfluve	-68.37170015	-26.29479604	**117.8**
364	Puna--wind erosion features	eolian modified interfluve	-68.3707206	-26.29932672	**122.4**
365	Puna--wind erosion features	eolian modified interfluve	-68.3666206	-26.30023898	**120.8**
366	Puna--wind erosion features	eolian modified interfluve	-68.3772455	-26.30388304	**125.8**
367	Puna--wind erosion features	eolian modified interfluve	-68.37294771	-26.30611173	**127.2**
368	Puna--wind erosion features	eolian modified interfluve	-68.37139711	-26.2962727	**125.9**
369	Puna--wind erosion features	eolian modified interfluve	-68.38518055	-26.29711724	**124.8**
370	Puna--wind erosion features	eolian modified interfluve	-68.36986115	-26.30849181	**126.3**
371	Puna--wind erosion features	eolian modified interfluve	-68.3673668	-26.30371808	**121.4**
372	Puna--wind erosion features	eolian modified interfluve	-68.36842004	-26.30097735	**118.5**
373	Puna--wind erosion features	eolian modified interfluve	-68.3803262	-26.30781823	**124.6**
374	Puna--wind erosion features	eolian modified interfluve	-68.37670659	-26.31071752	**121.7**
375	Puna--wind erosion features	eolian modified interfluve	-68.3830457	-26.30904379	**121.4**
376	Puna--wind erosion features	eolian modified interfluve	-68.37094503	-26.30271502	**123.1**
377	Puna--wind erosion features	eolian modified interfluve	-68.39173913	-26.30708052	**127.0**
378	Puna--wind erosion features	eolian modified interfluve	-68.38848042	-26.30892603	**125.0**
379	Puna--wind erosion features	eolian modified interfluve	-68.38726572	-26.31246038	**118.8**
380	Puna--wind erosion features	eolian modified interfluve	-68.38583191	-26.31196427	**123.4**
381	Puna--wind erosion features	eolian modified interfluve	-68.32975573	-26.33503335	**127.8**
382	Puna--wind erosion features	eolian modified interfluve	-68.35167139	-26.31797473	**126.2**

383	Puna--wind erosion features	eolian modified interfluve	-68.33283644	-26.32528207	**130.7**
384	Puna--wind erosion features	eolian modified interfluve	-68.31928264	-26.33614402	**128.9**
385	Puna--wind erosion features	eolian modified interfluve	-68.42678787	-26.30141369	**121.8**
386	Puna--wind erosion features	eolian modified interfluve	-68.42599541	-26.29710843	**120.0**
387	Puna--wind erosion features	eolian modified interfluve	-68.42406247	-26.29844642	**122.2**
388	Puna--wind erosion features	eolian modified interfluve	-68.42205452	-26.29961281	**120.9**
389	Puna--wind erosion features	eolian modified interfluve	-68.42487783	-26.30355579	**127.1**
390	Puna--wind erosion features	eolian modified interfluve	-68.42293774	-26.30430706	**122.9**
391	Puna--wind erosion features	eolian modified interfluve	-68.41095893	-26.30939877	**124.7**
392	Puna--wind erosion features	eolian modified interfluve	-68.40941874	-26.30947487	**120.9**
393	Puna--wind erosion features	eolian modified interfluve	-68.40681748	-26.30486565	**117.1**
394	Puna--wind erosion features	eolian modified interfluve	-68.42187383	-26.29714884	**112.7**
395	Puna--wind erosion features	eolian modified interfluve	-68.41772945	-26.29549181	**124.5**
396	Puna--wind erosion features	eolian modified interfluve	-68.41400741	-26.29783767	**125.9**
397	Puna--wind erosion features	eolian modified interfluve	-68.41903336	-26.29719335	**119.6**
398	Puna--wind erosion features	eolian modified interfluve	-68.42184419	-26.30263076	**122.2**
399	Puna--wind erosion features	eolian modified interfluve	-68.4189553	-26.30379311	**124.5**
400	Puna--wind erosion features	eolian modified interfluve	-68.41348387	-26.31345558	**121.7**
401	Puna--wind erosion features	eolian modified interfluve	-68.4124718	-26.31492944	**126.5**
402	Puna--wind erosion features	eolian modified interfluve	-68.4108299	-26.31285026	**125.5**
403	Puna--wind erosion features	eolian modified interfluve	-68.41034235	-26.31146858	**120.8**
404	Puna--wind erosion features	eolian modified interfluve	-68.40862144	-26.30692433	**124.7**
405	Puna--wind erosion features	eolian modified interfluve	-68.41495777	-26.31085504	**122.9**
406	Puna--wind erosion features	eolian modified interfluve	-68.0902589	-26.2102532	**114.5**
407	Puna--wind erosion features	eolian modified interfluve	-68.090357	-26.21795314	**103.5**
408	Puna--wind erosion features	eolian modified interfluve	-68.09370544	-26.22045203	**104.7**
409	Puna--wind erosion features	eolian modified interfluve	-68.09043718	-26.22098004	**110.2**
410	Puna--wind erosion features	eolian modified interfluve	-68.08299209	-26.2322414	**115.3**
411	Puna--wind erosion features	eolian modified interfluve	-68.06548894	-26.23464261	**108.7**
412	Puna--wind erosion features	eolian modified interfluve	-68.07343066	-26.23662737	**109.1**
413	Puna--wind erosion features	eolian modified interfluve	-68.07359666	-26.23716832	**107.1**
414	Puna--wind erosion features	eolian modified interfluve	-68.06428455	-26.23724917	**108.8**
415	Puna--wind erosion features	eolian modified interfluve	-68.07311152	-26.24408244	**107.3**
416	Puna--wind erosion features	eolian modified interfluve	-68.06648982	-26.24203028	**107.9**
417	Puna--wind erosion features	eolian modified interfluve	-68.06381664	-26.23612705	**105.0**
418	Puna--wind erosion features	eolian modified interfluve	-68.06123272	-26.2263335	**117.2**
419	Puna--wind erosion features	eolian modified interfluve	-68.07079585	-26.22273328	**105.8**
420	Puna--wind erosion features	eolian modified interfluve	-68.09988109	-26.20624186	**110.2**
421	Puna--wind erosion features	eolian modified interfluve	-68.11075923	-26.19706247	**114.9**
422	Puna--wind erosion features	eolian modified interfluve	-68.10806087	-26.19757568	**113.1**
423	Puna--wind erosion features	yardang	-68.11575116	-26.19234656	**111.0**
424	Puna--wind erosion features	yardang	-67.46974166	-25.09558414	**140.6**
425	Puna--wind erosion features	yardang	-67.46894866	-25.09604414	**132.2**
426	Puna--wind erosion features	yardang	-67.46860881	-25.09695874	**130.9**
427	Puna--wind erosion features	yardang	-67.46664469	-25.09903659	**123.5**
428	Puna--wind erosion features	yardang	-67.46530864	-25.10011572	**127.6**
429	Puna--wind erosion features	yardang	-67.46341916	-25.1008708	**129.9**
430	Puna--wind erosion features	yardang	-67.46433517	-25.09923586	**127.5**
431	Puna--wind erosion features	yardang	-67.46000478	-25.10252749	**110.9**
432	Puna--wind erosion features	yardang	-67.45871921	-25.0887336	**126.8**
433	Puna--wind erosion features	yardang	-67.4589981	-25.08888546	**129.7**
434	Puna--wind erosion features	yardang	-67.45919087	-25.08910178	**127.9**
435	Puna--wind erosion features	wind streak	-69.08195322	-26.24051581	**135.2**
436	Puna--wind erosion features	wind streak	-69.09793752	-26.25717774	**140.9**
437	Puna--wind erosion features	wind streak	-69.11148175	-26.2707291	**137.6**
438	Puna--wind erosion features	yardang	-68.3720324	-25.70983989	**152.6**
439	Puna--wind erosion features	yardang	-68.36204094	-25.71079339	**141.2**
440	Puna--wind erosion features	yardang	-68.36081964	-25.71308698	**139.6**
441	Puna--wind erosion features	yardang	-68.34429191	-25.71747041	**142.6**
442	Puna--wind erosion features	yardang	-68.34513991	-25.71906901	**141.2**
443	Puna--wind erosion features	yardang	-68.33900317	-25.73679294	**142.0**
444	Puna--wind erosion features	yardang	-68.33087463	-25.65698646	**194.2**
445	Puna--wind erosion features	yardang	-68.32959314	-25.6575146	**192.3**
446	Puna--wind erosion features	yardang	-67.52223119	-24.91003958	**140.3**

447	Puna--wind erosion features	yardang	-67.51781529	-24.9103182	**143.2**
448	Puna--wind erosion features	yardang	-67.51989852	-24.907052	**128.3**
449	Puna--wind erosion features	yardang	-67.51897658	-24.90786821	**142.7**
450	Puna--wind erosion features	yardang	-67.51642324	-24.90826354	**132.7**
451	Puna--wind erosion features	yardang	-67.5200577	-24.9119081	**129.0**
452	Puna--wind erosion features	yardang	-67.5259971	-24.91120429	**131.1**
453	Puna--wind erosion features	yardang	-67.51733344	-24.92184751	**133.4**
454	Puna--wind erosion features	yardang	-67.51797063	-24.92182307	**138.0**
455	Puna--wind erosion features	yardang	-67.52402121	-24.90523842	**136.3**
456	Puna--wind erosion features	yardang	-67.5226934	-24.903449	**129.9**
457	Puna--wind erosion features	yardang	-67.51984061	-24.90054109	**133.1**
458	Puna--wind erosion features	yardang	-67.52230993	-24.90538268	**132.4**
459	Puna--wind erosion features	yardang	-67.52155263	-24.90590664	**145.1**
460	Puna--wind erosion features	yardang	-67.51781237	-24.8967503	**129.3**
461	Puna--wind erosion features	yardang	-67.51920636	-24.89733705	**126.8**
462	Puna--wind erosion features	yardang	-67.51566109	-24.89644504	**133.4**
463	Puna--wind erosion features	yardang	-67.51698539	-24.89910557	**137.9**
464	Puna--wind erosion features	yardang	-67.5162335	-24.90030806	**133.4**
465	Puna--wind erosion features	yardang	-67.50687228	-24.89560736	**132.5**
466	Puna--wind erosion features	yardang	-67.51112976	-24.89222239	**129.8**
467	Puna--wind erosion features	yardang	-67.51091303	-24.89137789	**121.8**
468	Puna--wind erosion features	yardang	-67.50837601	-24.89129676	**138.1**
469	Puna--wind erosion features	yardang	-67.50923558	-24.89599311	**153.5**
470	Puna--wind erosion features	yardang	-67.51394567	-24.89427182	**131.1**
471	Puna--wind erosion features	yardang	-67.51414065	-24.89430314	**133.6**
472	Puna--wind erosion features	yardang	-67.51453352	-24.89420903	**135.5**
473	Puna--wind erosion features	yardang	-67.51476427	-24.89425197	**132.6**
474	Puna--wind erosion features	yardang	-67.51750434	-24.88819714	**138.9**
475	Puna--wind erosion features	yardang	-67.51737239	-24.88895149	**129.1**
476	Puna--wind erosion features	yardang	-67.51642726	-24.88961888	**133.0**
477	Puna--wind erosion features	yardang	-67.51673513	-24.88941156	**128.9**
478	Puna--wind erosion features	yardang	-67.52085457	-24.87291514	**127.5**
479	Puna--wind erosion features	yardang	-67.52168068	-24.87305995	**118.7**
480	Puna--wind erosion features	yardang	-67.52066613	-24.87409746	**129.5**
481	Puna--wind erosion features	yardang	-67.51552274	-24.87230862	**128.9**
482	Puna--wind erosion features	yardang	-67.51674833	-24.87258764	**124.3**
483	Puna--wind erosion features	wind streak	-67.00534444	-24.94294292	**128.1**
484	Puna--wind erosion features	wind streak	-66.9043959	-24.74203263	**125.1**
485	Puna--wind erosion features	yardang	-67.51679254	-24.87276183	**128.7**
486	Puna--wind erosion features	wind streak	-67.13307104	-23.8459766	**121.2**
487	Puna--wind erosion features	wind streak	-67.33032714	-23.70400183	**116.2**
488	Puna--wind erosion features	wind streak	-66.79669736	-23.55238934	**109.1**
489	Puna--wind erosion features	wind streak	-67.31631201	-23.68911089	**118.5**
490	Puna--wind erosion features	wind streak	-67.10571844	-23.46354858	**107.1**
491	Puna--wind erosion features	wind streak	-67.12249572	-23.10701006	**117.1**
492	Puna--wind erosion features	eolian modified interfluve	-67.24640509	-23.26702138	**109.4**
493	Puna--wind erosion features	eolian modified interfluve	-67.25061228	-23.26179952	**105.5**
494	Puna--wind erosion features	eolian modified interfluve	-67.25493465	-23.2722186	**120.2**
495	Puna--wind erosion features	eolian modified interfluve	-67.25001786	-23.25695474	**107.9**
496	Puna--wind erosion features	eolian modified interfluve	-67.25117942	-23.27098188	**109.1**
497	Puna--wind erosion features	eolian modified interfluve	-67.24309068	-23.23366036	**126.6**
498	Puna--wind erosion features	eolian modified interfluve	-67.25532402	-23.27405299	**118.7**
499	Puna--wind erosion features	eolian modified interfluve	-67.22554766	-23.24692534	**107.1**
500	Puna--wind erosion features	eolian modified interfluve	-67.1320997	-23.30946509	**112.6**
501	Puna--wind erosion features	eolian modified interfluve	-67.12705279	-23.32465346	**110.6**
502	Puna--wind erosion features	eolian modified interfluve	-67.13429905	-23.34940777	**105.0**
503	Puna--wind erosion features	eolian modified interfluve	-67.13288277	-23.35616354	**108.4**
504	Puna--wind erosion features	eolian modified interfluve	-67.13226002	-23.35762057	**110.0**
505	Puna--wind erosion features	eolian modified interfluve	-67.2818457	-23.57507464	**106.2**
506	Puna--wind erosion features	eolian modified interfluve	-67.28339399	-23.57263694	**106.6**
507	Puna--wind erosion features	eolian modified interfluve	-67.28745026	-23.57463539	**106.6**
508	Puna--wind erosion features	eolian modified interfluve	-67.27024668	-23.57787943	**108.0**
509	Puna--wind erosion features	eolian modified interfluve	-67.28490613	-23.57910815	**109.0**
510	Puna--wind erosion features	eolian modified interfluve	-67.28423693	-23.58056476	**109.5**

511	Puna--wind erosion features	eolian modified interfluve	-67.26232277	-23.59022177	**108.1**
512	Puna--wind erosion features	eolian modified interfluve	-67.24785544	-23.59747441	**106.8**
513	Puna--wind erosion features	eolian modified interfluve	-67.24588405	-23.6022159	**106.6**
514	Puna--wind erosion features	eolian modified interfluve	-67.24795274	-23.59462772	**107.7**
515	Puna--wind erosion features	eolian modified interfluve	-67.25760645	-23.58915396	**104.0**
516	Puna--wind erosion features	eolian modified interfluve	-67.25528099	-23.58428852	**106.0**
517	Puna--wind erosion features	eolian modified interfluve	-67.26684431	-23.57587484	**107.2**
518	Puna--wind erosion features	eolian modified interfluve	-67.27633618	-23.5807246	**111.6**
519	Puna--wind erosion features	eolian modified interfluve	-67.28096238	-23.58407274	**110.9**
520	Puna--wind erosion features	eolian modified interfluve	-67.28562273	-23.58205963	**110.6**
521	Puna--wind erosion features	eolian modified interfluve	-67.28427132	-23.57109758	**104.8**
522	Puna--wind erosion features	eolian modified interfluve	-67.28331227	-23.56945615	**106.6**
523	Puna--wind erosion features	eolian modified interfluve	-67.27852088	-23.57718251	**109.9**
524	Puna--wind erosion features	eolian modified interfluve	-67.27518732	-23.75336395	**119.7**
525	Puna--wind erosion features	eolian modified interfluve	-67.27350704	-23.77091504	**121.4**
526	Puna--wind erosion features	eolian modified interfluve	-67.27319958	-23.772777	**120.8**
527	Puna--wind erosion features	eolian modified interfluve	-67.2730889	-23.76978117	**121.4**
528	Puna--wind erosion features	eolian modified interfluve	-67.26692383	-23.76544491	**114.4**
529	Puna--wind erosion features	eolian modified interfluve	-67.25962301	-23.76456922	**115.9**
530	Puna--wind erosion features	eolian modified interfluve	-67.25182407	-23.76514498	**111.8**
531	Puna--wind erosion features	eolian modified interfluve	-67.2559341	-23.76133648	**113.0**
532	Puna--wind erosion features	eolian modified interfluve	-67.25874532	-23.76250023	**122.1**
533	Puna--wind erosion features	eolian modified interfluve	-67.28044843	-23.75666567	**113.4**
534	Puna--wind erosion features	eolian modified interfluve	-67.27620228	-23.7560812	**113.3**
535	Puna--wind erosion features	eolian modified interfluve	-67.28311916	-23.75407544	**109.2**
536	Puna--wind erosion features	eolian modified interfluve	-67.27887651	-23.76809127	**123.5**
537	Puna--wind erosion features	eolian modified interfluve	-67.27546338	-23.76876988	**116.2**
538	Puna--wind erosion features	eolian modified interfluve	-67.27038787	-23.76190801	**112.4**
539	Puna--wind erosion features	eolian modified interfluve	-67.2719281	-23.76922373	**115.5**
540	Puna--wind erosion features	eolian modified interfluve	-67.27447608	-23.74169527	**112.2**
541	Puna--wind erosion features	eolian modified interfluve	-67.17467509	-23.87647421	**113.1**
542	Puna--wind erosion features	eolian modified interfluve	-67.17388629	-23.87540966	**111.0**
543	Puna--wind erosion features	eolian modified interfluve	-67.17456567	-23.88183529	**113.8**
544	Puna--wind erosion features	eolian modified interfluve	-67.17155981	-23.88679957	**110.3**
545	Puna--wind erosion features	eolian modified interfluve	-67.17361898	-23.89850136	**118.7**
546	Puna--wind erosion features	eolian modified interfluve	-67.17464426	-23.88400507	**111.4**
547	Puna--wind erosion features	eolian modified interfluve	-67.17570826	-23.86852935	**114.1**
548	Puna--wind erosion features	eolian modified interfluve	-67.17531189	-23.89555554	**118.1**
549	Puna--wind erosion features	eolian modified interfluve	-67.17513953	-23.89457864	**113.4**
550	Puna--wind erosion features	eolian modified interfluve	-67.17356207	-23.8895163	**112.8**
551	Puna--wind erosion features	eolian modified interfluve	-67.17278038	-23.88808237	**114.6**
552	Puna--wind erosion features	eolian modified interfluve	-67.18281052	-23.91983922	**113.4**
553	Puna--wind erosion features	eolian modified interfluve	-67.18307368	-23.91799107	**110.1**
554	Puna--wind erosion features	eolian modified interfluve	-67.18212945	-23.91508675	**109.1**
555	Puna--wind erosion features	eolian modified interfluve	-67.18342818	-23.90901113	**112.4**
556	Puna--wind erosion features	eolian modified interfluve	-67.22526838	-23.94419757	**103.5**
557	Puna--wind erosion features	eolian modified interfluve	-67.22400396	-23.94200389	**106.0**
558	Puna--wind erosion features	eolian modified interfluve	-67.22463672	-23.94085349	**106.5**
559	Puna--wind erosion features	eolian modified interfluve	-67.22716287	-23.93890166	**100.1**
560	Puna--wind erosion features	eolian modified interfluve	-67.25646468	-23.91466558	**113.4**
561	Puna--wind erosion features	eolian modified interfluve	-67.27065778	-23.90890406	**110.6**
562	Puna--wind erosion features	eolian modified interfluve	-67.23857057	-23.91010563	**108.9**
563	Puna--wind erosion features	eolian modified interfluve	-67.25121015	-23.92795429	**108.4**
564	Puna--wind erosion features	eolian modified interfluve	-67.2341421	-23.93441864	**106.1**
565	Puna--wind erosion features	eolian modified interfluve	-67.22300882	-23.94363408	**104.6**
566	Puna--wind erosion features	eolian modified interfluve	-67.22646841	-23.94229392	**107.4**
567	Puna--wind erosion features	eolian modified interfluve	-67.22209642	-23.93597051	**101.5**
568	Puna--wind erosion features	eolian modified interfluve	-67.22415882	-23.93994011	**99.9**
569	Puna--wind erosion features	yardang	-67.41084963	-24.60843589	**137.8**
570	Puna--wind erosion features	yardang	-67.40981547	-24.60819948	**131.0**
571	Puna--wind erosion features	yardang	-67.41084022	-24.61649168	**149.1**
572	Puna--wind erosion features	yardang	-67.41061737	-24.61589232	**144.4**
573	Puna--wind erosion features	yardang	-67.40861436	-24.61163599	**144.1**
574	Puna--wind erosion features	yardang	-67.41249592	-24.61269811	**155.5**

575	Puna--wind erosion features	yardang	-67.41069661	-24.61798185	**159.0**
576	Puna--wind erosion features	yardang	-67.41450429	-24.62531472	**153.8**
577	Puna--wind erosion features	yardang	-67.41095669	-24.60756213	**134.0**
578	Puna--wind erosion features	yardang	-67.40901279	-24.60824931	**128.9**
579	Puna--wind erosion features	yardang	-67.40892994	-24.60672443	**134.1**
580	Puna--wind erosion features	yardang	-67.40983735	-24.606798	**133.3**
581	Puna--wind erosion features	yardang	-67.40960442	-24.60738544	**136.7**
582	Puna--wind erosion features	yardang	-67.4104305	-24.60675281	**137.5**
583	Puna--wind erosion features	yardang	-67.40892253	-24.61096396	**134.6**
584	Puna--wind erosion features	yardang	-67.40869245	-24.6101381	**142.2**
585	Puna--wind erosion features	yardang	-67.41726792	-24.60154183	**141.3**
586	Puna--wind erosion features	yardang	-67.42982019	-24.62429831	**153.3**
587	Puna--wind erosion features	yardang	-67.42942446	-24.61973502	**163.3**
588	Puna--wind erosion features	yardang	-67.42905073	-24.62139124	**172.1**
589	Puna--wind erosion features	yardang	-67.43019606	-24.62276889	**160.4**
590	Puna--wind erosion features	yardang	-67.43371933	-24.62032044	**157.8**
591	Puna--wind erosion features	yardang	-67.42971239	-24.62158742	**162.9**
592	Puna--wind erosion features	yardang	-67.42900425	-24.62211042	**166.0**
593	Puna--wind erosion features	yardang	-67.42939582	-24.62234616	**158.3**
594	Puna--wind erosion features	wind streak	-69.16247737	-26.28132723	**138.5**
595	Puna--wind erosion features	wind streak	-68.46215195	-27.76103499	**114.0**
596	Puna--wind erosion features	wind streak	-68.61140847	-27.63005688	**117.9**
597	Puna--wind erosion features	eolian modified interfluve	-67.1820568	-23.36797447	**108.1**
598	Puna--wind erosion features	eolian modified interfluve	-67.18118518	-23.3665256	**102.2**
599	Puna--wind erosion features	eolian modified interfluve	-67.18067883	-23.36384114	**103.1**
600	Puna--wind erosion features	eolian modified interfluve	-67.18306978	-23.37461735	**107.2**
601	Puna--wind erosion features	eolian modified interfluve	-67.28413416	-23.56491169	**106.2**
602	Puna--wind erosion features	eolian modified interfluve	-67.28285934	-23.56752121	**104.5**
603	Puna--wind erosion features	yardang	-67.47974644	-23.58218898	**119.7**
604	Puna--wind erosion features	yardang	-67.48049965	-23.58490539	**117.8**
605	Puna--wind erosion features	yardang	-67.49215044	-23.58353683	**116.1**
606	Puna--wind erosion features	yardang	-67.48523761	-23.58788445	**118.8**
607	Puna--wind erosion features	yardang	-67.48239994	-23.58910571	**123.7**
608	Puna--wind erosion features	yardang	-67.4973974	-23.58789161	**120.7**
609	Puna--wind erosion features	yardang	-67.49034439	-23.58997595	**119.5**
610	Puna--wind erosion features	yardang	-67.49441408	-23.59040602	**124.8**
611	Puna--wind erosion features	eolian modified interfluve	-67.29795164	-23.7320169	**117.2**
612	Puna--wind erosion features	eolian modified interfluve	-67.29061766	-23.73469736	**113.7**
613	Puna--wind erosion features	eolian modified interfluve	-67.30507048	-23.73033859	**116.9**
614	Puna--wind erosion features	eolian modified interfluve	-67.29646841	-23.73471398	**119.5**
615	Puna--wind erosion features	eolian modified interfluve	-67.30801034	-23.72696865	**116.9**
616	Puna--wind erosion features	eolian modified interfluve	-67.30833763	-23.72480876	**121.2**
617	Puna--wind erosion features	eolian modified interfluve	-67.28969188	-23.73386074	**119.9**
618	Puna--wind erosion features	eolian modified interfluve	-67.30249852	-23.72902465	**117.2**
619	Puna--wind erosion features	eolian modified interfluve	-67.29193033	-23.74951459	**122.1**
620	Puna--wind erosion features	eolian modified interfluve	-67.26271626	-23.76106375	**115.6**
621	Puna--wind erosion features	eolian modified interfluve	-67.26912473	-23.76414251	**117.8**
622	Puna--wind erosion features	eolian modified interfluve	-67.15442021	-23.83856724	**121.9**
623	Puna--wind erosion features	eolian modified interfluve	-67.16007909	-23.83705899	**120.2**
624	Puna--wind erosion features	eolian modified interfluve	-67.16015014	-23.82627434	**122.1**
625	Puna--wind erosion features	eolian modified interfluve	-67.17924945	-23.89119849	**111.7**
626	Puna--wind erosion features	eolian modified interfluve	-67.17737707	-23.89250581	**115.3**
627	Puna--wind erosion features	eolian modified interfluve	-67.20993445	-23.9072694	**108.7**
628	Puna--wind erosion features	eolian modified interfluve	-67.25490744	-23.90239008	**110.1**
629	Puna--wind erosion features	eolian modified interfluve	-67.28376619	-23.93436011	**107.6**
630	Puna--wind erosion features	eolian modified interfluve	-67.29181862	-23.93621247	**96.2**
631	Puna--wind erosion features	eolian modified interfluve	-67.29234195	-23.93951541	**91.8**
632	Puna--wind erosion features	eolian modified interfluve	-67.29078007	-23.93832408	**93.4**
633	Puna--wind erosion features	eolian modified interfluve	-67.29039628	-23.93901128	**90.3**
634	Puna--wind erosion features	yardang	-67.51101832	-24.89501022	**136.3**
635	Puna--wind erosion features	yardang	-67.52083451	-24.87459059	**134.9**
636	Puna--wind erosion features	yardang	-67.52278535	-24.87368533	**132.9**
637	Puna--wind erosion features	yardang	-67.5212359	-24.87458588	**143.4**
638	Puna--wind erosion features	yardang	-67.41163636	-24.60856092	**134.5**

639	Puna--wind erosion features	yardang	-67.40761828	-24.60941465	**151.7**
640	Puna--wind erosion features	yardang	-67.40971909	-24.61298655	**132.5**
641	Puna--wind erosion features	yardang	-67.69631634	-26.52462127	**120.1**
642	Puna--wind erosion features	yardang	-67.69615691	-26.52303887	**107.8**
643	Puna--wind erosion features	yardang	-67.69808509	-26.52065226	**108.3**
644	Puna--wind erosion features	yardang	-67.69931592	-26.52091028	**121.4**
645	Puna--wind erosion features	yardang	-67.70162035	-26.51745284	**115.5**
646	Puna--wind erosion features	yardang	-67.70030247	-26.51621868	**115.6**
647	Puna--wind erosion features	yardang	-67.69706598	-26.515211	**122.0**
648	Puna--wind erosion features	yardangs	-67.69643322	-26.5154427	**117.3**
649	Puna--wind erosion features	yardangs	-67.69674252	-26.51622149	**121.1**
650	Puna--wind erosion features	yardangs	-67.69842573	-26.51857524	**113.0**
651	Puna--wind erosion features	yardangs	-67.69816572	-26.51759471	**115.5**
652	Puna--wind erosion features	yardangs	-67.69737248	-26.51677893	**115.1**
653	Puna--wind erosion features	yardangs	-67.69817405	-26.51568058	**114.6**
654	Puna--wind erosion features	yardangs	-67.69825553	-26.5160565	**107.3**
655	Puna--wind erosion features	yardangs	-67.69819702	-26.51997185	**118.3**
656	Puna--wind erosion features	yardangs	-67.70082545	-26.51856031	**119.2**
657	Puna--wind erosion features	eolian modified interfluve	-67.74403818	-26.57898183	**115.1**
658	Puna--wind erosion features	eolian modified interfluve	-67.74404102	-26.5744657	**111.3**
659	Puna--wind erosion features	eolian modified interfluve	-67.72368415	-26.57257194	**109.3**
660	Puna--wind erosion features	eolian modified interfluve	-67.73874099	-26.57839155	**112.0**
661	Puna--wind erosion features	eolian modified interfluve	-67.73918623	-26.58335506	**115.0**
662	Puna--wind erosion features	eolian modified interfluve	-67.73272297	-26.59519405	**111.3**
663	Puna--wind erosion features	eolian modified interfluve	-67.73547567	-26.57294641	**117.5**
664	Puna--wind erosion features	eolian modified interfluve	-67.74369467	-26.53635924	**117.5**
665	Puna--wind erosion features	eolian modified interfluve	-67.74523488	-26.53659973	**124.1**
666	Puna--wind erosion features	eolian modified interfluve	-67.73639456	-26.53086958	**124.4**
667	Puna--wind erosion features	yardang	-67.69733081	-26.52204242	**119.8**
668	Puna--wind erosion features	yardang	-67.69708148	-26.52163554	**122.9**
669	Puna--wind erosion features	yardang	-67.69598161	-26.52268193	**120.9**
670	Puna--wind erosion features	yardang	-67.69850772	-26.51786228	**110.8**
671	Puna--wind erosion features	yardang	-67.69955314	-26.51742771	**117.8**
672	Puna--wind erosion features	yardang	-67.70130952	-26.51721026	**116.0**
673	Puna--wind erosion features	yardang	-67.69890173	-26.51412047	**108.3**
674	Puna--wind erosion features	yardang	-67.69807083	-26.51705057	**123.0**
675	Puna--wind erosion features	yardang	-67.69748465	-26.51704953	**115.2**
676	Puna--wind erosion features	yardang	-67.69950567	-26.51628469	**112.7**
677	Puna--wind erosion features	yardang	-67.69839198	-26.51718015	**117.2**
678	Puna--wind erosion features	yardang	-67.69971217	-26.51788833	**118.3**
679	Puna--wind erosion features	yardang	-67.69821581	-26.52320497	**123.0**
680	Puna--wind erosion features	yardang	-67.69115713	-26.53294973	**116.2**
681	Puna--wind erosion features	yardang	-67.68799875	-26.53091555	**124.9**
682	Puna--wind erosion features	yardang	-67.68887726	-26.52887988	**122.0**
683	Puna--wind erosion features	yardang	-67.56969781	-26.7419775	**136.1**
684	Puna--wind erosion features	yardang	-67.57219253	-26.73492875	**132.1**
685	Puna--wind erosion features	yardang	-67.57003792	-26.72316643	**141.2**
686	Puna--wind erosion features	yardang	-67.56776313	-26.72003739	**133.1**
687	Puna--wind erosion features	yardang	-67.57160382	-26.72057828	**131.8**
688	Puna--wind erosion features	yardang	-67.57244244	-26.71753998	**139.4**
689	Puna--wind erosion features	yardang	-67.57253896	-26.71677586	**140.5**
690	Puna--wind erosion features	yardang	-67.56168564	-26.71467325	**129.8**
691	Puna--wind erosion features	yardang	-67.57175354	-26.71493341	**136.0**
692	Puna--wind erosion features	yardang	-67.5711466	-26.71459456	**129.7**
693	Puna--wind erosion features	yardang	-67.57068863	-26.71398113	**128.1**
694	Puna--wind erosion features	yardang	-67.57064684	-26.7137682	**129.2**
695	Puna--wind erosion features	yardang	-67.57044461	-26.71309463	**136.4**
696	Puna--wind erosion features	yardang	-67.57025831	-26.71257984	**131.9**
697	Puna--wind erosion features	yardang	-67.57061255	-26.71143704	**131.0**
698	Puna--wind erosion features	yardang	-67.56922211	-26.71016002	**130.1**
699	Puna--wind erosion features	yardang	-67.56922863	-26.71062211	**131.8**
700	Puna--wind erosion features	yardang	-67.56968047	-26.71168347	**126.7**
701	Puna--wind erosion features	yardang	-67.57104763	-26.71098525	**129.3**
702	Puna--wind erosion features	yardang	-67.5711275	-26.71199438	**133.6**

703	Puna--wind erosion features	yardang	-67.56848949	-26.70761591	**129.3**
704	Puna--wind erosion features	Untitled Path	-67.56825538	-26.70734415	**132.3**
705	Puna--wind erosion features	yardang	-67.56987848	-26.70949812	**131.5**
706	Puna--wind erosion features	yardang	-67.57032232	-26.70647988	**126.7**
707	Puna--wind erosion features	yardang	-67.56738495	-26.70584347	**131.9**
708	Puna--wind erosion features	yardang	-67.56334677	-26.70263496	**127.1**
709	Puna--wind erosion features	yardang	-67.56207816	-26.70068872	**136.4**
710	Puna--wind erosion features	yardang	-67.56073147	-26.70108461	**125.9**
711	Puna--wind erosion features	yardang	-67.55137154	-26.69503315	**135.0**
712	Puna--wind erosion features	yardang	-67.55179571	-26.69519399	**134.9**
713	Puna--wind erosion features	yardang	-67.55165585	-26.6958138	**137.6**
714	Puna--wind erosion features	yardang	-67.55150319	-26.69376293	**134.0**
715	Puna--wind erosion features	yardang	-67.54908985	-26.69583082	**123.0**
716	Puna--wind erosion features	yardang	-67.54077134	-26.69899813	**132.4**
717	Puna--wind erosion features	yardang	-67.5403403	-26.69826034	**127.5**
718	Puna--wind erosion features	yardang	-67.54003031	-26.69804401	**127.8**
719	Puna--wind erosion features	yardang	-67.54763371	-26.69504069	**132.1**
720	Puna--wind erosion features	yardang	-67.54864754	-26.69496252	**131.3**
721	Puna--wind erosion features	yardang	-67.54701545	-26.69432984	**131.5**
722	Puna--wind erosion features	yardang	-67.54713161	-26.69269977	**132.7**
723	Puna--wind erosion features	yardang	-67.54765099	-26.69266696	**135.7**
724	Puna--wind erosion features	yardang	-67.5480174	-26.69296385	**132.4**
725	Puna--wind erosion features	yardang	-67.54814021	-26.69008937	**127.8**
726	Puna--wind erosion features	yardang	-67.54995562	-26.68938772	**130.7**
727	Puna--wind erosion features	yardang	-67.54605221	-26.69144009	**135.9**
728	Puna--wind erosion features	yardang	-67.54720985	-26.69096181	**134.0**
729	Puna--wind erosion features	yardang	-67.54800188	-26.69132027	**134.6**
730	Puna--wind erosion features	yardang	-67.54466178	-26.69102036	**134.3**
731	Puna--wind erosion features	yardang	-67.5437206	-26.69088829	**130.8**
732	Puna--wind erosion features	yardang	-67.54312569	-26.68979094	**127.4**
733	Puna--wind erosion features	yardang	-67.5438743	-26.68946416	**124.1**
734	Puna--wind erosion features	yardang	-67.54456317	-26.68977951	**130.3**
735	Puna--wind erosion features	yardang	-67.54555614	-26.69058702	**128.3**
736	Puna--wind erosion features	yardang	-67.5487045	-26.68787085	**130.9**
737	Puna--wind erosion features	yardang	-67.5466036	-26.68785753	**130.3**
738	Puna--wind erosion features	yardang	-67.54645799	-26.686617	**129.6**
739	Puna--wind erosion features	yardang	-67.54625207	-26.68655135	**135.6**
740	Puna--wind erosion features	yardang	-67.55012423	-26.68674394	**131.7**
741	Puna--wind erosion features	yardang	-67.55074925	-26.68688238	**128.3**
742	Puna--wind erosion features	yardang	-67.54712843	-26.68813594	**134.3**
743	Puna--wind erosion features	yardang	-67.54525397	-26.68846364	**130.3**
744	Puna--wind erosion features	yardang	-67.54709474	-26.68954964	**131.7**
745	Puna--wind erosion features	yardang	-67.54523045	-26.68659787	**133.0**
746	Puna--wind erosion features	yardang	-67.54624116	-26.68749852	**132.9**
747	Puna--wind erosion features	yardang	-67.54791418	-26.68509636	**128.7**
748	Puna--wind erosion features	yardang	-67.54529587	-26.68866874	**134.7**
749	Puna--wind erosion features	yardang	-67.54417969	-26.68716816	**129.3**
750	Puna--wind erosion features	yardang	-67.5434594	-26.6851452	**137.2**
751	Puna--wind erosion features	yardang	-67.54135613	-26.68775485	**138.0**
752	Puna--wind erosion features	yardang	-67.54135552	-26.68890503	**134.0**
753	Puna--wind erosion features	yardang	-67.53978216	-26.68742609	**122.7**
754	Puna--wind erosion features	yardang	-67.53915177	-26.68545934	**129.4**
755	Puna--wind erosion features	yardang	-67.53863171	-26.68479255	**132.0**
756	Puna--wind erosion features	yardang	-67.53909075	-26.68302972	**134.6**
757	Puna--wind erosion features	yardang	-67.53933887	-26.68316919	**132.4**
758	Puna--wind erosion features	yardang	-67.53798322	-26.68050662	**138.3**
759	Puna--wind erosion features	yardang	-67.53972694	-26.68245391	**132.0**
760	Puna--wind erosion features	yardang	-67.53925025	-26.6811269	**134.7**
761	Puna--wind erosion features	yardang	-67.53669205	-26.67958684	**134.2**
762	Puna--wind erosion features	yardang	-67.53371727	-26.68056721	**135.9**
763	Puna--wind erosion features	yardang	-67.53694172	-26.67974117	**123.8**
764	Puna--wind erosion features	yardang	-67.53252238	-26.67937564	**131.1**
765	Puna--wind erosion features	yardang	-67.53151695	-26.68103695	**130.7**
766	Puna--wind erosion features	yardang	-67.53129717	-26.67978951	**130.9**

767	Puna--wind erosion features	yardang	-67.53110802	-26.68130367	**128.3**
768	Puna--wind erosion features	yardang	-67.53214213	-26.67710592	**130.7**
769	Puna--wind erosion features	yardang	-67.53107107	-26.67775814	**129.0**
770	Puna--wind erosion features	yardang	-67.52795012	-26.6767371	**129.0**
771	Puna--wind erosion features	yardang	-67.53331294	-26.6741202	**130.7**
772	Puna--wind erosion features	yardang	-67.53597988	-26.67844318	**138.2**
773	Puna--wind erosion features	yardang	-67.52923535	-26.67905121	**128.3**
774	Puna--wind erosion features	yardang	-67.53284858	-26.6797985	**133.6**
775	Puna--wind erosion features	yardang	-67.52816634	-26.67748193	**125.4**
776	Puna--wind erosion features	yardang	-67.5222214	-26.68579594	**136.2**
777	Puna--wind erosion features	yardang	-67.52195727	-26.68343377	**135.0**
778	Puna--wind erosion features	yardang	-67.52251783	-26.68352463	**131.2**
779	Puna--wind erosion features	yardang	-67.52467693	-26.68615827	**134.4**
780	Puna--wind erosion features	yardang	-67.52396746	-26.68441624	**135.0**
781	Puna--wind erosion features	yardang	-67.52257275	-26.68385653	**133.5**
782	Puna--wind erosion features	yardang	-67.5240241	-26.68349529	**127.6**
783	Puna--wind erosion features	yardang	-67.52215727	-26.68694572	**125.0**
784	Puna--wind erosion features	yardang	-67.52196363	-26.6824951	**131.9**
785	Puna--wind erosion features	yardang	-67.52257177	-26.68022542	**131.6**
786	Puna--wind erosion features	yardang	-67.52171149	-26.68015732	**134.5**
787	Puna--wind erosion features	yardang	-67.52104909	-26.68016635	**128.1**
788	Puna--wind erosion features	yardang	-67.5191359	-26.68028842	**128.3**
789	Puna--wind erosion features	yardang	-67.52069751	-26.67839933	**127.1**
790	Puna--wind erosion features	yardang	-67.51950342	-26.67807386	**128.2**
791	Puna--wind erosion features	yardang	-67.52006679	-26.67727718	**129.0**
792	Puna--wind erosion features	yardang	-67.52114702	-26.67716495	**126.1**
793	Puna--wind erosion features	yardang	-67.51844709	-26.67626284	**130.0**
794	Puna--wind erosion features	yardang	-67.52376271	-26.69341869	**132.9**
795	Puna--wind erosion features	yardang	-67.52476703	-26.69201011	**128.7**
796	Puna--wind erosion features	yardang	-67.52374388	-26.69154448	**137.7**
797	Puna--wind erosion features	yardang	-67.52491367	-26.6930928	**135.5**
798	Puna--wind erosion features	yardang	-67.5214613	-26.69398397	**128.9**
799	Puna--wind erosion features	yardang	-67.52110767	-26.69288103	**133.7**
800	Puna--wind erosion features	yardang	-67.52423664	-26.69042969	**135.2**
801	Puna--wind erosion features	yardang	-67.52448315	-26.69165581	**130.2**
802	Puna--wind erosion features	yardang	-67.5232506	-26.69076363	**127.9**
803	Puna--wind erosion features	yardang	-67.52122055	-26.6910483	**136.3**
804	Puna--wind erosion features	yardang	-67.52274524	-26.69031438	**127.1**
805	Puna--wind erosion features	yardang	-67.52115722	-26.68872919	**134.4**
806	Puna--wind erosion features	yardang	-67.52150851	-26.68863101	**132.9**
807	Puna--wind erosion features	yardang	-67.50714597	-26.68641775	**128.1**
808	Puna--wind erosion features	yardang	-67.50636204	-26.68647087	**131.0**
809	Puna--wind erosion features	yardang	-67.50776431	-26.68583324	**123.7**
810	Puna--wind erosion features	yardang	-67.50725127	-26.68672265	**125.1**
811	Puna--wind erosion features	yardang	-67.50628046	-26.68723669	**136.0**
812	Puna--wind erosion features	yardang	-67.5063288	-26.68449495	**120.7**
813	Puna--wind erosion features	yardang	-67.50500149	-26.68549231	**131.9**
814	Puna--wind erosion features	yardang	-67.50401775	-26.68535653	**135.2**
815	Puna--wind erosion features	yardang	-67.50542482	-26.68497847	**135.5**
816	Puna--wind erosion features	yardang	-67.5045022	-26.68539122	**135.6**
817	Puna--wind erosion features	yardang	-67.50466491	-26.68445831	**134.5**
818	Puna--wind erosion features	yardang	-67.50004144	-26.68604835	**153.8**
819	Puna--wind erosion features	yardang	-67.52360403	-26.69983467	**137.0**
820	Puna--wind erosion features	yardang	-67.52462345	-26.69917278	**135.4**
821	Puna--wind erosion features	yardang	-67.52243832	-26.70044961	**137.8**
822	Puna--wind erosion features	yardang	-67.52214417	-26.70020505	**138.4**
823	Puna--wind erosion features	yardang	-67.52181935	-26.70014764	**137.0**
824	Puna--wind erosion features	yardang	-67.52025531	-26.70100298	**133.1**
825	Puna--wind erosion features	yardang	-67.52113535	-26.69882815	**140.3**
826	Puna--wind erosion features	yardang	-67.51965031	-26.70000196	**133.6**
827	Puna--wind erosion features	yardang	-67.52082232	-26.69853256	**133.9**
828	Puna--wind erosion features	yardang	-67.51839754	-26.6988441	**133.0**
829	Puna--wind erosion features	yardang	-67.52142345	-26.7008058	**130.1**
830	Puna--wind erosion features	yardang	-67.52319724	-26.69993319	**131.7**

831	Puna--wind erosion features	yardang	-67.52314956	-26.69922302	**134.7**
832	Puna--wind erosion features	yardang	-67.52308464	-26.69901274	**135.3**
833	Puna--wind erosion features	yardang	-67.52492433	-26.69945011	**140.6**
834	Puna--wind erosion features	yardang	-67.5198864	-26.70060926	**137.4**
835	Puna--wind erosion features	yardang	-67.52458288	-26.69399638	**140.6**
836	Puna--wind erosion features	yardang	-67.52214914	-26.69625656	**135.6**
837	Puna--wind erosion features	yardang	-67.5230596	-26.69754109	**132.6**
838	Puna--wind erosion features	yardang	-67.52270141	-26.69752746	**136.9**
839	Puna--wind erosion features	yardang	-67.52185006	-26.69679296	**134.3**
840	Puna--wind erosion features	yardang	-67.52268273	-26.69797043	**117.5**
841	Puna--wind erosion features	yardang	-67.52002644	-26.69650761	**131.9**
842	Puna--wind erosion features	yardang	-67.52124209	-26.69553358	**134.1**
843	Puna--wind erosion features	yardang	-67.52001701	-26.69583814	**131.2**
844	Puna--wind erosion features	yardang	-67.52015515	-26.69851093	**133.1**
845	Puna--wind erosion features	yardang	-67.51874018	-26.70852706	**141.9**
846	Puna--wind erosion features	yardang	-67.52437818	-26.70262814	**139.6**
847	Puna--wind erosion features	yardang	-67.52309218	-26.70414335	**132.2**
848	Puna--wind erosion features	yardang	-67.52765962	-26.70272876	**135.4**
849	Puna--wind erosion features	yardang	-67.52554619	-26.7060534	**136.0**
850	Puna--wind erosion features	yardang	-67.52321851	-26.70538156	**131.1**
851	Puna--wind erosion features	yardang	-67.5215386	-26.70403245	**132.0**
852	Puna--wind erosion features	yardang	-67.52974117	-26.70674845	**134.9**
853	Puna--wind erosion features	yardang	-67.52524632	-26.70830134	**139.6**
854	Puna--wind erosion features	yardang	-67.52545946	-26.70791144	**130.7**
855	Puna--wind erosion features	yardang	-67.52595109	-26.70779814	**139.7**
856	Puna--wind erosion features	yardang	-67.51589925	-26.71036996	**139.0**
857	Puna--wind erosion features	yardang	-67.51500739	-26.71037776	**143.8**
858	Puna--wind erosion features	yardang	-67.5278464	-26.72230274	**145.7**
859	Puna--wind erosion features	yardang	-67.55801139	-26.73523766	**124.5**
860	Puna--wind erosion features	yardang	-67.55897569	-26.73590526	**126.2**
861	Puna--wind erosion features	yardang	-67.56105405	-26.73659941	**124.4**
862	Puna--wind erosion features	yardang	-67.55621475	-26.73876299	**125.0**
863	Puna--wind erosion features	yardang	-67.55497168	-26.73990497	**114.3**
864	Puna--wind erosion features	yardang	-67.5524775	-26.74125689	**134.9**
865	Puna--wind erosion features	yardang	-67.55004549	-26.73747536	**115.3**
866	Puna--wind erosion features	yardang	-67.55549753	-26.73739036	**131.8**

Supplementary Table S2:

Sources of U-Pb Detrital Zircon Comparison Data

U-Pb comparison data for potential source areas was taken from the peer-reviewed literature. Published U-Pb detrital zircon data sets were compared to the new U-Pb data presented here without additional filtering. Comparison data sets were already filtered for substantial discordance, reverse discordance, high initial-Pb, high uncertainties on $^{206}Pb/^{207}Pb$ and $^{206}Pb/^{238}U$ ages. Properties of the comparison samples are provided if available in the referenced work, otherwise left blank. Significant figures on latitude and longitude measurements shown as presented in referenced papers. Geographical locations either stated in the text or assumed from maps provided in referenced papers.

U-Pb detrital zircon source area comparison data

Region	lat.	long.	location	Reference	Sample	Formation	Depositional Age
**southern Puna**							
			Pasto Ventura	Zhou et al., 2016	PT11T-53		Miocene
			Pasto Ventura	Zhou et al., 2016	PT11T-61		Miocene
			Pasto Ventura	Zhou et al., 2016	PT11T-51		Miocene
			Pasto Ventura	Zhou et al., 2016	PV1		late Eocene - early Oligocene
			Antofagasta de al Sierra	Zhou et al., 2017	A-DR-01		late Eocene - early Oligocene
			Antofagasta de al Sierra	Zhou et al., 2017	A-DR-02		late Eocene - early Oligocene
			Antofagasta de al Sierra	Zhou et al., 2017	A-DR-04		late Eocene - early Oligocene
			Antofagasta de al Sierra	Zhou et al., 2017	A-DR-05		late Eocene - early Oligocene
			Antofagasta de al Sierra	Zhou et al., 2017	A-DR-06		late Eocene - early Oligocene
			Antofagasta de al Sierra	Zhou et al., 2017	A-DR-07		late Eocene - early Oligocene
			Antofagasta de al Sierra	Zhou et al., 2017	A-DR-21		late Eocene - early Oligocene
			Antofagasta de al Sierra	Zhou et al., 2017	A-DR-25		late Eocene - early Oligocene
**central Puna**	24.614° S	67.222° W	Arizaro Basin	DeCelles et al., 2015	9AR386	Vizcachera Fm.	Miocene
	24.634° S	67.242° W	Arizaro Basin	DeCelles et al., 2015	3AR1	Vizcachera Fm.	Miocene
	24.557° S	67.319° W	Arizaro Basin	DeCelles et al., 2015	TG10	Vizcachera Fm.	Miocene
	24.690° S	67.330° W	Arizaro Basin	DeCelles et al., 2015	12AR70	Vizcachera Fm.	Miocene
	24.559° S	67.326° W	Arizaro Basin	DeCelles et al., 2015	TG246	Vizcachera Fm.	Miocene
	24.637° S	67.308° W	Arizaro Basin	DeCelles et al., 2015	4AR342	Vizcachera Fm.	Miocene
	24.884° S	68.031° W	Arizaro Basin	DeCelles et al., 2015	ARB09	Vizcachera Fm.	Miocene
			Copalayo	DeCelles et al., 2007	1SP0	Copaplayo Fm.	Ordovician
			Copalayo	DeCelles et al., 2007	1SP32	Geste Fm.	Eocene
			Copalayo	DeCelles et al., 2007	2SP38	Geste Fm.	Eocene
			Copalayo	DeCelles et al., 2007	2SP238	Geste Fm.	Eocene
			Copalayo	DeCelles et al., 2007	2SP277	Geste Fm.	Eocene
			Copalayo	DeCelles et al., 2007	3SP431	Geste Fm.	Eocene
			Copalayo	DeCelles et al., 2007	4SP7	Geste Fm.	Eocene
			Baros Arana Fold System	Henríquez et al. 2019	AT-NAJ	Naranja Fm.	Paleocene-Eocene
			Baros Arana Fold System	Henríquez et al. 2019	1AT433	Loma Amarilla Fm.	Oligocene
	23. 230° S	66.926° W	Paso de Jama	Henríquez et al. 2020	sh17-19	Angastaco Fm.	Miocene

	23.093° S	66.759° W	El Toro area	Henríquez et al. 2020	SH17-21	~Lumbrera Fm.	Paleogene
	23.126° S	66.769° W	El Toro area	Henríquez et al. 2020	SH16-44		≤Eocene
	23.185° S	66.509° W	Susques Range	Henríquez et al. 2020	SH17-10		Miocene
	23.242° S	66.955° W	Pso de Jama	Henríquez et al. 2020	PJ793DZ	~Quebrado de Los Colorados Fm.	≤Eocene
	23.197°	66.481° W	Susques Range	Henríquez et al. 2020	SH17-12		Paleocene
			Cianzo basin	Siks and Horton, 2011	SA01	Santa Bárbara Subgroup	Paleocene
			Cianzo basin	Siks and Horton, 2011	CA184	Casa Grande Fm.	Eocene-Oligocene
			Cianzo basin	Siks and Horton, 2011	RB835	Río Grande Fm.	mid-Miocene
			Cianzo basin	Siks and Horton, 2011	PA-01	Pisungo Fm.	late Miocene
	23.17111° S	65.75511° W	Sierra Aguilar	Streit et al., 2017	Aguilar catchment	active river	
	23.331997° S	65.344496° W	Humahuaca basin	Streit et al., 2017	Rio Grande	active river	
	23.380743° S	65.351381° W	Humahuaca basin	Streit et al., 2017	Rio Yacoraite	active river	
	23.30927° S	65.53719° W	Casa Grande basin	Streit et al., 2017	Casa Grande outlet	active river	
	23.383953° S	65.356253° W	Humahuaca basin	Streit et al., 2017	Rio Yacoraite section 46 m	Uquía Fm./Tilcara Fm.	Pliocene
	23.384293° S	65.358641° W	Humahuaca basin	Streit et al., 2017	Rio Yacoraite section 161 m	Uquía Fm./Tilcara Fm.	Pleistocene
	23.385875° S	65.361102° W	Humahuaca basin	Streit et al., 2017	Rio Yacoraite section 265 m	Uquía Fm./Tilcara Fm.	Pleistocene
	23.384527° S	65.365005° W	Humahuaca basin	Streit et al., 2017	Rio Yacoraite section 466 m	Uquía Fm./Tilcara Fm.	Pleistocene
	23.385115° S	65.366343° W	Humahuaca basin	Streit et al., 2017	Rio Yacoraite section 558 m	Uquía Fm./Tilcara Fm.	Pleistocene
	23.385191° S	65.368288° W	Humahuaca basin	Streit et al., 2017	Rio Yacoraite section 681 m	Uquía Fm./Tilcara Fm.	Pleistocene
**Chaco**	23.2499° S	64.1406° W	Chaco-Bermejo	Pepper et al., 2016	RBERAR02	active river	
	25.0146° S	58.1292° W	Chaco-lower Pilcomayo	Pepper et al., 2016	RPILAR01	active river	
	22.1571° S	62.7932° W	Chaco-Pilcomayo	Pepper et al., 2016	RPILAR02	active river	
	26.6607° S	58.6367° W	Chaco-lower Bermejo	Pepper et al., 2016	RBERAR01	active river	
			Chaco-Bermejo	McGlue et al., 2016	7633	active river	
			Chaco-Bermejo	McGlue et al., 2016	7103	active river	
			Chaco-Bermejo	McGlue et al., 2016	71274	active river	
			Chaco-Bermejo	McGlue et al., 2016	71584	active river	
			Chaco-Bermejo	McGlue et al., 2016	710172	active river	
**Sierras Pampeanas**	31.23225° S	67.74754° W	Bermejo basin	Capaldi et al., 2020	AMP03	Niquizanga Fm.	Pliocene-modern
	31.24070° S	67.75203° W	Bermejo basin	Capaldi et al., 2020	AMP02	Niquizanga Fm.	middle-Miocene
	31.24077° S	67.75351° W	Bermejo basin	Capaldi et al., 2020	AMP01	Niquizanga Fm.	Oligocene-Miocene
	31.36374° S	68.16521° W	Sierras Pampeans tributary	Capaldi et al., 2020	RPDP01	active river	modern
	31.24165° S	67.72928° W	Bermejo basin	Capaldi et al., 2020	AMP05	Río del Camperito Fm.	Pliocene-modern
	31.24081° S	67.74238° W	Bermejo basin	Capaldi et al., 2020	AMP04	Río del Camperito Fm.	Pliocene-modern
	31.24794° S	68.39191° W		Capaldi et al., 2021	VILE11		Miocene
	31.24608° S	68.40536° W		Capaldi et al., 2021	VILE10		Miocene
	30.88993° S	68.44434° W	Bermejo basin	Capaldi et al., 2020	MGN10	Quebrada del Jarillal Fm.	upper-Miocene
	30.92383° S	68.505° W	Bermejo basin	Capaldi et al., 2020	MGN09	Quebrada del Jarillal Fm.	middle-Miocene

	30.90485° S	68.51025° W	Bermejo basin	Capaldi et al., 2020	MGN08	Río Saldo Fm.	Oligocene-Miocene
	30.68884° S	68.39607° W	Bermejo basin	Capaldi et al., 2020	MGN05	Mogna Fm.	Pliocene-modern
	30.68128° S	68.42809° W	Bermejo basin	Capaldi et al., 2020	MGN04	Río Jachal Fm.	Pliocene-modern
	30.6761° S	68.44592° W	Bermejo basin	Capaldi et al., 2020	MGN03	Río Jachal Fm.	upper-Miocene
	30.68421° S	68.46669° W	Bermejo basin	Capaldi et al., 2020	MGN02	Quebrada del Cura Fm.	upper-Miocene
	30.67894° S	68.48889° W	Bermejo basin	Capaldi et al., 2020	MGN01	Huachipama Fm.	upper-Miocene
	30.62882° S	68.78916° W		Fosdick et al., 2017	12RF11	Cuculi Fm.	
	30.62838° S	68.78719° W		Fosdick et al., 2017	12RF09	upper Vallecito Fm.	
	30.625727° S	68.786769° W		Fosdick et al., 2017	12RF08	Patquia Fm.	
	30.60166° S	68.8098° W		Fosdick et al., 2017	15RF02	lower Vallecito Fm.	
	30.59939° S	68.82255° W		Fosdick et al., 2017	15RF04	upper Vallecito Fm.	
	30.59805° S	68.82497° W		Fosdick et al., 2017	15RF07	upper Vallecito Fm.	
	30.16606° S	68.44112° W		Fosdick et al., 2015	HC06	Quebrada del Cura Fm.	
	30.16332° S	68.45181° W		Fosdick et al., 2015	HC04	Huachipama Fm.	
	30.16201° S	68.46072° W		Fosdick et al., 2015	HC01	Quebrada del Jarillal Fm.	
	30.148586° S	68.52666° W		Fosdick et al., 2015	12RH10	lower Vallecito Fm.	
			Santuario des Aves	Reat and Fosdick, 2018	15SA06	Puesto La Flecha Fm.	≤late Eocene
	29°24'16"S	66°56'15"W		Adams et al., 2011	LRJ	Meson Group	Cambrian
**Precordillera**	30.922450° S	68.797250° W	Precordillera Talacasto basin	Capaldi et al., 2020	TAL 13		Miocene
			Precordillera Talacasto basin	Levina et al., 2014	Tal 14		Miocene
			Precordillera Talacasto basin	Levina et al., 2014	Tal 16		Miocene
			Precordillera Talacasto basin	Levina et al., 2014	Tal 06		Miocene
			Precordillera Talacasto basin	Levina et al., 2014	Tal 09		Miocene
			Precordillera Talacasto basin	Levina et al., 2014	QA20		
			Precordillera Talacasto basin	Levina et al., 2014	QA05		
	30.19791 S	68.81060 W	Jachal	Capaldi et al., 2017	RSJ01	Rio Jachal	modern
	30.21032 S	68.88143 W	Jachal	Capaldi et al., 2017	RZH01	Rio Jachal	modern
**Neuquén basin**			Chos Malal	Balgord, 2017	ND511	Arroyo Palao Fm.	Miocene
			Chos Malal	Balgord, 2017	NB-8	Tordillo Fm.	Late Jurassic
			Zapala	Balgord, 2017	NB111	Lotena Fm.	Middle–Late Jurassic
			Zapala	Balgord, 2017	BN1611	Lotena Fm.	Middle–Late Jurassic
			Zapala	Balgord, 2017	NB1811	Las Lajas Fm.	Middle Jurassic
			Zapala	Balgord, 2017	NB1911	Pecun Leufu Fm.	Early Cretaceous
**northern Patagonia**			Chaitén	Encinas et al., 2014	PUD 1	Padahuapi Fm.	
			Chaitén	Encinas et al., 2014	PUD 7	Padahuapi Fm.	
			Chaitén	Encinas et al., 2014	PNEG	La Cascada Fm.	
			Chaitén	Encinas et al., 2014	NONO2	La Cascada Fm.	
			La Junta	Encinas et al., 2014	VAR2	Vargas Fm.	
			La Junta	Encinas et al., 2014	VAR 5	Vargas Fm.	
			La Junta	Encinas et al., 2014	JUN1	La Junta Fm.	

**southern Patagonia**	-51.31009	-72.20272	Cerro Castillo	Leonard et al., 2020	15CCS302	Río Guillermo Fm.	Miocene
	-51.30816	-72.19702	Cerro Castillo	Leonard et al., 2020	15CCS306	Río Guillermo Fm.	Miocene
	-51.31296	-72.22133	Cerro Castillo	Leonard et al., 2020	15CCS105	Río Guillermo Fm.	Miocene
	-51.31315	72.21645	Cerro Castillo	Leonard et al., 2020	15CCS210	Río Guillermo Fm.	Miocene
	-51.30816	-72.19702	Cerro Castillo	Leonard et al., 2020	15CCS306	Río Guillermo Fm.	Miocene
	-51.30338	-72.1867	Cancha Carrera	Fosdick et al., 2020	JCF09-237B	Río Guillermo Fm.	Miocene
	-51.31373	-72.21932	Cancha Carrera	Fosdick et al., 2020	Rt28DZ6	Río Guillermo Fm.	Miocene
	-51.31163	-72.22042	Cancha Carrera	Fosdick et al., 2020	Rt28DZ5	Río Turbio (upper)	
	-51.29761	-72.23581	Cancha Carrera	Fosdick et al., 2020	Rt28DZ7	Río Turbio (upper)	
	-51.29667	-72.23819	Cancha Carrera	Fosdick et al., 2020	Rt28DZ8	Río Turbio (upper)	
	-51.31735	-72.29126	Cancha Carrera	Fosdick et al., 2020	17CCRT2-29	Río Turbio (lower)	
	-51.28071	-72.28936	Cancha Carrera	Fosdick et al., 2020	14LdCdz2	Río Turbio (lower)	
	-51.27997	-72.28916	Cancha Carrera	Fosdick et al., 2020	14LdCdz4	Río Turbio (lower)	
	-51.28001	-72.28927	Cancha Carrera	Fosdick et al., 2020	15LDC02/14DZ3	Cerro Dorotea	
	-51.28475	-72.30764	Cancha Carrera	Fosdick et al., 2020	14AVDZ2	Cerro Dorotea	
	-51.28473	-72.30828	Cancha Carrera	Fosdick et al., 2020	14AVDZ1	Cerro Dorotea	
	-51.27793	-72.31254	Cancha Carrera	Fosdick et al., 2020	15LDC05	Dorotea	
**Río Neuquén**	38.946038° S	68.03127511° W	lower Río Neuquén	Pepper et al., 2016	RNEQAR01	active river	modern
**Río Salado**	38.881630° S	61.599615° W	lower Río Salado	Pepper et al., 2016		active river	modern
**Río Limay**	38.97885256° S	68.0387335° W	Río Limay	Pepper et al., 2016	RLIMAR01	active river	modern
**Río Colorado**	37.67533435° S	67.76585762° W	lower Río Colorado	Pepper et al., 2016		active river	modern
	39.52600191° S	62.68103172° W	central Río Colorado	Pepper et al., 2016	RCOLAR01	active river	modern
**Río Negro**	40.79274679° S	63.01730244° W	lower Río Negro	Pepper et al., 2016	RNEGAR01	active river	modern
	40.80253199° S	62.99248673° W	central Río Negro	Pepper et al., 2016	RNEGAR02	active river	modern
**Río Chubut**	43.293830° S	65.302607° W	lower Río Chubut	Pepper et al., 2016	RCHUAR01	active river	modern
	43.720465° S	67.289337° W	central Río Chubut	Pepper et al., 2016	RCHUAR02	active river	modern
**Río Gallegos**	51.630252° S	69.615243° W	lower Río Gallegos	Pepper et al., 2016	RGALAR01	active river	modern
	51.700172° S	69.282634° W	lower Río Gallegos	Pepper et al., 2016	RGALAR02	active river	modern
**Río Santa Cruz**	50.003640° S	68.924979° W	lower Río Santa Cruz	Pepper et al., 2016	RSCRAR01	active river	modern
**Río Chico**	49.776628° S	68.642077° W	lower Río Chico	Pepper et al., 2016	RCHIAR02	active river	modern

**Río Deseado**	47.828961° S	66.594383° W	lower Río Deseado	Pepper et al., 2016	RDESAR01	active river	modern
	47.184053° S	67.247957° W	central Río Deseado	Pepper et al., 2016	RDESAR02	active river	modern

Supplementary Table T3

U-Pb Detrital Zircon Data

Analysis	U (ppm)	206Pb 204Pb	U/Th	206Pb* 207Pb*	± (%)	207Pb* 235U	± (%)	206Pb* 238U	± (%)	error corr.	206Pb* 238U	± (Ma)	207Pb* 235U	± (Ma)	206Pb* 207Pb*	± (Ma)	Best age (Ma)	± (Ma)	Conc (%)	Analysis Order	Lat.	Long.	Depth (cm)	Depth (m)	Modelled Age (Ma)
**19AR106**																					-26.94471	-65.76653	20	0.2	0.006
_19AR106_Spot_ 59	1257.621	39.65624	0.526612	4.371414	25.30778	-0.01014	48.06975	0.000233	3.975182	0.082696	1.501414	0.059677	NA	-4.9992	NA	NA	1.5	0.1		51					
_19AR106_Spot_ 200	692.9789	197.7744	0.818633	6.949604	25.78852	0.002944	89.93059	0.000299	9.46723	0.105273	1.924887	0.182206	2.985132	2.680611	972.7103	NA	1.9	0.2	0.2	175					
_19AR106_Spot_ 93	1161.72	161.2366	1.269509	6.233569	22.32008	0.00325	39.80242	0.00033	5.93693	0.14916	2.128939	0.126373	3.294247	1.309066	968.4225	835.4359	2.1	0.1	0.2	80					
_19AR106_Spot_ 502	976.1735	325.6	4.800908	6.453939	17.44969	0.005322	23.3007	0.000342	7.025642	0.301521	2.20277	0.154732	5.389758	1.252525	1848.101	407.1075	2.2	0.2	0.1	423					
_19AR106_Spot_ 363	1172.953	140.5956	1.591627	7.054431	33.63023	0.00166	222.7814	0.000344	6.210239	0.027876	2.216756	0.137642	1.684504	3.749668	NA	NA	2.2	0.1		307					
_19AR106_Spot_ 221	1771.166	481.2809	2.100489	6.396725	12.31079	0.006236	16.07056	0.000351	3.427915	0.213304	2.262513	0.077543	6.31226	1.011269	2082.972	278.0964	2.3	0.1	0.1	191					
_19AR106_Spot_ 491	2193.941	805.3909	5.892823	13.09852	14.73656	0.002894	31.14415	0.000362	3.171778	0.101842	2.334391	0.074028	2.934581	0.912631	529.1252	NA	2.3	0.1	0.4	413					
_19AR106_Spot_ 476	1571.982	263.0351	2.958264	8.029181	17.84196	0.003512	22.26636	0.000366	2.649536	0.118993	2.356739	0.062431	3.560132	0.791324	919.4532	459.9402	2.4	0.1	0.3	400					
_19AR106_Spot_ 103	2622.746	114.3859	0.901934	9.402852	12.57433	-0.00187	216.5025	0.000371	3.916809	0.018091	2.390593	0.093618	NA	-4.12033	NA	NA	2.4	0.1		88					
_19AR106_Spot_ 237	3123.945	733.3806	1.439873	13.39234	9.814574	0.002955	28.4833	0.000393	2.634309	0.092486	2.530144	0.066639	2.995569	0.85198	396.2358	NA	2.5	0.1	0.6	206					
_19AR106_Spot_ 126	2516.445	686.4435	1.419716	10.04504	14.708	0.004426	17.06441	0.000406	2.720315	0.159415	2.617252	0.071183	4.48431	0.763534	1174.298	335.6646	2.6	0.1	0.2	108					
_19AR106_Spot_ 591	1694.836	485.6899	2.289542	8.776472	11.86914	0.004985	23.24087	0.000427	4.000283	0.172123	2.752191	0.110072	5.048677	1.170445	1308.567	450.2527	2.8	0.1	0.2	497					
_19AR106_Spot_ 240	683.2076	356.3624	0.762294	6.102627	30.87467	0.009802	35.19978	0.000563	4.778316	0.135748	3.62884	0.173349	9.904507	3.46943	2047.223	637.4279	3.6	0.2	0.2	209					
_19AR106_Spot_ 245	1043.698	569.4549	3.770151	9.512146	25.03562	0.00799	32.66747	0.000724	2.854552	0.087382	4.664321	0.133097	8.080244	2.629142	1199.212	659.1435	4.7	0.1	0.4	213					
_19AR106_Spot_ 114	1782.657	1035.278	6.030303	11.29806	10.23443	0.007983	14.92856	0.000775	1.423319	0.095342	4.9914	0.071016	8.073506	1.20048	1062.566	300.5479	5.0	0.1	0.5	97					
_19AR106_Spot_ 7	2802.298	1766.015	4.498078	15.20799	5.461695	0.006228	7.722787	0.00079	1.245731	0.161306	5.088145	0.06336	6.3044	0.485367	500.4998	168.0374	5.1	0.1	1.0	7					
_19AR106_Spot_ 454	2886.719	2434.833	2.572872	14.95818	9.64015	0.007844	10.52343	0.000934	1.340755	0.127407	6.018623	0.080657	7.93326	0.831598	636.7571	225.1736	6.0	0.1	0.9	384					
_19AR106_Spot_ 28	502.1509	270.1506	4.539358	8.649344	13.52636	0.008331	41.53297	0.000976	4.713445	0.113487	6.291787	0.296415	8.423738	3.48417	670.6691	NA	6.3	0.3	0.9	25					
_19AR106_Spot_ 2	549.8027	460.3728	4.147085	7.886816	12.61096	0.01356	17.55179	0.001022	6.029547	0.343529	6.584323	0.396802	13.67574	2.38425	1553.164	311.6136	6.6	0.4	0.4	2					
_19AR106_Spot_ 598	291.1801	282.8738	4.751376	6.909345	31.00798	0.013559	37.82067	0.001031	8.882529	0.234859	6.645386	0.589974	13.67521	5.137429	1535.73	717.2692	6.6	0.6	0.4	503					
_19AR106_Spot_ 207	542.3718	395.9628	3.31979	7.421093	13.15215	0.014235	24.3186	0.001033	4.210477	0.173138	6.655328	0.280076	14.35234	3.46575	1623.971	452.2151	6.7	0.3	0.4	179					
_19AR106_Spot_ 10	818.6316	288.8204	3.487542	10.7299	11.56846	0.006093	26.33349	0.001065	1.747807	0.066372	6.864397	0.119913	6.167911	1.619305	NA	NA	6.9	0.1		10					
_19AR106_Spot_ 564	211.0238	278.7526	2.480918	8.710954	27.97537	0.010237	82.79579	0.001181	8.109658	0.097948	7.605869	0.616446	10.34168	8.519219	705.4708	NA	7.6	0.6	1.1	476					
_19AR106_Spot_ 99	698.6993	909.5601	2.31745	11.32599	26.58316	0.012029	27.34292	0.001209	3.360606	0.122906	7.785998	0.261499	12.14075	3.29988	992.0791	561.7634	7.8	0.3	0.8	85					
_19AR106_Spot_ 274	891.1211	271.6443	3.416506	9.928392	11.65115	0.007681	109.9773	0.001209	1.838676	0.016719	7.790956	0.143164	7.768953	8.511684	2.025993	NA	7.8	0.1	384.6	240					
_19AR106_Spot_ 488	324.5987	445.9625	4.153057	9.45545	8.184426	0.012669	15.16843	0.001252	6.717127	0.442836	8.063063	0.541268	12.78352	1.926907	1026.374	276.3107	8.1	0.5	0.8	410					
_19AR106_Spot_ 415	378.2664	230.9626	4.00736	9.21154	21.23202	0.007666	134.9709	0.001259	5.514259	0.040855	8.108233	0.446828	7.754184	10.4264	NA	NA	8.1	0.4		354					
_19AR106_Spot_ 55	192.3467	180.1665	1.518703	6.434749	27.50799	0.01804	38.01163	0.001705	6.029477	0.158622	10.98482	0.661763	18.15402	6.83942	1114.661	776.7623	11.0	0.7	1.0	47					
_19AR106_Spot_ 300	107.0821	194.5637	1.328415	4.851296	37.36814	0.03461	47.66482	0.001811	5.846341	0.122655	11.66465	0.681339	34.54788	16.19156	2210.487	877.4249	11.7	0.7	0.5	263					
_19AR106_Spot_ 282	236.004	371.9219	1.827819	5.1993	27.24559	0.039731	31.54011	0.001825	4.75486	0.150756	11.75592	0.558468	39.56166	12.23843	2433.686	543.1919	11.8	0.6	0.5	248					
_19AR106_Spot_ 150	218.1104	285.4438	1.913723	12.87042	10.0765	0.006255	131.7654	0.001849	4.015008	0.030471	11.9092	0.477714	6.331439	8.316876	NA	NA	11.9	0.5		131					
_19AR106_Spot_ 398	464.0503	656.4707	1.294049	13.7181	10.20165	0.012876	45.07651	0.001854	2.27524	0.050475	11.94176	0.271452	12.99071	5.818522	212.8666	NA	11.9	0.3	5.6	339					
_19AR106_Spot_ 506	477.0952	743.4164	0.423027	12.06069	13.43613	0.01632	24.62474	0.001873	3.50691	0.142414	12.06499	0.422713	16.43703	4.01501	715.3052	525.0276	12.1	0.4	1.7	426					
_19AR106_Spot_ 219	295.0373	429.1499	1.413071	8.698747	23.07968	0.021273	38.95291	0.00188	4.697229	0.120587	12.10807	0.56821	21.37389	8.238916	1247.977	787.267	12.1	0.6	1.0	189					
_19AR106_Spot_ 81	274.1082	339.8226	0.673525	6.850304	12.12388	0.02732	30.30766	0.001881	5.319468	0.175516	12.11209	0.643694	27.36812	8.184026	1721.368	561.3374	12.1	0.6	0.7	69					
_19AR106_Spot_ 27	1563.623	2443.212	0.379925	16.17489	5.270316	0.014539	7.287036	0.001891	1.017182	0.139588	12.17955	0.123771	14.65686	1.060379	443.7819	160.6129	12.2	0.1	2.7	24					
_19AR106_Spot_ 193	307.5502	459.5864	1.408603	13.07749	13.7827	0.01166	59.23879	0.001904	5.605168	0.09462	12.26177	0.68664	11.77095	6.932812	NA	NA	12.3	0.7		168					
_19AR106_Spot_ 270	432.5874	724.1198	0.961164	11.2114	17.46203	0.018169	31.08192	0.001909	2.954447	0.095054	12.29442	0.362886	18.28321	5.631974	900.4918	653.2213	12.3	0.4	1.4	236					
_19AR106_Spot_ 248	481.7143	790.9631	1.332479	13.23977	6.386277	0.015017	19.4896	0.001913	1.97623	0.101399	12.32059	0.243251	15.13508	2.927899	489.9108	431.366	12.3	0.2	2.5	216					
_19AR106_Spot_ 74	423.2396	564.4555	0.827746	11.49971	13.16577	0.016752	26.45915	0.001985	2.767213	0.104584	12.78412	0.353413	16.86873	4.42648	647.3695	574.4863	12.8	0.4	2.0	64					
_19AR106_Spot_ 109	150.2645	212.3744	1.046892	5.334591	39.72208	0.034934	52.75965	0.002038	6.4806	0.122832	13.12126	0.849471	34.86562	18.08466	2020.312	1008.185	13.1	0.8	0.6	93					
_19AR106_Spot_ 315	602.2163	952.3083	3.612513	13.05245	19.28634	0.018388	21.4847	0.002182	1.897363	0.088312	14.04985	0.266286	18.50139	3.938993	644.5415	464.8421	14.0	0.3	2.2	269					
_19AR106_Spot_ 577	175.122	485.8377	1.840389	8.318634	17.23154	0.027722	27.78133	0.002203	4.838521	0.174164	14.18385	0.685534	27.7651	7.609151	1453.216	530.6198	14.2	0.7	1.0	485					
_19AR106_Spot_ 85	1120.503	2805.787	5.794251	17.63788	4.351166	0.020261	10.99728	0.002859	0.842989	0.076654	18.40655	0.154944	20.36664	2.217467	259.3493	252.5637	18.4	0.2	7.1	72					
_19AR106_Spot_ 387	53.0242	255.7293	1.92407	8.339329	12.20378	0.0436	51.4746	0.005017	7.471371	0.145147	32.26096	2.404315	43.33315	21.83967	710.1679	NA	32.3	2.4	4.5	328					
_19AR106_Spot_ 329	125.594	427.2374	2.065678	9.842471	22.19371	0.050578	36.1596	0.005406	3.338712	0.092333	34.75715	1.157318	50.09919	17.6778	864.9488	771.0547	34.8	1.2	4.0	280					
_19AR106_Spot_ 588	241.5213	945.2256	0.807178	15.0739	7.685504	0.040181	12.64302	0.00576	4.191625	0.331537	37.02577	1.547533	40.00053	4.958993	223.295	NA	37.0	1.5	16.6	495					
_19AR106_Spot_ 190	162.1262	739.5305	0.601368	15.12825	8.967525	0.058319	25.45123	0.009126	1.682164	0.066094	58.56495	0.980697	57.55405	14.24173	16.7087	NA	58.6	1.0	350.5	166					
_19AR106_Spot_ 101	86.59339	632.0232	2.031555	11.44044	11.80755	0.087401	17.81258	0.00986	2.918155	0.163826	63.24743	1.836634	85.07923	14.53826	752.152	373.7264	63.2	1.8	8.4	87					
_19AR106_Spot_ 195	764.6421	6704.643	5.974983	16.8828	2.470228	0.083184	6.555876	0.010556	5.08355	0.775419	67.69422	3.423264	81.13397	5.112127	498.2954	91.22695	67.7	3.4	13.6	170					
_19AR106_Spot_ 295	178.9997	4893.875	5.02771	13.83099	3.153864	0.275615	4.045153	0.028893	1.43558	0.354889	183.6188	2.598806	247.173	8.874808	905.0938	77.95391	183.6	2.6	20.3	258					
_19AR106_Spot_ 559	206.2305	9337.027	2.911186	18.18969	3.742137	0.259174	4.387916	0.035146	0.758174	0.172787	222.6764	1.659449	234.0014	9.170779	350.2956	97.72597	222.7	1.7	63.6	473					
_19AR106_Spot_ 480	326.4806	10390.32	1.783648	19.43178	1.658107	0.293081	2.685912	0.042632	0.758653	0.282456	269.1273	1.99971	260.9816	6.181431	189.4484	59.96713	269.1	2.0	142.1	403					
_19AR106_Spot_ 474	14.18265	490.0724	0.721495	6.819795	10.93426	0.699523	15.23159	0.042722	4.026025	0.264321	269.6858	10.63365	538.5058	63.74117	1938.308	264.3398	269.7	10.6	13.9	399					
_19AR106_Spot_ 79	289.5274	9102.648	1.720356	18.7214	2.184225	0.316157	3.568278	0.044407	0.775761	0.217405	280.0916	2.126313	278.9424	8.703515	270.2916	79.87345	280.1	2.1	103.6	67					
_19AR106_Spot_ 531	82.62202	4008.609	2.136291	17.87667	3.794525	0.330344	6.23172	0.046051	0.97051	0.155737	290.2288	2.754235	289.8283	15.71357	287.5983	140.8173	290.2	2.8	100.9	446					
_19AR106_Spot_ 573	215.9668	11514.68	1.800133	18.58378	1.57839	0.394631	2.777534	0.05447	0.749444	0.269823	341.9097	2.495652	337.7465	7.980495	310.1756	60.88607	341.9	2.5	110.2	481					
_19AR106_Spot_ 373	246.749	9608.43	2.585822	17.45165	1.476027	0.475976	1.849835	0.06205	1.018131	0.55039	388.0812	3.834596	395.3083	6.057226	438.7434	34.37355	388.1	3.8	88.5	317					
_19AR106_Spot_ 77	285.5764	14323.57	13.30276	17.34504	1.576137	0.50236	1.917643	0.064363	0.828766	0.432179	402.1025	3.230682	413.2984	6.51095	477.246	38.23308	402.1	3.2	84.3	66					
_19AR106_Spot_ 100	263.7021	1545.419	0.459626	16.38231	1.305313	0.495606	14.85653	0.069982	0.826294	0.055618	436.0463	3.483867	408.7239	50.02858	258.1671	NA	436.0	3.5	168.9	86					
_19AR106_Spot_ 336	49.22047	3245.1	2.555474	15.92993	4.37137	0.587329	5.789829	0.073274	1.27967	0.22102	455.8518	5.631927	469.1603	21.75587	535.7393	123.6774	455.9	5.6	85.1	286					
_19AR106_Spot_ 156	342.0599	14208.07	14.20346	17.47169	1.01223	0.576914	2.101883	0.07374	0.78641	0.374145	458.649	3.48154	462.4761	7.808184	482.5102	43.05734	458.6	3.5	95.1	136					
_19AR106_Spot_ 462	535.4264	36185.97	29.54501	17.26496	0.784653	0.58957	1.54312	0.074415	1.312029	0.850244	462.7019	5.858034	470.5927	5.811523	510.2019	17.86544	462.7	5.9	90.7	389					
_19AR106_Spot_ 420	195.3448	9963.678	0.989254	17.89624	1.395947	0.557065	1.838204	0.074429	0.653803	0.355675	462.7853	2.91965	449.6141	6.677732	383.7502	38.58984	462.8	2.9	120.6	357					
_19AR106_Spot_ 214	293.4757	1644.553	17.03354	15.92668	1.083908	0.558403	3.335921	0.075154	1.06073	0.317972	467.1339	4.779759	450.4866	12.13765	367.3362	71.26746	467.1	4.8	127.2	185					
_19AR106_Spot_ 157	151.748	1257.091	1.31808	15.41745	1.942581	0.557897	8.80416	0.075413	0.928205	0.105428	468.6845	4.195973	450.1567	32.02411	357.5423	197.9739	468.7	4.2	131.1	137					
_19AR106_Spot_ 551	280.0116	13718.34	34.47543	17.58508	1.473346	0.580957	2.108865	0.075536	0.776791	0.368345	469.4225	3.51683	465.0764	7.868853	444.6221	43.61308	469.4	3.5	105.6	466					
_19AR106_Spot_ 131	184.4209	9583.771	5.699241	17.05969	1.527967	0.596729	2.878139	0.075742	0.763873	0.265405	470.6568	3.467114	475.1559	10.92206	497.8741	61.14948	470.7	3.5	94.5	113					
_19AR106_Spot_ 1	228.8178	2452.597	2.61691	16.7798	1.136003	0.557693	2.004573	0.075764	0.863662	0.430846	470.788	3.921093	450.024	7.287405	346.2679	40.91992	470.8	3.9	136.0	1					
_19AR106_Spot_ 87	268.1014	5743.005	4.146999	17.30873	1.47429	0.576787	2.11421	0.075831	0.740711	0.350349	471.1894	3.365648	462.3947	7.852886	419.892	44.23956	471.2	3.4	112.2	74					
_19AR106_Spot_ 446	193.1545	9553.633	1.662171	17.474	1.582404	0.584412	2.102818	0.075919	0.766976	0.364737	471.7203	3.48878	467.2925	7.875735	446.544	43.52286	471.7	3.5	105.6	378					
_19AR106_Spot_ 275	25.16628	411.8105	3.046183	14.05216	5.489933	0.36452	29.42989	0.076033	1.577876	0.053615	472.4014	7.187347	315.5838	79.99397	NA	NA	472.4	7.2		241					
_19AR106_Spot_ 227	452.861	25743.68	1.41553	17.44032	0.812757	0.595642	1.045754	0.076062	0.60953	0.582861	472.5716	2.777418	474.4645	3.9638	484.6095	18.75508	472.6	2.8	97.5	197					
_19AR106_Spot_ 39	311.5129	8460.146	2.404533	17.48673	0.887559	0.581413	2.28563	0.076132	0.704281	0.308134	472.9927	3.211922	465.3688	8.532675	428.8733	48.48072	473.0	3.2	110.3	34					
_19AR106_Spot_ 421	241.9512	17517.67	1.817019	17.55595	1.323013	0.588337	1.846994	0.076204	0.679995	0.368163	473.4251	3.103899	469.8049	6.946811	453.103	38.12537	473.4	3.1	104.5	358					
_19AR106_Spot_ 186	90.12242	3632.063	2.297715	17.05113	2.95862	0.575748	5.069298	0.076333	1.157352	0.228306	474.1971	5.291135	461.7251	18.80932	401.0994	110.617	474.2	5.3	118.2	162					
_19AR106_Spot_ 152	573.6346	9242.807	2.353908	17.35669	0.862959	0.594082	2.382538	0.076349	0.75626	0.317418	474.2962	3.458137	473.4713	9.016065	470.4612	50.01281	474.3	3.5	100.8	133					
_19AR106_Spot_ 246	201.104	10309.67	1.42093	17.51557	1.428974	0.587949	1.792384	0.076775	0.684071	0.381654	476.8441	3.144227	469.5573	6.738614	435.0422	36.9024	476.8	3.1	109.6	214					
_19AR106_Spot_ 185	200.5215	9731.379	4.901222	17.51957	1.800938	0.590812	2.430065	0.076991	0.881983	0.362946	478.1402	4.064518	471.3864	9.16411	439.5857	50.39443	478.1	4.1	108.8	161					
_19AR106_Spot_ 525	205.7511	9383.938	12.68483	17.55421	1.236762	0.585141	1.939304	0.076996	0.853474	0.440093	478.1668	3.933348	467.7598	7.269017	417.9525	38.89816	478.2	3.9	114.4	440					
_19AR106_Spot_ 6	280.924	15550.35	12.41211	17.33122	0.957144	0.601275	1.230637	0.077208	0.694178	0.56408	479.4349	3.207381	478.0426	4.692133	472.3322	22.48391	479.4	3.2	101.5	6					
_19AR106_Spot_ 184	86.84748	2992.646	1.345809	16.78859	2.632478	0.583536	4.791715	0.077281	0.906473	0.189175	479.8717	4.19195	466.7311	17.93104	403.5496	105.4229	479.9	4.2	118.9	160					
_19AR106_Spot_ 553	207.5257	16482.21	34.98631	17.51722	1.287218	0.600585	1.517351	0.077416	0.717561	0.472904	480.6783	3.323706	477.6049	5.781179	463.8592	29.6469	480.7	3.3	103.6	468					
_19AR106_Spot_ 242	89.12176	5443.943	1.939614	16.57111	3.052251	0.614745	4.729855	0.077483	0.624718	0.13208	481.0821	2.896004	486.5481	18.28587	513.3389	103.0591	481.1	2.9	93.7	211					
_19AR106_Spot_ 486	59.29118	3866.953	2.8306	16.97344	3.754464	0.587378	4.395812	0.077563	0.73514	0.167237	481.5576	3.411129	469.1921	16.51752	410.0955	96.97697	481.6	3.4	117.4	409					
_19AR106_Spot_ 584	251.7042	19006.95	12.89791	17.60972	1.316071	0.596928	1.787322	0.077571	0.813896	0.455372	481.6087	3.776952	475.2825	6.783845	445.8095	35.3722	481.6	3.8	108.0	491					
_19AR106_Spot_ 60	160.6946	8285.116	6.590141	17.49452	1.721144	0.59291	3.353994	0.077629	0.759095	0.226326	481.9532	3.525068	472.7243	12.67688	429.1213	72.86646	482.0	3.5	112.3	52					
_19AR106_Spot_ 587	209.8045	9454.528	11.67833	17.51933	1.449311	0.592565	1.962339	0.077672	0.908326	0.462879	482.212	4.220247	472.5046	7.413967	426.5828	38.8079	482.2	4.2	113.0	494					
_19AR106_Spot_ 530	260.8565	10664.3	1.837003	17.80915	0.868088	0.584567	1.351069	0.077774	0.687886	0.509142	482.8249	3.199952	467.3919	5.060977	393.2361	26.08629	482.8	3.2	122.8	445					
_19AR106_Spot_ 116	76.53066	4527.159	1.438973	17.06883	3.145413	0.593912	3.944094	0.077779	0.864072	0.21908	482.8497	4.019749	473.3626	14.92334	428.5843	85.8154	482.8	4.0	112.7	99					
_19AR106_Spot_ 404	323.6606	20285.63	19.4757	17.60352	1.053878	0.600159	1.361721	0.077794	0.609957	0.447931	482.9446	2.838117	477.3348	5.185912	451.4142	27.03647	482.9	2.8	107.0	344					
_19AR106_Spot_ 208	125.7483	4872.077	3.766055	17.90558	2.014555	0.567588	5.482617	0.077853	0.700995	0.127858	483.2925	3.263976	456.4533	20.15933	324.4642	123.5313	483.3	3.3	149.0	180					
_19AR106_Spot_ 403	150.1053	6873.759	2.013292	17.73931	1.782616	0.582283	3.325399	0.078037	0.741633	0.223021	484.3933	3.460769	465.9275	12.4264	376.8794	72.92265	484.4	3.5	128.5	343					
_19AR106_Spot_ 432	295.7083	14485.26	10.96395	17.40421	1.22585	0.606771	1.470495	0.078072	0.715833	0.486797	484.6036	3.341773	481.5214	5.638565	467.8213	28.46212	484.6	3.3	103.6	365					
_19AR106_Spot_ 124	257.862	22590.62	12.51852	17.43624	1.327186	0.611833	1.890037	0.07808	1.083323	0.573175	484.6518	5.057834	484.7155	7.284848	485.9815	34.1898	484.7	5.1	99.7	106					
_19AR106_Spot_ 239	141.4292	10354.94	5.246357	18.21789	1.91016	0.574427	2.171572	0.078083	0.580101	0.267134	484.6689	2.708475	460.8738	8.044995	344.9294	47.33355	484.7	2.7	140.5	208					
_19AR106_Spot_ 117	249.6983	12473.06	20.06852	17.61794	1.235318	0.598741	1.46437	0.078095	0.726048	0.495809	484.7401	3.390378	476.4344	5.5686	437.5902	28.31473	484.7	3.4	110.8	100					
_19AR106_Spot_ 334	218.6563	24515.28	13.87103	17.43389	1.276706	0.609558	1.661153	0.078104	0.784293	0.472137	484.7954	3.662762	483.2812	6.387835	477.0461	32.38711	484.8	3.7	101.6	284					
_19AR106_Spot_ 490	517.9638	16101.44	1.598699	17.55441	0.849603	0.600942	1.933303	0.078128	0.740659	0.383106	484.9415	3.459992	477.8314	7.368762	444.7945	39.725	484.9	3.5	109.0	412					
_19AR106_Spot_ 369	247.5774	14077.01	6.188229	17.44851	1.291107	0.605594	1.837487	0.078197	0.677437	0.368676	485.3529	3.167232	480.7775	7.037316	459.9968	37.87589	485.4	3.2	105.5	313					
_19AR106_Spot_ 534	145.0972	8178.289	16.02407	17.79577	2.019277	0.58414	3.341621	0.078209	0.772491	0.231172	485.4252	3.612157	467.1183	12.51216	379.0731	73.11679	485.4	3.6	128.1	449					
_19AR106_Spot_ 391	262.1069	14970.53	30.29944	17.84392	1.127031	0.59271	2.094376	0.078227	0.545739	0.260574	485.5297	2.552398	472.5967	7.914049	411.2336	45.23504	485.5	2.6	118.1	332					
_19AR106_Spot_ 381	140.944	8278.026	7.334949	17.7227	2.073375	0.588455	2.670841	0.078238	0.659737	0.247015	485.5996	3.085989	469.8804	10.04686	394.7635	58.04755	485.6	3.1	123.0	323					
_19AR106_Spot_ 368	159.4254	9927.786	3.491724	17.56632	1.300377	0.59854	2.416478	0.078292	0.742105	0.307102	485.9201	3.473482	476.307	9.187438	431.2216	51.27462	485.9	3.5	112.7	312					
_19AR106_Spot_ 112	116.4257	6004.663	2.911316	17.28984	2.412858	0.598026	3.059657	0.078364	0.809896	0.264702	486.3526	3.794031	475.98	11.62673	427.2458	65.82007	486.4	3.8	113.8	95					
_19AR106_Spot_ 57	130.6414	4789.742	3.3782	17.62969	2.838351	0.581325	3.172849	0.078396	0.636851	0.200719	486.5406	2.984496	465.3124	11.84396	362.8281	70.09434	486.5	3.0	134.1	49					
_19AR106_Spot_ 546	265.2358	16216.27	14.37085	17.70294	1.091044	0.598631	1.962838	0.078399	0.76428	0.389375	486.5619	3.581823	476.3646	7.463339	428.5058	40.31685	486.6	3.6	113.5	461					
_19AR106_Spot_ 236	131.745	5588.718	5.023352	17.8036	1.853624	0.577814	2.881778	0.078415	0.657009	0.227987	486.6571	3.079671	463.0558	10.71615	348.6326	63.43085	486.7	3.1	139.6	205					
_19AR106_Spot_ 317	220.9202	14405.95	10.87648	17.53591	1.078162	0.603225	1.44653	0.078419	0.672452	0.464873	486.6763	3.152181	479.2781	5.526452	444.9818	28.48461	486.7	3.2	109.4	271					
_19AR106_Spot_ 392	92.88072	5737.338	5.065727	17.22346	2.376147	0.599139	4.044268	0.078429	0.980224	0.242374	486.7394	4.595465	476.687	15.38666	429.5513	87.47943	486.7	4.6	113.3	333					
_19AR106_Spot_ 183	248.8237	10186.55	7.609487	17.44659	1.133138	0.605996	2.122697	0.078537	0.879644	0.414399	487.3841	4.129183	481.0315	8.13303	451.8108	42.90046	487.4	4.1	107.9	159					
_19AR106_Spot_ 600	267.3634	12662.64	7.772223	17.24346	0.998187	0.613583	1.602064	0.078559	0.640387	0.399726	487.5149	3.006851	485.8171	6.185815	478.7571	32.47846	487.5	3.0	101.8	505					
_19AR106_Spot_ 254	44.52923	2030.346	2.516986	16.22784	4.511481	0.587977	6.572581	0.078571	0.721561	0.109784	487.5887	3.388489	469.5746	24.71539	383.4116	146.939	487.6	3.4	127.2	222					
_19AR106_Spot_ 67	61.04476	2265.602	1.973846	16.77787	3.462757	0.576602	4.534441	0.078593	0.86375	0.190487	487.7207	4.057272	462.2756	16.84022	338.7069	100.8437	487.7	4.1	144.0	58					
_19AR106_Spot_ 151	113.7609	3913.989	8.48113	17.46646	2.636715	0.582905	3.18081	0.0786	0.809398	0.254463	487.7626	3.802278	466.3265	11.89407	363.0753	69.36975	487.8	3.8	134.3	132					
_19AR106_Spot_ 481	135.6936	7756.342	1.299656	17.7674	2.045308	0.587706	2.801555	0.078647	0.676908	0.241619	488.0425	3.181643	469.4015	10.53015	380.2042	61.11187	488.0	3.2	128.4	404					
_19AR106_Spot_ 118	176.7561	3312.269	2.687659	16.59771	1.755637	0.605623	2.854547	0.078706	0.694648	0.243348	488.397	3.26731	480.7957	10.93308	445.6544	61.57619	488.4	3.3	109.6	101					
_19AR106_Spot_ 507	55.77509	3353.259	2.838529	15.82526	3.293344	0.636563	7.536946	0.07871	0.840425	0.111507	488.4158	3.953124	500.1762	29.77546	555.281	163.5962	488.4	4.0	88.0	427					
_19AR106_Spot_ 578	106.3038	6349.87	2.113468	17.20091	1.779178	0.604744	2.118052	0.078753	0.889668	0.420041	488.6732	4.186871	480.2399	8.104786	441.1193	42.78471	488.7	4.2	110.8	486					
_19AR106_Spot_ 247	127.528	10491.6	7.099001	17.2152	1.419234	0.614577	2.608702	0.078805	0.606947	0.232662	488.9856	2.858114	486.4428	10.08292	475.4411	56.13568	489.0	2.9	102.8	215					
_19AR106_Spot_ 409	176.4049	6579.115	6.790603	17.52165	1.773853	0.594325	3.036289	0.078824	0.653368	0.215186	489.1024	3.077417	473.6261	11.49314	400.2879	66.44239	489.1	3.1	122.2	349					
_19AR106_Spot_ 337	129.463	7047.206	7.33335	17.58041	2.804886	0.59409	3.898805	0.078835	0.804297	0.206293	489.1638	3.788764	473.4762	14.75473	399.1069	85.51178	489.2	3.8	122.6	287					
_19AR106_Spot_ 113	203.6837	10944.69	1.472111	17.75655	1.907211	0.598225	2.625601	0.078889	0.788535	0.300326	489.4874	3.71688	476.1068	9.979282	413.0957	56.00309	489.5	3.7	118.5	96					
_19AR106_Spot_ 366	143.2484	6806.439	26.79161	17.18669	1.994162	0.610733	2.907966	0.07892	0.775376	0.266639	489.6736	3.656192	484.0226	11.19605	458.325	62.17376	489.7	3.7	106.8	310					
_19AR106_Spot_ 121	171.005	14672.43	1.553212	17.05198	1.532422	0.627546	1.774844	0.078925	0.752412	0.423931	489.7035	3.548117	494.5658	6.948788	518.0747	35.26884	489.7	3.5	94.5	103					
_19AR106_Spot_ 372	270.0404	14509.81	66.16096	17.77154	0.984345	0.601254	1.468643	0.079059	0.611756	0.416545	490.5017	2.889355	478.0295	5.599491	419.5781	29.81472	490.5	2.9	116.9	316					
_19AR106_Spot_ 375	91.61956	5460.555	2.873708	17.6952	2.445119	0.584941	3.717995	0.079085	0.738576	0.198649	490.656	3.489391	467.6319	13.93365	357.0864	82.28817	490.7	3.5	137.4	318					
_19AR106_Spot_ 354	60.73717	3156.639	1.609844	17.32065	4.341508	0.579379	5.778012	0.079086	0.741563	0.128342	490.6624	3.503547	464.0622	21.52533	335.4469	129.9488	490.7	3.5	146.3	299					
_19AR106_Spot_ 580	276.2261	10982.63	18.38295	17.67741	1.17011	0.600769	2.333384	0.079151	0.936533	0.401363	491.0536	4.428086	477.7213	8.892132	415.1625	47.78599	491.1	4.4	118.3	488					
_19AR106_Spot_ 493	275.005	15204.52	6.695223	17.71223	0.955035	0.603159	1.981552	0.07921	0.78365	0.395473	491.4055	3.707785	479.2365	7.57005	422.3696	40.64411	491.4	3.7	116.3	415					
_19AR106_Spot_ 298	56.79531	3443.239	1.038686	16.53806	3.403213	0.612946	3.967203	0.079259	1.049922	0.264651	491.6968	4.970467	485.4166	15.3091	456.8185	84.91791	491.7	5.0	107.6	261					
_19AR106_Spot_ 503	146.5255	11131.06	3.075847	17.82811	2.885795	0.596616	3.143924	0.079308	0.584511	0.185918	491.9898	2.768735	475.084	11.92934	395.2079	69.26642	492.0	2.8	124.5	424					
_19AR106_Spot_ 161	279.2174	27345.2	5.917553	17.53421	1.38654	0.621514	1.724822	0.079338	0.686773	0.39817	492.1692	3.254275	490.7956	6.712906	485.3486	34.93159	492.2	3.3	101.4	141					
_19AR106_Spot_ 21	135.078	6924.388	6.866352	17.69547	2.360784	0.594327	2.633459	0.079342	0.884148	0.335736	492.1933	4.189736	473.6271	9.968238	385.6152	55.72839	492.2	4.2	127.6	18					
_19AR106_Spot_ 311	89.00452	5000.845	1.535914	17.28066	1.962495	0.599259	3.814164	0.079356	0.715145	0.187497	492.2781	3.389438	476.7637	14.51292	403.7503	83.90184	492.3	3.4	121.9	265					
_19AR106_Spot_ 430	106.9718	8041.722	3.779568	17.71501	2.348666	0.597027	3.769241	0.079373	0.837212	0.222117	492.3765	3.968741	475.3452	14.3085	394.9212	82.42743	492.4	4.0	124.7	364					
_19AR106_Spot_ 332	107.1697	8573.926	1.40124	17.17117	3.66232	0.618742	3.848394	0.079447	0.869846	0.226028	492.8208	4.12702	489.0587	14.93733	472.4145	82.97162	492.8	4.1	104.3	282					
_19AR106_Spot_ 48	179.9454	9102.071	1.876369	17.61507	1.260381	0.603925	2.564388	0.07948	0.708349	0.276225	493.0179	3.362085	479.7215	9.804515	417.6073	55.07086	493.0	3.4	118.1	43					
_19AR106_Spot_ 356	133.3578	997.9915	3.976722	15.66676	2.513061	0.538676	22.8763	0.079496	0.73978	0.032338	493.1116	3.511911	437.5509	81.49427	155.7902	NA	493.1	3.5	316.5	301					
_19AR106_Spot_ 182	223.4346	9569.067	8.108461	17.41703	1.431314	0.612875	2.073872	0.079549	0.915305	0.441351	493.4285	4.347853	485.3718	8.001882	448.4532	41.36005	493.4	4.3	110.0	158					
_19AR106_Spot_ 358	339.6316	7467.791	7.785353	17.43897	1.162048	0.609363	2.117086	0.079597	0.834714	0.394275	493.7141	3.967243	483.1582	8.139537	434.3318	43.33597	493.7	4.0	113.7	303					
_19AR106_Spot_ 352	151.6315	10105.47	2.256824	18.0652	1.754668	0.592813	3.068359	0.079663	0.777673	0.253449	494.111	3.698996	472.6623	11.59599	370.7926	66.86022	494.1	3.7	133.3	297					
_19AR106_Spot_ 145	141.5825	8716.617	6.137417	17.48055	1.389363	0.614246	2.875977	0.079743	0.735407	0.255707	494.5912	3.501228	486.2343	11.11233	447.9844	61.80655	494.6	3.5	110.4	127					
_19AR106_Spot_ 273	127.4322	1954.834	8.559222	17.64836	2.412229	0.538627	5.052195	0.079813	0.73774	0.146024	495.0065	3.515174	437.5192	17.96017	146.2694	117.2819	495.0	3.5	338.4	239					
_19AR106_Spot_ 180	173.9634	4599.819	3.276248	17.31403	1.385127	0.602444	3.749754	0.080067	0.738549	0.196959	496.5227	3.529395	478.7836	14.31513	395.6433	82.43721	496.5	3.5	125.5	156					

_19AR106_Spot_ 357	118.1211	6249.049	1.962844	17.37576	2.330339	0.61067	4.063317	0.080117	0.727485	0.179037	496.8238	3.478554	483.9828	15.6439	424.5687	89.2142	496.8	3.5	117.0	302
_19AR106_Spot_ 176	287.5889	13954.69	8.703547	17.42468	1.209868	0.625333	1.499771	0.080186	0.745883	0.497331	497.2322	3.569342	493.1847	5.859071	475.3997	28.7958	497.2	3.6	104.6	152
_19AR106_Spot_ 595	218.9183	12700.58	1.628156	17.52945	1.462051	0.616418	1.665995	0.080257	0.70122	0.420902	497.6546	3.358357	487.5996	6.45106	441.5684	33.62015	497.7	3.4	112.7	500
_19AR106_Spot_ 365	105.3643	5565.215	1.973405	17.14342	2.953788	0.619194	4.457335	0.080489	0.893582	0.200475	499.0391	4.291089	489.3418	17.30912	445.1413	97.12085	499.0	4.3	112.1	309
_19AR106_Spot_ 498	104.9548	6975.479	3.00536	17.33791	2.565959	0.615575	2.945742	0.08054	0.681746	0.231434	499.3452	3.275757	487.0699	11.39716	430.675	63.90045	499.3	3.3	115.9	420
_19AR106_Spot_ 222	208.1939	9439.128	3.6349	17.17658	1.461918	0.629931	1.731415	0.080575	0.683331	0.394666	499.5568	3.28471	496.0527	6.794559	480.8528	35.14925	499.6	3.3	103.9	192
_19AR106_Spot_ 38	72.78385	4858.809	1.136743	17.64819	3.082259	0.595846	5.068334	0.080599	0.786703	0.155219	499.6961	3.782626	474.5942	19.2172	355.9949	113.1208	499.7	3.8	140.4	33
_19AR106_Spot_ 575	83.96159	5510.194	2.59643	17.15682	3.247307	0.620461	5.109522	0.081064	0.841573	0.164707	502.4707	4.068057	490.1359	19.86741	433.8403	112.3332	502.5	4.1	115.8	483
_19AR106_Spot_ 35	104.7093	9219.119	3.954423	17.86482	2.30803	0.612022	3.085916	0.081586	0.891261	0.288816	505.5803	4.333871	484.8348	11.8968	388.8783	66.33552	505.6	4.3	130.0	30
_19AR106_Spot_ 297	207.9285	14520.75	9.432482	17.39257	1.347879	0.639673	1.675565	0.082343	0.734278	0.438227	510.0953	3.601161	502.1038	6.637403	466.7913	33.37845	510.1	3.6	109.3	260
_19AR106_Spot_ 130	99.18136	5195.157	2.968704	17.10596	2.714214	0.634729	3.425064	0.082656	0.74399	0.217219	511.9569	3.661589	499.0376	13.50414	441.1584	74.39224	512.0	3.7	116.0	112
_19AR106_Spot_ 597	179.7601	15351.27	20.02194	17.12226	1.589967	0.654783	2.227674	0.082942	0.719089	0.322798	513.6614	3.550357	511.4177	8.950537	502.3576	46.42597	513.7	3.6	102.3	502
_19AR106_Spot_ 541	424.5022	4158.027	10.94664	16.90539	0.908337	0.634707	1.673364	0.083033	0.745464	0.445488	514.1984	3.684273	499.0238	6.5972	430.9604	33.38796	514.2	3.7	119.3	456
_19AR106_Spot_ 123	38.41214	2268.471	14.41897	15.34701	3.248776	0.67412	7.016474	0.083096	1.030628	0.146887	514.5746	5.097205	523.2146	28.69559	562.0323	151.3922	514.6	5.1	91.6	105
_19AR106_Spot_ 451	127.7541	7961.963	1.568029	17.70913	1.763497	0.628805	2.503229	0.083215	0.815305	0.325701	515.2816	4.0376	495.3513	9.812755	405.2059	53.00131	515.3	4.0	127.2	382
_19AR106_Spot_ 461	211.1015	18584.08	7.685027	17.4178	1.108051	0.657401	1.738844	0.084338	0.753941	0.433587	521.9627	3.780186	513.0228	7.003257	474.3698	34.66097	522.0	3.8	110.0	388
_19AR106_Spot_ 504	312.3135	31721.5	9.783975	17.26451	0.878567	0.667183	1.194026	0.084548	0.685838	0.574391	523.2105	3.446617	518.9982	4.851865	501.461	21.51901	523.2	3.4	104.3	425
_19AR106_Spot_ 238	110.0873	5756.784	3.636642	17.07158	2.865854	0.652116	3.022265	0.084558	0.747233	0.247243	523.2693	3.755554	509.7802	12.11344	450.671	65.04971	523.3	3.8	116.1	207
_19AR106_Spot_ 440	308.1638	2147.387	2.498727	16.18413	1.188383	0.642728	5.72769	0.084662	0.679194	0.118581	523.8893	3.417479	503.9937	22.7585	415.6208	127.1836	523.9	3.4	126.0	372
_19AR106_Spot_ 540	263.8663	19633.6	1.410377	17.05499	0.995902	0.675236	1.458025	0.084912	0.756157	0.518617	525.3734	3.815076	523.8909	5.967323	518.368	27.37111	525.4	3.8	101.4	455
_19AR106_Spot_ 562	212.3912	21087	5.266689	17.2703	1.470997	0.672872	1.642452	0.084986	0.675145	0.411059	525.8179	3.409106	522.4572	6.708088	508.7408	32.91516	525.8	3.4	103.4	475
_19AR106_Spot_ 511	141.0258	10775.75	2.036123	17.61918	1.723816	0.646659	2.11567	0.084995	0.762764	0.360531	525.8671	3.851879	506.4204	8.436441	420.4743	44.07059	525.9	3.9	125.1	430
_19AR106_Spot_ 433	88.86036	8611.072	1.474325	17.63776	3.128899	0.645321	3.394314	0.085074	0.754803	0.222373	526.3385	3.814959	505.5951	13.51863	413.7633	73.98464	526.3	3.8	127.2	366
_19AR106_Spot_ 107	160.5108	10439.68	3.42798	16.99924	2.38731	0.671692	2.772753	0.085136	0.785339	0.283234	526.7038	3.971939	521.7406	11.31287	501.0515	58.57741	526.7	4.0	105.1	91
_19AR106_Spot_ 4	165.4688	11510.07	2.675668	16.81787	1.599316	0.679869	1.848554	0.085147	0.680506	0.368129	526.769	3.442144	526.695	7.59661	527.3499	37.65412	526.8	3.4	99.9	4
_19AR106_Spot_ 425	288.8561	19371.37	0.969224	17.12664	1.100614	0.675122	1.523541	0.085154	0.768211	0.504227	526.8155	3.886102	523.8216	6.23484	511.7283	28.90632	526.8	3.9	102.9	360
_19AR106_Spot_ 272	134.144	2469.389	3.62202	16.85853	1.783415	0.624783	3.391052	0.085248	0.621834	0.183375	527.3697	3.148808	492.8408	13.24106	336.4082	75.53624	527.4	3.1	156.8	238
_19AR106_Spot_ 550	126.6623	7712.271	2.055535	17.9647	2.070286	0.632326	3.310532	0.085322	0.831801	0.251259	527.8134	4.215431	497.5435	13.02223	361.5401	72.28734	527.8	4.2	146.0	465
_19AR106_Spot_ 296	170.4897	9616.937	2.582171	17.37122	1.793465	0.657871	2.057067	0.085352	0.752675	0.365897	527.9891	3.815649	513.3108	8.288537	449.4167	42.55127	528.0	3.8	117.5	259
_19AR106_Spot_ 23	114.7132	8215.092	2.904636	17.68122	2.631547	0.643567	3.322097	0.08539	0.82507	0.248358	528.2178	4.184393	504.5123	13.2091	399.365	72.12228	528.2	4.2	132.3	20
_19AR106_Spot_ 292	150.4676	10674.08	5.172298	17.39496	2.029076	0.659426	2.178785	0.085472	0.725811	0.333126	528.7041	3.684244	514.2631	8.791509	451.532	45.63156	528.7	3.7	117.1	255
_19AR106_Spot_ 333	129.6991	8510.563	2.464027	17.20279	2.325854	0.664401	2.732393	0.085545	0.83282	0.304795	529.136	4.230742	517.3026	11.07548	466.3159	57.64979	529.1	4.2	113.5	283
_19AR106_Spot_ 266	190.0556	13548.82	4.844713	17.29723	1.461847	0.667162	2.056877	0.085588	0.866265	0.421155	529.3909	4.402677	518.9856	8.357987	474.4079	41.26628	529.4	4.4	111.6	232
_19AR106_Spot_ 521	214.2504	11052.53	1.639682	17.04275	1.292565	0.673478	1.732479	0.085611	0.856197	0.494203	529.5285	4.352592	522.8247	7.079593	494.6118	33.22014	529.5	4.4	107.1	437
_19AR106_Spot_ 115	393.5254	33624.27	22.6209	17.18846	0.903132	0.682195	1.285991	0.085661	0.721602	0.561125	529.823	3.670318	528.1	5.29546	521.5941	23.36135	529.8	3.7	101.6	98
_19AR106_Spot_ 196	241.8748	11460.11	2.3224	17.11845	1.134534	0.676332	2.105234	0.085691	0.663271	0.315058	530.0017	3.374717	524.555	8.624632	501.8715	43.96865	530.0	3.4	105.6	171
_19AR106_Spot_ 148	122.6601	1750.113	1.920253	16.20673	2.124306	0.634476	4.242265	0.085727	0.757865	0.178646	530.2191	3.857528	498.8801	16.72259	358.5116	94.25014	530.2	3.9	147.9	129
_19AR106_Spot_ 56	104.3633	6033.653	3.934437	17.67699	1.929623	0.643053	4.386261	0.08576	0.654651	0.14925	530.4124	3.333336	504.1944	17.4326	387.8841	97.40734	530.4	3.3	136.7	48
_19AR106_Spot_ 232	140.6587	8246.701	1.11305	17.53596	2.284223	0.652808	3.650997	0.085803	0.713999	0.195563	530.6652	3.637185	510.2053	14.64315	420.4846	79.95949	530.7	3.6	126.2	202
_19AR106_Spot_ 36	187.6182	12271.38	4.945102	17.39058	1.563536	0.666488	1.988091	0.085813	0.881114	0.443196	530.725	4.488971	518.575	8.073568	466.3661	39.48575	530.7	4.5	113.8	31
_19AR106_Spot_ 443	399.5373	20652.87	2.497414	17.18004	0.802287	0.681348	1.111817	0.085847	0.704012	0.633208	530.9264	3.588002	527.5886	4.574857	514.1118	18.89848	530.9	3.6	103.3	375
_19AR106_Spot_ 537	599.9822	28812.08	4.017162	17.27745	0.835295	0.67623	1.15812	0.08586	0.781285	0.674615	531.0078	3.982415	524.4931	4.744029	497.194	18.83543	531.0	4.0	106.8	452
_19AR106_Spot_ 24	99.02541	5242.253	1.739514	16.71202	2.691049	0.674613	4.177366	0.085863	0.842895	0.201777	531.0238	4.29658	523.513	17.08887	491.865	90.25806	531.0	4.3	108.0	21
_19AR106_Spot_ 529	272.8324	13844.05	13.50755	17.17737	1.01207	0.673642	1.604476	0.08589	0.825954	0.514781	531.1852	4.211449	522.9242	6.55746	487.9473	30.35378	531.2	4.2	108.9	444
_19AR106_Spot_ 140	123.7585	6360.412	2.20489	17.1144	2.196151	0.668544	3.024328	0.08592	0.997515	0.32983	531.3599	5.087829	519.827	12.30474	470.4464	63.21323	531.4	5.1	112.9	122
_19AR106_Spot_ 299	204.6742	17620.92	3.509448	17.06141	1.171002	0.684125	1.801859	0.086038	0.722713	0.401093	532.0622	3.690874	529.2647	7.432242	518.1545	36.22024	532.1	3.7	102.7	262
_19AR106_Spot_ 105	91.03003	7760.403	2.298124	17.31032	2.714512	0.662227	3.710918	0.0861	0.739011	0.199145	532.4314	3.776618	515.9752	15.01272	444.688	80.89337	532.4	3.8	119.7	90
_19AR106_Spot_ 61	192.6745	7131.288	4.329344	17.56176	1.283222	0.651677	2.121519	0.086106	0.776007	0.365779	532.4689	3.965951	509.5102	8.499519	408.7081	44.17698	532.5	4.0	130.3	53
_19AR106_Spot_ 146	109.6682	6738.759	1.700259	17.25127	2.054157	0.666747	2.974507	0.086139	0.710942	0.239012	532.6614	3.634681	518.7324	12.0825	458.8237	64.06821	532.7	3.6	116.1	128
_19AR106_Spot_ 533	330.917	20347.65	3.047574	17.30522	0.924426	0.675438	1.367476	0.086229	0.779231	0.569832	533.1991	3.987666	524.0131	5.597718	485.1223	24.83074	533.2	4.0	109.9	448
_19AR106_Spot_ 542	119.3287	7667.037	1.795346	16.92956	2.596069	0.677336	3.510891	0.086251	0.75343	0.214598	533.3243	3.856496	525.163	14.39662	490.7948	75.67184	533.3	3.9	108.7	457
_19AR106_Spot_ 519	120.0366	8363.174	3.459444	16.64521	1.873922	0.69057	2.625397	0.086308	0.989886	0.377042	533.6659	5.069934	533.1428	10.8897	531.8783	53.28517	533.7	5.1	100.3	435
_19AR106_Spot_ 177	205.4234	12709.75	2.700156	17.20518	1.179549	0.681066	2.17249	0.086365	0.753685	0.346922	534.0035	3.862517	527.4183	8.937236	499.9981	44.8807	534.0	3.9	106.8	153
_19AR106_Spot_ 260	108.9184	8620.891	2.032101	16.93854	1.486995	0.681188	1.810853	0.08646	0.609366	0.336508	534.5667	3.126065	527.4923	7.450265	497.9326	37.57054	534.6	3.1	107.4	226
_19AR106_Spot_ 548	150.0276	6792.503	2.953283	17.54027	1.512904	0.651962	4.048485	0.08651	0.785008	0.193902	534.8623	4.029247	509.6852	16.22488	399.2225	89.04675	534.9	4.0	134.0	463
_19AR106_Spot_ 233	314.7567	7194.946	1.604189	16.66313	0.792552	0.691794	2.626835	0.086636	0.837422	0.318795	535.6139	4.304066	533.8778	10.90709	527.4412	54.57587	535.6	4.3	101.5	203
_19AR106_Spot_ 261	139.6831	9603.129	2.429613	17.36726	2.278827	0.667647	2.74836	0.086687	0.608563	0.221428	535.9139	3.129491	519.2805	11.17284	447.7046	59.58376	535.9	3.1	119.7	227
_19AR106_Spot_ 215	307.2179	12276.17	1.758998	16.80575	0.949407	0.697057	1.664742	0.086724	0.687882	0.413207	536.1341	3.538776	537.0319	6.943136	541.7814	33.14157	536.1	3.5	99.0	186
_19AR106_Spot_ 133	167.3451	11322.71	2.689375	16.96211	1.243369	0.691857	1.700975	0.086791	0.763741	0.449002	536.529	3.931805	533.9156	7.062973	523.6988	33.32303	536.5	3.9	102.4	115
_19AR106_Spot_ 250	171.8773	14995.39	2.919564	17.16874	1.271835	0.683538	1.775464	0.086869	0.75037	0.422633	536.9929	3.866171	528.9105	7.319628	495.164	35.48667	537.0	3.9	108.4	218
_19AR106_Spot_ 599	95.07727	6873.558	3.449695	16.68387	2.809908	0.6926	3.273976	0.087171	0.579524	0.177009	538.7859	2.995471	534.3616	13.60378	516.4534	70.79517	538.8	3.0	104.3	504
_19AR106_Spot_ 218	85.08348	5606.06	1.8692	17.32825	2.786026	0.664978	4.58491	0.087506	0.763619	0.166551	540.7688	3.960956	517.6544	18.59551	417.8407	101.0255	540.8	4.0	129.4	188
_19AR106_Spot_ 137	160.1553	8091.475	1.764709	16.94437	1.834688	0.693222	2.368298	0.087555	1.109714	0.46857	541.0614	5.759164	534.7344	9.845513	508.7886	45.98685	541.1	5.8	106.3	119
_19AR106_Spot_ 340	119.2284	11099.17	1.907276	17.06702	2.805794	0.690805	3.081607	0.087556	0.786387	0.255187	541.064	4.081191	533.284	12.78474	501.1174	65.6355	541.1	4.1	108.0	290
_19AR106_Spot_ 41	107.3978	4885.851	2.105411	17.29278	2.097199	0.661395	3.287577	0.087583	0.703014	0.213839	541.2279	3.649558	515.4669	13.2898	403.7781	71.94823	541.2	3.6	134.0	36
_19AR106_Spot_ 448	381.1622	1650.582	11.6975	16.18239	0.910826	0.643232	13.64537	0.087912	1.000293	0.073306	543.1779	5.210758	504.305	54.28718	332.5878	309.8253	543.2	5.2	163.3	379
_19AR106_Spot_ 485	115.3431	7636.57	2.522613	17.63526	1.838125	0.662469	3.128504	0.088039	0.675819	0.21602	543.9274	3.525154	516.1231	12.65905	395.7919	68.51913	543.9	3.5	137.4	408
_19AR106_Spot_ 13	119.1971	6877.232	1.234459	16.99704	1.653572	0.686504	2.985399	0.08805	0.848426	0.284192	543.9966	4.426034	530.6978	12.33983	474.8897	63.33653	544.0	4.4	114.6	13
_19AR106_Spot_ 313	274.7376	18380.7	2.751086	17.11719	0.995307	0.701644	1.392124	0.088656	0.761106	0.546723	547.5863	3.995607	539.7725	5.828555	507.8798	25.63293	547.6	4.0	107.8	267
_19AR106_Spot_ 80	116.271	14865.46	3.776572	16.62041	1.852653	0.7227	2.238853	0.088842	0.73457	0.328101	548.6821	3.863697	552.2594	9.537104	568.0065	46.04156	548.7	3.9	96.6	68
_19AR106_Spot_ 326	172.2709	11071.39	4.231423	17.11267	1.221552	0.698438	2.464604	0.088855	0.7806	0.316724	548.759	4.106352	537.8578	10.2913	492.8409	51.55583	548.8	4.1	111.3	278
_19AR106_Spot_ 538	105.7897	7271.712	1.701078	16.88474	2.523239	0.703461	3.131475	0.08952	0.807771	0.257952	552.6978	4.278498	540.8563	13.1314	492.2294	66.73745	552.7	4.3	112.3	453
_19AR106_Spot_ 592	215.0621	10888.2	3.546893	17.24097	1.281219	0.698186	2.473757	0.089673	0.848697	0.34308	553.6022	4.502315	537.7066	10.32732	471.826	51.42404	553.6	4.5	117.3	498
_19AR106_Spot_ 517	180.9081	15292.29	192.0348	16.87669	1.872729	0.721948	2.202044	0.090216	0.860144	0.390612	556.8131	4.588381	551.8162	9.374632	532.2201	44.398	556.8	4.6	104.6	434
_19AR106_Spot_ 149	234.6258	12412.54	3.992799	17.20537	1.036728	0.713933	2.168559	0.090307	0.862307	0.397641	557.355	4.604207	547.0789	9.172262	505.464	43.79004	557.4	4.6	110.3	130
_19AR106_Spot_ 279	125.5888	9005.596	52.57637	17.02866	1.90531	0.711309	3.344394	0.090508	0.901693	0.269613	558.5435	4.824332	545.523	14.11578	492.4532	71.02825	558.5	4.8	113.4	245
_19AR106_Spot_ 316	430.723	3090.211	17.86963	16.13871	0.765071	0.714575	1.255749	0.090873	0.762448	0.607166	560.6964	4.094378	547.4592	5.314079	493.6932	21.9957	560.7	4.1	113.6	270
_19AR106_Spot_ 163	180.5145	10075.49	52.21397	16.67022	1.226407	0.739079	2.367958	0.091452	0.808525	0.341444	564.1207	4.367188	561.8679	10.21857	553.7184	48.5485	564.1	4.4	101.9	143
_19AR106_Spot_ 496	168.3787	9139.819	12.00529	16.74306	1.530712	0.736418	3.065386	0.092126	0.860844	0.280827	568.1008	4.681168	560.3133	13.20109	529.7835	64.46844	568.1	4.7	107.2	418
_19AR106_Spot_ 166	672.1512	34762.84	6.018103	16.44484	0.692036	0.769329	0.994344	0.092286	0.6886	0.692517	569.0451	3.750476	579.3778	4.390083	621.0205	15.49679	569.0	3.8	91.6	146
_19AR106_Spot_ 211	265.5612	15295.55	8.678609	16.18068	1.016403	0.775917	1.366657	0.092424	0.849919	0.621896	569.8599	4.635444	583.1518	6.062996	636.2144	23.05301	569.9	4.6	89.6	182
_19AR106_Spot_ 293	282.1781	23368.25	3.380538	17.26032	0.870627	0.73001	1.249929	0.092657	0.731596	0.58531	571.2293	3.999284	556.5593	5.355495	497.9355	22.30447	571.2	4.0	114.7	256
_19AR106_Spot_ 532	298.8378	19096.44	2.982832	16.70664	0.875398	0.756674	1.293379	0.093325	0.764138	0.590807	575.1694	4.204719	572.0895	5.656892	560.8207	22.72265	575.2	4.2	102.6	447
_19AR106_Spot_ 268	532.6569	41552	176.7466	16.76032	0.878538	0.760952	1.30106	0.093389	0.791959	0.608703	575.551	4.360572	574.5593	5.708759	571.6073	22.4574	575.6	4.4	100.7	234
_19AR106_Spot_ 510	79.9643	5662.15	3.33175	17.31976	2.228978	0.713845	3.192452	0.094233	1.105829	0.346389	580.5222	6.139019	547.0266	13.50244	410.7935	66.98797	580.5	6.1	141.3	429
_19AR106_Spot_ 406	280.5157	8804.683	1.004825	16.74931	0.840693	0.75406	3.010306	0.094433	0.609852	0.202588	581.7017	3.392171	570.5772	13.14093	527.4689	64.60349	581.7	3.4	110.3	346
_19AR106_Spot_ 469	212.1304	17157.12	4.146277	16.96047	1.128466	0.75741	1.454028	0.094813	0.7411	0.509687	583.9391	4.137355	572.515	6.363067	528.3866	27.41547	583.9	4.1	110.5	395
_19AR106_Spot_ 86	121.0736	4996.737	3.132625	16.41137	2.156989	0.758299	4.700546	0.094898	0.663023	0.141052	584.4414	3.704514	573.0283	20.58664	528.9859	102.0404	584.4	3.7	110.5	73
_19AR106_Spot_ 71	710.1448	18016.91	8.571017	16.60624	0.629353	0.782522	1.120949	0.095243	0.617266	0.550663	586.4729	3.460307	586.921	4.996675	589.6199	20.27281	586.5	3.5	99.5	61
_19AR106_Spot_ 110	146.3804	8406.096	7.128266	15.48711	1.394652	0.824132	1.713969	0.095355	0.742264	0.433067	587.1321	4.1655	610.3513	7.862891	698.4043	32.9112	587.1	4.2	84.1	94
_19AR106_Spot_ 241	188.2313	7416.069	2.386618	15.45872	1.010029	0.825577	2.286336	0.095656	0.763949	0.334137	588.9029	4.299537	611.155	10.49888	695.4413	45.9288	588.9	4.3	84.7	210
_19AR106_Spot_ 543	240.257	20535.35	3.363894	16.85725	1.31747	0.773788	1.55608	0.096108	0.765602	0.492007	591.558	4.327393	581.9339	6.892692	545.5178	29.61226	591.6	4.3	108.4	458
_19AR106_Spot_ 66	149.7426	8184.043	1.573109	16.52428	1.180575	0.78095	1.751292	0.096317	0.820763	0.468661	592.7905	4.648403	586.0254	7.797739	560.8703	33.71752	592.8	4.6	105.7	57
_19AR106_Spot_ 536	402.2397	17098.27	4.79308	16.46498	0.77402	0.804676	1.599116	0.097881	0.767531	0.479972	601.9792	4.411206	599.4628	7.240006	590.8905	30.40089	602.0	4.4	101.9	451
_19AR106_Spot_ 585	203.4751	12239.89	9.017183	15.93669	1.292261	0.828635	2.588005	0.09798	1.920636	0.74213	602.5615	11.04861	612.8547	11.90834	651.9897	37.25416	602.6	11.0	92.4	492
_19AR106_Spot_ 14	210.9153	10601.55	9.009076	16.31752	1.191852	0.81774	1.812982	0.099284	0.637782	0.351786	610.2111	3.713297	606.7871	8.281646	594.9679	36.78498	610.2	3.7	102.6	14
_19AR106_Spot_ 269	245.0236	21576.41	0.829052	16.50907	1.021118	0.822834	1.336276	0.100094	0.785673	0.587957	614.9595	4.608273	609.6285	6.124864	590.8319	23.41848	615.0	4.6	104.1	235
_19AR106_Spot_ 479	51.64035	5542.151	2.229852	15.89703	3.234421	0.832609	3.908417	0.100534	0.803318	0.205535	617.5351	4.730569	615.059	18.03217	606.8752	82.75922	617.5	4.7	101.8	402
_19AR106_Spot_ 429	234.1386	16747.14	4.234447	16.60411	0.994572	0.825115	1.61363	0.100965	0.758064	0.469788	620.0615	4.481481	610.898	7.407398	578.0117	30.95639	620.1	4.5	107.3	363
_19AR106_Spot_ 262	171.9409	6620.27	0.965759	16.12589	1.773354	0.831562	2.585764	0.101363	0.767905	0.296974	622.3944	4.555926	614.4786	11.92097	586.3801	53.59598	622.4	4.6	106.1	228
_19AR106_Spot_ 382	100.08	9274.759	0.997226	16.44533	1.88542	0.828188	2.379023	0.101597	0.692048	0.290896	623.7589	4.114447	612.606	10.94343	572.5387	49.51425	623.8	4.1	108.9	324
_19AR106_Spot_ 395	233.0309	18928.12	1.647687	16.5497	1.02211	0.834049	1.369398	0.101608	0.759616	0.554708	623.826	4.516619	615.8561	6.323329	587.5902	24.722	623.8	4.5	106.2	336
_19AR106_Spot_ 378	76.19184	5896.808	4.383054	16.20611	3.013355	0.828621	3.623936	0.101611	0.797314	0.220013	623.8453	4.740909	612.847	16.67561	573.378	76.90593	623.8	4.7	108.8	321
_19AR106_Spot_ 492	548.5339	13807.98	2.206798	16.32917	0.717123	0.840793	1.140623	0.101811	0.814029	0.71367	625.0116	4.848913	619.5829	5.290055	600.7223	17.29692	625.0	4.8	104.0	414
_19AR106_Spot_ 379	469.8744	55584.63	4.350282	16.54642	0.639816	0.84398	0.997375	0.101933	0.683045	0.684843	625.7279	4.073128	621.3395	4.63519	606.3101	15.70451	625.7	4.1	103.2	322
_19AR106_Spot_ 170	327.2622	16886.4	3.012812	16.59734	0.773766	0.838887	1.131418	0.102002	0.75761	0.669611	626.1285	4.520525	618.5312	5.240891	591.7718	18.19887	626.1	4.5	105.8	149
_19AR106_Spot_ 129	111.573	11472.01	1.494167	16.35056	2.305938	0.843104	3.133484	0.102017	1.699297	0.542303	626.216	10.14076	620.8572	14.55521	602.2929	56.9746	626.2	10.1	104.0	111
_19AR106_Spot_ 223	179.5957	12775.29	0.917724	16.60279	1.335059	0.831455	2.296613	0.102072	0.691482	0.301088	626.5411	4.128541	614.4192	10.58707	570.9351	47.65364	626.5	4.1	109.7	193
_19AR106_Spot_ 194	208.4232	20318.62	2.227054	16.54796	1.121951	0.844073	1.44541	0.102212	0.782524	0.541385	627.3611	4.677931	621.3909	6.717836	600.6298	26.29275	627.4	4.7	104.5	169
_19AR106_Spot_ 229	579.7211	50423.99	6.777554	16.30011	0.58325	0.859828	0.871026	0.102272	0.632072	0.725663	627.7106	3.780533	630.0291	4.08886	639.3154	12.89893	627.7	3.8	98.2	199
_19AR106_Spot_ 134	703.6981	40566.75	1.342143	16.51486	0.953697	0.851944	1.500316	0.102286	1.045613	0.696928	627.7919	6.254769	625.7157	7.008144	619.136	23.23418	627.8	6.3	101.4	116
_19AR106_Spot_ 494	230.6243	19165.55	1.92361	16.57303	1.318247	0.838991	1.901801	0.102613	0.809473	0.425635	629.7011	4.856216	618.5885	8.810155	579.0568	37.37582	629.7	4.9	108.7	416
_19AR106_Spot_ 19	32.63749	2903.175	7.552823	14.78162	5.512316	0.887149	8.345177	0.102796	0.968046	0.116001	630.7718	5.816936	644.8367	39.85464	695.3553	176.9239	630.8	5.8	90.7	17
_19AR106_Spot_ 414	267.9553	24418.32	4.06592	16.61317	0.849861	0.845359	1.205493	0.1032	0.621758	0.51577	633.1371	3.74944	622.0989	5.607375	583.0749	22.42042	633.1	3.7	108.6	353
_19AR106_Spot_ 159	173.2621	12962.38	5.171356	16.52698	1.189598	0.854359	2.184578	0.103754	0.748907	0.342815	636.3702	4.538138	627.0388	10.22019	594.4629	44.48595	636.4	4.5	107.0	139
_19AR106_Spot_ 64	264.8248	15469.46	0.957072	16.19362	1.00082	0.871752	1.434284	0.103852	0.664302	0.463159	636.9441	4.028914	636.5184	6.782912	635.9648	27.35823	636.9	4.0	100.2	55
_19AR106_Spot_ 225	240.7301	23623.39	1.391837	16.43424	0.727256	0.863214	0.975751	0.103893	0.61842	0.633789	637.1849	3.751996	631.8758	4.590158	613.8453	16.32349	637.2	3.8	103.8	195
_19AR106_Spot_ 220	338.905	3260.657	6.829585	15.04803	0.87893	0.89145	1.529507	0.104196	0.953742	0.623562	638.9539	5.801698	647.1482	7.319662	676.7916	25.58199	639.0	5.8	94.4	190
_19AR106_Spot_ 160	202.7409	12280.35	2.464805	16.33932	1.27114	0.867193	1.671257	0.1042	0.748191	0.447681	638.9774	4.551469	634.0417	7.881492	617.404	32.24222	639.0	4.6	103.5	140
_19AR106_Spot_ 388	165.4029	18114.33	4.734881	15.85299	1.294356	0.893037	2.129155	0.104292	1.531214	0.719165	639.5151	9.3223	647.9995	10.19911	678.582	31.61729	639.5	9.3	94.2	329
_19AR106_Spot_ 301	154.9581	10473.79	4.930591	16.3323	1.744279	0.862406	2.164949	0.104813	0.670813	0.309852	642.5559	4.102491	631.4353	10.17957	592.7385	44.62413	642.6	4.1	108.4	264
_19AR106_Spot_ 464	162.6201	12255.56	5.732491	16.42033	1.016195	0.864472	1.426148	0.105124	0.65495	0.459244	644.3654	4.016206	632.5613	6.714226	591.5444	27.46911	644.4	4.0	108.9	391
_19AR106_Spot_ 26	71.07733	6704.662	6.160703	15.83382	3.086543	0.883097	4.22804	0.105148	1.108261	0.262122	644.5074	6.797368	642.654	20.13546	637.1031	87.80733	644.5	6.8	101.2	23
_19AR106_Spot_ 590	247.8042	13202.58	1.518704	16.28013	1.228367	0.872609	1.957784	0.105177	0.834196	0.426092	644.6749	5.117694	636.983	9.263592	610.708	38.28007	644.7	5.1	105.6	496
_19AR106_Spot_ 394	360.5779	25551.42	11.24243	15.76994	0.927475	0.915073	1.624086	0.105672	1.066969	0.656966	647.5619	6.573589	659.7509	7.879852	702.5883	26.08534	647.6	6.6	92.2	335
_19AR106_Spot_ 226	238.1292	15425.39	2.155195	16.31425	1.141494	0.880491	1.745875	0.105758	0.628397	0.359932	648.0666	3.874418	641.2479	8.30054	618.2186	35.16656	648.1	3.9	104.8	196
_19AR106_Spot_ 287	237.4033	28001.31	4.618971	16.4977	0.814993	0.875767	1.290122	0.105957	0.676848	0.524639	649.2238	4.180229	638.6938	6.116114	602.5383	23.75057	649.2	4.2	107.7	251
_19AR106_Spot_ 267	104.6993	9394.06	1.154441	16.10577	2.001594	0.8848	2.441691	0.106365	0.786557	0.322136	651.6046	4.874722	643.5718	11.6391	616.4009	49.89489	651.6	4.9	105.7	233
_19AR106_Spot_ 188	383.1843	24905.58	1.556778	16.10509	0.926448	0.906792	1.319444	0.106538	0.861128	0.652644	652.6108	5.344712	655.3506	6.371343	665.729	21.38871	652.6	5.3	98.0	164
_19AR106_Spot_ 320	250.8049	19275.04	3.022936	16.42432	1.024735	0.881681	1.400396	0.106793	0.758285	0.541479	654.0951	4.716572	641.8904	6.662734	600.0946	25.46986	654.1	4.7	109.0	273
_19AR106_Spot_ 412	177.7171	14161.37	3.986883	16.33296	0.873135	0.885645	1.213354	0.107079	0.71266	0.587347	655.7594	4.443495	644.0269	5.786579	604.0192	21.23741	655.8	4.4	108.6	352
_19AR106_Spot_ 43	439.2907	35482.96	5.767897	16.27138	0.735299	0.916995	1.079529	0.109088	0.670126	0.620758	667.4476	4.248979	660.7693	5.243409	639.0009	18.2059	667.4	4.2	104.5	38
_19AR106_Spot_ 158	334.6961	19865.38	1.853668	16.2879	0.804417	0.920712	1.303649	0.109457	0.709454	0.544207	669.5927	4.512064	662.7363	6.34538	640.4374	23.54141	669.6	4.5	104.6	138
_19AR106_Spot_ 249	202.1383	16398.53	12.01639	16.00428	1.055489	0.930731	1.801767	0.109779	0.64705	0.35912	671.4618	4.126077	668.0194	8.819458	657.3776	36.08437	671.5	4.1	102.1	217
_19AR106_Spot_ 455	535.822	39217	13.40311	16.18642	0.725898	0.930267	1.174928	0.109835	0.782295	0.665824	671.7877	4.990801	667.775	5.74958	655.1695	18.79419	671.8	5.0	102.5	385
_19AR106_Spot_ 407	162.0163	9300.394	1.221339	16.12596	1.23767	0.918915	2.415249	0.110485	0.639862	0.264926	675.5643	4.103887	661.7859	11.7444	616.0369	50.2736	675.6	4.1	109.7	347
_19AR106_Spot_ 9	988.0209	72124.63	2.614766	16.06165	0.629285	0.944344	0.941562	0.110821	0.668461	0.709949	677.5155	4.29906	675.1532	4.643433	668.1922	14.17954	677.5	4.3	101.4	9
_19AR106_Spot_ 466	207.9649	18024.13	15.87138	15.44373	1.006009	0.986969	1.336066	0.112075	0.632285	0.473244	684.7858	4.107758	697.1726	6.738711	738.2188	24.88888	684.8	4.1	92.8	393
_19AR106_Spot_ 5	241.8333	21605.21	1.216719	15.86555	1.04187	0.964346	1.332503	0.112714	0.680194	0.510464	688.4887	4.441652	685.5452	6.642309	676.8272	24.50968	688.5	4.4	101.7	5
_19AR106_Spot_ 516	471.2346	28507.11	2.852393	15.89204	0.913158	0.966672	1.271748	0.112835	0.82968	0.652394	689.193	5.423042	686.747	6.347222	679.6516	20.57518	689.2	5.4	101.4	433
_19AR106_Spot_ 104	394.0771	33601	1.598964	15.87353	0.794732	0.973747	1.096658	0.113096	0.713539	0.650648	690.7041	4.673596	690.3932	5.493638	690.2882	17.75924	690.7	4.7	100.1	89
_19AR106_Spot_ 256	120.4421	12376.04	3.027596	14.8046	1.269341	1.036019	2.066771	0.113529	0.674456	0.326333	693.211	4.432792	721.9339	10.67884	813.1608	40.87026	693.2	4.4	85.2	224
_19AR106_Spot_ 271	277.6421	13429.29	2.181636	15.58447	0.738155	0.986461	1.361237	0.113929	0.887183	0.651747	695.5262	5.849363	696.9128	6.863887	702.3338	21.97721	695.5	5.8	99.0	237
_19AR106_Spot_ 143	414.0407	6381.534	4.262568	15.57299	0.664603	0.978759	1.094022	0.113994	0.781469	0.714308	695.9007	5.154996	692.9685	5.49469	684.4144	16.346	695.9	5.2	101.7	125
_19AR106_Spot_ 472	502.0495	6038.543	6.637327	14.65123	0.655785	1.032418	1.737371	0.114016	0.737515	0.424501	696.0271	4.865893	720.1362	8.96141	796.8766	32.98074	696.0	4.9	87.3	397
_19AR106_Spot_ 410	190.9377	15729.8	3.396798	15.44613	1.044436	1.010213	1.385527	0.115156	0.754493	0.544553	702.6219	5.02255	708.9819	7.070056	730.1202	24.62581	702.6	5.0	96.2	350
_19AR106_Spot_ 91	153.4125	17033	3.929999	14.9951	1.117356	1.064455	1.570281	0.117585	0.685861	0.436776	716.6462	4.651839	736.0171	8.221266	796.327	29.63093	716.6	4.7	90.0	78
_19AR106_Spot_ 65	158.0412	11394.93	2.404793	15.88345	1.162592	1.005972	1.602257	0.118145	0.762384	0.475819	719.8788	5.192896	706.8373	8.158916	666.5548	30.1773	719.9	5.2	108.0	56
_19AR106_Spot_ 350	122.2338	7577.31	1.607029	15.46364	1.538147	1.03038	3.159817	0.119045	1.008137	0.319049	725.0662	6.913575	719.1176	16.28355	701.551	63.74868	725.1	6.9	103.4	295
_19AR106_Spot_ 164	139.4803	9917.433	3.356509	14.96248	1.348918	1.074847	1.647668	0.119093	0.715914	0.434501	725.3415	4.911332	741.1155	8.667043	789.9678	31.16664	725.3	4.9	91.8	144
_19AR106_Spot_ 416	196.9638	26992.54	4.993183	14.81108	1.150621	1.101524	1.443073	0.119647	0.793067	0.549568	728.5293	5.463204	754.087	7.680437	831.5473	25.13497	728.5	5.5	87.6	355
_19AR106_Spot_ 281	225.0605	17100.98	9.307858	14.32638	1.131061	1.140968	1.562773	0.120226	1.024982	0.655874	731.8665	7.091344	772.9687	8.456664	894.543	24.35081	731.9	7.1	81.8	247
_19AR106_Spot_ 497	291.0068	35743.21	2.414506	15.12736	0.731872	1.089235	1.392298	0.120575	1.175596	0.844356	733.8716	8.154409	748.1322	7.370622	791.9219	15.65072	733.9	8.2	92.7	419
_19AR106_Spot_ 228	182.0785	17040.16	9.030956	15.47832	1.052595	1.060383	1.560627	0.120694	0.733844	0.470224	734.5591	5.094739	734.0123	8.155547	733.2848	29.16501	734.6	5.1	100.2	198

_19AR106_Spot_ 523	149.1842	19609.99	6.398071	14.66164	1.116106	1.125402	1.527841	0.12156	0.83284	0.545109	739.5383	5.819013	765.559	8.214556	843.163	26.6352	739.5	5.8	87.7	438
_19AR106_Spot_ 210	838.2833	59967.99	4.262289	15.40288	0.657865	1.12898	0.985903	0.126524	0.714697	0.724916	768.0078	5.174566	767.2672	5.308635	766.0086	14.30817	768.0	5.2	100.3	181
_19AR106_Spot_ 593	281.3846	9534.518	3.600558	14.22615	0.924591	1.21903	2.877608	0.128971	1.954505	0.679212	781.9922	14.39345	809.3316	16.05273	886.1808	43.64781	782.0	14.4	88.2	499
_19AR106_Spot_ 489	153.4976	17772.48	3.131874	15.29359	1.211626	1.147095	1.662899	0.129468	0.779788	0.468933	784.83	5.762139	775.8701	9.021013	751.0735	31.01941	784.8	5.8	104.5	411
_19AR106_Spot_ 127	128.2468	10867.98	2.856447	15.17801	1.12946	1.165686	1.660329	0.130666	0.761544	0.458671	791.6654	5.673391	784.6243	9.074479	765.5539	31.06626	791.7	5.7	103.4	109
_19AR106_Spot_ 216	256.8267	15947.15	4.062201	15.17181	0.844652	1.180531	1.572422	0.131764	0.659541	0.419443	797.9209	4.949954	791.5605	8.644198	774.582	30.0323	797.9	4.9	103.0	187
_19AR106_Spot_ 426	180.1741	26657.31	5.044849	14.4042	1.058663	1.254579	1.76342	0.132403	1.245584	0.706346	801.5613	9.388347	825.4692	9.963963	891.3357	25.78002	801.6	9.4	89.9	361
_19AR106_Spot_ 436	247.8485	36329.16	12.65821	15.178	0.731592	1.200969	2.322031	0.132854	2.140538	0.921839	804.1268	16.1824	801.0332	12.86587	793.3324	18.89696	804.1	16.2	101.4	368
_19AR106_Spot_ 376	80.28827	12420.38	4.037669	14.46642	1.840904	1.248244	2.465192	0.133676	0.90186	0.365838	808.7999	6.855209	822.6119	13.89837	861.0193	47.59466	808.8	6.9	93.9	319
_19AR106_Spot_ 547	122.73	7531.801	5.004486	14.41366	1.368933	1.263123	3.144521	0.136286	0.927879	0.295078	823.6241	7.174179	829.3096	17.82241	845.4625	62.51162	823.6	7.2	97.4	462
_19AR106_Spot_ 141	78.21062	4651.094	6.945199	14.28349	1.526053	1.266425	2.366757	0.136533	1.621863	0.685268	825.0266	12.55996	830.79	13.4291	847.1566	35.84879	825.0	12.6	97.4	123
_19AR106_Spot_ 367	154.5883	18550.26	3.307885	13.87207	1.044642	1.349272	1.737344	0.137019	1.279576	0.736512	827.7848	9.94027	867.2442	10.13203	970.3127	23.98351	827.8	9.9	85.3	311
_19AR106_Spot_ 338	212.3856	17788.38	3.622668	14.31695	0.855882	1.306769	1.448839	0.137496	0.893457	0.616671	830.4847	6.961952	848.7057	8.334022	897.5275	23.53181	830.5	7.0	92.5	288
_19AR106_Spot_ 383	561.2255	24443.07	27.58773	14.25708	0.567638	1.375214	0.984857	0.143724	0.721698	0.732795	865.6839	5.846298	878.3954	5.78996	911.4426	13.80678	865.7	5.8	95.0	325
_19AR106_Spot_ 32	51.11397	4981.541	1.605254	14.39362	2.028752	1.341653	2.849112	0.146206	0.889436	0.31218	879.6595	7.313665	863.9458	16.57661	824.7576	56.49983	879.7	7.3	106.7	27
_19AR106_Spot_ 96	103.6594	14103.07	21.70709	14.28258	1.221223	1.385154	1.644424	0.146369	0.678226	0.41244	880.5793	5.582365	882.6356	9.697026	888.7015	30.97228	880.6	5.6	99.1	83
_19AR106_Spot_ 252	364.0728	3298.899	2.529543	13.61483	0.57829	1.480727	1.28591	0.15567	0.72805	0.566175	932.6688	6.321945	922.5279	7.793721	899.2381	21.8635	899.2	21.9	103.7	220
_19AR106_Spot_ 45	69.45421	8919.875	2.508389	14.12615	1.648464	1.563316	1.95361	0.164054	0.81044	0.414842	979.2684	7.362979	955.7819	12.09854	902.9846	36.62732	903.0	36.6	108.4	40
_19AR106_Spot_ 187	41.82196	3272.189	1.91486	14.00574	2.472522	1.396784	2.850334	0.150966	0.945297	0.331644	906.3738	7.992862	887.5744	16.86809	841.9348	55.97736	906.4	8.0	107.7	163
_19AR106_Spot_ 442	81.24601	12214.57	1.700022	14.13179	1.434226	1.594386	1.959864	0.16599	0.77105	0.39342	989.9818	7.076019	968.0153	12.23026	919.3832	37.03211	919.4	37.0	107.7	374
_19AR106_Spot_ 535	63.69458	7252.198	2.280356	13.88822	1.952045	1.62192	3.015219	0.16871	0.816757	0.270878	1005.001	7.600559	978.7345	18.94127	921.1704	59.69639	921.2	59.7	109.1	450
_19AR106_Spot_ 235	157.6852	5205.657	2.26242	13.64289	0.759967	1.543153	3.627996	0.158965	1.194506	0.329247	951.0203	10.56178	947.7632	22.35648	941.1171	70.2059	941.1	70.2	101.1	204
_19AR106_Spot_ 561	113.0432	11166.43	1.337906	13.96151	0.853614	1.549625	1.614062	0.159547	0.983143	0.609111	954.2565	8.720361	950.3441	9.961262	942.2046	26.22159	942.2	26.2	101.3	474
_19AR106_Spot_ 576	374.8567	5863.718	4.528469	13.67718	0.774826	1.457718	1.508322	0.149936	0.723913	0.479946	900.6065	6.084685	913.0662	9.083998	944.204	27.10552	944.2	27.1	95.4	484
_19AR106_Spot_ 418	154.9184	7410.383	2.585316	13.75139	0.704225	1.504702	1.39546	0.154626	0.657918	0.47147	926.8401	5.679764	932.294	8.512399	946.0808	25.20065	946.1	25.2	98.0	356
_19AR106_Spot_ 49	84.45068	11634.33	3.996444	13.90369	1.042126	1.644907	1.708049	0.168703	0.722595	0.423053	1004.959	6.72405	987.598	10.78642	950.0892	31.67361	950.1	31.7	105.8	44
_19AR106_Spot_ 408	386.8057	28109.45	3.466569	13.97234	0.606591	1.580242	0.889343	0.161789	0.632524	0.711226	966.7139	5.678296	962.4644	5.530527	953.6636	12.78092	953.7	12.8	101.4	348
_19AR106_Spot_ 351	99.62143	11626.4	3.479953	13.84691	0.847332	1.61252	1.672567	0.164714	0.743841	0.44473	982.9212	6.781254	975.0878	10.48273	958.3537	30.59965	958.4	30.6	102.6	296
_19AR106_Spot_ 399	526.9919	6824.817	6.793055	13.66601	0.667401	1.540476	1.86241	0.157281	0.774727	0.415981	941.6498	6.78742	946.694	11.46736	959.312	34.61167	959.3	34.6	98.2	340
_19AR106_Spot_ 370	376.1002	44638.39	6.516928	13.9663	0.652112	1.436134	0.98276	0.146408	0.712378	0.724875	880.7988	5.864834	904.1094	5.882691	962.4113	13.82872	962.4	13.8	91.5	314
_19AR106_Spot_ 397	147.9906	21613.42	3.878635	13.89117	1.150611	1.490366	1.522582	0.15186	0.794036	0.521506	911.3841	6.748441	926.4657	9.252355	963.4436	26.53607	963.4	26.5	94.6	338
_19AR106_Spot_ 583	18.17707	4376.784	2.480713	13.38589	4.524315	1.657748	5.332924	0.168895	1.260148	0.236296	1006.019	11.73766	992.5156	33.78782	963.6967	105.9095	963.7	105.9	104.4	490
_19AR106_Spot_ 434	251.8313	19937.47	9.814272	13.91515	0.776158	1.507653	1.403463	0.153515	0.772817	0.55065	920.6344	6.63012	933.4895	8.567914	964.8696	23.90935	964.9	23.9	95.4	367
_19AR106_Spot_ 212	213.5802	19578.94	1.780558	13.87556	0.824711	1.613558	1.390138	0.164054	0.735789	0.529292	979.2652	6.68474	975.4913	8.71467	967.8882	24.07705	967.9	24.1	101.2	183
_19AR106_Spot_ 122	120.5428	12630.71	2.430335	13.79952	1.055038	1.553276	1.613165	0.157883	0.829237	0.514044	945.0009	7.288998	951.7968	9.964912	968.4198	28.22795	968.4	28.2	97.6	104
_19AR106_Spot_ 396	82.0929	8475.635	2.318216	13.6574	1.338815	1.573844	1.975754	0.159897	0.747532	0.378353	956.2025	6.64306	959.9437	12.26771	969.4125	37.29963	969.4	37.3	98.6	337
_19AR106_Spot_ 323	87.9416	9219.997	2.598273	13.67376	1.390524	1.77027	1.708153	0.179832	0.733274	0.429279	1066.059	7.204977	1034.619	11.08387	969.6405	31.4794	969.6	31.5	109.9	276
_19AR106_Spot_ 169	333.8137	21441.99	14.33423	13.93612	0.632092	1.391541	1.01709	0.141359	0.746136	0.733599	852.3406	5.957124	885.3511	6.009145	969.649	14.11155	969.6	14.1	87.9	148
_19AR106_Spot_ 88	79.97574	8172.251	1.87967	13.62917	1.806949	1.430192	2.942	0.14516	0.692219	0.235288	873.7729	5.656415	901.6301	17.58206	971.4352	58.33583	971.4	58.3	89.9	75
_19AR106_Spot_ 181	202.323	16610.25	4.962046	13.81536	0.97496	1.604806	1.477512	0.162776	0.972053	0.657899	972.1873	8.772101	972.0853	9.243153	972.7625	22.69871	972.8	22.7	99.9	157
_19AR106_Spot_ 280	146.3229	19993.11	2.261977	13.81815	0.952257	1.684549	1.494557	0.170838	0.847204	0.566859	1016.728	7.968826	1002.704	9.522862	973.0796	25.10907	973.1	25.1	104.5	246
_19AR106_Spot_ 58	86.22494	8112.668	2.372161	13.63057	0.964597	1.661486	1.331113	0.168308	0.667858	0.501729	1002.782	6.202256	993.9427	8.437764	975.3746	23.45328	975.4	23.5	102.8	50
_19AR106_Spot_ 73	68.21776	8623.336	1.37731	13.66201	1.135423	1.743818	1.494551	0.176591	0.66231	0.44315	1048.322	6.407988	1024.877	9.64494	976.058	27.29104	976.1	27.3	107.4	63
_19AR106_Spot_ 167	380.359	31347.12	1.789795	13.87095	0.662422	1.659811	0.987045	0.167852	0.682184	0.691137	1000.265	6.3206	993.3038	6.254325	978.8305	14.51905	978.8	14.5	102.2	147
_19AR106_Spot_ 344	142.815	11315.8	3.145806	13.70433	1.079116	1.609449	1.495847	0.162749	0.850605	0.568645	972.0344	7.675	973.8935	9.368237	978.957	25.06178	979.0	25.1	99.3	292
_19AR106_Spot_ 393	63.89241	8235.388	2.373328	13.58176	1.205442	1.70348	2.16492	0.172201	0.747027	0.34506	1024.229	7.074392	1009.839	13.852	979.6186	41.3984	979.6	41.4	104.6	334
_19AR106_Spot_ 499	84.00155	4219.028	1.85847	13.27618	1.001474	1.76814	1.557905	0.178548	0.757122	0.485987	1059.039	7.39422	1033.838	10.10448	981.8155	27.71034	981.8	27.7	107.9	421
_19AR106_Spot_ 346	423.8733	48285.3	2.712086	13.87697	0.807275	1.679431	1.353713	0.16958	1.058921	0.782235	1009.798	9.897541	1000.766	8.615623	981.9402	17.15564	981.9	17.2	102.8	293
_19AR106_Spot_ 347	111.029	15683.39	1.351534	13.74081	1.197096	1.774153	1.669472	0.178894	0.891961	0.534277	1060.929	8.7254	1036.041	10.84142	984.7579	28.72791	984.8	28.7	107.7	294
_19AR106_Spot_ 437	58.35093	8606.07	3.585705	13.59514	1.502712	1.797519	2.627888	0.181117	0.940873	0.358034	1073.073	9.300698	1044.558	17.14662	986.2477	49.93107	986.2	49.9	108.8	369
_19AR106_Spot_ 139	280.3406	34511.03	3.217638	13.87675	0.997258	1.517027	1.425715	0.152769	1.000993	0.702099	916.464	8.55148	937.2783	8.725266	987.4091	20.65708	987.4	20.7	92.8	121
_19AR106_Spot_ 527	136.1724	19154.1	2.559179	13.66298	0.786868	1.68875	1.31909	0.16997	0.825608	0.625892	1011.948	7.73198	1004.292	8.412582	988.4971	20.91871	988.5	20.9	102.4	442
_19AR106_Spot_ 42	189.5027	24908.86	3.220153	13.74248	0.846159	1.712488	1.157323	0.172273	0.683487	0.590576	1024.621	6.474949	1013.217	7.419111	989.5122	18.98527	989.5	19.0	103.5	37
_19AR106_Spot_ 284	225.8704	33113.75	2.065547	13.7476	0.807356	1.733853	1.24439	0.174346	0.815716	0.655515	1036.014	7.806822	1021.183	8.013692	990.4307	19.10823	990.4	19.1	104.6	249
_19AR106_Spot_ 335	73.1485	8823.422	2.882061	13.51759	1.410587	1.778315	2.391281	0.178747	0.859188	0.3593	1060.126	8.398951	1037.564	15.54253	991.23	45.36672	991.2	45.4	107.0	285
_19AR106_Spot_ 230	178.2132	15928.62	3.850828	13.66557	0.852464	1.571697	1.147105	0.157928	0.703237	0.613054	945.2527	6.18298	959.0964	7.118511	991.8599	18.40878	991.9	18.4	95.3	200
_19AR106_Spot_ 289	220.4615	33509.72	3.203736	13.72874	0.743671	1.678566	1.089357	0.168626	0.765538	0.702743	1004.535	7.120882	1000.438	6.931752	992.3362	15.74683	992.3	15.7	101.2	253
_19AR106_Spot_ 468	506.9501	9073.456	2.017693	13.51797	0.624422	1.662565	0.965131	0.166873	0.627016	0.64967	994.857	5.780413	994.3546	6.119272	994.1506	14.91463	994.2	14.9	100.1	394
_19AR106_Spot_ 8	548.9606	46493.63	3.884618	13.72155	0.632968	1.574754	1.018335	0.158034	0.774172	0.760233	945.8403	6.810582	960.3027	6.324167	994.476	13.42717	994.5	13.4	95.1	8
_19AR106_Spot_ 224	421.8223	52284.91	2.748865	13.76799	0.583403	1.695589	0.930049	0.169882	0.679331	0.730425	1011.463	6.359255	1006.871	5.940289	997.7558	12.90533	997.8	12.9	101.4	194
_19AR106_Spot_ 586	356.9529	40979.71	14.40926	13.64504	0.832974	1.741463	1.282156	0.173812	0.866574	0.675872	1033.083	8.271919	1024.006	8.270129	1005.521	19.17525	1005.5	19.2	102.7	493
_19AR106_Spot_ 135	134.065	13082.32	5.783062	13.60492	1.199895	1.705609	1.733079	0.170224	1.014692	0.585485	1013.345	9.514907	1010.638	11.09379	1005.639	28.49996	1005.6	28.5	100.8	117
_19AR106_Spot_ 40	326.5396	31363.45	5.13873	13.656	0.634699	1.727265	0.984371	0.172377	0.653727	0.664107	1025.195	6.196223	1018.733	6.330324	1005.736	14.91454	1005.7	14.9	101.9	35
_19AR106_Spot_ 108	262.1213	23544.26	17.6097	13.60692	0.703912	1.730969	1.190491	0.172709	0.674248	0.566361	1027.02	6.40122	1020.111	7.661903	1006.18	19.90657	1006.2	19.9	102.1	92
_19AR106_Spot_ 526	264.2322	28488.56	10.47474	13.58759	0.741007	1.739395	1.085331	0.173511	0.744006	0.68551	1031.425	7.091424	1023.239	6.997493	1006.637	16.01206	1006.6	16.0	102.5	441
_19AR106_Spot_ 288	145.8736	22513.02	0.788705	13.59406	0.970819	1.769381	1.355561	0.176386	0.922473	0.68051	1047.203	8.916351	1034.293	8.794246	1007.963	20.14754	1008.0	20.1	103.9	252
_19AR106_Spot_ 97	100.0461	12379.86	4.037313	13.4451	1.066927	1.751064	1.538551	0.174356	0.613841	0.398973	1036.067	5.875041	1027.555	9.943908	1010.342	28.58805	1010.3	28.6	102.5	84
_19AR106_Spot_ 509	200.9316	30576.09	3.732831	13.58484	0.81481	1.614474	1.204908	0.160733	0.769647	0.63876	960.8492	6.870411	975.8469	7.555072	1010.624	18.79486	1010.6	18.8	95.1	428
_19AR106_Spot_ 263	150.6934	22191.14	2.274382	13.55216	1.118067	1.646014	1.455501	0.163795	0.887806	0.609966	977.8329	8.054913	988.023	9.193816	1011.588	23.37265	1011.6	23.4	96.7	229
_19AR106_Spot_ 512	593.0755	8061.124	4.318426	13.34471	0.776594	1.706638	1.311942	0.169815	1.025729	0.78184	1011.093	9.598655	1011.024	8.399739	1011.74	16.56931	1011.7	16.6	99.9	431
_19AR106_Spot_ 386	416.5942	18581.23	9.634227	13.51708	0.55437	1.754792	0.836358	0.174589	0.604086	0.722281	1037.346	5.788254	1028.93	5.409572	1011.944	11.72642	1011.9	11.7	102.5	327
_19AR106_Spot_ 63	144.2013	20090.67	0.64561	13.59906	0.885964	1.775	1.24633	0.17659	0.73404	0.588961	1048.318	7.10197	1036.351	8.094826	1012.053	20.41476	1012.1	20.4	103.6	54
_19AR106_Spot_ 95	74.62861	11091.75	4.932576	13.40675	1.298328	1.788667	1.856666	0.177907	0.694509	0.374063	1055.53	6.762051	1041.34	12.09253	1012.541	34.91324	1012.5	34.9	104.2	82
_19AR106_Spot_ 342	150.1949	31875.53	4.997549	13.6129	0.861772	1.769085	1.171959	0.175916	0.727007	0.620335	1044.624	7.011095	1034.185	7.602612	1013.04	18.61522	1013.0	18.6	103.1	291
_19AR106_Spot_ 545	113.1932	2059.435	2.097869	12.47129	0.885798	1.697128	2.393805	0.168621	0.870917	0.363821	1004.507	8.100887	1007.45	15.29553	1014.721	45.19446	1014.7	45.2	99.0	460
_19AR106_Spot_ 400	267.4334	36927.37	2.951299	13.60394	0.749301	1.757269	1.061123	0.174504	0.72617	0.684341	1036.88	6.955175	1029.843	6.866909	1015.787	15.67537	1015.8	15.7	102.1	341
_19AR106_Spot_ 278	215.0859	8654.391	2.057972	13.35508	0.642354	1.815921	1.09619	0.18031	0.601805	0.548996	1068.667	5.926483	1051.215	7.177936	1015.999	18.56241	1016.0	18.6	105.2	244
_19AR106_Spot_ 331	212.2073	29414.01	4.778582	13.57519	0.764071	1.78442	1.297906	0.177024	0.818557	0.630675	1050.698	7.936238	1039.793	8.445892	1017.802	20.40081	1017.8	20.4	103.2	281
_19AR106_Spot_ 25	120.8491	5848.419	2.144407	13.21687	0.963922	1.81936	1.902057	0.180452	0.773954	0.406904	1069.442	7.626872	1052.455	12.4636	1018.238	35.18919	1018.2	35.2	105.0	22
_19AR106_Spot_ 253	139.7499	24060.33	4.015383	13.52398	0.772424	1.779857	1.254454	0.17652	0.683076	0.54452	1047.936	6.606662	1038.127	8.155615	1018.392	21.30953	1018.4	21.3	102.9	221
_19AR106_Spot_ 90	115.0011	12557.91	2.410554	13.42694	0.920665	1.715972	1.352355	0.170173	0.61581	0.455361	1013.063	5.773048	1014.52	8.675932	1018.527	24.36456	1018.5	24.4	99.5	77
_19AR106_Spot_ 44	500.7907	22389.66	3.190995	13.54379	0.634869	1.580645	1.181015	0.156715	0.963258	0.815618	938.4963	8.412899	962.6232	7.345124	1019.009	13.83739	1019.0	13.8	92.1	39
_19AR106_Spot_ 89	326.2147	11113.88	2.584235	13.39556	0.546371	1.730435	1.204302	0.171543	0.675698	0.56107	1020.608	6.378027	1019.913	7.749918	1019.281	20.18664	1019.3	20.2	100.1	76
_19AR106_Spot_ 483	152.5269	19176.79	5.544524	13.47532	0.912992	1.764464	1.244089	0.174901	0.635529	0.510839	1039.059	6.09881	1032.489	8.062921	1019.46	21.65634	1019.5	21.7	101.9	406
_19AR106_Spot_ 68	135.1245	17224.9	6.183829	13.53598	0.918442	1.857713	1.490626	0.184124	0.773644	0.519006	1089.464	7.754829	1066.174	9.839482	1019.688	25.79966	1019.7	25.8	106.8	59
_19AR106_Spot_ 411	336.9131	34285.97	14.21373	13.5415	0.643565	1.764	0.955766	0.174775	0.689848	0.721775	1038.365	6.616008	1032.319	6.193663	1020.393	13.39338	1020.4	13.4	101.8	351
_19AR106_Spot_ 495	138.4092	17281.7	3.292693	13.4631	0.845456	1.750622	1.3653	0.173341	1.001375	0.733447	1030.49	9.536553	1027.392	8.823285	1021.662	18.78611	1021.7	18.8	100.9	417
_19AR106_Spot_ 37	107.5808	17732.49	0.873008	13.50696	1.268009	1.772617	1.671241	0.175511	0.746101	0.446436	1042.402	7.181135	1035.479	10.84952	1021.752	30.27756	1021.8	30.3	102.0	32
_19AR106_Spot_ 449	239.7041	25362.41	5.395654	13.58555	0.702855	1.749658	1.173984	0.17317	0.842601	0.717728	1029.556	8.017754	1027.036	7.585334	1022.534	16.54485	1022.5	16.5	100.7	380
_19AR106_Spot_ 75	240.3719	21234.32	1.96459	13.49306	0.669604	1.761605	1.215604	0.174055	0.653237	0.537376	1034.413	6.242897	1031.438	7.873682	1025.998	20.75691	1026.0	20.8	100.8	65
_19AR106_Spot_ 441	72.07859	14367.09	3.202618	13.45431	1.271482	1.754324	2.001453	0.173319	0.780038	0.389736	1030.375	7.427887	1028.758	12.94475	1026.182	37.30541	1026.2	37.3	100.4	373
_19AR106_Spot_ 582	107.8198	10693.64	3.529419	13.32877	1.066779	1.80168	1.438085	0.177934	0.836695	0.581812	1055.68	8.147498	1046.067	9.390442	1026.904	23.6756	1026.9	23.7	102.8	489
_19AR106_Spot_ 136	116.3895	9903.838	1.058799	13.39928	1.180344	1.742946	1.632191	0.172097	0.978538	0.599524	1023.653	9.262015	1024.555	10.53133	1027.343	26.4394	1027.3	26.4	99.6	118
_19AR106_Spot_ 389	270.7786	24510.39	5.564047	13.47916	0.573668	1.760835	0.872177	0.173862	0.60858	0.69777	1033.355	5.810632	1031.155	5.648295	1027.354	12.6352	1027.4	12.6	100.6	330
_19AR106_Spot_ 174	424.9317	11889.32	3.865103	13.44167	0.717167	1.607705	1.181545	0.158696	0.892753	0.755581	949.5233	7.88216	973.2149	7.396664	1027.95	15.65099	1027.9	15.7	92.4	151
_19AR106_Spot_ 484	242.231	29590.66	1.508059	13.46512	0.727904	1.807113	1.001222	0.17835	0.635901	0.635125	1057.955	6.204498	1048.034	6.544732	1028.277	15.63775	1028.3	15.6	102.9	407
_19AR106_Spot_ 294	318.093	50632.7	2.114849	13.51822	0.652154	1.800155	1.061418	0.177646	0.820221	0.77276	1054.101	7.976087	1045.514	6.928687	1028.475	13.62202	1028.5	13.6	102.5	257
_19AR106_Spot_ 255	174.2182	38596.86	3.113249	13.49413	0.841627	1.762143	1.110241	0.173847	0.696961	0.627757	1033.275	6.654013	1031.636	7.191996	1029.025	17.49348	1029.0	17.5	100.4	223
_19AR106_Spot_ 325	227.8701	22913.8	2.97581	13.45835	0.717609	1.785368	1.194299	0.176088	0.742239	0.621485	1045.568	7.163946	1040.138	7.773141	1029.606	18.93472	1029.6	18.9	101.6	277
_19AR106_Spot_ 120	94.10728	15174.11	2.121721	13.41765	0.844083	1.820686	1.553215	0.179394	0.683581	0.440107	1063.665	6.702828	1052.932	10.1802	1031.6	28.20895	1031.6	28.2	103.1	102
_19AR106_Spot_ 557	184.7934	19509.27	1.889322	13.42983	0.793372	1.808963	1.139569	0.178239	0.739434	0.648872	1057.348	7.210866	1048.703	7.451811	1031.602	17.54521	1031.6	17.5	102.5	472
_19AR106_Spot_ 355	187.8929	24718.88	3.199193	13.4587	0.79521	1.84918	1.13577	0.181821	0.703605	0.619496	1076.914	6.978135	1063.138	7.484926	1035.831	18.02717	1035.8	18.0	104.0	300
_19AR106_Spot_ 565	175.9994	28967.34	2.316934	13.47521	0.57685	1.820379	0.858787	0.178871	0.606054	0.70571	1060.806	5.927951	1052.822	5.628263	1037.156	12.30654	1037.2	12.3	102.3	477
_19AR106_Spot_ 402	127.2222	20241.38	0.523652	13.38277	0.843865	1.81569	1.127295	0.178362	0.59738	0.529924	1058.018	5.828975	1051.132	7.381286	1037.71	19.32404	1037.7	19.3	102.0	342
_19AR106_Spot_ 377	103.3653	12280.43	3.034377	13.27658	1.098881	1.802524	1.317637	0.176919	0.661979	0.502398	1050.123	6.414918	1046.373	8.605338	1039.413	23.01886	1039.4	23.0	101.0	320
_19AR106_Spot_ 473	240.8294	22705.99	3.348446	13.3667	0.774743	1.800101	1.121703	0.176619	0.756689	0.674589	1048.475	7.322111	1045.495	7.322145	1040.131	16.73217	1040.1	16.7	100.8	398
_19AR106_Spot_ 84	121.5005	18417.81	2.637799	13.36871	0.945921	1.805712	1.391168	0.177135	0.761983	0.547729	1051.303	7.391648	1047.528	9.091313	1040.523	23.51178	1040.5	23.5	101.0	71
_19AR106_Spot_ 3	177.4005	23240.47	1.493349	13.34719	0.992479	1.784803	1.393611	0.175066	0.750681	0.538659	1039.962	7.209628	1039.932	9.069408	1040.729	23.71706	1040.7	23.7	99.9	3
_19AR106_Spot_ 362	305.9997	20771.32	1.185137	13.43185	0.719854	1.788522	1.098062	0.175409	0.796345	0.725228	1041.844	7.660945	1041.287	7.151288	1040.978	15.27765	1041.0	15.3	100.1	306
_19AR106_Spot_ 549	48.88046	6753.133	2.227552	13.09545	2.0104	1.868952	2.437253	0.183264	0.791149	0.324607	1084.779	7.898988	1070.16	16.12285	1041.346	46.52455	1041.3	46.5	104.2	464
_19AR106_Spot_ 12	196.0787	21663.89	1.631952	13.31956	0.729114	1.836425	1.068495	0.180054	0.660094	0.61778	1067.27	6.4927	1058.582	7.02443	1041.572	16.97498	1041.6	17.0	102.5	12
_19AR106_Spot_ 142	111.0483	12216.57	3.173957	13.36972	0.957598	1.883584	1.459558	0.184576	0.694824	0.476051	1091.924	6.979188	1075.325	9.680921	1042.685	25.91876	1042.7	25.9	104.7	124
_19AR106_Spot_ 318	250.7467	32784.89	7.943746	13.37039	0.697352	1.85495	1.024651	0.1817	0.716181	0.698952	1076.253	7.098853	1065.192	6.759986	1043.465	14.80306	1043.5	14.8	103.1	272
_19AR106_Spot_ 444	430.6476	57543.51	13.40081	13.49375	0.671029	1.797777	1.03656	0.176025	0.758216	0.731473	1045.224	7.315931	1044.652	6.763221	1044.313	14.27687	1044.3	14.3	100.1	376
_19AR106_Spot_ 439	399.7509	57906.61	2.813243	13.46396	0.649994	1.773996	0.989115	0.173626	0.697213	0.704886	1032.056	6.649181	1035.984	6.422872	1045.178	14.17124	1045.2	14.2	98.7	371
_19AR106_Spot_ 243	254.4548	44811.13	3.870892	13.40291	0.625223	1.829629	0.887812	0.179032	0.599664	0.67544	1061.686	5.869917	1056.146	5.828934	1045.611	13.2223	1045.6	13.2	101.5	212
_19AR106_Spot_ 198	212.4795	28309.94	3.058727	13.41962	0.670106	1.847009	0.974543	0.180686	0.627366	0.643754	1070.721	6.189121	1062.364	6.419729	1046.136	15.05856	1046.1	15.1	102.4	173
_19AR106_Spot_ 566	65.54604	8457.771	4.181693	13.20214	1.717442	1.887236	1.88794	0.184576	0.673088	0.35652	1091.926	6.760873	1076.61	12.53095	1046.628	35.58857	1046.6	35.6	104.3	478
_19AR106_Spot_ 556	221.8757	25700.68	1.991835	13.39598	0.704269	1.813638	1.030539	0.177327	0.730473	0.708827	1052.354	7.092508	1050.392	6.745015	1047.211	14.67637	1047.2	14.7	100.5	471
_19AR106_Spot_ 438	252.9654	34828.39	2.514462	13.39756	0.656745	1.807171	1.094074	0.176533	0.75299	0.688245	1048.004	7.283311	1048.055	7.151781	1049.059	16.01917	1049.1	16.0	99.9	370
_19AR106_Spot_ 72	53.64577	7182.339	3.263126	13.15505	1.074618	1.89667	1.435531	0.185254	0.692125	0.482139	1095.616	6.973643	1079.923	9.544386	1049.286	25.37064	1049.3	25.4	104.4	62
_19AR106_Spot_ 128	369.868	41315.1	4.142745	13.41223	0.65085	1.853892	1.079762	0.180944	0.761813	0.705538	1072.132	7.524584	1064.816	7.122161	1050.751	15.43963	1050.8	15.4	102.0	110
_19AR106_Spot_ 264	173.7633	27607.08	4.815386	13.30935	0.975639	1.774212	1.255698	0.173067	0.770985	0.613989	1028.988	7.332567	1036.063	8.15437	1051.922	19.98929	1051.9	20.0	97.8	230
_19AR106_Spot_ 82	269.7608	42161.66	5.728521	13.33955	0.851895	1.86192	1.182124	0.181543	0.810257	0.685425	1075.397	8.025477	1067.668	7.809172	1052.807	17.36066	1052.8	17.4	102.1	70
_19AR106_Spot_ 265	338.1803	44884.44	2.689376	13.33	0.753876	1.867013	1.103178	0.18192	0.755135	0.684509	1077.456	7.492656	1069.473	7.294584	1054.126	16.21746	1054.1	16.2	102.2	231
_19AR106_Spot_ 405	229.8939	26924.48	2.548112	13.30035	0.616897	1.880808	0.972208	0.183159	0.693849	0.713684	1084.208	6.924171	1074.347	6.44503	1055.287	13.73409	1055.3	13.7	102.7	345
_19AR106_Spot_ 286	81.778	9313.317	4.967366	13.11537	1.126762	1.898054	1.790642	0.184697	1.300232	0.726126	1092.586	13.06751	1080.408	11.9086	1056.821	24.7899	1056.8	24.8	103.4	250
_19AR106_Spot_ 189	196.6891	17112	2.973911	13.32944	0.973171	1.806476	1.334799	0.175647	0.852812	0.638907	1043.151	8.213651	1047.804	8.724236	1058.41	20.6796	1058.4	20.7	98.6	165
_19AR106_Spot_ 34	383.5259	63822.24	17.75005	13.36523	0.840793	1.830108	1.239358	0.177887	0.894337	0.721613	1055.422	8.706839	1056.318	8.137852	1059.064	17.28917	1059.1	17.3	99.7	29
_19AR106_Spot_ 50	59.47125	7719.777	2.79179	13.10486	1.405243	1.933977	1.891297	0.187954	0.832413	0.440128	1110.282	8.49003	1092.916	12.65922	1059.38	34.202	1059.4	34.2	104.8	45
_19AR106_Spot_ 477	228.5743	3846.259	1.8745	12.6549	0.734812	1.991825	1.246886	0.192574	0.693364	0.556077	1135.302	7.217579	1112.742	8.429111	1069.82	20.80648	1069.8	20.8	106.1	401
_19AR106_Spot_ 153	343.2059	39874.51	2.528761	13.37603	0.739216	1.854635	1.108969	0.179092	0.805917	0.726727	1062.01	7.891078	1065.08	7.315846	1072.268	15.2844	1072.3	15.3	99.0	134
_19AR106_Spot_ 154	383.476	39880.43	3.908328	13.3619	0.699873	1.897288	1.04911	0.183016	0.753858	0.718569	1083.427	7.51805	1080.139	6.975883	1074.403	14.63252	1074.4	14.6	100.8	135
_19AR106_Spot_ 51	272.8051	5124.444	2.587596	12.80154	0.717581	1.958103	1.434087	0.188079	0.711821	0.496358	1110.959	7.264128	1101.232	9.639192	1082.915	24.95082	1082.9	25.0	102.6	46
_19AR106_Spot_ 204	302.0916	14039.09	3.142558	13.08395	0.667285	1.820112	1.042526	0.174627	0.724105	0.694567	1037.556	6.939557	1052.726	6.832116	1085.195	15.02753	1085.2	15.0	95.6	177
_19AR106_Spot_ 574	299.205	40707.68	5.345793	13.12823	0.660621	1.938021	1.02976	0.185816	0.744626	0.723106	1098.668	7.521793	1094.315	6.897257	1086.539	14.2395	1086.5	14.2	101.1	482
_19AR106_Spot_ 16	410.1065	30285.26	8.558019	13.09213	0.63622	1.964911	0.949009	0.188298	0.68506	0.721869	1112.148	6.997886	1103.566	6.38612	1087.54	13.14349	1087.5	13.1	102.3	16
_19AR106_Spot_ 579	158.8813	15501.1	2.879164	12.99218	0.811284	1.89982	1.290111	0.181808	0.829779	0.643184	1076.842	8.228987	1081.026	8.582401	1090.321	19.77106	1090.3	19.8	98.8	487
_19AR106_Spot_ 390	138.404	23628.95	3.433134	13.05551	0.821146	2.017154	1.07826	0.192913	0.576342	0.534511	1137.136	6.008293	1121.302	7.319857	1091.603	18.23537	1091.6	18.2	104.2	331
_19AR106_Spot_ 199	87.97847	8810.858	3.034385	12.93877	0.901214	2.037683	1.262976	0.194774	0.816116	0.646185	1147.183	8.576592	1128.187	8.602596	1092.657	19.2854	1092.7	19.3	105.0	174
_19AR106_Spot_ 555	179.7726	29573.32	2.319462	13.10316	0.738301	1.943779	1.066393	0.185686	0.631658	0.592331	1097.965	6.376908	1096.303	7.149835	1093.895	17.18603	1093.9	17.2	100.4	470
_19AR106_Spot_ 596	310.2034	53941.09	2.944106	13.07966	0.546108	1.995883	0.846223	0.190656	0.631103	0.745788	1124.927	6.514515	1114.118	5.724392	1093.98	11.30066	1094.0	11.3	102.8	501
_19AR106_Spot_ 452	588.3112	55446.5	4.560681	13.13369	0.622412	1.864034	1.038299	0.177811	0.825491	0.795042	1055.004	8.033661	1068.418	6.861746	1096.755	12.5861	1096.8	12.6	96.2	383
_19AR106_Spot_ 179	222.6686	20943.91	0.947387	12.98255	0.892303	1.858517	1.33801	0.176597	0.791716	0.591711	1048.359	7.660271	1066.46	8.833364	1104.524	21.57812	1104.5	21.6	94.9	155
_19AR106_Spot_ 528	68.01384	8821.53	2.661246	12.74047	1.500603	2.093364	1.848387	0.198504	0.830394	0.449253	1167.28	8.866102	1146.631	12.70162	1108.633	32.99605	1108.6	33.0	105.3	443
_19AR106_Spot_ 92	171.1206	28974.48	6.676243	12.94763	0.816384	1.98549	1.048213	0.188175	0.628933	0.600005	1111.479	6.42101	1110.589	7.07846	1109.7	16.76703	1109.7	16.8	100.2	79
_19AR106_Spot_ 132	39.162	7307.061	1.719445	12.7357	2.13764	2.149395	3.129172	0.203422	0.854543	0.273089	1193.676	9.31176	1164.858	21.68775	1112.513	60.12026	1112.5	60.1	107.3	114
_19AR106_Spot_ 202	72.61153	9059.162	1.620683	12.7743	0.879245	2.138035	2.00307	0.201592	0.772608	0.385712	1183.865	8.355901	1161.189	13.85831	1120.01	36.86392	1120.0	36.9	105.7	176
_19AR106_Spot_ 46	50.43005	6220.611	1.607745	12.60557	1.760592	2.165855	2.846901	0.20408	0.687856	0.241616	1197.202	7.515561	1170.151	19.7786	1121.326	55.10033	1121.3	55.1	106.8	41
_19AR106_Spot_ 445	329.9468	6752.285	3.235384	12.55595	0.719468	2.069473	2.538047	0.193112	0.816333	0.321638	1138.21	8.517524	1138.758	17.37672	1140.656	47.81212	1140.7	47.8	99.8	377
_19AR106_Spot_ 162	305.953	15620.87	1.155038	12.67342	0.729367	2.120265	1.434592	0.196894	0.8866	0.618015	1158.613	9.40206	1155.423	9.898531	1150.325	22.39604	1150.3	22.4	100.7	142
_19AR106_Spot_ 339	153.314	26116.81	1.850082	12.69478	0.958585	2.224058	1.346198	0.206407	0.824169	0.61222	1209.645	9.090017	1188.649	9.429627	1151.499	21.14081	1151.5	21.1	105.0	289
_19AR106_Spot_ 213	242.6018	28679.36	5.313692	12.68831	0.672942	2.188057	1.012484	0.202715	0.7303	0.721296	1189.891	7.93494	1177.247	7.055983	1154.926	13.90619	1154.9	13.9	103.0	184
_19AR106_Spot_ 192	159.164	22510.67	5.769341	12.73427	0.872149	1.878432	1.328084	0.173949	0.87681	0.660207	1033.834	8.375238	1073.509	8.80047	1155.842	19.79156	1155.8	19.8	89.4	167

_19AR106_Spot_ 312	50.73333	9578.388	1.726939	12.46583	1.628009	2.137361	2.367214	0.197786	0.822046	0.347263	1163.413	8.750443	1160.971	16.3764	1157.259	44.04377	1157.3	44.0	100.5	266
_19AR106_Spot_ 463	198.6033	32068.22	4.99689	12.66686	0.786679	2.121486	1.041833	0.196245	0.645316	0.619405	1155.119	6.82449	1155.82	7.189755	1157.979	16.22347	1158.0	16.2	99.8	390
_19AR106_Spot_ 178	305.7145	45351.7	1.865906	12.69374	0.707042	2.166465	1.043167	0.199775	0.735165	0.704743	1174.114	7.891239	1170.346	7.247165	1164.228	14.67641	1164.2	14.7	100.8	154
_19AR106_Spot_ 231	75.30951	7459.953	2.236676	12.37196	0.916669	2.262703	1.463065	0.2082	1.045725	0.714749	1219.221	11.61657	1200.747	10.30288	1168.504	20.28311	1168.5	20.3	104.3	201
_19AR106_Spot_ 70	467.4198	69471.72	3.051553	12.60525	0.556289	2.167971	0.829465	0.198129	0.600455	0.723907	1165.26	6.400932	1170.829	5.763745	1182.022	11.31099	1182.0	11.3	98.6	60
_19AR106_Spot_ 422	121.4257	15350.82	3.046734	12.34908	0.714378	2.309505	1.016195	0.209728	0.688689	0.677714	1227.367	7.696794	1215.209	7.200624	1194.555	14.7333	1194.6	14.7	102.7	359
_19AR106_Spot_ 524	67.07546	3941.423	3.968078	11.90832	1.04231	2.246297	3.613451	0.203729	0.834521	0.230949	1195.323	9.105009	1195.629	25.39344	1197.059	69.35845	1197.1	69.4	99.9	439
_19AR106_Spot_ 125	103.1379	13714.04	2.289218	12.37051	0.910983	2.206566	1.185567	0.200087	0.712535	0.601008	1175.789	7.658282	1183.125	8.284046	1197.439	18.68732	1197.4	18.7	98.2	107
_19AR106_Spot_ 552	191.088	27271.69	4.560262	12.44005	0.643904	2.268495	0.955772	0.205635	0.659681	0.690207	1205.519	7.253253	1202.548	6.735667	1198.054	13.63839	1198.1	13.6	100.6	467
_19AR106_Spot_ 457	63.01512	7432.472	1.856048	12.15909	1.401584	2.334129	1.913192	0.210911	0.61255	0.320172	1233.667	6.877751	1222.736	13.60056	1204.342	35.71073	1204.3	35.7	102.4	386
_19AR106_Spot_ 384	107.8441	16594.95	2.662142	12.18441	0.775262	2.362838	1.406576	0.211905	0.595063	0.423058	1238.96	6.707401	1231.441	10.03541	1219.176	25.04921	1219.2	25.0	101.6	326
_19AR106_Spot_ 568	440.3306	79537.95	9.681078	12.33204	0.473255	2.295048	0.782407	0.2057	0.619753	0.792111	1205.868	6.816042	1210.764	5.533465	1220.34	9.393595	1220.3	9.4	98.8	479
_19AR106_Spot_ 427	72.72998	11029.6	3.788558	12.12629	1.214152	2.358192	1.703074	0.211167	0.799422	0.469399	1235.029	8.984947	1230.038	12.14389	1222.135	29.56073	1222.1	29.6	101.1	362
_19AR106_Spot_ 571	334.2788	29982	11.06904	12.20747	0.64038	2.394643	1.121819	0.212957	0.849629	0.757368	1244.553	9.616003	1241	8.035414	1235.709	14.35933	1235.7	14.4	100.7	480
_19AR106_Spot_ 554	133.4241	22471.2	2.428953	12.14205	0.842486	2.430066	1.380614	0.2154	1.06698	0.77283	1257.521	12.18992	1251.54	9.931882	1242.128	17.18576	1242.1	17.2	101.2	469
_19AR106_Spot_ 364	144.9178	31412.48	1.795664	12.09844	0.75973	2.461153	1.150916	0.216825	0.773932	0.672448	1265.074	8.890003	1260.702	8.310004	1254.079	16.64551	1254.1	16.6	100.9	308
_19AR106_Spot_ 47	49.90694	7178.664	3.167355	11.79799	1.219048	2.312556	1.93397	0.202779	0.826105	0.427155	1190.231	8.978224	1216.145	13.70992	1263.275	34.16316	1263.3	34.2	94.2	42
_19AR106_Spot_ 470	78.95677	18546.3	1.516611	11.66622	0.934886	2.717073	1.322707	0.232648	0.687277	0.519599	1348.364	8.362014	1333.133	9.817645	1309.579	21.93366	1309.6	21.9	103.0	396
_19AR106_Spot_ 458	123.8808	17374.93	4.813747	11.22147	0.618025	2.633427	1.117515	0.216643	0.822103	0.735653	1264.112	9.436833	1310.023	8.224277	1386.808	14.53296	1386.8	14.5	91.2	387
_19AR106_Spot_ 165	115.7935	18751.82	2.154208	11.19422	0.888604	2.653926	1.350151	0.217035	0.685111	0.507433	1266.189	7.876011	1315.736	9.957617	1398.215	22.30225	1398.2	22.3	90.6	145
_19AR106_Spot_ 22	131.7038	38148.1	3.364005	10.89404	0.851921	3.242417	1.219152	0.257575	0.859495	0.704994	1477.423	11.34832	1467.364	9.461408	1453.647	16.45057	1453.6	16.5	101.6	19
_19AR106_Spot_ 258	87.64097	19472.45	2.227189	10.76311	0.660623	3.239875	0.999694	0.255803	0.699808	0.700023	1468.333	9.189281	1466.755	7.756762	1465.28	13.56133	1465.3	13.6	100.2	225
_19AR106_Spot_ 353	313.7952	4353.177	3.618383	10.32262	0.629017	2.821858	3.483779	0.218344	0.939633	0.269717	1273.118	10.85546	1361.361	26.12384	1503.548	63.41546	1503.5	63.4	84.7	298
_19AR106_Spot_ 359	172.391	35825.51	2.168273	10.26195	0.708345	3.633944	1.105174	0.270891	0.786924	0.712037	1545.323	10.8128	1556.997	8.80033	1573.668	14.52881	1573.7	14.5	98.2	304
_19AR106_Spot_ 371	154.8883	41781.97	3.590774	9.997811	0.633046	3.99618	0.951975	0.291291	0.679608	0.713893	1647.975	9.882788	1633.42	7.731622	1615.524	12.41309	1615.5	12.4	102.0	315
_19AR106_Spot_ 291	85.08549	24465.46	3.116843	9.32943	0.835178	4.233583	1.166365	0.288828	0.767575	0.658092	1635.669	11.08878	1680.556	9.580457	1737.837	16.10097	1737.8	16.1	94.1	254
_19AR106_Spot_ 322	232.5658	29122.81	1.717563	9.30417	0.670111	4.301645	1.023116	0.292793	0.758839	0.741694	1655.469	11.07897	1693.676	8.429247	1742.078	12.57448	1742.1	12.6	95.0	275
_19AR106_Spot_ 500	117.005	27300.83	1.280691	9.190005	0.549418	4.844688	0.912532	0.325042	0.598323	0.655673	1814.306	9.461591	1792.693	7.680524	1768.415	12.58367	1768.4	12.6	102.6	422
_19AR106_Spot_ 251	158.3221	36167.53	2.840827	9.030447	0.57707	4.12266	0.839943	0.271919	0.59931	0.713513	1550.536	8.259431	1658.805	6.863858	1799.525	10.70849	1799.5	10.7	86.2	219
_19AR106_Spot_ 94	239.4574	13408.77	1.264698	8.944457	0.611143	4.295287	1.104798	0.28261	0.780429	0.7064	1604.494	11.08524	1692.458	9.099702	1803.992	14.21972	1804.0	14.2	88.9	81
_19AR106_Spot_ 520	111.3132	37416.02	1.747129	8.738784	0.798731	5.326967	1.180122	0.340506	0.815686	0.691188	1889.105	13.35667	1873.2	10.08917	1856.371	15.40973	1856.4	15.4	101.8	436
_19AR106_Spot_ 482	218.1669	33840.02	2.184371	8.739168	0.56187	5.109698	0.86822	0.326228	0.643048	0.740652	1820.071	10.19682	1837.719	7.372972	1858.533	10.53684	1858.5	10.5	97.9	405
_19AR106_Spot_ 544	140.2268	39239	6.482343	8.722902	0.689439	5.285897	1.164745	0.336523	0.924872	0.794056	1869.919	15.012	1866.587	9.945484	1863.649	12.7807	1863.6	12.8	100.3	459
_19AR106_Spot_ 138	193.3671	26040.58	1.221929	8.787557	0.948911	4.987299	1.454107	0.317453	1.099634	0.756227	1777.276	17.08092	1817.171	12.29934	1863.99	17.17565	1864.0	17.2	95.3	120
_19AR106_Spot_ 314	183.6257	76017.4	0.922456	8.681615	0.650459	5.473277	0.950808	0.346547	0.683622	0.718991	1918.089	11.34162	1896.413	8.163101	1873.54	11.91697	1873.5	11.9	102.4	268
_19AR106_Spot_ 321	167.4856	39931.45	0.879943	8.598479	0.697648	5.428744	1.039394	0.340983	0.765628	0.73661	1891.396	12.55007	1889.404	8.9124	1887.984	12.65286	1888.0	12.7	100.2	274
_19AR106_Spot_ 144	326.5183	14273.1	7.042035	8.596113	0.641855	4.459118	0.964222	0.277904	0.710388	0.736747	1580.8	9.958896	1723.397	7.997284	1902.007	11.71542	1902.0	11.7	83.1	126
_19AR106_Spot_ 513	104.1473	8037.509	1.187842	8.187713	0.788265	5.777726	1.192124	0.349416	0.859464	0.720952	1931.808	14.34641	1943.079	10.31904	1955.879	14.75079	1955.9	14.8	98.8	432
_19AR106_Spot_ 31	61.94097	19283.74	1.383368	8.247535	0.904296	5.891649	1.279628	0.354657	0.892152	0.697196	1956.799	15.05695	1960.004	11.10823	1964.15	16.36393	1964.1	16.4	99.6	26
_19AR106_Spot_ 206	68.48375	15423.18	0.922678	8.249254	0.745829	5.808482	1.233602	0.349206	0.734212	0.595178	1930.805	12.25021	1947.677	10.68645	1966.422	17.67905	1966.4	17.7	98.2	178
_19AR106_Spot_ 33	62.10095	17863.35	1.209313	8.234378	0.919527	6.073932	1.322385	0.364923	0.888355	0.671782	2005.467	15.31083	1986.512	11.52964	1967.602	17.46688	1967.6	17.5	101.9	28
_19AR106_Spot_ 173	456.3892	108687.2	6.450875	8.258491	0.595218	5.837219	0.914189	0.348883	0.691576	0.756491	1929.262	11.53092	1951.953	7.925039	1976.868	10.6507	1976.9	10.7	97.6	150
_19AR106_Spot_ 465	478.6237	169504	3.770142	7.939365	0.597477	6.502926	0.906403	0.375165	0.679879	0.750085	2053.658	11.95688	2046.294	7.976975	2039.634	10.59909	2039.6	10.6	100.7	392
_19AR106_Spot_ 450	217.7776	62507.88	3.202553	7.865508	0.645726	6.527422	0.970067	0.371845	0.707787	0.729627	2038.074	12.36738	2049.604	8.541558	2061.977	11.70196	2062.0	11.7	98.8	381
_19AR106_Spot_ 539	98.00886	24660.59	1.063501	7.791832	0.750545	6.714638	1.089938	0.382492	0.776529	0.712452	2087.916	13.84957	2074.549	9.632782	2062.056	13.48942	2062.1	13.5	101.3	454
_19AR106_Spot_ 197	70.56843	17805.69	7.241523	7.719506	0.677465	6.993659	1.133968	0.393147	0.904496	0.797638	2137.408	16.45448	2110.625	10.07404	2085.374	12.03065	2085.4	12.0	102.5	172
_19AR106_Spot_ 11	112.9222	24912.6	1.628076	6.846579	0.626415	8.747352	0.996623	0.437812	0.698179	0.700545	2340.84	13.70477	2312.023	9.081603	2287.401	12.23823	2287.4	12.2	102.3	11
_19AR106_Spot_ 15	110.0084	28142.6	1.372867	6.092306	0.579882	10.01953	0.918239	0.445277	0.696151	0.758137	2374.22	13.82616	2436.583	8.477742	2489.791	10.09094	2489.8	10.1	95.4	15
_19AR106_Spot_ 277	438.4196	73730.82	1.428767	5.666322	0.566885	12.26831	0.985534	0.506491	0.803835	0.815634	2641.631	17.42173	2625.15	9.253003	2613.176	9.493877	2613.2	9.5	101.1	243
_19AR106_Spot_ 328	371.3759	40558.86	3.451462	5.475174	0.708794	12.39382	1.088397	0.493916	0.812406	0.746424	2587.596	17.31487	2634.709	10.22663	2671.81	11.9923	2671.8	12.0	96.8	279
_19AR106_Spot_ 276	233.0627	37473.15	3.091834	5.46386	0.581869	10.912	1.083034	0.434771	0.901373	0.832266	2327.191	17.60767	2515.659	10.07409	2672.159	9.940689	2672.2	9.9	87.1	242
_19AR106_Spot_ 360	146.869	53772.36	1.669025	5.465559	0.700556	13.26234	1.04691	0.525114	0.77515	0.740417	2720.831	17.20506	2698.505	9.885131	2682.536	11.63587	2682.5	11.6	101.4	305
**19AR107**																					-26.94419	-65.76612	510	5.1	
19AR107 175	1440.319	238.3277	3.853559	4.479605	60.60138	0.006858	63.94166	0.000292	4.137572	0.064709	1.882255	0.077868	6.939283	4.421993	2561.422	1227.632	1.9	0.1	0.1	147					0.149
19AR107 208	1753.214	324.6647	2.479836	8.250388	27.10355	0.003356	38.12483	0.000315	6.079682	0.159468	2.029586	0.123373	3.402155	1.294896	1129.873	777.4358	2.0	0.1	0.2	178
19AR107 534	2168.81	376.6878	3.242201	9.159455	18.65037	0.003187	32.92512	0.000326	4.496615	0.136571	2.10264	0.094532	3.231349	1.062235	954.3683	684.8525	2.1	0.1	0.2	467
19AR107 363	2295.609	284.6715	0.81168	5.372285	21.99515	0.006347	30.39206	0.000329	5.119244	0.16844	2.120764	0.108549	6.423868	1.946185	2226.615	531.9785	2.1	0.1	0.1	319
19AR107 193	3227.568	419.4297	2.300288	11.11436	8.745564	0.002533	25.10863	0.000334	2.729983	0.108727	2.149826	0.05868	2.569014	0.644229	416.5954	NA	2.1	0.1	0.5	163
19AR107 184	3065.38	754.7405	4.392564	10.12198	13.06918	0.003676	17.34339	0.000334	2.657217	0.153212	2.151283	0.057155	3.725812	0.644998	1194.88	340.5502	2.2	0.1	0.2	156
19AR107 527	1970.052	489.8596	3.561992	6.478852	14.55372	0.005934	17.79872	0.000339	3.986382	0.22397	2.18767	0.087194	6.007189	1.066047	2054.7	308.6822	2.2	0.1	0.1	461
19AR107 420	2361.967	328.2395	4.084639	9.320506	14.46747	0.003052	21.16506	0.00035	4.166744	0.196869	2.258328	0.094082	3.09458	0.653973	715.2434	445.2288	2.3	0.1	0.3	369
19AR107 279	1475.008	313.2674	3.124186	7.023566	18.19111	0.004901	36.53052	0.000361	8.948935	0.244971	2.327393	0.208239	4.964321	1.809069	1595.531	683.8783	2.3	0.2	0.1	242
19AR107 468	1745.816	306.406	1.163883	8.220829	14.64185	0.003829	29.03331	0.000371	4.796604	0.16521	2.391856	0.114707	3.880513	1.124492	1064.903	587.6617	2.4	0.1	0.2	412
19AR107 310	1984.586	365.257	4.859122	8.610543	23.19258	0.003978	24.53827	0.000374	4.646813	0.18937	2.407991	0.111874	4.030882	0.987149	1127.65	486.9631	2.4	0.1	0.2	270
19AR107 489	2057.759	635.2537	4.674605	11.13817	18.25148	0.005123	26.63903	0.000554	7.856209	0.294913	3.569207	0.280327	5.188774	1.378714	841.5528	538.1074	3.6	0.3	0.4	429
19AR107 521	1404.832	521.2453	2.056443	9.223493	11.51416	0.006703	21.4734	0.0006	4.031451	0.187742	3.868111	0.155894	6.783865	1.451872	1222.289	419.0477	3.9	0.2	0.3	456
19AR107 211	1715.286	1009.6	3.916755	10.15238	22.01766	0.007863	27.09232	0.000675	4.605916	0.170008	4.352882	0.200422	7.952876	2.146206	1303.274	528.0481	4.4	0.2	0.3	181
19AR107 576	1028.8	357.2278	3.029847	6.377422	32.35793	0.011433	35.3661	0.000697	4.548092	0.1286	4.490362	0.204155	11.54351	4.059392	1942.093	648.9916	4.5	0.2	0.2	504
19AR107 118	3165.713	1061.726	4.22906	13.20464	6.534996	0.006893	10.42092	0.000806	1.712235	0.164307	5.19443	0.088905	6.975422	0.724412	675.7556	220.2822	5.2	0.1	0.8	98
19AR107 277	1265.607	644.7824	2.826071	8.981124	12.14122	0.010296	22.56822	0.000831	4.524925	0.2005	5.356273	0.242267	10.40072	2.335279	1422.783	427.6908	5.4	0.2	0.4	241
19AR107 598	319.0723	172.2288	1.529536	3.84574	12.54052	0.025395	30.31774	0.000973	6.965623	0.229754	6.272382	0.436698	25.4634	7.624078	2735.903	497.9594	6.3	0.4	0.2	524
19AR107 274	928.6567	596.0101	1.270398	12.85031	4.716072	0.007831	32.45448	0.001065	2.902825	0.089443	6.863871	0.19914	7.920242	2.56048	343.0666	NA	6.9	0.2	2.0	238
19AR107 466	1335.541	971.5912	2.084527	10.52757	17.05458	0.011984	22.08512	0.001086	2.328313	0.105425	6.99861	0.162861	12.09595	2.655563	1198.223	438.2662	7.0	0.2	0.6	410
19AR107 331	2924.201	1916.096	2.69967	16.68412	8.873217	0.008243	10.03336	0.001144	1.158289	0.115444	7.369373	0.08531	8.335967	0.832954	298.1871	227.8826	7.4	0.1	2.5	288
19AR107 2	1330.211	905.1076	2.516208	13.90223	6.932675	0.010019	21.43872	0.001302	1.582953	0.073836	8.385596	0.132654	10.12229	2.159311	446.2956	NA	8.4	0.1	1.9	2
19AR107 248	716.1515	610.0632	0.776714	13.66168	10.30039	0.012136	29.54983	0.001801	3.272048	0.11073	11.60089	0.379245	12.24837	3.597645	142.4157	NA	11.6	0.4	8.1	214
19AR107 372	487.199	490.6346	1.091265	11.75493	14.22524	0.013698	27.55504	0.001803	4.022554	0.145983	11.61061	0.466623	13.81427	3.780768	417.6204	NA	11.6	0.5	2.8	328
19AR107 357	776.7123	908.6424	0.908985	10.33291	14.48335	0.020326	19.3194	0.001823	2.139523	0.110745	11.74087	0.25097	20.432	3.90791	1219.122	380.8991	11.7	0.3	1.0	313
19AR107 16	975.3185	1525.267	0.570219	12.14338	13.16796	0.01841	13.78821	0.00183	2.194808	0.15918	11.78658	0.258457	18.52361	2.530924	1013.751	277.0918	11.8	0.3	1.2	12
19AR107 17	1006.745	1362.035	0.595936	16.36639	9.020028	0.012763	13.81498	0.001841	2.929404	0.212045	11.85645	0.347004	12.87706	1.767733	209.0258	NA	11.9	0.3	5.7	13
19AR107 367	467.0635	609.1237	1.329452	7.003171	10.00096	0.0307	12.79497	0.001849	3.98911	0.311772	11.91022	0.474673	30.70328	3.869694	1962.948	217.7401	11.9	0.5	0.6	323
19AR107 81	1054.351	1157.455	0.643189	12.35253	10.08898	0.017597	13.68202	0.00187	2.12293	0.155162	12.04357	0.255438	17.71182	2.402326	876.9415	280.9951	12.0	0.3	1.4	66
19AR107 533	558.2436	552.554	1.224364	9.987759	16.65254	0.019216	20.57015	0.001882	2.333162	0.113425	12.11949	0.282502	19.32658	3.93793	1043.986	416.482	12.1	0.3	1.2	466
19AR107 494	571.6961	577.6689	1.590927	10.8246	9.021187	0.018175	15.47826	0.001955	2.234988	0.144395	12.59198	0.281154	18.28869	2.805438	851.5625	320.0519	12.6	0.3	1.5	434
19AR107 238	478.567	620.1323	0.732805	12.06429	11.55276	0.016183	33.49325	0.001984	3.029305	0.090445	12.77606	0.386642	16.30029	5.415959	574.0287	NA	12.8	0.4	2.2	206
19AR107 418	223.3777	369.2548	0.904567	5.013249	58.00905	0.046568	59.48456	0.00204	7.887587	0.132599	13.13457	1.034946	46.21599	26.8814	2514.302	1111.111	13.1	1.0	0.5	367
19AR107 406	712.3055	496.423	1.559461	11.48435	12.32407	0.016942	47.3824	0.00213	3.48911	0.073637	13.71915	0.478167	17.05815	8.015215	518.3462	NA	13.7	0.5	2.6	355
19AR107 264	536.1139	1975.157	2.794206	11.99514	12.95642	0.056772	13.86932	0.005384	1.652455	0.119145	34.61291	0.57043	56.06837	7.565642	1108.507	276.3954	34.6	0.6	3.1	228
19AR107 594	577.0392	1748.924	3.271592	12.15498	5.61664	0.056761	9.253483	0.00557	6.621663	0.715586	35.80497	2.364313	56.05745	5.046717	1039.921	130.6243	35.8	2.4	3.4	520
19AR107 415	279.2238	1106.891	1.967485	9.681594	14.62241	0.072416	20.26202	0.005781	2.467734	0.121791	37.16133	0.914405	70.98991	13.89355	1444.206	387.0369	37.2	0.9	2.6	364
19AR107 148	530.5782	3023.209	0.694267	16.21173	3.968865	0.063117	7.258681	0.008057	1.371057	0.188885	51.73211	0.706438	62.14657	4.375773	485.3133	157.5542	51.7	0.7	10.7	121
19AR107 361	1786.826	8952.643	3.814296	16.27735	1.905044	0.077643	4.85397	0.009448	3.708652	0.764045	60.62205	2.237723	75.92685	3.551059	590.033	67.92968	60.6	2.2	10.3	317
19AR107 209	2808.2	12880.25	5.646674	14.95108	0.923039	0.091953	1.793346	0.010155	1.124611	0.627102	65.13473	0.728824	89.32118	1.533405	796.8057	29.29031	65.1	0.7	8.2	179
19AR107 164	325.1906	3237.88	1.751181	16.1358	5.416366	0.092916	6.740082	0.011763	1.07956	0.16017	75.38638	0.809101	90.21628	5.818409	503.6366	146.5814	75.4	0.8	15.0	136
19AR107 455	71.95292	1745.871	0.792799	14.89301	8.607293	0.334783	10.25926	0.041338	1.099211	0.107143	261.1238	2.812945	293.2106	26.13328	558.2703	222.9233	261.1	2.8	46.8	401
19AR107 501	698.6267	13485.02	3.372539	17.53814	1.17581	0.360366	2.014935	0.046818	1.502387	0.745626	294.9563	4.331535	312.4876	5.419792	446.3631	29.85327	295.0	4.3	66.1	440
19AR107 96	191.042	5809.133	1.370988	16.76326	3.85926	0.385825	4.107447	0.048849	0.984216	0.239617	307.4513	2.954959	331.3148	11.61188	503.4172	87.79477	307.5	3.0	61.1	79
19AR107 301	228.8388	5671.322	8.566403	17.71535	2.740834	0.397089	3.001657	0.053543	0.798446	0.266002	336.2354	2.615846	339.5345	8.662935	363.1449	65.26569	336.2	2.6	92.6	262
19AR107 350	331.7992	13240.98	3.484503	17.33639	2.51853	0.457979	2.784081	0.058817	0.996327	0.357866	368.4272	3.567813	382.8516	8.880089	471.9774	57.55491	368.4	3.6	78.1	306
19AR107 322	226.3786	3034.331	6.422478	17.43118	2.56828	0.498586	3.751687	0.068851	1.033542	0.275487	429.2318	4.291821	410.7447	12.67469	309.1369	82.138	429.2	4.3	138.8	281
19AR107 124	462.7814	2747.326	4.077685	16.97415	1.049018	0.528669	1.744122	0.071726	0.783035	0.448957	446.5471	3.378264	430.9263	6.12467	349.2516	35.21453	446.5	3.4	127.9	104
19AR107 454	251.1482	833.1756	0.500205	15.18412	2.072474	0.484893	4.084742	0.073047	2.707632	0.662865	454.4843	11.882	401.424	13.54474	107.4139	72.26173	454.5	11.9	423.1	400
19AR107 119	183.3735	8084.609	1.468785	17.5915	1.974992	0.554603	2.17886	0.073309	0.708234	0.325048	456.0601	3.118367	448.0074	7.892793	407.8255	46.13087	456.1	3.1	111.8	99
19AR107 12	507.7283	18063.24	12.78067	16.68249	1.027977	0.598652	1.3963	0.073431	0.829976	0.59441	456.7947	3.660076	476.378	5.309251	572.7556	24.41019	456.8	3.7	79.8	8
19AR107 170	187.3918	5878.528	1.476434	17.62239	2.128429	0.54841	4.420993	0.073567	0.701735	0.158728	457.6078	3.099866	443.9547	15.90028	374.7534	98.27603	457.6	3.1	122.1	142
19AR107 71	214.0682	9505.914	1.261421	17.38069	1.827096	0.566325	2.38679	0.073618	0.745645	0.312405	457.9137	3.295963	455.6351	8.762719	445.1046	50.41396	457.9	3.3	102.9	57
19AR107 306	304.8061	15204.8	20.3624	17.26111	1.255236	0.581556	1.57214	0.074009	0.726933	0.462384	460.2656	3.229171	465.461	5.869932	492.1532	30.74011	460.3	3.2	93.5	267
19AR107 82	448.9497	9862.548	10.43266	17.149	1.39377	0.578795	2.166231	0.074161	0.830401	0.383339	461.1758	3.695834	463.6871	8.06386	477.0847	44.23613	461.2	3.7	96.7	67
19AR107 284	82.15251	3019.609	2.04441	16.89737	4.514147	0.554661	5.14935	0.074198	1.084153	0.210542	461.3971	4.82743	448.0451	18.65619	381.0266	113.2362	461.4	4.8	121.1	246
19AR107 24	192.146	5031.885	6.199746	17.02937	2.59565	0.572878	2.829911	0.07439	0.632148	0.223381	462.55	2.821562	459.8739	10.4661	447.4735	61.31797	462.5	2.8	103.4	20
19AR107 73	142.4322	7438.755	1.549214	16.52085	3.896524	0.599919	4.336541	0.074583	0.818938	0.188846	463.7087	3.664123	477.1824	16.51225	543.432	93.14383	463.7	3.7	85.3	59
19AR107 185	570.005	18975.91	17.59088	17.44296	1.192791	0.581328	1.50449	0.074672	0.658333	0.437579	464.24	2.948793	465.3144	5.615943	471.56	29.94963	464.2	2.9	98.4	157
19AR107 308	213.204	7314.474	41.10612	17.76945	2.298299	0.559431	2.999818	0.074729	0.721168	0.240404	464.5842	3.23255	451.1563	10.92753	384.2417	65.4143	464.6	3.2	120.9	269
19AR107 66	307.9684	9973.976	11.82283	17.76182	1.75807	0.562941	2.716857	0.07474	0.749165	0.275747	464.6508	3.358506	453.4388	9.936438	397.9418	58.54042	464.7	3.4	116.8	52
19AR107 26	155.339	5130.625	1.43695	16.96716	2.996714	0.578688	5.215086	0.07478	0.712075	0.136541	464.8908	3.193823	463.6179	19.41299	458.3042	114.6675	464.9	3.2	101.4	22
19AR107 241	91.51697	4114.156	1.82868	16.2368	3.615593	0.595828	5.640486	0.074812	0.796835	0.141271	465.0812	3.575404	474.5825	21.38678	521.7116	122.6136	465.1	3.6	89.1	208
19AR107 224	163.5454	6448.989	1.828202	17.03897	2.033368	0.580804	2.283561	0.074894	0.657157	0.287777	465.5748	2.951685	464.9783	8.519311	463.02	48.48202	465.6	3.0	100.6	193
19AR107 428	233.6796	3171.876	12.93584	17.24992	2.228545	0.551342	5.725982	0.074971	0.837602	0.146281	466.0343	3.765751	445.8757	20.66587	344.122	128.261	466.0	3.8	135.4	376
19AR107 327	212.8397	4780.483	2.332671	17.36634	1.758646	0.563835	5.459341	0.07504	0.853553	0.156347	466.4473	3.840746	454.0195	19.98883	392.5318	121.0538	466.4	3.8	118.8	286
19AR107 109	211.5991	9193.452	1.202165	17.75095	2.423163	0.566844	3.768295	0.075042	0.788217	0.209171	466.4622	3.546862	455.9711	13.84329	404.3849	82.50508	466.5	3.5	115.4	90
19AR107 247	374.5534	16411.41	6.005627	17.69677	1.527118	0.574181	1.871741	0.07511	0.692793	0.370133	466.8705	3.1201	460.7147	6.932286	431.0835	38.75156	466.9	3.1	108.3	213
19AR107 228	322.0297	15360.41	7.24953	17.3161	1.694448	0.586245	1.914384	0.075138	0.719246	0.375706	467.0375	3.24035	468.4669	7.184147	476.4592	39.22937	467.0	3.2	98.0	197
19AR107 446	293.7442	12257.94	17.96421	17.27776	1.615776	0.588049	2.179674	0.075149	0.683137	0.313412	467.1048	3.078099	469.621	8.195596	482.9197	45.74045	467.1	3.1	96.7	392
19AR107 195	458.167	11522.22	34.73077	17.41145	1.432445	0.582835	1.854808	0.075221	0.756867	0.408057	467.5365	3.413355	466.2814	6.934981	461.0493	37.54202	467.5	3.4	101.4	165
19AR107 285	402.5097	17892.69	11.00431	17.63413	1.327653	0.578467	2.009265	0.075244	0.688181	0.342504	467.672	3.104458	463.4758	7.476839	443.6836	41.99959	467.7	3.1	105.4	247
19AR107 113	121.2564	3999.43	1.782034	16.18761	3.078259	0.604052	5.860067	0.075266	0.802435	0.136933	467.8032	3.620848	479.8022	22.41089	538.5248	127.0881	467.8	3.6	86.9	94
19AR107 216	422.8386	11809.61	186.8217	17.57001	1.12394	0.577307	1.546155	0.075365	0.610452	0.394819	468.3997	2.757946	462.7294	5.746169	435.6302	31.63893	468.4	2.8	107.5	186
19AR107 346	229.0055	10108.22	17.54298	17.73708	2.392211	0.571286	3.320567	0.075446	1.003996	0.302357	468.8857	4.540465	458.846	12.25919	409.833	70.7907	468.9	4.5	114.4	302
19AR107 97	287.1838	14153.19	1.727151	17.28329	2.525949	0.592967	2.790214	0.075495	0.827132	0.29644	469.1738	3.742833	472.7604	10.54646	491.1454	58.79973	469.2	3.7	95.5	80
19AR107 151	392.513	18255.13	12.96875	17.68595	1.280073	0.58112	1.694376	0.075542	0.599942	0.354079	469.4585	2.716371	465.1809	6.323337	445.0672	35.22855	469.5	2.7	105.5	124
19AR107 536	318.9883	12163.69	2.693621	17.13924	1.850162	0.593481	2.928451	0.075585	0.895757	0.305881	469.7177	4.057897	473.0884	11.07504	490.4277	61.51759	469.7	4.1	95.8	469
19AR107 591	389.5762	18248.88	1.5548	17.94655	1.515558	0.572582	1.740244	0.075724	0.749108	0.430461	470.549	3.399344	459.6831	6.43384	406.6867	35.15496	470.5	3.4	115.7	518
19AR107 43	100.64	5125.553	1.462326	16.43262	2.944104	0.604381	3.71897	0.075742	0.878976	0.236349	470.6568	3.989551	480.0103	14.22605	525.8759	79.24698	470.7	4.0	89.5	37
19AR107 404	158.0278	6286.628	1.643767	17.42369	3.220086	0.575243	3.818551	0.075778	0.777211	0.203536	470.8698	3.529192	461.4001	14.15992	415.4758	83.57271	470.9	3.5	113.3	353
19AR107 153	495.7253	18975.09	3.078774	17.64183	1.149495	0.584348	1.609722	0.075788	0.66732	0.414556	470.9336	3.030588	467.252	6.02847	450.149	32.55939	470.9	3.0	104.6	126
19AR107 391	122.2783	3283.324	2.045851	17.26488	4.203824	0.556584	5.256627	0.075826	0.990138	0.18836	471.1623	4.498749	449.3008	19.08739	339.8978	116.9714	471.2	4.5	138.6	342
19AR107 441	389.1212	18291.46	10.00054	17.67387	0.883108	0.584327	1.371567	0.075872	0.651034	0.474664	471.4355	2.959663	467.2382	5.136434	447.613	26.83395	471.4	3.0	105.3	387
19AR107 376	381.8788	17328.73	17.74483	17.25406	1.112861	0.597271	1.515185	0.075979	0.629913	0.415734	472.0767	2.867403	475.5004	5.752981	492.9891	30.36192	472.1	2.9	95.8	331
19AR107 33	362.8439	16507.01	3.843482	17.65033	1.528001	0.583225	2.166134	0.075984	0.728646	0.336381	472.1073	3.317048	466.5322	8.102484	440.1341	45.39213	472.1	3.3	107.3	27
19AR107 41	172.8359	4980.699	0.895638	17.15882	2.350879	0.578797	5.819653	0.076031	0.759376	0.130485	472.3895	3.458933	463.6879	21.6667	421.7658	128.8668	472.4	3.5	112.0	35
19AR107 290	218.7951	8119.001	5.143724	17.2855	2.320536	0.586083	3.220016	0.076031	0.75628	0.234868	472.3904	3.444834	468.363	12.08207	449.6174	69.54154	472.4	3.4	105.1	252
19AR107 580	57.71449	2656.42	2.351116	16.02662	4.019548	0.59624	10.31424	0.076107	1.124991	0.109072	472.8441	5.129049	474.8448	39.13859	485.4618	226.8489	472.8	5.1	97.4	507
19AR107 223	131.8945	6123.987	3.010421	16.66339	2.482074	0.60327	2.940604	0.076161	0.757607	0.257637	473.1657	3.456338	479.3067	11.23541	509.7313	62.48118	473.2	3.5	92.8	192
19AR107 451	163.5924	7275.153	6.919605	16.6646	4.229451	0.607902	4.427378	0.076239	0.675773	0.152635	473.6339	3.085936	482.2361	16.99773	524.2729	95.99523	473.6	3.1	90.3	397
19AR107 374	578.3022	31488.69	102.6119	17.68494	1.086151	0.588574	1.498172	0.076255	0.721525	0.481603	473.7301	3.295508	469.9567	5.636243	452.5145	29.17242	473.7	3.3	104.7	329
19AR107 149	273.2243	10807.32	1.496192	17.54211	2.753166	0.585426	3.409832	0.076288	0.828564	0.242993	473.9303	3.785943	467.9428	12.78534	439.6219	73.62976	473.9	3.8	107.8	122
19AR107 259	564.712	36041.19	70.1352	17.6466	0.95857	0.593181	1.321251	0.076296	0.630581	0.477261	473.9744	2.881561	472.8971	4.995061	468.6413	25.72027	474.0	2.9	101.1	224
19AR107 473	104.4463	3626.495	2.118411	16.70699	2.575884	0.586336	3.631545	0.07631	0.79951	0.220157	474.0572	3.654129	468.5249	13.63009	442.456	78.80916	474.1	3.7	107.1	416
19AR107 332	116.9305	5431.257	0.993479	17.08714	3.678214	0.587928	4.099957	0.076361	0.790269	0.19275	474.364	3.614145	469.5433	15.41472	446.9927	89.43749	474.4	3.6	106.1	289
19AR107 307	158.5117	7247.494	4.196828	17.16968	2.326092	0.592462	3.358114	0.076375	0.900347	0.268111	474.4479	4.118269	472.4386	12.68642	463.676	71.70531	474.4	4.1	102.3	268
19AR107 342	58.96909	2990.15	2.096717	15.21033	4.588499	0.641268	8.472896	0.076389	1.166201	0.137639	474.5317	5.335217	503.0907	33.6264	636.1213	180.9069	474.5	5.3	74.6	298
19AR107 198	116.9864	6111.591	2.16454	16.65294	3.80092	0.607852	5.522924	0.076407	0.850554	0.154004	474.6397	3.892027	482.2048	21.20382	519.2684	119.8747	474.6	3.9	91.4	168

19AR107 196	436.9953	18167.68	8.482559	17.47503	0.924768	0.594979	1.481022	0.076444	0.76133	0.514057	474.8621	3.485323	474.0425	5.609737	471.0618	28.11582	474.9	3.5	100.8	166
19AR107 334	352.7675	3600.165	6.36785	17.60143	1.943447	0.557269	7.334885	0.076543	0.812981	0.110838	475.4567	3.72627	449.7473	26.65779	321.3421	165.8109	475.5	3.7	148.0	290
19AR107 233	107.0936	3467.535	0.928195	16.36896	3.935448	0.598215	5.646913	0.076587	0.73588	0.130315	475.7169	3.374655	476.1005	21.46485	478.8901	123.8248	475.7	3.4	99.3	201
19AR107 375	394.1532	11233.61	7.505971	17.44719	1.382062	0.590485	2.467751	0.076589	0.717815	0.290878	475.7282	3.291889	471.1772	9.30299	450.0169	52.45941	475.7	3.3	105.7	330
19AR107 255	167.3196	8131.355	3.530929	17.95213	3.075641	0.567672	3.931874	0.076595	0.758522	0.192916	475.7657	3.478836	456.5079	14.45776	361.6441	87.05651	475.8	3.5	131.6	220
19AR107 53	330.924	20974.6	9.104091	17.47536	1.13964	0.595268	1.459556	0.076669	0.817689	0.560231	476.2089	3.753559	474.226	5.530109	465.6093	26.80061	476.2	3.8	102.3	41
19AR107 222	149.4243	7208.267	1.829482	17.62395	2.652281	0.57702	3.448462	0.076708	0.861706	0.249881	476.4408	3.957475	462.5446	12.81245	395.0705	74.89821	476.4	4.0	120.6	191
19AR107 398	411.8754	17656.95	2.006151	17.39161	1.378112	0.597514	1.7852	0.076804	0.869251	0.486921	477.0168	3.996776	475.6551	6.779954	470.0366	34.5228	477.0	4.0	101.5	347
19AR107 85	196.4934	6864.451	5.048073	17.25159	2.119548	0.590394	3.158818	0.07683	0.790399	0.25022	477.1729	3.635361	471.119	11.90725	442.6819	68.05178	477.2	3.6	107.8	68
19AR107 488	19.12968	718.8842	2.088228	10.57738	26.56391	0.791655	28.82471	0.076893	2.519722	0.087415	477.5536	11.59814	592.1105	130.0294	1060.523	589.7384	477.6	11.6	45.0	428
19AR107 200	365.6826	15365.67	5.607913	17.76044	1.774423	0.586141	2.022055	0.076922	0.626616	0.309891	477.7251	2.885271	468.4002	7.587369	423.906	42.92056	477.7	2.9	112.7	170
19AR107 403	152.0233	7122.458	1.349911	17.39227	2.445013	0.588577	3.541858	0.077007	0.738988	0.208644	478.2341	3.406183	469.9584	13.32542	430.6894	77.20777	478.2	3.4	111.0	352
19AR107 519	510.0584	15020.76	5.898438	17.20169	1.008974	0.605542	1.956961	0.077092	0.773569	0.395291	478.7392	3.569206	480.7445	7.494498	491.2593	39.66765	478.7	3.6	97.5	455
19AR107 103	97.12793	5275.014	1.344904	17.62162	4.007598	0.57421	4.582557	0.077101	1.163416	0.253879	478.7978	5.368571	460.7335	16.97408	372.5967	99.83525	478.8	5.4	128.5	84
19AR107 325	206.4718	9944.003	1.776418	17.58586	2.717423	0.588628	2.996872	0.077113	0.829823	0.276896	478.8698	3.829765	469.9911	11.27547	427.8114	64.22858	478.9	3.8	111.9	284
19AR107 582	100.5465	4822.701	1.662877	16.62817	4.378155	0.606342	4.697549	0.077122	0.804557	0.171272	478.9236	3.71356	481.2502	18.00637	493.3058	102.1027	478.9	3.7	97.1	509
19AR107 526	202.0361	6904.783	4.924653	16.54153	1.546268	0.618545	2.369257	0.077147	0.81067	0.342162	479.0714	3.742887	488.9348	9.193913	536.3829	48.74727	479.1	3.7	89.3	460
19AR107 323	181.3267	5996.057	1.635845	17.55253	2.327198	0.581261	4.645593	0.077325	0.816243	0.175703	480.1338	3.776668	465.2716	17.34127	393.5275	102.6297	480.1	3.8	122.0	282
19AR107 267	369.5289	16394.84	3.268759	17.40478	2.083598	0.605673	2.678035	0.077329	0.861798	0.321802	480.1593	3.987649	480.8272	10.25751	484.9965	56.00858	480.2	4.0	99.0	231
19AR107 138	285.7299	11117.15	3.656551	17.2157	1.56529	0.605136	2.248755	0.077331	0.786964	0.349955	480.1733	3.641486	480.4881	8.608426	482.9724	46.55015	480.2	3.6	99.4	116
19AR107 305	351.9803	15632.63	195.7108	17.62956	1.699244	0.59564	1.863823	0.077413	0.708292	0.380021	480.6623	3.280665	474.4633	7.064653	445.5458	38.34595	480.7	3.3	107.9	266
19AR107 312	146.9923	4424.587	1.240692	16.87917	3.058517	0.597391	4.35346	0.077422	1.100049	0.252684	480.7162	5.095756	475.5769	16.53295	451.8082	93.56789	480.7	5.1	106.4	272
19AR107 19	152.5675	5604.298	2.210655	17.63197	2.767365	0.57805	4.062617	0.07749	0.930782	0.229109	481.1245	4.315187	463.2072	15.11166	376.2748	89.01675	481.1	4.3	127.9	15
19AR107 480	173.644	10640.55	9.25807	17.25061	1.697122	0.603948	2.864998	0.077515	0.827484	0.288825	481.2755	3.83745	479.7363	10.95419	473.3498	60.70725	481.3	3.8	101.7	420
19AR107 197	269.6553	15176.44	1.461037	17.17836	2.167495	0.613088	2.476335	0.077627	0.67763	0.273642	481.9413	3.146686	485.5057	9.556898	503.3382	52.44784	481.9	3.1	95.7	167
19AR107 365	306.4783	9710.786	7.855512	17.33094	2.104501	0.599681	3.37419	0.077635	0.817881	0.242393	481.9926	3.798356	477.031	12.84427	454.2333	72.69654	482.0	3.8	106.1	321
19AR107 525	337.0265	17107.09	11.26601	17.43183	2.03434	0.603292	2.301401	0.077689	0.824352	0.358196	482.3134	3.830864	479.3206	8.793218	465.9979	47.61045	482.3	3.8	103.5	459
19AR107 456	171.2912	7669.409	5.555259	17.02024	2.812271	0.609392	3.026886	0.077851	0.667852	0.22064	483.2841	3.109604	483.1767	11.63804	483.6502	65.21599	483.3	3.1	99.9	402
19AR107 265	227.9407	9975.486	5.030795	17.3313	1.819005	0.606491	2.393547	0.077885	0.96591	0.403548	483.4854	4.499206	481.3446	9.175501	472.1223	48.46301	483.5	4.5	102.4	229
19AR107 506	144.6567	6377.47	2.736913	18.02056	1.773153	0.570199	3.954262	0.07793	0.684683	0.173151	483.7558	3.190968	458.1434	14.58133	332.6054	88.32264	483.8	3.2	145.4	445
19AR107 512	80.02531	2743.757	1.699236	16.18848	5.133905	0.606713	6.596576	0.078057	1.020745	0.154739	484.5163	4.764388	481.485	25.29783	468.0439	144.4418	484.5	4.8	103.5	450
19AR107 503	288.8094	1736.397	3.021761	16.72507	1.475146	0.55221	2.651751	0.078179	1.313687	0.495404	485.2415	6.140547	446.4432	9.579199	252.1674	53.00413	485.2	6.1	192.4	442
19AR107 554	45.22021	2874.203	2.214685	15.46847	7.258559	0.641742	9.087467	0.078217	1.173597	0.129145	485.4693	5.488208	503.3844	36.08372	586.621	195.9029	485.5	5.5	82.8	485
19AR107 518	239.7698	8292.328	3.760699	17.42611	1.836975	0.5992	2.201139	0.078354	0.752335	0.341793	486.2909	3.523949	476.7259	8.374451	431.9133	46.09473	486.3	3.5	112.6	454
19AR107 532	74.06755	5465.56	0.924942	15.55043	5.551641	0.664953	6.049597	0.078472	1.089578	0.180108	486.9951	5.110717	517.6394	24.53748	656.2245	127.7186	487.0	5.1	74.2	465
19AR107 490	108.0683	5084.606	1.519005	17.46738	3.796058	0.588593	4.444628	0.078503	0.833891	0.187618	487.1823	3.912853	469.9689	16.72273	387.7103	98.07341	487.2	3.9	125.7	430
19AR107 481	309.6532	16413.12	12.19928	17.40527	1.432988	0.613666	1.684902	0.078811	0.618275	0.36695	489.0192	2.911649	485.8698	6.506227	472.0241	34.70456	489.0	2.9	103.6	421
19AR107 509	267.9694	13590.93	8.50601	17.52777	2.033751	0.609495	2.21281	0.07918	0.673615	0.304416	491.2272	3.186046	483.2415	8.508727	446.4833	46.8641	491.2	3.2	110.0	447
19AR107 485	124.0849	7628.996	1.86855	17.42141	3.295159	0.606499	4.671024	0.079273	0.660112	0.141321	491.7799	3.125562	481.3496	17.90757	432.9199	103.0581	491.8	3.1	113.6	425
19AR107 493	526.3945	22181.79	1.382751	17.6978	1.03016	0.61137	1.66974	0.079399	0.804064	0.48155	492.5368	3.812799	484.4237	6.432702	447.1646	32.52257	492.5	3.8	110.1	433
19AR107 177	437.216	25557.21	8.597172	17.255	0.923996	0.63063	1.394422	0.079994	0.687099	0.492748	496.0892	3.280769	496.4883	5.475798	499.2663	26.74177	496.1	3.3	99.4	149
19AR107 572	538.1425	9860.261	71.82556	17.33626	0.922351	0.621638	1.658838	0.080344	0.779773	0.470072	498.1771	3.738343	490.8732	6.456885	457.9098	32.487	498.2	3.7	108.8	500
19AR107 499	179.8885	6422.399	11.15803	17.31016	2.733884	0.617427	4.432919	0.080734	1.148294	0.259038	500.5016	5.529786	488.2335	17.18392	432.0153	95.4586	500.5	5.5	115.9	438
19AR107 61	263.0113	17728.23	1.610394	16.91634	1.617192	0.648297	2.247177	0.080934	0.748572	0.333117	501.6923	3.613109	507.4302	8.974639	534.3069	46.38059	501.7	3.6	93.9	48
19AR107 487	234.2404	9561.994	7.19063	17.49613	1.773531	0.621879	3.059168	0.081077	0.797469	0.260682	502.5466	3.855419	491.0246	11.9108	438.5761	65.75964	502.5	3.9	114.6	427
19AR107 547	150.1162	1217.57	0.547297	16.31849	2.356376	0.549604	5.010759	0.081198	0.754505	0.150577	503.268	3.652746	444.7371	18.04713	153.2065	116.0893	503.3	3.7	328.5	478
19AR107 221	126.5421	4916.813	2.132282	16.54287	3.271894	0.642334	3.883483	0.081268	0.859902	0.221425	503.684	4.166302	503.7501	15.42354	504.9958	83.35182	503.7	4.2	99.7	190
19AR107 230	145.3839	6772.676	2.543624	16.50671	2.863665	0.655096	3.881862	0.08162	0.75232	0.193804	505.7856	3.65968	511.6097	15.6022	538.6519	83.3211	505.8	3.7	93.9	199
19AR107 88	359.9948	2847.946	1.404026	16.60449	1.1935	0.619371	2.138146	0.081652	0.861143	0.402752	505.9733	4.190549	489.4528	8.303883	413.8026	43.75839	506.0	4.2	122.3	71
19AR107 440	360.3631	17207.92	3.857264	17.361	1.343609	0.640233	1.926612	0.081709	0.727178	0.377439	506.3176	3.540952	502.4506	7.635985	485.8184	39.38673	506.3	3.5	104.2	386
19AR107 213	167.7615	5821.09	2.436825	17.15342	3.764216	0.627605	4.189126	0.081764	0.712977	0.170197	506.6405	3.47393	494.6029	16.4032	440.2	91.87855	506.6	3.5	115.1	183
19AR107 351	172.9447	4736.63	1.407445	16.90511	2.360498	0.631141	2.945191	0.081781	0.832578	0.282691	506.743	4.057466	496.8063	11.57169	452.2194	62.73589	506.7	4.1	112.1	307
19AR107 434	462.1485	25990.03	19.3336	17.1864	1.242324	0.649362	1.523612	0.081803	0.724573	0.475562	506.8756	3.532005	508.0862	6.090888	514.4738	29.44743	506.9	3.5	98.5	380
19AR107 349	156.9221	5840.909	2.527545	16.89632	1.970713	0.639933	4.321791	0.081914	1.085414	0.251149	507.5356	5.297583	502.2648	17.1255	479.2648	92.48495	507.5	5.3	105.9	305
19AR107 311	339.6716	13236.66	4.555193	17.22242	1.230561	0.643833	1.834577	0.081952	0.767427	0.418313	507.762	3.747188	504.6766	7.296068	491.6973	36.7695	507.8	3.7	103.3	271
19AR107 22	229.2096	8567.504	1.395841	17.17933	1.353181	0.639373	1.878236	0.082071	0.676431	0.360142	508.471	3.307305	501.918	7.438139	473.1125	38.77675	508.5	3.3	107.5	18
19AR107 345	295.7829	8628.618	6.264727	16.68574	1.647854	0.658976	2.677023	0.082078	1.016908	0.379865	508.5171	4.97245	513.9877	10.79762	539.3194	54.17363	508.5	5.0	94.3	301
19AR107 299	387.981	16112	3.739605	17.02746	1.149334	0.654806	1.664617	0.08234	0.691775	0.415576	510.0775	3.392597	511.4317	6.688301	518.4234	33.23241	510.1	3.4	98.4	260
19AR107 171	132.675	6832.662	1.087488	16.46313	3.559968	0.663863	4.673875	0.082439	0.972425	0.208055	510.6631	4.774223	516.974	18.9373	545.8961	99.9539	510.7	4.8	93.5	143
19AR107 302	172.8879	7001.102	1.871082	17.5237	2.486407	0.625336	2.981102	0.082446	0.805	0.270034	510.7036	3.95253	493.1866	11.64652	413.599	64.16896	510.7	4.0	123.5	263
19AR107 162	109.1989	4034.346	2.327348	17.33403	3.551195	0.612751	7.303799	0.082447	0.761664	0.104283	510.7132	3.739822	485.2937	28.18428	367.9285	163.8686	510.7	3.7	138.8	134
19AR107 437	287.9576	9439.969	4.015757	17.13801	1.592193	0.646235	1.844822	0.08258	0.708753	0.384185	511.5035	3.485196	506.159	7.353438	483.0626	37.61921	511.5	3.5	105.9	383
19AR107 298	186.6566	2393.735	2.900531	16.95044	2.158589	0.600959	10.75565	0.082629	0.795875	0.073996	511.7968	3.915767	477.8421	41.01733	318.9615	244.4179	511.8	3.9	160.5	259
19AR107 314	204.6641	3854.202	0.960691	17.09315	2.092975	0.62411	6.968999	0.082666	0.789482	0.113285	512.019	3.88593	492.4201	27.1988	403.2195	155.2276	512.0	3.9	127.0	274
19AR107 330	159.1147	5301.489	1.299916	17.40915	2.267995	0.623455	3.5624	0.08268	0.752426	0.211213	512.0999	3.704102	492.0108	13.892	400.5023	78.03208	512.1	3.7	127.9	287
19AR107 172	372.6702	15719.37	21.86691	16.81237	1.074981	0.665231	1.536656	0.082695	0.85587	0.55697	512.1914	4.214067	517.8086	6.233184	543.629	27.91804	512.2	4.2	94.2	144
19AR107 129	186.3333	5936.256	3.803617	16.45161	2.019878	0.665979	2.696418	0.082771	0.906065	0.336025	512.6419	4.46498	518.2644	10.94523	544.0611	55.51189	512.6	4.5	94.2	108
19AR107 407	358.5755	14098.05	190.7633	17.18834	1.586552	0.653442	2.334571	0.082808	0.751106	0.321732	512.8608	3.70288	510.5944	9.36844	501.3955	48.69076	512.9	3.7	102.3	356
19AR107 392	342.0338	12421.11	1.136338	17.30221	1.387796	0.644018	1.732046	0.082854	0.898499	0.51875	513.1378	4.431812	504.7906	6.889494	468.0873	32.8105	513.1	4.4	109.6	343
19AR107 38	274.9861	12141.88	2.250083	16.73295	2.055772	0.667854	2.300025	0.082865	0.756029	0.328704	513.2018	3.729528	519.4071	9.351871	547.7225	47.45156	513.2	3.7	93.7	32
19AR107 188	228.1793	12837.42	2.947402	16.47527	2.921921	0.681228	3.320123	0.082929	0.673712	0.202918	513.5843	3.325838	527.5163	13.6608	589.1898	70.54892	513.6	3.3	87.2	159
19AR107 25	130.1963	7628.629	1.765339	16.92134	3.067631	0.654792	3.345577	0.083	0.784906	0.23461	514.0019	3.87778	511.4231	13.44269	500.8922	71.64413	514.0	3.9	102.6	21
19AR107 394	187.9848	7383.229	2.852326	17.60588	2.076457	0.624495	3.313109	0.083026	0.926026	0.279504	514.1602	4.57633	492.6606	12.93301	394.8715	71.3551	514.2	4.6	130.2	344
19AR107 563	226.8026	12497.91	2.517037	17.05266	1.745765	0.656491	1.977005	0.083062	0.634486	0.320933	514.3718	3.136807	512.4655	7.955846	504.9437	41.20067	514.4	3.1	101.9	493
19AR107 167	325.8765	12459.91	7.219021	17.48069	2.04624	0.639818	2.375976	0.083072	0.67897	0.285765	514.4314	3.357102	502.1936	9.413373	447.7609	50.59957	514.4	3.4	114.9	139
19AR107 567	120.1229	5228.971	1.291776	16.1265	4.069536	0.676816	4.753265	0.083092	0.70263	0.14782	514.5516	3.474865	524.8481	19.48321	570.8216	102.3531	514.6	3.5	90.1	496
19AR107 353	138.412	5565.838	4.365606	16.5163	2.246662	0.661617	3.775827	0.083115	0.821928	0.217681	514.6915	4.065918	515.6026	15.26689	520.5766	80.89388	514.7	4.1	98.9	309
19AR107 359	107.5193	3727.341	1.638742	15.79979	3.67376	0.678503	6.01269	0.083133	0.879858	0.146334	514.7967	4.353345	525.8693	24.68391	575.1932	129.4204	514.8	4.4	89.5	315
19AR107 206	497.868	22623.14	2.29574	17.25615	0.737627	0.655953	1.092901	0.083178	0.668831	0.611977	515.0668	3.310893	512.1356	4.395802	500.0526	19.03373	515.1	3.3	103.0	176
19AR107 448	330.0393	11837.28	4.192126	17.10456	1.470684	0.657865	1.749682	0.083271	0.683401	0.390586	515.6173	3.386494	513.3073	7.049911	503.9711	35.42899	515.6	3.4	102.3	394
19AR107 229	446.1131	17912.3	10.81319	17.21595	0.987398	0.655066	1.803613	0.083276	0.661699	0.366874	515.6454	3.279124	511.5914	7.248537	494.4688	36.99195	515.6	3.3	104.3	198
19AR107 183	437.5153	21567.5	2.505857	17.22181	0.915376	0.657423	1.349914	0.083278	0.661595	0.490102	515.6621	3.278713	513.0362	5.4369	502.2962	25.91557	515.7	3.3	102.7	155
19AR107 122	371.9869	20663.23	6.845252	14.75698	1.414334	0.768746	1.71219	0.083393	0.63463	0.370654	516.345	3.149077	579.0436	7.556269	834.241	33.14604	516.3	3.1	61.9	102
19AR107 336	435.1751	17772.31	2.605467	17.17217	1.200511	0.661206	1.733956	0.083445	0.823876	0.475143	516.6508	4.090458	515.3513	7.007901	510.5314	33.55299	516.7	4.1	101.2	292
19AR107 205	130.6963	6171.717	0.711203	17.35479	2.906471	0.634566	5.125065	0.083448	0.863503	0.168486	516.6688	4.287345	498.9359	20.20509	419.3394	112.8854	516.7	4.3	123.2	175
19AR107 101	873.9455	55480.68	0.838117	17.16745	0.740264	0.669026	1.019617	0.08348	0.618558	0.606657	516.86	3.072269	520.1203	4.150012	535.4159	17.75371	516.9	3.1	96.5	83
19AR107 282	235.6373	2619.263	19.63865	16.43474	1.725131	0.637675	3.236388	0.083484	0.888992	0.274686	516.8879	4.415696	500.8657	12.79632	429.2609	69.38448	516.9	4.4	120.4	245
19AR107 161	110.4863	6496.768	1.696122	17.77352	3.274228	0.621833	3.784746	0.083512	0.937975	0.24783	517.0508	4.660409	490.9955	14.73549	372.1647	82.58242	517.1	4.7	138.9	133
19AR107 154	163.4122	10152.97	3.405094	17.47021	2.368563	0.643171	2.969819	0.083581	0.640199	0.215568	517.4638	3.183324	504.2677	11.80384	445.7857	64.48375	517.5	3.2	116.1	127
19AR107 364	154.8665	5118.251	2.553494	17.03441	2.461733	0.641712	3.961129	0.083584	0.763595	0.192772	517.4787	3.797001	503.3657	15.72269	440.6678	86.51611	517.5	3.8	117.4	320
19AR107 599	298.8005	19662.61	3.838145	17.20214	1.208523	0.659697	1.549589	0.083587	0.695349	0.448731	517.4956	3.457754	514.4289	6.254144	501.7814	30.49472	517.5	3.5	103.1	525
19AR107 413	373.8348	21480.17	4.069463	17.42724	1.199175	0.654929	1.65797	0.083607	0.676816	0.40822	517.6173	3.366356	511.5074	6.662353	485.2495	33.41561	517.6	3.4	106.7	362
19AR107 355	247.0133	14697.51	4.822636	16.77274	1.760597	0.673695	1.956916	0.083628	0.726991	0.371498	517.7421	3.616755	522.9563	7.998303	546.7203	39.70326	517.7	3.6	94.7	311
19AR107 426	313.1474	11839.1	3.160649	17.21916	1.553966	0.656028	2.359325	0.083669	0.739395	0.313392	517.9839	3.680113	512.1816	9.490413	487.3496	49.47117	518.0	3.7	106.3	374
19AR107 10	429.007	21230.28	2.075499	17.04102	0.989454	0.670047	1.564008	0.083725	0.8142	0.520585	518.3192	4.054953	520.7409	6.371639	532.3517	29.23964	518.3	4.1	97.4	6
19AR107 595	176.5378	7035.577	4.621007	16.88244	2.265928	0.658435	2.989904	0.083757	0.605664	0.20257	518.5075	3.017436	513.6566	12.05375	493.1003	64.57122	518.5	3.0	105.2	521
19AR107 150	154.5308	10868.76	1.661747	17.33088	2.048204	0.651604	2.476689	0.083776	0.850101	0.343241	518.6237	4.236139	509.4652	9.921858	469.5334	51.50177	518.6	4.2	110.5	123
19AR107 315	248.4917	11075.65	10.6174	16.70866	1.279293	0.676734	2.026531	0.08379	0.961772	0.474591	518.7043	4.793325	524.7984	8.305134	552.3724	38.92545	518.7	4.8	93.9	275
19AR107 390	302.3721	11034.76	5.035052	17.06243	1.613949	0.658953	2.679417	0.08379	0.969528	0.361843	518.7057	4.831992	513.9735	10.80705	493.9413	55.07621	518.7	4.8	105.0	341
19AR107 452	244.3174	8790.005	3.101016	17.0047	2.025177	0.658874	2.184225	0.083839	0.557074	0.255044	518.9994	2.777888	513.9251	8.809012	492.3599	46.57327	519.0	2.8	105.4	398
19AR107 410	165.355	6929.99	2.671646	17.69865	1.988171	0.62902	3.004923	0.083841	0.77551	0.25808	519.0097	3.867205	495.4854	11.78205	389.1552	65.17288	519.0	3.9	133.4	359
19AR107 257	174.6161	12352.13	1.556758	17.23716	2.119325	0.658031	2.937	0.083918	0.690907	0.235242	519.4647	3.448222	513.409	11.83606	487.4842	63.01357	519.5	3.4	106.6	222
19AR107 203	126.9056	7468.932	1.056759	17.42708	3.060959	0.640254	4.231097	0.083935	1.073226	0.253652	519.5702	5.357364	502.4632	16.77117	426.2488	91.31557	519.6	5.4	121.9	173
19AR107 400	202.1439	12727.49	1.734282	17.60502	1.535587	0.643305	2.068507	0.084002	0.896585	0.433445	519.9644	4.478862	504.3503	8.222332	435.0851	41.53051	520.0	4.5	119.5	349
19AR107 245	245.9744	10784.33	1.985283	17.0491	1.646748	0.661902	2.080597	0.084037	0.752887	0.361861	520.1737	3.762478	515.7765	8.414258	497.3124	42.74559	520.2	3.8	104.6	212
19AR107 381	183.8514	14663.97	2.651803	17.31641	2.050535	0.656868	2.645191	0.084092	0.667495	0.252343	520.5028	3.337765	512.6961	10.64861	478.9796	56.5777	520.5	3.3	108.7	335
19AR107 405	147.6475	6590.013	2.925565	17.53538	3.191864	0.635536	3.549721	0.084115	0.687106	0.193566	520.641	3.436708	499.5385	14.00657	404.9343	77.99071	520.6	3.4	128.6	354
19AR107 6	214.3142	10444.03	2.011761	17.50056	1.574966	0.647586	2.020577	0.084143	0.807522	0.399649	520.8049	4.040214	506.992	8.064244	446.1011	41.17093	520.8	4.0	116.7	5
19AR107 422	132.0057	4329.674	1.808822	16.95186	2.093179	0.64583	3.981841	0.084173	0.730301	0.183408	520.9849	3.655072	505.9095	15.86655	439.2626	87.13401	521.0	3.7	118.6	371
19AR107 573	373.6199	12731.28	3.105683	17.13304	1.167062	0.663396	1.407499	0.084238	0.5979	0.424796	521.3665	2.994527	516.6888	5.6998	497.0085	28.06652	521.4	3.0	104.9	501
19AR107 111	186.5929	10173.23	1.548249	17.1717	2.098464	0.660346	2.515522	0.084255	0.802467	0.319006	521.4702	4.019845	514.8256	10.15887	486.3754	52.62969	521.5	4.0	107.2	92
19AR107 408	148.7764	7461.7	3.501859	16.9625	3.858114	0.663088	4.223288	0.084338	0.789997	0.187057	521.9641	3.960978	516.5008	17.09926	493.3724	91.51988	522.0	4.0	105.8	357
19AR107 414	207.2827	8351.854	3.24474	17.25721	2.125832	0.65511	3.452861	0.084374	0.73857	0.213901	522.1775	3.704579	511.6184	13.87789	465.6723	74.74791	522.2	3.7	112.1	363
19AR107 368	229.1251	9620.014	1.613862	17.16974	1.652507	0.658134	2.763395	0.084394	0.658951	0.238457	522.2992	3.305962	513.4721	11.13743	475.3253	59.37252	522.3	3.3	109.9	324
19AR107 575	148.1526	5449.363	1.730198	17.39182	2.636537	0.636943	3.078998	0.084398	0.648917	0.210756	522.3185	3.255733	500.412	12.16542	402.3888	67.42388	522.3	3.3	129.8	503
19AR107 87	157.1933	11304.06	1.452812	17.20574	2.430271	0.660058	2.866309	0.0844	0.895307	0.312355	522.3345	4.492049	514.6497	11.57259	481.6067	60.17416	522.3	4.5	108.5	70
19AR107 570	586.488	35673.5	4.211279	17.19576	0.72687	0.670982	0.93255	0.084409	0.541618	0.580793	522.3867	2.717739	521.3092	3.802272	517.5265	16.66049	522.4	2.7	100.9	499
19AR107 270	373.4618	21135.62	2.586358	17.09641	1.268728	0.675916	1.601381	0.084424	0.82337	0.514163	522.4737	4.132178	524.3031	6.557994	533.2339	30.07386	522.5	4.1	98.0	234
19AR107 449	347.424	13884.16	2.491113	17.45725	1.33731	0.652714	1.613535	0.084445	0.63848	0.395703	522.5978	3.205017	510.1476	6.470524	455.674	32.89499	522.6	3.2	114.7	395
19AR107 163	388.652	6282.208	9.283811	16.80153	1.268368	0.66421	2.031391	0.084479	0.673588	0.331589	522.8019	3.382516	517.1858	8.232469	493.4022	42.25378	522.8	3.4	106.0	135
19AR107 317	345.5689	15133.27	3.222465	17.07474	1.394781	0.671566	1.792721	0.084497	0.776487	0.433133	522.9075	3.899993	521.6642	7.313334	517.1588	35.47836	522.9	3.9	101.1	276
19AR107 453	162.7298	6848.236	3.447553	17.5819	2.288246	0.6375	3.937802	0.08458	0.779803	0.19803	523.4014	3.9202	500.7571	15.56742	399.5155	86.50554	523.4	3.9	131.0	399
19AR107 105	78.61771	3784.497	1.326824	16.30027	4.015509	0.67165	5.538397	0.084652	1.165564	0.210451	523.8313	5.864106	521.7149	22.59869	513.4163	119.0554	523.8	5.9	102.0	86
19AR107 225	253.8929	14044.74	1.24595	17.04934	1.602746	0.672319	1.999918	0.08491	0.625428	0.312727	525.3635	3.155445	522.1214	8.164089	508.915	41.77638	525.4	3.2	103.2	194
19AR107 523	184.4529	6462.319	1.445237	17.28485	1.790769	0.648658	2.808831	0.084935	0.857981	0.305458	525.5113	4.329903	507.6528	11.2217	428.9354	59.65542	525.5	4.3	122.5	458
19AR107 484	370.0442	15296.33	4.8615	17.37101	1.370288	0.663216	1.579855	0.084963	0.662919	0.419607	525.6812	3.346537	516.5795	6.396751	477.4597	31.71434	525.7	3.3	110.1	424
19AR107 511	98.15853	4107.898	1.567585	16.9661	4.298334	0.64773	5.574522	0.085037	0.796732	0.142924	526.1174	4.025254	507.0808	22.25436	423.0643	123.2145	526.1	4.0	124.4	449
19AR107 461	171.0036	8298.286	1.894235	17.12528	2.169853	0.663282	2.568737	0.085164	0.865333	0.336871	526.8737	4.377874	516.6198	10.40153	472.4621	53.54072	526.9	4.4	111.5	406
19AR107 300	658.2697	4930.251	1.670711	16.80328	0.877892	0.663852	1.520831	0.085256	0.766762	0.504173	527.417	3.883023	516.9674	6.161304	472.0247	29.08489	527.4	3.9	111.7	261
19AR107 507	222.2677	5692.465	5.333056	16.91159	2.022567	0.662956	3.135057	0.085272	0.661598	0.211032	527.5125	3.351032	516.4207	12.69118	468.5838	67.87334	527.5	3.4	112.6	446
19AR107 417	169.9158	3837.705	1.892976	16.78065	2.348298	0.657624	5.602851	0.085313	0.812063	0.144938	527.7573	4.114983	513.1595	22.57367	449.5986	123.2217	527.8	4.1	117.4	366
19AR107 14	161.8216	7842.225	1.895794	17.30631	2.784307	0.657784	3.588197	0.085318	0.695621	0.193864	527.7852	3.525112	513.2577	14.45742	450.0172	78.2298	527.8	3.5	117.3	10
19AR107 40	378.3955	17503.7	1.263077	16.89234	1.281431	0.685004	1.641777	0.085334	0.758502	0.462001	527.8833	3.844452	529.7945	6.777084	538.9668	31.85262	527.9	3.8	97.9	34
19AR107 166	147.9988	7601.67	1.47774	17.19178	2.441247	0.66091	2.716939	0.085481	0.952075	0.350422	528.7581	4.833247	515.1704	10.97799	456.3259	56.48061	528.8	4.8	115.9	138
19AR107 541	587.3631	30384.03	3.769195	17.26132	0.692912	0.676528	1.001923	0.08554	0.544073	0.543029	529.1033	2.763739	524.6737	4.105266	506.4144	18.507	529.1	2.8	104.5	473
19AR107 460	252.6975	11967.27	4.986555	17.45072	1.452184	0.660911	1.714448	0.085696	0.610683	0.356198	530.0305	3.107311	515.1713	6.927201	450.7312	35.60238	530.0	3.1	117.6	405
19AR107 558	311.0053	14316.01	1.499688	17.01638	1.155394	0.68226	1.686295	0.085802	0.804179	0.476891	530.6591	4.096526	528.1397	6.944281	518.201	32.55897	530.7	4.1	102.4	488
19AR107 483	112.2087	3879.807	1.482974	17.80311	2.88539	0.620041	4.78526	0.085943	0.771166	0.161154	531.4984	3.934315	489.8729	18.59852	300.6511	107.7267	531.5	3.9	176.8	423
19AR107 585	99.35547	5964.792	1.028426	15.77786	2.698455	0.72316	4.875067	0.086176	1.164448	0.238858	532.8817	5.955587	552.5309	20.77687	635.3213	101.9398	532.9	6.0	83.9	512
19AR107 539	243.8906	11934.85	5.435373	17.37312	1.686088	0.668557	2.010087	0.086203	0.599403	0.298198	533.039	3.066523	519.8349	8.178086	463.2097	42.53333	533.0	3.1	115.1	472
19AR107 190	101.305	4553.257	3.357936	15.95537	3.088757	0.707986	3.398485	0.086297	0.832844	0.245063	533.598	4.265085	543.5496	14.30489	586.4664	71.51656	533.6	4.3	91.0	161
19AR107 462	138.2865	7405.097	2.323669	16.99304	2.315117	0.674099	2.546174	0.086341	0.722532	0.283772	533.8608	3.701913	523.2018	10.4106	477.8865	53.99677	533.9	3.7	111.7	407
19AR107 467	269.0146	10631.67	7.489137	16.16785	1.631718	0.718427	2.107355	0.086568	0.65205	0.309416	535.2089	3.348888	549.7378	8.946029	611.326	43.28794	535.2	3.3	87.5	411
19AR107 504	244.1515	10729.72	4.22928	17.05944	1.557911	0.684539	1.879192	0.08692	0.631491	0.336044	537.2943	3.255417	529.5139	7.754015	497.0561	38.99954	537.3	3.3	108.1	443
19AR107 445	182.5403	8070.478	2.423209	16.84832	2.742009	0.690887	3.993775	0.086969	0.943377	0.236212	537.5862	4.865762	533.3331	16.57083	516.1153	85.25166	537.6	4.9	104.2	391
19AR107 273	387.3867	15179.14	5.530803	17.2358	2.127916	0.685917	2.511625	0.087005	0.789052	0.31416	537.8026	4.07135	530.3445	10.37613	499.3182	52.5423	537.8	4.1	107.7	237
19AR107 139	571.84	21904.56	14.56177	16.70818	1.09089	0.710173	1.366002	0.087085	0.716846	0.524777	538.2723	3.701882	544.8488	5.759835	573.4207	25.26923	538.3	3.7	93.9	117
19AR107 471	170.5961	11206.41	3.342747	17.10525	2.234409	0.686898	3.023909	0.087385	0.792458	0.262064	540.0508	4.105312	530.9348	12.50328	492.9122	64.36402	540.1	4.1	109.6	414
19AR107 215	268.5073	10420.43	8.636862	16.83398	1.443234	0.698307	1.776269	0.087502	0.752504	0.423643	540.7478	3.903156	537.7793	7.416118	526.1871	35.28246	540.7	3.9	102.8	185
19AR107 297	217.1525	19649.47	30.25499	16.37556	1.905045	0.726457	2.183866	0.08757	0.830083	0.380098	541.1511	4.308629	554.4715	9.330871	610.4749	43.6406	541.2	4.3	88.6	258
19AR107 256	331.5432	23931.68	21.77599	16.03811	1.638548	0.74354	2.128171	0.087588	1.096461	0.515213	541.2574	5.69236	564.4691	9.215539	660.0599	39.10048	541.3	5.7	82.0	221
19AR107 500	323.1699	17820.78	6.172529	16.83973	1.441795	0.709798	1.694671	0.088072	0.764463	0.451098	544.1258	3.988927	544.6261	7.143519	547.6493	33.05285	544.1	4.0	99.4	439
19AR107 108	112.746	5330.343	2.837405	17.87314	2.690081	0.647317	4.102747	0.088174	0.836072	0.203783	544.7268	4.367195	506.8261	16.37125	340.2469	90.98346	544.7	4.4	160.1	89
19AR107 58	126.243	5079.697	304.0585	16.56656	3.142618	0.697604	4.971533	0.08829	0.734583	0.147758	545.4132	3.841703	537.3591	20.74691	504.3011	108.2562	545.4	3.8	108.2	45

19AR107 543	245.0922	11734.28	1.645063	17.00931	1.835135	0.702031	2.238044	0.088612	0.585706	0.261705	547.3227	3.073388	540.0033	9.373456	510.1716	47.46764	547.3	3.1	107.3	475
19AR107 214	38.12822	2230.922	-301.643	15.00153	7.735495	0.736233	11.52504	0.088943	1.53657	0.133324	549.2822	8.090527	560.205	49.66219	605.7496	247.8138	549.3	8.1	90.7	184
19AR107 396	195.8497	9440.798	1.858522	16.94558	1.863099	0.702734	2.253706	0.089074	1.099956	0.488065	550.0555	5.799426	540.4227	9.444606	500.9685	43.2972	550.1	5.8	109.8	345
19AR107 474	321.708	13636.48	1.700994	16.65362	1.057477	0.728882	1.43028	0.089773	0.7255	0.507243	554.1929	3.852692	555.897	6.122774	563.8102	26.86134	554.2	3.9	98.3	417
19AR107 304	640.5628	12735.42	2.073723	16.704	0.941793	0.73287	1.428562	0.090487	0.781972	0.547384	558.4158	4.182872	558.2364	6.134727	558.456	26.0671	558.4	4.2	100.0	265
19AR107 496	168.4614	4128.35	1.406201	16.76894	2.97601	0.699768	3.800716	0.09049	0.804404	0.211645	558.4349	4.303004	538.6521	15.88894	456.7228	82.45823	558.4	4.3	122.3	435
19AR107 235	493.0593	23030.26	3.780221	16.89328	0.937181	0.728094	1.487343	0.090629	0.743822	0.500101	559.256	3.984533	555.434	6.363071	540.7294	28.1754	559.3	4.0	103.4	203
19AR107 399	310.3352	22863.64	180.3036	17.3799	1.44996	0.709625	1.952503	0.090851	0.856584	0.438711	560.5677	4.598879	544.5238	8.229231	478.8683	38.78121	560.6	4.6	117.1	348
19AR107 134	327.1235	15913.24	3.423191	16.59397	1.443042	0.745469	2.204991	0.091221	0.770214	0.349305	562.7514	4.150591	565.5922	9.562406	577.9892	44.89723	562.8	4.2	97.4	112
19AR107 431	102.2704	4890.69	0.870531	16.16516	3.284621	0.740319	3.930585	0.09124	0.807951	0.205555	562.8646	4.354785	562.5917	16.97922	562.4152	83.86021	562.9	4.4	100.1	378
19AR107 31	317.5603	4154.261	2.300244	16.45528	1.57111	0.722311	2.175246	0.091523	1.102421	0.506803	564.5374	5.958854	552.0302	9.263241	501.6966	41.30608	564.5	6.0	112.5	25
19AR107 52	154.3859	11959.3	5.849351	16.55104	2.448476	0.745465	3.171783	0.091842	0.805308	0.253898	566.4196	4.366769	565.5896	13.75549	563.1797	66.8596	566.4	4.4	100.6	40
19AR107 42	303.2543	14917.11	2.453333	16.64252	1.400027	0.750469	1.858867	0.09231	0.830748	0.446911	569.1821	4.525734	568.4963	8.092182	566.7217	36.18668	569.2	4.5	100.4	36
19AR107 288	281.2626	16311.89	0.69093	16.83206	2.11763	0.745643	2.272961	0.092569	0.726855	0.319783	570.7154	3.969947	565.6932	9.858503	546.481	47.05493	570.7	4.0	104.4	250
19AR107 438	394.4162	10510.85	6.284506	14.31896	0.884394	0.875876	1.532631	0.092616	0.771585	0.503439	570.9907	4.216201	638.7527	7.266299	887.3251	27.38657	571.0	4.2	64.3	384
19AR107 201	369.8165	15796.22	1.112668	16.9124	1.115244	0.74217	1.477935	0.092625	0.67796	0.458721	571.0446	3.704932	563.6711	6.392987	534.9759	28.74429	571.0	3.7	106.7	171
19AR107 179	136.3995	5442.642	2.545464	16.18279	2.665292	0.757001	4.716917	0.093128	0.813523	0.172469	574.0105	4.467841	572.2785	20.63823	566.3726	101.2161	574.0	4.5	101.3	151
19AR107 458	641.4343	29208.93	0.980134	16.8344	0.856012	0.760311	1.342722	0.093869	0.689352	0.513399	578.3773	3.813426	574.1897	5.888749	558.6185	25.1016	578.4	3.8	103.5	403
19AR107 155	99.91931	7209.111	1.04087	16.71416	3.688618	0.749037	4.73078	0.093956	0.912486	0.192883	578.8912	5.052071	567.6655	20.57436	523.8782	101.8523	578.9	5.1	110.5	128
19AR107 280	529.0425	4502.402	7.911369	14.996	0.774169	0.830223	1.736466	0.094658	1.189792	0.68518	583.0266	6.632362	613.7359	7.998269	729.6824	26.78422	583.0	6.6	79.9	243
19AR107 450	156.5203	7732.036	2.206334	16.74889	2.161154	0.759422	2.509906	0.095544	0.632093	0.251839	588.2407	3.553625	573.6767	11.00063	517.3209	53.33554	588.2	3.6	113.7	396
19AR107 186	196.3057	9042.13	4.048961	16.2706	2.081899	0.788782	2.284377	0.09574	0.66541	0.291287	589.3972	3.747957	590.4806	10.2285	595.5924	47.35348	589.4	3.7	99.0	158
19AR107 421	313.5963	19566.92	4.160965	15.38342	1.564232	0.85124	2.054529	0.096028	0.984696	0.479281	591.0882	5.561552	625.3293	9.592778	752.1339	38.06353	591.1	5.6	78.6	370
19AR107 263	675.9156	39174.31	33.85609	16.02197	0.909691	0.832552	1.315364	0.096912	0.877046	0.66677	596.2849	4.995111	615.0271	6.067868	685.5966	20.91877	596.3	5.0	87.0	227
19AR107 72	508.6099	9002.582	1.731023	16.15688	0.876627	0.802936	1.530197	0.096962	0.877407	0.573395	596.5778	4.99951	598.4836	6.919657	606.6318	27.1248	596.6	5.0	98.3	58
19AR107 212	305.7688	17013.2	1.352579	16.45601	1.193054	0.800796	1.424978	0.09716	0.676046	0.474425	597.7462	3.859343	597.2778	6.434299	596.4581	27.15507	597.7	3.9	100.2	182
19AR107 169	93.92347	4781.409	0.817063	16.01025	3.485075	0.796714	3.821723	0.097585	0.841809	0.22027	600.2432	4.824794	594.9735	17.20895	575.8578	81.05022	600.2	4.8	104.2	141
19AR107 292	568.1065	10207.71	9.439853	15.89967	0.816986	0.825326	1.284719	0.097629	0.754962	0.587647	600.5014	4.328806	611.0154	5.898314	651.1331	22.32102	600.5	4.3	92.2	254
19AR107 55	386.3755	18750.16	4.542062	16.71598	1.027875	0.791971	1.394938	0.09765	0.745685	0.534565	600.6244	4.276448	592.2891	6.259914	561.4343	25.69196	600.6	4.3	107.0	43
19AR107 326	212.4713	10802.75	1.071636	16.5449	1.847132	0.796883	2.760287	0.097858	0.898752	0.325601	601.8453	5.164278	595.0689	12.43027	570.2741	56.79118	601.8	5.2	105.5	285
19AR107 90	535.6191	16384.06	3.208015	16.35187	0.95428	0.813738	1.741176	0.098045	0.903095	0.51867	602.9444	5.198274	604.5486	7.932155	611.4875	32.18016	602.9	5.2	98.6	73
19AR107 517	256.5273	18902.85	3.838138	15.81427	1.567127	0.842835	2.427488	0.098122	1.726532	0.711242	603.3957	9.945129	620.709	11.27356	685.3002	36.41599	603.4	9.9	88.0	453
19AR107 199	245.5754	11689.33	3.165397	16.50842	1.321292	0.805944	1.790781	0.098643	0.962292	0.537359	606.4502	5.569729	600.1763	8.114884	577.4699	32.82403	606.5	5.6	105.0	169
19AR107 133	495.4636	25901.56	0.723624	16.3603	1.024382	0.824445	1.615343	0.09877	0.858523	0.53148	607.1951	4.974936	610.5251	7.411963	623.8174	29.5113	607.2	5.0	97.3	111
19AR107 433	321.9036	17004.23	3.373423	16.69953	1.139322	0.802924	1.674586	0.098771	0.749574	0.447618	607.2001	4.343638	598.4766	7.572553	566.5278	32.60195	607.2	4.3	107.2	379
19AR107 234	382.9563	20287.5	2.573669	16.62905	1.167448	0.805783	1.476842	0.098854	0.762319	0.516182	607.6885	4.420877	600.0855	6.691487	572.4078	27.50133	607.7	4.4	106.2	202
19AR107 75	278.962	12167.89	1.117342	16.01609	1.28818	0.837414	1.765878	0.09957	0.744852	0.421802	611.887	4.348032	617.7174	8.172089	640.0986	34.45609	611.9	4.3	95.6	61
19AR107 244	99.27676	4639.509	0.993344	15.79341	3.659471	0.824635	4.076455	0.099858	0.87674	0.215074	613.5747	5.131377	610.6312	18.70887	600.6483	86.22139	613.6	5.1	102.2	211
19AR107 95	762.7517	20398.23	3.448138	16.16768	0.811197	0.844146	1.314926	0.099896	0.799466	0.607993	613.798	4.680733	621.4307	6.111652	650.2614	22.42522	613.8	4.7	94.4	78
19AR107 581	23.47943	1306.405	5.073991	13.43842	7.800998	0.87152	10.50433	0.099914	1.707317	0.162535	613.9031	9.997677	636.3923	49.70816	717.9947	220.59	613.9	10.0	85.5	508
19AR107 271	335.0722	18186.56	2.419333	16.60559	1.297492	0.825335	1.989605	0.100277	0.710351	0.357031	616.0345	4.17342	611.0207	9.134741	593.4425	40.28438	616.0	4.2	103.8	235
19AR107 157	156.8578	8262.412	3.719113	16.53665	2.092705	0.814086	2.457648	0.100288	0.713407	0.29028	616.0957	4.191771	604.7438	11.19901	563.3538	51.22776	616.1	4.2	109.4	130
19AR107 29	84.86499	7051.16	0.827297	15.60766	2.714206	0.85903	3.117471	0.100511	0.79959	0.256487	617.4052	4.707675	629.5933	14.62798	674.5565	64.44836	617.4	4.7	91.5	24
19AR107 459	480.754	28592.9	1.272551	16.48781	0.693322	0.833435	1.038114	0.100777	0.603667	0.581503	618.9625	3.562696	615.5164	4.791643	603.7807	18.25298	619.0	3.6	102.5	404
19AR107 384	351.2207	11916.13	1.729516	16.48384	1.308006	0.827551	2.392864	0.101283	0.953111	0.398314	621.9263	5.650689	612.2525	11.00247	577.5891	47.68423	621.9	5.7	107.7	336
19AR107 566	504.0084	24971.34	4.706493	16.48062	0.788495	0.838199	1.221803	0.101432	0.632649	0.517799	622.7942	3.755758	618.1513	5.657054	602.1068	22.61607	622.8	3.8	103.4	495
19AR107 59	270.7364	13567.97	1.150396	16.32571	1.617827	0.839245	1.872959	0.10148	0.780625	0.416787	623.0788	4.636243	618.7292	8.677969	603.7693	36.83972	623.1	4.6	103.2	46
19AR107 207	546.8418	38605.45	2.608006	16.34431	0.851478	0.849547	1.129985	0.101564	0.685585	0.60672	623.5659	4.074817	624.4002	5.270217	628.3424	19.37725	623.6	4.1	99.2	177
19AR107 158	150.3717	8535.991	3.591588	16.35257	2.147369	0.833388	2.330839	0.101688	0.770846	0.330716	624.291	4.586644	615.4906	10.75849	584.173	47.76579	624.3	4.6	106.9	131
19AR107 337	271.0012	7338.343	1.38536	16.27114	1.603129	0.837283	2.393927	0.101992	0.817814	0.34162	626.07	4.879317	617.6449	11.07782	587.8099	48.83819	626.1	4.9	106.5	293
19AR107 18	268.9848	17044.55	2.658114	16.58423	1.277309	0.837611	1.703139	0.102051	0.731105	0.429269	626.4144	4.36427	617.8264	7.882746	587.4469	33.37113	626.4	4.4	106.6	14
19AR107 36	92.96278	6510.89	1.067489	15.90612	3.809808	0.851956	4.987741	0.10208	0.80061	0.160515	626.5868	4.780425	625.7219	23.30218	623.5145	106.249	626.6	4.8	100.5	30
19AR107 419	192.5549	13008.95	4.191723	15.45875	1.77941	0.895645	2.436234	0.102131	0.74421	0.305476	626.8882	4.445702	649.3975	11.68818	729.3845	49.16923	626.9	4.4	85.9	368
19AR107 560	240.1727	4402.407	4.619292	15.94882	1.568	0.842433	5.18414	0.102989	0.663361	0.12796	631.9032	3.992904	620.4875	24.07308	579.9917	111.7797	631.9	4.0	109.0	490
19AR107 362	276.7561	9523.428	4.64149	16.22496	0.9503	0.851101	1.534925	0.103119	0.869416	0.566423	632.6639	5.239187	625.2531	7.165972	599.4518	27.38509	632.7	5.2	105.5	318
19AR107 590	437.1072	32526.09	1.17421	16.39043	0.815375	0.863726	1.287986	0.103639	0.758922	0.589232	635.6969	4.594201	632.155	6.060946	620.4279	22.45996	635.7	4.6	102.5	517
19AR107 378	61.4777	3663.148	47.39418	15.34057	5.169081	0.877045	6.621236	0.104128	0.792836	0.119741	638.5582	4.82005	639.3857	31.42352	643.2664	141.4341	638.6	4.8	99.3	333
19AR107 559	113.6902	10552.99	1.398853	15.64706	3.353549	0.905556	4.047097	0.105162	0.676906	0.167257	644.5869	4.152199	654.6924	19.53083	690.6233	85.13058	644.6	4.2	93.3	489
19AR107 79	248.5365	12206.7	10.85969	15.96325	1.090745	0.897396	1.924314	0.106336	0.762325	0.396154	651.4309	4.723346	650.3348	9.241532	647.4878	37.97641	651.4	4.7	100.6	65
19AR107 294	537.2529	33845.48	17.68411	16.23183	0.89133	0.893595	1.151123	0.106388	0.711954	0.618487	651.7348	4.413207	648.2991	5.515812	637.3101	19.48389	651.7	4.4	102.3	255
19AR107 397	380.2906	24375.71	12.38656	15.57472	1.201342	0.929698	1.972301	0.106465	1.461416	0.74097	652.1866	9.064883	667.476	9.648701	720.3493	28.11267	652.2	9.1	90.5	346
19AR107 313	232.264	15245.87	3.954307	14.89407	1.469174	0.974413	2.166896	0.106688	1.528444	0.705361	653.4836	9.498561	690.7358	10.85899	814.93	32.11924	653.5	9.5	80.2	273
19AR107 377	436.6697	25020.66	8.385764	15.55693	0.915568	0.939599	1.336758	0.10717	0.818443	0.61226	656.2913	5.106999	672.672	6.575356	728.8638	22.38174	656.3	5.1	90.0	332
19AR107 569	495.6715	30653.69	6.580131	16.17768	0.736065	0.909167	1.103339	0.107669	0.516631	0.468243	659.1965	3.237273	656.6146	5.335098	648.6846	20.95713	659.2	3.2	101.6	498
19AR107 354	1296.447	18329.12	33.24254	15.99475	0.646977	0.914638	1.005551	0.107838	0.716338	0.712384	660.1777	4.495012	659.5202	4.877527	658.2028	15.11146	660.2	4.5	100.3	310
19AR107 538	459.7757	34939.79	5.061791	15.86623	0.832638	0.93194	1.17964	0.108212	0.668213	0.566455	662.3535	4.206148	668.6547	5.778012	690.8563	20.73843	662.4	4.2	95.9	471
19AR107 1	182.2971	9139.48	5.589199	14.82845	1.593233	0.989712	2.022681	0.108886	0.867443	0.428858	666.2762	5.490919	698.5731	10.21622	804.8109	38.26498	666.3	5.5	82.8	1
19AR107 107	409.7842	5830.793	2.152511	15.63766	0.858131	0.929294	1.771198	0.109891	0.651753	0.367973	672.1138	4.159897	667.2631	8.66288	651.8685	35.35835	672.1	4.2	103.1	88
19AR107 60	194.2873	14804.09	2.177294	16.09037	1.932644	0.926753	2.291861	0.110255	0.791115	0.345185	674.2279	5.064457	665.9251	11.1937	638.8798	46.2942	674.2	5.1	105.5	47
19AR107 443	1177.003	50007.66	3.72759	15.94334	0.593343	0.954511	0.949012	0.11074	0.666306	0.702105	677.0416	4.282353	680.4488	4.705951	692.644	14.42146	677.0	4.3	97.7	389
19AR107 204	451.8972	24732.67	4.191582	16.03349	0.809216	0.947512	1.406245	0.111443	0.696023	0.494951	681.1208	4.498898	676.8061	6.947079	663.3891	26.15805	681.1	4.5	102.7	174
19AR107 338	372.5755	27514.87	2.600379	15.90968	1.046809	0.966854	1.564379	0.112485	0.889682	0.568713	687.164	5.799012	686.8408	7.808529	686.7314	27.46558	687.2	5.8	100.1	294
19AR107 513	170.1448	9999.6	2.383302	15.69826	2.032042	0.96883	2.615001	0.113176	0.756243	0.289194	691.1643	4.956428	687.8608	13.06668	677.9818	53.52807	691.2	5.0	101.9	451
19AR107 152	132.9548	7621.098	3.019282	15.86968	2.177232	0.959614	2.570385	0.113868	0.682826	0.265651	695.1751	4.499849	683.0965	12.78136	644.4587	53.24637	695.2	4.5	107.9	125
19AR107 530	355.3181	18169.55	0.844859	15.79034	1.138506	0.986465	1.485265	0.114788	0.863993	0.58171	700.4927	5.734973	696.9149	7.489319	686.3422	25.77335	700.5	5.7	102.1	464
19AR107 463	336.6623	17923.95	7.760318	15.10744	0.9239	1.038155	1.444339	0.115719	0.705258	0.488291	705.8788	4.715392	722.9983	7.470194	777.3677	26.50671	705.9	4.7	90.8	408
19AR107 502	402.0236	18936.38	2.010249	15.8523	0.893972	0.994378	1.316274	0.116054	0.778695	0.59159	707.8112	5.219876	700.9516	6.663869	679.9325	22.67391	707.8	5.2	104.1	441
19AR107 565	133.4127	5968.699	5.874239	14.95828	1.394008	1.028852	2.10978	0.116111	0.927704	0.439716	708.1433	6.221505	718.3532	10.86391	751.2735	40.01949	708.1	6.2	94.3	494
19AR107 168	738.7116	10403.25	2.351171	15.16582	0.713298	1.03183	1.252256	0.116323	0.986267	0.787592	709.3655	6.625046	719.8423	6.457279	753.5299	16.26975	709.4	6.6	94.1	140
19AR107 251	125.6551	8015.927	1.602563	14.85088	1.765609	1.052974	2.734396	0.116817	1.360249	0.497458	712.2172	9.171933	730.3542	14.24144	787.3148	49.82831	712.2	9.2	90.5	217
19AR107 528	384.9777	26115.65	4.89776	15.68762	1.291466	1.015512	1.607793	0.116899	0.786527	0.489197	712.6907	5.306756	711.6548	8.225629	709.2937	29.81727	712.7	5.3	100.5	462
19AR107 486	376.3796	16567.44	4.171996	14.84522	0.770427	1.084674	1.532036	0.118312	1.0216	0.666825	720.8414	6.967317	745.9132	8.09411	822.8276	23.83554	720.8	7.0	87.6	426
19AR107 112	156.8252	10779.83	1.523875	15.1735	1.785409	1.078053	2.045733	0.12093	0.752067	0.367627	735.9117	5.230328	742.6829	10.77652	764.0473	40.0961	735.9	5.2	96.3	93
19AR107 439	287.547	13485.75	3.201374	15.57137	0.729731	1.068591	1.265519	0.122565	0.751385	0.593737	745.3123	5.288555	738.049	6.638065	716.9732	21.61551	745.3	5.3	104.0	385
19AR107 123	255.1437	11963.27	0.93483	14.75639	1.411833	1.125934	1.952025	0.123031	1.09701	0.561986	747.9871	7.74734	765.8135	10.4977	819.113	33.73015	748.0	7.7	91.3	103
19AR107 258	136.4255	7210.741	0.712721	15.54157	2.49473	1.071794	3.004789	0.124415	0.725448	0.241431	755.9267	5.174536	739.6203	15.78497	691.4953	62.19309	755.9	5.2	109.3	223
19AR107 249	246.9569	23290.62	4.83812	15.60111	0.989209	1.087695	1.347468	0.124592	0.632828	0.469642	756.9374	4.519576	747.3834	7.128459	719.7836	25.24856	756.9	4.5	105.2	215
19AR107 34	223.2232	11325.04	0.797126	14.19156	0.983468	1.203762	2.000175	0.126405	1.162214	0.581056	767.322	8.407618	802.321	11.09408	901.6463	33.54703	767.3	8.4	85.1	28
19AR107 28	249.2253	14338.7	3.676678	14.2493	0.877574	1.215384	1.834374	0.127431	0.787182	0.429128	773.192	5.735589	807.6617	10.21873	904.7869	34.13395	773.2	5.7	85.5	23
19AR107 553	510.0028	26010.56	2.317036	15.3219	0.786305	1.142071	1.072372	0.128212	0.625065	0.582881	777.6568	4.579109	773.4915	5.805494	762.3837	18.36941	777.7	4.6	102.0	484
19AR107 492	93.50399	4626.214	3.596297	14.96773	2.593628	1.126038	3.069395	0.12822	0.814318	0.265302	777.7023	5.96587	765.863	16.50832	732.4172	62.68036	777.7	6.0	106.2	432
19AR107 596	539.0214	44219.38	2.302426	15.18846	0.638962	1.157012	0.917799	0.128427	0.606532	0.660855	778.8866	4.449953	780.5495	4.998808	786.2014	14.46492	778.9	4.4	99.1	522
19AR107 70	381.5723	26812.45	4.923588	14.3158	0.855936	1.228843	1.315996	0.129055	0.936722	0.711797	782.4704	6.902209	813.8121	7.367312	901.3874	19.06004	782.5	6.9	86.8	56
19AR107 65	116.0135	10663.19	1.075429	14.51751	1.998628	1.208933	3.401724	0.130327	2.436632	0.716293	789.7322	18.11091	804.701	18.90597	847.2426	49.37483	789.7	18.1	93.2	51
19AR107 320	500.6321	2728.106	3.976213	13.62191	0.692311	1.225445	1.259112	0.130468	0.903502	0.717571	790.5356	6.721923	812.2625	7.040089	873.1529	18.16525	790.5	6.7	90.5	279
19AR107 369	450.2789	26261.26	38.96584	13.69385	0.704497	1.316217	1.092917	0.132033	0.776828	0.710784	799.4516	5.840712	852.8559	6.306246	995.3145	15.62363	799.5	5.8	80.3	325
19AR107 62	126.5538	11529.08	2.883256	14.28737	1.869533	1.271581	2.462121	0.134794	1.41871	0.576214	815.157	10.8634	833.0975	13.99531	882.1693	41.64138	815.2	10.9	92.4	49
19AR107 371	232.1873	16339.49	2.249398	14.47336	0.9913	1.278632	1.476161	0.136286	0.793781	0.537734	823.6242	6.137361	836.2444	8.410947	870.8364	25.78296	823.6	6.1	94.6	327
19AR107 176	609.8009	16921.52	2.488941	14.07378	0.64626	1.341454	0.96744	0.139081	0.677306	0.700102	839.4618	5.331085	863.8598	5.627927	927.8936	14.20571	839.5	5.3	90.5	148
19AR107 303	675.3026	5362.253	2.211506	14.12886	0.65751	1.353438	2.401331	0.144096	1.075254	0.447774	867.7822	8.730091	869.0431	14.02313	873.141	44.48193	867.8	8.7	99.4	264
19AR107 57	70.23243	5827.016	3.190013	14.03256	2.20101	1.483454	2.772474	0.156683	0.822123	0.296531	938.3161	7.178976	923.6436	16.81725	889.6575	54.70457	889.7	54.7	105.5	44
19AR107 430	81.02179	7606.159	1.657735	14.28518	1.790575	1.391198	2.164593	0.148243	0.722026	0.333562	891.1059	6.009121	885.2055	12.788	871.3927	42.26913	891.1	6.0	102.3	377
19AR107 550	433.1444	35997.43	2.938266	14.44968	0.723848	1.459927	1.042849	0.15409	0.632716	0.606719	923.8512	5.445813	913.9783	6.284426	891.1289	17.10195	891.1	17.1	103.7	481
19AR107 121	300.8168	31549.44	1.31423	14.13111	0.776184	1.419739	1.040707	0.146909	0.64611	0.620838	883.6113	5.335113	897.2532	6.200176	931.9211	16.74369	931.9	16.7	94.8	101
19AR107 236	163.4485	19396.25	3.305967	14.03653	1.613671	1.412062	2.934684	0.145998	2.416561	0.823448	878.4883	19.84628	894.0264	17.44613	933.5619	34.17211	933.6	34.2	94.1	204
19AR107 98	585.0738	3000.893	2.909369	13.35494	0.820377	1.488604	1.31896	0.153613	0.879352	0.666701	921.1874	7.548315	925.7469	8.011129	937.4962	20.1379	937.5	20.1	98.3	81
19AR107 64	206.9585	16716.42	1.269633	13.99363	0.904315	1.533985	1.17924	0.158181	0.705923	0.598625	946.659	6.215172	944.0964	7.248644	938.9967	19.34463	939.0	19.3	100.8	50
19AR107 32	144.546	10041.98	2.486117	13.91214	1.470789	1.476996	1.821563	0.152129	0.723715	0.397305	912.8878	6.160239	920.9996	11.02923	941.3908	34.25789	941.4	34.3	97.0	26
19AR107 194	255.5583	7734.025	1.645173	13.85487	1.070629	1.433921	1.853537	0.147665	1.016382	0.548347	887.8606	8.430195	903.187	11.08836	941.7756	31.7758	941.8	31.8	94.3	164
19AR107 510	58.63922	4874.454	3.407285	13.59617	2.731886	1.668416	3.265073	0.171813	0.837946	0.256639	1022.093	7.920132	996.5833	20.73166	941.7769	64.6693	941.8	64.7	108.5	448
19AR107 286	76.61831	6261.222	0.698151	13.71617	1.972609	1.652071	2.24395	0.170068	0.755775	0.336805	1012.485	7.081445	990.3447	14.19433	942.5062	43.29333	942.5	43.3	107.4	248
19AR107 335	106.4862	9358.341	3.061134	13.87293	1.703237	1.509787	3.842896	0.15505	3.31148	0.861714	929.2119	28.65597	934.3534	23.47712	947.3709	39.93345	947.4	39.9	98.1	291
19AR107 344	578.4979	35619.38	4.7786	14.04369	0.984079	1.441755	1.839697	0.147597	1.499675	0.815175	887.4819	12.43384	906.45	11.03018	953.8429	21.7752	953.8	21.8	93.0	300
19AR107 250	531.4085	38500.64	2.112607	14.00589	0.681891	1.460866	0.899291	0.149553	0.565569	0.628906	898.4585	4.743193	914.3661	5.420715	953.85	14.30752	953.8	14.3	94.2	216
19AR107 239	175.5149	26637.65	3.727437	13.91356	1.49529	1.417105	1.770313	0.144814	0.871683	0.492389	871.8247	7.108066	896.1472	10.53906	957.4992	31.49738	957.5	31.5	91.1	207
19AR107 535	157.6762	10613.13	1.396264	13.76556	1.374655	1.586503	2.181516	0.161956	0.854241	0.391582	967.6355	7.67548	964.9252	13.58758	959.6257	41.00296	959.6	41.0	100.8	468
19AR107 94	114.915	10922.14	0.942461	13.76354	1.508056	1.671897	1.922867	0.169882	0.804908	0.418598	1011.462	7.534777	997.9071	12.21771	969.1482	35.63099	969.1	35.6	104.4	77
19AR107 321	667.502	4641.905	1.555945	13.41459	0.642169	1.559544	1.41476	0.158342	1.193415	0.843546	947.5556	10.51645	954.2866	8.753029	970.7329	15.49944	970.7	15.5	97.6	280
19AR107 67	327.2734	30500.72	6.896133	13.84531	1.008667	1.484995	1.761444	0.15067	1.427685	0.81052	904.7203	12.05114	924.2735	10.68844	972.0934	21.02331	972.1	21.0	93.1	53
19AR107 77	303.3598	6091.432	6.509787	13.49088	0.895686	1.645415	1.53314	0.166931	0.913708	0.595972	995.1804	8.425933	987.7929	9.682921	972.2847	25.1083	972.3	25.1	102.4	63
19AR107 542	397.3414	15406.77	4.933456	13.76929	0.627709	1.596627	1.160987	0.161768	0.916594	0.789496	966.5959	8.227527	968.8919	7.248674	975.0103	14.50707	975.0	14.5	99.1	474
19AR107 180	157.3737	17737.83	1.777512	13.77103	1.131774	1.646468	1.384628	0.166783	0.703135	0.507815	994.3624	6.479166	988.1972	8.747029	975.4225	24.30529	975.4	24.3	101.9	152
19AR107 491	425.4827	12310.86	2.413888	13.73882	0.852203	1.58781	1.442495	0.160781	1.013986	0.702939	961.116	9.053885	965.4384	8.987145	976.2	20.91221	976.2	20.9	98.5	431
19AR107 218	302.4152	16355.57	1.893855	13.74435	0.807458	1.523002	1.109765	0.154055	0.646078	0.582176	923.6538	5.559718	939.6855	6.802216	978.3407	18.37789	978.3	18.4	94.4	188
19AR107 20	258.9701	26102.5	2.535534	13.83339	0.99975	1.386787	1.394687	0.140219	0.793015	0.568597	845.8994	6.286631	883.3307	8.228342	979.1637	23.37495	979.2	23.4	86.4	16
19AR107 78	123.2754	11527.24	2.720635	13.66826	1.314334	1.571793	1.575859	0.158924	0.685814	0.4352	950.7967	6.062621	959.1343	9.779578	979.169	28.90393	979.2	28.9	97.1	64
19AR107 141	343.0491	21761.5	3.278975	13.79124	0.894543	1.545841	1.250064	0.156146	0.818431	0.654711	935.3216	7.125539	948.836	7.707334	981.2255	19.22626	981.2	19.2	95.3	118
19AR107 386	89.64074	7983.713	1.579238	13.54314	1.745957	1.727457	2.316147	0.174441	1.010777	0.436405	1036.533	9.67812	1018.805	14.89623	981.8071	42.44436	981.8	42.4	105.6	338
19AR107 352	457.8379	27107.79	1.977236	13.76047	0.756402	1.590218	1.456606	0.160402	1.230617	0.844853	959.0114	10.96587	966.3827	9.080375	984.0643	15.86483	984.1	15.9	97.5	308
19AR107 478	313.5308	25894.01	3.960125	13.7461	0.78472	1.431638	1.076634	0.143962	0.705141	0.65495	867.0277	5.720455	902.2341	6.436327	990.3672	16.52807	990.4	16.5	87.5	418
19AR107 266	179.0591	7878.538	3.550037	13.57329	1.014246	1.64076	1.480995	0.164894	0.949368	0.641034	983.9186	8.663082	986.0046	9.343546	991.5335	23.10021	991.5	23.1	99.2	230
19AR107 220	149.2333	15859.49	3.236909	13.64296	1.328453	1.650886	1.663934	0.165867	0.707288	0.425069	989.3015	6.486738	989.8908	10.52223	992.0784	30.60552	992.1	30.6	99.7	189
19AR107 144	135.0587	9349.997	4.451879	13.56552	1.166618	1.618367	1.935448	0.162563	0.893618	0.461711	971.0016	8.055167	977.3578	12.14727	992.5695	34.90756	992.6	34.9	97.8	119
19AR107 181	346.9332	12114.48	1.921412	13.58608	0.859514	1.542	1.28793	0.154872	0.895176	0.69505	928.2158	7.738662	947.3027	7.933041	992.8151	18.81341	992.8	18.8	93.5	153
19AR107 128	66.97642	6959.483	1.831303	13.4254	2.729415	1.671315	3.986419	0.167563	0.971075	0.243596	998.6708	8.983989	997.6858	25.33006	996.3919	78.59025	996.4	78.6	100.2	107
19AR107 551	168.6987	8581.721	3.48893	13.49515	1.201908	1.746958	1.561829	0.175139	0.647462	0.414554	1040.362	6.220507	1026.038	10.08575	996.5107	28.88215	996.5	28.9	104.4	482
19AR107 549	164.8045	15506.25	3.71421	13.63322	1.106681	1.805021	1.678752	0.180856	0.649509	0.3869	1071.651	6.412687	1047.277	10.96932	997.6441	31.45084	997.6	31.5	107.4	480
19AR107 92	271.6971	18837.58	2.630051	13.65363	0.745027	1.6066	1.155051	0.160961	0.843512	0.730282	962.1149	7.538983	972.7843	7.22889	997.8244	16.01015	997.8	16.0	96.4	75
19AR107 76	205.4243	9491.162	5.681876	13.47318	1.092471	1.618576	1.374816	0.162092	0.770739	0.560612	968.3914	6.930207	977.439	8.628852	998.6891	23.12567	998.7	23.1	97.0	62
19AR107 156	99.03418	12633.85	4.336939	13.58955	1.478954	1.677769	1.737226	0.167923	0.672848	0.387312	1000.658	6.236364	1000.136	11.05255	999.8567	32.51082	999.9	32.5	100.1	129
19AR107 469	141.0242	9843.362	1.222335	13.48732	1.366718	1.576616	2.026795	0.157719	1.155703	0.570212	944.0855	10.14949	961.0367	12.59326	1000.894	33.80285	1000.9	33.8	94.3	413
19AR107 482	133.4026	11715.8	1.931182	13.53513	1.305236	1.793123	1.547046	0.179265	0.750332	0.485009	1062.958	7.352853	1042.961	10.0848	1002.161	27.46682	1002.2	27.5	106.1	422
19AR107 202	187.4741	17940.13	5.573969	13.61519	1.684515	1.718371	1.907964	0.171765	0.688024	0.360606	1021.831	6.50155	1015.416	12.24699	1002.475	36.12123	1002.5	36.1	101.9	172
19AR107 116	271.7376	20813.45	1.52968	13.65631	0.75001	1.630856	1.157727	0.162879	0.733268	0.633369	972.7584	6.620834	982.1894	7.287225	1004.195	18.18303	1004.2	18.2	96.9	96
19AR107 505	56.16994	5550.483	1.270032	13.2606	3.587821	1.824386	3.741371	0.182101	0.63696	0.170248	1078.44	6.325387	1054.263	24.54358	1005.391	74.84488	1005.4	74.8	107.3	444
19AR107 389	274.4616	30651.44	10.86273	13.59805	1.042465	1.717982	1.487451	0.171267	1.002735	0.67413	1019.089	9.45198	1015.271	9.546792	1007.908	22.27244	1007.9	22.3	101.1	340
19AR107 472	202.6263	16375.44	2.584606	13.5388	1.051647	1.703069	1.46084	0.169678	0.604271	0.413646	1010.338	5.650792	1009.685	9.345873	1009.131	26.95402	1009.1	27.0	100.1	415
19AR107 237	124.5016	9796.609	3.138001	13.39574	1.262551	1.717938	1.641169	0.171092	0.711933	0.433797	1018.123	6.70495	1015.255	10.53335	1009.936	29.98664	1009.9	30.0	100.8	205
19AR107 387	100.5119	8151.073	2.26467	13.36728	1.908831	1.817916	2.84145	0.181037	1.001568	0.352485	1072.637	9.896979	1051.934	18.61506	1010.067	53.94658	1010.1	53.9	106.2	339
19AR107 45	298.0299	29222.9	2.614776	13.57563	0.969847	1.611296	1.402909	0.160452	0.767338	0.546962	959.2887	6.839485	974.6119	8.790011	1010.174	23.79567	1010.2	23.8	95.0	39
19AR107 385	224.109	23689.25	4.637058	13.56872	1.108939	1.706043	1.561062	0.169816	0.967935	0.620049	1011.097	9.057853	1010.801	9.993543	1011.025	24.8313	1011.0	24.8	100.0	337
19AR107 135	297.6774	29649.33	3.983242	13.61124	0.672373	1.654197	1.062216	0.164601	0.738036	0.694808	982.2952	6.724367	991.1583	6.722072	1011.691	15.46839	1011.7	15.5	97.1	113
19AR107 548	219.929	21679.78	5.278333	13.54533	1.12889	1.797489	1.329252	0.178661	0.666194	0.50118	1059.654	6.509676	1044.547	8.672526	1013.942	23.29174	1013.9	23.3	104.5	479
19AR107 74	740.5472	71915.7	2.037597	13.60672	0.69359	1.675101	1.045613	0.166438	0.770949	0.737318	992.4577	7.091461	999.1238	6.648259	1014.645	14.31297	1014.6	14.3	97.8	60

19AR107 593	392.4751	40576.54	6.054146	13.59148	0.789875	1.718988	1.100423	0.170671	0.755947	0.68696	1015.809	7.104527	1015.647	7.064185	1016.16	16.20058	1016.2	16.2	100.0	519					
19AR107 412	459.501	13750.43	3.016351	13.50715	0.685418	1.681129	1.222027	0.166781	0.660038	0.540117	994.3518	6.081979	1001.409	7.780413	1017.755	20.82468	1017.8	20.8	97.7	361					
19AR107 435	421.3728	36186.17	1.43175	13.59206	0.796921	1.693731	1.137842	0.168021	0.769754	0.676504	1001.198	7.138107	1006.171	7.264561	1017.881	16.9714	1017.9	17.0	98.4	381					
19AR107 189	81.54894	6576.617	2.679579	13.28539	2.0719	1.721897	3.340228	0.170798	0.81197	0.243088	1016.506	7.635882	1016.733	21.45884	1018.083	65.63532	1018.1	65.6	99.8	160					
19AR107 275	515.5375	33178.88	8.292483	13.61537	0.632127	1.720827	0.956382	0.17058	0.673795	0.704524	1015.304	6.329541	1016.333	6.141905	1019.412	13.74391	1019.4	13.7	99.6	239					
19AR107 537	220.6908	26111.79	3.197773	13.51699	0.885136	1.739909	1.2442	0.172452	0.533623	0.428888	1025.605	5.059707	1023.43	8.022684	1019.642	22.74419	1019.6	22.7	100.6	470					
19AR107 356	71.85601	6454.498	2.32158	13.21184	1.615519	1.678926	2.88285	0.16629	0.833698	0.289192	991.6359	7.662776	1000.575	18.34721	1021.075	55.86627	1021.1	55.9	97.1	312					
19AR107 358	446.1617	34376.98	4.784579	13.52158	0.693362	1.715258	1.044883	0.169757	0.716655	0.68587	1010.775	6.70442	1014.253	6.702278	1022.633	15.38938	1022.6	15.4	98.8	314					
19AR107 23	290.7321	32002.16	1.28607	13.56121	0.739894	1.695461	1.076571	0.167788	0.6323	0.587328	999.9117	5.856504	1006.822	6.875967	1022.755	17.63415	1022.8	17.6	97.8	19					
19AR107 137	254.9522	23627.25	2.707171	13.51298	0.856096	1.708338	1.2345	0.169034	0.729751	0.591131	1006.784	6.802038	1011.662	7.906801	1023.1	20.15103	1023.1	20.2	98.4	115					
19AR107 217	268.5884	22142.09	2.391769	13.4934	0.746406	1.70014	1.015725	0.168216	0.640503	0.630587	1002.275	5.945436	1008.584	6.493972	1023.176	15.9533	1023.2	16.0	98.0	187					
19AR107 4	173.5197	9991.104	3.711384	13.39567	1.227414	1.694935	1.705975	0.167659	0.739814	0.43366	999.2013	6.847818	1006.624	10.89492	1023.682	31.1164	1023.7	31.1	97.6	3					
19AR107 5	26.40777	3037.624	1.407837	12.82579	5.138287	1.80934	5.622354	0.178836	0.944322	0.167958	1060.61	9.235047	1048.839	36.78357	1025.268	112.1948	1025.3	112.2	103.4	4					
19AR107 182	576.701	44644.93	2.252793	13.52784	0.606318	1.739246	0.926482	0.171895	0.650757	0.702396	1022.546	6.153367	1023.184	5.973127	1025.41	13.34201	1025.4	13.3	99.7	154					
19AR107 529	224.0718	28287.62	3.097658	13.48966	0.878743	1.746709	1.26422	0.172631	0.898092	0.710392	1026.593	8.523092	1025.947	8.163375	1025.43	17.9996	1025.4	18.0	100.1	463					
19AR107 546	105.1177	14241.6	1.9501	13.40669	1.827583	1.783471	2.028609	0.176253	0.699268	0.344703	1046.473	6.754576	1039.446	13.19873	1025.561	38.54341	1025.6	38.5	102.0	477					
19AR107 587	199.5778	22836.76	2.184331	13.48631	1.024989	1.703613	1.357561	0.168302	0.650523	0.479185	1002.748	6.041082	1009.889	8.686132	1026.272	24.12203	1026.3	24.1	97.7	514					
19AR107 319	328.1055	24425.52	1.869034	13.50451	0.845562	1.744541	1.218764	0.172317	0.817738	0.670957	1024.867	7.748486	1025.145	7.866283	1026.598	18.29809	1026.6	18.3	99.8	278					
19AR107 54	261.3493	27147.82	3.329458	13.46342	1.033444	1.78096	1.372922	0.17582	0.780395	0.568419	1044.098	7.522464	1038.53	8.927838	1027.691	22.86209	1027.7	22.9	101.6	42					
19AR107 574	208.2048	22993.44	3.64194	13.47417	0.856773	1.745068	1.159832	0.172245	0.687551	0.592802	1024.471	6.512571	1025.34	7.48673	1028.056	18.90796	1028.1	18.9	99.7	502					
19AR107 11	199.5312	14436.95	2.99396	13.43708	1.002751	1.74525	1.688166	0.172201	0.777869	0.460777	1024.224	7.366434	1025.407	10.89777	1028.793	30.31459	1028.8	30.3	99.6	7					
19AR107 281	186.8671	14187.76	1.79916	13.4427	1.506384	1.762158	1.781513	0.173797	0.777373	0.436355	1032.997	7.419877	1031.641	11.54075	1029.629	32.42768	1029.6	32.4	100.3	244					
19AR107 343	275.4805	13040.06	3.030002	13.39347	0.990025	1.703704	1.362724	0.168008	0.824696	0.605182	1001.126	7.647092	1009.923	8.71934	1029.916	21.95183	1029.9	22.0	97.2	299					
19AR107 324	236.6826	10360.73	3.358209	13.33021	0.872658	1.681693	1.625027	0.165728	1.080383	0.66484	988.5301	9.901359	1001.623	10.34768	1031.25	24.555	1031.2	24.6	95.9	283					
19AR107 464	201.1735	19986.95	2.216941	13.3901	0.89923	1.782934	1.30959	0.175685	0.63414	0.484228	1043.356	6.108666	1039.25	8.519375	1031.484	23.17924	1031.5	23.2	101.2	409					
19AR107 289	366.6698	9176.213	2.111188	13.23465	0.807253	1.732229	1.178919	0.170214	0.657082	0.55736	1013.291	6.161239	1020.58	7.589449	1037.105	19.7852	1037.1	19.8	97.7	251					
19AR107 13	200.9843	14062.93	3.805994	13.35859	1.091138	1.495713	2.140552	0.146923	1.316482	0.61502	883.6922	10.87149	928.6435	13.02666	1037.798	34.1037	1037.8	34.1	85.2	9					
19AR107 226	96.34213	9790.478	1.286933	13.23504	1.739701	1.72689	2.565132	0.169577	1.190784	0.464219	1009.782	11.12988	1018.593	16.49586	1038.442	45.88255	1038.4	45.9	97.2	195					
19AR107 318	348.3441	26876.27	4.496219	13.42698	0.710505	1.753369	1.144683	0.171968	0.7829	0.683944	1022.946	7.405536	1028.406	7.401707	1040.898	16.87349	1040.9	16.9	98.3	277					
19AR107 600	699.8989	30251.04	7.332152	13.39836	0.559281	1.738234	1.151983	0.170434	0.791693	0.687244	1014.503	7.431648	1022.809	7.425424	1041.483	16.90705	1041.5	16.9	97.4	526					
19AR107 110	350.8564	7726.11	2.714036	13.19581	0.700717	1.444846	1.76092	0.141631	1.567758	0.890306	853.8777	12.53805	907.7345	10.56709	1042.006	16.20039	1042.0	16.2	81.9	91					
19AR107 370	119.1468	12175.78	1.730071	13.25598	1.151607	1.754782	1.462993	0.17179	0.715118	0.488805	1021.965	6.7584	1028.926	9.462824	1044.615	25.76467	1044.6	25.8	97.8	326					
19AR107 89	159.6868	17611.2	1.25632	13.31197	1.172602	1.702572	1.642857	0.166378	0.707521	0.430665	992.1238	6.506003	1009.498	10.50929	1048.293	29.90494	1048.3	29.9	94.6	72					
19AR107 442	40.12988	3079.256	1.041125	12.69291	3.694641	1.725707	5.001971	0.168536	0.966236	0.193171	1004.039	8.983632	1018.153	32.16653	1049.525	98.972	1049.5	99.0	95.7	388					
19AR107 159	1207.737	54558.46	6.980787	13.40916	0.576489	1.754336	0.856777	0.171256	0.602075	0.702721	1019.027	5.674959	1028.762	5.541126	1050.417	12.3034	1050.4	12.3	97.0	132					
19AR107 262	253.5517	15535.39	3.12742	13.29425	0.972444	1.709971	1.652592	0.166804	1.20139	0.726973	994.4782	11.07162	1012.274	10.58852	1051.88	22.88436	1051.9	22.9	94.5	226					
19AR107 340	509.825	31010.3	2.981001	13.36166	0.820388	1.781256	1.344128	0.173742	0.89095	0.662846	1032.695	8.501656	1038.638	8.741109	1052.064	20.29833	1052.1	20.3	98.2	296					
19AR107 243	122.8735	10804	2.269554	13.1271	1.567564	1.581761	2.181031	0.154025	0.815427	0.373872	923.4865	7.015838	963.0622	13.56883	1055.439	40.74128	1055.4	40.7	87.5	210					
19AR107 114	291.7017	39298.77	2.081897	13.35583	0.74231	1.796079	1.123341	0.174637	0.702933	0.625753	1037.612	6.736993	1044.035	7.326978	1058.398	17.65776	1058.4	17.7	98.0	95					
19AR107 39	32.12199	2915.415	1.812584	12.54999	5.747866	1.729413	6.197758	0.168096	1.295513	0.209029	1001.614	12.01822	1019.532	39.89489	1059.103	122.1156	1059.1	122.1	94.6	33					
19AR107 106	246.1221	21344.55	1.320187	13.27028	0.833033	1.907274	1.165214	0.185091	0.617916	0.530303	1094.728	6.221303	1083.633	7.761953	1062.288	19.89374	1062.3	19.9	103.1	87					
19AR107 268	236.5066	28902.64	5.213173	13.3284	0.838432	1.758586	1.450486	0.170569	1.15781	0.798222	1015.247	10.87575	1030.328	9.38929	1063.379	17.57272	1063.4	17.6	95.5	232					
19AR107 126	68.53147	3939.567	1.850478	12.6821	2.08787	1.865917	4.14827	0.180317	0.934005	0.225155	1068.709	9.198283	1069.085	27.43034	1070.747	81.22133	1070.7	81.2	99.8	106					
19AR107 295	78.73367	7907.841	1.495385	12.97655	1.901211	1.874356	2.832481	0.181103	0.70817	0.250018	1072.998	6.999936	1072.07	18.75676	1071.077	55.112	1071.1	55.1	100.2	256					
19AR107 296	60.48858	5823.248	1.840866	12.78279	2.25059	2.031221	3.004634	0.194893	0.79204	0.263606	1147.828	8.32786	1126.025	20.44654	1085.101	58.14068	1085.1	58.1	105.8	257					
19AR107 287	152.2362	12596.5	1.99981	12.99958	1.095378	1.895892	1.471624	0.181849	0.7504	0.509913	1077.065	7.443191	1079.65	9.782987	1085.734	25.3609	1085.7	25.4	99.2	249					
19AR107 231	293.4193	28362.67	1.768303	13.0806	0.804933	1.867793	1.083146	0.179061	0.690112	0.637137	1061.841	6.756193	1069.749	7.163167	1086.768	16.73388	1086.8	16.7	97.7	200					
19AR107 544	810.4038	62247.08	1.388033	13.1519	0.527709	1.84152	0.786248	0.176299	0.555246	0.706198	1046.722	5.364575	1060.404	5.173911	1089.54	11.15398	1089.5	11.2	96.1	476					
19AR107 341	380.7292	30562.36	11.4788	13.07764	0.858282	1.844323	1.331766	0.17606	0.943945	0.708792	1045.413	9.109532	1061.406	8.768532	1095.325	18.78846	1095.3	18.8	95.4	297					
19AR107 173	289.8841	25967.59	2.31553	13.05921	0.75572	1.97704	1.25263	0.188693	0.860226	0.686736	1114.293	8.802741	1107.711	8.446825	1095.702	18.21854	1095.7	18.2	101.7	145					
19AR107 291	450.5198	32970.76	4.111445	13.04118	0.706332	1.905944	1.16923	0.181781	0.848226	0.725457	1076.698	8.410887	1083.168	7.786841	1097.068	16.09201	1097.1	16.1	98.1	253					
19AR107 104	107.2402	14929.08	1.861668	12.95372	1.193455	2.0103	1.763927	0.191104	0.642549	0.364272	1127.354	6.64577	1118.992	11.96139	1103.652	32.8472	1103.7	32.8	102.1	85					
19AR107 516	297.5728	21353.95	1.737538	12.941	0.697112	1.946169	1.179293	0.184754	0.772664	0.655193	1092.894	7.767392	1097.127	7.910124	1106.423	17.82019	1106.4	17.8	98.8	452					
19AR107 125	65.92274	9378.381	1.774654	12.8007	2.332193	2.032192	2.754311	0.192895	0.817728	0.29689	1137.039	8.524055	1126.35	18.74564	1106.649	52.55313	1106.6	52.6	102.7	105					
19AR107 497	246.2853	19865.62	3.202989	12.93583	0.852761	1.981284	1.386251	0.187695	0.817792	0.589931	1108.877	8.331234	1109.158	9.354647	1110.558	22.36042	1110.6	22.4	99.8	436					
19AR107 562	213.608	18654.93	1.832828	12.88455	0.959667	1.987455	1.226711	0.187957	0.636913	0.519204	1110.299	6.496154	1111.257	8.286623	1113.983	20.93158	1114.0	20.9	99.7	492					
19AR107 436	569.3439	67477.15	2.616894	12.94218	0.680243	1.946224	1.081572	0.183353	0.836773	0.773663	1085.265	8.357935	1097.146	7.254706	1121.678	13.66576	1121.7	13.7	96.8	382					
19AR107 347	367.0924	50170.37	4.920767	12.88904	0.946888	1.662909	1.563816	0.156071	1.211814	0.774909	934.9058	10.54612	994.4857	9.916113	1129.199	19.7079	1129.2	19.7	82.8	303					
19AR107 21	196.41	21736.14	1.697503	12.80638	0.913109	1.956168	1.14878	0.18325	0.656022	0.571059	1084.703	6.549426	1100.568	7.718842	1132.932	18.77429	1132.9	18.8	95.7	17					
19AR107 69	119.1836	10520.79	1.515208	12.6396	1.569104	2.070313	1.83998	0.193797	0.753609	0.409574	1141.91	7.88643	1139.036	12.59849	1134.412	33.41233	1134.4	33.4	100.7	55					
19AR107 589	198.803	19101.62	2.062343	12.74709	0.870023	2.067341	1.144785	0.193199	0.700431	0.611845	1138.682	7.310993	1138.052	7.834525	1137.736	17.99559	1137.7	18.0	100.1	516					
19AR107 366	258.0641	19857.62	2.143653	12.7221	0.764933	2.069029	1.339094	0.193271	0.681027	0.508573	1139.072	7.110672	1138.611	9.166811	1138.617	22.91567	1138.6	22.9	100.0	322					
19AR107 146	149.9496	16344.35	2.167163	12.68409	1.075862	2.109564	1.393102	0.196366	0.826853	0.593533	1155.767	8.748791	1151.934	9.596636	1145.612	22.26805	1145.6	22.3	100.9	120					
19AR107 254	346.7371	35762.08	1.985999	12.72719	0.724382	2.049576	0.98519	0.1907	0.645457	0.655159	1125.164	6.663972	1132.155	6.723276	1146.453	14.76989	1146.5	14.8	98.1	219					
19AR107 242	473.7427	38179.23	8.24854	12.65207	0.658041	2.059427	1.032416	0.190839	0.70351	0.681421	1125.918	7.267795	1135.429	7.056641	1154.503	14.98077	1154.5	15.0	97.5	209					
19AR107 99	128.0121	19094.39	2.299729	12.66437	1.072227	2.062456	1.464543	0.190487	0.851745	0.581577	1124.013	8.785543	1136.434	10.01523	1161.078	23.62135	1161.1	23.6	96.8	82					
19AR107 44	17.07662	2681.871	0.445644	11.88268	6.781959	2.163875	7.474891	0.199712	1.342741	0.179633	1173.772	14.40914	1169.516	51.95489	1162.488	145.9442	1162.5	145.9	101.0	38					
19AR107 192	405.6009	9590.601	2.033582	12.48492	0.642212	2.164955	1.09043	0.199678	0.814727	0.747161	1173.589	8.741697	1169.862	7.573858	1163.816	14.36272	1163.8	14.4	100.8	162					
19AR107 269	77.63729	7886.069	1.476693	12.44176	2.054716	2.151397	2.830976	0.197666	0.809337	0.285886	1162.768	8.610799	1165.503	19.62627	1171.469	53.69289	1171.5	53.7	99.3	233					
19AR107 348	328.1293	18494.46	2.487108	12.47266	0.966837	2.15298	1.612507	0.1967	1.018415	0.631573	1157.569	10.79103	1166.013	11.18067	1182.609	24.72794	1182.6	24.7	97.9	304					
19AR107 401	245.1444	18502.62	2.492821	12.44727	1.017986	1.958502	1.597732	0.178872	1.206491	0.755127	1060.81	11.80099	1101.369	10.73995	1183.275	20.6994	1183.3	20.7	89.7	350					
19AR107 424	142.513	15903.95	1.489918	12.44215	1.10956	2.211838	1.446716	0.201752	0.694539	0.48008	1184.727	7.516549	1184.793	10.11643	1185.791	25.07011	1185.8	25.1	99.9	373					
19AR107 131	114.9382	12705.17	1.614092	12.38965	1.451458	1.957603	2.186988	0.178315	0.923419	0.422233	1057.762	9.008315	1101.06	14.69912	1188.53	39.14523	1188.5	39.1	89.0	109					
19AR107 557	51.48909	7604.186	2.109108	12.21876	2.088762	2.195743	2.472077	0.199373	0.765881	0.309813	1171.95	8.207136	1179.692	17.24818	1194.779	46.35789	1194.8	46.4	98.1	487					
19AR107 117	107.7687	12230.54	3.195211	12.32757	1.069339	2.195681	1.855733	0.198933	1.112583	0.599538	1169.585	11.90044	1179.672	12.94717	1199.091	29.28445	1199.1	29.3	97.5	97					
19AR107 276	357.424	39478.57	2.915139	12.4447	0.687713	2.231655	0.985744	0.201913	0.696169	0.706238	1185.587	7.539178	1191.038	6.911983	1201.822	13.75425	1201.8	13.8	98.6	240					
19AR107 568	427.3131	14630.41	2.830121	12.29653	0.692102	2.276821	1.341357	0.205943	0.606234	0.451956	1207.166	6.673886	1205.131	9.463746	1202.342	23.56215	1202.3	23.6	100.4	497					
19AR107 561	69.18539	10318.15	2.314682	12.16102	1.29924	2.415539	1.780954	0.216956	0.692115	0.38862	1265.772	7.954153	1247.231	12.7897	1216.233	32.25818	1216.2	32.3	104.1	491					
19AR107 584	134.0047	18198.49	2.124696	12.19501	1.175458	2.326226	1.391783	0.208024	0.709171	0.509541	1218.283	7.872401	1220.326	9.883602	1224.777	23.53083	1224.8	23.5	99.5	511					
19AR107 132	160.1415	11901.51	2.828348	12.0433	0.924936	2.259332	1.34594	0.200142	0.928684	0.689989	1176.084	9.983725	1199.698	9.473701	1243.336	19.1041	1243.3	19.1	94.6	110					
19AR107 588	267.8375	29413.51	3.330018	11.98753	0.769379	2.291598	1.391108	0.200644	1.034573	0.743705	1178.778	11.1453	1209.7	9.834128	1266.151	18.14422	1266.2	18.1	93.1	515					
19AR107 583	219.5072	26885.66	2.385176	11.78813	0.844266	2.304246	1.377301	0.198525	1.047778	0.760747	1167.389	11.18807	1213.594	9.752783	1297.565	17.37905	1297.6	17.4	90.0	510					
19AR107 260	235.3036	28711.18	2.731997	11.73323	0.812608	2.611489	1.135005	0.223022	0.751111	0.661769	1297.825	8.829507	1303.874	8.333725	1314.667	16.50337	1314.7	16.5	98.7	225					
19AR107 379	239.2223	42987.37	1.76442	11.46903	0.76183	2.639483	1.072893	0.220784	0.683789	0.637332	1286.017	7.972039	1311.714	7.900854	1354.793	15.94531	1354.8	15.9	94.9	334					
19AR107 586	210.3854	29320.51	1.183998	11.42946	0.715265	2.721767	1.035568	0.227201	0.662576	0.639819	1319.815	7.907683	1334.415	7.689864	1358.738	15.33987	1358.7	15.3	97.1	513					
19AR107 174	94.26129	8530.325	1.387733	10.93245	1.301166	3.038041	1.79479	0.246162	0.720698	0.40155	1418.652	9.177366	1417.231	13.71175	1415.909	31.43515	1415.9	31.4	100.2	146					
19AR107 253	66.20135	9417.48	0.673043	10.60994	1.030825	3.054017	1.906429	0.239438	0.750769	0.393809	1383.774	9.349596	1421.24	14.58365	1478.634	33.23073	1478.6	33.2	93.6	218					
19AR107 447	9.446677	1364.855	1.499808	9.05338	6.482252	4.036334	9.145943	0.291255	1.605887	0.175585	1647.796	23.3505	1641.548	74.5608	1634.347	167.6061	1634.3	167.6	100.8	393					
19AR107 210	64.68352	14276.93	2.184796	8.87535	1.030335	4.675888	1.342158	0.303974	0.649675	0.484052	1710.985	9.762976	1762.935	11.22746	1825.831	21.30045	1825.8	21.3	93.7	180					
19AR107 423	156.3936	25480.26	2.617318	8.924275	0.747412	4.663815	1.309044	0.302367	1.040753	0.795048	1703.038	15.57643	1760.773	10.94544	1830.752	14.39099	1830.8	14.4	93.0	372					
19AR107 165	57.96044	13133.93	0.903279	8.812813	1.355553	5.132154	1.568027	0.33208	0.768889	0.490354	1848.455	12.35647	1841.444	13.32585	1834.303	24.75823	1834.3	24.8	100.8	137					
19AR107 272	165.8192	22343.95	1.423216	8.879203	0.64639	4.749289	1.459786	0.306111	1.262472	0.864833	1721.541	19.07392	1775.982	12.24488	1841.361	13.2662	1841.4	13.3	93.5	236					
19AR107 93	300.4263	38336.21	2.465609	8.842006	0.738033	4.831769	1.823834	0.311221	1.661038	0.91074	1746.714	25.4152	1790.446	15.34455	1842.558	13.6319	1842.6	13.6	94.8	76					
19AR107 120	44.9579	7877.401	1.010215	8.682453	1.359601	5.209153	1.598094	0.334027	0.724369	0.45327	1857.873	11.69219	1854.114	13.61423	1850.673	25.75863	1850.7	25.8	100.4	100					
19AR107 35	92.25156	15480.41	0.580293	8.697515	0.717266	5.146295	1.021042	0.327736	0.665751	0.652031	1827.399	10.59359	1843.783	8.680915	1863.091	13.97622	1863.1	14.0	98.1	29					
19AR107 360	343.3256	23495.51	0.605402	8.663205	0.645389	5.200274	0.942469	0.329534	0.680808	0.722367	1836.123	10.87788	1852.661	8.026409	1872.048	11.75493	1872.0	11.8	98.1	316					
19AR107 339	103.4064	17701.02	0.627976	8.646002	0.82279	4.543554	1.308425	0.286902	0.948094	0.724607	1626.031	13.62569	1738.981	10.88937	1878.423	16.24966	1878.4	16.2	86.6	295					
19AR107 178	247.7484	55510	3.909186	8.662052	0.696071	5.261249	0.980022	0.332216	0.680202	0.694068	1849.114	10.93459	1862.598	8.36187	1878.45	12.71355	1878.4	12.7	98.4	150					
19AR107 15	231.1683	77965.86	1.92802	8.687568	0.638912	5.230325	0.985367	0.330104	0.740513	0.751509	1838.888	11.84723	1857.571	8.399546	1879.318	11.71279	1879.3	11.7	97.8	11					
19AR107 555	73.70534	13168.77	1.711428	8.552423	1.043527	5.390417	1.377904	0.337865	0.756403	0.548952	1876.389	12.31409	1883.332	11.80216	1891.762	20.72269	1891.8	20.7	99.2	486					
19AR107 444	96.2913	23921.81	1.20241	8.587734	0.943729	5.361402	1.179412	0.33435	0.67074	0.568707	1859.431	10.83438	1878.711	10.09335	1900.862	17.43554	1900.9	17.4	97.8	390					
19AR107 578	410.1071	6341.042	4.392496	8.341447	0.588618	4.528347	0.913703	0.279317	0.687265	0.752176	1587.922	9.673019	1736.192	7.599542	1920.562	10.79497	1920.6	10.8	82.7	505					
19AR107 522	75.35608	15233.96	1.735077	8.338851	0.967698	5.713747	1.308654	0.349316	0.759575	0.580425	1931.33	12.67635	1933.449	11.30912	1936.483	19.0721	1936.5	19.1	99.7	457					
19AR107 86	141.2209	30572.94	1.133442	8.274152	0.778776	5.607356	1.117486	0.338396	0.730591	0.653781	1878.947	11.90785	1917.23	9.629764	1959.655	15.09165	1959.7	15.1	95.9	69					
19AR107 409	255.8128	25810.34	1.310716	8.260647	0.637301	5.655595	0.972962	0.340191	0.668762	0.687346	1887.587	10.94324	1924.616	8.395127	1965.497	12.60372	1965.5	12.6	96.0	358					
19AR107 498	116.4815	26947.38	3.494298	8.237561	0.836433	5.509026	1.225752	0.330626	0.845164	0.689507	1841.417	13.53759	1902.005	10.53433	1969.526	15.82669	1969.5	15.8	93.5	437					
19AR107 427	204.8099	38813.1	2.486682	8.193137	0.596112	5.686477	0.887768	0.338739	0.643969	0.725379	1880.6	10.50395	1929.316	7.666264	1982.821	10.87845	1982.8	10.9	94.8	375					
19AR107 68	104.4273	19632.29	1.89356	8.028783	0.772549	5.900858	1.10504	0.346914	0.683648	0.618664	1919.843	11.35096	1961.36	9.59473	2006.213	15.41117	2006.2	15.4	95.7	54					
19AR107 552	84.65333	15856.06	0.825553	7.67417	0.797408	6.738403	1.076565	0.3784	0.672119	0.624318	2068.807	11.89435	2077.672	9.518937	2087.221	14.79056	2087.2	14.8	99.1	483					
19AR107 91	43.75805	8291.246	1.390847	7.629278	0.949327	6.760376	1.58647	0.379143	0.80405	0.506817	2072.279	14.24937	2080.551	14.03387	2089.499	24.04634	2089.5	24.0	99.2	74					
19AR107 227	283.0209	75545.3	1.647362	7.612257	0.481116	6.803034	0.78117	0.377348	0.611268	0.782503	2063.882	10.79564	2086.117	6.915463	2108.885	8.532371	2108.9	8.5	97.9	196					
19AR107 411	336.3426	21008.62	1.466542	7.577427	0.649274	6.321519	1.61891	0.348965	1.474446	0.910765	1929.655	24.58842	2021.443	14.19388	2117.274	11.71667	2117.3	11.7	91.1	360					
19AR107 597	114.9653	20742	1.238444	7.543503	0.660614	6.60684	0.89265	0.36417	0.563266	0.631005	2001.911	9.693223	2060.261	7.872436	2119.897	12.13402	2119.9	12.1	94.4	523					
19AR107 479	83.2258	18281.14	2.223231	7.454096	0.78124	6.811882	1.232679	0.370861	0.942699	0.764756	2033.45	16.4403	2087.268	10.91461	2141.528	13.88346	2141.5	13.9	95.0	419					
19AR107 37	106.8597	26817.64	1.884312	7.012771	0.794879	7.781494	1.177027	0.397887	0.824683	0.700649	2159.301	15.1319	2206.068	10.59075	2250.544	14.50613	2250.5	14.5	95.9	31					
19AR107 579	155.7252	33210.26	1.012032	6.350385	0.597398	9.248398	0.975482	0.427766	0.758063	0.777116	2295.639	14.64109	2362.92	8.938628	2422.219	10.41429	2422.2	10.4	94.8	506					
19AR107 136	84.60082	21137.78	1.137494	5.978423	0.624824	10.7639	1.010457	0.469012	0.757219	0.749382	2479.229	15.58473	2502.955	9.388119	2522.976	11.23841	2523.0	11.2	98.3	114					
19AR107 402	411.417	109925.5	1.874626	5.452563	0.616548	12.42082	1.006548	0.491786	0.79448	0.789312	2578.4	16.88387	2636.754	9.45906	2682.562	10.21884	2682.6	10.2	96.1	351					
19AR107 416	275.9105	69593.93	1.534233	5.218774	0.509293	12.78169	0.803035	0.483298	0.619567	0.771531	2541.616	13.01349	2663.696	7.562378	2758.448	8.390879	2758.4	8.4	92.1	365					
**19AR108**																					-26.94419	65.76612	590	5.9	
_19AR108_Spot_ 386	1292.76	28.59506	0.22234	4.274366	16.83268	-0.02079	38.18553	0.000165	5.556032	0.145501	1.065823	0.059213	NA	-8.23347	NA	NA	1.1	0.1		323					0.172
_19AR108_Spot_ 149	2473.885	106.9818	0.478455	8.558199	13.16378	-0.00156	60.27293	0.000316	3.023286	0.05016	2.034086	0.061487	NA	-0.9565	NA	NA	2.0	0.1		127					
_19AR108_Spot_ 23	1808.855	248.4669	3.044264	8.109665	11.59232	0.002876	60.39601	0.00032	6.161266	0.102014	2.062124	0.127033	2.915755	1.758475	781.3536	NA	2.1	0.1	0.3	19					
_19AR108_Spot_ 533	2021.981	318.3516	2.871669	10.52089	25.17818	0.002276	71.6434	0.000338	3.38268	0.047216	2.178021	0.073663	2.308023	1.65167	141.2288	NA	2.2	0.1	1.5	434					
_19AR108_Spot_ 271	1292.86	319.1432	2.133559	5.283864	17.04411	0.007293	19.8636	0.000355	6.688388	0.336716	2.285792	0.152856	7.378212	1.460268	2336.857	323.2218	2.3	0.2	0.1	225					
_19AR108_Spot_ 182	1632.146	396.8714	3.404001	9.36195	8.56199	0.00346	34.06451	0.000355	6.549671	0.192273	2.287941	0.149826	3.506676	1.192472	949.1429	703.4395	2.3	0.1	0.2	150					
_19AR108_Spot_ 148	1032.297	250.005	2.602253	6.45466	25.86788	0.00488	38.96415	0.000356	7.46674	0.191631	2.291618	0.171079	4.943054	1.921341	1616.395	740.9059	2.3	0.2	0.1	126					
_19AR108_Spot_ 38	1300.202	253.3282	4.192326	7.87191	28.50096	0.003472	53.99296	0.000357	5.756213	0.10661	2.303019	0.132543	3.519338	1.896908	943.0635	NA	2.3	0.1	0.2	31					
_19AR108_Spot_ 548	1804.166	332.1337	3.034465	7.711691	8.580007	0.004336	18.25476	0.000359	3.101847	0.16992	2.310901	0.071668	4.393155	0.800228	1376.938	348.6593	2.3	0.1	0.2	449					
_19AR108_Spot_ 291	2397.992	495.8921	5.402484	11.30648	13.75079	0.003039	21.03355	0.000374	5.344452	0.254092	2.407697	0.128654	3.081185	0.6471	568.2088	447.0102	2.4	0.1	0.4	243					
_19AR108_Spot_ 540	2319.699	488.8164	5.537745	11.35473	9.419417	0.003021	47.93042	0.000375	2.910651	0.060727	2.415776	0.070302	3.063332	1.466056	548.2145	NA	2.4	0.1	0.4	441					
_19AR108_Spot_ 190	3220.012	760.1318	5.570178	10.98979	10.70426	0.003766	16.80539	0.000378	2.258014	0.134362	2.436563	0.055008	3.816401	0.640158	993.6695	340.7577	2.4	0.1	0.2	158					
_19AR108_Spot_ 99	1925.082	428.0468	2.998011	9.217054	15.1691	0.003976	28.51182	0.000382	4.532691	0.158976	2.46003	0.111484	4.029436	1.146589	1084.226	575.6376	2.5	0.1	0.2	86					
_19AR108_Spot_ 100	2774.756	651.8228	2.766046	11.68564	12.5767	0.00336	31.76423	0.000383	2.648542	0.083381	2.470741	0.065426	3.406201	1.080141	728.4114	687.279	2.5	0.1	0.3	87					
_19AR108_Spot_ 211	2351.93	457.4115	3.194632	11.58419	15.02088	0.002893	43.19045	0.000386	3.931613	0.09103	2.490433	0.097895	2.933526	1.265175	384.706	NA	2.5	0.1	0.6	176					
_19AR108_Spot_ 368	2600.655	736.3464	4.320342	11.01439	12.16567	0.003822	14.03265	0.000389	2.95727	0.210742	2.508072	0.074156	3.873052	0.542457	964.8411	281.3382	2.5	0.1	0.3	308					
_19AR108_Spot_ 529	1844.899	367.2099	3.512197	9.349361	15.3897	0.003829	50.84402	0.000409	5.352663	0.105276	2.635307	0.14103	3.880198	1.969086	866.8129	NA	2.6	0.1	0.3	430					
_19AR108_Spot_ 208	2298.365	356.5491	0.509658	12.78653	12.75735	0.002662	42.75074	0.000534	1.989311	0.046533	3.440114	0.068416	2.699397	1.15248	NA	NA	3.4	0.1		173					
_19AR108_Spot_ 421	599.1624	223.6281	0.449515	4.824652	45.62016	0.011602	50.00819	0.000562	7.480422	0.149584	3.619051	0.270644	11.71298	5.823861	2344.898	911.1787	3.6	0.3	0.2	353					
_19AR108_Spot_ 96	907.2503	213.0797	2.852754	7.513115	25.24361	0.005281	47.89541	0.000584	5.44642	0.113715	3.766611	0.205086	5.34808	2.554756	792.3468	NA	3.8	0.2	0.5	84					
_19AR108_Spot_ 184	1318.124	202.4571	1.942716	7.687974	19.56992	0.00531	39.16699	0.000662	4.364578	0.111435	4.266964	0.186173	5.377657	2.100702	536.9765	NA	4.3	0.2	0.8	152					
_19AR108_Spot_ 404	412.2005	246.4588	1.383564	5.815714	27.47357	0.013578	38.52485	0.00084	7.55181	0.196024	5.410455	0.408416	13.69418	5.240292	1916.009	705.2582	5.4	0.4	0.3	339					
_19AR108_Spot_ 584	1282.173	719.1763	3.142789	10.29259	9.296369	0.009261	22.05873	0.000869	3.879605	0.175876	5.599324	0.217137	9.36068	2.055361	1129.799	437.5324	5.6	0.2	0.5	479					
_19AR108_Spot_ 542	233.158	123.6511	2.334556	4.159825	38.37082	0.016271	49.7169	0.000877	9.044305	0.181916	5.653773	0.51112	16.38874	8.082739	2158.248	916.4214	5.7	0.5	0.3	443					
_19AR108_Spot_ 37	748.6451	307.1084	2.634688	7.971361	16.86116	0.00996	25.19413	0.000913	4.268247	0.169414	5.880354	0.250874	10.06359	2.522917	1176.957	498.7162	5.9	0.3	0.5	30					
_19AR108_Spot_ 137	1924.181	780.6014	2.295992	16.74265	7.466866	0.005172	42.27169	0.000925	1.991054	0.047101	5.960445	0.118621	5.238332	2.208633	NA	NA	6.0	0.1		117					
_19AR108_Spot_ 325	881.0198	544.6005	1.047317	9.225119	17.35812	0.010662	23.27773	0.000939	3.671589	0.15773	6.052139	0.222105	10.76871	2.493472	1254.144	455.5879	6.1	0.2	0.5	271					
_19AR108_Spot_ 597	1707.39	233.4245	0.509343	11.36902	10.44404	0.002967	62.44491	0.000951	1.829465	0.029297	6.124518	0.111993	3.008197	1.875688	NA	NA	6.1	0.1		491					
_19AR108_Spot_ 412	177.4427	106.7433	1.347331	5.878093	22.38508	0.004119	292.673	0.001056	9.308549	0.031805	6.802514	0.632881	4.17412	12.19203	NA	NA	6.8	0.6		345					
_19AR108_Spot_ 348	454.3011	327.4737	4.113589	7.407408	29.33699	0.013572	45.90946	0.001065	5.264432	0.11467	6.863352	0.361124	13.68772	6.241872	1476.468	916.2804	6.9	0.4	0.5	292					
_19AR108_Spot_ 263	275.97	197.0405	1.222902	4.907503	18.53004	0.021865	24.89349	0.001153	8.913936	0.358083	7.431113	0.662023	21.96186	5.408425	2196.511	409.8598	7.4	0.7	0.3	217					
_19AR108_Spot_ 301	469.5258	321.2477	2.904603	7.901425	13.76568	0.013618	24.65535	0.001197	4.396165	0.178305	7.710946	0.338783	13.73373	3.363315	1258.707	481.263	7.7	0.3	0.6	252					

_19AR108_Spot_ 197	300.7185	257.0012	2.07568	5.535623	46.28376	0.021856	53.47803	0.001226	6.856829	0.128218	7.896022	0.541085	21.95353	11.61481	2089.717	1016.237	7.9	0.5	0.4	164
_19AR108_Spot_ 389	751.0433	686.3846	1.571842	10.81407	10.63319	0.016351	12.57147	0.001657	2.482433	0.197466	10.67343	0.264741	16.46817	2.053596	974.4466	252.1656	10.7	0.3	1.1	325
_19AR108_Spot_ 9	427.9855	392.1405	1.381679	8.740473	17.87477	0.018861	37.11978	0.001751	4.182291	0.11267	11.27601	0.471183	18.97313	6.977504	1151.216	758.3725	11.3	0.5	1.0	7
_19AR108_Spot_ 507	1059.6	846.961	2.06374	17.15385	6.184957	0.010425	17.53627	0.001853	1.461511	0.083342	11.93136	0.174217	10.5305	1.837116	NA	NA	11.9	0.2		412
_19AR108_Spot_ 587	449.6894	541.2453	1.457321	9.004513	16.05578	0.021747	18.21763	0.001857	3.084422	0.16931	11.95685	0.368458	21.84488	3.937137	1315.374	350.9658	12.0	0.4	0.9	482
_19AR108_Spot_ 339	519.8402	534.0147	1.146502	11.98967	11.3865	0.014348	28.65634	0.001858	3.38369	0.118078	11.9665	0.404534	14.4656	4.115945	453.6321	NA	12.0	0.4	2.6	284
_19AR108_Spot_ 438	524.8271	645.156	1.177805	10.88673	9.833066	0.017948	17.49938	0.001868	3.393928	0.193946	12.03333	0.408022	18.06205	3.132809	919.4867	355.4465	12.0	0.4	1.3	367
_19AR108_Spot_ 496	485.1109	457.9046	1.306117	10.03107	19.80481	0.017734	31.65608	0.001871	2.665913	0.084215	12.04728	0.32087	17.84905	5.600995	892.4272	667.4125	12.0	0.3	1.3	408
_19AR108_Spot_ 349	449.7287	513.4983	1.488742	11.4563	12.58979	0.015268	22.04717	0.001881	4.504956	0.204333	12.11112	0.545089	15.38536	3.366479	563.7335	475.1877	12.1	0.5	2.1	293
_19AR108_Spot_ 431	462.5181	766.0709	1.262486	10.28928	13.51273	0.020438	25.95815	0.001884	3.309016	0.127475	12.13287	0.401101	20.54334	5.279135	1165.041	518.6106	12.1	0.4	1.0	361
_19AR108_Spot_ 169	555.5385	656.0533	0.755796	10.5408	11.74943	0.019167	17.59656	0.001901	4.397842	0.249926	12.24166	0.537858	19.2772	3.360139	1018.475	347.4527	12.2	0.5	1.2	138
_19AR108_Spot_ 338	423.9818	367.3925	1.402125	9.575234	11.48788	0.01713	56.62982	0.001909	4.103939	0.07247	12.29679	0.504172	17.24629	9.684363	777.4433	NA	12.3	0.5	1.6	283
_19AR108_Spot_ 435	812.4574	936.4754	1.154783	13.57304	9.887233	0.015329	25.41424	0.001912	2.224945	0.087547	12.31213	0.273677	15.44625	3.895858	536.4497	562.2665	12.3	0.3	2.3	364
_19AR108_Spot_ 393	214.1073	263.3091	1.51964	6.343384	19.8928	0.02785	26.49299	0.001913	5.927513	0.223739	12.31738	0.729417	27.89151	7.288856	1725.739	482.5468	12.3	0.7	0.7	329
_19AR108_Spot_ 129	621.613	680.4672	0.943238	10.3268	16.89702	0.02076	23.10855	0.001988	2.385802	0.103243	12.80188	0.305124	20.86311	4.772003	1089.085	466.5521	12.8	0.3	1.2	110
_19AR108_Spot_ 544	1427.787	1703.841	1.350616	16.21393	7.871301	0.014794	15.40292	0.002017	1.089027	0.070703	12.99075	0.14133	14.91177	2.280067	337.9104	349.8484	13.0	0.1	3.8	445
_19AR108_Spot_ 162	281.6399	307.9105	0.494741	7.873818	13.42976	0.022858	27.63186	0.002049	4.755094	0.172087	13.19719	0.626897	22.94845	6.270043	1219.79	545.0997	13.2	0.6	1.1	133
_19AR108_Spot_ 241	1026.869	1035.675	0.989703	13.63815	7.693487	0.018318	22.14917	0.002237	1.313093	0.059284	14.40432	0.188931	18.43181	4.045683	582.4072	485.5583	14.4	0.2	2.5	201
_19AR108_Spot_ 311	921.0695	1054.908	4.771727	12.96509	3.599439	0.019979	8.431479	0.00229	2.455779	0.291263	14.74724	0.361746	20.08613	1.676919	718.1266	171.4927	14.7	0.4	2.1	260
_19AR108_Spot_ 161	259.0323	310.2801	1.405041	6.805669	39.08438	0.034251	41.07559	0.002425	3.0481	0.074207	15.61275	0.475316	34.19555	13.81299	1669.648	793.4371	15.6	0.5	0.9	132
_19AR108_Spot_ 238	327.6831	689.5901	0.831087	10.87719	9.487905	0.025243	14.43169	0.002569	2.797434	0.19384	16.53869	0.462066	25.31288	3.607932	966.019	290.4136	16.5	0.5	1.7	198
_19AR108_Spot_ 39	329.2122	389.1469	1.485252	9.058294	11.57925	0.026279	35.39321	0.00259	5.001908	0.141324	16.67564	0.833022	26.33892	9.202572	1030.951	730.485	16.7	0.8	1.6	32
_19AR108_Spot_ 577	216.628	458.7529	1.115756	8.671578	5.988906	0.030484	21.90549	0.002612	6.99068	0.319129	16.81545	1.173983	30.49008	6.579812	1308.272	407.321	16.8	1.2	1.3	473
_19AR108_Spot_ 143	377.0563	650.4288	1.583828	8.841712	10.76873	0.033673	13.45052	0.002671	2.33447	0.17356	17.19262	0.400822	33.62746	4.449018	1456.745	253.0033	17.2	0.4	1.2	122
_19AR108_Spot_ 434	625.3188	1327.208	2.964257	14.93443	10.16403	0.02122	15.16766	0.002749	1.618421	0.106702	17.69557	0.285996	21.32074	3.200152	452.8433	336.4954	17.7	0.3	3.9	363
_19AR108_Spot_ 546	336.8835	847.2469	2.49495	11.62505	12.70369	0.026342	18.12236	0.002758	3.046696	0.168118	17.75274	0.540128	26.40064	4.72279	907.9111	370.7768	17.8	0.5	2.0	447
_19AR108_Spot_ 322	103.9746	373.1222	1.410889	10.15301	21.09028	0.043473	67.38564	0.005308	3.599147	0.053411	34.12569	1.224988	43.20956	28.51369	582.8518	NA	34.1	1.2	5.9	269
_19AR108_Spot_ 427	288.766	1252.115	3.346281	13.2882	18.03962	0.047942	24.06121	0.005455	2.261723	0.093999	35.0672	0.79097	47.54865	11.17749	734.1452	514.1798	35.1	0.8	4.8	357
_19AR108_Spot_ 240	394.3712	1295.707	1.513503	16.18196	6.027256	0.038058	8.290866	0.005461	2.317585	0.279535	35.10875	0.811463	37.92602	3.086406	221.0344	184.381	35.1	0.8	15.9	200
_19AR108_Spot_ 140	317.8186	1871.516	1.920712	13.78326	11.48231	0.048833	14.52757	0.005475	1.448828	0.09973	35.19981	0.508595	48.41169	6.86812	765.0114	306.061	35.2	0.5	4.6	119
_19AR108_Spot_ 445	1302.012	3977.567	0.287168	18.57625	3.937112	0.039995	5.496236	0.005783	0.853505	0.155289	37.17298	0.31636	39.8187	2.146176	203.3064	126.1039	37.2	0.3	18.3	374
_19AR108_Spot_ 453	101.0671	468.4683	1.937541	10.08092	16.70795	0.058476	31.08793	0.006192	6.503496	0.209197	39.79542	2.580122	57.704	17.44048	884.2927	642.8482	39.8	2.6	4.5	382
_19AR108_Spot_ 285	305.6209	1444.678	1.176155	18.31365	4.418551	0.059924	9.899272	0.009798	1.425524	0.144003	62.8546	0.891654	59.09228	5.682801	NA	NA	62.9	0.9		237
_19AR108_Spot_ 159	418.4614	2650.083	1.488766	19.44534	3.028291	0.083516	5.49622	0.013238	0.834191	0.151775	84.77926	0.702591	81.44479	4.301588	NA	NA	84.8	0.7		130
_19AR108_Spot_ 187	283.9902	2153.097	2.675911	17.14779	2.551451	0.122542	6.81223	0.017225	0.969289	0.142287	110.0947	1.058075	117.3739	7.551084	268.5509	154.7823	110.1	1.1	41.0	155
_19AR108_Spot_ 135	48.02056	827.6081	1.154591	11.09768	11.30483	0.243362	14.78759	0.024273	2.77552	0.187693	154.606	4.240072	221.1696	29.39704	1006.888	296.1552	154.6	4.2	15.4	115
_19AR108_Spot_ 227	70.21708	1267.945	1.828922	15.93148	7.25717	0.276261	21.24858	0.0391	1.376417	0.064777	247.2505	3.338761	247.6874	46.73548	252.8513	NA	247.3	3.3	97.8	189
_19AR108_Spot_ 422	64.30613	1768.563	2.059022	16.15523	6.532364	0.318592	15.80203	0.043057	1.157754	0.073266	271.7537	3.080847	280.8187	38.78626	357.9704	357.7119	271.8	3.1	75.9	354
_19AR108_Spot_ 326	443.0114	8040.409	2.05202	19.12015	1.481661	0.305147	2.844113	0.04392	0.808311	0.284205	277.0877	2.192276	270.4122	6.752001	214.0382	63.19476	277.1	2.2	129.5	272
_19AR108_Spot_ 535	375.9716	2919	2.438229	18.85542	1.975942	0.292036	9.864234	0.043956	0.991552	0.10052	277.3068	2.69134	260.1609	22.64265	109.4043	NA	277.3	2.7	253.5	436
_19AR108_Spot_ 150	97.74306	3702.178	2.226268	16.75057	3.847072	0.365317	6.431046	0.047608	0.936583	0.145635	299.8181	2.743747	316.1764	17.47392	439.5058	141.684	299.8	2.7	68.2	128
_19AR108_Spot_ 126	437.9612	12364.72	2.195055	19.02892	0.832974	0.338011	1.416389	0.047763	0.640731	0.452369	300.7721	1.882873	295.6638	3.633172	256.5307	29.02343	300.8	1.9	117.2	107
_19AR108_Spot_ 196	592.7822	20390.14	2.336383	19.09291	0.860437	0.355121	1.32983	0.049768	0.73349	0.551567	313.0959	2.241649	308.5652	3.538554	275.4382	25.43296	313.1	2.2	113.7	163
_19AR108_Spot_ 265	74.36317	1935.1	1.457556	16.11329	5.453596	0.374938	8.394617	0.049819	0.920983	0.109711	313.4081	2.817393	323.3065	23.24785	396.1799	187.3561	313.4	2.8	79.1	219
_19AR108_Spot_ 290	263.4355	9936.719	2.101782	18.57456	2.063297	0.360368	2.334014	0.049977	0.693026	0.296925	314.3782	2.12645	312.4892	6.278104	299.3922	50.84224	314.4	2.1	105.0	242
_19AR108_Spot_ 457	143.7228	5826.256	1.654179	17.26194	2.728903	0.401332	4.068086	0.052587	0.77606	0.190768	330.3826	2.499372	342.6137	11.83048	427.385	89.05892	330.4	2.5	77.3	385
_19AR108_Spot_ 218	234.5515	3663.804	0.729927	17.34862	2.345997	0.48852	4.191625	0.065913	0.659023	0.157224	411.4829	2.627031	403.9012	13.96906	361.727	93.42943	411.5	2.6	113.8	182
_19AR108_Spot_ 50	1512.694	10950.06	10.92225	16.96422	0.60811	0.53217	2.387829	0.066849	0.738421	0.309244	417.1455	2.982743	433.2488	8.421442	520.7205	49.84293	417.1	3.0	80.1	42
_19AR108_Spot_ 392	311.2868	1112.095	1.977565	16.33879	1.535058	0.464841	5.657575	0.070299	1.265606	0.223701	437.9546	5.358694	387.619	18.23143	98.21782	NA	438.0	5.4	445.9	328
_19AR108_Spot_ 21	167.1961	9305.239	1.516956	17.60705	3.347039	0.567554	3.608827	0.074413	0.74658	0.206876	462.6877	3.333284	456.4316	13.268	426.0022	78.77378	462.7	3.3	108.6	17
_19AR108_Spot_ 526	74.03853	2618.937	2.452365	17.18009	9.755218	0.542641	16.02359	0.074529	8.64238	0.539354	463.3854	38.6425	440.1646	57.29255	321.49	307.778	463.4	38.6	144.1	427
_19AR108_Spot_ 471	83.81013	1375.746	3.781242	17.26209	3.751852	0.489242	8.359269	0.075062	0.806312	0.096457	466.5818	3.629184	404.3941	27.89113	64.00178	NA	466.6	3.6	729.0	396
_19AR108_Spot_ 429	259.3393	14713.21	7.931179	17.41308	1.575495	0.587207	2.243156	0.075437	0.841824	0.375285	468.8288	3.806612	469.0824	8.426687	471.2652	46.01742	468.8	3.8	99.5	359
_19AR108_Spot_ 144	430.8013	5177.884	3.26369	17.3108	0.916427	0.573251	5.215039	0.075798	0.792292	0.151924	470.9947	3.59859	460.1148	19.29685	407.1019	115.4146	471.0	3.6	115.7	123
_19AR108_Spot_ 430	141.3132	8945.976	1.274687	17.64642	2.039103	0.575581	2.292235	0.075824	0.715322	0.312063	471.1474	3.250009	461.6176	8.502859	415.4211	48.68292	471.1	3.3	113.4	360
_19AR108_Spot_ 469	43.45577	952.0524	2.451587	14.38894	8.458718	0.568022	29.25804	0.075986	1.002509	0.034264	472.1188	4.563872	456.7343	108.0245	381.0043	NA	472.1	4.6	123.9	394
_19AR108_Spot_ 510	212.1398	10253.18	3.949509	17.55734	1.72588	0.585861	2.65596	0.07631	1.133748	0.426869	474.0571	5.18175	468.2207	9.963102	440.6534	53.45202	474.1	5.2	107.6	415
_19AR108_Spot_ 516	214.1045	9078.114	1.276824	17.46679	1.946079	0.587475	2.226563	0.076407	0.784097	0.352156	474.6418	3.587944	469.2538	8.366755	443.9314	46.34862	474.6	3.6	106.9	420
_19AR108_Spot_ 71	336.4267	12777.53	13.03105	17.65837	1.075395	0.583596	1.79241	0.076547	0.851188	0.474885	475.4762	3.901545	466.77	6.707208	425.1208	35.19086	475.5	3.9	111.8	61
_19AR108_Spot_ 456	370.2409	17093.78	9.278789	17.56901	1.662507	0.592548	2.0054	0.076669	0.695044	0.346586	476.2125	3.190585	472.4937	7.576527	455.44	41.75735	476.2	3.2	104.6	384
_19AR108_Spot_ 527	281.3657	9390.64	5.585554	17.74682	8.645827	0.582051	12.42252	0.076775	8.628787	0.694608	476.8458	39.66155	465.7787	46.43901	412.5354	200.1479	476.8	39.7	115.6	428
_19AR108_Spot_ 116	292.8246	15375.45	36.73347	17.47039	1.643597	0.596481	2.034139	0.076818	0.825203	0.405677	477.1007	3.794887	474.9983	7.717059	465.8406	41.18736	477.1	3.8	102.4	100
_19AR108_Spot_ 491	81.59423	2908.175	2.887839	18.03915	2.805624	0.53787	10.14065	0.076846	0.841247	0.082958	477.27	3.869992	437.0189	36.0276	231.0997	233.8532	477.3	3.9	206.5	403
_19AR108_Spot_ 51	39.34741	2128.793	3.118133	15.48026	4.035806	0.613827	9.054822	0.0769	1.333067	0.147222	477.592	6.136501	485.9706	34.98408	526.6518	196.7032	477.6	6.1	90.7	43
_19AR108_Spot_ 405	187.5823	7465.854	1.828871	17.36074	2.815082	0.591255	3.043457	0.077062	0.622271	0.204462	478.565	2.87012	471.6688	11.48288	439.2019	66.31548	478.6	2.9	109.0	340
_19AR108_Spot_ 217	505.1746	10321.78	2.750508	17.66865	0.820688	0.587213	1.160505	0.077096	0.689625	0.594246	478.764	3.182053	469.086	4.359532	422.9531	20.83042	478.8	3.2	113.2	181
_19AR108_Spot_ 340	134.3756	6199.628	2.763349	17.48341	3.655786	0.584594	4.085135	0.07734	0.707956	0.173301	480.2243	3.276233	467.4093	15.30402	405.8997	90.09607	480.2	3.3	118.3	285
_19AR108_Spot_ 242	97.7553	4414.662	1.613352	17.39661	3.231681	0.579442	6.573369	0.077409	0.885723	0.134744	480.6385	4.102296	464.1029	24.49111	384.0439	146.4803	480.6	4.1	125.2	202
_19AR108_Spot_ 41	464.1278	19532.85	8.970529	17.48573	0.892282	0.602466	1.703792	0.077519	0.632382	0.371162	481.2993	2.932806	478.7976	6.504235	467.7949	35.04112	481.3	2.9	102.9	33
_19AR108_Spot_ 36	661.9062	40472.89	8.677438	17.50074	0.96744	0.606834	1.246246	0.077619	0.72829	0.584387	481.8935	3.381615	481.5616	4.778987	480.9217	22.35341	481.9	3.4	100.2	29
_19AR108_Spot_ 446	181.7289	8529.001	3.06222	17.53926	2.144418	0.593155	2.519996	0.077742	0.806032	0.319854	482.6307	3.748099	472.8802	9.526919	426.7904	53.25371	482.6	3.7	113.1	375
_19AR108_Spot_ 513	93.94601	4706.56	1.916912	17.21191	3.436034	0.590845	4.358457	0.077746	0.836034	0.191819	482.6551	3.8878	471.4072	16.4379	417.9666	95.57771	482.7	3.9	115.5	417
_19AR108_Spot_ 95	261.3668	12726.91	0.347009	17.38051	1.368732	0.606788	1.720292	0.077775	0.969267	0.563432	482.8256	4.508907	481.5322	6.596545	476.3442	31.44735	482.8	4.5	101.4	83
_19AR108_Spot_ 210	232.6267	4578.09	1.457944	17.45334	1.185147	0.581587	2.216283	0.077936	0.764686	0.345031	483.7932	3.564088	465.4806	8.275346	377.084	46.77991	483.8	3.6	128.3	175
_19AR108_Spot_ 134	172.7712	8598.278	4.608033	17.35473	1.860332	0.600125	2.311374	0.077972	0.944999	0.408847	484.0089	4.406389	477.3129	8.802354	446.2115	46.89168	484.0	4.4	108.5	114
_19AR108_Spot_ 47	155.9712	5510.702	2.670324	17.03192	1.657128	0.604206	4.290854	0.077994	0.805331	0.187685	484.1383	3.756104	479.8995	16.41105	460.6408	93.47779	484.1	3.8	105.1	39
_19AR108_Spot_ 97	239.4587	9406.756	8.235823	17.35511	0.958249	0.605205	2.625509	0.078014	0.694856	0.264656	484.2587	3.241619	480.5314	10.05147	463.7708	56.1126	484.3	3.2	104.4	85
_19AR108_Spot_ 537	208.9108	9569.969	12.25565	17.67645	2.01683	0.594849	2.451634	0.078054	0.656851	0.267924	484.4989	3.065782	473.9597	9.285063	424.2096	52.70658	484.5	3.1	114.2	438
_19AR108_Spot_ 341	194.6774	7086.322	2.394912	17.07285	1.709633	0.608148	2.691933	0.078073	0.732984	0.272289	484.6107	3.421887	482.3917	10.33695	472.8293	57.33546	484.6	3.4	102.5	286
_19AR108_Spot_ 327	94.76887	3846.857	1.860849	16.66709	4.546076	0.604127	5.889338	0.078075	1.252197	0.212621	484.6196	5.845902	479.8496	22.52461	458.085	127.7431	484.6	5.8	105.8	273
_19AR108_Spot_ 363	315.5804	9896.254	10.5771	17.6481	1.632909	0.593828	2.457575	0.078095	0.780594	0.317628	484.7407	3.645093	473.309	9.297536	419.2161	52.05082	484.7	3.6	115.6	304
_19AR108_Spot_ 302	239.4068	11378.45	3.482342	17.67978	1.586516	0.595193	2.007566	0.078242	0.70476	0.351052	485.6209	3.296731	474.1787	7.605935	420.1401	41.99598	485.6	3.3	115.6	253
_19AR108_Spot_ 22	267.5477	11201.69	5.752447	17.48709	1.755838	0.604021	2.009018	0.078265	0.70718	0.352003	485.7559	3.308936	479.782	7.681816	452.2763	41.75821	485.8	3.3	107.4	18
_19AR108_Spot_ 554	253.8137	14303.2	1.41011	17.6315	1.191228	0.603086	1.670659	0.078312	0.692819	0.414698	486.039	3.243559	479.1901	6.381837	447.4931	33.78364	486.0	3.2	108.6	453
_19AR108_Spot_ 105	132.2782	3958.361	1.747454	17.69723	2.548808	0.57134	3.344374	0.078332	0.830768	0.248408	486.1612	3.890335	458.8807	12.34783	325.4804	73.54236	486.2	3.9	149.4	92
_19AR108_Spot_ 186	152.3143	5652.398	0.826906	16.99658	1.54272	0.608989	2.661836	0.078354	0.678357	0.254845	486.2908	3.177433	482.9222	10.23016	467.9474	57.00264	486.3	3.2	103.9	154
_19AR108_Spot_ 489	90.20283	3145.667	0.737223	17.53144	2.77833	0.570888	8.295425	0.078454	0.973331	0.117334	486.887	4.564481	458.5889	30.62006	320.1865	187.4615	486.9	4.6	152.1	401
_19AR108_Spot_ 266	428.1963	18107.91	7.974781	17.6574	1.0046	0.604775	1.722701	0.078468	0.803264	0.466282	486.9706	3.767565	480.2598	6.592129	449.2917	33.87673	487.0	3.8	108.4	220
_19AR108_Spot_ 428	254.0024	13646.1	3.043558	17.50702	1.346539	0.607028	1.935291	0.078501	0.697339	0.360328	487.1679	3.272021	481.6838	7.422821	456.6289	40.07024	487.2	3.3	106.7	358
_19AR108_Spot_ 362	293.6386	12026.27	10.17731	17.72181	1.224424	0.598126	1.738644	0.07853	0.772385	0.444245	487.3423	3.625393	476.0441	6.607368	422.9146	34.78521	487.3	3.6	115.2	303
_19AR108_Spot_ 560	194.1798	7889.748	22.27717	17.65641	1.959161	0.594354	2.927185	0.078567	0.802513	0.274159	487.5646	3.768465	473.6442	11.08045	407.7153	62.98885	487.6	3.8	119.6	458
_19AR108_Spot_ 83	123.9738	5639.048	1.096904	17.90875	2.190208	0.57665	2.932575	0.078606	0.679998	0.231877	487.7983	3.194626	462.3063	10.89112	338.521	64.62446	487.8	3.2	144.1	72
_19AR108_Spot_ 539	111.4159	5673.153	1.500795	17.96363	2.957567	0.57768	3.654593	0.078618	0.822835	0.225151	487.8663	3.866192	462.9695	13.58824	342.2524	80.60868	487.9	3.9	142.5	440
_19AR108_Spot_ 107	343.7458	16894.24	2.123486	17.46812	1.143407	0.612632	1.434696	0.078738	0.757752	0.528162	488.5842	3.565436	485.2184	5.534243	470.295	26.96732	488.6	3.6	103.9	94
_19AR108_Spot_ 583	134.0075	7365.534	1.38272	17.87102	2.611891	0.585754	2.99417	0.078765	0.714645	0.238679	488.7493	3.363701	468.1525	11.23061	369.3355	65.51331	488.7	3.4	132.3	478
_19AR108_Spot_ 172	153.5373	8058.621	2.833721	16.80817	2.036848	0.627251	4.084107	0.078793	2.253357	0.551738	488.915	10.6096	494.3822	15.98637	520.718	74.76759	488.9	10.6	93.9	141
_19AR108_Spot_ 79	238.5657	12503.92	11.73393	17.77999	1.530032	0.597277	2.349452	0.078821	0.718925	0.305997	489.0817	3.386058	475.504	8.920785	411.4741	50.02225	489.1	3.4	118.9	68
_19AR108_Spot_ 316	318.5588	15570.79	12.05735	17.5611	1.403852	0.607826	1.887377	0.078828	0.743309	0.393832	489.1251	3.501203	482.1882	7.244963	450.2866	38.54476	489.1	3.5	108.6	264
_19AR108_Spot_ 189	91.77836	6590.636	1.64995	17.12672	2.957747	0.611351	3.336556	0.078831	0.857378	0.256965	489.1433	4.038648	484.4121	12.85441	463.0734	71.48124	489.1	4.0	105.6	157
_19AR108_Spot_ 130	186.2963	8496.954	5.552213	17.14205	1.764317	0.614247	2.046643	0.078842	0.658833	0.321909	489.2096	3.103814	486.2352	7.90777	473.2153	42.87107	489.2	3.1	103.4	111
_19AR108_Spot_ 571	84.59595	3700.562	1.391937	18.20164	3.441589	0.555269	4.327891	0.078973	0.846092	0.195498	489.9918	3.992139	448.4427	15.69058	241.5706	97.88284	490.0	4.0	202.8	467
_19AR108_Spot_ 563	239.917	9803.731	15.11494	17.30019	1.086126	0.614255	1.970536	0.079025	0.669444	0.339727	490.2986	3.16056	486.2398	7.613753	468.099	41.04952	490.3	3.2	104.7	460
_19AR108_Spot_ 558	93.77415	3815.129	1.502941	17.36979	3.137188	0.586913	4.285553	0.07909	0.777757	0.181484	490.6891	3.67474	468.8941	16.09509	364.518	95.05588	490.7	3.7	134.6	456
_19AR108_Spot_ 332	177.2025	10741.73	1.771818	17.14164	1.778041	0.620857	2.132269	0.079155	0.87595	0.410807	491.0742	4.141808	490.3843	8.29332	488.1437	42.92147	491.1	4.1	100.6	278
_19AR108_Spot_ 179	276.4274	12633.01	13.4987	17.80692	1.063436	0.60068	1.97945	0.079191	0.801943	0.405134	491.2906	3.79348	477.665	7.542599	413.7126	40.46201	491.3	3.8	118.8	147
_19AR108_Spot_ 357	153.2185	5976.291	2.808571	17.28608	1.73708	0.605727	2.695349	0.079212	0.66549	0.246903	491.4154	3.148777	480.8618	10.32441	431.7977	58.22079	491.4	3.1	113.8	300
_19AR108_Spot_ 324	69.0734	3436.468	1.92844	17.14746	3.501878	0.589753	4.297998	0.079238	0.943596	0.219543	491.5756	4.466043	470.7102	16.191	371.1762	94.44338	491.6	4.5	132.4	270
_19AR108_Spot_ 591	96.86858	4389.77	2.756779	16.60695	2.324797	0.620997	3.519969	0.079268	0.833198	0.236706	491.7505	3.944879	490.4722	13.69312	485.448	75.53774	491.8	3.9	101.3	486
_19AR108_Spot_ 329	226.4605	6811.473	1.322765	17.19577	1.72103	0.611661	2.0254	0.079328	0.760529	0.375496	492.113	3.603374	484.607	7.805241	450.2073	41.71842	492.1	3.6	109.3	275
_19AR108_Spot_ 103	84.17246	4016.926	1.532591	17.56238	3.314971	0.584485	3.905583	0.079398	0.720959	0.184597	492.528	3.418663	467.3395	14.62955	346.4068	86.8274	492.5	3.4	142.2	90
_19AR108_Spot_ 292	313.3143	15425.72	17.21092	17.36099	1.535558	0.619859	2.337663	0.079449	0.791882	0.338749	492.8331	3.757205	489.7587	9.0832	476.3598	48.63648	492.8	3.8	103.5	244
_19AR108_Spot_ 272	107.9799	5151.716	6.744801	17.33392	2.621133	0.602245	3.94231	0.079491	0.801229	0.203238	493.0835	3.803414	478.6576	15.04724	411.0737	86.3383	493.1	3.8	120.0	226
_19AR108_Spot_ 314	104.7757	3882.609	1.933177	17.50497	2.949584	0.584465	6.433125	0.079554	0.894945	0.139115	493.4581	4.251387	467.3268	24.09957	341.9098	144.3205	493.5	4.3	144.3	263
_19AR108_Spot_ 534	124.4109	5619.265	2.584147	17.89393	2.875773	0.586995	4.985606	0.079573	1.043712	0.209345	493.5744	4.959222	468.9468	18.72647	351.1272	110.2561	493.6	5.0	140.6	435
_19AR108_Spot_ 581	77.03165	3289.153	1.218907	16.98058	4.650293	0.59697	7.5456	0.079598	0.854728	0.113275	493.7202	4.062414	475.3089	28.64802	388.3544	168.5161	493.7	4.1	127.1	476
_19AR108_Spot_ 261	91.08318	4322.31	3.313292	16.74857	2.533623	0.619254	6.198185	0.079599	0.717426	0.115748	493.7262	3.409874	489.3798	24.07297	470.0263	136.371	493.7	3.4	105.0	215
_19AR108_Spot_ 195	122.4793	7106.994	1.884216	17.77818	2.644681	0.59603	3.377245	0.079616	0.747558	0.221351	493.8312	3.553814	474.7112	12.80686	384.2923	74.0278	493.8	3.6	128.5	162
_19AR108_Spot_ 595	178.4766	9229.345	4.572526	17.71753	1.72562	0.602765	2.091795	0.079727	0.64326	0.307516	494.495	3.061948	478.9869	7.987966	406.3602	44.55199	494.5	3.1	121.7	489
_19AR108_Spot_ 569	73.19692	3272.182	1.705659	17.32135	3.29131	0.586155	5.288119	0.079787	0.906927	0.171503	494.8541	4.320035	468.4094	19.8451	341.787	117.9825	494.9	4.3	144.8	465
_19AR108_Spot_ 360	209.5762	9270.86	4.424532	17.48571	1.755708	0.612001	2.262116	0.07979	0.714106	0.315681	494.8693	3.401656	484.8214	8.72052	438.5442	47.7754	494.9	3.4	112.8	302
_19AR108_Spot_ 411	170.1373	7436.324	4.241327	17.81102	1.634657	0.596718	2.309813	0.079916	0.858454	0.371655	495.623	4.095248	475.1489	8.765131	378.4274	48.23384	495.6	4.1	131.0	344
_19AR108_Spot_ 236	109.5158	5933.984	2.998023	17.26114	3.634127	0.612114	4.817442	0.079931	0.73664	0.152911	495.7088	3.514721	484.8927	18.57512	435.0364	106.083	495.7	3.5	113.9	196
_19AR108_Spot_ 284	57.25847	4366.364	1.576718	15.83029	3.052299	0.6605	5.686215	0.08001	0.79009	0.138948	496.1846	3.773228	514.92	22.97002	599.9246	122.006	496.2	3.8	82.7	236
_19AR108_Spot_ 101	202.7133	3603.907	2.529841	17.40891	2.14109	0.591158	6.189953	0.080032	0.681091	0.110032	496.3156	3.253508	471.607	23.35523	354.0666	139.0822	496.3	3.3	140.2	88
_19AR108_Spot_ 45	219.5647	12907.99	6.804574	17.35165	1.498875	0.624859	1.798616	0.080043	0.700159	0.389277	496.3782	3.345001	492.8884	7.023309	477.6467	36.62615	496.4	3.3	103.9	37
_19AR108_Spot_ 287	99.53	4486.726	1.920688	16.24631	4.142277	0.64414	6.744148	0.080082	0.840097	0.124567	496.612	4.015371	504.8659	26.83486	543.4118	146.4171	496.6	4.0	91.4	239
_19AR108_Spot_ 328	55.79557	1882.785	1.178377	15.24349	6.90724	0.640213	14.90649	0.080381	1.268087	0.085069	498.3993	6.081996	502.4382	59.14533	521.8073	327.4948	498.4	6.1	95.5	274
_19AR108_Spot_ 166	140.1772	4731.786	2.948644	17.39724	2.093811	0.604634	4.72136	0.080667	2.251966	0.476974	500.103	10.83639	480.1705	18.0659	387.0321	93.20322	500.1	10.8	129.2	136
_19AR108_Spot_ 235	81.16849	4010.161	1.184799	16.94358	3.424808	0.617094	4.289307	0.080715	1.132343	0.263992	500.3879	5.451782	488.0244	16.62157	431.3371	92.204	500.4	5.5	116.0	195
_19AR108_Spot_ 552	572.5573	2817.116	164.4739	16.70908	0.866895	0.612418	1.718584	0.081047	0.766052	0.445746	502.3702	3.702283	485.0837	6.627917	405.1701	34.44041	502.4	3.7	124.0	452
_19AR108_Spot_ 192	223.9521	13957.07	2.269741	17.03585	1.466106	0.649041	1.719092	0.081545	0.77094	0.448458	505.3365	3.747059	507.8885	6.87031	520.3311	33.71291	505.3	3.7	97.1	159
_19AR108_Spot_ 398	156.502	6473.291	1.780308	17.31615	2.344676	0.630222	4.179389	0.082344	0.711202	0.170169	510.099	3.488015	496.2342	16.40693	433.7146	91.77794	510.1	3.5	117.6	334
_19AR108_Spot_ 508	115.9127	4310.333	2.220251	17.52023	2.900179	0.612017	5.874365	0.082456	1.083513	0.184448	510.765	5.320639	484.8316	22.64941	364.9892	130.262	510.8	5.3	139.9	413
_19AR108_Spot_ 13	162.7906	6854.261	2.38706	17.44193	2.145309	0.63004	2.950569	0.082514	0.691531	0.234372	511.1087	3.397987	496.1211	11.58043	428.4879	63.97093	511.1	3.4	119.3	10
_19AR108_Spot_ 460	277.1074	7622.229	2.93328	17.06876	1.264784	0.645018	2.738459	0.082514	0.734584	0.268247	511.1123	3.609565	505.4083	10.9032	480.6146	58.29708	511.1	3.6	106.3	388
_19AR108_Spot_ 450	200.0873	9166.348	3.95469	17.49074	1.528741	0.63582	2.908401	0.082973	0.712701	0.245049	513.8443	3.520018	499.7148	11.47893	436.4602	62.80981	513.8	3.5	117.7	379
_19AR108_Spot_ 518	154.3329	6284.861	2.473005	17.39197	1.63794	0.636016	2.063126	0.083364	0.857211	0.415491	516.1704	4.252159	499.8366	8.144143	426.6757	41.86233	516.2	4.3	121.0	422
_19AR108_Spot_ 3	69.29717	3287.83	1.389659	15.68218	3.93257	0.681531	5.332377	0.083373	0.907836	0.17025	516.2239	4.503731	527.6991	21.9482	578.5757	114.2403	516.2	4.5	89.2	3
_19AR108_Spot_ 177	286.3665	11033.25	7.673876	16.40552	1.639819	0.687476	1.993514	0.083438	1.076126	0.539814	516.6133	5.342481	531.283	8.246673	595.72	36.37306	516.6	5.3	86.7	145
_19AR108_Spot_ 8	202.3006	10589.4	1.864019	17.3681	1.467691	0.647102	2.354013	0.083566	0.732361	0.311112	517.3744	3.640985	506.6939	9.390818	459.7544	49.61388	517.4	3.6	112.5	6
_19AR108_Spot_ 15	164.4717	10250.25	4.20152	17.05697	2.019799	0.661358	2.694108	0.083585	0.705146	0.261736	517.4829	3.506392	515.4441	10.89017	507.3506	57.1957	517.5	3.5	102.0	12
_19AR108_Spot_ 369	247.922	18396.76	5.276616	17.7538	1.656692	0.639608	2.026539	0.083654	0.936711	0.462222	517.8945	4.661423	502.0632	8.027264	431.4923	40.04738	517.9	4.7	120.0	309
_19AR108_Spot_ 449	219.1106	11579.79	3.566173	17.54806	1.653612	0.644764	2.494383	0.083858	0.66321	0.265881	519.1125	3.307831	505.2514	9.928965	443.9245	53.48975	519.1	3.3	116.9	378
_19AR108_Spot_ 514	270.079	15207.09	3.353561	17.14886	1.411895	0.66566	1.850275	0.083995	0.952795	0.514948	519.922	4.759288	518.0702	7.508274	510.8732	34.86231	519.9	4.8	101.8	418
_19AR108_Spot_ 506	170.529	1408.818	5.860586	16.20896	2.216395	0.598401	4.217066	0.08427	1.902435	0.451128	521.5569	9.53151	476.2184	16.03182	264.3388	86.43307	521.6	9.5	197.3	411
_19AR108_Spot_ 12	140.3747	7073.678	2.764621	16.96244	2.822171	0.662768	3.098507	0.084271	0.731725	0.236154	521.5657	3.66612	516.3056	12.54106	494.0733	66.38373	521.6	3.7	105.6	9
_19AR108_Spot_ 400	436.6999	18437.17	8.71692	16.93083	0.912843	0.678417	1.287668	0.084479	0.691614	0.537106	522.8041	3.473051	525.8174	5.284876	539.8954	23.75754	522.8	3.5	96.8	336
_19AR108_Spot_ 10	176.0873	11236.28	2.137627	16.9816	1.727569	0.670637	1.905115	0.084513	0.648402	0.340348	523.0027	3.257245	521.0998	7.765424	513.7447	39.34467	523.0	3.3	101.8	8
_19AR108_Spot_ 226	221.6896	10389.24	1.444681	17.27658	1.526801	0.659206	2.129446	0.084576	0.709428	0.333151	523.3778	3.56626	514.1285	8.590687	474.203	44.4153	523.4	3.6	110.4	188
_19AR108_Spot_ 407	80.30155	4489.423	2.172676	16.88991	3.25034	0.652482	4.553978	0.084622	0.818238	0.179675	523.6546	4.115332	510.005	18.25994	450.2141	99.54932	523.7	4.1	116.3	341
_19AR108_Spot_ 138	211.0303	16438.81	3.388737	17.35274	1.605979	0.660978	1.916744	0.084645	0.955386	0.498442	523.7893	4.806304	515.2119	7.74507	478.2838	36.73101	523.8	4.8	109.5	118
_19AR108_Spot_ 410	181.3355	9704.803	3.202998	17.30694	1.555719	0.65677	2.522081	0.08468	0.694559	0.275391	523.9975	3.495482	512.6365	10.15207	463.2786	53.73679	524.0	3.5	113.1	343
_19AR108_Spot_ 439	116.8306	5681.008	1.8729	17.14826	1.718059	0.652316	3.72855	0.084751	0.814134	0.218351	524.4198	4.100432	509.9028	14.94741	446.2703	80.91538	524.4	4.1	117.5	368
_19AR108_Spot_ 381	262.8284	13995.3	4.582674	17.49798	1.515805	0.654692	1.742146	0.084812	0.739672	0.424575	524.7799	3.727854	511.3618	6.999081	452.7582	35.01991	524.8	3.7	115.9	319

_19AR108_Spot_ 334	214.4493	7501.339	1.521865	17.0242	1.664956	0.663782	2.324893	0.084823	1.273706	0.547856	524.849	6.420132	516.9247	9.418341	483.0318	42.98004	524.8	6.4	108.7	279
_19AR108_Spot_ 447	351.8143	24274.15	2.309684	17.45179	1.042026	0.664815	1.4736	0.084991	0.621842	0.421988	525.8425	3.140097	517.555	5.975159	482.1157	29.51333	525.8	3.1	109.1	376
_19AR108_Spot_ 33	209.1499	8095.839	1.516479	16.76503	1.544251	0.679556	2.512784	0.085233	0.74118	0.294963	527.2851	3.752567	526.5057	10.32359	524.0614	52.66577	527.3	3.8	100.6	26
_19AR108_Spot_ 118	131.5808	6261.626	2.137143	17.04737	2.25013	0.662437	3.995371	0.085307	0.728119	0.182241	527.7245	3.689388	516.1037	16.16677	465.9606	87.05148	527.7	3.7	113.3	102
_19AR108_Spot_ 375	857.9041	38029.51	48.46136	17.24918	0.758774	0.676639	1.245756	0.085356	0.967858	0.776924	528.0153	4.906744	524.7408	5.10486	511.4951	17.2525	528.0	4.9	103.2	313
_19AR108_Spot_ 141	57.08257	4009.783	2.624487	15.55952	5.483617	0.712362	7.222229	0.085364	0.970288	0.134347	528.0613	4.919474	546.1476	30.51665	623.2603	154.5106	528.1	4.9	84.7	120
_19AR108_Spot_ 494	116.3153	7105.719	2.678918	16.80024	1.894882	0.685848	3.567157	0.085519	0.900359	0.252403	528.9791	4.572542	530.3027	14.73642	536.9686	75.55165	529.0	4.6	98.5	406
_19AR108_Spot_ 488	172.6853	7471.226	1.540627	17.55339	2.215311	0.653507	3.627661	0.085521	0.691641	0.190658	528.9903	3.512623	510.6344	14.55896	430.2158	79.38227	529.0	3.5	123.0	400
_19AR108_Spot_ 443	106.5899	7308.173	2.105573	16.98964	2.979502	0.671878	4.152395	0.085577	0.714674	0.172111	529.3259	3.631807	521.8537	16.94551	490.2642	90.2809	529.3	3.6	108.0	372
_19AR108_Spot_ 59	116.0443	3904.705	1.748822	17.42828	2.553012	0.6346	2.987277	0.085735	0.802513	0.268643	530.2667	4.085137	498.9571	11.77642	358.7414	64.94042	530.3	4.1	147.8	49
_19AR108_Spot_ 536	138.2017	8162.469	2.321772	17.29117	2.616852	0.666821	3.282066	0.085936	0.991531	0.302106	531.4588	5.05821	518.7775	13.33283	464.2628	69.34535	531.5	5.1	114.5	437
_19AR108_Spot_ 52	274.1916	9810.545	10.47046	17.3978	0.949493	0.66716	1.585034	0.085996	0.717726	0.452814	531.8121	3.663753	518.9843	6.440613	463.8947	31.33674	531.8	3.7	114.6	44
_19AR108_Spot_ 289	285.7099	9913.172	2.748971	16.79386	1.296704	0.687896	2.596692	0.086008	0.698185	0.268875	531.8861	3.564477	531.5355	10.74592	531.0045	54.80406	531.9	3.6	100.2	241
_19AR108_Spot_ 420	241.1383	18090.44	5.201818	17.01055	1.419484	0.68804	1.732841	0.086009	0.673197	0.388493	531.8899	3.436929	531.6223	7.171779	531.4484	34.97498	531.9	3.4	100.1	352
_19AR108_Spot_ 219	243.4163	11078.79	2.420713	17.12542	1.743826	0.677641	2.60199	0.086014	0.641029	0.246361	531.9217	3.272884	525.3478	10.67216	497.8367	55.56109	531.9	3.3	106.8	183
_19AR108_Spot_ 296	398.6358	28153.82	4.392064	17.17333	0.75911	0.686407	1.208686	0.086368	0.697727	0.577261	534.0226	3.575865	530.6395	4.995354	517.0518	21.67362	534.0	3.6	103.3	247
_19AR108_Spot_ 174	159.6158	10976.87	1.860609	16.92366	1.74252	0.689376	2.698757	0.086386	0.749628	0.277768	534.1277	3.842583	532.4254	11.18255	526.1129	56.84452	534.1	3.8	101.5	143
_19AR108_Spot_ 163	360.4945	25231.02	2.816554	17.2445	1.16421	0.68344	1.628129	0.086392	0.844446	0.51866	534.1627	4.328888	528.8513	6.711624	506.9398	30.62363	534.2	4.3	105.4	134
_19AR108_Spot_ 568	235.0759	10946.26	3.04683	17.05319	1.418188	0.68387	1.946482	0.086449	0.724782	0.372355	534.5016	3.717717	529.1106	8.027017	506.9023	39.72274	534.5	3.7	105.4	464
_19AR108_Spot_ 70	144.2051	6047.742	1.739346	16.84378	2.292619	0.677056	3.370152	0.086505	0.846113	0.251061	534.8351	4.34267	524.9934	13.81603	483.3996	72.08089	534.8	4.3	110.6	60
_19AR108_Spot_ 300	125.6091	6679.508	2.895948	17.24521	2.780934	0.665499	3.559626	0.086579	0.598595	0.168162	535.2756	3.074714	517.972	14.44331	443.3044	78.05102	535.3	3.1	120.7	251
_19AR108_Spot_ 85	112.5616	7894.794	2.413466	17.04229	3.324963	0.67901	3.551884	0.086596	0.750806	0.211382	535.3742	3.857233	526.1759	14.58618	487.434	76.6345	535.4	3.9	109.8	74
_19AR108_Spot_ 350	218.1408	14416.24	7.819742	17.22986	1.182071	0.681441	1.622942	0.086701	0.693314	0.427195	535.9953	3.565832	527.645	6.678608	492.6285	32.35635	536.0	3.6	108.8	294
_19AR108_Spot_ 366	153.848	6771.69	2.507946	17.45309	2.017233	0.659468	2.631116	0.086799	0.732483	0.278393	536.58	3.77123	514.2888	10.61722	417.3622	56.47999	536.6	3.8	128.6	306
_19AR108_Spot_ 54	367.6385	14079.22	1.262725	17.33735	1.019088	0.680012	2.030963	0.086806	0.725957	0.357445	536.6184	3.737885	526.7819	8.347301	485.3414	41.8985	536.6	3.7	110.6	46
_19AR108_Spot_ 358	200.8279	10476.58	4.123136	17.1117	1.40062	0.683098	2.506838	0.086822	0.806002	0.321521	536.712	4.150724	528.6449	10.33106	494.904	52.32468	536.7	4.2	108.4	301
_19AR108_Spot_ 89	187.1944	11645.84	3.548249	17.27706	1.400578	0.680783	2.18289	0.086898	0.760455	0.348371	537.1661	3.919347	527.2474	8.977801	485.4965	45.19609	537.2	3.9	110.6	78
_19AR108_Spot_ 588	140.4762	11852.69	2.465845	16.79867	1.816116	0.699238	2.105909	0.087028	0.830591	0.39441	537.9362	4.286707	538.3359	8.799364	540.9894	42.3218	537.9	4.3	99.4	483
_19AR108_Spot_ 188	354.6105	16176.09	2.841985	17.0477	0.88517	0.693887	1.266108	0.087037	0.62909	0.496869	537.9888	3.247055	535.1333	5.266342	523.9139	24.09765	538.0	3.2	102.7	156
_19AR108_Spot_ 160	125.5272	4037.294	0.567088	17.55829	2.821819	0.639969	4.222	0.087192	0.872494	0.206654	538.9099	4.510785	502.2867	16.73056	339.7427	93.56575	538.9	4.5	158.6	131
_19AR108_Spot_ 306	192.9224	11128.93	53.37039	17.15032	1.779582	0.68381	2.271304	0.087221	0.785908	0.346016	539.0815	4.064377	529.0743	9.366121	487.082	47.0363	539.1	4.1	110.7	256
_19AR108_Spot_ 88	492.6284	5012.707	24.83562	16.32897	0.952788	0.703724	1.850046	0.087291	0.7599	0.410747	539.4984	3.932789	541.0127	7.759325	548.3697	36.85494	539.5	3.9	98.4	77
_19AR108_Spot_ 229	140.8068	5949.145	3.029728	17.31991	1.74825	0.669097	3.647372	0.087524	0.727036	0.199331	540.8748	3.771903	520.1632	14.84733	431.1602	79.66699	540.9	3.8	125.4	190
_19AR108_Spot_ 231	314.9659	17815.29	7.602608	17.18537	1.481926	0.694954	1.994395	0.087603	0.90736	0.454955	541.3435	4.711345	535.7728	8.303269	513.1028	39.02482	541.3	4.7	105.5	191
_19AR108_Spot_ 56	281.8077	14327.34	4.298044	17.16154	1.315481	0.693843	1.689102	0.087628	0.770569	0.4562	541.4959	4.002157	535.1069	7.02556	508.9144	33.05238	541.5	4.0	106.4	47
_19AR108_Spot_ 492	156.3482	5268.853	1.521483	17.18957	1.350365	0.674506	2.343107	0.087644	0.858031	0.366193	541.5884	4.457142	523.4484	9.583719	446.0212	48.46781	541.6	4.5	121.4	404
_19AR108_Spot_ 267	146.1022	7766.423	3.267718	17.20791	2.262339	0.681435	3.082118	0.087769	0.731476	0.237329	542.331	3.804731	527.6414	12.6837	465.5701	66.35867	542.3	3.8	116.5	221
_19AR108_Spot_ 550	614.6083	21499.85	2.607597	17.18561	0.761697	0.697434	1.54398	0.087814	0.66249	0.42908	542.5945	3.447513	537.2573	6.44151	515.5996	30.62625	542.6	3.4	105.2	451
_19AR108_Spot_ 335	111.2371	4674.878	2.244887	17.18708	2.536232	0.666635	4.18198	0.087872	0.815008	0.194886	542.9381	4.243769	518.6643	16.98634	414.0731	91.70218	542.9	4.2	131.1	280
_19AR108_Spot_ 264	176.5174	10564.14	2.004872	17.66065	1.709706	0.670741	2.083571	0.087948	0.919722	0.441416	543.3927	4.792864	521.1628	8.493643	425.8365	41.71362	543.4	4.8	127.6	218
_19AR108_Spot_ 204	232.0816	9915.932	2.840776	16.78932	1.324793	0.708784	1.67578	0.088403	0.725164	0.432732	546.0848	3.796921	544.0242	7.057987	536.3704	33.07419	546.1	3.8	101.8	170
_19AR108_Spot_ 318	213.3857	10380.33	2.221655	16.89721	1.28757	0.703586	2.296153	0.088438	0.876353	0.381661	546.2903	4.590192	540.9307	9.629349	519.3404	46.59293	546.3	4.6	105.2	266
_19AR108_Spot_ 247	103.9817	6409.949	3.118724	17.25946	2.46164	0.679454	3.354197	0.088478	0.857128	0.255539	546.5287	4.491372	526.4441	13.77961	441.2157	72.16061	546.5	4.5	123.9	205
_19AR108_Spot_ 48	118.4739	7651.942	2.303326	17.28085	2.074091	0.684471	2.340816	0.088523	0.858374	0.366699	546.796	4.500008	529.4732	9.658328	456.4683	48.33383	546.8	4.5	119.8	40
_19AR108_Spot_ 288	185.8061	12603.22	1.309347	17.07863	2.008346	0.70586	2.381221	0.089241	0.651761	0.273709	551.0464	3.442286	542.285	10.00504	506.6019	50.39946	551.0	3.4	108.8	240
_19AR108_Spot_ 276	348.4854	13746.52	32.91995	16.79256	1.069168	0.723559	1.471763	0.089583	0.655689	0.445513	553.071	3.475216	552.7655	6.273658	552.4589	28.76022	553.1	3.5	100.1	230
_19AR108_Spot_ 345	119.1103	5930.197	1.828002	17.18497	2.734486	0.691933	4.22282	0.090081	0.660394	0.156387	556.0142	3.517989	533.9609	17.53705	441.764	92.80762	556.0	3.5	125.9	290
_19AR108_Spot_ 336	305.3552	14826.18	2.954852	16.89151	0.932024	0.732959	1.226914	0.091378	0.643795	0.524727	563.6796	3.474809	558.2887	5.269136	537.3025	22.85657	563.7	3.5	104.9	281
_19AR108_Spot_ 522	63.48663	3055.804	0.878562	16.96456	8.989828	0.687628	13.25574	0.091791	8.656151	0.653011	566.1229	46.91513	531.3744	54.89511	385.7665	226.0036	566.1	46.9	146.8	424
_19AR108_Spot_ 504	42.79287	1735.567	0.769201	15.03785	8.089672	0.757752	12.87584	0.094168	1.34102	0.10415	580.1404	7.440003	572.7123	56.41852	544.2838	280.9241	580.1	7.4	106.6	409
_19AR108_Spot_ 396	249.6524	15328.31	1.900991	16.80696	1.185969	0.766957	1.640494	0.094984	0.706284	0.430531	584.9485	3.949501	578.0158	7.230311	551.8029	32.32095	584.9	3.9	106.0	332
_19AR108_Spot_ 317	408.0608	1957.227	1.923737	15.74942	0.95897	0.739042	1.838477	0.095756	1.043657	0.567674	589.4911	5.879349	561.8466	7.933344	452.3561	33.61717	589.5	5.9	130.3	265
_19AR108_Spot_ 371	331.2296	16269.89	1.351903	16.60881	1.003161	0.782781	1.425412	0.095848	0.935918	0.656595	590.0326	5.27704	587.0685	6.35504	576.5493	23.36099	590.0	5.3	102.3	311
_19AR108_Spot_ 223	284.5534	2327.406	3.009789	16.33181	1.261929	0.726831	1.712492	0.095861	0.763144	0.445634	590.1072	4.303399	554.6914	7.318962	412.8002	34.27507	590.1	4.3	143.0	186
_19AR108_Spot_ 464	50.74048	1332.691	1.04479	16.76774	3.575652	0.643296	14.84412	0.096089	1.164	0.078415	591.4456	6.578055	504.3447	59.07027	127.466	NA	591.4	6.6	464.0	392
_19AR108_Spot_ 387	354.8089	27182.06	26.67194	15.95381	1.021958	0.823604	1.509662	0.096177	0.814119	0.539272	591.965	4.604645	610.0571	6.923156	678.7348	27.14778	592.0	4.6	87.2	324
_19AR108_Spot_ 532	212.6605	11993.7	1.070654	16.76262	1.441517	0.779252	1.772237	0.09646	0.972802	0.548912	593.6273	5.516905	585.0564	7.881354	552.8885	32.35148	593.6	5.5	107.4	433
_19AR108_Spot_ 198	200.3689	11408.92	1.130091	17.00364	1.643206	0.768098	2.129809	0.096641	0.73209	0.343735	594.6947	4.15892	578.6712	9.394921	517.1866	43.91684	594.7	4.2	115.0	165
_19AR108_Spot_ 574	439.5732	23861.91	35.93252	16.60754	0.968039	0.794486	1.276342	0.096647	0.699601	0.54813	594.7296	3.974577	593.7136	5.737832	590.7788	23.12873	594.7	4.0	100.7	470
_19AR108_Spot_ 245	1281.428	42834.51	2.641107	16.68228	0.649922	0.796365	1.083236	0.096851	0.709552	0.65503	595.93	4.038873	594.7759	4.876113	591.3387	17.7301	595.9	4.0	100.8	203
_19AR108_Spot_ 481	155.2409	12545.34	10.51221	16.32807	1.605251	0.810798	2.011952	0.097467	0.868405	0.431623	599.5487	4.971732	602.9018	9.14749	616.4473	39.20983	599.5	5.0	97.3	397
_19AR108_Spot_ 414	160.8728	14419.17	4.075795	16.67227	1.764266	0.796057	1.925695	0.097904	0.672147	0.349041	602.1161	3.863848	594.6017	8.666661	567.0036	39.27058	602.1	3.9	106.2	347
_19AR108_Spot_ 183	334.9512	29359.19	30.62349	16.60912	0.907136	0.808839	1.089852	0.098137	0.564775	0.518213	603.4816	3.253643	601.8028	4.948383	596.4467	20.19344	603.5	3.3	101.2	151
_19AR108_Spot_ 269	748.841	26528.86	5.175872	15.48679	0.752328	0.869412	1.170727	0.098325	0.822998	0.702981	604.5833	4.749511	635.2477	5.528536	746.8236	17.57652	604.6	4.7	81.0	223
_19AR108_Spot_ 382	213.9729	11520.6	5.407009	16.63593	1.743779	0.798356	2.087477	0.098603	0.873278	0.418341	606.2154	5.052651	595.9008	9.409898	557.7973	41.3621	606.2	5.1	108.7	320
_19AR108_Spot_ 509	131.8306	6146.004	3.620493	15.98026	2.458844	0.826184	4.027801	0.099238	1.277085	0.317068	609.9388	7.432289	611.4926	18.50453	618.1704	82.49943	609.9	7.4	98.7	414
_19AR108_Spot_ 452	279.5411	5392.266	1.601546	16.24373	0.910307	0.806027	1.243927	0.099275	0.703841	0.565822	610.1562	4.097552	600.2226	5.637083	563.8027	22.36248	610.2	4.1	108.2	381
_19AR108_Spot_ 147	314.8819	18276.05	9.728467	16.36875	1.007727	0.826556	1.430731	0.099599	0.819362	0.572688	612.058	4.784258	611.6993	6.574051	611.2918	25.34639	612.1	4.8	100.1	125
_19AR108_Spot_ 32	651.8082	15065.97	3.142409	16.24282	0.742636	0.831523	1.224413	0.099657	0.770922	0.629626	612.3993	4.503807	614.4568	5.644481	622.965	20.53728	612.4	4.5	98.3	25
_19AR108_Spot_ 319	337.5714	17239.42	1.925008	16.64466	0.820513	0.81262	1.188488	0.099659	0.660553	0.555793	612.413	3.859104	603.9226	5.410152	573.11	21.47836	612.4	3.9	106.9	267
_19AR108_Spot_ 463	199.466	7624.683	2.628409	16.31839	1.436424	0.815568	2.083132	0.099752	0.879976	0.422429	612.9546	5.145355	605.5731	9.501828	578.9916	41.01704	613.0	5.1	105.9	391
_19AR108_Spot_ 415	1425.234	129776.3	23.43028	16.58155	0.692356	0.828654	1.001858	0.099845	0.705216	0.703908	613.4988	4.126998	612.8653	4.609789	611.4453	15.35669	613.5	4.1	100.3	348
_19AR108_Spot_ 378	218.1224	14942.01	3.843458	16.48187	1.252753	0.827656	1.729124	0.100676	0.737107	0.426289	618.3669	4.346243	612.3106	7.950974	590.901	33.90941	618.4	4.3	104.6	316
_19AR108_Spot_ 251	1071.193	33623.55	19.46302	16.46857	0.631905	0.838364	1.152511	0.100726	0.676523	0.587	618.6609	3.990824	618.2422	5.336788	617.6282	20.15729	618.7	4.0	100.2	207
_19AR108_Spot_ 313	105.0491	6960.684	3.528801	16.23607	2.235113	0.825862	2.65119	0.100804	0.765641	0.288791	619.1195	4.519719	611.3137	12.17674	583.4213	55.11578	619.1	4.5	106.1	262
_19AR108_Spot_ 545	702.3278	37634.25	17.90118	16.4804	0.63905	0.841	1.159832	0.100842	0.939792	0.810283	619.3402	5.549651	619.6973	5.379862	621.9197	14.68274	619.3	5.5	99.6	446
_19AR108_Spot_ 512	110.6813	7708.965	11.40672	16.30532	1.504435	0.830729	3.475856	0.101079	0.936944	0.269558	620.728	5.544645	614.0167	16.01633	590.2882	72.60648	620.7	5.5	105.2	416
_19AR108_Spot_ 67	302.4777	15307.68	24.43865	16.22537	1.268518	0.843625	1.716011	0.101152	0.973546	0.567331	621.1559	5.76503	621.1439	7.973256	622.018	30.5004	621.2	5.8	99.9	57
_19AR108_Spot_ 178	160.5046	13323.29	3.627167	16.36797	1.571684	0.838972	2.505727	0.101295	0.746684	0.297991	621.9908	4.427288	618.578	11.60793	607.03	51.7223	622.0	4.4	102.5	146
_19AR108_Spot_ 114	131.2599	15131.18	3.581304	16.33372	1.626228	0.844045	1.983727	0.101534	0.874958	0.441068	623.3913	5.198984	621.3756	9.219722	614.9619	38.44318	623.4	5.2	101.4	98
_19AR108_Spot_ 541	288.7117	15992.38	6.952558	16.65881	0.92992	0.831551	1.697804	0.101627	0.65647	0.386658	623.9365	3.903982	614.4723	7.827012	580.6969	34.01128	623.9	3.9	107.4	442
_19AR108_Spot_ 225	284.0394	19380.71	3.252327	16.73373	1.084992	0.831009	1.590548	0.102047	0.701749	0.4412	626.3952	4.18891	614.1717	7.329922	570.2903	31.06077	626.4	4.2	109.8	187
_19AR108_Spot_ 598	190.744	10977.59	3.772706	16.38735	1.749344	0.840164	2.5177	0.102116	0.676844	0.268834	626.7992	4.042729	619.2364	11.67241	592.6125	52.59637	626.8	4.0	105.8	492
_19AR108_Spot_ 409	540.5147	11478.2	22.40185	16.27248	0.80741	0.848872	1.171844	0.102336	0.745164	0.63589	628.0871	4.459505	624.0297	5.463101	610.2674	19.52663	628.1	4.5	102.9	342
_19AR108_Spot_ 34	150.3742	7918.562	1.723788	15.31223	2.000284	0.895026	3.164484	0.102389	0.875551	0.276681	628.392	5.24224	649.0659	15.17699	722.5455	64.53306	628.4	5.2	87.0	27
_19AR108_Spot_ 320	561.0514	24148.93	11.58038	15.63146	0.754974	0.894189	1.288693	0.102503	0.764549	0.593275	629.0624	4.582281	648.6172	6.177186	718.1838	22.02416	629.1	4.6	87.6	268
_19AR108_Spot_ 63	967.7986	21001.65	3.771454	16.22888	0.746846	0.8616	1.098144	0.102895	0.675956	0.615544	631.3523	4.06534	630.9958	5.160743	630.677	18.65169	631.4	4.1	100.1	53
_19AR108_Spot_ 200	348.4436	22579.22	1.511664	16.4265	0.812323	0.855428	1.200629	0.102898	0.722708	0.601941	631.3708	4.346633	627.6241	5.6206	615.0659	20.71349	631.4	4.3	102.7	167
_19AR108_Spot_ 425	497.9696	28034.51	3.778948	16.49938	0.842248	0.855002	1.145268	0.103117	0.713136	0.62268	632.6502	4.297339	627.3908	5.35999	609.3963	19.36215	632.7	4.3	103.8	355
_19AR108_Spot_ 254	177.5462	7146.875	2.275236	16.30185	1.568338	0.843794	3.659378	0.103143	0.638893	0.17459	632.8027	3.850832	621.2369	17.00598	580.2942	78.2911	632.8	3.9	109.0	210
_19AR108_Spot_ 212	244.0045	13340.08	3.73248	16.45804	1.123026	0.850526	1.471525	0.103235	0.854004	0.580353	633.3367	5.151518	624.9379	6.867466	595.5951	25.94485	633.3	5.2	106.3	177
_19AR108_Spot_ 90	434.1844	20855.68	2.001488	16.42904	0.789559	0.86449	1.052778	0.103851	0.65022	0.617623	636.9404	3.943489	632.5712	4.956445	617.9054	17.89426	636.9	3.9	103.1	79
_19AR108_Spot_ 29	231.0047	12111.88	0.907654	16.33616	1.040981	0.859781	1.484311	0.104051	0.743586	0.500964	638.1066	4.517592	630.0031	6.967668	601.945	27.79319	638.1	4.5	106.0	23
_19AR108_Spot_ 270	293.5547	19664.2	3.841337	16.52833	1.089087	0.864668	1.416072	0.104762	0.812383	0.573687	642.2562	4.966086	632.6677	6.667595	599.4971	25.10343	642.3	5.0	107.1	224
_19AR108_Spot_ 351	247.4089	12010.47	3.154115	16.44118	1.380599	0.865514	2.10291	0.105343	0.749902	0.356602	645.6449	4.60714	633.1285	9.90695	589.6547	42.6345	645.6	4.6	109.5	295
_19AR108_Spot_ 255	125.4961	7777.493	2.771632	14.7456	1.817531	0.959656	3.30129	0.105474	0.929629	0.281596	646.4114	5.717768	683.1184	16.41675	806.8976	66.33428	646.4	5.7	80.1	211
_19AR108_Spot_ 402	243.6002	18285.45	1.132949	16.09922	1.17921	0.893437	1.496679	0.105746	0.67609	0.451727	647.9955	4.168037	648.2144	7.170986	649.9327	28.67155	648.0	4.2	99.7	337
_19AR108_Spot_ 24	449.6165	23535.49	0.926393	16.32999	0.763555	0.889072	1.373321	0.106405	0.645628	0.470122	651.8331	4.002641	645.8704	6.562908	625.9974	26.13237	651.8	4.0	104.1	20
_19AR108_Spot_ 600	190.8319	11063.94	3.49043	15.39829	1.77886	0.935382	2.642343	0.106615	1.175808	0.444987	653.0591	7.302586	670.462	12.96776	730.3331	50.14899	653.1	7.3	89.4	493
_19AR108_Spot_ 490	285.3997	20068.89	0.553331	16.27986	0.991765	0.902529	1.366759	0.107045	0.792172	0.579599	655.5664	4.93788	653.0782	6.583515	645.4463	23.9539	655.6	4.9	101.6	402
_19AR108_Spot_ 77	1033.587	55034.27	1.560586	16.30889	0.635871	0.90202	1.057146	0.10741	0.785447	0.742988	657.6871	4.911004	652.8063	5.090606	636.9282	15.23056	657.7	4.9	103.3	66
_19AR108_Spot_ 547	512.2368	34826.64	1.912916	16.10443	0.752512	0.917536	1.080902	0.107546	0.718974	0.665161	658.4815	4.500541	661.0562	5.2517	670.794	17.25993	658.5	4.5	98.2	448
_19AR108_Spot_ 259	232.7771	16406.89	4.470789	16.29269	1.130412	0.90286	1.628839	0.108082	0.606591	0.372407	661.5997	3.814134	653.2549	7.847482	625.4534	32.58567	661.6	3.8	105.8	214
_19AR108_Spot_ 275	301.7474	18692.25	1.250891	16.20928	0.913792	0.924074	1.269801	0.109855	0.679692	0.535275	671.9056	4.336945	664.5122	6.192356	640.4682	23.06662	671.9	4.3	104.9	229
_19AR108_Spot_ 194	126.8868	7874.262	1.978821	15.56144	2.529624	0.95196	3.264375	0.110602	1.916441	0.587078	676.2413	12.30319	679.1228	16.16644	689.5963	56.35836	676.2	12.3	98.1	161
_19AR108_Spot_ 20	134.052	11242.74	4.307945	15.84088	1.880858	0.945145	2.412514	0.110667	0.7355	0.304869	676.6195	4.724273	675.5714	11.90326	672.9895	49.15212	676.6	4.7	100.5	16
_19AR108_Spot_ 66	488.1473	43011.41	1.864336	16.17662	0.787443	0.940065	1.18436	0.111258	0.702322	0.592997	680.0513	4.532858	672.9164	5.827204	650.0468	20.48385	680.1	4.5	104.6	56
_19AR108_Spot_ 61	608.1622	5598.25	15.04025	14.77785	0.757905	1.000472	1.311759	0.111372	0.773686	0.589808	680.7134	4.998054	704.0493	6.661356	780.1354	22.26708	680.7	5.0	87.3	51
_19AR108_Spot_ 308	186.078	11725.65	27.20436	15.21265	1.457861	1.000596	1.806604	0.112745	0.913842	0.505834	688.669	5.968847	704.1126	9.174958	754.6001	32.89546	688.7	6.0	91.3	257
_19AR108_Spot_ 573	437.3418	28747.76	6.11433	15.55251	0.909541	0.997113	1.527902	0.113423	1.071375	0.701207	692.5964	7.035595	702.3431	7.745965	734.591	23.06715	692.6	7.0	94.3	469
_19AR108_Spot_ 549	157.8614	11572.31	10.82702	15.37371	1.513933	1.006142	2.786151	0.114101	1.315192	0.472046	696.5219	8.683065	706.9232	14.18928	741.0102	51.94733	696.5	8.7	94.0	450
_19AR108_Spot_ 572	296.1225	21277.67	1.545713	16.12895	0.878909	0.987794	1.441691	0.116802	0.78475	0.544326	712.1324	5.29084	697.5938	7.274519	651.9481	25.95974	712.1	5.3	109.2	468
_19AR108_Spot_ 131	353.4091	8295.007	2.935623	14.27478	0.803998	1.142433	2.009445	0.121523	0.767642	0.382017	739.3261	5.362022	773.6633	10.88043	874.998	38.437	739.3	5.4	84.5	112
_19AR108_Spot_ 35	69.03383	3575.62	1.283138	14.11708	2.894836	1.124032	3.960815	0.122167	0.929273	0.234617	743.0268	6.521677	764.9044	21.28608	830.2668	80.31317	743.0	6.5	89.5	28
_19AR108_Spot_ 80	98.10602	9873.175	5.780884	15.09828	2.306864	1.102486	2.938043	0.123709	1.065613	0.362695	751.8791	7.562529	754.5521	15.64453	763.3812	57.71712	751.9	7.6	98.5	69
_19AR108_Spot_ 6	829.2285	10532.1	3.928887	14.63921	0.608851	1.142621	0.998115	0.123934	0.743428	0.744831	753.1659	5.284526	773.7522	5.4047	834.5374	13.87741	753.2	5.3	90.2	4
_19AR108_Spot_ 273	375.1861	26234.39	5.960198	15.45426	0.706556	1.107136	1.016442	0.124993	0.70641	0.694983	759.2385	5.059543	756.7949	5.422817	750.4808	15.41604	759.2	5.1	101.2	227
_19AR108_Spot_ 397	67.68618	3818.204	4.426277	14.69638	2.64625	1.108989	4.76808	0.125119	0.829449	0.173959	759.959	5.946099	757.6878	25.46346	751.8901	99.18811	760.0	5.9	101.1	333
_19AR108_Spot_ 312	165.8638	14368.35	3.53652	15.48218	1.831321	1.112445	2.172465	0.127084	0.758675	0.349223	771.2078	5.514527	759.3503	11.61702	725.4987	43.17368	771.2	5.5	106.3	261
_19AR108_Spot_ 579	103.9953	3266.257	3.168059	13.99818	1.391114	1.178296	4.357146	0.12761	2.478724	0.568887	774.2172	18.08313	790.5194	23.93591	837.7094	74.65495	774.2	18.1	92.4	475
_19AR108_Spot_ 379	130.722	10943.01	3.956692	14.53965	1.303189	1.201315	2.36114	0.129426	1.019654	0.431848	784.5898	7.532426	801.1928	13.08429	848.5272	44.2915	784.6	7.5	92.5	317
_19AR108_Spot_ 441	287.3425	26034.32	29.67208	14.45648	0.880316	1.252479	1.156365	0.132206	0.721726	0.624133	800.4373	5.432702	824.5228	6.528908	890.9558	18.66049	800.4	5.4	89.8	370
_19AR108_Spot_ 403	82.17896	8868.729	2.383261	14.67853	2.22282	1.212437	2.421592	0.132228	0.713064	0.294461	800.5608	5.368278	806.3101	13.47549	823.1493	48.31756	800.6	5.4	97.3	338
_19AR108_Spot_ 432	228.8988	14159.03	6.809635	14.18636	1.111854	1.292639	1.554389	0.134893	0.917116	0.590017	815.7207	7.027133	842.4668	8.89902	914.5338	25.84534	815.7	7.0	89.2	362
_19AR108_Spot_ 170	526.353	35357.06	2.598775	14.19029	0.652478	1.344509	2.455516	0.139065	2.35172	0.957729	839.3738	18.50865	865.1833	14.29925	932.7863	14.47616	839.4	18.5	90.0	139
_19AR108_Spot_ 123	554.4431	24450.26	9.230035	14.69241	0.637768	1.301443	1.04285	0.13998	0.728925	0.698974	844.5505	5.769929	846.3587	5.987998	852.0127	15.49286	844.6	5.8	99.1	106
_19AR108_Spot_ 86	163.4371	22922.72	2.06333	14.51566	1.114401	1.329354	1.400164	0.14115	0.722246	0.515829	851.1617	5.758922	858.5989	8.113783	878.7614	24.81671	851.2	5.8	96.9	75
_19AR108_Spot_ 111	392.165	2176.296	3.707179	13.47138	0.842074	1.318893	1.648304	0.141349	0.970956	0.589064	852.2843	7.751598	854.0286	9.519382	859.4465	27.64723	852.3	7.8	99.2	96
_19AR108_Spot_ 185	86.7706	6717.33	1.613292	14.20255	1.608322	1.478148	2.019341	0.156709	0.664655	0.329145	938.4633	5.804771	921.4716	12.2307	881.9311	39.44098	881.9	39.4	106.4	153
_19AR108_Spot_ 250	85.64616	7523.005	3.015392	14.13634	1.767387	1.515352	3.242565	0.159459	1.447738	0.446479	953.7721	12.83523	936.6022	19.83758	897.3058	59.86665	897.3	59.9	106.3	206
_19AR108_Spot_ 592	177.441	13329.62	6.597316	14.27067	0.983757	1.499873	1.21731	0.157701	0.683002	0.561074	943.9894	5.997617	930.3347	7.416099	898.9979	20.79872	899.0	20.8	105.0	487
_19AR108_Spot_ 44	305.8343	3665.757	2.115843	13.708	0.714862	1.494761	5.413525	0.157101	1.835605	0.339078	940.6472	16.06595	928.2561	32.94619	899.8184	105.102	899.8	105.1	104.5	36
_19AR108_Spot_ 331	61.37132	4988.883	1.581695	14.0973	2.592984	1.404079	4.596534	0.149868	0.939299	0.204349	900.2233	7.891931	890.6603	27.26513	867.9066	93.33205	900.2	7.9	103.7	277
_19AR108_Spot_ 373	58.0502	5951.788	2.61396	13.8849	1.449833	1.622903	3.191161	0.169198	0.935119	0.293034	1007.69	8.723526	979.1155	20.05143	916.4825	62.7702	916.5	62.8	110.0	312
_19AR108_Spot_ 7	30.35825	5117.304	2.17873	13.78636	3.067308	1.624441	4.246066	0.169132	0.777471	0.183104	1007.326	7.250441	979.7107	26.69217	919.234	85.86352	919.2	85.9	109.6	5
_19AR108_Spot_ 104	719.9499	33358.26	1.216641	14.27745	0.670949	1.500565	1.047973	0.156088	0.715943	0.683169	934.9986	6.231247	930.6156	6.385612	921.1614	15.74021	921.2	15.7	101.5	91
_19AR108_Spot_ 102	212.2448	2225.536	3.82812	13.13881	0.844102	1.562244	3.137477	0.162305	1.284695	0.409467	969.5726	11.56458	955.357	19.42637	923.6753	58.82853	923.7	58.8	105.0	89
_19AR108_Spot_ 53	82.47775	4969.948	5.564404	13.78328	1.628132	1.605078	2.130587	0.166577	0.810291	0.380313	993.2254	7.458673	972.1914	13.32998	925.8538	40.46751	925.9	40.5	107.3	45
_19AR108_Spot_ 466	135.8563	7113.868	3.201332	13.88706	1.031486	1.574339	2.786191	0.163342	0.788474	0.282993	975.3219	7.136672	960.139	17.30276	926.4209	54.91241	926.4	54.9	105.3	393
_19AR108_Spot_ 222	186.5387	5103.211	2.344386	13.7402	1.302472	1.613992	1.745927	0.167106	0.888122	0.508682	996.1459	8.197334	975.6597	10.94637	930.6942	30.8752	930.7	30.9	107.0	185
_19AR108_Spot_ 213	87.39337	9855.244	2.65407	13.96155	1.392778	1.558695	2.342727	0.160929	1.74994	0.746967	961.9389	15.63765	953.9495	14.49184	936.4792	31.94829	936.5	31.9	102.7	178
_19AR108_Spot_ 65	243.5684	23242.92	6.795828	14.03848	0.898955	1.571484	1.211289	0.161924	0.775976	0.64062	967.4615	6.971091	959.0122	7.516433	940.5966	19.04331	940.6	19.0	102.9	55
_19AR108_Spot_ 81	60.45298	7196.913	4.033359	13.78749	2.383142	1.665452	3.135811	0.171594	0.707958	0.225765	1020.887	6.684215	995.4547	19.89742	940.7483	62.60713	940.7	62.6	108.5	70
_19AR108_Spot_ 274	431.8909	39427.92	2.209474	14.11859	0.625978	1.505749	1.057127	0.154754	0.82831	0.783548	927.5554	7.155875	932.7185	6.450274	945.8338	13.4342	945.8	13.4	98.1	228
_19AR108_Spot_ 426	196.4517	14161.25	5.144052	13.96196	0.923703	1.585824	1.465604	0.162757	0.725692	0.495149	972.0775	6.548175	964.6586	9.126708	948.6987	26.04501	948.7	26.0	102.5	356
_19AR108_Spot_ 367	531.9234	44853.47	21.92384	14.04687	0.776816	1.445924	1.301611	0.148081	0.99948	0.767879	890.1998	8.310373	908.1821	7.813079	953.0369	17.05163	953.0	17.1	93.4	307
_19AR108_Spot_ 214	82.69609	8291.544	2.791444	13.77495	1.87186	1.530874	2.679098	0.1565	1.031345	0.38496	937.2957	8.996857	942.8489	16.45604	956.7114	50.55635	956.7	50.6	98.0	179
_19AR108_Spot_ 454	178.3105	3363.497	2.842594	13.29537	1.013168	1.535286	5.734554	0.156933	0.785895	0.137046	939.71	6.872091	944.6173	35.27496	956.944	116.2124	956.9	116.2	98.2	383
_19AR108_Spot_ 181	285.7646	26236.25	4.072665	13.97146	0.704201	1.592681	1.054544	0.162597	0.754223	0.715213	971.1918	6.799877	967.3479	6.577794	959.4951	15.08055	959.5	15.1	101.2	149
_19AR108_Spot_ 593	156.2539	15777.42	3.515508	13.88009	1.211208	1.548156	1.441723	0.15788	0.741664	0.514429	944.9827	6.519113	949.7587	8.894295	961.752	25.26211	961.8	25.3	98.3	488
_19AR108_Spot_ 298	103.9388	5209.133	5.191343	13.51202	1.011226	1.600778	1.694504	0.16311	0.706243	0.416784	974.0372	6.384581	970.5139	10.59049	963.4536	31.45385	963.5	31.5	101.1	249
_19AR108_Spot_ 354	34.09423	4546.902	2.098359	13.46126	3.734793	1.655116	4.669808	0.168613	1.010928	0.216482	1004.462	9.402817	991.5098	29.56627	963.8687	93.16134	963.9	93.2	104.2	298
_19AR108_Spot_ 17	156.6192	4218.487	2.820786	13.43988	1.051521	1.614949	3.226236	0.164406	0.751653	0.232981	981.2161	6.841465	976.0316	20.23388	965.2896	64.07081	965.3	64.1	101.6	14
_19AR108_Spot_ 582	431.4044	46589.53	2.399242	13.95867	0.659302	1.565698	1.023242	0.159373	0.72967	0.713096	953.2895	6.466	956.7249	6.340398	965.5412	14.64255	965.5	14.6	98.7	477

_19AR108_Spot_ 19	160.4123	12001.4	3.165712	13.81866	1.207055	1.629777	1.471801	0.165806	0.771474	0.52417	988.9605	7.073153	981.773	9.261904	966.6128	25.56678	966.6	25.6	102.3	15
_19AR108_Spot_ 94	66.83689	4408.547	2.132006	13.41387	1.451223	1.734219	2.002032	0.175492	0.650595	0.324967	1042.301	6.26134	1021.319	12.89422	977.5268	38.58672	977.5	38.6	106.6	82
_19AR108_Spot_ 87	285.4676	19928.04	2.261286	13.80511	0.941461	1.565957	1.271617	0.158262	0.741777	0.583334	947.1126	6.533747	956.8276	7.879987	980.0973	21.0395	980.1	21.0	96.6	76
_19AR108_Spot_ 281	78.0166	8095.671	3.25893	13.60613	2.046724	1.678869	2.442315	0.169473	0.796905	0.326291	1009.205	7.444483	1000.553	15.54285	982.5475	47.01174	982.5	47.0	102.7	234
_19AR108_Spot_ 309	116.3093	12508.95	2.480459	13.65728	1.171084	1.663388	1.643335	0.167731	0.745931	0.453913	999.5995	6.906983	994.6683	10.4215	984.7055	29.80861	984.7	29.8	101.5	258
_19AR108_Spot_ 505	88.50125	11292.38	1.599692	13.70939	1.764592	1.664051	2.366546	0.167726	1.018447	0.430352	999.5723	9.430121	994.9209	15.01069	985.5781	43.46818	985.6	43.5	101.4	410
_19AR108_Spot_ 82	347.8556	24731.53	2.059346	13.73603	0.73825	1.600347	1.188959	0.161053	0.767556	0.64557	962.6234	6.863478	970.3457	7.429979	988.765	18.47235	988.8	18.5	97.4	71
_19AR108_Spot_ 515	279.958	24150.95	1.548888	13.78135	0.897984	1.71415	1.334816	0.172376	0.970558	0.72711	1025.187	9.199182	1013.838	8.560056	990.3074	18.63802	990.3	18.6	103.5	419
_19AR108_Spot_ 451	82.10748	10565.41	0.932451	13.59176	1.239351	1.705604	2.036329	0.171295	0.65148	0.319929	1019.243	6.141835	1010.636	13.03514	992.9297	39.2119	992.9	39.2	102.7	380
_19AR108_Spot_ 164	214.0092	22388.4	3.198481	13.70161	0.786084	1.700819	1.362945	0.170617	0.865285	0.634864	1015.508	8.129883	1008.839	8.715287	995.2676	21.39423	995.3	21.4	102.0	135
_19AR108_Spot_ 202	55.13897	7494.728	0.682667	13.50678	2.248195	1.650271	2.769841	0.165545	0.709112	0.256012	987.5203	6.492639	989.6552	17.5143	995.2727	54.42449	995.3	54.4	99.2	169
_19AR108_Spot_ 68	225.7737	21477.35	3.725565	13.66603	0.915331	1.725677	1.230585	0.173093	0.750253	0.609672	1029.133	7.136317	1018.142	7.911074	995.4749	19.82376	995.5	19.8	103.4	58
_19AR108_Spot_ 342	133.6487	13825.92	1.605756	13.60668	0.915223	1.734655	1.546267	0.173695	0.66347	0.429079	1032.438	6.329536	1021.481	9.95953	998.9401	28.35789	998.9	28.4	103.4	287
_19AR108_Spot_ 299	455.4843	27274.54	8.087184	13.67527	0.557974	1.708624	0.842868	0.170908	0.586524	0.695866	1017.112	5.518785	1011.769	5.398728	1001.083	12.28026	1001.1	12.3	101.6	250
_19AR108_Spot_ 310	264.9828	22624.37	6.151619	13.64277	0.777896	1.774404	1.140594	0.177479	0.796432	0.698261	1053.186	7.738566	1036.133	7.407159	1001.187	16.5616	1001.2	16.6	105.2	259
_19AR108_Spot_ 117	107.8058	4539.818	1.586267	13.22181	1.269787	1.693836	3.28239	0.169389	0.684622	0.208574	1008.742	6.39285	1006.21	20.95955	1001.565	65.21133	1001.6	65.2	100.7	101
_19AR108_Spot_ 246	319.4751	45651.38	1.625728	13.73552	0.630221	1.707632	0.958088	0.17076	0.662802	0.691797	1016.296	6.231896	1011.397	6.135437	1001.666	14.02791	1001.7	14.0	101.5	204
_19AR108_Spot_ 60	121.2659	8320.133	3.036556	13.46945	1.074197	1.812209	1.771037	0.181067	0.769596	0.434545	1072.798	7.605801	1049.876	11.58876	1003.357	32.37805	1003.4	32.4	106.9	50
_19AR108_Spot_ 64	64.43726	5535.599	2.039373	13.26017	2.347169	1.796734	3.073586	0.179482	0.764916	0.248867	1064.144	7.503459	1044.273	20.0523	1003.789	60.44168	1003.8	60.4	106.0	54
_19AR108_Spot_ 343	286.5834	46149.58	2.932005	13.69911	0.701913	1.734835	0.951948	0.173173	0.599413	0.62967	1029.573	5.703795	1021.548	6.131626	1005.257	14.9885	1005.3	15.0	102.4	288
_19AR108_Spot_ 530	274.4893	27595	7.268326	13.69535	0.901329	1.750286	1.432424	0.17466	0.956285	0.667599	1037.736	9.166155	1027.268	9.256453	1005.903	21.63154	1005.9	21.6	103.2	431
_19AR108_Spot_ 234	206.9766	10483.66	1.892287	13.52118	0.753719	1.780751	1.420859	0.177696	0.730096	0.513841	1054.378	7.101395	1038.454	9.239189	1005.946	24.713	1005.9	24.7	104.8	194
_19AR108_Spot_ 78	118.792	18857.11	2.452102	13.59267	0.932035	1.810216	1.308123	0.180633	0.751438	0.57444	1070.432	7.411287	1049.156	8.556168	1005.986	21.71788	1006.0	21.7	106.4	67
_19AR108_Spot_ 589	172.734	10297.68	4.04994	13.4868	0.921408	1.737424	1.718455	0.173322	0.675549	0.393114	1030.391	6.432988	1022.508	11.07513	1006.538	32.04067	1006.5	32.0	102.4	484
_19AR108_Spot_ 193	217.6072	20225.15	4.586819	13.63335	0.813527	1.711562	1.24598	0.170677	0.710216	0.570006	1015.842	6.674943	1012.87	7.985886	1007.314	20.74912	1007.3	20.7	100.8	160
_19AR108_Spot_ 575	223.9793	19858.06	3.643632	13.61338	0.895499	1.737272	1.243159	0.173177	0.768585	0.618252	1029.592	7.3137	1022.452	8.011533	1008.063	19.81092	1008.1	19.8	102.1	471
_19AR108_Spot_ 293	254.3969	18698.74	3.957945	13.55824	0.847683	1.543729	1.252207	0.15358	0.860085	0.686855	921.001	7.381542	947.9934	7.716398	1012.07	18.44903	1012.1	18.4	91.0	245
_19AR108_Spot_ 295	196.8877	21872.93	3.099671	13.57596	0.814325	1.788278	1.288812	0.177874	0.7172	0.556482	1055.35	6.981887	1041.198	8.393216	1012.475	21.68743	1012.5	21.7	104.2	246
_19AR108_Spot_ 43	128.8036	10814.34	1.850311	13.45225	1.061139	1.725723	1.592793	0.171643	0.644671	0.404742	1021.155	6.088162	1018.159	10.23984	1012.585	29.50449	1012.6	29.5	100.8	35
_19AR108_Spot_ 69	203.4203	22654.13	2.854936	13.53735	0.863449	1.786268	1.260955	0.177509	0.844366	0.669624	1053.35	8.205491	1040.466	8.208477	1014.364	18.96386	1014.4	19.0	103.8	59
_19AR108_Spot_ 142	170.4296	6683.008	2.654063	13.27905	1.001375	1.698792	1.621982	0.168752	0.830063	0.511759	1005.229	7.726008	1008.077	10.36721	1015.134	28.22822	1015.1	28.2	99.0	121
_19AR108_Spot_ 391	410.4986	30173.96	3.09474	13.60084	0.724207	1.762261	1.059715	0.175046	0.708678	0.668745	1039.854	6.80558	1031.679	6.864848	1015.252	15.9631	1015.3	16.0	102.4	327
_19AR108_Spot_ 417	257.8663	16968.02	2.88322	13.53267	0.744433	1.763445	1.294457	0.175076	0.675906	0.522154	1040.017	6.491797	1032.115	8.387614	1016.269	22.34617	1016.3	22.3	102.3	349
_19AR108_Spot_ 220	251.7954	23188.18	10.35605	13.58341	0.602419	1.775164	0.90788	0.176175	0.647419	0.713111	1046.046	6.251395	1036.412	5.896754	1017.006	12.89189	1017.0	12.9	102.9	184
_19AR108_Spot_ 14	301.1541	25068.55	6.483925	13.6073	0.71957	1.734941	1.106246	0.172021	0.719878	0.65074	1023.237	6.8112	1021.587	7.125664	1018.914	17.00923	1018.9	17.0	100.4	11
_19AR108_Spot_ 531	96.50593	11003.8	1.521532	13.45141	1.331124	1.780264	1.712725	0.176322	0.947305	0.553098	1046.848	9.153513	1038.276	11.13611	1021.134	28.90507	1021.1	28.9	102.5	432
_19AR108_Spot_ 586	77.32886	7612.354	2.035198	13.29671	1.517776	1.698698	2.31261	0.168184	0.762596	0.329756	1002.1	7.077625	1008.041	14.78172	1021.838	44.20396	1021.8	44.2	98.1	481
_19AR108_Spot_ 570	189.9912	17576.84	3.194574	13.50375	1.101554	1.77664	1.550768	0.175883	0.692848	0.446777	1044.445	6.680609	1036.951	10.0756	1022.048	28.08206	1022.0	28.1	102.2	466
_19AR108_Spot_ 566	176.9843	17828.85	2.118828	13.50066	1.012789	1.768978	1.351585	0.174924	0.659262	0.487769	1039.184	6.327259	1034.146	8.767726	1024.369	23.8927	1024.4	23.9	101.4	462
_19AR108_Spot_ 470	351.4694	18878.21	18.24432	13.49665	0.688094	1.827243	1.124351	0.180558	0.714171	0.635185	1070.024	7.041266	1055.29	7.378577	1025.792	17.56713	1025.8	17.6	104.3	395
_19AR108_Spot_ 1	102.5316	9782.49	2.09839	13.33424	0.99863	1.758996	2.125076	0.173768	0.817545	0.384713	1032.84	7.802226	1030.479	13.75767	1026.33	39.69887	1026.3	39.7	100.6	1
_19AR108_Spot_ 113	134.0697	17578.66	2.296528	13.46843	1.123955	1.776935	1.458891	0.175416	0.891556	0.611119	1041.884	8.57719	1037.059	9.479192	1027.763	23.37217	1027.8	23.4	101.4	97
_19AR108_Spot_ 106	1276.9	9600.156	2.932326	13.3598	0.579415	1.744412	0.93847	0.17216	0.704798	0.751007	1024.002	6.673109	1025.097	6.056964	1028.298	12.53074	1028.3	12.5	99.6	93
_19AR108_Spot_ 277	408.3098	34553.12	1.959974	13.54298	0.675554	1.744289	1.079981	0.172041	0.667995	0.618525	1023.345	6.320921	1025.051	6.970138	1029.556	17.1757	1029.6	17.2	99.4	231
_19AR108_Spot_ 26	206.2512	19951.83	1.74296	13.4614	0.836656	1.78473	1.168962	0.175998	0.682934	0.584223	1045.074	6.588679	1039.906	7.607256	1029.919	19.19923	1029.9	19.2	101.5	21
_19AR108_Spot_ 127	327.2554	38959.05	4.24561	13.51837	0.668099	1.7822	1.05669	0.175738	0.735694	0.696225	1043.648	7.08876	1038.982	6.873095	1030.041	15.35237	1030.0	15.4	101.3	108
_19AR108_Spot_ 42	496.0991	57720.12	4.73618	13.53121	0.609881	1.774199	0.891337	0.17494	0.602094	0.675496	1039.273	5.779054	1036.058	5.788167	1030.14	13.28574	1030.1	13.3	100.9	34
_19AR108_Spot_ 136	245.5179	19457.06	8.045907	13.43196	0.879021	1.783112	1.539458	0.175765	0.925832	0.601401	1043.797	8.922002	1039.315	10.01521	1030.763	24.88224	1030.8	24.9	101.3	116
_19AR108_Spot_ 419	259.9973	20340.8	5.88581	13.46664	0.718348	1.705953	1.166999	0.168104	0.887857	0.760804	1001.655	8.236787	1010.767	7.470595	1031.428	15.32716	1031.4	15.3	97.1	351
_19AR108_Spot_ 92	266.4243	41989.89	2.823673	13.56618	0.748226	1.770063	1.063917	0.174384	0.748975	0.703979	1036.223	7.16941	1034.543	6.903087	1031.854	15.29095	1031.9	15.3	100.4	81
_19AR108_Spot_ 344	390.2269	45055.26	3.454824	13.51002	0.591599	1.803942	0.907324	0.177534	0.679215	0.748592	1053.49	6.601375	1046.887	5.927216	1033.992	12.1739	1034.0	12.2	101.9	289
_19AR108_Spot_ 115	217.1681	2837.944	2.683871	12.69751	1.1186	1.738201	1.613748	0.170881	0.881385	0.546173	1016.963	8.292112	1022.797	10.40197	1036.16	27.3225	1036.2	27.3	98.1	99
_19AR108_Spot_ 157	1041.128	49812.56	4.308191	13.47206	0.701692	1.743452	1.184322	0.171324	0.891076	0.752393	1019.402	8.401839	1024.742	7.642238	1037.021	15.77374	1037.0	15.8	98.3	129
_19AR108_Spot_ 448	531.2767	58502.07	1.91328	13.50967	0.678297	1.742781	0.941458	0.171231	0.644346	0.684413	1018.89	6.072635	1024.493	6.07418	1037.341	13.87942	1037.3	13.9	98.2	377
_19AR108_Spot_ 75	691.6561	57450.97	2.585295	13.46772	0.750841	1.799976	1.104874	0.176827	0.77172	0.698469	1049.618	7.47506	1045.449	7.212113	1037.606	15.98543	1037.6	16.0	101.2	64
_19AR108_Spot_ 215	279.2027	30672.43	6.930252	13.46837	0.780908	1.778228	1.177073	0.174616	0.673967	0.572579	1037.495	6.458707	1037.532	7.649991	1038.471	19.50377	1038.5	19.5	99.9	180
_19AR108_Spot_ 121	436.1466	54605.5	4.817035	13.46762	0.590581	1.787432	0.860588	0.175291	0.622328	0.723142	1041.196	5.983441	1040.89	5.603444	1041.108	12.01542	1041.1	12.0	100.0	105
_19AR108_Spot_ 171	570.5432	24945.81	5.611304	13.4273	0.622798	1.782441	2.313638	0.174786	2.222617	0.960659	1038.425	21.31726	1039.071	15.05034	1041.289	12.98637	1041.3	13.0	99.7	140
_19AR108_Spot_ 62	103.5729	12538.67	2.242452	13.28008	1.152031	1.825669	1.49205	0.17896	0.748182	0.501445	1061.292	7.32121	1054.724	9.788758	1042.013	26.06977	1042.0	26.1	101.9	52
_19AR108_Spot_ 73	149.3733	13047.98	3.358998	13.27977	0.956743	1.84527	1.637078	0.180844	0.803178	0.490617	1071.582	7.929416	1061.744	10.78083	1042.436	28.80546	1042.4	28.8	102.8	63
_19AR108_Spot_ 168	60.70701	6379.658	3.565961	13.11449	1.094618	1.911676	3.648657	0.187158	2.263822	0.620453	1105.963	23.00715	1085.169	24.32861	1044.519	57.72789	1044.5	57.7	105.9	137
_19AR108_Spot_ 487	393.13	39785.14	4.527044	13.53112	0.661345	1.704392	1.127938	0.166856	0.885537	0.785094	994.7637	8.162989	1010.181	7.218093	1044.627	14.11121	1044.6	14.1	95.2	399
_19AR108_Spot_ 119	345.529	36300.98	3.404715	13.41968	0.617523	1.824193	0.973909	0.178572	0.626417	0.643199	1059.17	6.118426	1054.194	6.387491	1044.758	15.06145	1044.8	15.1	101.4	103
_19AR108_Spot_ 380	1005.9	95516.82	2.853778	13.43772	0.593986	1.83	0.974978	0.179103	0.753523	0.772862	1062.072	7.378464	1056.279	6.401694	1045.221	12.49782	1045.2	12.5	101.6	318
_19AR108_Spot_ 385	273.0568	25806.34	4.70856	13.37746	0.743813	1.838369	1.033335	0.179808	0.704346	0.681624	1065.926	6.919938	1059.278	6.795811	1046.502	15.26716	1046.5	15.3	101.9	322
_19AR108_Spot_ 206	192.6503	23120.23	2.968825	13.38328	0.738454	1.826193	1.026692	0.178571	0.681296	0.663583	1059.162	6.654396	1054.912	6.736297	1047.025	15.50703	1047.0	15.5	101.2	171
_19AR108_Spot_ 201	81.73052	8814.2	3.210118	13.20679	1.353729	1.825614	1.843663	0.1785	0.879156	0.476853	1058.774	8.584055	1054.704	12.09562	1047.187	32.67768	1047.2	32.7	101.1	168
_19AR108_Spot_ 91	253.7588	30644.39	3.148512	13.43174	0.67362	1.863218	1.118352	0.182042	0.712101	0.636741	1078.119	7.069651	1068.128	7.389672	1048.678	17.4032	1048.7	17.4	102.8	80
_19AR108_Spot_ 120	175.1303	20284.09	2.007975	13.31641	1.094541	1.821509	1.531772	0.177665	0.674917	0.440612	1054.206	6.563704	1053.228	10.04126	1052.099	27.7068	1052.1	27.7	100.2	104
_19AR108_Spot_ 437	132.0054	19606.16	3.152457	13.32814	1.119581	1.789175	1.46699	0.174504	0.701251	0.47802	1036.88	6.716496	1041.525	9.555355	1052.18	25.96529	1052.2	26.0	98.5	366
_19AR108_Spot_ 394	107.6931	12606.72	2.685975	13.22925	1.436114	1.927484	1.732414	0.187629	0.605859	0.349719	1108.518	6.170335	1090.667	11.58235	1056.095	32.69271	1056.1	32.7	105.0	330
_19AR108_Spot_ 279	64.13011	6026.177	2.866819	12.98326	1.541971	1.912249	2.349981	0.185456	0.879334	0.374188	1096.712	8.868044	1085.369	15.66912	1063.567	43.85697	1063.6	43.9	103.1	232
_19AR108_Spot_ 458	516.6904	30431.45	3.190909	13.27539	0.661167	1.831466	1.024787	0.177387	0.666072	0.649961	1052.683	6.46907	1056.805	6.730655	1066.222	15.65509	1066.2	15.7	98.7	386
_19AR108_Spot_ 557	290.8054	5097.215	5.639572	12.88807	0.846145	1.512599	1.534467	0.146384	1.084603	0.706827	880.6642	8.927992	935.4903	9.379947	1067.851	21.81872	1067.9	21.8	82.5	455
_19AR108_Spot_ 337	283.3542	31746.27	8.935904	13.22857	0.617455	1.685228	0.991081	0.16278	0.706062	0.712416	972.2063	6.371828	1002.96	6.315706	1071.688	13.96568	1071.7	14.0	90.7	282
_19AR108_Spot_ 57	76.26294	6557.379	3.572456	12.9807	1.422163	2.01727	2.009742	0.19477	0.739463	0.367939	1147.164	7.770926	1121.341	13.64414	1072.537	37.54172	1072.5	37.5	107.0	48
_19AR108_Spot_ 495	116.8257	17507.49	5.081002	13.26081	1.096973	1.885689	1.41407	0.182025	0.696185	0.492327	1078.029	6.911105	1076.066	9.382825	1072.986	24.71752	1073.0	24.7	100.5	407
_19AR108_Spot_ 355	460.4143	53633.56	6.988977	13.26656	0.664467	1.886776	0.955153	0.182071	0.678696	0.710563	1078.276	6.738916	1076.448	6.338924	1073.642	13.49625	1073.6	13.5	100.4	299
_19AR108_Spot_ 562	383.5299	21987.87	2.006434	13.20004	0.674076	1.62986	1.024554	0.157059	0.742175	0.724389	940.4123	6.494299	981.8049	6.447456	1076.446	14.16094	1076.4	14.2	87.4	459
_19AR108_Spot_ 199	82.33215	12788.83	2.7719	13.09416	1.868559	1.880922	2.218623	0.180902	0.794592	0.358147	1071.898	7.846776	1074.387	14.70899	1080.335	41.58028	1080.3	41.6	99.2	166
_19AR108_Spot_ 576	295.4959	29259.31	2.262247	13.14024	0.658706	1.866191	1.089919	0.17895	0.676132	0.620351	1061.237	6.615862	1069.182	7.205803	1086.316	17.12866	1086.3	17.1	97.7	472
_19AR108_Spot_ 585	76.96921	8851.928	2.024145	12.91313	1.219396	2.031063	2.337506	0.194459	0.79304	0.339268	1145.484	8.322816	1125.972	15.90547	1089.378	44.04351	1089.4	44.0	105.2	480
_19AR108_Spot_ 268	271.9975	25813.6	2.352441	13.11452	0.75989	1.9838	1.14858	0.189814	0.769618	0.67006	1120.369	7.914863	1110.014	7.754032	1090.638	17.06765	1090.6	17.1	102.7	222
_19AR108_Spot_ 523	82.34337	8200.895	6.240939	12.92291	8.691127	1.965027	12.26434	0.187672	8.632451	0.703866	1108.752	87.9392	1103.606	82.71286	1094.366	174.743	1094.4	174.7	101.3	425
_19AR108_Spot_ 578	303.0239	32729.56	3.588361	13.06348	0.747875	1.98314	1.070777	0.189067	0.672303	0.627865	1116.319	6.89117	1109.79	7.227962	1097.872	16.67424	1097.9	16.7	101.7	474
_19AR108_Spot_ 459	134.8085	18344.08	1.807507	12.98219	1.041889	2.026605	1.41764	0.192696	0.697421	0.491959	1135.962	7.263668	1124.477	9.638788	1103.215	24.67944	1103.2	24.7	103.0	387
_19AR108_Spot_ 543	429.7826	55432.48	3.905774	13.10496	0.628349	1.972669	0.914422	0.187421	0.646904	0.707445	1107.39	6.582209	1106.219	6.161545	1104.781	12.93903	1104.8	12.9	100.2	444
_19AR108_Spot_ 596	228.0223	22721.52	2.132434	12.93722	0.648849	1.965743	1.089942	0.186044	0.656372	0.602208	1099.91	6.637175	1103.851	7.335575	1112.476	17.36181	1112.5	17.4	98.9	490
_19AR108_Spot_ 525	175.0792	1619.591	3.283106	11.71518	8.668536	2.16771	12.86921	0.204502	8.636565	0.671103	1199.462	94.53248	1170.746	89.65282	1118.91	190.7469	1118.9	190.7	107.2	426
_19AR108_Spot_ 556	345.1032	9834.982	4.084432	12.72579	0.666945	2.067089	0.980848	0.193806	0.677335	0.69056	1141.96	7.088512	1137.969	6.712287	1131.252	14.12576	1131.3	14.1	100.9	454
_19AR108_Spot_ 27	421.9043	44554.2	6.538338	12.86733	0.558229	2.047632	0.907867	0.191945	0.688347	0.758202	1131.902	7.145722	1131.507	6.193656	1131.596	11.77392	1131.6	11.8	100.0	22
_19AR108_Spot_ 253	183.2388	18745.57	2.591346	12.73853	0.804671	2.111052	1.274553	0.196873	0.721087	0.565757	1158.501	7.646178	1152.42	8.781937	1141.883	20.87722	1141.9	20.9	101.5	209
_19AR108_Spot_ 528	58.76678	7176.165	3.146055	12.56311	8.85359	2.112635	12.44846	0.196883	8.62962	0.693228	1158.551	91.5155	1152.937	85.99603	1143.263	178.6748	1143.3	178.7	101.3	429
_19AR108_Spot_ 109	147.22	19517.33	4.36833	12.67416	0.907265	2.17559	1.53014	0.201755	0.90625	0.592266	1184.74	9.807868	1173.268	10.64461	1153.011	24.47257	1153.0	24.5	102.8	95
_19AR108_Spot_ 49	414.6485	49587.96	2.524611	12.74761	0.626177	2.11568	0.976006	0.19537	0.702534	0.719805	1150.399	7.401861	1153.93	6.729545	1161.411	13.45071	1161.4	13.5	99.1	41
_19AR108_Spot_ 493	210.5571	23874.71	4.151138	12.71636	0.77252	2.142044	1.142293	0.197609	0.726212	0.63575	1162.462	7.724552	1162.485	7.907372	1163.372	17.47505	1163.4	17.5	99.9	405
_19AR108_Spot_ 46	354.6161	50216.19	2.099171	12.69142	0.628888	2.116715	0.995394	0.195142	0.760457	0.763975	1149.168	8.004303	1154.267	6.864312	1164.698	12.74505	1164.7	12.7	98.7	38
_19AR108_Spot_ 395	410.9545	40582.77	2.610736	12.65164	0.665291	2.185942	0.997919	0.201434	0.684819	0.686247	1183.019	7.401621	1176.573	6.952369	1165.58	14.39307	1165.6	14.4	101.5	331
_19AR108_Spot_ 16	964.402	130281	5.754099	12.63765	0.670053	2.184965	0.97481	0.199923	0.699401	0.717474	1174.906	7.511969	1176.262	6.790411	1179.637	13.44387	1179.6	13.4	99.6	13
_19AR108_Spot_ 233	113.9972	14685.56	2.277875	12.48079	0.888564	2.280681	1.296941	0.208583	0.782814	0.603585	1221.264	8.709226	1206.327	9.155085	1180.563	20.46215	1180.6	20.5	103.4	193
_19AR108_Spot_ 76	586.7346	61077.99	1.108466	12.50723	0.737668	2.232133	1.108818	0.203553	0.803828	0.724942	1194.377	8.763817	1191.189	7.775518	1186.286	15.07007	1186.3	15.1	100.7	65
_19AR108_Spot_ 462	320.0708	28613.92	4.672624	12.45229	0.728656	2.044688	1.1176	0.186061	0.760886	0.680822	1100.004	7.694622	1130.526	7.620939	1190.491	16.15854	1190.5	16.2	92.4	390
_19AR108_Spot_ 297	136.3175	15270.83	6.455567	12.12026	0.752658	2.472469	1.275121	0.220077	0.594934	0.466571	1282.284	6.917917	1264.016	9.219043	1233.934	22.12621	1233.9	22.1	103.9	248
_19AR108_Spot_ 418	405.9671	51555.4	3.977653	12.21858	0.567036	2.39822	0.840545	0.213177	0.606779	0.721888	1245.719	6.873288	1242.069	6.023288	1236.616	11.42545	1236.6	11.4	100.7	350
_19AR108_Spot_ 209	292.4881	34015.22	2.884189	12.12095	0.734936	2.492194	1.103441	0.220137	0.745197	0.675339	1282.599	8.667104	1269.767	7.995976	1248.942	15.92199	1248.9	15.9	102.7	174
_19AR108_Spot_ 232	54.03886	2050.949	3.682475	11.20814	1.050481	2.59655	5.119899	0.227992	0.708168	0.138317	1323.968	8.475766	1299.665	37.54912	1260.59	99.15674	1260.6	99.2	105.0	192
_19AR108_Spot_ 384	417.0262	30292.99	3.11242	12.01882	0.685275	2.184107	1.157799	0.191583	0.802586	0.6932	1129.946	8.318466	1175.988	8.064149	1262.606	16.28631	1262.6	16.3	89.5	321
_19AR108_Spot_ 436	82.5674	9751.471	2.605946	11.7828	1.326042	2.555553	2.186318	0.22199	0.686161	0.313843	1292.383	8.035453	1288.024	15.9572	1281.595	40.45138	1281.6	40.5	100.8	365
_19AR108_Spot_ 444	195.9124	27071.35	6.000944	11.88189	0.705314	2.538544	1.018704	0.219851	0.652357	0.640379	1281.089	7.579242	1283.155	7.420716	1287.445	15.22251	1287.4	15.2	99.5	373
_19AR108_Spot_ 377	451.3867	71173.09	2.555948	11.85171	0.59064	2.578283	0.908376	0.222519	0.677617	0.745965	1295.172	7.950863	1294.495	6.645956	1294.2	11.76734	1294.2	11.8	100.1	315
_19AR108_Spot_ 239	167.3251	15619.2	3.248026	11.70585	0.811391	2.531687	1.152716	0.216773	0.782984	0.679252	1264.801	8.992223	1281.186	8.390537	1309.596	16.41797	1309.6	16.4	96.6	199
_19AR108_Spot_ 84	150.9309	17574.88	2.158003	11.58272	0.835538	2.752919	1.238144	0.23328	0.718102	0.579983	1351.666	8.756295	1342.879	9.22226	1329.729	19.52106	1329.7	19.5	101.6	73
_19AR108_Spot_ 559	157.6569	22052.85	1.536568	11.55583	0.923005	2.428384	1.350121	0.204742	0.853278	0.632001	1200.746	9.348076	1251.042	9.710546	1339.495	20.22072	1339.5	20.2	89.6	457
_19AR108_Spot_ 590	242.2227	36337.9	1.78225	11.41147	0.756165	2.807172	1.080194	0.23362	0.727487	0.673478	1353.442	8.881207	1357.452	8.087374	1364.592	15.37821	1364.6	15.4	99.2	485
_19AR108_Spot_ 282	579.7474	96707.08	3.060019	11.4577	0.567636	2.90328	0.869303	0.24128	0.653948	0.752267	1393.35	8.194342	1382.766	6.565479	1367.288	11.02692	1367.3	11.0	101.9	235
_19AR108_Spot_ 364	274.0329	23677.62	3.3224	11.34668	0.729706	2.585895	1.106772	0.214402	0.707445	0.639197	1252.226	8.051499	1296.652	8.104207	1371.789	16.37642	1371.8	16.4	91.3	305
_19AR108_Spot_ 352	536.5073	11221.06	5.025832	11.19106	0.638724	2.811173	0.981319	0.231418	0.705663	0.719097	1341.925	8.548844	1358.518	7.349818	1385.544	13.09466	1385.5	13.1	96.9	296
_19AR108_Spot_ 180	63.61487	7007.762	1.965983	10.93309	1.212173	2.944379	2.131541	0.238623	0.742742	0.348453	1379.534	9.224218	1393.402	16.15755	1415.508	38.21249	1415.5	38.2	97.5	148
_19AR108_Spot_ 346	114.9235	16844.52	2.941596	11.06524	0.736328	3.021102	1.151418	0.244806	0.701138	0.608935	1411.633	8.888786	1412.962	8.784036	1415.781	17.4662	1415.8	17.5	99.7	291
_19AR108_Spot_ 2	92.07759	10937.02	2.18392	10.89053	1.016892	3.137828	1.449478	0.251751	0.829887	0.572542	1447.498	10.75946	1442.018	11.16132	1434.757	22.66837	1434.8	22.7	100.9	2
_19AR108_Spot_ 280	106.2502	24449.69	1.852755	10.96038	0.74171	3.261977	1.162002	0.260518	0.718532	0.618357	1492.493	9.573119	1472.035	9.03063	1443.466	17.39868	1443.5	17.4	103.4	233
_19AR108_Spot_ 262	265.6145	45138.88	1.85277	10.71488	0.725014	3.334853	1.128362	0.259855	0.852051	0.755122	1489.1	11.32908	1489.25	8.81438	1490.27	14.00512	1490.3	14.0	99.9	216
_19AR108_Spot_ 173	152.7999	21860.51	2.832311	10.53861	0.846083	3.352557	2.436691	0.257721	2.261665	0.928171	1478.174	29.87554	1493.388	19.05957	1515.86	17.10867	1515.9	17.1	97.5	142
_19AR108_Spot_ 390	246.4109	36644.87	3.012442	10.39571	0.678664	3.584158	1.059368	0.271436	0.749071	0.707093	1548.089	10.30897	1546.029	8.410354	1544.014	14.08164	1544.0	14.1	100.3	326
_19AR108_Spot_ 286	165.8648	29718.28	3.289392	10.33971	0.680682	3.751951	1.015881	0.282625	0.655975	0.64572	1604.567	9.317857	1582.531	8.14455	1554.085	14.56232	1554.1	14.6	103.2	238
_19AR108_Spot_ 370	54.32865	881.513	0.870485	8.436791	1.297291	4.601383	4.387594	0.324307	0.957391	0.218204	1810.727	15.11387	1749.519	36.6132	1677.933	79.13623	1677.9	79.1	107.9	310
_19AR108_Spot_ 440	300.4791	54448.78	1.817983	9.586614	0.645132	3.482495	0.993674	0.242413	0.739965	0.744676	1399.229	9.307213	1523.257	7.838866	1700.88	12.21531	1700.9	12.2	82.3	369
_19AR108_Spot_ 72	278.4858	38673.57	2.600662	9.333997	0.739048	4.6838	1.135261	0.319245	0.826639	0.728149	1786.041	12.89535	1764.35	9.499435	1739.548	14.26296	1739.5	14.3	102.7	62
_19AR108_Spot_ 330	331.7093	51202.6	1.769158	9.275897	0.669192	4.05236	1.211972	0.273763	1.004177	0.828548	1559.874	13.91282	1644.774	9.870743	1755.818	12.41502	1755.8	12.4	88.8	276
_19AR108_Spot_ 304	223.3403	10623.21	1.218365	8.820437	0.663786	4.780012	1.255636	0.310026	0.994709	0.792196	1740.837	15.17518	1781.394	10.5441	1830.017	13.88966	1830.0	13.9	95.1	255
_19AR108_Spot_ 399	152.7374	27081.87	1.205141	8.845664	0.667929	4.841995	1.14485	0.312146	0.857492	0.748999	1751.261	13.14998	1792.225	9.635066	1841.012	13.73107	1841.0	13.7	95.1	335
_19AR108_Spot_ 128	428.9907	90571.54	28.28755	8.81901	0.484569	5.26658	0.818753	0.337724	0.649982	0.793868	1875.709	10.57828	1863.462	6.986951	1850.6	9.001287	1850.6	9.0	101.4	109
_19AR108_Spot_ 303	156.0573	35598.5	1.089545	8.756661	0.741063	5.250676	1.035059	0.335406	0.698706	0.67504	1864.531	11.31282	1860.882	8.82865	1857.576	13.79581	1857.6	13.8	100.4	254
_19AR108_Spot_ 565	76.04755	11947.9	1.662992	8.724598	0.737835	5.345176	1.325791	0.341244	1.044434	0.787782	1892.653	17.13002	1876.118	11.34074	1858.626	14.7512	1858.6	14.8	101.8	461
_19AR108_Spot_ 461	125.3711	22947.04	1.378565	8.749648	0.689556	5.158643	0.980599	0.328958	0.659212	0.672254	1833.328	10.51895	1845.821	8.340296	1860.7	13.10986	1860.7	13.1	98.5	389
_19AR108_Spot_ 132	211.6564	39930.44	1.138901	8.69995	0.526141	5.413106	0.883206	0.343287	0.696749	0.788886	1902.462	11.47845	1886.931	7.569692	1870.651	9.791262	1870.7	9.8	101.7	113
_19AR108_Spot_ 256	136.0968	25609.03	0.585628	8.688895	0.601184	5.435791	0.96899	0.344021	0.681856	0.703677	1905.987	11.25099	1890.516	8.310355	1874.337	12.41453	1874.3	12.4	101.7	212
_19AR108_Spot_ 237	106.1209	23048.4	1.149732	8.677611	0.622844	5.566742	1.000192	0.351733	0.750533	0.750389	1942.87	12.58958	1910.969	8.609435	1877.286	11.91686	1877.3	11.9	103.5	197
_19AR108_Spot_ 442	195.8955	48016.99	0.857813	8.698165	0.670733	5.241537	1.058926	0.331053	0.817321	0.77184	1843.484	13.1043	1859.396	9.029715	1878.005	12.135	1878.0	12.1	98.2	371
_19AR108_Spot_ 257	239.1382	55399.66	0.447519	8.694986	0.526247	5.520856	0.893038	0.34868	0.719571	0.805756	1928.292	11.99253	1903.849	7.677329	1878.082	9.532559	1878.1	9.5	102.7	213
_19AR108_Spot_ 521	232.4602	37531.1	1.318179	8.677268	8.61819	5.310556	12.19538	0.334284	8.627077	0.707405	1859.114	139.3533	1870.563	104.5754	1884.075	155.529	1884.1	155.5	98.7	423
_19AR108_Spot_ 517	290.178	57126.88	1.380015	8.677516	0.725005	5.294068	1.1124	0.333167	0.841907	0.756838	1853.714	13.56315	1867.906	9.500836	1884.504	13.09229	1884.5	13.1	98.4	421
_19AR108_Spot_ 30	28.2222	4777.543	1.122	7.704805	1.343153	6.716067	2.071801	0.384025	0.782763	0.377818	2095.06	14.00119	2074.737	18.31235	2055.373	33.86201	2055.4	33.9	101.9	24
_19AR108_Spot_ 252	81.87135	16157.16	1.52075	7.811365	0.68894	6.885266	1.123825	0.39248	0.757743	0.674254	2134.318	13.76794	2096.762	9.964304	2060.853	14.64007	2060.9	14.6	103.6	208
_19AR108_Spot_ 207	129.1461	29005.25	2.384123	7.751286	0.613326	6.812032	0.926624	0.38488	0.678661	0.732401	2099.039	12.15863	2087.287	8.204564	2076.465	11.10891	2076.5	11.1	101.1	172
_19AR108_Spot_ 538	29.28216	5674.643	0.964334	7.540951	1.506585	7.139527	2.128803	0.396186	0.87391	0.410517	2151.453	15.98606	2128.986	18.96209	2108.116	34.06493	2108.1	34.1	102.1	439
_19AR108_Spot_ 376	147.3332	31453.3	3.184475	7.34513	0.67804	7.530442	1.027687	0.403072	0.763066	0.742508	2183.171	14.13136	2176.617	9.211955	2171.189	11.99556	2171.2	12.0	100.6	314
_19AR108_Spot_ 146	215.6804	65026.17	1.000872	5.746647	0.698843	10.20561	1.153026	0.42661	0.914782	0.793375	2290.419	17.63449	2453.586	10.66322	2592.436	11.70668	2592.4	11.7	88.4	124
_19AR108_Spot_ 567	104.8982	20872.8	1.467821	5.675229	0.623666	12.04729	1.016092	0.497032	0.741607	0.729862	2601.028	15.87253	2608.093	9.526753	2614.294	11.56388	2614.3	11.6	99.5	463
_19AR108_Spot_ 413	213.2576	105639.9	1.728553	5.514554	0.645843	12.58777	0.968221	0.503798	0.720619	0.744271	2630.099	15.56295	2649.307	9.107863	2664.717	10.71399	2664.7	10.7	98.7	346
_19AR108_Spot_ 175	410.2971	91765.52	3.467139	5.435422	0.629019	11.56207	1.09389	0.455242	0.89407	0.817331	2418.516	18.0301	2569.611	10.22334	2691.766	10.41268	2691.8	10.4	89.8	144
_19AR108_Spot_ 483	119.8793	35406.95	1.410696	5.439232	0.61815	12.64132	0.922827	0.496312	0.677903	0.734594	2597.927	14.49504	2653.301	8.683544	2696.501	10.34008	2696.5	10.3	96.3	398
_19AR108_Spot_ 353	749.8752	201835.3	4.196058	5.170562	0.59389	14.38521	0.898683	0.539286	0.674136	0.750138	2780.459	15.22533	2775.455	8.532171	2772.522	9.747887	2772.5	9.7	100.3	297

19AR109 -26.94369 -65.76593 1900 19 0.555

19AR109	Spot 599	970.1788	161.9986	4.993606	3.679611	23.67933	0.006995	28.33208	0.000258	7.777017	0.274495	1.660616	0.12913	7.077918	1.998351	2801.506	455.3061	1.7	0.1	0.1	531
19AR109	Spot 105	1170.341	206.5344	0.50234	6.806828	40.7203	0.003334	75.00976	0.00031	7.532817	0.100424	1.996208	0.150347	3.379705	2.5309	1149.651	NA	2.0	0.2	0.2	91
19AR109	Spot 590	2031.56	461.0288	3.747818	4.569321	20.79127	0.008268	23.11243	0.000313	7.220596	0.312412	2.014968	0.14547	8.361103	1.924521	2758.569	365.5281	2.0	0.1	0.1	523
19AR109	Spot 72	2276.199	657.9279	0.923681	7.66269	31.40943	0.004918	35.20837	0.000326	3.23177	0.09179	2.102216	0.067928	4.981765	1.749704	1789.678	660.4591	2.1	0.1	0.1	70
19AR109	Spot 237	1159.01	151.3247	1.937564	6.470903	25.41313	0.002675	160.9381	0.000334	9.338805	0.058027	2.152943	0.201026	2.712798	4.360125	533.9994	NA	2.2	0.2	0.4	210
19AR109	Spot 30	2534.475	704.5484	0.782204	7.86405	12.92041	0.004978	18.52731	0.000335	6.88459	0.371592	2.162111	0.148827	5.041712	0.931778	1760.285	316.9071	2.2	0.1	0.1	29
19AR109	Spot 164	2034.921	479.7987	2.637352	7.338782	21.08666	0.005001	31.18548	0.000338	5.566385	0.178493	2.175408	0.121071	5.065635	1.57581	1757.749	575.5175	2.2	0.1	0.1	143
19AR109	Spot 429	1612.574	539.6214	2.24981	4.556903	28.13153	0.009166	29.87398	0.000338	7.393379	0.247486	2.179916	0.161142	9.264841	2.755194	2798.54	485.2478	2.2	0.2	0.1	379
19AR109	Spot 445	2104.649	376.047	1.869809	8.502155	34.30823	0.003734	38.56555	0.00034	4.27301	0.110799	2.191883	0.093643	3.784753	1.456895	1189.019	786.2355	2.2	0.1	0.2	393
19AR109	Spot 442	1876.662	550.4166	5.374256	6.258891	24.9415	0.006389	27.18498	0.000342	6.123556	0.225255	2.204056	0.134944	6.466726	1.752394	2171.278	470.474	2.2	0.1	0.1	390
19AR109	Spot 576	1316.513	510.0254	4.949101	4.94954	24.65373	0.008391	26.58986	0.000343	6.451091	0.242615	2.20913	0.142489	8.485033	2.246761	2630.982	436.8966	2.2	0.1	0.1	509
19AR109	Spot 120	2397.113	700.5601	2.670913	9.908323	12.58842	0.003816	14.18777	0.000343	4.440021	0.312947	2.210628	0.098136	3.867208	0.547627	1214.751	266.2381	2.2	0.1	0.2	105
19AR109	Spot 489	2670.644	881.207	4.612019	9.308747	14.56858	0.004331	17.66951	0.000345	3.522205	0.199338	2.221539	0.078234	4.388628	0.773776	1450.414	332.0484	2.2	0.1	0.2	430
19AR109	Spot 157	1171.25	362.495	4.180133	4.95839	20.09147	0.007919	24.93687	0.000345	8.319771	0.333633	2.221767	0.184814	8.00906	1.989355	2524.621	400.9242	2.2	0.2	0.1	136
19AR109	Spot 334	1953.545	552.8138	1.982082	5.375532	24.99259	0.008005	26.55911	0.000356	5.115834	0.192621	2.294069	0.11734	8.095357	2.14151	2488.842	447.68	2.3	0.1	0.1	291
19AR109	Spot 134	2336.088	459.5886	1.812742	8.088249	24.43803	0.004668	36.41211	0.000364	4.454579	0.122338	2.344741	0.104429	4.728675	1.717809	1489.921	708.3893	2.3	0.1	0.2	118
19AR109	Spot 425	957.0155	206.781	1.881653	4.347093	27.48187	0.008529	33.1269	0.000366	6.76119	0.2041	2.358328	0.159422	8.62314	2.844491	2548.973	559.9779	2.4	0.2	0.1	375
19AR109	Spot 506	1435.743	528.3795	2.432723	8.323923	23.74906	0.004741	32.50265	0.000368	7.265078	0.223523	2.371801	0.172282	4.802135	1.557137	1497.443	615.1636	2.4	0.2	0.2	447
19AR109	Spot 561	2711.028	1045.384	3.052569	10.2836	12.42241	0.004394	17.20238	0.000382	4.522373	0.262892	2.462755	0.111354	4.451737	0.764128	1279.417	325.6802	2.5	0.1	0.2	495
19AR109	Spot 389	2772.677	226.5753	1.030686	9.121015	16.79323	0.002389	128.4338	0.000393	6.80523	0.052986	2.534051	0.172414	2.423237	3.108555	NA	NA	2.5	0.2		340
19AR109	Spot 338	1382.297	421.7326	4.837195	4.289775	14.73182	0.011152	17.55208	0.000395	8.52668	0.485793	2.545022	0.216963	11.2613	1.965674	2865.829	251.1796	2.5	0.2	0.1	295
19AR109	Spot 587	3387.028	779.072	0.410789	9.856913	10.25317	0.004643	15.22565	0.000405	2.614857	0.17174	2.612425	0.068297	4.703094	0.714421	1271.733	294.2739	2.6	0.1	0.2	520
19AR109	Spot 122	4341.262	1623.748	2.34567	13.38928	10.36943	0.003799	11.44091	0.000419	2.238775	0.195682	2.699915	0.060432	3.850173	0.439661	800.0627	235.772	2.7	0.1	0.3	107
19AR109	Spot 295	2810.62	2567.812	2.652093	10.33366	28.72354	0.00904	29.3613	0.000718	2.007597	0.068376	4.627273	0.092864	9.137475	2.670852	1453.752	569.8252	4.6	0.1	0.3	260
19AR109	Spot 186	2547.332	2035.292	3.870076	11.49654	15.94376	0.008588	17.06362	0.00078	1.575955	0.092358	5.023933	0.079144	8.682944	1.475308	1195.145	337.5558	5.0	0.1	0.4	162
19AR109	Spot 375	938.5311	593.3875	4.612484	7.412837	11.6875	0.012148	21.42061	0.000785	8.408385	0.392537	5.059449	0.425251	12.26007	2.610397	1836.284	360.5094	5.1	0.4	0.3	327
19AR109	Spot 351	2046.001	1355.434	2.593642	11.53974	6.601434	0.008993	8.72217	0.000858	2.680523	0.307323	5.528179	0.148121	9.090496	0.78935	1096.598	166.4009	5.5	0.1	0.5	308
19AR109	Spot 387	1709.191	1563.704	2.640345	10.72972	18.12196	0.010905	19.67957	0.000939	2.732186	0.138834	6.050248	0.165226	11.0125	2.155505	1298.636	382.4094	6.1	0.2	0.5	338
19AR109	Spot 2	1422.766	1094.638	2.506028	12.51736	12.96599	0.008828	23.43981	0.000963	3.303883	0.140952	6.20579	0.204933	8.924781	2.082788	822.442	490.7332	6.2	0.2	0.8	2
19AR109	Spot 161	635.1417	537.9674	2.895892	9.345285	21.94146	0.010993	25.8119	0.000991	4.005025	0.155162	6.386993	0.255674	11.1014	2.849882	1208.478	510.2931	6.4	0.3	0.5	140
19AR109	Spot 581	491.056	474.9052	4.219572	7.387764	34.80584	0.014672	44.62699	0.001	7.831635	0.175491	6.443884	0.504409	14.7894	6.552322	1739.407	850.3625	6.4	0.5	0.4	514
19AR109	Spot 287	651.0438	656.5542	1.494992	7.890801	21.81954	0.014704	29.78147	0.001008	6.059176	0.203455	6.496614	0.393443	14.82105	4.381897	1728.4	547.4764	6.5	0.4	0.4	254
19AR109	Spot 400	672.1927	741.1921	3.414204	6.952087	16.6599	0.018217	18.16164	0.001054	4.728414	0.260352	6.793458	0.321054	18.33053	3.299267	2033.867	312.84	6.8	0.3	0.3	351
19AR109	Spot 374	441.0432	384.4227	1.605209	10.01795	20.94325	0.009304	64.22987	0.001085	8.273005	0.128803	6.991535	0.578096	9.403845	6.012264	681.4426	NA	7.0	0.6	1.0	326
19AR109	Spot 260	2450.006	1970.514	2.018147	14.77343	7.067757	0.009153	8.673434	0.001104	1.416681	0.163336	7.113883	0.100725	9.251672	0.798793	608.9253	185.2956	7.1	0.1	1.2	231
19AR109	Spot 399	1014.553	941.9725	1.717827	10.79581	13.78127	0.012269	15.14747	0.001152	2.836639	0.187268	7.425165	0.210504	12.38158	1.864109	1127.393	298.0545	7.4	0.2	0.7	350
19AR109	Spot 111	915.6015	1602.152	4.796905	10.45608	13.82689	0.014593	15.21509	0.001218	4.069845	0.267488	7.845196	0.319093	14.70999	2.222007	1359.37	284.0691	7.8	0.3	0.6	96
19AR109	Spot 280	1265.871	1512.463	5.790425	13.84118	10.44909	0.010846	12.25918	0.001255	2.561868	0.208976	8.082124	0.206924	10.95308	1.335542	699.0308	256.2009	8.1	0.2	1.2	248
19AR109	Spot 444	1968.995	2237.267	4.92887	14.81101	9.209208	0.011026	10.14434	0.001315	2.185243	0.215415	8.471415	0.184999	11.13421	1.123323	633.5449	213.7759	8.5	0.2	1.3	392
19AR109	Spot 271	1558.555	1509.373	2.870844	13.99607	6.507887	0.011239	13.19123	0.001321	1.739113	0.131839	8.508429	0.147874	11.34853	1.488677	665.3442	281.1477	8.5	0.1	1.3	240
19AR109	Spot 4	1384.673	1889.978	3.082446	12.40468	10.54638	0.014693	15.41823	0.001463	9.602427	0.622797	9.422367	0.904115	14.81067	2.266972	1010.767	245.4463	9.4	0.9	0.9	4
19AR109	Spot 597	840.4156	1151.602	1.399682	11.44617	14.66376	0.015121	17.13084	0.00147	3.439655	0.200787	9.467124	0.325397	15.239	2.591081	1059.244	340.0787	9.5	0.3	0.9	529
19AR109	Spot 580	748.3194	1022.561	0.9524	11.92285	13.0204	0.016128	20.72186	0.001678	3.967709	0.191475	10.80655	0.428413	16.24516	3.339526	920.9437	422.2334	10.8	0.4	1.2	513
19AR109	Spot 414	438.6884	743.5234	1.79547	9.606979	17.10021	0.020451	21.38559	0.00175	4.587839	0.214529	11.27367	0.516766	20.55627	4.351905	1310.426	409.7566	11.3	0.5	0.9	365
19AR109	Spot 579	1076.663	718.856	0.711918	10.89155	12.71165	0.017357	28.81417	0.001754	1.675629	0.058153	11.29621	0.189117	17.47302	4.991675	980.4843	597.9511	11.3	0.2	1.2	512
19AR109	Spot 102	584.8817	681.9841	1.022306	10.1418	14.54796	0.019203	18.8584	0.001791	3.569686	0.189289	11.5338	0.411352	19.31339	3.607792	1141.931	371.2522	11.5	0.4	1.0	88
19AR109	Spot 398	288.6288	506.7866	1.243018	9.684596	18.42848	0.018542	37.94708	0.001797	8.537325	0.22498	11.57397	0.987221	18.65477	7.01442	1064.948	769.9152	11.6	1.0	1.1	349
19AR109	Spot 500	417.2959	540.0416	1.852092	8.264848	26.81891	0.023611	28.04781	0.001804	5.816067	0.207363	11.62081	0.675265	23.69507	6.569094	1527.014	527.1023	11.6	0.7	0.8	441
19AR109	Spot 248	980.3418	572.8228	0.934861	7.740775	23.90024	0.026319	27.58018	0.001815	3.970534	0.143963	11.68777	0.463646	26.37802	7.181541	1718.348	511.5938	11.7	0.5	0.7	220
19AR109	Spot 222	699.1812	1515.827	1.036212	11.99143	15.61491	0.018924	16.32485	0.001866	2.676812	0.163972	12.01828	0.321407	19.03562	3.078598	1030.014	327.5929	12.0	0.3	1.2	197
19AR109	Spot 159	514.1654	707.4059	1.757655	6.376927	6.861428	0.036303	10.47495	0.001909	3.05912	0.292042	12.29753	0.375838	36.20787	3.725945	2201.578	174.4414	12.3	0.4	0.6	138
19AR109	Spot 175	560.8533	974.6344	1.052641	10.34691	23.4582	0.02202	24.67934	0.00195	3.021597	0.122434	12.5567	0.379043	22.11583	5.399081	1244.182	487.0547	12.6	0.4	1.0	154
19AR109	Spot 236	327.659	472.7329	0.952992	8.233967	19.06609	0.025	29.5993	0.001976	5.712262	0.192986	12.72302	0.726055	25.07224	7.330462	1463.587	563.9881	12.7	0.7	0.9	209
19AR109	Spot 264	543.3847	1063.442	2.71683	9.65507	21.42554	0.024958	23.45692	0.002003	3.791604	0.161641	12.90074	0.488656	25.03067	5.79971	1433.954	447.6524	12.9	0.5	0.9	235
19AR109	Spot 362	245.5418	673.7084	1.390329	6.683775	39.63752	0.037699	40.79334	0.002112	6.144663	0.150629	13.60174	0.8349	37.57541	15.04921	2091.248	742.7336	13.6	0.8	0.7	314
19AR109	Spot 364	1752.681	2132.686	2.521174	13.69243	7.546632	0.019407	12.6038	0.002129	5.427279	0.430607	13.7126	0.74343	19.5167	2.436361	810.389	238.6791	13.7	0.7	1.7	316
19AR109	Spot 481	190.2298	347.7155	1.684244	6.571835	14.98798	0.033387	22.7466	0.002149	6.576097	0.289102	13.83528	0.908846	33.34688	7.462209	1844.24	399.0007	13.8	0.9	0.8	423
19AR109	Spot 278	654.5073	2024.301	1.741021	13.2497	10.39467	0.020556	14.17873	0.002181	2.364242	0.166746	14.0454	0.331706	20.66057	2.899811	880.1117	290.5821	14.0	0.3	1.6	246
19AR109	Spot 418	266.7894	504.2447	2.263007	6.457916	19.53214	0.046961	28.39032	0.002661	5.675687	0.199916	17.12942	0.970922	46.59731	12.93086	2071.526	500.6018	17.1	1.0	0.8	369
19AR109	Spot 217	488.0454	2077.367	1.345536	15.30348	6.344086	0.04546	9.629361	0.005681	1.303099	0.135326	36.52139	0.474564	45.14088	4.251597	531.9486	209.3698	36.5	0.5	6.9	192
19AR109	Spot 339	1198.077	5536.744	3.255105	16.44145	3.976523	0.05016	6.724307	0.006254	4.444666	0.660985	40.18847	1.780687	49.69533	3.261221	537.2117	110.4841	40.2	1.8	7.5	296
19AR109	Spot 201	503.6395	3198.758	4.343777	15.63688	5.440203	0.085397	6.541843	0.010435	3.455886	0.528274	66.92192	2.300782	83.20643	5.226224	580.9917	120.7169	66.9	2.3	11.5	177
19AR109	Spot 277	150.8274	2324.519	1.896511	14.25717	5.311664	0.124973	10.42766	0.014178	2.318478	0.222339	90.75268	2.089339	119.5708	11.76282	740.2623	215.5929	90.8	2.1	12.3	245
19AR109	Spot 133	476.7747	4197.562	3.272039	17.76607	3.062896	0.277675	3.509662	0.038249	0.957069	0.272695	241.971	2.272905	248.8113	7.74497	314.7995	76.82243	242.0	2.3	76.9	117
19AR109	Spot 62	98.02137	3096.221	2.376352	15.81231	6.258936	0.327031	6.914754	0.040677	1.196558	0.173044	257.0265	3.014966	287.2963	17.30438	542.3516	149.0566	257.0	3.0	47.4	60
19AR109	Spot 239	636.6083	2250.929	1.9768	17.36863	1.652212	0.290194	3.748399	0.041319	1.24378	0.331816	261.0023	3.181454	258.712	8.560898	238.9937	81.57902	261.0	3.2	109.2	212
19AR109	Spot 346	373.4254	14808.85	1.842389	18.65436	2.497823	0.303691	2.850711	0.041929	0.868315	0.304596	264.7785	2.252534	269.2791	6.742902	309.5949	61.80891	264.8	2.3	85.5	303
19AR109	Spot 27	213.0814	8078.373	1.337253	18.06856	3.494283	0.31022	4.431429	0.042126	0.89426	0.201799	265.9953	2.330283	274.3512	10.65405	347.2348	98.19744	266.0	2.3	76.6	27
19AR109	Spot 335	424.5047	14458.49	1.698893	18.64863	1.943858	0.307518	2.110221	0.042423	0.729786	0.345834	267.8315	1.91455	272.2557	5.039469	311.436	45.04661	267.8	1.9	86.0	292
19AR109	Spot 473	868.0108	38695.74	1.084679	19.3478	1.270786	0.314843	1.551791	0.044708	0.878901	0.566378	281.9492	2.424643	277.9279	3.772987	245.2142	29.44655	281.9	2.4	115.0	416
19AR109	Spot 402	477.7276	20064.71	1.1386	19.18527	1.463118	0.315522	1.665061	0.044765	0.731318	0.439214	282.3021	2.019974	278.4518	4.055023	247.2224	34.46418	282.3	2.0	114.2	353
19AR109	Spot 121	213.007	9116.489	1.846634	18.18991	3.171418	0.332938	4.437549	0.045259	0.806386	0.181719	285.3467	2.250812	291.8063	11.25496	344.8224	98.75421	285.3	2.3	82.8	106
19AR109	Spot 108	168.017	4364.413	1.757744	18.32759	3.474371	0.327053	4.313692	0.046306	0.90678	0.21021	291.8006	2.586997	287.3136	10.79507	251.9956	97.0665	291.8	2.6	115.8	94
19AR109	Spot 511	236.16	9947.635	2.209558	17.75946	2.542892	0.353583	2.96359	0.04695	0.884177	0.298347	295.7706	2.556048	307.4124	7.860733	397.6572	63.39915	295.8	2.6	74.4	451
19AR109	Spot 416	451.7805	1378.106	2.542688	15.5287	1.477435	0.348659	3.178538	0.047244	1.506451	0.473945	297.5816	4.381024	303.7118	8.343831	352.0731	63.25023	297.6	4.4	84.5	367
19AR109	Spot 395	212.9614	8052.224	1.701285	17.55339	3.266153	0.38411	4.232271	0.050699	0.898918	0.212396	318.813	2.796155	330.0574	11.92636	411.0632	92.50908	318.8	2.8	77.6	346
19AR109	Spot 538	390.9608	15236.16	7.899989	17.35199	1.296414	0.408011	2.954892	0.052422	2.096847	0.709619	329.3713	6.732944	347.4417	8.694562	471.0784	46.08021	329.4	6.7	69.9	477
19AR109	Spot 18	234.7068	15807.24	1.361884	17.33426	2.375108	0.411612	2.980154	0.052672	0.921176	0.309103	330.9031	2.971287	350.0352	8.823729	479.9426	62.66003	330.9	3.0	68.9	18
19AR109	Spot 73	103.1276	5141.548	0.859707	16.34994	4.286579	0.434834	4.807848	0.054281	1.006922	0.209433	340.7486	3.34197	366.6035	14.79563	534.5186	102.9893	340.7	3.3	63.7	71
19AR109	Spot 306	982.4249	48063.27	3.757023	13.65449	0.751511	0.566646	1.439236	0.056209	1.219232	0.847138	352.5314	4.182764	455.8433	5.285756	1017.984	15.48888	352.5	4.2	34.6	271
19AR109	Spot 193	386.4033	4035.191	9.921285	16.86948	1.641923	0.515334	2.217824	0.067191	1.008471	0.454712	419.2092	4.093076	422.0299	7.658538	438.4177	43.98784	419.2	4.1	95.6	169
19AR109	Spot 384	277.4518	3317.917	6.352197	16.79654	1.700449	0.525419	2.035616	0.069074	0.864298	0.424588	430.5722	3.599867	428.7652	7.119504	420.0153	41.15338	430.6	3.6	102.5	335
19AR109	Spot 370	20.89463	229.4177	0.458891	10.1339	14.99285	0.321094	119.1674	0.069639	2.305909	0.01935	433.9813	9.677807	282.7434	302.7577	NA	NA	434.0	9.7		322
19AR109	Spot 303	19.24012	1023.975	0.646875	11.87172	8.05164	0.696505	10.43847	0.071719	3.574631	0.342448	446.5015	15.4206	536.7013	43.54134	941.9841	201.4152	446.5	15.4	47.4	268
19AR109	Spot 140	312.3587	9188.098	13.2659	17.4933	1.905134	0.554987	2.408075	0.072647	0.857783	0.356211	452.0823	3.745031	448.2584	8.727035	429.6338	50.16178	452.1	3.7	105.2	124
19AR109	Spot 336	704.1815	3190.18	1.281864	16.45685	1.071867	0.564374	1.959461	0.07288	1.013437	0.517202	453.486	4.437869	454.3694	7.177946	459.8021	37.20221	453.5	4.4	98.6	293
19AR109	Spot 373	479.1615	7327.424	5.860132	17.26789	1.226985	0.562788	1.5714	0.072968	0.808337	0.514406	454.0105	3.543685	453.3396	5.746008	450.8844	29.92776	454.0	3.5	100.7	325
19AR109	Spot 31	226.2508	13591.31	11.47423	17.74334	2.562023	0.562435	2.768507	0.073897	0.856517	0.309379	459.5934	3.799446	453.1102	10.11953	421.2987	58.77744	459.6	3.8	109.1	30
19AR109	Spot 12	499.4979	14732.82	2.739937	17.67613	1.43518	0.565786	2.20309	0.073922	0.847807	0.384827	459.7397	3.761967	455.2857	8.083347	433.8112	45.29707	459.7	3.8	106.0	12
19AR109	Spot 522	405.0431	22445.27	1.479794	17.66857	1.262571	0.570901	1.628497	0.074297	0.642383	0.394464	461.9927	2.863915	458.5971	6.009439	442.5653	33.30095	462.0	2.9	104.4	461
19AR109	Spot 42	215.6413	13884.03	1.881734	18.53056	1.799286	0.541707	2.055727	0.074388	0.789329	0.383966	462.54	3.523058	439.5495	7.334417	321.8742	43.10209	462.5	3.5	143.7	41
19AR109	Spot 401	168.4235	9578.497	1.447375	17.06397	2.836719	0.583603	3.517885	0.074491	0.893923	0.254108	463.1542	3.995008	466.7743	13.16458	485.6	75.14415	463.2	4.0	95.4	352
19AR109	Spot 49	408.797	7672.782	6.656252	17.42401	1.260912	0.571474	2.525333	0.074766	0.606207	0.24005	464.8069	2.718508	458.9672	9.325021	430.7858	54.65345	464.8	2.7	107.9	48
19AR109	Spot 67	125.0652	6506.297	1.002099	17.77025	3.624346	0.554607	4.725295	0.074768	0.847702	0.179397	464.8154	3.801547	448.0104	17.11847	363.5868	104.8546	464.8	3.8	127.8	65
19AR109	Spot 47	526.394	32844.48	27.36848	17.70656	0.962857	0.577382	1.228963	0.074871	0.666835	0.5426	465.4383	2.994309	462.7774	4.567703	450.5383	22.92886	465.4	3.0	103.3	46
19AR109	Spot 422	332.1578	29359.85	30.40666	17.38719	1.763101	0.586758	1.978951	0.07493	0.796105	0.402286	465.7894	3.577374	468.7952	7.430552	484.5215	40.00602	465.8	3.6	96.1	372
19AR109	Spot 132	184.7906	13144.59	1.938415	17.41111	2.751499	0.581758	3.221956	0.075096	0.607473	0.188542	466.787	2.735374	465.5907	12.03295	460.6399	70.18935	466.8	2.7	101.3	116
19AR109	Spot 403	311.2567	18047.37	15.91758	17.48248	1.489872	0.580941	1.78765	0.075098	0.764621	0.427724	466.7978	3.443071	465.0659	6.67014	457.511	35.84715	466.8	3.4	102.0	354
19AR109	Spot 458	318.9393	23127.96	8.547084	17.54089	1.680653	0.582844	2.012823	0.075332	0.747324	0.371281	468.1994	3.374923	466.2874	7.525888	457.844	41.48058	468.2	3.4	102.3	403
19AR109	Spot 39	113.317	7630.874	1.423457	17.51349	3.215177	0.572468	4.110677	0.075369	0.840718	0.204521	468.4231	3.798442	459.6093	15.19655	416.7393	89.94683	468.4	3.8	112.4	38
19AR109	Spot 233	174.6084	11430.87	2.157171	17.62957	1.897664	0.574823	2.439029	0.075386	0.829259	0.339996	468.5223	3.747435	461.129	9.039839	425.4144	51.19276	468.5	3.7	110.1	207
19AR109	Spot 330	339.4342	23651.78	8.017093	17.49815	1.165824	0.586157	1.458211	0.075419	0.663083	0.454724	468.7222	2.997714	468.4104	5.471699	467.8283	28.77141	468.7	3.0	100.2	287
19AR109	Spot 118	144.0813	8672.809	1.872609	16.70544	2.736762	0.604581	3.010165	0.07542	0.91671	0.304538	468.7281	4.144382	480.1367	11.51679	535.9265	62.76343	468.7	4.1	87.5	103
19AR109	Spot 451	128.678	11024.89	1.998189	16.48479	3.687507	0.614452	4.029872	0.075448	0.739607	0.183531	468.8924	3.344841	486.3641	15.57464	570.4995	86.23669	468.9	3.3	82.2	397
19AR109	Spot 224	336.5329	23785.36	1.264108	17.16561	1.536731	0.596872	1.7618	0.075454	0.808432	0.458867	468.9294	3.656376	475.2468	6.68658	506.8179	34.42967	468.9	3.7	92.5	199
19AR109	Spot 128	875.0938	47022.04	2.708645	17.57726	0.797341	0.587566	1.086512	0.075487	0.599699	0.551949	469.128	2.713427	469.3124	4.083117	471.1976	20.05116	469.1	2.7	99.6	112
19AR109	Spot 316	309.848	24882.96	7.522388	17.46317	1.892854	0.590856	2.268515	0.075518	0.871949	0.38437	469.3173	3.946796	471.4144	8.555252	482.6193	46.28096	469.3	3.9	97.2	277
19AR109	Spot 383	252.3413	16099.09	1.082034	17.53514	1.791239	0.584736	2.168523	0.075529	0.685498	0.316113	469.3785	3.103231	467.5003	8.124675	459.2517	45.6282	469.4	3.1	102.2	334
19AR109	Spot 117	151.7992	14605.17	1.948939	17.102	3.972168	0.598747	4.110906	0.075577	0.934785	0.227392	469.6703	4.234286	476.4381	15.63382	510.1094	88.04389	469.7	4.2	92.1	102
19AR109	Spot 190	67.3838	4020.97	1.118285	16.08253	3.694844	0.610148	5.432885	0.075611	0.948663	0.174615	469.8745	4.298951	483.6533	20.90697	550.4599	116.8776	469.9	4.3	85.4	166
19AR109	Spot 213	208.8104	13871.62	2.761493	17.23607	2.552117	0.591139	2.703231	0.075644	0.674337	0.249456	470.0726	3.057059	471.5945	10.19786	479.9478	57.87413	470.1	3.1	97.9	188
19AR109	Spot 294	485.9723	34975.65	2.166344	17.37372	0.977647	0.595935	1.219809	0.075693	0.580758	0.476106	470.3607	2.634381	474.6505	4.624964	496.4103	23.62566	470.4	2.6	94.8	259
19AR109	Spot 8	307.2604	18204.33	9.083931	17.26469	1.447352	0.595069	1.63818	0.075723	0.689379	0.42082	470.5403	3.128248	474.0994	6.205612	492.3195	32.76702	470.5	3.1	95.6	8
19AR109	Spot 359	40.53712	2500.428	1.788151	13.56323	8.814462	0.707773	11.69998	0.075767	1.610739	0.13767	470.8078	7.31318	543.423	49.27421	861.8099	241.1779	470.8	7.3	54.6	312
19AR109	Spot 560	233.1209	10678.58	6.472539	17.74479	2.002853	0.572126	2.457787	0.07578	0.7161	0.29136	470.8863	3.251804	459.3888	9.082182	403.2405	52.65543	470.9	3.3	116.8	494
19AR109	Spot 371	239.7487	16353.79	4.942238	17.88032	1.657516	0.574869	2.124487	0.075792	0.68858	0.324116	470.9576	3.127293	461.1588	7.874396	413.5897	44.94397	471.0	3.1	113.9	323
19AR109	Spot 185	168.7518	7526.327	6.772419	17.1038	3.466725	0.589855	3.828596	0.075795	0.747716	0.195298	470.9765	3.396002	470.7751	14.42402	470.7784	83.12863	471.0	3.4	100.0	161
19AR109	Spot 229	163.5397	10109.53	2.5633	17.12009	3.25598	0.593492	3.485834	0.075802	0.783813	0.224857	471.0135	3.560216	473.0952	13.18336	484.186	75.02481	471.0	3.6	97.3	203
19AR109	Spot 427	256.64	17472.15	15.25061	17.39271	1.941807	0.590528	2.080255	0.075852	0.705125	0.338961	471.3179	3.204796	471.2049	7.842496	471.62	43.31265	471.3	3.2	99.9	377
19AR109	Spot 406	157.0549	3414.359	1.034285	16.57431	3.021158	0.583561	6.332178	0.075871	0.810151	0.127942	471.4304	3.682989	466.7475	23.6981	444.725	139.7392	471.4	3.7	106.0	357
19AR109	Spot 210	107.8769	6290.664	1.457854	16.45056	4.191883	0.608749	4.505469	0.075885	1.108275	0.245984	471.5119	5.039115	482.7711	17.31259	537.5261	95.61381	471.5	5.0	87.7	185
19AR109	Spot 91	208.444	15369.07	2.524632	17.36447	2.197358	0.590309	2.415068	0.07589	0.823983	0.341184	471.5419	3.746723	471.0651	9.102674	469.6883	50.25869	471.5	3.7	100.4	80
19AR109	Spot 391	415.2253	29129.13	12.4312	17.63089	1.46508	0.587565	1.734637	0.075892	0.818056	0.471601	471.5548	3.719872	469.3116	6.518818	459.3398	33.93026	471.6	3.7	102.7	342
19AR109	Spot 372	364.6593	15445.58	3.758807	17.59916	1.071442	0.584474	1.351674	0.075897	0.694954	0.514144	471.5874	3.160312	467.3327	5.062739	447.4315	25.76325	471.6	3.2	105.4	324
19AR109	Spot 546	155.5492	6292.359	1.933382	17.83482	2.454986	0.560218	3.211578	0.075975	0.881416	0.27445	472.0511	4.012048	451.6686	11.70953	350.1568	69.79795	472.1	4.0	134.8	484
19AR109	Spot 476	239.5935	12143.45	8.209124	17.58585	1.825317	0.581183	2.299392	0.076006	0.916926	0.398769	472.2393	4.175284	465.2214	8.581909	431.677	46.99062	472.2	4.2	109.4	419
19AR109	Spot 150	289.0313	25132.31	1.258109	17.46449	1.742887	0.591442	2.023484	0.076027	0.736196	0.363826	472.3621	3.353157	471.7882	7.635885	469.9401	41.73175	472.4	3.4	100.5	132
19AR109	Spot 392	240.5423	21782.56	4.733738	17.53061	2.272461	0.590502	2.611089	0.07606	0.975313	0.373527	472.562	4.444082	471.188	9.843561	465.4441	53.6621	472.6	4.4	101.5	343
19AR109	Spot 188	368.797	5365.182	1.373342	16.80767	1.669863	0.595108	4.560021	0.076078	0.76893	0.168624	472.6714	3.504463	474.1243	17.27604	482.1444	99.34415	472.7	3.5	98.0	164
19AR109	Spot 536	396.362	37521.2	5.474961	17.65854	1.256145	0.587484	1.594405	0.076093	0.765705	0.480245	472.7571	3.490374	469.2596	5.991288	453.1336	31.06964	472.8	3.5	104.3	475
19AR109	Spot 258	94.5435	5501.502	1.385608	16.32949	3.930316	0.613754	4.956326	0.076198	1.181082	0.238298	473.3881	5.39075	485.9247	19.14248	546.4217	105.2373	473.4	5.4	86.6	229
19AR109	Spot 231	190.4354	15995.65	0.874907	17.58121	2.507524	0.586147	2.817892	0.076206	0.733877	0.260435	473.4377	3.349932	468.404	10.57384	444.7572	60.51584	473.4	3.3	106.4	205
19AR109	Spot 113	221.1962	14185.17	9.166828	17.46528	2.01633	0.590663	2.748697	0.076211	0.737284	0.26823	473.4655	3.365676	471.2911	10.36415	461.6597	58.70701	473.5	3.4	102.6	98
19AR109	Spot 478	167.6985	10747.25	3.708396	17.44053	2.856241	0.586693	3.078412	0.076279	0.978984	0.318016	473.874	4.472743	468.7536	11.55829	444.7037	64.90343	473.9	4.5	106.6	421
19AR109	Spot 477	274.8871	13254.54	4.625824	17.45772	1.473045	0.58938	2.307771	0.076367	0.876252	0.379696	474.3987	4.007657	470.4715	8.689622	452.304	47.42714	474.4	4.0	104.9	420
19AR109	Spot 349	297.3567	21177.8	1.33444	17.69299	1.648166	0.587918	1.892351	0.076388	0.842348	0.445133	474.5281	3.853604	469.5373	7.114222	446.1599	37.68762	474.5	3.9	106.4	306
19AR109	Spot 16	183.1381	10814.3	2.017886	16.89864	3.114899	0.608605	3.420747	0.076405	0.915768	0.26771	474.6269	4.190331	482.6797	13.14199	522.0432	72.32292	474.6	4.2	90.9	16
19AR109	Spot 272	377.5569	46466.97	6.992526	17.35017	1.510859	0.602978	1.67698	0.076434	0.693236	0.413383	474.8039	3.173216	479.122	6.405272	500.8217	33.6154	474.8	3.2	94.8	241
19AR109	Spot 174	754.6913	53485.71	10.43879	17.42526	0.788768	0.600341	0.981202	0.076458	0.567043	0.577907	474.9487	2.596346	477.4499	3.737454	490.4634	17.67073	474.9	2.6	96.8	153
19AR109	Spot 206	77.45539	4871.171	1.315699	15.71449	6.18949	0.637902	6.377331	0.076462	0.894867	0.14032	474.97	4.097545	501.0066	25.22461	622.6818	136.3024	475.0	4.1	76.3	182
19AR109	Spot 563	211.3856	8118.89	2.002185	16.92951	3.096236	0.60134	3.389793	0.076476	0.819693	0.241812	475.0568	3.753987	478.0838	12.92597	493.6115	72.55011	475.1	3.8	96.2	497
19AR109	Spot 497	191.9154	12254.8	3.694115	18.03145	2.131287	0.569811	2.353076	0.076492	0.76704	0.325973	475.149	3.513508	457.8923	8.67281	373.1511	50.07379	475.1	3.5	127.3	438
19AR109	Spot 584	126.3779	8136.021	1.476108	16.92937	3.313661	0.602293	4.623299	0.076509	0.875145	0.18929	475.2541	4.009547	478.688	17.64784	496.1432	100.0925	475.3	4.0	95.8	517
19AR109	Spot 112	432.4334	21963.02	5.288643	17.36437	1.096947	0.601277	1.425736	0.076572	0.745737	0.523054	475.6266	3.419233	478.044	5.436024	490.6028	26.79032	475.6	3.4	96.9	97
19AR109	Spot 505	281.6231	17771.18	2.956301	17.76855	2.126369	0.583342	2.461729	0.076595	0.74423	0.30232	475.764	3.413276	466.6072	9.209388	422.748	52.38778	475.8	3.4	112.5	446
19AR109	Spot 156	427.6353	30744.95	5.626566	17.34406	1.306411	0.601443	1.487621	0.076601	0.687048	0.461843	475.8049	3.151281	478.1491	5.672962	490.3477	29.10461	475.8	3.2	97.0	135
19AR109	Spot 388	166.8943	14056.08	5.538823	16.94747	3.186654	0.61211	3.562797	0.076613	0.905408	0.254128	475.8748	4.153421	484.89	13.7367	528.7051	75.50829	475.9	4.2	90.0	339
19AR109	Spot 419	392.5904	30987.36	6.920791	17.2659	1.098592	0.604475	1.529131	0.076614	0.782861	0.511965	475.882	3.591309	480.0695	5.849578	501.0994	28.9204	475.9	3.6	95.0	370
19AR109	Spot 98	348.2845	22722.91	11.53669	17.11957	1.267644	0.607724	1.589232	0.076624	0.87237	0.548925	475.9403	4.002395	482.1238	6.099827	512.5914	29.18573	475.9	4.0	92.8	85
19AR109	Spot 97	241.8732	18000.58	5.806291	17.01775	1.831862	0.610164	2.105282	0.07669	0.763887	0.362843	476.3356	3.507485	483.6634	8.100758	519.4769	43.04749	476.3	3.5	91.7	84
19AR109	Spot 126	160.0247	9861.193	4.454416	17.38823	2.254706	0.591393	3.487734	0.076693	0.734436	0.210577	476.3519	3.372368	471.7566	13.16122	450.4102	75.76603	476.4	3.4	105.8	110
19AR109	Spot 433	281.7311	21629.73	4.933575	17.71226	1.457935	0.587989	1.680491	0.076699	0.602276	0.358393	476.387	2.765713	469.5828	6.318202	437.4077	34.94651	476.4	2.8	108.9	383

19AR109	Spot 343	543.9707	31332.25	40.47972	17.50958	1.014857	0.599503	1.43281	0.076779	0.877332	0.612316	476.8677	4.032718	476.9182	5.452916	478.1008	25.0479	476.9	4.0	99.7	300
19AR109	Spot 352	291.1376	17186.54	7.609409	17.40856	1.790239	0.597045	1.983198	0.076782	0.739538	0.372902	476.8891	3.399485	475.3566	7.528246	468.9343	40.75178	476.9	3.4	101.7	309
19AR109	Spot 52	310.6189	21550.41	14.12185	17.35911	2.049838	0.601164	2.412097	0.076784	0.838681	0.347698	476.8987	3.855296	477.9722	9.19589	484.1086	49.94476	476.9	3.9	98.5	51
19AR109	Spot 323	142.0879	10611.98	2.364284	16.5122	3.906242	0.624809	4.142863	0.076785	0.773027	0.186592	476.906	3.553546	492.8571	16.17753	568.6453	88.64095	476.9	3.6	83.9	281
19AR109	Spot 249	594.2356	38922.92	14.84384	16.13039	0.850546	0.652441	1.454036	0.076808	1.142207	0.785542	477.04	5.252059	509.9797	5.829415	661.4552	19.26279	477.0	5.3	72.1	221
19AR109	Spot 41	260.6161	19810.93	3.58305	17.0095	1.690658	0.613759	2.16623	0.076813	0.742822	0.34291	477.0721	3.415844	485.928	8.365724	528.8865	44.57941	477.1	3.4	90.2	40
19AR109	Spot 475	196.3639	8998.605	1.297267	17.76385	1.831437	0.577424	2.502598	0.076883	0.993455	0.39697	477.4908	4.572234	462.8044	9.302063	391.5154	51.52732	477.5	4.6	122.0	418
19AR109	Spot 361	246.985	15572.81	1.666048	17.36849	1.468245	0.598361	1.806824	0.076892	0.755375	0.418067	477.5435	3.476871	476.1927	6.868164	470.6754	36.32886	477.5	3.5	101.5	313
19AR109	Spot 191	286.2493	12375.72	3.81422	17.7318	1.657367	0.585038	2.686649	0.076901	0.744205	0.277001	477.5965	3.425824	467.6937	10.0693	420.3235	57.64554	477.6	3.4	113.6	167
19AR109	Spot 550	361.5818	39351.33	3.862415	17.51541	1.426946	0.599663	1.716261	0.077	0.776922	0.452683	478.1932	3.58074	477.0196	6.532776	472.3391	33.86329	478.2	3.6	101.2	487
19AR109	Spot 467	102.2919	8119.313	1.326323	16.43085	3.944488	0.624877	4.266128	0.077018	0.81166	0.190257	478.2972	3.741623	492.8997	16.66007	562.2593	91.27944	478.3	3.7	85.1	410
19AR109	Spot 432	1069.321	67057.3	2.43985	17.48099	0.72556	0.60336	0.951891	0.077063	0.604042	0.63457	478.5676	2.786053	479.3636	3.637178	484.1561	16.24541	478.6	2.8	98.8	382
19AR109	Spot 379	112.5671	6982.709	3.4313	17.28809	4.114082	0.593666	4.233318	0.0771	0.867723	0.204975	478.7919	4.004053	473.206	16.0137	447.1574	92.10056	478.8	4.0	107.1	331
19AR109	Spot 38	130.2023	11397.82	1.58996	17.54454	3.449746	0.591494	3.823825	0.077107	0.785908	0.205529	478.8328	3.626819	471.8213	14.4312	438.8094	83.30774	478.8	3.6	109.1	37
19AR109	Spot 494	247.4633	16500.53	8.466802	17.99672	2.090766	0.578858	2.51417	0.077129	1.071287	0.4261	478.9629	4.945086	463.7272	9.359781	389.9162	51.05847	479.0	4.9	122.8	435
19AR109	Spot 14	321.0839	22377.4	8.967486	17.45668	1.604953	0.601657	1.943114	0.077131	1.012003	0.520815	478.9757	4.671549	478.2848	7.411659	475.9386	36.69319	479.0	4.7	100.6	14
19AR109	Spot 439	315.4034	25216.84	3.062331	17.49917	1.935597	0.600087	2.270047	0.077181	0.657429	0.28961	479.2743	3.036609	477.2888	8.644619	468.7375	48.10992	479.3	3.0	102.2	388
19AR109	Spot 54	262.766	17962.6	7.528657	17.30006	1.389868	0.604694	2.058159	0.077195	0.840787	0.408514	479.356	3.884159	480.208	7.875184	485.2399	41.4974	479.4	3.9	98.8	53
19AR109	Spot 574	252.428	19052	2.129784	17.65853	1.394747	0.594006	1.661726	0.077242	0.768706	0.462595	479.6366	3.553173	473.4225	6.287756	444.3647	32.75685	479.6	3.6	107.9	507
19AR109	Spot 313	336.8029	18366.8	5.977529	17.48885	1.169887	0.601757	1.672665	0.07731	1.126758	0.673631	480.0438	5.212449	478.3481	6.380712	471.2037	27.35816	480.0	5.2	101.9	275
19AR109	Spot 407	135.0434	11419.99	3.229656	16.76345	4.226435	0.619628	4.355781	0.077363	0.909488	0.2088	480.3663	4.210065	489.6141	16.92201	534.0672	93.29912	480.4	4.2	89.9	358
19AR109	Spot 573	179.6806	7393.016	3.515974	17.28055	2.800718	0.596828	3.649763	0.077609	0.749404	0.20533	481.8375	3.479264	475.2184	13.85199	444.3383	79.44077	481.8	3.5	108.4	506
19AR109	Spot 309	284.1234	16138.33	2.488122	17.54391	2.253024	0.602175	2.635712	0.077667	0.858952	0.32589	482.1793	3.990587	478.613	10.059	462.5475	55.2359	482.2	4.0	104.2	273
19AR109	Spot 435	249.1818	26591.2	2.40448	17.71058	1.982901	0.597669	2.191016	0.077736	0.733579	0.334812	482.595	3.410946	475.7531	8.322597	443.8447	45.92064	482.6	3.4	108.7	384
19AR109	Spot 182	719.2379	40176.8	5.520568	17.50976	0.852009	0.60769	1.195721	0.077875	0.793529	0.663641	483.4259	3.695818	482.1024	4.58926	476.7787	19.7977	483.4	3.7	101.4	158
19AR109	Spot 585	359.545	38755.61	3.676959	17.36627	1.307391	0.612575	1.575012	0.077917	0.806738	0.512211	483.6784	3.759228	485.1826	6.075167	493.2332	29.80234	483.7	3.8	98.1	518
19AR109	Spot 251	632.4539	43470.11	267.5369	17.535	0.853886	0.614502	1.165228	0.078737	0.787209	0.675584	488.5769	3.703986	486.3957	4.503274	477.0727	18.98738	488.6	3.7	102.4	223
19AR109	Spot 413	192.8009	15752.56	2.275893	17.50303	2.07219	0.607427	2.304862	0.07877	0.906049	0.393103	488.7772	4.264836	481.9359	8.843994	450.4675	47.07661	488.8	4.3	108.5	364
19AR109	Spot 195	438.0261	5585.974	6.500799	14.82417	1.071587	0.708272	1.860495	0.079245	1.373798	0.738405	491.616	6.502705	543.7198	7.832674	769.4771	26.41865	491.6	6.5	63.9	171
19AR109	Spot 578	352.666	25865.29	13.84625	17.53123	1.033081	0.617592	1.444509	0.079531	0.976757	0.676187	493.3226	4.638807	488.3371	5.599991	465.9889	23.59467	493.3	4.6	105.9	511
19AR109	Spot 214	378.1442	24725.66	3.38718	17.16602	1.172445	0.63467	1.499159	0.080233	0.81989	0.5469	497.5114	3.925615	499.0008	5.910176	506.7969	27.6284	497.5	3.9	98.2	189
19AR109	Spot 173	260.0932	14165.26	3.9514	17.30966	1.683105	0.627554	2.141925	0.080487	0.853311	0.398385	499.0261	4.097599	494.5712	8.386098	474.9412	43.45371	499.0	4.1	105.1	152
19AR109	Spot 539	488.4634	39078	1.846143	17.30639	0.836821	0.635026	1.156305	0.0805	0.729322	0.630735	499.1072	3.502753	499.2216	4.560073	500.7251	19.7569	499.1	3.5	99.7	478
19AR109	Spot 589	359.4889	21314.45	2.224334	17.17244	1.54685	0.637008	1.83787	0.080578	0.845296	0.459932	499.5703	4.063366	500.4517	7.261828	505.441	35.90573	499.6	4.1	98.8	522
19AR109	Spot 368	156.4468	1114.542	1.565378	15.24637	2.839916	0.582307	3.751556	0.080604	0.706581	0.188344	499.7258	3.397579	465.943	14.01943	303.7221	83.9753	499.7	3.4	164.5	320
19AR109	Spot 283	496.0326	31866.35	49.00303	17.25161	0.919041	0.642654	1.487895	0.081065	0.878854	0.59067	502.4761	4.248311	503.9482	5.91069	511.6133	26.37104	502.5	4.2	98.2	251
19AR109	Spot 446	380.8433	16845.93	6.232588	17.34611	1.107995	0.632795	1.531204	0.081115	0.67527	0.441006	502.7724	3.266051	497.8353	6.025587	476.1495	30.4032	502.8	3.3	105.6	394
19AR109	Spot 127	53.22381	3001.546	0.425772	14.04081	7.810612	0.744289	8.019894	0.08141	1.133274	0.141308	504.5361	5.499747	564.9051	34.76091	817.0135	166.1544	504.5	5.5	61.8	111
19AR109	Spot 254	289.1974	14488.08	3.587321	16.74342	1.661219	0.657735	2.217273	0.081501	0.774573	0.349336	505.0737	3.762834	513.2274	8.932972	550.678	45.36106	505.1	3.8	91.7	226
19AR109	Spot 119	291.9245	16438.16	3.129101	16.9365	1.637144	0.654594	2.01335	0.081656	0.798243	0.396475	506.0005	3.884662	511.302	8.087959	536.0091	40.45999	506.0	3.9	94.4	104
19AR109	Spot 253	1209.232	91644.15	7.802667	17.26114	0.75279	0.648635	1.069679	0.081693	0.743884	0.695427	506.2188	3.621621	507.6387	4.273285	514.9761	16.90516	506.2	3.6	98.3	225
19AR109	Spot 298	417.0263	33244.8	4.913804	17.02045	1.093677	0.658328	1.332456	0.081904	0.732597	0.549809	507.4769	3.57519	513.5905	5.371044	541.8414	24.35606	507.5	3.6	93.7	263
19AR109	Spot 437	145.2131	9795.361	3.539297	16.59744	2.753856	0.662568	2.886357	0.081993	0.697769	0.241747	508.007	3.408642	516.1833	11.68019	553.4825	61.11276	508.0	3.4	91.8	386
19AR109	Spot 146	356.0993	25422.57	4.702508	17.41016	1.513034	0.640689	1.793962	0.082002	0.904865	0.504395	508.0626	4.420787	502.7325	7.113302	479.4873	34.24474	508.1	4.4	106.0	130
19AR109	Spot 255	384.7233	28395.41	1.975099	17.38495	1.473731	0.643541	1.725591	0.08214	0.76528	0.443488	508.8846	3.744644	504.4959	6.860724	485.6157	34.14444	508.9	3.7	104.8	227
19AR109	Spot 33	297.3074	24237.59	3.721543	17.02231	1.778368	0.657765	1.979764	0.082167	0.740659	0.374115	509.0436	3.62526	513.2458	7.976273	532.9785	40.22545	509.0	3.6	95.5	32
19AR109	Spot 166	570.2795	39563.63	203.2617	17.12821	0.759589	0.655685	1.184569	0.082232	0.784315	0.66211	509.4323	3.841758	511.971	4.763332	524.2556	19.46978	509.4	3.8	97.2	145
19AR109	Spot 265	612.6426	62314.99	1.056962	17.02426	0.981976	0.66309	1.254655	0.082266	0.724308	0.577296	509.6342	3.54918	516.5025	5.079427	547.9763	22.38788	509.6	3.5	93.0	236
19AR109	Spot 88	168.4358	11845.2	1.576014	17.08625	2.65649	0.647252	2.925444	0.082286	0.868733	0.296958	509.7503	4.257806	506.7863	11.67224	494.4084	61.59862	509.8	4.3	103.1	77
19AR109	Spot 101	183.6257	17950.21	2.075849	17.0856	2.760211	0.654093	2.965706	0.082378	0.794649	0.267946	510.3033	3.898769	510.9943	11.90851	515.0231	62.79061	510.3	3.9	99.1	87
19AR109	Spot 75	339.4223	28232.82	1.782228	16.88303	1.151422	0.664403	1.530407	0.082409	0.735212	0.480403	510.4845	3.608384	517.3034	6.203193	548.5076	29.31833	510.5	3.6	93.1	73
19AR109	Spot 385	448.3122	29132.29	5.701199	17.25439	1.352149	0.65378	1.577073	0.082486	0.757199	0.480129	510.9445	3.719514	510.802	6.330546	511.1001	30.42844	510.9	3.7	100.0	336
19AR109	Spot 184	186.9258	11682.89	2.393104	16.95925	2.293582	0.655498	2.490582	0.082497	0.70359	0.2825	511.0073	3.456587	511.8563	10.01354	516.5851	52.4898	511.0	3.5	98.9	160
19AR109	Spot 179	214.8955	11258.6	4.770568	17.3908	1.927865	0.637815	2.512964	0.08252	0.65809	0.261878	511.1489	3.233916	500.9527	9.937109	455.5914	53.84513	511.1	3.2	112.2	157
19AR109	Spot 299	628.9105	26993.46	5.9815	17.13294	0.822075	0.657552	1.231523	0.082527	0.709891	0.576433	511.1908	3.488742	513.1158	4.960654	522.6331	22.05862	511.2	3.5	97.8	264
19AR109	Spot 464	241.3704	17263.78	1.843071	17.33492	1.721646	0.644591	1.92355	0.082565	0.765865	0.398152	511.412	3.765393	505.1448	7.6554	477.8052	39.00791	511.4	3.8	107.0	408
19AR109	Spot 290	262.95	30135.55	6.300433	17.33854	1.87527	0.650521	2.011831	0.082604	0.557798	0.277259	511.6497	2.74365	508.7995	8.05139	496.9768	42.60121	511.6	2.7	103.0	256
19AR109	Spot 588	160.3994	9831.365	2.994705	16.5192	3.148371	0.67071	3.408793	0.082606	0.806086	0.236473	511.6561	3.964957	521.1438	13.89605	563.8726	72.16702	511.7	4.0	90.7	521
19AR109	Spot 320	204.2198	11856.59	2.048945	16.40358	1.949677	0.678764	2.532134	0.082612	0.721895	0.285094	511.6929	3.551086	526.0273	10.39588	589.6979	52.65289	511.7	3.6	86.8	279
19AR109	Spot 23	193.353	22351.68	7.41081	17.47079	3.096313	0.642929	3.319705	0.082613	0.81651	0.245958	511.701	4.016567	504.1182	13.19162	470.8366	71.24279	511.7	4.0	108.7	23
19AR109	Spot 139	165.9266	12168.52	2.581315	17.08269	2.035325	0.651659	2.356107	0.082675	0.906575	0.384777	512.0702	4.462706	509.4991	9.439248	498.9147	47.90371	512.1	4.5	102.6	123
19AR109	Spot 43	229.6635	16365.81	2.969561	17.24311	1.712593	0.650221	2.05063	0.082733	0.733151	0.357525	512.4126	3.611326	508.615	8.204378	492.5373	42.24897	512.4	3.6	104.0	42
19AR109	Spot 104	148.7165	11911.82	1.599423	16.87743	1.956537	0.661509	2.29441	0.082736	0.704799	0.307181	512.4338	3.471813	515.5365	9.275684	530.2879	47.83223	512.4	3.5	96.6	90
19AR109	Spot 526	225.5954	12354.4	2.218929	16.84329	1.763095	0.661131	2.687747	0.082807	0.728316	0.270977	512.8559	3.590496	515.3054	10.86221	527.1579	56.69634	512.9	3.6	97.3	465
19AR109	Spot 205	216.5322	12067.45	3.929795	17.37823	2.186233	0.641626	2.328892	0.082815	0.66779	0.286742	512.9024	3.292397	503.3125	9.242703	460.8976	49.47557	512.9	3.3	111.3	181
19AR109	Spot 40	225.2577	18846.42	1.121888	17.05548	2.355365	0.659657	2.529852	0.082837	0.753766	0.297949	513.0348	3.717205	514.4043	10.21033	521.4286	52.98614	513.0	3.7	98.4	39
19AR109	Spot 595	362.9021	7781.479	9.619269	16.11815	1.303412	0.684252	3.395356	0.082858	1.45361	0.428117	513.1585	7.170156	529.3412	14.00722	600.6581	66.44413	513.2	7.2	85.4	528
19AR109	Spot 382	268.2568	28694.32	2.564334	17.27277	1.918668	0.657213	2.107883	0.082929	0.692417	0.328489	513.5796	3.418146	512.9075	8.488173	510.8743	43.75089	513.6	3.4	100.5	333
19AR109	Spot 270	109.4801	12505.83	2.334812	16.35457	2.671951	0.68475	2.974253	0.082946	0.748982	0.251822	513.6827	3.698094	529.641	12.27511	599.9505	62.31792	513.7	3.7	85.6	239
19AR109	Spot 293	87.71101	6543.719	1.835605	17.36106	2.723616	0.632474	3.516463	0.08295	0.848565	0.241312	513.7077	4.189981	497.6358	13.83436	425.3185	76.1443	513.7	4.2	120.8	258
19AR109	Spot 246	458.3353	25065.37	8.181975	17.21925	1.354357	0.657036	1.822323	0.082955	0.80425	0.441332	513.7388	3.971394	512.7996	7.337029	509.5822	35.96405	513.7	4.0	100.8	218
19AR109	Spot 22	114.3497	6915.737	2.049843	17.09073	4.517663	0.644378	4.92993	0.083039	0.87702	0.177897	514.2368	4.334767	505.0129	19.6184	464.4274	107.5551	514.2	4.3	110.7	22
19AR109	Spot 411	210.6069	17648.16	2.228117	17.51005	1.276602	0.641271	1.632715	0.083046	0.840512	0.514794	514.2775	4.154637	503.0925	6.477498	453.487	31.09529	514.3	4.2	113.4	362
19AR109	Spot 510	209.1918	15029.84	2.645115	16.88335	2.494612	0.664554	2.666248	0.083057	0.840753	0.315332	514.3466	4.156368	517.3959	10.80885	531.8581	55.44516	514.3	4.2	96.7	450
19AR109	Spot 474	155.8891	14033.12	2.488804	17.07286	2.718682	0.656885	2.980956	0.08311	0.894749	0.300155	514.6605	4.425895	512.7067	12.00059	504.966	62.59654	514.7	4.4	101.9	417
19AR109	Spot 89	269.9227	17126.53	1.832793	16.85337	1.690884	0.667858	2.239118	0.08315	0.896131	0.400216	514.8969	4.434688	519.4094	9.104239	540.2777	44.89497	514.9	4.4	95.3	78
19AR109	Spot 95	317.0841	23801.52	4.3379	17.07673	1.15699	0.661538	1.46047	0.083156	0.769989	0.52722	514.934	3.810711	515.5546	5.904349	519.2417	27.2184	514.9	3.8	99.2	82
19AR109	Spot 232	187.0212	11654.42	1.752612	16.90603	2.438527	0.662194	2.661802	0.083215	0.781157	0.293469	515.2846	3.868511	515.9554	10.76777	519.8622	55.84605	515.3	3.9	99.1	206
19AR109	Spot 207	238.0252	18262.89	2.985946	17.24692	1.544025	0.653256	2.069189	0.08322	0.840683	0.406286	515.3122	4.163514	510.4804	8.302006	489.8768	41.73233	515.3	4.2	105.2	183
19AR109	Spot 363	524.419	29288.41	27.24858	17.1071	0.917037	0.663781	1.216911	0.083276	0.703556	0.57815	515.6458	3.486557	516.9242	4.929704	523.5119	21.76545	515.6	3.5	98.5	315
19AR109	Spot 501	365.5409	25967.35	6.653345	17.47585	1.440116	0.648788	1.694733	0.08338	0.791384	0.466967	516.2674	3.926338	507.7326	6.771356	470.4571	33.17795	516.3	3.9	109.7	442
19AR109	Spot 381	161.3868	12681.79	3.579985	17.11281	2.047996	0.659897	2.479973	0.08341	0.955873	0.385437	516.446	4.744	514.5512	10.0112	507.081	50.35306	516.4	4.7	101.8	332
19AR109	Spot 542	147.0576	10959.71	1.279436	17.91516	2.320112	0.624599	3.150971	0.083424	0.851274	0.270162	516.5308	4.225542	492.7256	12.30129	384.5019	68.17452	516.5	4.2	134.3	481
19AR109	Spot 583	747.747	67748.27	10.44301	17.10447	0.922977	0.66877	1.2189	0.083462	0.758767	0.622501	516.7536	3.767919	519.964	4.960001	535.0539	20.89394	516.8	3.8	96.6	516
19AR109	Spot 503	389.3548	3236.834	11.27443	15.98888	1.014357	0.665716	4.698569	0.083498	2.723345	0.579612	516.9656	13.52905	518.1043	19.06929	524.0622	83.99611	517.0	13.5	98.6	444
19AR109	Spot 135	271.4579	25531.94	2.267167	16.95344	1.73387	0.670513	1.967815	0.083506	0.84363	0.428714	517.0157	4.191377	521.0246	8.020116	539.569	38.88629	517.0	4.2	95.8	119
19AR109	Spot 586	331.693	21801.97	4.450542	17.21506	1.256304	0.659567	1.50713	0.083581	0.724156	0.480487	517.463	3.600788	514.3494	6.082052	501.4961	29.10029	517.5	3.6	103.2	519
19AR109	Spot 450	297.9624	18890.51	3.684278	17.14015	1.028455	0.660411	1.27522	0.083589	0.660137	0.517665	517.5073	3.282727	514.8654	5.150122	504.0915	24.03117	517.5	3.3	102.7	396
19AR109	Spot 114	446.4361	5303.831	3.830558	16.94668	0.962945	0.64829	1.883614	0.083594	0.911282	0.483794	517.5401	4.531896	507.4256	7.522554	463.0794	36.55658	517.5	4.5	111.8	99
19AR109	Spot 129	241.5723	16298.1	1.949189	17.22563	1.397686	0.657325	2.18948	0.083613	0.769094	0.351268	517.6539	3.82559	512.9763	8.817678	493.1417	45.20618	517.7	3.8	105.0	113
19AR109	Spot 515	210.2756	20119.29	2.518522	17.09632	2.478198	0.663463	2.652038	0.083655	0.709212	0.267421	517.9027	3.529355	516.73	10.74062	512.5127	56.1743	517.9	3.5	101.1	455
19AR109	Spot 337	189.3253	14867.63	1.754495	16.76935	2.471458	0.676195	2.78964	0.083667	0.844231	0.302631	517.9719	4.20181	524.4721	11.4273	553.8002	58.02316	518.0	4.2	93.5	294
19AR109	Spot 421	160.9234	18615.85	5.070927	17.07153	2.537126	0.664213	2.684828	0.083679	0.769086	0.286456	518.0426	3.82831	517.1879	10.88082	514.3497	56.51927	518.0	3.8	100.7	371
19AR109	Spot 324	172.3198	13529.84	3.126504	17.00189	2.698562	0.663893	2.84359	0.08371	0.771286	0.271237	518.2316	3.840607	516.9926	11.52095	512.4939	60.1535	518.2	3.8	101.1	282
19AR109	Spot 282	647.9523	44322.5	1.042219	17.26183	0.89849	0.665555	1.143628	0.083751	0.686968	0.600692	518.4737	3.422278	518.0062	4.640262	516.8792	20.06191	518.5	3.4	100.3	250
19AR109	Spot 390	359.5749	33618.46	2.489196	17.16272	1.401156	0.667091	1.64422	0.083758	0.80211	0.487836	518.5133	3.996176	518.9421	6.680699	521.7643	31.49867	518.5	4.0	99.4	341
19AR109	Spot 405	223.7215	14820.13	4.527178	17.22377	1.751799	0.656322	1.996852	0.083825	0.769032	0.385122	518.9134	3.83422	512.3618	8.034466	484.2202	40.69384	518.9	3.8	107.2	356
19AR109	Spot 281	240.1962	16576.76	1.963035	17.22668	1.803605	0.661477	2.34037	0.083865	0.785855	0.335783	519.1518	3.919826	515.517	9.461224	500.4164	48.56842	519.2	3.9	103.7	249
19AR109	Spot 552	113.5335	8696.434	1.870763	16.10673	5.329733	0.696529	5.848538	0.083894	0.915498	0.156535	519.3221	4.567921	536.7155	24.38588	612.2466	124.8851	519.3	4.6	84.8	488
19AR109	Spot 328	155.7881	11788.06	2.044432	16.88321	2.698921	0.668997	3.327414	0.083911	0.727033	0.218498	519.4261	3.628261	520.1021	13.5435	524.0056	71.24028	519.4	3.6	99.1	286
19AR109	Spot 227	211.9721	15892.72	2.365264	16.91532	1.376832	0.671187	1.617585	0.084033	0.72646	0.449102	520.1488	3.630248	521.4341	6.596624	528.0424	31.68168	520.1	3.6	98.5	201
19AR109	Spot 423	381.0659	28056.82	2.28614	17.18897	1.188127	0.667099	1.542766	0.084237	0.878475	0.569416	521.3636	4.399734	518.9469	6.268509	509.2955	27.89179	521.4	4.4	102.4	373
19AR109	Spot 530	211.318	14482	0.785558	17.21797	1.997976	0.659266	2.693997	0.084303	0.780594	0.289753	521.7552	3.912328	514.165	10.86897	481.5061	56.96824	521.8	3.9	108.4	469
19AR109	Spot 194	430.6336	17890.29	1.517888	17.40265	0.969188	0.658663	1.307745	0.084306	0.706016	0.539873	521.7767	3.538682	513.7959	5.27305	479.3901	24.33	521.8	3.5	108.8	170
19AR109	Spot 355	396.7101	29616.06	3.213018	17.2485	1.196703	0.666341	1.467701	0.08437	0.689865	0.470031	522.1561	3.460148	518.4852	5.959435	503.3209	28.52165	522.2	3.5	103.7	310
19AR109	Spot 509	324.1047	32266.67	11.73574	17.10916	1.476391	0.672268	1.721344	0.084407	0.843181	0.489839	522.3736	4.230822	522.0906	7.026535	521.7865	32.92629	522.4	4.2	100.1	449
19AR109	Spot 502	289.094	24985.69	10.2529	17.43168	1.895361	0.657951	2.059939	0.084408	0.671269	0.325868	522.3813	3.368268	513.3599	8.30072	474.3749	43.07948	522.4	3.4	110.1	443
19AR109	Spot 212	244.6461	13695.14	2.21031	17.2326	1.400838	0.659615	1.667743	0.084494	0.666422	0.399595	522.8893	3.34707	514.3783	6.730517	477.6796	33.81471	522.9	3.3	109.5	187
19AR109	Spot 158	143.3706	9812.51	3.188768	16.58999	2.890681	0.683027	3.603014	0.084521	0.850706	0.23611	523.0529	4.27391	528.6024	14.84819	553.6083	76.43997	523.1	4.3	94.5	137
19AR109	Spot 559	248.5967	18046.92	1.484015	17.03891	2.005609	0.671816	2.168046	0.084534	0.706842	0.326027	523.1302	3.551646	521.8162	8.8465	517.0045	44.99087	523.1	3.6	101.2	493
19AR109	Spot 537	398.8094	27430.35	1.269249	17.21304	0.875619	0.668292	1.187941	0.084568	0.723763	0.609259	523.3325	3.63802	519.6732	4.831947	504.5568	20.70684	523.3	3.6	103.7	476
19AR109	Spot 348	117.3089	10316.85	1.8463	16.61774	3.370874	0.684205	3.563889	0.084575	0.857315	0.240556	523.3739	4.309649	529.3127	14.70198	555.9721	75.49478	523.4	4.3	94.1	305
19AR109	Spot 534	176.6833	13942.4	1.888759	17.82997	2.172624	0.640738	2.516715	0.084802	0.830814	0.330118	524.7225	4.18676	502.7633	9.979742	404.9859	53.18337	524.7	4.2	129.6	473
19AR109	Spot 521	150.8834	8109.924	4.784868	17.11467	2.313433	0.659973	3.64603	0.084847	0.878431	0.240928	524.9884	4.428875	514.5978	14.71993	469.6718	78.37424	525.0	4.4	111.8	460
19AR109	Spot 428	254.3443	18991.24	1.279163	16.71838	1.760252	0.68827	1.948941	0.084862	0.72107	0.36998	525.0779	3.636086	531.7602	8.067791	561.4988	39.45995	525.1	3.6	93.5	378
19AR109	Spot 197	542.971	2493.381	6.210948	15.75723	0.974189	0.675761	1.85888	0.085108	1.058172	0.569252	526.5388	5.350213	524.2092	7.611505	515.0091	33.59146	526.5	5.4	102.2	173
19AR109	Spot 211	286.5594	20421.04	1.778329	16.99849	1.299506	0.679562	1.498838	0.085241	0.627913	0.418933	527.3328	3.179376	526.5099	6.157766	523.8773	29.865	527.3	3.2	100.7	186
19AR109	Spot 26	143.3291	11720.2	1.378193	17.51713	3.45166	0.655933	3.619546	0.085386	0.845162	0.233499	528.1924	4.286093	512.1232	14.55896	441.9668	78.30449	528.2	4.3	119.5	26
19AR109	Spot 499	686.8014	66979.42	104.1063	16.84209	1.185964	0.694765	1.509913	0.085555	0.904996	0.59937	529.193	4.597875	535.6593	6.285153	564.2175	26.32778	529.2	4.6	93.8	440
19AR109	Spot 284	201.6851	13291.73	2.203068	17.09916	1.86507	0.681418	2.064901	0.086217	0.671005	0.324957	533.1278	3.433384	527.6312	8.497226	504.8805	42.99223	533.1	3.4	105.6	252
19AR109	Spot 412	330.4591	27044.93	5.13069	17.13189	1.161275	0.68926	1.800523	0.086882	1.089462	0.605081	537.0721	5.614084	532.356	7.45973	513.1421	31.48294	537.1	5.6	104.7	363
19AR109	Spot 570	588.6797	39799.03	2.797292	17.00831	0.947844	0.700484	1.189963	0.087225	0.677328	0.569201	539.106	3.502999	539.0801	4.977285	539.9438	21.39888	539.1	3.5	99.8	503
19AR109	Spot 431	287.6747	26943.92	3.205216	16.92906	1.329686	0.705467	1.735608	0.087728	0.910042	0.524336	542.0882	4.731505	542.0508	7.289913	542.8239	32.31452	542.1	4.7	99.9	381
19AR109	Spot 327	176.9204	16052.3	3.402532	16.60958	1.997268	0.719134	2.461138	0.088291	1.12277	0.4562	545.4209	5.871918	550.1556	10.45397	570.7644	47.65745	545.4	5.9	95.6	285
19AR109	Spot 504	1728.22	141714.1	16.9297	16.89589	0.684893	0.722124	1.04581	0.089001	0.789541	0.754957	549.6258	4.159676	551.9199	4.452798	562.3257	14.96333	549.6	4.2	97.7	445
19AR109	Spot 582	321.6538	21681.53	5.906644	16.97381	1.44908	0.71552	2.579565	0.089242	2.075804	0.804711	551.0521	10.96351	548.019	10.92495	536.3876	33.52489	551.1	11.0	102.7	515
19AR109	Spot 154	214.8565	16351.23	1.548977	16.76428	1.603163	0.723604	1.89202	0.089682	0.728686	0.385137	553.6587	3.866038	552.7925	8.065447	550.1912	38.11627	553.7	3.9	100.6	134
19AR109	Spot 17	430.7997	41509.62	6.153791	16.42845	1.10745	0.761933	1.459579	0.091405	0.936482	0.641611	563.8417	5.055945	575.1247	6.409006	620.8821	24.1546	563.8	5.1	90.8	17
19AR109	Spot 350	207.9572	19482.69	6.53812	16.65144	1.682302	0.746691	2.109798	0.091458	0.957771	0.453963	564.1563	5.173641	566.3024	9.158141	575.857	40.86717	564.2	5.2	98.0	307
19AR109	Spot 262	266.6386	19719.3	10.46901	15.69615	1.789438	0.801134	2.213854	0.092496	0.96639	0.436519	570.28	5.274392	597.4681	9.998898	703.0148	42.37757	570.3	5.3	81.1	233
19AR109	Spot 204	562.4491	62479.56	7.667355	16.78078	0.810414	0.75929	1.095654	0.093012	0.66392	0.605958	573.3252	3.642066	573.6004	4.801499	575.6284	18.93517	573.3	3.6	99.6	180
19AR109	Spot 106	301.0172	22006.65	1.620156	16.58251	1.505073	0.768038	1.724641	0.09346	0.696974	0.404127	575.968	3.840237	578.6366	7.60725	590.0794	34.19264	576.0	3.8	97.6	92
19AR109	Spot 498	64.1315	4139.184	2.191587	16.79967	4.510108	0.724288	5.549094	0.094206	1.187713	0.214037	580.3613	6.591849	553.1949	23.67184	443.8267	120.619	580.4	6.6	130.8	439
19AR109	Spot 415	307.735	34565.77	6.058588	15.79808	1.010936	0.813491	1.554966	0.094298	1.011823	0.650704	580.9085	5.620714	604.4104	7.082642	694.4677	25.16583	580.9	5.6	83.6	366
19AR109	Spot 577	849.0125	11593.6	1.409272	16.30391	0.690121	0.782661	2.309043	0.094624	0.740369	0.320639	582.8291	4.125769	587.0001	10.29394	604.0849	47.33615	582.8	4.1	96.5	510
19AR109	Spot 331	919.2942	74023.29	3.994092	16.52382	0.712919	0.78503	1.062612	0.094663	0.774832	0.729176	583.0531	4.319401	588.3486	4.745134	609.7512	15.70742	583.1	4.3	95.6	288
19AR109	Spot 369	290.8663	23241.16	2.989125	15.61389	0.961048	0.827794	1.422432	0.094703	0.938786	0.659987	583.2919	5.235436	612.3874	6.541274	722.4203	22.6738	583.3	5.2	80.7	321
19AR109	Spot 463	211.1425	15586.66	8.976779	15.2047	1.665217	0.844141	2.100441	0.094784	1.123516	0.534895	583.7666	6.270511	621.4284	9.762808	761.9803	37.41595	583.8	6.3	76.6	407
19AR109	Spot 508	523.0502	46372.05	5.604076	16.74378	1.007781	0.777596	1.288634	0.09531	0.740336	0.574513	586.8622	4.152855	584.1114	5.723809	574.3823	22.93144	586.9	4.2	102.2	448
19AR109	Spot 45	136.3908	8559.16	4.00555	16.40822	2.267598	0.781566	2.782478	0.095843	0.762921	0.274188	590.001	4.301403	586.3763	12.39501	573.3353	58.22522	590.0	4.3	102.9	44
19AR109	Spot 575	208.6072	6572.865	1.997402	16.27146	2.300823	0.788236	2.835808	0.096639	0.759662	0.267882	594.684	4.315478	590.1705	12.6929	573.7787	59.44526	594.7	4.3	103.6	508
19AR109	Spot 145	251.601	10996.77	3.608222	15.93762	1.104194	0.816872	2.495129	0.096783	0.851204	0.341146	595.5303	4.842075	606.3018	11.39124	647.7249	50.38505	595.5	4.8	91.9	129
19AR109	Spot 35	187.7246	16358.8	1.103462	16.66397	1.602242	0.792621	1.84463	0.097409	0.854602	0.463292	599.2095	4.890066	592.6577	8.281822	568.6222	35.57133	599.2	4.9	105.4	34
19AR109	Spot 46	324.2268	13866.42	7.221557	13.8442	0.976495	0.954914	2.005232	0.097446	1.189057	0.592977	599.4256	6.806172	680.6581	9.94592	960.3908	33.00052	599.4	6.8	62.4	45
19AR109	Spot 452	322.6964	31388.89	1.40511	16.51216	0.894585	0.809122	1.124926	0.097998	0.653931	0.58131	602.6653	3.762403	601.9614	5.10862	600.2333	19.81579	602.7	3.8	100.4	398
19AR109	Spot 136	125.0191	13067.58	2.27212	16.42762	1.745644	0.805372	2.471232	0.098039	1.266662	0.512563	602.9049	7.290531	599.8542	11.19414	589.2622	46.04045	602.9	7.3	102.3	120
19AR109	Spot 565	821.4627	60460.65	1.168644	16.44069	0.897823	0.815574	1.180387	0.09806	0.754634	0.63931	603.0313	4.344315	605.5762	5.384034	616.0307	19.61538	603.0	4.3	97.9	499
19AR109	Spot 247	296.3141	28606.22	5.617229	16.39412	1.108496	0.82135	1.408247	0.098564	0.8065	0.572698	605.9864	4.664603	608.8014	6.448361	620.2088	24.92389	606.0	4.7	97.7	219
19AR109	Spot 94	161.3142	10721.82	2.836512	16.51705	2.816171	0.803129	3.44822	0.098803	0.755808	0.219188	607.3874	4.381047	598.5921	15.59615	566.3786	73.28227	607.4	4.4	107.2	81
19AR109	Spot 519	455.3902	33018	4.866392	15.98746	0.751461	0.842908	1.041292	0.098812	0.702019	0.67418	607.4442	4.069623	620.7489	4.835955	670.5089	16.46603	607.4	4.1	90.6	458
19AR109	Spot 544	193.5254	14200.32	3.654534	16.4985	1.845811	0.808493	2.126971	0.098854	0.700841	0.329502	607.6876	4.064347	601.6084	9.655262	579.7268	43.61344	607.7	4.1	104.8	483
19AR109	Spot 593	346.1466	31522.82	2.491624	16.65435	1.269997	0.809294	1.636479	0.09895	0.783669	0.478875	608.2535	4.548721	602.0581	7.432679	579.7475	31.19112	608.3	4.5	104.9	526
19AR109	Spot 486	778.2963	82156.07	2.590884	16.37422	0.757052	0.827891	0.953646	0.099037	0.575543	0.603519	608.7606	3.343333	612.4416	4.385738	626.9949	16.41002	608.8	3.3	97.1	427
19AR109	Spot 525	369.7493	32796.7	14.43217	16.00333	0.948024	0.843792	1.371379	0.099126	0.805508	0.587371	609.2872	4.683063	621.236	6.372599	665.9565	23.74835	609.3	4.7	91.5	464

19AR109	Spot 141	426.1561	3270.039	1.018019	15.6833	0.988283	0.808951	1.462933	0.099194	0.921897	0.630171	609.6845	5.363056	601.8655	6.642873	573.4656	24.69264	609.7	5.4	106.3	125
19AR109	Spot 377	198.8501	17924.16	4.437555	16.5005	1.478445	0.819742	1.842224	0.099271	0.729224	0.395839	610.1373	4.245198	607.9045	8.426548	600.5133	36.64744	610.1	4.2	101.6	329
19AR109	Spot 235	126.9074	15175.5	1.868754	16.49435	2.93283	0.815406	3.221735	0.099294	0.793026	0.246149	610.2719	4.617594	605.4821	14.69434	588.5332	67.76033	610.3	4.6	103.7	208
19AR109	Spot 21	116.4368	9916.656	1.213206	16.15092	2.412023	0.828839	2.683834	0.099656	0.94423	0.351822	612.3941	5.51625	612.9677	12.35098	616.0062	54.26612	612.4	5.5	99.4	21
19AR109	Spot 558	625.809	42996.84	1.58928	16.35919	0.703279	0.834375	1.078177	0.099969	0.691737	0.64158	614.2264	4.052692	616.037	4.979627	623.6151	17.85555	614.2	4.1	98.5	492
19AR109	Spot 100	109.9923	10137.18	4.484168	16.28189	3.071078	0.827586	3.302573	0.100284	0.755262	0.228689	616.075	4.437559	612.2718	15.18623	599.1908	69.62491	616.1	4.4	102.8	86
19AR109	Spot 137	180.4361	23537.14	1.536851	16.31821	1.527809	0.836958	1.786226	0.100369	0.871294	0.487785	616.5729	5.123251	617.4658	8.263814	621.6587	33.64142	616.6	5.1	99.2	121
19AR109	Spot 24	27.9125	2912.518	1.368455	14.36752	5.437671	0.89569	6.15838	0.100702	1.496348	0.242977	618.523	8.825113	649.4217	29.55354	759.2454	126.0875	618.5	8.8	81.5	24
19AR109	Spot 297	690.5319	6497.196	1.056028	16.05017	0.711755	0.83735	1.323624	0.101124	0.653903	0.494024	620.9906	3.871221	617.682	6.125123	606.4956	24.88968	621.0	3.9	102.4	262
19AR109	Spot 266	212.7036	20830.41	0.964787	16.25811	1.44103	0.847572	1.708475	0.101165	0.671216	0.392875	621.2342	3.975206	623.3156	7.958342	631.8372	33.83087	621.2	4.0	98.3	237
19AR109	Spot 491	610.2951	53923.49	8.723698	16.32854	0.929697	0.847039	1.274741	0.101249	0.852804	0.669002	621.7237	5.054433	623.0228	5.93587	628.6616	20.43675	621.7	5.1	98.9	432
19AR109	Spot 340	326.3192	27174.34	10.52789	15.45887	1.225547	0.898825	1.647457	0.101638	0.973421	0.590862	624.0014	5.789436	651.0995	7.918504	747.0958	28.08815	624.0	5.8	83.5	297
19AR109	Spot 276	280.3798	24445.06	3.002866	16.49539	1.43516	0.842385	1.742756	0.101758	0.741985	0.425754	624.7025	4.41769	620.461	8.091072	605.9407	34.12703	624.7	4.4	103.1	244
19AR109	Spot 569	625.767	49246.12	9.42062	15.70074	0.931023	0.887025	1.564655	0.101798	1.210396	0.773587	624.9359	7.20912	644.7697	7.468173	715.782	21.07935	624.9	7.2	87.3	502
19AR109	Spot 322	809.6633	9981.164	6.362256	15.1246	0.795432	0.914879	1.42752	0.102853	1.002474	0.702248	631.1043	6.026828	659.6482	6.925341	759.3915	21.43451	631.1	6.0	83.1	280
19AR109	Spot 86	390.4606	24470.83	200.0858	15.96454	1.207741	0.876351	1.473561	0.102884	0.780613	0.529746	631.2887	4.694317	639.01	6.988257	667.3254	26.74257	631.3	4.7	94.6	75
19AR109	Spot 523	196.0746	14213.88	1.897919	16.22249	1.097631	0.863538	1.617133	0.103779	0.814008	0.503365	636.5165	4.933709	632.0523	7.608989	617.0359	30.18888	636.5	4.9	103.2	462
19AR109	Spot 68	358.2018	34040.18	20.58388	15.21917	1.045988	0.934754	1.349744	0.104294	0.74069	0.548764	639.5252	4.509517	670.1326	6.621538	775.3412	23.73657	639.5	4.5	82.5	66
19AR109	Spot 492	280.1736	22997.97	2.231468	16.23111	1.051923	0.876805	1.363735	0.104795	0.792597	0.581196	642.4465	4.846501	639.2557	6.469187	628.912	23.93052	642.4	4.8	102.2	433
19AR109	Spot 32	156.4508	12463.45	2.180103	14.99356	2.4	0.946241	3.268335	0.104873	1.768934	0.541234	642.9036	10.82385	676.1432	16.13608	789.3757	57.67114	642.9	10.8	81.4	31
19AR109	Spot 37	198.7445	21310.42	1.195813	16.10158	1.939518	0.887783	2.119525	0.105012	0.745238	0.351606	643.7167	4.565482	645.1774	10.12133	651.208	42.61861	643.7	4.6	98.8	36
19AR109	Spot 163	270.3763	27549.64	5.557675	15.34118	1.45577	0.934427	1.963809	0.105147	1.08016	0.550033	644.4997	6.624939	669.9613	9.632418	757.4564	34.60231	644.5	6.6	85.1	142
19AR109	Spot 199	339.4672	47496.33	1.11007	16.42932	1.098012	0.885686	1.584696	0.106041	1.13046	0.71336	649.7131	6.986749	644.0489	7.557782	625.1555	23.95705	649.7	7.0	103.9	175
19AR109	Spot 332	660.5571	12130.71	1.429505	16.08249	0.677991	0.891396	1.833414	0.106318	0.787988	0.429793	651.3262	4.881612	647.1189	8.773835	633.4252	35.65978	651.3	4.9	102.8	289
19AR109	Spot 196	500.7367	20612.59	3.871895	15.15688	0.857008	0.971257	1.171591	0.108005	0.743387	0.63451	661.154	4.67129	689.1115	5.861405	782.3681	19.02731	661.2	4.7	84.5	172
19AR109	Spot 485	299.7743	29317.64	3.712979	14.93554	0.932441	0.988659	1.194499	0.108359	0.702562	0.588165	663.21	4.427792	698.0359	6.029857	812.7831	20.21166	663.2	4.4	81.6	426
19AR109	Spot 571	88.24026	13502.05	3.526699	15.29787	2.823111	0.960557	3.187241	0.108687	0.817798	0.256585	665.12	5.168138	683.5851	15.85711	745.758	65.14641	665.1	5.2	89.2	504
19AR109	Spot 484	38.93551	3757.421	4.770755	14.49029	5.727532	0.974374	7.209812	0.108891	1.117574	0.155007	666.3059	7.07455	690.7156	36.14377	771.926	150.1081	666.3	7.1	86.3	425
19AR109	Spot 90	138.4341	19748.78	2.480651	16.11234	2.657586	0.919064	2.884628	0.109119	0.88684	0.307436	667.629	5.624518	661.8649	14.02826	643.233	59.01287	667.6	5.6	103.8	79
19AR109	Spot 527	315.7717	45957.09	11.62975	15.27665	0.971519	0.983067	1.801234	0.109993	1.455609	0.808117	672.7072	9.298394	695.1765	9.06687	769.4333	22.33994	672.7	9.3	87.4	466
19AR109	Spot 170	344.1272	31322.84	1.807639	15.97548	1.03295	0.939948	1.350193	0.110041	0.761014	0.563633	672.9846	4.863237	672.8549	6.642716	673.338	23.85182	673.0	4.9	99.9	149
19AR109	Spot 424	586.0877	51300.61	2.460311	15.9551	0.745202	0.946553	1.01609	0.110414	0.676344	0.665635	675.1528	4.335368	676.3062	5.017001	681.0584	16.18972	675.2	4.3	99.1	374
19AR109	Spot 591	357.3652	27908.56	2.210086	15.96565	1.247029	0.951365	1.671087	0.11154	0.780847	0.467269	681.6838	5.051131	678.8132	8.272701	670.2374	31.61753	681.7	5.1	101.7	524
19AR109	Spot 471	1220.586	145218.4	3.478851	15.8474	0.571797	0.964538	0.928088	0.11154	0.713981	0.769303	681.6869	4.61861	685.6445	4.626801	699.5598	12.61107	681.7	4.6	97.4	414
19AR109	Spot 453	273.582	24262.91	2.813275	15.91796	1.071288	0.960246	1.333701	0.112283	0.67624	0.50704	685.9929	4.400657	683.424	6.633862	675.9156	24.57742	686.0	4.4	101.5	399
19AR109	Spot 279	328.3343	41501.95	2.185141	16.03423	1.258696	0.965291	1.48556	0.112821	0.741716	0.499284	689.1125	4.847545	686.0336	7.408992	676.8599	27.50204	689.1	4.8	101.8	247
19AR109	Spot 301	361.7423	33531.82	10.71904	15.04743	1.013424	1.049025	1.356499	0.114877	0.893297	0.658531	701.0103	5.933638	728.3993	7.05172	814.5635	21.35785	701.0	5.9	86.1	266
19AR109	Spot 261	170.1923	25586.18	2.896981	15.06212	1.229288	1.054887	1.73928	0.116584	1.045269	0.600978	710.8703	7.035467	731.3001	9.066276	795.3184	29.16228	710.9	7.0	89.4	232
19AR109	Spot 461	322.5149	31560.52	5.235876	14.4876	0.956549	1.102563	1.372243	0.117171	0.849174	0.618822	714.2607	5.741373	754.589	7.306727	876.9353	22.31275	714.3	5.7	81.4	405
19AR109	Spot 61	169.7317	21918.61	2.008631	15.52361	1.185714	1.042239	1.438267	0.118816	0.670947	0.466497	723.7432	4.593268	725.0311	7.453119	729.9593	26.93903	723.7	4.6	99.1	59
19AR109	Spot 176	569.4022	62209.2	11.13981	14.49792	0.595458	1.12794	0.959246	0.119352	0.742052	0.773578	726.8333	5.100536	766.7711	5.162861	885.8907	12.56388	726.8	5.1	82.0	155
19AR109	Spot 241	376.6841	45847.29	6.439181	14.61371	1.006341	1.121321	1.4163	0.119676	0.951016	0.671479	728.697	6.552697	763.6078	7.601809	868.096	21.74151	728.7	6.6	83.9	213
19AR109	Spot 543	298.3644	29302.06	2.731831	15.62309	1.0478	1.055403	1.312821	0.120929	0.708455	0.539643	735.9079	4.927001	731.5552	6.84484	719.1476	23.44731	735.9	4.9	102.3	482
19AR109	Spot 15	93.84432	8346.782	5.790895	14.16423	2.332971	1.15347	2.831258	0.121574	0.934516	0.330071	739.6194	6.53009	778.8806	15.39962	893.9821	55.19094	739.6	6.5	82.7	15
19AR109	Spot 465	384.5237	31899.91	4.015987	14.50606	1.090723	1.185478	1.384576	0.126032	0.823574	0.59482	765.1892	5.94225	793.8618	7.626091	876.1629	23.02219	765.2	5.9	87.3	409
19AR109	Spot 529	428.0637	47351.83	3.891503	14.19593	0.848437	1.212748	1.36212	0.126191	1.028103	0.754781	766.0995	7.42628	806.4531	7.580396	920.4809	18.36948	766.1	7.4	83.2	468
19AR109	Spot 514	312.9914	34719.52	11.48746	15.11923	0.940467	1.14385	1.2314	0.126767	0.740167	0.601078	769.3933	5.368082	774.3344	6.671294	789.5028	20.65508	769.4	5.4	97.5	454
19AR109	Spot 555	540.3416	58628.49	7.426795	14.75518	0.781591	1.196373	1.148695	0.12904	0.778536	0.677757	782.3892	5.736059	798.9107	6.353323	846.1978	17.58785	782.4	5.7	92.5	490
19AR109	Spot 65	117.8596	11806.24	4.193034	15.1897	1.739022	1.14587	2.353421	0.129172	0.727222	0.309006	783.1418	5.362834	775.2906	12.76099	753.6459	47.23444	783.1	5.4	103.9	63
19AR109	Spot 168	505.5859	44240.94	6.49648	14.43907	0.6495	1.224095	1.056082	0.129192	0.802614	0.759993	783.2531	5.9196	811.6463	5.901929	891.2449	14.17565	783.3	5.9	87.9	147
19AR109	Spot 28	248.63	28933.02	3.787843	15.13079	1.41997	1.168351	1.770055	0.129434	0.985265	0.55663	784.6375	7.278804	785.8728	9.684417	790.2746	30.8814	784.6	7.3	99.3	28
19AR109	Spot 1	277.8514	4585.088	2.346404	14.0247	0.907212	1.218195	1.709358	0.130053	0.690844	0.404154	788.1665	5.125299	808.9492	9.532189	867.4967	32.41311	788.2	5.1	90.9	1
19AR109	Spot 178	146.6047	18234.92	1.790814	14.70568	1.380406	1.214165	1.536288	0.131352	0.512628	0.333679	795.5761	3.836723	807.1029	8.554224	839.9245	30.17422	795.6	3.8	94.7	156
19AR109	Spot 259	955.4389	60193.42	69.06412	13.91089	1.162709	1.30592	2.097683	0.132606	1.729655	0.824555	802.7125	13.05453	848.3318	12.0632	970.5234	24.21538	802.7	13.1	82.7	230
19AR109	Spot 223	251.0484	26118.1	5.490084	14.39106	1.127666	1.257384	1.484669	0.132944	0.929067	0.625774	804.6413	7.027926	826.7316	8.397151	887.4991	23.91138	804.6	7.0	90.7	198
19AR109	Spot 592	275.5606	19403.44	8.648136	14.28336	0.89595	1.305925	1.405552	0.137339	0.915704	0.65149	829.5948	7.128134	848.3344	8.082751	898.553	21.99692	829.6	7.1	92.3	525
19AR109	Spot 116	198.1343	34565.13	1.711229	14.61487	1.384944	1.318634	1.568191	0.140672	0.706607	0.450587	848.4595	5.617486	853.9151	9.055917	869.0002	29.01495	848.5	5.6	97.6	101
19AR109	Spot 495	199.5918	18055.51	1.081421	14.63367	1.260769	1.324174	1.558053	0.142825	0.871074	0.559078	860.6162	7.017738	856.3384	9.013634	846.2012	26.85031	860.6	7.0	101.7	436
19AR109	Spot 568	320.5261	3288.502	5.338796	13.60777	1.690423	1.387483	6.238536	0.146115	0.995864	0.159631	879.1506	8.184384	883.6266	36.82904	895.7487	127.2125	879.2	8.2	98.1	501
19AR109	Spot 285	85.53979	15697.51	2.101304	14.36409	2.705523	1.507167	2.867363	0.159286	0.76215	0.265802	952.8075	6.750655	933.293	17.50386	888.386	57.10709	888.4	57.1	107.3	253
19AR109	Spot 300	125.4028	12350.64	1.072955	14.29329	1.758118	1.512221	2.04707	0.158919	0.745497	0.364178	950.7671	6.590029	935.3377	12.51244	900.0396	39.32269	900.0	39.3	105.6	265
19AR109	Spot 459	36.51431	4944.717	4.665438	14.19654	6.324937	1.412556	7.020429	0.152403	2.672018	0.380606	914.4186	22.77971	894.2343	41.76059	845.5452	135.1765	914.4	22.8	108.1	404
19AR109	Spot 245	290.0038	37871.41	2.517192	14.24094	0.879109	1.432959	1.198439	0.148935	0.791339	0.660308	894.9923	6.612761	902.7854	7.167241	922.8186	18.5166	922.8	18.5	97.0	217
19AR109	Spot 540	320.0727	37179.97	7.102517	14.10152	0.730406	1.521066	0.996779	0.156952	0.674503	0.676683	939.8177	5.898679	938.9064	6.106578	937.6424	15.041	937.6	15.0	100.2	479
19AR109	Spot 598	53.4143	7820.854	0.830165	13.75432	3.025721	1.4973	3.314733	0.153946	0.915217	0.276106	923.0431	7.870901	929.289	20.18245	945.0476	65.2641	945.0	65.3	97.7	530
19AR109	Spot 3	188.5256	6463.267	2.723822	13.69324	1.175671	1.419313	1.475258	0.145741	0.747199	0.506487	877.0456	6.12703	897.0741	8.788098	947.6225	26.03016	947.6	26.0	92.6	3
19AR109	Spot 409	189.3936	18292.89	2.033899	13.87912	1.052995	1.508216	1.729656	0.154251	0.895314	0.517625	924.7464	7.712947	933.7177	10.56097	955.8235	30.26094	955.8	30.3	96.7	360
19AR109	Spot 496	70.66123	13128.96	1.615403	13.81248	1.789311	1.640166	2.155047	0.167555	0.959404	0.445189	998.6236	8.875625	985.7762	13.59469	958.1502	39.42626	958.2	39.4	104.2	437
19AR109	Spot 13	278.1034	23334.86	1.817904	13.91751	1.186187	1.410011	1.660511	0.143701	0.985511	0.593499	865.5552	7.982274	893.1626	9.864811	963.0456	27.30774	963.0	27.3	89.9	13
19AR109	Spot 10	239.5834	29048.46	3.749945	13.91818	0.888664	1.548744	1.103149	0.157765	0.629685	0.570807	944.3449	5.531361	949.993	6.806501	964.0106	18.50932	964.0	18.5	98.0	10
19AR109	Spot 63	464.9357	6313.266	3.173027	13.55157	0.646819	1.448333	0.98723	0.14748	0.67342	0.68213	886.8227	5.579466	909.1814	5.929954	964.7907	14.73558	964.8	14.7	91.9	61
19AR109	Spot 436	390.2237	35054.69	5.064912	13.91832	0.800135	1.490599	1.123334	0.151744	0.744524	0.66278	910.7329	6.323431	926.5607	6.826566	965.3284	17.17417	965.3	17.2	94.3	385
19AR109	Spot 148	153.8416	20572.31	1.574185	13.79498	1.121887	1.551305	1.419083	0.157261	0.805101	0.567339	941.5357	7.052739	951.0127	8.761594	973.9027	23.82748	973.9	23.8	96.7	131
19AR109	Spot 524	171.2848	13768.97	2.344754	13.68587	1.323289	1.690802	1.551277	0.171093	0.6987	0.450403	1018.133	6.580381	1005.066	9.89792	977.5863	28.20793	977.6	28.2	104.1	463
19AR109	Spot 238	280.2313	46394.1	1.358771	13.85438	0.800099	1.601327	1.145885	0.161995	0.734479	0.640971	967.8552	6.600784	970.7284	7.16248	978.1364	17.91333	978.1	17.9	98.9	211
19AR109	Spot 566	296.6289	30075.79	3.706225	13.7525	0.83169	1.521153	1.363044	0.153388	0.914589	0.670991	919.9279	7.840808	938.9413	8.350705	984.7115	20.55458	984.7	20.6	93.4	500
19AR109	Spot 263	503.1599	77711.5	1.072728	13.84573	0.727447	1.555119	1.048992	0.156689	0.733773	0.699502	938.3501	6.407694	952.5294	6.48277	986.2989	15.24553	986.3	15.2	95.1	234
19AR109	Spot 454	168.9889	18767.3	3.38965	13.69436	0.844159	1.598469	1.154222	0.160952	0.767059	0.664568	962.0665	6.855351	969.6121	7.209632	987.6294	17.54232	987.6	17.5	97.4	400
19AR109	Spot 250	968.1542	74339.68	3.104526	13.82228	0.644699	1.517401	0.970424	0.152765	0.717933	0.739814	916.4447	6.13318	937.4292	5.939426	987.9823	13.26886	988.0	13.3	92.8	222
19AR109	Spot 490	541.4646	30243.14	3.832131	13.71095	0.808624	1.564276	1.126018	0.157336	0.750238	0.666275	941.9564	6.574861	956.1619	6.974782	989.8562	17.05929	989.9	17.1	95.2	431
19AR109	Spot 553	24.36521	4568.06	0.532819	13.22931	5.472314	1.700446	6.012426	0.170899	1.587046	0.263961	1017.063	14.93237	1008.699	38.46047	991.4588	118.0019	991.5	118.0	102.6	489
19AR109	Spot 142	52.96495	7436.305	2.975758	13.45641	2.796616	1.674053	3.284383	0.168224	0.85085	0.259059	1002.319	7.898292	998.7261	20.88066	991.7193	64.51957	991.7	64.5	101.1	126
19AR109	Spot 198	79.98231	11315.27	1.39148	13.60314	2.250422	1.691151	2.749447	0.169906	0.792672	0.288302	1011.595	7.421135	1005.198	17.54538	992.1803	53.55779	992.2	53.6	102.0	174
19AR109	Spot 600	136.89	18422.35	2.284707	13.61016	1.072438	1.671069	1.469414	0.167587	0.870314	0.592287	998.8021	8.052775	997.5922	9.334595	995.8386	24.06036	995.8	24.1	100.3	532
19AR109	Spot 304	369.029	5731.916	2.919833	13.39501	0.692904	1.589019	1.345085	0.159309	0.999445	0.743035	952.9357	8.853574	965.9124	8.382685	996.4663	18.28797	996.5	18.3	95.6	269
19AR109	Spot 532	667.6273	89172.65	4.575442	13.70601	0.694108	1.665805	1.035481	0.167	0.760952	0.734878	995.5598	7.019736	995.5894	6.570129	996.5381	14.25031	996.5	14.3	99.9	471
19AR109	Spot 468	314.1283	33491.3	1.441535	13.66998	0.767155	1.625714	1.158499	0.162835	0.824523	0.711716	972.5142	7.443058	980.2031	7.283329	998.3319	16.51219	998.3	16.5	97.4	411
19AR109	Spot 393	455.1268	43483.01	6.3655	13.7271	0.902384	1.456985	1.636718	0.145886	1.361693	0.831965	877.861	11.17558	912.7632	9.855305	999.0142	18.44593	999.0	18.4	87.9	344
19AR109	Spot 345	109.572	16542.59	2.023985	13.61783	1.457909	1.707308	1.83942	0.170907	0.93138	0.506344	1017.106	8.7636	1011.275	11.77889	999.5314	32.19886	999.5	32.2	101.8	302
19AR109	Spot 219	587.0447	67401.61	2.259739	13.67685	0.615619	1.636184	0.891514	0.163702	0.632975	0.71	977.3171	5.740067	984.2436	5.618478	1000.588	12.74811	1000.6	12.7	97.7	194
19AR109	Spot 76	246.9038	32748.74	3.821002	13.59337	0.809118	1.692744	1.120342	0.168577	0.718253	0.641102	1004.269	6.67941	1005.798	7.15128	1009.996	17.42881	1010.0	17.4	99.4	74
19AR109	Spot 107	130.1516	15860.62	2.968287	13.54235	1.125217	1.701634	1.458956	0.169457	0.771975	0.529128	1009.121	7.211033	1009.146	9.330911	1010.062	25.09218	1010.1	25.1	99.9	93
19AR109	Spot 455	187.1586	30648.11	2.286437	13.59704	1.076216	1.667241	1.301679	0.165999	0.675917	0.519265	990.0296	6.203246	996.136	8.26189	1010.469	22.53712	1010.5	22.5	98.0	401
19AR109	Spot 487	193.4226	24810.77	4.35001	13.55973	0.795045	1.614098	1.124017	0.160687	0.674825	0.600369	960.5936	6.022477	975.701	7.047222	1010.732	18.21216	1010.7	18.2	95.0	428
19AR109	Spot 397	408.7427	51252.01	4.913991	13.61745	0.823101	1.657458	1.249724	0.164944	0.890283	0.712383	984.1909	8.125999	992.4049	7.914602	1011.47	17.78057	1011.5	17.8	97.3	348
19AR109	Spot 438	276.7352	36886.33	8.866115	13.59476	0.704899	1.666148	1.018112	0.165627	0.62804	0.616868	987.9697	5.752761	995.7199	6.460416	1013.692	16.24008	1013.7	16.2	97.5	387
19AR109	Spot 367	436.7691	16137.23	3.117413	13.48967	0.678499	1.53368	1.015529	0.152249	0.74025	0.728931	913.5555	6.305271	943.974	6.241809	1016.482	14.08386	1016.5	14.1	89.9	319
19AR109	Spot 218	413.8758	54375.12	2.781894	13.56397	0.624678	1.68106	0.972047	0.166807	0.735289	0.756433	994.4954	6.776287	1001.383	6.1887	1017.356	12.87778	1017.4	12.9	97.8	193
19AR109	Spot 11	491.0478	31878.9	3.443469	13.5716	0.651059	1.708648	0.9959	0.16954	0.682119	0.684927	1009.574	6.374333	1011.778	6.378984	1017.417	14.69792	1017.4	14.7	99.2	11
19AR109	Spot 183	389.7246	54762.97	2.115649	13.58408	0.701258	1.697718	1.024603	0.168222	0.662017	0.646121	1002.305	6.14531	1007.673	6.547272	1020.226	15.83257	1020.2	15.8	98.2	159
19AR109	Spot 189	247.37	58332.82	5.770655	13.60155	0.853272	1.709294	1.214111	0.169309	0.802406	0.660899	1008.301	7.489666	1012.02	7.777813	1020.94	18.44562	1020.9	18.4	98.8	165
19AR109	Spot 209	245.166	34925.62	1.233312	13.50806	1.032959	1.689602	1.304248	0.167304	0.626284	0.480187	997.2371	5.786431	1004.613	8.319483	1021.601	23.15795	1021.6	23.2	97.6	184
19AR109	Spot 426	126.8589	22570.39	1.835112	13.48221	1.319054	1.781642	1.662023	0.176325	0.797927	0.480094	1046.867	7.710252	1038.779	10.80942	1022.659	29.51999	1022.7	29.5	102.4	376
19AR109	Spot 512	148.8113	20976.38	2.453966	13.46907	0.999793	1.762364	1.271464	0.174392	0.728378	0.572866	1036.262	6.972487	1031.717	8.236797	1022.956	21.0905	1023.0	21.1	101.3	452
19AR109	Spot 244	484.3542	53531.57	6.218986	13.57491	0.835363	1.724151	1.169358	0.170607	0.795202	0.680033	1015.453	7.47103	1017.573	7.515006	1022.999	17.35135	1023.0	17.4	99.3	216
19AR109	Spot 9	285.1681	44598.46	3.477272	13.55209	0.851287	1.739144	1.11197	0.172065	0.69717	0.626969	1023.481	6.597797	1023.146	7.168876	1023.294	17.53121	1023.3	17.5	100.0	9
19AR109	Spot 172	157.7323	16663.37	1.658121	13.43342	1.101169	1.669161	1.352878	0.164999	0.651162	0.481316	984.4965	5.945145	996.8666	8.590575	1025.041	24.01203	1025.0	24.0	96.0	151
19AR109	Spot 257	285.541	28049.96	2.177305	13.4688	0.881147	1.682552	1.230649	0.16632	0.777849	0.632064	991.8031	7.150561	1001.948	7.837778	1025.072	19.29373	1025.1	19.3	96.8	228
19AR109	Spot 378	481.9582	7858.821	3.124002	13.30332	0.644692	1.491714	2.261283	0.147414	1.903169	0.841633	886.4535	15.76218	927.015	13.74671	1025.637	24.72418	1025.6	24.7	86.4	330
19AR109	Spot 7	238.8414	33551.44	2.609538	13.50722	0.768273	1.762645	1.101134	0.174134	0.696605	0.632625	1034.848	6.659945	1031.821	7.133738	1026.271	17.24928	1026.3	17.2	100.8	7
19AR109	Spot 69	353.7981	51128.23	2.396749	13.50188	0.73148	1.728146	1.054928	0.170563	0.74681	0.707926	1015.21	7.01484	1019.061	6.785346	1028.206	15.06657	1028.2	15.1	98.7	67
19AR109	Spot 200	370.256	85384.47	2.59616	13.56414	0.681143	1.727068	0.988709	0.170439	0.697715	0.705683	1014.529	6.549627	1018.66	6.35796	1028.409	14.1647	1028.4	14.2	98.7	176
19AR109	Spot 376	182.2604	22478.59	3.032598	13.51012	0.856556	1.60661	1.339619	0.158534	1.017975	0.759899	948.623	8.979838	972.7883	8.384083	1028.634	17.62745	1028.6	17.6	92.2	328
19AR109	Spot 310	298.4557	29447.23	2.984631	13.54484	0.801233	1.552608	1.446474	0.15316	1.169176	0.808294	918.6551	10.01049	951.5315	8.933659	1029.223	17.23912	1029.2	17.2	89.3	274
19AR109	Spot 202	224.4179	8326.166	2.829469	13.27823	0.793983	1.734885	1.30979	0.171125	0.757725	0.578509	1018.307	7.137411	1021.566	8.43671	1029.416	21.6192	1029.4	21.6	98.9	178
19AR109	Spot 325	534.4793	20016.51	3.888187	13.41693	0.592437	1.645118	1.076383	0.162254	0.88676	0.823834	969.2905	7.980292	987.6791	6.797592	1029.625	12.33471	1029.6	12.3	94.1	283
19AR109	Spot 228	111.4789	23579.87	4.112495	13.42782	1.422447	1.730224	1.630937	0.170577	0.712971	0.437154	1015.289	6.697467	1019.834	10.4951	1030.463	29.67211	1030.5	29.7	98.5	202
19AR109	Spot 564	348.9528	62188.63	6.746576	13.49076	0.81975	1.742463	1.132063	0.171767	0.759325	0.670744	1021.839	7.175374	1024.376	7.303498	1030.659	16.99182	1030.7	17.0	99.1	498
19AR109	Spot 58	21.44599	2465.267	2.146957	12.57404	7.707076	1.619258	8.059657	0.159573	1.690901	0.209798	954.4037	15.00025	977.7031	50.6342	1031.274	159.495	1031.3	159.5	92.5	57
19AR109	Spot 520	313.095	39148.35	1.509642	13.45891	0.839661	1.774241	1.127324	0.174725	0.683086	0.605936	1038.091	6.549565	1036.074	7.320733	1032.68	18.14108	1032.7	18.1	100.5	459
19AR109	Spot 314	32.52909	4567.122	1.092304	13.03262	5.798541	1.668295	6.080542	0.16417	1.296196	0.213171	979.9093	11.7833	996.5371	38.62073	1034.172	120.1057	1034.2	120.1	94.8	276
19AR109	Spot 443	885.0817	9511.915	4.715194	13.25668	0.63368	1.633279	1.127489	0.160717	0.89276	0.791813	960.7591	7.968715	983.124	7.100888	1034.265	13.93077	1034.3	13.9	92.9	391
19AR109	Spot 396	278.8594	42635.21	1.970214	13.44262	1.024808	1.698847	1.501837	0.167009	1.009716	0.67232	995.6124	9.31503	1008.097	9.599349	1036.192	22.47382	1036.2	22.5	96.1	347
19AR109	Spot 541	790.7108	93635.9	1.440657	13.47535	0.624468	1.754856	0.99511	0.172451	0.748765	0.752444	1025.603	7.099623	1028.954	6.436496	1036.945	13.25514	1036.9	13.3	98.9	480
19AR109	Spot 187	135.1337	21911.62	2.176677	13.40147	1.006595	1.700999	1.364538	0.167053	0.760325	0.557203	995.8522	7.015855	1008.907	8.725809	1038.225	22.897	1038.2	22.9	95.9	163
19AR109	Spot 44	142.7945	18541.1	1.936456	13.38249	1.310055	1.739889	1.6807	0.170801	0.863284	0.513646	1016.523	8.118569	1023.422	10.8374	1039.066	29.13307	1039.1	29.1	97.8	43
19AR109	Spot 34	572.6244	49966.51	5.762047	13.45797	0.744463	1.707598	1.137153	0.167621	0.796719	0.700626	998.9916	7.373106	1011.384	7.282118	1039.184	16.40019	1039.2	16.4	96.1	33
19AR109	Spot 66	773.4942	102107.4	3.961286	13.44018	0.62158	1.725618	0.913926	0.169252	0.665507	0.728185	1007.987	6.210068	1018.12	5.875239	1040.833	12.66098	1040.8	12.7	96.8	64
19AR109	Spot 115	191.9609	32461.06	4.425557	13.43616	0.941518	1.742982	1.328015	0.170915	0.75615	0.569384	1017.15	7.115099	1024.568	8.568664	1041.305	22.05308	1041.3	22.1	97.7	100
19AR109	Spot 341	234.6306	30891.27	2.454934	13.43096	0.811991	1.789942	1.204348	0.175497	0.847928	0.704056	1042.327	8.16066	1041.804	7.845747	1041.568	17.27846	1041.6	17.3	100.1	298
19AR109	Spot 457	161.9764	29326.14	2.494582	13.37688	0.921721	1.772989	1.244092	0.173766	0.794219	0.638392	1032.827	7.579523	1035.615	8.076991	1042.365	19.34167	1042.4	19.3	99.1	402
19AR109	Spot 20	323.7186	53337.94	2.061314	13.43377	0.830066	1.694212	1.282893	0.166037	0.928305	0.723603	990.2375	8.521209	1006.352	8.191545	1042.468	17.8862	1042.5	17.9	95.0	20
19AR109	Spot 167	414.3115	47205.61	5.823882	13.38196	0.72461	1.549264	1.071332	0.151459	0.751769	0.701714	909.1364	6.374545	950.2003	6.611054	1047.462	15.40811	1047.5	15.4	86.8	146
19AR109	Spot 273	350.4711	74364.07	3.919691	13.41904	0.861085	1.768619	1.18437	0.1728	0.805757	0.680325	1027.518	7.653165	1034.014	7.682398	1048.673	17.51804	1048.7	17.5	98.0	242
19AR109	Spot 138	549.2845	89213.21	3.382576	13.40304	0.810512	1.75839	1.178071	0.171792	0.852385	0.723543	1021.975	8.055755	1030.255	7.625512	1048.772	16.41214	1048.8	16.4	97.4	122
19AR109	Spot 394	317.856	3328.363	1.733874	12.70024	0.894789	1.849992	1.386067	0.180721	0.894901	0.64564	1070.913	8.829883	1063.427	9.135913	1048.996	21.35536	1049.0	21.4	102.1	345
19AR109	Spot 103	179.1575	19385.57	3.424629	13.32193	1.13275	1.754754	1.387195	0.171345	0.726252	0.52354	1019.52	6.848466	1028.916	8.972471	1049.844	23.84242	1049.8	23.8	97.1	89
19AR109	Spot 242	161.7403	20457.86	2.50221	13.30559	1.425509	1.727341	1.722505	0.168645	0.901653	0.523454	1004.639	8.387793	1018.761	11.07761	1050.132	29.58249	1050.1	29.6	95.7	214
19AR109	Spot 408	77.02481	10859.03	1.260371	13.15337	2.380789	1.784036	2.719224	0.174041	0.846056	0.311139	1034.34	8.085129	1039.653	17.69488	1051.733	52.09363	1051.7	52.1	98.3	359
19AR109	Spot 171	235.5519	39973.63	2.060597	13.34539	0.793734	1.768264	1.063322	0.172476	0.635978	0.598105	1025.741	6.030958	1033.884	6.896692	1052.043	17.18941	1052.0	17.2	97.5	150
19AR109	Spot 366	255.7847	40116.93	2.655162	13.34933	0.789828	1.7853	1.086849	0.17412	0.7324	0.673875	1034.769	7.001668	1040.113	7.073676	1052.259	16.19843	1052.3	16.2	98.3	318
19AR109	Spot 386	317.9762	11409	2.371016	13.23113	0.843232	1.761969	1.263887	0.171834	0.838919	0.663761	1022.207	7.930147	1031.572	8.187041	1052.378	19.06572	1052.4	19.1	97.1	337
19AR109	Spot 153	148.2632	15191.15	2.658692	13.22161	1.117507	1.730782	1.610902	0.168751	0.895203	0.555715	1005.224	8.332281	1020.042	10.36739	1052.875	26.98328	1052.9	27.0	95.5	133
19AR109	Spot 516	433.5703	72254.8	3.23921	13.33931	0.616444	1.84476	0.932143	0.179719	0.683858	0.733641	1065.439	6.715828	1061.561	6.137786	1054.494	12.77693	1054.5	12.8	101.0	456
19AR109	Spot 64	162.0404	18728.3	1.854008	13.22035	1.422119	1.832888	1.708911	0.178289	0.611131	0.357614	1057.621	5.961083	1057.315	11.22726	1057.58	32.13438	1057.6	32.1	100.0	62
19AR109	Spot 556	962.3426	149560.6	19.45734	13.30295	1.073966	1.811433	1.879925	0.175737	1.541791	0.820134	1043.645	14.85587	1049.596	12.29946	1062.894	21.65689	1062.9	21.7	98.2	491
19AR109	Spot 347	135.7172	6227.167	1.040343	12.87842	1.370089	1.881489	1.998127	0.181351	0.796982	0.398864	1074.351	7.886926	1074.587	13.24837	1075.959	36.78958	1076.0	36.8	99.9	304
19AR109	Spot 594	143.4002	22395.3	2.11528	13.10305	1.313783	1.867116	1.701438	0.17978	0.880134	0.517288	1065.772	8.645842	1069.51	11.25097	1077.995	29.21718	1078.0	29.2	98.9	527
19AR109	Spot 25	1084.306	165719.6	5.065992	13.22618	0.739306	1.815273	1.127297	0.174736	0.850317	0.754298	1038.151	8.153437	1050.982	7.380695	1078.636	14.85297	1078.6	14.9	96.2	25
19AR109	Spot 448	276.3618	30668.46	3.045825	13.09768	0.912739	1.888584	1.174441	0.181156	0.701033	0.596908	1073.285	6.931089	1077.084	7.79688	1085.665	18.88323	1085.7	18.9	98.9	395
19AR109	Spot 162	386.8211	43906.08	1.714519	13.0871	0.731394	1.891623	1.038231	0.180978	0.72617	0.69943	1072.317	7.17367	1078.152	6.896416	1090.82	14.84643	1090.8	14.8	98.3	141
19AR109	Spot 417	264.2059	45049.29	6.724252	13.07025	0.845195	1.915921	1.153621	0.183284	0.772763	0.669859	1084.893	7.716161	1086.648	7.696677	1091.022	17.13944	1091.0	17.1	99.4	368
19AR109	Spot 71	276.6478	50849.05	2.841128	13.07827	0.976153	1.758846	1.304067	0.168128	0.818877	0.627941	1001.788	7.597778	1030.424	8.441903	1092.574	20.30706	1092.6	20.3	91.7	69
19AR109	Spot 404	167.8085	38166.47	2.733248	13.04405	0.966764	1.898115	1.167878	0.181373	0.62802	0.537744	1074.474	6.21553	1080.429	7.766811	1093.314	19.69922	1093.3	19.7	98.3	355

19AR109	Spot 302	347.8722	80082.52	13.56491	13.20933	0.712568	1.861667	0.976123	0.177835	0.64261	0.658329	1055.134	6.254568	1067.578	6.447971	1093.984	14.70062	1094.0	14.7	96.4	267
19AR109	Spot 493	394.8378	59284.12	14.0886	13.04601	0.768665	1.921995	1.163657	0.183324	0.846903	0.727794	1085.111	8.458017	1088.761	7.772058	1096.961	15.95819	1097.0	16.0	98.9	434
19AR109	Spot 70	205.7091	37283.7	2.624556	13.01991	0.860336	1.865477	1.148973	0.177757	0.686136	0.597173	1054.712	6.675757	1068.929	7.595228	1098.943	18.43956	1098.9	18.4	96.0	68
19AR109	Spot 203	534.3854	106910.3	5.068671	13.05746	0.568172	1.93367	0.874684	0.183916	0.662628	0.757563	1088.33	6.635685	1092.81	5.854057	1102.636	11.41766	1102.6	11.4	98.7	179
19AR109	Spot 55	255.6251	42818.5	0.69622	13.00596	0.933291	1.889425	1.246384	0.179567	0.806951	0.647434	1064.606	7.918968	1077.38	8.275792	1104.183	19.00483	1104.2	19.0	96.4	54
19AR109	Spot 469	106.7646	16481.58	2.334307	12.86732	1.256697	1.726437	1.4545	0.163659	0.612642	0.421204	977.0802	5.554431	1018.425	9.352145	1109.255	26.35415	1109.3	26.4	88.1	412
19AR109	Spot 53	142.8905	24962.19	1.376118	12.83891	1.530027	1.956499	1.842154	0.184103	0.790343	0.429032	1089.353	7.921479	1100.681	12.37882	1123.995	33.1716	1124.0	33.2	96.9	52
19AR109	Spot 110	70.23825	10145.99	0.947697	12.738	1.882314	1.97242	2.126411	0.18544	0.790684	0.37184	1096.627	7.973447	1106.135	14.32832	1125.753	39.32731	1125.8	39.3	97.4	95
19AR109	Spot 356	115.9085	16743.34	2.928151	12.74569	1.419948	2.001641	1.682711	0.187718	0.762131	0.452919	1109.001	7.764983	1116.068	11.39423	1130.704	29.87793	1130.7	29.9	98.1	311
19AR109	Spot 74	186.8031	26560.48	2.936201	12.78432	0.893917	1.831559	1.266326	0.171702	0.873816	0.69004	1021.482	8.254612	1056.838	8.317265	1131.483	18.25835	1131.5	18.3	90.3	72
19AR109	Spot 549	206.7906	21269.66	2.134539	12.75734	0.945182	2.04076	1.344844	0.191265	0.757144	0.562998	1128.226	7.836546	1129.215	9.16481	1131.978	22.13002	1132.0	22.1	99.7	486
19AR109	Spot 342	392.5049	28654.35	3.526702	12.82731	0.80003	1.932807	1.234581	0.181015	0.850162	0.688624	1072.519	8.400016	1092.511	8.2616	1133.431	17.82913	1133.4	17.8	94.6	299
19AR109	Spot 535	86.58145	17281.68	2.183616	12.66242	1.45705	2.139362	1.70896	0.199403	0.802184	0.469399	1172.11	8.597217	1161.618	11.82563	1142.986	30.01668	1143.0	30.0	102.5	474
19AR109	Spot 274	112.2861	18263.25	3.262106	12.68692	1.519115	2.048974	1.715259	0.190376	0.707336	0.412378	1123.413	7.292433	1131.954	11.70473	1149.244	31.03529	1149.2	31.0	97.8	243
19AR109	Spot 365	98.05602	7179.767	2.506361	12.42913	1.588816	2.017691	2.463679	0.186928	0.95459	0.387465	1104.715	9.691392	1121.482	16.72758	1154.958	45.0922	1155.0	45.1	95.6	317
19AR109	Spot 472	96.89237	16528.38	2.165829	12.47474	1.270111	2.245536	1.631992	0.206288	0.893749	0.547643	1209.013	9.852752	1195.391	11.46568	1171.724	27.03135	1171.7	27.0	103.2	415
19AR109	Spot 479	133.5298	7329.825	3.007702	12.2823	1.347056	1.724034	2.46871	0.158005	1.085285	0.439616	945.6789	9.546012	1017.529	15.86604	1176.421	43.87211	1176.4	43.9	80.4	422
19AR109	Spot 50	135.4961	22247.12	2.250591	12.50099	1.184341	2.22944	1.410654	0.204155	0.653252	0.463084	1197.603	7.139651	1190.342	9.888553	1178.059	24.73056	1178.1	24.7	101.7	49
19AR109	Spot 96	507.5781	60852.81	3.990259	12.53118	0.731119	2.006026	0.985733	0.183645	0.654844	0.664322	1086.859	6.549603	1117.55	6.67943	1178.609	14.58854	1178.6	14.6	92.2	83
19AR109	Spot 517	84.09951	18387.67	17.79107	12.43065	1.644477	2.138326	1.942236	0.195484	1.00013	0.514938	1151.017	10.54249	1161.283	13.43795	1181.364	32.91232	1181.4	32.9	97.4	457
19AR109	Spot 548	278.287	42277.19	2.359968	12.43597	0.830447	2.248834	1.131445	0.204708	0.758775	0.670624	1200.56	8.311582	1196.422	7.952473	1189.831	16.5707	1189.8	16.6	100.9	485
19AR109	Spot 220	478.9453	21406.11	0.662038	12.28113	0.584363	2.160591	1.11654	0.195127	0.937252	0.839425	1149.089	9.864568	1168.461	7.750269	1205.378	11.95224	1205.4	12.0	95.3	195
19AR109	Spot 51	63.73314	13642.03	1.570024	12.14906	2.042216	2.283139	2.258503	0.204136	0.771697	0.341685	1197.498	8.433514	1207.087	15.94885	1225.12	41.69743	1225.1	41.7	97.7	50
19AR109	Spot 130	173.4087	34175.45	4.692269	12.20957	0.870731	2.324774	1.179844	0.207429	0.687111	0.582374	1215.104	7.609441	1219.883	8.376894	1229.181	18.83793	1229.2	18.8	98.9	114
19AR109	Spot 36	59.09002	9029.13	77.82924	11.92131	2.015476	2.373907	2.356401	0.209521	1.134646	0.481517	1226.264	12.67048	1234.778	16.83641	1250.512	40.41424	1250.5	40.4	98.1	35
19AR109	Spot 305	218.3427	38299.92	7.804813	11.78829	0.730056	2.394976	1.144232	0.205159	0.862551	0.753826	1202.974	9.465624	1241.099	8.1963	1308.73	14.59344	1308.7	14.6	91.9	270
19AR109	Spot 482	183.6746	32193.84	1.930468	11.67415	0.704943	2.578483	0.912749	0.220416	0.564446	0.618402	1284.073	6.571671	1294.551	6.678096	1312.796	13.91475	1312.8	13.9	97.8	424
19AR109	Spot 192	261.2733	67228.41	2.645793	11.70498	0.655977	2.627409	0.990573	0.223764	0.710701	0.717464	1301.732	8.377172	1308.34	7.285425	1320.015	13.37227	1320.0	13.4	98.6	168
19AR109	Spot 60	320.2644	64471.51	2.911919	11.60638	0.820153	2.659794	1.132235	0.224958	0.753984	0.665925	1308.018	8.926065	1317.365	8.355411	1333.431	16.33917	1333.4	16.3	98.1	58
19AR109	Spot 216	279.8227	46905.67	2.880141	11.51788	0.77018	2.339661	1.303314	0.19713	1.050768	0.806228	1159.884	11.15416	1224.419	9.271319	1340.788	14.89923	1340.8	14.9	86.5	191
19AR109	Spot 333	133.2704	43700.38	2.649323	11.33463	0.884629	2.641835	1.212093	0.21833	0.819829	0.676375	1273.043	9.470866	1312.37	8.928172	1378.034	17.16257	1378.0	17.2	92.4	290
19AR109	Spot 488	155.5192	37377	1.136739	11.27901	1.002033	2.831999	1.198287	0.233689	0.652912	0.544872	1353.803	7.972704	1364.052	8.992278	1380.964	19.30803	1381.0	19.3	98.0	429
19AR109	Spot 430	243.3706	57368	2.355836	11.16489	0.675599	2.963722	0.970703	0.241505	0.668136	0.688301	1394.514	8.378389	1398.369	7.369845	1405.066	13.48589	1405.1	13.5	99.2	380
19AR109	Spot 269	162.7901	39672.29	3.94842	10.89331	0.725781	3.15691	1.010778	0.250843	0.667628	0.660509	1442.822	8.630826	1446.689	7.794455	1453.187	14.43982	1453.2	14.4	99.3	238
19AR109	Spot 144	601.5421	162572.2	4.3231	10.86511	0.658144	3.084688	1.007828	0.244162	0.761003	0.755092	1408.299	9.627338	1428.893	7.728174	1460.512	12.55969	1460.5	12.6	96.4	128
19AR109	Spot 410	190.1855	30556.72	1.755341	10.69105	0.814931	3.074775	1.202619	0.240781	0.831264	0.691212	1390.755	10.39883	1426.426	9.21467	1480.874	16.47462	1480.9	16.5	93.9	361
19AR109	Spot 48	245.7185	44698.07	2.399525	10.69209	0.693486	2.644825	0.937302	0.206159	0.613536	0.654577	1208.322	6.760145	1313.203	6.906155	1489.613	13.4167	1489.6	13.4	81.1	47
19AR109	Spot 441	344.892	77931.31	5.547222	10.6137	0.687615	3.028738	1.081559	0.234327	0.830023	0.767432	1357.135	10.15781	1414.889	8.256239	1503.748	13.10461	1503.7	13.1	90.3	389
19AR109	Spot 318	565.9099	35971.95	3.946976	9.947577	0.835186	1.090362	1.26744	0.078907	0.90019	0.710243	489.5944	4.244076	748.6797	6.712939	1628.963	16.58584	1629.0	16.6	30.1	278
19AR109	Spot 307	222.6488	38166.16	2.787254	9.589907	1.115184	3.514842	1.876652	0.244024	1.497322	0.797869	1407.584	18.93385	1530.558	14.83569	1705.702	20.82606	1705.7	20.8	82.5	272
19AR109	Spot 160	446.5937	120565.1	2.374022	9.429789	0.677072	4.16867	1.859155	0.286327	1.730983	0.931059	1623.148	24.83843	1667.884	15.22638	1725.441	12.45744	1725.4	12.5	94.1	139
19AR109	Spot 5	147.8126	29382.13	2.900632	9.260013	0.57152	4.53502	0.929841	0.3067	0.680797	0.732165	1724.449	10.30088	1737.417	7.735831	1753.844	11.58975	1753.8	11.6	98.3	5
19AR109	Spot 215	121.4538	27918.62	1.649497	9.081333	0.809485	4.667464	1.075644	0.310372	0.664993	0.618227	1742.536	10.15367	1761.427	8.995022	1784.683	15.4106	1784.7	15.4	97.6	190
19AR109	Spot 131	44.19457	16071.2	1.527371	8.834836	1.951303	5.133964	2.08484	0.332263	0.718505	0.344633	1849.342	11.55156	1841.744	17.71978	1833.942	35.46217	1833.9	35.5	100.8	115
19AR109	Spot 296	689.5948	135604.3	6.033826	8.904054	0.586872	4.455289	0.982815	0.288073	0.785214	0.798943	1631.891	11.32057	1722.684	8.150218	1835.623	10.70795	1835.6	10.7	88.9	261
19AR109	Spot 6	465.7704	16610.62	1.68156	8.673344	0.536307	5.018909	0.992998	0.318802	0.783114	0.788636	1783.876	12.20352	1822.517	8.407751	1867.737	11.01807	1867.7	11.0	95.5	6
19AR109	Spot 19	216.1686	78520.77	1.021233	8.64168	0.791951	5.294269	1.220334	0.333064	0.924365	0.757469	1853.217	14.8881	1867.939	10.4228	1885.128	14.34571	1885.1	14.3	98.3	19
19AR109	Spot 531	1581.818	269460.5	2.591382	8.612956	0.662828	5.126054	0.992875	0.322368	0.738434	0.743732	1801.281	11.60458	1840.433	8.436008	1885.766	11.9495	1885.8	11.9	95.5	470
19AR109	Spot 513	348.9819	91636.98	1.968243	8.576085	0.553232	5.297413	0.862556	0.331344	0.660124	0.765312	1844.893	10.59091	1868.446	7.367605	1895.511	9.983698	1895.5	10.0	97.3	453
19AR109	Spot 56	261.5682	71136	1.586585	8.479011	0.731731	4.966481	1.163961	0.306881	0.904507	0.777094	1725.34	13.69194	1813.634	9.838127	1917.408	13.1398	1917.4	13.1	90.0	55
19AR109	Spot 326	477.851	144275.2	2.531643	8.304922	0.596577	5.745685	0.911082	0.347693	0.686948	0.753991	1923.573	11.42477	1938.268	7.879743	1954.773	10.68675	1954.8	10.7	98.4	284
19AR109	Spot 470	206.4638	28627.95	2.135424	8.251245	0.62208	5.03181	1.078568	0.303664	0.862075	0.799278	1709.455	12.94471	1824.691	9.1362	1959.643	11.56785	1959.6	11.6	87.2	413
19AR109	Spot 252	475.5581	121993.6	15.25123	8.267015	0.647974	5.540551	0.989079	0.333186	0.74583	0.754065	1853.804	12.01584	1906.911	8.507648	1965.951	11.58518	1966.0	11.6	94.3	224
19AR109	Spot 243	254.8502	62495.87	1.777713	8.214341	0.764956	4.808216	1.121807	0.28764	0.804619	0.717253	1629.725	11.58682	1786.336	9.429788	1975.26	13.92568	1975.3	13.9	82.5	215
19AR109	Spot 289	286.2179	49079.65	2.976623	8.216569	0.45392	5.887385	0.799543	0.3521	0.656449	0.821031	1944.617	11.01988	1959.376	6.939795	1975.762	8.132161	1975.8	8.1	98.4	255
19AR109	Spot 230	211.4674	84009.89	0.871618	8.183887	0.6226	5.944662	0.943187	0.35447	0.70202	0.744306	1955.91	11.84346	1967.785	8.198102	1981.054	11.21548	1981.1	11.2	98.7	204
19AR109	Spot 169	161.3325	40013.89	2.062382	8.076099	0.629291	5.893653	0.916474	0.347168	0.655485	0.715224	1921.063	10.88929	1960.3	7.955991	2002.739	11.37386	2002.7	11.4	95.9	148
19AR109	Spot 572	231.4864	89173.85	3.968495	8.016966	0.63837	6.014371	0.933903	0.351237	0.677622	0.72558	1940.504	11.35468	1977.927	8.130973	2018.042	11.39367	2018.0	11.4	96.2	505
19AR109	Spot 221	110.8467	30076.36	0.67308	7.923045	0.660885	6.188429	0.952128	0.358898	0.680575	0.714794	1976.947	11.58721	2002.815	8.323027	2030.362	11.78615	2030.4	11.8	97.4	196
19AR109	Spot 87	307.6075	66117.11	2.871561	7.886419	0.725654	5.738783	1.15299	0.330211	0.88886	0.770918	1839.402	14.22404	1937.228	9.970292	2044.284	12.97866	2044.3	13.0	90.0	76
19AR109	Spot 344	702.6271	23525.47	4.501147	7.86817	0.751967	6.354216	1.131968	0.364296	0.844247	0.745823	2002.506	14.53231	2025.967	9.931237	2050.706	13.3188	2050.7	13.3	97.6	301
19AR109	Spot 462	293.3624	78594.93	2.372795	7.648905	0.695689	6.626398	1.197857	0.370077	0.972198	0.811614	2029.762	16.92859	2062.868	10.56839	2096.856	12.29554	2096.9	12.3	96.8	406
19AR109	Spot 225	31.07298	10254.13	1.223805	6.686279	1.165421	8.424733	1.584216	0.41395	0.848284	0.53546	2232.957	16.00938	2277.847	14.38005	2319.117	22.94476	2319.1	22.9	96.3	200
19AR109	Spot 125	144.3951	31368.54	1.05469	6.330543	0.692048	8.117158	1.043712	0.374364	0.777253	0.744701	2049.902	13.64814	2244.157	9.43556	2427.096	11.81083	2427.1	11.8	84.5	109
19AR109	Spot 562	243.6692	88278.7	21.26424	5.761778	0.696927	10.65348	1.109992	0.447786	0.861645	0.776262	2385.401	17.17962	2493.38	10.30388	2583.266	11.68447	2583.3	11.7	92.3	496
19AR109	Spot 143	424.8154	121421.9	4.631972	5.571572	0.647283	10.51911	1.039831	0.426629	0.813635	0.782469	2290.502	15.68512	2481.603	9.641971	2642.707	10.74716	2642.7	10.7	86.7	127
19AR109	Spot 528	282.1477	10851.49	1.761569	5.525133	0.596813	10.44544	0.867767	0.423519	0.619066	0.713401	2276.437	11.87313	2475.089	8.041487	2643.183	10.09255	2643.2	10.1	86.1	467
19AR109	Spot 292	227.463	95724.47	2.71687	5.543341	0.468813	12.28425	0.791264	0.49489	0.635819	0.803549	2591.798	13.56913	2626.368	7.429692	2653.83	7.811704	2653.8	7.8	97.7	257
19AR109	Spot 165	138.835	79930.09	1.113662	5.239791	0.624572	12.64251	0.961884	0.482377	0.730536	0.759484	2537.61	15.32457	2653.389	9.051133	2743.589	10.28798	2743.6	10.3	92.5	144
19AR109	Spot 533	158.0021	96695.7	2.106372	5.159744	0.674396	13.36464	1.023782	0.502957	0.769865	0.751981	2626.49	16.60801	2705.762	9.671924	2766.211	11.07612	2766.2	11.1	94.9	472
19AR109	Spot 124	23.50776	10018.81	1.919451	5.00916	1.163226	14.38858	1.576647	0.526846	0.966675	0.613121	2728.148	21.50247	2775.677	14.96977	2811.113	20.35971	2811.1	20.4	97.0	108
19AR109	Spot 57	95.78073	57233.31	2.218205	4.973991	0.725961	14.38898	1.09843	0.521741	0.822538	0.748831	2706.559	18.17983	2775.704	10.42888	2827.062	11.87882	2827.1	11.9	95.7	56

19AR110 -26.94385 -65.76501 2870 28.7 0.846

19AR110	Spot 447	1120.821	81.80401	2.179746	4.733502	40.73565	0.000968	469.1279	0.000275	8.179314	0.017435	1.773135	0.14501	0.98244	4.606703	NA	NA	1.8	0.1		391
19AR110	Spot 186	1308.101	229.4823	2.783315	5.139577	47.23013	0.005849	54.22623	0.000309	7.24876	0.133676	1.988934	0.144151	5.921696	3.201778	2196.333	1021.122	2.0	0.1	0.1	160
19AR110	Spot 418	2363.796	225.5528	0.748964	7.430198	13.79075	0.00305	37.68815	0.000313	4.751371	0.126071	2.017364	0.095837	3.091985	1.16354	948.7864	792.6771	2.0	0.1	0.2	366
19AR110	Spot 209	1478.872	191.7161	1.585484	4.680224	10.52781	0.006579	25.67859	0.000327	5.670007	0.220807	2.108589	0.119538	6.658709	1.70427	2298.693	437.9423	2.1	0.1	0.1	179
19AR110	Spot 519	2049.344	263.6445	3.643116	7.462913	21.23429	0.003656	55.25318	0.000331	5.115396	0.092581	2.134057	0.109147	3.705668	2.043771	1199.999	1181.906	2.1	0.1	0.2	454
19AR110	Spot 520	927.6769	256.5045	0.671628	4.396307	36.3652	0.008451	40.54804	0.000342	9.395234	0.231706	2.205717	0.207197	8.544486	3.450098	2645.191	685.6961	2.2	0.2	0.1	455
19AR110	Spot 441	2677.172	585.3001	4.158582	8.164984	28.2779	0.004718	35.84862	0.000346	2.782818	0.077627	2.233182	0.062135	4.77981	1.709471	1601.805	690.0186	2.2	0.1	0.1	385
19AR110	Spot 216	3226.496	733.1188	4.873337	8.226622	12.32021	0.00492	19.06246	0.000348	2.800904	0.146933	2.240636	0.062747	4.983371	0.947626	1673.245	351.7493	2.2	0.1	0.1	186
19AR110	Spot 531	1666.699	434.0488	5.545217	4.936592	11.06028	0.008547	13.89252	0.000357	7.504967	0.540216	2.303724	0.172863	8.641602	1.195442	2591.731	195.7669	2.3	0.2	0.1	465
19AR110	Spot 268	1990.956	366.0446	6.306157	6.962377	25.19084	0.005318	29.54154	0.000362	5.60144	0.189612	2.33165	0.130582	5.385217	1.586666	1743.037	543.4872	2.3	0.1	0.1	234
19AR110	Spot 278	3004.386	858.8128	3.366763	8.928118	17.14048	0.004789	21.07611	0.000362	3.844315	0.182402	2.332291	0.089644	4.8512	1.020006	1548.32	393.5853	2.3	0.1	0.2	243
19AR110	Spot 417	1212.829	274.8311	5.274984	7.159175	17.16162	0.004434	44.36364	0.000365	8.253638	0.186045	2.350753	0.193987	4.492635	1.988696	1387.196	881.1787	2.4	0.2	0.2	365
19AR110	Spot 34	2662.305	494.077	4.872743	10.51686	17.24516	0.003317	27.46851	0.000366	3.615887	0.131638	2.35595	0.085173	3.362828	0.922191	801.5859	580.6063	2.4	0.1	0.3	32
19AR110	Spot 312	2999.127	764.1942	4.154738	7.985918	10.62568	0.005428	16.18731	0.000368	3.422453	0.211428	2.375052	0.08127	5.496519	0.887335	1746.821	291.631	2.4	0.1	0.1	271
19AR110	Spot 21	2528.588	541.2209	4.462476	8.311763	15.61166	0.004881	23.05787	0.000375	3.327718	0.14432	2.4157	0.080373	4.944491	1.137323	1518.108	436.1221	2.4	0.1	0.2	20
19AR110	Spot 523	1461.297	398.5523	3.936195	6.072575	29.42489	0.006793	36.66836	0.000376	6.744074	0.183921	2.421141	0.163253	6.873711	2.511971	2114.083	655.5776	2.4	0.2	0.1	458
19AR110	Spot 254	1706.905	729.6658	3.914163	8.43305	14.73488	0.005194	23.65202	0.000378	4.651299	0.196655	2.435979	0.113283	5.260289	1.240948	1618.736	437.7296	2.4	0.1	0.2	221
19AR110	Spot 385	2200.801	514.1571	1.327276	9.047673	13.26295	0.004343	21.26834	0.00038	4.689861	0.220509	2.451347	0.114943	4.400606	0.933911	1265.892	409.4463	2.5	0.1	0.2	336
19AR110	Spot 57	1686.428	471.6379	4.331206	5.382301	13.77256	0.008753	16.40557	0.000399	6.322686	0.385399	2.574375	0.162737	8.84942	1.44549	2445.153	257.8515	2.6	0.2	0.1	51
19AR110	Spot 501	1111.272	67.4086	0.659083	5.620431	8.387437	-0.00426	102.9606	0.000409	7.943522	0.077151	2.639223	0.209604	NA	-4.4683	NA	NA	2.6	0.2		439
19AR110	Spot 19	2660.334	733.4778	3.981425	10.18918	16.44375	0.004459	19.38896	0.000411	2.86649	0.147841	2.650559	0.075962	4.517979	0.874043	1164.059	383.452	2.7	0.1	0.2	18
19AR110	Spot 472	1840.327	637.6128	3.399339	10.65399	18.69818	0.004067	24.66417	0.000414	3.810137	0.154481	2.665475	0.101537	4.121166	1.014392	967.5687	504.5496	2.7	0.1	0.3	414
19AR110	Spot 142	1665.691	450.0475	2.780632	9.129517	15.22309	0.005185	33.72092	0.000483	3.719408	0.1103	3.110624	0.115669	5.251193	1.766181	1145.568	685.2497	3.1	0.1	0.3	124
19AR110	Spot 457	1918.428	581.6225	4.775289	9.191993	12.7177	0.00598	29.18902	0.000513	2.427518	0.083165	3.3073	0.080265	6.054154	1.761892	1305.339	577.1796	3.3	0.1	0.3	401
19AR110	Spot 383	1825.311	607.6638	0.807492	10.75979	12.30082	0.006193	26.29336	0.00065	3.556869	0.135276	4.187127	0.148882	6.268962	1.643245	903.5001	545.8788	4.2	0.1	0.5	334
19AR110	Spot 465	2355.262	1222.837	2.228134	12.94021	9.575569	0.006511	16.09058	0.000722	2.249318	0.139791	4.651149	0.104581	6.590225	1.056972	789.105	336.3901	4.7	0.1	0.6	409
19AR110	Spot 256	730.3342	335.9149	1.126002	6.028788	37.59636	0.014604	39.31526	0.000839	6.079361	0.154631	5.404743	0.328436	14.72121	5.74599	2047.531	716.217	5.4	0.3	0.3	223
19AR110	Spot 396	473.7806	292.1955	3.453088	7.196985	19.73445	0.011957	28.45114	0.000955	7.096514	0.249428	6.15029	0.436248	12.06852	3.41332	1444.257	535.1998	6.2	0.4	0.4	345
19AR110	Spot 154	570.4242	288.209	1.672949	7.349013	31.81753	0.011575	45.9666	0.000959	6.754856	0.146951	6.178926	0.417178	11.68538	5.34063	1373.244	925.5198	6.2	0.4	0.4	133
19AR110	Spot 288	580.9517	571.4811	1.182909	7.264429	26.27213	0.01708	30.78321	0.001088	5.692655	0.184927	7.011215	0.398907	17.19619	5.249012	1862.273	559.9251	7.0	0.4	0.4	251
19AR110	Spot 566	354.4659	275.066	1.179066	9.044863	27.58771	0.010096	41.65857	0.001275	7.766821	0.18644	8.213064	0.637488	10.19975	4.227826	509.2397	NA	8.2	0.6	1.6	494
19AR110	Spot 549	269.9059	231.9661	1.069511	7.495238	33.57546	0.015056	81.1957	0.00153	9.940753	0.12243	9.854475	0.978861	15.17381	12.22947	969.1704	NA	9.9	1.0	1.0	480
19AR110	Spot 152	239.936	247.4739	1.285662	5.090582	24.63801	0.031283	28.82468	0.001571	9.571951	0.332075	10.12223	0.968134	31.27731	8.878369	2281.131	477.8434	10.1	1.0	0.4	132
19AR110	Spot 329	241.585	264.757	0.777824	5.378278	24.80617	0.031385	35.20239	0.001672	9.374324	0.266298	10.7686	1.008641	31.37758	10.87715	2179.514	610.1186	10.8	1.0	0.5	286
19AR110	Spot 199	387.1117	384.91	1.437874	8.130673	21.50411	0.020452	38.59566	0.001717	7.993444	0.207107	11.06046	0.883353	20.55664	7.854352	1347.451	756.8451	11.1	0.9	0.8	172
19AR110	Spot 255	428.6616	331.7514	1.337228	7.114575	30.28664	0.023725	31.79003	0.001739	3.909338	0.122974	11.2033	0.437594	23.80815	7.480709	1604.682	604.0254	11.2	0.4	0.7	222
19AR110	Spot 72	398.3122	384.7502	1.02871	7.013192	19.85737	0.026004	24.59163	0.001764	4.154944	0.168958	11.36093	0.471625	26.06671	6.328728	1748.362	450.7219	11.4	0.5	0.6	64
19AR110	Spot 115	190.0238	217.9833	1.745949	7.30419	9.429287	0.017435	47.84778	0.001785	5.829828	0.121841	11.49376	0.669469	17.55092	8.325752	954.2495	1030.831	11.5	0.7	1.2	100
19AR110	Spot 293	610.4684	782.9383	0.884521	8.723036	17.24366	0.024299	22.04445	0.001823	4.125875	0.187162	11.74273	0.48405	24.37768	5.309975	1561.397	411.0186	11.7	0.5	0.8	255
19AR110	Spot 490	862.955	1004.142	1.079114	13.38471	11.63448	0.015145	20.00645	0.001825	1.873389	0.093639	11.75342	0.219987	15.2627	3.030697	611.3421	434.3776	11.8	0.2	1.9	429
19AR110	Spot 536	210.3161	303.1466	1.515717	4.631138	17.54816	0.043982	23.16075	0.001828	8.032826	0.346829	11.77309	0.944849	43.70419	9.907783	2602.017	366.8674	11.8	0.9	0.5	470
19AR110	Spot 88	381.1101	550.2453	1.132795	10.4965	12.18857	0.01769	20.43105	0.001852	3.914381	0.19159	11.9309	0.466589	17.80491	3.606038	907.2873	417.0782	11.9	0.5	1.3	79
19AR110	Spot 65	350.6019	274.4438	1.667582	7.218082	13.97646	0.022328	52.35899	0.001855	6.261889	0.119595	11.94793	0.747473	22.42194	11.61173	1367.746	1080.391	11.9	0.7	0.9	57
19AR110	Spot 551	792.1893	861.4065	1.060952	12.94099	11.77535	0.017042	13.8572	0.002055	2.441336	0.176178	13.2336	0.322745	17.15797	2.357643	609.7358	296.0953	13.2	0.3	2.2	482
19AR110	Spot 525	715.5403	725.5019	1.718396	10.80958	13.68838	0.021227	15.62112	0.002122	1.753005	0.11222	13.66233	0.239248	21.32795	3.296929	1002.661	316.9103	13.7	0.2	1.4	459
19AR110	Spot 66	202.811	338.4813	0.797481	6.886779	30.33162	0.030707	41.91946	0.00213	8.431505	0.201136	13.71595	1.155232	30.70977	12.6813	1707.346	791.8986	13.7	1.2	0.8	58
19AR110	Spot 107	117.6211	148.5589	1.807335	4.690215	25.05703	0.043842	56.01048	0.002592	7.590947	0.135527	16.68861	1.265185	43.56819	23.89103	1996.123	1084.378	16.7	1.3	0.8	92
19AR110	Spot 593	405.4038	654.5444	2.153098	10.64306	12.29865	0.044386	22.44966	0.004483	2.297427	0.102337	28.8353	0.660991	44.09739	9.688121	981.39	460.3177	28.8	0.7	2.9	520
19AR110	Spot 453	894.2905	2079.002	5.657373	16.40205	1.144354	0.247659	5.392544	0.033322	2.64877	0.491191	211.3077	5.506316	224.6725	10.86921	367.9842	105.8807	211.3	5.5	57.4	397
19AR110	Spot 572	58.39098	1509.264	0.668341	13.42116	8.563218	0.338326	9.578783	0.03781	2.207433	0.23045	239.2417	5.184303	295.9028	24.59229	771.932	196.5632	239.2	5.2	31.0	499
19AR110	Spot 515	124.6445	2182.219	1.30226	17.28442	6.732694	0.271463	8.501826	0.038577	1.908127	0.224437	244.0058	4.568925	243.8627	18.43303	243.5109	191.1655	244.0	4.6	100.2	450
19AR110	Spot 188	97.34569	2241.686	0.850921	16.73787	3.703065	0.306837	6.987712	0.041869	1.126917	0.161271	264.4074	2.919373	271.7266	16.66059	336.2664	156.4288	264.4	2.9	78.6	162
19AR110	Spot 141	92.92287	2431.154	1.749564	14.00252	7.940395	0.380619	11.37812	0.042178	1.19259	0.104814	266.3218	3.111415	327.4931	31.86098	789.6739	238.19	266.3	3.1	33.7	123
19AR110	Spot 224	115.1708	2661.388	1.093715	16.74622	5.674527	0.324556	6.571846	0.043505	1.023242	0.155701	274.5214	2.750046	285.4013	16.35213	376.4483	146.1694	274.5	2.8	72.9	193
19AR110	Spot 241	195.842	5101.924	1.345094	17.25941	2.801569	0.33615	3.417176	0.044408	0.845254	0.247355	280.0978	2.316841	294.2499	8.729421	409.0947	74.10678	280.1	2.3	68.5	209
19AR110	Spot 575	258.5579	5860.13	1.897722	18.7513	2.341588	0.319807	3.345892	0.045677	0.841507	0.251505	287.925	2.369598	281.7542	8.232449	231.8286	74.79237	287.9	2.4	124.2	502
19AR110	Spot 389	668.2965	25070.26	9.890186	18.88716	1.224711	0.378079	1.486255	0.052554	0.783635	0.527255	330.1819	2.522275	325.6234	4.140318	294.1498	28.85255	330.2	2.5	112.2	339
19AR110	Spot 310	145.4228	8011.001	1.142883	16.92884	2.936837	0.565939	3.277352	0.072088	0.805609	0.245811	448.7204	3.491989	455.3845	12.02729	490.1393	70.10345	448.7	3.5	91.5	269
19AR110	Spot 137	64.26744	2882.422	1.045003	16.88689	4.533742	0.541168	7.374424	0.072411	0.872257	0.118281	450.6622	3.796676	439.1941	26.29886	380.477	164.84	450.7	3.8	118.4	120
19AR110	Spot 244	240.8761	6875.59	0.459224	16.99233	2.298667	0.577233	2.70683	0.074051	0.813445	0.300516	460.5184	3.61539	462.6814	10.05912	474.4034	57.13189	460.5	3.6	97.1	211
19AR110	Spot 239	76.00754	4181.974	1.021091	15.13587	5.855781	0.641062	6.981309	0.074496	1.031984	0.147821	463.187	4.612331	502.9635	27.69807	689.148	147.4462	463.2	4.6	67.2	207
19AR110	Spot 302	323.1655	16566.8	17.00165	17.75404	1.350391	0.568951	1.984604	0.074932	0.831892	0.419173	465.8005	3.738275	457.3361	7.307636	415.9774	40.27611	465.8	3.7	112.0	262
19AR110	Spot 414	196.7132	793.0727	1.763583	15.29352	1.764914	0.481391	10.30742	0.07502	0.742399	0.072026	466.3292	3.339767	399.0266	34.02284	26.66743	NA	466.3	3.3	1748.7	362
19AR110	Spot 75	81.3468	3048.964	1.715154	16.40129	5.631991	0.579707	9.076599	0.075028	0.848896	0.093526	466.3739	3.819213	464.2733	33.83342	454.8831	200.9232	466.4	3.8	102.5	66
19AR110	Spot 35	468.4799	6839.071	6.001059	17.28233	1.287685	0.576618	2.801102	0.075092	0.627923	0.22417	466.7604	2.827305	462.2858	10.40245	441.0607	60.73809	466.8	2.8	105.8	33
19AR110	Spot 38	541.2394	18041.79	7.255852	17.65189	1.167099	0.579457	1.348277	0.075112	0.602521	0.446882	466.8781	2.713589	464.1126	5.022573	451.3968	26.78469	466.9	2.7	103.4	36
19AR110	Spot 143	247.1684	7655.789	80.19143	17.33506	2.059434	0.577389	2.262654	0.075117	0.725181	0.3205	466.9112	3.266237	462.782	8.409838	443.2877	47.66618	466.9	3.3	105.3	125
19AR110	Spot 133	331.2005	14910.99	9.74656	17.46464	1.604454	0.583265	2.253228	0.075146	0.757536	0.3362	467.0837	3.413183	466.5578	8.428642	464.9129	47.03883	467.1	3.4	100.5	116
19AR110	Spot 261	235.5004	8394.757	6.839037	17.26229	1.45043	0.582615	1.961082	0.075153	0.766409	0.390809	467.1256	3.453459	466.1405	7.330602	462.2351	40.02034	467.1	3.5	101.1	227
19AR110	Spot 69	81.79304	3905.094	1.691071	15.40742	5.898467	0.634329	6.945808	0.075287	0.943141	0.135786	467.9323	4.256891	498.7891	27.37998	643.9591	148.0615	467.9	4.3	72.7	61
19AR110	Spot 458	557.0196	18307.2	2.484653	17.61171	0.949894	0.580722	1.347386	0.075299	0.789965	0.586294	468.0028	3.566046	464.9254	5.026185	450.6997	24.25223	468.0	3.6	103.8	402
19AR110	Spot 380	759.1935	17173.11	10.92657	17.50336	0.770164	0.584139	1.698074	0.075368	0.740315	0.435974	468.4156	3.344761	467.1181	6.357923	461.6902	33.88908	468.4	3.3	101.5	331
19AR110	Spot 343	112.7855	4604.981	1.405086	16.41524	3.197327	0.601143	5.646999	0.075389	0.579419	0.102606	468.5434	2.618516	477.9589	21.53082	524.3278	123.2707	468.5	2.6	89.4	299
19AR110	Spot 24	636.0479	36297.88	22.42431	17.42847	0.940377	0.592842	1.244576	0.07545	0.800973	0.643571	468.9046	3.622455	472.6808	4.703493	492.034	21.00575	468.9	3.6	95.3	23
19AR110	Spot 266	463.4102	26017.82	37.56097	17.34904	0.95101	0.59613	1.220328	0.075472	0.646281	0.529597	469.04	2.923666	474.775	4.627882	503.5479	22.80272	469.0	2.9	93.1	232
19AR110	Spot 467	394.6031	15199.61	1.75739	17.48333	1.062673	0.585652	1.643387	0.075569	0.716125	0.435761	469.6218	3.243499	468.0873	6.163211	461.512	32.79821	469.6	3.2	101.8	410
19AR110	Spot 463	437.0452	21216.96	116.1841	17.69138	1.6854	0.58148	2.006215	0.07557	0.751858	0.374764	469.6281	3.405386	465.4121	7.490077	445.6118	41.35319	469.6	3.4	105.4	407
19AR110	Spot 590	519.688	17361.32	11.33421	17.56962	0.765164	0.582073	1.176099	0.075606	0.646255	0.54949	469.8436	2.928373	465.7927	4.393668	446.8196	21.85268	469.8	2.9	105.2	517
19AR110	Spot 387	333.6193	10486.69	5.567096	17.91112	1.563264	0.565871	1.909934	0.075619	0.773814	0.405152	469.9214	3.506944	455.3409	7.008367	383.3533	39.24128	469.9	3.5	122.6	337

19AR110	Spot 366	216.0701	9056.563	7.720289	17.78812	1.626647	0.56807	1.855847	0.075657	0.713824	0.384635	470.1475	3.236566	456.7656	6.826765	390.9401	38.42436	470.1	3.2	120.3	318
19AR110	Spot 49	264.1943	8169.748	1.978532	17.44949	1.710307	0.580631	3.369916	0.075749	0.716757	0.212693	470.6999	3.253546	464.8671	12.57017	437.1044	73.32028	470.7	3.3	107.7	45
19AR110	Spot 123	127.3433	4239.341	1.382903	17.69369	3.382436	0.551691	4.997563	0.075781	0.718783	0.143827	470.8878	3.264	446.1039	18.04366	321.2398	112.4174	470.9	3.3	146.6	106
19AR110	Spot 67	180.4283	8792.681	7.730502	17.72624	2.068034	0.571289	2.546286	0.075786	0.780628	0.306575	470.9189	3.545065	458.8477	9.400463	399.7994	54.32522	470.9	3.5	117.8	59
19AR110	Spot 155	255.9547	13719.81	2.223232	17.42208	2.127467	0.590233	2.592079	0.075854	0.90291	0.348334	471.33	4.103833	471.0165	9.769094	470.4742	53.78525	471.3	4.1	100.2	134
19AR110	Spot 164	107.5962	3961.909	1.968454	17.08361	2.921326	0.575298	5.363688	0.075894	1.052216	0.196174	471.567	4.784762	461.435	19.89202	412.2581	117.6778	471.6	4.8	114.4	141
19AR110	Spot 191	436.9684	24111.26	6.035879	17.43267	1.125447	0.593595	1.391704	0.076039	0.63743	0.458021	472.4338	2.903732	473.1608	5.263722	477.6293	27.35874	472.4	2.9	98.9	165
19AR110	Spot 25	199.7889	7001.512	8.635165	16.99975	1.876155	0.595082	4.004833	0.076041	0.74452	0.185905	472.4499	3.391682	474.1078	15.17192	483.1206	86.95053	472.4	3.4	97.8	24
19AR110	Spot 499	295.6921	11158.91	1.787733	17.99227	1.591806	0.567703	2.752868	0.076044	0.745343	0.270751	472.4684	3.395557	456.5276	10.12248	378.0135	59.61601	472.5	3.4	125.0	437
19AR110	Spot 313	123.4418	6337.551	1.137604	16.85273	3.126005	0.595853	3.914019	0.076081	0.873981	0.223295	472.6897	3.983393	474.5988	14.83987	484.8192	84.2822	472.7	4.0	97.5	272
19AR110	Spot 352	214.137	8094.445	1.507315	17.66372	2.658241	0.576043	3.345397	0.076147	0.70877	0.211864	473.0838	3.232994	461.9152	12.41612	407.7037	73.16221	473.1	3.2	116.0	305
19AR110	Spot 408	313.8502	18837.91	11.65219	17.2319	1.671052	0.598126	1.887802	0.076227	0.656243	0.347623	473.5612	2.996307	476.0439	7.174233	488.9761	39.07652	473.6	3.0	96.8	356
19AR110	Spot 84	171.5894	8961.539	1.168505	17.3777	2.502125	0.588658	3.772509	0.076228	0.97279	0.257863	473.5694	4.441691	470.01	14.19453	453.6102	80.9439	473.6	4.4	104.4	75
19AR110	Spot 23	376.2818	15343.89	25.06179	17.44933	1.844819	0.592269	2.004356	0.076236	0.706642	0.352553	473.6139	3.22677	472.3153	7.570337	466.9973	41.55692	473.6	3.2	101.4	22
19AR110	Spot 48	328.9864	12504.41	5.352453	17.55513	1.647201	0.589313	2.775449	0.076283	0.88263	0.318013	473.8978	4.032718	470.4289	10.44997	454.5067	58.42503	473.9	4.0	104.3	44
19AR110	Spot 237	354.5243	12379.42	1.437013	17.39259	1.36976	0.59158	1.742711	0.076301	0.82283	0.472155	474.0057	3.760318	471.8763	6.577283	462.5208	34.06463	474.0	3.8	102.5	205
19AR110	Spot 109	132.7048	6410.39	10.02981	17.53572	2.767191	0.573762	3.632326	0.076316	0.658934	0.181408	474.0958	3.011867	460.445	13.44724	393.8523	80.14247	474.1	3.0	120.4	94
19AR110	Spot 498	148.5491	5241.14	2.449141	17.29165	2.611251	0.579309	3.332963	0.076378	0.738783	0.22166	474.4648	3.379377	464.0172	12.41438	413.5862	72.67484	474.5	3.4	114.7	436
19AR110	Spot 440	240.1908	14089.68	9.737435	16.8786	1.768743	0.612712	2.578078	0.076411	0.679953	0.263744	474.6617	3.111519	485.2694	9.945799	536.6277	54.41061	474.7	3.1	88.5	384
19AR110	Spot 43	38.20658	2195.623	1.673601	12.79506	7.031836	0.757954	8.410479	0.076458	1.432347	0.170305	474.946	6.558314	572.8288	36.83633	983.9524	168.9763	474.9	6.6	48.3	39
19AR110	Spot 28	559.4212	19406.64	11.7346	17.59651	1.060293	0.591557	1.520857	0.076459	0.688944	0.452997	474.9539	3.154533	471.8617	5.73981	457.8132	30.07661	475.0	3.2	103.7	27
19AR110	Spot 15	348.0135	13300.49	10.41084	17.45079	1.485599	0.59116	2.269125	0.076467	0.708848	0.312388	475.0008	3.245977	471.6082	8.560318	456.0975	47.85431	475.0	3.2	104.1	14
19AR110	Spot 243	123.0761	5057.638	1.787316	16.95178	4.225743	0.589736	5.284528	0.076473	0.700947	0.132641	475.0377	3.210036	470.6987	19.90784	450.5432	116.4218	475.0	3.2	105.4	210
19AR110	Spot 160	241.5043	7453.129	5.542602	17.17546	2.558724	0.592157	3.658671	0.076478	0.75335	0.205908	475.0656	3.450214	472.2442	13.81755	459.5255	79.43635	475.1	3.5	103.4	138
19AR110	Spot 277	197.8259	6158.808	1.66456	17.4839	3.307808	0.579808	4.223606	0.076483	0.891187	0.211002	475.0988	4.081762	464.3383	15.74085	412.4173	92.32652	475.1	4.1	115.2	242
19AR110	Spot 512	290.3712	16554.06	2.819569	17.53671	1.622044	0.590042	1.826198	0.076488	0.683012	0.374008	475.1238	3.128447	470.8942	6.88111	451.2782	37.63086	475.1	3.1	105.3	448
19AR110	Spot 125	206.6826	9997.313	1.83335	17.24883	1.962997	0.593528	2.389799	0.076497	0.630345	0.263765	475.1811	2.88755	473.1185	9.038253	464.0836	51.08372	475.2	2.9	102.4	108
19AR110	Spot 591	121.6669	4953.631	0.860181	16.51691	4.028231	0.605568	5.923213	0.076536	0.802301	0.13545	475.4123	3.676984	480.7613	22.68788	507.2894	129.1924	475.4	3.7	93.7	518
19AR110	Spot 358	64.4486	2845.057	2.69682	16.23673	4.442583	0.595255	5.638964	0.076567	0.87229	0.15469	475.6005	3.999272	474.2179	21.36811	468.5193	123.4261	475.6	4.0	101.5	310
19AR110	Spot 299	185.4547	11885.31	4.7963	17.19718	2.670283	0.597662	3.252444	0.076574	0.765024	0.235215	475.6418	3.507774	475.7492	12.3547	477.2089	69.90156	475.6	3.5	99.7	260
19AR110	Spot 64	83.06927	2449.437	1.382496	16.65263	5.263033	0.570403	10.43595	0.076594	0.934427	0.089539	475.7597	4.285543	458.2751	38.50708	372.4896	234.575	475.8	4.3	127.7	56
19AR110	Spot 214	162.8528	3974.674	1.632501	16.85612	2.936515	0.587088	5.329106	0.076623	1.195485	0.224331	475.9359	5.484782	469.0064	20.01902	436.181	115.687	475.9	5.5	109.1	184
19AR110	Spot 328	310.1381	13563.98	10.69017	17.39041	1.872541	0.594742	2.275243	0.076636	0.760608	0.334297	476.0126	3.490144	473.8914	8.616012	464.5965	47.52274	476.0	3.5	102.5	285
19AR110	Spot 353	104.3869	5195.076	1.881796	16.4997	3.363585	0.611398	4.14756	0.076772	0.69404	0.167337	476.8261	3.189932	484.4414	15.9801	521.5686	89.74774	476.8	3.2	91.4	306
19AR110	Spot 127	255.158	11458.57	4.807088	17.46815	1.577813	0.590017	2.40301	0.076783	0.667626	0.277829	476.8897	3.068923	470.8787	9.054404	442.6336	51.35614	476.9	3.1	107.7	110
19AR110	Spot 248	64.86938	3046.311	2.373567	15.1826	4.719372	0.645687	8.814532	0.076786	0.969998	0.110045	476.9128	4.459068	505.8209	35.12991	639.726	188.7493	476.9	4.5	74.5	215
19AR110	Spot 86	76.41543	3550.744	1.945911	15.45129	5.892964	0.643266	8.353842	0.076812	1.043131	0.124868	477.0675	4.796759	504.3263	33.2165	630.9174	178.8276	477.1	4.8	75.6	77
19AR110	Spot 583	242.4619	6271.878	1.160655	17.32397	2.375065	0.585256	3.922834	0.076818	0.894028	0.227904	477.1031	4.111416	467.8336	14.70641	423.5511	85.25737	477.1	4.1	112.6	510
19AR110	Spot 517	227.4661	12019.23	8.151391	17.18187	2.408397	0.602682	3.179788	0.076843	0.837916	0.263513	477.2489	3.854505	478.9343	12.14198	487.9513	67.713	477.2	3.9	97.8	452
19AR110	Spot 404	316.2576	15063.03	2.253489	17.224	1.085452	0.601223	1.551629	0.076858	0.604211	0.389404	477.3426	2.779963	478.0096	5.915704	482.1942	31.58041	477.3	2.8	99.0	352
19AR110	Spot 348	375.9291	15765.5	4.011702	17.40939	1.048188	0.599267	1.453739	0.07688	0.742002	0.51041	477.4701	3.414815	476.7683	5.531209	474.3785	27.66237	477.5	3.4	100.7	301
19AR110	Spot 272	234.1478	12581.1	19.51172	17.38428	1.712354	0.59973	2.687419	0.076889	0.724559	0.269611	477.5246	3.334903	477.0626	10.23032	475.8211	57.2462	477.5	3.3	100.4	237
19AR110	Spot 422	199.5168	8239.926	4.692382	17.5115	1.664849	0.584364	1.961063	0.076913	0.780542	0.39802	477.6689	3.593622	467.2618	7.344417	417.3989	40.2004	477.7	3.6	114.4	370
19AR110	Spot 595	371.0424	15901.12	9.407593	17.10458	1.334354	0.607965	1.578094	0.076953	0.62165	0.393925	477.9081	2.863463	482.2761	6.058569	504.0304	31.92091	477.9	2.9	94.8	522
19AR110	Spot 508	363.5191	10849.58	5.723441	17.51705	1.218312	0.590682	2.160548	0.076965	0.677282	0.313477	477.9806	3.120171	471.303	8.146545	439.8646	45.65598	478.0	3.1	108.7	445
19AR110	Spot 252	298.6097	10081.64	11.23751	17.34537	1.38005	0.597435	2.017462	0.076978	0.852417	0.422519	478.0599	3.92763	475.6044	7.661449	464.7643	40.53502	478.1	3.9	102.9	219
19AR110	Spot 362	275.1953	13616.61	6.822967	17.54096	0.844229	0.593987	1.707297	0.077196	0.731679	0.42856	479.3646	3.380176	473.4108	6.460069	445.6043	34.29122	479.4	3.4	107.6	314
19AR110	Spot 395	349.2384	20012.43	4.689043	17.8396	1.174138	0.586838	1.790195	0.077201	0.653685	0.365147	479.3918	3.020027	468.8461	6.722365	418.4906	37.22383	479.4	3.0	114.6	344
19AR110	Spot 55	207.8299	12909.46	3.060433	17.60367	2.529678	0.592633	2.706684	0.077221	0.729191	0.269404	479.5135	3.369687	472.548	10.2271	439.8117	58.0084	479.5	3.4	109.0	50
19AR110	Spot 294	144.0582	7242.33	1.721512	18.10467	2.310834	0.563457	3.194973	0.077222	0.804403	0.251771	479.5195	3.717299	453.774	11.69206	326.3291	70.18869	479.5	3.7	146.9	256
19AR110	Spot 309	157.5197	5924.547	2.14348	17.59384	2.156206	0.57606	3.229714	0.077263	1.010273	0.312806	479.7671	4.670987	461.9264	11.98696	375.1142	69.03449	479.8	4.7	127.9	268
19AR110	Spot 1	166.0494	7795.36	1.791652	17.8578	1.853043	0.575948	3.30836	0.077303	0.729232	0.220421	480.0035	3.373197	461.8541	12.27737	373.5257	72.6429	480.0	3.4	128.5	1
19AR110	Spot 226	392.9133	14836.67	2.318209	17.59964	1.153579	0.593895	2.10844	0.07731	0.726912	0.344763	480.0452	3.362748	473.3519	7.977192	441.9833	44.03054	480.0	3.4	108.6	195
19AR110	Spot 247	166.179	9212.055	3.105952	17.26302	2.764987	0.599976	3.056831	0.077389	0.827142	0.270588	480.5185	3.830048	477.2187	11.63968	462.3333	65.25893	480.5	3.8	103.9	214
19AR110	Spot 565	120.2328	4959.191	2.810697	17.43531	3.073625	0.579804	4.184717	0.077392	0.819903	0.195928	480.5358	3.796663	464.3355	15.59582	385.9448	92.20687	480.5	3.8	124.5	493
19AR110	Spot 249	81.55187	3887.041	0.539852	16.31458	3.969942	0.613521	7.39746	0.077452	0.860337	0.116302	480.8976	3.986787	485.7781	28.56813	509.8471	161.7381	480.9	4.0	94.3	216
19AR110	Spot 563	135.9905	4228.459	1.991441	17.24778	3.563863	0.582315	4.161603	0.077562	0.735118	0.176643	481.5531	3.410994	465.9483	15.55212	390.7198	91.98407	481.6	3.4	123.2	491
19AR110	Spot 339	298.0199	8432.108	6.171683	17.47981	1.258243	0.595195	1.929081	0.077715	0.585078	0.303293	482.4709	2.719782	474.1801	7.308591	435.2111	40.93911	482.5	2.7	110.9	295
19AR110	Spot 31	106.4661	6755.402	4.16949	17.5449	3.114634	0.588862	4.545301	0.077751	0.879468	0.19349	482.6876	4.090049	470.1405	17.10649	410.2952	99.78056	482.7	4.1	117.6	30
19AR110	Spot 3	128.8915	3987.379	4.322249	16.59364	3.284348	0.606776	6.846873	0.077956	0.847897	0.123837	483.9093	3.952832	481.5246	26.25983	471.172	150.5103	483.9	4.0	102.7	3
19AR110	Spot 449	367.066	1321.845	3.024417	16.11451	1.032282	0.547472	2.341584	0.07803	0.892233	0.381038	484.3504	4.163174	443.3395	8.411801	236.6889	49.94897	484.4	4.2	204.6	393
19AR110	Spot 384	127.1108	6058.276	1.987484	16.93601	4.044916	0.607873	4.297173	0.078071	1.179857	0.274566	484.5957	5.50792	482.2177	16.49727	471.8776	91.46736	484.6	5.5	102.7	335
19AR110	Spot 62	320.0618	14608.4	3.347	17.47157	1.439235	0.609277	1.870758	0.078633	0.626549	0.334917	487.9576	2.94445	483.1041	7.191821	461.0841	39.10203	488.0	2.9	105.8	54
19AR110	Spot 176	421.7639	27190.59	42.42113	17.64891	1.770593	0.608308	2.120649	0.078742	0.896373	0.422688	488.6111	4.217908	482.4926	8.144462	454.497	42.65911	488.6	4.2	107.5	153
19AR110	Spot 233	87.4323	5107.131	2.0585	16.30889	4.270874	0.634406	4.66018	0.078803	1.110733	0.238346	488.9711	5.230295	498.8369	18.36908	545.3287	98.96344	489.0	5.2	89.7	201
19AR110	Spot 4	173.3103	9771.153	3.969257	17.07195	3.027567	0.619966	3.718439	0.078992	0.904588	0.243271	490.1046	4.269086	489.826	14.45047	489.4616	79.61094	490.1	4.3	100.1	4
19AR110	Spot 130	226.1402	15124.1	5.183268	17.02901	1.629828	0.627855	2.101624	0.079032	0.716072	0.340723	490.34	3.380973	494.7587	8.230723	516.1962	43.38096	490.3	3.4	95.0	113
19AR110	Spot 471	197.2917	8220.964	2.805713	17.40495	2.323196	0.607623	2.569879	0.079212	0.829553	0.322799	491.4149	3.925045	482.0599	9.862936	438.7595	54.15908	491.4	3.9	112.0	413
19AR110	Spot 529	249.0358	16363.17	2.35888	17.50775	2.234773	0.612405	2.639881	0.079245	1.119359	0.424019	491.6149	5.298341	485.0759	10.18107	455.2706	53.07418	491.6	5.3	108.0	463
19AR110	Spot 235	164.3885	7034.87	1.924099	17.14235	2.305449	0.615066	3.932045	0.079406	0.882425	0.224419	492.5746	4.184689	486.7498	15.2059	460.365	84.98432	492.6	4.2	107.0	203
19AR110	Spot 229	260.0178	11611.79	3.625745	17.58863	1.623317	0.609261	2.64791	0.079648	1.484218	0.560524	494.0241	7.058479	483.0937	10.17945	432.5058	48.88092	494.0	7.1	114.2	198
19AR110	Spot 46	163.4076	8512.053	8.059933	17.0775	1.917952	0.632656	2.405398	0.080382	0.844764	0.351195	498.4006	4.051663	497.7493	9.464605	495.6939	49.63694	498.4	4.1	100.5	42
19AR110	Spot 582	204.5368	9476.462	11.51948	16.85802	1.778279	0.642273	2.891494	0.080749	0.988347	0.341812	500.5896	4.76034	503.7125	11.48273	518.8568	59.63941	500.6	4.8	96.5	509
19AR110	Spot 228	241.7186	2984.54	2.810415	16.99444	1.974436	0.603899	8.328392	0.081428	0.749733	0.090021	504.6392	3.639149	479.7052	31.85084	363.1715	187.3394	504.6	3.6	139.0	197
19AR110	Spot 139	140.267	5564.093	1.799382	17.04601	2.324973	0.632511	3.290089	0.0819	0.804467	0.244512	507.4534	3.925752	497.6587	12.94413	453.8415	70.82883	507.5	3.9	111.8	121
19AR110	Spot 436	256.6467	11967.19	8.648769	17.21485	1.720866	0.646023	2.794723	0.082417	0.607845	0.217497	510.5309	2.983533	506.0281	11.13776	486.6904	60.21743	510.5	3.0	104.9	381
19AR110	Spot 505	219.3682	12096.44	0.469589	17.12144	1.991264	0.650901	2.316994	0.082582	0.626092	0.270218	511.5176	3.078808	509.0331	9.276	498.8256	49.16059	511.5	3.1	102.5	442
19AR110	Spot 379	142.9694	6746.751	125.2884	17.51051	3.318046	0.626361	3.969641	0.08278	0.792481	0.199635	512.6977	3.905659	493.8263	15.52468	408.1934	87.03618	512.7	3.9	125.6	330
19AR110	Spot 105	542.6148	16541.08	10.46428	17.4715	0.982034	0.642385	1.650356	0.082919	0.686179	0.415776	513.5238	3.386998	503.7817	6.554417	460.7239	33.28125	513.5	3.4	111.5	90
19AR110	Spot 80	131.266	7295.81	1.606992	17.02791	2.841743	0.649627	3.5066	0.083063	1.072776	0.30593	514.3808	5.303738	508.249	14.02239	481.7363	73.78808	514.4	5.3	106.8	71
19AR110	Spot 198	290.986	16251.04	5.983684	16.90905	2.100586	0.666235	2.289634	0.083145	0.753088	0.328912	514.8662	3.726597	518.4204	9.296065	535.0683	47.31392	514.9	3.7	96.2	171
19AR110	Spot 231	471.7832	23630.47	5.677904	17.2235	1.178345	0.65766	1.588547	0.083215	1.003927	0.631978	515.2835	4.971723	513.1814	6.399433	504.7825	27.11066	515.3	5.0	102.1	200
19AR110	Spot 171	113.1419	3942.919	3.827552	17.33925	3.207111	0.619452	3.708984	0.083384	0.71863	0.193754	516.2885	3.565517	489.5037	14.40634	366.9831	82.01416	516.3	3.6	140.7	148
19AR110	Spot 376	130.4411	7258.208	2.326587	17.35152	3.445015	0.637999	3.962553	0.083411	0.751613	0.189679	516.4497	3.730283	501.0665	15.67277	432.3601	86.69596	516.4	3.7	119.4	327
19AR110	Spot 335	237.4687	10356.88	4.384392	17.34447	1.832974	0.647128	2.509903	0.083416	0.71596	0.285254	516.4779	3.553524	506.7097	10.01299	463.8439	53.3124	516.5	3.6	111.3	292
19AR110	Spot 443	145.4524	6925.916	7.436124	16.5781	2.7415	0.670764	4.381034	0.083638	0.716136	0.163463	517.7991	3.563128	521.1771	17.86101	536.9726	94.63824	517.8	3.6	96.4	387
19AR110	Spot 480	119.115	6221.936	0.971737	15.8041	4.035366	0.702937	5.51379	0.083656	1.029388	0.186693	517.9082	5.122749	540.5438	23.11389	638.1519	116.6134	517.9	5.1	81.2	421
19AR110	Spot 32	292.8509	8618.916	2.967338	17.05443	0.939007	0.658217	2.677301	0.083688	0.69221	0.258548	518.1003	3.446011	513.5228	10.79124	494.1496	57.02256	518.1	3.4	104.8	31
19AR110	Spot 482	141.2895	7126.01	1.512911	17.1404	3.311708	0.649919	4.601554	0.083734	0.768747	0.167063	518.3738	3.828972	508.4289	18.40681	464.8959	100.5794	518.4	3.8	111.5	423
19AR110	Spot 456	231.388	12447.79	0.742772	17.28491	2.332409	0.654086	2.544196	0.083736	0.702187	0.275996	518.383	3.497512	510.9903	10.21579	478.9891	54.05224	518.4	3.5	108.2	400
19AR110	Spot 215	173.6199	5956.916	2.749413	17.42054	2.880964	0.63353	3.564319	0.083744	0.867088	0.243269	518.4345	4.319273	498.2922	14.03699	407.7503	77.37474	518.4	4.3	127.1	185
19AR110	Spot 10	340.3398	20938.01	0.904618	17.19769	1.383014	0.662222	1.80035	0.083749	0.685538	0.38078	518.4636	3.415094	515.9724	7.28298	505.8908	36.63772	518.5	3.4	102.5	10
19AR110	Spot 470	241.8382	9842.81	1.556229	17.25878	1.843954	0.651737	2.67601	0.083789	0.712778	0.266358	518.6973	3.552328	509.5474	10.72174	469.6628	57.127	518.7	3.6	110.4	412
19AR110	Spot 340	72.49863	3766.619	1.138371	16.38189	5.064593	0.662407	6.894185	0.083915	0.820329	0.118988	519.4483	4.094022	516.0855	27.90037	502.1897	150.8611	519.4	4.1	103.4	296
19AR110	Spot 166	497.0415	20424.94	8.807587	17.2013	0.878626	0.666222	1.550821	0.083959	0.754804	0.486713	519.7093	3.768827	518.4129	6.296272	513.6518	29.7542	519.7	3.8	101.2	143
19AR110	Spot 377	156.865	5782.74	1.892447	16.86803	2.138637	0.655403	4.333725	0.08396	0.677599	0.156355	519.7168	3.383377	511.7982	17.42363	477.5236	94.67261	519.7	3.4	108.8	328
19AR110	Spot 190	443.0539	22246.96	5.434889	17.19721	1.031835	0.663949	1.448102	0.083963	0.694008	0.479253	519.7322	3.465409	517.0265	5.867168	506.0467	27.96134	519.7	3.5	102.7	164
19AR110	Spot 42	292.3879	9012.002	5.183033	17.35645	2.226794	0.65093	3.114166	0.084015	1.346733	0.432454	520.0457	6.728578	509.0509	12.46807	460.9057	62.26223	520.0	6.7	112.8	38
19AR110	Spot 553	372.5775	9055.426	1.25454	17.03484	1.545029	0.659261	3.191784	0.084095	0.679316	0.212833	520.5162	3.396963	514.1618	12.87743	486.9713	68.86228	520.5	3.4	106.9	484
19AR110	Spot 369	93.03373	4275.896	1.139748	16.75452	3.704564	0.650813	4.842528	0.084096	0.740566	0.15293	520.5264	3.703314	508.979	19.38712	458.3765	106.2117	520.5	3.7	113.6	320
19AR110	Spot 263	139.7815	6033.609	1.709124	16.92934	2.720562	0.659346	5.064188	0.084098	0.731129	0.144372	520.5363	3.656188	514.2137	20.435	487.176	110.6642	520.5	3.7	106.8	229
19AR110	Spot 412	92.12047	5875.105	1.534895	15.32924	3.058746	0.723114	3.606741	0.084109	0.800874	0.222049	520.601	4.005447	552.5038	15.3699	687.1826	75.06719	520.6	4.0	75.8	360
19AR110	Spot 227	430.3681	20522.84	9.114758	17.47689	1.173146	0.653943	1.44201	0.084115	0.706518	0.489954	520.6367	3.533772	510.9026	5.78925	468.5466	27.83535	520.6	3.5	111.1	196
19AR110	Spot 275	267.7904	13882.11	6.909802	17.15767	2.010104	0.66623	2.565059	0.084187	0.909223	0.354465	521.0636	4.551215	518.4178	10.41434	507.7537	52.76426	521.1	4.6	102.6	240
19AR110	Spot 332	133.8332	9521.97	1.735503	17.02815	3.124967	0.663149	3.349874	0.084247	0.779336	0.232646	521.4245	3.903648	516.5382	13.56326	495.9505	71.83134	521.4	3.9	105.1	289
19AR110	Spot 424	279.1621	11116.55	1.839071	16.98118	1.430856	0.667323	2.115702	0.084256	0.643685	0.304242	521.4739	3.224474	519.0837	8.598276	509.5563	44.32214	521.5	3.2	102.3	372
19AR110	Spot 140	144.3935	6914.061	18.58998	16.84081	2.037142	0.665407	2.884803	0.084311	0.874652	0.303193	521.8037	4.384138	517.916	11.70394	501.7642	60.52889	521.8	4.4	104.0	122
19AR110	Spot 388	213.6112	8830.649	5.053476	17.6171	1.685601	0.638767	2.707075	0.084357	0.814096	0.300729	522.0745	4.082635	501.5427	10.7145	409.8786	57.75794	522.1	4.1	127.4	338
19AR110	Spot 552	76.60796	3957.886	1.770134	16.37891	3.928009	0.664721	4.549295	0.084441	1.03641	0.227818	522.5775	5.202336	517.4979	18.44675	496.1131	97.66013	522.6	5.2	105.3	483
19AR110	Spot 330	123.1872	6018.821	1.980916	16.62827	3.200761	0.672005	3.743036	0.084595	0.89041	0.237884	523.4926	4.476989	521.9309	15.27642	516.0386	79.88376	523.5	4.5	101.4	287
19AR110	Spot 81	154.1856	9549.557	2.92571	16.8386	2.466122	0.675334	2.94291	0.08466	1.045034	0.355102	523.8769	5.258143	523.9504	12.04604	525.2036	60.34608	523.9	5.3	99.7	72
19AR110	Spot 336	164.9435	8885.158	5.235403	16.43597	2.781837	0.691681	2.902158	0.084701	0.649245	0.223711	524.122	3.268177	533.8099	12.04921	576.3563	61.51731	524.1	3.3	90.9	293
19AR110	Spot 541	264.7366	14989.06	1.878234	17.60322	1.33606	0.648476	1.971741	0.084735	0.694413	0.352183	524.3219	3.496825	507.5403	7.875889	433.5661	41.11661	524.3	3.5	120.9	474
19AR110	Spot 351	116.7314	7195.287	1.382982	17.1031	3.364059	0.660896	3.951703	0.084972	0.737028	0.186509	525.7307	3.720991	515.1621	15.96764	469.4898	85.9852	525.7	3.7	112.0	304
19AR110	Spot 532	164.6418	10104.38	1.433355	16.40219	2.948241	0.695397	3.346976	0.084989	1.02409	0.305975	525.8302	5.171202	536.0382	13.94028	580.6553	69.20937	525.8	5.2	90.6	466
19AR110	Spot 514	351.2332	16928.83	2.434966	16.96058	0.87685	0.682737	1.189412	0.085384	0.71745	0.603197	528.1814	3.638348	528.4273	4.900074	530.4646	20.80257	528.2	3.6	99.6	449
19AR110	Spot 371	638.8735	18699.62	2.560938	17.1591	0.862913	0.678279	1.300716	0.085788	0.609238	0.468387	530.5795	3.103041	525.7337	5.337778	505.7243	25.30414	530.6	3.1	104.9	322
19AR110	Spot 537	168.1672	6599.05	2.390377	16.41497	1.576866	0.693661	2.719435	0.085952	0.98213	0.361152	531.5509	5.011083	534.9976	11.3096	550.6926	55.3616	531.6	5.0	96.5	471
19AR110	Spot 145	98.96375	5601.084	1.429983	17.3367	2.536078	0.656361	5.145918	0.08607	0.854141	0.165984	532.2517	4.363565	512.3854	20.70812	425.6558	113.249	532.3	4.4	125.0	127
19AR110	Spot 518	130.7967	5447.619	33.38201	16.5633	2.710436	0.691747	3.629814	0.087177	0.857862	0.236338	538.8224	4.434445	533.8498	15.07156	513.6216	77.51987	538.8	4.4	104.9	453
19AR110	Spot 476	44.71089	1610.295	1.767077	14.94628	5.686771	0.698383	6.925352	0.087437	1.100496	0.158908	540.3624	5.704252	537.8245	28.92325	528.0525	150.0028	540.4	5.7	102.3	418
19AR110	Spot 481	322.7298	14514.38	1.948148	16.96992	1.099326	0.701604	1.489577	0.087854	0.910968	0.611562	542.8308	4.742539	539.7485	6.236372	527.7249	25.83546	542.8	4.7	102.9	422
19AR110	Spot 400	350.7306	21189.74	1.796853	17.12494	1.172254	0.69929	1.408459	0.088381	0.702603	0.498845	545.9576	3.677971	538.367	5.885303	507.2791	26.86339	546.0	3.7	107.6	348
19AR110	Spot 20	163.3789	10143.63	1.128433	16.70788	2.484798	0.716564	3.430902	0.089125	0.66071	0.192576	550.363	3.485406	548.6364	14.5433	542.4453	73.61466	550.4	3.5	101.5	19
19AR110	Spot 437	211.9683	10573.46	2.049749	16.89528	1.606165	0.717441	2.013305	0.090039	0.939761	0.466775	555.7672	5.004075	549.1551	8.539925	522.7544	39.03822	555.8	5.0	106.3	382
19AR110	Spot 538	487.477	6572.18	0.752667	16.44008	0.906687	0.725995	1.308902	0.090196	0.733327	0.560261	556.6986	3.911114	554.1995	5.590302	544.9174	23.71423	556.7	3.9	102.2	472
19AR110	Spot 581	600.8042	24428.24	2.483419	16.77585	0.821223	0.737991	1.188388	0.090849	0.808753	0.680546	560.5585	4.342015	561.2325	5.123837	564.8927	18.96414	560.6	4.3	99.2	508
19AR110	Spot 378	616.0828	38688.17	3.858213	16.76166	0.691406	0.742529	1.208327	0.091031	0.792978	0.656261	561.6338	4.265139	563.8801	5.228191	573.9035	19.82936	561.6	4.3	97.9	329
19AR110	Spot 234	334.245	19177.22	4.304972	14.24937	1.199722	0.873727	1.552147	0.09141	0.93279	0.600967	563.873	5.036276	637.5887	7.349195	909.2505	25.54931	563.9	5.0	62.0	202
19AR110	Spot 251	95.73319	2286.858	1.468467	15.34649	3.914129	0.746884	6.932546	0.09214	3.923406	0.56594	568.1786	21.33788	566.415	30.10503	560.2654	124.6675	568.2	21.3	101.4	218
19AR110	Spot 430	199.9888	7408.791	2.244984	16.69835	1.946998	0.742078	2.715142	0.093087	0.757563	0.279014	573.7674	4.158824	563.6172	11.74421	523.7892	57.1811	573.8	4.2	109.5	378
19AR110	Spot 405	261.1963	12270.53	4.608393	15.81984	1.521485	0.798901	2.053053	0.093994	1.25745	0.612478	579.1136	6.964553	596.2087	9.25823	662.703	34.77801	579.1	7.0	87.4	353
19AR110	Spot 403	139.7045	6871.25	0.728026	16.33552	1.500593	0.769274	2.144406	0.094991	0.716164	0.333968	584.9889	4.005013	579.3463	9.467503	558.2255	44.07559	585.0	4.0	104.8	351
19AR110	Spot 589	274.5596	18074.51	0.85605	16.50175	1.227479	0.787308	1.551241	0.095858	0.607894	0.391876	590.0865	3.427819	589.6435	6.938426	588.8911	30.94485	590.1	3.4	100.2	516
19AR110	Spot 264	248.7435	10612.12	0.995387	16.30585	1.064234	0.795611	2.328489	0.095886	0.721996	0.310071	590.2535	4.072324	594.3497	10.4763	610.935	47.8658	590.3	4.1	96.6	230
19AR110	Spot 452	342.7904	19498.2	0.723477	16.58533	1.040282	0.788143	1.340019	0.096052	0.69372	0.517694	591.2322	3.919033	590.118	5.9972	586.7818	24.87833	591.2	3.9	100.8	396
19AR110	Spot 212	974.2701	52008.15	9.331312	16.47327	0.639259	0.800159	1.091435	0.096219	0.878079	0.804518	592.212	4.968384	596.9181	4.926026	615.7594	13.99828	592.2	5.0	96.2	182
19AR110	Spot 203	69.67711	4133.443	1.822194	15.88295	3.239432	0.791007	5.609933	0.096739	1.184898	0.211214	595.2672	6.737453	591.7427	25.16283	579.1919	119.2172	595.3	6.7	102.8	175
19AR110	Spot 324	1120.457	40222.89	4.809754	16.60177	0.60988	0.797247	0.993787	0.096777	0.671014	0.675209	595.4943	3.816845	595.2745	4.476216	595.3849	15.87069	595.5	3.8	100.0	282
19AR110	Spot 555	84.46832	4597.069	2.088907	16.70522	3.78869	0.753834	4.2579	0.096834	0.763961	0.179422	595.8311	4.34789	570.4468	18.58494	471.4922	92.73179	595.8	4.3	126.4	485
19AR110	Spot 303	79.6454	3655.126	10.23272	16.52932	4.072778	0.75065	5.76896	0.096906	0.897337	0.155546	596.2527	5.110413	568.6014	25.122	460.4468	126.429	596.3	5.1	129.5	263
19AR110	Spot 322	411.6877	27798.57	0.870154	16.50672	0.844244	0.799729	1.202188	0.096927	0.576152	0.479253	596.3743	3.281875	596.6758	5.424279	598.7522	22.84941	596.4	3.3	99.6	280
19AR110	Spot 427	117.4308	5268.24	1.874643	16.36412	4.030931	0.78128	4.428916	0.097336	0.764526	0.172621	598.7779	4.37164	586.2136	19.7268	538.8127	95.49277	598.8	4.4	111.1	375
19AR110	Spot 63	135.3124	10039.3	2.255216	16.58253	2.594809	0.790542	3.147776	0.097558	0.714678	0.227042	600.0795	4.095077	591.4795	14.11245	559.5678	66.82729	600.1	4.1	107.2	55
19AR110	Spot 204	174.0544	19330.48	0.791182	16.76643	2.118641	0.791518	2.291936	0.097726	0.795481	0.347078	601.0682	4.565246	592.0327	10.28222	558.4903	46.85555	601.1	4.6	107.6	176
19AR110	Spot 506	259.7367	9289.688	2.833785	15.6919	1.09185	0.837639	1.815765	0.097782	0.781907	0.430621	601.4003	4.489709	617.8418	8.404196	679.4626	34.99668	601.4	4.5	88.5	443
19AR110	Spot 355	342.4549	18055.73	2.325614	16.39557	1.158829	0.812388	1.514501	0.097978	0.658146	0.434563	602.5474	3.785952	603.793	6.893162	609.3923	29.50811	602.5	3.8	98.9	308
19AR110	Spot 167	414.6613	11627.82	1.803539	16.03167	0.995602	0.827702	1.611635	0.09798	0.752898	0.467164	602.5584	4.331081	612.3361	7.410932	649.607	30.59874	602.6	4.3	92.8	144
19AR110	Spot 574	233.7644	684.3568	1.582885	15.11569	3.11186	0.604734	47.02643	0.098421	0.934358	0.019869	605.1491	5.39698	480.2336	181.8624	NA	NA	605.1	5.4		501
19AR110	Spot 564	214.0208	8715.843	1.076969	16.49265	1.324889	0.799314	1.88849	0.098449	0.730914	0.387036	605.3113	4.222937	596.4416	8.518543	563.7811	37.91233	605.3	4.2	107.4	492
19AR110	Spot 533	384.4015	25923.9	3.202842	16.64181	1.259193	0.805918	1.763536	0.098557	0.95966	0.544168	605.9452	5.550085	600.1612	7.991272	579.3132	32.14705	605.9	5.6	104.6	467
19AR110	Spot 363	154.5206	5773.076	2.462302	15.70506	1.590439	0.829771	3.158288	0.098655	0.755763	0.239295	606.5215	4.374831	613.4851	14.54363	640.231	65.95288	606.5	4.4	94.7	315
19AR110	Spot 475	209.1445	11680.07	7.146207	16.41019	1.736035	0.815221	2.45462	0.098975	0.907464	0.369696	608.3989	5.268481	605.3787	11.1938	595.0459	49.4471	608.4	5.3	102.2	417
19AR110	Spot 193	696.2881	33119.31	3.306065	16.42995	0.711013	0.823394	0.968321	0.099074	0.621987	0.642335	608.9826	3.614381	609.9404	4.439963	614.4187	16.05056	609.0	3.6	99.1	166

19AR110	Spot 494	322.6751	20115.31	1.039814	16.37601	1.334986	0.824686	1.683581	0.099086	0.773967	0.459715	609.05	4.49802	610.6593	7.726319	617.5522	32.27699	609.1	4.5	98.6	433
19AR110	Spot 82	130.5582	6569.632	5.784693	15.93666	3.664809	0.829178	4.186744	0.099346	1.043536	0.249248	610.573	6.079113	613.1559	19.27304	623.6257	87.49031	610.6	6.1	97.9	73
19AR110	Spot 30	115.2628	5580.259	1.613244	15.88775	2.653133	0.828832	3.057356	0.099445	0.776715	0.254048	611.1566	4.528874	612.9637	14.07007	620.5653	63.82859	611.2	4.5	98.5	29
19AR110	Spot 451	197.8398	19973.17	4.494046	16.24697	1.708186	0.833108	2.035398	0.099483	0.766177	0.376426	611.3783	4.468971	615.335	9.393011	630.8796	40.63937	611.4	4.5	96.9	395
19AR110	Spot 450	390.5119	18840.37	1.385608	16.28378	1.056162	0.830753	1.390274	0.099501	0.676028	0.486255	611.4838	3.943797	614.0301	6.405873	624.3495	26.19561	611.5	3.9	97.9	394
19AR110	Spot 426	621.9929	31477.75	1.41228	16.4804	0.804714	0.824773	1.136073	0.099576	0.627057	0.551951	611.9242	3.660622	610.7079	5.213934	607.1199	20.48612	611.9	3.7	100.8	374
19AR110	Spot 100	53.3191	3994.982	1.236729	16.04392	5.489255	0.806005	7.338679	0.099607	1.028038	0.140085	612.1053	6.003157	600.2107	33.26765	556.4946	158.6685	612.1	6.0	110.0	86
19AR110	Spot 492	732.7225	56785.81	1.355281	16.52352	0.93235	0.827379	1.269477	0.099644	0.850609	0.670047	612.3215	4.968747	612.1569	5.836272	612.4683	20.35992	612.3	5.0	100.0	431
19AR110	Spot 556	272.945	29613.24	1.845579	16.67717	1.562106	0.81497	1.851192	0.099805	0.691991	0.373808	613.2685	4.048153	605.2382	8.440407	576.2072	37.3265	613.3	4.0	106.4	486
19AR110	Spot 168	194.207	9321.417	3.693855	16.42197	1.815001	0.819182	2.053657	0.100025	0.733071	0.356959	614.558	4.297066	607.5919	9.390189	582.6425	41.64541	614.6	4.3	105.5	145
19AR110	Spot 194	318.804	7996.809	1.661434	16.43218	1.091506	0.81318	1.927201	0.100128	0.707246	0.366981	615.1592	4.149555	604.2363	8.776342	564.3998	39.05012	615.2	4.1	109.0	167
19AR110	Spot 314	213.3843	17610.69	4.478652	16.52186	1.587781	0.82271	1.953929	0.100367	0.806498	0.412757	616.5602	4.742152	609.5593	8.955279	584.5487	38.63584	616.6	4.7	105.5	273
19AR110	Spot 500	435.7139	20468.89	6.195384	16.30497	0.983344	0.837022	1.335904	0.100384	0.697986	0.522482	616.6584	4.104734	617.5009	6.180632	621.509	24.57386	616.7	4.1	99.2	438
19AR110	Spot 7	300.0496	16082.48	0.934915	16.38141	1.220206	0.830701	1.408576	0.100463	0.638117	0.453023	617.1232	3.755347	614.001	6.489981	603.4236	27.19352	617.1	3.8	102.3	7
19AR110	Spot 79	956.1455	16028.4	2.990384	16.26571	0.970684	0.842573	1.645272	0.1009	0.980633	0.596031	619.6822	5.793872	620.5646	7.63939	624.7011	28.48028	619.7	5.8	99.2	70
19AR110	Spot 197	368.2276	14251.34	4.402485	16.29519	1.146862	0.837919	1.702496	0.100922	0.719712	0.422739	619.8116	4.253119	617.9966	7.881344	612.2746	33.3394	619.8	4.3	101.2	170
19AR110	Spot 189	184.2414	16580.19	3.470751	16.59065	1.440434	0.824654	1.880285	0.100941	0.681543	0.362468	619.9184	4.02822	610.6414	8.628893	577.3422	38.08944	619.9	4.0	107.4	163
19AR110	Spot 357	152.4604	8361.311	3.827937	15.97788	2.799385	0.844992	3.215694	0.100945	0.700238	0.217756	619.9448	4.138882	621.8966	14.95526	629.9218	67.64809	619.9	4.1	98.4	309
19AR110	Spot 290	93.13791	5113.483	1.105239	15.80009	3.365367	0.842917	4.221242	0.101417	1.088707	0.257912	622.7055	6.462299	620.7543	19.60665	614.5661	88.10489	622.7	6.5	101.3	253
19AR110	Spot 478	185.5735	13527.63	19.14814	15.57623	1.669152	0.883809	2.15417	0.101548	0.761426	0.353466	623.4762	4.524963	643.0377	10.26234	713.3056	42.80246	623.5	4.5	87.4	420
19AR110	Spot 483	370.2238	21931.08	1.423497	16.42995	1.160265	0.842516	1.645781	0.101554	0.874805	0.531544	623.5082	5.199002	620.5332	7.641476	610.618	30.13869	623.5	5.2	102.1	424
19AR110	Spot 543	314.4223	18446.7	3.466231	16.55813	1.322999	0.830081	1.69945	0.101602	0.815336	0.479765	623.7895	4.847656	613.6569	7.827029	577.4	32.39708	623.8	4.8	108.0	476
19AR110	Spot 579	140.5868	7122.297	0.989748	16.25625	2.297273	0.83424	2.811277	0.101895	0.88821	0.315945	625.5061	5.294774	615.962	12.98351	581.9797	57.95384	625.5	5.3	107.5	506
19AR110	Spot 18	414.4436	24149.18	1.641137	16.55301	0.870873	0.838526	1.368262	0.101907	0.713041	0.521129	625.5726	4.250991	618.3318	6.336532	592.836	25.29321	625.6	4.3	105.5	17
19AR110	Spot 345	232.9467	13367.81	3.667254	16.49832	1.389189	0.838689	2.011678	0.102152	0.857562	0.426292	627.0101	5.12378	618.422	9.317368	588.0606	39.46017	627.0	5.1	106.6	300
19AR110	Spot 2	158.1093	7582.029	4.205058	16.09793	1.73202	0.847465	1.964759	0.102251	0.660006	0.335922	627.5848	3.946859	623.2566	9.151587	608.4964	40.0032	627.6	3.9	103.1	2
19AR110	Spot 413	408.5587	4411.749	2.01174	15.54156	0.787573	0.855825	1.799686	0.102265	0.702767	0.390494	627.6707	4.203121	627.841	8.427225	629.372	35.69792	627.7	4.2	99.7	361
19AR110	Spot 267	729.6957	50324.61	4.107095	16.39189	0.752967	0.860022	1.124257	0.10236	0.696711	0.619708	628.223	4.170389	630.1349	5.278262	637.9591	18.9899	628.2	4.2	98.5	233
19AR110	Spot 596	208.4681	9454.944	4.267821	16.33544	1.430981	0.838878	1.80254	0.102383	0.583127	0.323503	628.3563	3.491204	618.5264	8.349695	583.639	37.03256	628.4	3.5	107.7	523
19AR110	Spot 373	280.0576	18435.09	3.723214	16.42217	1.192469	0.84714	1.525302	0.102429	0.594958	0.390059	628.6257	3.563488	623.078	7.103104	603.9051	30.40167	628.6	3.6	104.1	324
19AR110	Spot 219	275.3592	6231.388	1.783897	15.2107	1.240899	0.894305	2.436733	0.102451	0.789433	0.323972	628.7571	4.729235	648.6793	11.68134	719.5412	48.94043	628.8	4.7	87.4	189
19AR110	Spot 407	232.3122	13738.36	2.828239	15.45749	1.451859	0.893345	2.000408	0.102495	1.042133	0.52096	629.0122	6.245486	648.1647	9.584089	716.3588	36.26676	629.0	6.2	87.8	355
19AR110	Spot 13	251.4782	20718.03	5.894553	16.47976	1.51874	0.847513	1.973176	0.102733	0.676916	0.343059	630.4038	4.06529	623.2831	9.191078	598.4395	40.15346	630.4	4.1	105.3	13
19AR110	Spot 464	897.1633	42431.15	6.186237	15.29012	0.930469	0.924579	2.169587	0.103125	1.899048	0.875304	632.694	11.44438	664.7789	10.58354	776.0427	22.06807	632.7	11.4	81.5	408
19AR110	Spot 183	193.3376	15438.98	2.050356	16.18141	1.672208	0.864719	2.412872	0.103147	1.091255	0.452264	632.8236	6.577591	632.6957	11.36172	633.1973	46.33091	632.8	6.6	99.9	158
19AR110	Spot 287	309.4075	22884.74	4.405833	16.17853	1.097204	0.874517	1.386779	0.103618	0.771528	0.556345	635.5787	4.669683	638.0171	6.569345	647.6191	24.77299	635.6	4.7	98.1	250
19AR110	Spot 334	115.5028	6651.546	2.031136	15.8713	2.970023	0.87281	3.681524	0.104032	0.973334	0.264383	637.9923	5.912402	637.0921	17.42313	634.858	76.44144	638.0	5.9	100.5	291
19AR110	Spot 409	266.2117	16097.66	1.955957	15.9723	0.862582	0.881544	1.281382	0.104244	0.60941	0.475588	639.2307	3.708623	641.8162	6.095977	651.8814	24.19515	639.2	3.7	98.1	357
19AR110	Spot 295	125.7561	2484.545	2.915067	14.70864	2.806291	0.8925	4.446585	0.104604	1.921505	0.43213	641.3339	11.73011	647.7118	21.29574	670.9171	85.81184	641.3	11.7	95.6	257
19AR110	Spot 47	379.2015	29372.78	6.826394	15.59989	1.004346	0.93547	1.48742	0.106217	1.029213	0.691945	650.7423	6.370572	670.5082	7.299858	738.4054	22.7182	650.7	6.4	88.1	43
19AR110	Spot 391	255.3678	13825.98	1.936215	15.80201	1.500982	0.908652	2.516594	0.106261	1.984499	0.788565	650.9987	12.28817	656.3405	12.16563	675.6813	33.06927	651.0	12.3	96.3	341
19AR110	Spot 298	324.565	20727.13	5.70831	15.24071	1.140756	0.953406	1.585447	0.107021	1.055041	0.665454	655.424	6.575082	679.8746	7.857342	762.5975	24.94669	655.4	6.6	85.9	259
19AR110	Spot 561	48.48952	2914.377	0.919484	14.07561	3.487106	0.980883	7.036495	0.107898	1.061003	0.150786	660.5296	6.661146	694.0579	35.39326	805.137	145.7935	660.5	6.7	82.0	489
19AR110	Spot 485	239.0918	18336.87	3.639571	15.79955	1.248918	0.930361	1.642727	0.107984	0.652411	0.397151	661.0299	4.098886	667.8247	8.039283	691.7496	32.13964	661.0	4.1	95.6	425
19AR110	Spot 315	608.2969	3543.24	2.31479	15.40956	0.773913	0.905346	1.2414	0.108432	0.779855	0.628206	663.6349	4.917906	654.5804	5.989457	624.4114	20.84793	663.6	4.9	106.3	274
19AR110	Spot 165	32.28567	1904.376	2.244547	14.30815	5.96032	0.939449	7.241438	0.109105	1.286104	0.177603	667.5496	8.155818	672.5939	35.63098	690.4491	152.1893	667.5	8.2	96.7	142
19AR110	Spot 530	137.8529	11889.99	2.218281	15.63651	2.559822	0.948432	2.792581	0.10999	0.995626	0.356525	672.6881	6.359863	677.2858	13.80332	693.5489	55.60772	672.7	6.4	97.0	464
19AR110	Spot 592	697.1706	3926.748	1.955972	15.44337	0.736997	0.936029	1.307095	0.111598	0.579684	0.443491	682.0232	3.751625	670.8017	6.416826	634.2489	25.24194	682.0	3.8	107.5	519
19AR110	Spot 382	248.8571	15717.93	1.730106	15.92912	1.104213	0.956306	1.377973	0.112267	0.758743	0.550622	685.8983	4.936903	681.3809	6.839705	667.4211	24.60626	685.9	4.9	102.8	333
19AR110	Spot 432	36.81338	2139.168	1.094373	14.24814	6.448308	0.982405	7.037774	0.112511	0.911394	0.1295	687.3171	5.941787	694.8375	35.42743	720.1514	148.2997	687.3	5.9	95.4	379
19AR110	Spot 230	237.158	17100.52	2.414334	15.86168	1.03375	0.963026	1.468052	0.112537	0.723847	0.493066	687.4662	4.720051	684.863	7.31292	677.2571	27.30941	687.5	4.7	101.5	199
19AR110	Spot 206	145.648	9137.515	5.534456	14.65783	1.856581	1.060504	5.057386	0.115694	4.543991	0.898486	705.7344	30.37569	734.0718	26.43583	822.4892	46.36099	705.7	30.4	85.8	177
19AR110	Spot 285	59.68347	4045.19	4.852589	15.33012	4.18374	0.998301	5.379779	0.11736	0.850402	0.158074	715.3523	5.757985	702.9472	27.29604	664.4529	113.8593	715.4	5.8	107.7	249
19AR110	Spot 52	871.3108	74961.05	5.259552	15.63556	0.694408	1.0323	0.955944	0.117376	0.647413	0.67725	715.4423	4.384087	720.0773	4.93042	735.4859	14.89145	715.4	4.4	97.3	48
19AR110	Spot 416	354.1123	27029.27	4.747674	15.67616	1.091513	1.020858	1.373107	0.11776	0.667816	0.486354	717.6588	4.535497	714.3446	7.043215	704.8773	25.5489	717.7	4.5	101.8	364
19AR110	Spot 442	309.9915	20865.88	2.987547	15.70435	1.002592	1.049306	1.604715	0.120949	0.735439	0.458299	736.0217	5.115413	728.5383	8.343202	706.4847	30.35051	736.0	5.1	104.2	386
19AR110	Spot 144	291.5403	21058.28	1.209674	15.55561	0.990023	1.065329	1.234845	0.121292	0.605609	0.490433	737.996	4.223027	736.4466	6.467594	732.6781	22.775	738.0	4.2	100.7	126
19AR110	Spot 129	168.9555	10946	2.349124	15.42688	1.617896	1.068495	2.785462	0.122335	1.27091	0.456266	743.9914	8.930233	738.0021	14.61084	720.7705	52.60732	744.0	8.9	103.2	112
19AR110	Spot 8	503.8906	20367.95	7.367238	15.55998	0.760211	1.073338	1.327049	0.122701	0.930144	0.700911	746.0883	6.553163	740.3765	6.975737	724.0413	20.0775	746.1	6.6	103.0	8
19AR110	Spot 327	110.7249	5278.984	2.024471	14.20021	1.887767	1.153898	3.434801	0.123869	1.630701	0.474759	752.7942	11.58617	779.0824	18.68628	856.0355	62.81193	752.8	11.6	87.9	284
19AR110	Spot 540	433.1701	26388.31	6.852225	14.6599	0.754164	1.151383	1.347058	0.12414	0.966385	0.717405	754.3459	6.87953	777.8958	7.320246	846.9809	19.53712	754.3	6.9	89.1	473
19AR110	Spot 545	261.25	21821.11	3.611529	15.15228	1.164891	1.135251	1.598504	0.126643	0.63958	0.400111	768.6864	4.634554	770.2534	8.629727	775.695	30.81708	768.7	4.6	99.1	477
19AR110	Spot 308	71.97963	5544.661	2.481165	13.79946	3.871466	1.230988	4.440161	0.128459	1.313252	0.295767	779.0672	9.637066	814.7888	24.88125	914.5503	87.31366	779.1	9.6	85.2	267
19AR110	Spot 262	284.5459	12058.4	3.569534	14.7436	1.170431	1.188084	1.762608	0.129032	0.61573	0.349329	782.3413	4.536285	795.0716	9.718118	831.8549	34.45204	782.3	4.5	94.0	228
19AR110	Spot 446	483.9093	29504.34	3.293142	14.0584	0.863924	1.271847	1.402278	0.130775	0.959679	0.684372	792.2833	7.154709	833.2165	7.971292	944.9048	20.94119	792.3	7.2	83.8	390
19AR110	Spot 320	71.53743	7418.598	2.577151	14.54974	2.871896	1.229012	3.24128	0.133937	0.758733	0.234084	810.2847	5.777211	813.8887	18.14832	824.6566	65.80293	810.3	5.8	98.3	278
19AR110	Spot 37	143.1839	11515.36	3.946747	14.0335	1.190157	1.292771	1.412619	0.13401	0.589445	0.417271	810.7039	4.49038	842.5256	8.087703	928.2323	26.36143	810.7	4.5	87.3	35
19AR110	Spot 9	361.5069	28073.83	1.014952	15.17402	0.781462	1.214079	1.071446	0.134905	0.660582	0.616533	815.789	5.061915	807.0637	5.96567	783.9611	17.72096	815.8	5.1	104.1	9
19AR110	Spot 406	241.3616	7158.499	1.60095	14.12221	1.064538	1.291125	1.419752	0.136947	0.827782	0.583047	827.3736	6.427558	841.796	8.124021	880.9083	23.86091	827.4	6.4	93.9	354
19AR110	Spot 73	225.5197	18047.12	4.696247	14.18001	1.195575	1.339946	2.000506	0.139663	1.381898	0.690774	842.7592	10.91693	863.2055	11.63243	916.9886	29.73814	842.8	10.9	91.9	65
19AR110	Spot 253	229.9153	21446.12	1.22749	14.40022	1.08048	1.340628	1.389121	0.14137	0.729131	0.524886	852.4026	5.821749	863.5013	8.078952	892.9868	24.41618	852.4	5.8	95.5	220
19AR110	Spot 318	68.96464	7824.611	2.90794	14.30096	2.503247	1.327029	2.916401	0.141838	0.891354	0.305635	855.0498	7.137691	857.5849	16.88869	865.0672	57.61557	855.0	7.1	98.8	276
19AR110	Spot 240	135.6432	16293.62	2.259047	14.46442	1.627862	1.338658	1.783069	0.142707	0.660514	0.370437	859.9497	5.317529	862.6465	10.36373	870.4884	34.30224	859.9	5.3	98.8	208
19AR110	Spot 217	64.81646	9652.299	1.426666	14.66366	3.288252	1.313524	3.539052	0.143043	1.011697	0.285867	861.8456	8.161548	851.6748	20.40513	826.1884	70.78169	861.8	8.2	104.3	187
19AR110	Spot 460	310.535	24377.31	2.011479	14.6092	0.898132	1.344314	1.421114	0.143803	0.761637	0.535943	866.1311	6.172807	865.0989	8.27472	863.3781	24.90185	866.1	6.2	100.3	404
19AR110	Spot 201	244.5414	19162.98	4.931117	14.36545	1.086335	1.370642	1.491479	0.144574	0.740579	0.49654	870.4746	6.030252	876.4389	8.756204	892.4381	26.7331	870.5	6.0	97.5	174
19AR110	Spot 600	240.9574	20633.91	2.912594	14.21564	0.769842	1.38553	1.047875	0.144934	0.642834	0.613464	872.5004	5.245728	882.7958	6.179819	909.5681	17.03694	872.5	5.2	95.9	526
19AR110	Spot 542	220.4289	3482.823	3.077786	13.61907	1.090552	1.490836	5.347624	0.156912	0.875301	0.16368	939.5925	7.652995	926.6575	32.51052	896.9038	108.9269	896.9	108.9	104.8	475
19AR110	Spot 118	102.7045	9135.803	1.607718	14.08952	1.445743	1.527158	2.636931	0.160272	0.613851	0.23279	958.2862	5.466111	941.3568	16.18143	902.8314	52.85526	902.8	52.9	106.1	102
19AR110	Spot 236	106.7341	9224.426	1.869732	14.39332	1.465233	1.406533	1.911062	0.150422	0.855064	0.447429	903.3271	7.207265	891.6965	11.34177	863.8859	35.4469	903.3	7.2	104.6	204
19AR110	Spot 157	119.8641	8394.797	2.363232	14.06767	1.197873	1.549475	1.520461	0.162233	0.791914	0.520838	969.1719	7.125932	950.2843	9.383211	907.6696	26.74334	907.7	26.7	106.8	136
19AR110	Spot 534	171.2672	1753.566	3.305873	13.30861	1.175461	1.396177	2.532177	0.151716	1.30818	0.516623	910.5754	11.10892	887.3176	14.98225	830.663	45.22055	910.6	11.1	109.6	468
19AR110	Spot 305	97.17721	10493.37	4.132078	14.03395	1.959462	1.519267	2.477811	0.158605	1.14706	0.462933	949.0197	10.12246	938.1816	15.17366	913.6932	45.19025	913.7	45.2	103.9	265
19AR110	Spot 111	99.10547	10572.79	1.530532	14.05731	2.07738	1.548844	2.280617	0.161632	0.693852	0.304239	965.8373	6.223616	950.033	14.07261	914.51	44.71051	914.5	44.7	105.6	96
19AR110	Spot 97	84.92247	6673.581	1.647402	13.91098	1.583086	1.503143	3.210797	0.156321	0.822841	0.256273	936.3017	7.170912	931.6619	19.57994	921.6141	63.80813	921.6	63.8	101.6	84
19AR110	Spot 51	43.43505	3091.311	4.701045	13.45226	3.374778	1.496	4.223374	0.155508	0.982042	0.232525	931.7647	8.51977	928.7601	25.70806	922.5482	84.45277	922.5	84.5	101.0	47
19AR110	Spot 576	336.3363	4316.768	1.343892	13.65327	0.832813	1.44228	1.212634	0.149717	0.671519	0.553769	899.3791	5.63713	906.668	7.271455	925.3438	20.76215	925.3	20.8	97.2	503
19AR110	Spot 282	453.9325	31943.84	1.652049	14.25145	0.710076	1.442778	1.031729	0.149667	0.71773	0.695657	899.0966	6.023282	906.875	6.187519	926.7747	15.24243	926.8	15.2	97.0	246
19AR110	Spot 124	53.12221	4148.909	0.580388	13.5744	4.483938	1.635598	5.020587	0.169662	0.778597	0.155081	1010.25	7.280414	984.0181	31.64626	926.8388	101.9249	926.8	101.9	109.0	107
19AR110	Spot 367	131.34	8659.808	1.683363	13.92125	0.993029	1.554958	2.120719	0.161132	0.67549	0.318519	963.0669	6.042801	952.4658	13.10606	928.9428	41.27942	928.9	41.3	103.7	319
19AR110	Spot 577	174.2301	16881.7	2.726554	14.05142	0.987826	1.398531	1.49329	0.144373	0.978649	0.655365	869.3408	7.959075	888.3143	8.841208	936.7512	23.14974	936.8	23.1	92.8	504
19AR110	Spot 300	410.2135	9102.072	1.837898	13.82545	0.705722	1.502055	1.081913	0.154889	0.669527	0.618836	928.3133	5.78852	931.2205	6.59504	938.981	17.4038	939.0	17.4	98.9	261
19AR110	Spot 200	157.7082	13075.98	1.905266	13.96593	1.654172	1.483474	2.384268	0.152822	0.985575	0.413366	916.7652	8.422338	923.6516	14.46221	941.0422	44.48569	941.0	44.5	97.4	173
19AR110	Spot 92	287.2443	8957.332	2.473727	13.91329	1.014841	1.618871	2.626705	0.16651	1.093902	0.416454	992.8528	10.0658	977.5531	16.48836	944.2244	48.91329	944.2	48.9	105.2	82
19AR110	Spot 584	349.8877	29049.24	4.552693	14.02376	0.828103	1.562113	1.282256	0.160484	0.893815	0.697065	959.4632	7.968151	955.3052	7.938302	946.6402	18.82569	946.6	18.8	101.4	511
19AR110	Spot 184	69.35364	6504.079	1.154438	13.72676	2.691439	1.53508	3.17104	0.157634	0.640595	0.202014	943.6165	5.623172	944.5349	19.49954	947.5479	63.58528	947.5	63.6	99.6	159
19AR110	Spot 27	63.74216	5889.684	2.851495	13.65567	2.093319	1.581734	3.594829	0.161987	2.064018	0.574163	967.8078	18.54859	963.0515	22.36662	953.1183	60.20476	953.1	60.2	101.5	26
19AR110	Spot 271	253.8157	8542.825	1.075014	13.82147	0.916746	1.540951	2.623446	0.157551	0.765515	0.291797	943.154	6.71666	946.8839	16.1559	956.4356	51.30764	956.4	51.3	98.6	236
19AR110	Spot 397	41.88231	4762.674	2.288132	13.46839	4.231664	1.632472	5.005312	0.166758	0.900987	0.180006	994.2212	8.301215	982.8131	31.527	958.2884	100.7067	958.3	100.7	103.7	346
19AR110	Spot 245	144.6333	10470.3	3.129475	13.78382	1.376606	1.627939	2.022127	0.166212	0.716609	0.354384	991.2088	6.583951	981.0628	12.71991	959.294	38.62445	959.3	38.6	103.3	212
19AR110	Spot 393	43.13571	2153.765	2.172355	12.82615	4.826801	1.7056	6.142911	0.174005	0.873824	0.142249	1034.139	8.348992	1010.635	39.34006	960.9389	124.3532	960.9	124.4	107.6	342
19AR110	Spot 429	57.70146	7212.07	2.307769	13.6443	2.816887	1.580835	3.119197	0.161079	0.715947	0.229529	962.7702	6.402895	962.6977	19.40224	963.4407	62.01422	963.4	62.0	99.9	377
19AR110	Spot 68	164.4357	15421.23	3.466455	13.8507	1.153154	1.589665	1.450265	0.161922	0.710794	0.490113	967.4514	6.385456	966.1661	9.03963	964.1508	25.83376	964.2	25.8	100.3	60
19AR110	Spot 274	97.8171	11039.93	3.29793	13.81946	1.435283	1.693729	1.790655	0.172366	0.780247	0.435733	1025.131	7.394992	1006.17	11.43273	966.0079	32.88654	966.0	32.9	106.1	239
19AR110	Spot 291	464.6447	5183.646	2.239163	13.45277	0.697005	1.585751	1.144734	0.161149	0.774133	0.676256	963.1581	6.925854	964.63	7.128365	968.8554	17.1888	968.9	17.2	99.4	254
19AR110	Spot 5	40.81144	5323.121	2.977471	13.46566	2.490811	1.700899	2.824031	0.172831	0.995821	0.352624	1027.689	9.459872	1008.869	18.0599	969.1294	53.91125	969.1	53.9	106.0	5
19AR110	Spot 104	180.7951	3893	2.450558	13.27823	1.374493	1.556822	3.498618	0.158066	0.709392	0.202764	946.0202	6.241802	953.2061	21.63369	970.7276	69.90745	970.7	69.9	97.5	89
19AR110	Spot 258	278.2836	27784.62	2.320152	13.89734	0.843217	1.603678	1.259618	0.162715	0.792024	0.628781	971.8473	7.145148	971.6454	7.877845	972.0584	19.96518	972.1	20.0	100.0	225
19AR110	Spot 175	46.04444	6674.848	3.359341	13.53867	2.993691	1.695559	3.625079	0.171742	1.787391	0.493063	1021.702	16.8882	1006.859	23.15724	975.5849	64.30383	975.6	64.3	104.7	152
19AR110	Spot 297	198.0821	18616.35	4.407947	13.74389	1.12655	1.593244	2.155654	0.16127	1.584722	0.735147	963.83	14.18705	967.5681	13.44848	976.9529	29.78325	977.0	29.8	98.7	258
19AR110	Spot 103	62.22038	5705.302	1.560955	13.43898	2.573635	1.777826	2.844275	0.179841	0.802607	0.282184	1066.106	7.886547	1037.385	18.4856	978.2153	55.58999	978.2	55.6	109.0	88
19AR110	Spot 415	383.6487	33334.56	2.725938	13.76552	0.768745	1.539002	1.493631	0.155504	1.265907	0.847536	931.7447	10.98225	946.1046	9.193087	980.539	16.13482	980.5	16.1	95.0	363
19AR110	Spot 78	342.275	27568.27	3.331357	13.81844	1.008105	1.627126	1.432312	0.164337	0.972545	0.679004	980.8348	8.848819	980.749	9.007809	981.4627	21.39655	981.5	21.4	99.9	69
19AR110	Spot 106	240.3558	18339.95	1.82488	13.7315	0.899781	1.471641	1.267749	0.148588	0.645391	0.509084	893.0443	5.382214	918.802	7.664569	982.0833	22.20233	982.1	22.2	90.9	91
19AR110	Spot 528	87.48486	9572.769	4.728293	13.60404	1.740724	1.72001	2.153648	0.173505	0.681052	0.316232	1031.395	6.491211	1016.028	13.82904	983.9475	41.58432	983.9	41.6	104.8	462
19AR110	Spot 401	430.6494	39064.93	2.238402	13.74586	0.692132	1.632123	1.063782	0.164458	0.736564	0.692401	981.5034	6.705942	982.6782	6.697853	986.1709	15.61158	986.2	15.6	99.5	349
19AR110	Spot 181	164.3942	18601.41	3.059072	13.71781	1.048797	1.665402	1.586097	0.167811	0.857453	0.540606	1000.036	7.942838	995.4359	10.06306	986.1801	27.15677	986.2	27.2	101.4	156
19AR110	Spot 132	45.17425	4249.714	3.101371	13.2357	4.113122	1.764969	5.396888	0.17774	0.736093	0.136392	1054.618	7.161233	1032.675	34.99388	987.3791	108.861	987.4	108.9	106.8	115
19AR110	Spot 316	646.3925	82130.09	1.778049	13.78573	0.664938	1.553517	1.157867	0.156362	0.940851	0.812572	936.5265	8.201172	951.8929	7.152749	988.4732	13.7223	988.5	13.7	94.7	275
19AR110	Spot 588	501.0918	18999.91	2.359953	13.67361	0.671947	1.582487	1.240031	0.159174	0.641637	0.517436	952.1823	5.679758	963.3477	7.715653	989.7837	21.57158	989.8	21.6	96.2	515
19AR110	Spot 491	470.133	51094.74	3.494383	13.80104	0.79195	1.618803	1.14044	0.162776	0.789838	0.692573	972.1873	7.127731	977.5268	7.15815	990.4462	16.72471	990.4	16.7	98.2	430
19AR110	Spot 462	169.2444	12680.41	6.450197	13.6417	0.91754	1.624542	1.277075	0.163344	0.683581	0.535271	975.3332	6.18733	979.7496	8.026619	990.5663	21.93965	990.6	21.9	98.5	406
19AR110	Spot 259	148.7097	14472.81	2.272297	13.67497	1.370628	1.623722	1.818286	0.163153	0.688277	0.378531	974.2755	6.223576	979.4323	11.42626	991.8844	34.22255	991.9	34.2	98.2	226
19AR110	Spot 526	50.26179	5855.487	1.663278	13.35903	3.058171	1.667813	3.67891	0.167556	0.787256	0.213992	998.6292	7.283095	996.3537	23.35703	992.231	73.09123	992.2	73.1	100.6	460
19AR110	Spot 119	104.2861	10144.82	5.310772	13.5278	1.626614	1.688558	2.043045	0.169619	0.615763	0.301395	1010.014	5.756557	1004.219	13.02951	992.4981	39.59231	992.5	39.6	101.8	103
19AR110	Spot 114	97.48543	8496.196	2.82109	13.47978	2.555088	1.726442	3.288431	0.173398	0.713487	0.216969	1030.809	6.796797	1018.427	21.14644	992.7843	65.28558	992.8	65.3	103.8	99
19AR110	Spot 151	101.1568	8897.57	3.078269	13.60314	2.356578	1.635283	2.705004	0.164164	0.923775	0.341506	979.8748	8.397464	983.8965	17.04528	993.7457	51.69974	993.7	51.7	98.6	131
19AR110	Spot 558	283.8324	35135.01	4.486059	13.71489	0.763902	1.640371	1.101948	0.164534	0.708899	0.643314	981.9229	6.456624	985.855	6.951445	995.5025	17.13748	995.5	17.1	98.6	487
19AR110	Spot 182	61.19414	7286.952	1.839528	13.43655	1.896021	1.667655	2.650797	0.167256	0.80639	0.304207	996.972	7.448658	996.2935	16.82762	995.6648	51.34195	995.7	51.3	100.1	157
19AR110	Spot 6	213.8812	24355.95	1.731267	13.66873	0.763005	1.644508	1.045486	0.16482	0.656217	0.627667	983.5085	5.985733	987.4449	6.60154	997.0683	16.53653	997.1	16.5	98.6	6
19AR110	Spot 428	191.206	13373.68	4.889019	13.57782	0.955429	1.665958	1.747453	0.166897	1.405885	0.804534	994.9912	12.96237	995.6476	11.08826	997.96	21.07696	998.0	21.1	99.7	376
19AR110	Spot 522	142.7412	14932.31	0.911161	13.60145	1.520256	1.734983	1.831415	0.173793	0.662114	0.361531	1032.975	6.319629	1021.602	11.79714	998.1812	34.67004	998.2	34.7	103.5	457
19AR110	Spot 156	99.10919	22758.98	2.843334	13.70837	1.849819	1.690237	2.182697	0.169213	1.072974	0.491582	1007.772	10.01031	1004.853	13.9254	999.3565	38.58927	999.4	38.6	100.8	135
19AR110	Spot 434	71.49015	7604.375	2.249069	13.44896	1.689676	1.730871	3.086058	0.173268	0.677753	0.219618	1030.091	6.452239	1020.075	19.86337	999.5062	61.17888	999.5	61.2	103.1	380
19AR110	Spot 516	231.4322	4698.645	2.161253	13.21998	0.823476	1.561924	1.38956	0.156306	0.875893	0.630339	936.2169	7.632609	955.2302	8.602232	1000.146	21.89895	1000.1	21.9	93.6	451
19AR110	Spot 102	104.2704	10859.03	1.850808	13.53128	1.680685	1.7306	2.272762	0.173153	0.708232	0.311617	1029.461	6.738605	1019.974	14.62691	1000.533	43.85901	1000.5	43.9	102.9	87
19AR110	Spot 546	285.1805	25000.41	1.673266	13.61517	0.852807	1.669358	1.216193	0.166958	0.836348	0.687677	995.3298	7.713618	996.9417	7.722957	1001.355	17.90922	1001.4	17.9	99.4	478
19AR110	Spot 77	199.8377	19421.98	6.355131	13.61066	1.042091	1.677385	1.456043	0.167724	0.943548	0.648022	999.559	8.736505	999.9904	9.26271	1001.802	22.51548	1001.8	22.5	99.8	68
19AR110	Spot 120	173.5391	17538.44	2.786721	13.56609	0.984847	1.683197	1.266449	0.168191	0.589405	0.465399	1002.138	5.470428	1002.192	8.066947	1003.177	22.75373	1003.2	22.8	99.9	104
19AR110	Spot 180	97.38033	8768.473	3.945916	13.44683	2.222488	1.675619	2.54297	0.167422	0.888666	0.34946	997.8887	8.215625	999.3203	16.17182	1003.328	48.38866	1003.3	48.4	99.5	155
19AR110	Spot 211	96.67065	10681.18	4.222462	13.48113	1.742131	1.628344	2.151625	0.16254	1.168522	0.543088	970.8785	10.53195	981.2195	13.53587	1005.296	36.66351	1005.3	36.7	96.6	181
19AR110	Spot 455	109.3044	10760.43	2.020826	13.50466	1.812381	1.694846	2.135867	0.16912	0.760383	0.356007	1007.262	7.090667	1006.591	13.64038	1005.994	40.50081	1006.0	40.5	100.1	399
19AR110	Spot 87	68.95949	4148.062	3.127273	13.15697	2.572472	1.727958	3.49312	0.17242	0.882399	0.252611	1025.43	8.365415	1018.991	22.47035	1006.048	68.61318	1006.0	68.6	101.9	78
19AR110	Spot 76	181.4619	17017.45	2.551517	13.55464	1.061313	1.59239	1.660748	0.158788	0.900534	0.542246	950.0394	7.954863	967.2337	10.35852	1007.379	28.30738	1007.4	28.3	94.3	67
19AR110	Spot 173	255.0442	24277.07	2.405105	13.59765	0.7493	1.722687	1.245517	0.171633	0.70967	0.569779	1021.1	6.701675	1017.027	8.001978	1009.133	20.74833	1009.1	20.7	101.2	150
19AR110	Spot 402	283.3081	26375.66	2.188934	13.55457	0.880899	1.729839	1.158267	0.172342	0.62787	0.542077	1025.002	5.95011	1019.691	7.452723	1009.17	19.73907	1009.2	19.7	101.6	350
19AR110	Spot 510	49.97306	4229.17	1.495811	13.10978	3.038713	1.760061	4.622661	0.175335	0.792446	0.171426	1041.438	7.620697	1030.87	29.94034	1009.379	92.39891	1009.4	92.4	103.2	447
19AR110	Spot 493	101.5858	7169.286	1.586113	13.34893	1.727235	1.780805	2.731922	0.177231	0.771727	0.282485	1051.829	7.489623	1038.473	17.76594	1011.329	53.15787	1011.3	53.2	104.0	432
19AR110	Spot 147	191.3153	19396.7	7.170086	13.6409	0.845914	1.695403	1.139094	0.168729	0.684729	0.601117	1005.105	6.372552	1006.801	7.27522	1011.356	18.44493	1011.4	18.4	99.4	128
19AR110	Spot 333	232.9984	11560.09	2.448779	13.48052	0.983986	1.712869	1.933927	0.170427	0.720095	0.372349	1014.466	6.759319	1013.359	12.39901	1011.831	36.40602	1011.8	36.4	100.3	290
19AR110	Spot 289	152.7762	17796.55	6.094828	13.52762	1.279624	1.675442	1.660119	0.166678	0.811048	0.488548	993.7793	7.469499	999.2532	10.55647	1012.148	29.34542	1012.1	29.3	98.2	252
19AR110	Spot 83	159.6627	18417.3	2.561308	13.57778	1.455612	1.710719	1.866543	0.170181	1.060482	0.568153	1013.107	9.942132	1012.554	11.9614	1012.222	31.11868	1012.2	31.1	100.1	74
19AR110	Spot 117	110.3203	12587.75	1.782123	13.44615	1.423614	1.753131	1.67824	0.174321	0.567818	0.338341	1035.872	5.433623	1028.318	10.85146	1013.144	32.00873	1013.1	32.0	102.2	101
19AR110	Spot 486	93.19524	11676.55	0.503623	13.47732	2.251922	1.754192	2.756805	0.174383	0.719468	0.260979	1036.217	6.88692	1028.709	17.8305	1013.639	53.94306	1013.6	53.9	102.2	426

19AR110 Spot 162	169.2428	15059.3	1.363478	13.51185	1.332217	1.703002	1.682354	0.169254	0.768494	0.456797	1007.998	7.171145	1009.659	10.76298	1014.127	30.32062	1014.1	30.3	99.4	140
19AR110 Spot 12	479.1911	73510.53	3.477786	13.63243	0.689501	1.684289	1.001956	0.167383	0.719974	0.718568	997.6785	6.654782	1002.605	6.383687	1014.255	14.12116	1014.3	14.1	98.4	12
19AR110 Spot 521	181.2808	11269.52	2.05271	13.4391	1.042381	1.720481	1.258463	0.170976	0.651839	0.517964	1017.486	6.135438	1016.204	8.08135	1014.307	21.80219	1014.3	21.8	100.3	456
19AR110 Spot 108	54.49677	6408.82	2.088946	13.24263	3.638253	1.72097	3.795588	0.170999	0.748	0.197071	1017.612	7.041362	1016.387	24.38046	1014.613	75.40833	1014.6	75.4	100.3	93
19AR110 Spot 454	341.9743	42064.77	3.131226	13.62563	0.731498	1.651994	1.030386	0.164105	0.598918	0.581256	979.5493	5.442711	990.315	6.51736	1015.107	16.98932	1015.1	17.0	96.5	398
19AR110 Spot 496	260.3983	14532.61	2.345686	13.49388	0.928027	1.671946	1.391337	0.165942	0.823555	0.591917	989.7124	7.555968	997.9257	8.840315	1016.881	22.69704	1016.9	22.7	97.3	435
19AR110 Spot 504	214.8613	17054.25	3.016544	13.51359	0.840008	1.728553	1.077264	0.171468	0.625413	0.580557	1020.197	5.901181	1019.213	6.929615	1017.962	17.76502	1018.0	17.8	100.2	441
19AR110 Spot 250	296.7075	33424.39	4.590688	13.57726	0.745632	1.682813	1.151327	0.166759	0.818347	0.710786	994.2308	7.53988	1002.047	7.332998	1020.049	16.39682	1020.0	16.4	97.5	217
19AR110 Spot 70	192.8459	17292.41	4.267098	13.48664	0.989701	1.722275	1.608869	0.170613	0.88166	0.548	1015.486	8.283568	1016.873	10.33561	1020.725	27.26462	1020.7	27.3	99.5	62
19AR110 Spot 50	141.7569	11077.32	2.69397	13.43066	1.145571	1.783225	1.570538	0.176602	0.71547	0.455558	1048.386	6.92272	1039.356	10.21764	1021.277	28.31054	1021.3	28.3	102.7	46
19AR110 Spot 477	151.7673	12206.84	12.92538	13.44817	1.234777	1.554191	2.050289	0.153901	1.362266	0.664426	922.792	11.71258	952.1607	12.66831	1021.529	31.01784	1021.5	31.0	90.3	419
19AR110 Spot 331	638.4243	32181.3	3.618734	13.53816	0.656423	1.716628	1.037482	0.169897	0.708751	0.683145	1011.542	6.635128	1014.765	6.656756	1022.589	15.33422	1022.6	15.3	98.9	288
19AR110 Spot 425	273.3299	22384.7	12.44283	13.49629	0.80271	1.7323	1.057782	0.171446	0.663534	0.627288	1020.076	6.260188	1020.606	6.809686	1022.604	16.67353	1022.6	16.7	99.8	373
19AR110 Spot 444	320.808	40644.65	4.870983	13.55189	0.671883	1.716887	0.972854	0.169917	0.698024	0.717502	1011.654	6.535377	1014.862	6.242424	1022.652	13.7141	1022.7	13.7	98.9	388
19AR110 Spot 311	66.14134	9563.713	2.302019	13.30044	3.429191	1.781108	3.859565	0.176257	0.953318	0.247001	1046.492	9.20873	1038.584	25.10321	1022.837	75.6955	1022.8	75.7	102.3	270
19AR110 Spot 131	50.32764	5222.771	3.275243	13.12076	3.834867	1.689694	4.855775	0.167173	0.779256	0.16048	996.5135	7.194957	1004.648	30.98336	1023.298	97.043	1023.3	97.0	97.4	114
19AR110 Spot 599	50.56908	4010.627	1.476879	12.95502	2.240274	1.722043	4.322608	0.170371	0.740297	0.171262	1014.156	6.946995	1016.787	27.77366	1023.319	86.21903	1023.3	86.2	99.1	525
19AR110 Spot 473	200.6278	18788.45	1.37402	13.50494	1.128963	1.707451	1.396485	0.168921	0.75771	0.542583	1006.165	7.058625	1011.329	8.942625	1023.395	23.75789	1023.4	23.8	98.3	415
19AR110 Spot 562	188.0778	19493.46	3.535482	13.47875	0.955096	1.774455	1.287514	0.175509	0.799863	0.621246	1042.393	7.698524	1036.152	8.361401	1023.869	20.41517	1023.9	20.4	101.8	490
19AR110 Spot 448	159.508	3975.995	1.826002	12.98107	1.281623	1.679677	1.80016	0.166104	0.691288	0.384015	990.6106	6.347766	1000.859	11.45784	1024.238	33.63373	1024.2	33.6	96.7	392
19AR110 Spot 423	221.3165	15129.15	4.749347	13.41615	1.131534	1.609774	3.827327	0.15919	3.634766	0.949688	952.2762	32.1781	974.0202	23.97553	1024.252	24.27352	1024.3	24.3	93.0	371
19AR110 Spot 126	553.2121	74816.36	93.3646	13.5388	0.647655	1.692718	1.024184	0.167374	0.760372	0.742417	997.6277	7.027855	1005.789	6.537438	1024.475	13.88294	1024.5	13.9	97.4	109
19AR110 Spot 337	426.1197	73734.86	3.307972	13.60033	0.539064	1.684088	0.771971	0.166509	0.540784	0.700524	992.8497	4.976142	1002.529	4.918159	1024.617	11.14617	1024.6	11.1	96.9	294
19AR110 Spot 468	115.5276	10415.8	2.338411	13.37289	0.986641	1.739937	1.901398	0.172029	0.730155	0.384009	1023.282	6.908709	1023.44	12.26075	1024.64	35.54218	1024.6	35.5	99.9	411
19AR110 Spot 284	217.5646	25508.07	1.750404	13.53594	1.042935	1.692593	1.39102	0.167341	0.798415	0.573978	997.4415	7.378206	1005.742	8.878838	1024.734	23.06571	1024.7	23.1	97.3	248
19AR110 Spot 535	177.4345	16116.8	2.28634	13.4093	1.150586	1.797917	1.813073	0.177523	0.943183	0.520212	1053.427	9.166408	1044.702	11.83041	1027.362	31.33228	1027.4	31.3	102.5	469
19AR110 Spot 174	412.1971	33140.64	6.490037	13.50337	0.805802	1.743937	1.195769	0.172084	0.781358	0.653436	1023.586	7.395224	1024.921	7.716885	1028.635	18.3217	1028.6	18.3	99.5	151
19AR110 Spot 459	63.79444	12093.68	0.907802	13.37418	3.078149	1.758481	3.405839	0.173455	1.411224	0.414354	1031.117	13.44728	1030.289	22.04906	1029.393	62.68358	1029.4	62.7	100.2	403
19AR110 Spot 44	490.311	52793.47	6.490018	13.62417	0.618619	1.639642	0.988118	0.161658	0.718503	0.727143	965.9837	6.445635	985.5748	6.2323	1030.324	13.71271	1030.3	13.7	93.8	40
19AR110 Spot 507	374.1401	36839.76	1.122566	13.52068	0.717781	1.732033	0.962313	0.170754	0.617705	0.641896	1016.264	5.807704	1020.507	6.194721	1030.479	14.93602	1030.5	14.9	98.6	444
19AR110 Spot 122	131.5497	15336.36	2.466856	13.36125	1.484707	1.779877	1.648366	0.17541	0.633061	0.384054	1041.851	6.090169	1038.135	10.71677	1031.176	30.78326	1031.2	30.8	101.0	105
19AR110 Spot 585	79.84453	12451.28	3.009384	13.32718	2.056907	1.797167	2.481445	0.176945	0.909395	0.366478	1050.261	8.813583	1044.43	16.1898	1033.113	46.64534	1033.1	46.6	101.7	512
19AR110 Spot 45	143.866	17354.21	2.841451	13.47649	1.270327	1.718812	1.725347	0.169176	0.787582	0.456478	1007.568	7.346367	1015.581	11.07574	1033.761	31.03731	1033.8	31.0	97.5	41
19AR110 Spot 503	72.28033	7015.641	1.114526	13.1943	1.714532	1.854703	2.892138	0.182489	0.773203	0.267346	1080.56	7.692227	1065.104	19.08154	1034.441	56.31216	1034.4	56.3	104.5	440
19AR110 Spot 360	335.0892	8788.446	2.134461	13.25302	0.640944	1.728675	1.034678	0.170075	0.661908	0.639724	1012.526	6.202164	1019.258	6.655842	1034.607	16.08498	1034.6	16.1	97.9	312
19AR110 Spot 134	318.9559	27959.17	2.00028	13.48338	0.830604	1.704378	1.143444	0.167673	0.712548	0.62316	999.2785	6.595917	1010.176	7.317299	1034.746	18.08587	1034.7	18.1	96.6	117
19AR110 Spot 223	104.8609	7241.761	1.49611	13.18117	1.610345	1.829885	1.939605	0.179958	0.815746	0.420573	1066.747	8.02008	1056.238	12.73565	1035.442	35.56653	1035.4	35.6	103.0	192
19AR110 Spot 586	270.4066	28815.59	2.751978	13.42722	0.792719	1.7318	1.145746	0.170302	0.708648	0.618503	1013.777	6.6477	1020.42	7.375214	1035.557	18.20407	1035.6	18.2	97.9	513
19AR110 Spot 196	94.90345	12493.04	3.822432	13.31697	2.018384	1.83909	2.376653	0.180767	0.837029	0.352188	1071.165	8.260656	1059.536	15.63341	1036.517	44.93302	1036.5	44.9	103.3	169
19AR110 Spot 36	345.2549	32425.37	4.740862	13.45625	0.707878	1.736299	1.140534	0.170584	0.670977	0.588301	1015.329	6.303214	1022.091	7.348632	1037.459	18.64335	1037.5	18.6	97.9	34
19AR110 Spot 158	179.6885	21069.53	2.431686	13.38769	0.768705	1.781609	1.226599	0.174919	0.778661	0.634813	1039.154	7.472996	1038.767	7.977333	1038.812	19.15316	1038.8	19.2	100.0	137
19AR110 Spot 17	131.1787	11643.26	1.686232	13.27612	1.168596	1.797896	1.899442	0.176406	0.723291	0.380791	1047.313	6.991786	1044.695	12.39397	1040.084	35.47407	1040.1	35.5	100.7	16
19AR110 Spot 90	300.9131	29562.09	8.623217	13.4814	0.801186	1.689939	1.332481	0.165763	1.037117	0.778335	988.7233	9.506559	1004.74	8.500212	1040.704	16.9045	1040.7	16.9	95.0	81
19AR110 Spot 279	325.5365	55917.83	4.705852	13.50902	0.757599	1.697292	1.095603	0.166404	0.745207	0.68018	992.2688	6.853472	1007.512	7.000333	1041.673	16.22689	1041.7	16.2	95.3	244
19AR110 Spot 169	640.2112	55860.09	2.110278	13.44493	0.672367	1.729831	0.997259	0.169549	0.721576	0.72356	1009.625	6.743369	1019.688	6.416694	1042.214	13.90912	1042.2	13.9	96.9	146
19AR110 Spot 136	129.6686	9578.269	1.875385	13.25384	1.303065	1.730017	2.051604	0.169485	0.670686	0.326908	1009.273	6.265763	1019.757	13.20179	1043.19	39.13145	1043.2	39.1	96.7	119
19AR110 Spot 148	76.35468	7510.712	2.051068	13.21995	1.756867	1.773316	2.68358	0.173671	0.707052	0.263473	1032.306	6.744515	1035.735	17.42505	1043.839	52.23405	1043.8	52.2	98.9	129
19AR110 Spot 110	164.3665	16893.67	2.78564	13.29631	0.997314	1.788653	1.65581	0.175156	0.699742	0.422598	1040.46	6.723365	1041.335	10.78421	1044.031	30.29427	1044.0	30.3	99.7	95
19AR110 Spot 341	67.10789	6791.266	2.353669	13.14184	2.163022	1.741187	2.85593	0.170456	0.744321	0.260623	1014.622	6.987718	1023.903	18.42179	1044.651	55.62821	1044.7	55.6	97.1	297
19AR110 Spot 570	298.0581	25743.44	2.586745	13.37461	0.666719	1.779727	1.019124	0.174081	0.696111	0.683048	1034.556	6.65349	1038.08	6.625435	1046.404	15.03063	1046.4	15.0	98.9	497
19AR110 Spot 304	374.8678	37057.21	2.32249	13.32984	0.794362	1.779413	1.221176	0.17396	0.898654	0.735892	1033.891	8.584332	1037.965	7.938544	1047.451	16.69127	1047.5	16.7	98.7	264
19AR110 Spot 364	105.8097	11892.5	2.187771	13.22604	1.724724	1.787543	1.933042	0.174703	0.698946	0.361578	1037.973	6.700922	1040.931	12.58718	1048.042	36.35745	1048.0	36.4	99.0	316
19AR110 Spot 354	575.7128	51177.47	4.527805	13.33288	0.604285	1.807224	1.123062	0.175565	0.932087	0.829951	1042.7	8.973589	1048.075	7.341353	1060.188	12.60819	1060.2	12.6	98.4	307
19AR110 Spot 225	148.3173	4742.405	3.390125	12.84804	1.175391	1.945339	1.839152	0.18894	0.689505	0.374904	1115.628	7.063481	1096.841	12.33471	1060.642	34.33104	1060.6	34.3	105.2	194
19AR110 Spot 375	49.08853	6177.278	3.373994	12.9551	2.833612	1.783869	4.128103	0.173226	0.823871	0.199576	1029.863	7.841696	1039.592	26.86556	1060.997	81.43461	1061.0	81.4	97.1	326
19AR110 Spot 419	177.4556	18222.86	4.435558	13.19472	0.891654	1.829196	1.274843	0.177616	0.691942	0.542767	1053.936	6.727691	1055.991	8.369386	1061.135	21.5639	1061.1	21.6	99.3	367
19AR110 Spot 26	476.2941	53049.94	2.591438	13.32048	0.682432	1.818077	1.020556	0.176238	0.712509	0.698157	1046.391	6.881982	1051.992	6.68548	1064.532	14.69275	1064.5	14.7	98.3	25
19AR110 Spot 474	308.2338	38940.62	2.831599	13.30122	0.964852	1.807311	1.270953	0.175083	0.792653	0.623669	1040.058	7.613385	1048.106	8.308284	1065.81	19.96667	1065.8	20.0	97.6	416
19AR110 Spot 342	124.5011	13659.38	2.162098	13.17921	1.172522	1.829397	1.323472	0.177086	0.549503	0.415198	1051.034	5.329224	1056.063	8.688991	1067.366	24.20489	1067.4	24.2	98.5	298
19AR110 Spot 149	245.2844	23048.31	2.087005	13.35208	0.733837	1.795868	0.993068	0.173811	0.649235	0.653767	1033.075	6.197254	1043.958	6.476981	1067.704	15.09493	1067.7	15.1	96.8	130
19AR110 Spot 128	304.1952	47579.96	4.362694	13.24454	0.85386	1.825179	1.125805	0.176624	0.685198	0.608629	1048.503	6.630493	1054.548	7.385165	1067.979	17.9546	1068.0	18.0	98.2	111
19AR110 Spot 321	156.512	16399.54	3.297284	13.14011	1.202167	1.82027	1.545378	0.176114	0.775408	0.50176	1045.711	7.485026	1052.782	10.12801	1068.372	26.88824	1068.4	26.9	97.9	279
19AR110 Spot 411	297.826	23612.83	2.130837	13.12981	0.805712	1.854655	1.100114	0.179114	0.65267	0.593274	1062.134	6.391254	1065.087	7.25746	1072.033	17.77663	1072.0	17.8	99.1	359
19AR110 Spot 349	178.9189	18999.26	2.726223	13.17077	1.049001	1.894445	1.336255	0.18293	0.777451	0.581813	1082.961	7.750271	1079.142	8.880696	1072.333	21.83216	1072.3	21.8	101.0	302
19AR110 Spot 461	241.2141	23274.5	2.424943	13.16757	0.855486	1.826142	1.119605	0.175969	0.652503	0.582798	1044.917	6.29422	1054.894	7.345862	1076.496	18.26454	1076.5	18.3	97.1	405
19AR110 Spot 319	220.3461	32460.15	1.554625	13.13081	0.782795	1.910669	1.147607	0.183682	0.74311	0.64753	1087.06	7.433682	1084.818	7.649342	1081.195	17.52577	1081.2	17.5	100.5	277
19AR110 Spot 370	36.14212	5381.446	0.650503	12.7711	2.699639	1.789208	3.244794	0.171974	0.954143	0.294054	1022.976	9.025601	1041.537	21.1378	1081.562	62.22671	1081.6	62.2	94.6	321
19AR110 Spot 398	227.8578	23147.78	2.436814	13.09458	0.8936	1.882109	1.217034	0.180832	0.712111	0.58512	1071.521	7.029985	1074.806	8.070051	1082.334	19.79647	1082.3	19.8	99.0	347
19AR110 Spot 587	62.08357	6153.814	0.721482	12.80921	1.358428	1.824383	1.71141	0.175189	0.723551	0.422781	1040.638	6.953232	1054.262	11.2252	1083.441	31.08534	1083.4	31.1	96.0	514
19AR110 Spot 325	141.5255	19533.38	3.356395	13.08078	0.880541	1.906365	1.248987	0.182982	0.703043	0.562891	1083.243	7.010193	1083.315	8.318661	1084.352	20.68138	1084.4	20.7	99.9	283
19AR110 Spot 220	106.5511	9418.332	1.935912	12.94172	1.800178	1.909489	2.264246	0.18314	0.780311	0.344623	1084.105	7.786334	1084.406	15.08989	1085.887	42.61914	1085.9	42.6	99.8	190
19AR110 Spot 22	96.43746	6499.188	1.153291	12.82735	1.497251	1.980659	2.135764	0.189932	0.836137	0.391493	1121.006	8.603436	1108.945	14.41152	1086.26	39.38944	1086.3	39.4	103.2	21
19AR110 Spot 29	262.8557	24813.21	1.558388	13.13851	0.743513	1.899695	1.122593	0.182091	0.638431	0.568711	1078.387	6.339715	1080.982	7.467782	1087.085	18.50359	1087.1	18.5	99.2	28
19AR110 Spot 361	226.5298	21305.97	3.040469	13.07873	0.657362	1.959166	0.900595	0.187773	0.592367	0.657751	1109.298	6.036824	1101.597	6.054337	1087.269	13.57803	1087.3	13.6	102.0	313
19AR110 Spot 594	393.4785	39065.25	173.039	13.09156	0.729998	1.860194	1.025365	0.178242	0.631899	0.616268	1057.364	6.162289	1067.055	6.771388	1087.811	16.17031	1087.8	16.2	97.2	521
19AR110 Spot 85	30.20206	4892.574	1.133012	12.73426	4.74105	1.758069	5.276405	0.168336	1.070263	0.20284	1002.936	9.940734	1030.137	34.16352	1089.247	103.5811	1089.2	103.6	92.1	76
19AR110 Spot 170	357.3791	22908.26	2.55375	13.01579	0.684657	1.899612	1.282004	0.181089	0.795496	0.620509	1072.922	7.862596	1080.953	8.528146	1098.031	20.13538	1098.0	20.1	97.7	147
19AR110 Spot 39	351.9369	50891.88	5.035847	13.11405	0.788839	1.950036	1.067213	0.185518	0.71356	0.66862	1097.049	7.198244	1098.459	7.163144	1102.143	15.87062	1102.1	15.9	99.5	37
19AR110 Spot 276	374.3808	43430.68	3.702236	13.08518	0.687145	1.936504	1.046582	0.184054	0.700145	0.668983	1089.086	7.015865	1093.791	7.008062	1104.024	15.57001	1104.0	15.6	98.6	241
19AR110 Spot 210	291.1031	15208.58	4.853089	12.90063	0.715035	1.941807	1.096485	0.184366	0.787897	0.718566	1090.784	7.906486	1095.623	7.349066	1106.13	15.25931	1106.1	15.3	98.6	180
19AR110 Spot 410	173.7804	14582.32	1.290311	12.80073	0.816807	1.979159	1.138461	0.187205	0.696486	0.611779	1106.214	7.07981	1108.434	7.67968	1113.642	17.97984	1113.6	18.0	99.3	358
19AR110 Spot 172	372.3029	40213.84	3.038649	12.89623	0.724642	2.004502	1.008725	0.188665	0.676197	0.670348	1114.137	6.918671	1117.035	6.833502	1123.547	14.94039	1123.5	14.9	99.2	149
19AR110 Spot 135	276.4866	29467.01	1.505452	12.89673	0.885629	2.041041	1.097039	0.191929	0.614269	0.559933	1131.817	6.376279	1129.309	7.476345	1125.371	18.126	1125.4	18.1	100.6	118
19AR110 Spot 323	254.4826	32553.1	3.143801	12.85393	0.728681	1.95721	1.002793	0.18402	0.640463	0.638679	1088.899	6.416801	1100.925	6.739117	1125.657	15.38799	1125.7	15.4	96.7	281
19AR110 Spot 509	58.57297	6901.384	1.131614	12.56117	2.537043	2.107093	3.47287	0.197329	0.678733	0.195439	1160.957	7.210998	1151.127	23.91818	1133.509	67.80518	1133.5	67.8	102.4	446
19AR110 Spot 11	366.4969	26052.91	2.476228	12.79527	0.622591	2.085137	1.174381	0.195235	0.786828	0.669994	1149.673	8.285198	1143.927	8.059499	1133.895	17.34277	1133.9	17.3	101.4	11
19AR110 Spot 59	216.9829	25538.72	2.762407	12.79095	0.795012	2.106126	1.115169	0.196839	0.727831	0.652664	1158.316	7.716566	1150.811	7.677932	1137.584	16.7899	1137.6	16.8	101.8	52
19AR110 Spot 269	331.882	51900.41	3.661671	12.88078	0.745659	1.873718	1.130647	0.175027	0.810239	0.716616	1039.748	7.780156	1071.845	7.48557	1138.621	15.67667	1138.6	15.7	91.3	235
19AR110 Spot 222	203.0761	30204.29	2.251213	12.72932	0.921225	2.156428	1.194079	0.20066	0.729466	0.610903	1178.866	7.858948	1167.123	8.283456	1146.262	18.78368	1146.3	18.8	102.8	191
19AR110 Spot 246	72.12342	11294.49	1.161706	12.58354	2.053055	2.218303	2.479515	0.206129	0.705678	0.284603	1208.163	7.77447	1186.835	17.35533	1149.062	47.19669	1149.1	47.2	105.1	213
19AR110 Spot 359	124.0144	11840.14	2.574558	12.57755	1.144938	2.117813	1.508928	0.196451	0.651232	0.431586	1156.226	6.893072	1154.624	10.40762	1152.467	27.02172	1152.5	27.0	100.3	311
19AR110 Spot 550	360.8194	27778.41	26.52563	12.58934	0.821546	1.990718	1.200852	0.183711	0.743538	0.619176	1087.218	7.438954	1112.366	8.116388	1162.696	18.70973	1162.7	18.7	93.5	481
19AR110 Spot 578	365.1223	41647.37	4.170584	12.6502	0.806859	2.162894	1.167869	0.199585	0.831478	0.711961	1173.091	8.91797	1169.201	8.109312	1162.848	16.27044	1162.8	16.3	100.9	505
19AR110 Spot 161	314.658	31362.15	2.824272	12.52223	0.684489	2.167935	1.011549	0.198408	0.737071	0.728656	1166.765	7.866527	1170.818	7.029001	1179.199	13.69924	1179.2	13.7	98.9	139
19AR110 Spot 60	223.8923	24730.57	2.357392	12.48221	0.743426	2.254	1.218553	0.205582	0.940601	0.7719	1205.238	10.33981	1198.035	8.570794	1185.944	15.29859	1185.9	15.3	101.6	53
19AR110 Spot 559	536.3701	27069.16	0.907143	12.44612	0.644139	2.044606	1.025361	0.186282	0.768944	0.749925	1101.202	7.78388	1130.499	6.991851	1188.086	13.38557	1188.1	13.4	92.7	488
19AR110 Spot 489	39.64472	4989.9	1.166705	12.10637	2.546162	2.320264	2.960721	0.2111	0.865586	0.292357	1234.677	9.726075	1218.505	21.01136	1190.839	55.90503	1190.8	55.9	103.7	428
19AR110 Spot 438	67.00177	10204.47	1.728154	12.31683	2.110193	2.27224	2.798434	0.20672	0.66722	0.238426	1211.317	7.368219	1203.711	19.73371	1190.94	53.63858	1190.9	53.6	101.7	383
19AR110 Spot 71	115.1386	11064.57	2.572082	12.28656	1.236276	2.314853	1.568067	0.209878	0.837285	0.53396	1228.17	9.363066	1216.848	11.11913	1197.704	26.12826	1197.7	26.1	102.5	63
19AR110 Spot 257	545.1639	90316.78	1.586083	12.32555	0.589787	2.281467	0.895931	0.204401	0.663741	0.740839	1198.919	7.261538	1206.57	6.324938	1221.157	11.83763	1221.2	11.8	98.2	224
19AR110 Spot 394	677.5635	98391.07	2.50893	11.88757	0.623025	2.538588	0.967688	0.219819	0.728514	0.75284	1280.918	8.463031	1283.168	7.04911	1287.766	12.39999	1287.8	12.4	99.5	343
19AR110 Spot 598	104.6773	16096.6	1.331577	11.70647	1.200754	2.591937	1.645984	0.223303	0.745754	0.453075	1299.305	8.775561	1298.362	12.06068	1297.632	28.52736	1297.6	28.5	100.1	524
19AR110 Spot 283	273.0886	15118.73	1.539049	11.70083	0.753573	2.066384	1.164173	0.177118	0.740475	0.636052	1051.209	7.182414	1137.736	7.966008	1307.561	17.43894	1307.6	17.4	80.4	247
19AR110 Spot 495	75.53429	10062.37	1.040872	11.59706	1.560894	2.693841	1.843981	0.230468	0.835032	0.452842	1336.954	10.08237	1326.767	13.65546	1311.186	31.9035	1311.2	31.9	102.0	434
19AR110 Spot 208	60.63375	9163.499	1.819197	11.32387	2.029097	2.831493	2.316381	0.237242	0.756213	0.326463	1372.345	9.347604	1363.918	17.38321	1351.561	42.24852	1351.6	42.2	101.5	178
19AR110 Spot 445	112.9963	16923.13	1.592379	11.41917	0.828421	2.789896	1.117089	0.233629	0.668877	0.598767	1353.492	8.165954	1352.834	8.350035	1352.617	17.2606	1352.6	17.3	100.1	389
19AR110 Spot 54	240.2499	30356.85	3.439602	11.47525	0.757643	2.311155	1.644317	0.193433	1.45565	0.885262	1139.945	15.20927	1215.715	11.65424	1353.672	14.75201	1353.7	14.8	84.2	49
19AR110 Spot 571	120.8064	19284	2.596787	11.01979	1.107731	3.026868	1.338245	0.244081	0.622345	0.465046	1407.878	7.871091	1414.417	10.21424	1425.093	22.62812	1425.1	22.6	98.8	498
19AR110 Spot 569	497.1512	6694.655	3.288233	10.36912	0.578777	3.313555	0.951533	0.254888	0.726527	0.763533	1463.637	9.512954	1484.249	7.421992	1514.635	11.5942	1514.6	11.6	96.6	496
19AR110 Spot 112	119.1273	6656.348	1.513359	9.91492	0.819335	3.848346	1.881456	0.284001	0.779256	0.414177	1611.479	11.111	1602.922	15.1648	1592.489	31.98645	1592.5	32.0	101.2	97
19AR110 Spot 381	405.5109	45892.52	2.411136	9.459921	0.597141	3.514891	1.332629	0.24215	1.183636	0.888197	1397.866	14.87469	1530.569	10.53463	1719.908	11.25223	1719.9	11.3	81.3	332
19AR110 Spot 527	275.8278	37519.54	2.577895	9.422254	0.610488	4.126733	0.901777	0.283883	0.655947	0.727394	1610.89	9.349789	1659.612	7.370596	1722.613	11.36824	1722.6	11.4	93.5	461
19AR110 Spot 374	119.4119	38910.96	1.479659	9.260112	0.808748	4.525802	1.07412	0.305772	0.696689	0.648614	1719.867	10.51691	1735.725	8.932933	1755.668	14.95524	1755.7	15.0	98.0	325
19AR110 Spot 96	99.16146	4948.947	0.971245	8.99389	0.590078	4.197632	1.265458	0.280769	0.66902	0.528678	1595.234	9.454449	1673.557	10.37747	1774.002	19.60568	1774.0	19.6	89.9	83
19AR110 Spot 306	86.69964	12429.23	0.915081	8.980263	0.791531	4.687009	1.118335	0.310156	0.75462	0.674771	1741.474	11.51606	1764.923	9.358922	1793.564	15.02783	1793.6	15.0	97.1	266
19AR110 Spot 372	495.5897	102812.7	2.529753	9.051355	0.562616	4.864003	0.93179	0.320569	0.738863	0.79295	1792.509	11.56227	1796.043	7.847952	1800.924	10.32873	1800.9	10.3	99.5	323
19AR110 Spot 281	173.7677	34750.63	2.038536	8.954357	0.686248	4.271522	1.269136	0.277604	1.046492	0.824571	1579.286	14.65835	1687.891	10.44239	1826.368	13.02075	1826.4	13.0	86.5	245
19AR110 Spot 16	95.80549	14244.19	1.352488	8.735884	0.886423	5.140692	1.158805	0.329242	0.678772	0.585751	1834.707	10.83811	1842.857	9.850499	1852.844	16.97757	1852.8	17.0	99.0	15
19AR110 Spot 218	438.3074	90245.55	2.691375	8.794336	0.706449	5.103772	1.077906	0.326586	0.811203	0.752573	1821.812	12.87391	1836.733	9.151989	1854.454	12.82787	1854.5	12.8	98.2	188
19AR110 Spot 547	231.1069	40319.83	1.050085	8.721316	0.659044	5.286084	0.981293	0.337005	0.673586	0.686428	1872.247	10.945	1866.618	8.378999	1861.124	12.8859	1861.1	12.9	100.6	479
19AR110 Spot 273	86.43646	16452.99	1.496667	8.742989	0.781115	5.255823	1.142141	0.334436	0.781435	0.684184	1859.848	12.62487	1861.718	9.743596	1864.575	15.03577	1864.6	15.0	99.7	238
19AR110 Spot 350	228.6528	44762.15	1.04516	8.731548	0.727029	5.091545	1.170778	0.323496	0.896335	0.765589	1806.781	14.12329	1834.697	9.93666	1867.289	13.59106	1867.3	13.6	96.8	303
19AR110 Spot 420	280.8236	54978.63	1.712301	8.70037	0.575771	5.229152	0.851503	0.332069	0.625804	0.734941	1848.4	10.05676	1857.38	7.25815	1868.217	10.41969	1868.2	10.4	98.9	368
19AR110 Spot 89	158.1647	31530.8	0.857029	8.703531	0.696347	5.20978	1.024266	0.328993	0.715231	0.698286	1833.501	11.41378	1854.217	8.725627	1878.302	13.21234	1878.3	13.2	97.6	80
19AR110 Spot 573	386.1863	83811.28	1.71204	8.642421	0.6003	5.231643	0.871251	0.328854	0.628543	0.721426	1832.826	10.02719	1857.785	7.427049	1886.607	10.8611	1886.6	10.9	97.1	500
19AR110 Spot 265	458.4106	75840.31	0.842114	8.668265	0.608733	5.31269	0.936242	0.333315	0.708135	0.756359	1854.428	11.41187	1870.906	8.000685	1890.028	11.02073	1890.0	11.0	98.1	231
19AR110 Spot 195	530.9946	78286.02	3.176063	8.414864	0.536386	4.932813	0.918873	0.302168	0.741939	0.807445	1702.052	11.09861	1807.888	7.757609	1932.95	9.704595	1932.9	9.7	88.1	168
19AR110 Spot 567	111.3844	18915.37	1.96365	8.308885	0.800655	5.43899	1.170935	0.330175	0.815104	0.696114	1839.232	13.0427	1891.021	10.04332	1949.128	15.02211	1949.1	15.0	94.4	495
19AR110 Spot 187	165.6788	36716.34	1.945174	8.293185	0.613505	5.492421	0.930554	0.331484	0.664501	0.714092	1845.572	10.66452	1899.412	7.993512	1959.522	11.62618	1959.5	11.6	94.2	161
19AR110 Spot 365	349.1106	43207.42	0.543469	7.88219	0.514032	6.366565	0.877295	0.365846	0.709354	0.80857	2009.823	12.24836	2027.671	7.698814	2046.638	9.1218	2046.6	9.1	98.2	317
19AR110 Spot 390	64.7782	10786.31	1.605192	7.543879	1.229266	6.945561	1.505156	0.3849	0.819639	0.544554	2099.131	14.6849	2104.497	13.36039	2110.497	22.14583	2110.5	22.1	99.5	340
19AR110 Spot 178	188.2698	44452.18	1.153188	7.268508	0.740553	7.291008	1.067385	0.385753	0.760032	0.712051	2103.102	13.63875	2147.709	9.531125	2191.387	13.02836	2191.4	13.0	96.0	154
19AR110 Spot 98	93.42937	38807.13	2.002236	6.056276	0.564419	10.71342	0.859878	0.471522	0.641077	0.745544	2490.233	13.24233	2498.589	7.985833	2506.109	9.639059	2506.1	9.6	99.4	85
19AR110 Spot 487	157.0303	45343.9	1.612211	5.950393	0.604612	11.11775	0.914239	0.480727	0.683896	0.74805	2530.43	14.31306	2533.047	8.517157	2535.861	10.17823	2535.9	10.2	99.8	427
19AR110 Spot 213	195.0886	44397.39	1.287874	5.530206	0.579776	12.41956	0.909111	0.49971	0.697234	0.76694	2612.55	14.97642	2636.658	8.543286	2655.923	9.674049	2655.9	9.7	98.4	183
19AR110 Spot 580	108.867	32941.75	2.136815	5.525262	0.777947	12.61504	1.107721	0.507418	0.787349	0.710783	2645.596	17.08514	2651.343	10.42186	2656.438	12.92017	2656.4	12.9	99.6	507
19AR110 Spot 113	88.26665	27412.95	2.445036	5.467001	0.627952	12.73238	0.958482	0.508149	0.683405	0.713007	2648.721	14.84375	2660.056	9.023796	2669.395	11.12974	2669.4	11.1	99.2	98
19AR110 Spot 238	376.8944	125288.8	2.981757	4.407957	0.701409	18.08719	1.036219	0.579786	0.762015	0.735381	2947.878	18.02821	2994.382	9.97067	3026.447	11.26412	3026.4	11.3	97.4	206
19AR110 Spot 421	46.80203	15544.32	2.943238	3.63726	0.563289	25.19398	0.845927	0.669027	0.623773	0.737384	3302.117	16.11855	3315.763	8.261668	3324.699	8.948837	3324.7	8.9	99.3	369

**19AR111**																				-26.94385	-65.76501	3030	30.3	0.894
_19AR111_Spot_ 492	671.2792	22.40117	0.857906	3.446807	17.92459	-0.03025	69.05377	8.73E-05	6.258221	0.090628	0.562662	0.035211	NA	-21.8738	NA	NA	0.6	0.0		425					
_19AR111_Spot_ 305	3408.445	180.8744	9.150176	9.196611	21.31472	0.000487	126.1787	0.000143	4.287451	0.033979	0.921468	0.039505	0.494602	0.623931	NA	NA	0.9	0.0		270					
_19AR111_Spot_ 75	1002.836	239.7986	4.764222	4.878505	46.36764	0.005919	48.91911	0.000284	9.784116	0.200006	1.827805	0.178809	5.992114	2.922665	2362.259	877.277	1.8	0.2	0.1	64					
_19AR111_Spot_ 249	951.6013	176.8361	3.598655	3.735916	34.36991	0.00846	39.75555	0.000307	6.570866	0.165282	1.978194	0.129964	8.554024	3.386428	2826.231	670.2539	2.0	0.1	0.1	223					
_19AR111_Spot_ 82	893.7745	137.4667	3.609008	5.325926	31.79933	0.003677	47.27951	0.000312	8.69925	0.183996	2.011406	0.17495	3.726715	1.758745	1326.732	954.5209	2.0	0.2	0.2	71					
_19AR111_Spot_ 531	1762.803	314.9672	2.80586	10.01147	15.83998	0.002348	34.09785	0.00032	8.889993	0.26072	2.062561	0.183332	2.381683	0.811151	339.3486	NA	2.1	0.2	0.6	458					
_19AR111_Spot_ 372	1148.116	298.2567	4.546103	6.458338	8.361846	0.004846	16.99629	0.000322	6.1131	0.359673	2.076256	0.126903	4.908705	0.832284	1785.337	290.9437	2.1	0.1	0.1	325					
_19AR111_Spot_ 62	733.0777	160.8267	4.089106	6.085846	36.48155	0.003386	84.56045	0.000325	9.029934	0.106787	2.093323	0.188995	3.432069	2.897282	1085.583	NA	2.1	0.2	0.2	54					
_19AR111_Spot_ 235	1445.663	294.2739	2.157758	9.480913	25.8438	0.002521	48.05684	0.000328	4.492262	0.093478	2.114463	0.094972	2.556903	1.227221	443.0236	NA	2.1	0.1	0.5	210					
_19AR111_Spot_ 162	1427.535	263.2203	2.269931	10.44077	5.987225	0.001772	88.95379	0.00033	5.221266	0.058696	2.125472	0.110958	1.797194	1.597259	NA	NA	2.1	0.1		143					
_19AR111_Spot_ 91	1925.028	418.3772	3.45453	9.067293	18.87089	0.003506	32.94483	0.000333	3.364388	0.102122	2.147294	0.072231	3.553911	1.168784	1104.582	673.1586	2.1	0.1	0.2	78					
_19AR111_Spot_ 368	1192.885	276.0355	1.345965	10.50502	17.1501	0.001902	110.7199	0.000335	4.853884	0.043839	2.159503	0.104802	1.929172	2.133952	NA	NA	2.2	0.1		323					

_19AR111_Spot_ 26	1453.432	202.7198	3.264927	8.203212	13.57846	0.002314	124.3514	0.00034	4.010533	0.032252	2.193551	0.087958	2.346665	2.914749	163.5033	NA	2.2	0.1	1.3	21
_19AR111_Spot_ 125	1561.728	367.7558	1.117709	11.80457	10.55748	0.002105	77.2938	0.000345	4.312046	0.055788	2.223725	0.095872	2.134778	1.648319	NA	NA	2.2	0.1		110
_19AR111_Spot_ 69	2436.835	525.4714	1.824572	6.848659	17.53592	0.00574	25.45937	0.000346	2.893523	0.113653	2.227502	0.064442	5.81181	1.475425	1963.83	459.0732	2.2	0.1	0.1	60
_19AR111_Spot_ 128	2732.118	69.31961	0.855441	7.754082	15.18478	-0.00634	60.02322	0.000346	2.039623	0.033981	2.232353	0.045524	NA	-3.88917	NA	NA	2.2	0.0		112
_19AR111_Spot_ 545	2601.239	798.4816	7.406112	12.06183	12.01582	0.003109	15.00953	0.000349	3.548524	0.236418	2.246852	0.079716	3.151998	0.472367	764.9728	308.8112	2.2	0.1	0.3	471
_19AR111_Spot_ 306	1269.765	344.8174	2.495176	5.548562	43.58123	0.006944	46.18661	0.000352	8.300384	0.179714	2.271267	0.188491	7.026302	3.23402	2263.544	833.3216	2.3	0.2	0.1	271
_19AR111_Spot_ 390	1294.854	444.195	3.025935	10.96562	17.05167	0.003013	33.22853	0.000375	9.992945	0.300734	2.418147	0.241599	3.055108	1.013642	540.1858	NA	2.4	0.2	0.4	340
_19AR111_Spot_ 438	1173.561	90.18651	0.616621	6.960578	11.53394	-0.00197	367.267	0.000375	8.026929	0.021856	2.419082	0.194142	NA	-7.35078	NA	NA	2.4	0.2		381
_19AR111_Spot_ 16	2737.456	477.9052	3.470841	13.03143	13.54642	0.002387	33.78213	0.000378	3.488547	0.103266	2.435117	0.084934	2.420866	0.816846	NA	NA	2.4	0.1		13
_19AR111_Spot_ 126	2174.342	596.4581	3.104586	9.486089	20.5139	0.004247	21.99148	0.000378	2.665158	0.12119	2.436174	0.064916	4.30312	0.944318	1234.143	433.3252	2.4	0.1	0.2	111
_19AR111_Spot_ 96	2676.496	640.6838	4.947262	10.26088	15.56696	0.003937	17.65286	0.000379	2.934441	0.16623	2.445021	0.071734	3.989896	0.702949	1076.684	352.0194	2.4	0.1	0.2	82
_19AR111_Spot_ 600	1070.687	277.969	4.442146	9.166482	15.95187	0.002966	76.12424	0.000381	7.467087	0.098091	2.45536	0.183309	3.007444	2.286011	471.9001	NA	2.5	0.2	0.5	512
_19AR111_Spot_ 132	2654.087	705.1644	4.372328	13.35404	11.92233	0.002888	14.01906	0.000388	1.902645	0.135719	2.500712	0.04757	2.928672	0.409981	371.7098	314.0913	2.5	0.0	0.7	116
_19AR111_Spot_ 533	1571.997	451.0237	6.159668	10.80573	15.27767	0.003264	23.05496	0.000393	4.443024	0.192715	2.535919	0.112649	3.308693	0.761576	610.3674	494.7727	2.5	0.1	0.4	460
_19AR111_Spot_ 256	1240.594	354.1296	1.257769	5.986044	16.37839	0.007146	21.86984	0.000401	3.44756	0.15764	2.582777	0.089025	7.229632	1.575495	2089.538	384.5436	2.6	0.1	0.1	229
_19AR111_Spot_ 453	1731.941	594.9495	4.087599	9.824447	14.38593	0.004799	24.03904	0.000446	5.138622	0.213762	2.873468	0.147624	4.861138	1.165778	1149.415	472.7678	2.9	0.1	0.2	395
_19AR111_Spot_ 85	358.2744	43.4143	1.369911	3.821781	32.30808	-0.01106	232.4344	0.000459	9.366905	0.040299	2.958424	0.277049	NA	-26.3957	NA	NA	3.0	0.3		73
_19AR111_Spot_ 450	688.4461	242.3886	1.422156	6.335885	41.45018	0.007187	51.64329	0.000515	7.030655	0.136139	3.31919	0.233301	7.271338	3.741761	1647.171	1023.759	3.3	0.2	0.2	393
_19AR111_Spot_ 54	1305.513	403.5135	3.785725	8.357267	13.71278	0.006052	38.41811	0.000519	4.008304	0.104334	3.344574	0.134026	6.126334	2.34654	1306.653	770.8584	3.3	0.1	0.3	47
_19AR111_Spot_ 447	555.4197	256.7883	0.674686	6.306803	13.68547	0.007583	41.20915	0.000521	9.936891	0.241133	3.355281	0.333324	7.671034	3.149267	1726.315	767.4906	3.4	0.3	0.2	390
_19AR111_Spot_ 18	428.6773	126.8871	2.067009	4.547083	13.54071	0.010273	33.1315	0.000657	8.474697	0.25579	4.232421	0.358567	10.37744	3.420704	1856.011	595.1248	4.2	0.4	0.2	15
_19AR111_Spot_ 544	647.5628	231.3504	6.388012	5.036255	33.99951	0.013417	40.51462	0.000685	7.034725	0.173634	4.417213	0.310632	13.53322	5.446609	2252.081	721.6057	4.4	0.3	0.2	470
_19AR111_Spot_ 28	702.9818	362.4693	3.074396	8.593327	20.64582	0.00845	44.82293	0.000793	3.757392	0.083827	5.108525	0.191871	8.543976	3.813611	1129.927	937.9581	5.1	0.2	0.5	23
_19AR111_Spot_ 517	908.2559	583.4107	2.164923	8.092261	12.09054	0.010987	13.9332	0.000798	3.154047	0.226369	5.139377	0.162034	11.09537	1.537526	1623.248	253.6636	5.1	0.2	0.3	444
_19AR111_Spot_ 293	373.0846	262.4335	3.365547	8.199311	19.78652	0.011207	34.07803	0.001216	5.275439	0.154805	7.833888	0.413021	11.31622	3.834954	833.877	721.3727	7.8	0.4	0.9	260
_19AR111_Spot_ 566	666.133	575.223	4.606901	11.04091	17.27409	0.012057	23.89292	0.001335	2.229137	0.093297	8.600496	0.191589	12.16934	2.890264	791.3533	505.8627	8.6	0.2	1.1	491
_19AR111_Spot_ 208	491.5266	520.295	1.788163	10.00728	16.76574	0.013989	38.92025	0.001403	4.430377	0.113832	9.036064	0.400051	14.10533	5.451924	996.0768	816.1987	9.0	0.4	0.9	186
_19AR111_Spot_ 384	225.9299	285.5443	1.349365	4.645538	10.84853	0.034085	28.27121	0.001443	7.847186	0.277568	9.297691	0.729081	34.03262	9.462253	2570.85	463.6387	9.3	0.7	0.4	334
_19AR111_Spot_ 507	342.2271	415.8064	1.210321	6.988809	20.93553	0.022495	30.36756	0.001473	5.179322	0.170554	9.490685	0.491191	22.58768	6.783696	1812.27	556.826	9.5	0.5	0.5	436
_19AR111_Spot_ 15	151.9014	241.7472	1.375845	4.203693	49.70616	0.045343	53.18003	0.00175	9.223563	0.17344	11.26984	1.038573	45.0273	23.42651	2724.967	941.7388	11.3	1.0	0.4	12
_19AR111_Spot_ 90	627.3316	646.3405	1.092844	13.86501	8.075406	0.012505	41.55173	0.001837	2.618901	0.063027	11.83242	0.309595	12.61834	5.210739	166.1933	NA	11.8	0.3	7.1	77
_19AR111_Spot_ 179	461.3883	549.9436	1.343125	8.927009	9.673009	0.022233	25.44172	0.001868	2.476626	0.097345	12.03322	0.29774	22.32782	5.618638	1345.818	496.9392	12.0	0.3	0.9	159
_19AR111_Spot_ 210	249.81	269.9728	1.789872	8.186885	18.93079	0.01778	34.65569	0.001874	4.886801	0.14101	12.07009	0.589289	17.89488	6.147342	893.8674	729.1927	12.1	0.6	1.4	187
_19AR111_Spot_ 102	436.2848	436.6911	1.427369	11.22188	10.8121	0.014396	47.89384	0.001876	4.362809	0.091093	12.08334	0.526679	14.51359	6.901777	439.4297	NA	12.1	0.5	2.7	88
_19AR111_Spot_ 529	730.9118	976.2904	1.059602	12.69895	9.430399	0.016604	14.03934	0.001887	1.973529	0.140571	12.15139	0.239585	16.72089	2.328284	736.7836	295.5667	12.2	0.2	1.6	456
_19AR111_Spot_ 296	415.2216	543.9624	1.523714	13.10057	11.12634	0.013642	43.11114	0.002016	2.523223	0.058528	12.97975	0.327179	13.75799	5.891292	153.0905	NA	13.0	0.3	8.5	263
_19AR111_Spot_ 252	1012.934	1174.351	2.839531	14.7511	7.448156	0.015611	10.5757	0.002054	1.344209	0.127103	13.22561	0.177597	15.7286	1.650594	418.4287	234.8301	13.2	0.2	3.2	225
_19AR111_Spot_ 164	176.0283	187.134	0.719218	6.152868	28.74407	0.025243	37.8433	0.002095	8.890874	0.234939	13.49279	1.198372	25.31352	9.461326	1369.691	733.6042	13.5	1.2	1.0	145
_19AR111_Spot_ 76	461.1007	759.0201	2.300911	11.43001	12.30441	0.022812	25.29375	0.002416	2.028932	0.080215	15.55304	0.31518	22.9029	5.728217	884.4229	529.2852	15.6	0.3	1.8	65
_19AR111_Spot_ 94	708.8271	1087.589	1.097153	12.36553	8.050626	0.023301	12.87244	0.002499	1.352907	0.105101	16.0908	0.217422	23.38842	2.976259	857.8335	266.8084	16.1	0.2	1.9	80
_19AR111_Spot_ 286	312.7081	751.4515	1.107027	12.38659	10.60667	0.022201	18.63143	0.002629	3.057421	0.1641	16.92335	0.516739	22.29544	4.108708	649.0513	397.7825	16.9	0.5	2.6	253
_19AR111_Spot_ 290	566.9763	809.1917	1.28516	14.1946	6.07652	0.019285	21.42628	0.002682	2.154058	0.100533	17.26865	0.371479	19.39531	4.116277	292.6685	NA	17.3	0.4	5.9	257
_19AR111_Spot_ 224	425.4727	1162.102	1.431142	12.44275	13.73975	0.025427	19.57321	0.002711	2.504463	0.127954	17.45378	0.436532	25.49543	4.928183	870.0407	405.8595	17.5	0.4	2.0	200
_19AR111_Spot_ 364	667.0103	1152.079	5.143705	15.03619	8.579591	0.020809	10.59267	0.002812	1.403088	0.132458	18.10384	0.253657	20.91201	2.192488	357.9311	237.5495	18.1	0.3	5.1	320
_19AR111_Spot_ 521	1467.064	3367.569	2.843221	17.05038	3.196254	0.026572	7.834849	0.003557	2.922637	0.37303	22.88712	0.667722	26.62808	2.059155	379.6652	163.6276	22.9	0.7	6.0	448
_19AR111_Spot_ 345	447.9132	1325.129	2.214856	14.04765	5.826813	0.032648	10.8488	0.003934	2.20177	0.20295	25.31073	0.556191	32.62037	3.482691	611.2771	230.1517	25.3	0.6	4.1	304
_19AR111_Spot_ 594	775.1889	3124.828	1.838571	16.59893	4.709291	0.044896	5.324599	0.005867	0.711305	0.133589	37.70757	0.267433	44.59342	2.32304	433.5597	117.6167	37.7	0.3	8.7	507
_19AR111_Spot_ 169	292.9549	1625.484	1.130146	18.62082	5.049282	0.050312	14.13696	0.0082	1.05415	0.074567	52.6428	0.552674	49.84231	6.876149	NA	NA	52.6	0.6		150
_19AR111_Spot_ 262	1101.43	7498.558	3.636729	16.81703	2.207102	0.066004	3.158761	0.008342	0.921634	0.291771	53.55412	0.491529	64.90056	1.985913	507.2579	66.4634	53.6	0.5	10.6	234
_19AR111_Spot_ 303	1215.811	6585.921	5.182944	17.71878	2.59098	0.066005	8.518117	0.008807	8.022576	0.941825	56.5229	4.514771	64.90095	5.355414	386.8584	64.28596	56.5	4.5	14.6	268
_19AR111_Spot_ 258	124.5305	1240.914	3.479506	13.62888	7.910907	0.119838	15.53747	0.014114	2.246745	0.144602	90.3499	2.015773	114.9254	16.88458	660.4882	331.3018	90.3	2.0	13.7	231
_19AR111_Spot_ 560	345.1204	3002.175	1.993156	15.80685	3.607836	0.15338	6.329927	0.019073	1.188771	0.187802	121.7956	1.434279	144.8916	8.547405	542.8673	136.0307	121.8	1.4	22.4	486
_19AR111_Spot_ 186	239.0255	2642.132	1.892887	18.46178	2.263113	0.163976	10.84099	0.024538	1.012718	0.093416	156.2718	1.563564	154.1775	15.50846	123.1058	NA	156.3	1.6	126.9	165
_19AR111_Spot_ 241	213.2684	4467.212	1.556666	17.74387	2.751803	0.270893	3.910088	0.037053	0.760937	0.194609	234.5397	1.752624	243.4072	8.462816	330.8385	87.00831	234.5	1.8	70.9	216
_19AR111_Spot_ 500	124.7753	3924.605	2.435689	17.51311	4.3116	0.290197	6.431951	0.039394	0.818301	0.127224	249.0771	1.999325	258.7144	14.69061	347.9266	144.3911	249.1	2.0	71.6	430
_19AR111_Spot_ 108	166.4512	4558.499	1.222816	18.71239	2.353252	0.288613	3.175129	0.041701	0.793866	0.250026	263.3699	2.04867	257.4672	7.220914	205.0488	71.35907	263.4	2.0	128.4	93
_19AR111_Spot_ 145	102.5594	2818.305	1.042522	17.64479	2.747749	0.297068	6.934383	0.041965	1.120768	0.161625	265.002	2.90984	264.1073	16.1275	257.1566	157.4281	265.0	2.9	103.1	127
_19AR111_Spot_ 278	568.9801	5454.414	1.501826	18.82885	0.8833	0.299477	2.463671	0.04317	0.871994	0.353941	272.4542	2.326278	265.9917	5.765171	210.4793	53.42289	272.5	2.3	129.4	247
_19AR111_Spot_ 391	120.3964	3744.139	0.936091	17.18519	3.848658	0.33186	6.138276	0.044434	0.990256	0.161325	280.2604	2.715832	290.9846	15.53124	378.957	136.3237	280.3	2.7	74.0	341
_19AR111_Spot_ 546	332.7657	11877.13	1.668781	18.48161	1.664341	0.329625	2.218048	0.045296	0.80787	0.364226	285.5776	2.256741	289.2793	5.583362	320.2988	46.94523	285.6	2.3	89.2	472
_19AR111_Spot_ 236	209.4698	8587.684	1.509989	18.62701	2.272964	0.336422	3.114634	0.046973	0.741769	0.238156	295.9095	2.145348	294.4568	7.961343	283.9423	69.20761	295.9	2.1	104.2	211
_19AR111_Spot_ 150	156.6734	3075.61	2.084771	18.02497	2.147817	0.368005	8.850182	0.0527	0.82912	0.093684	331.0776	2.675733	318.1738	24.17855	225.7564	204.0056	331.1	2.7	146.7	132
_19AR111_Spot_ 71	154.4076	7815.364	1.276865	17.99935	2.212857	0.398367	3.108169	0.053829	0.829396	0.266844	337.9894	2.731049	340.4628	8.990996	358.3462	67.60152	338.0	2.7	94.3	61
_19AR111_Spot_ 196	467.9989	1474.829	5.177073	16.21383	1.209352	0.501944	13.60555	0.070545	0.833473	0.06126	439.4367	3.540541	413.0177	46.20057	268.9115	NA	439.4	3.5	163.4	174
_19AR111_Spot_ 528	378.9815	6429.879	2.264269	17.37291	1.16506	0.541234	1.716108	0.071048	0.894733	0.521374	442.4654	3.826077	439.2377	6.11922	423.3075	32.69021	442.5	3.8	104.5	455
_19AR111_Spot_ 11	390.2097	1708.222	9.961896	16.55408	1.072243	0.526324	3.779096	0.073452	0.806022	0.213284	456.9166	3.55536	429.367	13.23271	285.063	84.43249	456.9	3.6	160.3	9
_19AR111_Spot_ 530	201.3835	1083.555	5.045394	15.63242	1.259937	0.510149	8.39018	0.073467	0.753577	0.089817	457.0071	3.32466	418.5493	28.78689	212.7368	193.8898	457.0	3.3	214.8	457
_19AR111_Spot_ 55	130.5555	7571.106	1.553185	17.50617	2.842034	0.564414	3.276308	0.074324	0.84537	0.258025	462.1563	3.770172	454.3956	12.00276	416.2762	70.71645	462.2	3.8	111.0	48
_19AR111_Spot_ 525	233.5915	1477.274	4.19552	16.12553	1.715611	0.541327	3.544914	0.075391	0.941211	0.26551	468.5532	4.253618	439.2994	12.64222	289.7387	78.10755	468.6	4.3	161.7	452
_19AR111_Spot_ 376	477.0793	23945.12	195.0542	17.39148	0.899451	0.592447	1.331007	0.075567	0.754154	0.566604	469.6105	3.415661	472.429	5.028033	487.0868	24.20618	469.6	3.4	96.4	327
_19AR111_Spot_ 66	330.1244	14133.42	27.34512	17.8821	1.541662	0.571822	1.71301	0.075633	0.653951	0.381755	470.0041	2.964225	459.1922	6.327804	406.4029	35.43642	470.0	3.0	115.6	57
_19AR111_Spot_ 445	136.5614	9869.351	1.502284	16.90584	2.112723	0.60443	2.891724	0.075699	0.83389	0.288371	470.3981	3.782904	480.0414	11.06189	527.3459	60.68983	470.4	3.8	89.2	388
_19AR111_Spot_ 238	208.5095	2830.103	5.831058	16.76418	1.292246	0.569442	6.615592	0.075836	1.061378	0.160436	471.2178	4.822984	457.6538	24.37735	391.0629	146.6766	471.2	4.8	120.5	213
_19AR111_Spot_ 352	132.8202	3617.095	1.686264	17.4396	1.786909	0.558459	2.39072	0.076173	0.649167	0.271536	473.2408	2.962067	450.5228	8.698916	337.1491	52.1347	473.2	3.0	140.4	311
_19AR111_Spot_ 149	496.0112	11386.32	185.5762	17.24747	0.887832	0.595196	1.546033	0.076262	0.76276	0.493366	473.774	3.484158	474.1806	5.857327	477.0896	29.73259	473.8	3.5	99.3	131
_19AR111_Spot_ 309	104.3125	4375.746	1.430998	17.924	3.030094	0.553989	6.188619	0.076266	0.700351	0.113168	473.7941	3.199217	447.6063	22.40512	316.1377	139.9472	473.8	3.2	149.9	273
_19AR111_Spot_ 449	217.639	7234.222	9.725129	17.24965	1.62444	0.590721	3.987274	0.0763	0.783429	0.196482	473.9974	3.580195	471.3277	15.03578	459.336	86.72781	474.0	3.6	103.2	392
_19AR111_Spot_ 550	80.86108	3727.8	1.805641	16.98561	2.511108	0.576915	5.259935	0.076304	0.931699	0.177131	474.0222	4.257991	462.4772	19.54194	406.4918	115.9207	474.0	4.3	116.6	476
_19AR111_Spot_ 7	97.97521	4011.611	1.217962	17.3294	2.882199	0.569594	6.764183	0.076377	0.704015	0.10408	474.4628	3.220326	457.752	24.92933	375.6691	151.5525	474.5	3.2	126.3	5
_19AR111_Spot_ 116	48.88748	3004.125	1.727334	14.60297	7.302116	0.669119	7.498645	0.076383	0.853272	0.11379	474.4992	3.903349	520.1765	30.5323	727.0409	158.1549	474.5	3.9	65.3	101
_19AR111_Spot_ 37	136.3215	6717.149	3.211441	17.49657	2.62564	0.581178	2.824626	0.076424	0.624046	0.220931	474.7454	2.856169	465.2181	10.54229	419.4127	61.52638	474.7	2.9	113.2	31
_19AR111_Spot_ 233	178.6434	10753.1	1.146771	17.50739	1.635623	0.587914	2.002302	0.076489	0.687247	0.343229	475.1345	3.147915	469.5347	7.52756	443.2004	41.8455	475.1	3.1	107.2	208
_19AR111_Spot_ 420	169.2806	12048.11	1.799256	17.54881	2.450117	0.586188	2.7088	0.076494	0.857652	0.316617	475.1629	3.928678	468.4302	10.16491	436.5182	57.23217	475.2	3.9	108.9	367
_19AR111_Spot_ 317	711.0222	42675.75	1.267636	17.54557	0.776772	0.600465	1.132659	0.07653	0.765546	0.675884	475.377	3.508285	477.5286	4.314923	488.8338	18.39671	475.4	3.5	97.2	280
_19AR111_Spot_ 121	485.4305	27942.21	2.018077	17.56799	0.89638	0.594291	1.257551	0.076596	0.776137	0.617181	475.7739	3.55968	473.6041	4.759815	464.0688	21.93358	475.8	3.6	102.5	106
_19AR111_Spot_ 524	332.5976	24401.6	0.808585	17.37055	0.807357	0.602014	1.383913	0.07669	0.695115	0.502282	476.3326	3.191689	478.5113	5.280597	489.9235	26.37762	476.3	3.2	97.2	451
_19AR111_Spot_ 457	259.2728	12203.91	1.776751	17.77767	1.501652	0.582937	2.277291	0.076722	0.80807	0.354838	476.5249	3.711774	466.347	8.515623	417.4943	47.57541	476.5	3.7	114.1	399
_19AR111_Spot_ 481	287.1161	13463.33	12.4209	17.39698	1.081704	0.596982	1.775978	0.076766	0.850302	0.478779	476.7894	3.907851	475.3166	6.741166	469.1738	34.52287	476.8	3.9	101.6	419
_19AR111_Spot_ 194	104.5237	4965.511	1.929404	17.70166	2.151686	0.566804	3.478452	0.076928	0.775978	0.223081	477.7618	3.573276	455.9454	12.77783	348.3776	76.68758	477.8	3.6	137.1	172
_19AR111_Spot_ 511	373.4523	34175.51	5.417292	17.66891	0.906718	0.5965	1.365088	0.076928	0.733895	0.537618	477.7631	3.379502	475.0104	5.178882	462.7236	25.53153	477.8	3.4	103.3	440
_19AR111_Spot_ 246	44.40857	1808.991	3.321741	16.42834	5.203126	0.558765	6.104047	0.07693	1.271005	0.208223	477.7738	5.852959	450.7225	22.22111	315.9294	135.8996	477.8	5.9	151.2	221
_19AR111_Spot_ 519	284.8184	18290.24	3.214608	17.77365	1.193196	0.587636	1.746174	0.077002	0.689275	0.394735	478.2058	3.176864	469.3571	6.562677	427.2616	35.80045	478.2	3.2	111.9	446
_19AR111_Spot_ 451	88.01739	4453.956	3.009597	18.26146	3.152187	0.547722	3.995507	0.077015	0.823429	0.206089	478.2796	3.795745	443.5032	14.35813	267.8294	89.713	478.3	3.8	178.6	394
_19AR111_Spot_ 347	283.7857	10770.97	14.81156	17.40312	1.433709	0.595923	1.68627	0.077065	0.796498	0.472343	478.5773	3.6738	474.6433	6.393534	456.6339	32.97647	478.6	3.7	104.8	306
_19AR111_Spot_ 510	308.3337	19571.46	3.278857	17.6962	0.951036	0.593275	1.685364	0.077069	0.784536	0.465499	478.6033	3.618817	472.9572	6.372282	446.6044	33.15297	478.6	3.6	107.2	439
_19AR111_Spot_ 167	100.2605	4418.19	1.901373	16.99856	3.167646	0.589376	4.227928	0.077148	0.739392	0.174883	479.0787	3.413844	470.469	15.92058	429.6326	92.82588	479.1	3.4	111.5	148
_19AR111_Spot_ 513	167.8296	9564.003	2.224166	17.58471	1.687514	0.588712	1.92967	0.077161	0.765341	0.396618	479.155	3.534198	470.045	7.260694	426.7528	39.51933	479.2	3.5	112.3	441
_19AR111_Spot_ 348	194.4997	7133.426	1.003458	17.07316	2.132587	0.601377	3.71433	0.077189	0.862742	0.232274	479.3198	3.985294	478.1073	14.16419	473.2497	79.96716	479.3	4.0	101.3	307
_19AR111_Spot_ 508	261.2955	13733.67	1.812389	17.56951	1.649766	0.595356	2.470576	0.077213	0.717169	0.290284	479.4641	3.313808	474.2825	9.361809	450.2405	52.53823	479.5	3.3	106.5	437
_19AR111_Spot_ 427	225.6617	11668.46	1.068913	17.56142	1.529396	0.5917	2.342887	0.077234	1.037678	0.442906	479.5912	4.796	471.9527	8.843673	435.9292	46.79673	479.6	4.8	110.0	372
_19AR111_Spot_ 31	145.294	8621.194	1.771634	17.32801	1.937967	0.598022	3.350653	0.077234	0.891334	0.266018	479.5926	4.119629	475.9775	12.73258	459.5578	71.6608	479.6	4.1	104.4	25
_19AR111_Spot_ 321	94.37836	4725.992	4.466124	17.65218	3.165284	0.570579	3.932739	0.077235	0.849855	0.216098	479.5987	3.927967	458.3891	14.5081	354.3934	86.75669	479.6	3.9	135.3	284
_19AR111_Spot_ 38	262.7719	14632.44	1.810488	17.43731	1.7092	0.601129	2.158878	0.077262	0.711024	0.329349	479.7619	3.287376	477.9498	8.23017	470.2063	45.12263	479.8	3.3	102.0	32
_19AR111_Spot_ 382	134.0522	7129.865	2.158828	17.87222	2.47755	0.57333	3.456534	0.077272	0.750967	0.21726	479.8185	3.472447	460.1659	12.79023	364.1632	76.08295	479.8	3.5	131.8	332
_19AR111_Spot_ 230	164.3244	9967.784	7.480764	17.18532	2.327592	0.604675	2.887405	0.077273	0.823251	0.285118	479.8222	3.806711	480.1961	11.04815	482.964	61.15525	479.8	3.8	99.3	206
_19AR111_Spot_ 592	158.1276	6908.065	1.987648	17.37752	1.715681	0.590336	2.133695	0.077317	0.737109	0.345461	480.0907	3.410227	471.0823	8.042327	428.3806	44.65775	480.1	3.4	112.1	506
_19AR111_Spot_ 315	209.2881	12200.35	5.206579	17.29242	1.274368	0.60696	2.111811	0.077381	0.765524	0.362496	480.4712	3.544392	481.6411	8.09933	488.1944	43.45435	480.5	3.5	98.4	278
_19AR111_Spot_ 199	82.51857	3833.505	1.862826	17.58531	2.019073	0.564548	6.59292	0.077385	1.004813	0.152408	480.497	4.652551	454.4821	24.16025	325.9381	148.068	480.5	4.7	147.4	177
_19AR111_Spot_ 14	212.6332	7758.08	9.771377	17.5756	1.525213	0.588078	1.915307	0.077389	0.893036	0.466262	480.52	4.135181	469.6395	7.201761	417.7587	37.86898	480.5	4.1	115.0	11
_19AR111_Spot_ 273	243.3029	14139.94	2.743654	17.36168	1.532434	0.602382	1.766306	0.077394	0.675774	0.382592	480.547	3.129327	478.7445	6.742304	471.1032	36.11818	480.5	3.1	102.0	243
_19AR111_Spot_ 302	134.0257	9236.919	5.996265	17.56026	2.695828	0.590485	2.940812	0.07745	0.713335	0.242564	480.8862	3.305505	471.1775	11.08649	425.1128	63.67662	480.9	3.3	113.1	267
_19AR111_Spot_ 444	131.0857	7749.628	1.9119	17.49446	2.120606	0.592683	2.326459	0.077463	0.736628	0.316631	480.9638	3.413974	472.5798	8.790822	433.0228	49.18663	481.0	3.4	111.1	387
_19AR111_Spot_ 416	172.1624	10385.22	5.242067	16.91798	1.490017	0.614991	1.939064	0.077477	0.929023	0.479109	481.043	4.306331	486.7029	7.49771	514.3752	37.39061	481.0	4.3	93.5	364
_19AR111_Spot_ 572	27.11219	1237.887	2.333355	14.63271	8.184881	0.604993	12.61924	0.077483	1.619091	0.128303	481.0823	7.505614	480.3974	48.33573	478.0706	277.5827	481.1	7.5	100.6	496
_19AR111_Spot_ 472	181.0844	9326.794	7.564816	17.5296	1.445626	0.593386	2.551231	0.077495	0.708211	0.277596	481.1507	3.283502	473.0279	9.647378	434.765	54.59128	481.2	3.3	110.7	412
_19AR111_Spot_ 158	256.7367	9864.266	7.870091	17.49066	1.003048	0.59495	2.21351	0.077567	0.911455	0.411769	481.5848	4.229481	474.0237	8.384066	438.5391	44.91892	481.6	4.2	109.8	139
_19AR111_Spot_ 107	168.9801	7350.744	1.62565	17.27817	1.536694	0.598052	2.38982	0.077575	0.716698	0.299896	481.6294	3.326032	475.9968	9.081443	449.8892	50.66638	481.6	3.3	107.1	92
_19AR111_Spot_ 575	99.45038	4418.621	1.410492	17.43021	3.259978	0.579949	5.564093	0.077582	0.805775	0.144817	481.6736	3.739751	464.4289	20.74109	380.9928	123.8503	481.7	3.7	126.4	498
_19AR111_Spot_ 463	238.8898	11455.64	3.458313	17.51423	1.260581	0.598474	1.756931	0.077645	0.798895	0.45471	482.0504	3.710609	476.2647	6.679292	449.4408	34.78304	482.1	3.7	107.3	403
_19AR111_Spot_ 115	233.0447	10568.83	2.869521	17.66432	1.361573	0.590225	2.115047	0.077681	0.726674	0.343573	482.268	3.376633	471.0114	7.971093	417.482	44.3733	482.3	3.4	115.5	100
_19AR111_Spot_ 389	172.8488	10273.51	4.414361	17.42588	2.097821	0.598451	2.45679	0.077724	0.780873	0.317843	482.5202	3.630311	476.2501	9.339837	447.1092	51.77144	482.5	3.6	107.9	339
_19AR111_Spot_ 535	94.77039	5497.09	1.655572	17.55626	2.543912	0.581007	4.14274	0.077729	0.887293	0.21418	482.5523	4.125325	465.1087	15.45962	380.8368	91.00643	482.6	4.1	126.7	462
_19AR111_Spot_ 114	193.288	9567.591	4.087504	17.33084	1.815238	0.601047	3.342724	0.077772	0.758715	0.226975	482.8131	3.52936	477.898	12.74259	455.3446	72.28072	482.8	3.5	106.0	99
_19AR111_Spot_ 595	223.0079	11331.82	3.214537	17.75275	1.810053	0.589626	2.078426	0.077777	0.750177	0.360935	482.8392	3.489824	470.6289	7.82807	412.4669	43.35154	482.8	3.5	117.1	508
_19AR111_Spot_ 168	212.6502	9404.854	27.59151	17.43558	1.214759	0.597553	3.057164	0.077798	0.770582	0.252058	482.9633	3.585632	475.6796	11.61151	441.6517	65.84146	483.0	3.6	109.4	149
_19AR111_Spot_ 514	282.0313	16855.56	1.497934	17.40236	1.241498	0.607108	1.64918	0.077806	0.746814	0.45284	483.0139	3.47539	481.7343	6.325929	476.6139	32.5312	483.0	3.5	101.3	442
_19AR111_Spot_ 30	179.4766	9042.607	6.389153	17.45765	1.920772	0.59908	2.99175	0.077858	0.9241	0.308883	483.3247	4.303078	476.6498	11.3812	445.6025	63.26436	483.3	4.3	108.5	24
_19AR111_Spot_ 118	112.9151	5597.734	1.170636	17.8458	1.546768	0.572626	4.925097	0.077864	0.811611	0.164791	483.3577	3.779519	459.7113	18.21113	344.1603	109.9754	483.4	3.8	140.4	103
_19AR111_Spot_ 518	211.0564	3462.261	4.146286	16.94305	1.458242	0.588217	2.35451	0.07793	0.723296	0.307196	483.753	3.370905	469.7285	8.854605	402.7216	50.16462	483.8	3.4	120.1	445
_19AR111_Spot_ 597	258.9987	13191.07	3.662986	17.44503	1.620726	0.603709	1.908469	0.077968	0.736157	0.385732	483.9824	3.432409	479.5848	7.294987	459.5713	39.04812	484.0	3.4	105.3	510
_19AR111_Spot_ 56	209.0894	10504.25	3.922589	17.48281	1.684264	0.598848	3.08398	0.07799	0.859211	0.278605	484.1129	4.007203	476.5022	11.72925	440.9773	65.90347	484.1	4.0	109.8	49
_19AR111_Spot_ 344	205.2764	11114.94	1.048234	17.53351	2.112061	0.600798	2.614388	0.078147	0.798774	0.30553	485.0543	3.732312	477.7399	9.963359	443.7245	55.38128	485.1	3.7	109.3	303
_19AR111_Spot_ 237	311.2165	17672.45	7.921077	17.50371	1.071818	0.607277	1.415647	0.078274	0.685485	0.484221	485.8138	3.207792	481.8412	5.431063	463.9363	27.4462	485.8	3.2	104.7	212
_19AR111_Spot_ 78	145.6307	7733.024	4.075401	17.27939	1.818232	0.604261	2.256139	0.078285	0.815327	0.361382	485.8788	3.815892	479.9342	8.628906	452.5763	46.73064	485.9	3.8	107.4	67
_19AR111_Spot_ 234	132.7607	7163.11	0.99891	17.20867	2.575571	0.605041	3.35699	0.078305	0.722388	0.215189	485.9948	3.381695	480.4275	12.84995	454.9164	72.79425	486.0	3.4	106.8	209
_19AR111_Spot_ 229	98.51284	6138.56	2.899335	17.47724	2.748958	0.593166	3.42207	0.078411	0.868912	0.253914	486.6294	4.072728	472.8877	12.93772	407.7011	74.07899	486.6	4.1	119.4	205
_19AR111_Spot_ 588	124.4716	7393.086	1.846264	17.65762	3.153066	0.592921	4.109991	0.078545	0.812802	0.197763	487.4337	3.815793	472.7315	15.53486	402.9376	90.25056	487.4	3.8	121.0	502
_19AR111_Spot_ 589	261.0411	16659.45	5.803432	17.71097	1.40629	0.603654	1.675281	0.078668	0.694462	0.414535	488.1676	3.264955	479.5497	6.403251	439.496	33.93622	488.2	3.3	111.1	503
_19AR111_Spot_ 53	195.9527	10219.73	3.877657	17.60698	1.794372	0.599619	2.253683	0.078715	0.849441	0.376912	488.4462	3.995771	476.9922	8.578121	423.2351	46.58673	488.4	4.0	115.4	46
_19AR111_Spot_ 400	176.5332	8794.999	2.24822	16.88667	1.573962	0.623227	2.40701	0.07872	0.66012	0.274249	488.4758	3.105387	491.8678	9.383979	508.6439	50.90745	488.5	3.1	96.0	349
_19AR111_Spot_ 380	140.345	7735.314	3.123092	17.1206	1.919467	0.613194	2.673436	0.07881	0.758768	0.283817	489.0166	3.573256	485.5724	10.31872	470.2909	56.76947	489.0	3.6	104.0	330
_19AR111_Spot_ 342	207.1777	13477.97	4.877703	18.02675	1.518164	0.591318	2.054098	0.078857	0.868296	0.422714	489.2969	4.091312	471.7091	7.750393	387.9833	41.79767	489.3	4.1	126.1	301
_19AR111_Spot_ 165	169.601	6967.889	2.853409	17.62583	1.932724	0.593487	2.569888	0.078951	0.901051	0.350619	489.8587	4.250339	473.0922	9.718969	393.5272	53.97227	489.9	4.3	124.5	146
_19AR111_Spot_ 206	374.0495	9648.94	3.463734	17.1077	0.99488	0.61803	2.396843	0.078991	0.814047	0.339633	490.0981	3.841742	488.6117	9.296182	482.6317	49.81858	490.1	3.8	101.5	184
_19AR111_Spot_ 491	124.5811	9825.267	1.971672	17.35626	2.239503	0.612282	2.476494	0.079189	0.879121	0.354986	491.2776	4.158458	484.9984	9.549721	456.3889	51.39433	491.3	4.2	107.6	424
_19AR111_Spot_ 22	143.6316	5958.558	2.120369	17.12485	1.895681	0.613824	3.967314	0.079313	0.722848	0.182201	492.0199	3.424218	485.9691	15.32312	458.5191	86.5478	492.0	3.4	107.3	18
_19AR111_Spot_ 365	171.7437	13198.48	3.416792	17.37241	2.258828	0.617966	2.74356	0.079558	0.851098	0.310217	493.4831	4.043291	488.5715	10.64034	466.547	57.77629	493.5	4.0	105.8	321
_19AR111_Spot_ 556	365.0019	8226.971	2.45782	17.11879	1.092633	0.622656	2.460098	0.07997	0.834028	0.339022	495.9444	3.981209	491.5104	9.585543	471.8598	51.21902	495.9	4.0	105.1	482
_19AR111_Spot_ 141	153.748	10382.14	3.2566	17.54233	2.244481	0.617559	2.613215	0.080729	0.882004	0.337517	500.4728	4.247192	488.3159	10.13065	432.6219	54.81031	500.5	4.2	115.7	123
_19AR111_Spot_ 440	227.6758	10460.51	2.976413	17.22768	1.50262	0.633303	2.930329	0.080888	0.740262	0.252621	501.4215	3.571145	498.1516	11.53745	484.1373	62.61859	501.4	3.6	103.6	383
_19AR111_Spot_ 340	177.3612	15400.54	4.445039	17.60985	1.58709	0.626971	2.148493	0.08144	0.865032	0.402623	504.7142	4.199401	494.2074	8.407009	446.7615	43.73007	504.7	4.2	113.0	299
_19AR111_Spot_ 431	152.2826	7142.971	2.37563	17.26344	2.082929	0.630967	3.056372	0.082084	1.128302	0.369164	508.5515	5.517503	496.698	12.00654	443.3763	63.17612	508.6	5.5	114.7	376
_19AR111_Spot_ 10	211.951	12605.28	3.028683	17.22771	1.268931	0.649974	1.880503	0.082773	0.710468	0.377807	512.6559	3.501192	508.4626	7.521956	490.5957	38.42679	512.7	3.5	104.5	8
_19AR111_Spot_ 86	260.7234	15281.15	0.673428	17.40722	1.200336	0.644992	1.660242	0.082821	0.731929	0.440857	512.9405	3.608876	505.392	6.609946	472.3334	32.98495	512.9	3.6	108.6	74

_19AR111_Spot_ 104	253.4586	3231.473	3.351504	16.72072	1.783156	0.632355	2.279982	0.082977	0.734733	0.322254	513.8693	3.629005	497.5615	8.968477	424.1675	48.16479	513.9	3.6	121.1	90
_19AR111_Spot_ 77	151.745	8185.993	1.307864	16.56796	1.779514	0.671555	2.30458	0.083195	0.814008	0.353213	515.1636	4.03029	521.6573	9.401453	551.1618	47.04923	515.2	4.0	93.5	66
_19AR111_Spot_ 139	174.0343	13636.06	2.156564	16.94166	1.093187	0.664148	2.01207	0.083286	0.708388	0.352069	515.7055	3.510891	517.1479	8.153707	524.4602	41.30164	515.7	3.5	98.3	121
_19AR111_Spot_ 17	73.90747	2631.792	1.976428	16.76826	2.834875	0.622268	3.355463	0.083289	0.969302	0.288873	515.7229	4.80418	491.2677	13.06956	379.7595	72.24962	515.7	4.8	135.8	14
_19AR111_Spot_ 516	318.0001	17674.8	7.81131	17.18739	0.821786	0.661236	1.676951	0.083621	0.667932	0.398301	517.6987	3.322672	515.3697	6.777693	506.0251	33.85012	517.7	3.3	102.3	443
_19AR111_Spot_ 426	169.7109	9294.502	1.602568	17.19471	2.022176	0.652552	2.437769	0.083826	1.051414	0.431302	518.9178	5.242156	510.0477	9.774527	471.4439	48.67604	518.9	5.2	110.1	371
_19AR111_Spot_ 422	154.6021	1735.627	4.982784	16.21445	2.413177	0.613475	15.29614	0.083836	0.874637	0.05718	518.9788	4.361269	485.7494	59.12033	332.8404	348.0396	519.0	4.4	155.9	369
_19AR111_Spot_ 223	294.198	25468.23	152.0063	17.30737	1.140197	0.661867	1.543786	0.083931	0.922126	0.597315	519.5411	4.602855	515.7554	6.243053	499.9968	27.27212	519.5	4.6	103.9	199
_19AR111_Spot_ 148	187.5117	14125.78	4.86052	17.13373	1.929363	0.662237	2.199165	0.083983	0.979876	0.445567	519.855	4.893952	515.9815	8.896502	499.8438	43.36019	519.9	4.9	104.0	130
_19AR111_Spot_ 381	275.0761	14840.62	5.231249	17.10696	1.315277	0.664561	1.728926	0.084003	0.90601	0.52403	519.9715	4.526005	517.3999	7.008873	506.9959	32.37661	520.0	4.5	102.6	331
_19AR111_Spot_ 35	76.30016	3855.595	1.26177	16.73283	3.54283	0.65062	4.196811	0.084022	0.959491	0.228624	520.0858	4.794183	508.86	16.79845	459.6781	90.65136	520.1	4.8	113.1	29
_19AR111_Spot_ 478	170.8366	12933.7	2.900941	17.29719	1.673978	0.656176	2.058317	0.08404	0.738616	0.358844	520.1921	3.691283	512.2721	8.280671	478.0257	42.47043	520.2	3.7	108.8	417
_19AR111_Spot_ 247	79.95471	4403.442	1.510076	16.75265	3.163554	0.652376	4.863911	0.084089	0.700587	0.144038	520.4857	3.50313	509.9401	19.50106	463.9111	106.7141	520.5	3.5	112.2	222
_19AR111_Spot_ 50	558.024	36911.81	9.639871	17.1354	0.634581	0.671068	1.000721	0.08415	0.670071	0.669589	520.8465	3.352773	521.3618	4.08054	524.5538	16.28312	520.8	3.4	99.3	43
_19AR111_Spot_ 310	126.6101	7398.312	3.702059	16.98552	2.391996	0.663608	2.693003	0.084199	0.780745	0.289916	521.1348	3.908617	516.8186	10.90798	498.7195	56.7981	521.1	3.9	104.5	274
_19AR111_Spot_ 124	202.162	9254.995	4.826964	17.32958	1.574919	0.650255	1.908031	0.08425	0.773077	0.40517	521.4395	3.872401	508.6354	7.634066	452.4203	38.73259	521.4	3.9	115.3	109
_19AR111_Spot_ 220	118.6475	5963.652	1.99376	17.31552	2.311635	0.643104	2.870012	0.084285	0.824175	0.287168	521.649	4.129948	504.2264	11.40639	426.887	61.32839	521.6	4.1	122.2	196
_19AR111_Spot_ 109	107.7442	8695.416	2.090675	16.73522	2.437393	0.674781	3.835517	0.084296	0.743683	0.193894	521.7129	3.727042	523.6153	15.69254	532.895	82.4214	521.7	3.7	97.9	94
_19AR111_Spot_ 1	152.9702	8616.685	2.788126	16.76728	2.396235	0.673668	3.372181	0.084302	0.741346	0.219842	521.7472	3.715561	522.9404	13.78302	529.129	72.10033	521.7	3.7	98.6	1
_19AR111_Spot_ 405	231.4761	12282.5	2.705837	17.30882	1.336481	0.65658	2.37853	0.084305	0.774966	0.325817	521.768	3.884212	512.5196	9.572523	472.4222	49.75997	521.8	3.9	110.4	354
_19AR111_Spot_ 484	290.0493	19282.61	1.363396	17.09171	1.270861	0.670857	1.534515	0.084331	0.693068	0.451653	521.9204	3.474703	521.2333	6.256006	519.1573	30.06795	521.9	3.5	100.5	422
_19AR111_Spot_ 277	333.1264	16072.35	6.27846	17.18757	1.093667	0.665299	1.427276	0.08436	0.701201	0.491286	522.0972	3.516623	517.8501	5.789848	500.1351	27.38648	522.1	3.5	104.4	246
_19AR111_Spot_ 548	634.755	34253.4	3.213916	17.07782	0.833925	0.675017	1.194987	0.084372	0.769632	0.64405	522.1688	3.860322	523.7582	4.88981	531.669	20.03097	522.2	3.9	98.2	474
_19AR111_Spot_ 429	214.8601	11244.16	2.260159	17.13155	1.558199	0.663133	2.023517	0.084468	0.990737	0.489612	522.7391	4.974555	516.5284	8.192561	490.1282	38.94313	522.7	5.0	106.7	374
_19AR111_Spot_ 541	249.8683	11647.55	7.250092	17.33295	1.358655	0.655647	2.354173	0.084541	0.788104	0.334769	523.1734	3.960277	511.948	9.466366	463.1167	49.17656	523.2	4.0	113.0	467
_19AR111_Spot_ 6	83.32158	4839.489	1.7439	16.97881	3.23416	0.651742	3.70398	0.084545	0.856437	0.231221	523.1925	4.303805	509.55	14.84099	449.7342	80.08995	523.2	4.3	116.3	4
_19AR111_Spot_ 314	357.6626	23525.23	2.858122	17.17988	1.137145	0.675574	1.635563	0.084702	0.708553	0.433216	524.1305	3.566776	524.0961	6.69596	524.8797	32.32369	524.1	3.6	99.9	277
_19AR111_Spot_ 387	110.3926	6129.917	2.289212	16.74529	2.467186	0.668465	3.882752	0.084729	0.774114	0.199372	524.2875	3.897924	519.779	15.7967	500.9924	83.81952	524.3	3.9	104.6	337
_19AR111_Spot_ 334	219.0238	13489.48	4.517468	17.63002	1.67862	0.650799	2.18753	0.084838	0.66074	0.302049	524.9352	3.330995	508.9705	8.756842	438.819	46.42138	524.9	3.3	119.6	295
_19AR111_Spot_ 469	199.9205	9804.842	9.42293	16.85868	1.681168	0.677326	1.948143	0.084861	0.850503	0.436571	525.0727	4.288729	525.1568	7.988032	526.4969	38.40365	525.1	4.3	99.7	409
_19AR111_Spot_ 360	180.9016	12772.1	3.157576	17.4727	1.349869	0.655432	1.919191	0.084894	0.784643	0.408841	525.2691	3.958045	511.8163	7.715658	453.1149	38.90636	525.3	4.0	115.9	317
_19AR111_Spot_ 24	387.7217	39914.34	15.05505	17.15439	1.103865	0.680708	1.359042	0.084951	0.772372	0.568321	525.606	3.898542	527.2024	5.589022	535.0726	24.47166	525.6	3.9	98.2	19
_19AR111_Spot_ 21	212.2391	9046.605	1.252604	16.99635	1.856646	0.672667	2.533975	0.084975	0.739586	0.291868	525.7481	3.734024	522.3325	10.34756	508.4034	53.29372	525.7	3.7	103.4	17
_19AR111_Spot_ 201	114.015	4530.028	2.434861	16.62089	2.76126	0.666599	5.873143	0.085197	1.022257	0.174056	527.0699	5.173628	518.6422	23.85689	482.6716	127.8343	527.1	5.2	109.2	179
_19AR111_Spot_ 356	134.7523	12293.88	1.82435	17.00389	3.383497	0.677504	3.570299	0.085377	0.727594	0.203791	528.1388	3.689505	525.2647	14.64242	513.7608	76.82039	528.1	3.7	102.8	313
_19AR111_Spot_ 185	416.7939	23858.21	1.448876	17.14339	0.946915	0.678445	1.246842	0.085464	0.740056	0.593544	528.6536	3.756208	525.8344	5.117439	514.5478	22.04059	528.7	3.8	102.7	164
_19AR111_Spot_ 373	164.9436	6187.554	1.715443	17.6949	2.648735	0.637614	2.899696	0.085473	0.770497	0.265717	528.7098	3.911114	500.8279	11.46429	376.322	62.90666	528.7	3.9	140.5	326
_19AR111_Spot_ 353	192.8798	11747.62	1.421313	17.17221	1.257166	0.67166	1.687796	0.08558	0.701125	0.415408	529.3418	3.563057	521.7211	6.885858	489.4436	33.85014	529.3	3.6	108.2	312
_19AR111_Spot_ 89	163.2905	6522.538	1.296208	17.07704	2.027913	0.665817	3.32761	0.085641	0.752037	0.225999	529.7043	3.824297	518.1661	13.50565	468.5668	71.7853	529.7	3.8	113.0	76
_19AR111_Spot_ 239	283.6187	22975.99	4.095035	17.0731	1.05023	0.685986	1.471581	0.085962	0.669462	0.454927	531.6116	3.416144	530.3859	6.079679	526.0918	28.72087	531.6	3.4	101.0	214
_19AR111_Spot_ 92	113.2009	2098.251	1.15099	16.25945	2.398755	0.650345	5.985662	0.086369	1.176846	0.196611	534.0294	6.031431	508.6912	23.95476	397.2996	131.6802	534.0	6.0	134.4	79
_19AR111_Spot_ 163	146.6518	6424.78	2.118557	17.03599	2.131824	0.673497	3.661344	0.086748	0.982283	0.268285	536.2768	5.054599	522.8362	14.96278	465.5162	78.15593	536.3	5.1	115.2	144
_19AR111_Spot_ 268	199.0079	15926.85	1.184276	17.17657	1.736412	0.684014	2.118991	0.086768	1.01998	0.481352	536.3953	5.24969	529.1976	8.739551	499.2133	40.91543	536.4	5.2	107.4	239
_19AR111_Spot_ 509	483.0709	37201.88	2.070592	17.17632	0.901837	0.69409	1.289529	0.086947	0.714819	0.554325	537.4552	3.686039	535.2551	5.36469	526.8653	23.53954	537.5	3.7	102.0	438
_19AR111_Spot_ 34	200.2899	14285.67	2.830153	17.60257	1.321609	0.67537	1.798283	0.087527	0.890608	0.495254	540.8919	4.620665	523.9725	7.360831	451.8468	34.71164	540.9	4.6	119.7	28
_19AR111_Spot_ 410	139.1956	7013.726	3.042761	16.73091	2.088819	0.698393	2.471676	0.087978	0.894012	0.361703	543.5664	4.660307	537.8305	10.32044	514.519	50.62116	543.6	4.7	105.6	359
_19AR111_Spot_ 270	140.9	7111.767	4.543352	17.07592	2.396462	0.686656	3.775159	0.088262	0.915924	0.242619	545.2515	4.788717	530.7892	15.60673	470.0283	81.09035	545.3	4.8	116.0	240
_19AR111_Spot_ 329	98.18473	7122.622	1.759237	17.48179	2.888398	0.675665	4.566672	0.088714	0.7237	0.158474	547.9259	3.801494	524.151	18.6992	422.8235	100.6632	547.9	3.8	129.6	291
_19AR111_Spot_ 474	210.7289	10621.24	1.977291	16.81556	1.555778	0.714924	2.490225	0.08924	0.65372	0.262515	551.0384	3.452586	547.6661	10.54142	534.6066	52.58739	551.0	3.5	103.1	414
_19AR111_Spot_ 540	275.7275	16999.72	5.444125	16.6029	0.946494	0.731956	1.725965	0.089719	0.700498	0.405859	553.8727	3.717861	557.7006	7.406579	574.2966	34.28164	553.9	3.7	96.4	466
_19AR111_Spot_ 154	834.102	37262.17	9.524249	16.91311	0.781616	0.725613	1.308618	0.089805	0.81471	0.622573	554.3848	4.327867	553.975	5.587389	553.2475	22.32952	554.4	4.3	100.2	135
_19AR111_Spot_ 557	394.8076	25916.79	3.872252	16.73879	0.995301	0.731606	1.403806	0.089877	0.878372	0.625708	554.8113	4.669486	557.4955	6.02241	569.4378	23.84956	554.8	4.7	97.4	483
_19AR111_Spot_ 549	878.2798	25496.52	4.667109	16.66405	0.720565	0.740214	1.098622	0.090573	0.761426	0.693074	558.9246	4.076521	562.5302	4.744995	578.0659	17.22036	558.9	4.1	96.7	475
_19AR111_Spot_ 468	349.0688	4464.115	4.579553	16.43019	1.200888	0.721586	5.308333	0.090721	0.935737	0.176277	559.7983	5.017241	551.603	22.59536	518.8424	114.7842	559.8	5.0	107.9	408
_19AR111_Spot_ 272	87.93326	4739.175	2.453452	16.98109	3.720412	0.697776	3.938086	0.090815	0.572491	0.145373	560.3557	3.072511	537.4619	16.43572	442.4103	86.70695	560.4	3.1	126.7	242
_19AR111_Spot_ 213	540.4378	34704.6	13.8411	16.40167	0.748497	0.779736	1.319302	0.093479	1.053234	0.798326	576.0808	5.804269	585.3327	5.869092	622.3021	17.15786	576.1	5.8	92.6	190
_19AR111_Spot_ 192	84.21816	4866.935	0.989292	16.12023	3.129247	0.761775	4.789087	0.093709	0.919024	0.1919	577.4379	5.076056	575.0334	21.02911	566.5105	102.3857	577.4	5.1	101.9	170
_19AR111_Spot_ 425	59.50893	4798.142	0.55178	16.33678	3.390557	0.75152	4.786084	0.093893	1.089365	0.227611	578.5224	6.027707	569.1058	20.85435	532.632	102.1281	578.5	6.0	108.6	370
_19AR111_Spot_ 267	279.4854	17860.85	2.432316	16.71749	1.219772	0.767086	1.520957	0.094567	0.800319	0.526194	582.4919	4.457379	578.0898	6.704083	561.7949	28.19836	582.5	4.5	103.7	238
_19AR111_Spot_ 346	330.3751	5653.826	0.832362	16.34265	1.376822	0.767024	1.919294	0.094936	0.790517	0.411879	584.6649	4.41848	578.0542	8.459561	553.0844	38.17354	584.7	4.4	105.7	305
_19AR111_Spot_ 498	359.4849	23473.05	2.78772	16.57742	1.225013	0.785653	1.602537	0.095359	0.923986	0.576577	587.1551	5.185491	588.703	7.159438	595.6131	28.34917	587.2	5.2	98.6	429
_19AR111_Spot_ 43	478.9287	32276.68	1.76283	16.53994	0.841706	0.790177	1.165875	0.095417	0.740745	0.635355	587.4946	4.159422	591.2723	5.225335	606.7174	19.46597	587.5	4.2	96.8	37
_19AR111_Spot_ 160	14.34609	604.4717	52919.62	11.91598	10.67089	0.790272	39.98745	0.095692	1.81424	0.04537	589.1126	10.21411	591.3261	181.1273	600.7519	NA	589.1	10.2	98.1	141
_19AR111_Spot_ 596	115.042	2865.3	1.236827	16.62758	1.27808	0.728548	4.486507	0.096077	0.925247	0.206229	591.3761	5.228214	555.7004	19.20289	413.0491	98.19607	591.4	5.2	143.2	509
_19AR111_Spot_ 204	207.4168	8200.653	6.186362	16.3538	1.39765	0.785522	2.492213	0.096282	0.875798	0.351414	592.5838	4.958444	588.6286	11.13335	574.3337	50.74203	592.6	5.0	103.2	182
_19AR111_Spot_ 406	199.5964	18014.02	2.160248	17.02688	1.33143	0.771229	1.931474	0.096744	0.910485	0.471394	595.2981	5.177369	580.4676	8.539599	523.7841	37.36383	595.3	5.2	113.7	355
_19AR111_Spot_ 207	255.4697	20453.61	7.676759	16.54402	1.152573	0.79564	1.641382	0.09684	0.727373	0.443147	595.8622	4.139863	594.3659	7.384897	589.624	31.90349	595.9	4.1	101.1	185
_19AR111_Spot_ 338	498.005	23785.42	22.88669	16.1377	0.828209	0.819998	1.267155	0.096997	0.909012	0.717364	596.7854	5.181315	608.0472	5.797037	651.1614	18.95112	596.8	5.2	91.6	297
_19AR111_Spot_ 119	145.7095	6935.529	1.122694	16.46674	1.657603	0.782556	2.08114	0.097014	0.814665	0.391451	596.8867	4.644299	586.9403	9.27717	549.6155	41.80285	596.9	4.6	108.6	104
_19AR111_Spot_ 396	46.95086	2228.881	5.61678	15.35525	4.34545	0.786086	5.718143	0.097479	0.866356	0.15151	599.6176	4.960544	588.9493	25.55903	549.0183	123.5394	599.6	5.0	109.2	346
_19AR111_Spot_ 120	124.34	6819.992	2.450452	16.92867	2.255365	0.763959	3.026231	0.097531	0.728535	0.24074	599.9244	4.173448	576.2917	13.30879	485.1147	64.85908	599.9	4.2	123.7	105
_19AR111_Spot_ 138	395.7877	20336.8	4.101041	16.61371	0.920263	0.806646	1.241721	0.098574	0.731912	0.589434	606.0492	4.23362	600.5706	5.629477	580.8903	21.7672	606.0	4.2	104.3	120
_19AR111_Spot_ 388	258.4623	2368.904	16.31413	14.97387	1.061792	0.825438	2.088539	0.098911	1.094214	0.523913	608.0211	6.348932	611.0779	9.589649	623.3434	38.34785	608.0	6.3	97.5	338
_19AR111_Spot_ 436	377.9	23417.26	5.990113	16.56327	0.972027	0.8197	1.260243	0.09947	0.762444	0.604998	611.3037	4.446684	607.8811	5.764266	596.088	21.71883	611.3	4.4	102.6	380
_19AR111_Spot_ 99	286.2822	11902.09	1.065932	16.6039	1.008018	0.811294	2.081585	0.099483	0.72784	0.349657	611.3773	4.245352	603.1794	9.467294	573.4462	42.39495	611.4	4.2	106.6	85
_19AR111_Spot_ 489	140.4582	8400.656	3.175003	16.2602	2.066607	0.822153	3.055894	0.099855	0.820139	0.268379	613.5577	4.799978	609.249	14.00114	594.1987	63.78953	613.6	4.8	103.3	423
_19AR111_Spot_ 547	303.2485	16955.87	3.58228	16.38061	0.90221	0.830855	1.482447	0.100293	0.773445	0.521736	616.1241	4.544741	614.0863	6.831038	607.4985	27.3483	616.1	4.5	101.4	473
_19AR111_Spot_ 522	171.5049	12037.11	0.93328	16.36774	1.554075	0.827415	1.898452	0.10041	0.726692	0.382781	616.8097	4.274545	612.1771	8.728235	596.0114	38.01295	616.8	4.3	103.5	449
_19AR111_Spot_ 325	33.19301	2477.663	2.172566	14.82786	5.093093	0.85715	7.572199	0.100497	1.339477	0.176894	617.3192	7.885276	628.5661	35.50085	670.1943	159.6838	617.3	7.9	92.1	287
_19AR111_Spot_ 407	205.6055	15511.07	1.833273	16.44025	1.10534	0.833014	1.426836	0.101061	0.679133	0.475971	620.6245	4.018329	615.2832	6.584105	596.5988	27.1641	620.6	4.0	104.0	356
_19AR111_Spot_ 97	453.3434	39530.32	1.103247	16.49343	0.767497	0.841734	1.118537	0.101204	0.736933	0.658837	621.4618	4.365931	620.1023	5.190773	616.0622	18.18945	621.5	4.4	100.9	83
_19AR111_Spot_ 316	367.5484	21397.99	1.375296	16.46181	0.826356	0.842821	1.512088	0.101309	0.912264	0.603314	622.0752	5.409754	620.7011	7.022086	616.6142	26.05002	622.1	5.4	100.9	279
_19AR111_Spot_ 46	458.1867	18742.59	1.597308	16.36934	0.901196	0.840952	1.375606	0.101342	0.74069	0.538447	622.2662	4.393601	619.6708	6.380553	611.1236	25.05083	622.3	4.4	101.8	40
_19AR111_Spot_ 274	218.6052	12113.4	1.888497	16.49016	1.037132	0.831478	1.453774	0.101585	0.532553	0.366325	623.6934	3.165881	614.432	6.701657	581.4118	29.37981	623.7	3.2	107.3	244
_19AR111_Spot_ 283	493.0891	21508.75	1.748376	16.24465	0.71616	0.854857	1.364968	0.102025	0.736086	0.53927	626.2627	4.392988	627.3114	6.387649	632.0526	24.76764	626.3	4.4	99.1	250
_19AR111_Spot_ 520	368.8386	20470.32	15.25743	16.02979	0.779086	0.866141	1.472606	0.102067	0.823158	0.55898	626.5108	4.914491	633.4696	6.940124	659.3337	26.17723	626.5	4.9	95.0	447
_19AR111_Spot_ 255	269.7994	24258.58	2.349256	16.23622	1.114473	0.856155	1.35205	0.102079	0.688494	0.509222	626.5783	4.110932	628.0216	6.332372	634.1795	25.07102	626.6	4.1	98.8	228
_19AR111_Spot_ 200	258.2609	7380.956	3.059578	16.06324	1.03215	0.845995	2.125246	0.102141	0.935822	0.440336	626.9459	5.590824	622.4483	9.889858	607.0529	41.24569	626.9	5.6	103.3	178
_19AR111_Spot_ 357	657.9471	45342.23	2.459253	16.45178	0.624342	0.850267	1.059618	0.102239	0.706876	0.667105	627.5157	4.226701	624.7957	4.944287	615.8782	17.046	627.5	4.2	101.9	314
_19AR111_Spot_ 466	573.0884	29496.15	5.058548	16.36711	0.810333	0.856228	1.190311	0.10232	0.83481	0.701338	627.9921	4.995278	628.0615	5.5751	629.2285	18.30178	628.0	5.0	99.8	406
_19AR111_Spot_ 254	175.902	7104.695	5.554891	16.28057	1.596165	0.836092	2.536217	0.102391	0.781932	0.308307	628.4033	4.68179	616.9868	11.72723	576.2415	52.45253	628.4	4.7	109.1	227
_19AR111_Spot_ 205	129.953	8886.252	3.487198	16.18065	2.272843	0.84687	2.614641	0.102435	0.975456	0.373074	628.6603	5.842777	622.9294	12.17427	603.0889	52.5097	628.7	5.8	104.2	183
_19AR111_Spot_ 79	44.25737	2344.538	1.099141	14.70916	4.330323	0.874801	8.005003	0.102584	0.945163	0.118072	629.5369	5.668848	638.171	37.9444	669.8061	170.3496	629.5	5.7	94.0	68
_19AR111_Spot_ 211	120.1505	7580.516	0.930214	16.13176	2.134607	0.849708	3.67974	0.102708	0.698778	0.189899	630.2603	4.195676	624.4885	17.16544	604.5546	78.1823	630.3	4.2	104.3	188
_19AR111_Spot_ 414	299.162	14860.8	5.400703	16.36555	0.885124	0.851546	1.172242	0.10296	0.641096	0.546897	631.7333	3.857896	625.4973	5.474255	603.9301	21.21391	631.7	3.9	104.6	362
_19AR111_Spot_ 117	312.3556	20459.64	5.358107	16.35269	1.019005	0.856668	1.270693	0.103026	0.721661	0.567927	632.1201	4.345239	628.3022	5.953246	615.5032	22.60681	632.1	4.3	102.7	102
_19AR111_Spot_ 202	355.6237	28901.55	2.751562	16.24073	1.037939	0.863674	1.4736	0.103074	0.937923	0.636485	632.3971	5.64975	632.1264	6.934194	632.1168	24.49384	632.4	5.6	100.0	180
_19AR111_Spot_ 137	313.8533	22401.96	6.046288	15.6204	0.878958	0.898813	1.342685	0.103175	0.967135	0.720299	632.9906	5.830913	651.093	6.453525	715.262	19.78788	633.0	5.8	88.5	119
_19AR111_Spot_ 455	186.3868	13259.19	0.981529	16.29158	1.19494	0.861324	1.830919	0.1034	0.716362	0.391258	634.302	4.327504	630.8454	8.60308	619.3944	36.38558	634.3	4.3	102.4	397
_19AR111_Spot_ 193	46.49617	3756.53	5.971041	15.44766	3.514247	0.866774	6.330235	0.103421	0.892044	0.140918	634.4275	5.389801	633.8142	29.85307	632.585	135.0507	634.4	5.4	100.3	171
_19AR111_Spot_ 439	119.1113	9681.288	2.749913	16.43308	1.61525	0.852399	2.01182	0.103918	0.708	0.35192	637.3312	4.296419	625.9652	9.40026	586.0677	40.87005	637.3	4.3	108.7	382
_19AR111_Spot_ 526	207.2317	14161.7	1.344236	16.23965	1.304196	0.868182	1.90047	0.104052	0.869136	0.457327	638.11	5.280389	634.5796	8.967964	622.9435	36.46521	638.1	5.3	102.4	453
_19AR111_Spot_ 25	11.90741	740.5495	28.76409	11.85197	9.535542	0.93506	23.4617	0.104088	2.142817	0.091333	638.3199	13.02266	670.2933	115.6127	780.1987	497.4528	638.3	13.0	81.8	20
_19AR111_Spot_ 123	253.762	17603.4	3.318569	16.38352	1.07022	0.865987	1.497565	0.104586	0.814916	0.544161	641.229	4.973996	633.386	7.057087	606.4143	27.19386	641.2	5.0	105.7	108
_19AR111_Spot_ 480	511.5642	31772.23	1.787457	16.33408	0.635762	0.877359	1.076815	0.104714	0.71093	0.660216	641.9737	4.344088	639.5554	5.109808	631.9802	17.42933	642.0	4.3	101.6	418
_19AR111_Spot_ 569	664.7243	43529.32	17.78087	15.63231	0.673227	0.92172	1.333259	0.104971	1.071877	0.803953	643.4758	6.564203	663.2691	6.493205	732.0825	16.78655	643.5	6.6	87.9	493
_19AR111_Spot_ 67	295.3558	21611.48	8.44498	16.56287	0.83231	0.864069	1.185161	0.10507	0.72145	0.608736	644.0518	4.42194	632.3415	5.578251	591.6383	20.37759	644.1	4.4	108.9	58
_19AR111_Spot_ 591	693.6597	25981.66	6.390612	15.97345	0.6635	0.902218	1.033908	0.105588	0.773668	0.748295	647.0717	4.763135	652.9124	4.979281	674.0696	14.6684	647.1	4.8	96.0	505
_19AR111_Spot_ 155	236.2619	12985.79	10.92997	15.66646	1.220565	0.919289	3.666126	0.10655	3.409959	0.930126	652.68	21.1666	661.9838	17.83174	694.7319	28.6912	652.7	21.2	93.9	136
_19AR111_Spot_ 313	203.2383	12473.46	5.050319	15.99466	1.070517	0.928808	1.64448	0.109294	0.934512	0.568272	668.6441	5.93542	667.0071	8.040895	662.3921	28.98133	668.6	5.9	100.9	276
_19AR111_Spot_ 44	294.7126	20332.76	2.648684	15.90588	0.943702	0.935186	1.199365	0.109358	0.62958	0.524928	669.021	4.000829	670.3594	5.885203	675.7805	21.81096	669.0	4.0	99.0	38
_19AR111_Spot_ 13	604.6431	54412.23	4.875805	16.10427	0.815084	0.939088	1.264906	0.109967	0.94924	0.750443	672.5546	6.062416	672.4047	6.220174	672.8127	17.89852	672.6	6.1	100.0	10
_19AR111_Spot_ 214	274.3856	21706.08	1.48263	16.03946	0.891128	0.936249	1.355309	0.11019	0.777148	0.57341	673.849	4.972392	670.9169	6.654335	661.9962	23.79359	673.8	5.0	101.8	191
_19AR111_Spot_ 599	196.462	11087.74	4.383485	14.88123	1.097694	1.011031	2.365624	0.111388	0.930381	0.393292	680.803	6.011063	709.3947	12.07652	801.8731	45.57092	680.8	6.0	84.9	511
_19AR111_Spot_ 170	229.7401	17468.6	23.7161	15.77953	1.376098	0.98512	1.782756	0.114442	0.688246	0.386058	698.4943	4.556071	696.2271	8.983289	689.8225	35.08204	698.5	4.6	101.3	151
_19AR111_Spot_ 45	121.5159	8944.495	1.567814	15.57631	1.649885	0.990277	1.971467	0.115009	0.633845	0.321509	701.7746	4.214594	698.8618	9.960398	690.4351	39.82601	701.8	4.2	101.6	39
_19AR111_Spot_ 399	136.4528	8139.8	1.209003	15.4178	1.637245	1.014861	2.438373	0.116925	0.911067	0.373637	712.8431	6.148275	711.3267	12.47135	707.4494	48.09423	712.8	6.1	100.8	348
_19AR111_Spot_ 212	368.0382	5259.917	3.241475	14.96364	0.718587	1.032893	1.210381	0.117073	0.72149	0.596085	713.6934	4.874423	720.3738	6.244509	742.1327	20.55435	713.7	4.9	96.2	189
_19AR111_Spot_ 454	188.3314	23138.06	2.252096	15.49096	1.307244	1.050134	1.593983	0.118886	0.774138	0.485662	724.1466	5.302495	728.9488	8.290598	744.6437	29.43891	724.1	5.3	97.2	396
_19AR111_Spot_ 135	127.585	2988.941	3.423815	14.3431	1.412551	1.061809	4.054875	0.119009	1.000024	0.246623	724.8588	6.856083	734.7147	21.20648	765.7849	82.81997	724.9	6.9	94.7	118
_19AR111_Spot_ 448	617.417	3863.719	6.159382	14.90371	0.75785	1.050754	5.695904	0.119784	1.48489	0.260694	729.3184	10.23944	729.2556	29.64172	730.0056	116.6091	729.3	10.2	99.9	391
_19AR111_Spot_ 527	111.3565	8429.021	2.368438	15.5017	1.645575	1.041289	2.874382	0.120343	0.763324	0.265561	732.5403	5.28565	724.5587	14.88923	700.8427	58.99386	732.5	5.3	104.5	454
_19AR111_Spot_ 219	227.8009	18760.37	3.692656	15.44363	1.099169	1.06668	1.5035	0.120845	0.809111	0.538152	735.426	5.623541	737.1106	7.879578	743.133	26.79731	735.4	5.6	99.0	195
_19AR111_Spot_ 222	333.2508	20334.3	3.379666	15.50842	0.874355	1.063389	1.272543	0.120871	0.852417	0.669853	735.5771	5.92568	735.4922	6.659164	736.1759	20.00207	735.6	5.9	99.9	198
_19AR111_Spot_ 434	280.3131	18859.78	1.509488	15.71371	1.059494	1.04818	1.604829	0.120889	0.671356	0.418335	735.6757	4.667599	727.9806	8.339428	705.2741	31.03632	735.7	4.7	104.3	378
_19AR111_Spot_ 68	114.725	10966.04	4.716069	15.17413	1.735397	1.076194	2.583199	0.120936	0.77579	0.300322	735.9466	5.395557	741.7743	13.59679	760.3044	51.9848	735.9	5.4	96.8	59
_19AR111_Spot_ 534	813.0012	12500.1	89.69481	15.42236	0.711641	1.072591	1.090732	0.122355	0.81462	0.746857	744.1068	5.72488	740.0109	5.731563	728.5778	15.35374	744.1	5.7	102.1	461
_19AR111_Spot_ 284	97.77396	8492.093	2.433567	15.46568	2.083013	1.059873	2.681427	0.122416	0.617025	0.230111	744.4522	4.338147	733.7607	14.00995	702.1962	55.53668	744.5	4.3	106.0	251
_19AR111_Spot_ 442	184.3366	17953.08	1.770657	15.60211	1.279766	1.075122	1.725706	0.122923	0.802001	0.464738	747.3646	5.659469	741.25	9.078676	723.7259	32.40106	747.4	5.7	103.3	385
_19AR111_Spot_ 4	981.341	6313.473	15.06342	14.64211	0.613808	1.12	1.161149	0.122992	0.887483	0.764314	747.7599	6.265811	762.9752	6.228816	808.698	15.67684	747.8	6.3	92.5	2
_19AR111_Spot_ 542	381.2864	42420.25	12.21998	15.41325	0.579732	1.09667	0.973558	0.123672	0.663438	0.681457	751.6619	4.707053	751.7392	5.170604	752.8678	15.04261	751.7	4.7	99.8	468
_19AR111_Spot_ 217	246.2849	6634.972	6.051153	14.09691	1.081489	1.18305	2.06876	0.124865	1.095503	0.529546	758.5055	7.83922	792.7329	11.38407	891.1572	36.23483	758.5	7.8	85.1	194
_19AR111_Spot_ 571	281.2711	23199.14	3.055952	15.52304	0.987684	1.103682	1.261452	0.125378	0.742777	0.588827	761.4425	5.334565	755.1293	6.720024	737.4182	21.57852	761.4	5.3	103.3	495
_19AR111_Spot_ 432	227.8615	13595.34	6.352446	15.23424	0.978896	1.115889	1.926083	0.125624	1.005153	0.521863	762.8545	7.231536	761.0045	10.3145	756.4718	34.66939	762.9	7.2	100.8	377
_19AR111_Spot_ 173	638.8283	7467.821	15.72283	14.40789	0.674772	1.172219	1.50811	0.12625	0.717221	0.475576	766.4372	5.182843	787.6825	8.26376	849.2059	27.56146	766.4	5.2	90.3	154
_19AR111_Spot_ 336	337.7816	32886.13	8.606063	14.89363	0.807111	1.178237	1.224658	0.128118	0.896029	0.731657	777.1219	6.559895	790.4917	6.726334	829.319	17.40628	777.1	6.6	93.7	296
_19AR111_Spot_ 63	124.1631	9988.03	2.038229	15.2443	1.238186	1.145118	1.937283	0.129585	0.69352	0.357986	785.4964	5.128762	774.9347	10.50116	745.5233	38.21894	785.5	5.1	105.4	55
_19AR111_Spot_ 421	534.5926	52925.63	12.3367	14.29778	0.858418	1.252737	1.56474	0.130871	1.281421	0.818935	792.8324	9.559619	824.6391	8.835526	912.2683	18.46832	792.8	9.6	86.9	368
_19AR111_Spot_ 59	138.2589	12626.12	3.416257	14.64788	1.161564	1.214644	1.773271	0.131513	0.904065	0.509829	796.4895	6.773704	807.3224	9.875605	838.2309	31.79125	796.5	6.8	95.0	51
_19AR111_Spot_ 156	101.5322	9958.58	3.69681	14.95835	1.717256	1.185527	2.100231	0.131759	0.883726	0.420776	797.89	6.632252	793.8842	11.56834	783.548	40.04959	797.9	6.6	101.8	137
_19AR111_Spot_ 129	461.4943	34226.87	6.628521	15.00893	0.823913	1.205038	1.340507	0.132269	0.943634	0.703938	800.7971	7.10609	802.9088	7.438602	809.6618	19.9277	800.8	7.1	98.9	113
_19AR111_Spot_ 166	387.6899	28907.48	4.072404	14.47143	0.834585	1.24982	1.406559	0.132422	1.090341	0.775183	801.6664	8.219248	823.3236	7.934078	883.1926	18.35544	801.7	8.2	90.8	147
_19AR111_Spot_ 64	96.23809	10481.25	1.559711	15.23018	1.657718	1.174989	1.878928	0.132698	0.748085	0.398144	803.2409	5.649635	788.9765	10.30699	749.7672	36.40657	803.2	5.6	107.1	56
_19AR111_Spot_ 446	138.8345	12700.25	1.093818	15.33716	1.154849	1.181788	1.951703	0.133249	0.905858	0.464137	806.3735	6.866206	792.1459	10.73462	753.2085	36.49831	806.4	6.9	107.1	389
_19AR111_Spot_ 323	251.7855	11451.85	2.543589	14.45375	1.076676	1.257989	1.686142	0.133997	0.771763	0.457709	810.6293	5.878768	827.0037	9.538757	872.1622	31.03631	810.6	5.9	92.9	286
_19AR111_Spot_ 187	384.4015	46165.76	2.745848	14.67278	0.702886	1.251623	1.058419	0.134148	0.719488	0.679776	811.4848	5.486	824.137	5.974072	859.3106	16.11418	811.5	5.5	94.4	166
_19AR111_Spot_ 195	235.5438	1714.193	4.913866	13.42641	0.953288	1.239507	9.419763	0.136384	0.787127	0.083561	824.1837	6.089786	818.6586	52.98592	804.5658	196.9397	824.2	6.1	102.4	173
_19AR111_Spot_ 122	83.9066	6515.694	2.090932	14.61951	1.645116	1.247758	2.812208	0.137019	0.876023	0.311507	827.7834	6.805294	822.3926	15.85234	808.7336	55.94324	827.8	6.8	102.4	107
_19AR111_Spot_ 60	64.92154	4427.638	1.049371	14.48535	3.132202	1.249989	3.471058	0.138101	0.880569	0.253689	833.9174	6.888088	823.3999	19.58265	795.9942	70.43274	833.9	6.9	104.8	52
_19AR111_Spot_ 80	236.8959	20268.65	8.816228	14.13932	1.169856	1.341178	2.084912	0.138867	1.549178	0.743043	838.2513	12.17715	863.74	12.12803	930.5922	28.64251	838.3	12.2	90.1	69
_19AR111_Spot_ 477	130.8231	11184.34	2.572245	14.1742	0.938211	1.328963	1.826073	0.139138	0.812933	0.445181	839.789	6.400943	858.4281	10.58069	907.7594	33.68455	839.8	6.4	92.5	416
_19AR111_Spot_ 174	66.88123	6611.725	3.802802	14.46866	1.447094	1.313144	2.332731	0.142657	0.991602	0.425082	859.6724	7.980581	851.508	13.44713	831.1836	44.0342	859.7	8.0	103.4	155
_19AR111_Spot_ 203	181.8597	2261.972	4.442847	13.37193	1.180157	1.365987	3.4135	0.14524	1.467558	0.429928	874.2233	11.99783	874.443	20.01337	875.8894	63.81643	874.2	12.0	99.8	181
_19AR111_Spot_ 331	59.55805	6036.401	1.669389	14.07368	2.279167	1.484906	4.053421	0.15657	0.78287	0.193138	937.6847	6.831936	924.2371	24.59946	893.1845	82.13379	893.2	82.1	105.0	292

_19AR111_Spot_ 83	184.8587	23106.14	2.634518	14.34139	1.107423	1.453183	1.518685	0.152528	0.677601	0.446176	915.1209	5.780847	911.1911	9.134816	902.5593	28.02066	902.6	28.0	101.4	72
_19AR111_Spot_ 261	310.8841	4312.862	4.170613	13.66222	0.879663	1.434385	1.91148	0.14914	0.730975	0.382413	896.1395	6.115637	903.3803	11.43653	922.0442	36.2954	922.0	36.3	97.2	233
_19AR111_Spot_ 412	53.94093	5320.602	4.334549	14.22849	2.078654	1.432294	2.985519	0.153947	0.732057	0.245203	923.0521	6.295775	902.508	17.85298	853.4702	60.16342	923.1	6.3	108.2	361
_19AR111_Spot_ 378	170.3561	18844.13	3.650244	14.1248	1.187184	1.522983	1.433683	0.157873	0.729367	0.508736	944.9476	6.410798	939.6779	8.787689	928.2426	25.35314	928.2	25.4	101.8	328
_19AR111_Spot_ 411	277.0106	22760.64	3.843744	14.12469	0.839105	1.423114	1.687182	0.147411	1.322044	0.783581	886.4331	10.94901	898.6685	10.06171	929.7928	21.51996	929.8	21.5	95.3	360
_19AR111_Spot_ 243	271.5512	30364.51	2.617445	14.12825	0.883427	1.53825	1.228867	0.158847	0.742456	0.60418	950.3669	6.560585	945.8038	7.561977	936.0793	20.06401	936.1	20.1	101.5	218
_19AR111_Spot_ 506	154.1093	9326.837	4.283085	13.97639	1.078669	1.664941	2.328696	0.171911	0.791038	0.339691	1022.631	7.480394	995.26	14.77354	936.2963	44.94056	936.3	44.9	109.2	435
_19AR111_Spot_ 47	199.701	13569.3	2.646889	13.98281	0.771537	1.535455	1.332971	0.158391	0.672995	0.504883	947.8313	5.93207	944.6852	8.19674	938.2312	23.58949	938.2	23.6	101.0	41
_19AR111_Spot_ 523	128.6143	12812.32	3.15316	13.95458	1.111853	1.5176	1.383018	0.156211	0.802087	0.579954	935.6879	6.985781	937.5095	8.465221	942.6818	23.08299	942.7	23.1	99.3	450
_19AR111_Spot_ 251	515.6905	7672.552	5.643408	13.79057	0.672749	1.486879	2.568399	0.153046	0.747796	0.291152	918.0177	6.398492	925.0427	15.59364	942.7084	50.36539	942.7	50.4	97.4	224
_19AR111_Spot_ 465	346.7883	33601.45	2.778123	14.0911	0.881661	1.454539	1.329331	0.149341	0.955647	0.718893	897.2691	8.004727	911.7521	7.998852	947.8574	18.92697	947.9	18.9	94.7	405
_19AR111_Spot_ 403	282.6976	37328.25	4.092602	14.04574	0.731108	1.560587	1.069612	0.160132	0.756111	0.706902	957.5083	6.727807	954.7003	6.619282	949.1393	15.49569	949.1	15.5	100.9	352
_19AR111_Spot_ 215	89.40268	7526.913	4.093135	13.75876	1.744471	1.576908	2.394616	0.161721	0.948237	0.395987	966.3363	8.509443	961.1516	14.88005	950.1805	45.00632	950.2	45.0	101.7	192
_19AR111_Spot_ 564	317.2099	34598.62	3.915573	14.04508	0.835061	1.47135	1.495086	0.150675	1.203121	0.804717	904.7444	10.15584	918.6825	9.038351	953.2194	18.15513	953.2	18.2	94.9	489
_19AR111_Spot_ 307	84.19936	9714.846	2.329804	13.86259	1.382232	1.547866	1.862602	0.158257	0.8166	0.438419	947.082	7.192588	949.6434	11.49014	956.4564	34.22668	956.5	34.2	99.0	272
_19AR111_Spot_ 57	78.53772	8132.214	2.739329	13.70404	1.319445	1.686033	2.42699	0.172232	0.850418	0.3504	1024.398	8.054743	1003.265	15.46986	958.2463	46.46513	958.2	46.5	106.9	50
_19AR111_Spot_ 151	42.79326	3563.492	0.916585	13.29924	2.053674	1.698821	2.665065	0.173295	0.865385	0.324715	1030.239	8.239604	1008.088	17.03539	961.1572	51.50413	961.2	51.5	107.2	133
_19AR111_Spot_ 301	76.23684	6723.082	2.858133	13.62152	1.649013	1.682386	2.489565	0.171608	0.730476	0.293415	1020.967	6.897324	1001.885	15.85598	961.2744	48.62848	961.3	48.6	106.2	266
_19AR111_Spot_ 242	116.3866	3875.286	3.489079	13.35463	1.246109	1.470665	1.646591	0.149973	0.791218	0.480519	900.8125	6.651813	918.401	9.952435	961.8074	29.50031	961.8	29.5	93.7	217
_19AR111_Spot_ 483	110.8558	15122.43	2.681119	13.87192	1.082227	1.533752	1.812143	0.156352	1.326194	0.731838	936.473	11.55951	944.0027	11.1386	962.5154	25.20551	962.5	25.2	97.3	421
_19AR111_Spot_ 27	83.89355	10016.7	1.608303	13.81345	2.047176	1.547099	2.768241	0.157604	0.715907	0.258614	943.4454	6.283203	949.3377	17.07447	963.9315	54.61596	963.9	54.6	97.9	22
_19AR111_Spot_ 289	195.4748	19433.99	7.780168	13.84989	0.717013	1.619371	1.10427	0.164829	0.79143	0.716699	983.559	7.219431	977.7471	6.932046	965.622	15.73669	965.6	15.7	101.9	256
_19AR111_Spot_ 140	269.8106	20720.79	2.161882	13.85363	0.71871	1.559867	1.06855	0.158583	0.729357	0.682567	948.8983	6.435593	954.4146	6.611518	968.037	15.94813	968.0	15.9	98.0	122
_19AR111_Spot_ 131	65.31163	9501.904	2.067199	13.70066	1.32172	1.722329	1.719689	0.175083	0.930186	0.540904	1040.057	8.934376	1016.894	11.04772	968.2185	29.51935	968.2	29.5	107.4	115
_19AR111_Spot_ 339	392.1126	7310.408	3.617915	13.6343	0.797672	1.563897	1.28564	0.15889	0.894266	0.69558	950.6076	7.903882	956.0119	7.962801	969.3744	18.84629	969.4	18.8	98.1	298
_19AR111_Spot_ 502	71.17282	8437.107	1.069876	13.74022	1.62165	1.643547	2.563177	0.166867	0.718853	0.280454	994.8285	6.626868	987.0758	16.1823	970.7643	50.17668	970.8	50.2	102.5	432
_19AR111_Spot_ 52	221.9854	13616	0.705642	13.77196	0.909162	1.483352	1.337503	0.150533	0.853758	0.638323	903.9491	7.200877	923.602	8.112224	971.7003	20.99093	971.7	21.0	93.0	45
_19AR111_Spot_ 257	68.79545	5257.833	3.010907	13.43869	1.706882	1.686935	2.294017	0.171065	0.700066	0.30517	1017.974	6.592291	1003.606	14.62506	973.2661	44.54712	973.3	44.5	104.6	230
_19AR111_Spot_ 332	175.8264	4578.821	3.820351	13.4083	0.931858	1.643271	1.676975	0.166522	0.657116	0.391846	992.9211	6.046992	986.9697	10.58619	974.6662	31.45676	974.7	31.5	101.9	293
_19AR111_Spot_ 299	94.70624	3517.779	2.180901	13.16742	0.994916	1.754529	4.214823	0.177435	0.78581	0.18644	1052.945	7.633744	1028.833	27.26633	978.7794	84.41478	978.8	84.4	107.6	265
_19AR111_Spot_ 366	237.812	8664.414	3.093379	13.59789	0.755195	1.666789	1.104349	0.168515	0.755323	0.683953	1003.926	7.021926	995.9638	7.008658	979.3378	16.41238	979.3	16.4	102.5	322
_19AR111_Spot_ 464	255.5172	14982.71	5.749577	13.7531	0.953407	1.5483	1.598891	0.156271	0.91586	0.572809	936.0196	7.979317	949.816	9.864312	982.8293	26.68657	982.8	26.7	95.2	404
_19AR111_Spot_ 322	114.6808	16535.84	3.616305	13.7516	1.177921	1.676291	1.417895	0.169133	0.741446	0.52292	1007.333	6.914521	999.5756	9.01782	983.5056	24.60696	983.5	24.6	102.4	285
_19AR111_Spot_ 61	88.9726	9003.752	1.220753	13.57798	1.302074	1.77754	2.060681	0.179285	0.697978	0.338712	1063.069	6.840468	1037.28	13.39137	984.2028	39.45495	984.2	39.5	108.0	53
_19AR111_Spot_ 297	26.25799	2565.167	3.782012	12.87221	3.974039	1.693057	6.209123	0.170711	0.948601	0.152775	1016.027	8.916892	1005.917	39.65582	984.8231	125.017	984.8	125.0	103.2	264
_19AR111_Spot_ 495	362.2864	62903.56	2.209564	13.86533	0.691236	1.572982	1.132438	0.158552	0.882002	0.778852	948.7234	7.781147	959.6034	7.029724	985.4972	14.44495	985.5	14.4	96.3	426
_19AR111_Spot_ 161	90.27465	7788.321	3.291838	13.53216	1.331761	1.750039	1.801716	0.176333	0.881952	0.489506	1046.909	8.522477	1027.177	11.64244	986.2498	31.975	986.2	32.0	106.2	142
_19AR111_Spot_ 537	231.5081	31922.65	2.603122	13.78083	0.720346	1.640663	1.092214	0.165282	0.703688	0.644277	986.0619	6.434169	985.9676	6.890501	986.6622	16.97948	986.7	17.0	99.9	463
_19AR111_Spot_ 101	212.3192	26175.89	2.103284	13.79692	0.847227	1.608574	1.202154	0.162042	0.695454	0.578506	968.1152	6.251622	973.5532	7.527244	986.7495	19.93699	986.7	19.9	98.1	87
_19AR111_Spot_ 105	88.71597	10492	2.776984	13.60423	1.358686	1.723939	1.758749	0.173382	0.823213	0.468067	1030.72	7.841438	1017.494	11.30254	990.0074	31.61307	990.0	31.6	104.1	91
_19AR111_Spot_ 133	112.5055	13197.6	3.379474	13.61507	1.357693	1.740442	1.78396	0.174901	0.883688	0.495352	1039.056	8.480229	1023.627	11.50463	991.6756	31.49478	991.7	31.5	104.8	117
_19AR111_Spot_ 72	87.02748	10089.04	2.730329	13.57474	1.459012	1.742284	1.777754	0.174932	0.735461	0.413702	1039.231	7.058874	1024.309	11.46902	993.4717	32.88436	993.5	32.9	104.6	62
_19AR111_Spot_ 228	169.7401	16148.03	2.654935	13.66469	1.018404	1.677389	1.389347	0.16817	0.794772	0.572047	1002.021	7.375707	999.992	8.838405	996.4103	23.1362	996.4	23.1	100.6	204
_19AR111_Spot_ 103	80.24764	3269.127	1.735086	13.03185	1.215463	1.783821	2.499089	0.178836	0.761891	0.304867	1060.61	7.45095	1039.574	16.26143	996.5014	48.36786	996.5	48.4	106.4	89
_19AR111_Spot_ 172	87.2245	9425.633	3.044413	13.51546	1.545409	1.730959	2.241453	0.173534	0.803743	0.358581	1031.553	7.66169	1020.107	14.42648	996.5181	42.51997	996.5	42.5	103.5	153
_19AR111_Spot_ 294	263.3664	22759.96	1.557745	13.64411	0.774857	1.629303	1.123357	0.16333	0.774127	0.689119	975.2585	7.006395	981.5901	7.068317	996.6763	16.54086	996.7	16.5	97.9	261
_19AR111_Spot_ 191	368.0221	4766.212	1.676717	13.23652	0.794542	1.74985	3.744037	0.175193	0.83858	0.223977	1040.66	8.0588	1027.107	24.19602	999.2126	74.14243	999.2	74.1	104.1	169
_19AR111_Spot_ 470	66.20039	7734.756	4.182611	13.45991	1.207201	1.65471	2.622764	0.16549	1.391365	0.530495	987.2153	12.73572	991.3545	16.60094	1001.391	45.16606	1001.4	45.2	98.6	410
_19AR111_Spot_ 341	253.3702	38086.49	3.616169	13.71665	0.805629	1.686563	1.335092	0.168667	1.031813	0.772841	1004.763	9.599732	1003.465	8.510533	1001.496	17.19405	1001.5	17.2	100.3	300
_19AR111_Spot_ 398	290.5169	27773.56	8.609961	13.65649	0.645349	1.696872	1.121868	0.169633	0.898529	0.800923	1010.09	8.400634	1007.354	7.167496	1002.271	13.63495	1002.3	13.6	100.8	347
_19AR111_Spot_ 143	168.2048	16587.28	2.933026	13.59032	1.015598	1.729173	1.356951	0.172826	0.855144	0.630196	1027.663	8.123318	1019.443	8.729957	1002.698	21.36924	1002.7	21.4	102.5	125
_19AR111_Spot_ 171	366.1312	35480.11	1.380719	13.66149	0.694279	1.667131	1.011084	0.166403	0.692754	0.68516	992.2617	6.371035	996.0943	6.41724	1005.407	14.92592	1005.4	14.9	98.7	152
_19AR111_Spot_ 95	90.84369	10336.41	0.916215	13.51892	1.625213	1.754341	2.069922	0.175107	0.810922	0.391765	1040.187	7.789746	1028.764	13.38768	1005.416	38.63556	1005.4	38.6	103.5	81
_19AR111_Spot_ 565	102.9468	11170.2	4.32619	13.52517	0.953626	1.787218	1.344891	0.178299	0.720567	0.535781	1057.676	7.028879	1040.812	8.756578	1006.433	23.02097	1006.4	23.0	105.1	490
_19AR111_Spot_ 245	390.4458	36428.04	3.641015	13.62755	0.60966	1.678202	0.97104	0.167115	0.646337	0.665614	996.1947	5.965938	1000.3	6.178359	1010.171	14.69003	1010.2	14.7	98.6	220
_19AR111_Spot_ 558	129.7242	6376.543	3.200053	13.29339	1.176275	1.703237	2.256792	0.169505	0.850039	0.376658	1009.385	7.942153	1009.748	14.43916	1011.398	42.3867	1011.4	42.4	99.8	484
_19AR111_Spot_ 419	113.1092	13718.76	2.192379	13.47963	1.506111	1.730501	1.780646	0.172187	0.863303	0.484826	1024.148	8.174941	1019.937	11.45923	1011.772	31.57296	1011.8	31.6	101.2	366
_19AR111_Spot_ 253	288.6615	45331.01	3.64969	13.6225	0.590849	1.703079	0.96839	0.169444	0.651947	0.673228	1009.049	6.089456	1009.688	6.195291	1011.938	14.51494	1011.9	14.5	99.7	226
_19AR111_Spot_ 178	118.4418	9471.668	2.4869	13.40943	1.022367	1.741458	1.768586	0.173258	1.384097	0.782601	1030.036	13.17604	1024.004	11.4079	1011.996	22.31921	1012.0	22.3	101.8	158
_19AR111_Spot_ 504	42.3587	4980.743	3.988062	13.23897	3.305489	1.618337	4.42358	0.160989	0.891431	0.201518	962.2687	7.968438	977.3463	27.7687	1012.248	87.86865	1012.2	87.9	95.1	434
_19AR111_Spot_ 554	487.7498	56307.36	3.305506	13.62498	0.757728	1.72937	1.144624	0.172022	0.834755	0.729283	1023.24	7.898135	1019.517	7.364207	1012.394	15.8718	1012.4	15.9	101.1	480
_19AR111_Spot_ 409	200.4191	20645.13	2.883439	13.54726	0.840448	1.667958	1.385038	0.165789	0.66985	0.483633	988.8659	6.140885	996.4091	8.792425	1013.911	24.56834	1013.9	24.6	97.5	358
_19AR111_Spot_ 551	216.9221	21501.72	3.085407	13.54944	0.800946	1.702669	1.147035	0.169228	0.789556	0.688345	1007.854	7.36671	1009.534	7.337558	1014.045	16.86151	1014.0	16.9	99.4	477
_19AR111_Spot_ 8	554.7146	23407.74	2.661583	13.60251	0.61742	1.661535	1.014397	0.165116	0.726764	0.716449	985.1432	6.639435	993.9616	6.430151	1014.337	14.3407	1014.3	14.3	97.1	6
_19AR111_Spot_ 532	201.0575	22442.58	2.849485	13.55565	0.7461	1.715406	1.354464	0.170381	0.750955	0.55443	1014.21	7.047359	1014.308	8.688405	1015.383	22.83564	1015.4	22.8	99.9	459
_19AR111_Spot_ 379	272.9802	32861.61	1.51863	13.5823	0.78094	1.733615	1.476984	0.172084	1.179235	0.798408	1023.584	11.16095	1021.095	9.511166	1016.624	18.01529	1016.6	18.0	100.7	329
_19AR111_Spot_ 369	600.0735	76974.31	12.49604	13.64356	0.582671	1.715788	0.931504	0.1703	0.708064	0.76013	1013.762	6.642137	1014.451	5.975681	1016.802	12.26089	1016.8	12.3	99.7	324
_19AR111_Spot_ 279	98.47554	12475.61	1.717945	13.43749	1.457761	1.804601	1.768786	0.179087	0.718255	0.406072	1061.984	7.032584	1047.125	11.55671	1017.118	32.7531	1017.1	32.8	104.4	248
_19AR111_Spot_ 152	163.0066	19893.74	4.717487	13.53736	0.949854	1.71103	1.471073	0.169796	0.960183	0.65271	1010.988	8.98443	1012.67	9.42757	1017.174	22.56692	1017.2	22.6	99.4	134
_19AR111_Spot_ 328	138.907	15428.14	2.546115	13.55251	1.027579	1.723476	1.614158	0.171017	0.852121	0.527905	1017.712	8.022242	1017.321	10.37224	1017.344	27.78776	1017.3	27.8	100.0	290
_19AR111_Spot_ 349	253.0072	16388.61	16.17359	13.50066	0.823299	1.734524	1.294021	0.172056	0.820512	0.634079	1023.428	7.764691	1021.432	8.334503	1018.021	20.26677	1018.0	20.3	100.5	308
_19AR111_Spot_ 401	386.5784	41459.21	1.437064	13.57628	0.653826	1.732498	1.041704	0.171826	0.736857	0.707358	1022.163	6.965095	1020.68	6.706459	1018.362	14.91241	1018.4	14.9	100.4	350
_19AR111_Spot_ 311	200.8685	17911.41	4.316537	13.56875	0.759227	1.762939	1.203689	0.174605	0.821872	0.682794	1037.436	7.875681	1031.929	7.79864	1021.137	17.80291	1021.1	17.8	101.6	275
_19AR111_Spot_ 402	273.3636	28668.81	1.967354	13.5164	0.697042	1.728981	1.158654	0.171072	0.725809	0.626425	1018.016	6.834971	1019.372	7.453856	1023.147	18.27798	1023.1	18.3	99.5	351
_19AR111_Spot_ 503	85.60111	14100.1	2.503499	13.49524	0.91191	1.783685	1.289524	0.176428	0.815454	0.632368	1047.43	7.883513	1039.525	8.390103	1023.801	20.21398	1023.8	20.2	102.3	433
_19AR111_Spot_ 159	423.294	40587.1	1.928101	13.55535	0.797688	1.754023	1.164358	0.173478	0.833009	0.715423	1031.246	7.93849	1028.647	7.529953	1023.984	16.46138	1024.0	16.5	100.7	140
_19AR111_Spot_ 404	100.5699	11038.9	0.774147	13.3728	1.592008	1.765413	2.032586	0.174552	0.809033	0.398031	1037.141	7.750615	1032.838	13.17618	1024.599	37.74251	1024.6	37.7	101.2	353
_19AR111_Spot_ 5	111.8888	12233.65	2.721463	13.42211	0.967868	1.792694	1.450198	0.177214	0.66291	0.457117	1051.738	6.433042	1042.805	9.452626	1024.994	26.11379	1025.0	26.1	102.6	3
_19AR111_Spot_ 42	332.7909	39076.14	3.394052	13.57368	0.62732	1.668129	1.123386	0.164897	0.814973	0.725461	983.9338	7.436817	996.4742	7.131634	1025.038	15.64281	1025.0	15.6	96.0	36
_19AR111_Spot_ 586	82.6829	9808.958	2.559324	13.37169	1.512952	1.817932	2.118361	0.179697	0.780637	0.36851	1065.321	7.665472	1051.94	13.87727	1025.127	39.84901	1025.1	39.8	103.9	500
_19AR111_Spot_ 343	184.5753	24506.81	1.905685	13.51979	0.920811	1.795372	1.239126	0.177457	0.782713	0.631666	1053.066	7.604463	1043.779	8.081079	1025.249	19.43425	1025.2	19.4	102.7	302
_19AR111_Spot_ 428	149.3487	21954.07	2.537154	13.47715	1.172844	1.784338	1.651155	0.176295	1.01276	0.613365	1046.7	9.784717	1039.763	10.74457	1026.068	26.39958	1026.1	26.4	102.0	373
_19AR111_Spot_ 292	482.8674	16732.08	5.3897	13.42377	0.627723	1.765702	1.366279	0.174414	0.713712	0.522377	1036.385	6.832844	1032.944	8.857117	1026.525	23.5852	1026.5	23.6	101.0	259
_19AR111_Spot_ 265	153.0295	19027.15	1.828888	13.44242	0.937265	1.794021	1.274701	0.177208	0.799729	0.627386	1051.704	7.760529	1043.287	8.310854	1026.561	20.09683	1026.6	20.1	102.4	236
_19AR111_Spot_ 295	108.3888	10435.34	1.246997	13.33198	0.969794	1.766387	1.686689	0.174446	0.735426	0.436017	1036.56	7.041818	1033.195	10.9359	1026.939	30.72037	1026.9	30.7	100.9	262
_19AR111_Spot_ 327	583.9474	19515.57	3.296713	13.53023	0.620414	1.731994	0.95243	0.170962	0.70458	0.73977	1017.409	6.631397	1020.492	6.131053	1027.974	12.95943	1028.0	13.0	99.0	289
_19AR111_Spot_ 326	338.262	40151.88	7.967091	13.5824	0.742421	1.729062	1.05943	0.170461	0.744482	0.70272	1014.648	6.989401	1019.402	6.815628	1030.484	15.25627	1030.5	15.3	98.5	288
_19AR111_Spot_ 496	457.6958	45972.5	1.147983	13.53962	0.67129	1.715023	1.032811	0.168986	0.781963	0.757121	1006.52	7.286943	1014.165	6.624502	1031.57	13.65519	1031.6	13.7	97.6	427
_19AR111_Spot_ 110	107.5871	15123.62	3.011203	13.40687	1.204265	1.781012	1.580457	0.175473	0.675555	0.427443	1042.194	6.500939	1038.549	10.2776	1031.743	28.89748	1031.7	28.9	101.0	95
_19AR111_Spot_ 266	137.8004	14816.94	4.03269	13.36902	1.33454	1.774736	1.75627	0.174837	0.719643	0.409756	1038.708	6.903853	1036.255	11.40648	1031.944	32.39828	1031.9	32.4	100.7	237
_19AR111_Spot_ 443	176.6191	16301.44	2.359013	13.44254	0.806413	1.79771	1.22004	0.176947	0.751355	0.615845	1050.273	7.281978	1044.627	7.960305	1033.699	19.44022	1033.7	19.4	101.6	386
_19AR111_Spot_ 157	180.5097	13262.14	2.802379	13.35991	0.973354	1.785931	1.615375	0.175729	0.911927	0.56453	1043.602	8.786494	1040.343	10.51509	1034.365	26.95758	1034.4	27.0	100.9	138
_19AR111_Spot_ 395	154.733	13700.97	1.528343	13.34679	0.838096	1.780647	1.274058	0.17518	0.88847	0.697354	1040.586	8.537684	1038.416	8.284392	1034.71	18.46685	1034.7	18.5	100.6	345
_19AR111_Spot_ 291	154.7218	15394.37	2.411454	13.35659	1.412352	1.761235	1.951561	0.173264	1.189195	0.609356	1030.071	11.321	1031.302	12.64004	1034.775	31.2809	1034.8	31.3	99.5	258
_19AR111_Spot_ 559	459.5721	42242.75	12.15256	13.4773	0.748213	1.784295	1.172106	0.175482	0.861335	0.734861	1042.247	8.289107	1039.747	7.627046	1035.355	16.0768	1035.4	16.1	100.7	485
_19AR111_Spot_ 225	155.7101	13950.92	1.421412	13.38101	1.285964	1.80296	1.659526	0.177299	0.856239	0.515954	1052.201	8.312513	1046.531	10.83926	1035.574	28.73476	1035.6	28.7	101.6	201
_19AR111_Spot_ 287	221.4552	15913.86	3.096887	13.35572	0.713402	1.801566	1.144249	0.177087	0.748667	0.654287	1051.043	7.26083	1046.026	7.471494	1036.421	17.4959	1036.4	17.5	101.4	254
_19AR111_Spot_ 383	86.18192	11200.31	2.522062	13.28787	1.065974	1.797982	2.118728	0.17656	0.735956	0.347357	1048.156	7.119492	1044.726	13.82523	1038.42	40.11761	1038.4	40.1	100.9	333
_19AR111_Spot_ 574	234.284	25833.06	3.611833	13.47121	0.832399	1.78224	1.171364	0.174956	0.709652	0.605833	1039.359	6.811936	1038.997	7.619064	1039.096	18.8331	1039.1	18.8	100.0	497
_19AR111_Spot_ 244	315.5027	19242.37	3.610028	13.37225	0.63196	1.793643	0.988476	0.175985	0.662739	0.670465	1045.002	6.393439	1043.15	6.44417	1040.132	14.82321	1040.1	14.8	100.5	219
_19AR111_Spot_ 456	258.3935	39258.42	5.003603	13.48785	0.770842	1.784214	1.145848	0.175031	0.769494	0.67155	1039.771	7.389061	1039.717	7.456054	1040.463	17.15587	1040.5	17.2	99.9	398
_19AR111_Spot_ 280	433.2981	37041.83	4.443475	13.41849	0.59078	1.816443	0.942894	0.178166	0.674287	0.715125	1056.947	6.573266	1051.403	6.17474	1040.766	13.32051	1040.8	13.3	101.6	249
_19AR111_Spot_ 590	276.5827	38433.31	2.820528	13.44041	0.686102	1.740368	1.057116	0.170693	0.795873	0.752873	1015.927	7.480563	1023.6	6.816965	1040.901	14.06237	1040.9	14.1	97.6	504
_19AR111_Spot_ 49	283.2107	34367.21	9.357029	13.39948	0.611951	1.772912	0.920143	0.173788	0.668168	0.726157	1032.951	6.377276	1035.587	5.973673	1042.017	12.78549	1042.0	12.8	99.1	42
_19AR111_Spot_ 177	135.6909	15485.07	2.398265	13.30457	1.139298	1.850496	1.737846	0.18127	0.88687	0.510327	1073.911	8.773161	1063.606	11.45585	1043.386	30.17217	1043.4	30.2	102.9	157
_19AR111_Spot_ 318	166.2414	20883.26	2.411249	13.41863	1.047486	1.790441	1.479846	0.17534	0.770434	0.520617	1041.468	7.409211	1041.986	9.641546	1043.929	25.50839	1043.9	25.5	99.8	281
_19AR111_Spot_ 39	79.37111	8532.216	2.226529	13.21006	1.19173	1.795907	2.117807	0.175787	0.719269	0.339629	1043.92	6.93216	1043.973	13.81352	1044.98	40.18926	1045.0	40.2	99.9	33
_19AR111_Spot_ 51	104.5135	12184.95	3.073323	13.24967	1.073508	1.816823	1.612117	0.177721	0.670593	0.41597	1054.51	6.523379	1051.54	10.55833	1046.276	29.58428	1046.3	29.6	100.8	44
_19AR111_Spot_ 183	275.7048	31374.71	2.431389	13.36791	0.671982	1.856769	1.012886	0.181546	0.74243	0.732985	1075.417	7.353783	1065.839	6.684664	1047.182	13.91139	1047.2	13.9	102.7	162
_19AR111_Spot_ 147	829.5412	56739.59	4.671075	13.39479	0.69702	1.789195	1.019188	0.174847	0.734055	0.720235	1038.762	7.042448	1041.532	6.638487	1048.246	14.27309	1048.2	14.3	99.1	129
_19AR111_Spot_ 285	300.5757	38874.68	2.765642	13.36933	0.752355	1.833307	1.111022	0.179114	0.744251	0.66988	1062.133	7.288054	1057.465	7.299643	1048.738	16.64793	1048.7	16.6	101.3	252
_19AR111_Spot_ 188	95.82742	18856.21	4.204086	13.30756	1.132317	1.882868	1.468619	0.183953	0.826022	0.562448	1088.535	8.273375	1075.073	9.739744	1048.772	24.49899	1048.8	24.5	103.8	167
_19AR111_Spot_ 232	270.9351	31206.77	5.788725	13.39149	0.699349	1.804812	1.072675	0.176293	0.751294	0.700393	1046.691	7.258519	1047.202	7.008633	1049.165	15.45181	1049.2	15.5	99.8	207
_19AR111_Spot_ 497	190.2595	20079.59	2.083198	13.34722	0.730016	1.786814	1.098607	0.174479	0.772325	0.703004	1036.742	7.396327	1040.665	7.152385	1049.811	15.76694	1049.8	15.8	98.8	428
_19AR111_Spot_ 467	371.5048	42230.93	2.178698	13.41778	0.752983	1.792861	1.100635	0.175022	0.787915	0.715873	1039.723	7.565625	1042.866	7.174268	1050.353	15.50724	1050.4	15.5	99.0	407
_19AR111_Spot_ 263	124.725	17799.21	3.531819	13.27743	1.163196	1.850035	1.645052	0.180598	0.840322	0.510818	1070.242	8.286584	1063.442	10.84316	1050.413	28.50877	1050.4	28.5	101.9	235
_19AR111_Spot_ 543	132.9556	13094.04	4.08945	13.21097	0.900535	1.85332	1.805846	0.180791	1.019543	0.564579	1071.296	10.06302	1064.612	11.91052	1051.837	30.035	1051.8	30.0	101.8	469
_19AR111_Spot_ 175	652.8458	105009.8	4.990263	13.37714	0.699783	1.853291	1.090045	0.180632	0.821361	0.753511	1070.429	8.100904	1064.602	7.189175	1053.575	14.45493	1053.6	14.5	101.6	156
_19AR111_Spot_ 358	402.7486	17980.19	2.218918	13.26992	0.678904	1.822466	1.598157	0.177567	1.020867	0.638778	1053.669	9.923481	1053.572	10.47841	1054.268	24.77911	1054.3	24.8	99.9	315
_19AR111_Spot_ 552	124.764	16433.23	1.11498	13.23879	0.842009	1.836722	1.300411	0.178916	0.829467	0.63785	1061.052	8.114925	1058.688	8.549633	1054.715	20.19135	1054.7	20.2	100.6	478
_19AR111_Spot_ 20	454.7262	31651.91	2.707156	13.38547	0.83121	1.794161	1.204976	0.174673	0.851766	0.706874	1037.807	8.164834	1043.338	7.85646	1055.838	17.1824	1055.8	17.2	98.3	16
_19AR111_Spot_ 189	346.9549	42346.34	3.704179	13.32449	0.650136	1.844048	1.093953	0.179302	0.810073	0.740501	1063.16	7.939673	1061.307	7.202298	1058.394	14.81831	1058.4	14.8	100.5	168
_19AR111_Spot_ 562	212.5563	17267.72	2.524384	13.22596	0.781232	1.759065	1.830971	0.170691	1.535619	0.838691	1015.92	14.4335	1030.504	11.85362	1062.486	20.07035	1062.5	20.1	95.6	487
_19AR111_Spot_ 430	218.0791	29158.26	3.27602	13.252	1.026454	1.856016	1.465836	0.179989	1.021047	0.696563	1066.916	10.03998	1065.571	9.672743	1063.712	21.17443	1063.7	21.2	100.3	375
_19AR111_Spot_ 568	164.1168	19416.31	1.974575	13.24632	0.689646	1.858776	1.01797	0.18023	0.659456	0.647815	1068.23	6.491791	1066.552	6.72076	1064.016	15.59655	1064.0	15.6	100.4	492
_19AR111_Spot_ 32	84.79112	18071.8	2.610609	13.25526	1.217686	1.872377	1.867793	0.181459	1.001524	0.536207	1074.939	9.916062	1071.371	12.36323	1065.012	31.70611	1065.0	31.7	100.9	26
_19AR111_Spot_ 288	177.0442	14602.22	2.348151	13.11947	0.866376	1.910516	1.283904	0.184748	0.689284	0.536866	1092.86	6.928994	1084.765	8.557627	1069.436	21.75054	1069.4	21.8	102.2	255
_19AR111_Spot_ 539	266.1216	34541.22	2.503731	13.19537	0.650817	1.886528	0.894786	0.182167	0.579979	0.648176	1078.8	5.761304	1076.361	5.938018	1072.319	13.67988	1072.3	13.7	100.6	465
_19AR111_Spot_ 458	386.1229	39831.26	2.186346	13.24488	0.686109	1.87739	1.120249	0.180962	0.85173	0.760305	1072.227	8.413401	1073.142	7.421779	1075.896	14.59194	1075.9	14.6	99.7	400
_19AR111_Spot_ 36	311.2112	26788.22	5.390557	13.18296	0.896635	1.895098	1.289276	0.181907	0.879329	0.682033	1077.385	8.724413	1079.371	8.56948	1084.276	18.89022	1084.3	18.9	99.4	30
_19AR111_Spot_ 221	80.21575	13229.01	2.359871	13.0346	1.456862	1.910371	1.674531	0.183061	0.668423	0.39917	1083.674	6.667421	1084.714	11.16117	1087.694	30.7721	1087.7	30.8	99.6	197
_19AR111_Spot_ 198	162.174	25487.52	5.253427	13.05027	0.842845	1.931655	1.103255	0.184692	0.670136	0.607417	1092.556	6.734783	1092.112	7.381251	1092.082	17.53464	1092.1	17.5	100.0	176
_19AR111_Spot_ 386	247.1826	22571.85	2.187945	12.97566	0.789865	1.979716	1.170803	0.188259	0.762284	0.651078	1111.938	7.785383	1108.624	7.898609	1102.994	17.78887	1103.0	17.8	100.8	336
_19AR111_Spot_ 98	249.103	33224.5	1.479918	13.03567	0.741968	1.984171	1.063043	0.188459	0.693631	0.652495	1113.021	7.090535	1110.141	7.177009	1105.392	16.11114	1105.4	16.1	100.7	84
_19AR111_Spot_ 320	187.0335	30298.37	1.341185	13.00822	0.818523	1.967759	1.277484	0.186718	0.962228	0.753221	1103.573	9.759684	1104.541	8.600806	1107.337	16.79182	1107.3	16.8	99.7	283
_19AR111_Spot_ 9	72.63987	8495.992	3.959321	12.71733	1.301848	2.040439	1.862302	0.192078	0.850363	0.456619	1132.62	8.832729	1129.108	12.69083	1123.237	33.03225	1123.2	33.0	100.8	7
_19AR111_Spot_ 359	124.5612	5510.695	3.251296	12.51374	1.059214	2.038688	2.525286	0.191507	0.823271	0.326011	1129.533	8.529998	1128.523	17.20468	1127.43	47.57059	1127.4	47.6	100.2	316
_19AR111_Spot_ 197	114.9614	18826	2.967364	12.71358	0.928595	2.051253	1.307011	0.191509	0.780387	0.597078	1129.547	8.08577	1132.713	8.921982	1139.643	20.83369	1139.6	20.8	99.1	175
_19AR111_Spot_ 259	220.9263	33048.08	6.214745	12.73464	0.717168	2.11257	1.044679	0.196542	0.746165	0.714253	1156.717	7.900975	1152.915	7.199663	1146.631	14.50745	1146.6	14.5	100.9	232
_19AR111_Spot_ 385	389.726	55524.58	1.352821	12.71831	0.708214	2.157381	1.131202	0.200095	0.849684	0.751133	1175.83	9.132635	1167.429	7.848353	1152.725	14.80468	1152.7	14.8	102.0	335
_19AR111_Spot_ 482	102.7909	13864.74	1.851974	12.57297	1.363976	2.017159	1.79404	0.186325	0.93322	0.520178	1101.438	9.448673	1121.303	12.17937	1160.845	30.39337	1160.8	30.4	94.9	420
_19AR111_Spot_ 462	229.9576	35187.23	2.653119	12.61035	0.727356	2.150796	1.148078	0.197562	0.805297	0.701431	1162.208	8.56405	1165.31	7.957726	1171.958	16.21605	1172.0	16.2	99.2	402
_19AR111_Spot_ 146	281.078	28845.24	3.16775	12.54164	0.837093	2.084071	1.363455	0.191226	1.057089	0.775302	1128.014	10.93915	1143.576	9.355593	1174.093	17.04121	1174.1	17.0	96.1	128
_19AR111_Spot_ 563	82.24353	10102.35	3.794109	12.28612	1.177599	2.364531	1.656481	0.214264	0.905209	0.546465	1251.496	10.29684	1231.953	11.82106	1198.767	27.33647	1198.8	27.3	104.4	488
_19AR111_Spot_ 182	155.3335	25953.29	3.490185	12.29962	0.709735	2.336994	1.114844	0.210297	0.73857	0.662487	1230.399	8.272772	1223.608	7.927844	1212.52	16.4331	1212.5	16.4	101.5	161
_19AR111_Spot_ 304	241.9965	27107.85	1.724904	12.33174	0.62961	2.330924	0.986854	0.209264	0.719665	0.729252	1224.896	8.028277	1221.759	7.01218	1217.062	13.29729	1217.1	13.3	100.6	269
_19AR111_Spot_ 587	74.73681	12150.33	2.113392	12.13891	1.874069	2.369402	2.058524	0.211468	0.708426	0.344143	1236.635	7.971609	1233.421	14.69948	1228.648	37.94788	1228.6	37.9	100.7	501
_19AR111_Spot_ 408	186.2031	34733.47	0.600282	12.1403	0.659146	2.345014	1.020967	0.20795	0.64119	0.628022	1217.887	7.115658	1226.045	7.267695	1241.26	15.58783	1241.3	15.6	98.1	357
_19AR111_Spot_ 333	59.23305	9178.458	0.548549	11.93866	1.467851	2.588081	2.404787	0.227964	0.66692	0.27733	1323.821	7.981288	1297.271	17.61431	1254.443	45.18487	1254.4	45.2	105.5	294
_19AR111_Spot_ 459	59.50151	11587.36	1.343527	11.91646	1.330269	2.482488	1.765068	0.217414	0.783653	0.443979	1268.198	9.021771	1266.941	12.7765	1265.642	30.86995	1265.6	30.9	100.2	401
_19AR111_Spot_ 475	99.50312	13038.9	1.30328	11.33481	0.866906	2.873735	1.254656	0.239144	0.881866	0.702874	1382.246	10.97132	1375.051	9.451138	1364.715	17.18755	1364.7	17.2	101.3	415
_19AR111_Spot_ 555	235.0675	40442.39	2.300118	11.31051	0.738533	2.944086	1.129433	0.243216	0.834021	0.738443	1403.394	10.5182	1393.326	8.560608	1378.766	14.63944	1378.8	14.6	101.8	481
_19AR111_Spot_ 576	81.32891	20109.18	3.001705	10.8935	1.010802	3.267189	1.31018	0.259386	0.763918	0.583063	1486.7	10.14268	1473.275	10.1861	1454.797	20.24876	1454.8	20.2	102.2	499
_19AR111_Spot_ 87	205.4936	28570.48	1.90106	10.80907	0.710624	3.142176	1.100989	0.24769	0.74523	0.676873	1426.554	9.536985	1443.084	8.48057	1468.335	15.38888	1468.3	15.4	97.2	75

_19AR111_Spot_ 226	251.1514	50543.03	4.938072	10.3667	0.7541	3.586912	1.143435	0.270308	0.8492	0.742674	1542.367	11.64873	1546.639	9.079316	1553.281	14.37618	1553.3	14.4	99.3	202
_19AR111_Spot_ 184	16.81532	1851.753	5.123282	9.306925	2.71397	3.768584	3.678848	0.273959	1.053099	0.286258	1560.864	14.59882	1586.079	29.52929	1620.556	65.6175	1620.6	65.6	96.3	163
_19AR111_Spot_ 473	462.0017	49343.77	1.809781	9.849421	0.55068	3.193927	1.136177	0.228816	0.960512	0.845389	1328.292	11.52979	1455.691	8.785994	1647.699	11.25553	1647.7	11.3	80.6	413
_19AR111_Spot_ 362	115.7475	18278.53	2.40289	9.45406	0.70491	3.778432	1.157995	0.261258	0.90171	0.778683	1496.273	12.04068	1588.173	9.297684	1713.2	13.36146	1713.2	13.4	87.3	318
_19AR111_Spot_ 81	139.5082	26694.33	1.628259	9.196828	0.626002	4.716391	0.944462	0.315948	0.660951	0.699818	1769.91	10.22975	1770.155	7.91245	1771.224	12.3172	1771.2	12.3	99.9	70
_19AR111_Spot_ 393	234.5029	27581.52	3.329117	9.171373	0.691151	3.811682	1.115515	0.255266	0.856065	0.767417	1465.577	11.22233	1595.214	8.972977	1771.767	13.057	1771.8	13.1	82.7	343
_19AR111_Spot_ 130	55.73854	11076.5	0.595282	9.110453	0.99974	4.793004	1.583972	0.320912	0.846063	0.53414	1794.18	13.25054	1783.674	13.3078	1772.183	24.44771	1772.2	24.4	101.2	114
_19AR111_Spot_ 40	187.2141	32621.44	1.403	9.196448	0.624819	4.402234	1.044297	0.294506	0.825238	0.790233	1664.008	12.10286	1712.761	8.641	1773.681	11.68007	1773.7	11.7	93.8	34
_19AR111_Spot_ 142	59.43638	12568.56	1.181069	8.934156	1.042693	4.956408	1.442637	0.324983	0.794813	0.550945	1814.019	12.56708	1811.918	12.18963	1810.278	21.87713	1810.3	21.9	100.2	124
_19AR111_Spot_ 570	41.77173	8027.873	1.128692	8.87421	1.205286	4.721451	1.562823	0.308283	0.823491	0.526925	1732.251	12.50907	1771.054	13.09583	1817.886	24.1141	1817.9	24.1	95.3	494
_19AR111_Spot_ 417	271.3728	18723.01	2.001111	8.85262	0.776068	4.856293	1.149819	0.314567	0.79906	0.694944	1763.143	12.32619	1794.707	9.681771	1832.359	14.98445	1832.4	15.0	96.2	365
_19AR111_Spot_ 538	41.0314	9033.492	1.548246	8.676752	1.013543	5.461983	1.37104	0.349064	0.776241	0.56617	1930.129	12.94757	1894.64	11.76749	1856.747	20.41992	1856.7	20.4	104.0	464
_19AR111_Spot_ 144	516.5131	24868.1	1.072449	8.678808	0.712185	5.052824	1.216926	0.320529	0.981836	0.806817	1792.313	15.36306	1828.223	10.31538	1870.14	12.97065	1870.1	13.0	95.8	126
_19AR111_Spot_ 216	328.4623	82130.8	0.833723	8.72333	0.589955	5.133322	0.920641	0.325538	0.700902	0.761319	1816.719	11.09649	1841.637	7.824051	1870.68	10.76822	1870.7	10.8	97.1	193
_19AR111_Spot_ 41	159.8046	31640.07	1.081602	8.694276	0.635813	5.281968	0.900788	0.334019	0.618651	0.686789	1857.833	9.985581	1865.952	7.690607	1875.779	11.80403	1875.8	11.8	99.0	35
_19AR111_Spot_ 553	308.4485	81131.51	10.67829	8.664459	0.731587	5.438616	1.123597	0.343332	0.851469	0.757806	1902.679	14.02873	1890.962	9.637168	1878.89	13.21025	1878.9	13.2	101.3	479
_19AR111_Spot_ 111	102.1415	15706.7	2.265474	8.481331	0.717806	4.905929	1.223768	0.304128	0.738461	0.603432	1711.746	11.10152	1803.276	10.32232	1911.566	17.51626	1911.6	17.5	89.5	96
_19AR111_Spot_ 441	100.8396	20552.58	1.592819	8.482812	0.817774	5.316898	1.169284	0.328192	0.754226	0.645032	1829.613	12.014	1871.583	9.99352	1919.272	16.02344	1919.3	16.0	95.3	384
_19AR111_Spot_ 471	132.8304	31028.15	1.907794	8.252625	0.744021	5.47143	1.403017	0.328357	1.167674	0.83226	1830.414	18.60684	1896.123	12.04519	1969.596	13.86576	1969.6	13.9	92.9	411
_19AR111_Spot_ 74	196.1169	21853.51	1.249986	8.172513	0.666866	6.000705	1.108523	0.357707	0.759017	0.68471	1971.297	12.89116	1975.946	9.648243	1981.576	14.38425	1981.6	14.4	99.5	63
_19AR111_Spot_ 363	279.947	9053.431	1.230368	8.090451	0.641647	4.908049	1.567616	0.292124	1.233575	0.786911	1652.136	17.97828	1803.641	13.22388	1984.309	17.21505	1984.3	17.2	83.3	319
_19AR111_Spot_ 392	165.746	37322.51	2.202787	8.150393	0.63909	5.920028	1.056726	0.351753	0.81473	0.770994	1942.964	13.667	1964.177	9.179524	1987.36	11.97354	1987.4	12.0	97.8	342
_19AR111_Spot_ 112	243.4056	56715.07	3.124793	7.917926	0.619799	6.41649	1.099207	0.370004	0.888812	0.808594	2029.422	15.47441	2034.529	9.656539	2040.465	11.43444	2040.5	11.4	99.5	97
_19AR111_Spot_ 351	43.73816	13603.08	2.741071	7.865019	0.792632	6.899773	1.367765	0.39721	1.019587	0.74544	2156.18	18.68539	2098.628	12.1306	2043.413	16.11492	2043.4	16.1	105.5	310
_19AR111_Spot_ 227	131.9677	25772.41	4.366535	7.523389	0.743597	7.197142	1.152263	0.394006	0.857587	0.744263	2141.38	15.62555	2136.148	10.27292	2131.866	13.4691	2131.9	13.5	100.4	203
_19AR111_Spot_ 394	332.2537	67402.56	4.47053	7.110113	0.606077	7.509665	0.953475	0.38873	0.735418	0.771303	2116.934	13.27037	2174.141	8.54393	2229.315	10.50564	2229.3	10.5	95.0	344
_19AR111_Spot_ 180	76.48337	17590.07	2.31741	6.307449	0.770591	9.983175	1.120359	0.459682	0.772998	0.689955	2438.155	15.69266	2433.228	10.34053	2429.833	13.74728	2429.8	13.7	100.3	160
_19AR111_Spot_ 415	352.9023	80289.97	1.581494	6.106128	0.518992	8.340108	1.134649	0.370603	1.007721	0.888134	2032.236	17.56534	2268.688	10.28788	2489.968	8.78784	2490.0	8.8	81.6	363
_19AR111_Spot_ 33	143.769	27902.06	1.998075	5.69299	0.745625	11.59079	1.173608	0.478073	0.903339	0.769711	2518.866	18.83512	2571.93	10.97059	2614.731	12.47283	2614.7	12.5	96.3	27
_19AR111_Spot_ 501	133.9463	33288.61	1.17229	5.647616	0.612799	11.90671	1.069787	0.488225	0.857246	0.801324	2562.991	18.12909	2597.093	10.02115	2624.512	10.644	2624.5	10.6	97.7	431
_19AR111_Spot_ 100	99.67991	31799.06	2.11186	5.623937	0.655469	11.13525	1.022578	0.454795	0.771074	0.75405	2416.535	15.53922	2534.512	9.527746	2631.069	11.1604	2631.1	11.2	91.8	86
_19AR111_Spot_ 271	224.438	61075.53	2.149035	5.613569	0.480556	12.12124	0.839589	0.494867	0.687877	0.819303	2591.699	14.67966	2613.832	7.875488	2631.734	8.000697	2631.7	8.0	98.5	241
_19AR111_Spot_ 113	119.5528	36945.8	5.070708	5.592836	0.61062	11.96203	0.93904	0.487231	0.710747	0.756887	2558.686	15.01035	2601.436	8.799474	2635.604	10.19296	2635.6	10.2	97.1	98
_19AR111_Spot_ 435	381.5298	24404.2	3.185728	5.594234	0.5907	11.13862	1.039105	0.453126	0.819297	0.788464	2409.135	16.46934	2534.795	9.681992	2637.68	10.61339	2637.7	10.6	91.3	379
_19AR111_Spot_ 319	174.2298	42997.98	3.412723	5.574764	0.669957	11.78217	1.027632	0.476966	0.769936	0.749233	2514.035	16.02841	2587.248	9.618364	2645.801	11.29369	2645.8	11.3	95.0	282
_19AR111_Spot_ 240	253.8854	141870.2	1.932338	5.562359	0.574529	11.88374	0.896515	0.480163	0.686975	0.766273	2527.975	14.3661	2595.285	8.396695	2648.96	9.554965	2649.0	9.6	95.4	215
_19AR111_Spot_ 276	80.80546	29958.39	1.9078	5.550526	0.507552	12.53826	0.845237	0.506587	0.663671	0.785189	2642.042	14.38572	2645.601	7.948623	2649.03	8.682014	2649.0	8.7	99.7	245
_19AR111_Spot_ 350	24.08358	10975.24	1.372345	5.256824	1.036588	13.93011	1.368699	0.534964	0.843052	0.615951	2762.333	18.94088	2744.966	12.9674	2732.923	17.74799	2732.9	17.7	101.1	309

**19AR112**				
-26.94369	-65.76479	4200	42	1.032

19AR112 Spot 71	2282.289	141.7701	3.180168	4.089992	12.2812	0.003844	48.51796	0.000181	6.569801	0.13541	1.166365	0.076621	3.895159	1.886233	2392.139	877.6775	1.2	0.1	0.0	51
19AR112 Spot 81	1358.771	254.9454	0.971554	7.822296	25.72585	0.002884	73.4477	0.000293	8.570569	0.116689	1.886547	0.161664	2.923789	2.14437	971.3203	NA	1.9	0.2	0.2	57
19AR112 Spot 200	1197.496	161.4425	1.551993	6.086316	20.56813	0.003179	93.70835	0.000303	6.880666	0.073426	1.953739	0.13441	3.223055	3.015492	1097.791	NA	2.0	0.1	0.2	136
19AR112 Spot 482	2250.648	459.565	3.574846	7.820389	16.4152	0.004349	31.82023	0.000323	6.715646	0.21105	2.080835	0.139719	4.406748	1.3992	1581.671	596.7917	2.1	0.1	0.1	318
19AR112 Spot 113	1324.445	61.84576	10.55294	4.837208	28.93356	-0.00306	92.39799	0.000331	4.554624	0.049294	2.135179	0.097233	NA	-2.87534	NA	NA	2.1	0.1		77
19AR112 Spot 205	1743.957	340.7724	4.443681	5.362187	42.49961	0.006863	43.28169	0.000335	5.179667	0.119673	2.157758	0.111746	6.944508	2.995454	2331.479	777.1528	2.2	0.1	0.1	141
19AR112 Spot 41	1557.162	266.5604	0.959754	5.571015	29.96109	0.0061	43.43192	0.000341	4.750061	0.109368	2.199677	0.104468	6.175226	2.673886	2093.796	800.8454	2.2	0.1	0.1	30
19AR112 Spot 307	3197.115	387.7218	1.961212	8.664765	18.31521	0.003809	23.06284	0.000351	2.601304	0.112792	2.261332	0.058814	3.860356	0.888618	1166.47	459.8897	2.3	0.1	0.2	209
19AR112 Spot 581	2276.355	267.6049	2.458054	11.34426	11.7546	0.001555	61.76784	0.000355	4.552632	0.073706	2.287242	0.104111	1.577989	0.973933	NA	NA	2.3	0.1		379
19AR112 Spot 442	2098.478	484.961	4.634319	12.73114	11.44537	0.002392	49.57618	0.000361	3.172018	0.063983	2.329409	0.073876	2.426118	1.201342	100.6023	NA	2.3	0.1	2.3	289
19AR112 Spot 54	950.7485	231.235	1.625056	5.915897	34.8672	0.005542	42.264	0.000365	5.891903	0.139407	2.349524	0.138406	5.612112	2.365364	1804.628	799.3353	2.3	0.1	0.1	38
19AR112 Spot 345	2185.653	711.7644	4.882389	11.59813	12.46324	0.003315	14.76684	0.000365	2.524602	0.170964	2.355365	0.059453	3.360535	0.495425	800.6744	306.3188	2.4	0.1	0.3	231
19AR112 Spot 231	1371.818	267.9348	5.338634	5.674861	18.61038	0.006625	43.69283	0.00038	6.561491	0.150173	2.447666	0.160573	6.704281	2.919649	2050.841	805.1372	2.4	0.2	0.1	158
19AR112 Spot 137	1169.33	163.6685	1.861666	6.890088	20.05603	0.002936	139.2428	0.000382	6.777179	0.048672	2.463956	0.166955	2.976465	4.138469	441.1375	NA	2.5	0.2	0.6	94
19AR112 Spot 36	770.7085	205.5091	2.5294	7.477317	22.95209	0.003442	102.3262	0.000395	9.629304	0.094104	2.54771	0.245278	3.48917	3.564224	714.4252	NA	2.5	0.2	0.4	27
19AR112 Spot 18	1263.347	278.9076	3.25735	7.292755	13.45952	0.005123	45.94282	0.000428	4.404375	0.095866	2.75947	0.121511	5.188419	2.37763	1356.408	933.647	2.8	0.1	0.2	13
19AR112 Spot 57	1828.443	389.0755	2.157144	10.5568	14.42857	0.00566	24.34772	0.000717	2.389361	0.098135	4.619719	0.110342	5.730576	1.391335	502.4674	540.5783	4.6	0.1	0.9	40
19AR112 Spot 566	716.4637	374.8549	1.232783	8.223745	27.62594	0.008676	30.03017	0.00075	4.355149	0.145026	4.832178	0.21037	8.771374	2.62272	1291.351	591.2997	4.8	0.2	0.4	371
19AR112 Spot 368	908.9313	697.7322	4.614655	9.677605	22.80917	0.008885	28.28306	0.000777	6.443196	0.227811	5.005767	0.322406	8.981839	2.529141	1268.971	547.9611	5.0	0.3	0.4	245
19AR112 Spot 366	626.0202	382.606	2.781949	10.0931	18.93007	0.007876	45.32025	0.000937	3.624354	0.079972	6.036318	0.218675	7.965654	3.595946	639.2387	NA	6.0	0.2	0.9	243
19AR112 Spot 468	610.9178	338.6281	4.733571	5.800762	48.10209	0.017775	50.6944	0.000969	4.286015	0.084546	6.243103	0.267451	17.88949	8.989781	2139.317	954.0481	6.2	0.3	0.3	307
19AR112 Spot 23	1041.636	823.5707	4.67575	13.23607	11.15273	0.008938	28.0859	0.001122	2.647319	0.094258	7.228926	0.191265	9.035521	2.526455	522.2584	NA	7.2	0.2	1.4	18
19AR112 Spot 142	1180.175	605.5258	3.581883	15.61646	9.953121	0.006174	23.90983	0.001138	3.174416	0.132766	7.330515	0.232569	6.249827	1.489734	NA	NA	7.3	0.2		97
19AR112 Spot 595	499.4497	508.7367	4.174427	7.212971	34.84008	0.018825	38.79428	0.001215	5.47134	0.141035	7.827578	0.428013	18.93715	7.278571	1838.936	724.506	7.8	0.4	0.4	388
19AR112 Spot 72	3704.166	1890.934	2.564383	17.10845	5.894245	0.01084	7.800721	0.001555	2.355679	0.301982	10.01741	0.235795	10.94793	0.84943	221.6042	172.2114	10.0	0.2	4.5	52
19AR112 Spot 222	264.6775	318.9278	2.274373	7.6329	21.75172	0.022961	50.51382	0.001913	6.53818	0.129433	12.31934	0.804691	23.05042	11.51297	1362.57	1035.196	12.3	0.8	0.9	153
19AR112 Spot 63	738.4214	683.8003	1.955307	10.27121	17.42733	0.02199	20.48552	0.002086	2.056867	0.100406	13.43103	0.275971	22.08667	4.475743	1108.235	411.3973	13.4	0.3	1.2	44
19AR112 Spot 303	449.1053	1310.833	4.660781	13.74505	9.201007	0.028894	10.51037	0.003393	2.805741	0.26695	21.83661	0.611642	28.92276	2.99701	666.7112	217.3949	21.8	0.6	3.3	205
19AR112 Spot 65	154.9353	363.6506	4.358562	6.784827	28.92494	0.052077	34.4189	0.003438	7.467169	0.21695	22.12354	1.64917	51.54721	17.30083	1797.957	630.4809	22.1	1.6	1.2	46
19AR112 Spot 129	1832.668	3178.195	4.277686	16.31569	4.429988	0.028291	7.824973	0.003625	1.258766	0.160865	23.32631	0.293093	28.32774	2.186006	477.0407	170.9718	23.3	0.3	4.9	89
19AR112 Spot 310	199.85	609.3738	4.568919	9.016052	16.29173	0.059055	22.60736	0.004875	3.968719	0.17555	31.35206	1.241254	58.25958	12.8009	1380.064	433.0841	31.4	1.2	2.3	212
19AR112 Spot 502	332.254	1654.682	4.092493	11.24678	10.22615	0.068029	12.55938	0.006157	6.701056	0.53355	39.57055	2.643523	66.82773	8.123087	1201.119	209.9465	39.6	2.6	3.3	329
19AR112 Spot 552	159.4447	822.813	2.973655	11.1447	16.50055	0.083476	22.45148	0.008351	3.42253	0.152441	53.61119	1.82725	81.40809	17.56558	1000.782	455.9547	53.6	1.8	5.4	359
19AR112 Spot 209	265.2839	1365.081	1.490419	10.93503	9.440217	0.093967	10.76372	0.008399	3.949317	0.36691	53.91951	2.120571	91.1924	9.388092	1225.653	197.1441	53.9	2.1	4.4	143
19AR112 Spot 362	376.3445	545.1221	2.590531	14.81518	4.10481	0.061736	47.72389	0.011173	1.555265	0.032589	71.62698	1.107823	60.82641	28.18359	NA	NA	71.6	1.1		240
19AR112 Spot 577	367.0934	2868.784	1.894381	17.60529	4.151664	0.094036	7.531436	0.013213	1.052762	0.139782	84.61757	0.885001	91.25641	6.57321	269.4859	171.2133	84.6	0.9	31.4	378
19AR112 Spot 173	273.3026	2720.35	2.043066	15.68422	5.975899	0.149437	8.835666	0.018622	1.291293	0.146146	118.9436	1.521828	141.4145	11.66435	538.2311	191.5745	118.9	1.5	22.1	115
19AR112 Spot 220	120.0073	1552.593	1.939156	12.03188	8.783904	0.227198	14.39919	0.022275	2.154864	0.149652	142.0175	3.026819	207.8826	27.07447	1041.651	288.7123	142.0	3.0	13.6	151
19AR112 Spot 503	89.43672	2437.622	7.570771	14.37067	5.089048	0.26547	6.047848	0.030273	2.648142	0.437865	192.2596	5.016136	239.0654	12.88302	729.2716	115.3285	192.3	5.0	26.4	330
19AR112 Spot 325	575.8702	20081.44	5.292646	13.264	1.160802	0.399125	3.010865	0.038782	2.699151	0.89647	245.2795	6.496097	341.0133	8.721361	1059.74	26.86325	245.3	6.5	23.1	218
19AR112 Spot 568	76.94843	230.5573	1.778361	13.18121	8.075872	0.050414	60.33024	0.041019	1.142509	0.018938	259.1455	2.90204	49.94052	29.40852	NA	NA	259.1	2.9		373
19AR112 Spot 279	140.1238	3793.453	2.490731	18.57918	2.917548	0.293536	5.590657	0.042744	1.015847	0.181704	269.8221	2.68441	261.3392	12.88248	186.9353	128.034	269.8	2.7	144.3	189
19AR112 Spot 163	121.4111	1900.987	1.379903	16.84064	4.584676	0.313029	10.24995	0.044116	0.905547	0.088346	278.2962	2.466479	276.5263	24.81698	262.5624	234.9477	278.3	2.5	106.0	106
19AR112 Spot 83	360.2211	9242.535	3.961202	17.86993	1.994684	0.335825	2.246075	0.044951	0.797453	0.355043	283.447	2.211382	294.0033	5.733535	379.6847	47.20615	283.4	2.2	74.7	58
19AR112 Spot 22	1162.481	31042.17	6.060261	16.46203	0.779795	0.375456	1.898342	0.045214	1.669533	0.879469	285.0701	4.655641	323.6891	5.261632	612.6344	19.5209	285.1	4.7	46.5	17
19AR112 Spot 96	249.5374	8338.771	2.519836	17.39211	2.019748	0.386181	3.089426	0.050425	1.378892	0.446326	317.1273	4.267027	331.5761	8.739561	435.1921	61.59028	317.1	4.3	72.9	67
19AR112 Spot 278	350.3444	7470.939	2.281923	17.73477	1.81028	0.445011	2.165355	0.059478	0.96989	0.447913	372.4506	3.50999	373.7795	6.771188	382.9809	43.48972	372.5	3.5	97.3	188
19AR112 Spot 215	266.6368	8975.058	2.194137	17.54279	1.835215	0.49665	2.685082	0.065	0.841925	0.313557	405.9621	3.312501	409.4324	9.047515	429.9959	56.84955	406.0	3.3	94.4	147
19AR112 Spot 17	343.9493	12481.64	3.671325	17.52304	1.768045	0.525325	2.328069	0.068136	1.196043	0.513749	424.9149	4.918291	428.7026	8.141436	450.0589	44.37746	424.9	4.9	94.4	12
19AR112 Spot 328	138.7235	6680.784	2.151377	16.19402	3.277355	0.581509	3.980467	0.070858	1.142288	0.286973	441.3251	4.872513	465.4309	14.86209	587.1307	82.75269	441.3	4.9	75.2	219
19AR112 Spot 252	319.3834	6535.869	4.932375	14.91803	1.340692	0.638815	1.689922	0.071372	0.905147	0.535614	444.4152	3.887082	501.5721	6.688797	772.4874	30.03512	444.4	3.9	57.5	168
19AR112 Spot 466	388.7182	14306.84	2.020607	17.84663	1.297884	0.56187	2.138202	0.07437	0.76552	0.358021	462.4319	3.416021	452.7431	7.810489	404.7913	44.69911	462.4	3.4	114.2	305
19AR112 Spot 439	355.6362	15914.3	4.686722	17.80333	1.455433	0.572609	1.83902	0.075302	0.610678	0.332067	468.0173	2.756796	459.7002	6.799236	419.2948	38.73981	468.0	2.8	111.6	287
19AR112 Spot 301	318.4238	17538.6	58.43418	17.79995	1.881878	0.577382	2.065112	0.075381	0.80518	0.389896	468.4908	3.638384	462.7779	7.675531	435.4711	42.34934	468.5	3.6	107.6	204
19AR112 Spot 425	407.2664	31827.45	2.414444	17.65804	1.240059	0.581799	1.575452	0.075403	0.701519	0.445281	468.6268	3.170858	465.6169	5.88385	451.7465	31.346	468.6	3.2	103.7	280
19AR112 Spot 576	315.878	15938.84	8.339133	17.44904	1.419058	0.587183	2.15701	0.075607	0.841404	0.390079	469.8477	3.812685	469.067	8.102846	466.2149	44.01033	469.8	3.8	100.8	377
19AR112 Spot 411	370.9146	19789.33	3.224851	17.74815	1.402165	0.578471	2.068871	0.075655	0.771451	0.372885	470.1351	3.497767	463.4782	7.698684	431.5728	42.79126	470.1	3.5	108.9	272
19AR112 Spot 419	406.8053	11116.04	6.330466	17.6791	1.014874	0.574267	1.329203	0.07568	0.706973	0.531877	470.2834	3.206397	460.7701	4.92334	414.5652	25.15635	470.3	3.2	113.4	276
19AR112 Spot 405	260.4989	9261.761	2.203568	17.39217	1.274749	0.58231	2.191745	0.075704	0.814866	0.371789	470.4293	3.696841	465.9451	8.190156	444.8549	45.24771	470.4	3.7	105.7	268
19AR112 Spot 490	242.8873	10171.01	17.12694	17.33736	2.132344	0.586412	2.443592	0.075778	0.788239	0.322574	470.8702	3.579268	468.5739	9.171859	458.3187	51.30677	470.9	3.6	102.7	323
19AR112 Spot 500	283.4952	12985.72	12.50084	17.43543	2.25675	0.587089	2.507823	0.075812	0.860866	0.343272	471.0759	3.910706	469.0067	9.419802	459.8368	52.2555	471.1	3.9	102.4	327
19AR112 Spot 560	474.7997	30922.92	12.74522	17.31762	1.096013	0.5981	1.447322	0.07582	0.664525	0.459141	471.1217	3.019055	476.027	5.50008	500.7135	28.33178	471.1	3.0	94.1	367
19AR112 Spot 45	163.8206	7883.858	1.373679	17.42834	2.533721	0.580043	3.537771	0.075861	0.678463	0.191777	471.3706	3.08395	464.4893	13.1879	431.5584	77.40031	471.4	3.1	109.2	32
19AR112 Spot 201	36.79124	2078.546	3.812115	14.63082	7.832011	0.644527	9.602032	0.076151	1.339034	0.139453	473.1047	6.108156	505.1052	38.22948	653.7212	204.4522	473.1	6.1	72.4	137
19AR112 Spot 224	65.03023	3317.629	3.515022	16.06191	4.653435	0.608624	5.838211	0.076215	1.094263	0.187431	473.4926	4.995549	482.6918	22.43235	527.5949	125.8041	473.5	5.0	89.7	155
19AR112 Spot 217	153.3073	6745.118	2.297352	17.2035	2.113718	0.588723	3.820249	0.076238	0.842015	0.220408	473.6306	3.845061	470.052	14.37519	453.5616	82.74933	473.6	3.8	104.4	149
19AR112 Spot 535	293.6052	17627.66	15.00592	17.68136	1.916289	0.585562	2.114295	0.076291	0.81151	0.38382	473.9466	3.70814	468.0299	7.92856	440.0594	43.46454	473.9	3.7	107.7	351
19AR112 Spot 525	124.1788	7411.566	1.377118	16.60847	2.866339	0.612943	4.127615	0.076334	0.830344	0.201168	474.2058	3.796201	485.4142	15.92816	539.6164	88.46871	474.2	3.8	87.9	347
19AR112 Spot 21	181.8015	8513.333	1.489763	17.55818	1.918594	0.581046	2.24825	0.076352	0.688099	0.30606	474.3098	3.146544	465.1336	8.389778	421.0329	47.79921	474.3	3.1	112.7	16
19AR112 Spot 517	260.1572	14974.76	20.43581	17.64542	1.461665	0.586542	2.024548	0.076435	0.809223	0.399706	474.8109	3.704191	468.6572	7.600005	439.5745	41.29826	474.8	3.7	108.0	342
19AR112 Spot 412	243.862	11723.85	14.02912	17.91715	2.067703	0.572898	2.263754	0.076438	0.793157	0.350372	474.8253	3.630752	459.8869	8.372316	386.8877	47.59895	474.8	3.6	122.7	273
19AR112 Spot 585	178.3304	11850.22	4.975717	17.70043	2.192227	0.582785	2.602913	0.076513	0.952497	0.365935	475.2743	4.364119	466.2495	9.731712	422.9997	54.06503	475.3	4.4	112.4	382
19AR112 Spot 441	101.1925	7154.299	2.401955	17.15695	2.929426	0.592984	4.368753	0.076598	0.753624	0.172503	475.7844	3.456501	472.7712	16.51418	459.1426	95.49177	475.8	3.5	103.6	288
19AR112 Spot 554	148.0042	8365.254	1.5839	17.66293	2.699494	0.580024	3.771123	0.076652	0.740249	0.196294	476.1078	3.397382	464.4768	14.05758	408.3255	82.77747	476.1	3.4	116.6	361
19AR112 Spot 50	229.5595	9915.921	2.148055	17.27264	1.612599	0.596276	2.98481	0.076669	0.619013	0.207388	476.2087	2.841546	474.8675	11.3215	469.3644	64.66239	476.2	2.8	101.5	37
19AR112 Spot 551	104.4694	6411.111	18.30394	17.04912	2.880996	0.596739	3.209609	0.076708	0.868924	0.270726	476.4417	3.99063	475.1621	12.18017	469.9317	68.40709	476.4	4.0	101.4	358
19AR112 Spot 167	193.9158	10531.91	1.703936	17.33328	1.879668	0.593238	2.700168	0.076718	0.736264	0.272674	476.5034	3.381798	472.9336	10.20901	456.6184	57.64513	476.5	3.4	104.4	110
19AR112 Spot 305	287.5865	15321.18	25.38364	17.49827	1.561937	0.595251	1.941056	0.076726	0.789495	0.406735	476.5534	3.626665	474.2156	7.354391	463.907	39.29928	476.6	3.6	102.7	207
19AR112 Spot 521	272.208	12746.81	6.066725	17.05001	1.464021	0.607973	1.838195	0.076732	0.771125	0.419501	476.5896	3.542537	482.2812	7.057229	510.3781	36.68148	476.6	3.5	93.4	345
19AR112 Spot 7	89.27788	2997.763	1.895813	17.09023	3.856409	0.572763	6.926979	0.076747	0.846307	0.122175	476.6765	3.888605	459.8001	25.61992	377.2883	154.8179	476.7	3.9	126.3	5
19AR112 Spot 10	226.1535	12863.44	2.906095	17.56734	1.866321	0.594826	2.518631	0.076765	0.81142	0.322167	476.7865	3.729136	473.945	9.538586	461.1568	52.8655	476.8	3.7	103.4	6
19AR112 Spot 548	141.0626	7811.66	1.348709	17.44741	1.89581	0.586528	3.561965	0.076803	0.877145	0.246253	477.0129	4.03304	468.6479	13.37167	428.8292	76.99443	477.0	4.0	111.2	356
19AR112 Spot 549	70.86237	2576.599	3.171545	17.33828	2.932282	0.550238	8.55177	0.076838	1.055247	0.123395	477.2233	4.854	445.1523	30.82985	283.6253	194.4254	477.2	4.9	168.3	357
19AR112 Spot 37	683.3563	35164.75	23.43725	17.3762	0.78013	0.605098	1.10372	0.076935	0.663069	0.600758	477.8046	3.05361	480.4639	4.22489	494.1323	19.45705	477.8	3.1	96.7	28
19AR112 Spot 443	179.3306	10618.32	3.308055	16.75809	2.695912	0.617974	3.036069	0.077013	0.715004	0.235503	478.2716	3.295886	488.5764	11.77496	538.1661	64.54666	478.3	3.3	88.9	290
19AR112 Spot 6	218.7785	8040.079	6.551043	17.94905	1.724789	0.577507	2.307773	0.077016	0.699582	0.303142	478.2893	3.224911	462.8583	8.57866	387.9503	49.38465	478.3	3.2	123.3	4
19AR112 Spot 187	366.0885	13076.46	28.46704	17.69628	1.190868	0.587284	1.572854	0.077016	0.722651	0.459452	478.2896	3.331257	469.132	5.909038	425.5202	31.16425	478.3	3.3	112.4	127
19AR112 Spot 266	183.4824	7550.037	4.561306	16.80667	2.778726	0.610322	3.497011	0.077046	0.756828	0.216422	478.4648	3.490037	483.7634	13.45857	509.9027	75.09538	478.5	3.5	93.8	178
19AR112 Spot 13	94.52847	4025.392	2.491479	16.57494	3.774473	0.602737	4.043636	0.077049	0.64339	0.159112	478.483	2.967036	478.9691	15.44191	482.2796	88.21058	478.5	3.0	99.2	9
19AR112 Spot 512	380.8821	17446.41	13.52388	17.6355	1.183079	0.593564	1.887426	0.07705	0.817056	0.432895	478.4925	3.767983	473.1413	7.138472	448.2196	37.82066	478.5	3.8	106.8	338
19AR112 Spot 355	98.38084	6647.257	3.274735	17.39177	2.494079	0.587341	3.970661	0.077084	0.873613	0.220017	478.6928	4.030427	469.1679	14.91914	423.7837	86.45585	478.7	4.0	113.0	236
19AR112 Spot 505	134.1296	9794.488	2.133679	17.40027	2.483525	0.594573	3.35662	0.0771	0.807864	0.240678	478.7904	3.727827	473.7838	12.70912	450.5562	72.37454	478.8	3.7	106.3	332
19AR112 Spot 34	154.593	7751.709	1.811397	17.20001	2.870168	0.597134	3.510358	0.077103	0.84185	0.239819	478.8065	3.884777	475.4135	13.32714	460.0352	75.59032	478.8	3.9	104.1	26
19AR112 Spot 516	145.1569	6978.988	1.362393	17.29748	2.335189	0.592499	2.987294	0.077176	0.871851	0.291853	479.2433	4.026755	472.4624	11.28585	440.6087	63.59013	479.2	4.0	108.8	341
19AR112 Spot 272	288.0487	11818.4	5.227064	17.38892	1.539723	0.596624	2.143624	0.077184	0.702586	0.327756	479.2908	3.245294	475.089	8.133656	455.8379	44.96367	479.3	3.2	105.1	183
19AR112 Spot 472	392.4377	7978.785	27.74999	17.37943	1.039836	0.591445	1.914911	0.077191	0.669631	0.349693	479.3337	3.093336	471.79	7.226177	436.2105	39.9477	479.3	3.1	109.9	309
19AR112 Spot 191	172.5509	2593.51	2.059716	17.19174	2.333452	0.559191	5.262881	0.077218	0.935726	0.177797	479.4937	4.323944	450.9997	19.16752	309.1861	117.9674	479.5	4.3	155.1	130
19AR112 Spot 121	282.5859	15203.7	12.26535	17.80533	1.506234	0.588252	2.125702	0.077284	0.752999	0.354236	479.8879	3.482329	469.7509	7.99439	421.4694	44.38609	479.9	3.5	113.9	83
19AR112 Spot 339	316.0569	15423.96	6.444668	17.62225	1.437164	0.594484	1.713791	0.077304	0.836271	0.487965	480.0101	3.868376	473.7271	6.488044	444.358	33.26034	480.0	3.9	108.0	225
19AR112 Spot 444	148.2432	11691.29	1.514482	17.58209	2.399601	0.59233	3.18188	0.077314	0.78305	0.246097	480.0679	3.622608	472.3547	12.0189	436.0046	68.69166	480.1	3.6	110.1	291
19AR112 Spot 559	161.3239	7640.136	9.119707	17.45297	1.816789	0.59022	3.841253	0.077366	0.736147	0.191643	480.3828	3.407777	471.0081	14.47735	426.5343	84.12409	480.4	3.4	112.6	366
19AR112 Spot 398	63.05218	3474.731	1.627168	15.71859	3.535362	0.632884	4.809208	0.077376	1.11132	0.231082	480.4424	5.145144	497.8909	18.92878	579.858	101.6988	480.4	5.1	82.9	264
19AR112 Spot 267	88.84113	3730.572	1.146573	15.74851	4.197576	0.634724	4.554329	0.077387	0.840119	0.184466	480.5093	3.890067	499.0342	17.95725	585.8886	97.19771	480.5	3.9	82.0	179
19AR112 Spot 98	106.8418	4667.091	2.995806	16.76838	2.737326	0.600902	3.98428	0.077392	0.804279	0.201863	480.54	3.724345	477.8059	15.18626	465.6675	86.48513	480.5	3.7	103.2	69
19AR112 Spot 289	262.4161	14934.58	23.99153	17.71411	1.415122	0.593975	2.072619	0.077399	0.799811	0.385894	480.5809	3.703958	473.4028	7.84232	439.708	42.5708	480.6	3.7	109.3	195
19AR112 Spot 467	196.6457	8510.211	3.110534	17.75722	2.062452	0.580508	2.319346	0.077418	0.742908	0.320309	480.6912	3.441199	464.788	8.65003	387.9163	49.33933	480.7	3.4	123.9	306
19AR112 Spot 394	119.932	7427.291	6.739547	18.24297	2.469328	0.563419	3.74294	0.077445	0.736139	0.196674	480.8554	3.410966	453.7496	13.697	319.663	83.41669	480.9	3.4	150.4	261
19AR112 Spot 295	232.4445	9484.787	8.980732	17.84537	1.383991	0.584205	2.739238	0.077492	0.893311	0.326117	481.1336	4.141544	467.1604	10.25719	400.0159	58.02407	481.1	4.1	120.3	200
19AR112 Spot 255	337.6945	13293.51	6.198019	17.49187	1.109048	0.599499	1.49328	0.077566	0.770674	0.516094	481.5754	3.576135	476.9159	5.683034	455.548	28.40511	481.6	3.6	105.7	170
19AR112 Spot 332	334.9538	12251.98	8.402557	17.56097	1.202185	0.595857	2.258187	0.077567	0.779773	0.345309	481.5846	3.618424	474.6012	8.561469	441.9302	47.15732	481.6	3.6	109.0	222
19AR112 Spot 395	104.5331	5461.18	3.450636	17.45144	3.378335	0.583704	3.662134	0.077654	0.84695	0.231272	482.1039	3.934231	466.8393	13.70596	393.4045	79.95448	482.1	3.9	122.5	262
19AR112 Spot 586	188.0835	11198.57	10.70375	17.71912	2.01852	0.590109	2.346508	0.077659	0.658213	0.280507	482.1339	3.057698	470.9374	8.842365	417.688	50.31502	482.1	3.1	115.4	383
19AR112 Spot 385	102.4014	5207.754	1.620935	17.55019	3.188857	0.57918	3.969509	0.077679	0.900074	0.226747	482.254	4.182256	463.9344	14.78357	375.1947	87.05181	482.3	4.2	128.5	255

19AR112 Spot 5	236.1897	8864.188	18.64651	17.82704	1.309358	0.588573	1.87539	0.077697	0.766989	0.408976	482.3643	3.564655	469.9557	7.055398	410.7519	38.27551	482.4	3.6	117.4	3
19AR112 Spot 246	254.1502	14460.98	4.118129	17.08121	1.220344	0.616457	1.603243	0.077828	0.878198	0.547763	483.1461	4.087875	487.6245	6.208311	509.6587	29.48989	483.1	4.1	94.8	166
19AR112 Spot 492	250.2608	15709	3.678429	17.69885	1.989708	0.595307	2.261347	0.077861	0.822433	0.363691	483.3397	3.829775	474.2512	8.568488	431.4683	46.94444	483.3	3.8	112.0	324
19AR112 Spot 350	152.0493	7045.557	7.31151	17.4804	2.180496	0.590742	3.855896	0.077948	0.724855	0.187986	483.8612	3.378896	471.3412	14.54062	411.7859	84.7046	483.9	3.4	117.5	233
19AR112 Spot 66	222.3539	10186.43	7.818545	17.8882	1.808464	0.585929	2.614877	0.078093	0.882197	0.337376	484.7285	4.119442	468.2644	9.8097	389.2937	55.24354	484.7	4.1	124.5	47
19AR112 Spot 124	230.0537	8267.16	5.171346	17.72179	1.436577	0.590246	3.363744	0.078323	0.828326	0.246251	486.1028	3.878448	471.0245	12.6778	399.1645	73.06412	486.1	3.9	121.8	85
19AR112 Spot 450	147.5883	8593.905	13.10746	17.22449	2.958115	0.60861	4.080688	0.078537	0.818602	0.200604	487.3844	3.842647	482.6831	15.67784	461.3617	88.66453	487.4	3.8	105.6	293
19AR112 Spot 75	109.9576	3321.641	1.864076	16.20965	4.644854	0.619921	5.306242	0.078616	1.368403	0.257886	487.8565	6.42949	489.7979	20.62145	499.8687	112.9655	487.9	6.4	97.6	53
19AR112 Spot 89	350.1851	7638.199	2.871075	17.16974	1.47714	0.6093	3.101568	0.078668	0.882945	0.284677	488.1644	4.151069	483.1187	11.9241	460.2168	65.9586	488.2	4.2	106.1	62
19AR112 Spot 375	199.986	12485.66	8.627339	17.77859	2.12681	0.596694	2.360083	0.078707	0.904087	0.383074	488.4022	4.252457	475.1337	8.955679	412.5198	48.74451	488.4	4.3	118.4	249
19AR112 Spot 138	185.5709	7990.702	3.279998	17.26666	2.510145	0.609294	4.056556	0.078872	0.67862	0.16729	489.3839	3.198125	483.1149	15.596	454.4234	88.79771	489.4	3.2	107.7	95
19AR112 Spot 210	335.9643	19433.78	8.893469	17.56123	1.151109	0.613345	1.467015	0.079113	0.749187	0.510688	490.8233	3.540681	485.6676	5.662999	462.3992	27.9771	490.8	3.5	106.1	144
19AR112 Spot 176	212.9853	10974.85	7.34302	16.71618	1.837263	0.63563	2.457505	0.079122	0.906446	0.368848	490.8795	4.284366	499.5967	9.69743	540.6821	49.95867	490.9	4.3	90.8	117
19AR112 Spot 430	164.8929	7871.671	4.410027	17.78404	2.137204	0.595761	4.053928	0.079764	1.388682	0.342552	494.7146	6.613021	474.5403	15.36893	379.1059	85.67455	494.7	6.6	130.5	281
19AR112 Spot 134	130.2814	7844.684	2.461927	17.23851	2.576093	0.617462	3.064018	0.07988	0.748953	0.244435	495.4067	3.571377	488.2554	11.87728	455.8228	65.9395	495.4	3.6	108.7	92
19AR112 Spot 457	137.4761	6439.351	2.307458	16.98913	2.473863	0.621496	3.180538	0.079993	0.949644	0.29858	496.0838	4.534322	490.7846	12.37868	467.1124	67.24717	496.1	4.5	106.2	298
19AR112 Spot 553	316.6774	13754.7	3.770066	17.24595	1.646734	0.635706	1.904948	0.080979	0.775843	0.407278	501.9616	3.746669	499.6442	7.517477	489.9941	38.40206	502.0	3.7	102.4	360
19AR112 Spot 380	219.199	14323.24	7.67649	17.25501	1.617725	0.640889	1.965715	0.081777	0.777046	0.395299	506.7207	3.786676	502.8565	7.795836	486.2716	39.87911	506.7	3.8	104.2	252
19AR112 Spot 4	249.6448	11118.2	3.693589	17.27925	1.371056	0.645213	2.002971	0.081989	0.788576	0.393703	507.9846	3.852077	505.5285	7.976172	495.3857	40.5866	508.0	3.9	102.5	2
19AR112 Spot 574	438.3762	24018.1	7.428515	16.88605	1.263184	0.66443	1.674207	0.082314	0.798148	0.476732	509.9197	3.91311	517.3203	6.786243	551.0764	32.12927	509.9	3.9	92.5	376
19AR112 Spot 451	337.9434	14024.44	5.206076	17.36869	1.189956	0.640365	2.241548	0.082381	0.80515	0.359194	510.3164	3.950389	502.5319	8.88538	468.2271	46.32427	510.3	4.0	109.0	294
19AR112 Spot 493	197.5514	8231.272	2.516296	17.31738	2.270783	0.636049	2.6659	0.08259	0.723569	0.271416	511.5609	3.558439	499.8572	10.52407	447.5679	57.04027	511.6	3.6	114.3	325
19AR112 Spot 571	320.1008	30011.84	3.881351	17.13066	1.269599	0.662893	1.582833	0.083135	0.792511	0.500692	514.8075	3.92125	516.3821	6.406931	524.2891	30.04159	514.8	3.9	98.2	374
19AR112 Spot 263	335.8836	24303.43	12.2408	17.05942	1.372033	0.665463	1.741146	0.083362	1.024365	0.588328	516.1571	5.081198	517.95	7.064166	526.8417	30.85626	516.2	5.1	98.0	176
19AR112 Spot 304	321.5797	19839.6	1.798803	16.84251	1.526378	0.675049	1.803542	0.083386	0.635308	0.352256	516.3028	3.152198	523.7778	7.380261	557.4282	36.82137	516.3	3.2	92.6	206
19AR112 Spot 184	476.6464	27539.42	5.920913	17.41516	0.89739	0.653276	1.260633	0.083414	0.707255	0.561032	516.4708	3.510273	510.4927	5.05794	484.7894	23.06121	516.5	3.5	106.5	124
19AR112 Spot 257	105.1219	5041.651	1.584654	17.3674	2.86063	0.62971	3.412482	0.083573	0.774045	0.226828	517.4128	3.848498	495.9153	13.38923	398.8059	74.51462	517.4	3.8	129.7	171
19AR112 Spot 421	172.8236	6001.478	2.690734	17.56451	2.010037	0.625808	2.460861	0.083607	0.693169	0.281678	517.6138	3.447672	493.4812	9.618377	383.9516	53.06106	517.6	3.4	134.8	277
19AR112 Spot 182	191.8683	9050.914	1.980619	16.98173	1.758141	0.658727	3.370901	0.083629	0.919681	0.272829	517.7473	4.575425	513.8348	13.59353	497.4282	71.48376	517.7	4.6	104.1	122
19AR112 Spot 564	467.6671	25259.48	3.471597	17.17195	1.260378	0.664272	1.527244	0.083642	0.801447	0.524767	517.8268	3.987797	517.2235	6.189636	515.4938	28.56132	517.8	4.0	100.5	370
19AR112 Spot 488	213.7246	8632.03	4.240378	16.93698	1.557702	0.661544	1.971446	0.083743	0.721754	0.366104	518.4286	3.595275	515.5577	7.97022	503.8197	40.37375	518.4	3.6	102.9	322
19AR112 Spot 177	328.5353	17645.04	3.176769	17.08898	1.60433	0.663494	1.969768	0.083744	0.795306	0.403756	518.4324	3.961687	516.7489	7.977554	510.2707	39.59625	518.4	4.0	101.6	118
19AR112 Spot 349	203.6946	12727.62	3.833572	17.55127	1.576014	0.642702	2.046894	0.083795	0.709592	0.346668	518.737	3.536709	503.9778	8.131777	438.4739	42.73424	518.7	3.5	118.3	232
19AR112 Spot 259	284.3211	10852.47	1.91099	17.40234	1.38941	0.647449	1.923716	0.083841	0.714915	0.371632	519.0105	3.565044	506.9075	7.676665	453.6095	39.64674	519.0	3.6	114.4	173
19AR112 Spot 251	178.3543	7115.867	1.917516	17.41069	1.95253	0.640029	2.777184	0.083844	0.673014	0.242337	519.025	3.356191	502.3239	11.00524	427.9014	60.10766	519.0	3.4	121.3	167
19AR112 Spot 410	108.0907	6747.309	3.054456	18.03753	2.603892	0.614364	2.967679	0.083867	0.785372	0.264642	519.1606	3.917481	486.3085	11.46805	335.3029	64.8713	519.2	3.9	154.8	271
19AR112 Spot 508	234.0435	13169.47	5.561686	17.57104	1.492663	0.644437	1.947802	0.083881	0.716749	0.367979	519.2482	3.575764	505.0494	7.750794	442.1891	40.30683	519.2	3.6	117.4	334
19AR112 Spot 276	323.69	11484.8	12.24131	17.2803	1.404	0.652209	2.074445	0.083888	0.788443	0.380074	519.288	3.933726	509.8372	8.315022	468.6419	42.50659	519.3	3.9	110.8	186
19AR112 Spot 258	113.6017	6635.998	2.457502	17.47347	2.182377	0.637014	2.771341	0.084122	0.769487	0.277659	520.6797	3.849027	500.4555	10.95048	409.9579	59.53657	520.7	3.8	127.0	172
19AR112 Spot 277	78.54162	4245.284	1.190292	16.82953	4.212024	0.647046	5.966711	0.084134	0.824373	0.138162	520.7482	4.12409	506.6593	23.80532	444.4997	131.4993	520.7	4.1	117.2	187
19AR112 Spot 499	133.1873	6924.497	3.547177	17.29388	2.655184	0.646726	3.436555	0.084219	0.94051	0.273678	521.2591	4.709522	506.4621	13.70499	441.1285	73.54868	521.3	4.7	118.2	326
19AR112 Spot 112	172.7586	7598.485	1.851351	17.37675	1.667858	0.646987	2.534138	0.084256	0.852783	0.336518	521.4751	4.271937	506.623	10.10834	441.0666	53.09493	521.5	4.3	118.2	76
19AR112 Spot 47	244.2168	11096.22	3.710616	17.05375	1.506578	0.665412	1.823266	0.084296	0.613057	0.336241	521.7139	3.072399	517.9187	7.397014	502.1817	37.8073	521.7	3.1	103.9	34
19AR112 Spot 358	320.5015	21423.23	5.091425	17.2609	1.233526	0.663892	1.69647	0.084308	0.706656	0.416545	521.7875	3.541964	516.9917	6.873137	496.814	33.98241	521.8	3.5	105.0	238
19AR112 Spot 323	179.6179	8133.674	5.825171	17.08356	2.216014	0.659889	2.400334	0.084369	0.680975	0.2837	522.1466	3.415497	514.5462	9.689619	481.911	50.86099	522.1	3.4	108.3	217
19AR112 Spot 465	437.5522	15659.99	6.149072	17.2727	1.15286	0.660264	1.84093	0.084399	0.767022	0.416649	522.3248	3.848332	514.7755	7.433878	482.3817	36.9668	522.3	3.8	108.3	304
19AR112 Spot 587	248.2383	15664.38	13.53297	16.96955	1.600221	0.67502	1.829562	0.084442	0.745855	0.407668	522.5813	3.743896	523.7601	7.486548	529.8766	36.62435	522.6	3.7	98.6	384
19AR112 Spot 199	301.5006	18013.59	4.80656	17.19465	1.270749	0.668115	1.878449	0.084456	0.883901	0.470548	522.6662	4.437529	519.5654	7.63946	506.9189	36.44809	522.7	4.4	103.1	135
19AR112 Spot 232	144.3429	8221.802	3.409741	17.01585	2.677053	0.663841	3.328976	0.084518	0.796393	0.239231	523.0365	4.000924	516.9607	13.48709	491.1333	71.30379	523.0	4.0	106.5	159
19AR112 Spot 237	200.5537	11878.36	6.96617	16.98114	1.552368	0.671608	2.301635	0.084528	0.824089	0.358045	523.096	4.140516	521.6898	9.389883	516.4728	47.17539	523.1	4.1	101.3	163
19AR112 Spot 459	334.4049	15698.05	3.037378	17.1091	1.242163	0.668033	2.047338	0.084566	0.727872	0.355521	523.3178	3.658572	519.516	8.325737	503.8091	42.11606	523.3	3.7	103.9	300
19AR112 Spot 221	154.5429	7791.451	3.820666	17.15676	2.36043	0.658388	3.322579	0.08458	0.903649	0.271972	523.4033	4.542812	513.6272	13.39449	471.32	70.77835	523.4	4.5	111.1	152
19AR112 Spot 455	179.8496	6742.47	1.482181	17.64882	1.42502	0.63343	2.361564	0.084588	0.805315	0.341009	523.4526	4.048833	498.23	9.299073	384.9152	49.86321	523.5	4.0	136.0	297
19AR112 Spot 140	177.9044	7987.369	3.42915	17.55723	1.680962	0.643568	2.038877	0.084708	0.69883	0.342752	524.1661	3.518061	504.5127	8.106562	417.309	42.79053	524.2	3.5	125.6	96
19AR112 Spot 292	102.3611	6707.343	2.169623	16.36649	4.018865	0.691002	5.235592	0.084754	0.964301	0.184182	524.4375	4.856917	533.4021	21.72686	572.8568	111.9917	524.4	4.9	91.5	198
19AR112 Spot 136	240.0231	11350.21	3.362075	17.15333	1.82604	0.665244	2.665692	0.08476	0.772979	0.289973	524.4695	3.893507	517.8164	10.81332	489.5055	56.2885	524.5	3.9	107.1	93
19AR112 Spot 288	529.0065	29322.99	3.158667	17.17969	0.92817	0.67568	1.377637	0.084763	0.785462	0.570151	524.4927	3.956552	524.1599	5.640519	523.6444	24.84386	524.5	4.0	100.2	194
19AR112 Spot 2	399.7849	20738.5	13.23926	17.17287	1.402562	0.67798	1.811249	0.084779	0.844697	0.466361	524.5855	4.255654	525.553	7.43098	530.7331	35.09993	524.6	4.3	98.8	1
19AR112 Spot 131	212.7539	10861.26	2.123049	17.03542	2.459144	0.669812	2.655536	0.084826	0.80618	0.303585	524.8659	4.063688	520.5983	10.81643	502.8726	55.71909	524.9	4.1	104.4	91
19AR112 Spot 352	168.2058	8786.858	4.034379	16.48681	2.508233	0.688361	2.881679	0.084832	0.803617	0.278871	524.8996	4.05102	531.8151	11.93016	562.5244	60.29389	524.9	4.1	93.3	234
19AR112 Spot 116	147.2804	7135.878	2.973572	17.06242	1.757442	0.661573	3.997332	0.084887	0.824203	0.206188	525.2274	4.15728	515.5758	16.162	473.9633	86.56247	525.2	4.2	110.8	79
19AR112 Spot 28	334.043	18033.65	2.533885	17.43279	1.309217	0.661142	1.583674	0.084893	0.708056	0.447097	525.2649	3.571683	515.3125	6.400141	472.3598	31.34908	525.3	3.6	111.2	21
19AR112 Spot 300	290.7423	18878.54	2.930911	17.0598	1.016768	0.679642	1.623191	0.084898	0.848861	0.522958	525.2929	4.28217	526.558	6.669133	533.0209	30.2973	525.3	4.3	98.6	203
19AR112 Spot 484	388.82	20365.99	2.458364	17.14263	0.825929	0.675394	1.196641	0.085057	0.790287	0.660421	526.2364	3.99356	523.9867	4.898208	515.1298	19.72541	526.2	4.0	102.2	320
19AR112 Spot 44	171.5049	12061.72	1.35848	16.9724	2.070044	0.675474	2.416954	0.085074	0.757742	0.313511	526.3378	3.829805	524.0355	9.894255	514.9506	50.41462	526.3	3.8	102.2	31
19AR112 Spot 291	238.4936	11788.78	2.78961	17.55047	1.13011	0.656063	1.988779	0.085081	0.776026	0.390203	526.3782	3.922511	512.203	8.000076	450.3732	40.67223	526.4	3.9	116.9	197
19AR112 Spot 336	238.4139	14521.03	2.575022	17.37917	2.045442	0.663612	2.381822	0.085149	0.789111	0.331306	526.7848	3.991605	516.8207	9.647489	473.9473	49.71545	526.8	4.0	111.1	223
19AR112 Spot 343	158.1748	9155.956	2.630532	17.42709	3.386198	0.654865	3.538555	0.085172	0.847482	0.239499	526.9177	4.287902	511.4684	14.21915	443.9426	76.40987	526.9	4.3	118.7	229
19AR112 Spot 108	308.8779	15082.48	5.582089	17.42508	1.351071	0.662398	1.777752	0.085214	0.672281	0.378164	527.1684	3.403014	516.0798	7.192709	468.2485	36.45065	527.2	3.4	112.6	74
19AR112 Spot 118	189.6197	4797.305	4.539065	16.65936	1.605889	0.668918	2.878718	0.085217	0.822466	0.285706	527.1848	4.163358	520.0544	11.71618	489.8127	60.87988	527.2	4.2	107.6	81
19AR112 Spot 160	179.2968	3550.811	2.010528	16.80709	1.801525	0.650316	5.021835	0.085277	0.730274	0.14542	527.5417	3.69908	508.6734	20.09586	425.673	110.8876	527.5	3.7	123.9	103
19AR112 Spot 204	172.8933	10350	3.330312	17.67711	1.696619	0.649203	2.673669	0.085365	0.684909	0.256168	528.0648	3.47259	507.9882	10.6871	419.5451	57.71842	528.1	3.5	125.9	140
19AR112 Spot 312	186.6151	14529.91	4.978205	16.98314	2.037117	0.680764	2.41443	0.085395	0.727466	0.301299	528.2431	3.689555	527.236	9.929975	523.8121	50.50282	528.2	3.7	100.8	214
19AR112 Spot 230	155.1825	9350.129	4.094726	17.50592	2.547668	0.654069	3.169275	0.085424	1.169115	0.36889	528.4184	5.9314	510.9797	12.72572	434.6432	65.63894	528.4	5.9	121.6	157
19AR112 Spot 596	216.4793	18721.6	14.85658	17.13585	1.343766	0.681085	1.803277	0.085475	0.81977	0.4546	528.7202	4.16131	527.4303	7.418428	522.7862	35.24418	528.7	4.2	101.1	389
19AR112 Spot 38	202.0738	11165.7	6.76014	16.86451	1.563327	0.68314	2.355825	0.085527	0.750525	0.318582	529.0258	3.811917	528.6703	9.709025	528.1102	48.9641	529.0	3.8	100.2	29
19AR112 Spot 86	178.2526	8941.341	1.661806	17.2487	2.073591	0.663279	2.771228	0.085661	0.840643	0.303347	529.8268	4.27583	516.6177	11.2215	459.5703	58.56536	529.8	4.3	115.3	60
19AR112 Spot 254	266.5577	15403.3	4.542218	17.07184	1.728539	0.680617	2.43385	0.085679	0.697302	0.286502	529.9343	3.547436	527.1476	10.00857	516.0338	51.23575	529.9	3.5	102.7	169
19AR112 Spot 582	157.6457	8807.655	3.279523	17.26023	2.185972	0.666614	2.561016	0.085998	1.148035	0.448273	531.8251	5.860476	518.6518	10.40152	461.9813	50.76395	531.8	5.9	115.1	380
19AR112 Spot 388	166.3983	9507.016	2.121397	17.07087	2.593614	0.675927	2.896951	0.086061	0.884616	0.305361	532.2002	4.518828	524.31	11.86412	491.034	60.8679	532.2	4.5	108.4	258
19AR112 Spot 94	87.80904	4383.105	3.089346	17.182	3.828292	0.649094	5.80097	0.086062	0.937318	0.161579	532.2034	4.788071	507.9208	23.18825	400.9803	128.3602	532.2	4.8	132.7	65
19AR112 Spot 296	208.6775	11489.68	4.75847	17.34228	1.812066	0.671565	2.215485	0.086083	0.87353	0.394284	532.3288	4.463234	521.6635	9.038053	476.1786	45.02375	532.3	4.5	111.8	201
19AR112 Spot 282	146.6451	6123.941	2.265634	17.54078	2.485948	0.647234	4.98972	0.086179	0.803167	0.160964	532.9018	4.107957	506.7753	19.90985	391.4839	110.5567	532.9	4.1	136.1	190
19AR112 Spot 436	155.688	14949.67	2.238217	17.35368	2.011822	0.671638	2.513575	0.086237	0.873025	0.347324	533.2446	4.468016	521.7077	10.25485	472.4565	52.15859	533.2	4.5	112.9	284
19AR112 Spot 104	203.9724	14710.34	3.32584	17.85926	2.02186	0.654455	2.60154	0.086553	0.73813	0.283728	535.1165	3.79036	511.2166	10.44962	406.6628	55.84233	535.1	3.8	131.6	72
19AR112 Spot 558	210.4653	10654.38	4.423009	16.86186	1.486409	0.691515	2.404022	0.086651	0.770239	0.320396	535.6979	3.959367	533.71	9.979481	526.1978	49.92149	535.7	4.0	101.8	365
19AR112 Spot 168	260.1153	14071.74	3.621506	16.38718	1.481899	0.715314	1.844893	0.086766	0.679611	0.368374	536.382	3.497775	547.8965	7.812007	597.0339	37.15569	536.4	3.5	89.8	111
19AR112 Spot 97	417.8713	29106.92	2.719856	17.1158	0.93616	0.690927	1.308817	0.086825	0.759683	0.580435	536.7306	3.912321	533.3575	5.430256	519.8892	23.39017	536.7	3.9	103.2	68
19AR112 Spot 437	323.071	20581.62	8.080087	17.36001	1.445911	0.682271	1.754224	0.087225	0.93653	0.533871	539.1021	4.843502	528.146	7.224089	482.0542	32.76679	539.1	4.8	111.8	285
19AR112 Spot 95	661.0625	40540.91	7.315127	16.90421	0.855191	0.705563	1.310713	0.087348	0.818974	0.624831	539.8364	4.241063	542.1083	5.505667	552.6331	22.32447	539.8	4.2	97.7	66
19AR112 Spot 384	301.4636	13663.31	5.521557	16.88729	1.218402	0.699743	1.445827	0.087432	0.628236	0.434517	540.3344	3.256203	538.6371	6.043751	532.4325	28.51909	540.3	3.3	101.5	254
19AR112 Spot 61	220.601	7506.293	1.767301	16.81195	1.920645	0.698233	2.333941	0.08795	0.70215	0.300843	543.4047	3.659125	537.7347	9.743972	514.6967	48.87669	543.4	3.7	105.6	42
19AR112 Spot 149	205.8548	11555.28	3.668717	17.01077	1.963379	0.706019	2.81002	0.088871	0.766873	0.272907	548.8593	4.03485	542.3799	11.80842	516.1745	59.35445	548.9	4.0	106.3	99
19AR112 Spot 454	109.2729	7903.594	2.850635	16.88863	3.379762	0.708628	4.528315	0.089889	0.79067	0.174606	554.8788	4.203748	543.9313	19.07166	499.2567	98.26324	554.9	4.2	111.1	296
19AR112 Spot 30	214.7148	2740.533	11.28492	15.92932	1.573899	0.717039	2.908047	0.090581	0.903784	0.310787	558.9703	4.839054	548.9177	12.33149	508.3905	60.81073	559.0	4.8	109.9	22
19AR112 Spot 557	251.5629	20456.68	1.616177	17.05315	1.264335	0.728477	1.659305	0.091177	0.762759	0.459686	562.4937	4.108614	555.6589	7.100933	528.7273	32.30841	562.5	4.1	106.4	364
19AR112 Spot 403	142.8641	6873.421	3.02013	16.56948	2.327549	0.731878	2.862169	0.091366	0.881417	0.307954	563.61	4.756782	557.655	12.28197	534.3534	59.61618	563.6	4.8	105.5	266
19AR112 Spot 597	669.3806	2630.685	83.93738	15.69974	0.76287	0.741554	1.83565	0.092108	0.594461	0.323842	567.9901	3.232002	563.312	7.936601	545.3938	37.93111	568.0	3.2	104.1	390
19AR112 Spot 308	254.906	14932.34	24.09503	15.88068	1.843997	0.790876	2.543122	0.092575	1.710652	0.672658	570.748	9.343781	591.6689	11.40403	673.6903	40.23613	570.7	9.3	84.7	210
19AR112 Spot 46	285.1887	14314.68	3.642559	13.71565	1.056889	0.932685	1.519643	0.094193	0.860124	0.566004	580.2858	4.773122	669.0462	7.446517	981.6152	25.51482	580.3	4.8	59.1	33
19AR112 Spot 185	260.3414	6273.36	0.80289	16.70945	1.502408	0.746815	2.616904	0.0943	0.728495	0.27838	580.9207	4.046896	566.3749	11.36062	509.3377	55.25786	580.9	4.0	114.1	125
19AR112 Spot 382	256.611	18329.83	3.089002	16.55246	1.026117	0.777119	1.685771	0.094695	0.978473	0.580431	583.2456	5.456349	583.8389	7.48527	587.1124	29.78728	583.2	5.5	99.3	253
19AR112 Spot 270	270.2299	17132.94	5.959238	16.24538	1.238919	0.793559	1.64959	0.095161	0.904207	0.54814	585.9896	5.064872	593.1887	7.411008	621.7385	29.7856	586.0	5.1	94.3	181
19AR112 Spot 58	581.1733	20042.5	3.473067	16.59308	0.790546	0.782254	1.106833	0.09523	0.702114	0.634345	586.3915	3.935431	586.7681	4.932798	589.149	18.54889	586.4	3.9	99.5	41
19AR112 Spot 262	285.7894	20652.75	0.746897	16.51189	1.515733	0.789604	1.859291	0.095736	0.697511	0.375149	589.3741	3.928625	590.9471	8.329892	597.9218	37.34158	589.4	3.9	98.6	175
19AR112 Spot 92	213.0435	6871.588	5.72227	16.20249	1.882243	0.784631	2.803377	0.095741	0.847733	0.302397	589.4016	4.774936	588.122	12.51557	584.1104	58.01757	589.4	4.8	100.9	63
19AR112 Spot 19	165.052	9652.19	0.838646	17.1649	2.47627	0.751699	3.009783	0.096147	0.898534	0.298538	591.7907	5.080665	569.2097	13.11516	480.9396	63.48858	591.8	5.1	123.0	14
19AR112 Spot 414	93.88185	6141.113	1.815216	14.48274	5.569521	0.881585	8.459551	0.096267	5.369055	0.634674	592.4942	30.39344	641.8381	40.26661	820.505	136.6639	592.5	30.4	72.2	274
19AR112 Spot 418	340.9067	28071.85	3.79807	16.53572	0.807884	0.793016	1.057851	0.09634	0.641533	0.606449	592.9234	3.634107	592.8814	4.750677	593.6847	18.21017	592.9	3.6	99.9	275
19AR112 Spot 161	94.48624	6886.399	3.028851	17.03066	2.864895	0.752331	3.57258	0.096546	0.711296	0.199099	594.1366	4.037171	569.5762	15.57539	473.6875	77.46778	594.1	4.0	125.4	104
19AR112 Spot 485	254.2455	12797.22	4.640839	16.33877	1.164523	0.80086	1.778269	0.096741	1.14057	0.641394	595.2817	6.485552	597.3136	8.029953	605.9567	29.53136	595.3	6.5	98.2	321
19AR112 Spot 245	159.681	6633.642	3.463105	16.16747	1.628559	0.795221	3.42052	0.096813	0.891551	0.260648	595.7058	5.073015	594.129	15.38598	589.0767	71.65759	595.7	5.1	101.1	165
19AR112 Spot 78	306.7696	15930.1	0.831824	16.64708	1.157234	0.790851	1.525531	0.097148	0.787382	0.516136	597.6759	4.494424	591.6544	6.840577	569.5964	28.43219	597.7	4.5	104.9	54
19AR112 Spot 445	113.0532	7132.296	2.657808	16.00036	2.356251	0.818498	2.960348	0.098353	0.803196	0.271318	604.7471	4.636432	607.2103	13.53017	617.3321	61.52275	604.7	4.6	98.0	292
19AR112 Spot 376	260.0663	11857.84	1.65198	16.38193	1.314498	0.810131	2.062953	0.098391	0.705236	0.341857	604.9739	4.072412	602.5277	9.375121	594.2708	42.0378	605.0	4.1	101.8	250
19AR112 Spot 483	42.05079	2425.308	2.453716	14.19731	5.19079	0.879556	9.58732	0.099015	1.220707	0.127325	608.6345	7.089696	640.743	45.58554	756.5429	201.0707	608.6	7.1	80.4	319
19AR112 Spot 202	99.07027	5049.95	39.39265	15.89802	2.785477	0.820283	5.255208	0.099224	0.741657	0.141128	609.8569	4.315688	608.2063	24.0506	602.9838	112.633	609.9	4.3	101.1	138
19AR112 Spot 228	249.1279	11886.45	6.790726	15.4089	1.137403	0.889316	1.984831	0.101265	0.954805	0.481051	621.818	5.659791	646.0019	9.486753	732.4133	36.86038	621.8	5.7	84.9	156
19AR112 Spot 306	392.8029	22278.85	6.248631	16.3421	0.953938	0.850242	1.190007	0.101731	0.689774	0.579639	624.5456	4.105851	624.7816	5.552617	626.5538	20.92255	624.5	4.1	99.7	208
19AR112 Spot 590	345.9209	18131.12	5.922292	16.44042	1.289985	0.844868	1.736239	0.101961	0.673006	0.387623	625.8914	4.01426	621.8287	8.073693	607.992	34.61819	625.9	4.0	102.9	385
19AR112 Spot 285	230.1449	16359.36	3.985375	15.99296	1.495976	0.867697	2.129224	0.102037	0.920026	0.432095	626.3327	5.491339	634.3161	10.04447	663.8204	41.15766	626.3	5.5	94.4	192
19AR112 Spot 473	90.57399	6963.888	1.779474	16.04514	2.39301	0.853939	3.205462	0.103042	0.763195	0.238092	632.2138	4.595972	626.8088	14.99283	608.2721	67.3198	632.2	4.6	103.9	310
19AR112 Spot 322	66.42456	4162.02	3.023847	15.85437	4.49331	0.847892	4.974509	0.103259	0.825011	0.165848	633.4806	4.977706	623.4912	23.18032	588.3438	106.456	633.5	5.0	107.7	216
19AR112 Spot 189	347.2711	16565.43	4.812522	16.33312	0.9118	0.860108	1.257093	0.103379	0.67183	0.534431	634.1814	4.057753	630.182	5.902246	616.7773	22.94267	634.2	4.1	102.8	128
19AR112 Spot 518	236.3119	28324.95	10.78465	16.53913	1.261969	0.866573	1.742025	0.104819	0.856232	0.491515	642.5911	5.23673	633.705	8.212097	603.0552	32.84008	642.6	5.2	106.6	343
19AR112 Spot 271	268.122	11712.16	0.962245	15.94141	1.135953	0.885557	1.381373	0.104839	0.692397	0.501238	642.7043	4.235423	643.9794	6.587548	649.401	25.68894	642.7	4.2	99.0	182
19AR112 Spot 359	347.2009	24154.32	5.924222	16.4325	0.981015	0.870162	1.368732	0.105002	0.915412	0.668803	643.6532	5.60748	635.6555	6.46659	608.2412	22.00174	643.7	5.6	105.8	239
19AR112 Spot 33	302.8855	22809.98	2.844534	16.17946	1.073281	0.884868	1.579515	0.105074	0.908921	0.575443	644.0773	5.571205	643.6086	7.529381	642.9213	27.78588	644.1	5.6	100.2	25
19AR112 Spot 196	143.8951	8146.251	2.563513	15.7228	2.524437	0.895445	2.920495	0.105085	0.764838	0.261886	644.1407	4.688491	649.2904	14.01012	668.1472	60.36726	644.1	4.7	96.4	132
19AR112 Spot 513	214.5211	18643.42	7.874372	15.4032	1.194055	0.931861	2.074112	0.10544	1.503211	0.724749	646.2128	9.242937	668.6131	10.15902	745.7573	30.19045	646.2	9.2	86.7	339
19AR112 Spot 183	171.6517	11344.47	6.417295	16.28716	2.274184	0.872775	2.540819	0.105534	0.762882	0.30025	646.7605	4.694579	637.073	12.02377	603.7793	52.44837	646.8	4.7	107.1	123
19AR112 Spot 235	228.0371	15816.17	19.77983	15.60827	1.141269	0.920138	2.193038	0.10574	1.810607	0.825616	647.9606	11.16168	662.4329	10.67118	712.9106	26.27525	648.0	11.2	90.9	161
19AR112 Spot 357	304.9851	18927.24	22.92733	15.84745	1.098835	0.909759	2.143851	0.106119	1.414747	0.659909	650.1695	8.749612	656.9293	10.3702	681.1201	34.4036	650.2	8.7	95.5	237
19AR112 Spot 110	163.3419	12760.69	4.581889	15.96863	1.956084	0.900426	2.394035	0.106291	0.9145	0.381991	651.1702	5.664065	651.9552	11.51797	655.6279	47.46738	651.2	5.7	99.3	75
19AR112 Spot 340	386.2788	32225.79	4.434046	16.07193	0.985165	0.914736	1.353779	0.107453	0.823884	0.608581	657.9387	5.153208	659.5724	6.567054	666.1059	22.9827	657.9	5.2	98.8	226
19AR112 Spot 99	485.0286	34517.1	21.13495	15.5966	0.867121	0.959452	1.237475	0.109763	0.821472	0.663829	671.3717	5.237655	683.0125	6.152625	722.4638	19.61565	671.4	5.2	92.9	70
19AR112 Spot 330	997.9253	48283.11	1.578694	15.99596	0.692415	0.962296	1.090297	0.112261	0.76116	0.698121	685.8639	4.952395	684.4852	5.429039	680.8641	16.66942	685.9	5.0	100.7	220
19AR112 Spot 286	286.1475	21338.36	2.432914	16.04707	1.227094	0.959871	1.45595	0.112884	0.610629	0.419403	689.4765	3.992813	683.2297	7.240508	663.6185	28.30288	689.5	4.0	103.9	193
19AR112 Spot 479	260.708	16894.97	6.08157	15.13915	1.02706	1.025475	1.774125	0.114128	1.190582	0.671081	696.6767	7.862031	716.6615	9.120611	780.6478	27.646	696.7	7.9	89.2	315
19AR112 Spot 353	204.1826	18269.6	4.287745	14.7745	1.155475	1.057078	2.026544	0.114694	1.532281	0.756106	699.9506	10.16346	732.3821	10.57445	833.8584	27.64147	700.0	10.2	83.9	235
19AR112 Spot 290	387.5709	22139.53	2.283304	15.64953	0.847956	1.031416	1.308911	0.117886	0.826461	0.631411	718.3874	5.618325	719.6357	6.748097	724.4322	21.50757	718.4	5.6	99.2	196
19AR112 Spot 62	347.9752	13641.42	1.252657	14.75608	1.075121	1.086676	1.439944	0.118059	0.8755	0.60801	719.3813	5.95948	746.8878	7.614281	831.1058	23.85491	719.4	6.0	86.6	43
19AR112 Spot 506	311.4098	20748.9	5.198552	14.79186	0.891567	1.13885	1.545521	0.123558	0.764918	0.494926	751.008	5.422602	771.9636	8.356046	833.9812	27.98889	751.0	5.4	90.1	333
19AR112 Spot 216	207.9796	11398.03	5.311916	14.30013	1.030938	1.177079	1.805135	0.124231	1.344828	0.745001	754.8725	9.579898	789.9517	9.910254	891.2157	24.86878	754.9	9.6	84.7	148
19AR112 Spot 146	374.2983	26822.86	9.246457	15.32326	0.802501	1.158033	1.124611	0.129622	0.700393	0.622787	785.7068	5.180891	781.0297	6.127738	768.5861	18.52978	785.7	5.2	102.2	98
19AR112 Spot 203	278.3493	25298.86	6.7738	14.61229	0.998809	1.227474	1.338872	0.131185	0.634718	0.474069	794.6225	4.745149	813.1882	7.491636	865.2652	24.44673	794.6	4.7	91.8	139
19AR112 Spot 407	165.0881	16642.78	4.738917	14.46222	1.274282	1.251673	1.98985	0.133227	1.358055	0.682491	806.2461	10.29224	824.1595	11.2319	873.6805	30.11472	806.2	10.3	92.3	270
19AR112 Spot 556	399.6862	29046.99	12.47788	14.47224	0.761928	1.278912	1.223178	0.135183	0.894816	0.73155	817.3675	6.869247	836.3693	6.970108	888.059	17.22221	817.4	6.9	92.0	363
19AR112 Spot 25	223.6619	16722.98	2.666052	14.29227	1.023985	1.287919	1.742786	0.135308	0.97	0.55658	818.074	7.452454	840.3742	9.961751	900.6415	29.85829	818.1	7.5	90.8	20
19AR112 Spot 599	176.8269	14445.87	8.819925	14.26336	1.369661	1.323791	2.038147	0.138403	1.102174	0.540773	835.6254	8.638099	856.1708	11.78982	910.6446	35.31788	835.6	8.6	91.8	391
19AR112 Spot 68	179.9781	14274.23	5.775988	14.30909	1.051179	1.318712	4.737197	0.139032	4.55322	0.961163	839.1863	35.8278	853.9493	27.36275	893.379	26.99286	839.2	35.8	93.9	49
19AR112 Spot 274	124.9735	10010.74	4.16156	14.39938	2.117379	1.310234	2.488672	0.140245	0.880877	0.353955	846.0503	6.984318	850.2296	14.33241	862.0338	48.27757	846.1	7.0	98.1	185
19AR112 Spot 16	236.6399	11270.99	3.694051	14.30395	0.793923	1.334338	1.193066	0.140946	0.657518	0.551117	850.0072	5.236156	860.7691	6.924734	889.4811	20.56046	850.0	5.2	95.6	11
19AR112 Spot 208	137.9303	13170.84	6.614168	14.44655	1.893894	1.32629	2.361006	0.141097	0.89875	0.380664	850.8611	7.163939	857.2623	13.66872	874.7656	45.22753	850.9	7.2	97.3	142

19AR112 Spot 264	328.8572	28442.1	26.81648	14.20875	0.827723	1.367357	1.44627	0.142226	1.090816	0.754227	857.2399	8.755842	875.0308	8.482181	921.2348	19.54134	857.2	8.8	93.1	177					
19AR112 Spot 522	158.8195	19180.36	2.715759	14.08786	1.424858	1.376351	1.84511	0.142234	1.123491	0.608902	857.281	9.018525	878.8813	10.85144	934.5558	30.0448	857.3	9.0	91.7	346					
19AR112 Spot 543	546.5576	35328.19	27.0872	14.26541	0.604779	1.435471	1.104384	0.14955	0.799513	0.723945	898.4426	6.705075	903.8332	6.609485	917.946	15.68353	917.9	15.7	97.9	354					
19AR112 Spot 526	190.9183	18331.43	4.753935	14.03996	1.07486	1.54558	1.389398	0.159163	0.755434	0.543713	952.1231	6.686707	948.7319	8.565875	941.7836	23.89406	941.8	23.9	101.1	348					
19AR112 Spot 190	228.7198	19775.95	4.473733	14.03109	1.242984	1.410805	1.490913	0.145061	0.803244	0.53876	873.2192	6.559769	893.4973	8.859277	944.9234	25.71744	944.9	25.7	92.4	129					
19AR112 Spot 169	720.7016	13793.46	5.617866	13.91417	0.635824	1.449011	1.650131	0.148967	0.652612	0.395491	895.1681	5.454499	909.4625	9.913868	945.2078	31.03386	945.2	31.0	94.7	112					
19AR112 Spot 80	334.4523	18255.83	0.453714	13.9694	0.870302	1.523427	1.238316	0.156469	0.817554	0.660214	937.1253	7.130658	939.8567	7.591024	947.1616	19.04181	947.2	19.0	98.9	56					
19AR112 Spot 469	106.5698	11492.98	4.004346	13.82547	1.802901	1.604086	2.391519	0.164076	0.812006	0.339536	979.388	7.378041	971.8046	14.95917	955.5683	46.00078	955.6	46.0	102.5	308					
19AR112 Spot 197	170.7088	20313.48	2.453069	13.95439	1.103841	1.56431	1.481606	0.159958	0.9037	0.609946	956.5443	8.033536	956.1755	9.177549	956.1987	23.98582	956.2	24.0	100.0	133					
19AR112 Spot 179	250.479	17231.27	3.123486	13.89704	1.140409	1.488267	1.719827	0.152158	0.857673	0.498697	913.0494	7.301684	925.6093	10.44513	956.5243	30.49457	956.5	30.5	95.5	120					
19AR112 Spot 174	414.5841	19062.23	3.601285	13.88887	0.833789	1.394755	1.353146	0.142545	0.945222	0.698536	859.0396	7.602067	886.7147	8.002403	957.2731	19.80264	957.3	19.8	89.7	116					
19AR112 Spot 534	78.66878	8785.45	5.017968	13.70869	1.981593	1.587889	2.369043	0.161654	0.761711	0.321527	965.9634	6.833113	965.4692	14.76073	965.2519	45.78544	965.3	45.8	100.1	350					
19AR112 Spot 572	424.3221	50865.34	3.998874	13.95452	0.817147	1.440067	1.191767	0.146471	0.855794	0.718088	881.1517	7.048172	905.7476	7.141826	967.1083	16.91476	967.1	16.9	91.1	375					
19AR112 Spot 378	222.8878	6493.082	5.15969	13.5792	0.950602	1.460967	2.71546	0.148442	0.768092	0.282859	892.2221	6.399977	914.4078	16.36988	969.255	53.13316	969.3	53.1	92.1	251					
19AR112 Spot 315	125.3405	14413.18	4.392693	13.79593	1.795434	1.630246	2.074463	0.165632	0.76344	0.368018	988.0016	6.993218	981.954	13.05618	969.3639	39.35197	969.4	39.4	101.9	215					
19AR112 Spot 480	398.2212	12968.84	1.862331	13.78618	0.69773	1.606081	1.652016	0.163147	0.559015	0.338383	974.2421	5.054592	972.5823	10.33805	969.7348	31.71843	969.7	31.7	100.5	316					
19AR112 Spot 422	306.4946	10371.47	8.617092	13.68636	0.781049	1.642156	2.050457	0.166792	0.702931	0.342817	994.4101	6.477568	986.5414	12.94077	969.9539	39.30174	970.0	39.3	102.5	278					
19AR112 Spot 171	252.9832	26010.08	4.693657	13.83574	0.888079	1.5163	1.37913	0.153941	1.002202	0.726691	923.018	8.618758	936.9849	8.438551	970.8509	19.32654	970.9	19.3	95.1	114					
19AR112 Spot 541	99.63423	10969.72	4.657105	13.73249	1.594035	1.643195	2.18945	0.166823	0.610699	0.278928	994.5842	5.628558	986.9406	13.82139	970.8894	42.89028	970.9	42.9	102.4	353					
19AR112 Spot 475	151.3692	17554.39	3.775826	13.76303	1.46886	1.623969	1.593797	0.164283	0.573988	0.360139	980.5355	5.221025	979.5279	10.01604	978.1601	30.2972	978.2	30.3	100.2	312					
19AR112 Spot 180	387.5379	45909.34	2.967801	13.82754	0.831416	1.59232	1.198408	0.160808	0.815234	0.680264	961.2674	7.280291	967.2066	7.474537	981.6348	17.86947	981.6	17.9	97.9	121					
19AR112 Spot 195	369.425	4336.08	3.578127	13.30379	0.817313	1.616555	1.328522	0.163186	0.793431	0.597229	974.4583	7.175656	976.6548	8.334297	982.5038	21.68003	982.5	21.7	99.2	131					
19AR112 Spot 386	509.427	4151.856	3.724528	13.25906	0.934629	1.543238	3.560449	0.155774	1.954243	0.548875	933.2464	16.97926	947.7972	21.94058	982.6531	60.5947	982.7	60.6	95.0	256					
19AR112 Spot 372	72.64428	9079.718	2.315027	13.57759	2.61388	1.687682	2.908759	0.170075	0.744097	0.255812	1012.525	6.972283	1003.888	18.54807	985.9686	57.2391	986.0	57.2	102.7	248					
19AR112 Spot 213	128.5449	15186.78	2.173748	13.6918	1.125168	1.644523	1.604678	0.165322	0.773092	0.481774	986.2835	7.070232	987.4505	10.13269	990.9499	28.57861	990.9	28.6	99.5	146					
19AR112 Spot 390	745.4878	22924.02	5.459416	13.68205	0.564073	1.597402	1.019334	0.160054	0.698532	0.685283	957.0757	6.212871	969.1951	6.36542	997.6427	15.06243	997.6	15.1	95.9	259					
19AR112 Spot 555	267.1881	19364.51	4.840518	13.65738	0.795381	1.588036	1.202173	0.158873	0.756942	0.629645	950.5123	6.689535	965.5268	7.490224	1000.734	18.94604	1000.7	18.9	95.0	362					
19AR112 Spot 478	535.5036	12393.4	3.570541	13.56172	0.603614	1.60476	1.029686	0.160518	0.701068	0.680856	959.6545	6.251012	972.0674	6.44144	1001.095	15.30574	1001.1	15.3	95.9	314					
19AR112 Spot 273	301.0267	12125.6	14.65232	13.52554	0.711061	1.559715	1.190703	0.155956	0.896543	0.752953	934.2651	7.79742	954.3545	7.367065	1001.827	15.89933	1001.8	15.9	93.3	184					
19AR112 Spot 164	194.4545	20693.03	2.871444	13.59465	0.975095	1.696498	1.258768	0.169536	0.687323	0.546028	1009.557	6.422861	1007.213	8.041519	1002.983	21.38787	1003.0	21.4	100.7	107					
19AR112 Spot 520	348.0262	56987.72	6.706092	13.70424	0.822286	1.604237	1.201556	0.160125	0.855176	0.711724	957.4726	7.609017	971.8633	7.515705	1005.406	17.11981	1005.4	17.1	95.2	344					
19AR112 Spot 125	432.9967	5822.47	3.279928	13.30979	0.793227	1.73164	1.26099	0.172805	0.756136	0.599637	1027.549	7.182063	1020.361	8.116801	1005.835	20.46504	1005.8	20.5	102.2	86					
19AR112 Spot 396	142.3955	12379.82	2.16287	13.51594	0.873045	1.754503	1.366638	0.175073	0.722596	0.52874	1040.004	6.940155	1028.824	8.839046	1005.989	23.51779	1006.0	23.5	103.4	263					
19AR112 Spot 460	218.2174	25914.44	4.599366	13.59605	0.819537	1.741647	1.257704	0.173727	0.652688	0.518952	1032.612	6.227647	1024.074	8.112718	1006.736	21.79271	1006.7	21.8	102.6	301					
19AR112 Spot 510	277.1084	5003.702	4.987934	13.21509	0.876135	1.705303	1.367693	0.170093	0.932934	0.682122	1012.624	8.742497	1010.523	8.754172	1006.836	20.27122	1006.8	20.3	100.6	336					
19AR112 Spot 387	167.7549	15661.54	1.242873	13.54802	1.026758	1.719585	1.485448	0.171393	0.670823	0.451596	1019.784	6.327288	1015.87	9.537205	1008.305	26.86393	1008.3	26.9	101.1	257					
19AR112 Spot 88	107.1471	13472.3	5.629187	13.48394	1.122774	1.73899	1.734797	0.173173	0.868765	0.500788	1029.569	8.266824	1023.089	11.18414	1010.115	30.45011	1010.1	30.5	101.9	61					
19AR112 Spot 537	380.2737	34138.05	5.205059	13.63555	0.810122	1.63878	1.639465	0.163179	1.356419	0.827355	974.4207	12.26679	985.2432	10.33866	1010.297	18.665	1010.3	18.7	96.4	352					
19AR112 Spot 294	118.7052	9855.844	2.868816	13.53009	1.125174	1.769548	1.598157	0.176153	0.82113	0.513798	1045.926	7.927887	1034.354	10.36854	1010.836	27.78121	1010.8	27.8	103.5	199					
19AR112 Spot 545	132.5767	16835.21	1.994022	13.54056	1.350156	1.770704	1.556162	0.176107	0.692395	0.444937	1045.674	6.683487	1034.778	10.09846	1012.688	28.24246	1012.7	28.2	103.3	355					
19AR112 Spot 165	225.4006	20315.65	3.417557	13.5258	0.738624	1.714111	1.219917	0.17045	0.613267	0.502712	1014.59	5.75721	1013.824	7.823121	1013.034	21.37463	1013.0	21.4	100.2	108					
19AR112 Spot 393	374.515	29972.46	3.638266	13.60081	0.683671	1.684284	1.075306	0.167412	0.652228	0.606552	997.8362	6.029487	1002.603	6.85102	1013.903	17.30742	1013.9	17.3	98.4	260					
19AR112 Spot 370	173.1438	18047.06	2.602401	13.52139	0.948884	1.755723	1.288378	0.174304	0.829579	0.643894	1035.779	7.937829	1029.274	8.334956	1016.335	19.94998	1016.3	19.9	101.9	247					
19AR112 Spot 342	66.82101	7911.713	2.095045	13.33228	2.344934	1.789957	2.732277	0.17742	1.035284	0.378909	1052.864	10.05656	1041.81	17.80099	1019.553	51.22461	1019.6	51.2	103.3	228					
19AR112 Spot 128	481.8301	25444.79	6.342904	13.53899	0.769045	1.756943	1.249311	0.173986	0.837937	0.670719	1034.034	8.005359	1029.723	8.084246	1021.436	18.75753	1021.4	18.8	101.2	88					
19AR112 Spot 241	101.6253	7623.161	3.901376	13.28326	1.67059	1.754532	2.637374	0.173707	0.758942	0.287764	1032.503	7.240767	1028.834	17.05909	1021.904	51.13979	1021.9	51.1	101.0	164					
19AR112 Spot 64	108.3527	11186.49	2.810671	13.39324	1.184859	1.83114	1.759282	0.181134	0.766466	0.435669	1073.166	7.57725	1056.688	11.55432	1023.665	32.05083	1023.7	32.1	104.8	45					
19AR112 Spot 155	233.7209	22099.95	4.832594	13.52014	0.793478	1.803565	1.183272	0.178357	0.745158	0.629744	1057.994	7.270775	1046.75	7.729371	1024.22	18.59859	1024.2	18.6	103.3	100					
19AR112 Spot 79	337.7801	25821.36	3.015589	13.48833	0.7075	1.74646	1.186697	0.17259	0.814053	0.685982	1026.365	7.723969	1025.855	7.662374	1025.627	17.46759	1025.6	17.5	100.1	55					
19AR112 Spot 218	78.2455	8505.376	2.275232	13.32125	2.173185	1.76485	3.129412	0.174368	0.911095	0.291139	1036.131	8.720551	1032.631	20.28554	1026.084	60.5535	1026.1	60.6	101.0	150					
19AR112 Spot 311	69.92543	7786.868	2.876529	13.27507	1.49859	1.717193	2.728652	0.169556	0.948193	0.347495	1009.662	8.86148	1014.976	17.51136	1027.322	51.74118	1027.3	51.7	98.3	213					
19AR112 Spot 477	196.6036	16272.67	3.441891	13.42355	0.92484	1.746901	1.178813	0.172457	0.681542	0.578159	1025.636	6.462423	1026.018	7.612167	1027.693	19.4707	1027.7	19.5	99.8	313					
19AR112 Spot 309	514.25	13613.07	4.010176	13.37914	0.642721	1.639931	1.177313	0.161496	0.942387	0.800456	965.0841	8.446785	985.686	7.426131	1032.709	14.27933	1032.7	14.3	93.5	211					
19AR112 Spot 12	336.6851	39348.41	7.541029	13.47256	0.548173	1.79945	1.065815	0.176919	0.800196	0.750784	1050.122	7.754313	1045.259	6.956416	1035.966	14.23901	1036.0	14.2	101.4	8					
19AR112 Spot 170	101.483	6532.359	3.301151	13.11291	1.490385	1.760186	3.004655	0.173018	0.678613	0.225854	1028.719	6.452492	1030.916	19.45799	1036.442	59.11711	1036.4	59.1	99.3	113					
19AR112 Spot 406	412.1624	54549.39	4.122608	13.44002	0.755474	1.787713	1.093879	0.175305	0.768245	0.702313	1041.272	7.386883	1040.992	7.122886	1041.265	15.73416	1041.3	15.7	100.0	269					
19AR112 Spot 120	440.7819	45233.7	3.102797	13.45981	0.804567	1.780437	1.129544	0.174544	0.784573	0.694593	1037.099	7.516008	1038.339	7.344362	1041.809	16.41749	1041.8	16.4	99.5	82					
19AR112 Spot 432	179.1651	18183.54	2.262917	13.30904	0.826789	1.795946	1.064087	0.175847	0.615167	0.578117	1044.245	5.930553	1043.987	6.940304	1044.305	17.53461	1044.3	17.5	100.0	282					
19AR112 Spot 341	225.9923	21700.49	6.347791	13.34441	1.027395	1.64609	1.494523	0.160798	0.893716	0.597994	961.2105	7.980718	988.0523	9.440479	1049.052	24.16684	1049.1	24.2	91.6	227					
19AR112 Spot 567	413.8684	56622.07	17.90868	13.38103	0.744726	1.812342	1.087379	0.176661	0.766272	0.704696	1048.71	7.416371	1049.924	7.115247	1053.347	15.56087	1053.3	15.6	99.6	372					
19AR112 Spot 233	298.3094	35531.08	3.989243	13.35101	0.759283	1.787711	1.131003	0.174096	0.810992	0.717055	1034.642	7.752128	1040.992	7.364631	1055.244	15.89495	1055.2	15.9	98.0	160					
19AR112 Spot 458	415.1742	44188.51	11.00868	13.31274	0.666571	1.792885	1.004418	0.17455	0.726977	0.723779	1037.134	6.964471	1042.875	6.547115	1055.82	13.97657	1055.8	14.0	98.2	299					
19AR112 Spot 435	55.62459	8697.144	2.764995	13.08349	2.153415	1.781933	3.01297	0.173288	0.663407	0.220184	1030.205	6.316313	1038.885	19.59852	1058.091	59.19581	1058.1	59.2	97.4	283					
19AR112 Spot 130	350.7664	50157.65	20.15343	13.30734	0.911287	1.838763	1.244303	0.178431	0.81481	0.654833	1058.395	7.953168	1059.419	8.183932	1062.422	18.93162	1062.4	18.9	99.6	90					
19AR112 Spot 474	323.7545	35042.66	6.960779	13.23788	0.652829	1.842008	1.103518	0.178183	0.734422	0.665528	1057.038	7.160063	1060.579	7.262448	1068.765	16.54352	1068.8	16.5	98.9	311					
19AR112 Spot 399	486.3381	62632.8	2.894747	13.26651	0.618459	1.842492	0.8881	0.178142	0.6098	0.686635	1056.819	5.943956	1060.752	5.845247	1069.746	12.95824	1069.7	13.0	98.8	265					
19AR112 Spot 501	42.5313	6199.595	1.980985	12.91027	4.290895	1.772038	4.552191	0.171188	0.942996	0.207152	1018.653	8.885361	1035.267	29.55608	1071.419	89.49551	1071.4	89.5	95.1	328					
19AR112 Spot 367	170.3231	26043.22	2.133308	13.1552	0.950932	1.906137	1.335643	0.183618	0.857695	0.642159	1086.709	8.577384	1083.236	8.895482	1077.147	20.55359	1077.1	20.6	100.9	244					
19AR112 Spot 463	344.9917	36181.05	2.171951	13.11261	0.65758	1.924853	1.022798	0.184741	0.71364	0.697733	1092.823	7.173601	1089.754	6.834705	1084.518	14.67534	1084.5	14.7	100.8	303					
19AR112 Spot 462	87.77562	11830.41	2.486515	12.88339	1.462533	1.867706	1.699394	0.178073	0.687037	0.404283	1056.441	6.694603	1069.719	11.23869	1097.778	31.10314	1097.8	31.1	96.2	302					
19AR112 Spot 268	248.7785	27253.89	2.745617	13.01503	1.009188	1.913475	1.374894	0.182272	0.805678	0.585993	1079.374	8.00723	1085.796	9.16901	1099.555	22.29708	1099.6	22.3	98.2	180					
19AR112 Spot 504	390.7164	43424.57	8.669897	12.97502	0.723103	1.899989	1.166367	0.179728	0.815877	0.699502	1065.49	8.012676	1081.085	7.759406	1113.506	16.62864	1113.5	16.6	95.7	331					
19AR112 Spot 69	250.7225	17973.95	2.803561	12.87575	0.724592	1.993218	1.534081	0.188457	1.310423	0.854207	1113.009	13.39549	1113.214	10.37313	1114.466	15.91094	1114.5	15.9	99.9	50					
19AR112 Spot 48	103.4706	9196.358	2.948391	12.72025	0.960038	2.095448	1.569457	0.197447	0.57838	0.368522	1161.591	6.147889	1147.314	10.7882	1121.315	29.10482	1121.3	29.1	103.6	35					
19AR112 Spot 32	95.81217	15728.38	1.439182	12.79064	1.924107	2.054131	2.294304	0.192992	0.837803	0.365166	1137.561	8.73698	1133.67	15.66953	1127.078	42.54205	1127.1	42.5	100.9	24					
19AR112 Spot 365	127.1903	12971.2	5.119695	12.70303	1.173207	2.131529	1.937263	0.199617	0.837621	0.432373	1173.263	8.985059	1159.081	13.38993	1133.513	34.76037	1133.5	34.8	103.5	242					
19AR112 Spot 594	168.3605	19796.19	5.115073	12.84008	0.78331	2.038801	1.038	0.190928	0.648559	0.624815	1126.398	6.702718	1128.561	7.071424	1133.571	16.12393	1133.6	16.1	99.4	387					
19AR112 Spot 166	190.3716	18183.21	2.66983	12.70998	1.206573	1.884111	1.446074	0.176128	0.684876	0.473611	1045.787	6.611566	1075.511	9.59241	1137.145	25.34061	1137.1	25.3	92.0	109					
19AR112 Spot 67	288.6243	37273.27	3.019094	12.79755	0.665521	2.105212	1.06988	0.196548	0.767856	0.717704	1156.751	8.130881	1150.512	7.365072	1139.633	14.8083	1139.6	14.8	101.5	48					
19AR112 Spot 593	359.5666	30723.52	5.252592	12.81933	0.620118	2.071413	0.994857	0.192983	0.764289	0.76824	1137.517	7.970063	1139.399	6.812795	1143.861	12.63898	1143.9	12.6	99.4	386					
19AR112 Spot 338	82.19601	14444.39	1.253773	12.58286	1.54881	2.208496	2.051725	0.204258	0.808766	0.394189	1198.154	8.843035	1183.736	14.34079	1158.328	37.38274	1158.3	37.4	103.4	224					
19AR112 Spot 528	460.0049	48286.05	6.157123	12.63985	0.58776	2.147378	0.975596	0.197736	0.761316	0.78036	1163.144	8.102283	1164.208	6.758744	1167.03	12.10219	1167.0	12.1	99.7	349					
19AR112 Spot 14	173.7366	22318.91	3.061202	12.57319	0.86435	2.139313	1.091947	0.196588	0.61043	0.55903	1156.964	6.464971	1161.602	7.555774	1171.142	17.94247	1171.1	17.9	98.8	10					
19AR112 Spot 452	366.9952	49613.83	9.512682	12.53537	0.622549	2.194403	1.108844	0.200978	0.911007	0.821582	1180.571	9.827731	1179.266	7.73456	1177.75	12.51393	1177.8	12.5	100.2	295					
19AR112 Spot 562	309.0158	36885.59	3.778043	12.57041	0.778689	2.058818	1.098169	0.188556	0.749637	0.682625	1113.546	7.666371	1135.227	7.505357	1177.796	15.88511	1177.8	15.9	94.5	369					
19AR112 Spot 511	236.2232	18227.13	2.593039	12.4215	0.794515	2.111343	1.155576	0.192353	0.813237	0.70375	1134.111	8.457279	1152.515	7.962481	1188.176	16.19336	1188.2	16.2	95.4	337					
19AR112 Spot 509	88.2734	12314.18	2.477472	12.36247	1.556542	2.306212	1.863239	0.210073	0.811051	0.435291	1229.205	9.076627	1214.198	13.19749	1188.497	33.126	1188.5	33.1	103.4	335					
19AR112 Spot 331	220.4421	26818.93	5.063789	12.46661	0.850199	2.193398	1.092372	0.19978	0.668492	0.611964	1174.137	7.17571	1178.946	7.618559	1188.661	17.04134	1188.7	17.0	98.8	221					
19AR112 Spot 223	261.5708	13654.69	3.721015	12.39839	0.8979	1.977243	1.300621	0.180074	0.842551	0.647807	1067.379	8.288134	1107.78	8.770757	1188.857	19.58272	1188.9	19.6	89.8	154					
19AR112 Spot 55	107.9752	20666.99	3.331427	12.34152	0.932081	2.337515	1.223705	0.210888	0.739791	0.60455	1233.548	8.305686	1223.766	8.702587	1207.443	19.17747	1207.4	19.2	102.2	39					
19AR112 Spot 24	87.07526	9534.905	3.192235	12.17948	0.913848	1.864023	2.076217	0.167867	1.001699	0.482463	1000.348	9.281703	1068.414	13.72157	1210.973	35.77908	1211.0	35.8	82.6	19					
19AR112 Spot 105	157.1481	29658.11	3.256181	12.26421	1.26124	1.819123	2.214133	0.16324	1.790372	0.808611	974.7572	16.19644	1052.369	14.50812	1217.989	25.61722	1218.0	25.6	80.0	73					
19AR112 Spot 283	160.2264	21245.6	3.195039	12.22759	0.98891	2.102281	1.295128	0.188125	0.734059	0.566785	1111.208	7.49261	1149.553	8.91176	1223.452	20.96417	1223.5	21.0	90.8	191					
19AR112 Spot 31	118.3314	13006.84	4.223261	12.15585	1.384373	2.34992	1.81311	0.210176	0.797473	0.439837	1229.755	8.928302	1227.533	12.91505	1224.472	32.00665	1224.5	32.0	100.4	23					
19AR112 Spot 514	176.9714	38890.65	2.192083	12.06515	0.916718	2.436709	1.273538	0.214329	0.846599	0.664762	1251.842	9.632556	1253.505	9.168846	1257.232	18.58502	1257.2	18.6	99.6	340					
19AR112 Spot 438	50.81014	7000.44	3.67622	11.43393	1.859379	2.676106	2.276041	0.227673	0.670413	0.294552	1322.292	8.014754	1321.881	16.82538	1322.038	42.1434	1322.0	42.1	100.0	286					
19AR112 Spot 115	322.5859	9908.351	5.943	11.4093	0.856363	2.750504	1.485075	0.231515	0.888918	0.598568	1342.432	10.77256	1342.225	11.05906	1342.717	22.98088	1342.7	23.0	100.0	78					
19AR112 Spot 123	71.36183	8826.131	1.611517	11.34194	1.548796	2.8681	1.89714	0.240231	0.850008	0.448047	1387.898	10.61373	1373.573	14.28415	1352.192	32.72254	1352.2	32.7	102.6	84					
19AR112 Spot 369	46.94055	7471.362	3.550898	11.15195	2.146162	3.024828	3.00867	0.250247	0.81031	0.269325	1439.747	10.45544	1413.903	22.96317	1375.996	55.72535	1376.0	55.7	104.6	246					
19AR112 Spot 481	126.1781	16112.79	8.268633	11.23067	0.917158	2.842957	1.219241	0.233967	0.758998	0.622517	1355.259	9.277075	1366.951	9.158743	1386.093	18.32147	1386.1	18.3	97.8	317					
19AR112 Spot 20	107.671	13248.96	5.63931	10.27965	0.714153	3.571457	1.129016	0.269389	0.602729	0.533853	1537.699	8.245654	1543.212	8.956371	1551.571	17.92822	1551.6	17.9	99.1	15					
19AR112 Spot 261	101.1133	24330.63	2.820651	9.6412	0.685901	4.335879	1.088181	0.305462	0.775384	0.71255	1718.339	11.69577	1700.212	8.978704	1678.727	14.10406	1678.7	14.1	102.4	174					
19AR112 Spot 103	452.7849	33606.81	3.609238	8.959685	0.622165	4.355234	0.981293	0.28488	0.753543	0.767908	1615.895	10.77027	1703.888	8.103467	1814.64	11.41472	1814.6	11.4	89.0	71					
19AR112 Spot 424	88.37847	18301.98	1.206262	8.878995	0.826077	4.45722	1.281414	0.290031	0.956226	0.746227	1641.685	13.85874	1723.044	10.62742	1824.127	15.47351	1824.1	15.5	90.0	279					
19AR112 Spot 198	106.1349	10587.83	1.52942	8.815205	1.040513	4.14751	1.350982	0.268314	0.767768	0.568304	1532.236	10.47044	1663.718	11.05317	1834.669	20.13721	1834.7	20.1	83.5	134					
19AR112 Spot 85	279.0896	54420.96	2.892191	8.793465	0.700312	5.108923	1.131437	0.327648	0.878353	0.776317	1826.972	13.97374	1837.59	9.608106	1850.411	12.89533	1850.4	12.9	98.7	59					
19AR112 Spot 583	311.5923	58671.84	3.356802	8.738778	0.874948	5.183896	1.289546	0.329624	0.941942	0.730445	1836.557	15.05334	1849.976	10.97685	1865.866	15.89535	1865.9	15.9	98.4	381					
19AR112 Spot 236	245.9898	46844.39	4.401324	8.738594	0.662287	5.127155	0.973679	0.326001	0.711351	0.73058	1818.968	11.27398	1840.616	8.273187	1865.949	12.00056	1865.9	12.0	97.5	162					
19AR112 Spot 117	328.172	17024.84	1.732766	8.659657	0.781888	4.824524	1.448344	0.305349	1.197103	0.826532	1717.779	18.05182	1789.183	12.18194	1874.257	14.70063	1874.3	14.7	91.7	80					
19AR112 Spot 344	61.14888	18696.46	0.781932	8.659761	0.719984	5.301683	1.019771	0.335504	0.645991	0.633467	1865.006	10.4616	1869.134	8.711652	1874.497	14.22796	1874.5	14.2	99.5	230					
19AR112 Spot 404	29.56165	4926.202	2.36553	8.488686	1.315951	5.329607	2.475286	0.336735	0.81283	0.328378	1870.941	13.19961	1873.623	21.16589	1877.368	42.14463	1877.4	42.1	99.7	267					
19AR112 Spot 158	131.43	31392.16	6.547433	8.522759	0.700426	5.227471	1.019305	0.324826	0.690259	0.677186	1813.255	10.90995	1857.105	8.688095	1907.332	13.47079	1907.3	13.5	95.1	102					
19AR112 Spot 49	294.554	21145.58	10.54694	8.388217	0.730233	4.650081	3.685148	0.28476	3.585993	0.973093	1615.289	51.23814	1758.308	30.80519	1933.512	15.20114	1933.5	15.2	83.5	36					
19AR112 Spot 162	404.9	98052.23	4.281988	8.392188	0.578421	5.215084	0.867034	0.318993	0.642647	0.741202	1784.81	10.01912	1855.084	7.387334	1935.565	10.41658	1935.6	10.4	92.2	105					
19AR112 Spot 364	279.9577	67459.94	0.871588	8.288563	0.716525	5.812791	1.10103	0.350845	0.82342	0.747864	1938.635	13.7864	1948.319	9.538973	1959.391	13.04525	1959.4	13.0	98.9	241					
19AR112 Spot 211	126.319	20035.61	2.955383	8.268018	0.627604	5.307763	0.987982	0.319856	0.667337	0.675454	1789.027	10.42537	1870.113	8.44161	1962.217	12.99845	1962.2	13.0	91.2	145					
19AR112 Spot 561	53.70193	14528.02	3.274787	8.159463	1.11797	6.041355	1.387029	0.360599	0.791255	0.570468	1985.014	13.51855	1981.825	12.08409	1979.259	20.28631	1979.3	20.3	100.3	368					
19AR112 Spot 93	211.5986	35754.54	1.724607	8.142638	0.754406	6.002604	1.160362	0.3566	0.87437	0.753532	1966.038	14.81644	1976.222	10.09992	1987.657	13.56998	1987.7	13.6	98.9	64					
19AR112 Spot 178	302.3028	64756.31	1.752278	8.137984	0.776458	5.419206	1.145581	0.321466	0.827346	0.722207	1796.885	12.97434	1887.896	9.820276	1990.287	14.09122	1990.3	14.1	90.3	119					
19AR112 Spot 299	127.1205	33671.08	2.786378	7.981815	0.668304	6.431146	1.026842	0.371457	0.771095	0.750938	2036.252	13.46334	2036.534	9.023552	2037.571	11.993	2037.6	12.0	99.9	202					
19AR112 Spot 186	344.469	96349.65	1.867182	7.9023	0.650183	6.489868	1.041008	0.372963	0.80617	0.774413	2043.329	14.11734	2044.526	9.159197	2046.486	11.63668	2046.5	11.6	99.8	126					
19AR112 Spot 156	86.22281	18177.1	5.48252	7.685278	0.742752	6.8652	1.07068	0.384694	0.749912	0.700408	2098.175	13.43047	2094.175	9.489547	2090.995	13.43622	2091.0	13.4	100.3	101					
19AR112 Spot 127	65.62783	19025.45	2.578009	7.67292	1.034186	6.834231	1.55923	0.382399	1.12693	0.722748	2087.481	20.09555	2090.169	13.81212	2093.567	18.93928	2093.6	18.9	99.7	87					
19AR112 Spot 11	284.1387	47358.63	3.817584	5.43795	0.685078	12.51154	1.046857	0.494909	0.790005	0.754644	2591.879	16.86007	2643.595	9.843214	2684.131	11.35632	2684.1	11.4	96.6	7					
**19AR113**																					-26.94369	-65.76479	4320	43.2	1.048
19AR113 Spot 502	1919.235	163.5402	0.684788	6.373169	26.73781	0.002935	39.06192	0.000308	4.2308	0.10831	1.988341	0.08411	2.975806	1.160706	899.7184	831.9161	2.0	0.1	0.2	440					
19AR113 Spot 326	1876.966	268.4148	0.900419	8.919844	14.36156	0.002488	21.8541	0.000313	3.946505	0.180584	2.017416	0.079605	2.522984	0.550691	517.3117	476.929	2.0	0.1	0.4	288					
19AR113 Spot 100	1222.176	498.3984	0.830408	8.759368	22.61036	0.003891	26.96892	0.00033	6.92757	0.256872	2.12537	0.147212	3.943421	1.061436	1329.652	513.1575	2.1	0.1	0.2	92					
19AR113 Spot 461	2015.107	877.2805	2.253136	6.981314	13.48221	0.006047	14.49975	0.000344	3.219442	0.222034	2.216227	0.071338	6.121295	0.884902	2065.113	250.6034	2.2	0.1	0.1	406					
19AR113 Spot 367	1280.163	440.5971	4.768241	4.531273	30.82452	0.009136	31.98095	0.000344	6.678843	0.208838	2.219967	0.148243	9.234706	2.939966	2763.338	528.234	2.2	0.1	0.1	318					
19AR113 Spot 111	1333.803	586.2938	5.827836	7.220232	25.99149	0.005583	29.35696	0.000352	5.569983	0.189733	2.266523	0.126223	5.653223	1.655004	1882.97	530.917	2.3	0.1	0.1	102					
19AR113 Spot 316	1813.825	199.5287	1.113078	7.79953	12.04207	0.002647	26.66717	0.000352	5.099843	0.19124	2.269021	0.115696	2.684271	0.714874	394.149	NA	2.3	0.1	0.6	278					
19AR113 Spot 406	998.7538	278.4494	0.602466	4.25726	75.81884	0.009366	77.8296	0.000355	6.27361	0.080607	2.289561	0.143613	9.465605	7.332938	2753.38	NA	2.3	0.1	0.1	354					
19AR113 Spot 439	2822.988	1106.94	2.693543	11.91782	13.70056	0.003472	17.16019	0.000357	2.964885	0.172777	2.299692	0.068171	3.519458	0.6029	946.0852	348.4701	2.3	0.1	0.2	385					
19AR113 Spot 578	2053.956	1342.305	5.07017	9.652974	14.88768	0.004655	17.17389	0.000363	3.902242	0.227219	2.340604	0.091319	4.715987	0.80804	1488.166	318.945	2.3	0.1	0.2	507					
19AR113 Spot 350	3031.568	638.5179	2.062292	11.88248	8.728002	0.003079	26.50391	0.000366	2.223682	0.0839	2.356957	0.052402	3.121823	0.826135	642.6768	577.0868	2.4	0.1	0.4	312					
19AR113 Spot 319	2046.768	850.2556	4.044774	7.347065	12.7624	0.006055	14.42385	0.000366	2.710615	0.187926	2.358755	0.063925	6.129423	0.881436	1956.875	254.2704	2.4	0.1	0.1	281					
19AR113 Spot 159	1525.961	644.7282	3.222469	7.820449	19.98847	0.005394	21.53313	0.000369	4.738783	0.220069	2.375711	0.112559	5.461853	1.172951	1734.688	389.7294	2.4	0.1	0.1	138					
19AR113 Spot 122	1707.767	734.7476	3.558827	10.68301	12.30364	0.003803	24.60079	0.000372	4.659928	0.189422	2.39719	0.111687	3.854036	0.946326	1046.603	494.1077	2.4	0.1	0.2	110					
19AR113 Spot 493	2121.062	327.6546	0.941463	8.093446	16.24227	0.004126	32.37702	0.000374	3.71408	0.114713	2.409924	0.08949	4.180738	1.350817	1198.552	651.0972	2.4	0.1	0.2	433					
19AR113 Spot 554	2271.448	856.2447	3.469428	11.45624	15.29935	0.003642	17.66399	0.000375	4.339468	0.245668	2.416287	0.104834	3.691212	0.650832	942.5461	353.2726	2.4	0.1	0.3	483					
19AR113 Spot 575	2132.277	797.7246	3.67066	8.764539	18.93677	0.004995	20.61139	0.000375	2.937579	0.142522	2.418798	0.071041	5.059432	1.040226	1559.029	386.8028	2.4	0.1	0.2	504					
19AR113 Spot 129	1783.662	510.4464	2.667594	7.700193	21.10797	0.005324	24.36957	0.000377	3.554512	0.145859	2.427619	0.086274	5.391563	1.310419	1670.96	452.589	2.4	0.1	0.1	116					
19AR113 Spot 146	1370.558	634.0811	5.90959	6.907675	25.932	0.006538	28.0722	0.000385	5.065331	0.180439	2.483033	0.12575	6.617357	1.851599	2002.242	500.6345	2.5	0.1	0.1	131					
19AR113 Spot 90	1971.984	773.0623	6.455266	7.663896	37.48825	0.005984	38.31522	0.000386	3.361376	0.08773	2.490945	0.083714	6.057706	2.314118	1837.587	719.7176	2.5	0.1	0.1	83					
19AR113 Spot 504	1482.986	766.125	4.353619	10.79754	17.38356	0.004378	22.49563	0.000429	4.029787	0.179136	2.76631	0.111452	4.436128	0.995758	1041.929	451.9646	2.8	0.1	0.3	442					
19AR113 Spot 166	3585.938	1554.491	4.235919	13.41862	6.783971	0.003937	10.43377	0.00044	3.300229	0.316303	2.833245	0.093483	3.989817	0.415471	773.7811	208.7303	2.8	0.1	0.4	144					
19AR113 Spot 82	3319.125	1920.197	5.038402	12.16004	15.8771	0.004724	16.82948	0.000461	2.52972	0.150315	2.968858	0.075086	4.785657	0.803506	1052.725	337.4356	3.0	0.1	0.3	75					
19AR113 Spot 372	2471.003	357.3557	0.733236	10.12568	11.72775	0.003992	26.65765	0.000502	3.826716	0.14355	3.237153	0.123846	4.045738	1.076353	517.3133	NA	3.2	0.1	0.6	323					
19AR113 Spot 58	2529.411	1811.988	3.489121	12.75893	13.45069	0.005223	17.46734	0.00054	1.69432	0.096999	3.478308	0.058918	5.289598	0.92155	934.6999	359.1759	3.5	0.1	0.4	52					
19AR113 Spot 290	599.2718	451.4838	5.927667	6.520237	22.97017	0.011639	30.98437	0.000686	7.923781	0.255735	4.419122	0.350042	11.75027	3.619777	2002.421	545.1841	4.4	0.4	0.2	253					
19AR113 Spot 584	1403.542	1232.03	3.309995	9.254332	8.308896	0.009765	11.69776	0.000735	1.911778	0.163431	4.734413	0.090478	9.867463	1.148682	1556.388	217.2995	4.7	0.1	0.3	513					

19AR113 Spot 545	430.6129	394.9087	2.641481	5.206349	25.73625	0.016303	33.85751	0.000744	9.951122	0.293912	4.794446	0.476924	16.41989	5.514711	2444.906	564.4368	4.8	0.5	0.2	480
19AR113 Spot 208	1040.854	1879.604	2.925649	12.31638	11.85343	0.009517	13.24103	0.000937	4.462619	0.33703	6.037894	0.269322	9.617414	1.267434	1032.95	252.8468	6.0	0.3	0.6	184
19AR113 Spot 344	1644.032	2210.988	2.93418	12.58849	9.681595	0.011507	13.14828	0.001149	1.569604	0.119377	7.403762	0.116143	11.6173	1.518772	1004.331	266.0147	7.4	0.1	0.7	306
19AR113 Spot 223	664.8616	1362.624	4.762629	9.414619	21.23137	0.015225	22.49279	0.001152	3.52551	0.15674	7.423251	0.261557	15.3426	3.425049	1545.352	422.8213	7.4	0.3	0.5	199
19AR113 Spot 80	1425.085	1986.798	2.90515	11.87613	10.19682	0.013151	11.31196	0.001245	2.984455	0.263832	8.021197	0.23924	13.26599	1.490884	1111.777	218.5226	8.0	0.2	0.7	73
19AR113 Spot 314	258.1492	438.7994	1.021275	5.83879	34.04469	0.027515	36.27115	0.001417	9.682293	0.266942	9.127229	0.8831	27.5611	9.862582	2238.373	625.9305	9.1	0.9	0.4	276
19AR113 Spot 12	442.2562	834.4241	0.722901	9.005796	18.25423	0.019171	20.71597	0.001479	5.321493	0.256879	9.529132	0.506717	19.28172	3.956728	1508.749	381.9762	9.5	0.5	0.6	12
19AR113 Spot 54	479.2669	334.1123	1.403008	9.208978	21.264	0.016478	38.56822	0.001835	4.642265	0.120365	11.8212	0.548268	16.59494	6.348437	778.8313	834.7288	11.8	0.5	1.5	48
19AR113 Spot 86	834.4136	2419.182	0.800919	13.49911	13.49257	0.017771	14.20188	0.001902	2.00387	0.141099	12.2504	0.245249	17.8858	2.517885	862.0544	293.0613	12.3	0.2	1.4	79
19AR113 Spot 299	240.4819	621.8576	1.499545	5.033194	30.83552	0.046848	32.9552	0.001908	4.350974	0.132027	12.28676	0.534085	46.48839	14.97601	2635.954	559.7172	12.3	0.5	0.5	262
19AR113 Spot 534	779.8766	2302.34	0.985908	13.94459	13.79646	0.01906	14.17655	0.002128	1.569592	0.110718	13.70097	0.214821	19.17121	2.692326	774.3434	297.7927	13.7	0.2	1.8	470
19AR113 Spot 495	463.2279	1326.244	4.145856	10.80187	12.57733	0.024509	13.44899	0.002175	1.624193	0.120767	14.00656	0.227246	24.5859	3.266856	1239.729	262.8328	14.0	0.2	1.1	435
19AR113 Spot 471	979.4519	2589.468	4.486747	12.88871	8.61611	0.022344	11.48338	0.002258	6.364571	0.554242	14.53812	0.924246	22.43785	2.548369	980.4561	195.1341	14.5	0.9	1.5	415
19AR113 Spot 81	338.1609	940.7201	3.206083	11.61457	12.08845	0.02468	17.05432	0.002541	3.175686	0.18621	16.35734	0.518799	24.7557	4.17089	942.4994	345.6084	16.4	0.5	1.7	74
19AR113 Spot 65	390.0812	1300.178	1.672279	11.90218	12.95799	0.026667	17.8536	0.002665	3.772019	0.211275	17.15467	0.646217	26.72291	4.708799	1003.212	356.8191	17.2	0.6	1.7	58
19AR113 Spot 294	394.9411	1212.898	1.34991	11.23493	12.58238	0.030441	15.38913	0.002869	2.133151	0.138614	18.46903	0.393409	30.44783	4.616131	1120.764	305.6689	18.5	0.4	1.6	257
19AR113 Spot 50	133.9684	842.7711	2.259167	8.645023	9.317006	0.076943	15.08288	0.005642	3.274058	0.217071	36.26746	1.184084	75.26695	10.94229	1604.498	276.0868	36.3	1.2	2.3	45
19AR113 Spot 99	357.2048	2002.982	0.531293	14.23851	9.814932	0.049137	11.86585	0.005687	1.345823	0.11342	36.55935	0.490632	48.70624	5.643043	697.6679	251.9393	36.6	0.5	5.2	91
19AR113 Spot 220	1640.123	17377	7.288396	16.97926	1.686153	0.059316	2.78728	0.007421	1.968787	0.706347	47.65911	0.934846	58.51028	1.584748	529.7868	43.24389	47.7	0.9	9.0	196
19AR113 Spot 77	1661.258	15730.39	3.608918	13.87728	1.262811	0.115382	2.735793	0.011835	2.284169	0.834921	75.84553	1.722289	110.8767	2.873615	949.8722	30.79608	75.8	1.7	8.0	70
19AR113 Spot 552	159.1052	6413.581	0.98705	17.22161	4.127364	0.28831	4.467724	0.037519	0.718486	0.160817	237.4383	1.674926	257.228	10.15242	442.6431	98.1294	237.4	1.7	53.6	481
19AR113 Spot 544	47.76058	711.8251	1.306415	12.27764	8.504133	0.323471	12.24795	0.038657	1.771232	0.144615	244.5005	4.249573	284.5688	30.40491	629.1468	261.9915	244.5	4.2	38.9	479
19AR113 Spot 486	138.3661	6816.956	0.806062	16.42229	4.973101	0.323367	5.380453	0.040062	0.749619	0.139323	253.2145	1.861348	284.4895	13.35026	551.048	116.3895	253.2	1.9	46.0	426
19AR113 Spot 481	284.545	12582.4	2.500897	17.30372	2.489994	0.322346	3.615845	0.041423	2.29104	0.633611	261.6446	5.874364	283.7058	8.950112	470.692	61.92669	261.6	5.9	55.6	422
19AR113 Spot 598	120.9473	8991.081	1.940482	16.4676	3.650234	0.336942	3.824808	0.041587	0.709068	0.185387	262.6644	1.825036	294.8523	9.788047	559.2449	81.98184	262.7	1.8	47.0	525
19AR113 Spot 242	84.49449	4592.783	0.930558	15.40892	5.73972	0.352615	6.672829	0.04163	0.972766	0.14578	262.9301	2.506235	306.6864	17.66488	655.2887	141.7585	262.9	2.5	40.1	217
19AR113 Spot 324	209.9294	9749.011	1.465753	19.20528	2.077565	0.310466	2.36504	0.044623	0.697774	0.295037	281.427	1.921476	274.5419	5.689326	217.2593	52.31809	281.4	1.9	129.5	286
19AR113 Spot 189	169.3302	7098.456	1.339119	19.29671	2.810728	0.319973	3.266686	0.046634	0.867911	0.265686	293.8204	2.492858	281.8819	8.040716	184.9666	73.35797	293.8	2.5	158.9	167
19AR113 Spot 458	235.2377	14894.94	4.162539	18.26057	2.533762	0.36515	2.801617	0.049464	0.646586	0.23079	311.2294	1.964561	316.0523	7.609174	352.772	61.57654	311.2	2.0	88.2	403
19AR113 Spot 432	265.0304	21118.26	4.493064	17.08853	2.486995	0.42514	3.211297	0.053445	2.01284	0.6268	335.6365	6.582966	359.7199	9.727433	519.0662	54.93364	335.6	6.6	64.7	378
19AR113 Spot 41	184.4322	10537.14	1.398496	18.02151	3.080445	0.400848	3.249291	0.053764	0.723155	0.222558	337.5877	2.378463	342.2628	9.441031	375.0891	71.30524	337.6	2.4	90.0	37
19AR113 Spot 361	316.5012	22482.79	1.832166	18.74153	1.644493	0.389881	1.931565	0.053956	0.844053	0.436979	338.7661	2.785532	334.2825	5.501714	304.1879	39.5934	338.8	2.8	111.4	313
19AR113 Spot 389	258.0511	1575.855	2.692251	16.08439	2.063976	0.489531	3.761832	0.067469	1.551848	0.412524	420.8916	6.322937	404.5907	12.55399	313.5572	77.9666	420.9	6.3	134.2	340
19AR113 Spot 463	202.758	16434.36	4.382069	17.56444	1.87385	0.528698	2.414441	0.068679	0.877303	0.363357	428.191	3.634491	430.9454	8.478961	446.6437	50.00644	428.2	3.6	95.9	408
19AR113 Spot 179	284.8307	20988.07	13.48134	16.96809	1.488277	0.556628	1.781511	0.069433	0.895606	0.502723	432.7361	3.748394	449.3295	6.468516	536.1448	33.72788	432.7	3.7	80.7	157
19AR113 Spot 345	253.8978	1352.377	7.545814	15.45319	1.228747	0.551183	2.185847	0.074505	0.811743	0.371363	463.2419	3.628404	445.771	7.886622	357.5412	45.81844	463.2	3.6	129.6	307
19AR113 Spot 175	228.0058	19335.21	7.077641	17.51896	1.668853	0.586175	1.995657	0.075701	0.868426	0.435158	470.4139	3.939702	468.4219	7.488585	459.6442	39.86788	470.4	3.9	102.3	153
19AR113 Spot 98	156.5014	17682.52	1.57868	17.22042	2.464081	0.59494	2.738159	0.075775	0.763675	0.278901	470.855	3.467623	474.0174	10.37128	490.3103	58.03008	470.9	3.5	96.0	90
19AR113 Spot 186	228.1705	20413.91	4.034277	17.53337	1.718451	0.588506	1.977108	0.075784	0.765769	0.387318	470.9096	3.47752	469.9134	7.437557	466.0121	40.39285	470.9	3.5	101.1	164
19AR113 Spot 190	253.3093	17595.37	8.970076	17.46639	1.595709	0.590552	1.962357	0.07589	0.807923	0.41171	471.5442	3.673712	471.2201	7.398196	470.626	39.58741	471.5	3.7	100.2	168
19AR113 Spot 36	212.0421	17674.94	12.90142	17.87872	1.361046	0.578999	1.910776	0.076084	0.695994	0.364247	472.7087	3.172295	463.8181	7.114476	420.9833	39.72824	472.7	3.2	112.3	33
19AR113 Spot 184	189.9948	18118.11	3.353847	17.20844	2.35778	0.601261	2.531616	0.076092	0.8524	0.336702	472.7564	3.885557	478.0338	9.652545	504.4124	52.46105	472.8	3.9	93.7	162
19AR113 Spot 88	131.1783	6404.338	1.673001	17.42275	2.446886	0.574927	4.832798	0.076107	0.694537	0.143713	472.8423	3.166514	461.1958	17.91536	404.5485	107.1389	472.8	3.2	116.9	81
19AR113 Spot 408	304.9256	31717.23	2.579382	17.40423	1.395076	0.597488	1.733143	0.076107	0.671351	0.38736	472.8452	3.060823	475.638	6.582059	490.0885	35.22921	472.8	3.1	96.5	356
19AR113 Spot 308	79.75191	7133.91	1.850121	14.98788	4.292655	0.676058	4.524409	0.076194	0.797573	0.176282	473.3627	3.640131	524.3893	18.53254	754.13	94.06162	473.4	3.6	62.8	271
19AR113 Spot 32	99.96944	8784.276	2.341985	17.14689	2.835434	0.597376	3.040966	0.076206	0.769404	0.253013	473.4364	3.512091	475.5669	11.54784	486.8092	64.96688	473.4	3.5	97.3	31
19AR113 Spot 213	76.8645	8150.946	2.117098	17.00513	3.072493	0.599068	3.644502	0.07622	0.882603	0.242174	473.5203	4.029501	476.6424	13.86451	492.6371	77.98719	473.5	4.0	96.1	189
19AR113 Spot 24	206.5721	14826.86	7.595499	17.37204	1.392415	0.597105	1.622388	0.076281	0.714714	0.440532	473.8834	3.265421	475.3946	6.158954	483.6756	32.16507	473.9	3.3	98.0	24
19AR113 Spot 150	56.24757	5316.955	1.330774	15.02574	5.482434	0.667513	5.803866	0.076295	1.030769	0.1776	473.9676	4.71023	519.1991	23.59478	724.4123	121.2436	474.0	4.7	65.4	135
19AR113 Spot 66	156.1619	15197.84	1.666516	17.41504	2.552602	0.591606	2.82806	0.076449	0.676614	0.23925	474.8893	3.097668	471.893	10.67411	458.3237	60.91482	474.9	3.1	103.6	59
19AR113 Spot 531	283.3416	21451.42	9.537746	17.31392	1.270244	0.597915	1.518671	0.076501	0.603301	0.397256	475.2011	2.763776	475.9095	5.770109	480.2656	30.79619	475.2	2.8	98.9	468
19AR113 Spot 313	171.5275	10021.13	2.186697	17.27307	1.988137	0.592244	2.524115	0.076516	0.644837	0.255471	475.2934	2.954608	472.2999	9.533296	458.7446	54.15087	475.3	3.0	103.6	275
19AR113 Spot 94	186.7176	14237.32	18.98624	17.49842	2.338211	0.58944	2.530658	0.076545	0.756149	0.298796	475.4672	3.465856	470.5101	9.529536	447.3486	53.68875	475.5	3.5	106.3	86
19AR113 Spot 178	214.168	20166.77	2.59571	17.22096	1.490363	0.604196	1.986664	0.076583	0.864193	0.434997	475.6944	3.962907	479.8929	7.597709	500.9835	39.40702	475.7	4.0	95.0	156
19AR113 Spot 289	152.6222	12931.35	5.073268	17.19256	2.314122	0.599027	2.51787	0.076601	0.714766	0.283877	475.801	3.27839	476.6163	9.577823	481.4824	53.34554	475.8	3.3	98.8	252
19AR113 Spot 176	135.0319	14915.39	3.323542	17.48939	2.727565	0.592208	2.936552	0.076653	0.914509	0.311423	476.1131	4.197194	472.2765	11.09071	454.6425	61.9431	476.1	4.2	104.7	154
19AR113 Spot 519	90.14866	6836.402	2.358848	16.81931	3.52332	0.604657	4.236635	0.076666	1.234331	0.291347	476.1888	5.665905	480.1849	16.21119	500.2923	89.28713	476.2	5.7	95.2	456
19AR113 Spot 304	238.323	20525.05	1.514573	17.03098	1.448822	0.609848	1.697484	0.076698	0.750722	0.442256	476.3813	3.447353	483.4641	6.529474	518.1225	33.42393	476.4	3.4	91.9	267
19AR113 Spot 379	95.2919	7675.602	1.8936	16.40252	4.045395	0.62121	4.682111	0.076699	0.865986	0.184956	476.3895	3.976716	490.6052	18.21869	558.4797	100.3623	476.4	4.0	85.3	330
19AR113 Spot 287	399.2695	26495.63	7.005098	17.41595	0.817133	0.597241	1.356759	0.076705	0.651001	0.47982	476.4256	2.989695	475.4816	5.151285	471.8786	26.34127	476.4	3.0	101.0	251
19AR113 Spot 399	340.5452	26419.97	8.325294	17.45228	1.311359	0.597858	1.579614	0.076722	0.734711	0.465121	476.5276	3.374829	475.8734	6.001305	473.7014	30.94812	476.5	3.4	100.6	348
19AR113 Spot 272	261.6224	19340.19	11.30958	17.33096	1.418489	0.599835	1.61535	0.076762	0.716163	0.443349	476.7666	3.291221	477.1289	6.149763	479.8122	32.00724	476.8	3.3	99.4	237
19AR113 Spot 440	165.6182	12001.45	0.934185	17.10942	2.322084	0.603001	2.829497	0.076816	1.382961	0.488766	477.0908	6.359743	479.1362	10.80786	489.904	54.46905	477.1	6.4	97.4	386
19AR113 Spot 465	171.2529	14014.71	3.116618	17.6647	2.005953	0.58717	2.362179	0.076928	0.683921	0.28953	477.7624	3.14937	469.059	8.873486	427.6388	50.42137	477.8	3.1	111.7	410
19AR113 Spot 492	70.97696	5808.413	5.19928	16.15947	3.192249	0.627778	4.165148	0.076955	0.947549	0.227495	477.9212	4.364743	494.7106	16.31205	574.1338	88.21745	477.9	4.4	83.2	432
19AR113 Spot 44	105.3413	14408.45	0.814486	17.51778	3.308674	0.593581	3.513263	0.076966	0.73973	0.210554	477.9891	3.407924	473.1517	13.28835	450.7066	76.31818	478.0	3.4	106.1	39
19AR113 Spot 511	162.2951	14473.39	17.55547	17.7217	1.910674	0.588787	2.302976	0.077036	0.656098	0.284892	478.4056	3.025171	470.0927	8.666074	430.6552	49.20339	478.4	3.0	111.1	448
19AR113 Spot 131	74.41502	4658.689	3.667092	17.30623	4.863886	0.578368	5.979907	0.077077	0.863186	0.144348	478.6546	3.982017	463.4119	22.25306	389.5145	132.9134	478.7	4.0	122.9	118
19AR113 Spot 370	312.9651	25406.52	11.37096	17.37498	1.558645	0.602573	1.726767	0.0771	0.685681	0.397089	478.7868	3.163996	478.8655	6.592678	480.1836	35.01939	478.8	3.2	99.7	321
19AR113 Spot 517	141.4207	14927.32	2.422545	17.64923	2.186932	0.590786	2.437641	0.077106	0.947234	0.388586	478.8238	4.371229	471.3696	9.192423	436.1879	50.02633	478.8	4.4	109.8	454
19AR113 Spot 505	195.9371	19005.51	15.0956	17.59048	1.343077	0.596563	1.913847	0.077133	0.782594	0.408912	478.9844	3.61263	475.0504	7.261308	457.0416	38.75171	479.0	3.6	104.8	443
19AR113 Spot 203	118.6002	7578.94	2.244022	17.66896	2.928106	0.582059	3.291086	0.07714	0.722626	0.219571	479.0286	3.336097	465.784	12.29518	401.9639	71.92411	479.0	3.3	119.2	180
19AR113 Spot 134	95.07718	8595.456	1.618739	16.70029	2.655725	0.616502	3.839176	0.077165	0.63585	0.165621	479.1781	2.936368	487.6523	14.86816	528.6443	82.99517	479.2	2.9	90.6	121
19AR113 Spot 243	209.8612	14596.58	9.715757	17.44858	2.169547	0.596231	2.537808	0.077182	0.573362	0.225928	479.2787	2.648332	474.8389	9.625434	454.4044	54.8764	479.3	2.6	105.5	218
19AR113 Spot 424	284.6435	27233.48	15.50788	17.68729	1.35105	0.596583	1.505778	0.077239	0.61631	0.409296	479.6209	2.848664	475.063	5.71314	454.0998	30.5165	479.6	2.8	105.6	371
19AR113 Spot 249	98.21214	11334	2.047677	18.05992	2.586029	0.574084	2.738697	0.077272	0.710378	0.259385	479.819	3.284768	460.6525	10.14229	367.1245	59.61627	479.8	3.3	130.7	224
19AR113 Spot 193	147.3763	13901.65	8.088242	17.11524	2.343168	0.611512	2.665893	0.077274	0.70489	0.26441	479.8315	3.259473	484.5132	10.27209	507.6999	56.55109	479.8	3.3	94.5	171
19AR113 Spot 401	218.6167	18263.25	2.291646	17.52934	1.640243	0.597321	1.862109	0.077286	0.767618	0.41223	479.9037	3.550045	475.5319	7.070619	455.4642	37.65839	479.9	3.6	105.4	350
19AR113 Spot 85	97.98009	9533.405	1.777011	17.43984	3.968242	0.591891	4.296232	0.077337	0.806604	0.187747	480.2065	3.732615	472.0748	16.22121	433.6868	94.03203	480.2	3.7	110.7	78
19AR113 Spot 292	219.5292	16613.99	5.59935	17.36982	2.243638	0.601337	2.818766	0.077364	0.728077	0.258296	480.3674	3.370313	478.0819	10.74832	468.0829	60.3277	480.4	3.4	102.6	255
19AR113 Spot 515	330.1744	26917.75	2.404508	17.55162	1.580337	0.601226	1.847903	0.077425	0.898534	0.486245	480.7357	4.162441	478.0116	7.04533	465.91	35.77896	480.7	4.2	103.2	452
19AR113 Spot 573	110.4273	9380.126	2.813401	16.48834	4.176489	0.630221	4.545969	0.077435	0.634912	0.139665	480.7927	2.941551	496.2333	17.84627	569.0833	98.03358	480.8	2.9	84.5	502
19AR113 Spot 197	237.676	28331.08	6.295648	17.42972	1.891745	0.607918	2.031871	0.077467	0.719832	0.354271	480.9887	3.336298	482.2461	7.800377	489.1956	41.94442	481.0	3.3	98.3	174
19AR113 Spot 425	141.8081	11811.78	1.467675	17.79545	1.869817	0.587344	2.088531	0.077486	0.674608	0.323006	481.0987	3.12738	469.1698	7.846948	412.1745	44.2169	481.1	3.1	116.7	372
19AR113 Spot 574	98.65992	9298.962	1.531047	17.25648	3.612562	0.601594	3.906102	0.077535	0.820211	0.209982	481.393	3.804612	478.2447	14.89895	464.1495	84.64733	481.4	3.8	103.7	503
19AR113 Spot 95	276.5258	35077.54	11.21259	17.45364	1.536677	0.604923	1.748488	0.077546	0.772442	0.441777	481.4564	3.58349	480.3528	6.691823	476.0566	34.69437	481.5	3.6	101.1	87
19AR113 Spot 338	286.3496	37491.25	11.75625	17.83284	1.895825	0.594652	2.089842	0.077564	0.730866	0.349723	481.5634	3.391334	473.8338	7.913141	437.5259	43.58608	481.6	3.4	110.1	300
19AR113 Spot 366	175.3833	19203.58	2.160124	17.6974	1.588022	0.593495	1.850644	0.077603	0.755884	0.408444	481.8017	3.509095	473.0973	6.998842	432.0481	37.64098	481.8	3.5	111.5	317
19AR113 Spot 149	205.9323	26779.14	2.04894	17.77187	2.236387	0.593057	2.346198	0.077623	0.655996	0.2796	481.9204	3.046103	472.8182	8.868921	429.832	50.21605	481.9	3.0	112.1	134
19AR113 Spot 217	146.7289	15718.63	3.197913	17.38392	2.469323	0.605582	2.702554	0.077625	0.783635	0.289961	481.9286	3.638849	480.7701	10.35047	476.215	57.20982	481.9	3.6	101.2	193
19AR113 Spot 383	294.1726	26161.97	14.38799	17.38955	1.550876	0.606738	1.901031	0.077677	0.790234	0.415687	482.2416	3.671785	481.5007	7.289248	478.9157	38.21549	482.2	3.7	100.7	334
19AR113 Spot 529	174.0665	24910.53	5.535104	17.45969	1.450114	0.603146	1.80473	0.077699	0.700987	0.388417	482.3714	3.257949	479.2285	6.894429	465.1613	36.85366	482.4	3.3	103.7	466
19AR113 Spot 104	123.2669	13775.58	2.526042	17.51581	2.93165	0.599527	3.249979	0.077721	0.770161	0.236974	482.505	3.580401	476.9337	12.36942	451.174	70.13194	482.5	3.6	106.9	96
19AR113 Spot 539	138.3688	10292.42	4.888163	17.31636	2.261078	0.59969	3.238523	0.077727	0.875975	0.270486	482.5397	4.072603	477.0371	12.32791	451.6113	69.24778	482.5	4.1	106.8	475
19AR113 Spot 311	173.8977	14552.1	5.916831	17.20763	1.83033	0.608863	2.210726	0.077736	0.54178	0.245069	482.5955	2.519137	482.843	8.495241	485.0007	47.34573	482.6	2.5	99.5	273
19AR113 Spot 483	129.537	10269.94	6.114893	17.41752	1.776027	0.598046	2.617006	0.077741	0.761739	0.291073	482.6248	3.542092	475.9931	9.944751	445.107	55.68699	482.6	3.5	108.4	424
19AR113 Spot 349	604.4442	77727.96	3.961324	17.56205	1.160409	0.605153	1.382921	0.07777	0.744702	0.538499	482.7989	3.464074	480.4984	5.293951	470.521	25.8114	482.8	3.5	102.6	311
19AR113 Spot 83	283.6453	29054.93	3.485767	17.82413	1.288617	0.593357	1.57771	0.077819	0.746661	0.473256	483.092	3.475216	473.0093	5.96575	425.3406	31.01588	483.1	3.5	113.6	76
19AR113 Spot 485	139.6532	16776.45	7.864813	17.62529	2.121396	0.59747	2.425825	0.077852	0.704206	0.290296	483.2914	3.278922	475.6269	9.212651	439.7753	51.68087	483.3	3.3	109.9	425
19AR113 Spot 521	259.3934	27610.14	5.580225	17.37248	1.409317	0.611055	1.674941	0.077876	0.849535	0.507203	483.4318	3.956705	484.2253	6.450677	488.9479	31.8494	483.4	4.0	98.9	458
19AR113 Spot 417	102.3378	7442.299	13.88631	16.13186	2.142842	0.645325	2.691083	0.077894	0.900071	0.334464	483.5427	4.193005	505.5979	10.71766	607.5824	54.83527	483.5	4.2	79.6	364
19AR113 Spot 588	232.42	23274.17	7.658847	17.47738	1.257223	0.605552	1.578657	0.077942	0.607705	0.384951	483.8267	2.832612	480.7507	6.045742	467.0916	32.28935	483.8	2.8	103.6	516
19AR113 Spot 330	129.9444	18576.82	1.83357	17.52979	2.389762	0.604697	2.733794	0.077951	0.693597	0.253712	483.8836	3.233335	480.2099	10.46058	463.6927	58.60514	483.9	3.2	104.4	292
19AR113 Spot 248	345.0277	28670.07	8.452678	17.39074	1.321612	0.610022	1.542853	0.077973	0.661757	0.428918	484.0144	3.085708	483.5741	5.935717	482.4705	30.7937	484.0	3.1	100.3	223
19AR113 Spot 378	149.5104	14193.54	1.810303	17.34997	2.26552	0.605462	2.589072	0.077976	0.990441	0.382547	484.0312	4.618484	480.694	9.914588	465.7753	52.99536	484.0	4.6	103.9	329
19AR113 Spot 448	132.2952	15390.8	1.773118	17.25278	2.703201	0.609999	2.927611	0.078037	0.650757	0.222283	484.3931	3.036703	483.5594	11.26327	480.5496	63.08918	484.4	3.0	100.8	394
19AR113 Spot 565	166.6675	14432.51	5.8405	17.20367	2.321085	0.613751	2.534134	0.078042	0.814533	0.321425	484.4276	3.80121	485.9232	9.78653	493.9398	52.90496	484.4	3.8	98.1	494
19AR113 Spot 391	72.98458	7318.108	2.546974	15.95759	5.297838	0.649966	5.510716	0.078079	0.849726	0.154195	484.6489	3.967189	508.4577	22.04559	617.937	117.6226	484.6	4.0	78.4	341
19AR113 Spot 266	267.7616	23024.52	20.39293	17.55412	1.316205	0.605346	1.722354	0.078112	0.871584	0.506042	484.8421	4.070806	480.6207	6.594677	461.476	32.93236	484.8	4.1	105.1	232
19AR113 Spot 17	155.4052	13938.79	3.891293	17.34089	1.713285	0.608243	1.910965	0.078124	0.617	0.322874	484.9144	2.882162	482.4513	7.338639	471.725	40.03429	484.9	2.9	102.8	17
19AR113 Spot 450	67.6082	5916.455	2.142396	17.21295	3.173406	0.596566	3.910402	0.078168	0.843474	0.2157	485.1778	3.942139	475.0523	14.83727	427.3958	85.18025	485.2	3.9	113.5	396
19AR113 Spot 467	229.2746	22485.02	8.898236	17.37061	1.639037	0.611193	1.908709	0.078175	0.707184	0.370504	485.2214	3.30545	484.3125	7.352047	480.9549	39.17168	485.2	3.3	100.9	412
19AR113 Spot 364	164.947	14099.93	2.351773	17.31878	2.35965	0.608203	2.611226	0.078177	0.865478	0.331445	485.234	4.045428	482.426	10.02758	470.0472	54.5387	485.2	4.0	103.2	316
19AR113 Spot 93	112.4362	10829.07	4.830528	16.92775	3.287755	0.618874	3.850941	0.078203	0.802133	0.208295	485.3901	3.750501	489.141	14.94917	507.7258	82.86771	485.4	3.8	95.6	85
19AR113 Spot 62	185.6884	25474.9	7.972946	17.55825	1.940405	0.604506	2.05269	0.078208	0.606542	0.295486	485.4149	2.836124	480.0894	7.85274	455.6786	43.53959	485.4	2.8	106.5	56
19AR113 Spot 444	216.9117	12949.01	2.50292	17.46088	1.9684	0.602677	2.295152	0.078295	0.649956	0.283187	485.937	3.042273	478.9312	8.763766	446.4709	48.93928	485.9	3.0	108.8	390
19AR113 Spot 76	116.9794	13530.92	1.585965	16.98829	2.553532	0.62101	2.66197	0.078423	0.659442	0.247727	486.7046	3.091366	490.4803	10.35526	509.1152	56.73307	486.7	3.1	95.6	69
19AR113 Spot 466	273.0564	27188.36	10.14071	17.17038	1.497902	0.621647	1.66949	0.078432	0.683078	0.409154	486.7588	3.202514	490.879	6.498409	511.0871	33.49708	486.8	3.2	95.2	411
19AR113 Spot 267	155.7374	13339.73	3.561949	17.68726	1.942393	0.598304	2.460403	0.078441	0.705584	0.286776	486.8086	3.308354	476.157	9.352139	426.1144	52.57822	486.8	3.3	114.2	233
19AR113 Spot 373	149.5341	21440.83	2.515762	17.32041	1.91122	0.614466	2.274732	0.078549	0.726693	0.319463	487.4576	3.411703	486.3729	8.791034	482.2482	47.63848	487.5	3.4	101.1	324
19AR113 Spot 132	203.99	19421.32	4.972168	17.41581	1.33288	0.611512	1.888875	0.078593	0.731834	0.387444	487.721	3.437628	484.5132	7.277997	470.3095	38.56276	487.7	3.4	103.7	119
19AR113 Spot 445	145.8321	13130.17	2.945938	17.71136	2.217214	0.598214	2.614671	0.078832	0.687002	0.262749	489.147	3.236121	476.0998	9.937625	414.6692	56.39836	489.1	3.2	118.0	391
19AR113 Spot 228	88.84183	11582.12	2.50689	16.6051	3.761318	0.639801	4.259964	0.078888	0.981476	0.230395	489.4818	4.626285	502.183	16.87834	561.4306	90.36931	489.5	4.6	87.2	203
19AR113 Spot 251	99.62278	9833.989	3.144666	18.34067	3.050799	0.575565	4.041798	0.078912	0.735329	0.181931	489.6238	3.467015	461.6075	14.9932	325.4799	90.25161	489.6	3.5	150.4	226
19AR113 Spot 123	242.5597	21816.82	3.817355	17.16354	1.687986	0.628029	1.88358	0.079172	0.746368	0.39625	491.1785	3.529818	494.867	7.378005	512.9056	38.01115	491.2	3.5	95.8	111
19AR113 Spot 64	239.2971	28565.74	5.189344	17.46735	1.752296	0.615977	1.904253	0.07921	0.717374	0.376722	491.4075	3.394217	487.323	7.370411	469.141	39.07567	491.4	3.4	104.7	57
19AR113 Spot 430	117.6469	17831.67	2.06898	17.39291	2.492716	0.617835	2.840615	0.079247	0.683051	0.240459	491.6238	3.23319	488.4895	11.01533	474.7815	61.01341	491.6	3.2	103.5	376
19AR113 Spot 524	69.12933	9716.416	2.809552	16.3981	4.366455	0.647033	4.664915	0.079274	1.045257	0.224068	491.7863	4.949248	506.6514	18.61001	575.2587	98.89743	491.8	4.9	85.5	461
19AR113 Spot 286	59.4391	6551.912	1.829434	15.91884	4.628709	0.661221	5.596762	0.079682	1.162907	0.207782	494.225	5.532584	515.3609	22.62341	611.1364	118.4093	494.2	5.5	80.9	250
19AR113 Spot 139	109.5065	10762.61	2.818862	17.07	2.574417	0.627368	2.815213	0.079874	0.898225	0.319061	495.3688	4.282862	494.4552	11.02034	491.1649	58.8553	495.4	4.3	100.9	125
19AR113 Spot 252	84.3531	8801.798	3.175212	16.65356	4.111451	0.644796	4.899862	0.080157	0.725331	0.148031	497.061	3.469844	505.2715	19.50642	543.5889	105.9848	497.1	3.5	91.4	227
19AR113 Spot 52	543.5786	56467.93	14.67868	17.2376	0.956669	0.637951	1.189376	0.080331	0.652916	0.548956	498.0999	3.129704	501.0366	4.703689	515.4059	21.82171	498.1	3.1	96.6	47
19AR113 Spot 412	44.83806	5372.141	80.98211	14.85093	6.889231	0.717934	8.05838	0.080564	1.183286	0.146839	499.4912	5.68723	549.4463	34.20738	763.2379	168.2484	499.5	5.7	65.4	359
19AR113 Spot 307	281.1011	2968.593	106.5004	16.26688	1.180401	0.627347	1.668609	0.080906	0.760755	0.455922	501.5301	3.670772	494.4417	6.531578	462.7323	32.93732	501.5	3.7	108.4	270
19AR113 Spot 162	323.0768	36240.1	16.21501	17.08153	1.216382	0.650252	1.456071	0.081483	0.719286	0.493991	504.9683	3.493552	508.6339	5.825708	526.1241	27.7695	505.0	3.5	96.0	141
19AR113 Spot 579	254.0869	20349.81	43.15072	17.17056	1.509438	0.648294	1.825081	0.081977	0.870735	0.477094	507.9097	4.252808	507.4281	7.288811	506.2162	35.28555	507.9	4.3	100.3	508
19AR113 Spot 580	262.4959	24546.14	11.57762	16.94403	1.27044	0.659769	1.52297	0.082141	0.710376	0.466442	508.8868	3.476008	514.4725	6.147104	540.3364	29.47218	508.9	3.5	94.2	509
19AR113 Spot 404	202.2011	25041.93	459.0957	17.69489	1.566757	0.632893	1.860083	0.082194	0.699506	0.376062	509.2076	3.424889	497.8962	7.320526	447.1694	38.30421	509.2	3.4	113.9	353
19AR113 Spot 135	127.5705	9866.286	8.941185	16.68903	2.605822	0.661131	3.051789	0.082385	0.829136	0.271689	510.3418	4.068269	515.3058	12.33359	538.338	64.25974	510.3	4.1	94.8	122
19AR113 Spot 222	164.3688	23862.88	2.635904	17.78774	2.320978	0.634899	2.441923	0.082923	0.608456	0.249171	513.5484	3.003493	499.143	9.629166	434.5676	52.69232	513.5	3.0	118.2	198
19AR113 Spot 306	143.4281	14853.06	3.386586	16.45838	2.258877	0.680086	2.571068	0.082992	0.796756	0.309893	513.9562	3.935988	526.8266	10.56797	583.9159	53.0751	514.0	3.9	88.0	269
19AR113 Spot 13	130.7974	8082.654	3.757672	17.44636	2.39773	0.634725	2.766075	0.083151	0.671833	0.242883	514.901	3.324726	499.0348	10.90565	427.8563	59.8602	514.9	3.3	120.3	13
19AR113 Spot 322	435.6083	50565.18	7.123357	16.95993	0.998156	0.671568	1.19075	0.083246	0.619308	0.520099	515.4659	3.068023	521.6652	4.857576	549.8666	22.20168	515.5	3.1	93.7	284
19AR113 Spot 148	208.7825	22554.33	13.40383	17.36094	1.755258	0.650984	1.994642	0.083315	0.642673	0.322199	515.8786	3.18622	509.0842	7.986037	479.6221	41.73106	515.9	3.2	107.6	133
19AR113 Spot 337	214.7845	24143.74	3.691946	17.30187	1.614094	0.657597	1.94574	0.083388	0.73396	0.377214	516.3147	3.641756	513.143	7.837981	500.0194	39.70612	516.3	3.6	103.3	299
19AR113 Spot 2	107.9179	9129.922	3.281372	17.4189	2.544826	0.640707	3.862529	0.083401	0.66766	0.172856	516.3934	3.313273	502.744	15.31663	442.0417	84.66719	516.4	3.3	116.8	2
19AR113 Spot 173	132.5593	11422.87	3.127208	17.12393	2.886038	0.65483	3.197149	0.08342	0.911687	0.285156	516.506	4.525212	511.447	12.84669	489.8762	67.62019	516.5	4.5	105.4	151
19AR113 Spot 198	84.48422	11066.32	0.926244	16.47309	3.373718	0.684143	3.916865	0.08346	0.808201	0.206339	516.7395	4.013293	529.2756	16.15747	584.6698	83.2046	516.7	4.0	88.4	175
19AR113 Spot 35	106.5493	11725.5	0.965716	17.00987	2.384155	0.664062	2.639727	0.083559	0.756845	0.286714	517.3283	3.76239	517.0956	10.69656	517.0027	55.53522	517.3	3.8	100.1	32
19AR113 Spot 388	197.5344	23553.96	1.355781	17.05965	1.705168	0.665155	1.841412	0.083581	0.629641	0.341934	517.4602	3.130807	517.7622	7.468905	520.0291	37.96407	517.5	3.1	99.5	339
19AR113 Spot 497	120.4332	10737.77	2.859746	16.65554	2.382816	0.674899	2.761506	0.083606	0.75715	0.27418	517.6109	3.765879	523.687	11.29911	551.2202	57.97932	517.6	3.8	93.9	436
19AR113 Spot 7	203.788	17649.44	5.536081	16.89567	1.767321	0.671094	2.017988	0.083614	0.707602	0.350647	517.6554	3.51973	521.3774	8.228874	538.6492	41.35954	517.7	3.5	96.1	7
19AR113 Spot 523	99.52401	9153.338	2.708648	16.81798	2.573144	0.665227	2.822492	0.083665	0.900166	0.318926	517.9605	4.480109	517.8064	11.44926	518.0606	58.75449	518.0	4.5	100.0	460
19AR113 Spot 323	115.9096	7829.339	2.809868	16.72275	3.159496	0.666442	4.353707	0.083667	0.672506	0.154468	517.9735	3.347133	518.5468	17.68093	522.0062	94.4057	518.0	3.3	99.2	285

19AR113 Spot 490	335.7324	28142.66	7.463798	17.16313	1.077772	0.664888	1.373875	0.083817	0.799735	0.582102	518.8684	3.986964	517.5995	5.57115	512.98	24.54678	518.9	4.0	101.1	430
19AR113 Spot 543	155.5315	11867.75	3.741483	16.45594	1.737162	0.68481	2.034374	0.083971	0.779108	0.382972	519.7834	3.890711	529.6772	8.396335	573.4742	40.85165	519.8	3.9	90.6	478
19AR113 Spot 329	238.7893	39272.6	3.765519	17.23214	1.073241	0.669092	1.36004	0.08416	0.761764	0.560104	520.9064	3.811987	520.1601	5.535941	517.8192	24.73814	520.9	3.8	100.6	291
19AR113 Spot 8	276.8157	28981.91	1.124531	17.28849	1.014479	0.664153	1.430342	0.084195	0.713099	0.498551	521.1135	3.569822	517.1514	5.796281	500.6629	27.31231	521.1	3.6	104.1	8
19AR113 Spot 597	138.2866	14279.07	3.152827	17.21675	2.451674	0.658863	2.81499	0.084235	0.612784	0.217686	521.3522	3.068989	513.9182	11.35297	481.9744	60.7216	521.4	3.1	108.2	524
19AR113 Spot 300	184.8035	14956.03	1.259878	16.83191	2.602961	0.675961	2.997461	0.084298	0.908028	0.302933	521.7259	4.550783	524.3301	12.27615	536.6303	62.51761	521.7	4.6	97.2	263
19AR113 Spot 19	274.5034	23493.5	0.934557	17.45549	1.063863	0.659255	1.625222	0.0843	0.623199	0.383455	521.7354	3.123355	514.1583	6.556755	481.5563	33.16	521.7	3.1	108.3	19
19AR113 Spot 195	120.3818	15242.28	2.717361	17.49161	3.275215	0.654248	3.478969	0.084327	0.709607	0.20397	521.8984	3.557478	511.0897	13.97172	463.9707	75.50619	521.9	3.6	112.5	172
19AR113 Spot 533	99.78813	7878.35	2.481663	17.08009	2.671703	0.655315	3.003256	0.084403	0.859325	0.286131	522.3477	4.311622	511.744	12.07291	465.6135	63.77916	522.3	4.3	112.2	469
19AR113 Spot 336	220.2878	22357.39	3.62071	17.0277	2.312141	0.675916	2.469499	0.084408	0.710816	0.287838	522.3783	3.566688	524.3033	10.11332	533.6431	51.78741	522.4	3.6	97.9	298
19AR113 Spot 491	69.44983	6036.49	2.111681	16.78317	3.130322	0.663118	3.366035	0.084431	0.778516	0.231286	522.5174	3.907383	516.5193	13.62832	491.0111	72.24287	522.5	3.9	106.4	431
19AR113 Spot 382	179.7414	21105.13	5.675613	17.28884	2.872797	0.662429	3.072604	0.084543	0.771627	0.251131	523.1805	3.877529	516.0985	12.43238	485.8059	65.66644	523.2	3.9	107.7	333
19AR113 Spot 278	104.8406	13275.11	1.353875	16.35518	2.31717	0.696133	2.876348	0.084588	0.741389	0.257754	523.4502	3.72742	536.4788	11.98737	593.2236	60.25933	523.5	3.7	88.2	242
19AR113 Spot 49	103.1769	9783.187	2.246296	17.06464	2.675159	0.662488	3.319815	0.084611	0.757242	0.228098	523.587	3.808081	516.1347	13.43348	484.257	71.39545	523.6	3.8	108.1	44
19AR113 Spot 296	155.0606	15767.89	2.069004	17.27177	2.289588	0.661606	2.791103	0.084648	0.903685	0.323773	523.8086	4.546368	515.5957	11.28483	480.2979	58.35863	523.8	4.5	109.1	259
19AR113 Spot 394	244.5344	18864.09	2.546612	17.23758	1.393493	0.665144	1.736095	0.084683	0.757136	0.436114	524.0173	3.81055	517.7556	7.041646	491.1571	34.44495	524.0	3.8	106.7	344
19AR113 Spot 55	46.44854	3869.978	2.128285	14.83092	6.185311	0.742373	7.359733	0.08485	0.887056	0.120528	525.0049	4.472495	563.7895	31.85044	724.4439	155.1723	525.0	4.5	72.5	49
19AR113 Spot 133	243.1088	19490.81	3.954801	17.14584	1.344041	0.671071	1.59528	0.084919	0.711173	0.445798	525.4195	3.588415	521.3634	6.504985	504.5939	31.44414	525.4	3.6	104.1	120
19AR113 Spot 167	96.50295	6171.837	1.993644	16.51057	3.36125	0.678837	5.118759	0.084929	0.864305	0.168851	525.4769	4.361546	526.0711	21.01907	529.6261	110.6255	525.5	4.4	99.2	145
19AR113 Spot 282	155.6219	15878.11	4.201229	17.24985	1.626312	0.663633	1.810172	0.08499	0.698036	0.385618	525.8384	3.524825	516.8338	7.332091	478.1597	36.9199	525.8	3.5	110.0	246
19AR113 Spot 302	145.5557	14158.99	2.454676	17.12096	2.053645	0.668975	2.34918	0.085044	0.793452	0.337757	526.1594	4.008989	520.0892	9.56135	494.4688	48.75372	526.2	4.0	106.4	265
19AR113 Spot 279	199.3421	19135.67	3.195479	16.9538	2.063721	0.678628	2.23931	0.085165	0.668857	0.298689	526.8783	3.383894	525.9446	9.19249	522.8283	46.882	526.9	3.4	100.8	243
19AR113 Spot 535	198.4537	16034.37	2.907835	16.95595	1.822496	0.677104	1.991518	0.085175	0.674357	0.338615	526.9352	3.412074	525.0229	8.164303	517.6498	41.15143	526.9	3.4	101.8	471
19AR113 Spot 421	133.7462	15026.5	1.896831	17.23254	1.848331	0.671231	2.076641	0.085176	0.744326	0.358428	526.9463	3.766172	521.4606	8.469095	498.4421	42.71341	526.9	3.8	105.7	368
19AR113 Spot 239	159.2849	14502.1	1.686843	16.73358	1.837733	0.686919	2.376233	0.08522	0.544536	0.229159	527.2078	2.75658	530.9477	9.825256	548.0246	50.53736	527.2	2.8	96.2	214
19AR113 Spot 441	96.04942	11392.52	3.832519	16.18127	2.69603	0.707645	3.042898	0.085242	0.673915	0.221471	527.3372	3.412332	543.3472	12.80437	612.0054	64.12875	527.3	3.4	86.2	387
19AR113 Spot 437	80.10814	10680.98	2.789604	16.89913	3.354137	0.677549	4.037086	0.085309	0.771261	0.191044	527.733	3.90805	525.292	16.55777	515.6321	87.06083	527.7	3.9	102.3	383
19AR113 Spot 165	282.995	32646.78	4.021714	17.1727	1.248064	0.677313	1.436656	0.085367	0.68489	0.476725	528.0772	3.472572	525.1493	5.890645	513.4025	27.74575	528.1	3.5	102.9	143
19AR113 Spot 224	78.50457	7772.891	1.70013	16.76519	4.322457	0.680145	4.543946	0.085495	0.767892	0.168992	528.8398	3.898811	526.862	18.67956	519.2351	98.34383	528.8	3.9	101.8	200
19AR113 Spot 157	262.4042	19795.63	4.670694	16.9872	1.237027	0.684655	1.479264	0.085898	0.719012	0.486061	531.2296	3.666462	529.5838	6.10438	523.4304	28.33467	531.2	3.7	101.5	136
19AR113 Spot 526	96.08855	12446.44	1.597848	16.50394	3.22748	0.700549	3.71337	0.085923	0.982032	0.264458	531.3775	5.009016	539.119	15.53394	572.9235	77.93222	531.4	5.0	92.7	463
19AR113 Spot 332	518.316	46676.49	2.206044	17.0652	0.758936	0.693773	1.090001	0.086327	0.726357	0.666382	533.7749	3.720935	535.0649	4.53338	541.4989	17.78474	533.8	3.7	98.6	294
19AR113 Spot 369	169.8588	2915.4	4.684065	16.1994	2.464632	0.67213	3.263479	0.08636	0.674912	0.206808	533.9758	3.458646	522.0068	13.32045	470.962	70.68983	534.0	3.5	113.4	320
19AR113 Spot 368	145.7074	12944.69	4.514721	13.66253	1.757145	0.855243	2.37803	0.086494	0.798152	0.335636	534.7714	4.096046	627.5229	11.1315	978.6856	45.64686	534.8	4.1	54.6	319
19AR113 Spot 380	239.3481	19480.99	3.53019	16.84653	1.561286	0.69676	1.843514	0.086705	0.816018	0.442643	536.0223	4.197125	536.8538	7.686834	541.318	36.15436	536.0	4.2	99.0	331
19AR113 Spot 181	310.6488	33343.77	2.380957	17.04943	1.269696	0.694814	1.535517	0.086706	0.849392	0.553164	536.0273	4.368821	535.6888	6.392	535.2023	28.00099	536.0	4.4	100.2	159
19AR113 Spot 312	185.4808	18525.99	27.31921	16.79223	1.904948	0.698633	2.104169	0.086737	0.661618	0.314432	536.2103	3.404123	537.974	8.787611	546.3868	43.63849	536.2	3.4	98.1	274
19AR113 Spot 431	182.73	21332.22	2.288981	17.1078	2.037234	0.691068	2.240933	0.086963	0.692988	0.309241	537.5482	3.574059	533.4417	9.298878	516.8525	46.77669	537.5	3.6	104.0	377
19AR113 Spot 291	76.94417	11235.56	1.751714	15.77195	4.020551	0.74129	4.189059	0.087044	0.656304	0.156671	538.0308	3.387776	563.1583	18.10963	666.9232	88.61042	538.0	3.4	80.7	254
19AR113 Spot 397	86.89097	13258.01	1.922444	16.29677	3.586097	0.720475	3.85134	0.087093	0.790906	0.205359	538.3214	4.084692	550.9471	16.37759	604.4127	81.5931	538.3	4.1	89.1	346
19AR113 Spot 555	251.0962	21030.16	2.514215	16.7466	1.099842	0.708572	1.5822	0.087162	0.72726	0.459651	538.7303	3.758723	543.8982	6.662673	566.5817	30.57733	538.7	3.8	95.1	484
19AR113 Spot 295	182.1559	16834.44	1.430178	17.22576	1.508446	0.684114	2.134475	0.087202	0.728579	0.341339	538.9658	3.767121	529.2579	8.804181	488.5705	44.27635	539.0	3.8	110.3	258
19AR113 Spot 528	186.5852	11015.91	1.934103	17.10946	2.233924	0.683379	2.824336	0.087229	0.939458	0.33263	539.1263	4.858857	528.8145	11.64246	485.5113	58.81128	539.1	4.9	111.0	465
19AR113 Spot 229	245.0871	27461.72	5.336824	17.36813	1.539828	0.685305	1.778132	0.087486	0.600523	0.337727	540.6492	3.114304	529.9757	7.341872	485.2374	36.95156	540.6	3.1	111.4	204
19AR113 Spot 46	98.13853	11685.97	4.982837	17.20976	1.698293	0.684739	2.248306	0.087661	0.693241	0.308339	541.691	3.601777	529.6346	9.278758	478.9549	47.29342	541.7	3.6	113.1	41
19AR113 Spot 567	191.8424	21545.67	1.631759	17.15339	2.051539	0.698242	2.25434	0.08801	0.809187	0.358946	543.7582	4.219558	537.7404	9.411701	513.2573	46.22613	543.8	4.2	105.9	496
19AR113 Spot 510	233.7027	19020.59	1.488761	16.99234	1.541447	0.70504	1.996995	0.088013	0.619001	0.309966	543.7771	3.22793	541.7969	8.384865	534.4065	41.55488	543.8	3.2	101.8	447
19AR113 Spot 67	297.0965	32339.71	2.582885	17.05599	1.127514	0.708009	1.461167	0.08879	0.725495	0.496518	548.3762	3.813926	543.5635	6.150122	524.3662	27.81696	548.4	3.8	104.6	60
19AR113 Spot 142	203.4372	28708.85	4.00618	16.89659	1.363902	0.721075	1.766373	0.089541	0.753878	0.426794	552.8222	3.993906	551.3012	7.51451	545.9525	34.91105	552.8	4.0	101.3	127
19AR113 Spot 250	135.5379	14161.58	3.954885	15.50165	1.789146	0.795207	2.082182	0.091075	0.71381	0.342819	561.8944	3.84103	594.1214	9.365404	720.0798	41.52014	561.9	3.8	78.0	225
19AR113 Spot 285	83.50641	5715.805	1.095143	16.12873	3.399228	0.741545	5.141145	0.09108	0.782703	0.152243	561.9211	4.211935	563.3067	22.23115	569.8746	110.6364	561.9	4.2	98.6	249
19AR113 Spot 56	107.4167	11216	2.615581	17.0257	2.619846	0.724655	2.750502	0.091854	0.665155	0.24183	566.4929	3.607235	553.4115	11.7352	500.9208	58.7797	566.5	3.6	113.1	50
19AR113 Spot 183	190.8069	21087.9	2.268997	16.92357	1.611284	0.739855	1.859119	0.091943	0.827904	0.445321	567.0154	4.493815	562.3211	8.027492	544.3076	36.37211	567.0	4.5	104.2	161
19AR113 Spot 21	88.56356	7092.029	4.474642	16.82046	3.260003	0.738865	3.474484	0.093272	0.954016	0.274578	574.8575	5.246814	561.743	14.9917	509.9165	73.46434	574.9	5.2	112.7	21
19AR113 Spot 68	193.4564	3161.87	1.195766	15.61806	1.534369	0.770213	1.979638	0.094554	0.818858	0.41364	582.4113	4.560028	579.8851	8.746048	570.9406	39.19596	582.4	4.6	102.0	61
19AR113 Spot 205	288.4661	8347.742	23.86088	16.20296	1.070933	0.784575	1.518424	0.094774	0.97568	0.642561	583.7109	5.444919	588.0898	6.778427	605.9482	25.1756	583.7	5.4	96.3	182
19AR113 Spot 75	167.1928	20419.02	4.326531	16.34186	2.274745	0.785862	2.455236	0.094822	0.851609	0.346854	583.9942	4.754725	588.8217	10.9708	608.3956	49.77282	584.0	4.8	96.0	68
19AR113 Spot 117	338.2421	2456.419	0.857928	16.01938	1.229208	0.740489	5.421035	0.09525	0.816684	0.150651	586.5134	4.578521	562.6908	23.42267	468.4813	118.712	586.5	4.6	125.2	105
19AR113 Spot 91	226.049	18991.41	0.501363	16.63436	1.153628	0.775063	1.469004	0.095267	0.710805	0.483869	586.6092	3.985561	582.6634	6.513018	568.2826	27.98058	586.6	4.0	103.2	84
19AR113 Spot 147	279.2709	8278.543	6.063184	13.25912	0.793772	0.964014	2.100186	0.095347	0.818541	0.389747	587.0811	4.593176	685.3739	10.46747	1023.925	39.14444	587.1	4.6	57.3	132
19AR113 Spot 477	61.32584	6750.33	1.556977	16.48305	4.354344	0.769455	4.866812	0.095766	0.8516	0.174981	589.5504	4.797874	579.4504	21.49231	541.0033	104.8581	589.6	4.8	109.0	419
19AR113 Spot 59	150.7174	20083.88	58.00488	16.23687	1.7008	0.805869	1.894958	0.096393	0.791793	0.417842	593.2365	4.487555	600.1339	8.586536	627.1965	37.12119	593.2	4.5	94.6	53
19AR113 Spot 43	218.4622	27739.35	2.381101	16.5364	1.163382	0.796917	1.480582	0.096461	0.683179	0.461426	593.6373	3.874471	595.0879	6.667355	601.5427	28.43298	593.6	3.9	98.7	38
19AR113 Spot 70	242.4891	24136.51	2.217107	16.23962	1.071099	0.8083	1.439549	0.096738	0.648979	0.450821	595.2661	3.690153	601.4999	6.533781	625.9838	27.71705	595.3	3.7	95.1	63
19AR113 Spot 73	269.1733	38186.29	89.21658	16.46965	1.00293	0.800143	1.27997	0.096752	0.661722	0.516982	595.3482	3.763107	596.9095	5.776902	603.7669	23.68665	595.3	3.8	98.6	66
19AR113 Spot 309	406.4778	50053.51	0.697564	16.43338	0.913365	0.805645	1.166356	0.097004	0.706839	0.606024	596.8276	4.029216	600.0078	5.284162	612.9637	20.06493	596.8	4.0	97.4	272
19AR113 Spot 143	367.6416	46061.36	4.375396	16.35533	0.929765	0.811124	1.202627	0.097163	0.716003	0.595366	597.7629	4.087558	603.0841	5.46895	624.0534	20.85656	597.8	4.1	95.8	128
19AR113 Spot 343	150.996	22649.48	2.376287	16.57368	1.356382	0.798444	1.981015	0.097253	1.119252	0.564989	598.2925	6.395052	595.9507	8.930514	588.0132	35.45201	598.3	6.4	101.7	305
19AR113 Spot 398	224.8731	26530.08	1.010484	16.46464	1.77748	0.806522	2.088562	0.097623	0.820451	0.392831	600.4642	4.704034	600.5012	9.468102	601.5623	41.59617	600.5	4.7	99.8	347
19AR113 Spot 284	532.9358	3818.904	7.705923	15.52474	0.749281	0.812259	1.106384	0.097858	0.685673	0.619742	601.8441	3.939903	603.7203	5.035162	611.6906	18.78366	601.8	3.9	98.4	248
19AR113 Spot 57	307.3456	27666.96	2.937448	16.55503	0.951962	0.807752	1.161749	0.098137	0.65566	0.564373	603.4801	3.777218	601.1923	5.270907	593.539	20.78476	603.5	3.8	101.7	51
19AR113 Spot 136	148.575	20715.75	4.342561	16.39971	1.62728	0.813532	2.044891	0.098299	0.686732	0.335828	604.4348	3.962191	604.4332	9.314544	605.3486	41.68962	604.4	4.0	99.8	123
19AR113 Spot 320	172.384	19233.79	3.392386	16.69369	2.030851	0.801581	2.149652	0.098566	0.62016	0.288493	605.9981	3.586919	597.7199	9.711913	567.4032	44.81256	606.0	3.6	106.8	282
19AR113 Spot 201	169.8533	22683.93	1.971011	16.79292	1.331156	0.802026	1.685666	0.098647	0.72775	0.431729	606.4727	4.212355	597.9708	7.617929	566.8259	33.10632	606.5	4.2	107.0	178
19AR113 Spot 108	324.157	42706.68	13.65468	16.48524	0.697753	0.821777	0.947507	0.099078	0.59866	0.631826	609.0055	3.478952	609.0394	4.339839	610.0867	15.8665	609.0	3.5	99.8	100
19AR113 Spot 507	117.7664	16984.58	7.341306	16.55287	2.503235	0.816148	2.870365	0.09934	0.815571	0.284135	610.542	4.750875	605.8973	13.09812	589.4859	59.68526	610.5	4.8	103.6	444
19AR113 Spot 84	456.1311	52913.09	1.201421	16.514	0.820252	0.821294	1.076907	0.099356	0.691445	0.642066	610.6337	4.028391	608.7704	4.930946	602.7632	17.84685	610.6	4.0	101.3	77
19AR113 Spot 396	82.79824	15107.38	1.565851	16.14875	2.753526	0.834194	2.891114	0.099687	0.846597	0.292827	612.5714	4.947235	615.9367	13.35187	629.244	59.55371	612.6	4.9	97.4	345
19AR113 Spot 210	489.5007	76072	2.686925	16.50603	0.968509	0.832592	1.206897	0.099895	0.707339	0.586081	613.7959	4.141331	615.0495	5.567637	620.5865	21.10164	613.8	4.1	98.9	186
19AR113 Spot 268	268.2985	28839.42	20.62229	15.56812	0.889096	0.876458	1.285547	0.100086	0.812797	0.632258	614.911	4.767007	639.0681	6.096989	726.3342	21.11678	614.9	4.8	84.7	234
19AR113 Spot 237	71.82711	6363.826	1.081695	15.87074	3.518759	0.835835	4.15058	0.100321	0.770726	0.185691	616.2897	4.529923	616.8445	19.1901	619.7993	88.02094	616.3	4.5	99.4	212
19AR113 Spot 482	37.14657	3661.118	5.678804	15.83149	5.121087	0.81756	5.793482	0.100492	1.026524	0.177186	617.2915	6.042715	606.6864	26.46664	568.2406	124.2032	617.3	6.0	108.6	423
19AR113 Spot 583	372.8323	14368.99	5.779885	16.18643	0.826465	0.841935	1.411662	0.100865	0.762648	0.540248	619.4788	4.504542	620.2128	6.551957	623.8105	25.59966	619.5	4.5	99.3	512
19AR113 Spot 16	252.5606	32592.01	5.33932	16.5068	0.891019	0.834725	1.173573	0.10095	0.59405	0.506189	619.9742	3.511398	616.2306	5.421467	603.4215	21.88096	620.0	3.5	102.7	16
19AR113 Spot 74	168.5487	13972.85	6.100172	16.31092	0.965916	0.837717	1.457823	0.101433	0.627574	0.430487	622.8015	3.725671	617.8853	6.747763	600.8371	28.47527	622.8	3.7	103.7	67
19AR113 Spot 30	372.3978	41186.19	4.860477	16.3846	0.848921	0.85256	1.118287	0.101558	0.65775	0.588176	623.5319	3.909174	626.0529	5.225643	636.1296	19.48331	623.5	3.9	98.0	30
19AR113 Spot 211	454.8326	50906.42	3.370737	16.50357	0.756483	0.852984	1.052244	0.102486	0.699542	0.66481	628.9636	4.192035	626.2857	4.918346	617.5448	16.98937	629.0	4.2	101.8	187
19AR113 Spot 275	263.6771	29278.34	2.743117	16.28498	1.022472	0.854675	1.289297	0.102527	0.70016	0.543056	629.1993	4.197235	627.2118	6.03283	620.9698	23.36226	629.2	4.2	101.3	239
19AR113 Spot 512	486.8714	85517.3	3.770879	16.46948	1.011616	0.856581	1.357473	0.102588	0.869415	0.640465	629.5564	5.21468	628.2546	6.359477	624.4884	22.5005	629.6	5.2	100.8	449
19AR113 Spot 47	235.5308	29025.21	2.81799	16.57226	0.674644	0.84406	1.028105	0.102676	0.629421	0.612214	630.0738	3.778167	621.3839	4.778252	590.8265	17.60775	630.1	3.8	106.6	42
19AR113 Spot 342	238.339	30540.83	2.10832	16.24242	1.077363	0.863516	1.290525	0.102775	0.690734	0.535235	630.6504	4.149818	632.0406	6.072098	637.9743	23.47393	630.7	4.1	98.9	304
19AR113 Spot 4	97.23037	4842.253	1.245239	15.64814	3.733125	0.86219	4.541265	0.102918	0.883597	0.194571	631.4862	5.315207	631.3176	21.35255	631.6729	95.99522	631.5	5.3	100.0	4
19AR113 Spot 572	531.7261	35823.86	1.577581	16.35964	0.665783	0.861617	0.939625	0.102995	0.648466	0.690133	631.9369	3.903445	631.0051	4.415818	628.5856	14.67235	631.9	3.9	100.5	501
19AR113 Spot 536	249.0971	34924.49	1.939578	16.23715	1.30637	0.862987	1.476376	0.103052	0.641316	0.434386	632.2696	3.862343	631.7522	6.944294	630.8588	28.65765	632.3	3.9	100.2	472
19AR113 Spot 374	75.07591	11929.01	4.582061	16.14199	2.592874	0.860822	3.201177	0.103366	0.748433	0.233799	634.1075	4.519925	630.5715	15.03765	618.8313	67.1892	634.1	4.5	102.5	325
19AR113 Spot 436	69.72568	9219.007	2.646244	14.85336	2.815921	0.933888	3.217595	0.103369	0.754574	0.234515	634.1219	4.55711	669.678	15.77828	792.1148	65.63456	634.1	4.6	80.1	382
19AR113 Spot 28	175.5537	23299.93	2.445629	16.65204	1.244237	0.851096	1.448994	0.103708	0.597487	0.412346	636.1038	3.619138	625.2505	6.764762	587.131	28.62283	636.1	3.6	108.3	28
19AR113 Spot 333	270.2535	41384.78	1.900947	16.10907	1.326194	0.88457	1.615525	0.104045	0.8064	0.499157	638.0686	4.898939	643.4479	7.699664	663.2862	29.99182	638.1	4.9	96.2	295
19AR113 Spot 25	260.049	27992.01	2.055979	16.33671	1.082762	0.87397	1.327535	0.104085	0.632479	0.476431	638.3059	3.843712	637.7207	6.286593	636.6039	25.11761	638.3	3.8	100.3	25
19AR113 Spot 420	83.99154	13591.36	2.037794	16.55676	2.443178	0.852901	2.746997	0.104089	1.001154	0.364454	638.3281	6.084434	626.2403	12.83978	583.7411	55.54344	638.3	6.1	109.4	367
19AR113 Spot 427	273.4222	51504.25	7.087706	16.54357	1.245315	0.864165	1.440561	0.104143	0.662804	0.460101	638.6401	4.030012	632.3942	6.780792	611.0477	27.66288	638.6	4.0	104.5	374
19AR113 Spot 537	138.3986	20962.26	1.850168	16.36599	1.576949	0.862758	1.824969	0.104323	0.708159	0.388039	639.691	4.312521	631.6272	8.582784	603.7883	36.39508	639.7	4.3	105.9	473
19AR113 Spot 187	432.9548	46227.02	5.387628	16.34096	0.912547	0.877215	1.224096	0.104498	0.791165	0.646326	640.716	4.82535	639.4772	5.808207	636.0603	20.11249	640.7	4.8	100.7	165
19AR113 Spot 423	267.9226	22271.93	0.979385	16.34024	1.203933	0.874935	1.439206	0.104739	0.764836	0.531429	642.121	4.674496	638.2436	6.819446	625.459	26.30401	642.1	4.7	102.7	370
19AR113 Spot 452	162.8602	26201.49	1.227802	16.0693	1.29594	0.886238	1.521699	0.104745	0.695938	0.457343	642.1568	4.253635	644.3462	7.259722	652.9655	29.04283	642.2	4.3	98.3	398
19AR113 Spot 508	23.19077	2885.132	3.756866	14.87287	5.129702	0.901722	5.734159	0.105067	1.478541	0.257848	644.0329	9.062087	652.6473	27.61419	683.4966	118.3625	644.0	9.1	94.2	445
19AR113 Spot 362	140.4983	18277.5	10.47635	16.10887	1.658599	0.884042	2.1144	0.105201	0.781699	0.369703	644.8169	4.796634	643.1633	10.07427	638.3173	42.26213	644.8	4.8	101.0	314
19AR113 Spot 403	251.4963	30567.33	6.351542	16.27215	1.134223	0.883528	1.341176	0.105243	0.669447	0.49915	645.0643	4.109336	642.8862	6.388067	636.1985	25.02079	645.1	4.1	101.4	352
19AR113 Spot 137	521.2651	80764.22	3.67536	16.18759	0.80252	0.891001	1.097637	0.105355	0.701946	0.639506	645.7155	4.312962	646.907	5.251447	651.9813	18.11312	645.7	4.3	99.0	124
19AR113 Spot 102	136.8901	18696.2	0.856931	15.87254	1.632312	0.903134	1.861432	0.105697	0.717489	0.38545	647.71	4.42141	653.401	8.969562	674.0347	36.732	647.7	4.4	96.1	94
19AR113 Spot 18	210.3461	15549.68	0.805978	16.24883	1.359811	0.883109	1.865234	0.105714	0.648511	0.347684	647.8108	3.996933	642.6603	8.882045	625.5185	37.69064	647.8	4.0	103.6	18
19AR113 Spot 245	161.4794	23988.11	46.41015	16.12048	1.249223	0.89229	1.575292	0.105817	0.543769	0.345186	648.4116	3.354334	647.5989	7.542533	645.7229	31.77806	648.4	3.4	100.4	220
19AR113 Spot 280	394.38	54509.35	51.84027	16.06228	0.765236	0.904827	1.088408	0.106651	0.694031	0.637657	653.2709	4.31174	654.3041	5.249713	658.8233	17.96633	653.3	4.3	99.2	244
19AR113 Spot 174	185.1746	26442.57	1.32251	16.36231	1.294563	0.891816	1.716763	0.10711	0.838011	0.488134	655.9412	5.226446	647.3447	8.217625	618.3964	32.33581	655.9	5.2	106.1	152
19AR113 Spot 568	159.6932	26792.95	2.166793	16.37917	1.698483	0.912723	1.90952	0.109489	0.823219	0.431113	669.7778	5.236969	658.5043	9.25237	620.9981	37.19532	669.8	5.2	107.9	497
19AR113 Spot 263	141.1105	18814.88	4.774408	15.05554	1.327847	0.99056	1.766482	0.109671	0.904278	0.511909	670.8347	5.761245	699.006	8.925977	791.5632	31.85983	670.8	5.8	84.7	229
19AR113 Spot 5	377.0524	64337.15	8.806148	16.06315	0.832008	0.941068	1.160782	0.110322	0.788793	0.679535	674.618	5.052363	673.441	5.714332	670.418	18.23396	674.6	5.1	100.6	5
19AR113 Spot 246	211.096	29244.33	2.511252	16.08703	1.120662	0.936161	1.322936	0.110593	0.629287	0.475675	676.1894	4.039604	670.8711	6.495069	653.982	24.97033	676.2	4.0	103.4	221
19AR113 Spot 303	95.5634	12173.07	2.061175	15.16298	2.565021	0.996968	2.827388	0.112299	1.157886	0.409525	686.0869	7.535972	702.2692	14.33356	755.2905	54.42393	686.1	7.5	90.8	266
19AR113 Spot 434	595.7886	7844.518	3.600042	15.09148	0.631687	0.995031	1.922875	0.112424	0.967376	0.503089	686.8118	6.302365	701.2841	9.738256	748.8355	35.09053	686.8	6.3	91.7	380
19AR113 Spot 216	112.8724	13972.92	3.63044	16.19956	2.263856	0.943389	2.856381	0.112764	0.713616	0.249832	688.7827	4.661778	674.6544	14.08008	628.6877	59.58713	688.8	4.7	109.6	192
19AR113 Spot 426	71.12569	16664.23	0.539074	15.5613	2.909877	1.043086	3.221461	0.119297	0.722382	0.22424	726.5148	4.963279	725.4518	16.7015	723.0709	66.61086	726.5	5.0	100.5	373
19AR113 Spot 407	625.9621	14118.82	4.063649	14.76844	0.682225	1.100958	1.064936	0.119768	0.797929	0.749275	729.226	5.501667	753.8137	5.666452	828.3995	14.69127	729.2	5.5	88.0	355
19AR113 Spot 327	79.74748	11546.7	1.176523	15.49015	2.014973	1.059936	2.471461	0.121675	0.906534	0.366801	740.1983	6.33924	733.7918	12.91317	715.203	48.84606	740.2	6.3	103.5	289
19AR113 Spot 188	190.2711	29999.12	6.063283	14.65765	1.205434	1.161332	1.591761	0.124307	0.770613	0.484126	755.3085	5.492459	782.5808	8.684669	862.0307	28.8973	755.3	5.5	87.6	166
19AR113 Spot 418	108.7013	19300.4	1.862315	14.48856	1.734974	1.175454	2.188916	0.124782	0.835646	0.381763	758.0276	5.97618	789.1935	12.00977	879.1926	41.87671	758.0	6.0	86.2	365
19AR113 Spot 177	267.2552	30074.55	5.446477	14.59139	1.151344	1.172969	1.697936	0.125377	1.08568	0.639412	761.438	7.797227	788.0332	9.306713	864.9829	27.06088	761.4	7.8	88.0	155
19AR113 Spot 348	374.1234	56885.47	3.149267	15.22552	0.969006	1.143231	1.240385	0.127355	0.758705	0.611669	772.7601	5.525194	774.041	6.718274	778.6321	20.63212	772.8	5.5	99.2	310
19AR113 Spot 209	374.921	69462.6	7.121258	15.32807	0.854533	1.149426	1.132784	0.128072	0.72101	0.636494	776.8565	5.276871	776.972	6.150931	778.1981	18.37038	776.9	5.3	99.8	185
19AR113 Spot 518	179.8088	30241.86	1.63281	15.33279	1.366263	1.159442	1.637109	0.130109	0.878743	0.536765	788.4874	6.521801	781.6926	8.925368	763.2429	29.09463	788.5	6.5	103.3	455
19AR113 Spot 561	416.9668	49857.85	3.682541	15.24936	0.751142	1.172146	1.171529	0.130405	0.890844	0.760412	790.1733	6.624897	787.6483	6.419206	781.4008	15.98939	790.2	6.6	101.1	490
19AR113 Spot 274	117.2355	9000.47	4.81493	14.20772	1.833579	1.237766	2.282578	0.131471	0.772277	0.338335	796.253	5.78467	817.8687	12.82043	878.0366	44.45749	796.3	5.8	90.7	238
19AR113 Spot 560	114.3376	24455.36	2.59982	13.83658	1.532447	1.32487	1.887724	0.134235	0.92056	0.487656	811.9829	7.023197	856.6423	10.92345	974.969	33.58112	812.0	7.0	83.3	489
19AR113 Spot 392	307.6183	44651.29	1.408519	14.9767	0.930372	1.229323	1.204675	0.134875	0.749596	0.622239	815.6153	5.742862	814.0303	6.745267	810.5896	19.74622	815.6	5.7	100.6	342
19AR113 Spot 262	541.1151	59774.51	3.317051	14.82649	0.676464	1.259007	1.005913	0.135856	0.734326	0.730009	821.1882	5.661918	827.4614	5.692531	845.2549	14.30162	821.2	5.7	97.2	228
19AR113 Spot 200	567.8132	55402.57	8.268816	14.41996	0.630114	1.303592	0.909769	0.136712	0.641334	0.704942	826.0446	4.97233	847.3063	5.227584	904.2878	13.29944	826.0	5.0	91.3	177
19AR113 Spot 141	91.82599	14345.73	4.290376	14.82228	1.923434	1.249354	2.450296	0.136965	0.644842	0.263169	827.4773	5.007649	823.1134	13.81984	812.2718	49.43984	827.5	5.0	101.9	126
19AR113 Spot 118	40.57049	6638.187	4.833048	13.9985	4.201814	1.316534	4.549909	0.138041	0.922731	0.202802	833.5779	7.21514	852.995	26.2617	904.7089	91.84422	833.6	7.2	92.1	106
19AR113 Spot 474	225.1611	34033.95	11.57069	14.13251	0.956041	1.336724	1.346085	0.138413	0.90749	0.67417	835.6821	7.112746	861.8061	7.818894	930.4832	20.41931	835.7	7.1	89.8	417
19AR113 Spot 69	192.6103	46476.22	1.590881	14.76956	1.089372	1.291138	1.257252	0.139807	0.609731	0.484971	843.5687	4.821177	841.8018	7.194176	838.0459	22.87981	843.6	4.8	100.7	62
19AR113 Spot 276	114.9178	13661.99	4.965864	14.51696	1.331944	1.301845	1.773729	0.14023	0.876734	0.494288	845.9615	6.950785	846.5358	10.18626	848.9262	32.03904	846.0	7.0	99.7	240
19AR113 Spot 321	88.99941	19100.29	2.547957	14.1046	1.386299	1.383102	1.873499	0.143401	0.774554	0.413427	863.8631	6.262139	881.7615	11.04109	927.8267	35.05008	863.9	6.3	93.1	283
19AR113 Spot 269	95.61432	17864.29	3.488535	14.33823	2.302929	1.484712	2.511749	0.156946	0.756436	0.301159	939.782	6.614965	924.1576	15.2407	887.9672	49.49756	888.0	49.5	105.8	235
19AR113 Spot 72	137.4174	2934.043	2.085521	13.5181	2.191647	1.535736	6.760299	0.162197	0.76993	0.11389	968.9743	6.926798	944.7976	41.59597	889.7483	138.8868	889.7	138.9	108.9	65
19AR113 Spot 227	40.56634	6669.043	3.038956	14.05469	2.758266	1.542722	2.939695	0.162373	0.78813	0.268099	969.9527	7.097176	947.5911	18.11202	896.8991	58.44909	896.9	58.4	108.1	202
19AR113 Spot 103	196.8121	33076.88	3.427053	14.23401	0.92326	1.521388	1.211968	0.158282	0.604119	0.498461	947.2234	5.3218	939.0358	7.42556	920.7931	21.60927	920.8	21.6	102.9	95
19AR113 Spot 595	302.467	40943.29	9.69257	14.17174	0.612153	1.453044	0.900641	0.150981	0.613071	0.680704	906.4617	5.184222	911.1335	5.417007	923.3853	13.56223	923.4	13.6	98.2	522
19AR113 Spot 443	92.09397	19449.64	5.323487	14.06692	1.688699	1.521129	1.968837	0.157645	0.704312	0.35773	943.6772	6.182851	938.9315	12.06233	928.6885	37.74953	928.7	37.7	101.6	389
19AR113 Spot 218	117.8247	23999.64	2.448025	14.13913	1.453601	1.577882	1.785354	0.163362	0.685088	0.383727	975.4309	6.201545	961.5354	11.09642	930.7643	33.84146	930.8	33.8	104.8	194
19AR113 Spot 60	22.50904	4174.118	1.329682	13.48709	6.387092	1.58878	7.115185	0.163554	1.092726	0.153577	976.4993	9.901596	965.8191	44.3671	942.4746	144.2341	942.5	144.2	103.6	54
19AR113 Spot 298	160.7195	27363.28	4.797151	13.91845	1.193674	1.558214	1.534309	0.159255	0.853019	0.555963	952.6342	7.554243	953.7589	9.489554	957.225	26.0904	957.2	26.1	99.5	261
19AR113 Spot 563	129.84	5770.1	2.366055	13.59762	1.353971	1.678315	3.293934	0.17152	0.791025	0.240146	1020.483	7.465766	1000.343	20.96131	957.3337	65.37954	957.3	65.4	106.6	492
19AR113 Spot 235	192.1479	29542.97	6.287626	13.88694	1.032118	1.420217	1.403656	0.144745	0.707584	0.5041	871.437	5.767536	897.4538	8.36375	962.9925	24.76009	963.0	24.8	90.5	210
19AR113 Spot 10	204.5579	4878.736	2.048495	13.4632	1.014928	1.550765	1.483727	0.157985	0.760705	0.512699	945.5709	6.69034	950.7977	9.159489	963.8242	26.0153	963.8	26.0	98.1	10

19AR113 Spot 520	223.8806	35815.09	3.130077	13.93217	0.948688	1.594358	1.337526	0.162365	0.923261	0.690276	969.9041	8.313656	968.0045	8.346373	964.6017	19.74404	964.6	19.7	100.5	457
19AR113 Spot 386	252.5443	50204.66	3.406384	13.91156	0.800658	1.433355	1.296672	0.145881	0.957783	0.738647	877.834	7.860415	902.9508	7.755623	965.8227	17.86392	965.8	17.9	90.9	337
19AR113 Spot 376	65.52462	11769.25	1.095745	13.71901	1.885174	1.685841	2.090534	0.171531	0.786183	0.376068	1020.542	7.420464	1003.192	13.32442	966.3451	39.5303	966.3	39.5	105.6	327
19AR113 Spot 335	127.7159	18869.48	2.132107	13.86218	1.183737	1.52386	1.461241	0.154831	0.685116	0.468859	927.9852	5.921352	940.0308	8.958653	969.2713	26.3464	969.3	26.3	95.7	297
19AR113 Spot 402	44.46215	9494.447	0.646416	13.63017	2.727876	1.718152	2.933518	0.173936	0.800145	0.272759	1033.762	7.642448	1015.335	18.83026	976.6805	57.51166	976.7	57.5	105.8	351
19AR113 Spot 479	170.4241	27037.38	3.03851	13.80634	1.084154	1.516784	1.483847	0.153519	0.930994	0.627419	920.6597	7.987353	937.1804	9.080472	977.1157	23.53524	977.1	23.5	94.2	420
19AR113 Spot 110	37.25599	9472.812	2.74885	13.62551	3.829644	1.71132	4.170693	0.173132	0.879372	0.210846	1029.344	8.366066	1012.779	26.73554	978.0143	83.10632	978.0	83.1	105.2	101
19AR113 Spot 525	88.63638	20263.25	2.505361	13.7348	1.598995	1.669517	1.941371	0.168765	0.885772	0.456261	1005.305	8.245105	997.0022	12.32872	979.6522	35.19485	979.7	35.2	102.6	462
19AR113 Spot 593	75.73011	20111.32	2.580601	13.72873	1.16544	1.674953	1.488479	0.169303	0.644054	0.432693	1008.272	6.011446	999.0677	9.463934	979.7898	27.33178	979.8	27.3	102.9	520
19AR113 Spot 271	421.2446	95446.03	6.841412	13.84098	0.754573	1.541029	1.049084	0.155766	0.724301	0.690413	933.2047	6.292751	946.915	6.460218	979.7946	15.4524	979.8	15.5	95.2	236
19AR113 Spot 587	286.5199	55547.86	2.988478	13.82249	0.767982	1.650225	0.986075	0.166734	0.604473	0.613009	994.0911	5.568618	989.6375	6.234561	980.6448	15.85443	980.6	15.9	101.4	515
19AR113 Spot 78	571.1855	15857.65	5.093429	13.67484	0.642628	1.434544	1.105821	0.14491	0.864437	0.781715	872.3656	7.053062	903.4469	6.616331	981.1407	14.03324	981.1	14.0	88.9	71
19AR113 Spot 130	292.2136	44511.35	4.135566	13.80132	0.859111	1.574716	1.148456	0.158994	0.676325	0.588899	951.1865	5.981005	960.2877	7.132214	982.0958	18.88308	982.1	18.9	96.9	117
19AR113 Spot 586	36.17877	7133.873	1.568195	13.47857	3.517028	1.726101	4.0696	0.174146	0.792083	0.194634	1034.914	7.573212	1018.3	26.1699	983.6558	81.31873	983.7	81.3	105.2	514
19AR113 Spot 346	98.74199	18514.55	7.215702	13.68484	1.470261	1.681029	1.828117	0.169453	0.783117	0.428374	1009.097	7.314958	1001.372	11.63929	985.4008	33.60488	985.4	33.6	102.4	308
19AR113 Spot 538	141.9698	27060.6	2.078151	13.69677	1.027679	1.651787	1.322563	0.166467	0.731344	0.552975	992.6159	6.728157	990.2358	8.36511	985.8445	22.40849	985.8	22.4	100.7	474
19AR113 Spot 513	297.57	54483.6	2.007708	13.81351	0.921878	1.509344	1.401786	0.152073	1.031934	0.736157	912.5735	8.780973	934.1742	8.561501	986.3497	19.29772	986.3	19.3	92.5	450
19AR113 Spot 410	43.86195	6716.582	2.518052	13.4732	3.189516	1.591973	3.925638	0.160313	0.829806	0.211381	958.5135	7.39073	967.0704	24.48666	987.4411	78.11829	987.4	78.1	97.1	357
19AR113 Spot 455	45.12787	9510.893	1.926173	13.51954	3.324228	1.721183	3.812798	0.173245	0.928143	0.243428	1029.966	8.834983	1016.466	24.49217	988.4042	75.27815	988.4	75.3	104.2	400
19AR113 Spot 522	115.8011	24039.71	2.177787	13.73096	1.331431	1.574362	1.617028	0.158426	0.882218	0.54558	948.021	7.777703	960.148	10.04145	988.8998	27.54974	988.9	27.5	95.9	459
19AR113 Spot 120	46.25407	9251.841	3.45142	13.57687	2.986503	1.658289	3.309435	0.166816	0.807562	0.244018	994.5428	7.442678	992.7224	20.96542	989.5693	65.30455	989.6	65.3	100.5	108
19AR113 Spot 96	916.1789	171358.2	19.91944	13.77767	0.706197	1.603289	1.025066	0.161251	0.742556	0.724398	963.727	6.646982	971.4938	6.410282	989.9682	14.37284	990.0	14.4	97.3	88
19AR113 Spot 121	123.6658	27544.3	2.549916	13.76294	1.417305	1.645098	1.639116	0.165426	0.754735	0.460453	986.8627	6.906106	987.6714	10.35153	990.3735	29.57582	990.4	29.6	99.6	109
19AR113 Spot 509	107.3548	30044.01	3.232646	13.77661	1.172034	1.641591	1.365943	0.16507	0.677903	0.49629	984.89	6.191586	986.3242	8.619306	990.4209	24.11951	990.4	24.1	99.4	446
19AR113 Spot 161	185.1927	34421.99	2.967473	13.7138	0.901644	1.693104	1.158626	0.170225	0.717179	0.618991	1013.351	6.725117	1005.934	7.396248	990.7173	18.5067	990.7	18.5	102.3	140
19AR113 Spot 51	170.7386	23241.55	7.987964	13.6913	1.249026	1.647966	1.546098	0.165665	0.654062	0.42304	988.1845	5.99232	988.7718	9.770487	990.9808	28.47121	991.0	28.5	99.7	46
19AR113 Spot 144	275.7612	57797.71	3.025595	13.74201	0.736793	1.448505	0.963256	0.145591	0.592235	0.614827	876.2037	4.851979	909.253	5.786227	991.2787	15.44878	991.3	15.4	88.4	129
19AR113 Spot 435	98.78776	16124.13	1.166862	13.61574	1.627876	1.635946	1.934294	0.16412	0.667276	0.344971	979.6299	6.064379	984.1522	12.19006	995.1276	36.90165	995.1	36.9	98.4	381
19AR113 Spot 101	60.65501	13007.56	1.64312	13.55158	2.043756	1.674914	2.195875	0.167825	0.665467	0.303053	1000.117	6.164869	999.053	13.962	997.5847	42.51762	997.6	42.5	100.3	93
19AR113 Spot 384	77.09409	11905.49	2.61923	13.51181	1.567055	1.633599	1.923671	0.163487	0.789131	0.410221	976.1284	7.148093	983.2475	12.1165	1000.041	35.61153	1000.0	35.6	97.6	335
19AR113 Spot 570	130.2867	24820.3	3.173206	13.66169	1.1099	1.726248	1.342806	0.172534	0.657628	0.489741	1026.06	6.238043	1018.354	8.633593	1002.69	23.76679	1002.7	23.8	102.3	499
19AR113 Spot 164	268.7706	51114.88	15.18095	13.66521	0.836345	1.624547	1.167964	0.162306	0.773779	0.662502	969.5814	6.965469	979.7517	7.340826	1003.48	17.74562	1003.5	17.7	96.6	142
19AR113 Spot 225	88.93298	21096.1	1.818601	13.61546	1.208631	1.691349	1.401964	0.168806	0.558549	0.398404	1005.527	5.200244	1005.272	8.946256	1005.582	26.09255	1005.6	26.1	100.0	201
19AR113 Spot 26	175.1473	26393.31	3.035268	13.70367	0.781039	1.642478	1.147268	0.163911	0.696981	0.607514	978.4728	6.327426	986.6649	7.24086	1005.795	18.48073	1005.8	18.5	97.3	26
19AR113 Spot 127	126.9893	24836.74	1.932815	13.61053	1.080274	1.70641	1.399861	0.170231	0.679171	0.48517	1013.384	6.368903	1010.939	8.962227	1006.508	24.81572	1006.5	24.8	100.7	114
19AR113 Spot 556	683.7692	135789.8	50.58688	13.71382	0.608908	1.674632	0.914123	0.166984	0.674323	0.737673	995.4734	6.220095	998.9459	5.811585	1007.44	12.51548	1007.4	12.5	98.8	485
19AR113 Spot 480	143.3735	30564.42	1.895109	13.60962	0.939721	1.60443	1.232244	0.159925	0.775191	0.629089	956.3579	6.889899	971.9387	7.708021	1008.193	19.41959	1008.2	19.4	94.9	421
19AR113 Spot 542	112.3143	25751.42	1.258889	13.54738	0.934598	1.712631	1.293578	0.170689	0.779696	0.602744	1015.906	7.328376	1013.27	8.29288	1008.439	20.92674	1008.4	20.9	100.7	477
19AR113 Spot 240	59.05143	8839.188	3.151934	13.3754	2.442996	1.658584	2.919751	0.16528	0.60156	0.206031	986.0512	5.500301	992.8352	18.49741	1008.719	57.97572	1008.7	58.0	97.8	215
19AR113 Spot 48	113.1122	19493.43	2.319668	13.54946	1.438466	1.686679	1.621845	0.168028	0.67157	0.414078	1001.238	6.227859	1003.509	10.33882	1009.335	29.94042	1009.3	29.9	99.2	43
19AR113 Spot 199	201.7275	35182.33	8.381903	13.68022	0.930154	1.719553	1.232322	0.171295	0.653005	0.529898	1019.244	6.156215	1015.858	7.911901	1009.429	21.18498	1009.4	21.2	101.0	176
19AR113 Spot 591	188.3913	33608.36	2.246665	13.5787	0.889729	1.711681	1.117064	0.170475	0.576234	0.515847	1014.725	5.41022	1012.914	7.159778	1009.864	19.38615	1009.9	19.4	100.5	518
19AR113 Spot 315	232.06	41794.64	1.977744	13.59006	0.796418	1.602135	1.050991	0.15951	0.590198	0.561563	954.051	5.233938	971.0434	6.57059	1010.56	17.62483	1010.6	17.6	94.4	277
19AR113 Spot 281	434.2045	43055.75	3.3277	13.56139	0.692398	1.628539	1.045864	0.162104	0.775306	0.741307	968.4608	6.971742	981.2947	6.579528	1010.985	14.22277	1011.0	14.2	95.8	245
19AR113 Spot 233	438.1923	51729.56	5.763358	13.60906	0.681454	1.718457	1.014197	0.171034	0.683601	0.674032	1017.805	6.43626	1015.449	6.509901	1011.233	15.17903	1011.2	15.2	100.6	208
19AR113 Spot 212	51.92489	8658.062	6.707441	13.43293	2.205063	1.448503	2.848363	0.144163	0.874898	0.307158	868.1603	7.106268	909.252	17.11138	1011.276	54.9832	1011.3	55.0	85.8	188
19AR113 Spot 283	272.351	44434.07	7.048334	13.5597	0.718857	1.715001	1.068079	0.17059	0.696579	0.652179	1015.36	6.543902	1014.157	6.850692	1012.424	16.39969	1012.4	16.4	100.3	247
19AR113 Spot 192	222.9337	52749.3	1.007775	13.66489	0.920667	1.682572	1.180363	0.167336	0.671115	0.568566	997.4176	6.201675	1001.956	7.517542	1012.761	19.67507	1012.8	19.7	98.5	170
19AR113 Spot 241	454.4724	25701.18	15.81451	13.53955	0.501957	1.702095	0.887603	0.169268	0.537618	0.605697	1008.078	5.017109	1009.319	5.677231	1012.876	14.31524	1012.9	14.3	99.5	216
19AR113 Spot 89	151.6931	28958.81	2.356162	13.52499	0.915059	1.748195	1.205325	0.173649	0.740907	0.614695	1032.184	7.066687	1026.496	7.785469	1015.253	19.25	1015.3	19.3	101.7	82
19AR113 Spot 331	172.0806	31374.98	2.818851	13.60382	1.010975	1.699123	1.367428	0.168686	0.842474	0.616102	1004.868	7.838925	1008.201	8.740721	1016.315	21.82014	1016.3	21.8	98.9	293
19AR113 Spot 264	230.0767	50837.59	5.578796	13.58658	0.778433	1.752483	1.067519	0.173743	0.643315	0.602626	1032.702	6.138706	1028.079	6.90147	1019.116	17.25019	1019.1	17.3	101.3	230
19AR113 Spot 79	395.3815	51052.19	4.278045	13.51881	0.651782	1.72127	0.952835	0.170373	0.653747	0.686108	1014.168	6.13487	1016.499	6.119699	1022.384	14.02988	1022.4	14.0	99.2	72
19AR113 Spot 557	925.3441	8213.208	4.050892	13.31671	0.726623	1.476523	1.663247	0.146134	1.483402	0.891871	879.2529	12.19249	920.8059	10.06929	1022.585	15.22534	1022.6	15.2	86.0	486
19AR113 Spot 128	122.313	20115.81	2.067504	13.46968	1.099483	1.786444	1.479497	0.176755	0.678927	0.45889	1049.223	6.573962	1040.53	9.631546	1023.178	26.61176	1023.2	26.6	102.5	115
19AR113 Spot 576	372.406	58747.77	3.34101	13.56992	0.656762	1.732227	0.937966	0.171383	0.655363	0.698706	1019.725	6.181134	1020.579	6.038236	1023.274	13.5793	1023.3	13.6	99.7	505
19AR113 Spot 158	156.1079	34441.89	2.358082	13.48638	0.906789	1.760832	1.131143	0.17408	0.656339	0.580244	1034.553	6.273328	1031.154	7.325428	1024.814	18.63834	1024.8	18.6	101.0	137
19AR113 Spot 114	333.2807	8030.206	3.808211	13.2731	0.916209	1.448734	2.188178	0.143131	1.94471	0.888734	862.3413	15.69678	909.3476	13.14571	1026.151	20.31091	1026.2	20.3	84.0	104
19AR113 Spot 236	147.5273	32967.5	4.608491	13.47113	0.982939	1.730653	1.28949	0.170948	0.720494	0.558743	1017.333	6.780715	1019.994	8.298528	1026.568	21.64945	1026.6	21.6	99.1	211
19AR113 Spot 527	234.9763	23120.17	4.412483	13.43701	0.92806	1.712331	1.287548	0.169102	0.83034	0.6449	1007.163	7.742324	1013.158	8.253689	1027.001	19.92223	1027.0	19.9	98.1	464
19AR113 Spot 172	195.0722	47178.96	1.486164	13.50978	0.846014	1.778063	1.266452	0.175581	0.865906	0.683726	1042.786	8.337074	1037.472	8.230631	1027.152	18.71157	1027.2	18.7	101.5	150
19AR113 Spot 119	62.23863	14529.46	1.892699	13.42611	2.202994	1.770514	2.449298	0.174741	0.728018	0.297235	1038.18	6.980929	1034.709	15.89447	1028.242	47.29527	1028.2	47.3	101.0	107
19AR113 Spot 347	205.507	36947.91	3.205984	13.46386	1.036378	1.790168	1.300294	0.176586	0.775102	0.596097	1048.297	7.499111	1041.887	8.471202	1029.326	21.12787	1029.3	21.1	101.8	309
19AR113 Spot 214	596.8158	128256.4	2.208188	13.57085	0.693807	1.70261	0.99846	0.167885	0.705423	0.706511	1000.447	6.537015	1009.512	6.387014	1030.098	14.28451	1030.1	14.3	97.1	190
19AR113 Spot 71	188.5054	28714.85	2.991554	13.41775	1.114583	1.733927	1.300588	0.170927	0.629793	0.484237	1017.214	5.926469	1021.21	8.375745	1030.646	23.02149	1030.6	23.0	98.7	64
19AR113 Spot 293	62.62881	12710.24	3.104777	13.28523	2.509389	1.667885	2.970289	0.164061	0.977593	0.329124	979.3074	8.881923	996.3814	18.85721	1035.014	56.66973	1035.0	56.7	94.6	256
19AR113 Spot 277	288.3837	72104.49	4.03251	13.42298	0.756	1.801826	1.033079	0.177196	0.680188	0.658409	1051.638	6.600133	1046.12	6.745927	1035.474	15.72442	1035.5	15.7	101.6	241
19AR113 Spot 317	137.0422	28304.5	4.279243	13.39386	0.789322	1.725996	1.005805	0.169718	0.59676	0.593316	1010.558	5.581684	1018.261	6.466424	1035.718	16.37379	1035.7	16.4	97.6	279
19AR113 Spot 221	171.8017	35973.66	2.226592	13.46643	0.896132	1.796514	1.239081	0.17665	0.706729	0.570365	1048.645	6.839691	1044.193	8.082623	1035.749	20.57647	1035.7	20.6	101.2	197
19AR113 Spot 15	130.5139	19678.78	3.230122	13.38635	1.185412	1.768169	1.506136	0.173825	0.586209	0.389214	1033.149	5.596011	1033.849	9.768743	1036.189	28.04118	1036.2	28.0	99.7	15
19AR113 Spot 416	496.0613	101798.5	11.40661	13.53716	0.781426	1.673792	1.138803	0.164529	0.823131	0.722803	981.8942	7.496841	998.627	7.238688	1036.41	15.91338	1036.4	15.9	94.7	363
19AR113 Spot 577	124.5028	13631.12	4.669875	13.33682	1.095367	1.75577	1.404988	0.172568	0.783625	0.557745	1026.242	7.434431	1029.29	9.089474	1036.634	23.57117	1036.6	23.6	99.0	506
19AR113 Spot 400	162.8482	50580.59	2.340804	13.4465	1.004467	1.825238	1.324084	0.179305	0.780161	0.589208	1063.178	7.646619	1054.569	8.686016	1037.648	21.62325	1037.6	21.6	102.5	349
19AR113 Spot 171	166.2184	30469.4	2.348967	13.4083	0.845465	1.76426	1.175838	0.173289	0.807489	0.686735	1030.207	7.688133	1032.414	7.620251	1037.954	17.27772	1038.0	17.3	99.3	149
19AR113 Spot 39	401.1598	78370.52	4.34188	13.501	0.655801	1.741059	0.890629	0.170993	0.598246	0.671712	1017.582	5.631479	1023.856	5.744157	1038.152	13.34045	1038.2	13.3	98.0	36
19AR113 Spot 503	123.1781	25027.3	4.335445	13.44072	0.877022	1.751305	1.255366	0.171999	0.742246	0.591259	1023.117	7.022076	1027.644	8.113953	1038.155	20.46112	1038.2	20.5	98.6	441
19AR113 Spot 429	205.8628	48392.25	3.174247	13.44802	0.809901	1.791295	1.072811	0.175823	0.634206	0.591162	1044.113	6.113384	1042.296	6.990713	1039.349	17.48676	1039.3	17.5	100.5	375
19AR113 Spot 340	119.8278	29847.59	17.06509	13.40725	1.179231	1.819841	1.386412	0.178554	0.716264	0.516632	1059.068	6.995377	1052.628	9.085369	1040.152	23.98069	1040.2	24.0	101.8	302
19AR113 Spot 419	109.0999	31300.18	3.605702	13.45956	1.42591	1.816167	1.597695	0.178094	0.706889	0.442443	1056.551	6.888713	1051.304	10.46253	1041.28	28.936	1041.3	28.9	101.5	366
19AR113 Spot 449	217.6883	48295.04	3.709434	13.38493	0.840311	1.777952	1.136264	0.174215	0.666411	0.586493	1035.292	6.373796	1037.431	7.384348	1042.8	18.58894	1042.8	18.6	99.3	395
19AR113 Spot 559	163.8	24364.75	1.894069	13.38259	1.001043	1.806098	1.246014	0.176942	0.702766	0.564011	1050.248	6.810916	1047.667	8.143305	1043.149	20.77916	1043.1	20.8	100.7	488
19AR113 Spot 446	359.529	35886.97	3.25806	13.35719	0.58275	1.769324	0.974078	0.173301	0.717375	0.736466	1030.271	6.830549	1034.273	6.319209	1043.601	13.31254	1043.6	13.3	98.7	392
19AR113 Spot 244	442.248	106075.5	22.93924	13.40109	0.506539	1.783099	0.741808	0.174648	0.53732	0.724339	1037.671	5.150003	1039.311	4.825822	1043.62	10.33632	1043.6	10.3	99.4	219
19AR113 Spot 3	114.2391	33262.73	3.872561	13.39069	1.237654	1.783678	1.421773	0.174684	0.670062	0.471286	1037.869	6.423417	1039.522	9.250596	1043.858	25.31731	1043.9	25.3	99.4	3
19AR113 Spot 582	327.3472	59630.83	10.45033	13.41994	0.791346	1.679139	1.077345	0.164439	0.674501	0.626077	981.4002	6.140306	1000.655	6.856188	1043.943	16.96661	1043.9	17.0	94.0	511
19AR113 Spot 191	409.2419	12362.36	3.584271	13.2772	0.712812	1.760309	1.054848	0.172101	0.746112	0.707317	1023.674	7.062193	1030.962	6.83058	1047.351	15.05363	1047.4	15.1	97.7	169
19AR113 Spot 230	344.7885	60684.51	1.55228	13.36355	0.587286	1.796269	0.820228	0.175365	0.562907	0.686281	1041.603	5.414088	1044.104	5.350089	1050.238	12.0423	1050.2	12.0	99.2	205
19AR113 Spot 462	127.1881	28410.19	3.692348	13.31105	0.718247	1.803586	0.978443	0.176073	0.607848	0.62124	1045.487	5.866409	1046.758	6.391373	1050.307	15.47177	1050.3	15.5	99.5	407
19AR113 Spot 433	119.9959	25099.92	2.985975	13.31379	0.904519	1.807032	1.175273	0.176406	0.655281	0.557557	1047.313	6.334367	1048.005	7.682376	1050.344	19.68297	1050.3	19.7	99.7	379
19AR113 Spot 124	124.028	30267.95	1.374613	13.33193	1.328321	1.788616	1.542596	0.174508	0.748904	0.485483	1036.901	7.17305	1041.321	10.04673	1051.505	27.18246	1051.5	27.2	98.6	112
19AR113 Spot 381	77.52901	12973.09	2.896204	13.17781	1.422581	1.874132	1.728025	0.182778	0.793785	0.45936	1082.132	7.907541	1071.991	11.44173	1052.318	30.92767	1052.3	30.9	102.8	332
19AR113 Spot 168	158.3303	48938.5	5.204475	13.34278	0.87509	1.821142	1.264966	0.177576	0.906903	0.716939	1053.72	8.816067	1053.096	8.291578	1052.7	17.78549	1052.7	17.8	100.1	146
19AR113 Spot 38	74.98197	11398.46	2.185585	13.22208	1.633307	1.753971	1.88993	0.170803	0.634381	0.335664	1016.534	5.965955	1028.628	12.22249	1055.334	35.85708	1055.3	35.9	96.3	35
19AR113 Spot 501	177.8179	44541.95	5.108545	13.35032	0.842385	1.819006	1.154868	0.17709	0.758587	0.656861	1051.055	7.357114	1052.327	7.566731	1055.863	17.55423	1055.9	17.6	99.5	439
19AR113 Spot 472	23.32295	5777.845	1.612197	12.94106	4.965336	1.780992	5.16819	0.173332	0.946399	0.18312	1030.444	9.012613	1038.542	33.61935	1056.52	102.3683	1056.5	102.4	97.5	416
19AR113 Spot 6	270.207	54222.15	3.017425	13.32655	0.818828	1.819725	1.150517	0.176964	0.762419	0.662675	1050.366	7.389816	1052.586	7.539282	1058.089	17.36475	1058.1	17.4	99.3	6
19AR113 Spot 27	15.49443	2612.888	3.045585	12.53155	6.727721	1.717742	7.462558	0.166982	1.120606	0.150164	995.4611	10.33658	1015.181	47.92804	1058.863	148.7003	1058.9	148.7	94.0	27
19AR113 Spot 553	194.0672	39347.09	6.994178	13.32398	0.795905	1.768089	1.090225	0.171872	0.640281	0.587292	1022.417	6.053595	1033.82	7.070938	1058.913	17.77997	1058.9	17.8	96.6	482
19AR113 Spot 562	38.52814	9565.592	4.392275	13.11867	3.382194	1.929105	3.7828	0.187471	0.9349	0.247145	1107.659	9.51468	1091.229	25.30196	1059.486	73.77708	1059.5	73.8	104.5	491
19AR113 Spot 514	173.6762	40975.27	3.125531	13.30722	1.136232	1.763995	1.496929	0.171388	0.912265	0.609424	1019.754	8.604365	1032.317	9.700727	1059.924	23.88648	1059.9	23.9	96.2	451
19AR113 Spot 9	203.0764	45124.59	4.057556	13.25742	1.063831	1.795185	1.303352	0.173725	0.73618	0.564836	1032.605	7.024242	1043.71	8.499637	1067.929	21.61615	1067.9	21.6	96.7	9
19AR113 Spot 363	227.508	54367.05	2.636884	13.20571	0.845839	1.867579	1.145661	0.180396	0.762163	0.66526	1069.138	7.508709	1069.674	7.576306	1071.66	17.18413	1071.7	17.2	99.8	315
19AR113 Spot 180	89.58274	28146.44	2.912257	13.20417	1.326783	1.816164	1.59163	0.17539	0.858192	0.539191	1041.74	8.255161	1051.303	10.4228	1072.116	26.92076	1072.1	26.9	97.2	158
19AR113 Spot 393	257.7993	73603.68	2.885802	13.19669	0.806763	1.887283	1.109656	0.182037	0.751779	0.677488	1078.096	7.463426	1076.627	7.365015	1074.546	16.37115	1074.5	16.4	100.3	343
19AR113 Spot 219	208.242	36463.97	4.644529	13.20592	0.82892	1.888167	1.098008	0.182037	0.669751	0.609969	1078.092	6.649049	1076.938	7.288881	1075.496	17.45123	1075.5	17.5	100.2	195
19AR113 Spot 469	637.0107	134607.4	9.120722	13.2155	0.54583	1.814124	0.872624	0.174809	0.677995	0.776962	1038.551	6.503402	1050.567	5.711961	1076.525	11.00927	1076.5	11.0	96.5	414
19AR113 Spot 414	146.5089	29765.08	4.475446	13.1757	0.967356	1.89708	1.201019	0.1823	0.670367	0.558165	1079.526	6.663298	1080.066	7.985717	1082.01	19.98957	1082.0	20.0	99.8	361
19AR113 Spot 581	17.89796	3245.488	5.616641	12.44669	6.709915	1.625358	7.69066	0.155624	1.882464	0.244773	932.4106	16.342	980.0651	48.38185	1089.302	149.6177	1089.3	149.6	85.6	510
19AR113 Spot 107	600.9278	30647.25	2.720049	13.09825	0.615933	1.885602	0.974422	0.180452	0.74666	0.766259	1069.444	7.357919	1076.035	6.465418	1090.267	12.54038	1090.3	12.5	98.1	99
19AR113 Spot 11	80.67958	16489.63	2.851286	12.99894	1.435862	1.898107	1.714326	0.181618	0.787377	0.459293	1075.808	7.801589	1080.426	11.40113	1090.607	30.50776	1090.6	30.5	98.6	11
19AR113 Spot 305	19.25894	1203.425	0.980941	11.35061	4.299935	1.904872	8.14116	0.182085	0.890867	0.109428	1078.353	8.846185	1082.794	54.25854	1092.593	162.3064	1092.6	162.3	98.7	268
19AR113 Spot 20	66.08087	9918.402	2.129117	12.9116	1.475276	1.915235	1.992662	0.182564	0.632163	0.317245	1080.969	6.291272	1086.409	13.29343	1098.186	37.81779	1098.2	37.8	98.4	20
19AR113 Spot 566	163.8876	36956.34	5.600027	13.05222	0.88359	1.973484	1.237401	0.188029	0.854188	0.690309	1110.687	8.715037	1106.498	8.33908	1099.147	17.93099	1099.1	17.9	101.0	495
19AR113 Spot 377	434.2061	86974.81	1.258451	13.03238	0.786686	1.915568	1.161385	0.182461	0.852183	0.733765	1080.405	8.476853	1086.525	7.747985	1099.706	15.78626	1099.7	15.8	98.2	328
19AR113 Spot 125	43.92018	8396.307	1.915603	12.78629	2.965985	1.925569	3.476065	0.183003	0.836651	0.240689	1083.359	8.343248	1090.003	23.23499	1104.171	67.49055	1104.2	67.5	98.1	113
19AR113 Spot 234	133.012	29337.97	4.223683	12.9519	1.21816	1.924107	1.466167	0.182813	0.790463	0.539136	1082.324	7.87573	1089.495	9.796313	1104.738	24.69723	1104.7	24.7	98.0	209
19AR113 Spot 231	101.5966	13219.49	3.223203	12.84066	1.930588	1.600434	2.106567	0.151752	0.699685	0.332144	910.7771	5.942869	970.3797	13.16502	1108.764	39.70079	1108.8	39.7	82.1	206
19AR113 Spot 145	146.0122	35069.02	6.054863	12.92783	0.924223	1.998029	1.205699	0.189275	0.688768	0.571261	1117.445	7.06646	1114.845	8.159133	1110.63	19.77567	1110.6	19.8	100.6	130
19AR113 Spot 592	27.93094	7425.966	6.134604	12.66388	3.566174	1.992134	3.872918	0.188491	0.976243	0.252069	1113.194	9.980922	1112.846	26.18798	1113.018	74.87419	1113.0	74.9	100.0	519
19AR113 Spot 204	284.2275	13534.48	2.279248	12.86444	0.722373	1.946268	1.044261	0.183892	0.655341	0.627564	1088.204	6.562016	1097.161	7.004485	1115.861	16.21777	1115.9	16.2	97.5	181
19AR113 Spot 494	21.04181	4124.98	2.452102	12.38606	4.828688	1.784498	5.0896	0.168014	1.220248	0.239753	1001.158	11.31524	1039.821	33.13117	1122.9	98.57091	1122.9	98.6	89.2	434
19AR113 Spot 468	75.58077	22738.1	1.47538	12.78768	1.21818	2.069076	1.532989	0.194284	0.719352	0.469248	1144.541	7.543792	1138.627	10.4943	1128.259	26.96943	1128.3	27.0	101.4	413
19AR113 Spot 97	182.698	51291.96	1.629475	12.78706	0.799058	2.055277	1.088984	0.192306	0.729048	0.669475	1133.857	7.580188	1134.051	7.438388	1135.272	16.09975	1135.3	16.1	99.9	89
19AR113 Spot 459	301.536	12618.56	2.221304	12.57712	0.588027	2.086549	0.903125	0.193921	0.654501	0.724707	1142.583	6.85297	1144.391	6.199244	1148.667	12.34154	1148.7	12.3	99.5	404
19AR113 Spot 464	189.9182	52227.18	3.709105	12.68447	0.721577	2.085697	1.043205	0.19328	0.732488	0.702151	1139.118	7.648263	1144.111	7.15986	1154.434	14.73265	1154.4	14.7	98.7	409
19AR113 Spot 29	48.52551	10092.53	1.361304	12.58816	2.626651	2.16397	2.738899	0.200442	0.672849	0.245664	1177.695	7.242419	1169.546	19.02285	1155.335	52.69756	1155.3	52.7	101.9	29
19AR113 Spot 207	135.77	35364.73	3.921159	12.66554	0.726061	2.147005	1.060094	0.198178	0.695318	0.655902	1165.526	7.413724	1164.087	7.343742	1162.256	15.87044	1162.3	15.9	100.3	183
19AR113 Spot 422	78.75478	23844	3.388545	12.63207	1.925448	2.175306	2.124116	0.200473	0.789781	0.371816	1177.863	8.502163	1173.178	14.77657	1165.386	39.07079	1165.4	39.1	101.1	369
19AR113 Spot 499	156.567	45562.13	4.339322	12.59523	0.851338	2.129373	1.17426	0.195656	0.728264	0.620189	1151.944	7.68236	1158.382	8.113307	1171.326	18.2536	1171.3	18.3	98.3	437
19AR113 Spot 500	143.1378	28334.61	2.749208	12.56687	0.994292	2.138958	1.244219	0.196414	0.727188	0.584453	1156.028	7.695843	1161.488	8.609027	1172.565	19.98299	1172.6	20.0	98.6	438
19AR113 Spot 22	331.478	72731.52	3.265716	12.56315	0.699814	2.153192	0.946688	0.1965	0.614747	0.649366	1156.493	6.508271	1166.081	6.564102	1184.812	14.2234	1184.8	14.2	97.6	22
19AR113 Spot 45	79.6425	27531.45	2.067572	12.43804	1.343315	2.2677	1.614483	0.206645	0.666755	0.412984	1210.917	7.36088	1202.301	11.37692	1187.731	29.05507	1187.7	29.1	102.0	40
19AR113 Spot 318	51.3077	12719.74	0.688059	12.25655	1.709779	2.371717	2.171087	0.214514	0.71922	0.331272	1252.824	8.189057	1234.119	15.50789	1202.465	40.373	1202.5	40.4	104.2	280
19AR113 Spot 23	81.8595	16286.7	1.207346	12.34109	1.392667	2.22638	1.700393	0.201126	0.691244	0.40652	1181.364	7.461543	1189.38	11.9147	1204.82	30.60512	1204.8	30.6	98.1	23
19AR113 Spot 411	264.8508	112202.4	3.467875	12.37047	0.766361	2.140064	1.095544	0.192509	0.768	0.701022	1134.955	7.992267	1161.845	7.581514	1213.196	15.35485	1213.2	15.4	93.6	358
19AR113 Spot 265	116.0819	20209.38	1.14136	12.20752	1.075184	2.432786	1.292328	0.217736	0.681203	0.527113	1269.902	7.851859	1252.345	9.299767	1223.127	21.57333	1223.1	21.6	103.8	231
19AR113 Spot 14	145.1971	42320.29	4.22069	12.18863	0.856202	2.374905	1.075012	0.211383	0.612221	0.569502	1236.182	6.886768	1235.079	7.681325	1234.027	17.32731	1234.0	17.3	100.2	14
19AR113 Spot 530	39.49542	7887.63	3.732293	11.90772	2.470183	2.4097	2.640171	0.214101	0.746942	0.282914	1250.629	8.491204	1245.494	18.94782	1237.495	49.6423	1237.5	49.6	101.1	467
19AR113 Spot 334	163.3365	50608.89	1.972961	12.16907	0.794825	2.38026	1.032264	0.210794	0.644972	0.624813	1233.049	7.238494	1236.688	7.380789	1243.877	15.79513	1243.9	15.8	99.1	296
19AR113 Spot 541	155.761	45189.09	1.558735	12.01832	0.924882	2.408121	1.214088	0.212115	0.778403	0.641143	1240.077	8.781146	1245.023	8.710717	1254.421	18.21442	1254.4	18.2	98.9	476
19AR113 Spot 202	151.2417	33609.27	3.890633	11.82491	0.894343	2.105507	1.122738	0.181427	0.670321	0.597042	1074.763	6.635832	1150.609	7.729312	1297.301	17.51008	1297.3	17.5	82.8	179
19AR113 Spot 196	107.2594	27318.5	4.651366	11.39797	1.05881	2.804386	1.323239	0.232963	0.701909	0.530447	1350.01	8.549407	1356.709	9.904559	1368.103	21.59305	1368.1	21.6	98.7	173
19AR113 Spot 590	8.484951	1060.841	1.245209	9.893879	24.53369	1.104752	28.05733	0.091733	3.971073	0.141534	565.7803	21.5099	755.6458	150.6294	1368.931	545.246	1368.9	545.2	41.3	517
19AR113 Spot 371	74.48011	17785.39	3.695891	11.16694	1.516386	2.85008	1.855919	0.234094	0.715094	0.385305	1355.919	8.744266	1368.832	13.95094	1389.86	32.87039	1389.9	32.9	97.6	322
19AR113 Spot 451	79.57025	33591.57	2.653863	11.09261	1.213707	2.79429	1.453549	0.226998	0.669291	0.460453	1318.746	7.981999	1354.01	10.86969	1410.963	24.69178	1411.0	24.7	93.5	397
19AR113 Spot 594	74.21345	27720.82	2.953829	11.04905	0.814155	3.056053	1.083976	0.247726	0.572164	0.527838	1426.736	7.323023	1421.75	8.29309	1415.105	17.60821	1415.1	17.6	100.8	521
19AR113 Spot 185	176.4811	37712.83	3.663523	11.01329	0.819537	3.129011	1.153239	0.250953	0.792417	0.687122	1443.388	10.24763	1439.852	8.874043	1435.444	15.9806	1435.4	16.0	100.6	163
19AR113 Spot 301	102.1331	31381.7	2.401683	10.77033	1.099667	3.241956	1.42842	0.255695	0.881276	0.616959	1467.776	11.56826	1467.253	11.08521	1467.305	21.34921	1467.3	21.3	100.0	264
19AR113 Spot 232	77.37996	23755.69	0.941942	10.70297	1.403313	3.217008	1.557808	0.25233	0.645878	0.414607	1450.481	8.389171	1461.264	12.06735	1477.782	26.88479	1477.8	26.9	98.2	207
19AR113 Spot 415	43.03482	11893.57	1.6727	10.64719	2.191478	3.118728	2.536256	0.243687	0.84293	0.332352	1405.838	10.64712	1437.32	19.50252	1485.032	45.32427	1485.0	45.3	94.7	362
19AR113 Spot 325	11.25498	1033.089	3.153161	9.279987	19.70011	0.954893	22.9224	0.073565	3.379423	0.147429	457.5966	14.92805	680.6471	114.1685	1511.77	433.6638	1511.8	433.7	30.3	287
19AR113 Spot 385	43.503	10287.16	2.915246	10.35868	1.24913	3.55542	1.632923	0.272165	0.682072	0.4177	1551.784	9.406726	1539.643	12.94141	1523.812	27.96383	1523.8	28.0	101.8	336
19AR113 Spot 571	114.0663	6130.575	2.345878	9.617776	0.749347	3.874564	1.1266	0.276436	0.638674	0.566904	1573.388	8.916478	1608.398	9.092812	1655.339	17.19403	1655.3	17.2	95.0	500
19AR113 Spot 596	333.1706	140284	5.45263	9.364069	0.774855	3.667222	1.340449	0.250764	1.093192	0.815542	1442.417	14.12882	1564.263	10.69486	1733.624	14.22923	1733.6	14.2	83.2	523

19AR113 Spot 600	144.0372	68321.81	0.994883	9.102849	0.495636	4.715754	0.78139	0.313807	0.592182	0.757857	1759.414	9.118113	1770.042	6.546082	1783.38	9.292993	1783.4	9.3	98.7	526
19AR113 Spot 297	173.8645	68537.44	2.949274	9.027934	0.839087	4.730935	1.206101	0.311905	0.864917	0.717118	1750.075	13.25602	1772.736	10.10996	1800.307	15.29432	1800.3	15.3	97.2	260
19AR113 Spot 61	104.7854	33317.98	1.271272	9.019272	0.817177	4.213591	1.060193	0.277712	0.630488	0.594691	1579.828	8.833987	1676.67	8.700437	1800.865	15.50711	1800.9	15.5	87.7	55
19AR113 Spot 328	254.9289	64030.44	0.665727	8.987436	0.61733	4.103563	0.982476	0.268283	0.763147	0.776759	1532.081	10.40647	1655.012	8.021368	1815.556	11.2352	1815.6	11.2	84.4	290
19AR113 Spot 160	48.11099	25445.58	2.097085	8.789598	0.953768	4.656165	1.219484	0.299737	0.694455	0.569466	1690.004	10.32398	1759.401	10.19358	1843.605	18.14108	1843.6	18.1	91.7	139
19AR113 Spot 569	93.23915	24910.67	1.879748	8.823971	0.754433	5.022342	1.051297	0.323223	0.630485	0.599721	1805.45	9.928012	1823.096	8.902407	1844.089	15.22294	1844.1	15.2	97.9	498
19AR113 Spot 341	122.2578	51399.48	2.307021	8.793701	0.743494	5.086789	1.014573	0.326011	0.670209	0.660582	1819.018	10.62219	1833.904	8.609525	1851.617	13.77043	1851.6	13.8	98.2	303
19AR113 Spot 487	62.08765	31316.26	1.132782	8.749829	1.055356	5.175177	1.263956	0.330894	0.682664	0.540102	1842.711	10.94135	1848.543	10.75607	1855.881	19.22221	1855.9	19.2	99.3	427
19AR113 Spot 564	60.03057	27481.87	1.184662	8.714762	1.02333	5.336387	1.33402	0.339224	0.824244	0.617865	1882.933	13.45884	1874.711	11.40818	1866.379	18.93026	1866.4	18.9	100.9	493
19AR113 Spot 558	127.2689	51415.93	1.656605	8.731475	0.645632	5.207629	0.928713	0.33103	0.66034	0.711026	1843.374	10.58683	1853.865	7.911058	1866.424	11.78662	1866.4	11.8	98.8	487
19AR113 Spot 447	148.3227	72262.92	2.751356	8.65169	0.637097	5.231938	0.887707	0.330703	0.610147	0.687329	1841.788	9.774841	1857.834	7.567405	1876.609	11.62327	1876.6	11.6	98.1	393
19AR113 Spot 413	221.4756	62709.91	5.68993	8.696117	0.677662	5.222794	0.965837	0.329784	0.681265	0.705361	1837.335	10.89138	1856.343	8.231158	1878.472	12.33691	1878.5	12.3	97.8	360
19AR113 Spot 453	167.1028	63095.57	0.790819	8.639726	0.652274	5.340445	1.061695	0.337183	0.831505	0.783186	1873.104	13.51632	1875.361	9.080273	1878.63	11.89542	1878.6	11.9	99.7	399
19AR113 Spot 169	141.0263	43596.14	0.962066	8.644759	0.696386	5.248951	1.057609	0.331203	0.787746	0.744837	1844.21	12.63441	1860.602	9.020522	1879.737	12.71444	1879.7	12.7	98.1	147
19AR113 Spot 387	156.5148	55295.98	1.567722	8.590349	0.561248	5.504383	0.867649	0.345283	0.64702	0.745716	1912.036	10.70528	1901.281	7.455631	1890.331	10.40183	1890.3	10.4	101.1	338
19AR113 Spot 247	191.9589	15106.65	2.327568	8.507907	0.63418	5.164898	1.007879	0.322567	0.629232	0.624313	1802.251	9.893078	1846.851	8.574023	1898.235	14.15438	1898.2	14.2	94.9	222
19AR113 Spot 87	47.96504	10642.55	1.368413	8.252499	0.868694	5.512172	1.343662	0.335206	0.763065	0.5679	1863.567	12.34935	1902.496	11.54876	1945.988	19.76946	1946.0	19.8	95.8	80
19AR113 Spot 456	108.313	33928.32	2.606322	8.231142	0.638833	5.720826	1.006871	0.3446	0.766346	0.761117	1908.762	12.66095	1934.519	8.702626	1962.983	11.65066	1963.0	11.7	97.2	401
19AR113 Spot 37	129.3659	24014.74	0.729796	8.237983	0.624425	5.76858	0.975602	0.345712	0.69309	0.710423	1914.089	11.47811	1941.708	8.44275	1972.061	12.23603	1972.1	12.2	97.1	34
19AR113 Spot 215	210.3552	86930.97	2.994571	8.225094	0.613006	5.961222	0.909168	0.356172	0.662079	0.728226	1964.001	11.20917	1970.204	7.905556	1977.482	11.09886	1977.5	11.1	99.3	191
19AR113 Spot 457	171.2807	70383.67	2.409764	8.034892	0.561017	6.078544	0.845092	0.356582	0.62883	0.744097	1965.954	10.6553	1987.174	7.368808	2010.079	10.01878	2010.1	10.0	97.8	402
19AR113 Spot 112	130.3305	56393.52	28.04662	8.054248	0.756553	6.083465	1.071913	0.356415	0.752642	0.702148	1965.158	12.74884	1987.88	9.34775	2012.348	13.53838	2012.3	13.5	97.7	103
19AR113 Spot 339	184.0321	75519.89	0.773704	7.763545	0.708119	6.556632	0.975316	0.370633	0.662109	0.678866	2032.378	11.54174	2053.537	8.592872	2075.596	12.61108	2075.6	12.6	97.9	301
19AR113 Spot 170	46.54599	16542.51	2.085897	7.573309	0.810312	6.992813	1.161884	0.38785	0.813369	0.700043	2112.847	14.65303	2110.517	10.3219	2108.997	14.55633	2109.0	14.6	100.2	148
19AR113 Spot 442	47.87192	36884.17	2.002743	7.583971	0.83069	7.012787	1.100008	0.388836	0.718444	0.653127	2117.427	12.96663	2113.051	9.77566	2109.546	14.61295	2109.5	14.6	100.4	388
19AR113 Spot 105	94.15335	41105.66	1.788182	7.601916	0.713285	7.029126	0.92755	0.389103	0.590229	0.636331	2118.668	10.65785	2115.12	8.245363	2112.417	12.54807	2112.4	12.5	100.3	97
19AR113 Spot 182	71.07898	23059.53	2.198651	7.48987	0.887393	6.481309	1.254512	0.354187	0.840305	0.669826	1954.559	14.16803	2043.365	11.03588	2134.982	16.29511	2135.0	16.3	91.5	160
19AR113 Spot 476	142.7347	66140.21	2.489304	7.432673	0.600138	7.229958	0.830917	0.391968	0.568243	0.683874	2131.95	10.31511	2140.205	7.411962	2148.882	10.58788	2148.9	10.6	99.2	418
19AR113 Spot 438	30.82633	18041.72	1.199572	7.341873	1.064015	7.418121	1.354509	0.398657	0.678642	0.501024	2162.852	12.46945	2163.159	12.12024	2164.193	20.44106	2164.2	20.4	99.9	384
19AR113 Spot 1	54.27847	17885.72	2.343598	5.92326	0.702926	8.929253	1.038997	0.385876	0.760317	0.73178	2103.675	13.647	2330.797	9.487581	2536.82	11.87805	2536.8	11.9	82.9	1
19AR113 Spot 106	110.043	55036.39	3.443654	5.88056	0.582608	10.79654	0.849898	0.461811	0.608011	0.715392	2447.55	12.38235	2505.769	7.898333	2553.999	9.941437	2554.0	9.9	95.8	98
19AR113 Spot 516	230.7249	64016.03	2.477022	5.685172	0.838018	11.28314	1.267001	0.466698	0.948885	0.748922	2469.064	19.46383	2546.812	11.81809	2610.035	13.98055	2610.0	14.0	94.6	453
19AR113 Spot 238	146.2978	100932.6	3.347964	5.3836	0.630093	13.03602	0.883226	0.511661	0.617629	0.699287	2663.718	13.47642	2682.263	8.329381	2696.975	10.42588	2697.0	10.4	98.8	213
19AR113 Spot 489	33.4913	26491.63	1.835549	5.10425	0.768729	15.31953	1.097751	0.570534	0.753217	0.686146	2910.013	17.639	2835.318	10.46374	2783.344	13.08275	2783.3	13.1	104.6	429
19AR113 Spot 488	35.47844	33170.23	2.260429	5.105079	0.717281	14.70523	0.954868	0.547345	0.625241	0.654794	2814.12	14.25741	2796.359	9.078464	2784.269	11.82193	2784.3	11.8	101.1	428
19AR113 Spot 375	205.1903	156379.9	12.42832	4.713605	0.545546	14.69711	0.898223	0.505433	0.713514	0.794361	2637.103	15.44272	2795.834	8.539585	2913.132	8.838035	2913.1	8.8	90.5	326
19AR113 Spot 460	379.109	133414.2	2.744976	4.251757	0.515962	16.77876	0.879449	0.519921	0.712146	0.809765	2698.845	15.7038	2922.277	8.427693	3080.684	8.240153	3080.7	8.2	87.6	405
River Samples																				
**19AR105**																				
19AR105_Spot 433	96.94162	19.54853	0.194803	2.8238	10.75893	-0.06567	102.7481	4.35E-05	43.071	0.41919	0.280321	0.120734	NA	-73.4559	NA	NA	0.3	0.1		376
19AR105_Spot 579	855.3861	176.975	1.71986	5.353447	23.51208	0.001957	52.60261	0.000128	40.80421	0.775707	0.825996	0.337019	1.985334	1.043317	1812.934	621.3289	0.8	0.3	0.045561	500
19AR105_Spot 455	837.3833	167.5082	1.196501	3.179242	61.1588	0.004561	78.73713	0.000133	48.38419	0.614503	0.855819	0.414054	4.621039	3.630222	3180.21	NA	0.9	0.4	0.026911	396
19AR105_Spot 174	863.0073	97.97279	1.070875	8.020203	8.720483	-0.00101	276.91	0.000167	41.10653	0.148447	1.074211	0.441534	NA	-2.84488	NA	NA	1.1	0.4		150
19AR105_Spot 341	484.9747	169.6723	1.843124	2.602966	24.52153	0.01277	49.02831	0.000282	42.11434	0.85898	1.820608	0.766629	12.8839	6.276931	3607.858	392.4251	1.8	0.8	0.050462	294
19AR105_Spot 153	3350.457	959.4544	3.316237	5.923005	23.55849	0.006437	26.74389	0.000299	11.79002	0.440849	1.928816	0.227374	6.515351	1.736882	2413.637	414.2197	1.9	0.2	0.079913	130
19AR105_Spot 12	1658.203	453.4934	3.118365	7.999265	24.63929	0.005323	36.72162	0.00041	11.81945	0.321866	2.640896	0.312075	5.390865	1.97437	1513.401	677.5505	2.6	0.3	0.174501	12
19AR105_Spot 351	283.7022	195.3855	1.001783	4.039028	54.53624	0.014886	62.1662	0.000582	28.60687	0.460168	3.752742	1.07323	15.004	9.259098	2702.566	1005.775	3.8	1.1	0.138858	302
19AR105_Spot 225	1204.321	691.0268	2.473641	4.907584	39.42669	0.015273	40.38233	0.000593	8.255467	0.204433	3.822762	0.315493	15.39079	6.168371	2714.367	683.0116	3.8	0.3	0.140834	192
19AR105_Spot 408	308.7359	398.2268	1.550838	3.86537	53.11223	0.020056	64.74093	0.000632	35.94567	0.555223	4.071802	1.463175	20.16286	12.92557	3054.229	947.8522	4.1	1.5	0.133317	355
19AR105_Spot 32	285.3698	230.7048	2.077158	3.663084	29.81337	0.022961	40.28074	0.000748	25.85221	0.641801	4.817714	1.24502	23.05019	9.180445	3001.286	510.7188	4.8	1.2	0.160522	32
19AR105_Part2_Spot 164	2373.288	2666.867	4.186683	11.38736	16.38948	0.008605	17.70387	0.000758	3.636677	0.205417	4.883197	0.177519	8.700043	1.533666	1254.858	341.383	4.9	0.2	0.389143	648
19AR105_Part2_Spot 106	788.4818	556.5159	2.087833	4.480058	17.72817	0.025149	19.85187	0.000902	8.644431	0.435447	5.810648	0.502071	25.22054	4.945119	2845.275	293.8268	5.8	0.5	0.204221	601
19AR105_Spot 6	291.4426	341.84	1.140808	3.305379	20.96261	0.036462	31.18593	0.000976	22.36064	0.71701	6.288618	1.405489	36.36439	11.14035	3312.232	345.5135	6.3	1.4	0.18986	6
19AR105_Spot 8	556.2516	1081.016	0.47038	5.10814	21.73265	0.045834	24.30712	0.001806	9.184569	0.377855	11.6297	1.067175	45.50384	10.81691	2690.817	377.2846	11.6	1.1	0.4322	8
19AR105_Part2_Spot 257	378.2976	687.3505	1.558795	7.857437	40.91515	0.02805	41.86528	0.001905	7.201993	0.172028	12.26791	0.882694	28.08891	11.59884	1746.236	791.9696	12.3	0.9	0.702535	730
19AR105_Spot 289	642.9642	1083.88	0.670432	7.048059	18.66946	0.034294	19.92593	0.001917	5.220476	0.261994	12.34839	0.644028	34.23742	6.708488	2094.913	341.2939	12.3	0.6	0.589446	248
19AR105_Spot 124	500.9608	1199.96	1.001521	8.477395	23.51714	0.02836	24.75996	0.001919	6.353132	0.256589	12.36011	0.784502	28.39529	6.933373	1752.654	444.5976	12.4	0.8	0.705223	107
19AR105_Spot 383	352.0837	836.6377	1.688934	6.615745	12.15647	0.036087	18.36338	0.001926	10.27459	0.559515	12.40266	1.273098	35.99688	6.494533	2176.419	266.6893	12.4	1.3	0.569865	331
19AR105_Part2_Spot 232	1096.831	1723.323	0.96456	11.02678	11.63829	0.022022	13.65575	0.001945	3.751686	0.274733	12.5236	0.46939	22.11808	2.987739	1249.579	258.0946	12.5	0.5	1.002226	709
19AR105_Spot 362	1342.05	1002.011	2.616921	8.596385	8.63711	0.02861	10.65791	0.002012	3.530053	0.331214	12.95509	0.456862	28.64273	3.010072	1682.327	186.1491	13.0	0.5	0.77007	310
19AR105_Spot 544	937.8697	1807.102	2.86989	8.582063	17.79266	0.030911	18.8239	0.002036	3.698725	0.196491	13.11426	0.484567	30.91133	5.7311	1801.66	338.7302	13.1	0.5	0.727899	474
19AR105_Spot 203	1234.394	2704.794	10.25364	12.8629	12.22075	0.031276	12.71334	0.00312	2.899094	0.228036	20.0843	0.581357	31.27109	3.915023	1006.312	252.0651	20.1	0.6	1.995833	175
19AR105_Part2_Spot 211	142.8407	585.4783	2.628447	6.32159	19.85849	0.077113	21.95153	0.004118	6.891827	0.313957	26.49129	1.821988	75.42677	15.95862	2175.244	367.2814	26.5	1.8	1.217854	693
19AR105_Part2_Spot 77	215.9849	819.9399	1.463753	8.4595	28.24903	0.069447	28.7837	0.004958	4.279582	0.148681	31.88298	1.36109	68.17463	18.98112	1654.105	538.7981	31.9	1.4	1.927506	574
19AR105_Spot 412	904.8849	22464.05	3.034083	18.52803	2.477721	0.19789	2.804249	0.02691	0.936606	0.333995	171.1781	1.582164	183.3392	4.703881	344.0865	59.81018	171.2	1.6	49.74857	359
19AR105_Spot 299	100.2719	4412.768	0.873617	12.77388	8.383246	0.407433	8.840623	0.039395	2.019116	0.228391	249.0792	4.933278	347.0249	25.99178	1069.658	173.2809	249.1	4.9	23.28586	257
19AR105_Part2_Spot 65	732.7604	30539.39	3.074123	17.24661	1.557779	0.371934	1.857501	0.046955	0.835699	0.449905	295.7984	2.416128	321.0859	5.113229	509.8013	36.4605	295.8	2.4	58.02228	566
19AR105_Part2_Spot 184	168.0421	9269.471	2.009924	16.77178	3.970409	0.429344	4.531014	0.053627	0.905359	0.199814	336.752	2.970549	362.711	13.82042	533.1998	97.27692	336.8	3.0	63.15682	667
19AR105_Spot 380	484.178	31762.34	2.3366	18.02933	2.128046	0.474197	2.334788	0.062407	0.700649	0.300091	390.2498	2.653161	394.084	7.625862	417.594	49.75713	390.2	2.7	93.45195	328
19AR105_Spot 269	21.65012	143.253	11.21222	7.036798	11.60545	0.341797	21.2246	0.064898	2.864641	0.134968	405.3467	11.2542	298.5324	54.95086	NA	NA	405.3	11.3		230
19AR105_Part2_Spot 97	400.8479	24223.15	390.8499	17.90179	2.235352	0.511251	2.396654	0.067284	0.706092	0.294616	419.7729	2.86954	419.2898	8.232699	417.5845	51.18243	419.8	2.9	100.5241	592
19AR105_Part2_Spot 355	108.6516	2659.994	0.961416	15.68121	6.302596	0.558075	6.504922	0.069656	0.84849	0.130438	434.0852	3.561898	450.2728	23.66218	534.7746	141.3132	434.1	3.6	81.17163	812
19AR105_Part2_Spot 24	322.7436	1606.315	0.95951	15.06898	2.769649	0.555364	3.435175	0.070149	1.075993	0.313228	437.0556	4.546814	448.5044	12.45507	508.6031	71.7527	437.1	4.5	85.93254	533
19AR105_Spot 523	206.979	1831.022	152.4422	14.56335	3.918333	0.604841	4.189179	0.071719	1.004753	0.239845	446.5015	4.334398	480.301	16.0326	646.0207	87.4229	446.5	4.3	69.11566	457
19AR105_Spot 417	333.3077	13086.02	2.59143	16.9522	3.758263	0.587383	4.189272	0.073465	0.801313	0.191277	456.9961	3.535183	469.1949	15.74138	530.297	90.11621	457.0	3.5	86.17739	362
19AR105_Part2_Spot 110	223.5872	11042.31	2.187207	17.13754	3.292249	0.579403	3.752323	0.073472	1.094183	0.291601	457.0367	4.82766	464.078	13.97802	500.0526	79.06161	457.0	4.8	91.39773	604
19AR105_Spot 162	56.76867	5376.945	1.361588	13.43504	10.20277	0.729416	10.81208	0.073652	1.580934	0.146219	458.1209	6.991229	556.2106	46.33577	981.9553	218.4277	458.1	7.0	46.65394	139
19AR105_Part2_Spot 365	100.0769	4043.822	1.544419	14.37087	6.373841	0.66663	7.49885	0.073692	0.858482	0.114482	458.3583	3.79829	518.6614	30.46496	794.8288	156.3989	458.4	3.8	57.66755	820
19AR105_Spot 287	31.97513	2486.653	2.342719	12.42926	11.71568	0.761978	12.91501	0.073917	2.042807	0.158173	459.7122	9.064008	575.1506	56.77	1063.073	257.5087	459.7	9.1	43.2437	246
19AR105_Spot 52	87.9564	8277.914	1.066589	13.11657	5.666077	0.75911	6.042743	0.074053	1.015643	0.168076	460.5263	4.514139	573.4965	26.48343	1051.791	120.1374	460.5	4.5	43.78497	48
19AR105_Spot 161	197.1838	15799.74	0.760653	17.34775	2.574369	0.581962	2.977472	0.074326	0.89636	0.301047	462.1633	3.997635	465.7216	11.12228	484.2845	62.70603	462.2	4.0	95.43218	138
19AR105_Spot 572	123.8138	10268.32	1.433687	15.47851	4.708756	0.653959	5.007862	0.074405	0.915454	0.182803	462.6409	4.08686	510.912	20.10781	734.1221	104.3011	462.6	4.1	63.01961	493
19AR105_Spot 583	90.51747	6919.722	0.938989	15.63915	5.712919	0.642722	6.216601	0.074563	1.182628	0.190237	463.5861	5.290009	503.99	24.70175	692.7589	130.2302	463.6	5.3	66.91882	503
19AR105_Spot 77	153.7989	11768.42	0.999493	15.55001	5.829436	0.647495	6.242549	0.074577	1.865595	0.298851	463.6695	8.346435	506.936	24.91675	708.1233	126.8	463.7	8.3	65.47864	69
19AR105_Spot 84	106.8922	5876.678	1.56272	15.0373	5.520041	0.657887	6.088025	0.074582	1.281349	0.21047	463.702	5.732979	513.3204	24.53506	741.7302	126.0032	463.7	5.7	62.51626	74
19AR105_Spot 406	213.9536	16537.16	2.523868	16.76097	2.390003	0.606547	2.816983	0.074634	0.959416	0.340583	464.0113	4.295354	481.3802	10.79945	565.9437	57.67239	464.0	4.3	81.98895	353
19AR105_Spot 278	184.0583	14301.39	0.924902	16.30098	3.909238	0.620552	4.279454	0.074681	0.951767	0.222404	464.2952	4.263623	490.1935	16.64074	614.0346	90.16929	464.3	4.3	75.61384	238
19AR105_Spot 205	102.0403	7735.834	0.926347	15.29959	6.490681	0.65616	6.758982	0.074699	1.2784	0.189141	464.4011	5.7281	512.2624	27.19709	732.9043	140.7392	464.4	5.7	63.36449	177
19AR105_Spot 370	149.2149	7831.151	1.494756	15.58433	3.55148	0.644862	3.671014	0.074719	0.795154	0.216603	464.5265	3.563758	505.3122	14.61448	695.4048	76.39865	464.5	3.6	66.79944	318
19AR105_Spot 130	128.0322	9596.267	0.832601	17.96857	5.276054	0.558936	6.052201	0.074725	1.046354	0.172888	464.5604	4.689928	450.8339	22.03664	382.371	134.0829	464.6	4.7	121.4947	113
19AR105_Spot 164	174.5088	15234.28	0.959927	17.27729	4.161021	0.588624	4.395714	0.074754	0.932924	0.212235	464.7326	4.183013	469.9885	16.5392	496.7094	94.68616	464.7	4.2	93.56227	141
19AR105_Spot 93	126.3905	11325.38	1.922791	17.21937	4.460656	0.585505	4.935612	0.074763	0.796044	0.161286	464.7893	3.569693	467.993	18.50896	484.7207	107.6237	464.8	3.6	95.88807	82
19AR105_Part2_Spot 60	152.3763	11579.92	0.957514	16.94247	4.504602	0.593951	4.659111	0.074871	0.885216	0.189997	465.4349	3.974883	473.3876	17.63	513.0964	100.5524	465.4	4.0	90.711	562
19AR105_Spot 549	186.7866	11067.29	1.564194	17.76528	3.36135	0.570725	4.240587	0.07491	1.034352	0.243917	465.6689	4.6468	458.4836	15.6465	423.5829	91.79215	465.7	4.6	109.9357	478
19AR105_Spot 236	210.0432	14475.29	0.916979	17.04748	3.910725	0.602024	4.055175	0.074923	1.008196	0.24862	465.7479	4.530035	478.5176	15.47455	541.1586	85.91161	465.7	4.5	86.06495	202
19AR105_Spot 596	284.4493	13217	1.00024	17.61116	3.321379	0.578387	3.512436	0.074943	0.622063	0.177103	465.8655	2.795741	463.4242	13.06975	452.2884	76.79514	465.9	2.8	103.0019	514
19AR105_Part2_Spot 391	75.47975	1978.471	1.533395	16.75616	4.656263	0.536731	5.831288	0.074962	1.130951	0.193945	465.9806	5.084047	436.2669	20.68299	283.3271	130.9503	466.0	5.1	164.4673	840
19AR105_Spot 138	43.64616	3138.433	1.710178	12.21965	8.920657	0.801833	10.09065	0.075106	2.191083	0.21714	466.8441	9.867343	597.8619	45.62578	1133.132	196.5249	466.8	9.9	41.19945	118
19AR105_Spot 372	235.1201	16276.43	1.664518	17.86428	2.325446	0.573013	2.565936	0.075122	0.805901	0.314077	466.9385	3.630006	459.9614	9.491189	426.2106	54.34185	466.9	3.6	109.5558	320
19AR105_Spot 338	95.68434	7304.44	1.629719	15.45742	5.10117	0.649346	5.81386	0.075185	1.022622	0.175894	467.3168	4.609781	508.0762	23.24528	696.9466	122.0276	467.3	4.6	67.05202	291
19AR105_Spot 156	116.0534	6022.849	1.533195	15.12762	7.20124	0.662697	7.658411	0.075212	1.191706	0.155607	467.4807	5.373792	516.2622	31.00311	739.3454	160.2784	467.5	5.4	63.229	133
19AR105_Spot 575	168.2789	19862.65	1.048705	16.72707	3.676244	0.616035	3.827317	0.075284	0.894767	0.233784	467.912	4.038386	487.3589	14.81527	580.8073	80.8324	467.9	4.0	80.56235	496
19AR105_Spot 298	78.85092	5491.793	1.170861	12.24557	10.25994	0.821121	10.57426	0.075305	1.410628	0.133402	468.0388	6.368308	608.6736	48.44822	1175.083	207.886	468.0	6.4	39.83028	256
19AR105_Spot 258	151.5359	12724.06	1.130725	17.86572	3.902966	0.570104	4.122078	0.075312	1.152665	0.279632	468.0773	5.20414	458.0815	15.19862	409.1979	88.54078	468.1	5.2	114.389	220
19AR105_Spot 96	69.26427	4912.174	1.290136	11.45762	7.815076	0.87724	8.180258	0.075367	1.101914	0.134704	468.4115	4.97843	639.491	38.83356	1303.025	157.703	468.4	5.0	35.94801	85
19AR105_Spot 169	145.399	4518.531	1.602235	14.95422	6.456794	0.666866	7.459418	0.075505	0.999244	0.133957	469.2369	4.522237	518.8054	30.31112	744.3878	156.4616	469.2	4.5	63.03663	145
19AR105_Spot 440	146.9909	7425.531	1.197199	17.56879	4.641233	0.576201	5.033125	0.075517	0.826064	0.164126	469.3114	3.739058	462.0174	18.6844	426.865	110.7829	469.3	3.7	109.9438	383
19AR105_Part2_Spot 30	158.7858	12355.17	1.150774	16.28918	4.382938	0.628535	4.48745	0.07556	0.764094	0.170273	469.5679	3.460379	495.1829	17.58755	616.3562	95.53877	469.6	3.5	76.18451	537
19AR105_Spot 571	236.674	23115.99	0.873555	17.33136	3.423638	0.599981	3.585455	0.075597	0.797802	0.222511	469.7868	3.614657	477.2214	13.65284	514.0724	76.83203	469.8	3.6	91.38535	492
19AR105_Part2_Spot 354	257.3613	26376.69	0.856459	17.54108	2.859677	0.586467	2.950451	0.075605	0.672481	0.227925	469.8382	3.047181	468.6091	11.07511	463.5792	63.69279	469.8	3.0	101.3501	811
19AR105_Part2_Spot 39	92.43921	5796.085	0.826919	16.09568	6.267351	0.619622	6.475147	0.075633	1.150507	0.17768	470.0021	5.214988	489.6106	25.15832	583.3596	138.4742	470.0	5.2	80.56816	543
19AR105_Spot 141	238.7891	13975.63	0.823529	16.96138	3.062364	0.606451	3.205117	0.075714	0.873907	0.27266	470.4892	3.965181	481.3196	12.28636	534.1907	67.50374	470.5	4.0	88.07513	121
19AR105_Spot 16	130.5127	8922.589	0.992612	15.51075	6.483536	0.655475	6.729823	0.075732	0.854892	0.12703	470.5941	3.879739	511.8422	27.06262	701.5222	142.2633	470.6	3.9	67.08186	16
19AR105_Spot 184	127.927	8788.53	1.019885	15.89759	5.645569	0.64704	5.956175	0.07582	0.952844	0.159976	471.1255	4.328978	506.6554	23.76312	671.3403	125.8725	471.1	4.3	70.17685	157
19AR105_Spot 145	171.9823	14134.33	1.149133	17.68914	2.968141	0.581074	3.281503	0.075841	0.920284	0.280446	471.248	4.182098	465.1517	12.24625	436.1161	70.1496	471.2	4.2	108.0556	124
19AR105_Spot 392	102.913	8276.597	2.097867	15.33834	5.511678	0.664072	5.797549	0.075845	1.236616	0.2133	471.2749	5.619931	517.1018	23.49606	725.982	120.2143	471.3	5.6	64.91551	339
19AR105_Spot 385	103.0367	7186.845	1.868071	15.09162	7.633703	0.673329	7.912016	0.075857	1.418108	0.179235	471.3446	6.445661	522.7344	32.33775	754.9432	164.5309	471.3	6.4	62.43445	333
19AR105_Spot 182	206.045	16100.96	0.643013	16.75059	2.397478	0.620146	2.564762	0.075873	0.654325	0.255121	471.4434	2.974676	489.9387	9.968519	578.3018	53.91564	471.4	3.0	81.52204	156
19AR105_Spot 463	141.289	9367.886	2.15211	15.86119	3.991866	0.64552	4.179677	0.075883	0.835737	0.199953	471.5028	3.799866	505.7183	16.65015	664.5706	87.73409	471.5	3.8	70.94849	404
19AR105_Spot 127	292.844	21020.65	0.955475	17.44667	2.120781	0.595818	2.318981	0.075884	0.854701	0.368567	471.5061	3.886116	474.5764	8.79161	490.4037	47.57626	471.5	3.9	96.14652	110
19AR105_Spot 158	105.6023	9097.741	1.21752	16.14015	5.725655	0.632386	6.316387	0.075893	1.290869	0.204368	471.5594	5.869905	497.5809	24.85104	620.0667	133.5226	471.6	5.9	76.04978	135
19AR105_Spot 326	213.986	14538.35	1.547564	16.4683	3.928706	0.623443	4.015296	0.075894	0.771758	0.192205	471.5674	3.509436	492.0032	15.6582	589.219	85.49609	471.6	3.5	80.03262	280
19AR105_Spot 95	78.06186	6538.071	1.431847	13.44385	8.452073	0.75568	8.863479	0.075909	1.847127	0.208398	471.6594	8.401068	571.5148	38.75588	992.5082	176.567	471.7	8.4	47.52197	84
19AR105_Spot 252	93.69578	5561.503	1.872203	15.84153	4.969991	0.636516	6.118933	0.075914	1.159296	0.18946	471.6885	5.273	500.1468	24.17	633.5293	129.4494	471.7	5.3	74.45408	215
19AR105_Spot 361	78.32193	5623.971	1.523357	15.02853	9.80245	0.672836	10.10602	0.075931	1.473418	0.145796	471.7894	6.703152	522.4355	41.29577	751.3341	211.6276	471.8	6.7	62.79356	309
19AR105_Spot 447	311.964	27113.54	2.369574	18.13375	2.761103	0.573785	2.920691	0.075932	0.801156	0.274303	471.7945	3.644805	460.4593	10.81274	405.2482	62.87881	471.8	3.6	116.4211	389
19AR105_Spot 404	171.5607	12782.23	0.899791	16.04317	4.245313	0.642459	4.41032	0.075933	0.963526	0.218471	471.8004	4.383551	503.8276	17.51839	652.9628	92.39578	471.8	4.4	72.25532	351
19AR105_Spot 185	153.8594	9622.23	1.674811	16.95474	4.292063	0.608089	5.096321	0.075951	0.935346	0.183534	471.9078	4.256283	482.3541	19.5703	533.2585	109.7774	471.9	4.3	88.49513	158
19AR105_Part2_Spot 274	58.42823	3536.826	1.460337	13.97213	8.996798	0.704349	9.426173	0.075952	2.001406	0.212324	471.9161	9.107533	541.3851	39.57441	846.6822	191.9408	471.9	9.1	55.7371	742
19AR105_Spot 190	175.885	11829.94	0.781381	16.67369	2.736162	0.618427	2.861815	0.075968	0.694995	0.242851	472.0124	3.16324	488.8607	11.10411	569.582	60.44537	472.0	3.2	82.86996	163
19AR105_Spot 376	156.7254	15526.16	1.224298	16.98879	3.672819	0.6089	3.950313	0.075985	0.980119	0.248112	472.111	4.461869	482.8664	15.18137	535.2073	83.79233	472.1	4.5	88.21088	324
19AR105_Spot 274	148.1858	9601.43	2.049616	16.35911	5.375938	0.623791	5.623604	0.075998	0.867058	0.154182	472.189	3.947802	492.2204	21.93921	587.4864	120.6371	472.2	3.9	80.37446	234
19AR105_Part2_Spot 209	123.2665	7162.586	1.609251	15.83955	2.864059	0.640613	3.731587	0.076004	1.12262	0.300842	472.2242	5.11177	502.6858	14.79599	644.7984	76.47792	472.2	5.1	73.23595	691
19AR105_Part2_Spot 270	228.6145	11494.07	1.469775	17.66954	2.455954	0.576487	2.732073	0.076009	0.970978	0.3554	472.2594	4.421596	462.2012	10.14462	413.4723	57.11267	472.3	4.4	114.2179	739
19AR105_Spot 91	86.84912	5352.269	1.399289	15.55511	4.707024	0.645744	5.099859	0.076033	1.703069	0.333944	472.4028	7.757634	505.8563	20.32096	661.0668	103.0719	472.4	7.8	71.46068	80
19AR105_Part2_Spot 194	163.0329	7390.261	1.15136	16.7755	3.532293	0.60576	3.679058	0.076062	0.646934	0.175842	472.576	2.947882	480.8828	14.09336	521.6198	79.48405	472.6	2.9	90.59778	677
19AR105_Spot 457	130.6994	8750.137	1.901966	14.63267	6.194334	0.700023	6.774345	0.076063	1.898205	0.280205	472.581	8.64964	538.8045	28.33135	830.7997	135.7144	472.6	8.6	56.88266	398
19AR105_Spot 476	210.0607	11289.89	2.297265	18.46973	3.522428	0.558557	3.676432	0.076076	0.915551	0.249032	472.6565	4.172573	450.5866	13.37908	340.4725	80.62136	472.7	4.2	138.8237	416
19AR105_Spot 290	217.5241	19339.88	2.461368	17.68448	3.650069	0.585508	3.921876	0.076082	0.94737	0.241561	472.6939	4.317919	467.9951	14.70682	445.9585	84.63854	472.7	4.3	105.995	249
19AR105_Spot 390	165.1733	15665.27	0.915537	17.13318	3.974011	0.603008	4.079502	0.076083	0.807172	0.19786	472.6991	3.678961	479.1411	15.58327	511.0262	87.92403	472.7	3.7	92.49996	337
19AR105_Spot 340	183.1071	15705.5	2.379022	16.33788	4.420062	0.632146	4.585683	0.0761	0.782633	0.170669	472.8014	3.567862	497.432	18.03591	613.3596	97.67234	472.8	3.6	77.08389	293
19AR105_Spot 369	153.1664	11045.8	1.044305	16.28588	3.115924	0.633407	3.307094	0.076117	1.000589	0.302558	472.9024	4.562416	498.2157	13.02232	617.1834	68.06436	472.9	4.6	76.62268	317
19AR105_Part2_Spot 277	98.03678	7785.364	2.214024	13.79103	7.568942	0.738967	7.679159	0.076121	1.105855	0.144007	472.9262	5.042647	561.8024	33.14602	941.1789	155.9379	472.9	5.0	50.24828	745
19AR105_Spot 167	147.3178	11621.99	1.228419	15.99019	4.862508	0.645393	5.105088	0.076121	0.843265	0.165181	472.9274	3.845259	505.6395	20.33507	657.4325	108.0152	472.9	3.8	71.93551	143
19AR105_Part2_Spot 253	143.3907	10124.73	1.813891	17.56215	3.703909	0.579442	3.88617	0.076123	0.812123	0.208978	472.9377	3.703328	464.1027	14.47729	421.5682	84.85786	472.9	3.7	112.1853	727
19AR105_Part2_Spot 346	233.7239	24980.05	1.101616	17.59214	2.682799	0.589951	2.803651	0.076136	0.67318	0.240108	473.0167	3.070234	470.8365	10.56335	461.1671	60.34556	473.0	3.1	102.5695	803
19AR105_Spot 531	71.82254	5216.209	1.348645	12.70621	8.461281	0.802216	9.046264	0.076136	1.354814	0.149765	473.0181	6.179047	598.0778	40.90896	1106.916	179.0896	473.0	6.2	42.73297	465
19AR105_Part2_Spot 370	85.24897	7501.374	1.687326	14.34463	5.672531	0.706178	5.93243	0.076141	1.068009	0.180029	473.0459	4.871257	542.4742	24.93674	846.9344	121.4666	473.0	4.9	55.85391	823
19AR105_Spot 288	172.1552	13013.74	2.611924	16.76722	2.942773	0.614439	3.217951	0.076159	0.848743	0.263753	473.158	3.872057	486.3557	12.43621	549.9969	67.76745	473.2	3.9	86.02921	247
19AR105_Spot 261	213.9358	23123.81	1.677851	17.87148	3.277573	0.581831	3.507543	0.076169	0.83673	0.238552	473.2176	3.817714	465.6372	13.10067	429.3765	75.94809	473.2	3.8	110.2104	223
19AR105_Spot 90	275.7976	18448.27	1.501673	18.40183	2.046897	0.562622	2.229597	0.076187	0.759149	0.340487	473.3213	3.464472	453.2318	8.151331	353.5681	47.36066	473.3	3.5	133.8699	79
19AR105_Spot 503	217.9573	19385.58	8.364834	17.04273	3.733288	0.611244	3.85577	0.076188	0.761496	0.197495	473.3261	3.475214	484.3443	14.85338	537.7707	82.70722	473.3	3.5	88.01635	439
19AR105_Spot 561	112.5685	6917.93	1.490691	16.38714	6.268918	0.623833	6.457058	0.076188	1.021141	0.158143	473.3301	4.66019	492.2467	25.19304	582.2087	138.5823	473.3	4.7	81.29905	485

19AR105_Part2_Spot 294	120.5514	8633.308	2.525942	15.20751	3.92821	0.671122	4.260061	0.076217	0.89712	0.210589	473.5009	4.095617	521.3941	17.37326	738.0007	88.16111	473.5	4.1	64.15995	758
19AR105_Part2_Spot 179	160.289	12601.69	1.278885	17.20813	4.318367	0.598673	4.523942	0.076219	1.06428	0.235255	473.5126	4.858866	476.3913	17.20357	491.2151	97.03435	473.5	4.9	96.39618	662
19AR105_Part2_Spot 333	196.9227	9730.298	0.75507	17.24707	2.792656	0.591077	3.572073	0.076219	0.724601	0.202852	473.513	3.308098	471.5551	13.47499	463.0246	77.55949	473.5	3.3	102.2652	793
19AR105_Spot 197	138.4069	8921.054	1.170353	16.4677	5.323881	0.623063	5.43937	0.076222	0.735836	0.13528	473.53	3.359504	491.7651	21.20499	578.5423	117.1781	473.5	3.4	81.84881	170
19AR105_Part2_Spot 399	211.7671	16902.32	1.474782	16.36353	2.661023	0.628917	3.054309	0.076239	0.650502	0.212979	473.6354	2.970547	495.4208	11.9745	598.3618	64.64092	473.6	3.0	79.15536	848
19AR105_Spot 371	148.9758	13014.69	1.270258	16.78036	4.730242	0.61757	4.888441	0.076261	0.797895	0.163221	473.7665	3.644591	488.323	18.95282	558.1776	105.2252	473.8	3.6	84.87737	319
19AR105_Spot 61	142.266	8030.824	0.952467	15.04003	4.13728	0.679053	4.305734	0.076269	0.769821	0.17879	473.8167	3.516718	526.2017	17.68317	761.3567	89.35546	473.8	3.5	62.23321	56
19AR105_Spot 563	55.24171	2959.714	1.611315	12.16245	8.692014	0.817909	9.691707	0.076271	1.332412	0.13748	473.8242	6.086856	606.8812	44.30355	1142.014	191.2775	473.8	6.1	41.49024	486
19AR105_Spot 400	193.7467	11070.27	0.889662	16.31901	4.390295	0.630687	4.646887	0.076279	0.846111	0.182081	473.8767	3.865698	496.5236	18.25081	603.2693	98.91144	473.9	3.9	78.55144	347
19AR105_Part2_Spot 320	76.36668	7039.746	1.25103	15.5559	6.790139	0.65146	7.189931	0.076312	1.309744	0.182164	474.0695	5.986286	509.3771	28.80654	672.1275	151.4318	474.1	6.0	70.53267	781
19AR105_Spot 319	83.80428	5713.566	1.458781	15.42359	7.858434	0.65404	8.441175	0.07632	1.048001	0.124153	474.1167	4.790429	510.9617	33.90411	680.3122	179.2264	474.1	4.8	69.69105	274
19AR105_Spot 420	118.2799	9926.173	1.51965	17.19989	3.704861	0.599586	4.53374	0.076322	1.135217	0.250393	474.1334	5.18927	476.9708	17.25727	491.5778	96.85906	474.1	5.2	96.45135	365
19AR105_Spot 149	166.2256	14153.18	1.156282	16.92204	3.319312	0.611972	3.663543	0.076325	0.834652	0.227827	474.1495	3.815463	484.8031	14.12321	536.4233	78.07454	474.1	3.8	88.39093	128
19AR105_Spot 428	205.4384	14946.01	1.063254	17.53126	4.06301	0.59401	4.177569	0.076336	0.827952	0.19819	474.2167	3.78535	473.4253	15.80853	470.5768	90.66105	474.2	3.8	100.7735	372
19AR105_Spot 546	202.8104	17062.43	1.421476	16.9239	1.907977	0.617926	2.167997	0.07637	0.818389	0.377486	474.417	3.74315	488.5466	8.407682	556.3193	43.77966	474.4	3.7	85.27782	476
19AR105_Spot 48	247.0209	21354.67	1.179019	17.27647	2.832252	0.601426	3.12324	0.076377	0.821623	0.263068	474.4601	3.758273	478.1383	11.91051	496.7596	66.40476	474.5	3.8	95.511	45
19AR105_Spot 336	115.5743	9354.51	1.921909	16.71265	4.494752	0.613398	4.706575	0.076383	1.202717	0.25554	474.4953	5.50187	485.7011	18.17114	539.8999	99.58996	474.5	5.5	87.8858	289
19AR105_Spot 165	52.65611	4974.618	1.539312	14.43328	14.62891	0.701725	15.00329	0.076406	2.051569	0.136741	474.633	9.387598	539.821	62.89977	826.5317	311.6842	474.6	9.4	57.42466	142
19AR105_Spot 554	72.68485	4900.079	1.395328	14.99934	6.391216	0.676814	7.702572	0.076411	1.376535	0.178711	474.6653	6.299181	524.8468	31.57839	750.47	160.2899	474.7	6.3	63.24908	480
19AR105_Spot 459	327.857	21196.96	3.121808	17.32335	1.715421	0.601755	2.046946	0.076417	0.85697	0.418658	474.7	3.921864	478.3468	7.808516	496.8265	40.96085	474.7	3.9	95.54642	400
19AR105_Part2_Spot 311	199.9473	11366.45	0.823994	17.20855	3.052341	0.59531	3.291244	0.076417	1.055067	0.320568	474.7032	4.828475	474.2531	12.47125	473.06	69.00682	474.7	4.8	100.3473	774
19AR105_Part2_Spot 14	216.0251	18480.91	1.128109	17.40874	2.959645	0.595756	3.130024	0.076439	0.714213	0.228181	474.8298	3.269408	474.537	11.86587	474.1062	67.43917	474.8	3.3	100.1526	527
19AR105_Spot 377	188.6481	20487.05	1.161182	17.01824	3.058996	0.613895	3.252262	0.076443	0.903474	0.277799	474.8583	4.13602	486.0139	12.56194	539.9371	68.33702	474.9	4.1	87.94698	325
19AR105_Spot 314	81.87303	8702.116	2.15103	13.51812	8.048231	0.760772	8.196243	0.076444	1.386955	0.169218	474.8602	6.349374	574.4553	35.97309	991.8728	164.4906	474.9	6.3	47.87511	270
19AR105_Part2_Spot 360	139.3858	8840.195	1.091776	15.25759	6.117033	0.672455	6.219745	0.076446	0.70282	0.112998	474.872	3.217534	522.2038	25.39813	735.8921	130.9588	474.9	3.2	64.53012	816
19AR105_Spot 176	124.6194	11636.63	1.28116	16.17152	5.234494	0.644301	5.512091	0.076462	0.705194	0.127936	474.9728	3.22906	504.9654	21.93417	644.1961	117.5511	475.0	3.2	73.73109	151
19AR105_Spot 456	63.05913	7383.803	1.28485	13.048	9.323278	0.788884	9.566028	0.076482	1.746072	0.182528	475.0876	7.997074	590.5388	42.85983	1064.273	189.5766	475.1	8.0	44.63963	397
19AR105_Part2_Spot 186	188.8429	11828.84	1.022262	17.03135	2.742544	0.606418	3.322114	0.076495	0.827673	0.24914	475.166	3.791372	481.2986	12.73447	511.5949	70.72645	475.2	3.8	92.87936	669
19AR105_Part2_Spot 393	248.5424	14642.88	0.806764	16.75396	3.012897	0.615424	3.47454	0.076512	0.595369	0.171352	475.2722	2.727831	486.9753	13.44128	543.3995	74.82844	475.3	2.7	87.46275	842
19AR105_Spot 405	185.133	21989.39	0.885765	16.498	3.767692	0.634422	3.860075	0.076541	0.72764	0.188504	475.442	3.335012	498.847	15.21501	608.6437	81.97937	475.4	3.3	78.115	352
19AR105_Spot 51	156.1852	12260.2	1.752352	16.54135	3.79669	0.622654	3.950395	0.076544	0.95546	0.241864	475.463	4.379371	491.5098	15.39305	567.9685	83.45825	475.5	4.4	83.7129	47
19AR105_Spot 438	95.53409	5846.969	1.229045	15.15862	6.296819	0.674555	7.087527	0.076545	0.985448	0.13904	475.4648	4.516836	523.4783	28.99751	739.7144	148.6746	475.5	4.5	64.2768	381
19AR105_Spot 348	142.3395	11949.07	0.912337	16.75094	4.054814	0.617895	4.507369	0.076548	0.902583	0.200246	475.486	4.137202	488.527	17.48076	551.091	96.43774	475.5	4.1	86.28086	299
19AR105_Spot 209	114.5333	6088.369	1.230609	15.01096	9.355058	0.679416	9.53347	0.076554	1.332921	0.139815	475.5195	6.110174	526.4215	39.18086	754.6352	199.6675	475.5	6.1	63.01316	180
19AR105_Spot 99	199.8604	12246.36	0.815312	16.80116	3.37983	0.616544	3.573927	0.076554	1.054394	0.295024	475.5213	4.833407	487.6788	13.84137	546.1423	74.61865	475.5	4.8	87.06912	88
19AR105_Spot 129	91.51656	6764.869	1.366382	15.23743	6.038944	0.671776	6.348793	0.076566	0.963858	0.151818	475.5954	4.419049	521.7914	25.90965	730.4072	133.1091	475.6	4.4	65.11373	112
19AR105_Spot 163	180.7948	15683.18	1.918961	17.11956	3.121347	0.608862	3.61451	0.076569	1.141182	0.315722	475.6113	5.232204	482.8424	13.89014	518.2554	75.32911	475.6	5.2	91.7716	140
19AR105_Spot 315	191.5438	13981.06	1.437759	17.16926	3.240794	0.602685	3.66697	0.076578	0.869435	0.237099	475.6644	3.986701	478.9361	14.00254	495.5908	78.5374	475.7	4.0	95.97926	271
19AR105_Spot 135	215.4792	17796.01	0.892703	16.53623	4.605509	0.630715	4.725921	0.07658	0.886448	0.187571	475.6785	4.064826	496.541	18.56179	594.8618	100.6592	475.7	4.1	79.96455	116
19AR105_Part2_Spot 293	337.8654	25664.88	2.063793	17.18938	1.777259	0.60706	1.987992	0.076581	0.696421	0.350314	475.6841	3.193491	481.7043	7.625219	511.4378	40.92692	475.7	3.2	93.00917	757
19AR105_Part2_Spot 265	251.1235	17739.94	0.977211	17.18404	3.321877	0.602144	3.51795	0.076586	0.975683	0.277344	475.7148	4.474346	478.5933	13.4259	493.3764	74.55426	475.7	4.5	96.42026	735
19AR105_Part2_Spot 379	99.40827	6648.646	1.502751	13.18127	3.661477	0.775885	4.166433	0.076604	1.305934	0.313442	475.8178	5.990083	583.1336	18.48523	1027.516	80.02488	475.8	6.0	46.3076	831
19AR105_Part2_Spot 384	126.5403	11849.31	1.202679	16.04189	5.087049	0.641434	5.463824	0.07661	1.208042	0.221098	475.8561	5.541497	503.1937	21.68309	630.4562	114.858	475.9	5.5	75.47806	834
19AR105_Spot 7	136.3751	9241.362	2.23135	15.77946	4.634885	0.651487	4.992598	0.076626	1.118108	0.223953	475.9535	5.129965	509.3932	20.0006	663.3643	104.292	476.0	5.1	71.74844	7
19AR105_Part2_Spot 263	285.4857	26816.28	2.362017	17.4394	3.131567	0.596479	3.390116	0.076634	0.928842	0.273985	476.001	4.262009	474.9965	12.86173	471.1323	72.18384	476.0	4.3	101.0334	733
19AR105_Part2_Spot 67	180.3666	11490.07	1.305777	16.66341	3.646046	0.620372	3.802817	0.076662	0.84086	0.221115	476.1662	3.859592	490.0805	14.7844	556.6013	80.92394	476.2	3.9	85.54889	568
19AR105_Spot 364	56.1334	3859.146	1.453519	14.91768	6.550755	0.672088	7.574055	0.076662	1.37485	0.181521	476.1694	6.310679	521.9814	30.92144	728.7442	158.0855	476.2	6.3	65.34109	312
19AR105_Spot 54	197.7894	13302.24	0.814845	15.7111	4.257882	0.660154	4.445536	0.076683	0.868117	0.195278	476.2934	3.985728	514.7082	17.95131	690.0735	93.01815	476.3	4.0	69.02068	50
19AR105_Part2_Spot 167	451.0492	13007.07	107.5548	17.01335	2.08334	0.608536	2.245129	0.076696	0.781587	0.348125	476.3694	3.588999	482.6361	8.62456	513.4517	46.24548	476.4	3.6	92.77783	651
19AR105_Part2_Spot 241	227.9084	16826.76	1.061926	17.02193	3.003667	0.60998	3.14284	0.076728	0.632253	0.201172	476.5615	2.904394	483.5477	12.09116	517.7363	67.62362	476.6	2.9	92.04714	717
19AR105_Part2_Spot 273	432.5862	31845.67	3.016305	17.64288	2.225156	0.592399	2.397846	0.076734	0.731852	0.305212	476.5992	3.36218	472.3984	9.057846	452.9891	50.69835	476.6	3.4	105.2121	741
19AR105_Spot 507	136.388	8623.658	2.433619	17.1229	4.375647	0.604122	4.810343	0.076747	1.013447	0.210681	476.6765	4.656577	479.8464	18.39674	495.9794	103.6742	476.7	4.7	96.10812	443
19AR105_Spot 527	122.8022	20170.61	1.994198	15.93147	3.609789	0.661843	3.725958	0.07675	0.879415	0.236024	476.6922	4.040859	515.7405	15.06831	693.6691	77.2059	476.7	4.0	68.7204	461
19AR105_Spot 146	116.2135	758.2591	1.164247	12.45109	7.4663	0.648966	8.301209	0.076763	0.817541	0.098485	476.77	3.75714	507.8424	33.18457	651.2384	177.6209	476.8	3.8	73.20975	125
19AR105_Spot 432	137.3503	10349.51	1.794731	15.27838	5.624443	0.68183	5.720321	0.076777	0.786187	0.137438	476.8573	3.613687	527.8801	23.55171	755.9699	119.6477	476.9	3.6	63.07888	375
19AR105_Part2_Spot 219	252.2539	25662.04	2.212721	17.76602	2.687833	0.589059	2.881293	0.076782	0.788683	0.273725	476.8891	3.625392	470.2664	10.84557	439.0196	61.70265	476.9	3.6	108.6259	700
19AR105_Spot 250	90.1457	6368.428	0.967311	14.26959	9.014736	0.724162	9.201066	0.076791	1.584926	0.172255	476.9375	7.286251	553.1211	39.25927	881.4404	187.8486	476.9	7.3	54.10888	213
19AR105_Part2_Spot 323	258.7407	18309	1.701695	17.58063	2.487164	0.591222	2.595556	0.076825	0.678904	0.261564	477.1458	3.122383	471.6481	9.792505	445.933	55.69139	477.1	3.1	106.9994	784
19AR105_Part2_Spot 155	79.53271	6160.658	1.542012	13.494	7.149171	0.757001	7.819817	0.076855	0.986859	0.1262	477.325	4.540358	572.2786	34.2228	970.8228	158.4479	477.3	4.5	49.16706	640
19AR105_Part2_Spot 216	387.5946	24443.44	1.900202	17.73341	2.202013	0.589976	2.408711	0.076861	0.731205	0.303567	477.3586	3.364365	470.8524	9.075487	440.2101	51.09002	477.4	3.4	108.4388	697
19AR105_Part2_Spot 93	55.89935	4497.506	1.5279	12.63417	8.692926	0.805269	9.476817	0.076918	1.706612	0.180083	477.7023	7.85779	599.7967	42.94874	1094.099	187.0383	477.7	7.9	43.66169	588
19AR105_Spot 497	253.1805	21587.71	1.359372	16.83184	3.244671	0.626831	3.46928	0.076931	0.8531	0.245901	477.78	3.928559	494.12	13.57386	571.5021	73.19508	477.8	3.9	83.60074	433
19AR105_Part2_Spot 348	179.3705	14986.21	1.909026	16.93032	4.245396	0.615719	4.454837	0.07695	0.622775	0.139798	477.8942	2.868566	487.1605	17.23932	531.9583	96.65048	477.9	2.9	89.83677	805
19AR105_Part2_Spot 94	150.3442	9484.38	0.910027	16.34172	4.061623	0.630745	4.283615	0.076952	0.883724	0.206303	477.9015	4.070581	496.5598	16.82472	584.4546	91.02213	477.9	4.1	81.7688	589
19AR105_Spot 589	105.8608	5575.05	3.81133	15.02046	8.364309	0.68556	9.272884	0.076956	1.266293	0.136559	477.9295	5.833088	530.1295	38.31353	762.563	194.0186	477.9	5.8	62.6741	508
19AR105_Part2_Spot 117	159.6556	4662.7	0.969568	16.38741	3.133553	0.615524	4.850424	0.07702	0.752644	0.155171	478.308	3.469648	487.0378	18.76681	529.2977	105.066	478.3	3.5	90.36654	608
19AR105_Part2_Spot 362	58.4385	5477.018	1.427637	14.15651	7.797289	0.718917	8.077201	0.077036	1.562936	0.1935	478.4048	7.206454	550.0273	34.31468	859.7733	164.714	478.4	7.2	55.64313	817
19AR105_Part2_Spot 83	175.868	13321.84	1.138605	17.8268	3.232699	0.583649	3.639514	0.077052	0.974739	0.267821	478.5016	4.495244	466.8036	13.62047	410.6235	78.44086	478.5	4.5	116.5305	579
19AR105_Spot 122	188.2391	11827.66	1.585047	16.94882	4.579761	0.617321	4.726295	0.077061	0.744809	0.157588	478.5542	3.435227	488.1671	18.31947	534.5155	102.2442	478.6	3.4	89.53046	105
19AR105_Spot 119	191.4598	21747.24	0.966156	17.59681	3.59591	0.600105	3.72228	0.077064	0.876059	0.235356	478.5759	4.040763	477.3004	14.17575	472.1325	80.07241	478.6	4.0	101.3647	102
19AR105_Spot 14	233.3328	10077.81	0.78408	16.93156	3.623454	0.611504	3.755892	0.077069	0.679015	0.180787	478.6039	3.132086	484.5082	14.47239	513.4708	81.19129	478.6	3.1	93.20956	14
19AR105_Spot 139	122.0684	6939.771	1.113567	16.3409	4.2202	0.630075	4.518274	0.077082	1.024858	0.226825	478.681	4.728085	496.1423	17.735	578.4778	95.65393	478.7	4.7	82.74838	119
19AR105_Spot 416	73.9438	6618.234	1.328751	11.87747	12.41035	0.874269	12.56125	0.077121	1.867835	0.148698	478.9158	8.621156	637.8825	59.56261	1251.575	243.9863	478.9	8.6	38.26506	361
19AR105_Part2_Spot 59	91.15139	8019.372	1.444798	15.93497	6.079894	0.645698	6.238166	0.077145	1.18615	0.190144	479.0564	5.476325	505.8277	24.85723	629.7048	132.0329	479.1	5.5	76.07635	561
19AR105_Spot 244	99.76449	8454.664	1.077376	16.23355	7.224307	0.645142	7.432282	0.077178	1.207549	0.162474	479.2577	5.57738	505.4846	29.6024	626.9094	158.2575	479.3	5.6	76.44768	207
19AR105_Spot 506	362.6889	32241.81	1.167087	17.55757	2.4574	0.604026	2.588574	0.077183	0.692921	0.267684	479.2892	3.200638	479.7858	9.898027	483.1414	55.09639	479.3	3.2	99.20268	442
19AR105_Spot 260	71.44928	5126.284	1.351107	14.66493	6.608643	0.69677	7.237978	0.077184	1.29084	0.178343	479.2944	5.96252	536.8599	30.18851	790.4508	149.6075	479.3	6.0	60.63558	222
19AR105_Part2_Spot 157	145.059	10770.77	1.800762	16.08129	2.645325	0.64603	3.464802	0.077202	0.913231	0.263574	479.3972	4.219177	506.033	13.80862	629.2257	72.03723	479.4	4.2	76.18843	641
19AR105_Part2_Spot 357	153.2372	12613.91	1.08342	17.00746	3.824846	0.610671	3.944406	0.077213	0.613829	0.15562	479.4662	2.836318	483.9829	15.18603	506.4067	85.7587	479.5	2.8	94.68006	814
19AR105_Part2_Spot 101	49.30911	4855.765	3.522689	13.09294	7.948288	0.780436	8.746563	0.077224	1.903148	0.217588	479.5343	8.795074	585.7323	38.94855	1023.014	173.0568	479.5	8.8	46.87464	596
19AR105_Spot 309	89.259	5528.597	1.81886	13.96224	6.530016	0.733765	6.83028	0.077252	1.24853	0.182793	479.6988	5.771773	558.7603	29.36	896.3022	138.7226	479.7	5.8	53.51976	265
19AR105_Part2_Spot 192	168.8088	14044.95	1.777317	16.63542	3.385042	0.63013	3.489898	0.077266	0.724912	0.207717	479.781	3.351718	496.1771	13.69863	573.4883	74.23974	479.8	3.4	83.66012	675
19AR105_Part2_Spot 310	347.8983	23565.72	1.203826	17.32078	1.946279	0.605705	2.053952	0.077363	0.590539	0.287514	480.3607	2.733604	480.8481	7.867276	484.1554	43.46386	480.4	2.7	99.21622	773
19AR105_Spot 422	64.9324	4425.567	1.326634	13.58524	6.989404	0.754716	8.07721	0.077363	1.683786	0.208461	480.3624	7.794274	570.9569	35.28927	951.2029	161.8931	480.4	7.8	50.50052	367
19AR105_Spot 15	255.8436	17117.21	10.46559	17.37341	2.22241	0.604452	2.448278	0.077403	0.842668	0.344188	480.6024	3.902598	480.0553	9.365656	478.3837	50.83541	480.6	3.9	100.4638	15
19AR105_Part2_Spot 315	173.1777	14924.7	1.028946	16.6589	2.351656	0.626452	2.723804	0.077405	0.953357	0.35001	480.6173	4.415361	493.883	10.65291	556.8088	55.64725	480.6	4.4	86.31639	776
19AR105_Part2_Spot 124	189.5593	7958.007	1.870823	17.26467	3.804986	0.598304	5.001831	0.077413	0.891844	0.178303	480.66	4.13082	476.1572	19.01395	455.4821	109.2952	480.7	4.1	105.5277	614
19AR105_Part2_Spot 100	162.412	11713.95	0.846647	17.72368	3.656404	0.587858	3.972519	0.077425	0.697047	0.175467	480.7344	3.229048	469.4992	14.93442	415.8909	87.42352	480.7	3.2	115.5915	595
19AR105_Part2_Spot 349	141.7312	8855.747	0.959082	17.5834	2.998257	0.589144	4.163782	0.077435	0.889267	0.213572	480.7918	4.119975	470.3206	15.6751	420.4905	90.84396	480.8	4.1	114.3407	806
19AR105_Part2_Spot 2	360.7957	19600.83	1.513659	17.37958	1.74225	0.603014	1.926208	0.077435	0.738827	0.383566	480.7924	3.422992	479.1449	7.357513	472.2326	39.3745	480.8	3.4	101.8126	517
19AR105_Part2_Spot 129	143.9573	10017.6	1.915435	16.45974	4.544548	0.632481	4.75871	0.077453	0.785775	0.165123	480.9003	3.641285	497.6402	18.72267	576.3249	102.0499	480.9	3.6	83.44258	619
19AR105_Part2_Spot 340	63.73308	3722.775	1.755082	15.6366	5.053119	0.640147	5.504142	0.077465	1.291109	0.23457	480.9719	5.983866	502.3971	21.81641	602.1202	115.8564	481.0	6.0	79.87971	799
19AR105_Part2_Spot 37	300.7351	17634.75	1.757931	17.72947	2.338247	0.594028	2.475488	0.077473	0.612092	0.247261	481.0245	2.837142	473.4366	9.367291	437.776	53.39788	481.0	2.8	109.8792	541
19AR105_Spot 313	119.4109	8740.054	1.006121	15.07453	4.821998	0.689548	5.369447	0.077477	1.011656	0.18841	481.0434	4.689366	532.5287	22.25476	760.5823	111.2837	481.0	4.7	63.24673	269
19AR105_Spot 516	119.3212	9760.797	1.107843	16.56898	3.692777	0.631686	4.299495	0.077514	1.039334	0.241734	481.2643	4.819793	497.1456	16.90255	571.9051	90.79021	481.3	4.8	84.15108	450
19AR105_Part2_Spot 98	162.8188	11238.46	1.818558	17.47239	3.368019	0.596333	3.656894	0.077529	0.83983	0.229657	481.3555	3.895329	474.9041	13.87186	444.8056	79.1483	481.4	3.9	108.2171	593
19AR105_Part2_Spot 237	143.6872	15572.56	1.906404	16.49719	3.521062	0.635322	3.672234	0.07753	0.794387	0.216323	481.3618	3.684596	499.4058	14.48706	583.8987	77.85211	481.4	3.7	82.43927	713
19AR105_Spot 317	133.2241	12705.71	0.751765	16.1086	3.348879	0.650123	3.562574	0.077541	1.121551	0.314815	481.4304	5.202791	508.5544	14.25284	633.4092	72.82313	481.4	5.2	76.00622	272
19AR105_Part2_Spot 125	310.7121	25815.85	1.98067	17.70891	2.32097	0.597555	2.532794	0.077576	0.832201	0.32857	481.6355	3.862105	475.6812	9.619778	448.0142	53.17139	481.6	3.9	107.5045	615
19AR105_Part2_Spot 328	49.3689	5965.378	1.414519	14.32556	7.431811	0.718803	7.698484	0.077683	1.276031	0.165751	482.2785	5.929458	549.9601	32.7016	842.0913	158.205	482.3	5.9	57.27152	788
19AR105_Part2_Spot 51	151.5962	14493.01	4.06952	16.1864	4.384331	0.646672	4.740178	0.077723	0.848448	0.178991	482.5162	3.944435	506.4284	18.9039	616.8507	100.7368	482.5	3.9	78.22253	554
19AR105_Spot 25	78.86909	5527.84	1.455905	16.95834	4.33665	0.603216	5.806643	0.077725	1.527079	0.262988	482.5285	7.09957	479.2723	22.18732	464.6883	124.227	482.5	7.1	103.8392	25
19AR105_Spot 107	176.8853	13437.45	4.297173	17.22585	3.761597	0.609683	4.258532	0.077725	0.753285	0.176889	482.5309	3.502129	483.3603	16.37915	488.278	92.53113	482.5	3.5	98.82298	94
19AR105_Spot 325	207.7298	14789.33	0.608809	17.00033	3.779977	0.618475	4.18755	0.077744	0.863492	0.206204	482.6404	4.015369	488.8909	16.24961	519.2229	89.97047	482.6	4.0	92.95438	279
19AR105_Spot 2	109.4101	6993.293	0.904905	15.91374	4.760598	0.649514	4.98969	0.07778	0.947177	0.189827	482.8552	4.406407	508.1796	19.95225	624.7394	105.7065	482.9	4.4	77.28906	2
19AR105_Spot 382	125.5405	10429.4	2.056849	16.17442	4.27384	0.64941	4.487828	0.077785	0.942122	0.209928	482.8897	4.383194	508.1158	17.94328	624.2357	94.6362	482.9	4.4	77.35696	330
19AR105_Part2_Spot 392	90.31904	7710.614	2.010526	15.12531	5.697488	0.685069	6.113419	0.077814	0.969889	0.158649	483.0643	4.513951	529.8335	25.24179	737.664	127.8552	483.1	4.5	65.48568	841
19AR105_Part2_Spot 390	161.1511	13531.38	1.536026	16.57282	4.140174	0.632026	4.287454	0.077845	0.819995	0.191255	483.247	3.817719	497.3569	16.86075	563.7631	91.7167	483.2	3.8	85.71809	839
19AR105_Spot 358	81.72697	6536.824	3.521314	15.59689	4.219738	0.667536	4.610334	0.077855	1.37662	0.298594	483.3056	6.409995	519.2131	18.74179	681.3977	94.02608	483.3	6.4	70.92857	306
19AR105_Spot 552	233.9248	15856.67	1.740211	17.345	3.092834	0.616074	3.597776	0.0779	0.995257	0.276631	483.5729	4.63671	487.3838	13.92717	506.3013	76.07352	483.6	4.6	95.5109	479
19AR105_Part2_Spot 201	96.45035	10290.44	1.37546	15.20401	6.988009	0.69279	7.30251	0.078004	0.925251	0.126703	484.1964	4.315917	534.4751	30.35497	756.1717	153.0348	484.2	4.3	64.0326	683
19AR105_Part2_Spot 82	114.5525	9241.529	2.833633	16.26863	4.768875	0.644862	5.174721	0.078009	1.125953	0.217587	484.2288	5.252449	505.3118	20.6022	602.8431	109.3544	484.2	5.3	80.32418	578
19AR105_Spot 223	230.6587	12812.43	1.951272	17.833	2.595818	0.594963	2.848243	0.078029	0.822035	0.288611	484.3483	3.835614	474.032	10.78854	425.3555	60.86305	484.3	3.8	113.8691	191
19AR105_Part2_Spot 87	193.9113	11785.56	1.804902	16.46318	3.13322	0.638928	3.430036	0.078041	0.766383	0.223433	484.4202	3.576456	501.6423	13.57834	581.9478	72.61018	484.4	3.6	83.24118	583
19AR105_Part2_Spot 240	144.9851	8688.275	1.546893	16.33019	2.54389	0.638703	3.030943	0.07807	0.774548	0.255547	484.5923	3.615794	501.5026	11.99572	580.3873	63.68553	484.6	3.6	83.49465	716
19AR105_Part2_Spot 299	98.2726	6815.034	1.181315	15.62058	5.287045	0.66134	5.449987	0.078074	0.858128	0.157455	484.6147	4.006147	515.4335	22.0323	655.4676	115.5167	484.6	4.0	73.9342	763
19AR105_Part2_Spot 20	302.2167	7736.363	3.889236	17.01251	2.233571	0.611756	2.625645	0.078153	0.642134	0.244562	485.0874	3.000599	484.6667	10.1195	483.6602	56.25107	485.1	3.0	100.2951	531
19AR105_Spot 595	41.96631	4653.524	1.699317	13.88129	10.12917	0.747571	10.44216	0.078209	1.491008	0.142787	485.4255	6.971943	566.8139	45.38654	909.3159	213.3863	485.4	7.0	53.38359	513
19AR105_Spot 64	168.7987	10457.95	1.232544	15.87985	4.307203	0.663389	4.497326	0.078333	0.916429	0.203772	486.1643	4.291495	516.6847	18.21399	654.9519	94.4887	486.2	4.3	74.229	59
19AR105_Spot 55	253.3488	28124.7	0.796217	17.10918	2.692528	0.624721	2.830087	0.078369	0.699273	0.247085	486.3816	3.275997	492.8019	11.04979	523.6846	60.14491	486.4	3.3	92.87681	51
19AR105_Spot 192	125.8225	10987	1.211155	15.54056	5.540267	0.68473	5.758578	0.078394	0.954901	0.165822	486.5277	4.47487	529.629	23.76914	720.856	120.6146	486.5	4.5	67.49305	165
19AR105_Spot 578	118.173	9175.799	1.242268	16.73879	4.31766	0.632227	4.894303	0.078416	1.119472	0.22873	486.6581	5.24744	497.4825	19.25154	548.5758	104.1078	486.7	5.2	88.71301	499
19AR105_Part2_Spot 352	667.3626	39376.21	6.987319	17.62335	1.470863	0.609412	1.59154	0.078469	0.5686	0.357264	486.9775	2.66695	483.189	6.119222	466.2198	32.92645	487.0	2.7	104.4524	809
19AR105_Part2_Spot 144	221.4088	13999.04	5.047874	17.53684	3.165773	0.606001	3.496491	0.078473	0.857748	0.245317	487.0013	4.023359	481.0346	13.39722	453.6407	75.28218	487.0	4.0	107.354	632
19AR105_Part2_Spot 7	353.519	23124.5	2.85671	17.46436	2.407728	0.611091	2.640919	0.078514	0.695657	0.263415	487.2454	3.264628	484.2482	10.17151	471.0716	56.41162	487.2	3.3	103.4334	522
19AR105_Part2_Spot 376	122.9469	9115.523	1.015492	16.83958	4.004279	0.623072	4.358804	0.078603	1.105383	0.253598	487.777	5.192865	491.7711	16.99176	511.3948	92.70443	487.8	5.2	95.3817	829
19AR105_Part2_Spot 202	85.33006	8142.553	1.575604	15.37526	5.440284	0.687457	5.779956	0.07869	1.224332	0.211824	488.2993	5.757591	531.2713	23.91374	721.2827	119.9798	488.3	5.8	67.69874	684
19AR105_Spot 378	335.621	21327.66	4.169509	17.16554	2.625396	0.627432	2.95792	0.078826	0.780573	0.263893	489.1134	3.676646	494.4947	11.57974	520.4253	62.61752	489.1	3.7	93.98341	326
19AR105_Part2_Spot 203	359.8908	3280.68	18.46426	15.91987	2.258914	0.635884	2.595498	0.07896	0.982841	0.378671	489.9143	4.63666	499.7548	10.2445	546.0071	52.4985	489.9	4.6	89.72674	685
19AR105_Part2_Spot 332	48.91273	4067.975	1.600924	14.20525	9.075556	0.726172	9.745056	0.079171	1.196289	0.122759	491.173	5.657579	554.3037	41.64981	823.8032	202.3158	491.2	5.7	59.62261	792
19AR105_Spot 558	154.1363	10633.83	1.228958	16.70043	3.4249	0.645078	3.722411	0.079339	0.847977	0.227803	492.1733	4.018175	505.4455	14.82214	566.9362	78.95557	492.2	4.0	86.81282	483
19AR105_Spot 46	302.5945	22173.77	3.529221	16.2864	1.786185	0.665398	2.08077	0.079567	0.856678	0.411712	493.5388	4.070243	517.9103	8.441648	627.8381	40.86114	493.5	4.1	78.60925	44
19AR105_Part2_Spot 52	295.0107	24580.83	7.255119	17.41558	3.021686	0.622846	3.148382	0.080132	0.754908	0.239776	496.9109	3.610286	491.6295	12.26992	468.0594	67.70389	496.9	3.6	106.1641	555
19AR105_Spot 534	85.14084	9415.443	1.577835	15.05248	5.215461	0.723209	5.554076	0.080217	1.658159	0.298548	497.4197	7.937824	552.5594	23.67261	787.7219	111.3575	497.4	7.9	63.14661	468
19AR105_Spot 500	105.4566	10126.1	2.218916	13.98069	9.539462	0.779799	9.770975	0.080447	0.963245	0.098582	498.7915	4.623408	585.369	43.49559	938.0749	199.7987	498.8	4.6	53.17182	436
19AR105_Part2_Spot 27	260.7433	25393.81	4.421241	16.96512	3.215677	0.6487	3.392664	0.08045	0.913074	0.269132	498.807	4.382727	507.6785	13.55498	548.8143	71.37466	498.8	4.4	90.88813	536
19AR105_Spot 586	288.8751	25033.28	10.453	16.84111	2.226461	0.663916	2.52624	0.081014	0.780205	0.30884	502.1699	3.76924	517.0063	10.23531	584.0495	52.16723	502.2	3.8	85.98071	505
19AR105_Part2_Spot 321	208.535	13648.1	2.372033	16.46134	3.295992	0.665975	3.413825	0.081339	0.726087	0.21269	504.1098	3.520817	518.2623	13.85759	582.0767	72.45206	504.1	3.5	86.60539	782
19AR105_Part2_Spot 286	320.883	39058.05	3.97971	16.8291	2.020226	0.662868	2.20129	0.081569	0.720044	0.327101	505.4807	3.500641	516.367	8.910205	565.7632	45.30246	505.5	3.5	89.34493	752
19AR105_Part2_Spot 322	244.7572	20833.27	5.563049	17.11946	3.507392	0.646137	3.608882	0.081611	0.783303	0.217049	505.7308	3.810002	506.0989	14.38435	508.7208	77.49352	505.7	3.8	99.41224	783
19AR105_Spot 295	263.1602	18268.37	4.216498	17.69689	2.574619	0.627052	2.77078	0.081638	0.930071	0.335671	505.8929	4.525276	494.258	10.84303	441.6556	58.08666	505.9	4.5	114.5447	253
19AR105_Spot 277	199.9058	2882.586	2.031972	16.31722	2.366412	0.637853	2.763912	0.082383	0.88967	0.321888	510.3331	4.365213	500.9761	10.92991	459.4269	58.0381	510.3	4.4	111.0804	237
19AR105_Spot 186	257.3922	20500.59	6.469198	17.27883	2.824804	0.656766	3.002627	0.082468	0.785973	0.261762	510.8391	3.860095	512.6339	12.08652	521.5792	63.59684	510.8	3.9	97.94085	159
19AR105_Part2_Spot 81	115.6066	12070.61	3.883902	16.44713	4.169476	0.678339	4.419192	0.082472	1.064794	0.240948	510.8611	5.229664	525.7703	18.13787	591.9864	93.02324	510.9	5.2	86.29608	577
19AR105_Spot 530	182.3445	13941.4	2.571298	16.21647	3.758286	0.694428	4.054489	0.082595	0.808235	0.199343	511.5934	3.975061	535.4574	16.87369	639.4096	85.48763	511.6	4.0	80.01027	464
19AR105_Spot 418	255.3082	25487.64	2.513829	15.47024	3.263925	0.734011	3.89711	0.08276	2.026504	0.520002	512.5737	9.985101	558.9045	16.75184	753.2475	70.27764	512.6	10.0	68.0485	363
19AR105_Spot 21	245.8913	22037.11	3.178236	16.71469	3.531332	0.674437	3.733682	0.082834	0.764571	0.204777	513.0136	3.770341	523.4066	15.27118	569.9781	79.56127	513.0	3.8	90.00584	21
19AR105_Spot 282	133.6766	8038.816	1.735333	16.2502	4.290313	0.68313	4.533122	0.082996	1.029323	0.227067	513.9803	5.085105	528.6642	18.68366	593.5375	95.74846	514.0	5.1	86.5961	242
19AR105_Spot 526	345.0682	30747.16	7.18333	16.87937	2.589434	0.679428	2.720674	0.083187	0.698295	0.256663	515.1147	3.457061	526.4289	11.17651	576.7662	57.18484	515.1	3.5	89.31083	460
19AR105_Spot 280	298.4362	15424.34	2.128821	17.20283	2.395184	0.655588	2.763181	0.083198	0.713383	0.258175	515.1848	3.53222	511.912	11.11055	498.26	58.83295	515.2	3.5	103.3968	240
19AR105_Spot 386	114.4583	7960.191	1.980572	15.74112	4.980605	0.710098	5.225512	0.083298	1.091876	0.208951	515.7775	5.412249	544.8045	22.03557	669.0949	109.42	515.8	5.4	77.08585	334
19AR105_Part2_Spot 189	406.508	25922.35	2.983886	17.09467	1.748205	0.666203	1.902569	0.0833	0.689398	0.362351	515.7886	3.4173	518.4012	7.724271	530.9065	38.84682	515.8	3.4	97.15243	672
19AR105_Spot 172	117.8614	10581.63	3.048771	16.1596	4.541647	0.699154	4.772488	0.083301	0.827464	0.173382	515.7935	4.101721	538.2857	19.9421	635.703	101.2101	515.8	4.1	81.13751	148
19AR105_Part2_Spot 21	312.85	29883.95	5.49805	17.22867	2.561749	0.659749	2.704858	0.083324	0.653392	0.241562	515.9307	3.23968	514.4607	10.91761	508.8948	57.74212	515.9	3.2	101.3826	532

19AR105_Spot 264	207.1993	16119.68	1.496612	16.58721	3.273152	0.682561	3.488108	0.083331	0.86897	0.249124	515.9764	4.308938	528.3211	14.36877	582.9393	73.37435	516.0	4.3	88.51289	225
19AR105_Part2_Spot 200	358.5329	30254.23	20.67113	17.08318	2.654056	0.667882	2.813097	0.083379	0.896747	0.318776	516.2589	4.449009	519.4239	11.43846	534.3025	58.38189	516.3	4.4	96.62297	682
19AR105_Part2_Spot 283	477.7347	12446.36	1.78081	17.01665	1.541103	0.660162	2.500826	0.083421	0.84092	0.336257	516.5092	4.17398	514.7131	10.09782	507.7242	51.81258	516.5	4.2	101.7303	749
19AR105_Part2_Spot 318	220.7809	13552.62	4.865178	16.48891	2.965352	0.681726	3.217102	0.08346	0.981011	0.304936	516.7438	4.871457	527.8169	13.24262	576.9622	66.61686	516.7	4.9	89.56286	779
19AR105_Spot 207	320.4288	34460.17	1.597993	17.10318	2.20632	0.670515	2.428148	0.083603	0.747815	0.307977	517.5955	3.71934	521.0255	9.896393	537.0292	50.56812	517.6	3.7	96.38126	178
19AR105_Spot 407	70.57993	4882.673	1.726758	14.75312	6.132155	0.7477	6.663085	0.083619	1.217171	0.182674	517.6898	6.054796	566.8891	28.95233	770.4175	138.0317	517.7	6.1	67.19601	354
19AR105_Spot 475	104.5271	10921.21	2.829744	16.18657	5.982283	0.702689	6.378863	0.083665	0.984495	0.154337	517.9636	4.899841	540.3957	26.7363	637.1526	135.732	518.0	4.9	81.29349	415
19AR105_Spot 448	229.2553	19181.94	4.931122	17.2834	1.419463	0.661159	1.716702	0.083674	0.841245	0.490035	518.0156	4.187286	515.3224	6.93787	504.3767	32.94388	518.0	4.2	102.7041	390
19AR105_Part2_Spot 140	215.9379	13578.38	2.848692	17.48061	2.736889	0.646571	3.061829	0.083699	0.775858	0.253397	518.1616	3.862872	506.3662	12.20864	454.3991	65.75091	518.2	3.9	114.0323	629
19AR105_Part2_Spot 62	125.5447	10965.42	5.892873	16.39078	4.316629	0.687663	4.753481	0.083704	1.317186	0.277099	518.196	6.558473	531.3956	19.66917	589.4082	99.10895	518.2	6.6	87.91802	564
19AR105_Part2_Spot 350	231.6746	19195.06	1.161364	17.30278	2.993511	0.658456	3.200265	0.083821	0.805906	0.251825	518.8879	4.017875	513.6692	12.90215	491.4539	68.31815	518.9	4.0	105.5822	807
19AR105_Spot 191	393.3564	36215.69	2.916333	16.86215	1.941327	0.68433	2.088856	0.083842	0.725728	0.347428	519.0132	3.618982	529.3882	8.617619	575.329	42.5671	519.0	3.6	90.21156	164
19AR105_Spot 9	191.1266	13536.76	2.565696	16.61424	2.537734	0.682553	2.948881	0.083907	0.781033	0.264857	519.4018	3.897573	528.3165	12.14719	567.9865	61.91493	519.4	3.9	91.44616	9
19AR105_Spot 272	113.2461	7962.384	1.820103	16.3421	4.233402	0.686039	4.491762	0.08393	0.884034	0.196812	519.5353	4.412667	530.4176	18.55992	578.4577	95.71865	519.5	4.4	89.81388	232
19AR105_Part2_Spot 380	219.3134	20078.18	2.491694	17.01821	3.333674	0.6693	3.506162	0.08394	0.859049	0.245011	519.5992	4.288459	520.287	14.27503	524.2408	74.57737	519.6	4.3	99.11461	832
19AR105_Spot 545	128.3365	10688.65	2.344739	16.10814	3.311837	0.708799	3.585225	0.084009	0.984694	0.274653	520.0102	4.919427	544.0326	15.10116	646.9407	74.0665	520.0	4.9	80.37989	475
19AR105_Part2_Spot 18	358.3472	18011.54	13.16134	17.65061	2.315904	0.644606	2.435513	0.084016	0.577756	0.237221	520.0523	2.886629	505.1538	9.693173	439.1904	52.67903	520.1	2.9	118.4116	529
19AR105_Part2_Spot 327	153.8316	13120.62	2.035883	16.93244	3.546123	0.66794	3.691373	0.084024	0.762662	0.206607	520.0997	3.810806	519.4589	15.01089	517.5769	79.31891	520.1	3.8	100.4874	787
19AR105_Spot 593	289.1408	22521.03	5.145	17.12249	2.802356	0.675486	2.988399	0.084233	0.805626	0.269584	521.34	4.034704	524.0426	12.23391	536.8105	62.96663	521.3	4.0	97.11808	511
19AR105_Part2_Spot 115	288.5348	25724.75	7.155894	17.32003	1.835041	0.665341	2.002703	0.084325	0.666997	0.333048	521.887	3.343791	517.8758	8.124504	501.1974	41.57711	521.9	3.3	104.128	606
19AR105_Spot 27	170.7032	15132.24	1.14533	17.07802	3.65015	0.669455	3.936322	0.084374	0.818919	0.208042	522.1787	4.107613	520.3808	16.02888	513.4411	84.61928	522.2	4.1	101.7018	27
19AR105_Part2_Spot 185	310.3696	20251.39	43.69372	16.25669	2.783454	0.707823	3.07809	0.084423	0.728194	0.236573	522.4665	3.654478	543.4528	12.95438	633.4289	64.38436	522.5	3.7	82.48227	668
19AR105_Part2_Spot 344	167.846	12508.34	2.208739	17.44626	3.50785	0.652919	3.878346	0.084424	0.92084	0.237431	522.4761	4.621362	510.2736	15.55673	456.9209	83.60854	522.5	4.6	114.3472	802
19AR105_Spot 171	189.8944	13286.42	1.944447	17.00197	3.537282	0.675943	3.702769	0.084429	0.790408	0.213464	522.5079	3.967004	524.3196	15.16491	533.1501	79.22358	522.5	4.0	98.0039	147
19AR105_Spot 528	180.8048	15202.59	2.2546	15.30161	3.821935	0.75475	3.974989	0.08443	0.988323	0.248635	522.513	4.960372	570.9767	17.36185	769.8526	81.09746	522.5	5.0	67.87183	462
19AR105_Spot 442	293.2104	24988.8	1.13186	17.53326	2.548768	0.662066	2.717701	0.084471	0.850877	0.313087	522.7554	4.272436	515.8769	10.99263	486.504	56.98533	522.8	4.3	107.4514	385
19AR105_Part2_Spot 47	309.005	16113.82	1.601185	16.85362	2.196285	0.678855	2.406026	0.084471	0.741334	0.308116	522.7576	3.722412	526.0821	9.87888	541.4759	50.05512	522.8	3.7	96.54309	550
19AR105_Spot 443	75.41904	7243.172	1.507992	13.04871	6.312687	0.875898	6.550117	0.084485	1.161747	0.177363	522.8385	5.834269	638.7645	31.06409	1074.531	129.5952	522.8	5.8	48.65734	386
19AR105_Spot 94	264.1065	17477.27	6.501759	17.22799	2.12683	0.66773	2.393426	0.084534	0.834308	0.348583	523.1282	4.192105	519.3311	9.730568	503.621	49.35693	523.1	4.2	103.8734	83
19AR105_Part2_Spot 300	91.50459	5080.998	1.841369	15.24544	5.336925	0.726935	6.063674	0.084549	0.935124	0.154217	523.2167	4.699435	554.7527	25.92267	687.3272	127.9698	523.2	4.7	76.12337	764
19AR105_Spot 204	288.0673	27364.45	3.697557	17.08975	1.865489	0.678639	2.118063	0.084603	0.833571	0.393553	523.5381	4.191553	525.9518	8.694829	537.4157	42.6084	523.5	4.2	97.41772	176
19AR105_Part2_Spot 326	244.4957	15523.05	2.195992	16.91278	2.885564	0.675533	3.002515	0.084629	0.68435	0.227926	523.6929	3.442183	524.0708	12.29221	526.694	64.09082	523.7	3.4	99.4302	786
19AR105_Spot 462	59.76463	5830.36	81.99145	11.79103	10.23875	0.96336	11.13835	0.08463	3.725611	0.334485	523.7006	18.7396	685.0356	55.54851	1259.63	205.6984	523.7	18.7	41.57575	403
19AR105_Spot 181	170.9346	13600.9	1.607569	16.91366	3.123252	0.683017	3.363611	0.084633	0.947204	0.281603	523.7191	4.764528	528.5962	13.86135	550.6826	70.47824	523.7	4.8	95.10363	155
19AR105_Spot 491	258.7929	28230.7	7.752305	17.101	3.057279	0.682266	3.137673	0.084687	0.681645	0.217245	524.0363	3.430732	528.1428	12.92171	546.86	66.92204	524.0	3.4	95.82641	428
19AR105_Part2_Spot 238	246.5582	19051.03	1.477339	17.30426	3.018819	0.663049	3.222138	0.084696	0.866724	0.26899	524.0912	4.362681	516.4772	13.04483	483.9126	68.54469	524.1	4.4	108.3029	714
19AR105_Part2_Spot 228	197.623	16636.73	3.396517	16.91316	3.205432	0.678042	3.406392	0.08474	0.71441	0.209726	524.3541	3.597735	525.5906	13.97674	531.9395	72.96307	524.4	3.6	98.57401	706
19AR105_Spot 20	147.3588	10501.6	1.673033	16.13864	4.268698	0.707205	4.485135	0.084829	0.867401	0.193395	524.8823	4.372412	543.0855	18.86754	621.1541	94.95755	524.9	4.4	84.50113	20
19AR105_Spot 365	88.22876	3768.524	1.862102	13.72888	7.645717	0.811534	8.101095	0.084868	1.326759	0.163775	525.1156	6.690812	603.314	36.86587	910.1089	164.8228	525.1	6.7	57.6981	313
19AR105_Part2_Spot 43	122.9469	9019.529	1.952896	16.4309	4.151257	0.691386	4.354017	0.084925	0.839335	0.192773	525.4557	4.235373	533.6328	18.07356	569.715	93.03161	525.5	4.2	92.23132	546
19AR105_Part2_Spot 191	229.4392	14586.56	2.034608	17.18229	2.609078	0.670631	2.919003	0.08493	0.62519	0.214179	525.4825	3.15493	521.0959	11.89837	502.8706	62.78966	525.5	3.2	104.4965	674
19AR105_Part2_Spot 351	189.0985	10891.09	5.746251	17.52257	3.695227	0.652058	4.201417	0.084949	0.663887	0.158015	525.595	3.350899	509.7444	16.83939	440.2071	92.33454	525.6	3.4	119.3972	808
19AR105_Spot 194	121.8779	9307.969	2.739897	15.8937	4.85954	0.721106	5.189192	0.08497	1.131407	0.218031	525.7209	5.711966	551.3198	22.07953	659.4542	108.6133	525.7	5.7	79.7206	167
19AR105_Part2_Spot 177	145.6292	12938.73	1.926831	16.08232	4.149957	0.715796	4.508801	0.085007	1.131727	0.251004	525.9425	5.715893	548.1823	19.10143	642.6737	93.86579	525.9	5.7	81.83663	661
19AR105_Part2_Spot 302	174.3544	10144.59	1.655765	16.10792	3.90233	0.705638	4.28228	0.08501	0.792666	0.185104	525.9586	4.00355	542.1527	17.99059	611.7569	90.9806	526.0	4.0	85.9751	766
19AR105_Part2_Spot 56	294.875	20041.17	1.992143	17.10993	1.941228	0.671816	2.100019	0.085027	0.623006	0.296667	526.0569	3.147207	521.816	8.568905	504.2768	44.15515	526.1	3.1	104.3191	558
19AR105_Spot 273	117.3953	9103.414	1.582256	16.05566	4.28362	0.712979	4.863525	0.085027	1.063121	0.218591	526.0599	5.370545	546.5138	20.55726	633.6905	102.2281	526.1	5.4	83.01526	233
19AR105_Spot 384	368.6903	19605.93	6.185104	17.06284	2.598753	0.679681	2.73076	0.085029	0.805134	0.294839	526.071	4.067357	526.5816	11.22043	529.7703	57.16926	526.1	4.1	99.30171	332
19AR105_Part2_Spot 88	369.9081	27423.31	4.447956	17.16702	2.641873	0.676581	2.738817	0.085054	0.549251	0.200543	526.2187	2.775444	524.706	11.22292	519.0633	58.90863	526.2	2.8	101.3785	584
19AR105_Part2_Spot 99	426.3315	48931.13	4.839415	17.01772	1.796223	0.683324	1.946421	0.085114	0.644	0.330863	526.5749	3.256334	528.7813	8.02296	539.2478	40.20203	526.6	3.3	97.6499	594
19AR105_Spot 271	346.8837	15048.19	2.693226	16.91661	1.746512	0.681431	2.021314	0.085119	0.800474	0.396017	526.6025	4.04774	527.639	8.31795	533.0925	40.64813	526.6	4.0	98.78257	231
19AR105_Part2_Spot 85	375.2053	20552.08	6.08729	17.06832	1.578832	0.678692	1.898029	0.085233	0.898199	0.473227	527.2827	4.547532	525.9839	7.791895	521.2837	36.68203	527.3	4.5	101.1508	581
19AR105_Part2_Spot 95	300.663	18504.18	6.756724	17.76913	2.553446	0.650408	2.846776	0.085241	0.690337	0.242498	527.3289	3.495433	508.7299	11.39189	426.9246	61.61234	527.3	3.5	123.5181	590
19AR105_Spot 375	112.5729	12472.6	1.949783	15.35836	5.214959	0.754469	5.646774	0.085275	1.553861	0.275177	527.5329	7.870697	570.8138	24.66103	748.068	114.7812	527.5	7.9	70.51937	323
19AR105_Part2_Spot 151	290.8243	27756.84	2.684203	17.03942	2.270612	0.683125	2.421232	0.085337	0.774303	0.319797	527.9006	3.924663	528.6615	9.978477	532.9221	50.22007	527.9	3.9	99.05775	637
19AR105_Spot 267	53.79329	5077.183	1.753544	14.29572	10.20557	0.790059	10.65703	0.08534	1.285053	0.120583	527.9158	6.513648	591.2051	47.79459	843.131	220.7234	527.9	6.5	62.61374	228
19AR105_Part2_Spot 304	542.2361	41271.2	1.093254	17.11542	1.202928	0.679523	1.419327	0.0854	0.593568	0.418204	528.2758	3.010633	526.4862	5.830898	519.6673	28.31743	528.3	3.0	101.6565	768
19AR105_Spot 559	291.306	23977.77	7.604437	17.1835	2.549528	0.683402	2.821189	0.085401	0.670277	0.237587	528.2825	3.399749	528.8288	11.62973	532.1606	60.01603	528.3	3.4	99.27126	484
19AR105_Part2_Spot 79	150.2311	9574.842	3.245894	17.00394	3.536842	0.675713	3.960296	0.085462	0.957487	0.241772	528.6444	4.859717	524.18	16.2165	505.7582	84.58801	528.6	4.9	104.5251	575
19AR105_Part2_Spot 96	119.7369	17993.96	1.515409	17.05029	3.782473	0.679811	4.01273	0.08547	0.888426	0.221402	528.6928	4.509594	526.6599	16.49057	518.7914	85.92436	528.7	4.5	101.9086	591
19AR105_Part2_Spot 235	262.2534	19635.43	2.921284	16.96078	2.448974	0.683418	2.745576	0.085498	0.737852	0.268742	528.8583	3.746413	528.838	11.31816	529.7251	57.93928	528.9	3.7	99.83637	711
19AR105_Spot 331	256.0452	20316.47	1.777295	16.86314	3.286066	0.688982	3.409642	0.085503	0.742885	0.217878	528.8864	3.772164	532.1889	14.12374	547.303	72.70964	528.9	3.8	96.63502	285
19AR105_Part2_Spot 173	220.2183	14179.32	3.297754	16.2746	3.438678	0.712396	3.61946	0.085536	0.845712	0.233657	529.0804	4.295799	546.1679	15.29057	619.0315	75.97452	529.1	4.3	85.46906	657
19AR105_Spot 490	436.5993	46154.05	4.220447	17.05217	1.438838	0.694157	1.594263	0.08562	0.581802	0.364935	529.5817	2.957955	535.2951	6.632851	560.6807	32.3524	529.6	3.0	94.45335	427
19AR105_Spot 452	247.8331	21701.04	3.438166	17.55624	2.934565	0.665232	3.070744	0.08565	0.703825	0.229204	529.7564	3.579469	517.8095	12.45644	466.4	66.22374	529.8	3.6	113.5841	393
19AR105_Part2_Spot 55	124.9487	10759.71	1.583088	16.63245	3.624523	0.689135	3.832289	0.085654	0.803841	0.209755	529.7814	4.088307	532.2806	15.87682	543.9379	81.90149	529.8	4.1	97.3974	557
19AR105_Part2_Spot 206	363.8196	23605.96	3.747221	17.29725	1.858229	0.676019	2.231577	0.085696	0.853016	0.382248	530.0324	4.340382	524.3654	9.139732	500.744	45.40527	530.0	4.3	105.849	688
19AR105_Spot 268	251.486	20674.32	3.40099	17.24764	2.568048	0.676938	2.826091	0.085698	0.733171	0.25943	530.0469	3.730677	524.9219	11.58422	503.64	60.0753	530.0	3.7	105.2432	229
19AR105_Part2_Spot 215	127.662	10866.14	1.573572	17.56723	4.368521	0.655341	4.642939	0.0857	0.794262	0.171069	530.0587	4.041615	511.7602	18.66602	431.7783	101.9807	530.1	4.0	122.7618	696
19AR105_Part2_Spot 388	165.8465	2536.564	2.5423	14.62692	3.596091	0.736871	9.790461	0.085934	0.965259	0.098592	531.4463	4.924075	560.5778	42.19945	681.5746	208.5577	531.4	4.9	77.9733	837
19AR105_Part2_Spot 131	315.298	23144.64	4.442922	17.36072	2.193066	0.675162	2.330112	0.086066	0.762824	0.327377	532.2308	3.896902	523.8464	9.5361	488.4034	48.58512	532.2	3.9	108.9736	621
19AR105_Spot 429	131.4574	16836.52	0.920906	15.98409	4.290968	0.736392	4.488757	0.086087	0.859611	0.191503	532.3507	4.392289	560.2979	19.33169	676.4436	94.22494	532.4	4.4	78.69846	373
19AR105_Part2_Spot 364	131.5869	11761.23	2.770165	16.61405	5.261145	0.69578	5.690721	0.086141	0.781061	0.137252	532.6717	3.993238	536.2671	23.71256	552.5175	123.1322	532.7	4.0	96.40812	819
19AR105_Part2_Spot 230	233.9466	17654.57	2.65223	16.9697	2.648977	0.687188	2.821022	0.086149	0.716888	0.254124	532.7223	3.665483	531.1094	11.66722	525.1188	59.8345	532.7	3.7	101.448	708
19AR105_Spot 3	81.68396	7717.426	1.555002	14.30181	4.943725	0.806598	5.117867	0.086153	1.174077	0.229408	532.7459	6.003368	600.5436	23.20545	866.4965	103.3378	532.7	6.0	61.48275	3
19AR105_Part2_Spot 8	165.0113	13188.23	2.705601	15.92224	4.271953	0.732005	4.366373	0.086314	0.815118	0.186681	533.7017	4.175085	557.7292	18.7398	657.9861	91.9997	533.7	4.2	81.1114	523
19AR105_Part2_Spot 53	145.2208	12642.35	3.896145	17.82458	3.672856	0.651247	3.811882	0.08658	0.768772	0.201678	535.2803	3.948869	509.2458	15.26637	394.9386	83.75408	535.3	3.9	135.5351	556
19AR105_Part2_Spot 250	154.7474	10309.97	2.199653	17.4497	1.776019	0.664195	2.053522	0.086604	0.864367	0.420919	535.4208	4.44102	517.1766	8.322049	438.3105	41.46155	535.4	4.4	122.1556	724
19AR105_Spot 126	810.0035	8418.7	2.657947	16.01795	1.158548	0.729425	1.738666	0.086648	1.260807	0.725158	535.6812	6.480907	556.2155	7.446166	642.1301	25.74103	535.7	6.5	83.42254	109
19AR105_Spot 168	189.4478	16208.86	0.79416	16.35437	3.383441	0.723185	3.562236	0.086648	0.913966	0.256571	535.6826	4.698058	552.5452	15.18107	623.5841	74.26934	535.7	4.7	85.90383	144
19AR105_Spot 1	131.4095	10834.31	2.133029	16.14292	4.949967	0.721903	5.080718	0.086674	0.757401	0.149074	535.8378	3.894345	551.7898	21.63171	619.107	108.5073	535.8	3.9	86.5501	1
19AR105_Part2_Spot 41	208.3393	14967.33	2.329239	17.62053	3.258061	0.664371	3.471164	0.08669	0.725083	0.208887	535.9336	3.728814	517.2842	14.06998	436.6806	75.61358	535.9	3.7	122.729	545
19AR105_Part2_Spot 285	282.8639	23216.77	7.830377	17.00363	2.099135	0.694505	2.412261	0.086731	0.734715	0.304575	536.173	3.779968	535.5036	10.03924	533.616	50.29179	536.2	3.8	100.4792	751
19AR105_Spot 65	151.3068	10996.96	1.737693	16.6258	4.091573	0.702245	4.304708	0.086814	0.832512	0.193396	536.6669	4.286898	540.1307	18.03374	555.7491	92.1496	536.7	4.3	96.56641	60
19AR105_Spot 240	263.0478	28871.8	2.599489	17.41908	2.959414	0.689284	3.063969	0.086915	0.724204	0.236361	537.2676	3.733182	532.3705	12.695	512.3723	65.44707	537.3	3.7	104.8588	204
19AR105_Part2_Spot 368	833.5794	4467.061	5.645011	15.90278	0.920469	0.710382	1.725248	0.086926	1.278399	0.740994	537.3314	6.590744	544.9729	7.275913	578.006	25.16891	537.3	6.6	92.96295	822
19AR105_Spot 515	124.8006	11960.14	1.502741	16.6142	3.759253	0.7102	3.998522	0.086986	0.771325	0.192903	537.6884	3.979075	544.8653	16.86177	575.9229	85.29822	537.7	4.0	93.36119	449
19AR105_Spot 425	225.1379	21125.05	4.219198	16.56073	2.839313	0.722561	3.01153	0.087058	0.816923	0.271265	538.1156	4.217512	552.1779	12.82745	611.5278	62.65647	538.1	4.2	87.99528	370
19AR105_Part2_Spot 91	112.4468	911.246	2.058211	13.91539	4.497606	0.67049	7.146797	0.087079	1.203892	0.168452	538.2386	6.216676	521.0103	29.13459	447.1239	156.7206	538.2	6.2	120.3779	586
19AR105_Spot 524	141.8654	13791.69	1.635966	16.70404	2.563204	0.712092	2.704599	0.087123	0.774957	0.286533	538.5015	4.003609	545.9875	11.42247	578.2884	56.33576	538.5	4.0	93.1199	458
19AR105_Spot 148	431.2402	30830.68	1.316769	17.10968	1.506429	0.698507	1.683578	0.087277	0.731031	0.434213	539.412	3.782798	537.8991	7.030297	532.4651	33.21376	539.4	3.8	101.3047	127
19AR105_Part2_Spot 248	201.4526	26189.51	2.716736	16.25775	3.050292	0.733142	3.129578	0.087674	0.673837	0.215312	541.7632	3.50141	558.3959	13.44297	627.7008	65.87735	541.8	3.5	86.30915	722
19AR105_Part2_Spot 26	183.4261	11727.27	2.724061	17.63127	3.154244	0.676149	3.370376	0.088233	0.708905	0.210334	545.0799	3.705241	524.4444	13.80591	436.5283	73.39287	545.1	3.7	124.867	535
19AR105_Part2_Spot 353	176.1411	14342.81	3.27211	17.04752	2.794959	0.698881	2.908935	0.088238	0.587472	0.201954	545.1087	3.0707	538.1221	12.15136	509.5873	62.65101	545.1	3.1	106.9706	810
19AR105_Spot 198	500.649	31127.27	7.141276	16.9116	1.278213	0.718592	1.533863	0.088559	0.787042	0.513111	547.0085	4.127586	549.8352	6.512264	562.4914	28.68845	547.0	4.1	97.24744	171
19AR105_Part2_Spot 49	75.82738	3435.867	2.200886	14.74996	6.399887	0.77377	9.191804	0.088815	1.138022	0.123808	548.5268	5.984153	581.9234	40.73598	715.421	194.1131	548.5	6.0	76.67189	552
19AR105_Part2_Spot 251	234.6271	24555.74	2.980436	16.90603	2.401352	0.715892	2.558184	0.089098	0.727496	0.28438	550.2031	3.836648	548.2391	10.83767	541.0284	53.6455	550.2	3.8	101.6958	725
19AR105_Spot 157	155.876	10757.8	1.831673	16.65272	2.742085	0.724356	3.108149	0.089367	0.868968	0.279577	551.7898	4.595397	553.2352	13.25811	560.1381	65.05544	551.8	4.6	98.5096	134
19AR105_Spot 464	197.2224	16737.65	74.03603	16.0266	3.850849	0.762143	3.982104	0.08955	0.741114	0.186111	552.8753	3.926645	575.2455	17.48963	665.548	83.79951	552.9	3.9	83.07068	405
19AR105_Spot 5	118.2024	7135.143	1.240126	15.18719	5.043651	0.790834	5.423523	0.090079	0.987132	0.182009	556.0019	5.258443	591.6448	24.32341	731.7639	113.0652	556.0	5.3	75.98105	5
19AR105_Spot 332	127.4586	8935.001	2.138575	15.97396	3.667576	0.756413	3.989402	0.090183	0.982911	0.246381	556.6165	5.241501	571.9388	17.44669	634.2734	83.26247	556.6	5.2	87.75656	286
19AR105_Spot 155	805.0105	4516.426	2.441313	15.39828	1.208061	0.772595	1.958075	0.090478	1.419945	0.725174	558.3657	7.594826	581.2505	8.66587	672.661	28.84116	558.4	7.6	83.0085	132
19AR105_Spot 276	505.8801	2562.324	3.593861	15.05119	1.273144	0.759321	1.719667	0.09072	0.805108	0.468177	559.7964	4.316819	573.6183	7.536375	629.6876	32.71767	559.8	4.3	88.90066	236
19AR105_Spot 101	220.4967	16614.64	3.104833	16.66688	3.442154	0.73964	3.544693	0.090795	0.647506	0.182669	560.2344	3.474392	562.1957	15.30392	571.1001	75.85281	560.2	3.5	98.09742	89
19AR105_Spot 75	157.335	12491.81	4.941056	16.61015	2.418593	0.74353	2.725878	0.091495	0.762912	0.279877	564.3755	4.122591	564.4632	11.80388	565.7434	56.98479	564.4	4.1	99.75822	67
19AR105_Part2_Spot 284	290.6083	29333.96	15.11306	16.34624	1.944469	0.765504	2.180226	0.091663	0.835543	0.383237	565.3668	4.522662	577.1806	9.598936	624.8813	43.41264	565.4	4.5	90.47588	750
19AR105_Part2_Spot 330	850.6991	24207.87	1.303023	16.72951	0.873364	0.746006	1.106248	0.091904	0.654086	0.591265	566.7849	3.548957	565.9044	4.799349	563.2935	19.45335	566.8	3.5	100.6198	790
19AR105_Spot 173	51.31938	4393.075	0.295865	13.37254	6.632312	0.914244	7.036671	0.091913	2.114055	0.300434	566.8424	11.47163	659.3112	34.13701	990.8269	136.6482	566.8	11.5	57.20902	149
19AR105_Spot 293	473.6116	31438.35	2.993205	16.94297	1.338999	0.742454	1.617902	0.091918	0.869649	0.537516	566.873	4.719266	563.8366	6.999987	552.5324	29.78725	566.9	4.7	102.5954	252
19AR105_Spot 454	129.1246	11280.13	1.797054	16.54084	4.029824	0.750998	4.220768	0.091933	0.910326	0.215678	566.961	4.940743	568.8031	18.38323	577.1412	89.60885	567.0	4.9	98.2361	395
19AR105_Spot 92	58.84085	8201.513	262.5496	14.62357	10.29833	0.846557	10.90268	0.092131	3.405894	0.31239	568.1265	18.52169	622.7576	50.79476	827.5033	216.6188	568.1	18.5	68.6555	81
19AR105_Part2_Spot 387	362.7823	1773.659	3.286281	14.37672	1.389488	0.775238	2.027816	0.092151	0.825746	0.40721	568.2474	4.491416	582.7634	8.991836	640.6996	39.83126	568.2	4.5	88.69171	836
19AR105_Spot 283	273.614	16581.8	1.744466	16.40451	2.41398	0.763413	2.612759	0.092167	0.720548	0.275781	568.3398	3.919831	575.9771	11.48559	607.145	54.30916	568.3	3.9	93.60857	243
19AR105_Spot 242	361.9907	20488.62	46.11643	17.13011	1.888245	0.742388	2.131094	0.092373	0.660767	0.31006	569.5535	3.60196	563.7984	9.219997	541.5748	44.31877	569.6	3.6	105.1662	206
19AR105_Part2_Spot 57	697.877	17555.44	89.41295	16.48899	0.990888	0.764982	1.585692	0.093407	0.652959	0.411782	575.6527	3.595839	576.88	6.978571	582.6551	31.37861	575.7	3.6	98.7982	559
19AR105_Part2_Spot 45	111.0558	10561.49	2.504647	15.75651	3.240883	0.799181	3.554664	0.093576	1.046441	0.294385	576.6496	5.77228	596.3667	16.03374	673.0342	72.68303	576.6	5.8	85.67909	548
19AR105_Spot 368	432.5638	19019.38	1.532025	16.67759	1.794157	0.768564	2.271361	0.093781	0.697314	0.307003	577.8574	3.854158	578.9388	10.02281	584.1086	46.95231	577.9	3.9	98.9298	316
19AR105_Part2_Spot 152	184.4357	9628.215	2.303424	15.52846	3.345265	0.815326	3.611018	0.094243	0.838093	0.232093	580.5842	4.653152	605.4372	16.46927	700.5055	74.8113	580.6	4.7	82.88076	638
19AR105_Part2_Spot 303	74.17851	8928.647	1.173756	14.18316	7.172031	0.890008	7.457251	0.094277	1.253613	0.168107	580.7813	6.96241	646.3736	35.67119	883.6748	152.1726	580.8	7.0	65.72342	767
19AR105_Spot 353	510.8941	16658.5	8.357652	16.3737	1.515168	0.785767	1.971884	0.094278	0.878986	0.44576	580.7867	4.881823	588.7679	8.810309	620.576	38.10966	580.8	4.9	93.58833	304
19AR105_Part2_Spot 169	263.7898	13386.91	1.539935	15.96274	1.757645	0.803872	2.383597	0.094802	0.781647	0.327928	583.8771	4.363279	599.0104	10.78599	657.6515	48.31323	583.9	4.4	88.78214	653
19AR105_Part2_Spot 398	246.7696	18837.4	5.367581	16.11755	2.620355	0.796704	2.758093	0.094887	0.711106	0.257825	584.3731	3.972725	594.9678	12.41884	636.5281	57.36731	584.4	4.0	91.80633	847
19AR105_Spot 397	227.1886	27642.99	2.122432	16.65371	2.831468	0.780356	2.947409	0.095046	0.77958	0.264497	585.3135	4.361966	585.6867	13.11839	588.0749	61.66781	585.3	4.4	99.53044	344
19AR105_Spot 573	137.916	13259.52	2.38143	16.41335	3.836288	0.790689	4.035897	0.095067	0.764867	0.189516	585.4333	4.28048	591.5624	18.09678	616.0619	85.59655	585.4	4.3	95.02833	494
19AR105_Part2_Spot 266	42.86618	3548.228	1.316585	13.50072	10.1458	0.915665	10.43736	0.095402	1.976611	0.189378	587.409	11.09751	660.0649	50.69889	917.8067	211.3203	587.4	11.1	64.00138	736
19AR105_Part2_Spot 260	638.4443	4665.67	1.694722	16.0541	1.440742	0.774907	1.774326	0.095526	0.732195	0.412661	588.1364	4.115704	582.5741	7.865856	561.8767	35.19944	588.1	4.1	104.6736	731
19AR105_Spot 588	1378.718	115823.9	12.98399	16.71849	0.76516	0.794447	1.070591	0.095606	0.743838	0.694792	588.6069	4.184347	593.6914	4.812726	614.0892	16.63908	588.6	4.2	95.85039	507
19AR105_Spot 485	349.7732	40053.91	2.579744	16.4939	1.906442	0.799943	2.148879	0.095651	0.803463	0.373899	588.8743	4.521718	596.7965	9.697403	627.9384	42.9329	588.9	4.5	93.77899	423
19AR105_Spot 525	248.2719	16825.28	2.798703	15.76416	3.726473	0.83505	3.92898	0.096023	0.897853	0.228521	591.0625	5.070851	616.4106	18.15602	711.552	81.3067	591.1	5.1	83.06666	459
19AR105_Spot 142	128.3793	11147.9	2.234456	16.86587	3.809824	0.772035	4.328302	0.096269	0.850769	0.19656	592.5045	4.816125	580.9299	19.14978	536.8773	92.92559	592.5	4.8	110.3613	122
19AR105_Part2_Spot 4	201.0887	17037.55	1.392213	15.97868	2.738634	0.818531	2.939149	0.09647	0.720879	0.245268	593.6861	4.088598	607.2285	13.43356	659.0462	61.09897	593.7	4.1	90.08261	519
19AR105_Part2_Spot 193	241.6808	9746.861	0.589663	16.48199	2.475477	0.789333	2.847817	0.096615	1.151702	0.404416	594.54	6.541062	590.7937	12.75657	577.3941	56.63539	594.5	6.5	102.9695	676
19AR105_Part2_Spot 312	52.22005	4864.365	1.89601	13.99247	8.104422	0.908466	8.6842	0.096699	1.316296	0.151574	595.0327	7.481784	656.2419	41.99829	873.6253	178.0932	595.0	7.5	68.11075	775
19AR105_Spot 42	293.6838	27477.27	5.997874	16.55558	2.088719	0.799945	2.227904	0.097043	0.748514	0.335972	597.0594	4.268358	596.7975	10.05406	596.725	45.47093	597.1	4.3	100.056	41
19AR105_Spot 310	147.8797	12525.33	14.50789	15.96697	4.631938	0.823092	4.927923	0.097307	0.873963	0.177349	598.6075	4.996057	609.7721	22.59464	652.4	104.1324	598.6	5.0	91.75467	266
19AR105_Spot 116	109.2125	12811.18	1.339457	16.31822	4.841904	0.807902	5.155104	0.097313	0.91825	0.178124	598.6433	5.249525	601.2766	23.39527	612.1375	109.6476	598.6	5.2	97.79555	99
19AR105_Part2_Spot 160	416.7042	12086.25	1.219876	16.28172	1.76192	0.806785	2.107734	0.097358	0.822524	0.390241	598.9091	4.704262	600.6488	9.556742	608.1411	41.94347	598.9	4.7	98.48193	644
19AR105_Part2_Spot 371	2459.307	21262.94	3.100984	16.37716	0.560361	0.804195	0.811525	0.097365	0.559772	0.689777	598.9463	3.201691	599.1923	3.672921	601.045	12.715	598.9	3.2	99.65083	824
19AR105_Part2_Spot 172	293.19	24726	2.084144	15.93392	2.327293	0.834466	2.450165	0.097447	0.741538	0.302648	599.4288	4.24459	616.087	11.31728	678.7002	49.93003	599.4	4.2	88.32012	656
19AR105_Spot 439	251.8281	23644.42	4.790753	16.89832	2.890349	0.793013	3.000496	0.09753	0.714508	0.23813	599.9172	4.093047	592.8793	13.47551	567.0051	63.47906	599.9	4.1	105.8045	382
19AR105_Part2_Spot 9	256.2732	9812.33	1.102896	16.6092	2.685459	0.789214	3.028719	0.097781	0.738359	0.243786	601.3939	4.239611	590.726	13.56586	550.9241	64.12099	601.4	4.2	109.1609	524
19AR105_Spot 78	569.986	31903.22	7.313384	16.3912	1.010833	0.815937	1.428896	0.097821	0.944554	0.661037	601.6242	5.425553	605.7794	6.519173	622.2721	23.13821	601.6	5.4	96.68185	70
19AR105_Part2_Spot 5	378.4984	28037.82	3.60788	16.57603	1.682856	0.804469	1.873082	0.097862	0.649832	0.346932	601.8665	3.734092	599.3464	8.479235	590.7448	38.08349	601.9	3.7	101.8827	520
19AR105_Spot 394	737.7019	7280.733	30.05261	15.92952	1.06167	0.82346	1.334206	0.098169	0.715001	0.5359	603.6695	4.120314	609.9771	6.117931	634.4309	24.24898	603.7	4.1	95.15134	341
19AR105_Spot 520	294.9874	17184.26	2.10555	15.98367	1.893013	0.841579	2.12718	0.09832	0.796452	0.374417	604.5584	4.596132	620.0164	9.870797	677.7904	42.13991	604.6	4.6	89.19548	454
19AR105_Spot 140	126.1335	10071.65	0.987578	16.24023	4.347799	0.821049	4.558699	0.098722	1.067668	0.234205	606.9153	6.184164	608.6335	20.87274	615.9535	95.74158	606.9	6.2	98.53265	120
19AR105_Spot 117	297.9126	36834.4	4.81764	16.08777	2.199877	0.844953	2.337211	0.099114	0.723889	0.309723	609.2138	4.208059	621.8751	10.86905	669.1433	47.56986	609.2	4.2	91.04384	100
19AR105_Part2_Spot 188	89.84204	7646.188	3.301882	15.17585	4.789641	0.878853	4.945895	0.099443	0.868969	0.175695	611.1425	5.066674	640.3628	23.49501	745.7508	102.9692	611.1	5.1	81.94995	671
19AR105_Part2_Spot 86	414.8929	25540.95	3.586358	16.45501	2.015196	0.824143	2.145454	0.099505	0.564702	0.263209	611.5072	3.294466	610.3573	9.842523	607.0132	44.78068	611.5	3.3	100.7403	582
19AR105_Spot 31	372.3707	26937.02	1.094816	16.38238	1.695571	0.830394	1.893073	0.099754	0.617711	0.326301	612.9676	3.611927	613.8308	8.720623	617.9348	38.63952	613.0	3.6	99.19617	31
19AR105_Spot 266	395.4432	41692.78	1.389996	16.45022	1.399796	0.831625	1.591744	0.099759	0.697389	0.438129	612.9982	4.078018	614.5135	7.338407	621.0199	30.87355	613.0	4.1	98.70831	227
19AR105_Part2_Spot 84	840.4854	13847.68	18.1987	16.21173	0.963608	0.832246	1.344155	0.099764	0.908465	0.675863	613.0257	5.312523	614.8574	6.199441	622.528	21.38908	613.0	5.3	98.4736	580
19AR105_Spot 594	529.0251	45713.76	2.878621	16.52296	1.38628	0.833075	1.527014	0.09983	0.594971	0.389631	613.4148	3.481379	615.3172	7.046668	623.2426	30.32389	613.4	3.5	98.42312	512
19AR105_Spot 113	483.2082	70761.81	4.137983	16.45629	1.299378	0.835211	1.486965	0.099919	0.66476	0.447058	613.9356	3.892882	616.4994	6.871437	626.8431	28.6697	613.9	3.9	97.94086	98

19AR105_Spot 112	429.2131	33467.98	5.279873	16.30141	1.16947	0.842269	1.394139	0.099971	0.670394	0.480866	614.2362	3.927712	620.3969	6.472015	643.8927	26.28292	614.2	3.9	95.39418	97
19AR105_Spot 461	151.5959	13593.1	3.029434	16.30329	2.736295	0.832103	3.247024	0.100001	1.067625	0.328801	614.4141	6.256739	614.7786	14.97525	617.0412	66.22124	614.4	6.3	99.57424	402
19AR105_Part2_Spot 223	507.5184	7818.727	1.800729	15.82284	2.003427	0.844621	2.120181	0.100096	0.581538	0.274287	614.9697	3.410999	621.6927	9.857602	647.1971	43.80226	615.0	3.4	95.02047	702
19AR105_Spot 469	148.6622	14983.63	4.108268	16.05537	4.069621	0.852367	4.402563	0.10031	0.693722	0.157572	616.2228	4.076911	625.9475	20.57285	662.1625	93.19253	616.2	4.1	93.06218	410
19AR105_Part2_Spot 104	133.9201	9819.055	1.454639	15.77194	4.651986	0.858367	5.120619	0.100316	0.74404	0.145303	616.2577	4.372856	629.2308	24.0201	677.0709	108.3441	616.3	4.4	91.01819	599
19AR105_Spot 532	138.4423	12392.75	0.580567	15.39025	4.62688	0.888304	4.774797	0.10038	0.793569	0.1662	616.6341	4.666666	645.4578	22.81117	748.5384	99.54293	616.6	4.7	82.37842	466
19AR105_Spot 479	192.2756	15327.85	0.874782	16.61069	3.03884	0.826901	3.360106	0.100444	0.811688	0.241566	617.0104	4.775989	611.8912	15.44382	593.9486	70.70427	617.0	4.8	103.8828	419
19AR105_Part2_Spot 40	324.7249	44625.11	2.339217	16.53761	1.822576	0.830846	1.963121	0.100546	0.648887	0.330538	617.6055	3.821574	614.0816	9.046016	602.0299	40.12418	617.6	3.8	102.5872	544
19AR105_Part2_Spot 395	400.0195	12019.58	3.797171	16.05698	1.528398	0.843647	2.190906	0.100809	0.698266	0.318711	619.1469	4.12217	621.1559	10.18008	629.3994	44.74264	619.1	4.1	98.37106	844
19AR105_Part2_Spot 374	469.653	38217.49	1.454173	16.44889	1.251404	0.83557	1.423924	0.10083	0.598897	0.420596	619.2697	3.53622	616.6981	6.58165	608.1885	27.9317	619.3	3.5	101.822	827
19AR105_Spot 34	217.6751	25648.01	0.464165	16.28177	3.171624	0.844715	3.239204	0.100869	0.612497	0.189089	619.4969	3.617781	621.7441	15.06194	630.89	68.52773	619.5	3.6	98.19412	34
19AR105_Spot 354	404.8773	21811.31	0.821265	16.49952	1.656567	0.837755	1.840616	0.100952	0.725665	0.394251	619.9866	4.289449	617.9058	8.519865	611.2109	36.56754	620.0	4.3	101.4358	305
19AR105_Part2_Spot 33	153.7187	13418.2	0.864388	16.46521	3.07735	0.830744	3.622257	0.101049	0.638467	0.176262	620.5528	3.777299	614.0248	16.69121	590.9462	77.33796	620.6	3.8	105.01	539
19AR105_Spot 179	176.6725	15680.18	4.356578	16.07198	3.646975	0.861061	3.888376	0.101052	0.760955	0.1957	620.5724	4.502104	630.702	18.26915	668.0987	81.64256	620.6	4.5	92.88634	154
19AR105_Part2_Spot 181	848.2257	102511.2	2.616658	16.61093	1.242043	0.8376	1.616247	0.10113	1.013999	0.627378	621.0259	6.003381	617.8206	7.480514	607.0149	27.24002	621.0	6.0	102.3082	664
19AR105_Spot 248	306.392	43435.56	2.112844	16.48949	2.110493	0.849554	2.313628	0.101149	0.841478	0.363705	621.1375	4.982823	624.4043	10.79105	637.2158	46.37987	621.1	5.0	97.47679	211
19AR105_Part2_Spot 3	126.6855	8946.861	0.820707	15.59606	5.475292	0.869417	6.067023	0.101204	0.823139	0.135674	621.4623	4.87666	635.2507	28.6578	685.5699	128.4264	621.5	4.9	90.64901	518
19AR105_Spot 467	158.2597	26746.36	1.683503	15.51279	3.251679	0.894905	3.461926	0.101215	0.874483	0.2526	621.5281	5.181366	649.0011	16.60259	746.6636	70.80803	621.5	5.2	83.24072	408
19AR105_Part2_Spot 165	107.2101	14341.37	2.470373	15.25471	3.228329	0.899161	3.644748	0.101229	0.914165	0.250817	621.6095	5.417163	651.279	17.52331	756.3905	74.48836	621.6	5.4	82.18104	649
19AR105_Part2_Spot 73	115.5931	12888.06	2.654768	15.68303	4.199245	0.875869	4.349141	0.101283	0.849165	0.195249	621.9209	5.034387	638.7492	20.62196	699.6428	90.87587	621.9	5.0	88.8912	573
19AR105_Part2_Spot 386	157.9452	21007.21	1.051559	16.23988	2.888942	0.849141	3.039414	0.101797	0.788499	0.259425	624.929	4.696251	624.1774	14.17287	622.3715	63.34424	624.9	4.7	100.4109	835
19AR105_Spot 246	117.793	13770.65	4.367167	16.53409	4.721133	0.843729	4.921613	0.101947	0.81528	0.165653	625.8074	4.862256	621.2012	22.87266	605.3776	105.0345	625.8	4.9	103.3747	209
19AR105_Spot 125	275.9907	28715.1	2.882846	16.42998	1.474543	0.854212	1.746048	0.102061	0.684392	0.391966	626.4765	4.085808	626.9582	8.167737	629.6132	34.62887	626.5	4.1	99.5018	108
19AR105_Part2_Spot 123	404.7692	26838.88	1.535069	16.43593	1.989825	0.848989	2.135449	0.102106	0.725056	0.339534	626.7368	4.330285	624.094	9.956362	615.4448	43.40025	626.7	4.3	101.8348	613
19AR105_Part2_Spot 372	189.2858	24183.08	2.99372	15.90473	2.134295	0.870681	2.225147	0.102203	0.603826	0.271365	627.3045	3.609367	635.9372	10.51632	667.6676	45.86072	627.3	3.6	93.95461	825
19AR105_Spot 41	143.2114	12127.27	3.646291	15.95638	3.220779	0.86737	3.402476	0.102478	0.874662	0.257066	628.9162	5.241072	634.1384	16.04853	653.7361	70.57429	628.9	5.2	96.20338	40
19AR105_Spot 577	115.3103	11126.86	2.190659	15.73986	4.769558	0.884335	4.985722	0.102546	1.068035	0.214219	629.3132	6.403633	643.3212	23.76271	693.7607	103.8691	629.3	6.4	90.71042	498
19AR105_Spot 330	181.8852	17819.44	0.78912	15.96314	4.149852	0.873177	4.32056	0.102672	0.646747	0.14969	630.0467	3.882011	637.291	20.45278	664.0169	91.53651	630.0	3.9	94.88413	284
19AR105_Spot 484	1822.891	225239.5	3.867862	16.43551	0.827357	0.866178	1.097757	0.102698	0.719499	0.655427	630.2006	4.319703	633.49	5.173612	646.2017	17.83332	630.2	4.3	97.52382	422
19AR105_Spot 45	619.6046	63099.98	2.581165	16.15499	0.907999	0.872347	1.125059	0.102752	0.647716	0.575717	630.5191	3.890601	636.8407	5.322454	660.2513	19.69772	630.5	3.9	95.49683	43
19AR105_Spot 441	478.2591	51979.48	4.926996	16.67951	1.471414	0.851848	1.666981	0.102815	0.730268	0.438078	630.8861	4.388893	625.6628	7.786206	607.7436	32.40916	630.9	4.4	103.8079	384
19AR105_Part2_Spot 254	292.443	41446.54	1.309415	16.62407	1.767378	0.845293	1.929318	0.103059	0.725944	0.37627	632.3114	4.372287	622.0622	8.974019	585.919	38.79574	632.3	4.4	107.9179	728
19AR105_Spot 81	445.8539	47925.17	4.796573	16.46338	1.379944	0.859002	1.663951	0.103167	0.882036	0.530085	632.9415	5.317455	629.5778	7.807165	618.4342	30.46804	632.9	5.3	102.3458	71
19AR105_Spot 323	151.4437	13872.62	4.135284	16.42698	3.844353	0.850443	3.945764	0.103251	0.742464	0.188167	633.4326	4.479332	624.892	18.41527	595.0487	83.99205	633.4	4.5	106.4505	277
19AR105_Spot 483	127.5459	12600.52	2.902915	15.96158	3.902447	0.882807	4.057517	0.103415	0.844741	0.208192	634.39	5.103706	642.4978	19.3198	672.0571	84.90998	634.4	5.1	94.39524	421
19AR105_Spot 512	166.7962	12312.58	2.934969	15.94131	3.447038	0.881912	3.5757	0.103436	0.661464	0.184989	634.5128	3.99713	642.0147	17.016	669.4102	75.21544	634.5	4.0	94.78684	447
19AR105_Spot 600	538.3999	27146.59	0.597483	16.1382	1.610208	0.882623	1.733342	0.103681	0.604917	0.348989	635.9438	3.663267	642.3983	8.251548	666.1032	34.76646	635.9	3.7	95.47226	516
19AR105_Spot 389	364.8075	38883.43	15.22755	16.25244	1.908785	0.875062	2.064062	0.103695	0.742321	0.359641	636.0257	4.495918	638.3123	9.781136	647.3701	41.38083	636.0	4.5	98.24762	336
19AR105_Spot 76	333.3255	30440.43	0.795985	16.45624	2.013635	0.861872	2.155381	0.103863	0.678329	0.314714	637.0051	4.114365	631.1443	10.13121	611.1254	44.21621	637.0	4.1	104.2348	68
19AR105_Spot 426	363.7809	46697.44	2.110881	16.83678	1.79362	0.85361	1.985408	0.103918	0.817639	0.411824	637.3268	4.961721	626.6287	9.283956	589.1318	39.24636	637.3	5.0	108.1807	371
19AR105_Spot 517	755.742	73864.6	1.724037	16.54174	0.894452	0.870641	1.133123	0.104372	0.661587	0.583861	639.979	4.030633	635.9154	5.355004	622.4175	19.86451	640.0	4.0	102.8215	451
19AR105_Spot 232	116.3979	2789.623	1.2124	14.55724	3.860783	0.924255	6.589338	0.104494	1.591126	0.24147	640.6913	9.704002	664.6076	32.1474	747.4942	135.2442	640.7	9.7	85.71186	199
19AR105_Spot 57	364.7061	32716.79	2.282093	16.03165	1.324861	0.89145	1.521209	0.104724	0.716894	0.471266	642.0353	4.380934	647.148	7.279947	665.9686	28.71269	642.0	4.4	96.40625	53
19AR105_Spot 592	193.2619	17907.7	1.43299	16.61622	1.993508	0.863745	2.239072	0.104779	0.687522	0.307057	642.3526	4.203412	632.1654	10.5369	596.864	46.17671	642.4	4.2	107.6213	510
19AR105_Part2_Spot 13	214.7266	6970.323	5.564597	14.74648	2.35505	0.950044	2.725219	0.105034	0.660657	0.242424	643.8405	4.048065	678.1253	13.48205	794.5799	55.455	643.8	4.0	81.02905	526
19AR105_Spot 82	199.7625	27513.2	3.037564	16.35877	1.534278	0.878762	1.871261	0.105254	0.965655	0.516045	645.1233	5.928095	640.314	8.887404	624.2998	34.57581	645.1	5.9	103.3355	72
19AR105_Part2_Spot 176	205.9338	18369.01	1.078916	15.97071	2.546325	0.90837	2.775758	0.106541	1.03801	0.373956	652.6258	6.442698	656.1906	13.41643	669.384	55.10597	652.6	6.4	97.49648	660
19AR105_Spot 504	295.4753	50823.05	1.482966	16.53161	1.725854	0.891343	1.886238	0.106895	0.720942	0.382211	654.692	4.488184	647.0904	9.026355	621.575	37.61832	654.7	4.5	105.3279	440
19AR105_Spot 494	578.8671	60921.27	4.438379	16.36736	1.252917	0.909584	1.453479	0.107631	0.704419	0.484644	658.9726	4.412557	656.8363	7.029907	650.4404	27.31713	659.0	4.4	101.3118	431
19AR105_Spot 489	219.9109	20754.82	1.777129	15.63836	1.895435	0.951577	2.474507	0.108239	1.554867	0.628354	662.5109	9.789515	678.9236	12.25175	734.6791	40.74982	662.5	9.8	90.1769	426
19AR105_Spot 465	395.6161	50464.98	3.314143	16.36999	1.510477	0.916734	1.706597	0.109011	0.690057	0.404347	667.0006	4.372574	660.6314	8.288044	639.9073	33.57119	667.0	4.4	104.2339	406
19AR105_Part2_Spot 308	1094.543	14094.19	6.533358	15.60411	0.675019	0.951109	1.6739	0.110026	0.815979	0.487472	672.8985	5.213858	678.6798	8.285483	698.8543	31.1409	672.9	5.2	96.28594	772
19AR105_Spot 123	447.5262	48017.28	2.246653	16.40887	1.777052	0.92292	1.927879	0.110067	0.596219	0.309262	673.1395	3.810955	663.903	9.39561	633.6215	39.47303	673.1	3.8	106.2368	106
19AR105_Spot 213	252.7158	26181.77	5.193353	15.05206	1.922683	1.011978	2.198109	0.111194	1.007572	0.458381	679.6807	6.499612	709.8728	11.22651	807.4729	40.88728	679.7	6.5	84.17381	182
19AR105_Spot 367	357.4641	2704.847	5.090417	14.34769	1.707429	0.993349	1.960606	0.111408	0.737295	0.376055	680.9213	4.764348	700.4274	9.920934	764.4197	38.28581	680.9	4.8	89.0769	315
19AR105_Part2_Spot 276	110.1216	10769.14	3.365697	15.74376	4.24868	0.951893	4.796285	0.111456	0.809396	0.168755	681.1985	5.23228	679.0879	23.75452	673.0417	101.1498	681.2	5.2	101.2119	744
19AR105_Part2_Spot 275	133.2022	15994.22	2.488522	15.6221	3.992199	0.966894	4.140627	0.111538	0.798999	0.192966	681.6726	5.168477	686.8618	20.67064	704.841	86.48207	681.7	5.2	96.71296	743
19AR105_Spot 436	331.3374	34662.77	2.236587	16.02898	1.788199	0.961113	1.984012	0.111569	0.664451	0.334903	681.8525	4.299202	683.8729	9.873244	691.4753	39.86858	681.9	4.3	98.60837	379
19AR105_Spot 187	134.3778	1092.113	5.784649	12.52923	6.536367	1.040263	16.10466	0.112003	1.58726	0.098559	684.3718	10.30603	724.0483	83.56365	849.8921	335.1416	684.4	10.3	80.52455	160
19AR105_Part2_Spot 168	300.1407	42277.53	2.954447	15.52377	1.730166	0.988874	1.900364	0.112111	0.742166	0.390539	684.9937	4.82301	698.1453	9.594297	741.6193	36.98852	685.0	4.8	92.3646	652
19AR105_Spot 453	187.2068	5722.784	4.399936	14.78008	3.959448	1.011104	5.102035	0.112424	0.960185	0.188196	686.8098	6.255494	709.4319	26.05134	782.6034	105.347	686.8	6.3	87.75962	394
19AR105_Spot 188	317.7647	25365.3	3.080454	16.00262	1.424715	0.969246	1.692527	0.112645	0.82259	0.486013	688.0906	5.36855	688.0752	8.458818	688.9531	31.54908	688.1	5.4	99.87481	161
19AR105_Part2_Spot 224	563.2144	15449.93	4.279277	15.14208	0.885206	1.008214	1.790205	0.112694	1.011763	0.565166	688.3756	6.605761	707.9717	9.126141	771.5335	31.08796	688.4	6.6	89.22175	703
19AR105_Part2_Spot 90	455.9367	14338.79	3.615543	15.46746	1.062218	0.998221	1.685462	0.113845	0.620319	0.368041	695.0408	4.08718	702.9062	8.54953	729.0704	33.21236	695.0	4.1	95.33247	585
19AR105_Part2_Spot 396	159.2406	17323.61	3.65891	15.83001	3.269259	0.978493	3.415523	0.114554	0.845086	0.247425	699.1386	5.599207	692.8321	17.15346	673.3359	70.79858	699.1	5.6	103.8321	845
19AR105_Spot 307	429.7257	37543.14	4.585683	15.83951	1.286075	1.000011	1.443986	0.115823	0.636544	0.440824	706.4774	4.259378	703.8154	7.331159	696.2391	27.60789	706.5	4.3	101.4705	263
19AR105_Part2_Spot 397	540.127	17783.45	4.609642	15.67482	0.821089	1.001735	1.096592	0.116069	0.675836	0.616306	707.8955	4.530886	704.6902	5.572185	695.4216	18.39289	707.9	4.5	101.7937	846
19AR105_Spot 488	419.0411	59442.09	15.12066	14.6873	1.41405	1.099312	1.635422	0.116651	0.774303	0.473457	711.2601	5.214359	753.0179	8.695897	880.0271	29.78389	711.3	5.2	80.82252	425
19AR105_Spot 281	154.6105	16276.22	3.027772	15.21476	3.053303	1.05141	3.195648	0.11768	0.811318	0.253882	717.1984	5.506756	729.5805	16.63211	768.7073	65.1205	717.2	5.5	93.29928	241
19AR105_Spot 102	292.573	35485.26	7.617599	14.95005	2.17415	1.080276	2.318494	0.118099	0.783714	0.338027	719.6105	5.336303	743.7688	12.22561	818.1153	45.61297	719.6	5.3	87.95954	90
19AR105_Part2_Spot 306	502.0088	59489.68	1.099983	15.64317	1.170987	1.035827	1.331986	0.118715	0.621632	0.466696	723.1624	4.252431	721.8379	6.881489	718.6276	24.99028	723.2	4.3	100.631	770
19AR105_Spot 40	190.844	29248.8	2.063688	14.82657	2.042615	1.099569	2.483927	0.119286	1.308788	0.526903	726.4536	8.991597	753.1421	13.20948	834.1536	44.0045	726.5	9.0	87.08871	39
19AR105_Spot 222	55.87156	6900.843	5.204838	14.14522	5.64007	1.132367	6.202487	0.1193	2.20567	0.355611	726.5345	15.15494	768.8811	33.45632	894.8456	119.751	726.5	15.2	81.19105	190
19AR105_Spot 451	475.7841	27416.75	3.283239	15.70094	1.347192	1.045654	1.594411	0.119948	0.629874	0.395051	730.2664	4.34879	726.7274	8.275526	716.7286	31.08718	730.3	4.3	101.8888	392
19AR105_Part2_Spot 367	220.6499	23849.2	10.35107	15.36892	2.277751	1.063578	2.40729	0.120429	0.606067	0.251763	733.0341	4.199391	735.5853	12.59879	744.2626	49.24521	733.0	4.2	98.49133	821
19AR105_Spot 63	218.3271	25546.41	4.55963	14.7572	2.257409	1.117753	2.46076	0.120963	0.954633	0.387942	736.1069	6.640757	761.8984	13.18846	839.2146	47.23222	736.1	6.6	87.71378	58
19AR105_Spot 18	676.758	72517.09	4.588184	14.54307	0.713545	1.1419	1.090223	0.12103	0.82075	0.752828	736.4891	5.712218	773.4103	5.901723	882.4637	14.83559	736.5	5.7	83.45829	18
19AR105_Spot 510	792.5202	18354.79	3.335792	15.06585	0.850395	1.101529	1.14601	0.121055	0.743167	0.648482	736.6319	5.173204	754.0895	6.099357	807.1149	18.27827	736.6	5.2	91.26729	446
19AR105_Spot 582	340.7292	15894.79	3.389311	14.57166	1.217483	1.143871	1.506811	0.121528	0.838133	0.556229	739.3527	5.854604	774.3444	8.163504	877.5251	25.8982	739.4	5.9	84.25431	502
19AR105_Spot 259	238.5111	30770.07	3.515524	15.58267	2.038773	1.070337	2.113751	0.121688	0.543843	0.257288	740.2724	3.80336	738.9059	11.09636	735.7071	43.24855	740.3	3.8	100.6205	221
19AR105_Part2_Spot 400	335.7279	42048.33	3.827703	15.22909	1.605643	1.101255	1.790532	0.123142	0.617337	0.344779	748.6218	4.363265	753.9571	9.528704	770.7138	35.36439	748.6	4.4	97.13357	849
19AR105_Part2_Spot 183	1236.4	151817.6	5.165067	15.35115	0.756284	1.117837	1.03315	0.124656	0.700528	0.67805	757.3042	5.005364	761.9385	5.537113	776.4524	15.97129	757.3	5.0	97.53389	666
19AR105_Part2_Spot 229	142.3481	15996.88	5.402875	14.39189	3.215478	1.18011	3.361636	0.125335	0.683857	0.20343	761.1949	4.9099	791.3643	18.47875	878.2321	68.14902	761.2	4.9	86.67354	707
19AR105_Part2_Spot 338	206.9817	15909.14	3.056095	14.78805	1.262631	1.154669	1.602964	0.125852	0.766087	0.477919	764.1583	5.520457	779.4459	8.722507	824.3636	29.38999	764.2	5.5	92.69675	797
19AR105_Part2_Spot 289	855.0257	86918.34	41.57885	15.16834	0.827584	1.138945	1.311747	0.125868	0.991182	0.75562	764.2498	7.143313	772.0085	7.092348	795.4118	18.03623	764.2	7.1	96.08228	754
19AR105_Spot 257	98.99038	2130.316	1.717201	13.87172	4.385266	1.1421	4.678711	0.12635	0.684929	0.146393	767.0086	4.952964	773.5052	25.3344	793.199	97.12349	767.0	5.0	96.69814	219
19AR105_Spot 17	388.4256	54442.99	5.223749	15.29702	1.213989	1.157556	1.412228	0.12911	0.699035	0.494987	782.7842	5.152756	780.8054	7.693485	776.0488	25.81055	782.8	5.2	100.8679	17
19AR105_Part2_Spot 70	79.98535	6008.49	9.42193	14.60307	5.053619	1.181339	5.447845	0.129434	0.871324	0.159939	784.6385	6.43705	791.9365	29.96622	813.4669	112.5113	784.6	6.4	96.45611	571
19AR105_Part2_Spot 118	174.3099	27379.33	4.073724	14.74095	2.190693	1.227825	2.365135	0.132364	0.883295	0.373465	801.3361	6.655907	813.3479	13.23628	847.2436	45.63726	801.3	6.7	94.58155	609
19AR105_Part2_Spot 138	133.5207	8023.934	3.588102	14.52839	1.683177	1.220634	2.17276	0.132404	0.99096	0.456083	801.5665	7.469211	810.0652	12.12748	834.396	40.31433	801.6	7.5	96.06548	627
19AR105_Spot 458	289.8027	7697.611	3.336019	13.61221	1.861338	1.311207	2.135823	0.13263	0.863231	0.404168	802.8543	6.51629	850.6576	12.30407	978.3682	39.80962	802.9	6.5	82.06055	399
19AR105_Spot 291	122.6152	14932.51	5.86863	14.5916	3.349078	1.237917	3.680695	0.132818	0.828782	0.22517	803.9209	6.264048	817.9373	20.67604	857.1374	74.48312	803.9	6.3	93.79137	250
19AR105_Spot 396	272.0147	10985.62	2.076363	15.22865	1.853044	1.180861	2.224908	0.133026	0.765019	0.343843	805.1064	5.790122	791.7144	12.23303	755.0831	44.09455	805.1	5.8	106.6249	343
19AR105_Spot 567	373.6412	49095.47	2.850685	14.9247	0.968765	1.231601	1.231294	0.133305	0.730943	0.593638	806.6942	5.542456	815.0675	6.900043	838.8827	20.61418	806.7	5.5	96.16294	489
19AR105_Part2_Spot 212	89.94804	4277.552	2.624039	13.83787	4.2974	1.28232	5.38583	0.135221	1.183785	0.219796	817.5835	9.089836	837.8866	30.73513	892.9837	108.5512	817.6	9.1	91.55638	694
19AR105_Spot 23	259.6597	41128.76	3.46764	14.10364	1.479195	1.321044	2.202749	0.136021	1.629686	0.739842	822.1236	12.5789	854.9699	12.73068	942.0723	30.36775	822.1	12.6	87.26757	23
19AR105_Part2_Spot 109	139.921	17920.94	1.788184	15.27416	3.061577	1.223002	3.240073	0.136779	1.018461	0.314333	826.4231	7.899626	811.1475	18.10165	770.3522	64.76428	826.4	7.9	107.2786	603
19AR105_Part2_Spot 137	149.1875	21449.26	3.004584	14.66213	2.530794	1.268758	2.679267	0.136813	0.836622	0.312258	826.6132	6.490597	831.8348	15.2149	846.6994	52.94489	826.6	6.5	97.6277	626
19AR105_Part2_Spot 48	473.1029	52946.85	13.26839	14.76987	1.015486	1.275294	1.234011	0.137507	0.67616	0.547937	830.5517	5.269139	834.7557	7.023098	846.8499	21.45044	830.6	5.3	98.07543	551
19AR105_Spot 555	342.7439	57663.18	5.671014	14.66118	1.136456	1.305691	1.514411	0.138231	0.996102	0.657749	834.6532	7.798271	848.2313	8.708108	884.8386	23.58312	834.7	7.8	94.3283	481
19AR105_Spot 226	182.8924	8944.386	3.395486	13.98669	1.766304	1.34491	2.485405	0.139101	0.82104	0.330345	839.5787	6.463259	865.3571	14.47516	932.8449	48.15999	839.6	6.5	90.00196	193
19AR105_Spot 221	317.534	13201.9	7.303633	14.01746	1.169619	1.358042	1.49996	0.139742	0.894045	0.596046	843.2037	7.0664	871.0275	8.771665	943.3298	24.67025	843.2	7.1	89.38589	189
19AR105_Spot 505	57.29716	5623.905	3.332975	13.72629	5.811983	1.365661	6.645027	0.140236	2.072966	0.311958	845.9975	16.43525	874.3032	38.96998	947.5602	129.3515	846.0	16.4	89.28166	441
19AR105_Part2_Spot 389	93.18318	9558.605	2.509846	14.86169	4.267049	1.280915	4.465577	0.142134	0.868245	0.194431	856.7168	6.965319	837.2611	25.46887	786.888	92.00975	856.7	7.0	108.874	838
19AR105_Part2_Spot 112	34.63567	4734.284	2.662349	13.71354	7.668226	1.373969	8.414488	0.142297	1.444036	0.171613	857.6397	11.59616	877.8629	49.48838	930.0828	170.4626	857.6	11.6	92.21111	605
19AR105_Spot 344	232.0651	47371.27	5.092972	14.45657	1.63309	1.390399	1.848623	0.146425	0.800582	0.433069	880.8908	6.591633	884.8662	10.91853	895.7266	34.39218	880.9	6.6	98.34372	297
19AR105_Part2_Spot 121	47.87194	6987.089	1.136365	14.1057	7.040789	1.392758	7.172652	0.146792	0.910372	0.126923	882.9534	7.511971	885.8675	42.41691	894.0303	147.0442	883.0	7.5	98.76101	612
19AR105_Part2_Spot 339	52.91182	8500.41	2.204496	14.21707	5.506075	1.500848	5.661607	0.158889	0.885533	0.15641	950.5971	7.826615	930.7303	34.51327	884.8719	115.6875	884.9	115.7	107.4276	798
19AR105_Spot 335	135.8481	22388.71	6.535008	14.37394	2.609947	1.477068	2.871633	0.155518	0.848503	0.295477	931.8191	7.361644	921.0292	17.38857	896.1582	56.63116	896.2	56.6	103.9793	288
19AR105_Spot 539	185.507	20596.45	6.576319	14.53715	2.035485	1.419188	2.194991	0.149709	0.813165	0.370464	899.3296	6.825837	897.022	13.07546	892.2362	42.08532	899.3	6.8	100.795	472
19AR105_Part2_Spot 336	78.35102	12602.58	1.737799	14.18924	5.751318	1.581075	5.871454	0.165925	0.841313	0.143289	989.6182	7.71821	962.7924	36.53548	902.899	119.8846	902.9	119.9	109.6045	795
19AR105_Part2_Spot 120	155.145	11646.17	2.43601	14.09288	2.097781	1.464389	2.996309	0.152382	1.71233	0.57148	914.3007	14.59631	915.8184	18.08046	920.3926	50.54428	920.4	50.5	99.33811	611
19AR105_Spot 229	216.9723	30895.31	2.148999	14.35376	1.80727	1.405126	1.981098	0.145827	0.781841	0.39465	877.5314	6.414409	891.1024	11.75257	925.8218	37.39543	925.8	37.4	94.78405	196
19AR105_Part2_Spot 166	731.6834	18593.59	2.138055	14.09311	0.743972	1.402789	1.125143	0.145242	0.815624	0.724907	874.2355	6.66811	890.1152	6.669917	930.651	15.90364	930.7	15.9	93.93806	650
19AR105_Part2_Spot 210	464.7613	7731.268	3.644367	13.90975	0.969413	1.424049	1.439979	0.147412	0.883794	0.613755	886.4378	7.319512	899.0601	8.58974	931.0898	23.33395	931.1	23.3	95.20433	692
19AR105_Spot 216	258.2998	53768.49	2.742947	14.211	1.128686	1.475093	1.432534	0.152335	0.867733	0.605733	914.0408	7.394796	920.2195	8.669085	935.9604	23.37782	936.0	23.4	97.65807	184
19AR105_Part2_Spot 242	423.9538	45837.33	1.380045	14.10667	0.846957	1.465302	1.023225	0.15117	0.565244	0.552414	907.5165	4.784975	916.1945	6.175376	938.0293	17.46806	938.0	17.5	96.74714	718
19AR105_Part2_Spot 139	251.8056	28957.97	6.695158	14.08951	1.258863	1.505208	1.551542	0.155186	0.894246	0.57636	929.9701	7.744205	932.4991	9.465829	939.3541	26.01049	939.4	26.0	99.00102	628
19AR105_Spot 518	390.9724	36490.23	2.031285	14.19849	0.996613	1.477174	1.234208	0.152157	0.663729	0.537777	913.0419	5.650522	921.0728	7.47311	941.2679	21.324	941.3	21.3	97.00127	452
19AR105_Spot 381	40.86073	5074.003	2.349673	13.67451	7.825409	1.602018	8.078598	0.164935	1.270242	0.157235	984.1443	11.59355	970.9979	50.54539	942.2664	163.7104	942.3	163.7	104.4444	329
19AR105_Part2_Spot 227	351.2678	37063.4	3.148194	14.0693	1.193288	1.385938	1.619434	0.142684	1.070904	0.661283	859.8207	8.620202	882.9695	9.551917	942.3095	24.87516	942.3	24.9	91.24611	705
19AR105_Spot 514	101.7164	15629.86	1.562914	14.06126	3.583782	1.692568	3.693426	0.174156	0.772496	0.209154	1034.972	7.386324	1005.732	23.57853	943.4668	73.99067	943.5	74.0	109.6988	448
19AR105_Part2_Spot 226	109.3742	16504.05	1.96429	13.96381	2.242991	1.666192	2.436742	0.171397	0.615549	0.252611	1019.803	5.806035	995.7365	15.46346	943.9954	48.28242	944.0	48.3	108.0306	704
19AR105_Spot 26	257.2242	55248.15	3.006382	14.09973	1.020293	1.487461	1.292353	0.152797	0.765744	0.592519	916.6216	6.542791	925.2806	7.847098	946.8528	21.30432	946.9	21.3	96.80719	26
19AR105_Spot 324	46.79903	7658.988	3.151463	13.76277	4.199359	1.672427	4.600309	0.171755	0.829628	0.180342	1021.775	7.83926	998.1082	29.24005	947.3448	92.68274	947.3	92.7	107.8567	278
19AR105_Spot 333	108.6444	18194.01	2.566283	13.99112	2.871232	1.563613	2.978621	0.160536	0.748324	0.251232	959.7535	6.673002	955.8996	18.44887	947.913	59.02472	947.9	59.0	101.2491	287
19AR105_Spot 470	292.2192	14513.71	4.25714	14.00427	1.374231	1.389814	1.556321	0.142417	0.701647	0.450837	858.3151	5.638635	884.6174	9.19038	951.8975	28.40338	951.9	28.4	90.16887	411
19AR105_Spot 366	218.553	32766.84	2.706102	14.09384	1.588922	1.60045	1.781671	0.163863	0.777813	0.436564	978.2107	7.059487	970.3859	11.13444	953.6177	32.78719	953.6	32.8	102.5789	314
19AR105_Spot 409	312.8293	30186.99	1.325429	14.02317	2.015634	1.498249	2.170246	0.153387	0.775848	0.357493	919.9221	6.651331	929.6747	13.21635	953.7818	41.46632	953.8	41.5	96.44996	356
19AR105_Part2_Spot 262	217.268	27671.29	1.351687	13.92427	1.615907	1.672961	2.32673	0.17125	1.65854	0.71282	1018.993	15.63239	998.3114	14.78767	954.0542	33.36436	954.1	33.4	106.8067	732
19AR105_Spot 296	107.4194	15337.11	1.267183	13.92165	2.235326	1.593613	2.514083	0.163027	0.952456	0.378848	973.5769	8.606634	967.7129	15.68634	955.2881	47.58238	955.3	47.6	101.9145	254
19AR105_Spot 509	84.70601	13221.7	2.94828	13.94542	5.46376	1.699428	5.530261	0.173445	0.771933	0.139583	1031.064	7.355239	1008.316	35.36569	960.118	111.9723	960.1	112.0	107.3893	445
19AR105_Part2_Spot 38	86.03981	10976.24	3.259946	13.77604	3.440539	1.622367	3.653189	0.165335	1.144877	0.313391	986.3567	10.47108	978.9077	22.95258	963.1428	70.85795	963.1	70.9	102.4102	542
19AR105_Part2_Spot 175	268.8549	43829.79	2.649172	13.98849	1.458587	1.449175	1.796814	0.147678	0.99782	0.555328	887.9316	8.276856	909.5305	10.79569	963.2454	30.51447	963.2	30.5	92.18125	659
19AR105_Spot 580	105.2407	23806.58	2.367705	14.04211	3.599863	1.702018	3.699723	0.173426	0.781704	0.211287	1030.959	7.447637	1009.29	23.66757	963.455	73.87496	963.5	73.9	107.0064	501
19AR105_Part2_Spot 383	460.6198	73979.44	5.457082	13.94079	0.993245	1.590663	1.296149	0.162071	0.826536	0.637686	968.2783	7.431115	966.5571	8.080929	963.5528	20.39086	963.6	20.4	100.4904	833
19AR105_Spot 444	179.1292	34800.12	3.072171	14.03572	1.166908	1.651774	1.494618	0.168192	0.809624	0.541693	1002.139	7.514353	990.2308	9.453374	964.8849	25.67146	964.8	25.7	103.8653	387
19AR105_Part2_Spot 341	172.2669	36238.21	3.196794	13.93207	1.835902	1.407135	2.282825	0.143273	1.260881	0.552334	863.1432	10.18607	891.9504	13.55076	964.965	38.84014	965.0	38.8	89.44813	800
19AR105_Spot 346	310.427	48833.44	2.478122	13.98411	1.381555	1.651982	1.566283	0.168063	0.640243	0.408766	1001.43	5.938394	990.3104	9.907146	966.6291	29.1834	966.6	29.2	103.6002	298
19AR105_Spot 215	236.5123	46019.74	2.047358	13.99091	1.358065	1.577823	1.546268	0.160511	0.708345	0.4581	959.6167	6.315668	961.512	9.610208	966.7395	28.06708	966.7	28.1	99.26321	183
19AR105_Part2_Spot 334	67.28576	10230.52	2.667301	13.7122	5.270207	1.629614	5.509976	0.165777	0.756135	0.13723	988.8014	6.931486	981.7099	34.68496	966.7648	111.5025	966.8	111.5	102.2794	794
19AR105_Part2_Spot 295	131.3366	21276.02	0.573633	13.81351	2.44252	1.620605	2.608717	0.164739	0.773536	0.29652	983.061	7.052893	978.2255	16.38213	968.2843	50.82989	968.3	50.8	101.5261	759
19AR105_Spot 178	174.9618	29675.06	1.95245	13.99873	1.892588	1.545656	2.105213	0.15701	0.878946	0.417509	940.137	7.688997	948.7622	12.97964	969.7293	39.03343	969.7	39.0	96.9484	153
19AR105_Spot 434	120.3759	18839.51	1.12934	13.95929	2.158905	1.674582	2.274712	0.169957	0.65974	0.290032	1011.875	6.178182	998.9268	14.46226	971.4936	44.40777	971.5	44.4	104.1567	377
19AR105_Spot 556	94.72644	20578.42	3.374344	13.96448	2.44495	1.705426	2.683859	0.17302	0.736381	0.274374	1028.728	7.001823	1010.57	17.18021	972.2814	52.62478	972.3	52.6	105.8056	482
19AR105_Spot 312	106.7822	16483.06	1.516802	13.79427	2.330032	1.758768	2.551599	0.178308	0.665021	0.260629	1057.726	6.487329	1030.395	16.51863	973.7096	50.21766	973.7	50.2	108.6285	268
19AR105_Part2_Spot 377	98.15933	10857.79	1.914804	13.65322	2.329005	1.758731	2.795726	0.178256	0.952555	0.340718	1057.44	9.289936	1030.381	18.09925	974.2658	53.57999	974.3	53.6	108.5371	830
19AR105_Spot 284	214.2255	38143.19	1.548912	13.90385	1.382515	1.545574	1.575124	0.156612	0.69439	0.440848	937.9186	6.061194	948.7295	9.710956	974.7779	28.82456	974.8	28.8	96.2187	244
19AR105_Spot 337	93.86266	11041.89	2.952372	13.71124	4.717188	1.567882	4.860581	0.158835	1.036725	0.213292	950.2971	9.160214	957.5891	30.14283	975.258	96.87419	975.3	96.9	97.44059	290
19AR105_Part2_Spot 204	113.0891	14067.25	3.394073	13.76974	2.743666	1.693803	2.854031	0.171502	0.763122	0.267384	1020.38	7.201743	1006.198	18.22352	976.3455	56.04348	976.3	56.0	104.5101	686
19AR105_Spot 10	85.17152	16560.05	2.540913	13.76281	4.789526	1.586201	4.903773	0.160556	0.743948	0.151709	959.8649	6.634697	964.8067	30.54831	976.9645	98.85261	977.0	98.9	98.24973	10
19AR105_Spot 131	138.3528	24702.71	3.085773	13.86923	1.769385	1.742225	2.071008	0.176282	0.743057	0.35879	1046.629	7.178548	1024.288	13.36097	977.7251	39.39573	977.7	39.4	107.0474	114
19AR105_Spot 566	153.8437	32791.68	0.562617	13.92563	1.752658	1.641005	2.003148	0.166011	0.870186	0.434409	990.0946	7.986651	986.0988	12.63881	978.1201	36.7485	978.1	36.7	101.2242	488
19AR105_Spot 564	122.8734	24075.61	2.038586	13.88397	2.360777	1.691873	2.479923	0.171018	0.72659	0.292989	1017.716	6.840464	1005.47	15.82765	979.7414	48.29002	979.7	48.3	103.876	487

19AR105_Spot 435	116.934	17612.94	1.915074	13.88904	2.461566	1.722415	2.764793	0.174051	0.649224	0.234818	1034.391	6.204432	1016.926	17.76316	980.3751	54.75168	980.4	54.8	105.5097	378
19AR105_Spot 53	171.592	32614.87	0.754431	13.81227	2.18862	1.653875	2.370824	0.167022	0.795871	0.335694	995.6833	7.342706	991.0349	15.00317	981.6648	45.47223	981.7	45.5	101.428	49
19AR105_Spot 30	83.40269	16351.57	1.861966	13.71434	3.246237	1.739083	3.352347	0.175448	0.760293	0.226794	1042.059	7.315503	1023.123	21.61521	983.7432	66.48796	983.7	66.5	105.9279	30
19AR105_Spot 19	259.3799	48959.45	1.993856	13.82148	0.942118	1.479276	1.28349	0.149183	0.772578	0.601935	896.3824	6.465331	921.9338	7.775984	984.4727	20.84487	984.5	20.8	91.05203	19
19AR105_Part2_Spot 264	588.1276	14907.64	10.86776	13.62765	0.764478	1.517339	1.273454	0.152886	0.99732	0.783161	917.1185	8.525763	937.4042	7.794043	986.2569	16.09768	986.3	16.1	92.98981	734
19AR105_Spot 423	65.0543	16235.08	0.971835	13.81182	5.506273	1.707657	5.620796	0.17205	0.857351	0.152532	1023.396	8.113075	1011.406	36.00947	986.3959	113.1302	986.4	113.1	103.7511	368
19AR105_Part2_Spot 233	139.2649	32028.38	1.746907	13.73699	1.870818	1.694174	2.0446	0.170481	0.765872	0.374583	1014.76	7.190937	1006.338	13.05553	988.9491	38.54936	988.9	38.5	102.6099	710
19AR105_Spot 493	282.1886	33609.09	2.628731	13.88909	1.06077	1.455329	1.402141	0.146287	0.706127	0.503606	880.1173	5.809168	912.0788	8.43885	991.1593	24.62854	991.2	24.6	88.79675	430
19AR105_Part2_Spot 325	84.84521	13527.44	3.290914	13.58357	2.530918	1.738225	2.871638	0.174626	0.82381	0.286878	1037.551	7.895063	1022.805	18.51163	992.2568	55.94557	992.3	55.9	104.5648	785
19AR105_Spot 349	313.1043	36131.89	2.563069	13.77687	1.164693	1.713582	1.427789	0.172141	0.734522	0.514447	1023.898	6.953896	1013.626	9.155198	992.3622	24.87572	992.4	24.9	103.1779	300
19AR105_Spot 241	219.5383	29694.14	4.577648	13.90519	1.223015	1.685083	1.4785	0.16926	0.759284	0.51355	1008.034	7.085431	1002.906	9.421656	992.6025	25.78591	992.6	25.8	101.5547	205
19AR105_Spot 590	108.8798	25015.99	2.220279	13.85993	2.99814	1.723555	3.181108	0.173089	0.787414	0.247528	1029.109	7.489628	1017.351	20.44354	993.0095	62.69274	993.0	62.7	103.6353	509
19AR105_Spot 521	606.1277	100644.9	2.871696	13.89478	0.886357	1.611977	1.107759	0.161832	0.651805	0.5884	966.9479	5.852704	974.8769	6.941788	993.6528	18.18805	993.7	18.2	97.31246	455
19AR105_Spot 200	463.839	75079.88	1.865099	13.84933	0.826254	1.691843	1.078211	0.169848	0.667315	0.618909	1011.276	6.245695	1005.459	6.880986	993.669	17.21576	993.7	17.2	101.7719	173
19AR105_Part2_Spot 236	187.8314	21860.49	2.993874	13.65916	1.413604	1.646287	1.683003	0.165257	0.776004	0.461083	985.9229	7.09446	988.1277	10.63162	993.8927	30.33942	993.9	30.3	99.19812	712
19AR105_Spot 22	75.14548	14391.33	3.112874	13.62359	4.156871	1.728237	4.477201	0.173442	1.340898	0.299495	1031.05	12.77638	1019.095	28.80541	994.4058	86.86408	994.4	86.9	103.685	22
19AR105_Part2_Spot 329	121.4201	21041.28	1.656867	13.63843	2.312558	1.445011	2.472589	0.14501	0.806444	0.326154	872.9318	6.583874	907.8028	14.83895	994.5075	47.51454	994.5	47.5	87.77529	789
19AR105_Spot 574	489.2316	10138.53	3.980348	13.63856	0.972626	1.364409	1.339857	0.13692	0.903239	0.674131	827.2204	7.012244	873.7657	7.850893	994.5264	20.11527	994.5	20.1	83.17732	495
19AR105_Part2_Spot 154	101.871	25590.18	2.418237	13.6576	1.915352	1.672873	2.082349	0.1678	0.788622	0.378717	999.9807	7.304854	998.2777	13.23405	995.4425	39.1696	995.4	39.2	100.4559	639
19AR105_Part2_Spot 249	174.6505	20781.61	1.787262	13.63285	1.232383	1.575741	1.407379	0.158023	0.62296	0.442639	945.7787	5.480004	960.6919	8.742475	995.8886	25.64584	995.9	25.6	94.96832	723
19AR105_Part2_Spot 290	84.59277	14198	1.762943	13.61005	3.983859	1.719461	4.069459	0.172428	0.764465	0.187854	1025.472	7.247643	1015.823	26.13196	995.9537	81.24251	996.0	81.2	102.9639	755
19AR105_Part2_Spot 239	232.2827	15195.7	3.095318	13.58756	1.422161	1.605539	1.576849	0.16089	0.552724	0.350524	961.7202	4.938151	972.3709	9.86636	997.3798	29.98586	997.4	30.0	96.42467	715
19AR105_Part2_Spot 198	131.0136	15474.26	2.714747	13.64788	1.857102	1.658117	2.257839	0.166106	0.893325	0.395655	990.6185	8.203034	992.6566	14.30186	998.0282	42.12791	998.0	42.1	99.25757	681
19AR105_Spot 308	206.6213	28744.85	2.668519	13.6876	1.100943	1.716439	1.514451	0.171913	0.821054	0.542147	1022.643	7.764322	1014.694	9.716877	998.4452	25.83234	998.4	25.8	102.4235	264
19AR105_Part2_Spot 102	128.8863	20704.68	2.296569	13.68627	2.392182	1.556798	2.626957	0.155898	0.867982	0.330413	933.9423	7.54659	953.1966	16.24261	998.7788	50.36985	998.8	50.4	93.50842	597
19AR105_Spot 88	496.5243	67767.34	2.439656	13.762	0.818573	1.636234	1.073132	0.163844	0.643421	0.599573	978.1052	5.83915	984.2628	6.763177	998.8859	17.42571	998.9	17.4	97.91961	77
19AR105_Spot 292	308.1128	62989.58	9.649089	13.76734	1.221968	1.657296	1.404506	0.165936	0.634733	0.451926	989.6786	5.823372	992.3432	8.894568	999.1038	25.44007	999.1	25.4	99.05663	251
19AR105_Spot 399	221.5656	34365.42	2.858478	13.71797	1.705743	1.559519	1.938005	0.156131	0.90024	0.464519	935.2376	7.83714	954.2767	11.9905	999.302	34.84014	999.3	34.8	93.58908	346
19AR105_Spot 311	172.1151	32019.33	3.999844	13.68798	1.598463	1.748785	1.797164	0.175035	0.661761	0.368225	1039.794	6.354682	1026.714	11.61	999.8071	33.9169	999.8	33.9	103.9995	267
19AR105_Part2_Spot 116	300.5142	48186.54	8.929126	13.74917	1.139304	1.62549	1.37352	0.162601	0.673089	0.490047	971.2149	6.068531	980.1165	8.634743	1000.977	24.3012	1001.0	24.3	97.02665	607
19AR105_Spot 195	203.221	35251.51	2.840052	13.75873	1.269058	1.678665	1.48891	0.167916	0.597202	0.4011	1000.621	5.535045	1000.476	9.474506	1001.022	27.67668	1001.0	27.7	99.95998	168
19AR105_Part2_Spot 316	100.8546	14812.42	1.417393	13.52644	2.498326	1.72929	2.813607	0.172935	0.996295	0.354099	1028.264	9.46926	1019.487	18.10329	1001.551	53.43394	1001.6	53.4	102.6671	777
19AR105_Spot 477	151.3298	31223.03	2.27851	13.80561	1.278503	1.816482	1.586736	0.181652	0.873709	0.550633	1075.995	8.658376	1051.418	10.39139	1001.58	26.89287	1001.6	26.9	107.4298	417
19AR105_Part2_Spot 245	167.9027	33682.58	2.102031	13.6339	1.535848	1.746887	1.66165	0.174621	0.597277	0.359448	1037.522	5.723915	1026.012	10.73024	1002.41	31.47231	1002.4	31.5	103.5028	720
19AR105_Spot 424	108.196	22923.26	1.99454	13.77819	2.734858	1.70893	2.868409	0.170824	0.762257	0.265742	1016.648	7.169297	1011.884	18.37574	1002.45	56.16182	1002.4	56.2	101.4164	369
19AR105_Spot 71	187.3425	44603.98	5.052793	13.69518	1.455105	1.70352	1.651548	0.170252	0.771649	0.467228	1013.501	7.236884	1009.854	10.56707	1002.815	29.62396	1002.8	29.6	101.0656	63
19AR105_Part2_Spot 134	288.4184	41897.23	2.816749	13.6587	1.054422	1.690267	1.383014	0.168911	0.804908	0.581995	1006.11	7.497931	1004.864	8.823225	1003.013	22.82978	1003.0	22.8	100.3087	624
19AR105_Spot 211	298.5276	57663.26	2.849051	13.75614	1.239858	1.731535	1.50706	0.173029	0.805969	0.534796	1028.779	7.663857	1020.322	9.700589	1003.084	25.84994	1003.1	25.8	102.5617	181
19AR105_Spot 339	180.2868	35567.35	5.078434	13.69231	1.464938	1.6942	1.662174	0.169281	0.715243	0.430306	1008.148	6.675151	1006.347	10.61346	1003.294	30.43826	1003.3	30.4	100.4839	292
19AR105_Part2_Spot 143	111.767	23936.15	2.510119	13.63426	2.347029	1.721648	2.522409	0.171967	0.794168	0.314845	1022.941	7.512095	1016.64	16.20297	1003.957	48.61757	1004.0	48.6	101.8909	631
19AR105_Part2_Spot 268	61.07131	11831.31	2.333952	13.4595	4.500339	1.749133	4.628195	0.174582	0.93716	0.202489	1037.306	8.979414	1026.842	29.90847	1005.477	92.00943	1005.5	92.0	103.1655	738
19AR105_Part2_Spot 174	484.1376	11690.23	2.119851	13.52735	1.096786	1.620353	1.555831	0.161614	1.069635	0.687501	965.7412	9.593379	978.1278	9.769123	1006.907	22.91865	1006.9	22.9	95.91168	658
19AR105_Spot 286	309.341	24772.11	3.927532	13.64625	1.146576	1.687694	1.45512	0.168292	0.649945	0.446661	1002.691	6.035403	1003.892	9.278006	1007.38	26.39162	1007.4	26.4	99.53453	245
19AR105_Spot 143	262.6522	35134.35	2.879894	13.71315	1.588469	1.706895	1.767028	0.170201	0.710421	0.402043	1013.217	6.660932	1011.121	11.31428	1007.447	32.82123	1007.4	32.8	100.5727	123
19AR105_Spot 160	142.2887	24713.77	1.935147	13.65011	1.935502	1.757966	2.102506	0.17522	0.799694	0.380353	1040.809	7.686118	1030.099	13.60864	1008.29	39.44266	1008.3	39.4	103.2252	137
19AR105_Spot 263	119.9029	24092.23	3.527873	13.63166	2.497207	1.702553	2.670977	0.169641	0.745202	0.279	1010.131	6.967392	1009.491	17.08707	1008.965	52.04518	1009.0	52.0	100.1156	224
19AR105_Spot 303	522.7283	95307.29	2.289485	13.69085	0.794099	1.698974	1.098401	0.169266	0.735	0.669155	1008.066	6.859024	1008.145	7.020785	1009.182	16.5539	1009.2	16.6	99.88934	260
19AR105_Spot 230	202.3697	27940.11	2.044146	13.76671	1.626183	1.61021	1.805949	0.160338	0.729465	0.403923	958.6526	6.49791	974.1895	11.31255	1010.254	33.49342	1010.3	33.5	94.8922	197
19AR105_Part2_Spot 252	264.2802	51004.81	2.755016	13.61049	1.101277	1.594247	1.335212	0.158748	0.708102	0.53033	949.814	6.253643	967.9609	8.331711	1010.259	22.9472	1010.3	22.9	94.01692	726
19AR105_Part2_Spot 66	855.0429	10274.77	12.14074	13.45354	0.729025	1.561158	1.210086	0.155376	0.915339	0.756425	931.0296	7.935264	954.9264	7.489699	1011.26	16.03939	1011.3	16.0	92.06625	567
19AR105_Spot 59	124.1647	20660.93	1.781099	13.56726	1.919119	1.646229	2.106948	0.163827	0.75269	0.357242	978.0104	6.83018	988.1054	13.3098	1011.456	39.91702	1011.5	39.9	96.6933	55
19AR105_Part2_Spot 317	164.2292	24568.07	2.721899	13.52936	1.964603	1.772879	2.206871	0.17641	0.95469	0.432599	1047.331	9.228795	1035.575	14.32794	1011.699	40.3589	1011.7	40.4	103.522	778
19AR105_Part2_Spot 207	179.3905	26835.46	1.519176	13.62739	2.056846	1.731883	2.260408	0.172163	0.806143	0.356636	1024.02	7.632787	1020.451	14.55134	1013.666	42.82081	1013.7	42.8	101.0214	689
19AR105_Spot 499	140.0205	16910.63	4.157006	13.60111	2.116921	1.686857	2.271058	0.167677	0.734104	0.323243	999.2976	6.795576	1003.576	14.47843	1013.795	43.56407	1013.8	43.6	98.57	435
19AR105_Spot 245	486.1625	67288.7	2.555634	13.80133	0.918221	1.593041	1.202273	0.158339	0.77132	0.641552	947.5417	6.796827	967.4886	7.499952	1013.946	18.68151	1013.9	18.7	93.45091	208
19AR105_Part2_Spot 195	63.33703	11162.82	2.578826	13.47445	5.190295	1.793529	5.261939	0.178221	0.675605	0.128395	1057.248	6.587841	1043.109	34.31597	1014.469	105.8239	1014.5	105.8	104.2168	678
19AR105_Spot 38	157.7047	22101	1.695038	13.55905	2.605622	1.707103	2.807543	0.169586	0.775875	0.276354	1009.831	7.252175	1011.199	17.97863	1015.027	54.68038	1015.0	54.7	99.48809	37
19AR105_Spot 468	166.1046	18846.89	1.589638	13.60733	1.682238	1.723728	2.073658	0.171165	0.683669	0.329692	1018.529	6.44113	1017.416	13.32591	1015.883	39.68453	1015.9	39.7	100.2605	409
19AR105_Spot 374	487.2305	110522.1	5.259324	13.7093	0.940525	1.727663	1.137279	0.171515	0.630359	0.55427	1020.454	5.949234	1018.881	7.314297	1016.366	19.17621	1016.4	19.2	100.4022	322
19AR105_Part2_Spot 180	268.499	57781.93	7.620421	13.64318	1.156604	1.659068	1.567441	0.164691	1.036	0.66095	982.7936	9.443603	993.0199	9.93047	1016.541	23.81866	1016.5	23.8	96.68019	663
19AR105_Spot 132	122.0457	31457.13	2.157954	13.63088	2.527048	1.701514	2.707296	0.168891	0.756058	0.279267	1005.998	7.042166	1009.1	17.31554	1016.702	52.66752	1016.7	52.7	98.94718	115
19AR105_Spot 39	147.3366	22942.47	7.793001	13.54883	1.584607	1.749959	1.761454	0.173681	0.722285	0.41005	1032.359	6.890145	1027.147	11.38206	1016.926	32.55547	1016.9	32.6	101.5177	38
19AR105_Spot 24	263.5378	38206.91	1.850769	13.59723	1.408032	1.782251	1.607218	0.176806	0.673033	0.418756	1049.501	6.518477	1039.001	10.45424	1017.839	29.56784	1017.8	29.6	103.1106	24
19AR105_Spot 85	260.9268	34121.65	1.858199	13.58391	1.384523	1.49807	1.826053	0.1486	1.163787	0.637324	893.1109	9.706042	929.6021	11.11957	1018.031	28.51906	1018.0	28.5	87.72927	75
19AR105_Spot 159	85.97344	12571.89	4.656705	13.4805	3.416932	1.688683	3.69988	0.167472	0.764591	0.206653	998.1683	7.070391	1004.266	23.59958	1018.46	73.33207	1018.5	73.3	98.00757	136
19AR105_Part2_Spot 205	442.1747	72149.28	1.337758	13.63444	0.820006	1.667618	1.112277	0.165302	0.722723	0.649769	986.1764	6.608925	996.2795	7.060297	1019.447	17.11997	1019.4	17.1	96.73636	687
19AR105_Part2_Spot 190	228.8968	10360.37	3.322786	13.42945	1.223748	1.613993	1.663414	0.159973	0.800544	0.481266	956.6262	7.117084	975.6601	10.429	1019.623	29.52525	1019.6	29.5	93.82154	673
19AR105_Spot 343	110.8231	4350.551	2.373626	13.0726	2.921193	1.646866	3.86836	0.163223	0.768991	0.19879	974.6633	6.955978	988.3497	24.4437	1019.726	76.77745	1019.7	76.8	95.58091	296
19AR105_Spot 538	209.8331	41928.95	2.423555	13.69996	0.973792	1.734533	1.302349	0.171875	0.838335	0.64371	1022.432	7.926226	1021.435	8.388155	1020.164	20.17879	1020.2	20.2	100.2223	471
19AR105_Spot 492	546.8203	72755.95	6.668157	13.73261	0.86479	1.678741	1.094486	0.166307	0.667893	0.610234	991.7292	6.13934	1000.505	6.964657	1020.646	17.55506	1020.6	17.6	97.16679	429
19AR105_Spot 111	147.2483	28085.79	1.649293	13.59234	1.678554	1.693434	1.828928	0.167629	0.64612	0.353278	999.0354	5.979658	1006.059	11.67636	1022.25	34.6517	1022.2	34.7	97.72908	96
19AR105_Spot 363	103.3297	16485.6	2.102703	13.54919	2.781271	1.748583	2.986094	0.173079	0.807991	0.270585	1029.055	7.684981	1026.639	19.29136	1022.354	58.19103	1022.4	58.2	100.6555	311
19AR105_Part2_Spot 6	214.3579	43720.69	5.784366	13.54133	1.575811	1.679021	2.323115	0.16616	1.69043	0.727657	990.9163	15.52686	1000.611	14.78466	1022.774	32.25188	1022.8	32.3	96.88521	521
19AR105_Spot 201	257.6642	48237.95	3.250688	13.63099	1.125726	1.718435	1.333788	0.170036	0.679897	0.509749	1012.308	6.369461	1015.44	8.561332	1023.063	23.2415	1023.1	23.2	98.94872	174
19AR105_Part2_Spot 280	188.6448	40679.1	2.478125	13.5491	1.497501	1.781463	1.710891	0.176224	0.771641	0.451017	1046.312	7.452613	1038.714	11.12687	1023.62	30.90142	1023.6	30.9	102.2169	747
19AR105_Part2_Spot 337	125.4334	25211.97	2.956287	13.48768	2.215406	1.676876	2.443251	0.165847	0.75089	0.307332	989.1876	6.885897	999.7975	15.54192	1024	47.06848	1024.0	47.1	96.60039	796
19AR105_Spot 233	381.9236	67262.1	3.67987	13.71062	0.969398	1.684258	1.271095	0.166572	0.818928	0.64427	993.1966	7.537981	1002.594	8.098443	1024.058	19.67037	1024.1	19.7	96.98638	200
19AR105_Part2_Spot 218	327.644	58218.46	3.341012	13.56887	1.119848	1.730031	1.307808	0.171088	0.669144	0.511653	1018.101	6.301842	1019.763	8.415314	1024.193	22.75566	1024.2	22.8	99.40526	699
19AR105_Spot 196	119.1513	19656.92	2.322846	13.54262	2.796206	1.621714	2.922648	0.160356	0.722885	0.247339	958.7573	6.439953	978.655	18.35874	1024.438	57.31044	1024.4	57.3	93.58863	169
19AR105_Spot 379	87.54291	17102.02	2.534254	13.50244	4.447252	1.687933	4.552668	0.166864	0.901387	0.197991	994.81	8.30945	1003.983	29.03695	1024.924	90.33307	1024.9	90.3	97.06184	327
19AR105_Part2_Spot 126	275.185	59799.93	1.560563	13.57108	1.445194	1.754859	1.589884	0.173403	0.655247	0.412135	1030.835	6.242132	1028.955	10.28378	1025.821	29.30993	1025.8	29.3	100.4888	616
19AR105_Spot 251	134.9006	20689.72	2.702442	13.60573	2.585239	1.760732	2.690588	0.173929	0.644966	0.239712	1033.725	6.160072	1031.117	17.42564	1026.452	52.8393	1026.5	52.8	100.7086	214
19AR105_Part2_Spot 296	272.2447	53392.99	1.608837	13.49953	1.012809	1.728562	1.265547	0.170744	0.673242	0.531977	1016.208	6.329543	1019.216	8.140827	1026.543	21.69368	1026.5	21.7	98.99325	760
19AR105_Spot 74	241.3501	42642.87	5.729473	13.5297	1.82433	1.698531	2.045133	0.167726	0.902972	0.441522	999.5711	8.360891	1007.978	13.07138	1027.161	37.11498	1027.2	37.1	97.31395	66
19AR105_Spot 177	153.6271	33986.64	2.516825	13.6445	2.3012	1.713344	2.459815	0.169073	0.854446	0.347362	1007.004	7.965931	1013.537	15.77273	1028.543	46.64653	1028.5	46.6	97.90591	152
19AR105_Spot 328	83.18724	13250.53	3.612355	13.36725	3.596227	1.718556	3.702961	0.169509	0.765387	0.206696	1009.406	7.151364	1015.485	23.77299	1029.479	73.27211	1029.5	73.3	98.05023	282
19AR105_Spot 220	988.4689	215511.8	2.016123	13.63103	0.594862	1.729055	0.884554	0.170543	0.651839	0.736912	1015.103	6.122169	1019.399	5.690566	1029.498	12.08881	1029.5	12.1	98.60167	188
19AR105_Spot 118	601.4596	77713.34	3.412792	13.60494	0.694668	1.737615	0.95283	0.171366	0.633689	0.665059	1019.633	5.976216	1022.579	6.140899	1029.752	14.38522	1029.8	14.4	99.01735	101
19AR105_Part2_Spot 307	457.9748	87822.73	2.732214	13.49836	0.684621	1.68629	0.955217	0.166299	0.6601	0.691047	991.6861	6.067464	1003.362	6.088584	1029.817	13.95828	1029.8	14.0	96.29733	771
19AR105_Spot 275	169.5218	34214.14	2.520819	13.50046	1.570308	1.781805	1.866803	0.17571	0.921532	0.493642	1043.496	8.87821	1038.839	12.14179	1029.913	32.84107	1029.9	32.8	101.3188	235
19AR105_Spot 249	81.37404	10158.79	4.495668	13.40472	3.727015	1.461365	3.976066	0.144106	1.071368	0.269454	867.8372	8.699058	914.5718	23.97435	1029.978	77.41604	1030.0	77.4	84.25787	212
19AR105_Part2_Spot 292	262.9786	53137.23	10.47439	13.51789	1.074191	1.699223	1.303727	0.16754	0.732705	0.562008	998.541	6.77787	1008.239	8.333703	1030.234	21.81981	1030.2	21.8	96.9237	756
19AR105_Part2_Spot 272	382.044	35375.12	3.932895	13.44153	0.976075	1.738779	1.305474	0.17142	0.842668	0.645488	1019.929	7.949197	1023.011	8.415799	1030.47	20.17439	1030.5	20.2	98.97712	740
19AR105_Spot 199	56.21251	9360.174	2.490679	13.33789	4.723576	1.741952	4.968768	0.171683	1.100703	0.221524	1021.38	10.39697	1024.186	32.06264	1031.048	97.99278	1031.0	98.0	99.06235	172
19AR105_Spot 449	357.5939	61065.2	3.645952	13.54885	0.963201	1.775642	1.17832	0.174926	0.670535	0.56906	1039.197	6.435528	1036.586	7.654083	1031.945	19.60059	1031.9	19.6	100.7028	391
19AR105_Spot 56	79.12651	14495.97	2.416686	13.37729	4.13022	1.774392	4.269807	0.174796	0.687708	0.161063	1038.482	6.59617	1036.129	27.73498	1032.028	85.19993	1032.0	85.2	100.6254	52
19AR105_Spot 419	108.127	19118.26	2.41604	13.51466	3.146178	1.745834	3.337691	0.171944	0.972776	0.291452	1022.814	9.20051	1025.623	21.55112	1032.482	64.55594	1032.5	64.6	99.06369	364
19AR105_Spot 584	205.9835	41241.83	2.841179	13.66018	1.717128	1.752616	1.906391	0.172588	0.755086	0.396081	1026.356	7.164411	1028.128	12.3255	1032.761	35.39383	1032.8	35.4	99.37989	504
19AR105_Part2_Spot 197	155.7458	4667.313	2.474121	13.04417	1.433889	1.69637	1.823466	0.16702	0.61773	0.338767	995.6697	5.699104	1007.165	11.64897	1033.12	34.6885	1033.1	34.7	96.37502	680
19AR105_Part2_Spot 80	131.4663	27825.18	3.44073	13.49057	2.244972	1.859212	2.420822	0.183009	0.856915	0.353977	1083.394	8.545573	1066.707	15.98495	1033.6	45.73972	1033.6	45.7	104.8175	576
19AR105_Spot 403	330.8328	47487.21	3.844146	13.5514	1.262618	1.721555	1.488395	0.169437	0.780917	0.52467	1009.01	7.293822	1016.605	9.56015	1033.861	25.62109	1033.9	25.6	97.59629	350
19AR105_Spot 350	207.1631	38588.82	2.405593	13.51975	1.271246	1.738636	1.490352	0.171102	0.665555	0.446575	1018.182	6.268493	1022.958	9.607405	1034.05	26.9615	1034.1	27.0	98.46545	301
19AR105_Spot 445	137.527	28791.23	3.105818	13.52446	1.69513	1.79017	1.883189	0.17614	0.731911	0.388655	1045.851	7.06602	1041.887	12.26899	1034.444	35.07552	1034.4	35.1	101.1027	388
19AR105_Part2_Spot 342	695.3459	20814.7	2.739848	13.41106	0.677014	1.685624	1.036966	0.165823	0.780845	0.75301	989.0567	7.159716	1003.11	6.608698	1034.807	13.80253	1034.8	13.8	95.57882	801
19AR105_Spot 154	464.1904	76583.99	5.712443	13.56771	0.950812	1.840105	1.230263	0.180978	0.776522	0.631184	1072.317	7.67109	1059.898	8.093666	1035.274	19.29565	1035.3	19.3	103.5781	131
19AR105_Part2_Spot 208	139.7995	24600.39	2.447009	13.45771	1.639967	1.707814	1.93004	0.16795	0.915427	0.474305	1000.806	8.485901	1011.465	12.3606	1035.483	34.34231	1035.5	34.3	96.6511	690
19AR105_Spot 98	195.4766	35819.17	0.920821	13.48218	1.416458	1.775943	1.600708	0.174592	0.639408	0.399453	1037.36	6.126782	1036.697	10.39862	1036.158	29.65861	1036.2	29.7	100.116	87
19AR105_Spot 547	244.0385	35135.84	1.83525	13.57704	1.127889	1.740262	1.393769	0.171001	0.719172	0.515991	1017.625	6.770068	1023.56	8.987826	1037.131	24.13012	1037.1	24.1	98.11928	477
19AR105_Spot 471	199.5583	41741.28	3.76393	13.55189	1.454292	1.734792	1.608356	0.170441	0.673385	0.418679	1014.542	6.321307	1021.531	10.35978	1037.398	29.51494	1037.4	29.5	97.7968	412
19AR105_Part2_Spot 162	59.02496	10414.28	3.709169	13.26598	5.483835	1.809952	5.745226	0.177791	0.869833	0.151401	1054.895	8.464393	1049.061	37.59271	1037.794	114.753	1037.8	114.8	101.6478	646
19AR105_Spot 29	326.219	11797.27	6.26652	13.29662	0.929908	1.680987	1.192789	0.165102	0.675112	0.565995	985.0702	6.167136	1001.356	7.594011	1038.042	19.87475	1038.0	19.9	94.89693	29
19AR105_Part2_Spot 281	304.798	60482.23	4.0776	13.46773	1.061535	1.796679	1.277946	0.176383	0.705969	0.552425	1047.184	6.823576	1044.253	8.336426	1038.985	21.52497	1039.0	21.5	100.7891	748
19AR105_Spot 67	190.954	38979.5	1.620711	13.43111	1.453721	1.748938	1.616128	0.171676	0.640256	0.396167	1021.339	6.047474	1026.77	10.44072	1039.223	29.97692	1039.2	30.0	98.27907	61
19AR105_Part2_Spot 319	2441.899	44132.73	3.817413	13.39874	0.564433	1.800599	1.054809	0.176635	0.889761	0.843528	1048.567	8.610484	1045.675	6.886149	1040.498	11.45417	1040.5	11.5	100.7755	780
19AR105_Part2_Spot 163	233.2929	39028.54	1.662572	13.42511	1.313495	1.791665	1.607761	0.175658	0.80081	0.49809	1043.208	7.71319	1042.431	10.47756	1041.661	28.15488	1041.7	28.2	100.1485	647
19AR105_Spot 231	335.8464	55783.11	2.954384	13.59945	1.269767	1.808799	1.505034	0.17729	0.794071	0.52761	1052.151	7.708641	1048.644	9.841457	1042.206	25.81904	1042.2	25.8	100.9542	198
19AR105_Spot 104	241.2216	35692.1	2.095697	13.425	1.082566	1.795984	1.35916	0.175942	0.630581	0.463949	1044.765	6.081935	1044.001	8.865015	1043.26	24.31252	1043.3	24.3	100.1443	92
19AR105_Spot 430	69.24891	11957.82	1.716051	13.36299	5.400582	1.72414	5.7215	0.168878	1.777412	0.310655	1005.928	16.55434	1017.569	36.78518	1043.56	109.7893	1043.6	109.8	96.39391	374
19AR105_Spot 105	240.0678	33677.65	3.123726	13.41872	1.716673	1.772126	1.925576	0.173579	0.666286	0.346019	1031.798	6.352768	1035.299	12.49955	1043.56	36.46982	1043.6	36.5	98.87286	93
19AR105_Part2_Spot 159	100.7628	16311.81	2.654813	13.31962	2.413811	1.770274	2.769516	0.173367	0.970779	0.350523	1030.635	9.246366	1034.621	17.97202	1043.913	52.32572	1043.9	52.3	98.72804	643
19AR105_Spot 402	82.10727	17704.59	2.436451	13.3615	3.203268	1.721074	3.32571	0.168547	0.801397	0.24097	1004.1	7.451451	1016.425	21.36178	1043.932	65.13086	1043.9	65.1	96.18449	349
19AR105_Part2_Spot 182	1149.959	15830.99	3.235661	13.34311	0.698145	1.657941	0.995893	0.162364	0.692351	0.695206	969.902	6.234374	992.5897	6.307713	1043.933	14.46039	1043.9	14.5	92.90846	665
19AR105_Spot 387	243.2002	62332.88	3.10942	13.48614	0.953213	1.78241	1.179236	0.174551	0.645669	0.547532	1037.135	6.185546	1039.059	7.670532	1043.968	19.92559	1044.0	19.9	99.34544	335
19AR105_Spot 256	215.1495	33561.25	2.135634	13.47307	1.021359	1.792991	1.20452	0.175578	0.626025	0.51973	1042.771	6.027377	1042.913	7.851652	1044.07	20.77874	1044.1	20.8	99.87555	218
19AR105_Spot 279	413.8253	87576.4	3.330686	13.46154	0.794123	1.790644	1.07824	0.175324	0.715463	0.663547	1041.376	6.879998	1042.059	7.025174	1044.351	16.29098	1044.4	16.3	99.71515	239
19AR105_Spot 103	300.1125	54120.17	4.235285	13.43937	1.181604	1.809961	1.371936	0.177177	0.634455	0.462452	1051.533	6.155798	1049.064	8.973127	1044.783	24.55541	1044.8	24.6	100.6461	91
19AR105_Spot 352	299.8169	33533.09	2.945282	13.46629	1.119373	1.766719	1.468781	0.172931	0.840875	0.572499	1028.242	7.991921	1033.317	9.523621	1044.969	24.30988	1045.0	24.3	98.39927	303
19AR105_Spot 97	156.2807	31343.53	2.38308	13.41264	1.744082	1.779287	1.869508	0.174146	0.619842	0.331553	1034.914	5.926391	1037.919	12.1532	1045.15	35.58558	1045.1	35.6	99.0206	86
19AR105_Part2_Spot 363	114.7852	22164.2	1.187451	13.28056	1.264539	1.767867	1.463097	0.172843	0.683506	0.467164	1027.755	6.493398	1033.738	9.48899	1047.312	26.10383	1047.3	26.1	98.13264	818
19AR105_Part2_Spot 375	319.0487	67350.38	4.43085	13.36993	0.752669	1.774993	0.989781	0.173504	0.620998	0.627409	1031.386	5.918783	1036.349	6.428497	1047.73	15.55869	1047.7	15.6	98.44012	828
19AR105_Spot 121	507.9322	79090.81	0.9304	13.51219	0.759548	1.780578	0.989027	0.173936	0.624109	0.631034	1033.763	5.961077	1038.391	6.430869	1049.043	15.48464	1049.0	15.5	98.54336	104
19AR105_Spot 570	166.5727	33631.79	3.178311	13.50639	2.059269	1.692682	2.268708	0.165339	0.862047	0.379972	986.3813	7.884482	1005.775	14.48199	1049.173	42.30747	1049.2	42.3	94.01509	491
19AR105_Spot 11	219.7462	40162.2	1.100723	13.37959	1.821427	1.773495	2.004073	0.173165	0.80151	0.399941	1029.528	7.62657	1035.801	13.01277	1049.961	37.0342	1050.0	37.0	98.05391	11
19AR105_Part2_Spot 196	289.2398	51387.37	7.393558	13.41684	0.932213	1.764033	1.125249	0.17218	0.579393	0.514902	1024.114	5.486321	1032.33	7.292049	1050.675	19.45985	1050.7	19.5	97.47203	679
19AR105_Part2_Spot 288	128.8964	19763.99	2.891006	13.28927	2.422354	1.80566	2.593776	0.176238	0.727651	0.280537	1046.39	7.028232	1047.508	16.95133	1050.737	50.18119	1050.7	50.2	99.58629	753
19AR105_Part2_Spot 298	39.26103	4929.657	3.081167	12.88789	8.085248	1.805947	8.608156	0.176238	1.339545	0.155613	1046.387	12.93837	1047.612	56.31324	1051.063	171.6513	1051.1	171.7	99.55518	762
19AR105_Spot 68	278.7557	38562.36	4.850485	13.35324	0.975079	1.734378	1.139506	0.169238	0.583339	0.511922	1007.911	5.442941	1021.378	7.339041	1051.247	19.74545	1051.2	19.7	95.87758	62
19AR105_Spot 4	320.5754	57948.07	6.08817	13.37603	1.067278	1.757159	1.325817	0.171432	0.77024	0.580955	1019.999	7.266418	1029.802	8.579718	1051.581	21.76477	1051.6	21.8	96.99672	4
19AR105_Part2_Spot 19	98.74727	20243.67	2.084975	13.28265	1.765865	1.827131	2.012821	0.178076	0.660268	0.328031	1056.458	6.433865	1055.249	13.20941	1053.646	38.3059	1053.6	38.3	100.2669	530
19AR105_Part2_Spot 15	484.2046	93200.69	1.803934	13.38021	0.610331	1.828205	0.865335	0.17818	0.595396	0.688052	1057.025	5.80459	1055.635	5.679797	1053.657	12.66882	1053.7	12.7	100.3197	528
19AR105_Spot 136	165.345	28420.92	1.343972	13.36158	1.634309	1.793937	1.845846	0.174798	0.826405	0.447711	1038.492	7.926558	1043.257	12.03473	1054.146	33.24842	1054.1	33.2	98.51507	117
19AR105_Part2_Spot 148	726.3174	34813.19	2.51898	13.34879	0.661665	1.794105	0.949812	0.174782	0.661272	0.696214	1038.404	6.342163	1043.318	6.192669	1054.52	13.75142	1054.5	13.8	98.47173	634
19AR105_Part2_Spot 12	213.5729	46162.35	3.800748	13.30173	1.26503	1.878774	1.390787	0.182957	0.535355	0.38493	1083.111	5.337537	1073.63	9.21657	1055.323	25.85456	1055.3	25.9	102.6331	525
19AR105_Spot 217	172.5475	32353	2.735481	13.3718	1.396459	1.798814	1.622581	0.175165	0.706779	0.435589	1040.507	6.791265	1045.028	10.58923	1055.388	29.43495	1055.4	29.4	98.59003	185
19AR105_Spot 576	197.3228	38745.36	1.98588	13.42668	1.927562	1.814402	2.092307	0.17668	0.744895	0.356016	1048.812	7.210125	1050.667	13.69711	1055.422	39.39794	1055.4	39.4	99.37377	497
19AR105_Spot 395	227.0084	60371.55	3.679027	13.37555	1.125505	1.756929	1.347717	0.170907	0.677422	0.502644	1017.107	6.374048	1029.718	8.721031	1057.5	23.46865	1057.5	23.5	96.18039	342
19AR105_Spot 410	284.758	47405.15	2.447022	13.35829	1.43368	1.796599	1.641894	0.174748	0.729686	0.444417	1038.221	6.997178	1044.224	10.71056	1057.703	29.6127	1057.7	29.6	98.15809	357
19AR105_Spot 219	410.4811	55527.64	4.218678	13.40127	0.984487	1.810395	1.276552	0.176002	0.782624	0.613076	1045.095	7.550584	1049.221	8.349956	1058.715	20.31779	1058.7	20.3	98.71357	187
19AR105_Part2_Spot 297	502.9453	91165.99	1.506895	13.30198	0.905884	1.846002	1.187458	0.179402	0.760795	0.640692	1063.705	7.460201	1062.005	7.820847	1059.409	18.35427	1059.4	18.4	100.4055	761
19AR105_Part2_Spot 301	253.2121	3763.853	1.452286	12.67171	1.400612	1.822373	2.564349	0.177006	0.713035	0.278057	1050.596	6.912541	1053.539	16.81395	1060.538	49.57523	1060.5	49.6	99.06255	765
19AR105_Spot 411	218.7471	40118.74	2.150455	13.3218	1.109271	1.794907	1.317508	0.174232	0.70066	0.531807	1035.385	6.701923	1043.609	8.591483	1061.766	22.4677	1061.8	22.5	97.51542	358
19AR105_Spot 587	284.6271	26252.09	5.105721	13.42633	1.364172	1.748315	1.617435	0.169653	0.801721	0.495674	1010.201	7.496299	1026.54	10.44781	1062.426	28.26111	1062.4	28.3	95.08442	506
19AR105_Part2_Spot 305	82.19239	12446.6	7.981259	13.09251	3.799928	1.770765	3.985536	0.171776	0.755821	0.189641	1021.89	7.142592	1034.801	25.86853	1063.077	78.7274	1063.1	78.7	96.12568	769
19AR105_Part2_Spot 92	532.6293	128125	3.58515	13.31985	0.715823	1.812193	0.906905	0.175473	0.550133	0.606605	1042.198	5.294007	1049.87	5.934117	1066.759	14.47853	1066.8	14.5	97.6976	587

19AR105_Part2_Spot 187	176.59	34721.92	3.453352	13.27734	1.426176	1.814724	1.657794	0.175677	0.696649	0.420227	1043.313	6.710561	1050.783	10.85305	1067.236	30.25338	1067.2	30.3	97.75848	670
19AR105_Part2_Spot 34	89.40105	11184.34	1.469257	13.12903	3.682515	1.955648	3.766176	0.189263	0.635965	0.168862	1117.384	6.52439	1100.389	25.30803	1067.829	74.63067	1067.8	74.6	104.6407	540
19AR105_Spot 487	253.2889	48867.9	1.919982	13.36772	1.117558	1.74386	1.356915	0.168645	0.763934	0.562993	1004.64	7.106646	1024.893	8.756749	1069.283	22.51889	1069.3	22.5	93.95455	424
19AR105_Part2_Spot 158	377.2477	18176.93	0.861737	13.15557	1.031159	1.652117	1.325038	0.159588	0.798157	0.602366	954.4865	7.081139	990.3621	8.381392	1071.608	21.23055	1071.6	21.2	89.0705	642
19AR105_Part2_Spot 71	364.1335	51223.99	2.017042	13.289	0.867306	1.849219	1.128325	0.178601	0.715963	0.634536	1059.328	6.994008	1063.151	7.435917	1071.9	17.51128	1071.9	17.5	98.82708	572
19AR105_Part2_Spot 214	731.3184	9089.792	3.72919	13.00685	0.68599	1.770199	1.099152	0.170834	0.840613	0.764783	1016.703	7.906661	1034.593	7.131915	1073.494	14.22361	1073.5	14.2	94.70978	695
19AR105_Spot 342	176.0791	29992.37	4.338722	13.20436	1.819318	1.870646	2.027106	0.180389	0.716694	0.353555	1069.101	7.060532	1070.759	13.41355	1075.031	38.07909	1075.0	38.1	99.44843	295
19AR105_Spot 569	232.5212	61444.69	2.382548	13.3687	1.788321	1.856348	1.912323	0.179006	0.652902	0.341418	1061.54	6.390238	1065.689	12.62007	1075.085	36.09327	1075.1	36.1	98.74014	490
19AR105_Spot 329	166.2566	3022.438	2.353999	12.4798	2.230124	1.707627	2.657825	0.16459	0.637418	0.239827	982.2367	5.807301	1011.395	17.02163	1075.989	51.83019	1076.0	51.8	91.28685	283
19AR105_Part2_Spot 132	205.3851	44010.53	1.790352	13.21342	1.39389	1.99186	1.643051	0.191966	0.767426	0.467074	1132.016	7.967379	1112.754	11.10749	1076.202	29.16814	1076.2	29.2	105.1863	622
19AR105_Spot 501	226.5382	43891.91	2.424114	13.26046	1.276653	1.776938	1.478127	0.171173	0.706517	0.477981	1018.572	6.656647	1037.06	9.604189	1077.138	26.05586	1077.1	26.1	94.56278	437
19AR105_Part2_Spot 46	170.8532	43809.16	1.833847	13.18121	1.928488	1.855285	2.051515	0.178576	0.679999	0.331462	1059.189	6.641886	1065.311	13.53604	1078.75	38.86528	1078.8	38.9	98.18668	549
19AR105_Spot 327	124.5392	26102.85	1.464216	13.14296	1.956951	1.938449	2.086888	0.186567	0.674605	0.323259	1102.751	6.837707	1094.463	13.97954	1078.896	39.65314	1078.9	39.7	102.2111	281
19AR105_Spot 437	184.05	30189.09	3.157596	13.28673	1.359694	1.8769	1.52079	0.180524	0.65467	0.43048	1069.837	6.453584	1072.969	10.07465	1080.194	27.5232	1080.2	27.5	99.0412	380
19AR105_Spot 302	142.3449	26442.14	1.820409	13.14123	2.050427	1.930054	2.17866	0.185354	0.704218	0.323235	1096.157	7.098707	1091.558	14.5728	1083.251	41.36744	1083.3	41.4	101.1914	259
19AR105_Spot 28	69.2753	9317.911	2.254435	12.9552	3.481218	1.713629	3.988817	0.164557	0.942463	0.236276	982.0503	8.584953	1013.643	25.58187	1083.402	77.75723	1083.4	77.8	90.64508	28
19AR105_Spot 301	223.9536	37332.16	2.241386	13.16731	1.006848	1.917826	1.192083	0.184138	0.582159	0.488354	1089.54	5.835799	1087.312	7.956003	1083.706	20.84909	1083.7	20.8	100.5384	258
19AR105_Spot 218	248.6859	33686.8	1.78572	13.20176	2.008839	1.855424	2.148102	0.178066	0.744209	0.34645	1056.399	7.251438	1065.36	14.17378	1084.65	40.42733	1084.6	40.4	97.39541	186
19AR105_Part2_Spot 44	219.5936	35088.67	2.726485	13.13764	1.215809	1.924707	1.405488	0.184554	0.695876	0.495113	1091.808	6.989074	1089.703	9.391865	1086.381	24.45963	1086.4	24.5	100.4995	547
19AR105_Spot 128	92.21077	16745.1	2.855799	13.11945	2.412645	1.949848	2.76366	0.186788	0.801142	0.289884	1103.954	8.128389	1098.394	18.55086	1088.268	53.03352	1088.3	53.0	101.4414	111
19AR105_Spot 253	188.243	31268.73	1.776377	13.16571	2.297255	1.786022	2.436211	0.170951	0.777195	0.319018	1017.351	7.314459	1040.376	15.85924	1089.949	46.28349	1089.9	46.3	93.33937	216
19AR105_Part2_Spot 217	132.5559	18818.5	1.792006	13.0337	2.049552	1.916859	2.318518	0.183405	0.771626	0.33281	1085.55	7.709087	1086.975	15.47208	1090.685	43.78497	1090.7	43.8	99.52921	698
19AR105_Spot 254	217.3465	27403.26	2.167739	13.14714	0.975695	1.987643	1.222469	0.19014	0.634182	0.518771	1122.135	6.53143	1111.321	8.258229	1091.077	20.91728	1091.1	20.9	102.8465	217
19AR105_Spot 297	58.98598	8870.805	3.673734	12.91457	3.042044	1.789705	3.196741	0.171178	0.835339	0.26131	1018.598	7.870572	1041.718	20.82674	1091.399	61.82208	1091.4	61.8	93.32962	255
19AR105_Spot 247	280.3326	38872.25	1.451183	13.23215	1.285859	1.913621	1.522674	0.182709	0.791791	0.52	1081.757	7.885167	1085.847	10.15487	1094.952	26.03919	1095.0	26.0	98.79489	210
19AR105_Part2_Spot 142	584.8182	25441.59	6.251353	13.03843	0.699906	1.888101	0.994052	0.180198	0.670102	0.674112	1068.058	6.595611	1076.914	6.598694	1095.774	14.69364	1095.8	14.7	97.47057	630
19AR105_Spot 393	43.05532	6709.054	3.496523	12.79963	6.499241	1.873017	6.871876	0.178574	1.010817	0.147095	1059.181	9.873094	1071.597	45.51972	1097.821	136.1699	1097.8	136.2	96.4803	340
19AR105_Part2_Spot 133	346.8172	55315.56	2.694756	13.05964	0.76817	1.999586	1.002386	0.190544	0.634104	0.632595	1124.324	6.542284	1115.372	6.785004	1098.84	15.53414	1098.8	15.5	102.3191	623
19AR105_Spot 460	75.12197	20052.52	1.546865	13.02988	3.245111	1.973413	3.441001	0.187986	0.813657	0.236459	1110.455	8.299915	1106.474	23.19279	1099.512	66.90727	1099.5	66.9	100.9953	401
19AR105_Part2_Spot 69	564.4378	102880.2	3.63231	13.12728	0.67865	1.891588	1.037929	0.180131	0.774803	0.74649	1067.691	7.623743	1078.139	6.894367	1100.212	13.80714	1100.2	13.8	97.04409	570
19AR105_Part2_Spot 145	351.2239	80189.65	3.072975	13.0883	0.886308	1.930984	1.099077	0.183866	0.634652	0.577441	1088.061	6.354092	1091.88	7.352429	1100.355	17.96304	1100.4	18.0	98.88277	633
19AR105_Spot 227	622.5022	95803.76	5.416932	13.14258	0.750187	1.810312	1.056441	0.17234	0.738662	0.699198	1024.99	6.999966	1049.191	6.910045	1100.774	15.11979	1100.8	15.1	93.11541	194
19AR105_Spot 482	285.8487	49295.38	1.866186	13.15339	1.262285	1.952499	1.448212	0.18556	0.681751	0.470753	1097.279	6.878679	1099.306	9.724707	1104.212	25.54825	1104.2	25.5	99.3721	420
19AR105_Part2_Spot 61	263.8564	53011.72	4.47288	12.99708	1.039283	1.94351	1.357085	0.184587	0.800454	0.589833	1091.984	8.040595	1096.21	9.0985	1105.501	21.90612	1105.5	21.9	98.7773	563
19AR105_Spot 193	784.8048	17003.3	0.959641	13.00979	0.630696	1.755917	0.865961	0.166668	0.586553	0.677344	993.7275	5.401708	1029.345	5.602351	1106.72	12.73141	1106.7	12.7	89.79034	166
19AR105_Spot 414	90.95348	28673.03	3.313628	13.00858	3.084818	1.847649	3.207512	0.175119	0.749075	0.233538	1040.256	7.19608	1062.592	21.1346	1109.6	62.31767	1109.6	62.3	93.75049	360
19AR105_Spot 36	300.5017	48558.06	2.626487	12.98781	1.153131	1.912975	1.382204	0.181231	0.721337	0.521875	1073.696	7.134352	1085.622	9.216936	1110.479	23.54015	1110.5	23.5	96.68765	36
19AR105_Spot 87	141.696	19864.55	2.361747	12.91779	1.93523	1.880196	2.219192	0.177994	0.681984	0.307312	1056.007	6.642863	1074.132	14.7108	1111.955	42.15794	1112.0	42.2	94.96856	76
19AR105_Spot 58	283.0485	47726.96	1.454922	12.97288	1.036608	2.021858	1.254998	0.191332	0.662027	0.527513	1128.586	6.854071	1122.884	8.52628	1112.719	21.28928	1112.7	21.3	101.4259	54
19AR105_Part2_Spot 278	213.1886	39802.03	4.105703	12.9066	1.239982	2.053971	1.515206	0.193739	0.791635	0.52246	1141.598	8.282302	1133.617	10.34775	1119.253	25.77279	1119.3	25.8	101.9964	746
19AR105_Spot 529	650.1599	142632	2.318056	13.05446	0.708284	2.006052	0.981419	0.189087	0.676268	0.689071	1116.428	6.932427	1117.559	6.650226	1120.645	14.17354	1120.6	14.2	99.62368	463
19AR105_Spot 502	91.4337	19747.83	5.17921	12.90287	2.767985	1.959506	2.974354	0.184696	0.808301	0.271757	1092.577	8.123471	1101.713	19.9989	1120.688	57.09876	1120.7	57.1	97.49164	438
19AR105_Spot 265	249.5231	47068.15	1.964158	12.92713	1.03066	2.013421	1.229033	0.189451	0.657147	0.534686	1118.401	6.747323	1120.045	8.338313	1124.085	20.69161	1124.1	20.7	99.49433	226
19AR105_Spot 478	212.6809	42149.03	2.563937	13.00156	1.392814	2.042242	1.61655	0.192142	0.809065	0.500489	1132.97	8.406142	1129.71	11.01918	1124.336	27.89134	1124.3	27.9	100.7679	418
19AR105_Spot 472	37.87344	4300.434	1.064198	12.47453	7.173903	1.672281	7.485208	0.156993	1.406488	0.187902	940.0446	12.30281	998.0529	47.59692	1128.671	146.6333	1128.7	146.6	83.28773	413
19AR105_Part2_Spot 119	340.8611	52003.51	1.681667	12.89164	1.130522	2.070968	1.36083	0.194405	0.74605	0.548232	1145.194	7.82784	1139.253	9.318456	1128.808	22.66982	1128.8	22.7	101.4515	610
19AR105_Part2_Spot 130	130.7142	25245.75	2.486664	12.81693	1.511393	2.022362	1.757494	0.189614	0.746807	0.424927	1119.285	7.673466	1123.053	11.94142	1131.232	31.66325	1131.2	31.7	98.94392	620
19AR105_Spot 109	618.0777	108503.9	5.667953	12.92658	0.711487	2.001136	0.95611	0.187404	0.63632	0.66553	1107.296	6.474016	1115.897	6.473434	1133.535	14.19366	1133.5	14.2	97.68523	95
19AR105_Spot 474	217.6136	27407.91	1.914125	12.89097	1.149264	2.017607	1.345658	0.188466	0.693535	0.515387	1113.062	7.089799	1121.454	9.135877	1138.635	22.91933	1138.6	22.9	97.75409	414
19AR105_Spot 398	160.2618	29091.13	2.262809	12.74225	1.618453	1.992497	1.89613	0.185212	0.785835	0.414442	1095.383	7.916296	1112.97	12.81991	1148.332	34.29552	1148.3	34.3	95.38902	345
19AR105_Part2_Spot 359	384.2508	76394.82	11.91386	12.75278	0.895677	2.020559	1.102724	0.187657	0.616391	0.558971	1108.671	6.27839	1122.447	7.49012	1150.081	18.15157	1150.1	18.2	96.39933	815
19AR105_Part2_Spot 347	112.5262	20488.98	0.859036	12.65947	1.722619	2.181278	1.893782	0.202263	0.75074	0.396424	1187.468	8.1419	1175.086	13.18541	1153.198	34.51049	1153.2	34.5	102.9717	804
19AR105_Part2_Spot 64	41.50907	7014.604	0.583615	12.44602	5.136602	2.259626	5.565683	0.209423	1.096198	0.196957	1225.744	12.23642	1199.789	39.19519	1154.194	108.3634	1154.2	108.4	106.1992	565
19AR105_Part2_Spot 170	313.6477	60131.59	3.311191	12.72499	0.95842	2.091984	1.232021	0.193863	0.756064	0.613678	1142.265	7.91437	1146.178	8.46407	1154.432	19.32318	1154.4	19.3	98.94604	654
19AR105_Spot 401	88.23519	18088.19	4.422128	12.6436	3.978617	1.856475	4.072089	0.171949	0.781584	0.191937	1022.842	7.392406	1065.734	26.87864	1155.458	79.3253	1155.5	79.3	88.52266	348
19AR105_Part2_Spot 25	451.6624	67624.84	2.481334	12.72094	0.725302	2.105298	0.969518	0.194394	0.58797	0.606456	1145.133	6.168909	1150.541	6.674251	1161.589	15.30192	1161.6	15.3	98.58333	534
19AR105_Spot 13	349.7008	65412.17	2.859393	12.62221	0.82901	2.134946	0.998071	0.196227	0.548038	0.549097	1155.021	5.795281	1160.189	6.901682	1170.733	16.53259	1170.7	16.5	98.65797	13
19AR105_Part2_Spot 246	24.75152	4604.126	1.96319	12.1129	8.385416	1.880516	8.564192	0.172454	1.341651	0.156658	1025.617	12.72141	1074.244	56.82981	1175.183	167.6399	1175.2	167.6	87.27297	721
19AR105_Spot 228	79.42689	8034.516	3.596885	12.44348	2.654065	1.956846	3.10735	0.179209	0.895687	0.288248	1062.649	8.774918	1100.8	20.88379	1177.889	58.8582	1177.9	58.9	90.21643	195
19AR105_Spot 533	53.30677	9241.422	1.61582	12.43539	3.347796	2.101465	3.471673	0.191887	0.768963	0.221496	1131.587	7.980564	1149.286	23.88934	1183.71	66.90573	1183.7	66.9	95.59661	467
19AR105_Spot 466	104.4477	29210.26	1.842358	12.49425	2.309391	1.986311	2.470838	0.18052	0.858138	0.347306	1069.813	8.459152	1110.869	16.68882	1193.013	45.72825	1193.0	45.7	89.67318	407
19AR105_Spot 189	24.28184	2778.533	2.248074	11.82037	9.759502	1.91995	10.15355	0.174249	2.24626	0.221229	1035.481	21.48773	1088.05	67.89048	1195.683	195.7914	1195.7	195.8	86.60164	162
19AR105_Part2_Spot 127	204.6347	60984.83	1.64789	12.37107	1.319776	2.369324	1.479544	0.21334	0.65123	0.440156	1246.586	7.381454	1233.398	10.56465	1211.262	26.14878	1211.3	26.1	102.9162	617
19AR105_Spot 35	230.2941	9439.994	3.896115	12.14693	1.008851	2.148793	1.320267	0.193175	0.720395	0.545643	1138.549	7.518566	1164.664	9.148584	1214.397	21.78412	1214.4	21.8	93.7543	35
19AR105_Spot 373	779.3466	113158.6	2.846832	12.36933	0.673116	2.311775	0.918842	0.206918	0.61747	0.672009	1212.378	6.824257	1215.905	6.512709	1223.008	13.37818	1223.0	13.4	99.1308	321
19AR105_Spot 359	73.67172	15196.64	18.33511	12.251	2.010714	2.35182	2.266702	0.210413	0.856561	0.377889	1231.019	9.598782	1228.109	16.15044	1223.842	41.21627	1223.8	41.2	100.5864	307
19AR105_Spot 304	33.98203	5693.457	2.80871	11.7866	9.633058	1.970402	9.904101	0.17372	1.093969	0.110456	1032.573	10.43778	1105.445	66.80525	1252.62	193.0245	1252.6	193.0	82.43307	261
19AR105_Part2_Spot 256	133.297	23919.46	2.935661	11.98196	1.249462	2.348394	1.505497	0.206624	0.740042	0.491559	1210.806	8.169268	1227.071	10.72162	1256.64	25.61886	1256.6	25.6	96.35265	729
19AR105_Spot 537	177.0448	44767.07	2.171907	12.02314	1.068955	2.476721	1.29504	0.21519	0.694037	0.535919	1256.409	7.92281	1265.258	9.367696	1281.162	21.30093	1281.2	21.3	98.06793	470
19AR105_Spot 597	134.8315	22520.49	1.8029	11.75783	1.137808	2.553744	1.364084	0.218228	0.666672	0.488733	1272.504	7.698611	1287.507	9.953506	1313.45	23.08415	1313.4	23.1	96.88254	515
19AR105_Part2_Spot 105	186.5186	53331.69	7.761675	11.75129	1.211656	2.638139	1.425308	0.225083	0.712767	0.500079	1308.68	8.441968	1311.339	10.49476	1316.516	23.93093	1316.5	23.9	99.40478	600
19AR105_Spot 391	113.4948	26511.46	1.787753	11.631	1.866932	2.631806	1.993191	0.223211	0.676532	0.339422	1298.821	7.958326	1309.57	14.66697	1328.039	36.29524	1328.0	36.3	97.79996	338
19AR105_Part2_Spot 107	277.9127	4983.006	4.446945	11.12104	0.795693	2.338066	1.065823	0.193986	0.674812	0.633137	1142.929	7.067591	1223.934	7.580275	1370.481	15.87584	1370.5	15.9	83.39621	602
19AR105_Spot 33	211.5018	60380.52	2.493637	11.3265	1.002443	2.896327	1.236517	0.23894	0.687789	0.556231	1381.182	8.550893	1380.956	9.333283	1381.425	19.74478	1381.4	19.7	99.98246	33
19AR105_Part2_Spot 220	387.9847	98431.5	2.66516	11.31802	0.660274	2.93941	0.957008	0.242014	0.677301	0.707727	1397.16	8.507743	1392.122	7.250735	1385.227	12.98356	1385.2	13.0	100.8615	701
19AR105_Spot 150	177.6691	32200.36	1.744166	11.28064	1.307404	2.546657	1.615477	0.208745	0.886336	0.548653	1222.129	9.867311	1285.48	11.77881	1393.777	25.90688	1393.8	25.9	87.6847	129
19AR105_Part2_Spot 128	189.936	6115.161	1.847497	10.65448	1.511318	2.51219	1.701403	0.199081	0.660325	0.388106	1170.38	7.067357	1275.565	12.35757	1458.282	29.81603	1458.3	29.8	80.25748	618
19AR105_Spot 320	21.25359	1686.432	2.771519	9.935686	21.96286	0.933458	23.00673	0.073448	5.296547	0.230217	456.8965	23.36216	669.4525	113.2506	1471.8	430.3691	1471.8	430.4	31.04339	275
19AR105_Part2_Spot 356	142.9428	30663.21	2.374637	10.44571	1.132635	3.398984	1.266511	0.259638	0.560059	0.442206	1487.989	7.441745	1504.162	9.936871	1527.818	21.39821	1527.8	21.4	97.39307	813
19AR105_Part2_Spot 171	153.602	32006.58	1.368552	9.195973	0.944655	3.709886	1.283317	0.248679	0.844899	0.658371	1431.657	10.84704	1573.502	10.26429	1770.081	17.63897	1770.1	17.6	80.88092	655
19AR105_Part2_Spot 394	110.2327	9075.242	1.0666	8.816103	1.326102	4.022532	2.100995	0.262264	1.44056	0.685656	1501.417	19.29478	1638.761	17.08727	1820.536	27.75681	1820.5	27.8	82.47113	843
19AR105_Spot 305	236.6724	77888.68	5.006723	8.840992	0.766407	5.255183	1.018198	0.337922	0.667813	0.655877	1876.667	10.87326	1861.614	8.686019	1845.617	13.90547	1845.6	13.9	101.6824	262
19AR105_Spot 49	126.1194	46351.87	1.828755	8.754299	0.842414	5.130806	1.10435	0.327363	0.668573	0.605399	1825.586	10.62936	1841.221	9.38463	1859.707	15.87558	1859.7	15.9	98.16524	46
19AR105_Spot 498	61.28735	20230.41	0.736246	8.77495	1.855681	5.38996	2.000857	0.342969	0.7354	0.367543	1900.935	12.10685	1883.259	17.13857	1864.59	33.59161	1864.6	33.6	101.9492	434
19AR105_Spot 496	195.8296	67342.5	1.58285	8.766549	0.964879	4.955551	1.201487	0.313736	0.709977	0.590916	1759.066	10.92998	1811.772	10.15158	1873.716	17.47727	1873.7	17.5	93.88114	432
19AR105_Spot 535	217.3532	45283.3	3.513802	8.756583	0.723594	5.159927	0.954783	0.326134	0.618482	0.647773	1819.616	9.805155	1846.032	8.121042	1876.705	13.11221	1876.7	13.1	96.95804	469
19AR105_Part2_Spot 331	167.5503	57184.18	0.735156	8.647558	0.757134	5.418232	0.959336	0.34227	0.573618	0.597932	1897.58	9.429095	1887.742	8.223421	1877.707	13.85794	1877.7	13.9	101.0584	791
19AR105_Spot 43	108.899	44417.3	0.698628	8.654386	0.945722	5.307557	1.158627	0.334524	0.621592	0.53649	1860.27	10.0444	1870.08	9.899676	1881.769	17.61342	1881.8	17.6	98.85752	42
19AR105_Part2_Spot 50	324.3118	102905.1	2.061712	8.58706	0.689905	5.041688	1.009486	0.316626	0.73616	0.729243	1773.23	11.41235	1826.353	8.553782	1888.244	12.43257	1888.2	12.4	93.90898	553
19AR105_Spot 238	730.9624	68634.99	4.913637	8.700934	0.717899	5.019042	1.077824	0.314635	0.803195	0.745201	1763.475	12.392	1822.54	9.126048	1891.49	12.93144	1891.5	12.9	93.23206	203
19AR105_Part2_Spot 244	169.535	33458.16	1.557809	8.525866	0.809751	5.077903	1.039902	0.31658	0.63427	0.609933	1773.006	9.831712	1832.421	8.821933	1901.374	14.80952	1901.4	14.8	93.24864	719
19AR105_Spot 120	234.5803	65274.99	0.827653	8.51521	0.695466	5.24907	1.014142	0.322914	0.735959	0.725696	1803.942	11.5805	1860.621	8.649802	1925.324	12.50318	1925.3	12.5	93.69551	103
19AR105_Spot 508	96.39942	31827.67	1.690982	8.442478	0.849268	5.488683	1.236272	0.335215	0.89387	0.723037	1863.609	14.46657	1898.827	10.6187	1938.304	15.28079	1938.3	15.3	96.14637	444
19AR105_Part2_Spot 161	251.9934	33549.54	5.599825	8.348896	0.820814	5.082953	1.371719	0.309429	1.0741	0.783032	1737.894	16.36222	1833.264	11.639	1944.116	15.2572	1944.1	15.3	89.39252	645
19AR105_Spot 542	224.4562	12300.18	1.877171	8.366387	0.863217	4.868585	1.165704	0.295286	0.736645	0.631931	1667.889	10.82564	1796.836	9.819763	1950.719	16.14149	1950.7	16.1	85.50125	473
19AR105_Part2_Spot 135	449.0552	56906.03	3.205687	8.18688	0.774751	5.08183	1.115914	0.303563	0.794207	0.71171	1708.951	11.92255	1833.077	9.468017	1977.874	13.96283	1977.9	14.0	86.40343	625
19AR105_Spot 72	99.22505	34322.65	1.295336	8.184945	0.961286	5.812911	1.177033	0.34679	0.647285	0.549929	1919.253	10.74438	1948.337	10.19751	1980.151	17.50392	1980.2	17.5	96.92461	64
19AR105_Part2_Spot 68	323.9679	113339.1	3.054141	8.117835	0.759044	5.806672	1.105487	0.342269	0.798211	0.722044	1897.578	13.12093	1947.407	9.576109	2001.574	13.58353	2001.6	13.6	94.80428	569
19AR105_Spot 519	337.0127	132858.9	15.10395	8.156161	0.655705	5.6563	0.922043	0.332944	0.646755	0.701437	1852.635	10.414	1924.723	7.955901	2004.035	11.66782	2004.0	11.7	92.44524	453
19AR105_Part2_Spot 149	126.2775	30241.61	0.965552	8.054601	0.855845	6.166277	1.114631	0.362184	0.652495	0.585391	1992.518	11.1838	1999.681	9.73876	2007.847	16.03854	2007.8	16.0	99.23651	635
19AR105_Spot 73	28.82691	2364.524	2.002998	7.658882	29.38511	1.293453	29.80862	0.075238	2.93823	0.09857	467.6331	13.25362	842.8273	172.3356	2025.087	538.2437	2025.1	538.2	23.09199	65
19AR105_Spot 89	88.81501	30005.22	2.924329	7.710272	0.918158	6.650788	1.158394	0.373117	0.63955	0.552101	2044.051	11.20291	2066.11	10.2251	2088.932	16.98366	2088.9	17.0	97.85149	78
19AR105_Spot 147	94.18112	27274.02	5.618562	7.545921	0.907331	7.283822	1.191547	0.399595	0.748029	0.62778	2167.174	13.7675	2146.829	10.63863	2128.166	16.23967	2128.2	16.2	101.833	126
19AR105_Spot 522	192.6963	19524.79	3.611736	7.524773	0.784009	6.374574	1.230674	0.347657	0.938933	0.762942	1923.403	15.61442	2028.774	10.80199	2138.482	13.91176	2138.5	13.9	89.94244	456
19AR105_Part2_Spot 150	176.4147	58893.44	1.463628	7.459531	0.693794	7.237754	0.94569	0.392944	0.629407	0.665553	2136.466	11.44583	2141.166	8.436918	2146.427	12.33081	2146.4	12.3	99.53597	636
19AR105_Part2_Spot 267	490.2327	221191.8	3.226593	7.017688	0.660449	8.173564	1.140195	0.418748	0.928806	0.814602	2254.791	17.67223	2250.42	10.31567	2247.185	11.42927	2247.2	11.4	100.3385	737
19AR105_Spot 322	78.02533	32725.96	1.699059	6.01156	0.686626	11.03508	1.022993	0.483421	0.749357	0.732514	2542.151	15.74233	2526.096	9.524491	2513.939	11.70521	2513.9	11.7	101.1222	276
19AR105_Spot 318	43.38559	27543.92	1.692831	5.881574	1.141386	11.18245	1.420163	0.479266	0.825425	0.581218	2524.07	17.2396	2538.454	13.23717	2550.687	19.35624	2550.7	19.4	98.95647	273
19AR105_Spot 234	118.6213	27987.14	1.137122	5.862452	0.758435	10.79871	1.106144	0.455437	0.79146	0.715513	2419.377	15.96551	2505.955	10.28002	2577.584	12.91068	2577.6	12.9	93.8622	201
19AR105_Spot 170	20.83422	1482.502	2.303268	5.544441	21.81096	1.864803	22.87189	0.078195	5.97828	0.261381	485.3387	27.94974	1068.69	152.3036	2587.24	373.5325	2587.2	373.5	18.75893	146
19AR105_Spot 360	58.86454	18020.55	3.554747	5.740287	1.034571	10.25071	1.543515	0.426525	1.121171	0.726375	2290.032	21.61008	2457.665	14.2805	2600.119	17.68277	2600.1	17.7	88.07414	308
19AR105_Part2_Spot 373	127.5631	45469.39	1.77499	5.671915	0.585127	11.78791	0.880737	0.488638	0.655669	0.744455	2564.779	13.87398	2587.704	8.243714	2606.413	9.794364	2606.4	9.8	98.40263	826
19AR105_Spot 421	474.3465	183951.3	1.692927	5.661938	0.739463	11.07488	1.139355	0.45254	0.866659	0.760657	2406.537	17.4059	2529.448	10.61112	2630.292	12.29111	2630.3	12.3	91.49312	366
19AR105_Part2_Spot 103	189.3562	77346.59	1.829822	5.610033	0.6578	12.17185	0.961357	0.494678	0.698362	0.726434	2590.883	14.8996	2617.74	9.020607	2639.292	10.96962	2639.3	11.0	98.16582	598
19AR105_Spot 83	129.4117	34720.59	1.790994	5.563299	0.930621	11.28541	1.297242	0.456474	0.891622	0.687321	2423.971	18.01413	2546.999	12.10039	2647.188	15.63472	2647.2	15.6	91.56779	73
19AR105_Spot 208	99.77847	45782.75	3.648031	5.577735	0.785411	11.79252	1.105655	0.476898	0.767457	0.69412	2513.738	15.97527	2588.07	10.3494	2647.495	13.20555	2647.5	13.2	94.94779	179
19AR105_Part2_Spot 31	128.3549	18504.79	2.547677	5.522469	0.626721	10.90244	1.090913	0.437468	0.835141	0.765543	2339.298	16.38427	2514.843	10.14664	2660.461	11.63376	2660.5	11.6	87.92827	538
19AR105_Part2_Spot 58	226.0838	75009.09	2.414074	5.469047	0.608489	12.34522	0.912782	0.492098	0.678458	0.743286	2579.746	14.42435	2631.018	8.573939	2671.412	10.11061	2671.4	10.1	96.56863	560
19AR105_Spot 62	124.9645	72996.37	0.885132	5.266796	0.572083	13.24613	0.84581	0.508041	0.622191	0.735616	2648.263	13.51227	2697.35	7.985529	2735.046	9.427198	2735.0	9.4	96.827	57
**19AR114**																				
19AR114_Spot 397	2372.227	377.0666	0.950387	5.74949	18.93747	0.006155	20.52389	0.00032	6.510677	0.317224	2.065631	0.134465	6.230398	1.274805	2219.115	340.8506	2.1	0.1	0.093084	370
19AR114_Spot 76	1581.077	2804.027	2.452898	11.78102	16.7686	0.013153	18.30881	0.001196	4.055567	0.221509	7.705875	0.31233	13.26836	2.413481	1191.824	355.153	7.7	0.3	0.646562	71
19AR114_Spot 196	5175.596	9127.213	4.067477	17.34917	3.980945	0.011979	4.298097	0.001551	1.067718	0.248417	9.992048	0.106604	12.09145	0.51662	453.7376	92.45129	10.0	0.1	2.202164	183
19AR114_Spot 409	351.49	926.1417	2.070383	6.979768	30.2693	0.044339	31.25948	0.00249	5.946257	0.190223	16.02878	0.951928	44.0515	13.47663	2087.456	553.9599	16.0	1.0	0.767862	382
19AR114_Spot 442	152.9982	16771.54	1.66697	16.92949	4.246626	0.339327	4.582331	0.04206	0.922659	0.201352	265.589	2.400688	296.6614	11.78874	549.9349	98.06479	265.6	2.4	48.29462	414
19AR114_Spot 374	260.442	15090.48	6.481091	16.92507	3.21701	0.4714	4.970302	0.058718	3.618667	0.728058	367.8226	12.93768	392.1553	16.16993	539.2173	74.5378	367.8	12.9	68.21417	347
19AR114_Spot 441	337.6523	2909.857	1.691759	16.82783	2.39072	0.501659	2.708394	0.06657	0.917533	0.338774	415.4587	3.691732	412.8247	9.187362	399.0785	57.13081	415.5	3.7	104.1045	413
19AR114_Spot 62	251.004	12384.77	3.061604	17.48635	3.271174	0.543081	3.725311	0.070478	0.786506	0.211125	439.0348	3.338077	440.454	13.31353	448.8232	80.94129	439.0	3.3	97.81911	59
19AR114_Spot 93	91.98722	5371.256	1.879612	16.15634	4.422891	0.589176	5.159454	0.072264	1.170813	0.226926	449.7794	5.086563	470.3411	19.42493	572.8879	109.3503	449.8	5.1	78.5109	87
19AR114_Spot 428	18.47112	105.2981	2.916128	5.102042	9.637995	0.589437	21.37618	0.073332	4.767286	0.223019	456.1993	20.9967	470.5082	80.66152	541.8923	460.131	456.2	21.0	84.18635	401
19AR114_Spot 1	155.7848	8567.687	1.651465	16.64922	4.487583	0.5923	4.737855	0.073479	0.791869	0.167137	457.08	3.494137	472.3354	17.89672	548.1164	102.0955	457.1	3.5	83.39104	1
19AR114_Spot 66	465.1928	20386.98	1.062399	17.2436	1.697788	0.585258	1.878946	0.074184	0.685851	0.365019	461.3138	3.053372	467.8348	7.043662	500.9406	38.49674	461.3	3.1	92.08953	62
19AR114_Spot 122	104.6761	8405.933	1.75977	15.48854	6.780443	0.643745	6.928967	0.074382	1.048902	0.151379	462.503	4.681265	504.6224	27.56034	701.3547	145.9576	462.5	4.7	65.94424	115
19AR114_Spot 54	365.6316	30820.72	2.262917	17.4326	2.44447	0.581258	2.64589	0.074608	0.793446	0.299879	463.8551	3.551144	465.2697	9.876013	473.2253	55.86895	463.9	3.6	98.01992	53
19AR114_Spot 121	50.48042	4237.765	2.209781	13.40585	7.439132	0.734075	7.897606	0.074843	1.85623	0.235037	465.2696	8.332176	558.9423	33.9594	962.2171	157.0359	465.3	8.3	48.35391	114
19AR114_Spot 414	114.8809	5244.432	2.174338	15.74843	4.129516	0.62828	4.294914	0.074903	0.742226	0.172815	465.6284	3.334154	495.0239	16.82861	634.3545	91.09283	465.6	3.3	73.40193	387
19AR114_Spot 202	110.5914	6117.578	2.291615	16.34368	5.300185	0.607358	6.114741	0.074911	0.954919	0.156167	465.6765	4.290016	481.8924	23.46483	560.7309	131.7465	465.7	4.3	83.04813	189
19AR114_Spot 250	287.1897	19922.67	1.462411	17.49497	2.852559	0.581748	3.008992	0.074948	0.711103	0.236326	465.8974	3.196124	465.5845	11.23742	465.0014	64.80606	465.9	3.2	100.1927	229
19AR114_Spot 255	124.8938	10522.53	1.729206	16.48606	5.623814	0.610744	5.915226	0.074977	1.11098	0.187817	466.0699	4.995194	484.0291	22.77754	570.9374	126.5035	466.1	5.0	81.63239	233
19AR114_Spot 99	225.8296	32460.87	1.683582	17.68676	2.785334	0.584391	3.01919	0.074979	0.957771	0.317228	466.0823	4.306446	467.2794	11.30784	474.1483	63.35221	466.1	4.3	98.29884	92
19AR114_Spot 248	201.7669	17509.56	1.390695	17.49559	3.824057	0.585029	4.138656	0.075173	1.077757	0.260412	467.2458	4.857606	467.6883	15.51184	470.8462	88.47983	467.2	4.9	99.23533	227
19AR114_Spot 246	380.5009	34481.38	1.276327	17.5731	2.38108	0.586969	2.598544	0.075212	1.007924	0.38788	467.4827	4.545079	468.93	9.759328	477.0043	52.97911	467.5	4.5	98.00388	226
19AR114_Spot 87	786.8428	66731.05	1.279127	17.74333	0.817683	0.582212	1.079298	0.075263	0.686176	0.635762	467.7855	3.096138	465.8819	4.032644	457.4974	18.50257	467.8	3.1	102.2488	82
19AR114_Spot 212	121.3344	2236.033	1.593391	17.69974	3.987612	0.521726	4.862908	0.075295	0.80758	0.166069	467.9788	3.645386	426.3034	16.93059	207.7848	111.2566	468.0	3.6	225.2229	198
19AR114_Spot 36	75.77559	4647.952	1.912064	15.05645	5.608042	0.657868	6.256607	0.075306	0.991962	0.158546	468.0434	4.478272	513.3089	25.21429	721.2046	131.2223	468.0	4.5	64.89745	35
19AR114_Spot 68	111.5545	9622.562	1.844857	15.35683	6.442572	0.659705	6.602733	0.07532	0.925162	0.140118	468.1275	4.177426	514.4334	26.6546	726.7242	138.7482	468.1	4.2	64.41612	64
19AR114_Spot 86	661.1271	76318.13	1.269371	17.6755	1.29601	0.585372	1.509602	0.075344	0.748331	0.495714	468.273	3.379983	467.9076	5.659751	467.0999	29.04618	468.3	3.4	100.2512	81
19AR114_Spot 81	113.3626	17425.51	1.786815	16.79656	4.825162	0.609899	4.976677	0.075351	1.05208	0.211402	468.3155	4.752343	483.4961	19.14608	557.0823	106.1557	468.3	4.8	84.06577	76
19AR114_Spot 59	89.55478	8286.038	1.758104	16.19346	5.186779	0.621067	5.510372	0.075379	0.851286	0.154488	468.4838	3.846668	490.5156	21.4394	595.6936	118.0424	468.5	3.8	78.64511	56
19AR114_Spot 43	52.4875	6316.397	1.449278	15.17177	7.805887	0.660458	8.119034	0.075385	1.540629	0.189755	468.5147	6.962017	514.894	32.80217	727.3265	169.2443	468.5	7.0	64.41601	42
19AR114_Spot 252	135.5773	13177.72	1.62974	16.52189	4.765544	0.615293	5.178541	0.075403	0.861692	0.166397	468.6267	3.894834	486.8925	20.03199	574.7589	111.1	468.6	3.9	81.53449	231

19AR114_Spot 88	212.9359	10762.4	1.301358	17.96024	2.758236	0.565367	3.195269	0.075467	0.838529	0.262428	469.011	3.793135	455.0136	11.71846	385.866	69.29161	469.0	3.8	121.5476	83
19AR114_Spot 245	174.9625	22471.51	1.663947	17.03068	3.52524	0.605586	3.821961	0.075476	1.320129	0.345406	469.0615	5.972301	480.7725	14.63823	537.9654	78.4921	469.1	6.0	87.19177	225
19AR114_Spot 51	238.4493	11581.02	1.737287	16.67532	3.281043	0.607291	3.578537	0.075527	0.788549	0.220355	469.3705	3.569683	481.85	13.7298	542.6054	76.30992	469.4	3.6	86.5031	50
19AR114_Spot 144	306.0917	33891.45	9.966356	18.04009	2.305278	0.573034	2.550321	0.07555	0.848252	0.332606	469.5052	3.841016	459.975	9.433646	413.6019	53.77952	469.5	3.8	113.5162	134
19AR114_Spot 256	192.1669	21923.35	1.732022	17.27011	2.915986	0.594796	3.186479	0.07562	0.849379	0.266557	469.9256	3.849439	473.9258	12.06769	494.283	67.71159	469.9	3.8	95.07217	234
19AR114_Spot 100	62.25396	8389.568	3.708865	14.68037	6.800726	0.696015	7.073323	0.075632	1.560137	0.220566	469.9962	7.071664	536.408	29.4825	830.6882	143.9957	470.0	7.1	56.57914	93
19AR114_Spot 291	97.10632	8346.108	2.101759	18.29412	2.742015	0.554266	2.911218	0.075632	0.753236	0.258736	469.998	3.414218	447.7876	10.54176	336.2561	63.73197	470.0	3.4	139.7738	268
19AR114_Spot 258	132.8356	14317.92	1.970269	16.28878	5.922065	0.627198	6.011047	0.075651	0.848459	0.14115	470.1101	3.846723	494.3486	23.53	609.1702	128.7256	470.1	3.8	77.17222	236
19AR114_Spot 133	171.8514	12586.57	1.258243	16.63783	4.625087	0.615387	4.816223	0.075666	0.918364	0.190681	470.2028	4.164448	486.9521	18.6319	567.5286	102.9964	470.2	4.2	82.85094	124
19AR114_Spot 123	115.4554	10563.64	1.455167	15.54142	6.409871	0.656807	6.627013	0.075707	0.860517	0.12985	470.4496	3.904106	512.6587	26.68169	706.4785	139.9154	470.4	3.9	66.59078	116
19AR114_Spot 15	111.192	12180.18	1.922327	17.10654	5.759225	0.598401	5.986838	0.075746	0.965733	0.161309	470.6836	4.383567	476.2182	22.7618	503.9015	130.1446	470.7	4.4	93.40786	14
19AR114_Spot 109	154.6304	13982.48	1.684033	17.01931	4.675337	0.603111	4.795037	0.075753	0.926519	0.193225	470.7236	4.205917	479.2061	18.31904	520.9389	103.2827	470.7	4.2	90.36062	102
19AR114_Spot 217	220.9657	3260.567	1.443844	17.60864	2.77205	0.551633	2.943272	0.075818	0.705058	0.239549	471.1144	3.203158	446.0655	10.6252	319.8645	64.93772	471.1	3.2	147.2856	202
19AR114_Spot 135	83.2135	6519.969	1.82396	16.55313	5.997629	0.609671	6.270615	0.075879	0.918912	0.146542	471.4799	4.177842	483.3526	24.12016	541.0091	135.7595	471.5	4.2	87.14825	126
19AR114_Spot 216	265.6811	4120.619	1.344016	17.67504	2.588739	0.559807	2.795159	0.075889	0.769811	0.275409	471.5351	3.500351	451.4007	10.18635	351.0606	60.73738	471.5	3.5	134.3173	201
19AR114_Spot 95	98.99651	8047.759	1.838992	15.13052	5.902225	0.675791	6.044329	0.075912	0.952725	0.157623	471.6746	4.333301	524.2274	24.75464	761.1131	125.9502	471.7	4.3	61.97169	88
19AR114_Spot 153	315.7582	27065.72	10.06615	17.88262	2.436817	0.579639	2.666569	0.075923	0.696231	0.261096	471.7386	3.167095	464.2294	9.93565	428.2001	57.40617	471.7	3.2	110.1678	143
19AR114_Spot 181	94.0789	8619.705	1.726935	15.33452	6.934605	0.662136	7.192426	0.075945	1.013697	0.14094	471.8719	4.612478	515.9197	29.1008	717.0027	151.3808	471.9	4.6	65.81172	169
19AR114_Spot 322	252.0743	14051.33	1.686577	17.38619	3.621333	0.594163	3.698358	0.075946	0.617267	0.166903	471.8764	2.808684	473.5227	13.99714	482.4904	80.56566	471.9	2.8	97.80017	297
19AR114_Spot 124	56.73448	6536.7	2.231638	13.167	6.726322	0.772015	6.926801	0.075987	1.229367	0.17748	472.1224	5.596674	580.9181	30.65158	1033.753	137.9026	472.1	5.6	45.67072	117
19AR114_Spot 226	76.0595	7895.044	2.054411	15.72631	8.850245	0.653689	9.146755	0.076037	1.428115	0.156134	472.4262	6.505502	510.7466	36.72855	687.12	193.1475	472.4	6.5	68.75453	209
19AR114_Spot 195	102.7163	14596.84	1.838796	16.37189	5.396978	0.629644	5.673025	0.076037	1.04785	0.184708	472.4264	4.773281	495.8743	22.25956	606.5672	120.6633	472.4	4.8	77.88525	182
19AR114_Spot 149	63.55736	6726.173	2.081149	13.95672	7.510839	0.729422	7.784301	0.076086	1.437522	0.184669	472.7206	6.552288	556.2142	33.34907	915.4123	157.6408	472.7	6.6	51.64018	139
19AR114_Spot 27	299.2398	36432.31	1.26983	17.36185	2.367353	0.600521	2.498082	0.07609	0.71918	0.287893	472.7412	3.278191	477.5645	9.517356	501.7519	52.67244	472.7	3.3	94.2181	26
19AR114_Spot 80	220.3128	22938.42	1.798161	17.58952	3.06283	0.590186	3.253807	0.07614	0.878823	0.270091	473.0418	4.008335	470.9865	12.26264	461.9281	69.47929	473.0	4.0	102.4059	75
19AR114_Spot 108	131.5942	10678.03	1.815783	16.68059	4.178886	0.615542	4.616505	0.076162	0.989695	0.214382	473.1757	4.515259	487.0493	17.86189	553.7888	98.43368	473.2	4.5	85.44336	101
19AR114_Spot 237	115.2672	10403.72	1.863726	16.13669	4.18735	0.639271	4.424222	0.07618	0.963185	0.217707	473.2791	4.395235	501.8547	17.5204	635.31	92.98338	473.3	4.4	74.49577	218
19AR114_Spot 215	112.7561	1777.254	2.085166	15.39129	4.816089	0.601915	5.025651	0.076197	0.875497	0.174206	473.3854	3.995961	478.4485	19.17652	503.7447	108.9675	473.4	4.0	93.97328	200
19AR114_Spot 277	246.3603	28499.09	1.225143	16.99768	2.150883	0.611736	2.349774	0.076204	0.852559	0.362826	473.4222	3.89156	484.6541	9.056022	539.0574	47.91045	473.4	3.9	87.82407	254
19AR114_Spot 439	502.49	43463.84	1.435696	17.75338	1.442492	0.590322	1.630686	0.076205	0.715494	0.438769	473.4294	3.265965	471.0733	6.146241	460.5548	32.49623	473.4	3.3	102.7955	412
19AR114_Spot 331	417.5989	45471.5	1.327055	17.86094	1.263769	0.585124	1.497991	0.076229	0.692885	0.462543	473.5755	3.163703	467.7492	5.614723	440.2011	29.55069	473.6	3.2	107.5816	305
19AR114_Spot 4	96.06583	9876.208	1.902617	15.40247	4.376328	0.66988	4.741244	0.076242	1.166205	0.24597	473.6496	5.325681	520.6397	19.31463	733.4305	97.37754	473.6	5.3	64.58002	4
19AR114_Spot 160	159.6188	18230.86	1.644769	17.4045	3.536343	0.593702	3.822104	0.076287	0.799242	0.209111	473.9194	3.651882	473.2287	14.45849	470.8654	82.75782	473.9	3.7	100.6486	149
19AR114_Spot 111	80.37224	6188.735	1.961938	14.4525	7.157797	0.703812	7.814947	0.076301	0.96466	0.123438	474.0047	4.408471	541.0654	32.79004	835.5905	161.8018	474.0	4.4	56.72691	104
19AR114_Spot 113	124.3597	9650.312	1.748876	15.49345	3.728971	0.66248	3.943611	0.076302	0.952772	0.241599	474.0144	4.35423	516.1301	15.95792	708.1266	81.40988	474.0	4.4	66.93922	106
19AR114_Spot 207	108.3509	9674.368	1.898077	15.9044	5.132945	0.647523	5.274908	0.076324	0.908893	0.172305	474.142	4.154778	506.9531	21.0538	658.8023	111.4502	474.1	4.2	71.97031	194
19AR114_Spot 257	130.0527	12436.97	2.082222	17.32975	3.159059	0.59236	3.563886	0.076326	0.925443	0.259672	474.1576	4.230565	472.3735	13.46243	464.6835	76.27881	474.2	4.2	102.0388	235
19AR114_Spot 278	225.5725	25845.28	1.864341	16.63992	3.428672	0.626839	3.637171	0.076329	0.837773	0.230337	474.1721	3.829905	494.1251	14.23094	588.6396	76.79924	474.2	3.8	80.5539	255
19AR114_Spot 433	220.4032	18125.93	1.367251	17.45914	3.019135	0.596953	3.268048	0.076335	0.851128	0.260439	474.2084	3.891245	475.2981	12.40475	481.5016	69.71922	474.2	3.9	98.48532	406
19AR114_Spot 63	152.9602	27401.83	2.734149	17.05959	3.813438	0.610846	3.936999	0.07635	0.862561	0.219091	474.2967	3.94422	484.0934	15.16021	531.7237	84.16184	474.3	3.9	89.19985	60
19AR114_Spot 19	117.638	18589.12	1.769865	16.52182	4.215203	0.629482	4.372295	0.076358	0.855875	0.195749	474.3477	3.914052	495.7732	17.15199	596.9399	92.9053	474.3	3.9	79.46322	18
19AR114_Spot 218	247.5405	4450.955	1.524956	16.62908	2.811557	0.602768	2.963675	0.076364	0.816163	0.275389	474.3823	3.732705	478.9889	11.3177	502.0406	62.72949	474.4	3.7	94.49083	203
19AR114_Spot 281	121.6227	17354.94	1.793842	15.34707	6.010645	0.678359	6.126795	0.076386	0.702054	0.114588	474.5167	3.211709	525.782	25.14934	755.9689	128.5237	474.5	3.2	62.76934	258
19AR114_Spot 337	253.8449	30493.27	1.682852	17.56109	2.021417	0.595398	2.262502	0.076396	0.854551	0.377702	474.5749	3.909802	474.3089	8.573683	473.9645	46.34349	474.6	3.9	100.1288	311
19AR114_Spot 360	292.3049	25319.16	4.387574	17.53053	1.958619	0.594718	2.104806	0.076401	0.742739	0.352878	474.6074	3.398456	473.8764	7.970366	471.3087	43.58672	474.6	3.4	100.6999	334
19AR114_Spot 235	120.2901	11139.64	1.680057	16.41402	3.182012	0.631087	3.892156	0.076411	1.015609	0.260937	474.6657	4.647543	496.773	15.29204	600.9095	81.38315	474.7	4.6	78.99122	216
19AR114_Spot 415	168.1637	21149.02	1.522792	17.32374	2.469985	0.6019	2.85858	0.076416	0.822971	0.287895	474.6922	3.766211	478.4385	10.90651	497.3736	60.3289	474.7	3.8	95.43976	388
19AR114_Spot 182	116.6391	11226.02	1.586709	17.15083	4.1779	0.598101	4.585559	0.076455	0.764925	0.166812	474.9301	3.502262	476.028	17.42751	482.3043	99.91286	474.9	3.5	98.47106	170
19AR114_Spot 35	111.1163	10891.36	1.883727	15.60124	5.988472	0.662382	6.205295	0.076476	1.090964	0.175812	475.0539	4.996311	516.0698	25.11068	703.0121	130.1426	475.1	5.0	67.57406	34
19AR114_Spot 70	122.1978	16663.48	1.615522	15.83818	4.562613	0.656506	4.742515	0.076531	1.18649	0.250182	475.385	5.437444	512.4747	19.08691	682.4349	98.08895	475.4	5.4	69.66012	65
19AR114_Spot 169	92.03587	10113.54	1.754884	14.77887	3.557661	0.696471	3.763122	0.076533	1.070336	0.284428	475.3954	4.905234	536.6808	15.68806	807.309	75.526	475.4	4.9	58.88642	157
19AR114_Spot 354	256.003	18044.03	1.491586	16.90693	2.512744	0.615449	2.642713	0.076536	0.771385	0.291891	475.4139	3.535307	486.9909	10.22336	542.7809	55.26405	475.4	3.5	87.58854	328
19AR114_Spot 48	241.5999	14881	1.179728	17.29623	2.619167	0.59653	2.869483	0.07654	0.874904	0.304899	475.4358	4.009919	475.0293	10.88693	474.0084	60.48099	475.4	4.0	100.3011	47
19AR114_Spot 127	140.5088	21437.78	1.767499	16.12648	3.036046	0.646631	3.188294	0.076548	0.851712	0.267137	475.4847	3.904011	506.4034	12.71368	649.5204	65.98713	475.5	3.9	73.20551	119
19AR114_Spot 379	153.1592	14732.65	1.374761	17.0414	4.220042	0.610108	4.487982	0.076557	1.394004	0.310608	475.5407	6.390454	483.6285	17.2693	523.0794	93.6098	475.5	6.4	90.91177	352
19AR114_Spot 200	129.8903	15963.51	2.205002	16.58992	4.322623	0.627134	4.508878	0.076562	0.8972	0.198985	475.568	4.113209	494.3091	17.64734	583.0134	95.98259	475.6	4.1	81.57068	187
19AR114_Spot 249	120.6075	11707.52	1.206351	16.01713	3.91485	0.64606	4.087776	0.076577	0.998981	0.244383	475.6585	4.580665	506.051	16.29226	646.839	85.20345	475.7	4.6	73.53583	228
19AR114_Spot 61	341.5393	27150.92	2.275444	16.95362	2.603035	0.614645	2.830471	0.076585	0.66827	0.236098	475.7062	3.064537	486.4852	10.94089	538.5406	60.19202	475.7	3.1	88.33247	58
19AR114_Spot 130	125.5213	15510.88	1.786196	16.1227	5.965972	0.644903	6.039733	0.076611	0.843517	0.139661	475.8648	3.869427	505.3372	24.04822	642.021	128.6839	475.9	3.9	74.11982	122
19AR114_Spot 424	124.905	6075.046	1.934119	16.88034	4.463742	0.600875	5.138215	0.076613	0.917185	0.178503	475.8768	4.207465	477.7887	19.58496	487.9161	111.6263	475.9	4.2	97.53252	397
19AR114_Spot 259	153.0457	20783.64	1.796589	16.74838	4.99996	0.62084	5.101139	0.076624	0.712388	0.139653	475.9406	3.268405	490.3738	19.8423	559.3351	110.177	475.9	3.3	85.09042	237
19AR114_Spot 89	123.2586	12871.1	1.496163	16.11805	3.499936	0.642935	3.66134	0.076627	0.969284	0.264735	475.9606	4.447214	504.122	14.54945	634.9993	76.00986	476.0	4.4	74.95451	84
19AR114_Spot 223	195.3966	18298.23	17.14479	17.63796	2.98022	0.595063	3.319961	0.07665	0.678223	0.204286	476.0971	3.112642	474.096	12.5768	465.3903	72.02289	476.1	3.1	102.3006	207
19AR114_Spot 147	108.7458	11004.72	2.192519	15.64665	5.177509	0.661722	5.383588	0.076657	1.021389	0.189723	476.1401	4.687982	515.6665	21.77134	695.825	112.6892	476.1	4.7	68.42814	137
19AR114_Spot 60	79.51067	7391.069	1.773565	15.22085	6.213227	0.671112	6.423308	0.07667	1.236135	0.192445	476.2174	5.674512	521.3885	26.19839	725.3997	133.7725	476.2	5.7	65.64896	57
19AR114_Spot 417	122.7814	16806.85	1.685405	16.97885	5.024404	0.614852	5.314547	0.07672	1.330515	0.250353	476.5161	6.111457	486.6153	20.54912	535.4116	112.7089	476.5	6.1	88.99996	390
19AR114_Spot 405	285.0094	23487.19	1.633317	17.55392	2.139077	0.595412	2.441633	0.076723	0.601211	0.246233	476.5309	2.761625	474.3177	9.252667	464.5915	52.43698	476.5	2.8	102.5699	378
19AR114_Spot 197	197.9903	17614.84	1.674418	17.23422	3.181746	0.605037	3.407694	0.076756	0.968481	0.284204	476.7322	4.45047	480.4254	13.04402	499.0281	71.97737	476.7	4.5	95.53212	184
19AR114_Spot 418	113.6716	11654.07	1.822052	17.46551	5.197127	0.593516	5.571767	0.076785	0.958445	0.172018	476.907	4.405908	473.1108	21.0747	455.6866	121.8694	476.9	4.4	104.6568	391
19AR114_Spot 8	66.3051	7164.964	2.392108	14.90272	7.435786	0.690639	7.574176	0.076786	1.325964	0.175064	476.9103	6.095409	533.1841	31.42708	782.7559	156.8924	476.9	6.1	60.92708	8
19AR114_Spot 71	232.1828	20958.86	1.801813	18.92368	2.03021	0.551699	2.631856	0.076802	1.028049	0.390618	477.006	4.726817	446.1091	9.501659	290.7251	55.34723	477.0	4.7	164.0746	66
19AR114_Spot 403	229.6003	28354.14	1.344115	17.68417	2.902934	0.592755	2.993606	0.076808	0.658043	0.219816	477.0395	3.02579	472.6256	11.31277	452.1974	64.85442	477.0	3.0	105.4936	376
19AR114_Spot 7	168.9958	21776.79	6.313444	17.85133	2.922964	0.588326	3.063139	0.076828	0.810074	0.264459	477.1596	3.725755	469.7983	11.5211	434.9411	65.81241	477.2	3.7	109.7067	7
19AR114_Spot 411	93.62504	4746.12	1.532632	15.05178	7.193815	0.673029	8.22847	0.076871	1.047351	0.127284	477.4203	4.819597	522.5524	33.62312	725.8965	173.3303	477.4	4.8	65.76974	384
19AR114_Spot 231	121.9742	8467.553	1.74103	16.43384	4.078433	0.63139	4.312059	0.076884	0.963373	0.223414	477.494	4.433814	496.9611	16.94707	588.6261	91.21085	477.5	4.4	81.12009	213
19AR114_Spot 20	220.6854	20921.33	1.770527	17.74418	2.516716	0.590551	2.890302	0.076895	0.702969	0.243216	477.5598	3.235761	471.2195	10.89682	441.4048	62.39516	477.6	3.2	108.1909	19
19AR114_Spot 162	261.6259	27435.66	1.947797	17.52879	2.58333	0.595847	2.712897	0.076898	0.657425	0.242333	477.5811	3.026253	474.5947	10.28541	461.1291	58.35858	477.6	3.0	103.5678	151
19AR114_Spot 79	161.5082	26776.02	2.471828	16.47038	3.244407	0.638119	3.431671	0.076911	0.857299	0.24982	477.6577	3.946923	501.141	13.57431	610.776	71.83042	477.7	3.9	78.20504	74
19AR114_Spot 357	120.0271	20547.5	2.039246	18.12767	3.645269	0.577228	3.77163	0.076976	0.777802	0.206224	478.0462	3.583731	462.6786	14.0165	388.0488	82.87239	478.0	3.6	123.1923	331
19AR114_Spot 29	500.0537	68150.37	73.94472	17.44148	1.419625	0.60674	1.682531	0.076982	0.885431	0.526249	478.0823	4.079931	481.5022	6.451431	498.7565	31.52584	478.1	4.1	95.85484	28
19AR114_Spot 430	136.9087	13814.58	1.833242	17.12164	3.661984	0.610766	3.821002	0.076983	0.776431	0.203201	478.0916	3.577745	484.043	14.71228	513.2736	82.23879	478.1	3.6	93.14558	403
19AR114_Spot 419	86.02402	8399.636	2.065734	14.78812	5.867445	0.700472	6.00434	0.07699	1.143488	0.190444	478.1298	5.269521	539.0728	25.11916	806.8453	123.4677	478.1	5.3	59.25916	392
19AR114_Spot 40	166.2452	13939.72	1.434703	17.46624	3.192999	0.596183	3.412802	0.077031	0.958011	0.280711	478.374	4.416963	474.8083	12.94378	458.5744	72.68681	478.4	4.4	104.3176	39
19AR114_Spot 338	87.48154	10457	2.166897	14.91193	4.512831	0.698134	4.810862	0.07704	1.335711	0.277645	478.4317	6.159084	537.6755	20.0852	798.4704	96.90227	478.4	6.2	59.91852	312
19AR114_Spot 297	108.863	11453.2	2.667696	17.96627	3.029682	0.579155	3.457892	0.077049	1.045797	0.302438	478.4869	4.8228	463.9182	12.87759	393.3826	73.9343	478.5	4.8	121.634	274
19AR114_Spot 378	138.2493	14082.96	2.137425	15.60263	4.038559	0.670636	4.156417	0.077053	0.902473	0.217128	478.508	4.162023	521.0992	16.94316	713.366	86.24301	478.5	4.2	67.07749	351
19AR114_Spot 140	111.7734	17419.25	1.882151	17.49823	4.166689	0.599284	4.360961	0.077066	0.952167	0.218339	478.5832	4.391868	476.7795	16.59427	469.0615	94.25399	478.6	4.4	102.0299	130
19AR114_Spot 450	96.99502	9296.968	1.928457	15.98664	3.704169	0.652867	4.142405	0.077069	1.215564	0.293444	478.6034	5.607015	510.2412	16.61529	655.5922	84.96304	478.6	5.6	73.00321	421
19AR114_Spot 350	195.6879	16611.19	1.146946	17.14575	3.762656	0.610322	3.995163	0.077069	1.01693	0.25454	478.6059	4.6908	483.7633	15.37602	509.2347	84.97417	478.6	4.7	93.98532	324
19AR114_Spot 306	110.5205	4411.461	2.150197	15.18751	3.836144	0.665445	6.35244	0.077074	0.963695	0.151705	478.6341	4.445495	517.939	25.77778	696.2185	133.9195	478.6	4.4	68.74769	282
19AR114_Spot 364	117.114	10396.2	1.730685	16.9834	4.334597	0.611597	4.550267	0.077077	0.739321	0.162479	478.6539	3.410602	484.5666	17.53552	513.6053	98.68529	478.7	3.4	93.19491	338
19AR114_Spot 157	81.92268	12289.5	1.865836	16.33711	4.828212	0.636503	4.947925	0.077084	0.959406	0.193901	478.6941	4.426248	500.1386	19.54295	600.4255	105.1369	478.7	4.4	79.72582	147
19AR114_Spot 413	136.6231	8829.889	1.7287	17.12328	3.858589	0.604266	4.068359	0.0771	0.821592	0.201947	478.7882	3.791154	479.9377	15.56091	486.3724	87.99058	478.8	3.8	98.44066	386
19AR114_Spot 387	98.29896	13336.82	1.952826	17.2402	4.559664	0.605062	4.915895	0.077105	1.011724	0.205807	478.8214	4.668811	480.4412	18.81873	489.1445	106.2093	478.8	4.7	97.88956	360
19AR114_Spot 262	329.2929	40121.11	0.794632	17.29059	1.668	0.609396	1.929659	0.077121	0.872558	0.452183	478.913	4.027344	483.1791	7.419162	504.4479	37.8851	478.9	4.0	94.93806	240
19AR114_Spot 41	119.3026	11556.4	1.691254	15.86049	4.073401	0.656652	4.666336	0.077169	1.217422	0.260895	479.2044	5.622377	512.5638	18.78275	665.193	96.50371	479.2	5.6	72.0399	40
19AR114_Spot 445	128.5589	16909.25	1.74681	16.24549	4.730199	0.649899	4.941305	0.077172	0.919105	0.186004	479.218	4.244786	508.4166	19.76583	642.9508	104.4017	479.2	4.2	74.53416	417
19AR114_Spot 166	332.3266	32008.61	2.127991	18.02724	2.077891	0.583569	2.232479	0.077192	0.735075	0.329264	479.3377	3.395681	466.7522	8.353763	406.263	47.20598	479.3	3.4	117.987	155
19AR114_Spot 339	263.3766	28232.06	1.52336	17.4257	3.581477	0.605212	3.733629	0.0772	0.798233	0.213795	479.3863	3.687798	480.5357	14.29436	487.0068	80.54237	479.4	3.7	98.43522	313
19AR114_Spot 335	233.8004	11091.9	1.938943	17.34059	3.015419	0.600806	3.212292	0.077241	0.72899	0.226938	479.6348	3.36958	477.7451	12.24226	469.6568	69.2685	479.6	3.4	102.1245	309
19AR114_Spot 171	152.9769	17873.65	1.798042	15.66289	4.906202	0.668931	5.145109	0.077244	0.929987	0.180752	479.6529	4.298797	520.0623	20.94253	702.6777	107.7966	479.7	4.3	68.26073	159
19AR114_Spot 190	333.857	24513.19	2.15573	17.69498	2.101315	0.593502	2.404807	0.077266	0.789978	0.328499	479.7811	3.652556	473.1016	9.09476	441.7813	50.5278	479.8	3.7	108.6015	178
19AR114_Spot 380	151.2436	14686.44	2.244511	17.97699	3.9637	0.583493	4.25037	0.07732	0.673909	0.158553	480.1035	3.117914	466.704	15.9042	402.266	94.03771	480.1	3.1	119.3498	353
19AR114_Spot 332	174.02	19707.69	1.954612	16.53677	3.450196	0.637023	3.697073	0.077339	0.829742	0.224432	480.217	3.839767	500.4616	14.60893	595.0754	78.09631	480.2	3.8	80.69853	306
19AR114_Spot 368	116.5876	10940.76	1.404554	16.436	4.743456	0.635462	5.198581	0.07736	0.8876	0.170739	480.3439	4.108563	499.4928	20.51271	589.1445	111.1898	480.3	4.1	81.53245	341
19AR114_Spot 230	177.2858	14963.13	1.290995	17.96407	2.752469	0.587741	2.930098	0.077385	0.818862	0.279466	480.4981	3.791556	469.424	11.01375	416.5842	62.86441	480.5	3.8	115.3424	212
19AR114_Spot 375	218.5842	22166.46	1.599096	17.62129	2.804033	0.59971	3.074227	0.077401	0.796781	0.259181	480.5924	3.690011	477.0495	11.70267	460.9941	65.85841	480.6	3.7	104.2513	348
19AR114_Spot 449	127.5581	10907.64	2.386852	16.08985	4.809357	0.653918	4.912054	0.077411	0.678782	0.138187	480.6498	3.143903	510.8867	19.72227	649.5138	104.5202	480.6	3.1	74.00148	420
19AR114_Spot 352	85.0243	7950.234	1.755048	15.93509	8.131561	0.650732	8.230622	0.077427	1.097934	0.133396	480.7471	5.086276	508.9289	32.95645	638.594	175.7485	480.7	5.1	75.28212	326
19AR114_Spot 287	116.4586	10342.22	2.043117	15.57888	4.375509	0.67333	4.490553	0.077438	0.939886	0.209303	480.811	4.354659	522.7352	18.34947	711.2723	93.37693	480.8	4.4	67.59872	264
19AR114_Spot 401	90.23836	10180.54	1.907338	16.17505	5.830525	0.644959	5.962177	0.077442	0.826087	0.138555	480.837	3.827609	505.372	23.74056	618.9373	127.5206	480.8	3.8	77.68751	374
19AR114_Spot 77	191.8948	16949.59	1.968064	17.46583	2.612265	0.601949	2.828331	0.077482	0.97547	0.344893	481.0755	4.521923	478.47	10.79164	466.986	58.8254	481.1	4.5	103.0171	72
19AR114_Spot 391	97.78699	12204.8	1.955569	15.40025	3.922341	0.681207	4.292213	0.077496	0.92223	0.214861	481.1615	4.275858	527.5036	17.6609	734.3667	88.78426	481.2	4.3	65.52061	364
19AR114_Spot 25	216.2559	23963.02	1.763081	17.27238	3.212117	0.612986	3.32481	0.077546	0.762548	0.229351	481.4588	3.537607	485.4416	12.83039	505.2605	71.23794	481.5	3.5	95.28922	24
19AR114_Spot 136	354.8971	44555.51	1.652145	17.59896	2.086883	0.605151	2.272242	0.07757	0.806311	0.354853	481.5991	3.741679	480.4972	8.69847	476.1822	46.9773	481.6	3.7	101.1376	127
19AR114_Spot 399	92.52911	6680.811	2.052408	16.00387	5.658581	0.644952	5.868302	0.077617	1.083813	0.184689	481.8806	5.032259	505.3673	23.36646	614.0535	124.6474	481.9	5.0	78.47535	372
19AR114_Spot 241	1112.007	136341.3	1.735935	17.73316	1.139285	0.603485	1.521606	0.077626	1.000769	0.657705	481.9345	4.647174	479.4426	5.814847	468.5255	25.38271	481.9	4.6	102.862	221
19AR114_Spot 198	235.6055	19459.94	1.993509	17.87439	2.237811	0.590561	2.417675	0.077637	0.784949	0.324671	482.0011	3.64548	471.2258	9.11494	420.0348	51.06816	482.0	3.6	114.7527	185
19AR114_Spot 184	101.4441	12165.1	1.666524	15.76357	4.267799	0.664384	4.393913	0.077657	0.870567	0.19813	482.1208	4.044073	517.2918	17.81113	676.768	92.08308	482.1	4.0	71.23871	172
19AR114_Spot 304	240.051	16563.49	1.819663	17.21014	2.113836	0.613325	2.340849	0.077669	0.783145	0.334556	482.1961	3.638516	485.6548	9.036149	502.9706	48.55459	482.2	3.6	95.86963	281
19AR114_Spot 286	78.71194	9443.356	1.926384	15.72832	6.809652	0.667406	6.919825	0.077677	1.056672	0.152702	482.2417	4.909778	519.1338	28.13097	685.9043	146.1236	482.2	4.9	70.30744	263
19AR114_Spot 386	772.3184	58755.37	0.639302	17.49275	1.089585	0.609767	1.297329	0.077698	0.698091	0.538099	482.3651	3.244448	483.4133	4.989816	489.369	24.11191	482.4	3.2	98.56878	359
19AR114_Spot 394	195.4447	23720.05	1.261615	17.536	2.709769	0.605408	3.073488	0.077857	0.987636	0.32134	483.3171	4.598861	480.66	11.76911	468.9893	64.45731	483.3	4.6	103.055	367
19AR114_Spot 438	139.6871	20135.9	1.970502	15.85626	3.889	0.67254	4.085482	0.077896	0.874905	0.21415	483.5537	4.075859	522.2554	16.68221	696.2077	85.06665	483.6	4.1	69.45538	411
19AR114_Spot 173	88.8716	7692.774	1.964875	15.23956	5.331422	0.681392	5.592445	0.077902	1.309503	0.234156	483.5886	6.100911	527.6155	23.01623	723.8355	115.4281	483.6	6.1	66.80918	161
19AR114_Spot 328	177.0881	12024.35	1.743531	17.13164	3.434615	0.615962	3.887287	0.07791	0.915393	0.235484	483.636	4.265176	487.3131	15.04635	505.5819	83.16258	483.6	4.3	95.65928	302
19AR114_Spot 349	138.9704	20599.33	1.861652	16.70224	4.955357	0.636023	5.148461	0.077936	1.180291	0.229251	483.7931	5.501157	499.841	20.32586	574.9606	109.0303	483.8	5.5	84.14369	323
19AR114_Spot 373	76.68989	7404.595	2.557598	15.42166	5.867084	0.676523	6.111593	0.077942	0.984706	0.161121	483.8296	4.5899	524.6711	25.04642	707.5465	128.3735	483.8	4.6	68.38132	346
19AR114_Spot 26	247.1982	20432.43	1.468795	16.99873	2.42207	0.625853	2.764495	0.078046	0.842207	0.304651	484.4481	3.930519	493.5093	10.80571	536.7128	57.64463	484.4	3.9	90.26207	25
19AR114_Spot 272	119.9213	12473.88	3.721456	15.51314	4.802664	0.678411	5.189894	0.078048	0.916469	0.176587	484.4609	4.2772	525.8138	21.30329	710.5909	108.679	484.5	4.3	68.17719	249
19AR114_Spot 353	371.0579	32029.41	2.164917	17.31492	1.535529	0.616493	1.790652	0.078061	0.81334	0.454214	484.5413	3.796501	487.6469	6.934295	503.2273	35.12263	484.5	3.8	96.28676	327
19AR114_Spot 273	172.0506	22065.27	4.4906	15.89965	3.704806	0.66908	3.884764	0.0782	0.992115	0.255386	485.3711	4.63862	520.1529	15.81359	676.8745	80.30553	485.4	4.6	71.70769	250
19AR114_Spot 320	132.8933	13302.89	1.901346	15.53825	5.166531	0.685801	5.34409	0.07825	0.839618	0.157112	485.6676	3.927934	530.2746	22.07822	728.1095	111.9665	485.7	3.9	66.70255	296
19AR114_Spot 199	113.5655	12349.34	3.148998	16.81773	4.255599	0.629713	4.797372	0.078277	1.337395	0.278777	485.8317	6.258686	495.917	18.82412	543.7022	100.7701	485.8	6.3	89.35621	186
19AR114_Spot 362	89.5934	7662.394	2.312266	15.8161	5.422834	0.662528	5.740864	0.078335	0.917606	0.159838	486.1793	4.297134	516.1594	23.2337	652.1231	121.7035	486.2	4.3	74.55331	336
19AR114_Spot 275	119.4297	15292.62	1.644064	15.39605	5.04938	0.690564	5.540902	0.07838	1.817111	0.327945	486.447	8.514012	533.139	22.98565	739.2005	110.8181	486.4	8.5	65.80718	252
19AR114_Spot 431	51.53126	5954.444	2.312307	12.20883	12.06781	0.862671	12.32003	0.07841	0.949378	0.07706	486.6279	4.44987	631.58	57.99929	1192.741	243.2779	486.6	4.4	40.79913	404
19AR114_Spot 396	133.3352	11804.51	2.256811	16.78005	3.701862	0.630987	3.853258	0.078416	0.781109	0.202714	486.6581	3.661388	496.7103	15.1377	544.2647	82.47165	486.7	3.7	89.4157	369
19AR114_Spot 421	176.0496	13206.56	2.106576	17.42888	4.226436	0.611273	4.423752	0.07869	0.929104	0.210026	488.2985	4.369235	484.3628	17.04228	466.7236	95.83015	488.3	4.4	104.6226	394
19AR114_Spot 359	119.5672	10398.55	1.835215	16.57388	4.208471	0.640029	4.409174	0.078773	0.761502	0.172708	488.7964	3.584577	502.3241	17.47343	565.3528	94.61515	488.8	3.6	86.45864	333
19AR114_Spot 346	98.46008	12625.14	0.768582	16.44833	3.951777	0.648359	4.393044	0.078853	1.490204	0.339219	489.2716	7.021324	507.4684	17.54698	591.278	89.63803	489.3	7.0	82.74816	320
19AR114_Spot 370	673.3228	63260.45	4.285573	17.69788	1.310184	0.612578	1.507359	0.078894	0.659745	0.437683	489.5151	3.109976	485.1849	5.81423	465.7245	30.02537	489.5	3.1	105.1083	343
19AR114_Spot 313	460.836	28091.45	8.598972	17.43905	1.976212	0.619583	2.161564	0.079152	0.768558	0.355556	491.0598	3.633913	489.5858	8.396612	483.6773	44.64029	491.1	3.6	101.5263	289
19AR114_Spot 289	175.301	16906.78	2.743813	17.00527	3.942107	0.637158	4.055932	0.079389	0.703942	0.173559	492.4718	3.337603	500.5449	16.02924	538.5887	87.41438	492.5	3.3	91.43745	266
19AR114_Spot 446	435.7903	32850.81	2.873404	17.39621	1.596072	0.633364	1.749324	0.080032	0.609013	0.348142	496.3121	2.909179	498.1894	6.887748	507.8017	36.073	496.3	2.9	97.73739	418
19AR114_Spot 333	199.1674	25592.06	2.87844	17.44984	3.115636	0.634262	3.242398	0.081044	0.789591	0.243521	502.3529	3.815921	498.7474	12.7781	483.2176	69.46837	502.4	3.8	103.96	307
19AR114_Spot 119	78.39057	7987.168	2.192919	14.96902	6.608706	0.729673	7.351659	0.081514	1.686934	0.229463	505.1558	8.196315	556.361	31.50059	772.7078	150.7643	505.2	8.2	65.37475	112
19AR114_Spot 105	377.7444	11142.53	4.595846	17.1337	2.106766	0.648309	3.106926	0.082369	0.811088	0.261058	510.2478	3.979008	507.4376	12.40869	495.7532	66.10703	510.2	4.0	102.9238	98
19AR114_Spot 126	223.4909	32921.05	3.460745	16.93503	2.945996	0.665429	3.096568	0.082438	0.91934	0.29689	510.6595	4.513562	517.9297	12.56344	551.0679	64.54221	510.7	4.5	92.66727	118
19AR114_Spot 163	101.9372	11497.16	1.248378	16.45966	6.596717	0.67549	6.824641	0.082625	0.833801	0.122175	511.7748	4.102194	524.0447	27.9445	578.7972	147.3479	511.8	4.1	88.42039	152
19AR114_Spot 308	86.52585	4524.183	1.954114	15.92445	3.973638	0.678276	5.743065	0.082674	1.084514	0.188839	512.0646	5.338576	525.7317	23.5719	586.4979	122.475	512.1	5.3	87.30884	284
19AR114_Spot 102	349.8329	31108.31	5.712331	17.3371	1.480682	0.65347	1.707549	0.082735	0.814324	0.476896	512.426	4.011268	510.6122	6.852346	503.4553	33.02534	512.4	4.0	101.7818	95
19AR114_Spot 103	96.82241	11138.39	5.73992	16.41523	4.049018	0.680911	4.265299	0.082799	0.913271	0.214117	512.8083	4.501895	527.3248	17.5456	591.6205	90.35116	512.8	4.5	86.67859	96
19AR114_Spot 294	135.8648	17722.88	1.543249	17.86531	3.350975	0.632857	3.524909	0.082947	0.674361	0.191313	513.6918	3.329712	497.8739	13.87274	426.7397	77.18455	513.7	3.3	120.3759	271

19AR114_Spot 85	106.6374	9156.364	3.011056	16.10472	3.191556	0.692225	4.232263	0.083019	1.012424	0.239216	514.1205	5.002931	534.1363	17.58065	621.4817	88.68535	514.1	5.0	82.72496	80
19AR114_Spot 112	189.3803	7699.445	2.394398	16.36143	2.637473	0.678201	3.250214	0.083097	0.929067	0.285848	514.579	4.594952	525.6862	13.33771	575.1842	67.73065	514.6	4.6	89.46335	105
19AR114_Spot 74	334.1397	32326.15	1.300528	17.34455	1.89784	0.655707	2.155937	0.083208	0.966227	0.44817	515.2411	4.784641	511.9847	8.669676	498.4092	42.47507	515.2	4.8	103.3771	69
19AR114_Spot 83	62.76811	7771.932	1.7279	14.01836	8.557798	0.796874	8.701839	0.083231	1.354119	0.155613	515.3817	6.707199	595.0637	39.20385	912.6762	177.2578	515.4	6.7	56.46928	78
19AR114_Spot 204	264.8477	4999.739	1.410582	16.06515	2.829042	0.682176	3.058841	0.08324	0.930833	0.304309	515.4328	4.611021	528.089	12.59605	584.0983	63.2866	515.4	4.6	88.24419	191
19AR114_Spot 14	43.80024	2506.172	1.875742	13.59887	7.314904	0.779129	9.23126	0.083282	1.149444	0.124516	515.6812	5.696582	584.9865	41.07046	864.94	190.3398	515.7	5.7	59.62046	13
19AR114_Spot 180	86.19276	8113.878	1.314328	14.57937	5.024625	0.763748	5.275903	0.083298	1.15202	0.218355	515.7781	5.71038	576.17	23.20149	823.0454	107.5621	515.8	5.7	62.66703	168
19AR114_Spot 58	199.6098	20461.51	3.160415	16.69609	3.134847	0.677156	3.361772	0.083325	0.859015	0.255524	515.9379	4.259265	525.054	13.78288	565.8629	70.79066	515.9	4.3	91.17719	55
19AR114_Spot 243	87.16518	8830.178	1.445387	17.25829	5.065218	0.647924	5.324889	0.083326	1.206585	0.226593	515.9439	5.982697	507.2004	21.26135	468.9325	114.8909	515.9	6.0	110.0252	223
19AR114_Spot 82	198.8996	24332.12	3.760834	16.45172	3.547135	0.691918	3.721808	0.083375	0.821262	0.220662	516.2352	4.074329	533.9522	15.45584	611.3038	78.46171	516.2	4.1	84.44822	77
19AR114_Spot 402	138.9848	11698.34	2.682855	15.90665	4.701285	0.709328	4.923563	0.083386	0.793167	0.161096	516.3033	3.935448	544.3471	20.74873	664.5132	104.1401	516.3	3.9	77.69647	375
19AR114_Spot 203	65.26135	7139.881	1.766195	14.40931	9.511854	0.776354	9.789763	0.083426	1.175394	0.120064	516.5427	5.834537	583.4014	43.47079	853.8898	202.3553	516.5	5.8	60.4929	190
19AR114_Spot 6	89.02153	11479.91	2.608062	16.00308	5.608892	0.708465	5.806312	0.083633	1.143505	0.196942	517.7707	5.689202	543.8346	24.45269	655.5736	122.1951	517.8	5.7	78.9798	6
19AR114_Spot 234	107.342	11813.61	1.537186	14.96368	4.966255	0.760259	5.272338	0.083649	1.726132	0.327394	517.8684	8.589466	574.1593	23.12561	804.6337	104.345	517.9	8.6	64.36077	215
19AR114_Spot 47	488.4867	7188.759	1.722628	16.53854	1.653475	0.670462	2.147001	0.08366	0.786414	0.366285	517.9308	3.91375	520.9935	8.750051	535.401	43.72004	517.9	3.9	96.737	46
19AR114_Spot 120	65.38437	8792.166	1.907224	14.66522	6.374677	0.766815	6.650501	0.083666	1.069518	0.160818	517.9674	5.323042	577.9341	29.31598	822.2113	137.194	518.0	5.3	62.99687	113
19AR114_Spot 28	103.1783	9882.886	1.845376	14.82057	3.333822	0.763212	3.609878	0.083688	0.860776	0.23845	518.0974	4.285155	575.8617	15.86714	811.8253	73.34659	518.1	4.3	63.81883	27
19AR114_Spot 116	79.95084	10862.5	1.643759	15.77229	5.608325	0.715439	5.739076	0.083697	0.809873	0.141116	518.1524	4.032158	547.9706	24.30816	674.8685	121.5595	518.2	4.0	76.77827	109
19AR114_Spot 107	204.6832	20946.24	3.080922	17.1396	2.280445	0.666233	2.424853	0.083709	0.79004	0.325809	518.225	3.933941	518.4195	9.845082	520.2119	50.32864	518.2	3.9	99.61806	100
19AR114_Spot 448	294.5119	5905.237	7.064462	17.3787	2.978037	0.640293	3.177153	0.083797	0.817248	0.257227	518.746	4.073353	502.4878	12.59354	430.0704	68.44621	518.7	4.1	120.6188	419
19AR114_Spot 148	49.58441	5070.035	1.610823	13.68012	5.32178	0.813283	5.8179	0.08392	1.83451	0.315322	519.4808	9.156061	604.2939	26.50152	937.6102	113.2845	519.5	9.2	55.40478	138
19AR114_Spot 32	123.6136	5665.096	1.294709	14.27128	4.456051	0.782051	4.667918	0.083921	0.893914	0.191502	519.4825	4.461546	586.653	20.80314	856.809	95.1874	519.5	4.5	60.62991	31
19AR114_Spot 97	1136.423	10605.89	14.56095	16.82406	0.898768	0.674817	1.368564	0.083937	0.865362	0.632313	519.5768	4.319795	523.6369	5.599099	542.3272	23.18113	519.6	4.3	95.80505	90
19AR114_Spot 10	220.9637	26306.33	2.25159	17.14796	1.885802	0.670056	2.161859	0.08395	0.852722	0.394439	519.656	4.257319	520.7466	8.807411	526.508	43.53314	519.7	4.3	98.69859	9
19AR114_Spot 210	82.82007	4283.193	1.674467	17.74012	3.844041	0.616044	5.370545	0.084016	1.362768	0.253749	520.0509	6.808758	487.365	20.79063	337.4686	117.7562	520.1	6.8	154.1035	196
19AR114_Spot 296	173.7077	14880.73	3.355172	17.53502	3.115393	0.650885	3.278721	0.084021	0.665356	0.202932	520.0778	3.324464	509.0229	13.12641	460.607	71.21673	520.1	3.3	112.9114	273
19AR114_Spot 185	123.0542	14958.18	2.236175	16.36434	3.424782	0.694664	3.737547	0.084044	0.957034	0.256059	520.2176	4.783072	535.5991	15.55758	602.5739	78.21312	520.2	4.8	86.33258	173
19AR114_Spot 11	118.4282	16547.58	2.199235	16.27278	4.764377	0.703935	4.975768	0.084098	0.818199	0.164437	520.5361	4.091603	541.1386	20.87521	629.8238	105.8122	520.5	4.1	82.6479	10
19AR114_Spot 91	128.0132	6380.637	1.754154	16.7073	4.443769	0.666999	5.053488	0.084107	1.178275	0.233161	520.5935	5.892876	518.8859	20.53383	512.3275	108.0459	520.6	5.9	101.6134	86
19AR114_Spot 53	86.46728	7551.065	3.130141	15.01766	5.496517	0.745019	5.663848	0.084111	1.082653	0.191151	520.6138	5.414847	565.3304	24.55803	750.4826	117.5007	520.6	5.4	69.37054	52
19AR114_Spot 211	124.0003	1811.877	1.7235	17.47761	4.299069	0.572127	4.543499	0.084132	0.846049	0.186211	520.7391	4.232457	459.3893	16.79055	164.1039	104.3906	520.7	4.2	317.3227	197
19AR114_Spot 261	125.5214	14666.31	2.394598	17.52979	3.2933	0.647557	3.655218	0.084136	0.901602	0.246662	520.7639	4.510575	506.9743	14.5885	446.1847	78.75399	520.8	4.5	116.7149	239
19AR114_Spot 423	104.0252	6139.91	2.002802	16.25737	6.030955	0.686886	6.463472	0.084147	0.825047	0.127648	520.8281	4.12807	530.9278	26.72976	575.5301	139.4971	520.8	4.1	90.49537	396
19AR114_Spot 146	165.0922	21581.67	3.036524	17.89358	4.052033	0.640696	4.248448	0.084154	0.96672	0.227547	520.8712	4.837307	502.7367	16.84705	421.979	92.35202	520.9	4.8	123.4353	136
19AR114_Spot 191	100.4768	13344.26	3.141624	15.87273	4.852211	0.716042	4.943811	0.084187	0.892796	0.180589	521.0658	4.469006	548.3275	20.94903	664.2214	104.2097	521.1	4.5	78.44761	179
19AR114_Spot 412	73.89724	6285.823	1.835891	14.70036	5.575492	0.763954	5.798056	0.084198	1.34744	0.232395	521.129	6.745573	576.2886	25.50255	801.107	118.2242	521.1	6.7	65.05111	385
19AR114_Spot 183	124.7498	9379.97	2.473462	15.88898	4.926992	0.709844	5.060625	0.084233	0.852187	0.168396	521.3402	4.267892	544.6535	21.33557	644.3934	107.2568	521.3	4.3	80.90403	171
19AR114_Spot 129	79.324	7802.114	1.568078	14.82116	7.152335	0.761739	7.268927	0.084236	1.008798	0.138782	521.3602	5.05241	575.0129	31.92331	794.0537	151.1545	521.4	5.1	65.65806	121
19AR114_Spot 189	81.9086	8927.173	1.428481	14.73339	5.934998	0.767016	6.138094	0.084267	1.113968	0.181484	521.5415	5.580999	578.0496	27.06015	807.7575	126.4153	521.5	5.6	64.56659	177
19AR114_Spot 90	147.878	21877.27	3.711631	18.09851	2.956796	0.633707	3.16676	0.084288	0.823062	0.259907	521.6653	4.124494	498.4027	12.4733	393.8927	68.61756	521.7	4.1	132.4384	85
19AR114_Spot 42	88.32375	8672.332	1.907182	15.47506	4.078477	0.730832	4.685828	0.084308	1.265278	0.270022	521.7874	6.341935	557.0413	20.09257	704.7537	96.0472	521.8	6.3	74.03826	41
19AR114_Spot 50	162.3907	19618.23	3.392842	17.64053	3.568161	0.645947	3.873717	0.084313	0.839537	0.216727	521.8152	4.208215	505.9816	15.43733	435.9762	84.20572	521.8	4.2	119.6889	49
19AR114_Spot 150	46.94624	3192.688	2.298265	12.61688	11.13194	0.868963	11.55336	0.084379	1.821104	0.157625	522.2079	9.134959	635.004	54.59553	1061.079	230.2851	522.2	9.1	49.21479	140
19AR114_Spot 22	54.90786	5071.246	1.800961	13.36452	10.15121	0.838708	10.31131	0.08441	1.396655	0.135449	522.39	7.008196	618.4321	47.79276	988.6277	208.353	522.4	7.0	52.83991	21
19AR114_Spot 220	298.8369	18474.47	6.097652	17.79069	1.721904	0.649924	1.954515	0.084442	0.718326	0.367521	522.5796	3.605702	508.4321	7.81765	446.2416	40.40801	522.6	3.6	117.1069	205
19AR114_Spot 232	100.8857	11589.66	1.754766	16.59536	4.472532	0.691993	4.891951	0.08445	1.176954	0.24059	522.6303	5.908378	533.9974	20.31764	583.7833	103.1314	522.6	5.9	89.52471	214
19AR114_Spot 139	162.343	16912.47	2.97172	15.92371	3.066327	0.722395	3.215354	0.084451	0.756107	0.235155	522.6358	3.795738	552.0797	13.69389	676.38	66.82372	522.6	3.8	77.26955	129
19AR114_Spot 363	167.2355	18498.08	2.709735	16.95987	2.100227	0.678395	2.305385	0.084475	0.783264	0.339754	522.7775	3.933094	525.804	9.461814	539.9398	47.42899	522.8	3.9	96.82144	337
19AR114_Spot 23	109.9709	12704.08	1.654144	16.56678	4.217646	0.690285	4.441887	0.084478	0.824886	0.185706	522.7988	4.142256	532.9714	18.42101	577.6992	94.89145	522.8	4.1	90.49672	22
19AR114_Spot 309	226.6196	12899.82	4.555782	17.09304	3.355946	0.667332	3.699425	0.084483	0.675006	0.182462	522.8238	3.389772	519.0889	15.03543	503.6356	80.09189	522.8	3.4	103.8099	285
19AR114_Spot 73	56.87086	7048.255	1.79705	15.35378	7.22235	0.733973	7.895413	0.08451	2.248932	0.28484	522.9888	11.29722	558.8821	33.94722	708.7835	161.1633	523.0	11.3	73.78682	68
19AR114_Spot 224	171.9342	18069.51	1.693315	16.16892	3.055833	0.718278	3.239143	0.084522	0.770287	0.237806	523.0604	3.869943	549.6498	13.74946	662.3392	67.44214	523.1	3.9	78.97169	208
19AR114_Spot 393	89.58325	15659.42	4.588468	16.37337	6.211611	0.701105	6.394965	0.084537	1.031001	0.161221	523.1477	5.180604	539.4505	26.76828	609.8869	136.5307	523.1	5.2	85.77782	366
19AR114_Spot 340	243.1371	25100.36	1.882578	17.27631	2.086138	0.667899	2.304722	0.084553	0.641257	0.278236	523.243	3.222771	519.4343	9.371345	503.6583	48.72109	523.2	3.2	103.8885	314
19AR114_Spot 205	82.24262	2256.891	2.169153	14.70896	6.55446	0.719041	6.839534	0.084555	0.825433	0.120686	523.2559	4.148486	550.1008	29.05645	663.8227	145.6197	523.3	4.1	78.82464	192
19AR114_Spot 283	87.5591	9973.89	2.158022	14.68923	7.246292	0.778963	7.478046	0.084589	1.025476	0.137132	523.4565	5.155765	584.8919	33.26009	832.1105	154.6314	523.5	5.2	62.9071	260
19AR114_Spot 117	120.2455	16062.33	2.011325	17.03231	3.594108	0.673511	3.85507	0.084605	0.71476	0.185408	523.5492	3.594194	522.8448	15.75481	520.7025	83.1528	523.5	3.6	100.5467	110
19AR114_Spot 282	123.1476	14688.34	1.666781	16.75373	4.659041	0.685875	4.931522	0.084638	0.729261	0.147877	523.7457	3.668432	530.3192	20.37459	559.6454	106.3722	523.7	3.7	93.58527	259
19AR114_Spot 131	121.4115	14685.18	1.49944	16.59175	2.737924	0.691584	2.962208	0.084662	0.918239	0.309985	523.8916	4.620293	533.7518	12.29753	577.0873	61.24221	523.9	4.6	90.78204	123
19AR114_Spot 292	142.7338	5921.641	2.485685	16.11875	3.714571	0.69912	5.318581	0.084702	0.728847	0.137038	524.1305	3.668936	538.2653	22.22403	599.5436	114.1253	524.1	3.7	87.42159	269
19AR114_Spot 24	246.4978	30169.4	7.627673	16.70932	2.102975	0.694073	2.278544	0.084732	0.75336	0.330632	524.3057	3.793551	535.2445	9.479228	583.0622	46.71159	524.3	3.8	89.92278	23
19AR114_Spot 33	95.47267	10527.32	1.705823	16.08089	4.14353	0.71049	4.323244	0.084753	0.910799	0.210675	524.4309	4.587384	545.037	18.23575	633.1132	91.01337	524.4	4.6	82.83368	32
19AR114_Spot 114	82.34497	7345.508	2.482205	15.19057	6.905293	0.745709	7.508377	0.084785	1.120195	0.149193	524.6236	5.644033	565.7314	32.57785	735.5867	157.3732	524.6	5.6	71.32043	107
19AR114_Spot 186	221.177	18803.17	6.25952	16.30935	2.713476	0.705584	3.003065	0.084795	0.816374	0.271847	524.682	4.113686	542.1209	12.61516	617.0595	62.40386	524.7	4.1	85.0294	174
19AR114_Spot 21	114.2342	9888.774	1.679228	15.95878	5.379609	0.716236	5.476833	0.084819	0.829977	0.151543	524.8254	4.183329	548.4423	23.21208	648.7431	116.3384	524.8	4.2	80.8988	20
19AR114_Spot 115	54.56829	5594.821	1.540194	12.78025	9.417869	0.884353	9.61169	0.084825	1.364083	0.141919	524.8582	6.875798	643.331	45.83401	1085.728	191.1553	524.9	6.9	48.34161	108
19AR114_Spot 326	169.563	20815.18	1.526783	17.02759	3.314804	0.682289	3.477603	0.084881	0.654657	0.188249	525.1938	3.301889	528.1568	14.32209	541.9366	74.68271	525.2	3.3	96.91057	300
19AR114_Spot 227	128.2374	12679.41	1.814765	17.40682	3.798953	0.665482	4.278916	0.08491	0.859079	0.20077	525.3633	4.334274	517.9618	17.36211	486.3849	92.58119	525.4	4.3	108.0139	210
19AR114_Spot 384	74.04417	5836.945	2.719244	15.30975	7.887894	0.73639	8.267326	0.084984	1.332745	0.161206	525.801	6.729417	560.2968	35.61506	703.9356	173.9225	525.8	6.7	74.69447	357
19AR114_Spot 301	170.156	14880.33	5.993224	17.19057	3.72468	0.670727	4.080253	0.084987	0.77824	0.190733	525.8237	3.929724	521.1541	16.63397	501.6842	88.20873	525.8	3.9	104.8117	278
19AR114_Spot 392	113.9458	16869.56	1.822097	15.98729	4.615904	0.722933	4.768389	0.084989	0.867397	0.181906	525.8339	4.380001	552.3972	20.31839	664.4253	100.4857	525.8	4.4	79.14117	365
19AR114_Spot 110	362.4751	43351.52	6.92998	17.10464	1.64518	0.681602	1.822501	0.085007	0.73597	0.403824	525.9375	3.71705	527.7419	7.500895	536.5027	36.48943	525.9	3.7	98.03073	103
19AR114_Spot 267	547.1977	75089.05	3.10234	17.33505	1.236894	0.671274	1.468707	0.085034	0.786847	0.535741	526.0989	3.975177	521.487	5.98994	502.2862	27.32098	526.1	4.0	104.7409	244
19AR114_Spot 398	307.8011	24491.03	6.679213	17.05388	2.132337	0.680611	2.34337	0.085049	0.824028	0.351642	526.1895	4.163709	527.1437	9.636419	532.2511	48.04256	526.2	4.2	98.86115	371
19AR114_Spot 381	198.7007	11144.46	2.971371	17.10338	3.189822	0.672153	3.493629	0.085126	0.978441	0.280064	526.6449	4.94804	522.0205	14.26026	502.7879	73.8513	526.6	4.9	104.7449	354
19AR114_Spot 330	71.86288	7951.545	1.611619	15.24844	6.398429	0.748641	6.985479	0.085143	0.964129	0.138019	526.7492	4.876594	567.4356	30.37586	734.9827	146.6445	526.7	4.9	71.66825	304
19AR114_Spot 406	139.4426	16580.69	1.604944	16.33685	5.329963	0.707584	5.432051	0.085147	0.685658	0.126225	526.7712	3.468218	543.3108	22.85931	614.235	116.4656	526.8	3.5	85.76053	379
19AR114_Spot 420	150.4688	20831.05	2.895499	16.04969	3.012138	0.724258	3.178111	0.085156	0.789739	0.248493	526.8267	3.995084	553.1772	13.5555	664.1363	65.96527	526.8	4.0	79.3251	393
19AR114_Spot 404	113.4919	12121.6	1.726694	15.92915	2.641606	0.722538	3.288488	0.085185	0.837633	0.254717	526.9993	4.238704	552.164	14.00701	658.28	68.18045	527.0	4.2	80.05701	377
19AR114_Spot 325	90.63407	8565.062	1.2487	15.49684	5.208542	0.741596	5.602975	0.085193	1.093814	0.19522	527.0444	5.535518	563.3364	24.22986	713.6899	116.8301	527.0	5.5	73.84782	299
19AR114_Spot 158	213.0558	21403.16	1.150723	17.13842	2.537862	0.674957	2.690472	0.085206	0.63301	0.235279	527.1244	3.203972	523.7216	11.00899	509.8806	57.5168	527.1	3.2	103.3819	148
19AR114_Spot 345	94.89992	9912.934	1.932217	17.12391	3.466975	0.66898	3.680266	0.085244	0.98397	0.267364	527.3477	4.982375	520.0921	14.97969	489.3083	78.2795	527.3	5.0	107.7741	319
19AR114_Spot 310	635.0373	86933.34	10.05125	17.20819	1.106264	0.680253	1.260151	0.085275	0.596172	0.473096	527.5295	3.01974	526.9273	5.180265	525.2521	24.33208	527.5	3.0	100.4336	286
19AR114_Spot 302	171.222	19488.92	3.692155	16.97361	3.083133	0.68291	3.272489	0.085276	0.805433	0.246122	527.5379	4.079754	528.5317	13.48454	533.7784	69.45357	527.5	4.1	98.83088	279
19AR114_Spot 194	233.536	20813.95	2.133214	16.79563	2.284825	0.690622	2.619337	0.085289	0.842516	0.321652	527.6127	4.268173	533.1744	10.86506	557.9956	54.06514	527.6	4.3	94.55498	181
19AR114_Spot 300	104.4539	12297.85	2.451949	16.29228	4.843662	0.708663	4.994372	0.08531	0.986156	0.197453	527.738	4.996991	543.9518	21.03566	613.3986	105.8149	527.7	5.0	86.03509	277
19AR114_Spot 219	109.628	3065.914	2.024066	16.33123	6.39794	0.669407	6.630052	0.085329	1.097203	0.165489	527.8533	5.560848	520.3518	27.00083	488.5327	144.4305	527.9	5.6	108.0487	204
19AR114_Spot 312	108.0841	11802.83	2.454438	15.53191	4.784387	0.741112	4.962318	0.085349	0.781362	0.157459	527.9706	3.960946	563.0543	21.45044	708.3976	104.2878	528.0	4.0	74.53027	288
19AR114_Spot 382	220.8034	30292.25	1.69021	16.9544	2.70955	0.689787	2.819488	0.08536	0.724001	0.256784	528.036	3.670599	532.6727	11.68699	553.5126	59.47008	528.0	3.7	95.39728	355
19AR114_Spot 355	88.89477	9552.139	1.602199	15.37405	6.354816	0.747539	6.447524	0.085365	0.935107	0.145033	528.0688	4.741168	566.7953	28.01173	726.2982	135.3907	528.1	4.7	72.70689	329
19AR114_Spot 383	98.77712	15146.21	1.42505	15.3634	5.564092	0.755683	5.675292	0.085379	0.958411	0.168874	528.1516	4.860055	571.5163	24.80836	748.886	118.2398	528.2	4.9	70.52496	356
19AR114_Spot 75	96.73144	16893.85	1.769364	15.64292	4.610238	0.741962	4.837359	0.085395	1.162353	0.240287	528.2433	5.895217	563.5497	20.9239	709.7083	99.90464	528.2	5.9	74.43104	70
19AR114_Spot 151	163.2311	17160.33	2.901801	16.74511	2.988869	0.692182	3.307156	0.085402	0.820647	0.248143	528.2849	4.162468	534.1104	13.73676	559.9982	69.86929	528.3	4.2	94.33689	141
19AR114_Spot 179	139.7389	14556.06	1.458764	16.5808	3.231488	0.695265	3.582689	0.085424	0.981776	0.274033	528.4154	4.980923	535.9591	14.92049	569.1608	75.02767	528.4	5.0	92.84115	167
19AR114_Spot 78	154.8639	26284.02	2.447832	16.36508	4.01898	0.713602	4.134125	0.085441	0.937673	0.226813	528.5163	4.758043	546.883	17.48246	625.0789	86.84172	528.5	4.8	84.55194	73
19AR114_Spot 303	174.4188	26972.78	3.995682	16.79392	3.307493	0.695854	3.505282	0.085462	0.930659	0.265502	528.6445	4.723554	536.3113	14.60536	570.0194	73.57246	528.6	4.7	92.7415	280
19AR114_Spot 327	126.4441	13590.51	3.985769	16.22726	3.932626	0.717386	4.111247	0.085521	1.085972	0.264147	528.9908	5.515308	549.1226	17.43938	634.4989	85.37092	529.0	5.5	83.37144	301
19AR114_Spot 143	63.69731	11101.07	2.444293	14.99893	5.695003	0.772125	6.131524	0.085528	1.182902	0.192921	529.0367	6.008082	580.9812	27.13282	790.5343	126.3758	529.0	6.0	66.92141	133
19AR114_Spot 84	512.6746	13064.45	6.97987	16.80737	1.130947	0.688386	1.622954	0.085565	1.010925	0.622892	529.2552	5.136628	531.8305	6.718974	543.8618	27.76172	529.3	5.1	97.31428	79
19AR114_Spot 168	167.5322	17727.32	1.724626	16.39141	3.521035	0.707441	3.626987	0.085617	0.803629	0.221569	529.5638	4.085617	543.2258	15.25995	601.8913	76.5517	529.6	4.1	87.9833	156
19AR114_Spot 16	76.06803	11639.65	1.933992	16.42393	5.155062	0.704779	5.327064	0.085655	1.115975	0.209492	529.7874	5.675873	541.6416	22.36521	592.7725	112.9972	529.8	5.7	89.37449	15
19AR114_Spot 389	323.965	28702.53	1.685998	17.17569	2.66296	0.682563	2.82442	0.085773	0.781425	0.276667	530.4871	3.979378	528.3222	11.63455	519.9146	59.57579	530.5	4.0	102.0335	362
19AR114_Spot 377	102.3095	11174.9	2.321726	16.93343	3.356794	0.684514	3.997108	0.085804	0.967572	0.242068	530.6715	4.928968	529.4993	16.49384	525.3845	85.06199	530.7	4.9	101.0063	350
19AR114_Spot 206	206.8482	12941.31	3.740182	17.10493	3.008052	0.67941	3.134371	0.085805	0.776977	0.247889	530.6791	3.958101	526.4179	12.87594	508.9584	66.77995	530.7	4.0	104.2677	193
19AR114_Spot 161	777.0586	70048.84	11.15747	17.32481	1.027238	0.677958	1.285182	0.085818	0.759364	0.590861	530.7578	3.868929	525.5392	5.27254	503.8685	22.83914	530.8	3.9	105.3366	150
19AR114_Spot 270	120.2934	15056.5	1.957031	16.99957	4.486864	0.68278	4.7991	0.085942	1.000831	0.208546	531.4911	5.105952	528.453	19.77415	516.2866	103.1211	531.5	5.1	102.945	247
19AR114_Spot 347	191.1997	33268.88	3.118961	17.2266	2.951461	0.682692	3.119709	0.085962	0.830279	0.26614	531.6105	4.236756	528.4004	12.8525	515.4924	66.0762	531.6	4.2	103.1267	321
19AR114_Spot 290	263.7951	11463.95	1.959201	16.74417	3.254573	0.696065	3.399335	0.085971	0.737512	0.216958	531.6665	3.763764	536.438	14.1664	557.7049	72.39654	531.7	3.8	95.33116	267
19AR114_Spot 317	123.3286	17624.14	1.473147	16.41243	3.467901	0.711944	3.727563	0.085984	0.888427	0.23834	531.7409	4.534542	545.8998	15.74149	606.3714	78.31734	531.7	4.5	87.69228	293
19AR114_Spot 164	133.2255	10872.17	2.444143	16.36011	3.990336	0.706907	4.111054	0.086038	0.892867	0.217187	532.0608	4.559832	542.908	17.2893	589.6787	87.07446	532.1	4.6	90.22892	153
19AR114_Spot 390	239.7601	26804.02	2.974359	17.66446	2.391647	0.666865	2.561166	0.086263	0.815634	0.318462	533.3982	4.175451	518.8048	10.40448	456.0096	53.89802	533.4	4.2	116.9708	363
19AR114_Spot 307	144.0782	6359.179	2.036959	15.77337	5.269793	0.726594	5.64987	0.086347	0.72119	0.127647	533.8936	3.695255	554.5522	24.14637	641.2604	120.578	533.9	3.7	83.25691	283
19AR114_Spot 67	152.8327	18497.07	2.652869	16.83173	3.911578	0.69774	4.032512	0.086408	0.872307	0.216319	534.2608	4.472502	537.4402	16.82937	551.8839	85.96343	534.3	4.5	96.80674	63
19AR114_Spot 170	104.998	17343.13	1.588405	16.04978	4.871574	0.730172	5.003603	0.086463	0.93713	0.187291	534.5835	4.807645	556.6543	21.44437	648.8851	105.599	534.6	4.8	82.38492	158
19AR114_Spot 279	195.1881	22260.21	2.756892	16.60219	2.805896	0.711382	3.007355	0.086487	0.919544	0.305765	534.7248	4.718623	545.5667	12.69385	592.0528	62.06688	534.7	4.7	90.31709	256
19AR114_Spot 348	134.0749	9288.666	1.702999	17.36065	3.014354	0.668171	3.453936	0.086503	1.011164	0.292757	534.8229	5.189682	519.5996	14.04814	454.2061	73.34224	534.8	5.2	117.7489	322
19AR114_Spot 142	104.3921	9071.374	2.190864	15.58051	6.18611	0.749355	6.280923	0.086698	0.856294	0.136332	535.9804	4.403949	567.8501	27.32549	698.5454	132.651	536.0	4.4	76.72807	132
19AR114_Spot 436	183.0546	27385.93	9.067588	17.15886	2.421528	0.692705	2.577085	0.086714	0.812259	0.315185	536.0745	4.178179	534.4246	10.70883	528.3628	53.60859	536.1	4.2	101.4595	409
19AR114_Spot 334	242.902	26279.02	2.920766	16.9139	3.575823	0.702208	3.673908	0.086864	0.6661	0.181306	536.9657	3.431815	540.1089	15.39021	554.3371	78.87079	537.0	3.4	96.86628	308
19AR114_Spot 437	1589.433	121029.4	15.60609	17.23352	0.628212	0.697471	0.931893	0.087073	0.681606	0.731421	538.2014	3.519455	537.2792	3.887962	534.3018	13.93363	538.2	3.5	100.7298	410
19AR114_Spot 323	131.328	22364.6	4.604442	16.62471	3.555697	0.72587	3.780178	0.088295	0.822985	0.217711	545.4435	4.304254	554.126	16.14468	590.8969	80.0382	545.4	4.3	92.30772	298
19AR114_Spot 236	342.602	34427.5	5.498679	16.65369	1.572085	0.735649	1.790234	0.089066	0.811197	0.453124	550.0126	4.276651	559.8634	7.704718	601.0322	34.54894	550.0	4.3	91.51133	217
19AR114_Spot 329	212.7845	2628	3.198807	15.23119	2.264731	0.74911	3.293941	0.090327	1.099324	0.333741	557.4698	5.870895	567.7079	14.32528	609.8542	67.12661	557.5	5.9	91.41035	303
19AR114_Spot 314	49.87957	6823.214	0.462357	13.98653	7.093771	0.867805	7.591205	0.090775	1.37403	0.181003	560.1187	7.371326	634.3747	35.82708	909.6113	153.9657	560.1	7.4	61.57781	290
19AR114_Spot 34	280.8014	38362.72	2.636784	16.63411	1.909975	0.754387	2.144565	0.091519	0.91964	0.428824	564.5169	4.970704	570.7666	9.363758	596.662	41.99378	564.5	5.0	94.61252	33
19AR114_Spot 293	157.9963	18591	3.415162	17.38204	2.777705	0.725255	2.961709	0.092273	0.663519	0.224033	568.9633	3.613377	553.7646	12.64248	492.7087	63.66178	569.0	3.6	115.4766	270
19AR114_Spot 385	69.51213	10817.02	5.175573	15.72825	4.680726	0.793439	4.952246	0.092451	1.33048	0.268662	570.015	7.258309	593.1209	22.24993	683.4735	101.9102	570.0	7.3	83.39971	358
19AR114_Spot 221	287.7823	17564.5	1.897138	16.53992	2.371093	0.774138	2.636277	0.093472	0.659592	0.250199	576.0402	3.634705	582.1339	11.68076	606.901	55.18555	576.0	3.6	94.91502	206
19AR114_Spot 251	415.9122	39248.95	7.502479	16.55355	1.104541	0.778575	1.350863	0.094271	0.76823	0.568696	580.7476	4.266417	584.67	6.004466	600.8542	24.03432	580.7	4.3	96.65368	230
19AR114_Spot 435	578.2115	14262.67	1.069589	16.49815	1.027147	0.784647	1.271708	0.095177	0.694458	0.546083	586.0815	3.890553	588.1308	5.677324	597.0119	23.05962	586.1	3.9	98.16915	408
19AR114_Spot 52	191.4852	28305.97	1.187922	16.43139	2.252508	0.802116	2.451811	0.097233	0.738887	0.301364	598.1716	4.220951	598.0215	11.08124	598.3911	50.62897	598.2	4.2	99.96333	51
19AR114_Spot 39	205.5203	26660.54	215.5968	16.40288	2.847171	0.810511	3.025543	0.097421	0.776395	0.256614	599.2799	4.443062	602.7405	13.75363	616.697	63.13448	599.3	4.4	97.17575	38
19AR114_Spot 266	265.2588	33944.13	0.728681	16.89072	2.218755	0.786483	2.377145	0.097452	0.75883	0.319219	599.4574	4.343771	589.1748	10.62654	550.7299	49.17796	599.5	4.3	108.8478	243
19AR114_Spot 343	153.4696	20153.65	1.864421	16.73118	3.669994	0.794209	3.773488	0.097571	0.745893	0.197667	600.1593	4.274486	593.5566	16.96192	569.3666	80.54505	600.2	4.3	105.4083	317
19AR114_Spot 344	111.295	16106.04	1.166975	16.26983	3.906753	0.814528	4.029495	0.097641	0.789955	0.196043	600.5712	4.529953	604.9913	18.36836	622.5036	85.24709	600.6	4.5	96.47673	318
19AR114_Spot 208	189.8869	7373.294	5.171185	16.33276	3.28571	0.806509	3.634113	0.098294	0.693082	0.190715	604.4039	3.998632	600.494	16.47539	586.6822	77.44022	604.4	4.0	103.0207	195
19AR114_Spot 367	73.10252	10692.61	9.213175	15.41798	3.935747	0.862409	4.392843	0.098371	1.64634	0.374778	604.855	9.505081	631.4371	20.65731	728.7528	86.35339	604.9	9.5	82.99865	340
19AR114_Spot 376	1271.862	156953.2	1.046379	16.61074	0.709058	0.818462	0.957324	0.098575	0.641282	0.66987	606.0545	3.70942	607.1901	4.375077	612.351	15.33777	606.1	3.7	98.97175	349
19AR114_Spot 265	314.722	59464.2	9.766325	16.58013	1.638861	0.814577	1.903903	0.098761	0.940494	0.493982	607.1413	5.449475	605.0183	8.678448	598.016	35.87497	607.1	5.4	101.5259	242
19AR114_Spot 416	55.36339	5591.646	0.432874	14.97562	6.319263	0.876713	6.631819	0.098887	1.250626	0.18858	607.8832	7.254914	639.2058	31.46742	752.4411	137.6449	607.9	7.3	80.78814	389
19AR114_Spot 46	294.6187	42132.55	0.63028	16.40822	1.936213	0.824919	2.171609	0.099272	0.823648	0.37928	610.1407	4.794916	610.789	9.967659	614.1138	43.42526	610.1	4.8	99.35304	45
19AR114_Spot 295	46.60601	6061.236	2.502515	15.00917	6.650594	0.881792	7.433998	0.099302	1.388061	0.186718	610.3168	8.08291	641.95	35.38528	755.7952	154.3052	610.3	8.1	80.75162	272
19AR114_Spot 358	234.1323	9238.729	3.245788	16.16086	1.427174	0.828743	1.765338	0.099682	0.682618	0.386678	612.5465	3.988839	612.9146	8.123335	615.1951	35.14478	612.5	4.0	99.56948	332
19AR114_Spot 318	159.5897	17722.67	3.21393	16.19526	2.39378	0.840893	2.543689	0.099857	0.751845	0.295573	613.5702	4.400365	619.6386	11.79847	642.8229	52.25265	613.6	4.4	95.44934	294
19AR114_Spot 13	188.5684	26738.14	1.308389	16.51411	3.129826	0.839502	3.292134	0.101398	0.95245	0.289311	622.5947	5.65255	618.8706	15.25673	606.1931	68.16284	622.6	5.7	102.7057	12
19AR114_Spot 145	557.132	68588.94	2.646024	16.28556	1.025233	0.857667	1.320263	0.10164	0.789396	0.597908	624.0116	4.69502	628.8484	6.189373	647.2368	22.74166	624.0	4.7	96.41165	135
19AR114_Spot 2	158.584	19698.37	1.902851	16.43208	2.80121	0.848978	2.988186	0.10225	0.807374	0.270189	627.5825	4.828112	624.0881	13.93252	612.361	62.18277	627.6	4.8	102.4857	2
19AR114_Spot 138	392.6952	50547.78	2.663948	15.81999	1.212564	0.888828	1.441635	0.102327	0.685554	0.475539	628.0293	4.1024	645.7395	6.88838	709.0742	26.98747	628.0	4.1	88.57031	128
19AR114_Spot 3	179.9322	19139.69	3.066173	16.13669	2.271085	0.870687	2.674843	0.102737	0.806123	0.301372	630.4269	4.841428	635.9404	12.64188	656.534	54.73086	630.4	4.8	96.02349	3
19AR114_Spot 274	1105.137	144717.1	3.322736	16.33784	0.940742	0.867214	1.230304	0.103104	0.790193	0.642275	632.574	4.761138	634.0531	5.802018	640.2857	20.29201	632.6	4.8	98.79559	251
19AR114_Spot 299	592.4644	71268.93	4.270904	16.46249	0.9832	0.879251	1.211727	0.104984	0.706954	0.583427	643.5515	4.32989	640.5781	5.756616	631.0637	21.2075	643.6	4.3	101.9788	276
19AR114_Spot 410	51.9533	5557.556	3.490337	15.14016	6.71435	0.947297	6.935485	0.108099	1.042221	0.150274	661.6962	6.554207	676.6942	34.27091	727.8299	145.4914	661.7	6.6	90.91358	383
19AR114_Spot 38	392.4477	56734.27	4.948568	15.44588	1.248086	0.966489	1.471576	0.108855	0.776779	0.527856	666.0932	4.915737	686.6525	7.343878	755.5101	26.35754	666.1	4.9	88.1647	37
19AR114_Spot 37	269.0802	33449.37	4.177486	16.2223	1.818305	0.930817	1.976809	0.110208	0.755231	0.382045	673.9583	4.832906	668.0642	9.676782	649.185	39.25327	674.0	4.8	103.8161	36

19AR114_Spot 55	649.816	16737.31	78.07754	15.72699	0.803945	0.953128	1.135905	0.110966	0.784054	0.690246	678.3534	5.048381	679.7301	5.628555	685.2419	17.5645	678.4	5.0	98.99473	54
19AR114_Spot 165	186.9115	38365.31	2.248549	14.93741	2.879346	1.093509	3.018429	0.119537	0.779592	0.258277	727.8961	5.365971	750.2071	16.01011	818.2648	60.93506	727.9	5.4	88.95605	154
19AR114_Spot 72	297.7073	28021.94	2.27992	14.53677	1.628571	1.147568	2.352933	0.122005	1.610041	0.68427	742.0923	11.28594	776.0937	12.76714	876.0875	35.52275	742.1	11.3	84.70527	67
19AR114_Spot 178	81.68361	14980.23	15.29702	14.1972	5.243271	1.197838	5.476039	0.125413	1.267184	0.231405	761.6453	9.103101	799.5878	30.31293	907.7094	109.8139	761.6	9.1	83.9085	166
19AR114_Spot 187	120.724	18161.14	3.458101	14.54503	3.232332	1.194555	3.529233	0.127889	1.119024	0.317073	775.8125	8.179468	798.07	19.50847	861.6167	69.48642	775.8	8.2	90.04149	175
19AR114_Spot 356	330.4976	24754.53	3.253411	14.53254	1.158133	1.227549	1.602566	0.13053	0.856867	0.534684	790.8889	6.377644	813.2224	8.967444	875.7629	28.03652	790.9	6.4	90.30857	330
19AR114_Spot 242	203.1282	44168.94	2.854474	14.29654	2.107486	1.338977	2.466649	0.139235	1.248116	0.505997	840.335	9.833511	862.7848	14.33881	921.8184	43.73728	840.3	9.8	91.16059	222
19AR114_Spot 101	80.0316	17327.53	2.901568	14.51608	4.084013	1.449421	4.243402	0.153665	1.071005	0.252393	921.4752	9.196134	909.6327	25.50155	881.9146	84.96613	881.9	85.0	104.4858	94
19AR114_Spot 214	121.5169	3144.898	2.639926	14.10386	2.483777	1.3667	2.699444	0.148108	0.892174	0.330503	890.3478	7.419305	874.749	15.82958	836.3824	53.082	890.3	7.4	106.4522	199
19AR114_Spot 239	160.0731	5104.182	3.717687	13.7085	1.983776	1.392175	2.284429	0.143684	1.04739	0.458491	865.4612	8.48261	885.62	13.50001	937.213	41.63352	937.2	41.6	92.34413	220
19AR114_Spot 316	187.1178	24449.46	1.041562	14.0647	2.088013	1.399401	2.259026	0.144065	0.763742	0.338085	867.6068	6.199721	888.6825	13.37875	942.4105	43.56438	942.4	43.6	92.06251	292
19AR114_Spot 141	617.0812	108661.3	2.326173	14.02394	0.825056	1.567917	1.095998	0.159526	0.718274	0.655361	954.1422	6.370296	957.6028	6.794979	966.4632	16.9161	966.5	16.9	98.72514	131
19AR114_Spot 244	292.1753	40986.84	4.848767	13.9837	1.355528	1.498387	1.730022	0.152318	1.06668	0.61657	913.944	9.089323	929.7309	10.53565	968.2228	27.78783	968.2	27.8	94.39397	224
19AR114_Spot 427	486.438	26789.67	4.252063	13.83041	0.779263	1.492467	1.109426	0.150722	0.752428	0.678214	905.0076	6.353149	927.3219	6.745437	981.6674	16.58469	981.7	16.6	92.19086	400
19AR114_Spot 361	366.9263	43272.36	5.53604	13.85249	0.866854	1.491369	1.092655	0.150556	0.62866	0.575351	904.0785	5.30303	926.8744	6.6415	982.4089	18.17866	982.4	18.2	92.0267	335
19AR114_Spot 269	37.09609	8823.163	2.1544	13.53088	6.488656	1.750416	6.899031	0.176347	1.370626	0.198669	1046.986	13.24555	1027.316	44.61082	986.5638	137.7349	986.6	137.7	106.1245	246
19AR114_Spot 432	136.5752	47739.59	3.651052	13.84219	1.578287	1.73126	1.800684	0.17396	0.837345	0.465015	1033.892	7.99868	1020.22	11.59006	991.8746	32.41684	991.9	32.4	104.2361	405
19AR114_Spot 44	132.4673	30294.49	2.940087	13.68467	1.690573	1.681574	1.851546	0.168596	0.71284	0.384997	1004.371	6.629686	1001.578	11.7899	996.3536	34.7056	996.4	34.7	100.8046	43
19AR114_Spot 17	557.8297	70322.36	1.57139	13.79178	0.827791	1.625554	1.08188	0.162963	0.667294	0.616791	973.2197	6.027786	980.1411	6.801363	996.5563	17.30311	996.6	17.3	97.65827	16
19AR114_Spot 408	82.23715	18588.6	2.354263	13.67142	3.724204	1.621697	3.834029	0.162243	0.689038	0.179716	969.2275	6.200543	978.6482	24.0854	1000.712	76.62133	1000.7	76.6	96.85378	381
19AR114_Spot 152	147.4265	45305.2	2.694884	13.65575	1.95361	1.753831	2.108286	0.17487	0.723199	0.343027	1038.889	6.939082	1028.576	13.6344	1007.57	40.18835	1007.6	40.2	103.1084	142
19AR114_Spot 426	350.1525	72100.93	5.785586	13.65599	1.004794	1.659135	1.261266	0.164666	0.72752	0.576817	982.6561	6.630802	993.0456	7.990739	1016.93	20.85878	1016.9	20.9	96.62963	399
19AR114_Spot 18	297.3168	58008.95	3.827234	13.61625	1.138534	1.70643	1.314084	0.168872	0.609616	0.463909	1005.894	5.677608	1010.946	8.413074	1022.772	23.57928	1022.8	23.6	98.34979	17
19AR114_Spot 176	104.4597	19497.75	2.927522	13.44335	1.686763	1.771475	1.895048	0.175273	0.791481	0.417658	1041.098	7.609131	1035.061	12.29972	1023.192	34.84968	1023.2	34.8	101.75	164
19AR114_Spot 369	712.2536	171203.9	26.19041	13.63278	0.670645	1.735809	0.92607	0.171698	0.629316	0.679555	1021.462	5.944802	1021.909	5.966161	1023.73	13.74759	1023.7	13.7	99.7784	342
19AR114_Spot 263	150.7515	13228.15	2.209592	13.38507	2.270586	1.757709	2.439643	0.173848	0.716515	0.293697	1033.28	6.840729	1030.005	15.79028	1023.919	47.20242	1023.9	47.2	100.9142	241
19AR114_Spot 30	357.6283	62600.07	1.60484	13.58461	1.059567	1.723902	1.267006	0.17025	0.678452	0.535477	1013.486	6.362754	1017.48	8.142147	1026.945	21.66108	1026.9	21.7	98.68945	29
19AR114_Spot 128	227.9763	54661.49	2.238823	13.54962	1.269643	1.7702	1.557394	0.174714	0.865549	0.555768	1038.032	8.298621	1034.594	10.10541	1028.195	26.20072	1028.2	26.2	100.9568	120
19AR114_Spot 201	409.983	73567.31	3.492921	13.57984	0.776312	1.766187	0.97431	0.174296	0.560384	0.57516	1035.74	5.361847	1033.122	6.316666	1028.446	16.11577	1028.4	16.1	100.7092	188
19AR114_Spot 174	175.3128	38999.91	2.39303	13.4703	1.524532	1.706627	1.743237	0.168363	0.780959	0.447994	1003.086	7.25464	1011.02	11.16128	1029.113	31.53146	1029.1	31.5	97.47085	162
19AR114_Spot 351	418.3792	37195.23	1.993461	13.51554	0.909294	1.731066	1.313833	0.170608	0.929458	0.70744	1015.462	8.732463	1020.147	8.455932	1031.075	18.78825	1031.1	18.8	98.4858	325
19AR114_Spot 271	849.6874	50140.59	2.407966	13.46053	0.691957	1.645015	1.274823	0.161883	1.065173	0.835545	967.2305	9.567015	987.6397	8.050645	1034.13	14.16901	1034.1	14.2	93.53086	248
19AR114_Spot 254	322.7921	60029.75	1.433433	13.47282	1.148317	1.765991	1.38152	0.173703	0.762586	0.551991	1032.48	7.275383	1033.05	8.95645	1035.115	23.29047	1035.1	23.3	99.74548	232
19AR114_Spot 106	118.5465	22014.65	2.347169	13.44961	2.342306	1.833503	2.578462	0.180341	0.809304	0.313871	1068.838	7.971085	1057.535	16.94294	1035.139	49.48004	1035.1	49.5	103.2555	99
19AR114_Spot 400	699.4242	47244.71	3.287075	13.4994	0.832287	1.658724	1.144553	0.163061	0.783959	0.684948	973.7653	7.08532	992.8884	7.250605	1036.241	16.86228	1036.2	16.9	93.97094	373
19AR114_Spot 193	773.0194	32322.11	3.311127	13.40764	0.705747	1.747593	1.027397	0.171588	0.744081	0.724239	1020.856	7.025072	1026.273	6.635325	1038.703	14.32204	1038.7	14.3	98.28183	180
19AR114_Spot 5	287.2122	65074.32	1.206519	13.53924	0.945857	1.696039	1.188867	0.166467	0.695355	0.584889	992.6168	6.397072	1007.04	7.594184	1039.42	19.48522	1039.4	19.5	95.49721	5
19AR114_Spot 118	181.9579	48392.45	2.356904	13.48935	1.865028	1.624084	1.992913	0.159315	0.627946	0.315089	952.9708	5.562839	979.5724	12.52481	1040.552	38.18716	1040.6	38.2	91.58324	111
19AR114_Spot 429	58.12717	3777.031	2.432103	12.88987	5.871688	1.733032	6.502889	0.169724	0.987283	0.151822	1010.592	9.234655	1020.878	41.89329	1043.856	129.7942	1043.9	129.8	96.81337	402
19AR114_Spot 64	315.6123	73947.15	1.389423	13.44305	0.861627	1.780026	1.14631	0.174185	0.729695	0.63656	1035.128	6.978039	1038.189	7.452761	1045.539	17.84976	1045.5	17.8	99.00425	61
19AR114_Spot 276	272.8053	65886.89	10.55251	13.42084	0.995399	1.742206	1.298468	0.170366	0.798271	0.614779	1014.129	7.490845	1024.281	8.376652	1046.929	20.6708	1046.9	20.7	96.86707	253
19AR114_Spot 31	263.698	55905.45	2.127899	13.39514	0.865243	1.821029	1.243656	0.17739	0.871601	0.700838	1052.699	8.465341	1053.056	8.151714	1054.692	17.88685	1054.7	17.9	99.81102	30
19AR114_Spot 284	201.058	44199.19	2.228039	13.42819	1.18444	1.839883	1.33166	0.179196	0.601402	0.451619	1062.581	5.891501	1059.819	8.760395	1055.032	23.9345	1055.0	23.9	100.7156	261
19AR114_Spot 425	1470.737	56314.64	2.465862	13.38935	0.592608	1.815164	0.896383	0.176755	0.670099	0.747559	1049.223	6.488487	1050.942	5.868681	1055.412	12.00896	1055.4	12.0	99.41356	398
19AR114_Spot 175	149.0354	32041.13	2.985988	13.27664	2.429845	1.806024	2.584143	0.175779	0.799401	0.309348	1043.874	7.704145	1047.64	16.88957	1056.397	49.49824	1056.4	49.5	98.8146	163
19AR114_Spot 96	200.2193	15209.6	2.87736	13.26563	1.280498	1.711354	1.623711	0.166035	0.924826	0.569576	990.2305	8.489215	1012.792	10.40656	1062.804	26.86639	1062.8	26.9	93.17152	89
19AR114_Spot 407	508.0248	72758.66	2.551895	13.34927	0.602143	1.856421	0.846286	0.180018	0.541029	0.639299	1067.073	5.32067	1065.715	5.584773	1063.831	13.08942	1063.8	13.1	100.3047	380
19AR114_Spot 444	475.8967	100381	1.877278	13.42545	0.735138	1.819949	0.946322	0.176446	0.584052	0.617181	1047.531	5.646897	1052.667	6.201436	1064.231	14.97453	1064.2	15.0	98.43078	416
19AR114_Spot 372	34.10653	6541.857	3.236693	12.98123	5.131717	1.802743	5.238653	0.174523	0.898068	0.171431	1036.982	8.602365	1046.452	34.22666	1067.171	103.8206	1067.2	103.8	97.17113	345
19AR114_Spot 434	199.4497	24681.8	2.277276	13.26602	1.239296	1.829172	1.535669	0.176769	0.709034	0.46171	1049.3	6.865945	1055.982	10.08178	1070.718	27.3717	1070.7	27.4	97.99967	407
19AR114_Spot 228	220.2795	43107.52	2.428815	13.37251	0.90647	1.814672	1.118315	0.175226	0.591032	0.528502	1040.843	5.680767	1050.765	7.321025	1072.339	19.05243	1072.3	19.1	97.06286	211
19AR114_Spot 12	150.6457	49840.87	2.444198	13.28617	1.829384	1.937592	2.04194	0.187093	0.891707	0.436696	1105.608	9.059691	1094.167	13.67635	1072.366	36.90355	1072.4	36.9	103.0999	11
19AR114_Spot 156	449.1678	103002.4	10.84448	13.25892	0.745216	1.827021	1.149364	0.176046	0.862251	0.750199	1045.34	8.320607	1055.21	7.542405	1076.582	15.23715	1076.6	15.2	97.09805	146
19AR114_Spot 188	181.7937	53820.83	1.814523	13.20299	1.224516	1.823113	1.479043	0.175624	0.794031	0.536854	1043.026	7.646661	1053.805	9.698604	1077.102	25.04813	1077.1	25.0	96.83627	176
19AR114_Spot 280	342.7769	94171.55	1.451178	13.26245	1.050694	1.898869	1.310123	0.182789	0.781007	0.596133	1082.192	7.780652	1080.693	8.71403	1078.525	21.11013	1078.5	21.1	100.34	257
19AR114_Spot 177	344.6982	59369.29	4.220402	13.16396	0.781094	1.876128	1.058876	0.180555	0.673044	0.635621	1070.006	6.635672	1072.696	7.01352	1079.062	16.40472	1079.1	16.4	99.16075	165
19AR114_Spot 336	281.0234	67756.15	1.526285	13.14529	1.076269	1.960333	1.299337	0.187221	0.725096	0.558051	1106.303	7.371174	1101.997	8.736786	1094.397	21.58748	1094.4	21.6	101.0879	310
19AR114_Spot 371	1400.182	261425.3	3.295371	13.12272	0.613497	1.912531	0.880028	0.182024	0.629207	0.714985	1078.022	6.246177	1085.467	5.867719	1101.29	12.3062	1101.3	12.3	97.88727	344
19AR114_Spot 443	72.16204	13730.68	2.746489	12.84909	4.688892	2.024632	4.789933	0.190196	0.656598	0.137079	1122.436	6.763957	1123.815	32.5673	1127.331	94.58589	1127.3	94.6	99.56579	415
19AR114_Spot 319	236.484	52977.9	2.760402	12.95596	1.133287	1.984565	1.422991	0.186271	0.807961	0.56779	1101.142	8.178425	1110.275	9.60792	1129.077	23.32535	1129.1	23.3	97.52583	295
19AR114_Spot 311	184.3543	46388.15	2.178225	12.84889	1.089741	1.894336	1.292893	0.177498	0.646213	0.499819	1053.29	6.279524	1079.104	8.592331	1132.499	22.29411	1132.5	22.3	93.00582	287
19AR114_Spot 365	88.54557	25642.3	0.986446	12.77986	1.7584	2.069259	1.928785	0.192938	0.737306	0.382264	1137.272	7.687167	1138.687	13.20442	1142.258	35.44677	1142.3	35.4	99.56352	339
19AR114_Spot 98	44.90568	13412.41	4.466057	12.69468	6.741319	1.848281	6.917754	0.171777	1.040171	0.150363	1021.892	9.829753	1062.817	45.61127	1148.672	135.99	1148.7	136.0	88.96294	91
19AR114_Spot 341	89.66919	26943.68	1.467536	12.72388	2.180329	2.216862	2.354157	0.206002	0.785732	0.333764	1207.48	8.651982	1186.38	16.4744	1149.002	44.06852	1149.0	44.1	105.0894	315
19AR114_Spot 155	87.04955	20397.31	1.646946	12.49283	1.989775	2.113584	2.171549	0.193212	0.788861	0.363271	1138.752	8.234472	1153.246	14.9689	1181.47	39.97787	1181.5	40.0	96.38435	145
19AR114_Spot 238	386.7207	51474.89	6.357412	12.47538	0.842459	2.216446	1.201384	0.200602	0.831692	0.692278	1178.556	8.95814	1186.249	8.406254	1201.16	17.07775	1201.2	17.1	98.11808	219
19AR114_Spot 49	147.0638	26045.45	3.400533	12.15434	1.522452	2.371387	1.778048	0.212243	0.724917	0.407704	1240.757	8.181827	1234.02	12.69959	1223.107	31.8832	1223.1	31.9	101.443	48
19AR114_Spot 268	45.63017	8357.92	2.255609	11.59385	11.77953	0.93999	11.88767	0.080841	1.511176	0.127121	501.1377	7.286195	672.8771	58.55062	1301.054	229.8893	1301.1	229.9	38.51781	245
19AR114_Spot 288	342.939	71175.55	3.146222	11.87152	0.899118	2.429791	1.546396	0.208961	1.253542	0.810622	1223.278	13.96722	1251.459	11.12421	1301.099	17.59626	1301.1	17.6	94.0188	265
19AR114_Spot 342	151.1563	31645.04	0.781778	11.78668	1.207523	2.588471	1.449408	0.222491	0.774314	0.534228	1295.024	9.084523	1297.382	10.61623	1302.112	23.80414	1302.1	23.8	99.45565	316
19AR114_Spot 260	349.3698	66853.47	1.980516	10.89869	0.732872	3.088131	0.988896	0.245676	0.662954	0.670398	1416.137	8.428672	1429.749	7.585066	1450.879	13.96565	1450.9	14.0	97.60546	238
19AR114_Spot 45	31.37745	3568.528	2.029328	9.889293	16.10238	1.101006	16.20814	0.082567	1.772455	0.109356	511.4296	8.714604	753.8369	86.45159	1562.438	304.1023	1562.4	304.1	32.7328	44
19AR114_Spot 298	25.75774	185.2242	19.27409	5.49283	9.307224	1.075547	11.10227	0.071682	3.15165	0.283874	446.2793	13.58937	741.4577	58.48146	1780.578	194.7108	1780.6	194.7	25.06373	275
19AR114_Spot 172	437.257	38078.2	1.222054	9.08653	0.71638	3.861691	1.242146	0.256256	1.013756	0.816133	1470.656	13.33053	1605.713	10.01859	1788.483	13.07715	1788.5	13.1	82.22922	160
19AR114_Spot 285	301.1166	95800.83	5.358871	9.008604	0.63954	4.543179	0.957748	0.295946	0.706376	0.737539	1671.175	10.39873	1738.913	7.970596	1822.153	11.73444	1822.2	11.7	91.71431	262
19AR114_Spot 104	272.2982	82636.52	1.651308	8.896127	0.696285	4.655512	1.039855	0.300687	0.762571	0.733344	1694.716	11.36426	1759.284	8.691784	1837.62	12.80423	1837.6	12.8	92.22345	97
19AR114_Spot 315	15.11296	1766.941	0.676339	7.639522	34.39354	1.308597	34.69238	0.077017	3.985458	0.11488	478.294	18.37226	849.5099	202.3094	2004.268	632.42	2004.3	632.4	23.86377	291
19AR114_Spot 154	199.282	99593.22	3.311942	7.994732	0.592007	6.137289	1.143403	0.356307	0.974299	0.852105	1964.646	16.49977	1995.566	9.983581	2028.496	10.59455	2028.5	10.6	96.85236	144
19AR114_Spot 395	419.6644	185896.8	2.225966	7.993302	0.73986	6.395664	1.058465	0.371017	0.756675	0.714879	2034.184	13.20015	2031.674	9.29452	2029.881	13.10185	2029.9	13.1	100.212	368
19AR114_Spot 134	157.7484	34391.09	1.359919	6.621483	0.716416	7.213012	0.972418	0.346728	0.646626	0.664967	1918.954	10.732	2138.112	8.671771	2356.632	12.40707	2356.6	12.4	81.42783	125
19AR114_Spot 388	310.2222	153997	0.941542	5.707375	0.644013	11.46963	0.985063	0.474669	0.745299	0.7566	2504.002	15.46484	2562.112	9.20029	2609.132	10.72878	2609.1	10.7	95.97071	361
19AR114_Spot 422	595.7534	173486.3	1.335875	5.62341	0.587413	11.60134	0.890444	0.472796	0.669167	0.751498	2495.811	13.84793	2572.78	8.324109	2634.702	9.757675	2634.7	9.8	94.72838	395
**19AR115**																				
19AR115_Spot 303	764.755	2560.592	0.766546	9.427867	12.18652	0.026181	12.88219	0.001885	3.719688	0.288747	12.14118	0.451189	26.24185	3.337226	1638.293	229.8886	12.1	0.5	0.741087	277
19AR115_Spot 478	782.4647	2311.534	1.058597	9.003886	20.64488	0.027829	21.18103	0.001919	3.738299	0.176493	12.35905	0.461576	27.87122	5.823189	1718.172	387.5517	12.4	0.5	0.719314	435
19AR115_Spot 252	167.6635	1000.202	2.423575	4.924821	31.62678	0.135259	32.36015	0.005156	5.780806	0.17864	33.15235	1.911554	128.8125	39.16767	2745.13	539.2244	33.2	1.9	1.207679	233
19AR115_Spot 85	75.60089	5671.398	1.242891	13.20635	8.423394	0.491095	9.204256	0.048607	2.03396	0.22098	305.9639	6.077809	405.6564	30.79016	1022.472	182.0105	306.0	6.1	29.92393	77
19AR115_Spot 224	239.7196	3884.394	3.674167	15.64722	3.156855	0.559592	3.27558	0.067577	0.625431	0.190938	421.5421	2.552103	451.2606	11.93434	606.5822	69.54708	421.5	2.6	69.49464	205
19AR115_Spot 247	321.4145	6505.615	2.670379	16.59834	1.697682	0.542297	2.051121	0.067996	1.0963	0.534488	424.0696	4.499457	439.9382	7.323153	524.7711	37.99513	424.1	4.5	80.8104	228
19AR115_Spot 74	142.8061	22943.57	1.004928	17.01068	3.979849	0.593931	4.143587	0.074121	0.926542	0.223609	460.934	4.121638	473.3749	15.6786	535.0821	88.43644	460.9	4.1	86.14268	67
19AR115_Spot 121	611.4944	17329.16	13.1042	17.45892	1.60471	0.581815	1.782675	0.074459	0.761821	0.427347	462.9623	3.403276	465.6269	6.6579	479.7285	35.61709	463.0	3.4	96.50507	110
19AR115_Spot 157	119.5063	5094.683	2.958114	15.74737	3.593747	0.637603	3.954968	0.076537	1.012746	0.256069	475.4206	4.641543	500.821	15.63684	619.5456	82.54464	475.4	4.6	76.73698	144
19AR115_Spot 166	155.2998	11585.61	1.103104	16.73522	4.052956	0.619603	4.476659	0.077058	0.627142	0.140092	478.5404	2.892443	489.5988	17.39128	542.6117	96.96411	478.5	2.9	88.19205	152
19AR115_Spot 189	328.5739	23099.47	1.943761	17.26352	2.39253	0.61015	2.48261	0.077328	0.604473	0.243483	480.1519	2.796931	483.6545	9.552594	501.2675	53.03163	480.2	2.8	95.78755	173
19AR115_Spot 122	62.94048	6452.514	1.026082	14.8743	7.485278	0.697416	7.715789	0.077497	1.652138	0.214124	481.1648	7.660079	537.2464	32.20026	783.9108	158.5342	481.2	7.7	61.38005	111
19AR115_Spot 245	108.6007	13844.94	1.053923	15.24584	4.749276	0.688702	4.900197	0.077608	0.929953	0.189779	481.8282	4.317415	532.0201	20.29455	754.4222	101.6036	481.8	4.3	63.86718	226
19AR115_Spot 253	874.3761	51938.64	6.235537	16.84177	0.98667	0.630725	1.236249	0.077842	0.734915	0.594472	483.2265	3.421467	496.5477	4.855105	559.3779	21.68782	483.2	3.4	86.3864	234
19AR115_Spot 442	57.06522	3794.955	2.597752	12.2961	10.7791	0.840413	11.54141	0.0782	1.182962	0.102497	485.3722	5.530934	619.3734	53.5635	1146.291	228.8226	485.4	5.5	42.34283	404
19AR115_Spot 40	130.3833	10360.25	1.701813	17.07638	4.599127	0.6195	4.837416	0.078378	1.213307	0.250817	486.4314	5.684733	489.5339	18.79115	505.0165	103.082	486.4	5.7	96.31989	39
19AR115_Spot 486	215.8107	1360.942	3.229332	14.0387	2.667737	0.657626	3.702131	0.078825	0.839231	0.226689	489.1044	3.952864	513.161	14.91439	622.7188	77.80481	489.1	4.0	78.54339	442
19AR115_Spot 383	434.1386	3281.811	1.156417	15.81799	1.965194	0.643754	2.236214	0.07942	0.934801	0.418028	492.6588	4.433797	504.6279	8.892781	560.2052	44.28255	492.7	4.4	87.94256	351
19AR115_Spot 176	369.2197	28669.27	24.65344	18.13067	1.865308	0.601531	2.124711	0.080196	0.945884	0.445182	497.2924	4.526954	478.2048	8.103294	388.629	42.71696	497.3	4.5	127.9607	160
19AR115_Spot 326	393.9312	12548.15	4.063186	16.89713	1.925104	0.64565	2.075049	0.080796	0.657746	0.316979	500.8718	3.169729	505.7982	8.266609	529.1135	43.12057	500.9	3.2	94.66244	296
19AR115_Spot 398	136.0688	9572.769	1.637028	16.71226	3.330538	0.656505	3.601638	0.081434	0.73742	0.204746	504.6771	3.579639	512.4738	14.49455	548.3748	77.01654	504.7	3.6	92.03141	366
19AR115_Spot 241	470.8865	6112.63	5.565395	16.53639	1.356251	0.651685	2.065773	0.081563	1.421755	0.688243	505.4469	6.911712	509.515	8.27623	528.7877	32.85178	505.4	6.9	95.58597	222
19AR115_Spot 436	331.8406	3260.226	1.25309	16.15861	1.585974	0.653389	1.99425	0.082036	0.788987	0.395631	508.2618	3.856107	510.5623	8.002309	521.8109	40.1633	508.3	3.9	97.40345	399
19AR115_Spot 115	231.8625	8127.986	2.077828	17.43236	1.944965	0.630892	2.510964	0.082066	0.789497	0.31442	508.4415	3.859912	496.6517	9.863113	443.6142	53.01472	508.4	3.9	114.6134	104
19AR115_Spot 330	297.9787	15305.09	1.384177	17.17665	2.770128	0.648076	2.873633	0.082169	0.623088	0.216829	509.0589	3.049878	507.2941	11.47436	500.3258	61.78247	509.1	3.0	101.7455	300
19AR115_Spot 395	412.8435	52901.83	2.351903	17.16927	1.844126	0.662169	1.952293	0.082634	0.600124	0.307394	511.8267	2.952822	515.94	7.897279	535.171	40.65869	511.8	3.0	95.63798	363
19AR115_Spot 269	168.9835	19426.53	1.44412	16.19067	4.512868	0.695466	4.628497	0.082737	0.664561	0.14358	512.4412	3.273646	536.0795	19.28009	638.9197	98.57183	512.4	3.3	80.20433	247
19AR115_Spot 97	346.5499	19796.36	5.595925	17.20982	1.854257	0.657013	2.06188	0.082768	0.798906	0.387465	512.6211	3.936763	512.7854	8.301393	514.4521	41.77554	512.6	3.9	99.64409	88
19AR115_Spot 211	201.0993	21543.94	2.802759	17.33421	1.19497	0.651976	1.419672	0.082905	0.679563	0.478676	513.4412	3.353822	509.6938	5.689186	493.8774	27.46937	513.4	3.4	103.9613	195
19AR115_Spot 362	306.4662	38596.73	4.720782	16.8153	2.251117	0.67589	2.36459	0.083038	0.680427	0.287757	514.2311	3.363051	524.2871	9.683433	569.2948	49.28985	514.2	3.4	90.32775	330
19AR115_Spot 102	296.7936	33277.7	4.017803	17.07916	2.228393	0.667625	2.375077	0.08308	0.672185	0.283016	514.4784	3.323846	519.2673	9.655058	541.3213	49.79582	514.5	3.3	95.04123	91
19AR115_Spot 337	219.6184	21029.13	2.222122	17.09245	3.382015	0.661946	3.642076	0.083163	0.712423	0.195609	514.9759	3.526092	515.8033	14.73041	520.4052	78.41176	515.0	3.5	98.95671	306
19AR115_Spot 236	542.298	33897.23	4.296927	17.05893	1.304887	0.665022	1.53162	0.083191	0.574572	0.37514	515.1425	2.844687	517.681	6.211583	529.8742	31.12821	515.1	2.8	97.21977	217
19AR115_Spot 145	268.6711	17466.2	2.00062	17.36177	2.600368	0.651591	2.874992	0.083248	0.733195	0.255025	515.4777	3.632296	509.4575	11.51749	483.5105	61.40226	515.5	3.6	106.6115	132
19AR115_Spot 349	141.407	16168.42	2.350466	15.65446	4.76728	0.723162	4.85489	0.083254	0.867849	0.178758	515.5148	4.299681	552.5317	20.69087	709.0859	101.6411	515.5	4.3	72.70132	318
19AR115_Spot 308	810.1508	6762.924	4.267274	16.55288	1.072282	0.669808	1.204512	0.083278	0.4992	0.414442	515.662	2.473919	520.5958	4.906006	543.2753	23.98278	515.7	2.5	94.91726	280
19AR115_Spot 317	187.4145	32955.64	2.617359	16.61763	2.037876	0.687274	2.281283	0.083346	0.826713	0.36239	516.0625	4.100056	531.1613	9.435523	597.4968	46.06282	516.1	4.1	86.37075	289
19AR115_Spot 316	248.7018	22660.48	0.89537	17.18326	2.630726	0.662529	2.790057	0.083391	0.737848	0.264456	516.3341	3.661181	516.1599	11.29007	516.3229	59.09666	516.3	3.7	100.0022	288
19AR115_Spot 313	161.7323	19946.8	1.300854	16.26184	2.720471	0.699809	3.04755	0.083426	0.872197	0.286196	516.5414	4.329486	538.6766	12.74039	634.4713	62.86597	516.5	4.3	81.41288	285
19AR115_Spot 227	264.9833	45547.68	4.876164	16.94147	2.865174	0.674013	3.017016	0.083435	0.779202	0.258269	516.5934	3.868244	523.1492	12.33497	552.8397	63.62142	516.6	3.9	93.44363	208
19AR115_Spot 242	380.9469	14495.45	3.635531	17.16076	1.946237	0.657378	2.173446	0.083465	0.887198	0.408199	516.7712	4.405829	513.0088	8.753526	497.2262	43.70358	516.8	4.4	103.9308	223
19AR115_Spot 125	2078.89	39151.29	11.03486	17.10791	0.720016	0.671897	1.009081	0.083507	0.703475	0.697145	517.0244	3.495107	521.8654	4.11767	544.0477	15.80697	517.0	3.5	95.03292	114
19AR115_Spot 94	133.5158	17894.03	1.479957	14.87774	7.089546	0.767821	7.139701	0.083526	0.780428	0.109308	517.1374	3.878247	578.5119	31.49712	828.4147	148.1823	517.1	3.9	62.42494	85
19AR115_Spot 342	101.3321	18331.11	1.424933	16.57417	4.152091	0.685262	4.442244	0.083544	0.98233	0.221134	517.2432	4.882535	529.9497	18.34293	586.0146	94.05487	517.2	4.9	88.26456	311
19AR115_Spot 129	82.96769	14392.66	2.924641	16.48775	5.459571	0.690628	5.696332	0.083586	0.991355	0.174034	517.4946	4.929696	533.1776	23.63195	601.7816	121.4773	517.5	4.9	85.99376	118
19AR115_Spot 371	489.3488	33008.61	1.141124	17.26673	1.32476	0.661461	1.589026	0.083597	0.698194	0.439385	517.5543	3.472281	515.5075	6.423637	507.3804	31.3877	517.6	3.5	102.0052	339
19AR115_Spot 204	708.246	47072.74	0.940123	17.22272	1.094854	0.665988	1.310379	0.083606	0.604098	0.46101	517.6093	3.004628	518.27	5.318941	522.1183	25.49882	517.6	3.0	99.1364	188
19AR115_Spot 318	236.6405	28425.92	1.087995	16.8881	3.132107	0.677116	3.317371	0.083625	0.776486	0.234067	517.7214	3.862843	525.0301	13.60035	557.8921	70.32095	517.7	3.9	92.79956	290
19AR115_Spot 201	496.349	70176.87	4.267072	17.14308	1.353156	0.671028	1.474145	0.083666	0.578416	0.392374	517.9684	2.878806	521.3373	6.010798	537.0544	29.69258	518.0	2.9	96.44618	185
19AR115_Spot 341	135.7489	16463.46	1.257524	16.17307	4.103849	0.703152	4.301197	0.083745	0.867424	0.20167	518.4361	4.320962	540.6717	18.03269	636.5261	90.71326	518.4	4.3	81.44774	310
19AR115_Spot 24	151.695	20851.89	2.581453	17.27044	3.755578	0.662594	4.021564	0.083778	0.813009	0.202162	518.6312	4.051364	516.1994	16.27509	506.4181	86.68476	518.6	4.1	102.4117	24
19AR115_Spot 29	599.2944	52477.9	1.060866	17.26903	0.855442	0.668764	1.103719	0.083795	0.62513	0.566386	518.7352	3.115731	519.9608	4.491271	526.3243	19.92724	518.7	3.1	98.5581	28
19AR115_Spot 365	225.8065	19364.8	3.440879	16.82802	1.896438	0.676908	2.079194	0.083798	0.7814	0.375819	518.7518	3.894718	524.904	8.522275	552.7078	42.06768	518.8	3.9	93.85642	333
19AR115_Spot 452	338.6064	38722.5	4.472248	16.72678	1.918208	0.687064	2.100846	0.083843	0.689181	0.328049	519.022	3.43679	531.0348	8.687609	583.9182	43.06637	519.0	3.4	88.88607	413
19AR115_Spot 431	659.4951	50521.95	1.047965	17.45988	0.878952	0.662725	1.126118	0.083888	0.676758	0.600966	519.2885	3.376507	516.2792	4.55753	503.9181	19.78715	519.3	3.4	103.0502	395
19AR115_Spot 19	317.3295	29071.98	0.879834	17.46207	2.30443	0.657602	2.477603	0.083909	0.769066	0.310407	519.4123	3.83793	513.146	9.98064	486.2817	51.99492	519.4	3.8	106.8131	19
19AR115_Spot 445	316.2761	50634.27	5.402081	17.71037	1.737431	0.652786	1.913534	0.083923	0.730105	0.381548	519.4959	3.644062	510.1917	7.674118	469.6701	39.15815	519.5	3.6	110.6087	406
19AR115_Spot 412	128.9117	19105.16	1.728989	16.98035	3.958903	0.674004	4.134761	0.08393	0.780284	0.188713	519.5359	3.894803	523.144	16.90544	539.8982	88.8423	519.5	3.9	96.2285	380
19AR115_Spot 467	121.2571	31631.29	1.577498	16.01089	4.636741	0.718045	4.753391	0.083945	1.003362	0.211083	519.6249	5.009121	549.5119	20.17472	676.3372	99.36886	519.6	5.0	76.82927	426
19AR115_Spot 473	564.8563	73418.66	4.937638	17.13433	1.41939	0.6735	1.534536	0.083958	0.575154	0.374807	519.7024	2.871775	522.8381	6.270819	537.4905	31.15182	519.7	2.9	96.69053	432
19AR115_Spot 109	175.4375	20929.4	3.647865	16.67534	3.882339	0.689157	4.088916	0.083974	1.00153	0.244938	519.7984	5.00158	532.2937	16.94051	587.1594	86.0534	519.8	5.0	88.52765	98
19AR115_Spot 130	291.9189	30837.48	1.774606	17.30471	2.389979	0.666828	2.616471	0.083984	0.847348	0.323851	519.8583	4.232071	518.7823	10.62882	514.9818	54.40094	519.9	4.2	100.9469	119
19AR115_Spot 108	231.8327	39097.87	4.414447	17.12453	3.383149	0.674721	3.49464	0.083988	0.717952	0.205444	519.8854	3.585987	523.5787	14.29693	540.673	74.81308	519.9	3.6	96.15524	97
19AR115_Spot 344	106.0208	15191.39	1.49716	16.67862	5.414191	0.683009	5.562326	0.084013	0.991466	0.178247	520.0323	4.953459	528.5912	22.9245	566.6888	119.2543	520.0	5.0	91.76682	313
19AR115_Spot 474	197.7477	17615.47	2.316291	17.03373	3.066102	0.67193	3.30127	0.084065	0.753001	0.228094	520.3418	3.764218	521.8852	13.47232	529.6205	70.42468	520.3	3.8	98.24806	433
19AR115_Spot 377	238.6729	23354.43	3.685671	17.6611	2.662781	0.648099	2.95871	0.084068	0.808132	0.273136	520.3595	4.039943	507.308	11.81435	449.8384	63.24118	520.4	4.0	115.677	345
19AR115_Spot 425	417.0529	28655.77	4.512858	16.97659	1.612208	0.67876	1.908142	0.08407	0.748073	0.392043	520.3723	3.739791	526.0247	7.833881	551.5859	38.32704	520.4	3.7	94.34112	390
19AR115_Spot 423	196.6056	20297.87	2.734026	17.62663	3.480464	0.651556	3.620629	0.08408	0.734066	0.202745	520.4271	3.670137	509.4361	14.50448	461.3452	78.63312	520.4	3.7	112.8064	388

19AR115_Spot 471	222.6589	24468.61	1.556909	16.86155	2.505366	0.679946	2.683227	0.084108	0.892335	0.33256	520.5978	4.462847	526.7418	11.02766	554.4107	55.19678	520.6	4.5	93.90111	430
19AR115_Spot 255	348.5286	41282.62	3.75359	17.09673	2.107207	0.670689	2.237683	0.084137	0.663119	0.296342	520.7718	3.317532	521.1312	9.121488	523.6414	46.87775	520.8	3.3	99.45198	235
19AR115_Spot 389	279.0931	26892.94	3.405144	17.74127	1.800165	0.648382	1.982265	0.084146	0.599436	0.3024	520.825	2.999224	507.4827	7.917237	448.743	41.98037	520.8	3.0	116.0631	357
19AR115_Spot 472	216.7429	39080.97	1.360068	17.03896	2.980764	0.67567	3.055891	0.084147	0.620924	0.203189	520.8273	3.106748	524.1539	12.51226	539.6028	65.44799	520.8	3.1	96.52051	431
19AR115_Spot 287	130.0602	13058.34	1.679043	15.95266	4.073755	0.71335	4.224151	0.084153	0.886021	0.209751	520.8662	4.433464	546.7333	17.85955	656.9733	88.59175	520.9	4.4	79.2827	264
19AR115_Spot 314	365.1847	33886.87	1.482734	17.05322	1.258057	0.676259	1.460454	0.08416	0.721141	0.493779	520.9033	3.608685	524.5108	5.982664	541.199	27.78221	520.9	3.6	96.24986	286
19AR115_Spot 105	139.8143	18787.78	2.596826	16.43464	4.822751	0.701294	4.906557	0.08418	0.753769	0.153625	521.0218	3.772781	539.5634	20.53935	619.6248	104.6809	521.0	3.8	84.08667	94
19AR115_Spot 84	308.0222	47812.34	3.757822	17.3604	1.966559	0.665749	2.151382	0.084189	0.733834	0.341099	521.077	3.673378	518.1244	8.7309	506.0904	44.49265	521.1	3.7	102.9613	76
19AR115_Spot 321	713.8928	29704.51	0.858046	17.04631	0.729121	0.67475	1.001733	0.084196	0.541055	0.540118	521.1208	2.708594	523.5962	4.09805	535.3754	18.47452	521.1	2.7	97.33746	292
19AR115_Spot 270	303.9451	30881.61	3.245232	16.45906	2.297144	0.699298	2.418066	0.084202	0.664885	0.274965	521.1581	3.328732	538.3715	10.10427	612.8834	50.23338	521.2	3.3	85.03381	248
19AR115_Spot 13	242.5932	29377.8	1.959396	17.36887	2.099034	0.663662	2.286488	0.084218	0.851191	0.37227	521.2522	4.262209	516.8517	9.261747	498.3815	46.76138	521.3	4.3	104.589	13
19AR115_Spot 41	373.5963	29044.24	1.878475	17.61001	2.331569	0.655588	2.516657	0.084229	0.576187	0.228949	521.3158	2.885509	511.9115	10.11922	471.1154	54.22362	521.3	2.9	110.6556	40
19AR115_Spot 292	368.5749	32706.67	5.184508	17.05494	1.54232	0.675334	1.705935	0.084233	0.556655	0.326305	521.3398	2.787816	523.9507	6.982591	536.3192	35.27256	521.3	2.8	97.207	266
19AR115_Spot 205	536.8823	27187.33	0.971779	17.12308	1.5162	0.672374	1.705377	0.084244	0.641787	0.376331	521.403	3.214542	522.1549	6.96201	526.4201	34.62272	521.4	3.2	99.04694	189
19AR115_Spot 117	264.9744	33462.2	2.813952	17.3857	1.776767	0.665279	1.937635	0.084248	0.749878	0.387007	521.427	3.756109	517.8378	7.860087	502.9868	39.31989	521.4	3.8	103.6661	106
19AR115_Spot 5	124.2161	15132.66	1.391541	17.02055	4.204672	0.674126	4.343932	0.084278	0.983565	0.226423	521.6046	4.92825	523.218	17.76275	531.2406	92.71603	521.6	4.9	98.18613	5
19AR115_Spot 469	325.6464	33592.55	1.678571	17.06922	1.813966	0.674861	2.087992	0.084284	0.987132	0.472766	521.6441	4.946484	523.6634	8.542881	533.4244	40.27985	521.6	4.9	97.79158	428
19AR115_Spot 70	159.6767	13612.01	2.255569	16.34602	4.126984	0.698226	4.268169	0.084296	0.687443	0.161063	521.7148	3.445198	537.7308	17.82037	607.1657	91.12953	521.7	3.4	85.92626	64
19AR115_Spot 104	343.6839	54127.81	1.820569	17.06262	1.669277	0.682032	1.78591	0.084305	0.628248	0.351781	521.766	3.148836	528.002	7.353053	556.0225	36.46595	521.8	3.1	93.83902	93
19AR115_Spot 86	212.0968	35057.66	2.286085	18.02873	2.389976	0.640702	2.531884	0.084329	0.704977	0.27844	521.9075	3.534326	502.7408	10.03955	417.383	54.33557	521.9	3.5	125.0428	78
19AR115_Spot 388	301.4508	41379.92	5.231982	17.56337	2.416351	0.658136	2.503615	0.08433	0.585654	0.233923	521.9156	2.936157	513.4729	10.09037	477.0279	53.84262	521.9	2.9	109.4099	356
19AR115_Spot 404	173.2552	17530.12	2.327466	16.67881	3.066904	0.690168	3.232933	0.08433	0.734923	0.227324	521.9167	3.68452	532.9013	13.40531	581.1726	68.40401	521.9	3.7	89.80409	372
19AR115_Spot 180	303.7065	32823.69	1.560172	17.27347	1.696698	0.667077	1.91631	0.084352	0.67738	0.353481	522.046	3.396836	518.9336	7.786179	506.2141	39.41815	522.0	3.4	103.1275	164
19AR115_Spot 499	307.7719	32635.4	3.068742	17.05398	2.031826	0.675287	2.317919	0.08436	1.06487	0.459408	522.0935	5.340439	523.922	9.487245	532.8738	45.08179	522.1	5.3	97.97694	453
19AR115_Spot 248	303.5524	14390.63	1.946344	16.72851	3.131118	0.681631	3.270195	0.084367	0.779458	0.238352	522.1347	3.909361	527.7597	13.46008	553.0916	69.30241	522.1	3.9	94.40293	229
19AR115_Spot 475	245.8983	22258.95	1.106871	17.1069	2.668902	0.67343	2.802475	0.084368	0.657109	0.234474	522.1442	3.295778	522.796	11.45183	526.6187	59.73442	522.1	3.3	99.15032	434
19AR115_Spot 354	443.1556	48205.18	4.524942	17.19014	1.636405	0.672678	1.755041	0.084369	0.593018	0.337894	522.1493	2.974354	522.3394	7.166701	524.1039	36.22682	522.1	3.0	99.62706	323
19AR115_Spot 378	175.5414	6654.211	4.23075	15.95761	5.239715	0.703005	6.332984	0.084373	0.832237	0.131413	522.1739	4.174374	540.584	26.55092	619.9205	135.5864	522.2	4.2	84.23241	346
19AR115_Spot 415	283.635	12268.33	2.99021	17.18965	2.345108	0.665214	2.535542	0.0844	0.78809	0.310817	522.3341	3.954103	517.7983	10.28506	498.7714	53.10826	522.3	4.0	104.7242	382
19AR115_Spot 12	226.7636	34907.63	3.233856	17.33442	1.535182	0.66707	1.760674	0.0844	0.836112	0.474882	522.3345	4.195049	518.9295	7.153747	504.9408	34.07332	522.3	4.2	103.4447	12
19AR115_Spot 111	241.8976	21642.26	4.846413	17.7667	2.895229	0.650071	3.089333	0.08443	0.772264	0.249978	522.5105	3.875957	508.5227	12.35875	447.0506	66.49996	522.5	3.9	116.8795	100
19AR115_Spot 387	110.483	16830.53	1.185755	15.90565	3.723436	0.722124	3.90838	0.084472	0.905873	0.231777	522.7613	4.548628	551.9202	16.64229	675.0464	81.30296	522.8	4.5	77.4408	355
19AR115_Spot 322	324.9758	59561.59	4.673972	17.3272	1.751799	0.668747	1.857921	0.084473	0.505099	0.271862	522.7644	2.53625	519.9502	7.560254	508.5424	39.33197	522.8	2.5	102.7966	293
19AR115_Spot 193	132.2583	14173.32	1.317423	16.56039	4.262359	0.690579	4.506219	0.084475	0.808233	0.179359	522.7779	4.058476	533.1484	18.69258	578.7308	96.36682	522.8	4.1	90.33178	177
19AR115_Spot 217	202.331	17881.21	1.464208	16.58217	3.470932	0.692967	3.802388	0.084492	0.997753	0.262402	522.8775	5.011053	534.5813	15.80467	585.8057	79.66593	522.9	5.0	89.25785	201
19AR115_Spot 456	215.4022	20659.88	1.299283	17.01842	2.486414	0.676857	2.634818	0.084504	0.743668	0.282246	522.9535	3.735472	524.8733	10.79936	534.183	55.36672	523.0	3.7	97.89781	417
19AR115_Spot 355	341.8293	29375.56	8.082405	17.76283	1.859904	0.649699	2.088296	0.08451	0.620918	0.297332	522.9846	3.119073	508.2933	8.351012	443.677	44.35871	523.0	3.1	117.8751	324
19AR115_Spot 293	787.7894	94792.24	5.561428	17.04294	0.961323	0.681455	1.110371	0.084514	0.549813	0.495162	523.0117	2.762028	527.6535	4.569334	548.7557	21.06726	523.0	2.8	95.30865	267
19AR115_Spot 372	189.8828	25147.24	2.733062	17.40722	2.553336	0.661878	2.796029	0.08454	0.989893	0.354035	523.1626	4.97418	515.762	11.30754	484.0869	57.76545	523.2	5.0	108.072	340
19AR115_Spot 468	312.8691	21129.68	2.658051	16.75136	1.869841	0.688052	2.293497	0.084564	0.91505	0.398976	523.307	4.599316	531.6293	9.492403	568.4789	45.77935	523.3	4.6	92.05391	427
19AR115_Spot 113	141.6954	18140.32	1.206618	16.03453	2.607001	0.720252	2.962188	0.084566	0.974325	0.328921	523.3183	4.897348	550.8154	12.59381	667.1331	59.90334	523.3	4.9	78.44286	102
19AR115_Spot 7	147.5861	20732.46	1.221066	16.81951	2.679909	0.687698	2.922026	0.084566	0.885481	0.303037	523.3204	4.450801	531.4165	12.09032	567.3005	60.63341	523.3	4.5	92.24748	7
19AR115_Spot 449	307.133	46980.4	7.290329	17.10962	1.828508	0.681011	1.967411	0.084573	0.667577	0.339317	523.3635	3.355788	527.3854	8.093156	545.8046	40.45549	523.4	3.4	95.88843	410
19AR115_Spot 374	502.1228	57750.88	8.50658	17.56341	1.882084	0.660151	2.055474	0.084595	0.767602	0.373443	523.4902	3.859493	514.7066	8.299415	476.8321	42.1602	523.5	3.9	109.785	342
19AR115_Spot 209	796.5342	86266.87	5.055407	17.19338	0.999764	0.676828	1.207137	0.084613	0.654788	0.54243	523.5971	3.292909	524.8555	4.94743	531.3076	22.20614	523.6	3.3	98.54877	193
19AR115_Spot 158	374.8109	41431.06	3.108207	17.3799	2.124978	0.665176	2.266391	0.084628	0.754852	0.333063	523.6871	3.796756	517.7753	9.192912	492.754	47.14305	523.7	3.8	106.2776	145
19AR115_Spot 177	544.6744	77986.23	5.832894	17.09705	1.347726	0.677325	1.513001	0.084637	0.649035	0.428972	523.7407	3.264841	525.1561	6.203748	532.2888	29.91947	523.7	3.3	98.39409	161
19AR115_Spot 206	392.3417	86296.95	3.710522	17.81043	1.388596	0.65347	1.536826	0.084637	0.651139	0.423691	523.7423	3.275434	510.6122	6.16722	453.188	30.92573	523.7	3.3	115.5685	190
19AR115_Spot 370	542.8538	63690.62	1.580737	17.22786	1.427763	0.673814	1.626426	0.084653	0.731352	0.449668	523.8346	3.679553	523.0286	6.648187	520.4452	31.87379	523.8	3.7	100.6513	338
19AR115_Spot 124	151.8643	17107.43	1.768609	17.07739	3.001118	0.676753	3.392203	0.084656	0.79316	0.233819	523.8515	3.990641	524.8099	13.90272	529.9568	72.27698	523.9	4.0	98.84797	113
19AR115_Spot 274	554.5403	66653.79	121.1313	17.40485	0.915988	0.667103	1.153065	0.084662	0.620194	0.537866	523.8918	3.120626	518.9497	4.685086	498.1893	21.39821	523.9	3.1	105.1592	252
19AR115_Spot 417	858.9387	84635.44	177.3128	17.26352	0.902469	0.676319	1.081107	0.084674	0.586936	0.542903	523.9588	2.953641	524.5471	4.428902	528.0832	19.89774	524.0	3.0	99.21899	383
19AR115_Spot 93	312.1923	68942.62	3.489092	17.47145	2.580594	0.668147	2.721127	0.084682	0.788935	0.289929	524.0103	3.970538	519.5849	11.06709	501.1553	57.34561	524.0	4.0	104.5605	84
19AR115_Spot 11	225.0525	24409.24	4.207071	17.34784	2.865514	0.667008	3.111622	0.084689	1.154216	0.370937	524.0486	5.809329	518.8915	12.64248	497.2063	63.69605	524.0	5.8	105.3986	11
19AR115_Spot 54	112.1272	10320.36	1.638788	15.6475	3.367362	0.733729	3.608606	0.084702	0.914087	0.253308	524.1302	4.601414	558.7394	15.50806	703.2874	74.3197	524.1	4.6	74.52576	49
19AR115_Spot 138	156.1949	15167.24	1.480924	17.37326	2.15166	0.662285	2.340429	0.084706	0.755552	0.322826	524.1514	3.80351	516.0109	9.46842	481.0618	48.96376	524.2	3.8	108.9572	126
19AR115_Spot 400	163.0853	6940.433	1.174799	14.92773	3.023603	0.761143	3.125342	0.084713	0.638503	0.204299	524.1909	3.21451	574.6693	13.71594	780.5754	64.3177	524.2	3.2	67.15442	368
19AR115_Spot 498	134.1727	13339.01	2.221498	15.69251	4.654196	0.730904	4.900565	0.08473	1.37183	0.279933	524.292	6.907685	557.0837	21.01481	694.3671	100.3149	524.3	6.9	75.50645	452
19AR115_Spot 31	150.4779	16301.12	1.837254	16.12462	4.865048	0.717502	5.098057	0.084755	0.880593	0.172731	524.444	4.435352	549.1911	21.62848	654.1461	107.7833	524.4	4.4	80.1723	30
19AR115_Spot 230	356.5188	60264.29	2.932093	16.95212	1.912958	0.685237	2.006486	0.084766	0.588603	0.29335	524.5057	2.964997	529.9344	8.28429	554.3246	41.86629	524.5	3.0	94.6207	211
19AR115_Spot 57	155.2617	14520.03	2.168359	16.27234	3.2902	0.709438	3.607939	0.08477	0.812818	0.225286	524.5333	4.094657	544.4123	15.20486	629.4414	75.74389	524.5	4.1	83.33315	52
19AR115_Spot 185	170.0228	25138.42	2.276273	16.33523	4.111794	0.706054	4.198164	0.084785	0.75888	0.180765	524.6225	3.823561	542.4003	17.64323	618.7498	89.13024	524.6	3.8	84.78751	169
19AR115_Spot 163	288.6921	30788.5	3.961947	17.2752	2.383902	0.669114	2.530338	0.084791	0.797686	0.315249	524.6586	4.019345	520.1734	10.3	501.4876	52.88642	524.7	4.0	104.6205	150
19AR115_Spot 148	268.5559	20924.57	2.549266	17.54006	2.082013	0.658531	2.231634	0.084804	0.625546	0.280308	524.7323	3.152399	513.7152	8.997398	465.9751	47.4764	524.7	3.2	112.6095	135
19AR115_Spot 190	489.1259	34247.71	3.095598	17.20355	1.681076	0.673929	1.859287	0.084808	0.602426	0.324009	524.7589	3.036036	523.0984	7.600839	516.791	38.64764	524.8	3.0	101.5418	174
19AR115_Spot 298	372.6363	30311.29	2.766116	17.39477	1.524814	0.666257	1.721866	0.084816	0.698729	0.405797	524.807	3.521681	518.4338	6.990941	491.3857	34.71761	524.8	3.5	106.8014	272
19AR115_Spot 235	137.4048	13119.81	1.336876	15.91874	3.216686	0.719098	3.31241	0.084817	0.679655	0.205185	524.8132	3.425588	550.1341	14.06984	657.3589	69.53093	524.8	3.4	79.83663	216
19AR115_Spot 399	114.7503	12205.3	1.364918	17.04568	3.466172	0.674466	3.678765	0.084844	0.693364	0.188477	524.9715	3.495694	523.4242	15.04692	517.6143	79.35598	525.0	3.5	101.4214	367
19AR115_Spot 114	210.5054	19428.07	4.338268	16.4872	3.481524	0.703151	3.716265	0.084847	0.755698	0.203349	524.9899	3.810086	540.6714	15.57996	608.2757	78.68934	525.0	3.8	86.30788	103
19AR115_Spot 184	236.6297	25511.88	1.510864	16.77212	2.490273	0.688084	2.704514	0.084848	0.671869	0.248425	524.9976	3.387483	531.6488	11.19397	561.2685	57.08854	525.0	3.4	93.53768	168
19AR115_Spot 282	317.7583	2185.597	5.589018	15.21675	1.968061	0.691877	2.176058	0.08485	0.578586	0.265887	525.0073	2.917217	533.9277	9.035913	573.1929	45.60662	525.0	2.9	91.59348	259
19AR115_Spot 58	307.9309	27818.8	2.379436	16.79803	2.094759	0.692465	2.238755	0.084879	0.696901	0.31129	525.1802	3.514868	534.2807	9.300943	574.2777	46.24643	525.2	3.5	91.45058	53
19AR115_Spot 203	569.826	45100.59	5.430657	17.24121	1.123757	0.675267	1.374991	0.084882	0.572708	0.416518	525.1974	2.888584	523.91	5.627634	519.2359	27.4169	525.2	2.9	101.1481	187
19AR115_Spot 286	292.6608	21959.9	4.206645	16.97775	1.821125	0.680759	1.924765	0.084913	0.596139	0.30972	525.3799	3.007763	527.2332	7.915977	536.2385	40.03806	525.4	3.0	97.97505	263
19AR115_Spot 2	118.0888	7974.533	1.989961	16.51499	3.010241	0.690481	3.825436	0.084916	0.731011	0.191092	525.4006	3.688391	533.0893	15.86674	567.1008	81.7666	525.4	3.7	92.64677	2
19AR115_Spot 324	187.7102	28757.76	1.892504	16.24649	3.393982	0.713991	3.46164	0.084922	0.493677	0.142614	525.4349	2.491051	547.1135	14.64287	639.4054	73.70485	525.4	2.5	82.17556	295
19AR115_Spot 196	242.0973	29751.35	4.041195	17.4362	2.605057	0.666276	2.775959	0.084948	0.704618	0.253829	525.5924	3.556463	518.4457	11.27114	488.0155	59.25895	525.6	3.6	107.6999	180
19AR115_Spot 96	539.1642	72113.73	8.937638	17.26002	1.281557	0.678621	1.448731	0.084951	0.664751	0.458851	525.6091	3.355346	525.9407	5.946993	528.3554	28.18987	525.6	3.4	99.48022	87
19AR115_Spot 376	158.7343	16855.8	1.44779	17.27277	3.293727	0.667091	3.685374	0.084963	0.901379	0.244583	525.6791	4.550309	518.9421	14.97506	490.3358	78.86478	525.7	4.6	107.208	344
19AR115_Spot 107	343.848	28110.12	1.21217	17.32527	2.689329	0.67208	2.829563	0.084968	0.628793	0.222223	525.709	3.174422	521.9765	11.54867	506.6394	60.71423	525.7	3.2	103.7639	96
19AR115_Spot 160	230.0421	34592.42	5.373478	17.03318	2.40846	0.680755	2.558388	0.084972	0.829301	0.32415	525.7312	4.186848	527.2308	10.522	534.6841	52.97772	525.7	4.2	98.32557	147
19AR115_Spot 225	604.7237	36589.18	1.196117	17.03952	1.354711	0.681577	1.476006	0.084976	0.578572	0.391985	525.7565	2.92114	527.7269	6.074649	537.2073	29.73479	525.8	2.9	97.86844	206
19AR115_Spot 194	765.0597	71941.26	6.571878	17.24628	1.063862	0.677018	1.255827	0.084977	0.614566	0.489372	525.7628	3.102906	524.9705	5.147849	522.4601	24.02457	525.8	3.1	100.6321	178
19AR115_Spot 38	285.4471	28549.43	10.67716	16.98609	2.056034	0.6863	2.167925	0.084978	0.634477	0.292665	525.7672	3.203459	530.575	8.959103	552.2781	45.25093	525.8	3.2	95.19973	37
19AR115_Spot 465	228.6002	28826.16	3.154617	16.99648	2.949599	0.683916	3.096593	0.084983	0.897698	0.289899	525.7973	4.532709	529.1382	12.77083	544.5047	64.78125	525.8	4.5	96.56433	424
19AR115_Spot 343	341.9502	34458.85	1.550297	17.31156	2.349595	0.671323	2.478938	0.084986	0.708465	0.285794	525.8179	3.577356	521.5163	10.1107	503.66	52.29003	525.8	3.6	104.3994	312
19AR115_Spot 208	125.2104	20840.1	2.970601	15.54954	4.686665	0.745775	4.770852	0.084988	0.834776	0.174974	525.83	4.215248	565.7698	20.69691	730.7096	99.5781	525.8	4.2	71.96155	192
19AR115_Spot 353	357.9484	48687.15	1.144305	17.32221	1.858586	0.672557	1.997667	0.085001	0.62942	0.315077	525.906	3.178731	522.2659	8.156626	507.3153	41.71273	525.9	3.2	103.6645	322
19AR115_Spot 83	173.5443	18852.51	2.144319	16.67686	2.674126	0.694297	2.852955	0.085003	0.785913	0.275473	525.9164	3.96914	535.3788	11.87136	576.8668	59.64185	525.9	4.0	91.16773	75
19AR115_Spot 405	98.27631	10435.09	1.824402	17.40551	3.610914	0.659561	3.990421	0.085005	0.780924	0.1957	525.9275	3.944024	514.3455	16.10448	464.1688	86.7372	525.9	3.9	113.3052	373
19AR115_Spot 3	439.2998	49609.73	1.087776	17.41175	1.223877	0.672382	1.396988	0.085021	0.635483	0.454895	526.021	3.210025	522.1595	5.703054	506.2782	27.38541	526.0	3.2	103.8996	3
19AR115_Spot 182	505.4439	67970.8	4.545402	16.81674	1.277472	0.693521	1.424325	0.085029	0.609022	0.427586	526.0708	3.076642	534.9136	5.922607	573.741	27.99645	526.1	3.1	91.69134	166
19AR115_Spot 151	378.0468	48421.79	4.347907	17.41012	2.036502	0.667949	2.153393	0.085033	0.585947	0.272104	526.0949	2.960201	519.4645	8.756378	491.3505	45.71245	526.1	3.0	107.0712	138
19AR115_Spot 390	213.6038	23695.08	2.326897	17.91698	2.66179	0.647959	2.880324	0.085046	0.675846	0.234642	526.1698	3.414839	507.2223	11.49982	423.6233	62.49905	526.2	3.4	124.207	358
19AR115_Spot 289	571.4916	70794.28	8.18581	17.08152	1.268286	0.683584	1.388343	0.085046	0.541947	0.390355	526.1736	2.738308	528.9384	5.723855	541.8164	27.9565	526.2	2.7	97.11288	265
19AR115_Spot 446	99.97368	13818.54	1.657431	16.10381	4.058931	0.719019	4.273525	0.085058	1.092227	0.25558	526.2459	5.519448	550.0877	18.1519	650.9919	88.73018	526.2	5.5	80.83755	407
19AR115_Spot 312	376.9316	31527.95	3.052686	17.23518	2.102806	0.676446	2.234701	0.085061	0.728272	0.325892	526.2593	3.68033	524.624	9.155975	518.4484	46.38161	526.3	3.7	101.5066	284
19AR115_Spot 386	432.1514	35563.57	5.95769	17.1726	1.719601	0.678073	1.85228	0.085065	0.638066	0.344476	526.2817	3.224608	525.609	7.599941	523.6225	38.14106	526.3	3.2	100.5079	354
19AR115_Spot 484	544.5543	59065.8	5.835293	17.25369	1.163318	0.675931	1.290756	0.085068	0.543061	0.420731	526.3013	2.744574	524.312	5.285963	516.5932	25.73401	526.3	2.7	101.8792	441
19AR115_Spot 161	344.6433	40734.43	1.538239	17.2104	2.248471	0.675349	2.418596	0.085088	0.758917	0.313784	526.4183	3.83631	523.9593	9.899877	514.2135	50.43523	526.4	3.8	102.3735	148
19AR115_Spot 188	102.7208	15087.89	1.350692	16.01819	5.604666	0.719293	5.735212	0.085088	1.0919	0.190385	526.4212	5.51956	550.2497	24.36794	651.1092	120.9335	526.4	5.5	80.84991	172
19AR115_Spot 88	201.7924	19052.3	1.816783	17.78741	2.602085	0.651914	2.811035	0.085097	0.751354	0.267287	526.4722	3.798449	509.6557	11.26462	435.8502	60.34076	526.5	3.8	120.792	80
19AR115_Spot 220	232.8263	27815.43	1.488047	17.01833	2.80024	0.682183	2.954668	0.085103	0.918887	0.310995	526.5124	4.64575	528.093	12.1671	535.8613	61.47957	526.5	4.6	98.25536	202
19AR115_Spot 162	229.4036	21182.04	2.938645	16.90875	2.448523	0.684549	2.734807	0.085127	0.79143	0.289391	526.6507	4.002355	529.5201	11.28484	542.8373	57.23177	526.7	4.0	97.01814	149
19AR115_Spot 352	172.1275	23008.1	2.797814	17.15582	2.880047	0.676337	3.115698	0.085132	0.743735	0.238706	526.685	3.76139	524.5582	12.76468	516.2462	66.45642	526.7	3.8	102.0221	321
19AR115_Spot 167	97.65419	9891.154	1.285073	15.53624	5.622041	0.73608	5.90724	0.085134	0.850007	0.143892	526.6926	4.298915	560.1155	25.43669	699.2675	124.5828	526.7	4.3	75.32061	153
19AR115_Spot 373	418.9553	9841.418	6.380603	17.0444	2.061825	0.670243	2.82002	0.085137	0.715654	0.253776	526.7146	3.61957	520.86	11.49088	496.2275	60.11622	526.7	3.6	106.1438	341
19AR115_Spot 60	319.2515	27001.92	2.606838	17.5408	1.952476	0.664852	2.102606	0.085157	0.646462	0.307458	526.834	3.270328	517.5777	8.526043	477.8637	44.23043	526.8	3.3	110.2478	54
19AR115_Spot 305	245.3949	37435.73	4.284423	16.55242	2.976153	0.705139	3.081477	0.085165	0.728807	0.236512	526.8797	3.687204	541.856	12.9398	606.2915	64.77442	526.9	3.7	86.90205	278
19AR115_Spot 87	269.1606	24058.71	1.478042	16.92445	2.298194	0.687403	2.500241	0.085166	0.776433	0.310543	526.8866	3.928201	531.2388	10.34235	550.9414	51.88986	526.9	3.9	95.63387	79
19AR115_Spot 153	216.7325	20376.95	2.202879	16.1412	1.860451	0.716714	2.079221	0.085169	0.654728	0.314891	526.9035	3.312561	548.7254	8.814333	641.3422	42.44821	526.9	3.3	82.15637	140
19AR115_Spot 273	290.3316	44728.57	1.775203	17.31341	1.494068	0.674465	1.704299	0.085175	0.658414	0.386325	526.9393	3.331427	523.4233	6.97053	509.09	34.56194	526.9	3.3	103.5061	251
19AR115_Spot 450	83.16566	10869.07	1.237262	16.94793	3.148737	0.678527	3.373711	0.085178	0.936969	0.277727	526.9553	4.740996	525.8839	13.84853	522.1695	71.11672	527.0	4.7	100.9165	411
19AR115_Spot 75	906.978	68782.45	4.204597	17.16894	1.036627	0.681144	1.23727	0.085181	0.673106	0.544025	526.9708	3.405964	527.4657	5.090168	530.5818	22.74284	527.0	3.4	99.31943	68
19AR115_Spot 359	209.1067	40437.2	4.27224	16.66613	2.185753	0.700394	2.352964	0.08522	0.727352	0.309121	527.2032	3.682008	539.0262	9.841283	590.2998	48.53249	527.2	3.7	89.31108	328
19AR115_Spot 192	333.7117	41680.09	24.83011	16.83994	1.827723	0.692822	1.992687	0.08522	0.646345	0.324358	527.2032	3.271934	534.4943	8.281115	566.7027	41.04357	527.2	3.3	93.02995	176
19AR115_Spot 392	88.87272	11120.97	1.256652	15.47652	4.189274	0.745019	4.304997	0.085246	0.906031	0.21046	527.3612	4.587842	565.3302	18.66461	722.099	89.33443	527.4	4.6	73.0317	360
19AR115_Spot 36	499.6412	95466.03	3.337547	17.09093	1.592066	0.690164	1.730103	0.085251	0.67281	0.388885	527.3916	3.407077	532.8991	7.173525	557.4773	34.75929	527.4	3.4	94.60324	35
19AR115_Spot 42	197.6099	1944.872	1.720455	15.0003	4.523944	0.698068	12.89738	0.085253	0.984656	0.076345	527.4029	4.986349	537.6366	53.88673	582.231	280.3104	527.4	5.0	90.58311	41
19AR115_Spot 207	274.9529	9782.614	2.826513	16.94566	1.754321	0.676854	2.031789	0.085266	0.720673	0.354699	527.4771	3.650016	524.8713	8.327567	514.4874	41.74061	527.5	3.7	102.5248	191
19AR115_Spot 14	380.3434	54372.41	3.550155	17.4675	1.657651	0.670854	1.846953	0.085277	0.756583	0.409638	527.5428	3.832349	521.2313	7.529793	494.6265	37.14541	527.5	3.8	106.6548	14
19AR115_Spot 408	168.5204	17186.14	1.692093	17.95425	3.654907	0.646138	3.780362	0.08528	0.777628	0.205702	527.56	3.939076	506.0996	15.06796	411.1979	82.73689	527.6	3.9	128.2983	376
19AR115_Spot 396	151.1587	20683.33	2.128928	17.08474	2.79717	0.681777	3.105863	0.085286	0.78464	0.252632	527.5986	3.974874	527.8479	12.78525	529.9018	65.86254	527.6	4.0	99.56536	364
19AR115_Spot 229	236.0162	25313.28	3.326005	17.59156	3.236351	0.660436	3.309282	0.085296	0.636881	0.192453	527.6562	3.226687	514.8806	13.36587	459.5322	72.04987	527.7	3.2	114.8247	210
19AR115_Spot 299	240.5324	21485.09	4.229276	17.17755	3.184209	0.675248	3.293412	0.085303	0.704016	0.213765	527.6988	3.567092	523.898	13.47986	508.3448	70.76599	527.7	3.6	103.8073	273
19AR115_Spot 438	606.5823	82964.51	3.379071	17.09013	0.968466	0.689825	1.152035	0.085309	0.619321	0.537589	527.7319	3.138151	532.6951	4.775247	554.9389	21.19119	527.7	3.1	95.0973	401
19AR115_Spot 140	190.5609	22007.79	3.322535	17.06475	1.949566	0.682748	2.195418	0.085318	0.703439	0.320412	527.7853	3.564729	528.4342	9.044824	532.2122	45.54598	527.8	3.6	99.16821	127
19AR115_Spot 363	278.9858	32413.31	2.873296	17.01523	2.053178	0.685478	2.200341	0.085371	0.729249	0.331425	528.105	3.697673	530.0798	9.086611	539.5219	45.41881	528.1	3.7	97.88388	331
19AR115_Spot 311	493.1628	66266.08	6.146752	17.14267	0.919219	0.684921	1.161686	0.085379	0.673623	0.579867	528.1524	3.415916	529.7445	4.794938	537.541	20.72921	528.2	3.4	98.25342	283
19AR115_Spot 263	160.1668	13384.22	3.044231	17.11248	3.6385	0.672893	3.954447	0.085387	0.831914	0.210374	528.1968	4.218941	522.4702	16.15215	498.4546	85.20378	528.2	4.2	105.9669	241
19AR115_Spot 278	238.498	40265.69	4.241854	16.98023	2.993102	0.687965	3.134729	0.085388	0.748875	0.238896	528.2023	3.797857	531.5773	12.97348	547.0215	66.50976	528.2	3.8	96.55969	256
19AR115_Spot 249	73.70811	9110.479	1.383159	16.05126	4.539469	0.712196	4.84528	0.085394	1.45649	0.3006	528.2392	7.386964	546.0495	20.46698	622.002	99.73274	528.2	7.4	84.92563	230
19AR115_Spot 294	137.7309	23967.16	2.155316	16.39423	4.025758	0.709563	4.142523	0.085403	0.842183	0.203302	528.2894	4.271736	544.4867	17.45997	613.7933	87.64475	528.3	4.3	86.06958	268
19AR115_Spot 186	227.9904	33193.96	2.019316	17.16173	2.777622	0.678327	2.968573	0.085414	1.00366	0.338095	528.3554	5.091396	525.7627	12.18319	515.455	61.37816	528.4	5.1	102.5027	170
19AR115_Spot 409	120.3147	16261.77	1.660082	15.79101	4.134845	0.736775	4.246254	0.085421	0.860205	0.20258	528.401	4.364032	560.5216	18.29254	694.1108	88.67048	528.4	4.4	76.12632	377
19AR115_Spot 382	283.6504	42055.06	5.450002	17.66879	2.527545	0.661894	2.676207	0.085426	0.832584	0.311106	528.4309	4.224135	515.7718	10.82309	461.0134	56.39364	528.4	4.2	114.6237	350
19AR115_Spot 183	271.1286	24252.39	3.572911	17.28627	1.978799	0.673283	2.113599	0.085434	0.705164	0.333632	528.476	3.577958	522.7063	8.635569	498.5155	43.89991	528.5	3.6	106.0099	167
19AR115_Spot 150	211.836	31412.14	1.874225	16.63784	2.81807	0.700525	2.938847	0.085435	0.713214	0.242685	528.4798	3.618827	539.1044	12.29331	585.264	61.91173	528.5	3.6	90.29767	137
19AR115_Spot 434	204.6266	22454.39	2.197825	17.28659	3.361786	0.677769	3.496148	0.085437	0.662313	0.189441	528.4959	3.360657	525.4253	14.34162	513.0817	75.44594	528.5	3.4	103.0042	397
19AR115_Spot 391	368.3068	44038.26	4.722037	17.51304	1.432636	0.669506	1.600251	0.085441	0.583368	0.364548	528.5151	2.960184	520.4123	6.516143	485.9722	32.89565	528.5	3.0	108.7542	359
19AR115_Spot 394	320.6361	55288.24	1.924591	17.01803	1.89885	0.690309	2.020348	0.085448	0.675212	0.334206	528.5575	3.426489	532.986	8.378059	552.9277	41.5562	528.6	3.4	95.59252	362
19AR115_Spot 215	109.1631	13880.74	1.769264	15.73844	5.041368	0.736195	5.333428	0.085449	1.219073	0.228572	528.5655	6.186506	560.1828	22.96703	691.714	110.766	528.6	6.2	76.41387	199
19AR115_Spot 9	236.3368	30117.67	1.30363	17.5623	3.388183	0.667706	3.585329	0.085471	0.913428	0.254768	528.6935	4.636509	519.3167	14.57654	479.2195	76.65112	528.7	4.6	110.3239	9
19AR115_Spot 91	161.6022	13632.21	3.228867	16.90972	4.570231	0.687	4.703295	0.085501	0.901452	0.191664	528.8772	4.577246	530.9966	19.45033	541.0485	101.0109	528.9	4.6	97.75043	82
19AR115_Spot 135	261.5906	23646.56	4.134286	17.20185	2.370373	0.680351	2.485372	0.085507	0.685335	0.275748	528.9129	3.480109	526.9863	10.21807	519.5832	52.44582	528.9	3.5	101.7956	123
19AR115_Spot 119	156.6564	17054.82	1.712186	17.33319	3.352836	0.672944	3.516504	0.085511	0.720157	0.204793	528.9344	3.657073	522.5009	14.36374	495.4179	75.89188	528.9	3.7	106.7653	108
19AR115_Spot 358	265.987	33937.32	3.652723	17.21195	1.837031	0.679571	2.042697	0.085514	0.680702	0.333237	528.9512	3.456821	526.515	8.392285	516.9002	42.28788	529.0	3.5	102.3314	327
19AR115_Spot 72	553.3102	54463.85	1.12272	17.3174	1.538761	0.677274	1.720612	0.085514	0.70788	0.411412	528.9534	3.594852	525.1253	7.054723	509.4738	34.48124	529.0	3.6	103.8235	66
19AR115_Spot 6	113.5301	15317.43	2.205047	17.51077	3.921355	0.664677	4.078069	0.085516	0.915962	0.224607	528.9614	4.651633	517.4707	16.53497	468.0093	88.03123	529.0	4.7	113.0237	6
19AR115_Spot 171	344.1217	32611.45	4.067026	17.01065	1.646055	0.685251	1.804766	0.085518	0.659477	0.365409	528.9775	3.349195	529.943	7.4515	535.0605	36.77806	529.0	3.3	98.86312	157
19AR115_Spot 110	130.085	11211.66	1.661728	17.4527	4.730843	0.663046	4.871469	0.085519	0.954208	0.195877	528.9833	4.846051	516.4755	19.72349	462.4978	105.9538	529.0	4.8	114.3753	99
19AR115_Spot 159	177.5647	15725.17	1.245435	17.72139	3.120987	0.652616	3.330172	0.085549	0.830604	0.249418	529.1603	4.219668	510.0874	13.35388	426.4301	71.93489	529.2	4.2	124.0907	146
19AR115_Spot 424	505.4926	43591.66	2.915507	17.14122	1.135216	0.686062	1.355851	0.085549	0.714002	0.526608	529.1623	3.627318	530.4314	5.601909	536.8543	25.21878	529.2	3.6	98.56722	389
19AR115_Spot 133	193.5074	25572.57	1.82171	16.89615	3.48119	0.694283	3.616073	0.085551	0.928036	0.256642	529.1705	4.714735	535.3703	15.04698	562.8009	76.1824	529.2	4.7	94.02446	121

19AR115_Spot 198	199.51	22008.33	1.733203	16.96342	3.585093	0.688062	3.759198	0.085577	0.72953	0.194065	529.3284	3.707318	531.6352	15.55959	542.5018	80.62456	529.3	3.7	97.57174	182
19AR115_Spot 95	140.8238	13752.61	1.642222	17.51248	3.568114	0.66364	3.760277	0.085584	0.829858	0.220691	529.3674	4.217461	516.838	15.23195	462.8066	81.3192	529.4	4.2	114.382	86
19AR115_Spot 261	401.3004	30094.76	1.067475	16.82202	1.524016	0.695232	1.717912	0.085599	0.675644	0.393294	529.4545	3.434269	535.9394	7.153835	564.561	34.39141	529.5	3.4	93.78163	239
19AR115_Spot 264	681.3217	82936.67	6.245552	17.0814	1.282706	0.686591	1.449227	0.085607	0.650747	0.44903	529.5052	3.308019	530.7504	5.990457	537.0414	28.33508	529.5	3.3	98.59671	242
19AR115_Spot 239	257.0845	33434.9	2.01978	17.37299	2.601774	0.672636	2.813001	0.085618	1.025073	0.364405	529.5662	5.211452	522.3136	11.48674	491.7079	57.78245	529.6	5.2	107.6993	220
19AR115_Spot 453	263.5503	34054.93	1.693765	16.7089	2.20444	0.70192	2.348532	0.08562	0.695753	0.29625	529.5788	3.537277	539.9373	9.83532	584.888	48.70435	529.6	3.5	90.54363	414
19AR115_Spot 123	411.9072	48599.64	4.352249	16.90655	1.644497	0.697682	1.780137	0.08563	0.670346	0.37657	529.6412	3.408489	537.4054	7.428349	571.4213	35.87803	529.6	3.4	92.68839	112
19AR115_Spot 327	489.6504	69293.81	3.478561	17.11424	1.638507	0.686614	1.782249	0.085632	0.672415	0.377285	529.6503	3.419068	530.7641	7.367208	536.5109	36.1017	529.7	3.4	98.72127	297
19AR115_Spot 147	159.608	14605.95	2.093419	16.73226	4.847356	0.693947	4.931388	0.085632	0.797388	0.161696	529.6523	4.054538	535.1688	20.51561	559.6789	106.1315	529.7	4.1	94.63502	134
19AR115_Spot 8	238.2594	23968.66	2.129131	17.87434	1.835991	0.65572	2.126535	0.085633	0.847319	0.39845	529.6592	4.308478	511.9926	8.55154	434.817	43.44062	529.7	4.3	121.812	8
19AR115_Spot 348	185.6596	22916.1	5.132737	18.09068	3.069731	0.645008	3.183203	0.085637	0.7185	0.225716	529.6831	3.653612	505.4024	12.67401	397.9131	69.50174	529.7	3.7	133.1153	317
19AR115_Spot 461	246.4433	28716.35	2.451991	17.27703	3.117297	0.677894	3.213918	0.085641	0.677738	0.210876	529.7068	3.446484	525.501	13.18519	508.2487	69.11224	529.7	3.4	104.222	422
19AR115_Spot 463	500.4849	51350.19	4.324162	17.45804	1.022632	0.673447	1.236181	0.085642	0.652612	0.527926	529.714	3.318758	522.8059	5.051345	493.6936	23.12361	529.7	3.3	107.2961	423
19AR115_Spot 39	96.27257	10546.55	1.916147	15.98339	4.697323	0.725051	5.050044	0.085681	1.26816	0.251119	529.9428	6.451701	553.6446	21.55545	653.2973	104.9397	529.9	6.5	81.11817	38
19AR115_Spot 25	164.5263	10369.41	2.017639	17.84691	2.795031	0.647203	3.142421	0.085695	0.779318	0.247999	530.028	3.965355	506.7562	12.53747	404.0051	68.16624	530.0	4.0	131.1934	25
19AR115_Spot 169	256.9722	23815.48	2.817851	17.10732	3.411682	0.680876	3.52419	0.085702	0.703032	0.199488	530.0705	3.577468	527.304	14.4961	516.2802	75.85576	530.1	3.6	102.6711	155
19AR115_Spot 243	167.5024	20524.22	1.790437	17.08053	3.17106	0.681959	3.327513	0.085735	0.68804	0.206773	530.2612	3.502389	527.958	13.69995	518.9477	71.4889	530.3	3.5	102.1801	224
19AR115_Spot 154	343.4008	29473.63	3.207568	17.07222	1.839617	0.684487	2.001075	0.085767	0.617726	0.308697	530.4532	3.145553	529.4828	8.256582	526.2775	41.72895	530.5	3.1	100.7934	141
19AR115_Spot 141	355.503	31626.9	4.522608	17.10066	1.388824	0.687435	1.617577	0.085788	0.646753	0.399828	530.5803	3.294121	531.258	6.691225	535.106	32.4748	530.6	3.3	99.15424	128
19AR115_Spot 71	92.90741	11431.67	2.192449	15.2882	4.185718	0.758532	4.551856	0.085815	1.332089	0.292647	530.736	6.786669	573.1625	19.93874	746.0915	92.01466	530.7	6.8	71.13551	65
19AR115_Spot 127	291.0128	27441.53	2.43391	17.42894	2.128166	0.675816	2.429352	0.08582	0.85025	0.34999	530.7698	4.332079	524.2425	9.948013	496.8923	50.14575	530.8	4.3	106.8179	116
19AR115_Spot 237	420.9909	39480.57	3.238571	16.88106	1.811128	0.694201	1.923355	0.08584	0.618619	0.321635	530.8897	3.152589	535.3214	8.002373	555.179	39.73105	530.9	3.2	95.62497	218
19AR115_Spot 251	187.4254	22716.39	2.340176	16.84347	3.672904	0.693933	3.761645	0.085888	0.658031	0.174932	531.1699	3.355135	535.1608	15.64816	553.1713	80.86127	531.2	3.4	96.02268	232
19AR115_Spot 458	283.72	38373.17	4.213877	17.2597	2.651208	0.682125	2.79658	0.085898	0.740881	0.264924	531.2309	3.777988	528.0581	11.51547	515.301	59.23126	531.2	3.8	103.0914	419
19AR115_Spot 214	190.2461	24457.92	1.31982	16.51822	2.861296	0.710069	3.186674	0.085925	1.028203	0.322657	531.3889	5.244627	544.7873	13.43631	602.1584	65.2936	531.4	5.2	88.24735	198
19AR115_Spot 226	523.875	69367.07	3.568722	17.19027	1.565453	0.685395	1.704645	0.085935	0.611933	0.35898	531.4491	3.121668	530.0297	7.038982	524.8563	34.88758	531.4	3.1	101.2561	207
19AR115_Spot 101	481.961	32269.42	2.174072	17.02885	1.851122	0.692725	2.058513	0.085965	0.829806	0.40311	531.6321	4.234509	534.4365	8.553981	547.3539	41.16874	531.6	4.2	97.12767	90
19AR115_Spot 406	297.8285	25045.11	2.886646	17.76101	2.425821	0.663241	2.694309	0.085996	0.777826	0.288692	531.811	3.970535	516.5945	10.90963	450.7864	57.29775	531.8	4.0	117.9741	374
19AR115_Spot 410	559.8043	86285.54	4.602912	17.35381	1.228357	0.6826	1.422459	0.086005	0.684146	0.48096	531.8639	3.492663	528.3444	5.859488	514.1086	27.40631	531.9	3.5	103.4536	378
19AR115_Spot 137	250.7797	41057.49	3.764012	16.76662	2.399845	0.705436	2.527752	0.086013	0.746356	0.295265	531.9145	3.810601	542.0329	10.61702	585.7819	52.42407	531.9	3.8	90.80419	125
19AR115_Spot 144	383.8299	44098.7	2.900573	17.65695	2.076021	0.668455	2.174341	0.086034	0.606753	0.279051	532.0389	3.098537	519.7723	8.845574	467.1801	46.26507	532.0	3.1	113.883	131
19AR115_Spot 295	214.5137	20218.06	3.741809	17.21502	2.513136	0.680116	2.684444	0.086036	0.754045	0.280894	532.0481	3.850787	526.8444	11.03431	505.3116	56.69114	532.0	3.9	105.2911	269
19AR115_Spot 32	306.3156	40823.8	2.010225	17.34077	1.791046	0.682886	1.952082	0.08604	0.730385	0.374157	532.0758	3.730145	528.5171	8.043228	514.1145	39.76984	532.1	3.7	103.4936	31
19AR115_Spot 309	334.6712	41053.6	4.071694	17.11876	1.402371	0.68962	1.534362	0.086044	0.577671	0.37649	532.0967	2.950334	532.572	6.358934	535.5623	31.13595	532.1	3.0	99.35291	281
19AR115_Spot 401	92.4773	7010.272	1.677654	15.98511	4.801313	0.720465	5.308054	0.086046	1.010725	0.190413	532.1126	5.162209	550.9414	22.57376	630.522	112.3171	532.1	5.2	84.3924	369
19AR115_Spot 490	526.8513	63599.9	46.77012	17.16065	1.287948	0.688928	1.515114	0.086105	0.751737	0.496159	532.4619	3.841865	532.1561	6.275429	531.8201	28.81893	532.5	3.8	100.1207	446
19AR115_Spot 146	100.783	15553.11	1.44258	15.88498	5.457502	0.736414	5.56758	0.086108	0.810443	0.145565	532.4771	4.142002	560.3106	23.97981	675.9501	117.837	532.5	4.1	78.77461	133
19AR115_Spot 488	281.5841	41733.29	4.237779	16.97774	1.657023	0.696104	1.873777	0.086205	0.717741	0.383045	533.0514	3.672018	536.4613	7.808689	551.9146	37.78158	533.1	3.7	96.58223	444
19AR115_Spot 149	454.9161	46490.85	4.252655	16.94422	1.749532	0.697351	1.895939	0.086319	0.714037	0.376614	533.7296	3.657523	537.2075	7.909386	552.9362	38.33548	533.7	3.7	96.52643	136
19AR115_Spot 427	402.1361	54807.68	3.610428	17.47198	1.313822	0.680094	1.602412	0.086348	0.792407	0.494509	533.9053	4.060243	526.8315	6.586363	497.2371	30.70682	533.9	4.1	107.3744	392
19AR115_Spot 27	444.4735	59065.53	4.489778	17.32113	1.268197	0.68707	1.492871	0.086447	0.71211	0.477007	534.4885	3.65263	531.0384	6.173414	517.1743	28.78878	534.5	3.7	103.3478	26
19AR115_Spot 92	143.4187	8330.069	1.147092	16.12427	3.336123	0.721208	3.846653	0.086472	0.964796	0.250814	534.6352	4.950036	551.3799	16.36733	622.0793	80.33117	534.6	5.0	85.94327	83
19AR115_Spot 494	276.626	29658.42	2.152297	17.07976	2.303191	0.691615	2.486367	0.086504	0.796841	0.320484	534.8285	4.089732	533.7705	10.32222	530.2262	51.62164	534.8	4.1	100.868	450
19AR115_Spot 178	386.6302	33750.02	1.663081	17.08428	1.945606	0.692115	2.13713	0.086565	0.677579	0.317051	535.1882	3.479873	534.0704	8.87606	530.2732	44.42374	535.2	3.5	100.9269	162
19AR115_Spot 28	472.6626	40448.58	7.173904	17.11531	1.441153	0.696114	1.617831	0.086649	0.706936	0.436965	535.6907	3.633911	536.4673	6.742095	540.7097	31.84436	535.7	3.6	99.07177	27
19AR115_Spot 407	242.7099	27502.44	2.568154	17.06347	2.371152	0.696535	2.56573	0.086713	0.725694	0.282841	536.0683	3.73286	536.7194	10.69639	540.4245	53.81214	536.1	3.7	99.19394	375
19AR115_Spot 489	451.5772	34569.12	3.726278	17.28105	1.858097	0.687418	2.075972	0.08675	0.773767	0.372725	536.2865	3.981691	531.2481	8.587368	510.62	42.34067	536.3	4.0	105.0265	445
19AR115_Spot 65	301.3508	40819.95	2.614834	16.96813	1.856564	0.702584	2.008208	0.086753	0.757558	0.377231	536.3052	3.898414	540.3334	8.414697	558.3169	40.55363	536.3	3.9	96.0575	59
19AR115_Spot 366	187.6685	18002.84	2.271269	17.01937	3.509009	0.692483	3.822021	0.086799	0.757674	0.198239	536.5784	3.900916	534.2911	15.87973	525.4714	82.16167	536.6	3.9	102.1137	334
19AR115_Spot 259	368.2782	45569.29	6.885642	17.23378	1.917638	0.690171	2.101605	0.086827	0.70578	0.335829	536.7427	3.634801	532.903	8.714001	517.4318	43.45297	536.7	3.6	103.7321	237
19AR115_Spot 329	95.34678	13845.61	1.522739	15.55319	4.706327	0.756602	4.980899	0.086853	1.145555	0.22999	536.8992	5.901317	572.0476	21.78705	715.218	103.0433	536.9	5.9	75.06792	299
19AR115_Spot 164	275.7097	25595.43	7.407091	17.44132	2.007376	0.677968	2.207298	0.086893	0.880751	0.399018	537.1375	4.539113	525.5455	9.055822	476.4721	44.75466	537.1	4.5	112.7322	151
19AR115_Spot 421	81.97426	13831.15	1.351753	14.84837	6.511513	0.797211	6.603236	0.086914	1.026591	0.155468	537.2589	5.29187	595.2539	29.74987	823.8587	136.303	537.3	5.3	65.21251	386
19AR115_Spot 233	93.36176	9383.303	1.958846	16.28009	4.467932	0.716597	4.683516	0.087044	0.935881	0.199824	538.0335	4.830949	548.6562	19.85473	593.9619	99.51367	538.0	4.8	90.58383	214
19AR115_Spot 68	284.5247	16013.68	2.563228	16.71456	2.535313	0.708267	2.732012	0.087062	0.808755	0.296029	538.1403	4.175527	543.7164	11.50197	568.1282	56.81323	538.1	4.2	94.72163	62
19AR115_Spot 283	219.0485	16205.52	2.888252	16.90088	2.447725	0.699293	2.926649	0.087085	0.68398	0.233708	538.2737	3.532165	538.3684	12.2296	539.7424	62.25162	538.3	3.5	99.7279	260
19AR115_Spot 279	57.2296	5440.652	1.887233	12.6095	3.50138	0.922155	4.959874	0.087118	1.448771	0.292098	538.4686	7.484251	663.499	24.16563	1116.128	94.72879	538.5	7.5	48.24433	257
19AR115_Spot 357	131.3036	17949.38	2.272101	17.41873	3.951682	0.680041	4.034406	0.087194	0.740959	0.18366	538.9195	3.830813	526.7993	16.58301	475.5605	87.72836	538.9	3.8	113.323	326
19AR115_Spot 143	148.0447	11223.95	1.935098	17.10707	3.599755	0.688438	3.951855	0.087202	0.675808	0.17101	538.9709	3.4943	531.8614	16.36244	502.4506	85.73547	539.0	3.5	107.2684	130
19AR115_Spot 55	282.1467	33978.99	5.02295	16.98133	2.795539	0.70654	2.948762	0.087211	0.71822	0.243567	539.0231	3.713939	542.6899	12.39686	559.0907	62.33878	539.0	3.7	96.41067	50
19AR115_Spot 118	112.6545	18617.37	1.931454	14.94603	5.722165	0.798735	5.80905	0.087301	0.842051	0.144955	539.5548	4.358389	596.115	26.19793	818.5653	120.1864	539.6	4.4	65.91469	107
19AR115_Spot 347	224.7059	28290.96	1.266191	17.17873	2.940862	0.694887	3.110166	0.087371	0.766253	0.24637	539.9696	3.968986	535.7323	12.94823	518.6663	66.20008	540.0	4.0	104.1073	316
19AR115_Spot 448	295.5091	13791.79	1.955742	16.89906	2.723083	0.703589	3.059833	0.087426	0.976069	0.318994	540.2969	5.058717	540.9325	12.83231	544.5432	63.36826	540.3	5.1	99.22021	409
19AR115_Spot 131	199.5615	16018.62	1.963012	17.46808	3.111103	0.682975	3.335623	0.08751	0.78253	0.234598	540.7919	4.059215	528.5709	13.7455	477.0823	71.71219	540.8	4.1	113.354	120
19AR115_Spot 64	183.6574	25445.74	1.974265	17.59871	2.630942	0.680657	2.969742	0.087524	1.036844	0.349136	540.8757	5.379216	527.1714	12.2129	469.2034	61.63917	540.9	5.4	115.2753	58
19AR115_Spot 63	241.0943	30268.72	3.400297	17.35532	2.213287	0.691884	2.391731	0.087584	0.837446	0.350142	541.2326	4.347475	533.9315	9.931587	503.8224	49.30982	541.2	4.3	107.4253	57
19AR115_Spot 457	125.0025	12321.43	1.403148	15.1204	6.719374	0.785037	6.796535	0.087745	0.961227	0.141429	542.1848	4.998478	588.3529	30.35917	771.6227	141.7654	542.2	5.0	70.26552	418
19AR115_Spot 231	336.268	38104.72	3.034598	17.55207	2.193204	0.683948	2.309458	0.087831	0.588377	0.254769	542.6991	3.062405	529.1577	9.524606	472.1286	49.41971	542.7	3.1	114.9473	212
19AR115_Spot 106	190.9765	21419.22	3.545209	17.34088	2.714089	0.695099	2.844146	0.087957	0.729275	0.256413	543.4412	3.800725	535.8597	11.84277	504.6883	60.4989	543.4	3.8	107.6786	95
19AR115_Spot 56	211.8558	19745.48	1.988453	16.91521	2.488709	0.712336	2.629294	0.088044	0.783754	0.298085	543.9561	4.088363	546.1321	11.10662	556.1916	54.73679	544.0	4.1	97.80013	51
19AR115_Spot 496	389.5585	41545.65	5.627241	17.36452	1.609389	0.694922	1.791382	0.088163	0.774283	0.432227	544.6661	4.044011	535.7531	7.457822	498.9227	35.5724	544.7	4.0	109.1684	451
19AR115_Spot 441	440.0119	75933.1	1.854107	17.0896	1.520203	0.72766	1.658408	0.090078	0.659658	0.397766	556.0007	3.513984	555.1791	7.092491	552.7496	33.22965	556.0	3.5	100.5882	403
19AR115_Spot 228	226.1641	25780.63	2.787057	16.43744	3.553812	0.75205	3.689801	0.090618	0.65395	0.177232	559.1924	3.502719	569.4133	16.08309	611.3605	78.50115	559.2	3.5	91.46687	209
19AR115_Spot 216	273.1824	35908.14	1.694491	17.00009	2.151317	0.736129	2.355337	0.091408	0.915599	0.388734	563.8571	4.943327	560.1441	10.14073	546.0168	47.43945	563.9	4.9	103.2674	200
19AR115_Spot 79	382.6414	59498.53	1.144374	16.82248	1.435126	0.746371	1.638872	0.091435	0.779361	0.475547	564.0196	4.208938	566.1165	7.112139	575.5121	31.33821	564.0	4.2	98.00307	71
19AR115_Spot 78	969.8104	14808.31	8.725537	16.52894	0.740652	0.766368	1.045091	0.093366	0.6899	0.660133	575.4113	3.797745	577.6773	4.604086	587.5464	17.01906	575.4	3.8	97.93462	70
19AR115_Spot 336	251.2967	20789.97	2.112398	13.54496	2.125237	0.96624	2.466652	0.095953	1.227027	0.497447	590.6502	6.925331	686.524	12.30857	1015.753	43.35633	590.7	6.9	58.14901	305
19AR115_Spot 81	281.1921	28075.67	3.122058	16.59842	1.616682	0.796182	1.777571	0.096672	0.69654	0.391849	594.8742	3.958103	594.6729	8.000702	594.8528	35.45614	594.9	4.0	100.0036	73
19AR115_Spot 345	284.6646	28066.78	4.825417	16.36437	1.501106	0.819888	1.670032	0.098163	0.651007	0.389817	603.6348	3.75133	607.9861	7.639637	625.1541	33.15193	603.6	3.8	96.55776	314
19AR115_Spot 234	620.3978	49794.36	2.640906	16.46436	1.101813	0.821572	1.285324	0.098942	0.545794	0.424635	608.2032	3.16775	608.9252	5.886357	612.5329	25.15511	608.2	3.2	99.29314	215
19AR115_Spot 191	647.2247	72957.11	1.368129	16.70575	0.894715	0.814336	1.090587	0.099115	0.618404	0.567037	609.2186	3.594889	604.8834	4.970267	589.639	19.46259	609.2	3.6	103.3206	175
19AR115_Spot 455	431.155	71033.52	2.402209	16.82538	1.738911	0.812256	1.865055	0.099401	0.650251	0.34865	610.8957	3.789943	603.7186	8.487993	577.8304	37.96926	610.9	3.8	105.7223	416
19AR115_Spot 444	244.1385	21982.68	2.814218	16.55741	2.399722	0.822914	2.516905	0.099491	0.678402	0.269538	611.4226	3.957268	609.6727	11.53729	604.0964	52.45492	611.4	4.0	101.2128	405
19AR115_Spot 426	451.4109	48810.44	87.82942	16.8125	1.400094	0.814444	1.67187	0.099539	0.765306	0.457755	611.7066	4.466178	604.9442	7.620057	580.6394	32.268	611.7	4.5	105.3505	391
19AR115_Spot 200	229.886	25648.46	4.452852	16.20831	2.124139	0.840941	2.226183	0.099693	0.646787	0.290537	612.612	3.779851	619.6649	10.32598	646.4666	45.77028	612.6	3.8	94.76313	184
19AR115_Spot 481	310.9901	49844.46	1.80498	16.53232	1.651745	0.827932	1.803539	0.099737	0.650107	0.360462	612.8666	3.800759	612.4639	8.294678	611.8946	36.35303	612.9	3.8	100.1589	438
19AR115_Spot 51	394.8417	8039.307	1.683743	15.89141	1.485253	0.850491	1.680316	0.100237	0.703572	0.418714	615.7952	4.132061	624.9185	7.841735	659.0391	32.73026	615.8	4.1	93.43834	47
19AR115_Spot 266	333.2953	37235.7	0.962278	16.57371	1.721271	0.825932	1.870557	0.100268	0.662006	0.353908	615.9767	3.889038	611.3525	8.59154	595.1786	37.93208	616.0	3.9	103.4944	244
19AR115_Spot 155	347.5412	42231.2	4.344248	16.54675	1.396548	0.827911	1.574956	0.100268	0.619814	0.393543	615.9812	3.641199	612.4523	7.243262	600.3451	31.34764	616.0	3.6	102.6045	142
19AR115_Spot 49	352.737	32981.57	2.713214	16.36431	1.560782	0.844493	1.728909	0.100316	0.691396	0.399903	616.2624	4.063488	621.6222	8.037669	642.1386	34.07409	616.3	4.1	95.9703	45
19AR115_Spot 76	124.0943	17666.7	1.814365	16.35804	3.608913	0.842183	3.940774	0.101339	0.996768	0.252937	622.2523	5.912466	620.3496	18.29499	614.3333	82.37357	622.3	5.9	101.2891	69
19AR115_Spot 367	272.3453	47569.07	4.084329	16.80747	1.976612	0.832617	2.132762	0.102173	0.752302	0.352736	627.1327	4.49571	615.0632	9.839193	571.8241	43.39637	627.1	4.5	109.6723	335
19AR115_Spot 397	114.692	13031.4	3.61953	15.12096	3.950963	0.921578	4.244099	0.10252	0.806566	0.190044	629.1627	4.834839	663.1942	20.67043	781.5613	87.61706	629.2	4.8	80.50074	365
19AR115_Spot 315	221.6081	39404	2.78233	16.47728	2.005875	0.854485	2.13956	0.102619	0.712707	0.333109	629.737	4.275929	627.1078	10.01036	618.556	43.52882	629.7	4.3	101.8076	287
19AR115_Spot 250	319.1632	19951.78	1.359517	16.30462	1.344671	0.857779	1.718409	0.102881	0.6282	0.365571	631.2714	3.777663	628.9094	8.056508	621.3437	34.51812	631.3	3.8	101.5978	231
19AR115_Spot 179	404.4936	42543.68	4.099596	16.31194	1.613762	0.863938	1.779533	0.102946	0.71772	0.403319	631.6493	4.318448	632.2706	8.375234	635.4496	35.05414	631.6	4.3	99.40194	163
19AR115_Spot 116	237.8318	36013.98	3.620906	16.46264	2.31777	0.861816	2.447058	0.103244	0.752542	0.307529	633.3941	4.539873	631.1135	11.50192	623.8697	50.22422	633.4	4.5	101.5267	105
19AR115_Spot 480	307.4306	59961.11	2.260518	16.64013	1.74926	0.853399	1.899541	0.103394	0.644129	0.339097	634.268	3.890947	626.513	8.881223	599.5764	38.70468	634.3	3.9	105.786	437
19AR115_Spot 20	189.3192	34570.56	2.960071	16.25429	2.72734	0.875054	2.865322	0.10373	0.853178	0.29776	636.2314	5.168919	638.3079	13.57845	646.6211	58.76601	636.2	5.2	98.39322	20
19AR115_Spot 142	244.7343	41165.21	3.717447	16.47243	2.13611	0.865609	2.231197	0.10384	0.632719	0.283578	636.8708	3.836948	633.1803	10.51198	620.9452	46.1739	636.9	3.8	102.5647	129
19AR115_Spot 232	254.4769	35588.54	3.668047	16.23065	1.93693	0.881854	2.027689	0.104709	0.54444	0.268503	641.9473	3.326632	641.9835	9.648396	643.0674	42.01124	641.9	3.3	99.82582	213
19AR115_Spot 202	902.6969	101602.9	6.367102	16.11738	0.665412	0.91782	0.910535	0.107506	0.59215	0.650332	658.2495	3.705425	661.2064	4.424651	672.2556	14.80974	658.2	3.7	97.91655	186
19AR115_Spot 483	954.1833	6304.589	11.15693	14.6217	0.784059	0.981595	1.207531	0.10766	0.852659	0.706117	659.1413	5.342442	694.4225	6.073667	811.3272	17.90322	659.1	5.3	81.24235	440
19AR115_Spot 332	271.6148	32836.9	4.005094	15.55285	1.760624	0.962425	1.96536	0.109435	0.690459	0.351314	669.4635	4.390449	684.5517	9.787214	735.4133	38.96194	669.5	4.4	91.03229	302
19AR115_Spot 413	895.4053	128063.5	1.047435	15.96491	0.906685	0.951386	1.14177	0.110023	0.693089	0.607031	672.8818	4.428525	678.824	5.652317	699.4881	19.32334	672.9	4.4	96.19631	381
19AR115_Spot 61	402.8552	55984.73	4.247008	16.42238	1.617481	0.924229	1.764071	0.110252	0.699829	0.396712	674.2123	4.479974	664.5938	8.603576	633.0623	34.85292	674.2	4.5	106.5001	55
19AR115_Spot 267	255.0549	38578.05	1.129477	15.15858	1.595777	1.008001	1.827281	0.111833	0.823965	0.450924	683.382	5.34264	707.8639	9.314179	787.2241	34.26558	683.4	5.3	86.80908	245
19AR115_Spot 310	211.4983	35525.66	1.037431	16.02403	1.841025	0.966203	1.95791	0.112896	0.642812	0.328315	689.5439	4.20364	686.5046	9.769584	677.4676	39.51139	689.5	4.2	101.7826	282
19AR115_Spot 460	278.8407	46383.72	1.302316	15.70202	1.379086	0.987235	1.596704	0.112912	0.69305	0.43405	689.636	4.532744	697.3084	8.05443	723.0227	30.51864	689.6	4.5	95.38235	421
19AR115_Spot 447	244.3308	35553.76	2.722652	16.13498	2.015806	0.977526	2.131276	0.114662	0.665718	0.312357	699.7657	4.414535	692.3353	10.69773	669.1865	43.33766	699.8	4.4	104.5696	408
19AR115_Spot 15	744.9668	54500.51	52.16332	15.29933	0.817273	1.06092	1.28213	0.118046	0.939805	0.733003	719.3078	6.396582	734.2769	6.701775	781.1222	18.32911	719.3	6.4	92.08646	15
19AR115_Spot 82	242.6318	27104.19	8.081586	15.34668	2.086149	1.054564	2.275443	0.118338	0.846991	0.372231	720.9929	5.77763	731.1402	11.85957	763.2686	44.5172	721.0	5.8	94.46124	74
19AR115_Spot 103	57.89687	9997.496	2.612927	14.57724	6.061906	1.169771	6.256658	0.125593	1.100326	0.175865	762.6759	7.914514	786.5378	34.2629	855.7192	128.0079	762.7	7.9	89.12689	92
19AR115_Spot 437	522.6113	47619.53	4.784377	14.1659	0.803288	1.225625	1.082691	0.125725	0.660906	0.610429	763.432	4.758258	812.345	6.05404	949.7056	17.53638	763.4	4.8	80.38617	400
19AR115_Spot 197	325.0164	70480.76	5.794714	14.69633	1.089695	1.206476	1.31011	0.128932	0.719658	0.549311	781.7703	5.298317	803.5708	7.273856	865.4078	22.69121	781.8	5.3	90.33548	181
19AR115_Spot 18	211.8644	40236.65	1.775688	15.33966	2.019156	1.154464	2.159772	0.129063	0.74877	0.346689	782.5174	5.517598	779.3493	11.75164	771.1834	42.64702	782.5	5.5	101.4697	18
19AR115_Spot 301	388.0673	31479.51	1.858627	14.64617	1.405054	1.206247	1.698901	0.129211	0.767175	0.451571	783.3619	5.658961	803.4653	9.431754	860.4934	31.44036	783.4	5.7	91.03636	275
19AR115_Spot 66	331.9448	42961.92	4.633165	14.5978	1.492341	1.234546	1.780216	0.13099	0.938928	0.527424	793.512	7.010205	816.4064	9.986988	880.2014	31.29163	793.5	7.0	90.15119	60
19AR115_Spot 136	114.2611	22704.56	3.32543	14.65792	2.063653	1.256199	2.428645	0.134354	1.258605	0.518233	812.6573	9.609714	826.1982	13.73098	863.7328	43.06759	812.7	9.6	94.08665	124
19AR115_Spot 306	618.8451	22758.21	1.35014	14.14197	0.770007	1.323761	1.083823	0.13694	0.709757	0.654864	827.3334	5.510862	856.1576	6.269195	932.4581	16.79861	827.3	5.5	88.72607	279
19AR115_Spot 285	309.1499	56749.82	2.02315	14.28569	1.16949	1.368907	1.355133	0.142574	0.627992	0.463417	859.1998	5.051588	875.6954	7.951457	918.5511	24.69309	859.2	5.1	93.53859	262
19AR115_Spot 37	62.09714	11725.87	3.702364	14.46559	4.643603	1.498906	4.855457	0.159114	0.966039	0.19896	951.8494	8.548595	929.9417	29.58062	879.2772	98.50849	879.3	98.5	108.2536	36
19AR115_Spot 35	127.4261	21088.65	1.219365	14.48092	2.502505	1.395523	2.68225	0.147122	0.835161	0.311366	884.8107	6.904894	887.0401	15.86723	893.5009	52.62907	884.8	6.9	99.0274	34
19AR115_Spot 470	94.62799	19748.09	2.4171	14.29971	3.405156	1.436197	3.59105	0.150719	1.047411	0.291673	904.9898	8.843681	904.136	21.49898	902.9272	70.81764	902.9	70.8	100.2284	429
19AR115_Spot 120	253.8749	60305.83	1.401357	14.43639	1.515401	1.479857	1.674026	0.154835	0.699326	0.417751	928.0082	6.044312	922.1719	10.14378	909.1151	31.34173	909.1	31.3	102.0782	109
19AR115_Spot 402	210.2767	44176.13	1.877913	14.40695	1.445641	1.518998	1.650678	0.158686	0.778039	0.471345	949.4679	6.868964	938.0729	10.10732	912.2843	29.96214	912.3	30.0	104.0759	370
19AR115_Spot 152	76.58485	12994.2	1.778363	14.06916	3.043749	1.450652	3.408418	0.150898	0.823928	0.241733	905.9933	6.963911	910.1427	20.48912	921.136	67.99997	921.1	68.0	98.35608	139
19AR115_Spot 134	56.33113	11984.18	1.998403	14.08691	6.171362	1.54094	6.431218	0.159603	0.831979	0.129366	954.5723	7.38182	946.8793	39.62185	929.9129	131.0576	929.9	131.1	102.6518	122
19AR115_Spot 16	305.6881	42017.9	2.993527	14.16802	0.987029	1.499699	1.590814	0.15465	1.234837	0.776229	926.977	10.66173	930.264	9.691231	938.9342	20.54067	938.9	20.5	98.72651	16
19AR115_Spot 30	63.43812	16607.37	1.766128	14.10377	2.917211	1.611514	3.295203	0.166137	1.472295	0.446799	990.7933	13.52169	974.6969	20.6497	939.4658	60.42102	939.5	60.4	105.4635	29
19AR115_Spot 268	553.6451	113861.1	7.256032	14.15859	0.695794	1.430173	1.059543	0.147418	0.791347	0.746876	886.471	6.554097	901.622	6.331484	939.8087	14.42435	939.8	14.4	94.32462	246
19AR115_Spot 466	29.80269	7432.105	3.438777	13.75025	6.66029	1.69437	7.120832	0.173452	1.663197	0.233568	1031.102	15.84807	1006.412	45.49904	953.9044	141.7561	953.9	141.8	108.0928	425
19AR115_Spot 334	297.1246	59693.12	1.9674	14.02588	1.19176	1.388638	1.355143	0.141947	0.577277	0.42599	855.6624	4.625757	884.1176	7.999496	956.8877	25.06497	956.9	25.1	89.4214	303
19AR115_Spot 428	173.5062	38657.27	3.114882	14.05251	1.998022	1.660466	2.127797	0.169451	0.709067	0.33324	1009.083	6.623179	993.5538	13.48522	960.3351	40.97545	960.3	41.0	105.0761	393
19AR115_Spot 381	558.5838	88828.68	2.520665	13.99586	0.809502	1.537192	1.107741	0.156545	0.727647	0.656874	937.5469	6.349142	945.3804	6.814743	964.5772	17.0742	964.6	17.1	97.19771	349
19AR115_Spot 90	140.0131	26202.06	1.78021	13.9732	1.944854	1.428513	2.203137	0.145374	0.92827	0.42134	874.9813	7.595087	900.9281	13.1595	966.0207	40.78484	966.0	40.8	90.57584	81
19AR115_Spot 260	242.5432	66594.34	1.74253	13.95294	1.355872	1.500426	1.582483	0.152442	0.771732	0.487671	914.6379	6.580675	930.5589	9.642343	969.372	28.17084	969.4	28.2	94.35366	238
19AR115_Spot 385	150.9183	29487.23	2.57604	13.89947	2.330808	1.641766	2.460097	0.166655	0.578629	0.235206	993.657	5.328383	986.3916	15.52505	971.1379	48.76144	971.1	48.8	102.3188	353
19AR115_Spot 48	494.7978	28669.56	2.055409	13.95206	0.802049	1.434106	1.274357	0.145161	0.981229	0.76998	873.7787	8.018098	903.2638	7.623791	976.965	16.55344	977.0	16.6	89.43808	44
19AR115_Spot 459	182.6356	46018.2	2.768925	13.89888	1.494039	1.687282	1.652856	0.170719	0.664697	0.402151	1016.069	6.248421	1003.737	10.53792	977.7949	30.81976	977.8	30.8	103.9143	420
19AR115_Spot 62	258.984	47068.37	3.097352	13.92665	1.369549	1.574804	1.583763	0.159106	0.770332	0.486393	951.8077	6.816477	960.3224	9.835947	980.7404	28.17634	980.7	28.2	97.04991	56
19AR115_Spot 451	102.3916	23345.76	2.139162	13.8281	2.177409	1.725232	2.339481	0.174176	0.700671	0.299499	1035.077	6.700193	1017.976	15.03922	982.2814	45.45966	982.3	45.5	105.3748	412
19AR115_Spot 368	190.941	29890.11	1.82039	13.80637	1.676898	1.626751	1.952148	0.164136	0.938471	0.480738	979.7191	8.529803	980.6038	12.27625	983.4917	34.83457	983.5	34.8	99.61641	336
19AR115_Spot 350	173.6944	37979.22	2.903085	13.79946	1.310677	1.68279	1.460742	0.169341	0.625909	0.428487	1008.479	5.843197	1002.038	9.303765	988.8583	26.83097	988.9	26.8	101.9842	319
19AR115_Spot 422	422.1889	71854.1	5.878649	13.88057	0.909588	1.584217	1.123121	0.159406	0.651437	0.580024	953.4764	5.773784	964.0276	6.991154	989.0413	18.59233	989.0	18.6	96.40411	387
19AR115_Spot 284	199.5925	60729.68	3.691631	13.7959	1.065387	1.63338	1.236219	0.164227	0.620846	0.502214	980.2237	5.645581	983.1631	7.785875	990.6378	21.7417	990.6	21.7	98.94874	261
19AR115_Spot 491	367.548	79909.17	4.654686	13.8162	1.176672	1.671754	1.449291	0.167984	0.811741	0.560095	1000.995	7.526056	997.8526	9.208171	991.8402	24.39478	991.8	24.4	100.923	447
19AR115_Spot 213	426.4701	89988.01	1.632784	13.80567	0.915381	1.619232	1.134836	0.162518	0.657596	0.579463	970.7541	5.926235	977.6931	7.123696	994.2156	18.7927	994.2	18.8	97.6402	197
19AR115_Spot 379	561.259	5268.091	2.949932	13.29066	0.802752	1.326798	1.411796	0.132796	1.122347	0.794978	803.7967	8.481624	857.4838	8.174429	999.8464	17.3751	999.8	17.4	80.39201	347
19AR115_Spot 369	158.0287	45845	3.289062	13.71371	1.817418	1.704689	1.987427	0.170536	0.775072	0.389987	1015.061	7.279315	1010.293	12.71955	1000.834	37.1455	1000.8	37.1	101.4216	337
19AR115_Spot 174	231.4644	42392.79	2.679262	13.66164	1.258872	1.716533	1.450636	0.171637	0.667818	0.460363	1021.122	6.306578	1014.73	9.3076	1001.825	26.14703	1001.8	26.1	101.9262	159
19AR115_Spot 487	444.8818	50999.98	8.942091	13.70656	0.66022	1.64886	0.896135	0.164783	0.592957	0.661682	983.3029	5.407653	989.1145	5.664121	1002.893	13.63895	1002.9	13.6	98.04659	443
19AR115_Spot 210	193.7881	32831.98	2.508457	13.68074	1.302264	1.707717	1.490406	0.170392	0.649259	0.435626	1014.268	6.09331	1011.429	9.544648	1006.15	27.22047	1006.2	27.2	100.8068	194
19AR115_Spot 429	329.6134	88678.75	6.298363	13.79691	1.030095	1.726445	1.252327	0.172201	0.708917	0.56608	1024.229	6.713488	1018.428	8.052165	1006.841	20.93691	1006.8	20.9	101.727	394

19AR115_Spot 10	191.0243	60506.72	1.764486	13.74835	1.316535	1.655793	1.594079	0.16505	0.86032	0.539697	984.7787	7.856853	991.7684	10.09173	1008.126	27.20134	1008.1	27.2	97.68413	10
19AR115_Spot 258	769.6519	37165.01	1.808929	13.65725	0.670383	1.575396	1.039335	0.15702	0.79116	0.761218	940.1975	6.921466	960.5558	6.455606	1008.324	13.66206	1008.3	13.7	93.24361	236
19AR115_Spot 500	197.049	43681.78	1.393865	13.63965	1.661439	1.726256	1.960124	0.171932	1.019545	0.520143	1022.75	9.642287	1018.357	12.60301	1009.79	33.92983	1009.8	33.9	101.2834	454
19AR115_Spot 170	359.5837	20680.99	1.602358	13.54672	0.96684	1.647053	1.332349	0.163983	0.579783	0.435159	978.8743	5.26546	988.4215	8.417881	1010.542	24.32358	1010.5	24.3	96.86622	156
19AR115_Spot 67	387.6081	90484.22	4.027236	13.725	0.89046	1.666092	1.141394	0.165758	0.711542	0.623397	988.6989	6.522077	995.6985	7.242639	1012.011	18.07292	1012.0	18.1	97.6965	61
19AR115_Spot 212	112.7053	19809.47	2.248235	13.58329	2.461709	1.63953	2.628627	0.163111	0.705132	0.268251	974.0436	6.374578	985.5319	16.58022	1012.068	51.33963	1012.1	51.3	96.2429	196
19AR115_Spot 156	328.5677	51073.56	2.161797	13.5986	0.902328	1.770669	1.167674	0.175905	0.667405	0.571568	1044.563	6.435955	1034.766	7.577263	1014.983	19.4142	1015.0	19.4	102.9144	143
19AR115_Spot 375	237.7191	44818.7	2.170561	13.61454	1.35748	1.736279	1.576265	0.172453	0.75495	0.478948	1025.611	7.158323	1022.083	10.15624	1015.399	28.02506	1015.4	28.0	101.0058	343
19AR115_Spot 4	662.1883	20470.73	2.007124	13.61552	0.857164	1.551412	1.181046	0.154078	0.800412	0.677715	923.7817	6.888697	951.0554	7.292066	1015.575	17.59586	1015.6	17.6	90.96146	4
19AR115_Spot 328	173.03	35579.14	1.826273	13.61068	1.39529	1.730872	1.5273	0.171865	0.6013	0.393701	1022.378	5.68485	1020.075	9.8295	1015.998	28.43012	1016.0	28.4	100.628	298
19AR115_Spot 277	258.6787	33615.83	2.160995	13.5661	1.033846	1.726514	1.343158	0.17112	0.675048	0.502583	1018.282	6.358477	1018.453	8.63634	1019.686	23.4942	1019.7	23.5	99.86233	255
19AR115_Spot 275	96.12744	19933.56	3.255509	13.49673	2.261048	1.734496	2.485924	0.171705	0.866378	0.348514	1021.496	8.184452	1021.422	16.01215	1022.124	47.17512	1022.1	47.2	99.93851	253
19AR115_Spot 126	276.0902	44363.88	4.635616	13.64416	1.013882	1.746074	1.284802	0.172734	0.756522	0.588823	1027.155	7.183183	1025.712	8.29519	1023.498	21.01379	1023.5	21.0	100.3572	115
19AR115_Spot 339	98.44441	18931.15	1.478446	13.46818	1.810786	1.771287	2.090396	0.174951	0.716326	0.342675	1039.331	6.87583	1034.992	13.56725	1026.696	39.73654	1026.7	39.7	101.2307	308
19AR115_Spot 1	95.06147	16999.04	2.036964	13.51395	2.550246	1.80269	2.741434	0.177975	0.632627	0.230765	1055.904	6.161547	1046.433	17.906	1027.575	53.96626	1027.6	54.0	102.7569	1
19AR115_Spot 17	305.1032	15100.54	2.235863	13.45128	1.305602	1.609122	1.554791	0.158794	0.803136	0.516555	950.0686	7.094699	973.7665	9.736658	1028.482	26.9403	1028.5	26.9	92.37578	17
19AR115_Spot 272	418.5903	75023.17	1.734436	13.55488	0.814593	1.682041	1.051413	0.165937	0.64583	0.614249	989.6864	5.92522	1001.755	6.695465	1029.12	16.79437	1029.1	16.8	96.16825	250
19AR115_Spot 22	199.0372	49363.64	1.50895	13.56136	1.460402	1.815517	1.615658	0.179037	0.6863	0.42478	1061.714	6.71813	1051.07	10.57882	1029.879	29.59023	1029.9	29.6	103.0912	22
19AR115_Spot 199	708.9492	199557.5	2.587441	13.59537	0.570427	1.785176	0.803132	0.175947	0.562481	0.700359	1044.793	5.425247	1040.068	5.226961	1031.012	11.58782	1031.0	11.6	101.3366	183
19AR115_Spot 181	222.0598	23987.29	1.961738	13.45881	1.233881	1.706685	1.512053	0.168208	0.662404	0.438083	1002.228	6.148467	1011.042	9.681125	1031.049	27.49411	1031.0	27.5	97.20465	165
19AR115_Spot 195	293.6757	67298.37	2.50121	13.55283	0.919383	1.757625	1.13588	0.173195	0.635067	0.559097	1029.691	6.043705	1029.974	7.351249	1031.435	19.05342	1031.4	19.1	99.83096	179
19AR115_Spot 418	139.648	28967.18	1.583979	13.53639	1.668901	1.779511	1.801534	0.175248	0.625739	0.347336	1040.962	6.014989	1038.001	11.71181	1032.631	34.16003	1032.6	34.2	100.8068	384
19AR115_Spot 21	444.9753	120188.3	1.985343	13.5647	0.785475	1.794017	1.017579	0.176553	0.627641	0.616798	1048.114	6.071449	1043.286	6.634403	1034.049	16.2014	1034.0	16.2	101.3602	21
19AR115_Spot 360	332.2117	89268.39	2.030735	13.52602	0.884064	1.804951	1.09291	0.177555	0.628827	0.575369	1053.605	6.11226	1047.252	7.141045	1034.883	18.07631	1034.9	18.1	101.8091	329
19AR115_Spot 112	1048.951	22199.01	2.534796	13.49678	0.738314	1.675821	1.131063	0.164852	0.843622	0.745867	983.6829	7.696431	999.3972	7.192744	1034.894	15.23806	1034.9	15.2	95.05154	101
19AR115_Spot 335	763.6191	162367.1	2.905496	13.50638	0.625431	1.7555	0.890227	0.172426	0.62527	0.702371	1025.462	5.927919	1029.191	5.758852	1037.988	12.81384	1038.0	12.8	98.79325	304
19AR115_Spot 323	222.8327	32144.86	2.788362	13.4446	1.163229	1.787148	1.275692	0.175486	0.465109	0.364594	1042.269	4.476089	1040.787	8.305884	1038.537	24.00237	1038.5	24.0	100.3593	294
19AR115_Spot 351	596.0114	79797.82	2.908768	13.50114	0.679934	1.735385	0.916446	0.170383	0.579294	0.632109	1014.224	5.436463	1021.752	5.903628	1038.774	14.35607	1038.8	14.4	97.63662	320
19AR115_Spot 34	241.8629	68173.33	2.123199	13.57259	1.161559	1.757107	1.349768	0.172404	0.681744	0.505083	1025.341	6.46263	1029.783	8.73463	1040.091	23.53513	1040.1	23.5	98.58185	33
19AR115_Spot 439	576.9983	22867.42	2.772316	13.4629	0.900966	1.697985	1.077394	0.166442	0.587599	0.545389	992.4802	5.405057	1007.773	6.885024	1042.033	18.24194	1042.0	18.2	95.24462	402
19AR115_Spot 47	237.5802	41082.47	1.92251	13.52033	1.256549	1.747414	1.485718	0.171011	0.772875	0.520203	1017.678	7.275959	1026.207	9.59513	1045.334	25.61153	1045.3	25.6	97.3543	43
19AR115_Spot 493	515.7064	106629.7	1.794789	13.44308	1.040887	1.783923	1.342663	0.174471	0.845851	0.62998	1036.701	8.10017	1039.611	8.736279	1046.633	21.04707	1046.6	21.0	99.05102	449
19AR115_Spot 128	246.2774	64430.25	3.025442	13.51342	1.220297	1.812043	1.458861	0.177144	0.761172	0.521758	1051.351	7.384094	1049.816	9.545598	1047.52	25.11427	1047.5	25.1	100.3657	117
19AR115_Spot 411	423.4086	106607.9	2.178975	13.47817	0.744777	1.820938	1.019029	0.177991	0.686324	0.673508	1055.987	6.685021	1053.023	6.679199	1047.776	15.20511	1047.8	15.2	100.7837	379
19AR115_Spot 265	346.9269	80017.57	1.90852	13.38956	0.794301	1.796375	1.002765	0.175463	0.60671	0.605037	1042.142	5.838164	1044.143	6.540887	1049.23	16.11272	1049.2	16.1	99.32438	243
19AR115_Spot 262	118.0625	28518.47	2.482298	13.356	1.521485	1.789448	1.637318	0.174572	0.580431	0.354501	1037.252	5.561141	1041.624	10.66546	1051.704	30.85619	1051.7	30.9	98.62582	240
19AR115_Spot 492	358.775	70232.99	2.449208	13.39419	1.046605	1.855436	1.343118	0.180972	0.799227	0.595054	1072.282	7.895152	1065.365	8.861939	1052.124	21.76929	1052.1	21.8	101.916	448
19AR115_Spot 403	221.7548	42617.26	1.254666	13.41406	1.128695	1.810191	1.335623	0.176223	0.610984	0.457453	1046.307	5.900939	1049.147	8.736005	1055.96	23.91742	1056.0	23.9	99.08582	371
19AR115_Spot 482	213.7578	46146.3	2.295917	13.35455	1.261643	1.872075	1.424362	0.182045	0.611872	0.429576	1078.138	6.074693	1071.265	9.427367	1058.194	25.91171	1058.2	25.9	101.8847	439
19AR115_Spot 302	571.8332	116005.2	2.408549	13.36343	0.679444	1.820183	0.859537	0.176977	0.524525	0.610242	1050.436	5.08432	1052.751	5.632963	1058.45	13.72707	1058.4	13.7	99.24293	276
19AR115_Spot 356	139.2077	21274.3	2.236753	13.27596	1.338197	1.836256	1.49671	0.178529	0.589843	0.394093	1058.933	5.760006	1058.521	9.839407	1058.568	27.70438	1058.6	27.7	100.0345	325
19AR115_Spot 222	236.163	5652.481	2.726933	12.93418	1.347775	1.686077	1.497741	0.163766	0.598256	0.399439	977.6698	5.427035	1003.281	9.546373	1060.56	27.64369	1060.6	27.6	92.18432	203
19AR115_Spot 98	443.6691	132893.7	1.953683	13.39653	0.748949	1.849718	1.002421	0.179353	0.65568	0.654096	1063.441	6.427996	1063.33	6.606777	1063.996	15.24661	1064.0	15.2	99.94786	89
19AR115_Spot 419	207.9012	46033.77	3.140287	13.33997	1.087804	1.795105	1.287886	0.173829	0.642856	0.499156	1033.173	6.136903	1043.682	8.398639	1066.645	22.45596	1066.6	22.5	96.86195	385
19AR115_Spot 435	218.5304	72809.43	2.858462	13.38522	1.291105	1.856326	1.442726	0.179633	0.627312	0.43481	1064.969	6.158018	1065.681	9.520793	1068.035	26.13491	1068.0	26.1	99.71292	398
19AR115_Spot 52	248.7319	17188.2	1.688579	13.28723	1.116307	1.82128	1.581834	0.176173	0.695253	0.439523	1046.034	6.713203	1053.146	10.36899	1068.815	28.56775	1068.8	28.6	97.8686	48
19AR115_Spot 244	324.4677	96046.2	1.361222	13.28361	1.026417	1.864884	1.236232	0.180341	0.667905	0.540275	1068.837	6.578393	1068.719	8.171163	1069.371	20.88809	1069.4	20.9	99.9501	225
19AR115_Spot 331	503.9857	93901.5	2.889515	13.25276	0.813763	1.897166	1.0131	0.182931	0.599267	0.591518	1082.967	5.974019	1080.096	6.736286	1075.204	16.38353	1075.2	16.4	100.722	301
19AR115_Spot 271	136.5911	27434.81	1.283282	13.17579	2.223575	1.819943	2.35431	0.175266	0.657497	0.279274	1041.062	6.320831	1052.665	15.42924	1077.705	45.38935	1077.7	45.4	96.59997	249
19AR115_Spot 50	69.58114	16632.91	2.758752	13.21249	3.437287	1.782656	3.694829	0.171626	0.841361	0.227713	1021.063	7.945012	1039.149	24.03884	1078.284	72.24852	1078.3	72.2	94.69336	46
19AR115_Spot 346	387.8412	76223.62	2.37961	13.23574	0.902819	1.807257	1.086824	0.173962	0.591728	0.544456	1033.906	5.652513	1048.086	7.104506	1078.655	18.29248	1078.7	18.3	95.85141	315
19AR115_Spot 23	315.7386	67985.09	2.675678	13.17028	0.950633	1.921979	1.161732	0.183658	0.646437	0.556443	1086.927	6.465888	1088.756	7.759179	1093.269	19.31173	1093.3	19.3	99.41985	23
19AR115_Spot 338	343.2103	71549.86	1.032822	13.12527	1.077428	1.922302	1.273486	0.183682	0.666375	0.523268	1087.058	6.666045	1088.868	8.506104	1093.381	21.71357	1093.4	21.7	99.42166	307
19AR115_Spot 340	79.28009	17682.54	2.797957	13.00648	2.20545	1.811657	2.367147	0.172858	0.76657	0.323837	1027.841	7.28308	1049.677	15.48826	1096.251	44.81442	1096.3	44.8	93.7596	309
19AR115_Spot 432	214.8988	60311.21	2.859635	13.17231	1.16878	1.733877	1.387904	0.16526	0.704798	0.507815	985.9405	6.443585	1021.192	8.937993	1098.433	23.91479	1098.4	23.9	89.75878	396
19AR115_Spot 320	144.9561	37423.85	1.652283	13.04006	1.913806	1.841052	2.024153	0.175139	0.547852	0.270657	1040.364	5.263505	1060.237	13.31941	1102.262	38.95329	1102.3	39.0	94.38446	291
19AR115_Spot 280	212.7052	44259.54	4.907529	13.05311	1.026294	1.833012	1.236128	0.174096	0.65995	0.533885	1034.64	6.308333	1057.359	8.121186	1105.457	20.88407	1105.5	20.9	93.59388	258
19AR115_Spot 69	147.8592	30260.63	2.80814	12.95461	2.122046	1.942958	2.310426	0.183786	0.901644	0.39025	1087.622	9.023851	1096.02	15.48941	1113.587	42.45465	1113.6	42.5	97.66839	63
19AR115_Spot 80	419.0151	132336.3	2.671587	12.4983	0.68067	2.247677	0.948001	0.204009	0.649177	0.684784	1196.82	7.090888	1196.06	6.662023	1195.528	13.613	1195.5	13.6	100.1081	72
19AR115_Spot 479	511.9391	13867.93	2.409722	12.35288	0.68991	1.790994	1.020505	0.162479	0.738937	0.72409	970.5362	6.657896	1042.187	6.649458	1196.497	13.86924	1196.5	13.9	81.11484	436
19AR115_Spot 364	304.0744	89984.89	2.753712	12.24229	0.739308	2.271603	1.035219	0.20239	0.723607	0.69899	1188.145	7.851717	1203.513	7.298634	1232.073	14.52747	1232.1	14.5	96.43463	332
19AR115_Spot 44	96.00453	29829.51	3.311402	12.12177	1.837045	2.361402	1.974796	0.20748	0.654119	0.331234	1215.378	7.245554	1231.008	14.08736	1259.355	36.42489	1259.4	36.4	96.50799	42
19AR115_Spot 33	166.5084	59592.07	0.823245	12.08606	1.293667	2.498601	1.464737	0.218529	0.669698	0.457214	1274.095	7.742296	1271.628	10.62204	1268.292	25.42883	1268.3	25.4	100.4576	32
19AR115_Spot 454	56.37376	11775.65	1.5321	11.78559	4.264297	2.420304	4.450228	0.209705	0.886164	0.199128	1227.245	9.902884	1248.646	31.98608	1286.575	84.95335	1286.6	85.0	95.38846	415
19AR115_Spot 276	255.9427	45330.14	3.381026	11.80668	0.930023	2.249002	1.164494	0.193591	0.670176	0.575508	1140.796	7.007064	1196.474	8.18496	1299.313	18.50861	1299.3	18.5	87.79993	254
19AR115_Spot 246	741.6677	242506	5.089621	11.06573	0.636031	3.052277	0.913823	0.245844	0.655472	0.717285	1417.008	8.338131	1420.804	6.989141	1427.311	12.15754	1427.3	12.2	99.27812	227
19AR115_Spot 380	422.1359	86886.75	1.483311	11.04065	0.768207	2.670399	1.166789	0.214424	0.873886	0.748966	1252.347	9.946652	1320.303	8.619765	1433.19	14.75004	1433.2	14.8	87.38175	348
19AR115_Spot 393	177.8244	116189.1	0.848187	9.266739	0.607613	4.609975	0.847512	0.309832	0.583202	0.688134	1739.878	8.892989	1751.075	7.071645	1765.246	11.23705	1765.2	11.2	98.56289	361
19AR115_Spot 297	267.7556	98515.25	1.805793	8.973752	0.751818	4.458065	1.011613	0.290696	0.671959	0.664245	1645.004	9.756091	1723.201	8.389998	1820.318	13.72317	1820.3	13.7	90.36904	271
19AR115_Spot 172	124.46	43630.06	1.460343	8.696165	0.789325	5.285632	0.980557	0.335568	0.574339	0.585727	1865.311	9.302522	1866.544	8.372603	1868.687	14.34048	1868.7	14.3	99.81932	158
19AR115_Spot 168	111.7834	54764.05	1.30733	8.173985	0.709661	6.027877	0.98709	0.359244	0.68073	0.689633	1978.591	11.59808	1979.88	8.596812	1981.985	12.72604	1982.0	12.7	99.82876	154
19AR115_Spot 300	198.2523	27650.59	1.380043	8.051817	0.709403	5.634433	0.981383	0.330876	0.66012	0.672643	1842.625	10.5796	1921.382	8.463012	2008.222	12.88731	2008.2	12.9	91.75407	274
19AR115_Spot 240	100.8583	55989.61	0.542916	7.68919	0.980097	6.891297	1.198124	0.385996	0.681972	0.5692	2104.23	12.24351	2097.538	10.6243	2091.729	17.31901	2091.7	17.3	100.5977	221
19AR115_Spot 223	277.8638	120643.6	3.749245	7.508198	0.598121	7.064181	0.834346	0.385634	0.581041	0.696403	2102.549	10.42443	2119.543	7.421392	2136.817	10.47369	2136.8	10.5	98.39629	204
19AR115_Spot 296	209.7385	130045.1	1.873505	5.888023	0.652687	9.616842	0.943565	0.411178	0.681236	0.72198	2220.303	12.7957	2398.783	8.678599	2554.704	10.92909	2554.7	10.9	86.91037	270
19AR115_Spot 384	81.00461	46086.42	1.017868	5.555669	0.620745	12.47386	0.890806	0.503446	0.631325	0.708712	2628.591	13.62815	2640.759	8.373977	2650.804	10.42329	2650.8	10.4	99.16203	352
19AR115_Spot 187	112.5989	82530.73	1.703935	5.46977	0.677272	11.62689	1.241973	0.463488	1.038893	0.836486	2454.942	21.20997	2574.838	11.61257	2671.325	11.26911	2671.3	11.3	91.89976	171
19AR115_Spot 238	286.0275	188277.5	1.7167	5.440983	0.507246	11.91258	0.764805	0.471812	0.571971	0.747864	2491.504	11.81978	2597.555	7.164416	2682.039	8.394854	2682.0	8.4	92.89588	219
**19AR116**																				
19AR116_Spot 160	3640.71	771.5937	1.78406	6.966234	27.12517	0.002928	30.85264	0.000168	9.609463	0.311463	1.082108	0.103976	2.968709	0.914588	2050.611	530.138	1.1	0.1	0.05277	143
19AR116_Spot 406	1208.368	1170.464	2.236109	6.67615	18.27888	0.011538	19.18905	0.000602	5.426744	0.282804	3.876472	0.210303	11.64863	2.222491	2217.018	322.0342	3.9	0.2	0.174851	359
19AR116_Spot 450	1686.473	1818.799	8.364272	8.083354	11.75279	0.012452	12.83252	0.000778	3.792478	0.295537	5.013212	0.190051	12.56507	1.602481	1897.473	221.2862	5.0	0.2	0.264205	396
19AR116_Part2_Spot 5	433.9058	347.2162	0.78058	4.424254	28.35282	0.035065	29.79488	0.001325	7.472213	0.250788	8.533934	0.637252	34.99457	10.24931	2759.902	485.1319	8.5	0.6	0.309212	535
19AR116_Part2_Spot 218	638.1319	760.0334	1.002691	8.678025	17.33175	0.022611	25.20893	0.001691	5.462223	0.216678	10.88987	0.594327	22.70264	5.659643	1567.871	468.4228	10.9	0.6	0.694564	717
19AR116_Part2_Spot 272	1499.177	11456.17	1.02112	17.6766	4.001574	0.086334	4.092866	0.011398	0.646558	0.157972	73.06166	0.469719	84.08263	3.302765	410.5159	90.39799	73.1	0.5	17.79752	764
19AR116_Part2_Spot 243	230.8972	4632.758	1.325843	16.79125	3.500629	0.32772	4.805366	0.04235	0.907758	0.188905	267.3849	2.377558	287.8235	12.04406	458.217	104.71	267.4	2.4	58.35333	739
19AR116_Spot 311	53.08859	1430.044	0.861429	10.7967	11.90324	0.489406	13.65572	0.042804	2.364195	0.173129	270.1897	6.255799	404.5056	45.59239	1268.276	263.722	270.2	6.3	21.3037	277
19AR116_Spot 300	80.26386	6164.727	0.923811	13.71454	13.16428	0.422177	13.63414	0.043293	2.072991	0.152044	273.212	5.54532	357.6063	41.11834	950.3771	276.9466	273.2	5.5	28.74774	266
19AR116_Part2_Spot 372	297.4485	1046.804	2.69835	14.45429	3.870494	0.361594	6.338052	0.047455	2.118027	0.334176	298.8755	6.185761	313.4044	17.09231	423.8674	133.3943	298.9	6.2	70.51155	849
19AR116_Spot 104	100.3518	5414.989	1.982676	14.03947	5.959138	0.449842	6.789593	0.047532	2.249976	0.331386	299.3531	6.581382	377.1687	21.39327	888.7861	132.4773	299.4	6.6	33.68112	95
19AR116_Spot 408	671.5795	41105.9	2.492303	18.92868	1.833747	0.347228	1.990507	0.047937	0.736281	0.369896	301.841	2.171171	302.6337	5.209194	309.7425	42.1199	301.8	2.2	97.44901	361
19AR116_Spot 568	217.6649	19506.42	2.55753	18.04291	3.281282	0.403809	3.602884	0.05341	0.849079	0.235666	335.4256	2.775201	344.4074	10.52359	406.4108	78.38371	335.4	2.8	82.53365	502
19AR116_Part2_Spot 78	345.906	2712.634	1.785746	15.61963	2.277995	0.558833	3.088886	0.069332	0.953948	0.308832	432.128	3.987147	450.7668	11.24429	547.9684	64.16015	432.1	4.0	78.86002	598
19AR116_Spot 186	505.621	4322.607	3.010797	15.8093	1.307066	0.58656	2.46487	0.070871	1.995524	0.809586	441.4013	8.513481	468.6686	9.253201	605.448	31.31369	441.4	8.5	72.90491	165
19AR116_Spot 259	343.2836	16639.11	1.851089	16.89069	2.603619	0.572719	2.937118	0.07114	0.670692	0.22835	443.0219	2.871511	459.7712	10.86071	545.3233	62.47567	443.0	2.9	81.24023	230
19AR116_Part2_Spot 237	121.2329	1003.105	14.21599	13.06304	6.636546	0.619733	7.37577	0.072761	1.931817	0.261914	452.7679	8.446556	489.68	28.66248	667.2446	152.5992	452.8	8.4	67.85636	735
19AR116_Part2_Spot 194	312.4394	13921.78	2.678295	17.57763	3.260559	0.569502	3.690339	0.073928	1.172211	0.317643	459.7746	5.201819	457.6927	13.59739	448.2001	77.7897	459.8	5.2	102.5824	699
19AR116_Spot 123	263.5959	28833.34	2.204376	17.18485	2.900046	0.600286	3.051361	0.075384	0.640537	0.209918	468.5085	2.894513	477.4149	11.62259	521.3672	65.47864	468.5	2.9	89.86152	111
19AR116_Part2_Spot 343	179.0761	1656.224	1.694671	14.53866	3.836659	0.621754	4.151858	0.07572	0.751305	0.180956	470.5223	3.409128	490.9458	16.16376	588.3388	88.59279	470.5	3.4	79.97473	822
19AR116_Part2_Spot 83	139.2222	7876.153	1.324403	17.24815	4.160915	0.583509	5.313265	0.075741	0.969898	0.182543	470.652	4.40219	466.7137	19.88263	448.3323	116.1576	470.7	4.4	104.9784	602
19AR116_Spot 313	212.9668	14483.54	1.791819	16.82794	2.491369	0.610814	2.632502	0.07579	0.608463	0.231135	470.9451	2.76336	484.0734	10.13624	547.6543	55.93132	470.9	2.8	85.99314	279
19AR116_Part2_Spot 174	652.76	40017.72	16.09618	17.28283	1.841185	0.596759	2.015738	0.075816	0.684974	0.339813	471.1033	3.111842	475.1747	7.649476	495.841	41.77049	471.1	3.1	95.01096	682
19AR116_Part2_Spot 228	255.8267	16898.74	1.515277	17.02452	3.031468	0.602278	3.287017	0.07583	0.673533	0.204907	471.1816	3.060359	478.6784	12.54623	515.6909	70.69369	471.2	3.1	91.36901	727
19AR116_Part2_Spot 324	1121.049	91786.34	10.91283	17.57483	0.896667	0.588947	1.131679	0.075908	0.681482	0.602187	471.6544	3.099471	470.1951	4.259145	464.0403	20.04071	471.7	3.1	101.6408	806
19AR116_Spot 389	191.0367	24274.26	2.503364	17.20506	2.481419	0.605816	2.593842	0.075972	0.671153	0.258749	472.0371	3.05488	480.918	9.936475	524.4156	54.95197	472.0	3.1	90.01202	346
19AR116_Spot 392	90.05419	9870.107	2.754463	17.32625	4.417667	0.593399	4.889086	0.076112	0.950484	0.194409	472.8735	4.333698	473.0358	18.48961	474.787	106.1329	472.9	4.3	99.59698	348
19AR116_Part2_Spot 199	160.1089	14023.82	1.938739	15.24533	6.097956	0.679178	6.305921	0.076114	0.926802	0.146973	472.8847	4.225816	526.2776	25.90357	766.0494	131.5046	472.9	4.2	61.73031	703
19AR116_Spot 165	137.9106	9936.634	2.029685	16.32379	3.290674	0.628599	3.584785	0.076123	0.814732	0.227275	472.9379	3.715229	495.2225	14.05012	600.5416	75.60976	472.9	3.7	78.7519	148
19AR116_Spot 314	196.7205	24119.33	2.256919	16.89331	1.655107	0.618345	1.87134	0.076501	0.752535	0.402137	475.2053	3.44746	488.8096	7.260221	554.0262	37.39886	475.2	3.4	85.77307	280
19AR116_Spot 244	106.9296	11081.46	2.201861	16.02983	5.113544	0.647239	5.416346	0.076587	1.377278	0.254282	475.7181	6.316048	506.7783	21.61275	650.4781	112.5138	475.7	6.3	73.13361	216
19AR116_Spot 328	151.2641	22453.51	2.074291	17.06918	3.040664	0.612605	3.177144	0.076673	0.728063	0.229156	476.2328	3.342296	485.2017	12.25577	528.7664	67.80349	476.2	3.3	90.06487	292
19AR116_Spot 291	407.9628	35129.09	1.339219	17.83873	1.874662	0.589554	2.050806	0.076711	0.655352	0.319558	476.4624	3.009903	470.5828	7.723448	442.955	43.24073	476.5	3.0	107.5645	259
19AR116_Part2_Spot 332	384.8152	30194.34	63.06269	17.95642	1.714012	0.579336	1.986414	0.076725	0.747795	0.376455	476.5435	3.435038	464.0344	7.398831	403.5443	41.21134	476.5	3.4	118.0895	813
19AR116_Spot 283	449.1939	53557.44	2.072201	17.44107	2.815188	0.606074	2.89206	0.076743	0.601719	0.208059	476.6509	2.764632	481.081	11.08191	503.2166	62.29122	476.7	2.8	94.72083	251
19AR116_Part2_Spot 325	309.7359	15510.45	1.298839	17.44699	2.489362	0.59169	2.704378	0.076753	0.719471	0.26604	476.7104	3.306049	471.9462	10.20816	449.793	57.9353	476.7	3.3	105.9844	807
19AR116_Part2_Spot 360	381.7153	28820.01	6.942547	17.5382	2.536014	0.598803	2.712996	0.07676	0.730584	0.26929	476.7534	3.357403	476.4736	10.3177	476.0816	57.78191	476.8	3.4	100.1411	838
19AR116_Part2_Spot 116	180.6477	10377.5	0.666834	16.58162	3.868884	0.623063	3.988268	0.076827	0.768035	0.192573	477.1571	3.532387	491.7653	15.54693	561.3328	85.33696	477.2	3.5	85.00431	630
19AR116_Spot 397	167.147	9064.688	2.474923	16.7219	2.258635	0.620045	2.751762	0.076837	0.731693	0.2659	477.2159	3.36564	489.8756	10.69432	550.4762	57.91489	477.2	3.4	86.69145	353
19AR116_Spot 138	284.7199	3961.512	1.985775	16.16668	2.244312	0.617076	3.839343	0.076863	0.668827	0.174204	477.3723	3.077444	488.0131	14.87737	539.2179	82.70382	477.4	3.1	88.53051	123
19AR116_Spot 546	312.5868	10051.52	1.809839	17.17543	2.500858	0.602089	2.836741	0.076869	0.606188	0.213692	477.4057	2.789412	478.5588	10.82531	485.0708	61.19263	477.4	2.8	98.41981	483
19AR116_Spot 506	149.7034	15660.19	1.513417	17.92627	2.649973	0.580511	2.962085	0.07689	0.949066	0.320405	477.5309	4.368292	464.7895	11.04733	403.2723	62.82279	477.5	4.4	118.414	446
19AR116_Spot 448	558.9907	49860.96	3.344687	17.32201	1.042938	0.607409	1.333898	0.076922	0.681364	0.510806	477.7265	3.137369	481.9247	5.118127	502.9253	25.26168	477.7	3.1	94.98956	394
19AR116_Spot 561	167.2034	20928.53	2.187287	17.82281	3.17267	0.589354	3.304072	0.076948	0.763452	0.231064	477.8801	3.516438	470.4553	12.44105	435.3432	71.62276	477.9	3.5	109.7709	497
19AR116_Spot 84	256.0103	36354.28	1.678001	17.62576	2.057713	0.596745	2.280036	0.076972	0.716758	0.314362	478.0241	3.302322	475.1661	8.652378	462.3347	47.997	478.0	3.3	103.3935	76
19AR116_Spot 306	198.197	21168.67	1.392653	17.06745	3.720451	0.615987	3.886119	0.07701	0.793289	0.204134	478.251	3.656598	487.3288	15.0422	531.2164	83.35645	478.3	3.7	90.0294	272
19AR116_Spot 71	496.3463	37935.49	8.322205	17.29954	1.357524	0.607713	1.637691	0.077163	0.782118	0.477574	479.1695	3.611775	482.1168	6.285755	497.1134	31.70425	479.2	3.6	96.39038	64
19AR116_Spot 267	136.7086	10336.64	1.168655	17.24664	3.20591	0.602828	3.293325	0.077185	0.643851	0.195502	479.2998	2.974044	479.0271	12.57747	478.6619	71.40891	479.3	3.0	100.1333	236
19AR116_Spot 318	147.0964	7105.914	1.938085	15.98835	5.061841	0.644422	5.454533	0.077225	0.772361	0.1416	479.5363	3.569347	505.0402	21.70754	623.2032	116.5169	479.5	3.6	76.94701	283
19AR116_Spot 241	175.2267	28398.67	2.664374	16.59184	3.284829	0.638455	3.424481	0.077329	0.822662	0.24023	480.1622	3.806586	501.3489	13.55021	600.176	71.97488	480.2	3.8	80.00356	214
19AR116_Spot 253	122.1972	14970.32	2.036865	15.94619	4.454949	0.659019	4.578017	0.07735	0.735712	0.160705	480.2849	3.405092	514.0139	18.46724	667.8655	96.76944	480.3	3.4	71.91341	224
19AR116_Part2_Spot 347	101.5328	4093.3	1.207988	16.6235	4.383134	0.599624	5.279004	0.07742	0.940874	0.17823	480.7062	4.358324	476.9948	20.09553	460.1313	115.2602	480.7	4.4	104.4715	826
19AR116_Spot 483	140.9155	17283.73	8.255069	17.11041	3.184357	0.619007	3.463179	0.077859	0.783332	0.226189	483.3287	3.647616	489.2249	13.4455	517.8661	74.07765	483.3	3.6	93.33082	425
19AR116_Spot 531	278.9658	38770.76	1.33175	17.71273	2.19301	0.603189	2.366095	0.077878	0.782071	0.330532	483.4439	3.642581	479.2558	9.03946	460.228	49.53183	483.4	3.6	105.0444	469
19AR116_Part2_Spot 340	218.9927	9792.523	2.045456	16.00333	3.875845	0.650868	4.098037	0.078001	0.796603	0.194386	484.1797	3.715702	509.0129	16.40681	623.1006	86.74698	484.2	3.7	77.70489	819
19AR116_Spot 282	86.95363	8467.213	1.812302	16.4212	4.178048	0.640701	5.089941	0.078105	1.02051	0.200495	484.8011	4.765985	502.7403	20.18487	586.201	108.2879	484.8	4.8	82.7022	250
19AR116_Part2_Spot 399	226.8806	12529.25	1.744879	16.88371	3.334239	0.624285	3.534441	0.078326	0.997474	0.282215	486.1216	4.670618	492.5293	13.79424	523.3694	74.41036	486.1	4.7	92.88307	873
19AR116_Spot 97	294.3081	29268.5	1.610628	17.46594	2.786496	0.612757	2.92196	0.078338	0.767934	0.262815	486.1961	3.596342	485.2975	11.27305	482.0388	62.28515	486.2	3.6	100.8624	89
19AR116_Spot 467	180.8333	5011.313	1.649927	16.76656	2.937267	0.614928	3.295748	0.0784	0.768439	0.233161	486.5672	3.601349	486.6631	12.74317	488.0958	70.75355	486.6	3.6	99.68682	411
19AR116_Spot 526	171.627	16346.94	2.102179	16.83837	3.613654	0.634369	3.772823	0.078678	0.921509	0.244249	488.2253	4.332893	498.8141	14.87029	548.6714	79.90864	488.2	4.3	88.98319	464
19AR116_Spot 424	121.3792	5527.126	2.8015	16.98732	3.380365	0.614142	5.640867	0.079434	0.946402	0.167776	492.7452	4.489578	486.1689	21.79563	456.2523	123.4644	492.7	4.5	107.9984	375
19AR116_Part2_Spot 142	49.22361	3470.025	1.455463	12.02961	8.187255	0.889843	8.866527	0.082281	1.357193	0.153069	509.7263	6.651533	646.2849	42.4154	1158.758	174.0749	509.7	6.7	43.98901	655
19AR116_Part2_Spot 236	230.794	9578.471	6.469793	15.1993	3.696427	0.734198	4.276737	0.083331	1.078628	0.252208	515.9751	5.348547	559.0139	18.38672	739.2419	87.6013	516.0	5.3	69.79787	734
19AR116_Spot 501	399.016	50062.56	2.937033	17.39715	1.68836	0.660528	1.827594	0.083471	0.627301	0.343239	516.807	3.115386	514.9371	7.381803	507.5823	37.7648	516.8	3.1	101.8174	442
19AR116_Spot 246	544.4673	59754.27	4.128732	17.67693	1.599737	0.650297	1.789254	0.083522	0.67623	0.377939	517.1102	3.360275	508.6617	7.159109	471.833	36.67824	517.1	3.4	109.596	217
19AR116_Part2_Spot 151	196.1095	11998.67	1.289058	16.31947	2.692857	0.687684	3.012527	0.083722	1.062212	0.352598	518.2999	5.289936	531.408	12.46466	589.0626	61.1375	518.3	5.3	87.98722	662
19AR116_Spot 61	285.367	34068.61	4.754266	17.14889	2.52744	0.667416	2.712617	0.083875	0.691917	0.255073	519.211	3.45164	519.1403	11.02524	519.7643	57.57026	519.2	3.5	99.89354	54
19AR116_Spot 153	113.1011	13064.07	1.421952	17.27843	3.66049	0.65726	3.848937	0.083878	0.80414	0.208925	519.2287	4.011601	512.9363	15.50068	485.9649	83.13463	519.2	4.0	106.8449	137
19AR116_Part2_Spot 266	210.2742	19685.17	2.27139	17.55595	2.663763	0.645471	3.002507	0.083922	0.985451	0.328209	519.4913	4.91849	505.688	11.95971	444.6746	63.08705	519.5	4.9	116.825	759
19AR116_Part2_Spot 224	70.59769	4439.79	1.539979	14.67359	8.676468	0.750376	9.623106	0.084158	1.38787	0.144223	520.8936	6.944964	568.4423	41.91208	764.4158	201.0688	520.9	6.9	68.14271	723
19AR116_Spot 479	166.4927	18105.47	2.947254	15.97454	3.67643	0.717945	3.888816	0.084182	0.95576	0.245771	521.0362	4.783922	549.4528	16.50317	670.01	80.68612	521.0	4.8	77.76544	421
19AR116_Spot 394	193.5266	24732.04	4.98253	17.23536	3.349992	0.673183	3.484161	0.084345	0.610078	0.175101	522.0059	3.059116	522.6458	14.23463	526.4178	75.22003	522.0	3.1	99.16189	350

19AR116_Part2_Spot 107	58.68765	5322.554	1.300874	13.55639	6.713979	0.824839	7.399903	0.084353	1.449663	0.195903	522.0502	7.269641	610.7443	33.97524	955.985	148.5294	522.1	7.3	54.60862	622
19AR116_Spot 1	157.6218	20510.81	3.913419	17.78015	2.372504	0.643761	2.488469	0.084383	0.610943	0.245509	522.233	3.06473	504.6321	9.896049	426.5652	53.80708	522.2	3.1	122.4275	1
19AR116_Part2_Spot 252	219.3441	20050.97	1.29821	17.56981	3.391152	0.651545	3.49012	0.084384	0.809122	0.231832	522.2392	4.05892	509.4289	13.98143	453.2781	75.40699	522.2	4.1	115.2139	747
19AR116_Spot 352	77.45735	8388.818	1.884161	14.80429	4.969162	0.762299	5.351032	0.084425	0.857469	0.160244	522.4782	4.303344	575.3355	23.50665	790.9172	110.9042	522.5	4.3	66.05979	312
19AR116_Part2_Spot 355	324.828	23048.56	3.29948	17.23356	1.824501	0.66859	1.997215	0.084455	0.623019	0.311944	522.6603	3.127766	519.8547	8.125952	508.5009	41.71935	522.7	3.1	102.7845	833
19AR116_Part2_Spot 256	453.2132	27888.8	3.012399	16.90554	1.618089	0.680181	1.90764	0.084502	0.747422	0.391805	522.9402	3.75424	526.8837	7.841577	544.9602	38.34448	522.9	3.8	95.95933	749
19AR116_Part2_Spot 216	168.4702	11108.1	1.20624	16.94594	2.876573	0.67293	3.249026	0.084538	0.989116	0.304435	523.1556	4.970214	522.4924	13.27089	520.5283	67.93307	523.2	5.0	100.5048	716
19AR116_Spot 6	123.7821	13906.84	1.662602	17.33729	3.63932	0.657782	3.873253	0.084541	0.984019	0.254055	523.1696	4.944724	513.2562	15.6061	470.289	82.94898	523.2	4.9	111.2443	6
19AR116_Spot 554	104.5741	6808.858	1.656914	14.99101	6.211593	0.75485	6.721058	0.08458	0.922048	0.137188	523.4009	4.635288	571.0347	29.3636	766.4099	140.3921	523.4	4.6	68.29256	491
19AR116_Spot 268	921.6588	108947.3	11.10329	17.19561	0.927053	0.677261	1.142709	0.084595	0.642526	0.562283	523.4945	3.230636	525.1175	4.685156	533.1144	20.6944	523.5	3.2	98.19552	237
19AR116_Spot 191	110.1703	13519.67	3.036042	17.09733	4.289781	0.670083	4.393634	0.084622	0.757431	0.172393	523.655	3.809504	520.7628	17.90149	509.0329	95.19648	523.7	3.8	102.8725	169
19AR116_Spot 275	174.4688	1263.835	3.423215	13.9793	2.881941	0.703802	4.356874	0.084722	0.743189	0.170578	524.2457	3.741921	541.0594	18.27609	613.4673	92.79772	524.2	3.7	85.45619	244
19AR116_Spot 336	123.5223	17697.42	1.135038	16.23942	4.153822	0.70952	4.402409	0.084748	0.771284	0.175196	524.4006	3.884481	544.4611	18.55492	630.3009	93.39027	524.4	3.9	83.19844	299
19AR116_Part2_Spot 14	83.88407	6827.699	1.964168	17.41342	5.44228	0.646557	5.681601	0.084853	1.149846	0.202381	525.0236	5.797666	506.3578	22.65702	423.8554	124.2328	525.0	5.8	123.8685	543
19AR116_Part2_Spot 72	154.0389	8891.822	1.979627	16.1371	3.840931	0.703083	4.527405	0.084901	0.834892	0.184409	525.3113	4.211845	540.6307	18.98019	606.6877	96.28453	525.3	4.2	86.58678	592
19AR116_Part2_Spot 349	152.4905	6982.447	2.129642	16.01907	4.232125	0.702832	4.443329	0.084902	0.862597	0.194133	525.3142	4.351632	540.4813	18.62374	605.9056	94.3394	525.3	4.4	86.69902	828
19AR116_Part2_Spot 183	88.937	8104.118	1.168492	14.67483	4.735126	0.775068	5.029091	0.08492	0.874046	0.173798	525.4225	4.410264	582.6662	22.30047	813.4955	103.6254	525.4	4.4	64.58826	690
19AR116_Spot 276	185.1082	18237.63	3.331627	17.54819	2.39021	0.660209	2.589622	0.084975	0.739529	0.285574	525.7486	3.733737	514.742	10.45684	467.1495	54.96897	525.7	3.7	112.544	245
19AR116_Spot 433	150.8484	15870.42	5.585633	16.1493	3.812774	0.712826	4.040724	0.084983	0.883446	0.218636	525.8003	4.460769	546.4232	17.07656	634.3341	84.89894	525.8	4.5	82.89012	382
19AR116_Spot 92	65.86239	8533.056	1.928282	13.87651	6.223688	0.823387	6.353059	0.085001	1.028934	0.161959	525.9015	5.196339	609.9362	29.13782	936.7193	128.6989	525.9	5.2	56.1429	84
19AR116_Spot 550	381.3946	42789.21	1.143755	17.14036	1.710618	0.681367	1.809046	0.085019	0.577028	0.318968	526.0118	2.914705	527.6	7.443987	535.4249	37.55227	526.0	2.9	98.24193	487
19AR116_Spot 290	107.8638	7849.395	1.354041	15.95433	4.104255	0.715137	4.264132	0.085109	0.880338	0.206452	526.5479	4.451142	547.7918	18.05497	638.1148	89.8166	526.5	4.5	82.51618	258
19AR116_Part2_Spot 220	145.1365	7237.458	2.487981	16.36387	3.444195	0.693329	3.981061	0.08521	0.928236	0.233163	527.1443	4.698422	534.7985	16.55256	568.5488	84.3016	527.1	4.7	92.71751	719
19AR116_Spot 87	174.9055	19645.98	1.415281	16.91181	3.534941	0.684762	3.696822	0.085214	0.810389	0.219212	527.1688	4.102106	529.6483	15.2578	541.2866	78.87242	527.2	4.1	97.3918	79
19AR116_Spot 18	171.38	21373.96	2.477511	16.55844	3.081084	0.701974	3.248494	0.085287	0.642232	0.197701	527.604	3.253485	539.9696	13.60524	593.4953	69.03633	527.6	3.3	88.89776	16
19AR116_Spot 437	129.685	5672.235	2.441922	15.22568	3.546712	0.740107	4.069048	0.085343	0.823021	0.202264	527.9365	4.171868	562.468	17.57457	705.6602	84.81216	527.9	4.2	74.81454	385
19AR116_Spot 377	145.2393	25112.07	2.192786	16.22987	4.186243	0.71992	4.290671	0.085493	0.667782	0.155636	528.8266	3.390443	550.6194	18.23802	642.7768	91.15177	528.8	3.4	82.27221	334
19AR116_Spot 209	166.7139	25143.57	1.995714	17.20498	3.207291	0.677365	3.343524	0.085498	0.667066	0.19951	528.8564	3.386988	525.1804	13.71058	510.1809	72.03981	528.9	3.4	103.6606	183
19AR116_Part2_Spot 97	101.915	7718.612	1.558517	15.26759	6.465688	0.746972	6.72845	0.085503	0.951681	0.141441	528.8883	4.832385	566.4659	29.22019	721.2618	141.5065	528.9	4.8	73.3282	614
19AR116_Part2_Spot 204	285.3727	3435.276	2.374834	15.36867	2.760129	0.718798	3.191957	0.085523	1.170173	0.366601	529.0055	5.943097	549.9572	13.55485	638.6685	63.90773	529.0	5.9	82.82944	707
19AR116_Spot 171	96.53936	13304.02	1.700166	15.32076	4.679767	0.758495	4.826732	0.085585	0.996268	0.206406	529.3736	5.06324	573.1416	21.14256	751.648	99.76128	529.4	5.1	70.42839	154
19AR116_Part2_Spot 348	298.9771	25416.81	0.930827	17.11998	2.111774	0.677455	2.415362	0.085598	0.741698	0.307075	529.4481	3.769974	525.235	9.905022	507.9409	50.55176	529.4	3.8	104.2342	827
19AR116_Part2_Spot 258	256.3004	16764.75	1.62277	17.00593	3.097824	0.679745	3.305539	0.085614	0.754661	0.228302	529.5466	3.836547	526.6204	13.58316	514.8905	70.72531	529.5	3.8	102.8464	751
19AR116_Spot 514	138.8444	17322.74	2.091716	16.80066	3.615707	0.693254	3.788508	0.085765	0.850398	0.224468	530.4391	4.330244	534.7538	15.75082	554.1598	80.58638	530.4	4.3	95.71952	453
19AR116_Spot 497	72.55368	11887.27	2.34604	16.10353	5.429258	0.722343	5.704432	0.085772	1.152016	0.201951	530.4863	5.866594	552.0492	24.2968	642.9861	120.1828	530.5	5.9	82.50354	438
19AR116_Spot 249	157.4538	13141.56	1.543707	17.1863	3.148179	0.67691	3.466797	0.085849	0.645769	0.186272	530.9421	3.29126	524.9052	14.21045	499.7116	75.04533	530.9	3.3	106.2497	220
19AR116_Part2_Spot 106	120.0045	9118.544	2.960995	15.12975	5.064125	0.762426	5.437796	0.085955	0.926113	0.17031	531.568	4.725416	575.4087	23.8902	753.4623	113.1887	531.6	4.7	70.55004	621
19AR116_Spot 67	172.1834	12758.98	1.516671	17.20959	3.096369	0.671357	3.30586	0.086	0.779253	0.235719	531.8393	3.978022	521.5369	13.48416	477.6122	71.05747	531.8	4.0	111.3538	60
19AR116_Spot 405	243.7378	6433.644	5.391347	16.74743	2.675107	0.683721	3.01279	0.086002	0.713089	0.236687	531.8465	3.640307	529.0209	12.42308	517.7842	64.26049	531.8	3.6	102.7159	358
19AR116_Spot 580	163.9762	18609.84	4.254902	16.27576	4.06136	0.725176	4.359431	0.086025	1.127613	0.25866	531.9851	5.757884	553.7177	18.6088	645.0801	90.52605	532.0	5.8	82.46808	513
19AR116_Spot 212	168.8149	26191.79	3.976482	17.01419	3.580952	0.69023	3.756822	0.086101	0.622486	0.165695	532.4346	3.181153	532.9386	15.57875	536.0277	81.10107	532.4	3.2	99.32968	186
19AR116_Spot 415	154.014	15501.85	1.34136	17.07793	2.607283	0.686407	2.794174	0.086387	0.749	0.268058	534.1359	3.839419	530.6393	11.54839	516.5662	59.10512	534.1	3.8	103.4012	367
19AR116_Spot 96	225.3466	44094.51	2.808267	16.34657	3.080011	0.724487	3.183233	0.086397	0.667089	0.209563	534.1955	3.419905	553.3124	13.57985	633.7447	67.02484	534.2	3.4	84.29191	88
19AR116_Spot 481	178.9968	17780.75	2.168035	17.37778	2.292716	0.676958	2.729205	0.086477	0.718632	0.263312	534.666	3.687258	524.9342	11.18725	483.8082	58.1586	534.7	3.7	110.512	423
19AR116_Part2_Spot 362	100.7663	8167.988	1.374414	16.13498	4.055442	0.719814	4.495616	0.086532	0.938335	0.208722	534.9913	4.817348	550.557	19.10773	616.4057	94.986	535.0	4.8	86.79207	840
19AR116_Spot 188	46.50129	5590.204	1.811022	14.26016	6.553024	0.809871	7.181052	0.086844	1.52896	0.212916	536.8476	7.875699	602.3816	32.63892	858.2788	145.827	536.8	7.9	62.54933	167
19AR116_Spot 223	158.0715	7749.086	2.596554	16.81485	2.585486	0.691137	3.246184	0.087023	0.836701	0.257749	537.9084	4.318026	533.4832	13.47143	515.5393	68.92099	537.9	4.3	104.339	197
19AR116_Spot 309	81.33093	8467.073	1.598946	15.61881	5.048527	0.754375	5.394611	0.08763	0.940812	0.174398	541.5044	4.886432	570.7598	23.5577	690.0214	113.376	541.5	4.9	78.47647	275
19AR116_Part2_Spot 110	1252.337	63534.82	10.43938	16.70969	1.002388	0.726317	1.289558	0.08837	0.797132	0.618144	545.8871	4.172294	554.3892	5.509101	590.4127	21.96263	545.9	4.2	92.45858	624
19AR116_Part2_Spot 294	858.8785	40764.29	2.207166	16.67178	0.947489	0.730415	1.22882	0.089268	0.660976	0.537895	551.2064	3.491923	556.7967	5.266736	580.6595	22.49552	551.2	3.5	94.92765	783
19AR116_Part2_Spot 378	416.5713	29654.91	2.523067	17.00497	1.329987	0.719786	1.627908	0.089853	0.60553	0.371968	554.6662	3.218232	550.5407	6.918239	534.4831	33.08293	554.7	3.2	103.7762	855
19AR116_Part2_Spot 25	90.91327	8957.759	2.169342	13.24175	4.675367	0.923051	4.887466	0.090868	1.322414	0.270573	560.6713	7.101117	663.9721	23.82474	1033.398	95.13718	560.7	7.1	54.25513	553
19AR116_Spot 147	219.0391	13040.61	6.653955	16.55402	1.993441	0.750444	2.533052	0.091702	0.632391	0.249656	565.5951	3.424355	568.4822	11.0271	581.0034	53.28267	565.6	3.4	97.34799	131
19AR116_Spot 172	385.5849	5129.249	25.77532	15.85053	1.316801	0.764334	2.75153	0.091854	1.250639	0.454525	566.4952	6.782437	576.5076	12.10395	617.0896	52.91997	566.5	6.8	91.80113	155
19AR116_Part2_Spot 377	122.5853	3779.252	0.895303	14.7435	3.95507	0.820706	7.184963	0.093386	0.857981	0.119413	575.5314	4.723937	608.4424	32.89684	733.9198	151.2416	575.5	4.7	78.41884	854
19AR116_Part2_Spot 90	253.8618	22287.63	2.257138	16.98682	3.043877	0.747509	3.141769	0.093599	0.642493	0.2045	576.7856	3.544855	566.7782	13.64669	527.7965	67.42999	576.8	3.5	109.2818	608
19AR116_Part2_Spot 208	295.3535	15879.94	3.046922	16.18985	2.13494	0.787781	2.440094	0.093767	1.037356	0.42513	577.7784	5.732873	589.9123	10.91803	637.8313	47.53392	577.8	5.7	90.58483	710
19AR116_Spot 126	147.5761	1702.403	0.834205	13.92051	4.901871	0.820827	9.911542	0.093887	0.663732	0.066966	578.4843	3.672346	608.5097	45.39869	722.8516	210.279	578.5	3.7	80.02809	114
19AR116_Part2_Spot 58	114.7261	8725.156	2.333003	15.65358	3.643886	0.80302	4.070083	0.094234	1.056132	0.259487	580.5273	5.863173	598.531	18.40801	668.2987	84.14994	580.5	5.9	86.86643	580
19AR116_Spot 585	104.2465	9055.178	4.6867	15.80343	3.789124	0.809418	4.097978	0.094263	1.037552	0.253186	580.6992	5.761653	602.1276	18.61582	684.5999	84.66768	580.7	5.8	84.82316	517
19AR116_Part2_Spot 213	295.2848	27515.57	8.74497	16.86124	3.228285	0.76434	3.345122	0.094263	0.713534	0.213306	580.6998	3.962348	576.5111	14.71556	560.9813	71.24561	580.7	4.0	103.515	714
19AR116_Part2_Spot 182	359.327	27575.21	2.626666	16.54218	3.477807	0.776106	3.616784	0.094271	0.746867	0.2065	580.745	4.147755	583.2599	16.04874	593.9892	76.73776	580.7	4.1	97.77029	689
19AR116_Spot 351	879.7173	20245	6.461443	16.60849	0.935845	0.772563	1.177684	0.094428	0.670639	0.569456	581.6734	3.73011	581.2324	5.211883	580.4519	21.00854	581.7	3.7	100.2104	311
19AR116_Spot 331	560.0105	79697.73	1.293464	16.90417	0.640035	0.767576	0.867298	0.094459	0.576575	0.664795	581.8529	3.207872	578.3715	3.824227	565.6471	14.09829	581.9	3.2	102.865	294
19AR116_Spot 327	82.57973	5469.167	3.281962	15.28731	4.063929	0.817495	5.335954	0.094512	0.99367	0.186222	582.1639	5.531269	606.6499	24.37458	700.1133	111.7255	582.2	5.5	83.15281	291
19AR116_Spot 86	94.18011	13423.49	2.316704	15.06216	4.297441	0.850311	4.574821	0.094592	0.702623	0.153585	582.6363	3.914189	624.8196	21.35015	781.574	95.04604	582.6	3.9	74.54653	78
19AR116_Spot 51	473.2997	59897.51	7.39651	16.81134	0.812936	0.773559	1.04086	0.094932	0.577052	0.554399	584.642	3.225225	581.8025	4.609702	571.6904	18.84788	584.6	3.2	102.2655	45
19AR116_Part2_Spot 76	250.961	2840.596	2.263297	15.48121	2.951707	0.777127	3.502022	0.095083	0.720261	0.20567	585.5306	4.031489	583.8434	15.55093	578.2399	74.50451	585.5	4.0	101.2608	596
19AR116_Spot 62	284.8936	39009.34	1.411497	16.52908	1.586927	0.785283	1.7809	0.095096	0.638053	0.358275	585.6069	3.571789	588.4925	7.954216	600.5601	36.01801	585.6	3.6	97.51011	55
19AR116_Spot 570	404.6135	44104.64	0.828426	16.85104	1.590914	0.778825	1.707766	0.095366	0.578633	0.338825	587.1967	3.247562	584.8132	7.592295	576.4954	34.91994	587.2	3.2	101.8563	504
19AR116_Spot 548	174.6007	24664.62	1.492954	16.61252	3.291184	0.785794	3.380734	0.095555	0.589349	0.174326	588.3085	3.313683	588.7832	15.10603	591.5783	72.21618	588.3	3.3	99.44728	485
19AR116_Spot 79	211.5497	20947.06	3.51365	16.8267	1.774493	0.772459	1.952839	0.095592	0.725492	0.371506	588.5247	4.080596	581.1726	8.641837	553.4812	39.58785	588.5	4.1	106.3315	71
19AR116_Part2_Spot 264	163.8555	7282.373	1.669972	16.33933	3.359112	0.774775	3.706743	0.095647	0.948096	0.255776	588.8451	5.335431	582.4985	16.43208	558.7588	78.16876	588.8	5.3	105.3845	757
19AR116_Part2_Spot 168	168.731	14697.16	2.581075	16.56907	3.334209	0.777085	3.546784	0.09568	0.878472	0.247681	589.0445	4.945218	583.8191	15.74925	564.4746	74.86299	589.0	4.9	104.3527	676
19AR116_Part2_Spot 203	109.0407	13131.15	2.554623	14.97563	4.896867	0.868286	5.100236	0.095722	0.974713	0.191111	589.2874	5.489149	634.6362	24.07249	800.5515	104.9417	589.3	5.5	73.61019	706
19AR116_Spot 13	430.7648	49591.1	4.246693	16.66817	1.161833	0.789998	1.324766	0.095991	0.584513	0.44122	590.8739	3.300179	591.171	5.936734	593.2762	25.7484	590.9	3.3	99.59507	11
19AR116_Spot 41	216.7959	22799.02	1.098387	16.88166	3.324641	0.776399	3.41022	0.096153	0.710602	0.208374	591.8236	4.018237	583.427	15.13522	551.8152	72.82114	591.8	4.0	107.2503	37
19AR116_Spot 177	300.9814	15710.85	2.725366	16.08816	1.043127	0.817038	1.767321	0.096548	1.081249	0.611801	594.146	6.137039	606.3946	8.06924	653.3502	30.02147	594.1	6.1	90.93837	158
19AR116_Part2_Spot 286	154.8364	16946.63	1.57164	16.03215	4.589772	0.819385	4.69087	0.096897	0.824256	0.175715	596.196	4.693782	607.705	21.45414	651.8073	99.14928	596.2	4.7	91.46813	777
19AR116_Part2_Spot 23	227.3504	23087.54	6.016654	16.19372	2.660595	0.814746	2.827445	0.09692	0.680449	0.240659	596.3359	3.875736	605.1132	12.89003	639.0821	59.03776	596.3	3.9	93.31132	551
19AR116_Spot 564	1053.524	35133.6	4.2222	16.50932	0.663534	0.807175	1.009925	0.096957	0.600205	0.594307	596.5489	3.41984	600.8682	4.580252	618.1178	17.55312	596.5	3.4	96.51056	499
19AR116_Spot 382	579.5309	56298.27	2.893128	16.55744	0.923098	0.806082	1.098084	0.097118	0.554733	0.505183	597.4998	3.16556	600.2537	4.976347	611.5901	20.49051	597.5	3.2	97.69612	339
19AR116_Spot 426	377.0138	39426.32	1.425263	16.70621	1.378389	0.794539	1.607576	0.097143	0.76625	0.476649	597.6435	4.373574	593.7436	7.227219	579.7944	30.68175	597.6	4.4	103.0785	377
19AR116_Part2_Spot 276	829.7576	10878.74	0.843556	15.98067	0.771485	0.816124	1.057139	0.097155	0.610859	0.577841	597.7147	3.487036	605.8836	4.823652	637.5138	18.57368	597.7	3.5	93.75714	768
19AR116_Part2_Spot 201	1245.352	5767.71	1.989862	15.89447	0.765213	0.810734	1.170527	0.097181	0.665539	0.568581	597.8657	3.800089	602.866	5.321562	622.6316	20.7904	597.9	3.8	96.02239	704
19AR116_Part2_Spot 280	413.6463	34397.89	2.617812	16.18788	1.671495	0.818068	1.869631	0.097213	0.75794	0.405395	598.0573	4.329001	606.9703	8.542312	641.3429	36.75144	598.1	4.3	93.2508	772
19AR116_Part2_Spot 149	174.2574	2379.695	1.570222	14.87684	3.233924	0.81429	4.41079	0.09732	1.338461	0.303451	598.6861	7.652342	604.8576	20.10367	628.968	90.59106	598.7	7.7	95.18546	660
19AR116_Spot 49	151.7924	16427.48	1.471316	16.39668	3.510189	0.807246	3.588652	0.097562	0.599397	0.167026	600.1043	3.434658	600.9081	16.27748	604.8631	76.54418	600.1	3.4	99.21323	43
19AR116_Part2_Spot 136	787.0558	61015.17	1.486971	16.49891	1.117566	0.808538	1.31737	0.097749	0.673287	0.511084	601.2041	3.86481	601.6333	5.980196	604.1714	24.49442	601.2	3.9	99.50886	649
19AR116_Part2_Spot 33	536.7972	4309.378	1.114343	15.70371	1.25355	0.811022	1.707052	0.098013	0.626357	0.366923	602.751	3.604249	603.027	7.762364	604.9854	34.37272	602.8	3.6	99.63067	559
19AR116_Spot 551	1343.148	274802	0.772195	16.77867	0.62937	0.808014	0.888722	0.098207	0.623679	0.70177	603.8942	3.595328	601.3393	4.032881	592.6407	13.71807	603.9	3.6	101.8989	488
19AR116_Spot 330	67.1157	13196.51	375.7675	15.57147	5.073498	0.854823	5.190851	0.098276	0.763287	0.147045	604.3001	4.402951	627.2925	24.29545	712.017	109.2094	604.3	4.4	84.87158	293
19AR116_Spot 152	51.7926	2376.163	3.397838	14.85587	6.194501	0.832636	7.52443	0.098497	1.043661	0.138703	605.5926	6.032543	615.0739	34.72576	651.0639	160.1866	605.6	6.0	93.01585	136
19AR116_Part2_Spot 350	330.1753	19945.69	1.819627	16.18359	1.564738	0.831715	1.812726	0.098952	0.727458	0.401306	608.2649	4.22253	614.5632	8.357734	638.7961	35.72808	608.3	4.2	95.22051	829
19AR116_Part2_Spot 155	373.1482	15169.76	0.831789	16.13913	2.011836	0.825709	2.364913	0.099117	0.870064	0.367905	609.2302	5.057926	611.2285	10.86068	619.5589	47.46322	609.2	5.1	98.3329	666
19AR116_Spot 555	205.6755	14995.83	1.675118	16.17175	1.632674	0.833669	2.106396	0.09912	0.653328	0.310164	609.2482	3.798085	615.6459	9.724246	640.202	43.08202	609.2	3.8	95.16499	492
19AR116_Spot 539	196.2832	16168.93	2.020821	16.34979	2.641285	0.824628	2.894695	0.099303	0.815539	0.281736	610.3221	4.749055	610.6269	13.28437	612.6776	60.01702	610.3	4.7	99.61554	477
19AR116_Spot 37	131.7026	16044.82	2.15121	16.23879	3.755772	0.831766	3.86847	0.099364	0.802806	0.207525	610.6833	4.677548	614.5916	17.83795	629.9346	81.54548	610.7	4.7	96.94391	33
19AR116_Spot 597	370.2904	48390.4	3.985118	16.76571	1.421879	0.819252	1.565539	0.099549	0.628869	0.401695	611.7674	3.670306	607.631	7.15856	593.2016	31.07748	611.8	3.7	103.1298	527
19AR116_Part2_Spot 68	102.8234	6242.569	1.393086	15.32166	5.293682	0.858979	5.456176	0.099553	1.085024	0.198862	611.7904	6.332819	629.5656	25.60463	694.8781	114.0378	611.8	6.3	88.04285	589
19AR116_Spot 5	160.2922	17711.38	0.38269	16.52196	2.28783	0.816704	2.482921	0.099561	0.656578	0.264438	611.8383	3.832448	606.2082	11.33422	586.1833	51.97961	611.8	3.8	104.3766	5
19AR116_Part2_Spot 99	185.6188	19222.23	1.230339	15.52864	3.599262	0.872373	3.699944	0.099786	0.733256	0.19818	613.152	4.288779	636.8549	17.50564	722.7945	76.9427	613.2	4.3	84.83075	616
19AR116_Spot 83	329.079	53006.77	3.520577	16.39487	1.283574	0.833525	1.511746	0.099795	0.709698	0.469456	613.2102	4.151362	615.5661	6.978263	625.1597	28.78818	613.2	4.2	98.08857	75
19AR116_Part2_Spot 171	380.9564	34872.15	6.543787	16.67221	1.908209	0.813367	2.142161	0.099911	0.917922	0.428503	613.8893	5.375036	604.3408	9.756544	569.6598	42.10261	613.9	5.4	107.7642	679
19AR116_Part2_Spot 55	511.7195	13164.6	4.202714	16.34591	0.786632	0.821951	1.104297	0.099927	0.595235	0.539018	613.9807	3.485987	609.1362	5.058578	592.0842	20.1597	614.0	3.5	103.6982	577
19AR116_Spot 474	201.4053	40575.25	3.051555	16.31067	1.918604	0.840768	2.056328	0.099956	0.687395	0.334283	614.1487	4.026769	619.5694	9.537008	640.3797	41.66843	614.1	4.0	95.90383	417
19AR116_Spot 80	227.5843	35153.12	1.81018	16.08779	1.562681	0.849722	1.818789	0.100026	0.721184	0.396519	614.5611	4.22741	624.4967	8.483847	661.5728	35.78453	614.6	4.2	92.89396	72
19AR116_Spot 307	96.14261	17388.61	2.624826	16.11576	4.876068	0.847372	5.028856	0.10011	0.956892	0.19028	615.0564	5.61338	623.2059	23.42589	653.8571	105.986	615.1	5.6	94.06588	273
19AR116_Spot 247	415.1228	30649.27	1.783841	16.58914	1.340628	0.827778	1.474695	0.10013	0.582084	0.394715	615.1693	3.415256	612.3788	6.781555	602.9935	29.33134	615.2	3.4	102.0192	218
19AR116_Spot 472	382.4998	53939.95	4.062883	16.47069	1.195284	0.835205	1.398497	0.100169	0.64978	0.464628	615.3972	3.813795	616.496	6.462578	621.4519	26.72678	615.4	3.8	99.0257	416
19AR116_Part2_Spot 82	147.3311	14161.18	1.833696	15.56282	4.030497	0.869541	4.223448	0.100201	0.956323	0.226432	615.5852	5.614639	635.3178	19.94839	707.0957	87.53449	615.6	5.6	87.05825	601
19AR116_Part2_Spot 386	438.2798	24621.09	2.480163	16.32213	1.571846	0.833684	1.752654	0.100202	0.716238	0.408659	615.5929	4.205133	615.6541	8.091186	616.7989	34.53895	615.6	4.2	99.80448	863
19AR116_Spot 82	207.805	38589.27	2.644783	16.08843	2.00314	0.851911	2.221833	0.100228	0.943797	0.424783	615.7463	5.542482	625.6975	10.37841	662.7538	43.12122	615.7	5.5	92.90726	74
19AR116_Part2_Spot 382	314.0241	32335.67	3.896204	16.31119	1.946777	0.838911	2.159979	0.100358	0.587663	0.272069	616.5089	3.455141	618.5446	10.00573	626.9189	44.82403	616.5	3.5	98.3395	859
19AR116_Spot 4	178.8019	27851.36	2.449253	16.20479	3.276668	0.844296	3.355914	0.10043	0.649147	0.193434	616.9284	3.819114	621.5133	15.60051	639.1995	70.81804	616.9	3.8	96.51579	4
19AR116_Part2_Spot 341	405.7728	41553.01	3.435758	16.31243	1.626014	0.840104	1.794537	0.10077	0.685485	0.381984	618.9181	4.04529	619.2033	8.319225	621.1644	35.76876	618.9	4.0	99.63838	820
19AR116_Part2_Spot 130	265.6692	29055.01	1.776587	16.51813	2.90334	0.831474	3.007838	0.10087	0.767495	0.255165	619.5045	4.533349	614.4295	13.86625	596.6917	63.00446	619.5	4.5	103.8232	643
19AR116_Spot 63	431.0306	27642.63	1.6188	16.17154	1.812523	0.847786	1.950928	0.100903	0.675819	0.346409	619.6966	3.993029	623.4333	9.089024	637.9754	39.39554	619.7	4.0	97.13487	56
19AR116_Part2_Spot 47	284.0087	12989.93	2.727243	15.89338	1.567425	0.854152	1.911243	0.100945	0.711562	0.372303	619.9459	4.205821	626.9254	8.940191	653.1416	38.0894	619.9	4.2	94.91753	569
19AR116_Part2_Spot 387	434.1493	51295.37	2.706266	16.33656	1.521585	0.84411	1.732432	0.101036	0.732377	0.422745	620.4796	4.332406	621.4109	8.052064	625.7219	33.8507	620.5	4.3	99.1622	864
19AR116_Spot 520	148.6894	24240.86	2.200298	16.24496	3.064201	0.850223	3.174257	0.101097	0.774918	0.244126	620.8336	4.586549	624.7716	14.81193	640.0134	66.20301	620.8	4.6	97.00322	458
19AR116_Spot 383	368.1647	37292.15	4.323589	16.23583	2.002658	0.855497	2.143566	0.101113	0.758444	0.353824	620.9288	4.489701	627.6615	10.0355	652.9465	43.03422	620.9	4.5	95.09643	340
19AR116_Spot 216	71.05401	9420.94	1.530527	16.10751	6.221075	0.844028	6.602182	0.101168	1.096807	0.166128	621.2493	6.495876	621.366	30.69297	622.7098	140.5476	621.2	6.5	99.76547	190
19AR116_Part2_Spot 28	255.9389	11404.65	3.683632	15.70745	2.669644	0.867196	3.049155	0.10119	0.875783	0.287222	621.3809	5.187898	634.0437	14.38023	680.36	62.40814	621.4	5.2	91.33119	555
19AR116_Part2_Spot 186	271.3921	25061.13	0.695954	16.52413	2.244163	0.835143	2.391822	0.101202	0.75745	0.316683	621.4467	4.487379	616.4616	11.05266	599.1399	49.14519	621.4	4.5	103.7231	693
19AR116_Part2_Spot 126	225.6638	19052.69	3.272796	16.34312	2.149137	0.842676	2.288539	0.10122	0.720788	0.314955	621.5558	4.270893	620.6211	10.62712	618.1322	46.88908	621.6	4.3	100.5539	639
19AR116_Spot 88	459.7728	56856.47	2.407617	16.63135	0.934579	0.8341	1.127813	0.101262	0.619476	0.549272	621.8022	3.671973	615.8845	5.20794	595.1129	20.41051	621.8	3.7	104.4847	80
19AR116_Spot 94	219.2215	27827.72	1.720401	16.50179	3.396901	0.838041	3.479574	0.101386	0.679718	0.195345	622.5265	4.03353	618.0638	16.11026	602.6746	73.86824	622.5	4.0	103.294	86
19AR116_Part2_Spot 74	254.8379	4495.692	0.538675	15.83733	3.953644	0.834985	5.568198	0.101434	0.785927	0.141146	622.8062	4.665788	616.3742	25.73259	593.7823	119.545	622.8	4.7	104.888	594
19AR116_Spot 180	58.72279	7506.567	4.006284	15.67391	4.529516	0.867374	5.095936	0.101438	1.464992	0.287482	622.8287	8.697472	634.1403	24.03865	675.5976	104.4117	622.8	8.7	92.1893	160
19AR116_Spot 208	115.7151	12984.29	2.276939	16.05614	2.484321	0.854269	2.738856	0.10144	0.845796	0.308814	622.8436	5.021495	626.9893	12.8128	642.9306	56.01098	622.8	5.0	96.87571	182
19AR116_Part2_Spot 354	181.8774	5688.103	1.837584	15.74175	3.132975	0.853696	3.356475	0.101494	0.747326	0.222652	623.157	4.439004	626.6756	15.69686	640.354	70.38432	623.2	4.4	97.31445	832
19AR116_Spot 457	252.2554	34917.78	4.100803	16.57751	1.961637	0.841439	2.115438	0.101633	0.671431	0.317396	623.9697	3.993152	619.9394	9.815422	606.1731	43.39268	624.0	4.0	102.9359	401
19AR116_Part2_Spot 101	195.5638	13806.27	2.623838	15.7595	3.312996	0.871694	3.461896	0.101713	0.740553	0.213916	624.4377	4.407386	636.4867	16.37234	680.415	72.26544	624.4	4.4	91.77306	617
19AR116_Spot 596	145.1129	20275.33	0.622128	16.16322	2.087722	0.8629	2.47471	0.10175	0.741512	0.299636	624.6579	4.414577	631.7045	11.63976	657.9604	50.63306	624.7	4.4	94.93853	526
19AR116_Part2_Spot 255	454.3266	27070.4	3.219207	16.35971	1.152599	0.846404	1.465626	0.101753	0.744182	0.507757	624.6715	4.430561	622.6735	6.821983	616.3378	27.2842	624.7	4.4	101.3521	748
19AR116_Part2_Spot 114	431.2068	42108.23	5.525345	16.52465	1.47707	0.843587	1.669037	0.101838	0.631052	0.378094	625.169	3.759881	621.123	7.754798	607.3294	33.43325	625.2	3.8	102.9374	628
19AR116_Spot 430	257.1359	47900.55	3.029043	16.36178	1.766011	0.851889	1.951649	0.101979	0.747797	0.383162	625.996	4.461075	625.6856	9.116149	625.4818	38.87392	626.0	4.5	100.0822	381
19AR116_Part2_Spot 246	631.9852	17567.28	3.319749	16.25547	1.208647	0.849288	1.441689	0.101984	0.728418	0.505253	626.0264	4.345666	624.258	6.722925	618.7735	26.86405	626.0	4.3	101.1721	742
19AR116_Part2_Spot 77	512.5002	10482.82	3.391255	15.95326	1.518294	0.859164	1.817996	0.102017	0.823985	0.453238	626.2185	4.917247	629.6664	8.530831	643.0251	34.83719	626.2	4.9	97.38631	597
19AR116_Part2_Spot 317	784.593	62348.74	3.582333	16.25273	0.997701	0.853875	1.214925	0.102124	0.658683	0.542159	626.8435	3.934519	626.7734	5.681954	627.4373	22.00666	626.8	3.9	99.90536	799
19AR116_Part2_Spot 12	73.71794	4676.481	0.888053	15.43632	6.151977	0.867532	6.692033	0.102139	1.177804	0.176001	626.9326	7.036342	634.2263	31.57515	661.2535	141.3192	626.9	7.0	94.80972	541
19AR116_Part2_Spot 121	259.9677	26749.14	2.205545	16.44897	3.140077	0.847257	3.269888	0.102143	0.869641	0.265954	626.9556	5.195519	623.1426	15.22942	610.2429	68.14173	627.0	5.2	102.7387	635
19AR116_Spot 470	155.6118	25812.61	3.084076	16.24917	3.945392	0.859702	4.027812	0.102201	0.624513	0.15505	627.2934	3.73296	629.9602	18.90836	640.5016	85.58143	627.3	3.7	97.93784	414
19AR116_Spot 119	1523.038	202032.6	2.980087	16.46658	0.600862	0.856733	0.835183	0.102277	0.579038	0.693307	627.7397	3.463482	628.3379	3.912997	631.449	12.98304	627.7	3.5	99.41257	108
19AR116_Spot 591	1156.433	90453.4	4.960053	16.6195	0.625992	0.851317	0.860334	0.102334	0.580655	0.674919	628.0728	3.47491	625.3719	4.017074	616.5342	13.72802	628.1	3.5	101.8715	522
19AR116_Spot 201	109.9191	13592.84	1.271676	15.41681	3.522397	0.900299	3.715546	0.102481	0.743811	0.200189	628.9322	4.457106	651.8874	17.87567	733.1276	77.13821	628.9	4.5	85.78755	177
19AR116_Spot 469	129.344	18253.21	1.861607	16.14359	4.29403	0.864548	4.373241	0.10249	0.659605	0.150828	628.9843	3.952836	632.6025	20.59247	646.5087	92.91822	629.0	4.0	97.28938	413
19AR116_Spot 324	166.6262	24402.7	1.916701	16.43331	1.999945	0.850975	2.157683	0.102491	0.640274	0.296742	628.9886	3.837018	625.1838	10.07275	612.361	44.50255	629.0	3.8	102.7153	288
19AR116_Spot 56	938.6171	50931.3	4.27471	16.14294	0.672189	0.870007	0.952039	0.102576	0.634732	0.666708	629.4895	3.806686	635.571	4.497457	658.1915	15.21314	629.5	3.8	95.63927	50
19AR116_Spot 108	533.5341	77481.67	3.470593	16.46429	0.887878	0.858412	1.116392	0.102599	0.64815	0.580575	629.6231	3.887942	629.2556	5.236057	628.8527	19.60221	629.6	3.9	100.1225	98
19AR116_Spot 512	472.2925	41951.13	3.764484	16.29505	1.240159	0.861002	1.424497	0.102675	0.649398	0.455879	630.0673	3.898046	630.6695	6.691985	633.7865	27.30697	630.1	3.9	99.41318	451
19AR116_Spot 55	308.6644	80407.72	6.231594	16.22795	1.539931	0.868126	1.680936	0.102722	0.633059	0.376611	630.3409	3.80154	634.5491	7.931702	650.5104	33.4533	630.3	3.8	96.89944	49
19AR116_Part2_Spot 240	142.4744	5719.029	3.000737	15.79202	5.292597	0.856803	5.809359	0.102737	1.051491	0.180999	630.4291	6.315082	628.3761	27.22559	621.9116	123.3184	630.4	6.3	101.3696	737

19AR116_Part2_Spot 35	163.0337	15280.07	1.955849	16.42659	3.207649	0.844243	3.403752	0.102764	0.751036	0.220649	630.5841	4.511652	621.4846	15.8224	589.4119	72.02289	630.6	4.5	106.9853	560
19AR116_Spot 85	322.0286	46402.48	7.158744	16.22651	1.391165	0.867229	1.605998	0.102773	0.670773	0.417667	630.6422	4.029846	634.0617	7.573894	647.2268	31.34734	630.6	4.0	97.43759	77
19AR116_Spot 218	733.8595	43312.46	3.692949	16.37742	1.01071	0.860024	1.255266	0.102791	0.697301	0.555501	630.7435	4.189864	630.1358	5.893355	628.8735	22.49043	630.7	4.2	100.2974	192
19AR116_Spot 73	193.0893	29087.98	2.138299	16.40557	2.534641	0.854704	2.634629	0.10288	0.634262	0.240741	631.2657	3.814087	627.2276	12.32855	613.6134	55.25849	631.3	3.8	102.8768	65
19AR116_Spot 199	3160.69	63431.93	37.08363	16.35812	0.591074	0.863875	0.902331	0.102923	0.631138	0.699453	631.5169	3.796734	632.236	4.246512	635.7665	13.8997	631.5	3.8	99.33157	175
19AR116_Part2_Spot 9	1060.165	20147.01	6.490747	15.8921	0.863951	0.886201	1.162418	0.103018	0.671337	0.577535	632.0709	4.041932	644.3263	5.5455	688.4685	20.22705	632.1	4.0	91.80826	538
19AR116_Spot 557	137.9973	14510.22	3.967385	16.02742	3.393075	0.873896	3.490092	0.103054	0.578024	0.165619	632.2806	3.481222	637.6805	16.52797	657.8159	73.82571	632.3	3.5	96.11817	494
19AR116_Spot 334	188.6704	24334.62	4.222381	16.13294	2.690867	0.872649	2.775098	0.103163	0.552718	0.199171	632.9182	3.332009	637.0049	13.13155	652.4431	58.37749	632.9	3.3	97.00741	297
19AR116_Spot 192	240.9997	25401.81	2.408119	15.86956	2.11978	0.889343	2.349439	0.103263	0.509	0.216647	633.501	3.071144	646.0163	11.22976	690.9455	48.90088	633.5	3.1	91.6861	170
19AR116_Spot 535	449.9672	57199.22	3.971885	16.32773	1.348508	0.870803	1.580231	0.103405	0.784273	0.496303	634.3307	4.737955	636.0031	7.46879	642.9076	29.49999	634.3	4.7	98.66592	473
19AR116_Spot 522	118.9005	21912.83	3.138451	15.74598	2.669858	0.897015	2.905366	0.103455	0.908074	0.312551	634.6254	5.488287	650.1313	13.95041	705.2644	58.70937	634.6	5.5	89.98404	460
19AR116_Part2_Spot 51	296.9509	22083.81	2.054076	16.30095	1.980772	0.8598	2.104547	0.103531	0.638255	0.303274	635.0683	3.860097	630.0138	9.879469	612.8339	43.30856	635.1	3.9	103.6281	573
19AR116_Spot 475	111.9521	21279.38	2.230048	16.18469	3.689208	0.873049	3.802683	0.103531	0.829548	0.218148	635.0706	5.017035	637.2213	17.99927	645.8112	79.74187	635.1	5.0	98.33688	418
19AR116_Spot 466	104.6246	17626.36	7.335402	15.09519	6.091742	0.93779	6.166911	0.103684	0.83924	0.136088	635.9638	5.082442	671.7247	30.31276	794.4722	128.2375	636.0	5.1	80.04859	410
19AR116_Spot 109	67.297	12734.4	2.354428	15.83029	4.276753	0.888781	4.489744	0.103686	1.040907	0.231841	635.9712	6.303805	645.7143	21.45504	680.8445	93.3268	636.0	6.3	93.40918	99
19AR116_Part2_Spot 156	175.6452	19618.78	4.618985	16.03205	4.244365	0.873275	4.370799	0.103754	0.944935	0.216193	636.3712	5.726015	637.3441	20.6919	641.7523	91.79719	636.4	5.7	99.1615	667
19AR116_Spot 112	163.0673	9954.205	2.784484	15.4332	2.49675	0.910592	2.75117	0.103965	0.762436	0.277131	637.6035	4.628638	657.3724	13.3146	726.7113	56.05517	637.6	4.6	87.7382	102
19AR116_Part2_Spot 230	143.3337	16093.39	3.643005	15.8473	4.485486	0.886948	4.741156	0.10404	0.877343	0.185048	638.0403	5.329694	644.7286	22.63208	669.1465	99.75681	638.0	5.3	95.35136	728
19AR116_Spot 340	768.9735	74749.36	1.513636	16.48153	0.976967	0.86772	1.245185	0.104181	0.747204	0.600075	638.8631	4.544696	634.3287	5.874038	619.1232	21.50189	638.9	4.5	103.1884	302
19AR116_Spot 271	277.3068	31627.34	3.469706	16.48477	1.652703	0.86552	1.830416	0.104182	0.624228	0.34103	638.8731	3.796775	633.1316	8.623177	613.6052	37.17193	638.9	3.8	104.118	240
19AR116_Spot 476	184.5493	31009.84	2.167862	16.22313	2.552256	0.883557	2.702311	0.104663	0.733277	0.271352	641.6782	4.478675	642.9021	12.87195	648.1605	55.87091	641.7	4.5	98.99988	419
19AR116_Spot 428	161.9488	30323.6	1.757411	16.25532	3.708195	0.878098	3.842439	0.104668	0.759547	0.197673	641.7074	4.639331	639.9551	18.24351	634.7316	81.0989	641.7	4.6	101.099	379
19AR116_Spot 440	504.6234	48027.98	1.785956	16.22274	1.150218	0.882892	1.361295	0.104786	0.672105	0.493724	642.3959	4.109419	642.5436	6.481424	644.0191	25.45146	642.4	4.1	99.74797	387
19AR116_Spot 495	455.5267	47229.23	2.961763	16.43703	1.085972	0.877891	1.367384	0.104844	0.71681	0.52422	642.7351	4.384956	639.8432	6.490775	630.6036	25.10449	642.7	4.4	101.9238	436
19AR116_Spot 292	772.7362	10345.24	2.443277	15.91328	0.850948	0.890111	1.133824	0.104885	0.706846	0.623418	642.9737	4.325535	646.4292	5.421715	659.4463	18.98449	643.0	4.3	97.50205	260
19AR116_Part2_Spot 306	844.7263	46377.42	61.11778	16.19589	0.872579	0.882836	1.166006	0.104947	0.688879	0.590802	643.335	4.217837	642.5132	5.551399	640.5822	20.24199	643.3	4.2	100.4297	791
19AR116_Part2_Spot 320	379.9409	30925.86	2.566921	16.35619	1.18389	0.869905	1.340372	0.104968	0.591675	0.441426	643.4595	3.623345	635.5157	6.331597	608.2851	26.02008	643.5	3.6	105.7826	802
19AR116_Spot 285	104.2106	16550.61	4.533305	16.23955	5.178484	0.882405	5.290262	0.105124	0.732456	0.138454	644.3681	4.491496	642.2805	25.18552	635.9037	112.8287	644.4	4.5	101.3311	253
19AR116_Spot 365	574.9203	56487.93	3.101112	16.36521	0.841677	0.882828	1.021565	0.105128	0.569275	0.557257	644.3924	3.490975	642.5087	4.863676	636.85	18.25252	644.4	3.5	101.1843	325
19AR116_Spot 536	221.3535	42532.98	3.197444	16.2247	1.428577	0.89311	1.702111	0.105531	0.742006	0.435933	646.7424	4.565995	648.0386	8.153725	653.4922	32.89413	646.7	4.6	98.96712	474
19AR116_Part2_Spot 71	220.98	21084.57	1.610326	16.03724	1.700527	0.895131	2.1411	0.105763	0.951816	0.444545	648.0949	5.868724	649.1224	10.26902	653.6108	41.15789	648.1	5.9	99.15609	591
19AR116_Spot 99	196.198	26002.5	2.135749	15.79572	2.88309	0.914678	2.978269	0.105942	0.723736	0.243005	649.1382	4.469249	659.5415	14.4476	696.2024	61.55048	649.1	4.5	93.23986	90
19AR116_Spot 542	652.3994	89203.33	2.031566	16.43805	0.808872	0.886164	1.066716	0.106002	0.677919	0.63552	649.485	4.188444	644.3063	5.088819	627.1134	17.77112	649.5	4.2	103.5674	479
19AR116_Part2_Spot 148	800.6493	37034.04	52.46762	16.19041	1.086574	0.892238	1.340175	0.106049	0.758146	0.565707	649.7621	4.686017	647.5708	6.416556	640.8946	23.78479	649.8	4.7	101.3836	659
19AR116_Spot 181	99.44859	13162.04	0.869797	16.43316	4.482311	0.87648	4.623581	0.106146	0.891933	0.19291	650.3255	5.517484	639.0799	21.93179	600.4434	98.25216	650.3	5.5	108.3075	161
19AR116_Part2_Spot 344	532.5504	48127.77	1.342597	16.06109	1.467464	0.897033	1.64438	0.106162	0.720224	0.437991	650.4177	4.455893	650.1405	7.895405	650.0927	31.74122	650.4	4.5	100.05	823
19AR116_Spot 420	382.6717	48617.14	4.106522	16.33446	1.418472	0.891932	1.563482	0.1063	0.569206	0.364063	651.2267	3.525734	647.4067	7.484397	635.0647	31.36063	651.2	3.5	102.5449	371
19AR116_Part2_Spot 96	113.2624	14876.56	0.965548	14.94982	5.188461	0.963076	5.331822	0.106404	1.161648	0.217871	651.8298	7.201733	684.8888	26.56612	795.9688	109.1604	651.8	7.2	81.89137	613
19AR116_Spot 101	288.6689	6251.103	2.402012	15.87645	1.650239	0.890001	1.92392	0.106448	0.753713	0.391759	652.0839	4.674437	646.37	9.199356	627.3775	38.15815	652.1	4.7	103.9381	92
19AR116_Part2_Spot 319	518.0491	50321.09	163.5967	16.21959	1.23351	0.89309	1.424349	0.106545	0.590073	0.414276	652.6497	3.66258	648.0281	6.823024	632.9174	27.92815	652.6	3.7	103.1177	801
19AR116_Part2_Spot 263	306.2611	33804.57	1.913581	16.03825	1.495025	0.904208	1.818076	0.106737	0.932467	0.512887	653.7687	5.797243	653.974	8.766107	655.6365	33.49244	653.8	5.8	99.7151	756
19AR116_Spot 398	289.4077	42147.57	2.112791	16.18183	1.263532	0.911354	1.391933	0.106955	0.560875	0.402947	655.0424	3.493475	657.7771	6.73908	668.077	27.25582	655.0	3.5	98.04895	354
19AR116_Spot 387	320.1201	56487.07	0.911621	16.62728	1.66904	0.886685	1.804753	0.107068	0.659957	0.365677	655.6991	4.114533	644.5866	8.612473	606.7652	36.34987	655.7	4.1	108.0647	344
19AR116_Part2_Spot 293	413.6593	51207.3	2.410261	16.15804	1.536635	0.90774	1.693744	0.107122	0.63815	0.376769	656.0134	3.980384	655.8552	8.183315	656.2659	33.67389	656.0	4.0	99.96152	782
19AR116_Spot 459	326.959	40831.91	2.755685	16.34245	1.415743	0.901873	1.598959	0.107243	0.675709	0.422593	656.7181	4.218959	652.7278	7.699089	639.9136	31.16947	656.7	4.2	102.6261	403
19AR116_Spot 75	71.05593	14445.1	0.950347	16.0972	4.674942	0.904595	4.847721	0.107443	0.85422	0.176211	657.8779	5.342484	654.1801	23.38276	642.4034	102.6391	657.9	5.3	102.4088	67
19AR116_Part2_Spot 358	318.68	34101.33	3.570033	16.56223	1.749587	0.893103	1.962377	0.107916	0.615769	0.313787	660.636	3.866488	648.035	9.400522	605.2999	40.32906	660.6	3.9	109.1419	836
19AR116_Part2_Spot 195	186.065	16665.18	1.48057	16.39034	2.359046	0.899401	2.611555	0.10828	0.705463	0.270131	662.7529	4.443164	651.4074	12.55706	613.2132	54.31532	662.8	4.4	108.0787	700
19AR116_Spot 76	777.1362	105912.3	1.26756	16.13012	0.781867	0.923409	1.008834	0.10837	0.632375	0.626838	663.2719	3.985803	664.1613	4.917852	668.0931	16.84744	663.3	4.0	99.27836	68
19AR116_Part2_Spot 16	457.5187	41876.62	0.948172	16.31246	1.084112	0.910752	1.331327	0.108419	0.714032	0.536331	663.5608	4.502338	657.4571	6.443417	637.5283	24.17757	663.6	4.5	104.0833	545
19AR116_Part2_Spot 380	1711.016	102084.5	6.585756	15.97608	0.722212	0.930851	0.969703	0.108519	0.639113	0.659081	664.1396	4.033272	668.0824	4.746824	682.3137	15.57565	664.1	4.0	97.33639	857
19AR116_Spot 15	121.1345	23804.65	1.037709	15.04122	3.767942	0.989067	4.070665	0.10891	1.511197	0.371241	666.4158	9.567792	698.2441	20.55563	802.9849	79.20566	666.4	9.6	82.99233	13
19AR116_Part2_Spot 3	86.03403	7559.595	1.773935	15.42764	4.880365	0.948597	5.324338	0.108929	1.036327	0.19464	666.5274	6.562305	677.3719	26.32404	714.5341	111.0187	666.5	6.6	93.28141	533
19AR116_Spot 28	130.4392	25307.4	3.209643	16.4	2.459681	0.908576	2.672147	0.109051	0.657155	0.245928	667.234	4.165474	656.3006	12.91712	619.8154	55.91835	667.2	4.2	107.6504	25
19AR116_Spot 341	208.4786	26439.85	1.414864	16.18348	1.409854	0.919993	1.618904	0.109078	0.760253	0.469609	667.3918	4.820052	662.3562	7.876706	646.2082	30.71699	667.4	4.8	103.2781	303
19AR116_Part2_Spot 24	131.733	17179.36	0.819852	15.63679	4.681376	0.947102	4.831874	0.109217	0.894478	0.18512	668.199	5.677559	676.5922	23.86896	705.5353	101.0765	668.2	5.7	94.70809	552
19AR116_Part2_Spot 364	429.5303	46326.89	2.229058	15.96857	1.404953	0.939538	1.581238	0.109327	0.673852	0.426155	668.8411	4.281072	672.6405	7.77771	686.3352	30.51155	668.8	4.3	97.45109	842
19AR116_Spot 40	81.37103	12745.86	3.28207	15.44158	5.34015	0.965809	5.442435	0.110155	0.78796	0.144781	673.6491	5.040153	686.3014	27.15668	728.9469	114.225	673.6	5.0	92.41402	36
19AR116_Spot 538	1089.828	127874	3.512094	16.19863	0.625594	0.936231	0.923268	0.110163	0.668417	0.723968	673.6946	4.275772	670.9075	4.533007	662.4719	13.65294	673.7	4.3	101.6941	476
19AR116_Spot 237	286.7102	27724.98	2.524961	16.21327	1.745241	0.934781	1.941368	0.11063	0.726177	0.374054	676.4039	4.662978	670.1466	9.524199	650.0843	38.66539	676.4	4.7	104.0486	211
19AR116_Part2_Spot 137	598.7099	24542.5	5.977963	15.8488	1.196048	0.947928	1.442037	0.110686	0.633466	0.439285	676.7296	4.06951	677.0232	7.125509	678.909	27.68384	676.7	4.1	99.67899	650
19AR116_Part2_Spot 267	283.7536	22022.23	1.189801	16.27151	2.546278	0.920808	2.72746	0.110704	0.887474	0.325385	676.8371	5.702171	662.7872	13.27695	616.1997	55.70731	676.8	5.7	109.8405	760
19AR116_Spot 556	356.0841	61314.93	2.698492	16.25808	1.020926	0.937583	1.176049	0.110746	0.561161	0.477158	677.0803	3.606785	671.6163	5.778422	654.2643	22.16329	677.1	3.6	103.4873	493
19AR116_Spot 360	426.7806	65880.61	15.68812	16.24008	1.054597	0.936422	1.22497	0.110791	0.52851	0.431448	677.3398	3.398159	671.0076	6.014948	650.7177	23.74004	677.3	3.4	104.0912	320
19AR116_Part2_Spot 41	121.9081	12952.55	3.308796	14.82866	3.186407	1.006471	3.411697	0.110839	1.014717	0.297423	677.6202	6.526881	707.0897	17.37845	802.746	68.22678	677.6	6.5	84.41278	564
19AR116_Part2_Spot 102	123.7691	13978.42	2.010348	15.3985	4.852085	0.975428	5.113333	0.111165	0.701172	0.137126	679.5114	4.52203	691.2574	25.64249	730.6036	107.3859	679.5	4.5	93.00685	618
19AR116_Spot 225	210.313	22297.74	3.697489	15.82222	1.607456	0.95999	1.855064	0.111179	0.752044	0.405401	679.5917	4.850657	683.2912	9.226004	696.4053	36.14157	679.6	4.9	97.58566	199
19AR116_Spot 64	207.4479	49078.72	3.337454	15.68404	2.955342	0.967278	3.046105	0.111222	0.65291	0.214343	679.8413	4.212713	687.0596	15.20872	711.7138	63.23941	679.8	4.2	95.52173	57
19AR116_Spot 14	158.9744	18924.15	2.684242	15.51805	3.038464	0.980486	3.120655	0.111696	0.661694	0.212037	682.5917	4.285764	693.854	15.68844	731.466	64.62077	682.6	4.3	93.3183	12
19AR116_Spot 170	76.22472	15020.56	3.934498	15.03628	5.595558	1.01343	5.733703	0.111977	0.985618	0.171899	684.2186	6.398228	710.6055	29.31183	795.7879	118.5357	684.2	6.4	85.98002	153
19AR116_Spot 281	467.787	64081.69	1.546468	16.11655	1.062369	0.962714	1.210908	0.112352	0.573237	0.473394	686.3915	3.732419	684.7013	6.03096	680.0598	22.78969	686.4	3.7	100.931	249
19AR116_Part2_Spot 198	1407.984	15192.96	6.19997	15.66757	0.609397	0.976603	0.863328	0.112463	0.593064	0.686952	687.0363	3.864954	691.8615	4.331186	708.4928	13.33951	687.0	3.9	96.97153	702
19AR116_Part2_Spot 205	228.1236	27456.25	2.355596	15.54399	1.365813	0.991292	1.653529	0.112487	0.7553	0.456781	687.1765	4.923185	699.3795	8.358308	739.6932	31.12271	687.2	4.9	92.9002	708
19AR116_Part2_Spot 143	271.4241	21064.89	1.834098	15.96316	1.112253	0.95371	1.553296	0.112517	0.710792	0.457602	687.3515	4.63419	680.0328	7.699257	656.8328	29.64257	687.4	4.6	104.6463	656
19AR116_Spot 296	281.2942	13367.54	5.040512	15.66225	1.727703	0.977714	1.882237	0.112585	0.670407	0.356176	687.7424	4.373247	692.4322	9.448549	708.6363	37.40337	687.7	4.4	97.05154	263
19AR116_Part2_Spot 345	1191.162	16993.37	8.158899	15.56687	0.877586	0.978891	1.351867	0.112854	0.867959	0.642045	689.3024	5.674102	693.0362	6.790204	706.1164	22.04957	689.3	5.7	97.6188	824
19AR116_Spot 115	797.3256	16882.26	3.732228	15.92231	0.668735	0.966334	1.009259	0.11289	0.587002	0.581617	689.5109	3.838502	686.5727	5.036236	677.8925	17.53041	689.5	3.8	101.7139	105
19AR116_Spot 38	543.2144	71133.04	2.060879	16.0124	0.870896	0.97026	1.077034	0.113035	0.609956	0.56633	690.3506	3.993205	688.5978	5.385524	683.8251	18.95368	690.4	4.0	100.9543	34
19AR116_Spot 530	170.3199	27664.03	7.112721	15.32574	2.057222	1.011312	2.280028	0.113125	0.809444	0.355015	690.8735	5.302996	709.5365	11.64112	769.9508	44.90167	690.9	5.3	89.72957	468
19AR116_Part2_Spot 190	71.56399	7870.845	7.692337	15.27242	7.260202	0.993744	7.461651	0.113194	1.081494	0.14494	691.2712	7.089171	700.6289	37.78073	731.7011	156.5936	691.3	7.1	94.47454	696
19AR116_Part2_Spot 323	252.4242	19623.05	1.918488	15.85696	2.15201	0.968006	2.515401	0.113788	0.607461	0.241497	694.7078	4.000639	687.4355	12.56351	664.6606	52.2899	694.7	4.0	104.5207	805
19AR116_Part2_Spot 376	314.0561	37464.15	2.191746	16.15442	1.493143	0.961557	1.779396	0.113855	0.826509	0.464488	695.095	5.446129	684.1026	8.85702	649.0506	33.84149	695.1	5.4	107.0941	853
19AR116_Part2_Spot 177	310.9137	2039.496	1.957845	13.84158	2.080942	1.017618	4.128294	0.114022	0.979072	0.237161	696.0633	6.459926	712.7154	21.14507	766.4197	84.52194	696.1	6.5	90.82011	684
19AR116_Spot 582	161.4164	32422.43	2.489079	15.69934	2.97247	1.003038	3.167782	0.114117	1.029731	0.325064	696.6125	6.799254	705.351	16.1083	734.2209	63.45278	696.6	6.8	94.87778	515
19AR116_Part2_Spot 169	500.0666	53267.13	4.774946	15.61831	0.918075	0.995245	1.218553	0.114214	0.764002	0.626974	697.1756	5.048519	701.393	6.171825	715.8457	20.16411	697.2	5.0	97.39188	677
19AR116_Spot 434	286.9328	37424.07	2.467332	15.80073	1.474246	0.986973	1.660604	0.11423	0.621938	0.374525	697.2684	4.110276	697.1744	8.375661	697.7997	32.8029	697.3	4.1	99.92386	383
19AR116_Part2_Spot 172	198.9169	29632.97	1.558524	15.88609	2.888943	0.976503	3.024211	0.114388	0.801868	0.265149	698.1817	5.305976	691.8103	15.17227	672.1054	62.41322	698.2	5.3	103.8798	680
19AR116_Part2_Spot 64	749.289	14669.11	4.494298	15.71592	1.161615	0.989168	1.507127	0.11503	0.941034	0.624389	701.8963	6.258201	698.2955	7.610033	687.642	25.12151	701.9	6.3	102.0729	586
19AR116_Part2_Spot 369	1147.117	25767.67	7.235538	15.75185	0.719203	0.998588	1.026952	0.115066	0.709648	0.691024	702.1008	4.720707	703.0927	5.210109	707.1882	15.76838	702.1	4.7	99.28063	846
19AR116_Spot 155	89.64869	13988.84	1.556561	15.75921	3.750206	0.993288	3.876773	0.115266	0.709327	0.182968	703.2585	4.725933	700.3967	19.61822	692.1739	81.29361	703.3	4.7	101.6014	139
19AR116_Spot 446	190.1459	41989.36	1.792727	15.52886	3.044879	1.019459	3.138577	0.115772	0.696703	0.22198	706.1826	4.660086	713.6412	16.08917	738.0731	64.76673	706.2	4.7	95.67921	392
19AR116_Spot 347	422.1919	66439.12	3.353298	15.61046	1.148882	1.019193	1.305635	0.116134	0.587464	0.449945	708.2732	3.94042	713.5075	6.691704	730.9513	24.68545	708.3	3.9	96.89745	308
19AR116_Part2_Spot 287	155.5997	12762.25	3.757647	15.544	3.057427	1.012296	3.407218	0.116524	0.903124	0.265062	710.5238	6.075921	710.0334	17.40556	709.3873	69.85158	710.5	6.1	100.1602	778
19AR116_Part2_Spot 165	951.0899	105925.8	3.813154	15.83235	0.882755	1.00972	1.234754	0.11722	0.844035	0.683565	714.541	5.708748	708.7326	6.29914	691.2808	19.21693	714.5	5.7	103.3648	673
19AR116_Spot 393	1513.633	54370.91	21.81517	15.56548	0.60359	1.040839	0.878309	0.117355	0.631587	0.719095	715.3239	4.276253	724.3344	4.54836	753.2302	12.88532	715.3	4.3	94.9675	349
19AR116_Part2_Spot 375	164.4551	5032.218	1.322354	15.02355	3.161516	1.032924	4.766689	0.118115	0.927103	0.194496	719.704	6.313416	720.3892	24.59682	723.4273	99.25708	719.7	6.3	99.48531	852
19AR116_Part2_Spot 275	462.6597	59286.85	4.119749	15.0589	1.322323	1.073392	1.556319	0.118286	0.811259	0.521267	720.6883	5.531676	740.4031	8.181163	801.3916	27.84134	720.7	5.5	89.9296	767
19AR116_Spot 287	131.9763	3085.185	1.635245	14.3897	1.970564	1.067757	2.744929	0.119108	0.766584	0.279273	725.4299	5.259547	737.6398	14.39339	775.7905	55.46954	725.4	5.3	93.50848	255
19AR116_Part2_Spot 188	217.1172	15470.51	5.173401	14.60597	2.098558	1.114081	2.563935	0.119751	0.991032	0.386528	729.1279	6.832229	760.1364	13.72014	853.3711	49.1343	729.1	6.8	85.4409	694
19AR116_Part2_Spot 338	854.0673	17446.29	1.118729	15.24646	0.872649	1.068431	1.194167	0.120932	0.719662	0.602648	735.9258	5.005059	737.9704	6.263339	745.0802	20.14568	735.9	5.0	98.77136	817
19AR116_Spot 552	325.708	33487.88	1.12023	15.69688	0.88133	1.057097	1.130956	0.120955	0.588715	0.520546	736.0598	4.095057	732.3917	5.901204	722.0885	20.46898	736.1	4.1	101.9348	489
19AR116_Spot 451	143.2685	20117.24	2.646465	14.78384	2.320603	1.115292	2.486712	0.121083	0.81036	0.325876	736.7918	5.642096	760.7177	13.31369	832.5887	49.03043	736.8	5.6	88.49409	397
19AR116_Spot 563	46.81202	9574.952	2.721258	15.42821	6.314284	1.060872	6.415943	0.121088	1.074268	0.167437	736.8204	7.479821	734.2529	33.54751	727.3294	134.2393	736.8	7.5	101.3049	498
19AR116_Part2_Spot 295	195.8326	22746.77	2.374705	15.09449	2.982927	1.092378	3.063053	0.12137	0.660081	0.215498	738.4443	4.605516	749.6584	16.23881	784.1718	62.85984	738.4	4.6	94.16868	784
19AR116_Spot 46	29.77977	5545.892	1.298503	13.85494	7.056827	1.163699	7.411635	0.121402	1.728175	0.23317	738.6293	12.06067	783.6921	40.49654	915.1384	148.4955	738.6	12.1	80.7123	41
19AR116_Spot 504	163.4849	7510.212	4.451031	15.32548	2.367881	1.068626	2.759446	0.122592	0.947703	0.34344	745.4679	6.671635	738.0664	14.47521	716.5752	55.04649	745.5	6.7	104.0321	445
19AR116_Part2_Spot 61	69.42129	5684.146	1.516902	15.20055	5.638794	1.072059	6.689916	0.12377	1.1897	0.177835	752.227	8.446843	739.7499	35.15933	703.1063	140.2579	752.2	8.4	106.9862	583
19AR116_Spot 322	309.7589	36270.22	1.014574	15.3654	1.243624	1.103754	1.375963	0.1239	0.543	0.394633	752.9713	3.85888	755.1642	7.330296	762.5626	26.63187	753.0	3.9	98.74223	287
19AR116_Spot 89	173.1484	33472.16	1.708514	15.45003	2.078279	1.101168	2.184963	0.124451	0.655353	0.299938	756.1339	4.675763	753.9152	11.62749	748.2384	44.0433	756.1	4.7	101.0552	81
19AR116_Part2_Spot 21	324.8725	21880.26	3.346421	15.49357	1.680499	1.103122	1.846715	0.125303	0.687915	0.372508	761.0131	4.937926	754.859	9.835635	737.6097	36.25401	761.0	4.9	103.1729	550
19AR116_Spot 578	72.89523	9844.176	2.313389	14.38379	4.785201	1.194159	4.997942	0.126371	1.150504	0.230196	767.1297	8.320946	797.8868	27.62627	885.7	100.6102	767.1	8.3	86.61281	511
19AR116_Part2_Spot 379	54.20939	5120.303	1.747516	14.39197	5.41286	1.158356	5.85519	0.126479	1.461018	0.249525	767.7466	10.57472	781.1817	31.91779	820.6846	118.5116	767.7	10.6	93.54953	856
19AR116_Spot 493	129.6176	18811.38	2.749166	15.75054	2.977223	1.098564	3.165024	0.126615	0.771156	0.243649	768.5254	5.586891	752.6561	16.8248	706.6889	65.28983	768.5	5.6	108.7502	434
19AR116_Spot 210	625.78	67177.66	3.32307	15.45428	0.662471	1.129154	0.898867	0.12717	0.602844	0.670671	771.698	4.384468	767.3501	4.840325	755.6097	14.07035	771.7	4.4	102.1292	184
19AR116_Part2_Spot 19	233.7988	23971.67	2.841402	15.26758	2.020895	1.14195	2.172296	0.127687	0.741442	0.341317	774.6591	5.411962	773.434	11.75997	770.795	42.98744	774.7	5.4	100.5013	548
19AR116_Part2_Spot 141	134.1248	13737.99	2.279214	13.75008	2.454652	1.255776	2.65644	0.128047	0.762515	0.287044	776.714	5.579671	826.008	15.01681	962.0068	51.98746	776.7	5.6	80.73893	654
19AR116_Spot 190	95.83483	15688.59	3.454023	14.9009	3.424543	1.174145	3.647398	0.12861	0.799553	0.219212	779.9297	5.873482	788.5825	20.00334	814.0615	74.4362	779.9	5.9	95.80722	168
19AR116_Part2_Spot 337	101.4363	5197.138	1.763197	13.98437	2.658582	1.204873	3.208617	0.128825	0.953378	0.29713	781.1594	7.013861	802.8326	17.80533	864.3708	63.5737	781.2	7.0	90.37319	816
19AR116_Spot 50	388.1198	82986.89	4.095325	15.0986	0.686206	1.174181	0.904305	0.129228	0.559673	0.618898	783.4602	4.128834	788.5994	4.958927	804.0517	14.89291	783.5	4.1	97.43904	44
19AR116_Spot 224	102.7242	18911.16	2.500159	14.74828	3.892226	1.199486	4.009609	0.129611	0.847401	0.211343	785.6464	6.267876	800.3488	22.20624	842.423	81.5921	785.6	6.3	93.26032	198
19AR116_Part2_Spot 45	92.96804	10427.56	0.673891	14.17947	3.899671	1.238351	4.078512	0.130806	1.099533	0.269592	792.4618	8.1991	818.1341	22.91506	889.4867	81.16406	792.5	8.2	89.09204	567
19AR116_Spot 375	227.6119	41337.28	1.682566	15.46665	1.397704	1.160994	1.586896	0.130865	0.686284	0.432469	792.7995	5.119596	782.422	8.656959	753.8394	30.20523	792.8	5.1	105.1682	332
19AR116_Spot 480	192.1521	8716.452	1.984523	14.8835	1.78785	1.189945	2.239321	0.131568	1.266913	0.565758	796.8033	9.495862	795.935	12.35553	794.3975	38.73557	796.8	9.5	100.3029	422
19AR116_Spot 572	44.97543	6389.528	3.93739	14.80628	7.139909	1.189035	8.026683	0.131605	1.331662	0.165904	797.0128	9.983639	795.5128	44.29792	792.2059	166.2977	797.0	10.0	100.6068	506
19AR116_Spot 137	85.65885	13925.7	1.57711	14.65309	3.454185	1.221546	3.560717	0.131753	0.703704	0.19763	797.8567	5.281005	810.482	19.88279	846.2355	72.64084	797.9	5.3	94.28306	122
19AR116_Spot 284	80.06701	11998.63	3.345653	15.17434	4.26372	1.189173	4.353197	0.132917	0.782849	0.179833	804.4875	5.920796	795.577	24.01509	771.5908	90.19268	804.5	5.9	104.2635	252
19AR116_Spot 356	308.7831	37526.7	2.192788	15.03421	1.253905	1.21114	1.479571	0.133281	0.694062	0.469097	806.5573	5.261964	805.7147	8.229118	804.2776	27.38159	806.6	5.3	100.2834	316
19AR116_Part2_Spot 81	193.8378	22705.63	4.380161	14.7058	2.641641	1.249321	2.910896	0.135136	0.836082	0.287225	817.0974	6.41637	823.0981	16.41788	840.2307	58.05901	817.1	6.4	97.24678	600
19AR116_Spot 243	274.6934	78523.75	1.932426	15.17406	1.259811	1.227599	1.437378	0.135205	0.634536	0.441453	817.492	4.871844	813.2448	8.043212	802.5311	27.02079	817.5	4.9	101.8642	215
19AR116_Spot 91	126.7156	27153.98	4.485011	14.45056	2.986299	1.2881	3.068667	0.136294	0.68688	0.223837	823.6727	5.311113	840.4546	17.54274	885.9487	61.84007	823.7	5.3	92.9707	83
19AR116_Spot 418	151.4916	18681.13	4.437608	14.57191	2.485096	1.283491	2.75618	0.137258	1.03205	0.37445	829.1404	8.029694	838.4073	15.73134	863.958	53.01459	829.1	8.0	95.96999	369
19AR116_Part2_Spot 150	560.0024	39074.05	4.359077	14.6225	0.93281	1.280845	1.264254	0.137708	0.740067	0.585378	831.6863	5.774528	837.2302	7.208959	852.8508	21.29739	831.7	5.8	97.51838	661
19AR116_Spot 200	240.2402	45609.24	1.730325	14.9405	1.36918	1.269964	1.516968	0.138455	0.624368	0.411589	835.9209	4.894994	832.3744	8.617659	823.8463	28.85972	835.9	4.9	101.4656	176
19AR116_Part2_Spot 298	84.99088	11260.55	1.440311	15.02935	4.402196	1.245219	4.726777	0.13911	1.204034	0.254726	839.6262	9.478705	821.2449	26.62451	772.6815	96.25505	839.6	9.5	108.6639	787
19AR116_Part2_Spot 395	170.3067	13442.48	2.47215	13.91451	2.297276	1.359543	3.15917	0.139973	1.75871	0.5567	844.5118	13.92079	871.674	18.48485	942.2454	53.78552	844.5	13.9	89.62758	871
19AR116_Part2_Spot 59	306.6586	24428.91	1.259227	14.88546	1.370932	1.296668	1.61259	0.142191	0.705529	0.437513	857.0414	5.661965	844.2499	9.24478	811.6945	30.34669	857.0	5.7	105.5867	581
19AR116_Spot 205	185.9656	42538.96	1.080315	14.46035	1.674668	1.358589	1.836987	0.14321	0.680778	0.370595	862.7876	5.497566	871.263	10.74454	893.7574	35.228	862.8	5.5	96.53487	179
19AR116_Part2_Spot 2	337.7295	38911.75	1.129873	14.72827	1.323699	1.34448	1.528876	0.144012	0.65439	0.42802	867.3078	5.310337	865.1708	8.902688	860.5848	28.6639	867.3	5.3	100.7812	532
19AR116_Part2_Spot 135	207.4091	26072.1	1.186278	14.13324	1.37844	1.402573	1.646481	0.145814	0.862261	0.523699	877.4544	7.073622	890.0242	9.759981	922.3012	28.81532	877.5	7.1	95.13751	648
19AR116_Spot 527	226.3022	44764.63	1.930355	14.5548	1.380866	1.456102	1.605503	0.154487	0.805	0.501401	926.0692	6.944139	912.3984	9.664951	880.3477	28.72315	880.3	28.7	105.1936	465
19AR116_Spot 528	233.4495	30173.08	5.598101	14.45576	1.320784	1.44114	1.540158	0.152063	0.785205	0.509821	912.5184	6.681123	906.1941	9.232529	891.6923	27.36322	891.7	27.4	102.3356	466
19AR116_Part2_Spot 328	97.67802	3426.353	2.405132	13.89861	3.780216	1.378862	4.836655	0.14885	1.184804	0.244964	894.5158	9.895818	879.9535	28.47347	844.4257	97.61816	894.5	9.9	105.9319	810
19AR116_Spot 319	176.8647	31691.48	1.966496	14.39993	1.718401	1.477301	1.8039	0.155475	0.508552	0.281918	931.5819	4.411175	921.1246	10.92319	897.0408	35.72191	897.0	35.7	103.8506	284
19AR116_Spot 462	111.2655	39102.25	0.800742	14.48793	1.938396	1.517838	2.174456	0.159639	0.860938	0.395932	954.7717	7.640238	937.6054	13.31077	898.3626	41.19586	898.4	41.2	106.2791	406
19AR116_Spot 32	67.63406	15183.75	1.044971	14.82837	5.46195	1.377212	5.624428	0.15014	1.221268	0.217136	901.7455	10.27719	879.249	33.09748	823.9607	114.6781	901.7	10.3	109.4404	28
19AR116_Spot 326	95.00815	23895.62	1.335009	14.31783	3.163399	1.574693	3.31675	0.165044	0.58948	0.177728	984.7469	5.383257	960.2785	20.60026	905.5328	67.28056	905.5	67.3	108.7478	290
19AR116_Spot 277	159.2796	34229.83	1.642525	14.39636	1.488095	1.561689	1.656248	0.163577	0.678667	0.409762	976.6222	6.150374	955.1373	10.2527	906.8484	31.12296	906.8	31.1	107.6941	246
19AR116_Part2_Spot 193	104.2661	15982.76	0.986707	14.23642	2.55964	1.59048	2.896594	0.166273	0.829114	0.286238	991.5436	7.619986	966.4854	18.05973	910.7985	57.16587	910.8	57.2	108.8653	698
19AR116_Part2_Spot 365	73.65174	9343.551	1.15122	14.10336	3.046643	1.548129	3.584946	0.161804	0.756712	0.21108	966.7927	6.793673	949.748	22.11907	911.3299	72.17119	911.3	72.2	106.0859	843
19AR116_Spot 297	136.2193	26154.64	3.602875	14.32515	1.98082	1.431312	2.099426	0.149369	0.622988	0.296742	897.4251	5.219135	902.0976	12.55007	914.4731	41.26035	914.5	41.3	98.13576	264
19AR116_Part2_Spot 381	451.4301	52862.51	25.4121	14.2765	0.987083	1.45784	1.363393	0.152119	0.922175	0.676383	912.8309	7.849067	913.1168	8.211389	914.7239	20.68018	914.7	20.7	99.79306	858
19AR116_Spot 238	148.6974	26674.95	1.710794	14.30749	1.741708	1.594245	1.977474	0.166294	0.665858	0.336722	991.6612	6.12025	967.9602	12.33974	915.44	38.30625	915.4	38.3	108.3262	212
19AR116_Spot 525	141.2661	27057.59	1.040793	14.25331	2.583022	1.592978	2.73259	0.165988	0.841431	0.307924	989.9685	7.721819	967.4643	17.04733	917.5975	53.46214	917.6	53.5	107.887	463
19AR116_Spot 571	46.9432	11669.66	2.796167	14.14459	4.557412	1.584046	4.658579	0.164765	0.712461	0.152935	983.2051	6.496916	963.9602	29.00474	921.2347	94.68232	921.2	94.7	106.7269	505

19AR116_Part2_Spot 60	63.54971	6431.731	1.364892	13.8024	4.949026	1.545205	5.745586	0.160668	0.817284	0.142246	960.4878	7.293113	948.5822	35.43266	921.9693	116.9821	922.0	117.0	104.1779	582
19AR116_Spot 31	113.5069	28136.8	1.56086	14.21642	2.224233	1.590065	2.378929	0.165053	0.737701	0.310098	984.7967	6.737151	966.3229	14.83018	925.439	46.45747	925.4	46.5	106.414	27
19AR116_Part2_Spot 356	658.0976	14350.63	2.187485	14.1142	0.848888	1.404575	1.145462	0.14578	0.751995	0.656499	877.2636	6.16779	890.8697	6.793973	925.6773	17.76335	925.7	17.8	94.76992	834
19AR116_Spot 401	98.009	26100.89	3.127305	14.27838	2.448366	1.469363	2.585868	0.152485	0.756913	0.292711	914.8803	6.455901	917.8658	15.62481	925.9276	50.8014	925.9	50.8	98.80689	357
19AR116_Spot 410	76.43521	14648.41	1.033506	14.12559	4.51332	1.573041	4.622573	0.163218	0.897471	0.19415	974.6369	8.117949	959.6269	28.7027	926.2971	93.17557	926.3	93.2	105.2186	363
19AR116_Spot 523	164.9436	32080.66	3.351807	14.61245	1.762278	1.448805	1.9546	0.154559	0.826468	0.422832	926.4687	7.132184	909.3772	11.74256	868.9929	36.69185	926.5	7.1	106.6141	461
19AR116_Spot 203	34.65063	11157.19	0.497753	14.0343	6.97647	1.583994	7.227657	0.164213	1.147389	0.15875	980.1488	10.4329	963.9398	45.01672	928.0633	146.7197	928.1	146.7	105.6123	178
19AR116_Part2_Spot 132	221.9409	35599.8	0.736531	14.13836	1.622376	1.472968	1.834859	0.152555	0.757326	0.412743	915.2693	6.461979	919.3472	11.09749	930.0328	34.30971	930.0	34.3	98.41258	645
19AR116_Spot 168	201.0339	34628.55	1.138118	14.16651	1.607911	1.556875	1.745111	0.160676	0.659628	0.377986	960.5348	5.88652	953.2273	10.78981	937.2877	33.14176	937.3	33.1	102.4802	151
19AR116_Part2_Spot 318	133.4648	19301.81	1.979514	13.9307	1.714897	1.546867	1.944494	0.159279	0.694294	0.357056	952.7701	6.149402	949.2452	11.99233	941.9911	37.21945	942.0	37.2	101.1443	800
19AR116_Spot 269	467.6391	95120.18	6.113846	14.16466	0.823922	1.502029	1.052248	0.15451	0.646295	0.614204	926.1929	5.5758	931.2099	6.414163	943.9993	17.02755	944.0	17.0	98.11372	238
19AR116_Spot 348	237.3657	47607.39	1.672207	14.0786	1.329745	1.409981	1.514901	0.145019	0.704349	0.464947	872.9818	5.750674	893.1501	8.999641	944.3219	27.45343	944.3	27.5	92.44537	309
19AR116_Spot 516	582.9513	122927.5	2.124369	14.14076	0.723922	1.573537	0.986095	0.161808	0.66128	0.670604	966.8153	5.937023	959.8225	6.122102	944.7065	14.96328	944.7	15.0	102.3403	454
19AR116_Part2_Spot 17	77.99812	9485.581	1.855675	13.8699	2.523697	1.475616	2.991932	0.151686	0.929671	0.310726	910.4063	7.893298	920.4337	18.10997	945.4553	58.24491	945.5	58.2	96.2929	546
19AR116_Spot 164	260.8722	48318.28	1.346029	14.12518	1.308142	1.523698	1.442246	0.156519	0.597948	0.414595	937.4033	5.216703	939.9659	8.841826	946.8536	26.87106	946.9	26.9	99.00192	147
19AR116_Spot 159	436.4264	57375.95	21.4967	14.13184	0.992516	1.42701	1.181319	0.146559	0.606872	0.513724	881.6479	5.000718	900.2995	7.052767	947.2315	20.74166	947.2	20.7	93.07629	142
19AR116_Spot 127	49.71416	2096.652	1.30892	12.91976	5.020701	1.450552	5.34759	0.148939	1.068584	0.199825	895.0113	8.92972	910.1011	32.15159	947.7623	107.3346	947.8	107.3	94.43415	115
19AR116_Spot 42	79.97158	14357.91	3.559565	13.94696	2.210888	1.690985	2.532524	0.173563	0.690927	0.272821	1031.71	6.58719	1005.135	16.16027	948.5453	49.87984	948.5	49.9	108.7676	38
19AR116_Part2_Spot 63	325.1675	44542.13	2.250951	14.01171	1.211455	1.552682	1.596196	0.159208	0.948449	0.594194	952.3758	8.397246	951.5609	9.858608	950.5679	26.26145	950.6	26.3	100.1902	585
19AR116_Spot 149	117.6734	22208.04	3.343317	14.02516	2.2538	1.669709	2.462002	0.171164	0.696033	0.28271	1018.52	6.557564	997.0753	15.63614	951.0868	48.32663	951.1	48.3	107.0902	133
19AR116_Part2_Spot 223	709.1706	67199.34	9.498736	14.02607	0.728438	1.497927	1.307293	0.153346	1.074605	0.822008	919.6938	9.210447	929.5436	7.960174	953.8596	15.2457	953.9	15.2	96.41815	722
19AR116_Spot 388	181.9638	10907.38	2.4322	13.9058	1.566822	1.495901	1.793593	0.15301	0.693709	0.386771	917.8159	5.934486	928.7199	10.91556	955.5651	33.80314	955.6	33.8	96.04954	345
19AR116_Spot 235	123.5048	31752.87	0.74609	14.04132	2.14137	1.597395	2.328475	0.163371	0.752575	0.323205	975.4845	6.812796	969.1923	14.54137	955.8231	45.05806	955.8	45.1	102.057	209
19AR116_Part2_Spot 249	307.8171	53079.85	0.949076	13.98402	1.452562	1.592208	1.614626	0.162636	0.673569	0.417167	971.407	6.073966	967.1625	10.07038	958.3995	29.994	958.4	30.0	101.3572	745
19AR116_Part2_Spot 400	194.4489	17980.04	2.645604	13.86486	1.68249	1.48796	1.992977	0.151979	0.982776	0.49312	912.0479	8.358195	925.4841	12.10321	958.5079	35.43819	958.5	35.4	95.15288	874
19AR116_Part2_Spot 164	321.9407	4882.116	1.893919	13.40295	1.582019	1.563558	3.547359	0.159579	0.897224	0.252927	954.4347	7.959643	955.8778	21.97221	960.1086	70.12542	960.1	70.1	99.40903	672
19AR116_Part2_Spot 283	75.543	9038.666	0.664511	13.71994	3.765888	1.659829	3.938265	0.169389	1.083698	0.275171	1008.746	10.11938	993.3104	24.95928	960.2873	77.36831	960.3	77.4	105.0463	775
19AR116_Spot 390	513.1431	112154.9	4.384772	14.12042	0.773132	1.524407	1.038682	0.155558	0.692619	0.666825	932.0444	6.01054	940.2509	6.368833	960.4344	15.83455	960.4	15.8	97.04405	347
19AR116_Part2_Spot 31	219.7649	30330.16	1.491937	13.89171	1.514193	1.444373	1.690229	0.147311	0.68973	0.408069	885.8721	5.708889	907.5378	10.14149	961.5447	31.5154	961.5	31.5	92.13009	557
19AR116_Part2_Spot 268	194.9308	30147.47	2.311031	13.872	1.575299	1.577989	1.838097	0.160919	0.942569	0.512796	961.8796	8.422402	961.5777	11.42456	961.7958	32.24656	961.8	32.2	100.0087	761
19AR116_Spot 106	119.7685	25167.74	2.08712	13.94434	1.734193	1.683726	1.873398	0.171488	0.647894	0.345839	1020.307	6.113915	1002.393	11.93475	964.3301	35.88678	964.3	35.9	105.8047	96
19AR116_Part2_Spot 211	49.89991	5746.049	4.490555	13.57897	3.505052	1.545933	3.736519	0.157364	1.116195	0.298726	942.1113	9.783497	948.8724	23.04174	965.4972	72.82086	965.5	72.8	97.57784	712
19AR116_Spot 7	80.98116	17129.62	1.071404	13.82316	2.876727	1.722952	3.028062	0.175374	0.77828	0.257022	1041.651	7.485874	1017.126	19.45723	965.6087	59.75439	965.6	59.8	107.8751	7
19AR116_Spot 569	130.869	42351.91	0.97781	14.01443	1.488668	1.653875	1.639659	0.168287	0.626653	0.382185	1002.665	5.818969	991.0351	10.37578	966.2843	30.92225	966.3	30.9	103.765	503
19AR116_Spot 489	55.98308	12919.89	0.896108	13.85828	3.998737	1.746725	4.104562	0.177713	0.793178	0.193243	1054.468	7.715578	1025.953	26.50969	966.5132	82.24619	966.5	82.2	109.1002	431
19AR116_Spot 342	125.3422	28919.39	2.282888	13.91212	2.900316	1.73044	3.014034	0.17602	0.803188	0.266483	1045.195	7.749665	1019.915	19.3979	966.9262	59.30749	966.9	59.3	108.0946	304
19AR116_Spot 425	284.9483	31452.34	1.578991	13.87958	1.143625	1.580982	1.498772	0.160616	0.76879	0.512947	960.1983	6.858451	962.7557	9.322227	969.4698	26.27013	969.5	26.3	99.04365	376
19AR116_Part2_Spot 4	281.035	33597.12	4.444823	13.97189	1.133566	1.414533	1.397564	0.143629	0.735903	0.526562	865.1509	5.957939	895.066	8.313637	970.5887	24.23714	970.6	24.2	89.13671	534
19AR116_Part2_Spot 180	244.6978	24195.87	1.867942	13.83259	1.745025	1.549152	1.932976	0.157263	0.82018	0.42431	941.5475	7.184914	950.1555	11.92819	971.0548	35.69617	971.1	35.7	96.96132	687
19AR116_Spot 30	105.7066	16264.55	1.413722	13.82628	2.446684	1.664942	2.635518	0.168917	0.722275	0.274054	1006.141	6.728376	995.2604	16.72039	972.2296	51.68159	972.2	51.7	103.488	26
19AR116_Spot 121	39.32373	6883.609	1.992638	13.61268	6.820144	1.614151	7.116457	0.163731	1.314706	0.184742	977.4788	11.92411	975.7215	44.64645	972.6751	142.7956	972.7	142.8	100.4939	109
19AR116_Spot 33	76.28819	20157.92	2.126502	13.84522	2.818251	1.743977	3.048596	0.17685	0.754979	0.247648	1049.741	7.313688	1024.936	19.67635	973.256	60.24245	973.3	60.2	107.8587	29
19AR116_Spot 133	83.53893	1171.224	0.852173	11.94879	2.879558	1.668755	4.703723	0.169146	0.706043	0.150103	1007.406	6.584807	996.7122	29.87313	974.128	94.88409	974.1	94.9	103.4162	118
19AR116_Spot 274	111.0849	19766.58	2.875205	13.87193	2.123504	1.707101	2.282759	0.172989	0.676273	0.296252	1028.558	6.429309	1011.198	14.61756	974.6875	44.45424	974.7	44.5	105.5269	243
19AR116_Spot 234	229.065	51200.64	3.374494	13.94129	1.145659	1.589216	1.421254	0.160983	0.775422	0.54559	962.2395	6.931251	965.99	8.857827	975.4247	24.27585	975.4	24.3	98.64826	208
19AR116_Part2_Spot 91	201.4515	29493.69	3.557416	13.79946	1.154844	1.698428	1.466198	0.171999	0.674459	0.460006	1023.117	6.380773	1007.94	9.370685	975.9983	26.53082	976.0	26.5	104.8277	609
19AR116_Spot 441	385.913	18924.25	2.375864	13.76558	0.834532	1.661987	1.520659	0.168304	0.740471	0.486941	1002.76	6.876469	994.1339	9.64043	976.0548	27.05476	976.1	27.1	102.7361	388
19AR116_Part2_Spot 15	129.297	15340.4	0.92497	13.77585	1.864747	1.678563	2.300725	0.169976	0.769427	0.334428	1011.976	7.206019	1000.437	14.64066	976.1412	44.18073	976.1	44.2	103.6711	544
19AR116_Part2_Spot 234	291.5298	22735.71	1.388215	13.75737	1.620898	1.6158	1.977913	0.163611	1.060783	0.536315	976.8126	9.615011	976.3617	12.40628	976.254	34.01369	976.3	34.0	100.0572	732
19AR116_Spot 511	155.0692	29086.03	3.957246	13.85111	1.729095	1.508526	1.921722	0.152733	0.821783	0.427629	916.2643	7.019068	933.8429	11.73475	976.4437	35.38871	976.4	35.4	93.83688	450
19AR116_Part2_Spot 269	147.035	6124.478	2.620461	13.43018	2.149423	1.552822	2.58063	0.157159	1.121558	0.434606	940.9679	9.819422	951.6163	15.94015	977.2288	47.35924	977.2	47.4	96.28941	762
19AR116_Part2_Spot 18	245.8044	26699.67	4.379449	13.82914	1.459206	1.634742	1.631588	0.165439	0.682046	0.418026	986.933	6.24138	983.6883	10.27937	977.3277	30.20679	977.3	30.2	100.9828	547
19AR116_Spot 438	280.3057	56567.47	1.952728	13.84357	1.278032	1.672651	1.448608	0.169228	0.666262	0.459933	1007.855	6.216357	998.1935	9.205676	977.9386	26.19353	977.9	26.2	103.0591	386
19AR116_Spot 545	97.24162	13073.03	2.798707	13.72524	3.312483	1.661361	3.425781	0.167798	0.678977	0.198196	999.9663	6.289156	993.8952	21.71781	981.4269	68.42348	981.4	68.4	101.889	482
19AR116_Spot 399	98.77628	38012.94	2.011101	13.95947	2.271944	1.728645	2.377179	0.174559	0.682117	0.286944	1037.181	6.534985	1019.247	15.29268	981.8286	46.36593	981.8	46.4	105.6377	355
19AR116_Part2_Spot 94	383.3196	73138.68	1.297954	13.83199	0.776584	1.625819	0.970676	0.164163	0.565881	0.582977	979.8696	5.144046	980.2436	6.102626	981.987	16.04136	982.0	16.0	99.78437	611
19AR116_Part2_Spot 212	122.9931	16170.59	2.754604	13.77439	2.968115	1.718382	3.19264	0.173507	0.957982	0.30006	1031.405	9.130768	1015.421	20.49501	982.0121	62.01399	982.0	62.0	105.0298	713
19AR116_Spot 58	65.97419	1911.028	1.642085	12.57134	5.136411	1.369558	8.287227	0.138211	1.020706	0.123166	834.5395	7.989871	875.9744	48.67261	983.1111	167.7005	983.1	167.7	84.88761	52
19AR116_Spot 70	78.98597	11686.02	2.987355	13.61044	3.850138	1.429377	3.947562	0.144238	0.721231	0.182703	868.5831	5.860789	901.2895	23.58788	983.2521	79.06937	983.3	79.1	88.33778	63
19AR116_Part2_Spot 385	1037.196	235270.7	2.732934	13.81971	0.701278	1.650742	1.019934	0.166516	0.738357	0.723926	992.8853	6.794375	989.8355	6.449405	983.9663	14.3202	984.0	14.3	100.9064	862
19AR116_Spot 273	458.3721	127021.7	1.596447	13.90971	0.800185	1.612832	1.021328	0.162669	0.630363	0.617199	971.5947	5.685371	975.2091	6.401455	984.2313	16.33942	984.2	16.3	98.71609	242
19AR116_Spot 496	63.4046	17364.66	2.577954	13.78322	3.146614	1.676035	3.426147	0.168913	0.85629	0.249928	1006.122	7.976666	999.4782	21.79184	985.8193	67.53365	985.8	67.5	102.0595	437
19AR116_Part2_Spot 357	193.1486	22871.78	2.100997	13.80588	1.245336	1.676811	1.490313	0.168979	0.740224	0.49669	1006.483	6.897751	999.7728	9.47952	985.9743	26.32653	986.0	26.3	102.0801	835
19AR116_Spot 343	170.8151	52771.03	3.160536	13.81958	1.457733	1.759788	1.687329	0.177256	0.819413	0.485628	1051.967	7.953382	1030.77	10.92524	986.9255	30.01628	986.9	30.0	106.5903	305
19AR116_Spot 455	656.314	136100.9	2.014717	13.88364	0.809174	1.6413	0.988225	0.165266	0.565946	0.572689	985.9762	5.174306	986.2124	6.235359	987.6174	16.48367	987.6	16.5	99.83383	400
19AR116_Spot 302	208.8519	48527.73	2.500431	13.82862	1.659915	1.673154	1.867503	0.16839	0.812994	0.435338	1003.235	7.553261	998.3847	11.86924	988.6319	34.20401	988.6	34.2	101.4771	268
19AR116_Part2_Spot 40	368.3539	15283.23	1.430186	13.59958	0.96427	1.550929	1.363546	0.156044	0.646236	0.473938	934.7565	5.623192	950.8632	8.417892	989.1975	24.40511	989.2	24.4	94.49646	563
19AR116_Spot 594	162.7691	32710.76	2.619085	13.85774	1.168909	1.758524	1.394822	0.176801	0.681296	0.488446	1049.475	6.598357	1030.305	9.028833	990.7326	24.73429	990.7	24.7	105.9291	524
19AR116_Spot 379	82.63379	4697.66	4.462612	13.31294	2.775584	1.529405	4.250762	0.153738	0.764033	0.17974	921.8822	6.563029	942.2595	26.10337	991.0981	85.0717	991.1	85.1	93.01624	336
19AR116_Part2_Spot 278	85.71239	9794.095	0.777471	13.51078	4.397356	1.605442	4.928699	0.161352	0.99005	0.200875	964.2883	8.867214	972.3333	30.84673	991.445	98.23638	991.4	98.2	97.26089	770
19AR116_Spot 207	55.62048	18887.83	1.441684	13.70367	4.173339	1.737192	4.290122	0.174581	0.797874	0.185979	1037.302	7.644808	1022.422	27.65346	991.5767	85.76998	991.6	85.8	104.6113	181
19AR116_Spot 114	54.82107	13109.79	2.792314	13.67544	6.70387	1.751315	6.884154	0.175945	0.781838	0.113571	1044.785	7.540945	1027.648	44.52282	992.2258	139.2202	992.2	139.2	105.2971	104
19AR116_Part2_Spot 251	128.3183	14771.07	4.378427	13.60183	3.733908	1.704969	4.034457	0.171267	0.779325	0.193167	1019.089	7.346072	1010.398	25.82634	992.5058	80.53078	992.5	80.5	102.6784	746
19AR116_Part2_Spot 262	328.6935	31795.01	1.906402	13.66682	1.201559	1.741492	1.488054	0.174918	0.854463	0.574215	1039.154	8.20049	1024.016	9.598339	992.6718	24.76063	992.7	24.8	104.6825	755
19AR116_Spot 304	96.82544	17353.33	2.383047	13.71625	2.814528	1.596834	3.020278	0.160364	0.888428	0.294154	958.8002	7.915047	968.9729	18.86003	992.9984	58.69711	993.0	58.7	96.55607	270
19AR116_Spot 587	252.5338	37549.86	3.32579	13.89972	1.182531	1.62664	1.370386	0.163275	0.597537	0.436035	974.953	5.406562	980.5608	8.617359	994.0433	25.04986	994.0	25.0	98.07953	519
19AR116_Part2_Spot 153	229.8805	27359.9	2.753581	13.60914	1.223439	1.69107	1.597303	0.16974	1.0188	0.637825	1010.681	9.530229	1005.167	10.19221	994.0672	24.98728	994.1	25.0	101.6713	664
19AR116_Part2_Spot 219	98.49661	11755.38	1.967875	13.58989	5.008583	1.684787	5.162173	0.169107	0.952914	0.184595	1007.191	8.885465	1002.794	32.90407	994.0986	103.1937	994.1	103.2	101.317	718
19AR116_Part2_Spot 322	197.5258	27329.09	0.911899	13.61166	1.182609	1.689151	1.517445	0.169527	0.665283	0.438423	1009.505	6.216603	1004.443	9.678524	994.3189	27.70756	994.3	27.7	101.5273	804
19AR116_Spot 560	346.9554	7144.963	2.830027	13.49501	0.832396	1.557364	1.160677	0.156256	0.735265	0.633479	935.9395	6.405396	953.4212	7.17705	994.8872	18.24604	994.9	18.2	94.07494	496
19AR116_Spot 148	44.89766	11205	1.884134	13.60324	4.085228	1.670926	4.244073	0.167599	0.824436	0.194256	998.8676	7.628739	997.5379	26.96561	995.5002	84.65026	995.5	84.7	100.3383	132
19AR116_Spot 534	84.44411	17506.7	2.690631	13.69439	3.109454	1.556935	3.262229	0.156164	0.895138	0.274395	935.4222	7.794159	953.2512	20.17216	995.5141	63.77562	995.5	63.8	93.96374	472
19AR116_Spot 157	72.27754	24392.16	2.420182	13.73544	3.009001	1.747788	3.158455	0.175303	0.762283	0.241347	1041.266	7.329508	1026.345	20.4018	995.5404	62.3024	995.5	62.3	104.593	141
19AR116_Spot 407	173.3969	29268.41	2.411858	13.74786	1.568425	1.751335	1.805802	0.175576	0.708769	0.392496	1042.759	6.823969	1027.655	11.67199	996.5269	33.73018	996.5	33.7	104.6393	360
19AR116_Part2_Spot 147	123.3692	12616.14	2.697778	13.53276	2.580816	1.692256	2.745353	0.16965	0.748096	0.272495	1010.181	6.994765	1005.614	17.5235	996.5541	53.70006	996.6	53.7	101.3674	658
19AR116_Spot 593	107.1013	25897.11	3.308573	13.78498	2.142473	1.623742	2.254613	0.162768	0.585807	0.259826	972.1395	5.286258	979.4401	14.16857	996.7268	44.23667	996.7	44.2	97.53319	523
19AR116_Spot 449	137.1421	31975.67	3.525893	13.70477	1.565307	1.711348	1.72382	0.171531	0.635441	0.368624	1020.541	5.997664	1012.79	11.04821	996.9685	32.54059	997.0	32.5	102.3644	395
19AR116_Spot 337	54.04466	11483.07	2.776269	13.57732	5.654634	1.686238	5.809501	0.169005	1.115223	0.191965	1006.626	10.39354	1003.342	37.04552	997.0404	115.9098	997.0	115.9	100.9614	300
19AR116_Part2_Spot 176	189.0616	25759.33	1.404894	13.61501	1.434019	1.746506	1.626573	0.175019	0.725668	0.446133	1039.704	6.967807	1025.872	10.50288	997.3495	29.5568	997.3	29.6	104.2467	683
19AR116_Part2_Spot 162	539.5248	22085.02	3.493827	13.55585	0.887451	1.64493	1.285012	0.164808	0.872536	0.67901	983.4436	7.95842	987.6067	8.114833	997.7333	19.16531	997.7	19.2	98.56779	671
19AR116_Part2_Spot 178	79.20633	11295.51	1.543964	13.50655	3.753819	1.769268	3.924093	0.177259	1.032097	0.263016	1051.983	10.01787	1034.252	25.46179	997.8165	76.93734	997.8	76.9	105.4285	685
19AR116_Part2_Spot 166	435.2185	50793.84	13.04363	13.63485	0.905309	1.702859	1.156081	0.170533	0.715099	0.618554	1015.048	6.715984	1009.606	7.395734	998.6796	18.45222	998.7	18.5	101.639	674
19AR116_Spot 27	177.0318	6028.938	5.499246	13.37671	1.709549	1.652352	2.9788	0.165449	0.574363	0.192817	986.9875	5.256245	990.4522	18.84484	998.9988	59.37977	999.0	59.4	98.79767	24
19AR116_Part2_Spot 222	1809.806	17929.35	3.504125	13.63333	0.559452	1.5762	0.865295	0.157809	0.650043	0.751238	944.5911	5.711573	960.8727	5.375638	999.1882	11.5946	999.2	11.6	94.53586	721
19AR116_Part2_Spot 120	153.9421	27786.78	1.063841	13.68583	1.815368	1.656326	2.097186	0.165824	0.889384	0.424085	989.0604	8.154959	991.9723	13.27871	999.2837	38.55912	999.3	38.6	98.97694	634
19AR116_Spot 458	170.2866	38315.9	1.934205	13.76709	1.451582	1.665148	1.612045	0.166691	0.659803	0.409296	993.8513	6.076983	995.3389	10.22712	999.4827	29.86539	999.5	29.9	99.43658	402
19AR116_Spot 312	256.7136	55689.33	1.259921	13.78269	1.055448	1.651145	1.197395	0.165271	0.540312	0.451239	986.0045	4.940068	989.9901	7.572288	999.6991	21.68315	999.7	21.7	98.63013	278
19AR116_Spot 118	379.768	68722.6	1.321157	13.78645	0.82283	1.666883	1.01734	0.16683	0.591726	0.58164	994.6225	5.453882	995.9998	6.456589	999.8975	16.78686	999.9	16.8	99.47245	107
19AR116_Spot 423	198.9489	44545.04	4.227514	13.71287	1.409368	1.731985	1.536741	0.173346	0.571832	0.372107	1030.52	5.44596	1020.489	9.892591	999.8981	28.95795	999.9	29.0	103.0625	374
19AR116_Spot 419	66.86516	20297.54	2.372898	13.6457	3.095796	1.733698	3.315234	0.173433	0.826737	0.249375	1030.997	7.876966	1021.125	21.35163	1000.889	65.20644	1000.9	65.2	103.0081	370
19AR116_Spot 303	91.1062	28310.77	2.293217	13.71957	2.010991	1.81431	2.211767	0.181448	0.813836	0.367957	1074.879	8.057351	1050.634	14.47899	1001.439	41.74164	1001.4	41.7	107.3335	269
19AR116_Part2_Spot 310	92.47529	11778.22	1.253521	13.46717	3.190565	1.718253	3.43477	0.171782	0.724066	0.210805	1021.922	6.842697	1015.372	22.04921	1002.138	68.20147	1002.1	68.2	101.9741	794
19AR116_Spot 227	533.832	5253.911	3.584345	13.30092	0.887097	1.368196	1.50706	0.136775	1.147473	0.761398	826.3991	8.900053	875.3905	8.841017	1002.289	19.81565	1002.3	19.8	82.45116	201
19AR116_Spot 308	161.2921	35431.37	2.822974	13.73183	1.476326	1.698269	1.717752	0.169758	0.754686	0.439345	1010.779	7.060239	1007.88	10.97814	1002.448	31.31661	1002.4	31.3	100.8311	274
19AR116_Part2_Spot 87	166.8283	30992.54	1.331163	13.62595	2.034325	1.647073	2.20099	0.164634	0.771253	0.350412	982.4755	7.028202	988.4293	13.90663	1002.536	41.83219	1002.5	41.8	97.99899	606
19AR116_Part2_Spot 388	214.3731	22751.74	1.684824	13.585	1.614507	1.727558	1.849695	0.172649	0.761634	0.411762	1026.689	7.228699	1018.842	11.89621	1002.884	34.21884	1002.9	34.2	102.3736	865
19AR116_Part2_Spot 342	114.0576	17753.63	2.559766	13.51783	3.045708	1.781513	3.207791	0.178005	0.786528	0.245193	1056.069	7.661579	1038.732	20.86436	1003.291	63.13876	1003.3	63.1	105.2605	821
19AR116_Spot 491	157.0482	38046.76	3.921126	13.74625	1.249639	1.731079	1.512365	0.172914	0.788638	0.52146	1028.147	7.494803	1020.152	9.733801	1003.901	26.18086	1003.9	26.2	102.4152	433
19AR116_Spot 502	55.91262	15260.25	1.569252	13.61381	4.704346	1.741304	4.814199	0.173928	0.910002	0.189025	1033.718	8.691386	1023.947	31.06038	1003.985	95.99883	1004.0	96.0	102.9615	443
19AR116_Spot 286	85.27125	22773.89	10.18495	13.69289	1.978138	1.714907	2.13017	0.171287	0.692984	0.325319	1019.198	6.532846	1014.121	13.66331	1004.04	40.88491	1004.0	40.9	101.5097	254
19AR116_Spot 372	90.3384	24122.9	2.076188	13.67027	2.426123	1.736633	2.571545	0.173413	0.681653	0.265075	1030.887	6.49399	1022.215	16.5712	1004.557	50.34683	1004.6	50.3	102.621	331
19AR116_Part2_Spot 279	471.2916	4229.479	3.380812	13.08352	0.908858	1.480316	1.306863	0.147714	0.760973	0.58229	888.1385	6.313595	922.3596	7.919837	1005.978	21.55003	1006.0	21.6	88.28604	771
19AR116_Part2_Spot 210	103.8397	15120.01	1.062714	13.59864	3.164564	1.705374	3.359468	0.170163	0.778913	0.231856	1013.008	7.301729	1010.55	21.5059	1006.09	66.31996	1006.1	66.3	100.6876	711
19AR116_Part2_Spot 103	131.8437	21073.65	2.2268	13.59155	1.872839	1.834425	2.022003	0.183025	0.643487	0.318242	1083.481	6.417635	1057.866	13.28836	1006.248	38.89936	1006.2	38.9	107.6753	619
19AR116_Spot 521	265.3382	23198.69	4.404771	13.64401	1.158963	1.694793	1.460152	0.169032	0.795098	0.544531	1006.775	7.411083	1006.571	9.324629	1006.99	24.82674	1007.0	24.8	99.97868	459
19AR116_Part2_Spot 160	125.1902	19027.28	4.56796	13.47288	2.595744	1.755158	2.775189	0.175033	0.959609	0.345781	1039.782	9.214722	1029.065	17.95302	1007.217	52.85203	1007.2	52.9	103.2331	669
19AR116_Spot 461	134.6783	30831.75	2.478929	13.69745	1.409448	1.684765	1.627096	0.168005	0.789121	0.484987	1001.111	7.317117	1002.785	10.36791	1007.312	28.86416	1007.3	28.9	99.38446	405
19AR116_Spot 140	460.3933	81293.17	4.261167	13.72767	0.799378	1.712317	1.026777	0.170751	0.63431	0.617768	1016.247	5.963735	1013.153	6.581969	1007.335	16.36071	1007.3	16.4	100.8847	125
19AR116_Part2_Spot 66	191.5741	27987.23	6.889347	13.59089	1.894693	1.769606	2.194938	0.176431	0.96705	0.440582	1047.446	9.349221	1034.376	14.24096	1007.711	39.98256	1007.7	40.0	103.9431	588
19AR116_Spot 8	103.6417	26046.69	1.407339	13.59861	2.972459	1.762985	3.095836	0.175756	0.702055	0.226774	1043.747	6.76522	1031.946	20.06016	1007.883	61.19266	1007.9	61.2	103.5583	8
19AR116_Spot 132	203.2008	18352.26	2.122715	13.60017	1.173873	1.674836	1.398784	0.16691	0.691771	0.494552	995.0628	6.378603	999.0232	8.893376	1008.588	24.65159	1008.6	24.7	98.65897	117
19AR116_Part2_Spot 392	464.696	56951.62	2.626825	13.64597	1.067723	1.716516	1.406857	0.171063	0.854802	0.607597	1017.963	8.049312	1014.723	9.026657	1008.6	22.66305	1008.6	22.7	100.9283	868
19AR116_Part2_Spot 69	127.7982	16828.95	2.760707	13.51941	1.627527	1.758307	1.863836	0.175213	0.697303	0.374123	1040.769	6.701769	1030.225	12.06452	1008.769	35.0501	1008.8	35.1	103.1722	590
19AR116_Part2_Spot 273	137.7214	19973.21	1.542258	13.51485	1.920959	1.748278	2.140005	0.174123	0.671111	0.313602	1034.787	6.415856	1026.526	13.82361	1009.824	41.22852	1009.8	41.2	102.472	765
19AR116_Part2_Spot 329	189.2853	37526.1	3.461399	13.54563	1.589353	1.738525	1.816631	0.17307	0.761542	0.419205	1029.004	7.242858	1022.917	11.71063	1010.778	33.42263	1010.8	33.4	101.8031	811
19AR116_Spot 44	257.1437	70667.83	2.812342	13.67592	0.986901	1.726845	1.173149	0.171864	0.618578	0.52728	1022.371	5.848166	1018.577	7.543692	1011.292	20.19019	1011.3	20.2	101.0956	40
19AR116_Part2_Spot 248	338.7151	22946.29	1.082556	13.54064	0.962067	1.680235	1.258967	0.167224	0.69907	0.555273	996.7983	6.456297	1001.071	8.014022	1011.302	21.20935	1011.3	21.2	98.56584	744
19AR116_Part2_Spot 197	68.06874	13052.47	1.842144	13.53937	4.705859	1.684858	4.815017	0.167651	0.671525	0.139465	999.1574	6.215475	1002.821	30.69036	1011.699	96.69066	1011.7	96.7	98.76034	701
19AR116_Spot 232	117.586	28512.42	0.713759	13.63065	2.670902	1.760425	2.811577	0.175169	0.841817	0.299411	1040.528	8.088965	1031.004	18.20824	1011.718	54.4099	1011.7	54.4	102.8476	206
19AR116_Part2_Spot 352	243.7185	33430.69	2.784576	13.66991	1.18253	1.758733	1.444041	0.174998	0.649999	0.450125	1039.59	6.240607	1030.382	9.347856	1011.749	26.131	1011.7	26.1	102.7517	831
19AR116_Spot 266	309.3	55375.85	2.072009	13.68362	1.005408	1.694633	1.191086	0.168567	0.624163	0.524029	1004.211	5.804109	1006.511	7.606012	1012.384	20.5623	1012.4	20.6	99.19274	235
19AR116_Part2_Spot 326	305.5114	49922.66	1.832709	13.54691	1.026259	1.738523	1.20303	0.172907	0.607516	0.504988	1028.108	5.77331	1022.916	7.754948	1012.689	21.04483	1012.7	21.0	101.5226	808
19AR116_Spot 411	84.77271	20732.98	2.425405	13.60053	3.742907	1.787104	3.889944	0.177736	0.802935	0.206413	1054.596	7.811357	1040.771	25.33144	1012.715	77.16996	1012.7	77.2	104.1355	364
19AR116_Spot 230	117.0864	27990.12	3.317005	13.61897	2.372743	1.803259	2.486333	0.179299	0.720917	0.289952	1063.143	7.065733	1046.639	16.24131	1013.212	48.23397	1013.2	48.2	104.928	204
19AR116_Part2_Spot 140	937.7553	62652.85	6.026123	13.55969	0.621207	1.710735	0.906982	0.17009	0.651207	0.717993	1012.605	6.102341	1012.56	5.812047	1013.325	12.79471	1013.3	12.8	99.92898	653
19AR116_Spot 66	144.1268	38686.72	3.904397	13.55293	1.887452	1.671625	2.087196	0.166157	0.862275	0.413126	990.9042	7.920022	997.8038	13.26115	1013.86	38.53607	1013.9	38.5	97.73581	59
19AR116_Spot 134	129.7406	23467.18	1.277624	13.59462	2.061023	1.773066	2.204633	0.1762	0.649295	0.294514	1046.179	6.27024	1035.644	14.31396	1014.332	42.71715	1014.3	42.7	103.1397	119
19AR116_Part2_Spot 327	302.1577	52022.92	2.433435	13.53678	1.131372	1.729013	1.410001	0.171805	0.776084	0.550414	1022.049	7.335128	1019.384	9.070963	1014.528	23.83627	1014.5	23.8	100.7413	809
19AR116_Part2_Spot 30	286.8722	54357.29	9.145323	13.57368	0.999562	1.714656	1.230056	0.170362	0.70062	0.569584	1014.107	6.574371	1014.028	7.889068	1014.72	20.46701	1014.7	20.5	99.93957	556
19AR116_Spot 124	333.374	13734.02	1.865035	13.51527	0.893389	1.606343	1.153514	0.159591	0.615469	0.53356	954.5012	5.460432	972.6844	7.218833	1014.849	19.7673	1014.8	19.8	94.05352	112
19AR116_Spot 363	374.8326	105682.6	1.31192	13.68339	0.714345	1.710777	0.891113	0.169937	0.519265	0.582715	1011.762	4.862195	1012.576	5.710407	1015.199	14.67327	1015.2	14.7	99.66143	323
19AR116_Spot 333	97.11552	26604.56	1.431333	13.57559	2.523666	1.680959	2.609886	0.166972	0.57316	0.219611	995.4063	5.286613	1001.345	16.61718	1015.232	51.59613	1015.2	51.6	98.04716	296
19AR116_Spot 142	445.8639	60672.72	1.606418	13.65889	0.771376	1.693085	0.998318	0.168122	0.630285	0.631347	1001.755	5.847784	1005.927	6.372839	1015.89	15.68481	1015.9	15.7	98.60861	127
19AR116_Part2_Spot 221	183.6042	24088.85	3.209259	13.55913	1.649784	1.710066	2.188153	0.169796	1.368328	0.625334	1010.987	12.80342	1012.309	14.02065	1016.035	34.59453	1016.0	34.6	99.50315	720
19AR116_Part2_Spot 152	264.761	42512.76	1.115085	13.49484	1.413929	1.628546	1.682644	0.161615	0.849296	0.504739	965.7454	7.617223	981.2975	10.58577	1017.122	29.44368	1017.1	29.4	94.94878	663
19AR116_Spot 463	303.8927	7787.185	4.622182	13.38216	0.974688	1.625927	1.299785	0.161352	0.721785	0.555311	964.2835	6.464509	980.2854	8.172016	1017.167	21.8913	1017.2	21.9	94.80095	407
19AR116_Spot 264	69.25284	3571.573	1.591443	12.98963	4.290121	1.371297	6.677617	0.136079	0.813507	0.121826	822.451	6.281473	876.7193	39.22952	1017.227	134.3813	1017.2	134.4	80.85224	234
19AR116_Part2_Spot 144	171.455	32240.1	2.636012	13.49727	1.482782	1.677586	1.705052	0.166456	0.79076	0.463775	992.5534	7.27434	1000.067	10.84739	1017.437	30.60572	1017.4	30.6	97.55424	657
19AR116_Spot 384	182.2922	54338.97	2.044002	13.67033	1.473647	1.7779	1.627277	0.176384	0.68459	0.420697	1047.192	6.61698	1037.412	10.57542	1017.722	29.90051	1017.7	29.9	102.8957	341
19AR116_Spot 409	346.7317	71313.64	9.735199	13.65738	0.834602	1.870711	1.080697	0.18559	0.679606	0.628859	1097.442	6.85798	1070.782	7.15086	1017.744	17.01815	1017.7	17.0	107.8309	362
19AR116_Part2_Spot 233	336.1599	37377	1.218793	13.54294	1.379499	1.763773	1.682995	0.174971	0.927381	0.55103	1039.44	8.902559	1032.235	10.90611	1017.864	28.46546	1017.9	28.5	102.1197	731
19AR116_Spot 59	119.6633	25330.58	2.251219	13.53233	1.778004	1.774644	1.926461	0.17604	0.618951	0.321289	1045.305	5.972608	1036.221	12.51169	1017.968	36.95127	1018.0	37.0	102.6854	53
19AR116_Part2_Spot 261	115.7631	19537.69	2.121073	13.46053	2.160415	1.812303	2.384204	0.179773	0.806155	0.338123	1065.737	7.918879	1049.91	15.60186	1017.994	45.44772	1018.0	45.4	104.6899	754
19AR116_Spot 537	164.3425	46723.21	0.812436	13.63344	2.069771	1.766085	2.206301	0.175115	0.684854	0.310408	1040.231	6.578984	1033.084	14.3044	1018.849	42.47512	1018.8	42.5	102.0986	475

19AR116_Spot 567	138.5979	24004.4	2.362261	13.6088	1.542357	1.757091	1.70393	0.174201	0.658792	0.386631	1035.214	6.300486	1029.777	11.0266	1019.108	31.84122	1019.1	31.8	101.5805	501
19AR116_Spot 52	77.42993	14366.07	2.395122	13.44193	3.534168	1.730537	3.83793	0.171507	0.795176	0.207189	1020.408	7.504442	1019.95	24.70276	1019.831	76.06525	1019.8	76.1	100.0566	46
19AR116_Spot 368	183.3033	25604.99	6.500144	13.57154	1.337883	1.752237	1.536219	0.173645	0.687645	0.447622	1032.16	6.558541	1027.988	9.93125	1019.983	27.83347	1020.0	27.8	101.1939	328
19AR116_Part2_Spot 73	304.828	33736.58	4.045983	13.50359	0.890552	1.748206	1.125079	0.17313	0.593475	0.527496	1029.332	5.646069	1026.5	7.267141	1021.329	19.34866	1021.3	19.3	100.7836	593
19AR116_Part2_Spot 117	351.5181	58625.38	1.515546	13.58765	1.441326	1.708925	1.688217	0.169229	0.83689	0.495724	1007.862	7.808398	1011.882	10.81436	1021.457	29.69975	1021.5	29.7	98.66898	631
19AR116_Spot 295	456.9701	98726.63	1.365665	13.67199	0.885235	1.764116	1.112285	0.174674	0.663648	0.596653	1037.81	6.361598	1032.361	7.208154	1021.697	18.06846	1021.7	18.1	101.5771	262
19AR116_Part2_Spot 265	51.00489	6241.767	2.071508	13.13803	4.934911	1.721677	5.103251	0.170444	1.10912	0.217336	1014.56	10.41188	1016.651	32.79011	1022.018	100.8731	1022.0	100.9	99.27022	758
19AR116_Spot 23	157.3639	19949.42	2.688676	13.51701	1.478076	1.741829	1.673842	0.172416	0.709189	0.42369	1025.408	6.723202	1024.141	10.79757	1022.295	30.69068	1022.3	30.7	100.3045	21
19AR116_Spot 435	113.1517	20466.3	2.553308	13.46379	2.755974	1.729231	2.917218	0.1711	0.870108	0.298266	1018.169	8.19497	1019.465	18.76986	1023.112	56.35354	1023.1	56.4	99.51682	384
19AR116_Spot 381	607.7065	25830.87	2.423195	13.54449	0.691046	1.761511	0.939101	0.174172	0.587193	0.625272	1035.054	5.614938	1031.404	6.082556	1024.533	14.82896	1024.5	14.8	101.027	338
19AR116_Part2_Spot 95	501.1686	53060.14	1.603886	13.52471	0.787172	1.733789	1.089301	0.171414	0.74853	0.687166	1019.896	7.060947	1021.159	7.014809	1024.731	16.01112	1024.7	16.0	99.52814	612
19AR116_Part2_Spot 292	138.2578	20648.69	1.973773	13.47097	1.857138	1.768262	2.073648	0.174799	0.754729	0.363962	1038.501	7.239118	1033.883	13.45023	1024.99	39.09733	1025.0	39.1	101.3182	781
19AR116_Spot 256	94.83074	23075.76	1.222209	13.52186	2.282132	1.692627	2.448689	0.167263	0.664397	0.271328	997.0145	6.137308	1005.755	15.63086	1025.709	47.69722	1025.7	47.7	97.20249	227
19AR116_Part2_Spot 232	160.007	22369.85	1.711734	13.42724	1.66231	1.721167	1.947956	0.170083	0.986398	0.506376	1012.567	9.243033	1016.46	12.5112	1025.717	33.98664	1025.7	34.0	98.71805	730
19AR116_Part2_Spot 225	278.1619	46573.86	10.08826	13.51659	1.267478	1.729264	1.469878	0.170858	0.737548	0.501775	1016.839	6.938101	1019.477	9.456697	1026.006	25.73928	1026.0	25.7	99.10658	724
19AR116_Spot 487	318.6522	63337.09	2.704933	13.58572	0.892342	1.764186	1.085294	0.174305	0.609421	0.561526	1035.788	5.831291	1032.387	7.033333	1026.05	18.16547	1026.0	18.2	100.9491	429
19AR116_Part2_Spot 307	328.5013	58157.77	8.861973	13.46127	0.98177	1.756424	1.193028	0.173484	0.652011	0.546518	1031.281	6.213788	1029.531	7.719197	1026.677	20.2274	1026.7	20.2	100.4485	792
19AR116_Spot 150	133.7743	23167.15	3.029613	13.52091	1.855508	1.801053	1.996285	0.17788	0.706638	0.353976	1055.383	6.879257	1045.84	13.03411	1026.818	37.7852	1026.8	37.8	102.7819	134
19AR116_Spot 90	413.3659	88957.54	9.069856	13.56162	0.71296	1.755375	0.948326	0.173359	0.604482	0.63742	1030.591	5.757272	1029.145	6.134542	1026.934	14.77831	1026.9	14.8	100.3561	82
19AR116_Spot 465	1250.078	63382.96	21.00423	13.60901	0.604224	1.733755	0.836548	0.17121	0.575112	0.687482	1018.777	5.419586	1021.147	5.387076	1027.089	12.28681	1027.1	12.3	99.19071	409
19AR116_Spot 65	383.5122	78145.72	1.498859	13.49561	0.946268	1.78046	1.152004	0.17578	0.636805	0.552781	1043.88	6.137176	1038.347	7.490438	1027.58	19.43408	1027.6	19.4	101.5862	58
19AR116_Spot 355	211.4952	41946.76	1.788184	13.50272	1.203134	1.72406	1.429204	0.170198	0.735918	0.514915	1013.203	6.899911	1017.539	9.184844	1027.743	24.79715	1027.7	24.8	98.58522	315
19AR116_Spot 482	25.96851	4616.64	2.187853	13.07405	6.892494	1.765163	7.155477	0.174254	1.480226	0.206866	1035.506	14.16014	1032.746	46.41249	1027.766	141.7518	1027.8	141.8	100.7531	424
19AR116_Part2_Spot 32	510.2836	46857.34	4.365897	13.47909	0.839075	1.769815	1.053478	0.174712	0.598301	0.56793	1038.019	5.736258	1034.453	6.835007	1027.784	17.55324	1027.8	17.6	100.9958	558
19AR116_Spot 413	390.1655	83874.61	1.715023	13.5765	0.761693	1.807912	1.01584	0.1784	0.661981	0.651658	1058.226	6.460488	1048.323	6.641335	1028.607	15.57999	1028.6	15.6	102.8795	365
19AR116_Part2_Spot 170	204.4579	40765.31	1.996826	13.42232	1.305296	1.796546	1.559278	0.177264	0.772324	0.495309	1052.01	7.496608	1044.205	10.17148	1028.769	27.40906	1028.8	27.4	102.2592	678
19AR116_Spot 338	200.8367	56978.03	2.014582	13.53572	1.418583	1.787263	1.651563	0.176339	0.82951	0.502257	1046.944	8.015969	1040.829	10.75355	1028.871	28.89679	1028.9	28.9	101.7566	301
19AR116_Spot 141	277.4821	64785.12	3.066874	13.57341	0.919274	1.74725	1.126376	0.172376	0.638687	0.567028	1025.188	6.05363	1026.146	7.274071	1029.051	18.77757	1029.1	18.8	99.62465	126
19AR116_Spot 169	289.1131	56985.87	3.066524	13.57233	0.902818	1.687299	1.117232	0.166426	0.653058	0.584532	992.3865	6.006656	1003.743	7.122891	1029.487	18.34611	1029.5	18.3	96.39621	152
19AR116_Spot 421	435.3538	60195.13	5.990806	13.54032	0.74133	1.824413	0.978466	0.17994	0.589749	0.602728	1066.646	5.797662	1054.273	6.417654	1029.596	15.80509	1029.6	15.8	103.5985	372
19AR116_Part2_Spot 359	147.7091	2705.319	1.095746	12.70408	2.423757	1.721956	2.736224	0.169824	0.804245	0.293925	1011.14	7.526353	1016.755	17.57786	1029.726	52.88095	1029.7	52.9	98.19502	837
19AR116_Spot 174	221.1564	77065.33	2.462616	13.59407	1.107445	1.74281	1.279671	0.171873	0.609278	0.476121	1022.423	5.760515	1024.504	8.256428	1029.813	22.77048	1029.8	22.8	99.28245	157
19AR116_Spot 346	186.7811	53247.73	1.298146	13.50691	1.510717	1.733846	1.63604	0.170982	0.595536	0.364011	1017.521	5.605661	1021.18	10.536	1029.894	30.82645	1029.9	30.8	98.79857	307
19AR116_Part2_Spot 6	241.1569	28788.58	1.479911	13.55807	1.393655	1.696458	1.607725	0.167282	0.745144	0.463478	997.121	6.88388	1007.198	10.27084	1030.047	28.81958	1030.0	28.8	96.80342	536
19AR116_Spot 102	227.5986	23899.74	1.52918	13.48474	1.036757	1.753864	1.214422	0.172939	0.623476	0.513394	1028.286	5.925932	1028.588	7.853467	1030.091	21.08694	1030.1	21.1	99.82483	93
19AR116_Part2_Spot 281	506.6985	43396.34	3.660722	13.46158	0.716479	1.708835	1.023893	0.16846	0.723325	0.706446	1003.623	6.72258	1011.848	6.558557	1030.557	14.66772	1030.6	14.7	97.3865	773
19AR116_Spot 206	612.5126	202113.5	2.964197	13.57659	0.581272	1.753538	0.848404	0.172866	0.615879	0.725926	1027.881	5.851593	1028.468	5.486067	1030.576	11.79539	1030.6	11.8	99.73853	180
19AR116_Spot 69	199.5811	48483.18	2.304357	13.46647	1.131554	1.769325	1.349797	0.17441	0.689893	0.511108	1036.363	6.60467	1034.273	8.756751	1030.718	23.47031	1030.7	23.5	100.5477	62
19AR116_Part2_Spot 124	220.7265	26309.37	1.966323	13.48077	1.360705	1.715676	1.625757	0.169112	0.853431	0.524944	1007.218	7.958026	1014.409	10.42937	1030.828	27.99039	1030.8	28.0	97.70957	637
19AR116_Part2_Spot 109	163.4292	24340.98	1.497782	13.48132	1.769264	1.750849	2.0195	0.17257	0.706821	0.349998	1026.258	6.705866	1027.476	13.05207	1030.93	38.24784	1030.9	38.2	99.54675	623
19AR116_Spot 260	616.2567	107148.6	3.34035	13.57562	0.742408	1.716379	0.990682	0.169136	0.640919	0.646947	1007.348	5.977119	1014.672	6.356132	1031.373	15.28827	1031.4	15.3	97.6706	231
19AR116_Part2_Spot 239	304.4969	47956.52	7.954553	13.46069	0.996188	1.785178	1.32394	0.175871	0.809751	0.611622	1044.38	7.807378	1040.069	8.616626	1031.879	21.18826	1031.9	21.2	101.2115	736
19AR116_Part2_Spot 247	264.8474	55818.26	6.223761	13.47066	0.857719	1.825814	1.117729	0.179873	0.656519	0.587369	1066.278	6.452018	1054.776	7.333089	1031.904	18.30176	1031.9	18.3	103.3311	743
19AR116_Part2_Spot 128	422.759	62577.39	2.15492	13.51143	0.830605	1.781437	1.124228	0.175488	0.731029	0.65025	1042.28	7.035309	1038.704	7.311277	1032.047	17.28049	1032.0	17.3	100.9915	641
19AR116_Spot 217	239.2533	71294.47	11.05101	13.53447	1.229802	1.793266	1.415765	0.17662	0.69698	0.492299	1048.484	6.744391	1043.013	9.229231	1032.43	24.92345	1032.4	24.9	101.5549	191
19AR116_Part2_Spot 308	340.312	28585.94	4.074359	13.3959	0.988952	1.777917	1.229066	0.175095	0.663785	0.540072	1040.123	6.375974	1037.418	7.987417	1032.585	20.92308	1032.6	20.9	100.73	793
19AR116_Spot 145	164.6768	33259.69	1.510889	13.50904	1.408949	1.799642	1.577536	0.177182	0.660936	0.418967	1051.562	6.412894	1045.328	10.29693	1033.184	28.96384	1033.2	29.0	101.7788	129
19AR116_Spot 255	68.04043	19025.46	2.324129	13.44452	3.483556	1.794235	3.569673	0.176595	0.732797	0.205284	1048.345	7.090105	1043.365	23.27828	1033.812	70.6065	1033.8	70.6	101.4058	226
19AR116_Spot 154	158.9961	36050.36	2.031031	13.51255	1.445447	1.766407	1.616165	0.173829	0.642974	0.397839	1033.176	6.138047	1033.202	10.47866	1034.119	29.97696	1034.1	30.0	99.90879	138
19AR116_Spot 222	220.9623	54653.02	1.809386	13.50326	1.460085	1.789698	1.651224	0.176109	0.722196	0.43737	1045.682	6.971193	1041.715	10.7566	1034.265	30.01986	1034.3	30.0	101.1038	196
19AR116_Spot 229	219.3804	56460.88	5.438948	13.52425	1.11401	1.768499	1.31567	0.174003	0.684539	0.520297	1034.13	6.540397	1033.97	8.533902	1034.493	22.71913	1034.5	22.7	99.96487	203
19AR116_Spot 370	92.2216	7757.141	1.063376	13.24873	2.161147	1.824215	2.640307	0.179476	0.83479	0.316171	1064.111	8.188655	1054.202	17.31828	1034.595	50.62492	1034.6	50.6	102.8529	330
19AR116_Spot 39	768.885	10629.53	6.432723	13.31234	0.680141	1.800258	1.123816	0.177079	0.62208	0.553543	1050.996	6.032896	1045.552	7.336172	1035.053	18.92582	1035.1	18.9	101.5403	35
19AR116_Spot 183	629.3883	196166	2.691856	13.57581	0.656151	1.787624	0.951091	0.175825	0.686951	0.722276	1044.125	6.621881	1040.96	6.192979	1035.183	13.30608	1035.2	13.3	100.8638	162
19AR116_Spot 566	174.3476	42654.17	2.728739	13.5482	1.234708	1.760563	1.376817	0.173148	0.581477	0.422334	1029.43	5.532409	1031.055	8.916024	1035.363	25.22829	1035.4	25.2	99.42698	500
19AR116_Spot 447	466.6718	75836.46	3.313777	13.48426	0.836284	1.797718	1.083744	0.176776	0.664285	0.612954	1049.337	6.432828	1044.63	7.071007	1035.658	17.31535	1035.7	17.3	101.3208	393
19AR116_Part2_Spot 370	445.3569	4782.076	6.702871	13.03374	0.894938	1.570775	2.325186	0.154433	2.004571	0.862112	925.7633	17.28667	958.7324	14.4267	1036.013	23.81665	1036.0	23.8	89.35824	847
19AR116_Spot 248	270.8464	58879.67	2.20471	13.54498	1.016402	1.760025	1.161191	0.173033	0.535596	0.461247	1028.8	5.093001	1030.857	7.518779	1036.084	20.82812	1036.1	20.8	99.29697	219
19AR116_Part2_Spot 235	166.8108	2770.844	1.146271	12.60149	2.231697	1.767083	3.577369	0.173711	1.12074	0.313286	1032.526	10.69276	1033.451	23.20086	1036.267	68.61637	1036.3	68.6	99.63904	733
19AR116_Spot 173	266.8782	59361.56	1.856416	13.53299	1.188789	1.776019	1.363125	0.174544	0.603857	0.442995	1037.098	5.784794	1036.724	8.855272	1036.795	24.69986	1036.8	24.7	100.0292	156
19AR116_Spot 396	384.5547	39989.3	2.882293	13.56072	0.842587	1.749715	1.026015	0.171958	0.572666	0.558146	1022.892	5.416648	1027.057	6.629327	1036.8	17.21228	1036.8	17.2	98.65852	352
19AR116_Part2_Spot 1	104.8511	13832.98	2.589275	13.39965	2.893265	1.766337	3.184043	0.173589	0.854753	0.268449	1031.854	8.150127	1033.177	20.64606	1036.837	61.95078	1036.8	62.0	99.51934	531
19AR116_Spot 500	107.695	22040.98	2.475706	13.4793	1.791976	1.748787	1.975163	0.171856	0.701945	0.355386	1022.331	6.636101	1026.714	12.76003	1036.925	37.30628	1036.9	37.3	98.59258	441
19AR116_Part2_Spot 191	518.2949	3601.623	10.34033	12.86637	2.205065	1.734076	5.479561	0.17041	0.685745	0.125146	1014.369	6.436326	1021.266	35.30271	1036.935	109.8787	1036.9	109.9	97.82384	697
19AR116_Part2_Spot 129	230.0864	27587.83	2.848739	13.39721	1.112807	1.734543	1.315447	0.17044	0.68857	0.523449	1014.534	6.463805	1021.439	8.472538	1037.125	22.65391	1037.1	22.7	97.82175	642
19AR116_Spot 499	132.2096	27098.73	2.809977	13.51578	1.642191	1.810724	1.755193	0.17791	0.597138	0.340212	1055.548	5.814097	1049.34	11.48173	1037.298	33.35054	1037.3	33.4	101.7595	440
19AR116_Part2_Spot 54	155.1548	17091.28	1.451268	13.30093	1.847918	1.760021	1.997029	0.172894	0.649877	0.325422	1028.039	6.175489	1030.856	12.93133	1037.697	38.1526	1037.7	38.2	99.06925	576
19AR116_Part2_Spot 131	193.0996	25350.34	2.864379	13.38527	1.645184	1.762809	1.810361	0.173168	0.725545	0.400774	1029.542	6.903826	1031.881	11.72921	1037.701	33.5131	1037.7	33.5	99.21379	644
19AR116_Spot 559	322.3399	24460.18	1.924345	13.47112	0.918756	1.738488	1.204725	0.170771	0.600567	0.49851	1016.358	5.647057	1022.903	7.765819	1037.794	21.1064	1037.8	21.1	97.93444	495
19AR116_Spot 553	273.0143	108491.4	2.189913	13.54708	1.050361	1.740387	1.228008	0.170941	0.617889	0.503163	1017.294	5.814869	1023.607	7.919064	1037.989	21.4486	1038.0	21.4	98.00628	490
19AR116_Spot 503	98.55714	30981.17	99.75912	13.43445	3.20576	1.809427	3.303583	0.177708	0.70692	0.213986	1054.44	6.876339	1048.871	21.60747	1038.152	65.16622	1038.2	65.2	101.5689	444
19AR116_Spot 298	437.7424	139666.5	2.145543	13.56882	0.683246	1.750116	0.94192	0.171878	0.641566	0.681126	1022.452	6.065939	1027.205	6.086463	1038.205	13.9439	1038.2	13.9	98.48266	265
19AR116_Part2_Spot 390	110.0638	13919.82	2.270842	13.28033	3.960713	1.763161	4.140488	0.173151	0.742185	0.179251	1029.45	7.061582	1032.01	26.83294	1038.3	82.27788	1038.3	82.3	99.14771	866
19AR116_Part2_Spot 154	264.3484	28421.26	2.48394	13.32048	1.241263	1.790397	1.58758	0.175824	0.896476	0.564681	1044.119	8.641566	1041.97	10.34341	1038.325	26.47565	1038.3	26.5	100.5581	665
19AR116_Part2_Spot 302	261.6178	42296.95	1.567527	13.4072	1.285413	1.788099	1.487058	0.175561	0.720489	0.484507	1042.676	6.936301	1041.133	9.683989	1038.756	26.2834	1038.8	26.3	100.3773	789
19AR116_Part2_Spot 384	227.1103	14644.99	2.103316	13.28834	1.413578	1.700294	1.66462	0.166934	0.771997	0.463768	995.1984	7.119236	1008.641	10.64324	1038.819	29.79528	1038.8	29.8	95.80094	861
19AR116_Part2_Spot 231	282.2845	40280.03	2.340781	13.38914	1.43393	1.754874	1.755361	0.172259	0.958879	0.546257	1024.548	9.083251	1028.961	11.35425	1039.215	29.70342	1039.2	29.7	98.5886	729
19AR116_Spot 599	133.7106	29017.76	1.824459	13.49281	1.662367	1.829458	1.868585	0.179432	0.62055	0.332096	1063.871	6.085865	1056.085	12.26827	1040.885	35.59433	1040.9	35.6	102.2083	529
19AR116_Part2_Spot 244	253.2114	29443.86	2.752372	13.3866	1.228136	1.781037	1.447143	0.174625	0.68609	0.4741	1037.545	6.575176	1038.558	9.410655	1041.549	25.73333	1041.5	25.7	99.61561	740
19AR116_Part2_Spot 321	342.261	33169.59	2.643869	13.31372	0.97636	1.807725	1.26071	0.177208	0.666921	0.529004	1051.706	6.471776	1048.256	8.241995	1041.932	21.60888	1041.9	21.6	100.938	803
19AR116_Spot 125	409.2254	16245.4	3.31605	13.36619	0.851175	1.752163	1.065595	0.171755	0.624236	0.58581	1021.772	5.89847	1027.961	6.888569	1042.014	17.44672	1042.0	17.4	98.0574	113
19AR116_Spot 575	247.5951	52270.74	3.04523	13.53977	1.182958	1.816969	1.322367	0.178104	0.585338	0.442644	1056.608	5.70446	1051.593	8.6608	1042.053	23.94706	1042.1	23.9	101.3967	508
19AR116_Spot 321	426.1364	50700.74	2.685768	13.44686	0.694197	1.812758	0.841658	0.177664	0.473057	0.562053	1054.203	4.600557	1050.074	5.507791	1042.356	14.0654	1042.4	14.1	101.1365	286
19AR116_Spot 110	192.7169	18108.26	7.162961	13.39969	1.377786	1.788806	1.62396	0.175315	0.799133	0.492089	1041.331	7.684273	1041.391	10.57708	1042.376	28.54719	1042.4	28.5	99.89977	100
19AR116_Spot 211	334.2864	99630.53	2.043846	13.4657	0.924079	1.800291	1.117179	0.17642	0.597684	0.534994	1047.387	5.777971	1045.564	7.292891	1042.615	19.06394	1042.6	19.1	100.4577	185
19AR116_Part2_Spot 42	154.2986	3698.282	1.961078	12.75788	2.660678	1.776627	3.610872	0.174097	0.685728	0.189907	1034.643	6.554759	1036.946	23.46378	1042.666	71.55373	1042.7	71.6	99.23049	565
19AR116_Part2_Spot 214	365.1826	7623.803	3.383224	13.18353	1.215229	1.828447	1.695311	0.179148	0.984216	0.580552	1062.32	9.639464	1055.722	11.12836	1042.963	27.87091	1043.0	27.9	101.8559	715
19AR116_Spot 194	113.1171	26454.02	2.736093	13.40592	1.936568	1.826674	2.03847	0.178923	0.530214	0.260104	1061.09	5.187419	1055.085	13.37657	1043.538	39.71354	1043.5	39.7	101.682	172
19AR116_Spot 444	217.4835	20273.36	2.800709	13.32135	1.23169	1.856484	1.429829	0.181836	0.676383	0.473052	1076.998	6.708636	1065.737	9.435957	1043.618	25.43431	1043.6	25.4	103.1985	390
19AR116_Spot 122	241.549	5475.633	4.247652	13.06243	1.144254	1.730458	1.400668	0.169429	0.617142	0.440605	1008.966	5.763918	1019.921	9.013676	1044.367	25.38474	1044.4	25.4	96.61037	110
19AR116_Spot 184	298.0083	33283.4	2.400556	13.4489	0.993336	1.795432	1.247192	0.175774	0.609946	0.489056	1043.846	5.878151	1043.8	8.133783	1044.561	21.96428	1044.6	22.0	99.93156	163
19AR116_Spot 533	243.0705	67656.7	2.019739	13.47576	1.419463	1.836804	1.640836	0.179677	0.814977	0.496684	1065.208	8.001889	1058.718	10.7881	1046.259	28.73962	1046.3	28.7	101.811	471
19AR116_Part2_Spot 167	231.5895	33176.57	4.525984	13.29216	0.935897	1.761683	1.327255	0.172313	0.910197	0.685774	1024.845	8.624406	1031.467	8.597034	1046.432	19.49986	1046.4	19.5	97.93712	675
19AR116_Part2_Spot 123	376.9314	50026.57	2.513271	13.41041	1.005454	1.810242	1.318106	0.177048	0.828451	0.628516	1050.826	8.033069	1049.166	8.621518	1046.608	20.69332	1046.6	20.7	100.403	636
19AR116_Spot 74	200.1218	35870.3	3.331174	13.3979	1.318454	1.680097	1.537088	0.164287	0.627807	0.408439	980.5555	5.710672	1001.019	9.784213	1047.004	28.3111	1047.0	28.3	93.65342	66
19AR116_Part2_Spot 111	330.2296	44527.02	3.505648	13.42299	1.103393	1.799134	1.326351	0.175925	0.643235	0.484966	1044.674	6.203491	1045.144	8.65643	1047.022	23.40806	1047.0	23.4	99.77577	625
19AR116_Spot 325	465.9608	44893.85	1.874376	13.41549	0.631701	1.797524	0.821763	0.17576	0.521036	0.634046	1043.768	5.02096	1044.56	5.361443	1047.114	12.83272	1047.1	12.8	99.68045	289
19AR116_Spot 187	447.4242	29952.76	3.355147	13.42294	0.701892	1.777859	1.006289	0.173827	0.649415	0.645356	1033.164	6.199463	1037.397	6.539517	1047.226	15.51887	1047.2	15.5	98.65719	166
19AR116_Spot 490	484.9337	121683.2	6.012055	13.50146	0.73457	1.773297	1.014433	0.173355	0.683884	0.674154	1030.568	6.513387	1035.728	6.586345	1047.533	15.12531	1047.5	15.1	98.38053	432
19AR116_Spot 2	133.5832	47214.36	2.063078	13.37458	2.212467	1.794192	2.312237	0.17538	0.606105	0.262129	1041.685	5.829988	1043.35	15.07672	1047.735	44.99499	1047.7	45.0	99.42256	2
19AR116_Spot 354	195.7018	37496.05	1.402888	13.36294	1.394085	1.800854	1.567869	0.176025	0.663333	0.42308	1045.221	6.400404	1045.768	10.23629	1047.807	28.66368	1047.8	28.7	99.75313	314
19AR116_Part2_Spot 46	448.3772	73378.95	4.786368	13.34681	0.865426	1.779407	1.123167	0.173927	0.684655	0.609575	1033.711	6.539067	1037.963	7.301379	1047.824	17.97051	1047.8	18.0	98.65318	568
19AR116_Spot 315	536.2907	40839.53	0.951265	13.42004	0.66838	1.823645	0.895946	0.178244	0.57774	0.644839	1057.372	5.63417	1053.997	5.875526	1047.907	13.8257	1047.9	13.8	100.9032	281
19AR116_Spot 335	135.6487	31901.15	1.906632	13.38564	1.728567	1.734321	1.850346	0.169495	0.63239	0.341769	1009.326	5.908281	1021.357	11.91744	1048.126	35.06112	1048.1	35.1	96.29819	298
19AR116_Part2_Spot 50	191.5252	29190.34	2.010457	13.2838	1.420483	1.774664	1.570167	0.173402	0.657424	0.418697	1030.825	6.262823	1036.228	10.19756	1048.542	28.74623	1048.5	28.7	98.31041	572
19AR116_Spot 236	430.8596	25717.25	7.158543	13.39493	0.870188	1.776036	1.142655	0.17347	0.651931	0.570541	1031.204	6.212597	1036.731	7.423	1049.302	18.93555	1049.3	18.9	98.27518	210
19AR116_Spot 460	389.3193	56076.86	3.838694	13.45317	0.855262	1.80784	1.074118	0.176545	0.619888	0.577113	1048.072	5.996229	1048.297	7.022255	1049.665	17.6991	1049.7	17.7	99.84825	404
19AR116_Part2_Spot 393	369.4656	57773.72	1.802693	13.37121	1.029939	1.792879	1.375626	0.175054	0.858954	0.62441	1039.895	8.249009	1042.872	8.966858	1050.013	21.67589	1050.0	21.7	99.03636	869
19AR116_Spot 367	209.3015	44651.69	2.17661	13.41302	1.18373	1.829131	1.362998	0.178537	0.648545	0.475822	1058.977	6.333491	1055.968	8.948048	1050.648	24.18072	1050.6	24.2	100.7927	327
19AR116_Spot 361	167.767	7691.159	2.396239	13.11624	0.940455	1.726016	1.140835	0.168424	0.600272	0.526169	1003.423	5.577894	1018.268	7.334605	1051.22	19.56998	1051.2	19.6	95.45321	321
19AR116_Spot 53	431.0437	20474.31	2.436287	13.29176	0.773042	1.778253	1.015668	0.17344	0.635558	0.625754	1031.036	6.055663	1037.541	6.600997	1052.169	15.98196	1052.2	16.0	97.99153	47
19AR116_Part2_Spot 366	98.53797	18565.79	2.488302	13.31216	2.410023	1.785409	2.664023	0.174107	0.696677	0.261513	1034.702	6.659773	1040.153	17.34039	1052.521	51.82128	1052.5	51.8	98.30702	844
19AR116_Spot 316	147.1701	34580.47	2.532763	13.37778	1.71842	1.830777	1.879046	0.178525	0.632626	0.336674	1058.914	6.1777	1056.558	12.34009	1052.59	35.66234	1052.6	35.7	100.6008	282
19AR116_Part2_Spot 189	189.6743	16825.73	1.924047	13.29595	1.644343	1.720442	2.069754	0.167722	0.660701	0.319217	999.5488	6.117509	1016.19	13.2915	1053.119	39.51967	1053.1	39.5	94.91315	695
19AR116_Spot 163	101.4298	20204.95	2.094038	13.32902	1.684876	1.775276	1.9671	0.172987	0.64804	0.329439	1028.546	6.160832	1036.453	12.7773	1054.065	37.41342	1054.1	37.4	97.57897	146
19AR116_Part2_Spot 118	513.6835	79855.96	2.74595	13.36573	0.853673	1.791525	1.179607	0.174508	0.807244	0.684333	1036.899	7.731823	1042.38	7.687001	1054.786	17.34393	1054.8	17.3	98.30424	632
19AR116_Spot 195	190.6441	17416.81	2.158752	13.28598	0.92787	1.83337	1.115603	0.178571	0.531899	0.476781	1059.163	5.195202	1057.488	7.329829	1054.928	19.76973	1054.9	19.8	100.4015	173
19AR116_Part2_Spot 374	678.1071	49767.45	1.054476	13.32056	0.687407	1.785325	1.106681	0.173886	0.816172	0.737495	1033.486	7.79361	1040.122	7.202795	1054.99	15.07166	1055.0	15.1	97.96171	851
19AR116_Spot 359	374.6985	74161.58	6.414117	13.38204	0.71113	1.884116	0.914715	0.183502	0.565519	0.618246	1086.076	5.652445	1075.512	6.067581	1055.06	14.49809	1055.1	14.5	102.9397	319
19AR116_Spot 478	420.249	14539.04	2.305528	13.28026	0.793512	1.679457	1.105922	0.163551	0.72392	0.654585	976.4823	6.5596	1000.776	7.038551	1055.277	16.85685	1055.3	16.9	92.53324	420
19AR116_Spot 279	205.3513	51244.58	5.671148	13.44115	1.160766	1.811486	1.319071	0.176329	0.558531	0.423428	1046.89	5.39711	1049.615	8.629934	1056.185	24.08197	1056.2	24.1	99.11996	248
19AR116_Spot 366	141.8594	21199.41	2.203421	13.31336	2.036531	1.795364	2.191095	0.174756	0.637514	0.290957	1038.262	6.113532	1043.776	14.29006	1056.232	42.22076	1056.2	42.2	98.29859	326
19AR116_Spot 353	203.0808	53106.68	2.34229	13.3266	1.245493	1.815612	1.434748	0.176718	0.683737	0.476555	1049.021	6.619364	1051.104	9.394384	1056.329	25.41878	1056.3	25.4	99.30817	313
19AR116_Spot 3	115.7744	26042.3	3.221422	13.27458	1.954476	1.805843	2.083823	0.175758	0.58123	0.278925	1043.756	5.600962	1047.575	13.61863	1056.441	40.31262	1056.4	40.3	98.79929	3
19AR116_Part2_Spot 282	532.1097	73835.98	2.101488	13.3115	0.774282	1.795062	1.068417	0.174663	0.721607	0.675398	1037.754	6.916834	1043.666	6.967315	1056.958	15.88321	1057.0	15.9	98.18307	774
19AR116_Spot 219	183.2274	52657.94	2.377224	13.35078	1.536512	1.805486	1.710279	0.175672	0.737385	0.431149	1043.286	7.102779	1047.446	11.17636	1057.026	31.08379	1057.0	31.1	98.70007	193
19AR116_Spot 34	467.3716	13588.72	2.293535	13.22991	0.914815	1.821838	1.152925	0.177169	0.674499	0.585033	1051.488	6.54407	1053.347	7.558172	1058.096	18.84129	1058.1	18.8	99.3755	30
19AR116_Part2_Spot 336	155.2046	10147.18	1.709085	12.98755	1.761379	1.818687	2.426392	0.176849	0.746646	0.307719	1049.735	7.232929	1052.212	15.89782	1058.252	46.48093	1058.3	46.5	99.19516	815
19AR116_Spot 524	103.8548	29141.65	0.882872	13.31317	1.843366	1.928881	2.025003	0.187535	0.808172	0.399097	1108.009	8.22732	1091.151	13.54207	1058.558	37.39051	1058.6	37.4	104.6716	462
19AR116_Spot 146	264.8017	76147.89	1.991534	13.38392	0.783629	1.783943	0.980207	0.173438	0.578709	0.590395	1031.023	5.513939	1039.618	6.377848	1058.629	15.94476	1058.6	15.9	97.39232	130
19AR116_Part2_Spot 79	155.0658	29578.4	1.269978	13.24278	1.835252	1.82285	2.049234	0.177033	0.829694	0.40488	1050.747	8.04457	1053.711	13.43724	1060.751	37.71997	1060.8	37.7	99.05691	599
19AR116_Spot 20	298.3558	73702.49	4.032092	13.34259	0.831028	1.814765	1.028191	0.176193	0.564864	0.549377	1046.144	5.454728	1050.798	6.731134	1061.38	17.30639	1061.4	17.3	98.56455	18
19AR116_Spot 93	273.6247	109443.7	2.720458	13.33643	0.838472	1.852194	1.043651	0.179804	0.60781	0.582388	1065.905	5.971405	1064.211	6.881758	1061.636	17.08443	1061.6	17.1	100.4022	85
19AR116_Spot 417	311.3216	58144.54	1.97336	13.3351	0.86092	1.836924	1.094931	0.17821	0.665495	0.607796	1057.189	6.488931	1058.761	7.198925	1062.896	17.48987	1062.9	17.5	99.46312	368
19AR116_Spot 214	95.63816	5864.534	1.532893	12.94824	2.055758	1.778547	2.699657	0.172542	0.722498	0.267626	1026.1	6.853629	1037.649	17.54807	1062.954	52.3529	1063.0	52.4	96.53287	188
19AR116_Part2_Spot 26	370.8526	51602.48	1.842492	13.28	1.138209	1.837268	1.349095	0.178225	0.711643	0.527496	1057.271	6.939382	1058.884	8.870656	1063.103	23.05539	1063.1	23.1	99.4514	554
19AR116_Part2_Spot 312	251.1043	40740.94	4.506196	13.20267	1.051681	1.86135	1.231365	0.180553	0.629676	0.511364	1069.996	6.208052	1067.466	8.133607	1063.193	21.30592	1063.2	21.3	100.6399	796
19AR116_Spot 251	96.5473	25640.76	1.814513	13.28031	2.675803	1.812034	2.812137	0.175723	0.688837	0.244952	1043.567	6.636801	1049.813	18.40177	1063.725	54.86779	1063.7	54.9	98.10495	222
19AR116_Part2_Spot 346	481.4876	9725.558	3.196741	13.01577	0.954587	1.784565	1.380993	0.173038	0.848466	0.614388	1028.827	8.068297	1039.845	8.986856	1063.974	21.93482	1064.0	21.9	96.69663	825
19AR116_Part2_Spot 202	473.1492	39183.1	10.43877	13.30938	0.889483	1.788683	1.146024	0.173414	0.715848	0.624636	1030.892	6.81979	1041.346	7.463896	1064.246	17.99067	1064.2	18.0	96.86588	705
19AR116_Spot 111	66.43269	14322.04	3.16696	13.2183	2.786032	1.83543	2.928634	0.177915	0.760407	0.259646	1055.575	7.403947	1058.226	19.25156	1064.592	56.90403	1064.6	56.9	99.15295	101
19AR116_Part2_Spot 227	249.9471	38790.94	2.497892	13.24575	1.419308	1.803517	1.596697	0.174751	0.583944	0.36572	1038.234	5.599679	1046.733	10.43001	1065.408	29.90416	1065.4	29.9	97.44943	726
19AR116_Part2_Spot 85	306.1441	55951.11	2.424949	13.24339	1.11491	1.830621	1.381352	0.177325	0.807599	0.584644	1052.346	7.841307	1056.502	9.071155	1065.993	22.54574	1066.0	22.5	98.71982	604
19AR116_Part2_Spot 115	475.2504	75430.44	1.770504	13.3007	0.891647	1.837409	1.084912	0.177968	0.585239	0.539434	1055.863	5.699797	1058.934	7.13371	1066.163	18.36821	1066.2	18.4	99.03397	629
19AR116_Part2_Spot 179	93.99377	8713.273	0.694287	13.02361	3.476701	1.768285	4.055325	0.171267	0.883532	0.21787	1019.087	8.328328	1033.892	26.30839	1066.234	79.60558	1066.2	79.6	95.5781	686
19AR116_Spot 544	58.3007	16218.58	2.519289	13.20029	3.619525	1.885626	3.821812	0.182541	0.807435	0.21127	1080.843	8.03472	1076.044	25.36326	1067.228	75.12015	1067.2	75.1	101.2757	481
19AR116_Spot 179	242.579	36143.94	1.93795	13.30105	0.942444	1.912152	1.161943	0.185012	0.562169	0.483818	1094.299	5.658005	1085.335	7.746964	1068.281	20.4338	1068.3	20.4	102.4355	159
19AR116_Part2_Spot 333	506.2374	4585.151	2.519089	12.67574	0.773409	1.625312	1.474785	0.157188	1.223399	0.829544	941.1323	10.7128	980.0473	9.270996	1069.182	16.53532	1069.2	16.5	88.02355	814
19AR116_Spot 54	100.9506	25401.46	3.437404	13.19955	2.123958	1.783151	2.267798	0.17239	0.714599	0.315107	1025.265	6.773609	1039.33	14.75422	1069.923	43.25562	1069.9	43.3	95.8261	48
19AR116_Spot 468	119.6165	34003.04	2.345416	13.27128	1.445432	1.833281	1.603924	0.17713	0.668993	0.417098	1051.275	6.489444	1057.456	10.53825	1071.129	29.30864	1071.1	29.3	98.14644	412
19AR116_Spot 362	292.6905	63325.88	6.552738	13.2915	0.964971	1.895326	1.123676	0.183089	0.551918	0.491172	1083.828	5.506016	1079.451	7.469048	1071.52	19.64457	1071.5	19.6	101.1487	322
19AR116_Part2_Spot 57	642.715	14482.19	1.851509	13.02728	0.599193	1.886299	0.873763	0.182121	0.625569	0.715947	1078.551	6.212858	1076.281	5.798261	1072.577	12.25314	1072.6	12.3	100.557	579
19AR116_Spot 22	189.6776	36528.04	0.617096	13.23398	1.050512	1.842962	1.218546	0.177933	0.575337	0.472151	1055.673	5.602437	1060.919	8.020954	1072.62	21.57739	1072.6	21.6	98.42007	20
19AR116_Spot 301	473.027	100149.3	2.920008	13.304	0.666752	1.894564	0.878019	0.182811	0.564707	0.64316	1082.316	5.626384	1079.184	5.835322	1073.76	13.48394	1073.8	13.5	100.7968	267

19AR116_Spot 577	100.8628	19224.89	1.309072	13.24508	2.338559	1.847931	2.586446	0.178288	1.0854	0.419649	1057.616	10.58715	1062.692	17.04238	1074.024	47.17179	1074.0	47.2	98.47231	510
19AR116_Spot 454	234.1475	61793.36	2.771049	13.28008	0.740211	1.838148	0.988548	0.177325	0.647369	0.654868	1052.343	6.285547	1059.199	6.500983	1074.247	15.00185	1074.2	15.0	97.9609	399
19AR116_Spot 17	120.9706	22720.47	1.472912	13.1854	2.026649	1.820556	2.209937	0.175585	0.636447	0.287993	1042.813	6.127949	1052.885	14.48467	1074.727	42.51898	1074.7	42.5	97.03045	15
19AR116_Part2_Spot 241	39.66031	9859.996	0.32115	12.98225	7.53918	1.844528	7.802508	0.177883	0.911481	0.116819	1055.401	8.873586	1061.479	51.41741	1074.886	155.8255	1074.9	155.8	98.1873	738
19AR116_Spot 288	423.2992	51282.41	1.906033	13.27877	0.768051	1.876081	1.03248	0.180903	0.639549	0.61943	1071.906	6.315732	1072.68	6.838622	1075.144	16.25719	1075.1	16.3	99.69883	256
19AR116_Part2_Spot 65	231.8037	42385.38	2.447945	13.16954	1.45181	1.928326	1.724802	0.185827	0.921974	0.534539	1098.727	9.313732	1090.959	11.53317	1076.379	29.24647	1076.4	29.2	102.0763	587
19AR116_Part2_Spot 181	58.4818	10851.24	0.694595	13.00711	4.470371	1.773552	4.563826	0.170824	0.831317	0.182154	1016.651	7.818853	1035.821	29.6408	1077.367	90.11618	1077.4	90.1	94.36445	688
19AR116_Spot 117	129.2692	17727.21	4.355962	13.16326	1.508891	1.90397	1.719636	0.183313	0.658115	0.382706	1085.049	6.572245	1082.478	11.44861	1078.18	31.86924	1078.2	31.9	100.6371	106
19AR116_Part2_Spot 98	147.7902	24145.59	2.202598	13.11505	2.586726	1.900036	2.766682	0.182909	0.668104	0.241482	1082.847	6.659573	1081.102	18.40752	1078.439	53.90679	1078.4	53.9	100.4087	615
19AR116_Part2_Spot 373	62.90631	9329.001	0.504897	12.98437	4.517275	1.602488	5.025258	0.154248	1.091836	0.21727	924.7295	9.405789	971.1812	31.42917	1078.708	98.49643	1078.7	98.5	85.72562	850
19AR116_Spot 26	133.213	29546.74	3.549074	13.19081	1.892386	1.822285	2.02	0.175352	0.622312	0.308075	1041.534	5.985087	1053.507	13.24406	1079.287	38.58613	1079.3	38.6	96.50207	23
19AR116_Spot 581	285.4317	87367.31	6.711307	13.34104	1.160508	1.915266	1.549428	0.184277	1.02225	0.65976	1090.297	10.254	1086.42	10.33635	1079.529	23.35392	1079.5	23.4	100.9975	514
19AR116_Spot 305	448.0307	56554.7	2.176198	13.24465	0.863464	1.869441	1.14558	0.179842	0.736591	0.642985	1066.109	7.237876	1070.333	7.578406	1079.802	17.59756	1079.8	17.6	98.73191	271
19AR116_Spot 12	547.8243	138064.5	2.76819	13.23526	0.59346	1.882309	0.815681	0.18107	0.558633	0.684867	1072.818	5.520975	1074.876	5.408846	1079.909	11.90682	1079.9	11.9	99.34334	10
19AR116_Part2_Spot 119	140.4154	27242.49	1.759633	13.14652	1.676322	1.81219	1.892159	0.174249	0.831127	0.439248	1035.482	7.950543	1049.869	12.38135	1080.806	34.08685	1080.8	34.1	95.80641	633
19AR116_Spot 427	415.8344	87610.65	2.666408	13.18828	0.691168	1.953964	0.979576	0.187835	0.684666	0.698941	1109.636	6.979393	1099.81	6.579391	1081.275	14.05479	1081.3	14.1	102.623	378
19AR116_Spot 257	104.867	39170.51	2.273768	13.18497	1.488789	1.855468	1.661544	0.178364	0.626946	0.377328	1058.03	6.117529	1065.376	10.96314	1081.302	30.85075	1081.3	30.9	97.84773	228
19AR116_Spot 600	54.52279	14133.13	1.68883	13.13336	3.005022	1.839556	3.154758	0.176787	0.684675	0.217029	1049.396	6.630627	1059.702	20.75482	1081.881	61.78741	1081.9	61.8	96.99743	530
19AR116_Part2_Spot 127	64.29619	13457.43	1.542275	13.05757	5.214452	1.779413	5.326695	0.171005	0.905005	0.1699	1017.647	8.519613	1037.965	34.64015	1081.9	105.3801	1081.9	105.4	94.06102	640
19AR116_Part2_Spot 185	172.331	28279.59	1.963528	13.1352	1.647273	1.894105	1.853923	0.181909	0.710717	0.383358	1077.396	7.051566	1079.023	12.32063	1083.166	34.34203	1083.2	34.3	99.46736	692
19AR116_Spot 583	209.0332	58765.34	1.741505	13.32028	1.364198	1.802765	1.735749	0.173107	1.026929	0.591634	1029.209	9.768694	1046.46	11.33672	1083.508	28.04554	1083.5	28.0	94.9886	516
19AR116_Spot 345	176.099	73761.39	1.491244	13.16497	1.176337	1.959636	1.354662	0.188153	0.636948	0.47019	1111.362	6.502214	1101.758	9.107717	1083.694	23.96253	1083.7	24.0	102.5531	306
19AR116_Spot 540	855.8897	340114	1.377658	13.22223	0.656068	1.965922	0.933456	0.188756	0.663265	0.710548	1114.63	6.789102	1103.912	6.282546	1083.707	13.17143	1083.7	13.2	102.8535	478
19AR116_Spot 350	118.7897	30897.24	1.651284	13.11484	2.286388	1.865719	2.385829	0.17901	0.650363	0.272594	1061.564	6.365524	1069.015	15.77309	1085.139	46.0343	1085.1	46.0	97.82749	310
19AR116_Spot 272	157.1296	44714.45	2.657435	13.18432	1.148992	1.931057	1.349576	0.185276	0.656834	0.486696	1095.736	6.618725	1091.906	9.028373	1085.162	23.61923	1085.2	23.6	100.9744	241
19AR116_Spot 486	442.7857	82643.48	2.544129	13.2001	0.73798	1.916329	0.944734	0.183818	0.567763	0.600977	1087.8	5.683147	1086.79	6.303455	1085.654	15.11964	1085.7	15.1	100.1976	428
19AR116_Spot 414	70.24042	19223.47	1.220086	13.09977	2.532147	1.889203	2.677734	0.181213	0.849465	0.317233	1073.599	8.400893	1077.302	17.78043	1085.68	50.93629	1085.7	50.9	98.88724	366
19AR116_Part2_Spot 394	296.0446	40631.85	3.203533	13.08739	1.19015	1.923555	1.479954	0.184359	0.859082	0.580479	1090.742	8.620511	1089.303	9.887468	1087.281	24.13528	1087.3	24.1	100.3183	870
19AR116_Spot 103	552.2647	148613.5	2.794348	13.20141	0.662962	1.917071	0.89729	0.183622	0.604025	0.673165	1086.732	6.040667	1087.049	5.987685	1088.569	13.297	1088.6	13.3	99.8313	94
19AR116_Part2_Spot 257	179.2078	17548.79	1.691928	12.97461	1.907355	1.868756	2.155961	0.17898	0.908838	0.421546	1061.398	8.894101	1070.09	14.26127	1088.701	39.17956	1088.7	39.2	97.49217	750
19AR116_Spot 310	313.9244	82310.16	1.99026	13.19707	0.915717	1.936059	1.186299	0.185386	0.750949	0.633019	1096.332	7.570877	1093.637	7.943037	1089.166	18.3923	1089.2	18.4	100.6579	276
19AR116_Spot 144	274.7821	46508.71	3.585755	13.1519	1.029062	1.939706	1.203507	0.185643	0.612294	0.508758	1097.729	6.180198	1094.897	8.063429	1090.165	20.73932	1090.2	20.7	100.6938	128
19AR116_Part2_Spot 113	300.5028	59863.06	1.362251	13.13454	0.992379	1.966223	1.199226	0.18818	0.626506	0.522425	1111.508	6.396383	1104.015	8.071777	1090.172	20.46778	1090.2	20.5	101.9572	627
19AR116_Part2_Spot 49	139.4618	19234.22	4.178989	12.98585	3.016849	1.921585	3.137982	0.18389	0.648441	0.206643	1088.193	6.492868	1088.619	20.9596	1090.322	61.52286	1090.3	61.5	99.80476	571
19AR116_Part2_Spot 11	105.5285	17384.08	2.558373	13.01837	2.445997	1.845278	2.718219	0.176533	0.831476	0.30589	1048.007	8.042487	1061.746	17.90179	1090.946	51.86818	1090.9	51.9	96.06403	540
19AR116_Spot 595	114.6801	33112.39	1.881475	13.18126	1.3439	1.989469	1.537318	0.190181	0.663002	0.431272	1122.356	6.829475	1111.942	10.38848	1092.489	27.77803	1092.5	27.8	102.7338	525
19AR116_Spot 166	133.4884	33212.04	1.345098	13.11648	1.52614	1.939588	1.661831	0.185278	0.619977	0.373069	1095.745	6.247379	1094.857	11.13415	1093.982	30.86191	1094.0	30.9	100.1611	149
19AR116_Part2_Spot 259	190.2458	31555.63	2.05191	13.01129	1.853434	1.868769	2.006478	0.178475	0.73841	0.368013	1058.638	7.208968	1070.095	13.27239	1094.408	37.34238	1094.4	37.3	96.73156	752
19AR116_Part2_Spot 161	98.25783	18688.32	2.990716	12.90501	2.552217	1.887023	2.750767	0.180199	0.989211	0.359613	1068.06	9.736533	1076.535	18.25818	1094.626	51.40703	1094.6	51.4	97.57312	670
19AR116_Part2_Spot 206	222.6702	11557.88	1.97047	12.97795	1.977161	1.906821	2.115639	0.182084	0.678557	0.320734	1078.348	6.737944	1083.475	14.09259	1094.687	40.10391	1094.7	40.1	98.50739	709
19AR116_Spot 590	807.105	158842.3	3.344344	13.23982	0.626163	1.970752	0.855432	0.188169	0.581509	0.679783	1111.448	5.93669	1105.564	5.762168	1094.897	12.55785	1094.9	12.6	101.5117	521
19AR116_Part2_Spot 44	113.458	17364.53	1.554276	12.92114	1.983351	1.751297	2.13019	0.166924	0.686577	0.322308	995.1394	6.33116	1027.641	13.76883	1098.389	40.35411	1098.4	40.4	90.5999	566
19AR116_Spot 21	70.35098	11312.44	1.436813	12.91868	3.050615	1.78849	3.175104	0.170453	0.769758	0.242435	1014.609	7.226433	1041.275	20.6807	1098.567	61.65144	1098.6	61.7	92.35753	19
19AR116_Spot 252	110.7391	30237.13	0.732416	13.0844	2.394386	1.951218	2.471877	0.185961	0.56142	0.227123	1099.458	5.67489	1098.866	16.59586	1098.582	48.15614	1098.6	48.2	100.0798	223
19AR116_Part2_Spot 330	225.3703	36392.92	1.837273	12.96807	1.255812	1.970548	1.517474	0.187787	0.72049	0.474796	1109.378	7.343012	1105.495	10.22154	1098.727	26.72281	1098.7	26.7	100.9694	812
19AR116_Spot 385	125.4716	39820.81	0.953194	13.11158	2.040452	1.981782	2.150148	0.188781	0.663927	0.308782	1114.771	6.796672	1109.327	14.51135	1099.525	40.90912	1099.5	40.9	101.3866	342
19AR116_Part2_Spot 371	349.4568	91963.66	3.109965	13.10664	0.907972	1.918581	1.140782	0.182698	0.682686	0.598437	1081.695	6.798276	1087.574	7.614635	1100.245	18.30214	1100.2	18.3	98.31403	848
19AR116_Part2_Spot 89	589.9385	66907.04	3.849444	13.01221	0.670685	1.855827	0.960146	0.176522	0.66474	0.692332	1047.946	6.429377	1065.504	6.335463	1102.52	13.87221	1102.5	13.9	95.05003	607
19AR116_Part2_Spot 260	603.5509	91601.59	3.728997	12.98237	0.742209	1.971376	1.038262	0.187134	0.719101	0.692601	1105.829	7.30736	1105.778	6.99448	1106.532	14.98674	1106.5	15.0	99.93644	753
19AR116_Spot 357	224.7904	32141.51	1.891445	12.98136	0.865325	1.970035	1.212476	0.186942	0.748088	0.616992	1104.788	7.595361	1105.319	8.166293	1107.234	19.06272	1107.2	19.1	99.77908	317
19AR116_Spot 226	485.8046	148843.6	2.463748	13.06989	0.776383	1.957069	1.077539	0.18554	0.745305	0.691673	1097.171	7.519247	1100.877	7.241275	1109.061	15.5384	1109.1	15.5	98.92797	200
19AR116_Part2_Spot 53	76.71969	5992.334	3.102136	12.58405	3.410663	1.908735	4.504339	0.180813	0.74017	0.164324	1071.415	7.306325	1084.143	30.02127	1110.661	88.79484	1110.7	88.8	96.4665	575
19AR116_Spot 532	156.5988	7053.553	2.987148	12.73251	1.647892	1.946475	2.109467	0.18416	0.740454	0.351015	1089.66	7.423369	1097.233	14.15067	1113.134	39.42274	1113.1	39.4	97.89125	470
19AR116_Part2_Spot 39	177.3488	21410.28	2.755644	12.82773	1.47795	1.919597	1.645603	0.181406	0.699846	0.425282	1074.649	6.92743	1087.928	10.98649	1115.459	29.73817	1115.5	29.7	96.34135	562
19AR116_Spot 185	201.0167	47794.98	1.792263	12.98232	1.043472	2.019325	1.244282	0.190471	0.653587	0.525272	1123.928	6.741123	1122.032	8.449967	1119.249	21.13038	1119.2	21.1	100.4181	164
19AR116_Part2_Spot 391	405.2093	24370.71	3.741542	12.86094	0.937007	1.969729	1.264001	0.185787	0.735787	0.582109	1098.513	7.431547	1105.215	8.512891	1119.316	20.51217	1119.3	20.5	98.14145	867
19AR116_Spot 19	126.5344	6543.866	2.206529	12.62327	1.687076	1.836805	1.843706	0.173013	0.652335	0.353817	1028.689	6.202466	1058.718	12.12205	1122.037	34.36801	1122.0	34.4	91.68044	17
19AR116_Part2_Spot 84	140.3352	31929.39	2.878828	12.84599	3.111578	1.850058	3.218101	0.174239	0.785042	0.243946	1035.425	7.509319	1063.451	21.21409	1122.269	62.24149	1122.3	62.2	92.26178	603
19AR116_Spot 161	198.7295	38320.55	1.407615	12.93622	1.343964	1.834477	1.689012	0.172687	0.954397	0.565062	1026.898	9.059928	1057.884	11.09991	1123.26	27.76406	1123.3	27.8	91.42119	144
19AR116_Spot 508	79.05397	25178.63	1.950115	12.87079	1.871742	2.054136	2.11146	0.193341	0.860051	0.407325	1139.451	8.982621	1133.672	14.42059	1123.477	38.43375	1123.5	38.4	101.4218	448
19AR116_Spot 507	169.4086	35265.31	0.719731	12.86253	1.513075	2.009346	1.825212	0.189056	0.897451	0.491697	1116.257	9.198488	1118.671	12.37506	1124.212	31.68341	1124.2	31.7	99.2924	447
19AR116_Part2_Spot 313	170.6345	29161.01	2.389924	12.71709	1.926631	2.031902	2.091942	0.19061	0.726416	0.347245	1124.677	7.496856	1126.253	14.23625	1130.163	39.07427	1130.2	39.1	99.51459	797
19AR116_Spot 213	223.0096	40474.61	2.19753	12.85296	1.012222	2.113077	1.202887	0.197995	0.628732	0.522686	1164.541	6.698588	1153.081	8.290675	1132.486	20.41631	1132.5	20.4	102.8306	187
19AR116_Part2_Spot 297	218.4026	35602.63	1.500835	12.78629	1.449807	2.027301	1.648016	0.189948	0.715526	0.434174	1121.091	7.36291	1124.711	11.20654	1132.583	29.54939	1132.6	29.5	98.98533	786
19AR116_Part2_Spot 133	226.8987	43731.52	1.869604	12.7716	1.31286	2.044014	1.492634	0.191136	0.70052	0.469318	1127.527	7.246368	1130.301	10.17735	1136.518	26.22645	1136.5	26.2	99.20891	646
19AR116_Spot 364	155.6854	44241.01	1.996713	12.82858	0.891383	1.991537	1.092409	0.185888	0.595561	0.545181	1099.061	6.017998	1112.644	7.384427	1140.165	18.19438	1140.2	18.2	96.39489	324
19AR116_Spot 471	52.10016	16219.75	2.109152	12.73208	3.571029	1.883181	3.666915	0.175771	0.722632	0.197068	1043.831	6.964028	1075.183	24.32394	1140.197	71.50096	1140.2	71.5	91.54832	415
19AR116_Spot 376	267.0827	50298.3	2.604629	12.82347	0.724265	2.089396	1.018845	0.194917	0.640957	0.629102	1147.954	6.739976	1145.327	6.996682	1141.205	15.74789	1141.2	15.7	100.5914	333
19AR116_Part2_Spot 138	39.83723	10290.16	2.504308	12.54745	6.955381	1.895503	7.29098	0.17675	1.635013	0.224252	1049.194	15.83125	1079.513	48.50052	1142.087	141.4308	1142.1	141.4	91.86637	651
19AR116_Spot 131	174.6091	41425.98	2.045722	12.80434	1.21668	2.053197	1.438539	0.191447	0.732501	0.509198	1129.211	7.587547	1133.36	9.82293	1142.199	24.59562	1142.2	24.6	98.8629	116
19AR116_Spot 510	237.1826	52764.25	2.677384	12.77577	1.447863	2.09169	1.662031	0.194675	0.807644	0.485938	1146.651	8.483967	1146.081	11.41796	1145.885	28.86431	1145.9	28.9	100.0668	449
19AR116_Spot 254	408.2167	116129.4	14.92229	12.81876	0.865872	2.122142	1.061535	0.197454	0.604302	0.569272	1161.627	6.423608	1156.033	7.326446	1146.421	17.33541	1146.4	17.3	101.3264	225
19AR116_Spot 332	31.41914	8796.933	3.950775	12.53819	6.506006	1.835629	6.761689	0.170473	0.912395	0.134936	1014.717	8.566344	1058.297	44.47318	1150.16	133.1849	1150.2	133.2	88.22402	295
19AR116_Part2_Spot 86	173.8756	23672.8	1.965535	12.61485	1.205791	2.104792	1.446649	0.195023	0.64605	0.446584	1148.528	6.796635	1150.375	9.958262	1154.702	25.70087	1154.7	25.7	99.46533	605
19AR116_Spot 136	304.2214	8214.432	2.615096	12.5031	0.853046	1.907324	1.176051	0.17669	0.726854	0.618046	1048.864	7.035814	1083.65	7.834216	1155.115	18.34599	1155.1	18.3	90.80167	121
19AR116_Spot 57	30.3545	7872.848	2.484782	12.45392	7.008728	1.749064	7.358294	0.16202	1.315865	0.178827	967.9908	11.82726	1026.817	47.57135	1155.226	143.8356	1155.2	143.8	83.79236	51
19AR116_Spot 167	96.03537	31497.77	27.71687	12.68581	1.679932	2.104142	1.897248	0.194415	0.735138	0.387476	1145.25	7.713696	1150.162	13.05905	1160.278	34.68956	1160.3	34.7	98.70477	150
19AR116_Spot 68	33.52951	9136.644	0.945442	12.34066	5.910879	1.959475	6.100054	0.179679	1.086711	0.178148	1065.223	10.67006	1101.703	41.03221	1175.352	118.8408	1175.4	118.8	90.63007	61
19AR116_Part2_Spot 10	131.2185	14786.9	0.85047	12.49948	2.303815	2.05424	2.641087	0.187926	0.743856	0.281648	1110.129	7.585847	1133.706	18.03874	1180.013	50.1223	1180.0	50.1	94.07764	539
19AR116_Part2_Spot 351	50.50515	8520.821	1.983579	12.31338	5.316467	1.980907	5.404836	0.180903	0.763835	0.141324	1071.907	7.543107	1109.029	36.48504	1183.439	105.7886	1183.4	105.8	90.57562	830
19AR116_Part2_Spot 245	95.22426	14522.88	0.82467	12.32199	1.389334	2.254672	1.780571	0.204937	0.740136	0.415673	1201.785	8.114939	1198.245	12.52528	1192.706	31.96016	1192.7	32.0	100.7613	741
19AR116_Part2_Spot 339	370.5591	54771.73	5.200887	12.32838	0.861861	2.199355	1.09488	0.198878	0.671637	0.613434	1169.291	7.182321	1180.839	7.642538	1202.897	17.02357	1202.9	17.0	97.20626	818
19AR116_Spot 48	183.6719	51918.83	1.184144	12.39577	0.888622	2.288898	1.068735	0.206702	0.582758	0.545278	1211.222	6.43503	1208.867	7.552382	1205.503	17.62929	1205.5	17.6	100.4744	42
19AR116_Spot 549	209.4851	79549.88	0.910843	12.31813	0.911412	2.424676	1.096739	0.216708	0.606166	0.552698	1264.455	6.959814	1249.943	7.884533	1225.868	17.93485	1225.9	17.9	103.1477	486
19AR116_Part2_Spot 184	127.0687	27897.03	1.183668	12.2168	1.743716	2.261062	1.883746	0.201883	0.669664	0.355496	1185.428	7.251245	1200.236	13.26265	1227.829	34.59026	1227.8	34.6	96.5466	691
19AR116_Part2_Spot 383	91.14925	10768.18	1.520126	11.98391	1.831111	2.416221	2.110033	0.214295	0.919489	0.43577	1251.658	10.4605	1247.434	15.15451	1241.017	37.22218	1241.0	37.2	100.8575	860
19AR116_Spot 233	283.0796	53325.23	7.704643	12.14048	0.73223	2.245278	1.02894	0.198127	0.718511	0.698302	1165.253	7.659374	1195.31	7.228455	1250.905	14.39512	1250.9	14.4	93.15286	207
19AR116_Spot 135	419.0623	65226.2	1.96282	12.12778	0.641957	2.414568	0.932984	0.212851	0.665181	0.712961	1243.987	7.525331	1246.942	6.699061	1252.883	12.78096	1252.9	12.8	99.28992	120
19AR116_Spot 239	30.35001	5572.199	3.057098	11.77431	8.643365	2.001805	8.84803	0.175831	0.910219	0.102872	1044.161	8.774366	1116.123	59.98195	1259.926	172.3394	1259.9	172.3	82.8748	213
19AR116_Part2_Spot 75	61.59252	12187.9	0.844404	11.85974	2.72277	2.38372	2.972411	0.20864	0.811114	0.272881	1221.57	9.026135	1237.727	21.26489	1266.837	55.84544	1266.8	55.8	96.42676	595
19AR116_Spot 358	168.2527	41846.31	2.468756	11.9745	1.214997	2.48907	1.421637	0.21724	0.680747	0.478847	1267.273	7.831898	1268.858	10.29819	1272.377	24.3432	1272.4	24.3	99.5989	318
19AR116_Spot 443	108.987	14271.63	2.638073	11.80489	2.100216	2.522181	2.393647	0.21931	0.65975	0.275625	1278.226	7.649641	1278.449	17.40592	1279.655	44.83133	1279.7	44.8	99.88827	389
19AR116_Part2_Spot 8	229.61	60176.54	1.72843	11.73361	1.038719	2.617682	1.295322	0.222722	0.743256	0.5738	1296.24	8.727539	1305.614	9.517146	1321.873	20.55289	1321.9	20.6	98.06092	537
19AR116_Part2_Spot 289	183.6972	31497.8	2.006459	11.62475	1.084552	2.679586	1.336241	0.227639	0.763702	0.57153	1322.115	9.128916	1322.841	9.880917	1324.842	21.23623	1324.8	21.2	99.79416	779
19AR116_Spot 193	783.6319	220583.2	39.39596	11.56119	0.52213	2.763259	0.751731	0.231803	0.538977	0.716982	1343.939	6.538317	1345.672	5.604722	1349.251	10.11378	1349.3	10.1	99.6063	171
19AR116_Spot 139	323.1828	74628.92	2.469843	11.4544	0.820519	2.864662	1.040851	0.238295	0.637222	0.612213	1377.826	7.904965	1372.67	7.8341	1365.476	15.84825	1365.5	15.8	100.9044	124
19AR116_Spot 484	266.9372	74588.23	1.90726	11.32126	0.749795	2.953207	0.968359	0.242921	0.608654	0.628542	1401.866	7.668511	1395.672	7.34545	1387.037	14.4597	1387.0	14.5	101.0691	426
19AR116_Spot 221	147.7492	28071.64	2.409295	11.25742	1.038565	2.921941	1.310972	0.240205	0.71784	0.547563	1387.762	8.962611	1387.609	9.917621	1388.192	21.05731	1388.2	21.1	99.96908	195
19AR116_Spot 215	117.4165	30905.94	1.113775	11.00333	1.216239	3.136987	1.489238	0.251753	0.758888	0.509581	1447.508	9.839012	1441.811	11.46677	1434.231	24.44388	1434.2	24.4	100.9257	189
19AR116_Spot 250	362.2501	128850.1	3.475912	11.04882	0.605087	3.122674	0.816689	0.250385	0.546765	0.66949	1440.461	7.05803	1438.292	6.281159	1435.897	11.56975	1435.9	11.6	100.3178	221
19AR116_Part2_Spot 13	363.6034	95672.17	1.654345	10.7038	0.772675	3.328831	1.017511	0.258963	0.654711	0.643444	1484.535	8.681469	1487.838	7.945094	1493.357	14.74047	1493.4	14.7	99.40922	542
19AR116_Part2_Spot 284	144.281	38137.39	1.886832	10.49494	1.251916	3.396411	1.518591	0.26032	0.82815	0.545341	1491.48	11.02693	1503.568	11.91276	1521.444	23.99867	1521.4	24.0	98.03057	776
19AR116_Spot 151	77.47469	22190.2	1.76616	9.616205	1.734669	3.569627	1.889631	0.250142	0.694156	0.36735	1439.21	8.953717	1542.805	14.98928	1688.574	32.42484	1688.6	32.4	85.23227	135
19AR116_Part2_Spot 296	62.56289	14168.01	1.499791	9.45579	1.486676	4.247485	1.765999	0.295504	0.846579	0.479377	1668.974	12.44832	1683.25	14.51545	1701.873	28.54643	1701.9	28.5	98.06694	785
19AR116_Part2_Spot 56	145.6703	24973.98	5.11725	9.237733	0.895029	4.558292	1.134363	0.309106	0.64825	0.571466	1736.307	9.867196	1741.677	9.446163	1748.911	17.04353	1748.9	17.0	99.2793	578
19AR116_Spot 81	434.7284	126050.5	5.157931	9.218465	0.702203	3.963598	1.024084	0.265852	0.744985	0.727464	1519.712	10.08609	1626.777	8.303637	1768.936	12.83253	1768.9	12.8	85.91106	73
19AR116_Spot 35	168.9897	70898.44	1.636272	9.230049	0.997532	4.782308	1.190297	0.320539	0.647932	0.544345	1792.361	10.13859	1781.797	9.996215	1770.226	18.23313	1770.2	18.2	101.2504	31
19AR116_Spot 488	243.3305	46779.67	0.851307	9.232929	0.716669	4.729539	0.926391	0.316837	0.579061	0.625072	1774.263	8.981444	1772.488	7.764825	1771.178	13.2022	1771.2	13.2	100.1742	430
19AR116_Part2_Spot 20	17.30492	1354.05	5.448584	8.433287	12.26534	1.441298	13.6698	0.096376	3.895311	0.284957	593.1361	22.07352	906.2596	82.12409	1774.547	240.2032	1774.5	240.2	33.42465	549
19AR116_Part2_Spot 226	31.0805	9821.041	0.941242	8.917233	3.597889	4.75522	3.826142	0.312525	1.241464	0.324469	1753.122	19.05598	1777.029	32.11031	1806.006	65.8167	1806.0	65.8	97.07175	725
19AR116_Spot 400	71.06998	7185.191	0.568939	8.936997	1.964323	4.299206	2.634468	0.281831	1.004719	0.381374	1600.575	14.24035	1693.209	21.70534	1810.672	44.25463	1810.7	44.3	88.39672	356
19AR116_Spot 270	74.8298	22362.06	1.284526	8.951267	1.243655	4.78046	1.540868	0.311789	0.871087	0.565322	1749.502	13.34679	1781.473	12.93976	1819.906	23.06898	1819.9	23.1	96.13145	239
19AR116_Spot 289	353.4988	20223.49	0.921865	8.953216	0.681948	4.640683	0.97468	0.302636	0.693252	0.711261	1704.368	10.38264	1756.618	8.142385	1820.121	12.43359	1820.1	12.4	93.64037	257
19AR116_Part2_Spot 134	52.09481	5857.76	2.072789	8.684601	2.111304	5.022936	2.293454	0.324892	0.79109	0.344934	1813.575	12.50555	1823.196	19.42327	1834.978	39.00228	1835.0	39.0	98.83363	647
19AR116_Spot 445	58.20688	35905.72	0.531171	8.836436	2.090987	5.008227	2.210471	0.322984	0.670513	0.303335	1804.287	10.55244	1820.714	18.7112	1840.331	38.13697	1840.3	38.1	98.04145	391
19AR116_Spot 95	164.0129	75387.11	1.513778	8.859083	0.684084	5.100058	0.98429	0.328368	0.703008	0.714229	1830.467	11.20267	1836.115	8.356105	1843.297	12.46701	1843.3	12.5	99.30399	87
19AR116_Part2_Spot 397	218.8275	56878.99	1.26106	8.768863	0.874866	5.201166	1.315634	0.333169	0.968653	0.736263	1853.723	15.6051	1852.807	11.20495	1852.552	16.09373	1852.6	16.1	100.0632	872
19AR116_Spot 378	230.2641	84407.47	1.708498	8.793778	0.602009	5.321418	0.807193	0.339597	0.53218	0.659297	1884.728	8.696933	1872.309	6.899644	1859.323	10.96156	1859.3	11.0	101.3664	335
19AR116_Spot 517	255.125	100302.9	1.846526	8.778586	0.718947	5.282195	1.019742	0.337044	0.721477	0.70751	1872.435	11.72418	1865.989	8.706303	1859.586	13.01584	1859.6	13.0	100.691	455
19AR116_Part2_Spot 301	142.6221	21260.73	0.94011	8.701449	1.040418	5.002102	1.364852	0.319169	0.853257	0.625165	1785.668	13.30818	1819.678	11.55004	1859.606	19.24089	1859.6	19.2	96.02397	788
19AR116_Part2_Spot 93	524.5919	143335.1	0.591713	8.739203	0.561495	5.018686	0.840153	0.319986	0.624236	0.743003	1789.659	9.755037	1822.48	7.113509	1860.967	10.15371	1861.0	10.2	96.16822	610
19AR116_Part2_Spot 48	154.4989	34135.59	0.524217	8.683254	0.689216	5.313598	1.034101	0.338182	0.762538	0.737393	1877.916	12.42268	1871.052	8.837216	1864.207	12.60858	1864.2	12.6	100.7354	570
19AR116_Part2_Spot 271	95.92665	35106.12	0.875757	8.687811	1.227478	5.002649	1.548916	0.318351	0.93239	0.601963	1781.67	14.51415	1819.77	13.10808	1864.436	22.32766	1864.4	22.3	95.56081	763
19AR116_Spot 579	146.908	82072.22	1.245553	8.798576	1.211173	5.091894	1.5975	0.322871	1.040782	0.651507	1803.735	16.37534	1834.756	13.55887	1870.903	21.85887	1870.9	21.9	96.40986	512
19AR116_Spot 518	168.0793	94446.54	0.693648	8.710243	0.883698	5.402635	1.134401	0.342241	0.709448	0.625394	1897.439	11.66112	1885.271	9.719788	1872.664	15.96466	1872.7	16.0	101.323	456
19AR116_Part2_Spot 274	183.1554	62636.56	1.097543	8.671889	0.792623	5.251542	1.024388	0.332662	0.632366	0.617311	1851.27	10.17583	1861.023	8.737854	1872.705	14.53473	1872.7	14.5	98.85541	766
19AR116_Part2_Spot 290	43.43021	18282.55	0.896327	8.651873	1.774887	5.26116	1.979637	0.333051	0.759799	0.383807	1853.154	12.23719	1862.584	16.89204	1873.894	32.96263	1873.9	33.0	98.89321	780
19AR116_Part2_Spot 62	429.8682	34242.28	1.366776	8.637555	0.915556	5.102633	1.346507	0.322841	0.981709	0.729078	1803.587	15.44481	1836.544	11.43231	1874.872	16.61492	1874.9	16.6	96.19787	584
19AR116_Part2_Spot 52	171.3858	31099.53	0.811548	8.604358	0.724711	5.387476	1.024579	0.340439	0.675918	0.659703	1888.78	11.06636	1882.865	8.774902	1877.115	13.87776	1877.1	13.9	100.6214	574
19AR116_Spot 228	146.1385	67705.75	1.296345	8.699134	0.847392	5.276512	1.090288	0.333373	0.67938	0.623119	1854.707	10.94988	1865.07	9.307046	1877.409	15.36883	1877.4	15.4	98.79083	202
19AR116_Spot 588	102.5858	47550.48	1.001967	8.736189	0.904629	5.637654	1.066972	0.355121	0.554233	0.519445	1959.008	9.362889	1921.875	9.20192	1882.821	16.42148	1882.8	16.4	104.0464	520
19AR116_Spot 464	137.5683	24617.08	0.691024	8.632434	0.731223	5.382008	1.075619	0.337689	0.64699	0.601505	1875.541	10.52877	1881.995	9.210588	1889.895	15.46349	1889.9	15.5	99.24051	408
19AR116_Spot 586	208.1376	60687	5.098424	8.716337	0.593987	5.398822	0.829566	0.338686	0.567804	0.684459	1880.346	9.260532	1884.667	7.107012	1890.2	10.88277	1890.2	10.9	99.47871	518
19AR116_Part2_Spot 277	183.4184	64885.95	0.809934	8.582851	1.008191	5.318422	1.193435	0.333411	0.613747	0.514269	1854.891	9.892888	1871.828	10.20041	1891.447	18.41633	1891.4	18.4	98.06732	769
19AR116_Part2_Spot 311	155.7374	33697.18	0.395624	8.54292	0.728202	5.499715	1.080485	0.344281	0.76539	0.708376	1907.234	12.63644	1900.552	9.283387	1894.029	13.71741	1894.0	13.7	100.6972	795
19AR116_Part2_Spot 112	37.98033	11981.63	1.055539	8.544454	2.683066	5.538902	2.842818	0.34662	0.770253	0.270947	1918.441	12.7809	1906.655	24.45579	1894.621	49.22657	1894.6	49.2	101.2572	626
19AR116_Spot 113	205.2872	65487.69	1.170451	8.360688	0.617715	5.672417	0.940355	0.343811	0.703862	0.748507	1904.978	11.60882	1927.179	8.117382	1951.904	11.14081	1951.9	11.1	97.59591	103
19AR116_Spot 263	131.6536	51308.78	1.748621	8.314388	0.845567	5.720197	1.101969	0.345261	0.693036	0.628907	1911.931	11.4661	1934.424	9.524473	1959.367	15.29164	1959.4	15.3	97.57898	233
19AR116_Spot 43	85.10051	49180.89	2.160502	8.296312	1.140063	6.00934	1.328363	0.362629	0.668447	0.503211	1994.624	11.46755	1977.198	11.56419	1959.782	20.48842	1959.8	20.5	101.7779	39
19AR116_Spot 485	172.5215	90245.25	1.752756	8.226402	0.575025	6.018217	0.815022	0.359386	0.567859	0.696741	1979.263	9.677817	1978.483	7.096551	1978.427	10.41276	1978.4	10.4	100.0422	427
19AR116_Spot 261	180.3717	95941.38	2.999295	8.214126	0.715023	5.855632	0.98215	0.348907	0.668753	0.680908	1929.379	11.15097	1954.684	8.518125	1982.351	12.80535	1982.4	12.8	97.32782	232
19AR116_Spot 320	327.8117	229771	1.326317	8.191627	0.515066	6.174055	0.720029	0.367406	0.502915	0.698466	2017.182	8.710875	2000.783	6.292033	1984.65	9.170869	1984.7	9.2	101.6392	285
19AR116_Spot 100	125.0853	59443.05	1.623089	8.175384	0.69809	6.098786	0.965223	0.362481	0.660941	0.684755	1993.924	11.33538	1990.073	8.420287	1986.836	12.5163	1986.8	12.5	100.3567	91
19AR116_Part2_Spot 314	225.5829	26296.45	1.718884	8.049708	0.754021	5.54964	1.072106	0.328842	0.710169	0.662405	1832.765	11.32906	1908.321	9.224163	1992.241	14.27992	1992.2	14.3	91.99514	798
19AR116_Spot 380	266.7415	139601.3	1.462922	8.16717	0.558746	6.194795	0.773121	0.366871	0.532478	0.688738	2014.658	9.213096	2003.714	6.759155	1993.209	9.963972	1993.2	10.0	101.0761	337
19AR116_Spot 513	157.2703	70850.2	0.776789	8.114583	0.746551	6.138695	1.0286	0.362193	0.706021	0.68639	1992.558	12.10145	1995.766	8.981422	1999.848	13.28783	1999.8	13.3	99.63547	452
19AR116_Part2_Spot 305	197.0934	72145.61	6.142028	8.054957	0.666972	6.008147	0.983238	0.353945	0.716757	0.728976	1953.411	12.07886	1977.025	8.559258	2002.575	11.95211	2002.6	12.0	97.54496	790
19AR116_Spot 294	263.1521	112304.4	3.884044	8.087699	0.706224	6.448899	0.990353	0.37752	0.69353	0.700285	2064.687	12.25253	2038.956	8.706112	2013.781	12.54029	2013.8	12.5	102.5279	261
19AR116_Spot 258	33.27156	14470.38	1.346134	8.007767	1.926778	6.173456	2.063937	0.360845	0.718812	0.348272	1986.18	12.28702	2000.698	18.03732	2016.48	34.30582	2016.5	34.3	98.49736	229
19AR116_Part2_Spot 104	361.161	51121.31	1.124096	7.988055	0.623706	5.958861	0.956811	0.346931	0.719216	0.75168	1919.928	11.94197	1969.859	8.319376	2023.471	11.18078	2023.5	11.2	94.88291	620
19AR116_Part2_Spot 139	239.3685	85314.59	1.299095	7.947458	0.658649	6.261842	0.920041	0.364228	0.632239	0.687186	2002.182	10.88144	2013.133	8.05567	2025.14	11.83814	2025.1	11.8	98.86633	652
19AR116_Spot 529	272.2159	129188.9	2.233141	7.841449	0.702528	6.520499	1.028001	0.370513	0.750166	0.729733	2031.812	13.07363	2048.67	9.050426	2066.433	12.39035	2066.4	12.4	98.32461	467
19AR116_Spot 107	305.2555	202290.5	1.60813	7.724518	0.612011	6.834573	0.863894	0.382024	0.608196	0.704017	2085.733	10.83771	2090.213	7.652347	2095.378	10.78172	2095.4	10.8	99.53969	97
19AR116_Spot 16	241.1649	150773.2	2.627079	7.551211	0.574406	6.909089	0.8143	0.378723	0.57599	0.707344	2070.315	10.19948	2099.825	7.222974	2129.617	10.07662	2129.6	10.1	97.21534	14

19AR116_Spot 231	75.28781	30180.13	1.638812	7.514439	1.122909	7.253675	1.368598	0.396487	0.776717	0.567528	2152.842	14.21588	2143.127	12.21343	2134.574	19.71399	2134.6	19.7	100.8558	205
19AR116_Spot 494	224.912	125656	1.371965	7.484416	0.685851	7.375891	0.987971	0.398773	0.709391	0.718028	2163.388	13.03716	2158.052	8.834224	2153.722	12.00355	2153.7	12.0	100.4488	435
19AR116_Spot 574	198.7046	48010.37	2.417912	7.462488	0.652318	7.232297	0.903641	0.390082	0.615276	0.680885	2123.207	11.13022	2140.494	8.061027	2157.871	11.5474	2157.9	11.5	98.39357	507
19AR116_Part2_Spot 173	493.3826	69000.78	5.748283	7.236256	0.678484	7.261899	1.026947	0.384646	0.769983	0.749778	2097.951	13.78867	2144.138	9.165581	2189.427	11.81511	2189.4	11.8	95.82189	681
19AR116_Spot 36	9.893771	1489.37	0.114081	6.740698	44.18592	1.676717	44.56427	0.087075	5.514101	0.123734	538.2164	28.47284	999.7369	291.1758	2223.705	811.5111	2223.7	811.5	24.20359	32
19AR116_Spot 78	654.0437	77179.35	1.844265	6.234454	0.635924	9.037309	0.927409	0.409494	0.67451	0.727306	2212.606	12.63255	2341.787	8.478775	2457.049	10.75893	2457.0	10.8	90.05135	70
19AR116_Spot 220	440.1453	148580.1	44.04994	6.163005	0.663653	8.916021	0.947428	0.399232	0.675885	0.713389	2165.503	12.43161	2329.443	8.650085	2477.087	11.20043	2477.1	11.2	87.42135	194
19AR116_Spot 543	89.49358	45476.6	1.391361	6.106143	0.775873	10.61527	1.057086	0.47121	0.714301	0.675726	2488.865	14.74823	2490.045	9.809695	2491.728	13.12957	2491.7	13.1	99.8851	480
19AR116_Spot 162	1087.29	99290.98	6.500868	6.101718	0.585638	10.03308	0.844044	0.443794	0.606847	0.718975	2367.603	12.02469	2437.831	7.793656	2497.684	9.878503	2497.7	9.9	94.79194	145
19AR116_Spot 156	306.9693	197539.5	1.711943	6.002001	0.506742	11.01508	0.748888	0.479094	0.550632	0.735267	2523.319	11.49755	2524.408	6.97131	2525.999	8.523327	2526.0	8.5	99.89392	140
19AR116_Part2_Spot 361	431.3272	144367.6	3.433886	5.811591	0.586237	9.943624	0.849922	0.418852	0.615181	0.723808	2255.268	11.70699	2429.565	7.841538	2579.661	9.795947	2579.7	9.8	87.42497	839
19AR116_Spot 386	404.2765	199458.2	2.711631	5.7757	0.54934	10.84053	0.822274	0.452925	0.611785	0.744016	2408.244	12.29422	2509.548	7.644232	2593.268	9.162103	2593.3	9.2	92.86523	343
19AR116_Spot 422	246.2549	149257.6	2.311485	5.731034	0.493208	11.72951	0.738862	0.488316	0.549243	0.743363	2563.387	11.61688	2583.056	6.913029	2599.232	8.236675	2599.2	8.2	98.62095	373
19AR116_Part2_Spot 159	128.002	57225.65	0.691809	5.665619	0.857532	12.09166	1.22419	0.502578	0.870625	0.711185	2624.868	18.7723	2611.54	11.48123	2601.932	14.3418	2601.9	14.3	100.8815	668
19AR116_Spot 453	492.0853	192851.5	3.872317	5.691044	0.5244	11.50108	0.802536	0.474186	0.6065	0.755729	2501.891	12.57609	2564.669	7.497097	2615.386	8.748865	2615.4	8.7	95.66046	398
19AR116_Spot 11	168.7184	104674.5	3.555396	5.674861	0.566707	12.17438	0.821223	0.50152	0.592285	0.721222	2620.323	12.75282	2617.936	7.705774	2616.801	9.468311	2616.8	9.5	100.1346	9
19AR116_Part2_Spot 367	290.4815	67364.96	0.534271	5.65595	0.616896	11.82206	0.912217	0.48532	0.67033	0.734837	2550.395	14.11938	2590.412	8.540308	2622.572	10.29212	2622.6	10.3	97.24785	845
19AR116_Spot 25	68.82807	28617.27	1.215413	5.645382	0.879609	11.95044	1.083809	0.490217	0.621622	0.573553	2571.616	13.18208	2600.528	10.15539	2623.836	14.7672	2623.8	14.8	98.0098	22
19AR116_Spot 598	188.4288	176579.2	0.89907	5.668896	0.625867	12.34301	0.866284	0.504781	0.598793	0.69122	2634.31	12.94867	2630.85	8.137048	2628.901	10.40732	2628.9	10.4	100.2057	528
19AR116_Spot 547	153.071	93907.6	1.285664	5.599018	0.667084	11.92083	0.911638	0.484158	0.61998	0.680073	2545.353	13.03779	2598.204	8.540406	2640.382	11.09609	2640.4	11.1	96.40094	484
19AR116_Part2_Spot 125	450.0583	124426.3	1.455451	5.586466	0.756649	12.13731	1.111499	0.492431	0.814044	0.732384	2581.185	17.31483	2615.075	10.42726	2642.133	12.56291	2642.1	12.6	97.69321	638
19AR116_Part2_Spot 38	136.6236	54123.98	2.082634	5.491979	0.630698	11.42312	0.956045	0.458405	0.716674	0.749623	2432.512	14.52151	2558.318	8.926345	2660.303	10.48847	2660.3	10.5	91.43742	561
19AR116_Spot 369	270.286	85839.39	1.681233	5.344149	0.621791	12.76351	0.901033	0.494597	0.651281	0.722816	2590.535	13.8936	2662.356	8.484412	2718.087	10.2647	2718.1	10.3	95.30729	329
19AR116_Spot 429	128.4012	90443.22	2.161369	5.22479	0.749983	13.57292	1.08572	0.51664	0.782763	0.720962	2684.914	17.18917	2720.379	10.26808	2747.522	12.36652	2747.5	12.4	97.72129	380
19AR116_Spot 198	128.8258	22717.67	1.161725	5.134678	0.617286	12.15741	0.88249	0.453915	0.608198	0.689184	2412.636	12.24052	2616.627	8.279806	2779.202	10.47881	2779.2	10.5	86.81038	174
19AR116_Spot 77	195.4855	148632.4	1.819335	5.115652	0.650979	14.75974	0.927026	0.548372	0.659251	0.711146	2818.396	15.05114	2799.877	8.815811	2787.26	10.67278	2787.3	10.7	101.1171	69
19AR116_Spot 519	254.108	139540.9	1.876702	5.111715	0.691107	14.11134	0.996105	0.523095	0.717063	0.719867	2712.294	15.8756	2757.217	9.445234	2790.97	11.31885	2791.0	11.3	97.18103	457
19AR116_Spot 395	164.564	155609.7	0.618075	4.80589	0.558762	16.00673	0.807369	0.555015	0.582544	0.721534	2845.995	13.40349	2877.199	7.715999	2899.809	9.065612	2899.8	9.1	98.14422	351
19AR116_Spot 278	44.03233	42711.9	1.109657	4.298738	0.699881	19.81048	0.933082	0.61602	0.615524	0.659668	3094.061	15.12558	3082.151	9.019325	3075.085	11.20364	3075.1	11.2	100.6171	247
19AR116_Part2_Spot 363	216.501	124255.3	1.346683	4.214291	0.574967	20.01482	0.87188	0.611816	0.655333	0.751632	3077.268	16.03563	3092.073	8.431847	3102.38	9.162744	3102.4	9.2	99.19054	841
19AR116_Spot 498	816.7598	244919.2	2.249222	3.402138	0.562985	27.73196	0.840994	0.682151	0.62473	0.742848	3352.606	16.33154	3409.666	8.242282	3444.038	8.739008	3444.0	8.7	97.34522	439
19AR116_Spot 576	350.166	346749.7	3.717558	3.063188	0.559426	32.92377	0.872845	0.727496	0.669978	0.76758	3524.08	18.18836	3578.328	8.601673	3609.493	8.582963	3609.5	8.6	97.63366	509
